# An In silico Approach towards Finding the Cancer-Causing Mutations in Human MET Gene

**DOI:** 10.1155/2023/9705159

**Published:** 2023-05-09

**Authors:** Fayeza Sadia Laskar, Md. Nazmul Islam Bappy, Md. Sowrov Hossain, Zenifer Alam, Dilruba Afrin, Sudeb Saha, Kazi Md. Ali Zinnah

**Affiliations:** ^1^Faculty of Biotechnology and Genetic Engineering, Sylhet Agricultural University, Sylhet 3100, Bangladesh; ^2^Department of Animal and Fish Biotechnology, Sylhet Agricultural University, Sylhet 3100, Bangladesh; ^3^Department of Pharmaceuticals and Industrial Biotechnology, Sylhet Agricultural University, Sylhet 3100, Bangladesh; ^4^Faculty of Veterinary, Animal and Biomedical Sciences, Sylhet Agricultural University, Sylhet 3100, Bangladesh; ^5^Department of Dairy Science, Sylhet Agricultural University, Sylhet 3100, Bangladesh

## Abstract

Mesenchymal–epithelial transition (MET) factor is a proto-oncogene encoding tyrosine kinase receptor with hepatocyte growth factor (HGF) or scatter factor (SF). It is found on the human chromosome number 7 and regulates the diverse cellular mechanisms of the human body. The impact of mutations occurring in the MET gene is demonstrated by their detrimental effects on normal cellular functions. These mutations can change the structure and function of MET leading to different diseases such as lung cancer, neck cancer, colorectal cancer, and many other complex syndromes. Hence, the current study focused on finding deleterious non-synonymous single nucleotide polymorphisms (nsSNPs) and their subsequent impact on the protein's structure and functions, which may contribute to the emergence of cancers. These nsSNPs were first identified utilizing computational tools like SIFT, PROVEAN, PANTHER-PSEP, PolyPhen-2, I-Mutant 2.0, and MUpro. A total of 45359 SNPs of MET gene were accumulated from the database of dbSNP, and among them, 1306 SNPs were identified as non-synonymous or missense variants. Out of all 1306 nsSNPs, 18 were found to be the most deleterious. Moreover, these nsSNPs exhibited substantial effects on structure, binding affinity with ligand, phylogenetic conservation, secondary structure, and post-translational modification sites of MET, which were evaluated using MutPred2, RaptorX, ConSurf, PSIPRED, and MusiteDeep, respectively. Also, these deleterious nsSNPs were accompanied by changes in properties of MET like residue charge, size, and hydrophobicity. These findings along with the docking results are indicating the potency of the identified SNPs to alter the structure and function of the protein, which may lead to the development of cancers. Nonetheless, Genome-wide association study (GWAS) studies and experimental research are required to confirm the analysis of these nsSNPs.

## 1. Introduction

Mesenchymal–epithelial transition (MET) factor gene, which is also called c-MET, is a proto-oncogene that encodes a receptor tyrosine kinase for hepatocyte growth factor (HGF) or scatter factor (SF). It belongs to the MNNG HOS (N-methyl-N′-nitroso-guanidine human osteosarcoma) transforming gene family and is situated on human chromosome number 7 (7q21–q31) [1, 2]. The MET gene is 125 kb long, which comprises 21 exons and 20 introns, encoding a protein whose size is around 120 kDa [2]. The MET protein encoded by this gene is a single chain precursor of 1390 amino acid residues. Following the translation process, the MET protein is transferred to the cell apparatus, i.e., the Golgi reticulum, and goes through the glycosylation process [3].

The extracellular and intracellular portions of MET protein contain several domains. HGF binding to MET protein has an effect on receptor dimerization and phosphorylation of tyrosine residues of the protein, which eventually leads to the phosphorylation of intracellular docking sites [4]. MET gene stimulation prompts composite signaling processes that rely on the cellular environment and generates a range of responses related to cells [5]. This signaling pathway aids in various biological mechanisms like wound healing, hepatic regeneration, embryonic, neuronal, and muscle development. Still, disturbance in MET signaling pathways, due to any kind of alterations or mutations, intervenes cell proliferation, cell death, and migration and is involved in several malignancies [4]. The overexpression or activation of MET receptor, due to mutations, has been associated with the expansion of several human cancers, comprising carcinomas, sarcomas, hematopoietic malignancies, melanomas, and also central nervous system tumors [5].

Single-nucleotide polymorphisms (SNPs) are the most prevalent type of genetic alteration or mutation, affecting a single base pair in alleles in one or more individuals [6]. A subgroup of SNPs occurs in protein coding regions of the genome, and the non-synonymous single nucleotide polymorphisms (nsSNPs) or missense variants, which result in an amino acid change at the protein level, are particularly relevant from a medical perspective [7]. Thus, nsSNPs are of specific interest as candidates for further evaluation [6]. A major challenge in modern sequencing studies is highlighting missense variants for further experimental analysis [8]. The ability to computationally distinguish between pathogenic and benign variants could assist in the selection of prospective contenders from a group of data for targeting disease-causing mutations [7].

A concentrated comprehension of how MET gene activation manages tumor development will need additional investigation utilizing 3D molecular configurations, cell culture systems, human tumors, and also animal prototypes. With the help of these combined techniques, and also with novel medications targeting MET gene, shortly, the impact of MET on tumorigenesis will be recognized and may be controlled in a better way [5]. Correlating the various nsSNPs with their phenotypic features utilizing wet laboratory techniques can be perplexing. Identifying the functional nsSNPs from the large pool of data using wet laboratory techniques could be awkward. Most of the bioinformatics algorithms make their calculations based on sequence, structure, physicochemical, and evolutionary conservation to identify the deleterious nsSNP. These in silico-based modeling help to clarify the different properties of the protein molecules, for detailed information on the conformational changes of the protein [9]. They can control many more variables much more specifically within a short time than wet laboratory experiments, which enable researchers to explore the different components in various ways within the shortest possible time. Moreover, large datasets, which would be difficult or impossible for people to manually analyze by hand, may include patterns that bioinformatics has the ability to identify. In the case of SNPs analysis, more than 2 million SNPs have been documented by the SNP consortium [10, 11], and the total number is estimated to be >10 million [12], which is quite impossible to test the subsequent effects of those SNPs on their corresponding genes in a web lab setting. Thus, bioinformatics will allow us to screen the potentially damaging ones and then confirm their effects in vivo conditions, which will eventually lead to the saving of time and money. Moreover, it can be time-consuming to figure out where a test went wrong in the wet lab setting. Even if a failed test may not be the fault of a specific technician, it nonetheless adds to everyone's effort. Trying to figure out when, where, or how exactly went wrong can be quite irritating.

This study aims to determine and evaluate the effects of deleterious nsSNPs on the structure, function, and other various properties of MET gene, which can eventually lead to oncogenesis. The major intents of this research work are to identify and analyze the most deleterious nsSNPs of MET gene. Earlier the effects of oncogenic mutation on isocitrate dehydrogenase 1 [13], the impact of point mutation P29S in RAS-related C3 botulinum toxin substrate 1 [14], the role of T315I in BCR-ABL1 protein [15], effects of point mutation (R482W) in lamin A/C protein for laminopathy [16], and the impact of deleterious nsSNPs on human POLD1 gene [17] have been revealed using computational analysis. Bioinformatics analysis is not merely utilized in the deleterious mutation prediction rather it can further be expanded to the screening of suitable anticancer drugs [18, 19].

In the present study, the nsSNPs or missense variants were acquired from the SNP dataset of MET gene for analyzing their functional and structural effects of them. The most deleterious nsSNPs were identified and analyzed their impacts on normal properties of MET. Different types of computational tools, which are now broadly involved with identifying missense mutations, were utilized for the ample identification and analysis of damaging nsSNPs in this research work. In previous different studies, these tools were utilized, which helped us in selecting a research workflow synchronized with these tools. They were widely used for the first inspection of prospective candidates in many other research experiments related to mutation analysis [20, 21]. Each tool used in this study has the ability to provide a score-based outcomes, which can ultimately point out deleterious mutations with better prediction efficiency than other available computational tools.

## 2. Materials and Methods

**Figure 1:**
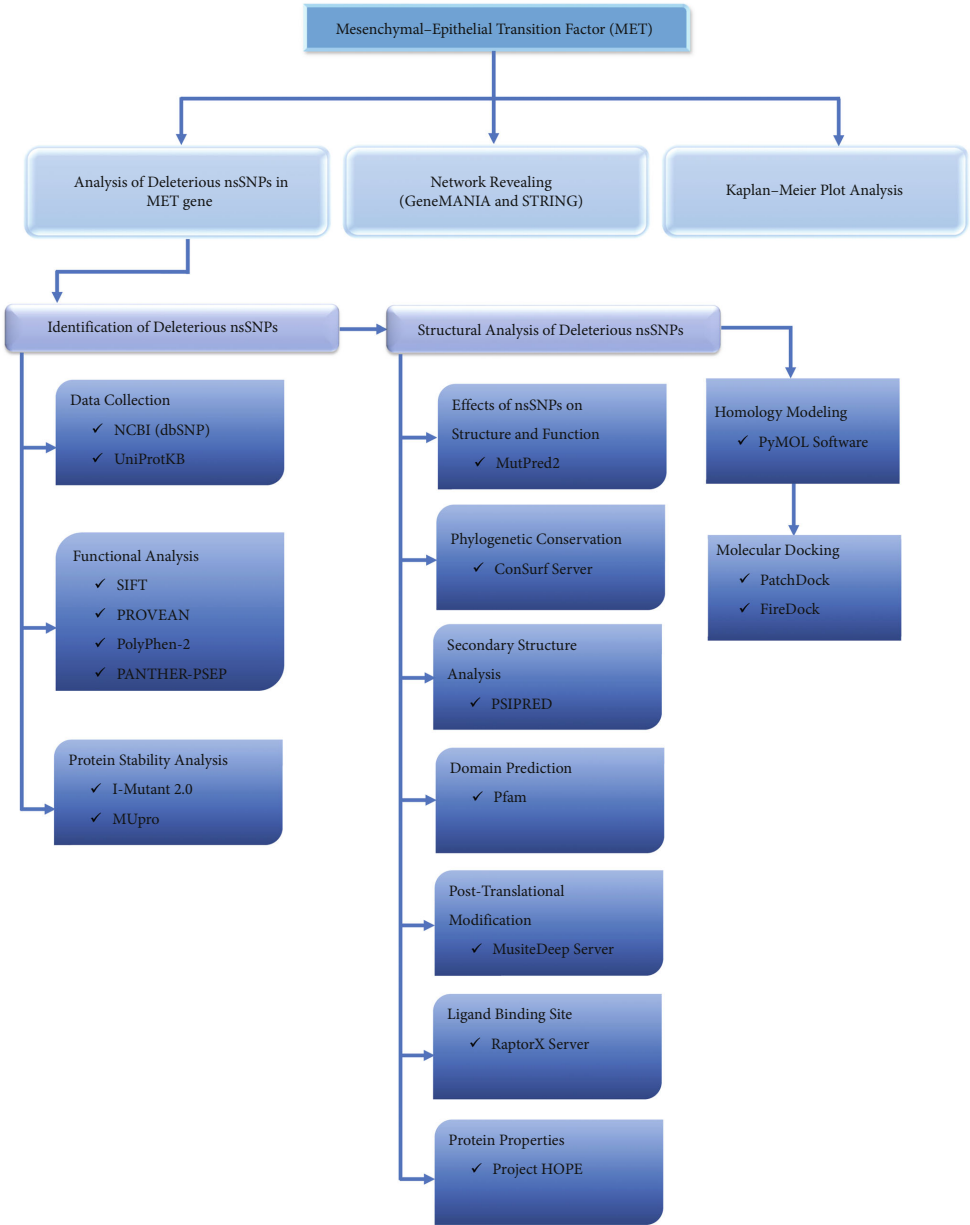
Schematic outline of the protocol.

The damaging effect of non-synonymous mutations in the structure and function of the MET protein was predicted and evaluated using various bioinformatics tools. An outline of the computational approaches used in this study is illustrated in Figure 1.

### 2.1. Data Collection

All data about the MET gene were collected from a web-based source, National Centre for Biotechnology Information (NCBI) (https://www.ncbi.nlm.nih.gov/). The comprehensive dataset of SNP mutations of the MET gene was retrieved from the dbSNP database of NCBI (https://www.ncbi.nlm.nih.gov/snp/). The protein sequence (P08581) of MET gene was retrieved from UniProtKB database (https://www.uniprot.org/).

### 2.2. Functional Analysis

#### 2.2.1. Screening of Deleterious nsSNPs

Only the missense mutations or nsSNPs from the total SNPs of the MET gene were retrieved from the dataset of SNP mutations of the MET gene for screening tolerated and deleterious nsSNPs, among which tolerated nsSNPs were excluded from the analysis. This screening was done by the following computational tools.

*(1) Sorting Intolerant from Tolerant*. Sorting Intolerant from Tolerant (SIFT) is a web-based tool (https://sift.bii.a-star.edu.sg/) [22] that analyzes the effect of an amino acid substitution on the protein function. It evaluates the data based on the physical properties of amino acids and sequence homologies of the protein sequence [21]. SIFT server aids in linking the divergence between mutations and phenotypic alterations [22]. This program can also be able to differentiate between neutral and deleterious mutations, which can have an effect on protein function [23]. The estimated output values of SIFT server are normally in the range between 0 and 1, while “0” indicates damaging and “1” indicates neutrality. The reference sequence identities (rsIDs) of nsSNPs were submitted in this server for the prediction of functional changes of amino acids.

*(2) Protein Variation Effect Analyzer*. Protein Variation Effect Analyzer (PROVEAN) is an online tool (http://provean.jcvi.org/) [24] for the prediction of the functional outcomes of amino acid alternatives [21]. It can make estimations for numerous amino acid changes such as substitutions, insertions, and deletions, along with single amino acid changes by means of a similar fundamental estimation pattern [24]. The PROVEAN server is centered on a cut-off value which is −2.5. The PROVEAN server includes three primary tools and these are PROVEAN protein, PROVEAN protein batch, and PROVEAN genome variants [25]. The input (FASTA sequence of protein and amino acid variations) was submitted in the PROVEAN protein tool of this server.

#### 2.2.2. Predicting Functional Effects of nsSNPs

The functional consequences of these nsSNPs of MET gene accumulated from dbSNP–NCBI database were analyzed by two in silico tools, i.e., PolyPhen-2 and PANTHER-PSEP server.

These tools will determine the damaging effects of non-synonymous mutations.

*(1) Polymorphism Phenotyping v2*. Polymorphism Phenotyping v2 (PolyPhen-2) server is an acclaimed bioinformatics tool (http://genetics.bwh.harvard.edu./pph2/) [26], which is used for determining the damaging effect of missense or non-synonymous mutations. An iterative greedy algorithm nominated eight sequences and three structure-based analytical attributes routinely, which were then utilized by the PolyPhen-2 server [26]. The FASTA sequence of protein or protein identifier of the MET gene was submitted in this server followed by the submission of the position of the amino acid substitution. The particular amino acid residues were put in apposite boxes, whereas wild type residue in AA1 and mutant residue in AA2.

*(2) PANTHER-PSEP*. PATHER-position-specific evolutionary preservation, version 17.0 (PANTHER-PSEP) is a web-based tool (http://www.pantherdb.org/tools/csnpScoreForm.jsp) [27], which is used for the prediction of damaging missense or non-synonymous mutations [27]. The prediction of this server is based on the position of amino acid variations with a particular value of evolutionary conservation, which is estimated from the sequence alignment of various evolutionary-linked proteins [28]. FASTA sequence of protein and amino acid substitutions was submitted in the PANTHER-PSEP server for the prediction of the damaging effect of substitutions.

### 2.3. Protein Stability Analysis

The missense or nsSNPs can change the stability of the protein, which directly has an effect on protein function. So, it is essential to evaluate the effects of changing the stability of deleterious mutations [20]. Hence, to analyze the protein stability change, two web-based tools were used in this study and these are I-Mutant 2.0 and MUpro.

#### 2.3.1. I-Mutant 2.0

I-Mutant 2.0 web server (https://folding.biofold.org/i-mutant/i-mutant2.0.html) is a standard programmed tool that is developed on the basis of support vector machine (SVM) and used for the evaluation of effects of protein stability changes for missense or non-synonymous mutations [29]. This online tool is usually applied to analyze the insignia of stability alterations of protein and the involvement of *ΔΔG* values upon missense mutation [20]. To predict the protein stability changes, the protein sequence (P08581) of the MET gene was submitted in this server followed by position and new amino acid residue after mutation.

#### 2.3.2. MUpro

MUpro (http://mupro.proteomics.ics.uci.edu/, MUpro 1.1) is an online tool that is used for predicting stability changes of protein upon mutations [30]. It is an SVM tool that can able to predict protein stability changes for single site amino acid mutations contingent on protein sequence and/or structural attributes based on *ΔΔG* values [21]. The protein sequence of MET gene was acquiesced followed by submission of substitution position with wild residue and mutant residue in apposite boxes.

### 2.4. Predicting Effects of nsSNPs on Structure and Function of Protein

Primarily, for characterizing mutations to analyze complex diseases, bioinformatics methods have been developed, which can identify the impact of pathogenicity of mutations by predicting statistical effects of nsSNPs on structure and function of protein. MutPred2 (MutPred 2.0) is a computational tool (http://mutpred.mutdb.org/) that advances the positioning of pathogenic nsSNPs over prevailing approaches, can generate mechanisms involved with potentially causative of ailment, and proceeds pathogenicity score on specific genomes [31]. The FASTA sequence of MET gene with 18 deleterious amino acid substitutions was submitted to the MutPred2 server.

### 2.5. Phylogenetic Conservation Analysis

The phylogenetic or evolutionary conservational score of an amino acid extremely influences the structure and function of a protein. This type of attribute of proteins is essential for interpreting the mutations that have a deleterious effect [21]. Hence, for the analysis of phylogenetic conservation, an in silico tool ConSurf was used. ConSurf server (https://consurf.tau.ac.il/) [32] is an online tool that is broadly used tool for exhibiting functional regions in macromolecules through the scrutiny of evolutionary conservation of amino acids or nucleotides substitutions based on the phylogenetic relationship among homologous sequences [32]. It estimates the conservation score within the range of 1–9 on the basis of the evolutionary proportion of sites by means of the empirical Bayesian or maximum likelihood (ML) approach [20]. To analyze the phylogenetic conservational scale of the deleterious nsSNPs within MET protein, the FASTA sequence of the protein was submitted to the ConSurf server.

### 2.6. Detecting the Presence of Deleterious nsSNPs in Secondary Structure

Secondary structures of proteins are considered native structural attributes that are alleviated through hydrogen bonds on the backbone and are also regarded as the linking networks between primary sequences and tertiary structures of proteins [33]. The two-phase neural network has been utilized to analyze secondary structures of proteins on the basis of position-specific scoring system formed by Position-Specific Iterated BLAST (PSI-BLAST) [34]. Hence, to predict the secondary structure of a protein, a web server PSIPRED or PSI-BLAST-based secondary structure prediction (http://bioinf.cs.ucl.ac.uk/psipred/) [34] is usually used. This server assimilates two feed-forward neural networks, which can carry out an investigation on predicted results acquired from PSI-BLAST [35]. The FASTA sequence of the protein was entered on the server to predict the secondary structure of MET protein.

### 2.7. Presence in Functional Domain

Functional domains of a protein can be predicted using a database named Pfam (https://pfam.xfam.org/) [36]. Pfam is a vast database that includes families and domains of proteins that are generally utilized to evaluate novel genomes and to monitor investigational operation on certain proteins [36]. It comprises multiple sequence alignments, along with profile hidden Markov models (profile HMMs), for uncovering these functional domains in new sequences of proteins [37]. The accession or protein ID was entered in this server for revealing the functional domains of the protein.

### 2.8. Prediction of Post-Translational Modification Sites

Post-translational modifications (PTMs) imply amino acid change with the addition of various side chains in proteins. These changes or modifications occur because of essential governing procedures to manage various cellular activities. Hence, PTMs have a major influence on the structure and function of proteins. Interference in these PTM sites can direct the disturbance of important biological mechanisms, which can cause several diseases [38]. In this study, MusiteDeep server (https://www.musite.net/) [39] was used for predicting PTM sites. MusiteDeep is an online tool that can generate a conventional deep-learning framework for the prediction and visualization of PTM site of protein [39]. The FASTA sequence of MET protein was submitted to the server for predicting the various PTM sites of MET protein.

### 2.9. Analyzing Effects of nsSNPs on Ligand Binding Sites

The interfaces between proteins and ligands ensue through amino acid residues at particular sites of the protein, which are generally positioned in pocket-like regions. These regions are known as ligand binding sites. The ligand binding sites have enormous importance in the fields of molecular dynamics, molecular docking, and drug designing. That's why the prediction of binding sites is essential for discovering the intermolecular procedures as well as for understanding the pathogenicity of diseases [40]. In this research work, RaptorX-Binding web server (http://raptorx.uchicago.edu/BindingSite/) [41] was used for predicting ligand binding sites. RaptorX server is an online platform for the prediction of secondary structure, structure modeling, quality evaluation, and also alignment sampling [41]. On the other hand, RaptorX-Binding, a version of RaptorX, is a bioinformatics tool that can predict the ligand binding sites of a protein based on a 3D model structure with multiplicity value configured by RaptorX [42]. The sequence of MET protein was entered on the server as input for predicting ligand binding sites.

### 2.10. Analyzing Impacts of nsSNPs on Protein Properties

To predict the effects of deleterious nsSNPs on the properties of protein such as residue size, charge, hydrophobicity, structure, etc., HOPE (https://www3.cmbi.umcn.nl/hope/, version 1.1.1) server was used [43]. HOPE server can construct an instinctive mutant scrutiny platform, which can give a conception of the structural effects of a mutation [42]. The protein sequence or accession number was entered followed by the submission of mutant residue with the position.

### 2.11. Homology Modeling

**Figure 2:**
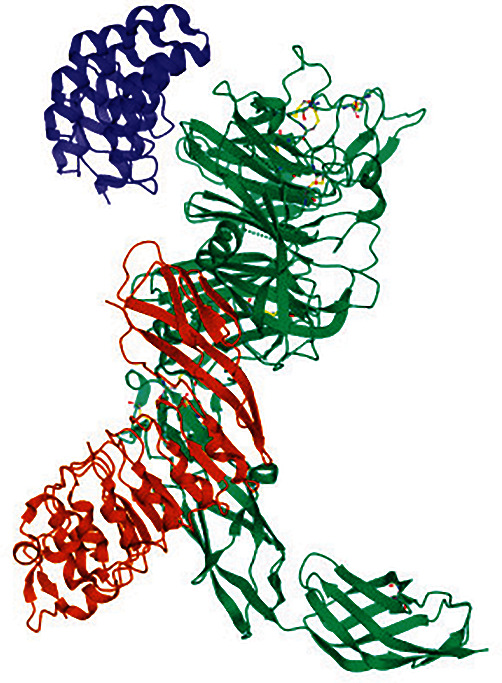
3D Structure of MET protein (PDB ID: 6GCU).

PyMOL software (PyMOL 2.5) was used for the homology modeling of variants of MET protein. For modeling of the variants, the structure of the MET protein (Figure 2) (PDB ID 6GCU) was acquired from Protein Data Bank (PDB) database (https://www.rcsb.org/) [44].

### 2.12. Predicting Effects of MET Mutant Complexes on Binding Affinity

Molecular docking is used to demonstrate the interaction between receptor and ligand molecules to analyze the effects of mutant complexes on binding affinity with their ligands. In this study, PatchDock (https://bioinfo3d.cs.tau.ac.il/PatchDock/) [45] server was used for docking purposes. This server intends to find docking alterations, which can acquire superior molecular shape complementarity [45]. For molecular docking, MET protein and its' mutant variants were entered as receptor molecules, and NK1 and NK2 dimer of HGF [46] were entered as ligand molecules in the PatchDock server. Clustering Root-mean-square deviation (RMSD) was set at 4.0, and complex type was set as default. After docking, FireDock (Fast Interaction Refinement) (https://bioinfo3d.cs.tau.ac.il/FireDock/) server was used for refinement. It marks the flexibility problem and solutions scoring generated by rigid body docking systems [47].

### 2.13. Analyzing Networking of MET with Other Genes

Gene Multiple Association Network Integration Algorithm (GeneMANIA) is a web tool (https://genemania.org/) that uncovers other genes associated with a set of genes that are submitted as input, expending a very large set of functional association data. These data comprise protein and genetic interactions, pathways, co-expression, co-localization, and also protein domain similarity [48]. It is an adaptable, accessible web platform for producing assumptions about gene function, scrutinizing gene lists, and arranging genes for functional tests [49]. The related gene name (MET) was entered into this server as input.

### 2.14. Predicting Interactions of MET with Other Proteins

Search Tool for the Retrieval of Interacting Genes (STRING) is an online database (https://string-db.org/, version 11.5) that combines gene–gene and/or protein–protein interactions into a network frame, with interfaces has their score of confidence [50]. The protein network encompasses high confidence interactors with scores ≥0.700 to avoid false negatives and false positives. Protein sequences were submitted, and the “minimum required interaction score” was set at “High confidence” (0.700).

### 2.15. Predicting Effects of MET Deregulation on the Survival Rate of Different Cancer Patients

The Kaplan–Meier Plot (https://kmplot.com/analysis/) [51] is an online tool that estimates the time of death, and this event may have a substantial inference while using these analytics for clinical decisions, medical policies, and also resource provision [52]. This plot is able to perform proportional survival analysis utilizing the data produced by genomic, transcriptomic, or proteomic investigations [51]. The prediction of the comprehensive survival rate of various types of cancer patients, i.e., lung, breast, ovarian, and gastric cancer, was done with MET gene dysregulation. The survival analyses of cancer patients were run against 1925, 4929, 1435, and 875 lung, breast, ovarian, and gastric cancer patients, respectively. The affymetrix ID was 217828_at for plot analysis.

## 3. Results

### 3.1. Retrieval of nsSNPs from dbSNP Database

**Figure 3:**
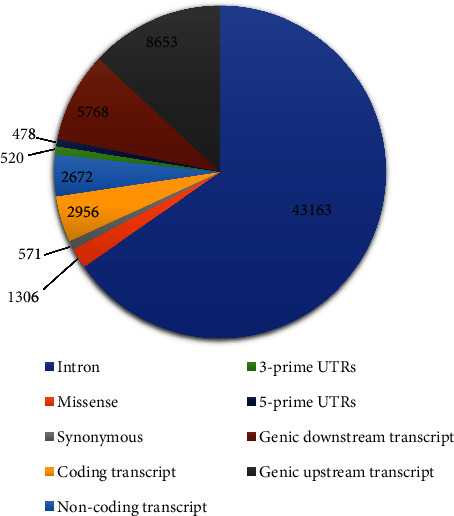
SNP types in MET.

A total of 45359 SNPs were obtained from the dbSNP database of NCBI. The obtained SNPs contain different variations. Among the 45359 nsSNPs, 1306 missense or non-synonymous, 571 synonymous, 2672 non-coding transcripts, 2956 coding transcripts, 520 3-prime UTR regions (Untranslated region), 478 5-prime UTR regions, 43163 intron, 5768 genic downstream transcript, 8653 genic upstream transcript, and other variants were found (Figure 3; Supplementary File 1).

### 3.2. Analyzing Functional Impacts of nsSNPs

#### 3.2.1. Prediction of Deleterious nsSNPs in MET

**Figure 4:**
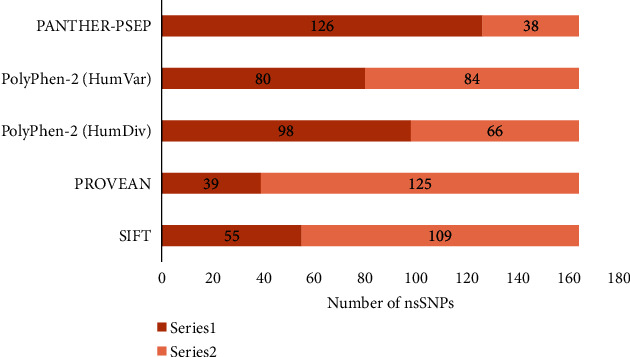
Distribution of deleterious and neutral nsSNPs of MET.

*(1) Sorting Intolerant from Tolerant*. The SIFT server postulates that the amino acids that are significant for protein function will be conserved and so then modifies at specific positions that are likely to be determined as deleterious mutations [23]. It estimates the possibility of an amino acid substitution at a specific position of the protein sequence. The estimated output values are normally in the range between 0 and 1, while “0” indicates a highly damaging mutation, and “1” indicates neutrality of mutation. If the values are less than 0.05, then the mutation is predicted to be deleterious but if the values are greater than 0.05, then the mutation is predicted to be tolerated [53]. The predicted deleterious nsSNPs usually have SIFT score of ≤0.05, and tolerated nsSNPs have SIFT score of ≥0.05. A total of 1306 missense or non-synonymous mutations were analyzed for the prediction of functional consequences using the SIFT server. Among the 1306 mutations, 164 nsSNPs gave the result. According to the result, 55 and 109 nsSNPs were predicted as “deleterious” and “tolerated”, respectively (Figure 4; Supplementary File 2). These 164 nsSNPs were determined by the tolerating index of SIFT server that ranges between 0 and 1, which indicates damaging and tolerated nsSNPs. But the other nsSNPs did not give values of the tolerating index of SIFT, which prevent them from showing prediction.

*(2) Protein Variation Effect Analyzer*. PROVEAN server utilizes the principal sequence of the protein and its sequence homologies, which are investigated by dint of BLAST in NCBI database. The prediction of PROVEAN is based on a scoring system where the cut-off value is −2.5 [54]. On the basis of this scoring system, amino acid substitution with PROVEAN score less than −2.5 will be determined as a deleterious mutation, while PROVEAN score greater than −2.5 will be determined as a neutral mutation [25]. Deleterious mutations have a ≤−2.5 score, and neutral mutations have a ≥−2.5 score in the prediction by PROVEAN server. According to this PROVEAN scoring scale, 39 and 125 nsSNPs were found as deleterious and neutral, respectively, among the 1306 missense mutations (Figure 4; Supplementary File 2).

#### 3.2.2. Prediction of Functional Consequences of nsSNPs

*(1) Polymorphism Phenotyping v2*. The PolyPhen-2 server accomplishes the prediction on the basis of a number of protein sequences, phylogenetic, and structural characteristics distinguishing amino acid substitutions. It extricates several sequences and structural attributes of amino acid substitutions and analyzes the probability of their damaging effect [55]. PolyPhen-2 server includes HumVar and HumDiv datasets that are based on naïve Bayes classifier administered by machine learning, where mutations are sorted as “probably damaging”, “possibly damaging”, or “benign” [42]. The predictive result of this server consists of a score ranging from zero to a positive number, while zero indicates the neutrality of amino acid substitutions, and a positive number indicates the damaging effect of mutations [56]. After the analysis of 164 amino acid substitutions in this server, two datasets (HumDiv and HumVar) with different mutations were found. In HumDiv dataset, 98 “damaging” (75 probably damaging and 23 possibly damaging) and 66 “benign” mutations were found, while, in HumVar dataset, 80 “damaging” (63 probably damaging and 17 possibly damaging) and 84 “benign” mutations were found (Figure 4; Supplementary File 3).

*(2) PANTHER-PSEP*. PANTHER-PSEP utilizes a metric-related arrangement, which is distinct from evolutionary preservation, whereas the potential sequences of proteins from the ancestral source at joints of a phylogenetic tree are restructured on the basis of homologous proteins [42]. It can construct a range of outcomes, while the most expedient being the possibility of a particular variant with a deleterious effect [56]. The PSEP result was generally categorized as “probably damaging”, “possibly damaging”, and “probably benign”, consequent to a false positive rate. After the exploration of 164 amino acid substitutions in this web server, three types of outcomes were found, i.e., 126 “damaging” (73 probably damaging and 53 possibly damaging) and 38 “probably benign” mutations (Figure 4; Supplementary File 4).

These two datasets of outcomes of PolyPhe-2 and PATHER-PSEP servers are distinguishable on the basis of their possible outcomes. PolyPhen-2 produces two different datasets based on a scoring system with sequences and structural attributes, whereas PATHER-PSEP constructs a range of outcomes on the basis of homologous proteins of a phylogenetic tree.

### 3.3. Prediction of Deleterious nsSNPs Based on Protein Stability

#### 3.3.1. I-Mutant 2.0

**Figure 5:**
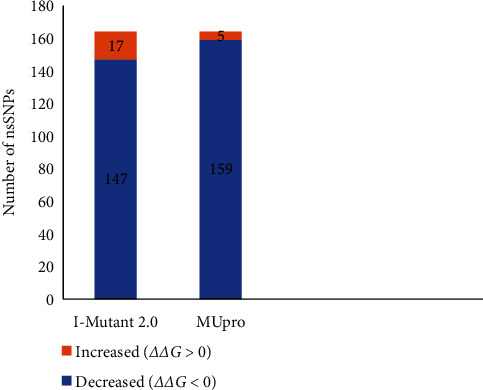
Distribution of nsSNPs based on protein stability.

The functionality of I-Mutant 2.0 depends on Gibbs free energy value (*ΔΔG*) of the protein [57]. I-Mutant 2.0 web server estimates the stability of the protein upon amino acid mutation by scrutinizing the Gibbs free energy or *ΔΔG* value, whereas *ΔG* (mutant protein)—*ΔG* (wild protein) in kcal/mol, which is evaluated at pH −7 and 25°C temperature [20]. The predicted positive value determines that the mutant protein has higher stability, while the negative value denotes that the mutant protein has lower stability. The outcomes of I-Mutant 2.0 are usually to be either increased or decreased value of Gibbs free energy upon amino acid mutations [57]. After analyzing 164 amino acid substitutions in this server, 147 nsSNPs were found with decreased stability, where *ΔΔG* value is negative or less than “0” and 17 nsSNPs were with increased stability, where *ΔΔG* value is positive or greater than “0” (Figure 5; Supplementary File 5).

#### 3.3.2. MUpro

MUpro server can evaluate stability changes of protein by utilizing information related to sequence or combined information of sequence and tertiary structure. The predicted value of Gibbs free energy or *ΔΔG* is similar to I-Mutant 2.0 server [42]. If the score acquired from MUpro server is less than “0” or negative, then the substitution decreases the structural stability of the protein, but if the score is greater than “0” or positive, then the substitution increases the structure stability [21]. In this analysis, among 164 nsSNPs, 159 nsSNPs were found with decreased stability (*ΔΔG* value less than “0”), and 5 nsSNPs were found with increased stability (*ΔΔG* value greater than “0”) (Figure 5; Supplementary File 5).

Among 1306 nsSNPs, only 164 nsSNPs gave prediction. After analyzing these 164 nsSNPs with the above six servers (SIFT, PROVEAN, PolyPhen-2, PANTHER-PSEP, MutPred2, I-Mutant 2.0, MUpro), only 18 nsSNPs were identified as the most deleterious mutations (Supplementary File 6) because they were predicted to decrease the protein stability and alter the function and thus can induce a deleterious effect on the encoded protein.

These 18 deleterious nsSNPs then went through structural analysis by utilizing the prominent in silico tools (ConSurf, PSIPRED, Pfam, MusiteDeep, RaptorX, and Project HOPE).

### 3.4. Prediction of Structural and Functional Effects of nsSNPs

MutPred2 server predicts molecular pathways associated with a pathogenic amino acid substitution [58]. It groups a mutation as pathogenic or neutral using a machine-learning-based technique. The MutPred2 prediction is based on the scoring system where the cutoff value of probability is 0.5. When the MutPred2 score of probability is greater than 0.5, the mutation will be determined as pathogenic, but when the MutPred2 score of probability is less than 0.5, the mutation will be determined as neutral [59]. Here, MutPred2 predicted the effects of the 18 selected nSNPs on the structure and function of the protein that includes loss of helix, loss of phosphorylation, gain of strand, gain of allosteric site, gain/loss of N-linked glycosylation and sulfation, altered transmembrane protein, altered metal binding and DNA binding, gain of relative solvent accessibility, and altered ordered interface (Supplementary File 7).

### 3.5. Assessment of Phylogenetic Conservation of Deleterious nsSNPs

**Figure 6:**
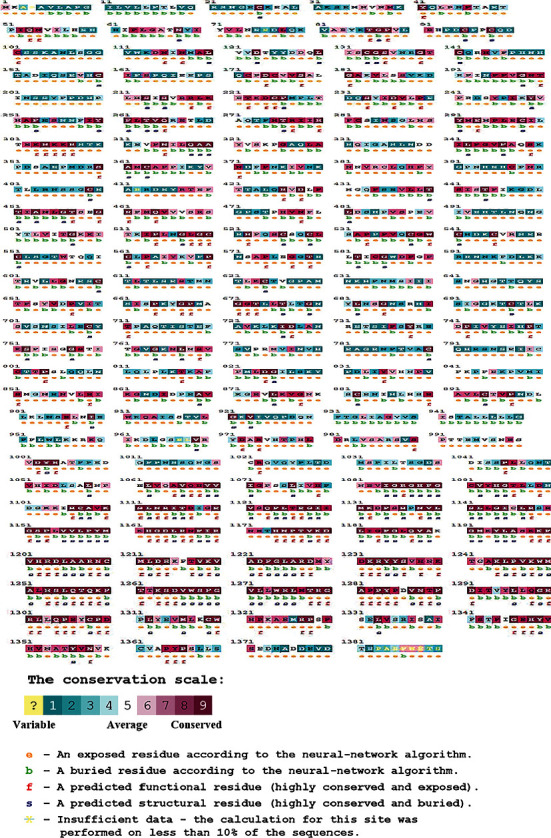
Phylogenetic conservation analysis by ConSurf server.

The phylogenetic conservation analysis of amino acids imparts a better interpretation of the importance of a specific amino acid residue and its evolutionary structure [60]. The conservation score on a scale of 7–9 is deemed to be conserved, but scores on a scale of 4–6 and 1–3 are contemplated to be average and variable, correspondingly [21]. Conservational analysis of the MET protein has been accomplished by means of the ConSurf tool, and the estimated conservational score revealed that the maximum amino acids of the MET protein are located in the highly conserved region. The predicted result of 18 deleterious nsSNPs showed that 11 residues were on the scale of “9”, 2 residues were on the scale of “8”, 2 residues were on the scale of “7”, 2 residues were on the scale of “6”, and 1 residue was on the scale of “5”. Since the highly damaging mutations are usually found in highly conserved regions, then the 11 residues with the score of “9” were perceived to be highly conserved with extreme damaging effects (Figure 6; Supplementary File 8).

### 3.6. Predicting Effects of nsSNPs on the Secondary Structure of MET

**Figure 7:**
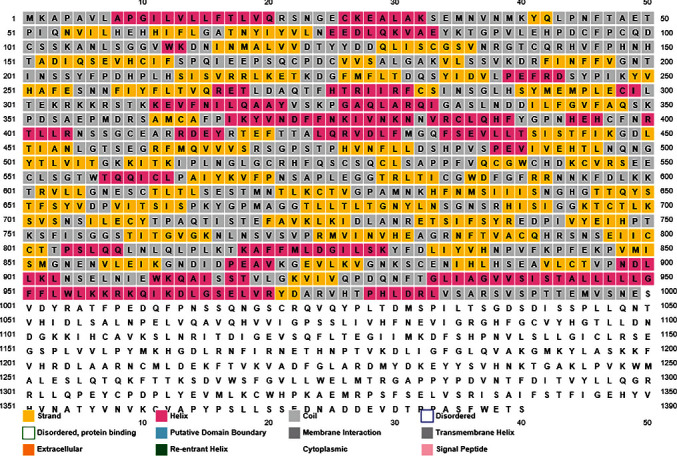
PSIPRED sequence plot of MET.

Secondary structures of MET protein were predicted using the PSIPRED server based on PSI-BLAST. The predicted sequence outline revealed the arrangement of alpha helix, beta sheet, extracellular, and coil structures. After the analysis of 18 amino acid substitutions by PSIPRED server, three different types of secondary structures were found. Among the 18 residues, 6 (33.33%) were predicted as “coil”, 6 (33.33%) were predicted as “helix”, and 6 (33.33%) were predicted as “extracellular” structures (Figure 7; Supplementary File 9).

### 3.7. Effects of Deleterious nsSNPs on Functional Domains of MET

Pfam database revealed the domains of MET protein upon the submission of the protein ID of MET. Six domains of MET protein were found and these domains were arranged between 317–493, 519–562, 563–654, 657–738, 742–833, and 1078–1337 amino acid residues, correspondingly (Table 1). Among the 18 deleterious mutations, 14 nsSNPs were uncovered in these predicted functional domains.

### 3.8. Predicting Impacts of nsSNPs on PTM Sites

The MusiteDeep server predicts PTM sites by using only protein sequences as input material and it results from a real time estimation for several proteins. The outcome is displayed at the level of amino acid residue for the various selected PTM sites [39]. This server predicts the PTM sites that have the possibility of transpiring due to the occurrence of highly damaging mutations in the MET protein. Phosphorylation is one of the fundamental forms of PTM sites and also the mostly analyzed PTM, which occurs usually in serine (S), threonine (T), and tyrosine (Y) residues [38]. Since phosphorylation is the most important PTM, the phosphorylation sites of the MET protein were analyzed in this server. In this PTM analysis, 21 amino acid residues were found in phosphorylation sites with a score greater than 0.5. But only one amino acid residue (Y1230) was found with phosphorylation, which was associated with highly deleterious nsSNPs among these 21 residues (Supplementary File 10).

### 3.9. Predicting Effects of Deleterious nsSNPs on Binding Sites of MET

The RaptorX server is different from other web servers by the value of the alignment amid a target protein sequence and distantly linked template proteins and also by a non-linear scoring system [41]. In this server, the multiplicity value is used as one of the conditions to predict the ligand binding sites of the protein. The multiplicity value of a pocket greater than 40 implies the accuracy of the predicted pocket [42]. After analyzing MET protein in the RaptorX-Binding server, four domains were found in total. The server found a sum of eight binding pockets based on its binding with different ligands. In the RaptorX-Binding server, the multiplicity value is used as one of the scoring systems to predict the ligand binding sites of the protein. This scoring value helps to determine the accuracy of predicted binding sites. In this assessment, only one residue M1131 (M1131T) was found under pocket 3 among the highly damaging 18 substitutions, and this residue was associated with M97 ligand and the multiplicity value was 97 (Table 2).

### 3.10. Predicting of Effects of Deleterious nsSNPs on Protein Properties

The HOPE server analyzed the effects of highly damaging nsSNPs on the properties of MET protein. In this study, 18 deleterious nsSNPs of MET were analyzed by this server based on different properties such as size, charge, hydrophobicity, etc. Among these 18 substitutions, nine mutants were found bigger than wild residues, while seven mutants were found smaller than wild residues. Furthermore, seven substitutions were less hydrophobic than wild types, whereas five substitutions were more hydrophobic than wild types. Besides, two mutations (P239R, H1094R) turned neutral to positively charged, while one mutation (G757E) turned neutral to negatively charged. On the other hand, three substitutions (D1228N, D340G, and D1180N) turned negatively charged to neutral (Supplementary File 11).

### 3.11. Modeling of Variants of MET Protein

**Figure 8:**
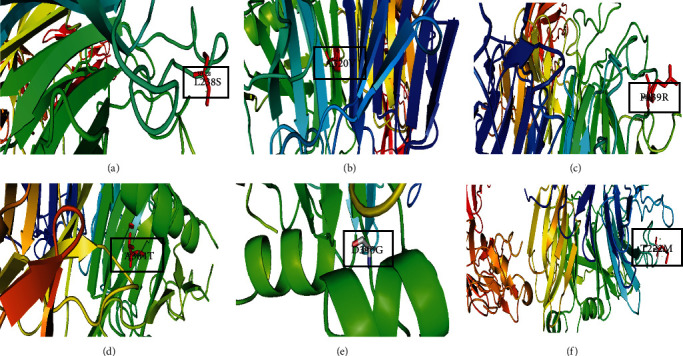
Homology modeling of (a) L238S variant, (b) A320V variant, (c) P239R variant, (d) A364T variant, (e) D340G variant, and (f) T222M variant of MET protein.

For homology modeling of proteins, six variants (L238S, A320V, P239R, A364T, D340G, and T222M) were selected because these variants were among 18 highly damaging residues. None of these lies in the binding pockets of MET gene as predicted by RaptorX-Binding site server. However, earlier experiments confirmed that 25–519 residues of MET protein are sufficient for binding HGF/SF, while 567–932 residues increase the binding affinity [61]. All these six SNPs were within the range of 25–519 residues and thus indicated a high possibility to affect the binding capacity. These six protein variants were modeled by creating mutations in the wild-type protein structure of MET using PyMOL software. The modeled structures of six mutant proteins were shown in Figure 8.

### 3.12. Analysis of Binding Affinity of Mutant Complexes

To analyze the effect of mutations on the structure and functions of MET protein, molecular docking analysis was carried out with these two specific NK1 and NK2, which are variants of HGF. Fourteen complexes were utilized and these are 2 native complexes (MET-NK1 and MET-NK1) and 12 mutant complexes (L238S-NK1, L238S-NK2, A320V-NK1, A320V-NK2, P239R-NK1, P239R-NK2, A364T-NK1, A364T-NK1, D340G-NK1, D340G-NK2, T222M-NK1, and T222M-NK2) for this analysis. PatchDock server was used for calculating binding energy and FireDock server for further refinement. Comparing the binding energy of native between mutant complexes, the mutant A364T-NK1 complex showed the weakest binding affinity with a binding energy of 6.31 when assessed with native and other mutant complexes (Table 3). This analysis confirmed that the mutant complex can show the least binding interaction than the native complex on the basis of their deleterious effect.

### 3.13. Analysis of Effects of Deleterious Mutations on Other Genes

**Figure 9:**
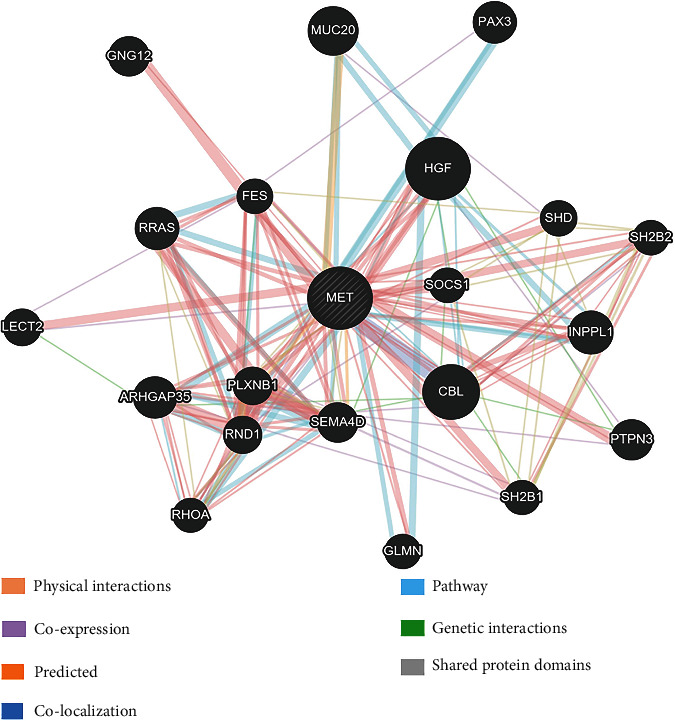
Interaction of MET gene with other genes.

GeneMANIA is a large collection of interaction networks from several data sources which identify genes and networks that are functionally associated [62]. This server analyzed the gene–gene interactions of MET by forming networking with other related genes. The GeneMANIA prediction of MET gene has shown that MET gene has interaction with other 20 different genes whose functions may be hampered as a result of mutation (Figure 9), and the detailed interaction data is provided in Supplementary File 12.

### 3.14. Analysis of Effects of Deleterious Mutations on Other Proteins

**Figure 10:**
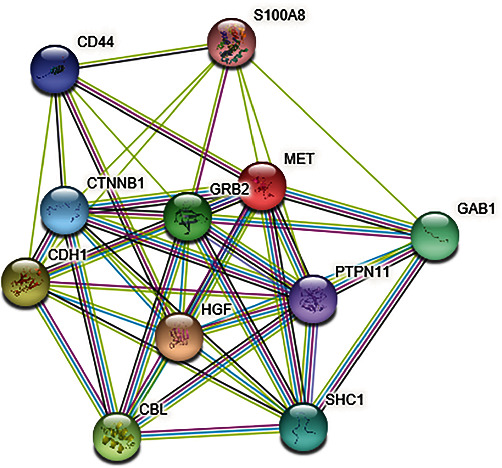
Protein–protein interaction network of MET protein.

Protein–protein interactions were investigated to interpret all functional interactions among cellular proteins [52]. The STRING database prediction showed that MET protein has interaction with other 10 proteins (Figure 10, Table 4) whose functions also might be interrupted by these mutations. It exhibited that MET protein interacts with 10 different proteins comprising HGF, growth factor receptor-bound protein 2 (GRB2), tyrosine–protein phosphatase non-receptor type 11 (PTPN11), GRB2-associated-binding protein 1 (GAB1), S100 calcium-binding protein A8 (S100A8), SHC-transforming protein 1 (SHC1), casitas B-lineage lymphoma (CBL), cell surface adhesion receptor (CD44), catenin beta 1 (CTNNB1), and cadherin 1 (CDH1).

### 3.15. Correlation of MET Deregulation with Various Types of Cancers

**Figure 11:**
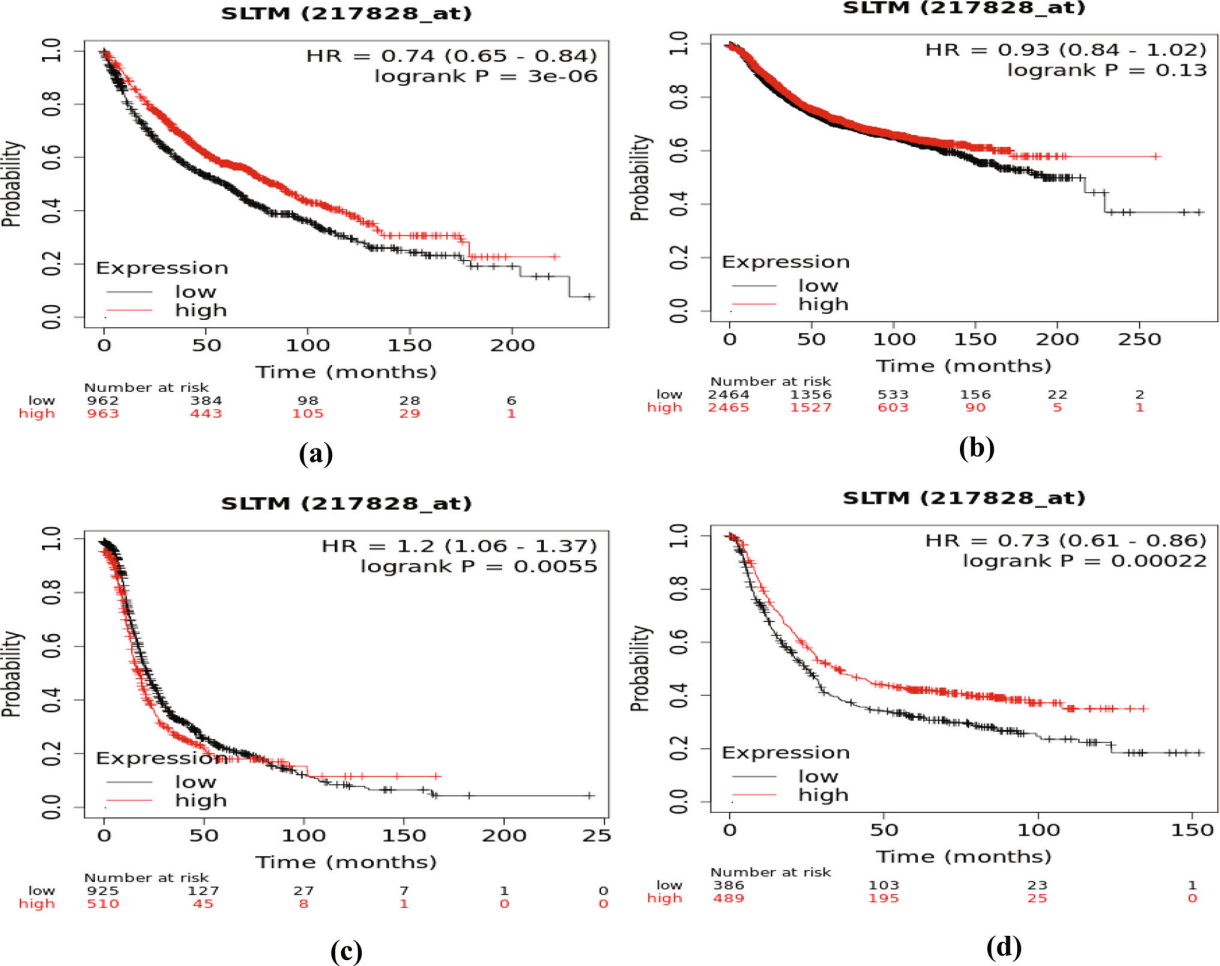
Clinical analysis of MET in (a) lungs, (b) breast, (c) ovarian, and (d) gastric cancer.

The clinical analysis discovered several consequences of MET gene deregulation in various kinds of cancers, i.e., lung, breast, ovarian, and gastric cancer. According to the results of the clinical investigation through Kaplan–Meier, MET gene deregulation was correlated with the survival rate of lung and gastric cancer patients. The expression curves in the lungs and gastric cancer plot had significant distances which defined the association of MET deregulation with the survival rate of cancer patients. But the expression level of both breast and ovarian cancer does not affect the survival rate of patients, since both of the expression curves were overlapped (Figure 11). Therefore, the deregulation of MET was predicted to be correlated more with lung and gastric cancer patients, which means they have less survival rate.

## 4. Discussion

The tertiary structure of a protein determines its function, hence any alteration to its amino acid sequence has the potential to alter the protein's structure and cause disease. The MET receptor with its ligand HGF synchronizes a range of functions of cells, where many cellular functions can be disturbed in human cancers. Disturbance in the MET signaling pathway can direct cellular movement and scattering, angiogenesis, proliferation, invasion, and eventually to metastasis [63]. Using various tools and algorithms, bioinformatics analysis enables us to anticipate the structural and functional impact of SNPs on a protein. In this study, the main focus was on the determination and evaluation of the effects of deleterious nsSNPs on the encoded protein of MET gene.

This analysis commenced with the separation of 1306 missense or nsSNPs from the 45359 SNPs of MET gene collected from the dbSNP database of NCBI. Then the MET protein sequence, accumulated from the UniProtKB database for conducting further analysis to identify the deleterious nsSNPs in MET gene. The functional and structural changes in protein occurred due to highly damaging nsSNPs, which were identified from the results acquired from various servers. At first, functional analysis of nsSNPs was carried out by some prominent computational tools, i.e., SIFT, PROVEAN, PolyPhen-2, and PANTHER-PSEP. In SIFT server, 55 deleterious and 109 neutral mutations were found among the total 164 amino acid substitutions, which have given the SIFT result. While in the PROVEAN, 39 and 125 mutations showed a deleterious and neutral effect on protein, respectively. On the other hand, PolyPhen-2 server showed two datasets of results, these are 98 and 66 damaging and benign mutations, respectively, in HumDiv dataset, whereas 80 and 84 damaging and benign mutations, respectively, in HumVar dataset. The PANTHER-PSEP server displayed 126 damaging and 38 benign mutations among 164 substitutions. Afterward, structural stability changes of MET protein were analyzed because the protein stability has a significant effect on the function and activity of proteins. Here, I-Mutant 2.0 and MUpro server were used for the prediction of stability changes of MET. In the I-Mutant 2.0 server, 147 mutations with decreased stability and 17 mutations with increased stability were found. While in the MUpro server, 159 and 5 mutations with decreased and increased stability, respectively, were found. But cautiousness should be maintained while analyzing the substitutions on the basis of *ΔΔG*. Even a substitution with a *ΔΔG* value except zero can cause substantial changes in the protein according to the comparative values of *ΔG* and *ΔΔG* [64]. An amino acid substitution that directs to a small extent of *ΔΔG* value may not cause prominent structural changes in a protein with a large *ΔG* value. Moreover, a few damaging mutations can be alleviated, which specifies that estimating pathogenicity via a particular method is very unreliable [65].

Then the results obtained from the above six servers were analyzed and compared, which eventually assorted 18 potentially deleterious mutations for MET protein. These 18 deleterious mutations went through the remaining six servers, i.e., MutPred2, ConSurf, PSIPRED, Pfam, MusiteDeep, RaptorX, and Project HOPE for further structural scrutiny. The MutPred2 predicted the statistical significance of nsSNPs on the structure and function of MET protein, where different characteristics of deleterious nsSNPs were revealed. The ConSurf server determined the conservational score of the MET protein for different amino acid residues, where 11 residues with score 9, 2 residues with score 8, 2 residues with score 7, 2 residues with score 6, and 1 residue with score 5. In conservation analysis, score 9 denotes highly conserved amino acid, and score 1 denotes the least conserved or variable amino acid. The highly conserved residue is predicted to be a highly damaging residue. The PSIPRED server estimated three types of secondary structures among the 18 deleterious substitutions, whereas six residues with coil structure, six residues with helix, and six residues with extracellular structure. After that, Pfam database showed six functional domains of MET protein upon submission of the protein accession number. The MusiteDeep server is able to predict the PTM sites, where only one amino acid residue (Y1230) as phosphorylation site in the prominent PTM sites among the 18 deleterious nsSNPs, which signifies the alteration in PTMs of MET protein due to Y1230H substitution. Then the RaptorX-Binding server determined the possible ligand binding sites of the MET protein. Any kind of change or variation at the ligand binding site of the protein can neutralize or reduce the activity of the protein. Among the 18 mutations, only one substitution (M1131T) was found in the binding sites of MET protein.

Since, the mutations can cause alterations in size, charge, and hydrophobicity of residue, which may lead to disruption of protein structure and interactions. The HOPE server can compare the wild and mutant residues based on different properties of the protein. From the result of HOPE, nine residues were bigger than the wild and seven residues smaller than the wild type; two positively charged, one negatively charged, and three neutral; seven residues were less hydrophobic; and five residues were more hydrophobic than the wild type among 18 deleterious substitutions.

The first 519 amino acid residues of MET protein are essential for HGF or SF binding, which comprises the first 212 amino acid residues of the beta (*β*) chain. Hence, it is feasible that the HGF or SF binding site is confined within this protein sequence. Within the longest structures, the strongest binding affinity was detected, but the binding affinity of 25–519 residues and 25–567 residues was easily assessable. On the other hand, 567–932 exhibited no binding affinity. As a result, the N-terminal portion of the MET ectodomain (25–519) is adequate for binding with HGF or SF, while the C-terminal part (567–932) has no binding affinity, but stimulates the binding affinity to the N-terminal portion (25–519). Therefore, after the structural analysis, six variants (L238S, A320V, P239R, A364T, D340G, and T222M) among 18 deleterious variants of MET protein were selected for homology modeling through PyMOL software, because MET protein has shown strong binding affinity between 25–519 residues. The modeled mutant variants then went through molecular docking via PatchDock and FireDock servers for analyzing the binding affinity of native and mutant complexes. The mutant complex A364T-NK1 with 6.31 binding energy showed the least binding interaction than native and other mutant complexes. This assessment showed that the mutant complexes can show the least binding interactions than native complexes on the basis of their deleterious effect. On the other hand, similar in silico analysis of the HGF gene revealed that five nsSNPs (D358G, G648R, I550N, N175S, and R220Q) of the HGF are the most deleterious that hinder MET–HGF interaction [66].

The gene–gene interactions analyzed by GeneMANIA server showed the networking of MET with other related genes, whereas the protein–protein interactions by STRING database revealed connections of MET with other proteins. These interactions demonstrate the significant alteration in functions and activity of other different networked proteins because of these deleterious nsSNPs of MET. However, the result of Kaplan–Meier Plot investigation specified that the MET gene deregulation can be regarded as a substantial diagnostic way in prognostic tool for identifying lung and gastric cancers patients because the plot showed less survival rate for them. This also revealed that the sex or gender-specific cancer's (such as ovarian and breast cancers that are common among females) survival percentage is not influenced by the deregulation of the MET gene. Considering that the deleterious nsSNPs of MET have a major influence on the structure and function of MET protein, they can affect the normal cellular functions in MET dysregulation. Among the identified 18 deleterious nsSNPs, most nsSNPs have no existing literature. Only 5 (H1094R, H1094Y, D1228N, Y1230H, and L1195V) of 18 deleterious nsSNPs have been found associated with resistance mechanisms to MET kinase inhibitors, which ultimately affects cancer treatments [67, 68]. The findings of this study showed that 5 of 18 deleterious nsSNPs were associated with cancer development and resistance mechanisms of cancer drugs. This comparative analysis of the findings of this research validated the outcomes and justified these nsSNPs being deleterious.

In this research work, the mutations or variations found in MET gene were explored and screened, which may have detrimental effects on human normal cellular functions, preceding different cancers, and other diseases. Since laboratory experiments on mutations require a huge time and labor, this computational study was carried out to identify the most deleterious mutations, for further research purposes. However, none of these predicted deleterious nsSNPs were found to be well studied in the broad spectrum. All the computational tools have shown us simply the predicted results. Yet, the sophistication of these algorithms solely depends on raw experimental data. The downstream structural and functional analysis may be faulty as a result of unreliable and inaccurate raw data. Hence, it is advised to employ a number of tools and come to a decision by comparing the outcomes of these tools. Furthermore, some computational tools may not be usable over time or developed with newer versions, which can make the experimental outcomes less specific. Also, several in vitro and in vivo studies should be used in the laboratory to validate the outcomes of the bioinformatics analysis.

## 5. Conclusion

Since the alterations due to non-synonymous mutations in MET gene have a negative effect on human cellular functions and can cause diverse types of cancers in humans, the whole screening of these mutations was required. The present study focused on the analysis of the detrimental effects of deleterious nsSNPs or mutations in MET. Eighteen most deleterious nsSNPs were detected in MET among 45359 SNPs for further practical field application in research intent. The effects on binding affinity also imply that mutation prompts changes in wild type proteins. The altered structure and function of MET protein can lead to dysregulated activities of the MET gene, which was further supported by the docking results that may induce complex diseases. Along with this, changes in structural stability can alter the conformation and other properties of proteins concerning cancer. These nsSNPs may have implications for treatment approaches and personalized medicine, and they may be employed in future experimental research to examine how they contribute to the pathophysiology of associated diseases.

## Figures and Tables

**Table 1:** Functional domains predicted by Pfam.

Family name	Family description	Entry type	Start position	End position	*E*-value	Amino acid substitutions
Sema	Semaphorins	Domain	317	493	3.9 × 10^−15^	A320V, D340G, A364T
PSI	Plexin repeat	Domain	519	562	0.19	
TIG	Ig-like fold	Domain	563	654	3.7 × 10^−10^	
TIG	Ig-like fold	Domain	657	738	4.2 × 10^−10^	
TIG	Ig-like fold	Domain	742	833	6.5 × 10^−5^	G757E
PK Tyr Ser-Thr	Receptor tyrosine kinase	Domain	1078	1337	1.6 × 10^−89^	H1094R, H1094Y, M1131T, S1141L, D1180N, V1188L, L1195V, D1228N, Y1230H, and T1261A

**Table 2:** Ligand binding sites predicted by RaptorX.

Domain	Pocket	Multiplicity	Ligand	Binding residues	Deleterious nsSNPs
1	1	63	ALQ	P51 Q53 N54 T67 I116 N117 M118 S135 L180 A182 K248 S487 G507	
	2	15	UNX	H61 V81 H150 T151 A152	
2	3	97	M97	I1084 V1092 A1108 K1110 E1127 M1131 L1140 L1157 P1158 Y1159 M1160 K1161 G1163 D1164 R1208 N1209 M1211 A1221 D1222 F1223	M1131T
3	4	77	BMA	S663 K665	
	5	76	NAG	S663 T676 T678 G679 I706	
	6	72	MAN	K665 L674 T676 S771	
	7	17	GAL	Y666 G667 G672 T673 L674 D824	
4	8	79	DA	D866 P867 E868 S889	

M97: 1-[(3R,4R)-4-(1H-indol-3-yl)-2,5-dioxopyrrolidin-3-yl]pyrrolo[3,2,1ij]quinolinium; ALQ: 2-METHYL-PROPIONIC ACID; BMA: beta-d-mannopyranose; NAG: 2-acetamido-2-deoxy-beta-D-glucopyranose; MAN: alpha-d-mannopyranose; GAL: beta-d-galactopyranose; DA: 2′-DEOXYADENOSINE-5′-MONOPHOSPHATE.

**Table 3:** Docking analysis of native and mutant complexes.

Protein (receptor)	Ligand	Global binding energy
Native MET receptor	NK1	−4.00
	NK2	−8.41
Mutant A238S	NK1	−4.00
	NK2	−8.41
Mutant A320V	NK1	−0.60
	NK2	−8.41
Mutant P239R	NK1	−4.00
	NK2	−8.41
Mutant A364T	NK1	6.31
	NK2	−8.41
Mutant D340G	NK1	−4.00
	NK2	−8.41
Mutant T222M	NK1	−4.00
	NK2	−11.86

**Table 4:** List of interacting proteins with their corresponding scores.

Interacting proteins	Full form of the proteins	Interacting scores
HGF	Hepatocyte growth factor	0.999
CDH1	Cadherin-1	0.999
CBL	E3 ubiquitin–protein ligase CBL	0.999
GRB2	Growth factor receptor-bound protein 2	0.999
GAB1	GRB2-associated-binding protein 1	0.998
SHC1	SHC-transforming protein 1	0.996
CTNNB1	Catenin beta-1	0.994
CD44	CD44 antigen	0.993
PTPN11	Tyrosine–protein phosphatase non-receptor type 11	0.989
S100A8	Protein S100-A8	0.987

## Data Availability

Dataset of this study is available from the corresponding author on reasonable request.

## Supplementary Materials

Supplementary Materials Supplementary File 1: All SNPs of MET gene.

Supplementary Materials Supplementary File 2: SIFT and PROVEAN results. Supplementary File 3: PolyPhen-2 prediction. Supplementary File 4: PANTHER-PSEP prediction. Supplementary File 5: I-Mutant 2.0 and MUpro prediction. Supplementary File 6: Deleterious nsSNPs of MET gene predicted by all computational tools. Supplementary File 7: Effects of nsSNPs on structural and functional properties of MET by MutPred2 server. Supplementary File 8: Conservancy scale of deleterious nsSNPs. Supplementary File 9: Position of deleterious nsSNPs in secondary structure. Supplementary File 10: Deleterious nsSNPs present in post-translational modification sites. Supplementary File 11: Effects of deleterious nsSNPs on different properties of MET protein. Supplementary File 12: Gene interactions data predicted by GeneMANIA.
