# Supplementary material for: An In silico Approach towards Finding the Cancer-Causing Mutations in Human MET Gene
Source: Int J Genomics. 2023 May 9;2023:9705159. doi: 10.1155/2023/9705159 (PMC10188262; doi:10.1155/2023/9705159)
Supplement: Supplementary Materials — Supplementary File 1: All SNPs of MET gene. [file 9705159.f1.pdf]

rsID	Functional Consequence
rs1621	non_coding_transcript_variant,3_prime_UTR_variant,genic_downstream_transcript_variant
rs6566	non_coding_transcript_variant,3_prime_UTR_variant,genic_downstream_transcript_variant
rs14456	non_coding_transcript_variant,3_prime_UTR_variant,genic_downstream_transcript_variant
rs41736	non_coding_transcript_variant,coding_sequence_variant,missense_variant,synonymous_variant,genic_downstream_transcript_variant
rs41737	genic_downstream_transcript_variant,non_coding_transcript_variant,synonymous_variant,coding_sequence_variant
rs41738	non_coding_transcript_variant,3_prime_UTR_variant,genic_downstream_transcript_variant
rs41739	non_coding_transcript_variant,3_prime_UTR_variant,genic_downstream_transcript_variant
rs1129355	non_coding_transcript_variant,3_prime_UTR_variant,genic_downstream_transcript_variant
rs1858830	upstream_transcript_variant,5_prime_UTR_variant,intron_variant,genic_upstream_transcript_variant
rs2023748	genic_downstream_transcript_variant,non_coding_transcript_variant,synonymous_variant,coding_sequence_variant
rs11762213	synonymous_variant,coding_sequence_variant,non_coding_transcript_variant,intron_variant,genic_upstream_transcript_variant
rs12540703	3_prime_UTR_variant,genic_downstream_transcript_variant,non_coding_transcript_variant
rs13223756	coding_sequence_variant,synonymous_variant,non_coding_transcript_variant
rs17138978	intron_variant
rs28444388	genic_upstream_transcript_variant,synonymous_variant,intron_variant,non_coding_transcript_variant,coding_sequence_variant
rs33917957	genic_upstream_transcript_variant,missense_variant,intron_variant,non_coding_transcript_variant,coding_sequence_variant
rs34349517	genic_upstream_transcript_variant,missense_variant,intron_variant,non_coding_transcript_variant,coding_sequence_variant
rs34589476	coding_sequence_variant,missense_variant,genic_downstream_transcript_variant,non_coding_transcript_variant
rs35225896	genic_upstream_transcript_variant,missense_variant,intron_variant,non_coding_transcript_variant,coding_sequence_variant
rs35284565	genic_upstream_transcript_variant,missense_variant,synonymous_variant,intron_variant,non_coding_transcript_variant,coding_sequence_variant
rs35469582	genic_upstream_transcript_variant,missense_variant,intron_variant,non_coding_transcript_variant,coding_sequence_variant
rs35601148	genic_upstream_transcript_variant,missense_variant,intron_variant,non_coding_transcript_variant,coding_sequence_variant
rs35763409	genic_upstream_transcript_variant,synonymous_variant,intron_variant,non_coding_transcript_variant,coding_sequence_variant
rs35775721	genic_upstream_transcript_variant,synonymous_variant,intron_variant,non_coding_transcript_variant,coding_sequence_variant
rs35776110	genic_upstream_transcript_variant,missense_variant,intron_variant,non_coding_transcript_variant,coding_sequence_variant
rs36080330	intron_variant,genic_upstream_transcript_variant
rs41281081	3_prime_UTR_variant,genic_downstream_transcript_variant,non_coding_transcript_variant
rs45440991	coding_sequence_variant,missense_variant,non_coding_transcript_variant
rs45441497	coding_sequence_variant,non_coding_transcript_variant,missense_variant,5_prime_UTR_variant
rs45446492	coding_sequence_variant,missense_variant,non_coding_transcript_variant
rs45460604	coding_sequence_variant,missense_variant,non_coding_transcript_variant
rs45483396	genic_upstream_transcript_variant,missense_variant,intron_variant,non_coding_transcript_variant,coding_sequence_variant
rs45499391	coding_sequence_variant,non_coding_transcript_variant,synonymous_variant,5_prime_UTR_variant
rs45520237	coding_sequence_variant,synonymous_variant,non_coding_transcript_variant
rs45551737	genic_upstream_transcript_variant,missense_variant,intron_variant,non_coding_transcript_variant,coding_sequence_variant
rs45552236	genic_upstream_transcript_variant,synonymous_variant,intron_variant,non_coding_transcript_variant,coding_sequence_variant
rs45553236	coding_sequence_variant,missense_variant,non_coding_transcript_variant
rs45561544	missense_variant,synonymous_variant,non_coding_transcript_variant,coding_sequence_variant,genic_downstream_transcript_variant
rs45564937	coding_sequence_variant,missense_variant,genic_downstream_transcript_variant,non_coding_transcript_variant
rs45571634	coding_sequence_variant,synonymous_variant,genic_downstream_transcript_variant,non_coding_transcript_variant
rs45572835	coding_sequence_variant,synonymous_variant,non_coding_transcript_variant
rs45575240	coding_sequence_variant,missense_variant,non_coding_transcript_variant
rs45575438	coding_sequence_variant,synonymous_variant,genic_downstream_transcript_variant,non_coding_transcript_variant
rs45578433	coding_sequence_variant,missense_variant,genic_downstream_transcript_variant,non_coding_transcript_variant
rs45585831	coding_sequence_variant,missense_variant,non_coding_transcript_variant
rs45586239	coding_sequence_variant,missense_variant,non_coding_transcript_variant
rs45587940	coding_sequence_variant,missense_variant,non_coding_transcript_variant
rs45592846	coding_sequence_variant,missense_variant,genic_downstream_transcript_variant,non_coding_transcript_variant
rs45602940	coding_sequence_variant,missense_variant,synonymous_variant,non_coding_transcript_variant
rs45607832	coding_sequence_variant,missense_variant,genic_downstream_transcript_variant,non_coding_transcript_variant
rs45612435	coding_sequence_variant,missense_variant,genic_downstream_transcript_variant,non_coding_transcript_variant
rs45628136	coding_sequence_variant,missense_variant,genic_downstream_transcript_variant,non_coding_transcript_variant
rs55755322	coding_sequence_variant,synonymous_variant,non_coding_transcript_variant
rs55985569	genic_upstream_transcript_variant,missense_variant,synonymous_variant,intron_variant,non_coding_transcript_variant,coding_sequence_variant
rs56311081	genic_upstream_transcript_variant,missense_variant,stop_gained,intron_variant,non_coding_transcript_variant,coding_sequence_variant
rs56340719	genic_upstream_transcript_variant,missense_variant,synonymous_variant,intron_variant,non_coding_transcript_variant,coding_sequence_variant
rs56391007	coding_sequence_variant,missense_variant,genic_downstream_transcript_variant,non_coding_transcript_variant
rs57349036	intron_variant
rs59399612	non_coding_transcript_variant,3_prime_UTR_variant,genic_downstream_transcript_variant
rs73469198	intron_variant
rs73471130	intron_variant,genic_downstream_transcript_variant
rs73471132	intron_variant,genic_downstream_transcript_variant
rs74994656	intron_variant
rs76322625	non_coding_transcript_variant,3_prime_UTR_variant,genic_downstream_transcript_variant
rs76731671	intron_variant,genic_downstream_transcript_variant,downstream_transcript_variant
rs77398083	intron_variant,genic_downstream_transcript_variant
rs77523018	non_coding_transcript_variant,intron_variant,coding_sequence_variant,missense_variant,genic_upstream_transcript_variant
rs80048442	non_coding_transcript_variant,intron_variant,synonymous_variant,coding_sequence_variant,missense_variant,genic_upstream_transcript_variant
rs80153920	non_coding_transcript_variant,intron_variant,initiator_codon_variant,missense_variant,genic_upstream_transcript_variant
rs80256822	non_coding_transcript_variant,intron_variant,coding_sequence_variant,missense_variant,genic_upstream_transcript_variant
rs112241458	intron_variant
rs115574135	missense_variant,non_coding_transcript_variant,coding_sequence_variant
rs118057172	synonymous_variant,non_coding_transcript_variant,missense_variant,coding_sequence_variant

rs121913243	genic_downstream_transcript_variant,non_coding_transcript_variant,missense_variant,coding_sequence_variant
rs121913244	genic_downstream_transcript_variant,non_coding_transcript_variant,missense_variant,coding_sequence_variant
rs121913245	genic_downstream_transcript_variant,non_coding_transcript_variant,missense_variant,coding_sequence_variant
rs121913246	genic_downstream_transcript_variant,non_coding_transcript_variant,missense_variant,coding_sequence_variant
rs121913247	genic_downstream_transcript_variant,non_coding_transcript_variant,missense_variant,coding_sequence_variant
rs121913668	genic_downstream_transcript_variant,non_coding_transcript_variant,missense_variant,coding_sequence_variant
rs121913669	genic_downstream_transcript_variant,non_coding_transcript_variant,missense_variant,coding_sequence_variant
rs121913670	genic_downstream_transcript_variant,non_coding_transcript_variant,missense_variant,coding_sequence_variant
rs121913671	genic_downstream_transcript_variant,non_coding_transcript_variant,missense_variant,coding_sequence_variant
rs121913673	genic_downstream_transcript_variant,non_coding_transcript_variant,missense_variant,coding_sequence_variant
rs121913675	genic_downstream_transcript_variant,non_coding_transcript_variant,missense_variant,coding_sequence_variant
rs121913676	genic_downstream_transcript_variant,non_coding_transcript_variant,missense_variant,coding_sequence_variant
rs121913677	genic_downstream_transcript_variant,non_coding_transcript_variant,missense_variant,coding_sequence_variant
rs139377965	genic_downstream_transcript_variant,non_coding_transcript_variant,3_prime_UTR_variant
rs139535303	genic_downstream_transcript_variant,non_coding_transcript_variant,3_prime_UTR_variant
rs140853140	intron_variant
rs141733128	synonymous_variant,intron_variant,coding_sequence_variant
rs141961972	intron_variant,genic_upstream_transcript_variant
rs143472935	genic_downstream_transcript_variant,non_coding_transcript_variant,3_prime_UTR_variant
rs144126521	genic_upstream_transcript_variant,intron_variant
rs144156652	genic_downstream_transcript_variant,3_prime_UTR_variant,non_coding_transcript_variant
rs144559852	genic_downstream_transcript_variant,3_prime_UTR_variant,non_coding_transcript_variant
rs146651797	missense_variant,non_coding_transcript_variant,genic_downstream_transcript_variant,synonymous_variant,coding_sequence_variant
rs147343288	genic_downstream_transcript_variant,3_prime_UTR_variant,non_coding_transcript_variant
rs149188493	genic_downstream_transcript_variant,3_prime_UTR_variant,non_coding_transcript_variant
rs149713628	genic_downstream_transcript_variant,3_prime_UTR_variant,non_coding_transcript_variant
rs150534017	genic_downstream_transcript_variant,3_prime_UTR_variant,non_coding_transcript_variant
rs150588908	non_coding_transcript_variant,genic_upstream_transcript_variant,intron_variant,synonymous_variant,coding_sequence_variant
rs180985111	missense_variant,non_coding_transcript_variant,genic_upstream_transcript_variant,intron_variant,coding_sequence_variant
rs181068390	genic_downstream_transcript_variant,3_prime_UTR_variant,non_coding_transcript_variant
rs185301166	missense_variant,non_coding_transcript_variant,coding_sequence_variant,intron_variant,synonymous_variant,genic_upstream_transcript_variant
rs185897782	genic_downstream_transcript_variant,non_coding_transcript_variant,3_prime_UTR_variant
rs186175651	genic_downstream_transcript_variant,non_coding_transcript_variant,3_prime_UTR_variant
rs186524917	genic_downstream_transcript_variant,intron_variant
rs188625702	non_coding_transcript_variant,coding_sequence_variant,intron_variant,synonymous_variant,genic_upstream_transcript_variant
rs190739281	genic_downstream_transcript_variant,non_coding_transcript_variant,3_prime_UTR_variant
rs191198682	coding_sequence_variant,synonymous_variant,intron_variant,genic_upstream_transcript_variant,non_coding_transcript_variant
rs199502137	coding_sequence_variant,non_coding_transcript_variant,missense_variant
rs199542598	coding_sequence_variant,synonymous_variant,non_coding_transcript_variant,missense_variant
rs199585804	coding_sequence_variant,synonymous_variant,non_coding_transcript_variant
rs199643166	coding_sequence_variant,non_coding_transcript_variant,missense_variant
rs199701987	missense_variant,coding_sequence_variant,intron_variant,genic_upstream_transcript_variant,non_coding_transcript_variant
rs199736573	missense_variant,coding_sequence_variant,intron_variant,genic_upstream_transcript_variant,non_coding_transcript_variant
rs199761604	coding_sequence_variant,non_coding_transcript_variant,missense_variant
rs199763277	coding_sequence_variant,non_coding_transcript_variant,genic_downstream_transcript_variant,missense_variant
rs199771406	coding_sequence_variant,non_coding_transcript_variant,missense_variant
rs199776656	coding_sequence_variant,synonymous_variant,non_coding_transcript_variant
rs199883640	coding_sequence_variant,synonymous_variant,non_coding_transcript_variant,genic_downstream_transcript_variant
rs199892100	coding_sequence_variant,synonymous_variant,non_coding_transcript_variant,5_prime_UTR_variant
rs199922867	coding_sequence_variant,synonymous_variant,non_coding_transcript_variant
rs200003116	coding_sequence_variant,synonymous_variant,non_coding_transcript_variant,5_prime_UTR_variant
rs200016433	missense_variant,coding_sequence_variant,intron_variant,genic_upstream_transcript_variant,non_coding_transcript_variant
rs200050499	coding_sequence_variant,synonymous_variant,non_coding_transcript_variant
rs200074800	missense_variant,coding_sequence_variant,intron_variant,genic_upstream_transcript_variant,non_coding_transcript_variant
rs200138253	coding_sequence_variant,synonymous_variant,intron_variant,genic_upstream_transcript_variant,non_coding_transcript_variant
rs200218511	coding_sequence_variant,non_coding_transcript_variant,missense_variant
rs200255054	coding_sequence_variant,synonymous_variant,non_coding_transcript_variant
rs200283364	coding_sequence_variant,non_coding_transcript_variant,missense_variant
rs200315561	missense_variant,coding_sequence_variant,synonymous_variant,stop_gained,non_coding_transcript_variant,genic_downstream_transcript_variant
rs200345440	coding_sequence_variant,synonymous_variant,non_coding_transcript_variant,missense_variant
rs200494620	coding_sequence_variant,synonymous_variant,intron_variant,genic_upstream_transcript_variant,non_coding_transcript_variant
rs200524064	coding_sequence_variant,non_coding_transcript_variant,missense_variant
rs200601372	intron_variant
rs200624981	intron_variant,genic_downstream_transcript_variant
rs200633053	coding_sequence_variant,non_coding_transcript_variant,missense_variant
rs200659843	coding_sequence_variant,synonymous_variant,non_coding_transcript_variant,genic_downstream_transcript_variant
rs200690492	missense_variant,coding_sequence_variant,intron_variant,genic_upstream_transcript_variant,non_coding_transcript_variant
rs200740468	coding_sequence_variant,non_coding_transcript_variant,missense_variant
rs200754673	coding_sequence_variant,synonymous_variant,stop_gained,non_coding_transcript_variant,genic_downstream_transcript_variant
rs200776610	missense_variant,coding_sequence_variant,intron_variant,genic_upstream_transcript_variant,non_coding_transcript_variant
rs200819547	coding_sequence_variant,non_coding_transcript_variant,missense_variant
rs200861145	missense_variant,coding_sequence_variant,intron_variant,genic_upstream_transcript_variant,non_coding_transcript_variant
rs200865810	coding_sequence_variant,synonymous_variant,non_coding_transcript_variant,genic_downstream_transcript_variant
rs201037977	coding_sequence_variant,non_coding_transcript_variant,genic_downstream_transcript_variant,missense_variant

rs201111132	coding_sequence_variant,synonymous_variant,non_coding_transcript_variant,genic_downstream_transcript_variant
rs201154533	coding_sequence_variant,non_coding_transcript_variant,5_prime_UTR_variant,missense_variant
rs201180585	coding_sequence_variant,synonymous_variant,non_coding_transcript_variant
rs201191014	missense_variant,coding_sequence_variant,intron_variant,genic_upstream_transcript_variant,non_coding_transcript_variant
rs201245213	coding_sequence_variant,synonymous_variant,non_coding_transcript_variant,genic_downstream_transcript_variant
rs201271860	coding_sequence_variant,non_coding_transcript_variant,missense_variant
rs201274041	missense_variant,coding_sequence_variant,intron_variant,genic_upstream_transcript_variant,non_coding_transcript_variant
rs201304108	coding_sequence_variant,synonymous_variant,intron_variant
rs201315884	missense_variant,coding_sequence_variant,intron_variant,genic_upstream_transcript_variant,non_coding_transcript_variant
rs201467281	missense_variant,coding_sequence_variant,intron_variant,genic_upstream_transcript_variant,non_coding_transcript_variant
rs201519623	coding_sequence_variant,synonymous_variant,non_coding_transcript_variant,genic_downstream_transcript_variant
rs201628326	missense_variant,coding_sequence_variant,intron_variant,genic_upstream_transcript_variant,non_coding_transcript_variant
rs201632219	coding_sequence_variant,synonymous_variant,non_coding_transcript_variant
rs201687037	missense_variant,coding_sequence_variant,intron_variant,genic_upstream_transcript_variant,non_coding_transcript_variant
rs201789039	coding_sequence_variant,non_coding_transcript_variant,initiator_codon_variant,missense_variant
rs201861645	coding_sequence_variant,non_coding_transcript_variant,missense_variant
rs201878238	coding_sequence_variant,synonymous_variant,non_coding_transcript_variant
rs201975130	coding_sequence_variant,non_coding_transcript_variant,missense_variant
rs201980687	coding_sequence_variant,non_coding_transcript_variant,5_prime_UTR_variant,missense_variant
rs202026780	coding_sequence_variant,synonymous_variant,intron_variant,genic_upstream_transcript_variant,non_coding_transcript_variant
rs202047059	missense_variant,coding_sequence_variant,intron_variant,genic_upstream_transcript_variant,non_coding_transcript_variant
rs202166889	coding_sequence_variant,non_coding_transcript_variant,genic_downstream_transcript_variant,missense_variant
rs202194405	intron_variant
rs367628460	coding_sequence_variant,non_coding_transcript_variant,5_prime_UTR_variant,missense_variant
rs367634278	coding_sequence_variant,non_coding_transcript_variant,genic_downstream_transcript_variant,missense_variant
rs367764141	coding_sequence_variant,synonymous_variant,non_coding_transcript_variant
rs368144654	missense_variant,coding_sequence_variant,intron_variant,genic_upstream_transcript_variant,non_coding_transcript_variant
rs368150874	intron_variant,genic_downstream_transcript_variant
rs368328347	missense_variant,coding_sequence_variant,intron_variant,genic_upstream_transcript_variant,non_coding_transcript_variant
rs368750834	coding_sequence_variant,genic_upstream_transcript_variant,intron_variant,missense_variant,non_coding_transcript_variant
rs368787826	non_coding_transcript_variant,coding_sequence_variant,missense_variant
rs368891381	non_coding_transcript_variant,coding_sequence_variant,missense_variant
rs368942722	coding_sequence_variant,genic_upstream_transcript_variant,intron_variant,missense_variant,non_coding_transcript_variant
rs369148519	genic_downstream_transcript_variant,non_coding_transcript_variant,coding_sequence_variant,synonymous_variant
rs369312680	genic_downstream_transcript_variant,non_coding_transcript_variant,coding_sequence_variant,missense_variant
rs369639118	non_coding_transcript_variant,coding_sequence_variant,synonymous_variant
rs369758288	non_coding_transcript_variant,coding_sequence_variant,missense_variant
rs369838973	genic_downstream_transcript_variant,non_coding_transcript_variant,coding_sequence_variant,missense_variant
rs370170168	coding_sequence_variant,genic_upstream_transcript_variant,intron_variant,missense_variant,non_coding_transcript_variant
rs370237186	coding_sequence_variant,intron_variant,missense_variant
rs370314484	coding_sequence_variant,genic_upstream_transcript_variant,intron_variant,missense_variant,non_coding_transcript_variant
rs370368651	coding_sequence_variant,synonymous_variant,genic_downstream_transcript_variant,missense_variant,non_coding_transcript_variant
rs370499060	coding_sequence_variant,genic_upstream_transcript_variant,intron_variant,missense_variant,non_coding_transcript_variant
rs370529693	genic_downstream_transcript_variant,non_coding_transcript_variant,coding_sequence_variant,missense_variant
rs370767911	coding_sequence_variant,synonymous_variant,genic_downstream_transcript_variant,missense_variant,non_coding_transcript_variant
rs370833501	intron_variant
rs370883654	non_coding_transcript_variant,coding_sequence_variant,missense_variant
rs371015065	genic_downstream_transcript_variant,non_coding_transcript_variant,coding_sequence_variant,synonymous_variant
rs371124109	non_coding_transcript_variant,coding_sequence_variant,missense_variant
rs371165052	genic_downstream_transcript_variant,non_coding_transcript_variant,coding_sequence_variant,synonymous_variant
rs371379572	genic_downstream_transcript_variant,non_coding_transcript_variant,coding_sequence_variant,synonymous_variant
rs371463233	5_prime_UTR_variant,non_coding_transcript_variant,coding_sequence_variant,missense_variant
rs371939364	non_coding_transcript_variant,coding_sequence_variant,missense_variant
rs372116735	non_coding_transcript_variant,coding_sequence_variant,missense_variant
rs372229471	5_prime_UTR_variant,non_coding_transcript_variant,coding_sequence_variant,synonymous_variant
rs372238373	genic_downstream_transcript_variant,non_coding_transcript_variant,coding_sequence_variant,synonymous_variant
rs372608840	genic_downstream_transcript_variant,non_coding_transcript_variant,coding_sequence_variant,synonymous_variant
rs372830789	genic_downstream_transcript_variant,non_coding_transcript_variant,coding_sequence_variant,missense_variant
rs373030463	non_coding_transcript_variant,coding_sequence_variant,missense_variant
rs373312981	non_coding_transcript_variant,coding_sequence_variant,missense_variant
rs373419003	genic_downstream_transcript_variant,non_coding_transcript_variant,coding_sequence_variant,missense_variant
rs373517956	genic_downstream_transcript_variant,non_coding_transcript_variant,coding_sequence_variant,missense_variant
rs373586966	non_coding_transcript_variant,coding_sequence_variant,synonymous_variant
rs373842614	non_coding_transcript_variant,coding_sequence_variant,synonymous_variant
rs374050750	coding_sequence_variant,genic_upstream_transcript_variant,intron_variant,missense_variant,non_coding_transcript_variant
rs374081963	5_prime_UTR_variant,non_coding_transcript_variant,coding_sequence_variant,synonymous_variant
rs374383028	genic_downstream_transcript_variant,non_coding_transcript_variant,coding_sequence_variant,missense_variant
rs374578653	coding_sequence_variant,genic_upstream_transcript_variant,intron_variant,missense_variant,non_coding_transcript_variant
rs374733251	non_coding_transcript_variant,coding_sequence_variant,missense_variant
rs375353223	coding_sequence_variant,genic_upstream_transcript_variant,intron_variant,missense_variant,non_coding_transcript_variant
rs375391602	5_prime_UTR_variant,non_coding_transcript_variant,coding_sequence_variant,missense_variant
rs375395179	genic_downstream_transcript_variant,non_coding_transcript_variant,coding_sequence_variant,missense_variant
rs375523121	coding_sequence_variant,intron_variant,missense_variant
rs375576430	stop_gained,coding_sequence_variant,genic_downstream_transcript_variant,missense_variant,non_coding_transcript_variant

rs375716972	coding_sequence_variant,genic_upstream_transcript_variant,intron_variant,synonymous_variant,non_coding_transcript_variant
rs375951814	non_coding_transcript_variant,coding_sequence_variant,missense_variant
rs376069508	genic_downstream_transcript_variant,non_coding_transcript_variant,coding_sequence_variant,synonymous_variant
rs376097698	coding_sequence_variant,genic_upstream_transcript_variant,intron_variant,missense_variant,non_coding_transcript_variant
rs376104371	non_coding_transcript_variant,coding_sequence_variant,missense_variant
rs376183825	genic_downstream_transcript_variant,intron_variant
rs376235685	coding_sequence_variant,genic_upstream_transcript_variant,intron_variant,synonymous_variant,non_coding_transcript_variant
rs376243090	genic_downstream_transcript_variant,non_coding_transcript_variant,coding_sequence_variant,missense_variant
rs376244358	coding_sequence_variant,genic_upstream_transcript_variant,intron_variant,missense_variant,non_coding_transcript_variant
rs376364468	5_prime_UTR_variant,non_coding_transcript_variant,coding_sequence_variant,missense_variant
rs376418811	genic_downstream_transcript_variant,non_coding_transcript_variant,coding_sequence_variant,missense_variant
rs376459715	non_coding_transcript_variant,coding_sequence_variant,missense_variant
rs376589619	non_coding_transcript_variant,coding_sequence_variant,missense_variant
rs376966336	coding_sequence_variant,genic_upstream_transcript_variant,intron_variant,synonymous_variant,non_coding_transcript_variant
rs377284387	non_coding_transcript_variant,coding_sequence_variant,synonymous_variant
rs377336878	stop_gained,non_coding_transcript_variant,coding_sequence_variant,synonymous_variant
rs386833405	intron_variant
rs398123567	coding_sequence_variant,genic_upstream_transcript_variant,intron_variant,synonymous_variant,non_coding_transcript_variant
rs398123568	non_coding_transcript_variant,coding_sequence_variant,missense_variant
rs398123569	genic_downstream_transcript_variant,non_coding_transcript_variant,coding_sequence_variant,missense_variant
rs398123570	genic_downstream_transcript_variant,non_coding_transcript_variant,coding_sequence_variant,missense_variant
rs527496385	genic_downstream_transcript_variant,non_coding_transcript_variant,3_prime_UTR_variant
rs527638748	intron_variant,coding_sequence_variant,missense_variant,non_coding_transcript_variant,genic_upstream_transcript_variant
rs529823741	intron_variant
rs530932258	intron_variant,coding_sequence_variant,missense_variant,non_coding_transcript_variant,genic_upstream_transcript_variant,synonymous_variant
rs531456758	genic_downstream_transcript_variant,non_coding_transcript_variant,3_prime_UTR_variant
rs534514626	genic_downstream_transcript_variant,non_coding_transcript_variant,3_prime_UTR_variant
rs534974144	genic_downstream_transcript_variant,non_coding_transcript_variant,coding_sequence_variant,missense_variant
rs536185931	genic_downstream_transcript_variant,non_coding_transcript_variant,3_prime_UTR_variant
rs536603694	5_prime_UTR_variant,intron_variant,upstream_transcript_variant,genic_upstream_transcript_variant
rs538856434	intron_variant,genic_downstream_transcript_variant
rs539345989	coding_sequence_variant,missense_variant,genic_downstream_transcript_variant,non_coding_transcript_variant
rs540354779	synonymous_variant,intron_variant,non_coding_transcript_variant,genic_upstream_transcript_variant,coding_sequence_variant
rs543293293	intron_variant
rs544081614	coding_sequence_variant,synonymous_variant,genic_downstream_transcript_variant,non_coding_transcript_variant
rs544274181	coding_sequence_variant,missense_variant,non_coding_transcript_variant
rs544545520	coding_sequence_variant,synonymous_variant,genic_downstream_transcript_variant,non_coding_transcript_variant
rs545332056	intron_variant,non_coding_transcript_variant,genic_upstream_transcript_variant,coding_sequence_variant,missense_variant
rs549459978	synonymous_variant,intron_variant,non_coding_transcript_variant,coding_sequence_variant,genic_upstream_transcript_variant
rs552870344	synonymous_variant,intron_variant,non_coding_transcript_variant,coding_sequence_variant,genic_upstream_transcript_variant
rs553431706	intron_variant
rs553602374	3_prime_UTR_variant,genic_downstream_transcript_variant,non_coding_transcript_variant
rs554029534	3_prime_UTR_variant,genic_downstream_transcript_variant,non_coding_transcript_variant
rs554190225	synonymous_variant,intron_variant,non_coding_transcript_variant,coding_sequence_variant,genic_upstream_transcript_variant
rs555099334	genic_downstream_transcript_variant,synonymous_variant,coding_sequence_variant,non_coding_transcript_variant
rs555920594	genic_upstream_transcript_variant,missense_variant,intron_variant,coding_sequence_variant,non_coding_transcript_variant
rs559231345	genic_downstream_transcript_variant,3_prime_UTR_variant,non_coding_transcript_variant
rs560568639	genic_downstream_transcript_variant,3_prime_UTR_variant,non_coding_transcript_variant
rs561131509	coding_sequence_variant,non_coding_transcript_variant,synonymous_variant
rs561295443	genic_upstream_transcript_variant,missense_variant,intron_variant,coding_sequence_variant,non_coding_transcript_variant
rs561588772	missense_variant,coding_sequence_variant,non_coding_transcript_variant
rs563978117	genic_downstream_transcript_variant,3_prime_UTR_variant,non_coding_transcript_variant
rs565768785	genic_downstream_transcript_variant,3_prime_UTR_variant,non_coding_transcript_variant
rs565938550	non_coding_transcript_variant,missense_variant,genic_downstream_transcript_variant,coding_sequence_variant
rs566540058	non_coding_transcript_variant,coding_sequence_variant,synonymous_variant
rs567551556	non_coding_transcript_variant,genic_downstream_transcript_variant,coding_sequence_variant,synonymous_variant
rs570036369	synonymous_variant,non_coding_transcript_variant,genic_upstream_transcript_variant,coding_sequence_variant,intron_variant
rs573510587	genic_downstream_transcript_variant,3_prime_UTR_variant,non_coding_transcript_variant
rs574911056	non_coding_transcript_variant,intron_variant,genic_upstream_transcript_variant,synonymous_variant,coding_sequence_variant
rs575907920	missense_variant,non_coding_transcript_variant,genic_downstream_transcript_variant,coding_sequence_variant
rs576502224	non_coding_transcript_variant,intron_variant,genic_upstream_transcript_variant,synonymous_variant,coding_sequence_variant
rs587777950	intron_variant
rs587777951	intron_variant
rs587777952	intron_variant
rs587778441	missense_variant,non_coding_transcript_variant,genic_downstream_transcript_variant,coding_sequence_variant
rs587778442	non_coding_transcript_variant,intron_variant,genic_upstream_transcript_variant,missense_variant,coding_sequence_variant
rs587778443	non_coding_transcript_variant,intron_variant,genic_upstream_transcript_variant,missense_variant,coding_sequence_variant
rs587778444	missense_variant,non_coding_transcript_variant,genic_downstream_transcript_variant,coding_sequence_variant
rs587778445	missense_variant,non_coding_transcript_variant,coding_sequence_variant
rs587778446	missense_variant,non_coding_transcript_variant,coding_sequence_variant
rs587780532	synonymous_variant,non_coding_transcript_variant,genic_downstream_transcript_variant,coding_sequence_variant
rs587780540	missense_variant,intron_variant,stop_gained,coding_sequence_variant
rs587780548	non_coding_transcript_variant,intron_variant,genic_upstream_transcript_variant,missense_variant,coding_sequence_variant
rs587780733	missense_variant,non_coding_transcript_variant,coding_sequence_variant

rs587780734	missense_variant,non_coding_transcript_variant,coding_sequence_variant
rs587780735	missense_variant,non_coding_transcript_variant,coding_sequence_variant
rs587780736	intron_variant
rs587780737	non_coding_transcript_variant,intron_variant,genic_upstream_transcript_variant,synonymous_variant,coding_sequence_variant
rs587780738	non_coding_transcript_variant,intron_variant,genic_upstream_transcript_variant,synonymous_variant,coding_sequence_variant
rs587780739	non_coding_transcript_variant,intron_variant,genic_upstream_transcript_variant,missense_variant,coding_sequence_variant
rs587780740	non_coding_transcript_variant,intron_variant,genic_upstream_transcript_variant,missense_variant,coding_sequence_variant
rs587782086	non_coding_transcript_variant,intron_variant,genic_upstream_transcript_variant,missense_variant,coding_sequence_variant
rs587782205	missense_variant,non_coding_transcript_variant,coding_sequence_variant
rs587782807	non_coding_transcript_variant,intron_variant,genic_upstream_transcript_variant,missense_variant,coding_sequence_variant
rs727504014	missense_variant,non_coding_transcript_variant,genic_downstream_transcript_variant,coding_sequence_variant
rs745345346	missense_variant,non_coding_transcript_variant,coding_sequence_variant
rs745437003	missense_variant,non_coding_transcript_variant,genic_downstream_transcript_variant,coding_sequence_variant
rs745479104	missense_variant,non_coding_transcript_variant,coding_sequence_variant
rs745547523	intron_variant
rs745740862	non_coding_transcript_variant,intron_variant,genic_upstream_transcript_variant,missense_variant,coding_sequence_variant
rs746040712	synonymous_variant,non_coding_transcript_variant,coding_sequence_variant
rs746216803	non_coding_transcript_variant,intron_variant,genic_upstream_transcript_variant,missense_variant,coding_sequence_variant
rs746806941	non_coding_transcript_variant,intron_variant,genic_upstream_transcript_variant,missense_variant,coding_sequence_variant
rs746880479	genic_upstream_transcript_variant,intron_variant
rs747239403	missense_variant,non_coding_transcript_variant,genic_downstream_transcript_variant,coding_sequence_variant
rs747390610	splice_acceptor_variant
rs747777018	non_coding_transcript_variant,intron_variant,genic_upstream_transcript_variant,missense_variant,coding_sequence_variant
rs747803111	synonymous_variant,non_coding_transcript_variant,coding_sequence_variant
rs747811080	synonymous_variant,non_coding_transcript_variant,genic_downstream_transcript_variant,coding_sequence_variant
rs747850853	3_prime_UTR_variant,non_coding_transcript_variant,genic_downstream_transcript_variant
rs747940150	non_coding_transcript_variant,intron_variant,genic_upstream_transcript_variant,synonymous_variant,coding_sequence_variant
rs747989932	missense_variant,non_coding_transcript_variant,coding_sequence_variant
rs748086754	missense_variant,intron_variant,coding_sequence_variant
rs748382550	missense_variant,non_coding_transcript_variant,genic_downstream_transcript_variant,coding_sequence_variant
rs748508881	synonymous_variant,5_prime_UTR_variant,non_coding_transcript_variant,coding_sequence_variant
rs748950533	missense_variant,synonymous_variant,non_coding_transcript_variant,coding_sequence_variant
rs749081367	intron_variant
rs749126070	missense_variant,intron_variant,coding_sequence_variant
rs749383450	non_coding_transcript_variant,intron_variant,genic_upstream_transcript_variant,synonymous_variant,missense_variant,coding_sequence_variant
rs749484691	missense_variant,synonymous_variant,non_coding_transcript_variant,coding_sequence_variant
rs749678495	coding_sequence_variant,missense_variant,genic_downstream_transcript_variant,non_coding_transcript_variant
rs749728359	missense_variant,genic_upstream_transcript_variant,intron_variant,non_coding_transcript_variant,coding_sequence_variant
rs749730267	coding_sequence_variant,missense_variant,non_coding_transcript_variant
rs749738523	missense_variant,genic_upstream_transcript_variant,intron_variant,non_coding_transcript_variant,coding_sequence_variant
rs750004615	coding_sequence_variant,intron_variant,missense_variant
rs750102016	missense_variant,genic_upstream_transcript_variant,intron_variant,non_coding_transcript_variant,coding_sequence_variant
rs750256779	coding_sequence_variant,missense_variant,non_coding_transcript_variant
rs750268644	intron_variant
rs750337451	coding_sequence_variant,missense_variant,genic_downstream_transcript_variant,non_coding_transcript_variant
rs750531377	missense_variant,genic_upstream_transcript_variant,intron_variant,non_coding_transcript_variant,coding_sequence_variant
rs750584257	coding_sequence_variant,non_coding_transcript_variant,synonymous_variant
rs750586651	genic_upstream_transcript_variant,synonymous_variant,intron_variant,non_coding_transcript_variant,coding_sequence_variant
rs750893461	3_prime_UTR_variant,genic_downstream_transcript_variant,non_coding_transcript_variant
rs751158831	missense_variant,genic_upstream_transcript_variant,synonymous_variant,intron_variant,non_coding_transcript_variant,coding_sequence_variant
rs751158986	coding_sequence_variant,missense_variant,non_coding_transcript_variant
rs751186512	coding_sequence_variant,missense_variant,genic_downstream_transcript_variant,non_coding_transcript_variant
rs751307227	coding_sequence_variant,missense_variant,non_coding_transcript_variant
rs751556706	coding_sequence_variant,missense_variant,genic_downstream_transcript_variant,non_coding_transcript_variant
rs751564290	missense_variant,genic_upstream_transcript_variant,intron_variant,non_coding_transcript_variant,coding_sequence_variant
rs751961995	coding_sequence_variant,missense_variant,genic_downstream_transcript_variant,non_coding_transcript_variant
rs752013993	coding_sequence_variant,missense_variant,non_coding_transcript_variant,synonymous_variant
rs752109953	coding_sequence_variant,missense_variant,genic_downstream_transcript_variant,non_coding_transcript_variant
rs752226182	missense_variant,genic_upstream_transcript_variant,synonymous_variant,intron_variant,non_coding_transcript_variant,coding_sequence_variant
rs752292538	intron_variant,genic_downstream_transcript_variant
rs752351789	coding_sequence_variant,missense_variant,genic_downstream_transcript_variant,non_coding_transcript_variant
rs752641437	coding_sequence_variant,missense_variant,genic_downstream_transcript_variant,non_coding_transcript_variant
rs752669237	coding_sequence_variant,missense_variant,genic_downstream_transcript_variant,non_coding_transcript_variant
rs752694306	coding_sequence_variant,missense_variant,genic_downstream_transcript_variant,non_coding_transcript_variant
rs752791731	coding_sequence_variant,missense_variant,genic_downstream_transcript_variant,non_coding_transcript_variant
rs752842662	coding_sequence_variant,missense_variant,non_coding_transcript_variant
rs753101831	coding_sequence_variant,genic_downstream_transcript_variant,non_coding_transcript_variant,synonymous_variant
rs753407699	coding_sequence_variant,missense_variant,non_coding_transcript_variant
rs753697730	missense_variant,genic_upstream_transcript_variant,intron_variant,non_coding_transcript_variant,coding_sequence_variant
rs753740913	intron_variant
rs753757242	coding_sequence_variant,genic_downstream_transcript_variant,non_coding_transcript_variant,synonymous_variant
rs753958204	coding_sequence_variant,missense_variant,non_coding_transcript_variant
rs754228039	coding_sequence_variant,missense_variant,non_coding_transcript_variant
rs754467674	coding_sequence_variant,genic_downstream_transcript_variant,non_coding_transcript_variant,synonymous_variant

rs754635450	missense_variant,genic_upstream_transcript_variant,intron_variant,non_coding_transcript_variant,coding_sequence_variant
rs754686930	intron_variant
rs754909964	coding_sequence_variant,genic_downstream_transcript_variant,non_coding_transcript_variant,synonymous_variant
rs754969545	coding_sequence_variant,missense_variant,non_coding_transcript_variant
rs755043272	coding_sequence_variant,missense_variant,genic_downstream_transcript_variant,non_coding_transcript_variant
rs755139610	coding_sequence_variant,missense_variant,non_coding_transcript_variant
rs755165691	coding_sequence_variant,non_coding_transcript_variant,synonymous_variant
rs755166920	genic_upstream_transcript_variant,synonymous_variant,intron_variant,non_coding_transcript_variant,coding_sequence_variant
rs755234697	coding_sequence_variant,missense_variant,genic_downstream_transcript_variant,non_coding_transcript_variant
rs755500683	coding_sequence_variant,genic_downstream_transcript_variant,non_coding_transcript_variant,synonymous_variant
rs755571526	coding_sequence_variant,missense_variant,non_coding_transcript_variant
rs755804127	genic_upstream_transcript_variant,synonymous_variant,intron_variant,non_coding_transcript_variant,coding_sequence_variant
rs755954919	missense_variant,genic_upstream_transcript_variant,intron_variant,non_coding_transcript_variant,coding_sequence_variant
rs755985210	coding_sequence_variant,non_coding_transcript_variant,synonymous_variant
rs756031094	coding_sequence_variant,missense_variant,genic_downstream_transcript_variant,non_coding_transcript_variant
rs756154521	coding_sequence_variant,missense_variant,non_coding_transcript_variant,synonymous_variant
rs756526057	coding_sequence_variant,missense_variant,non_coding_transcript_variant
rs756670611	coding_sequence_variant,missense_variant,genic_downstream_transcript_variant,non_coding_transcript_variant
rs757012119	coding_sequence_variant,genic_downstream_transcript_variant,non_coding_transcript_variant,synonymous_variant
rs757338659	coding_sequence_variant,genic_downstream_transcript_variant,non_coding_transcript_variant,synonymous_variant
rs757402499	missense_variant,genic_upstream_transcript_variant,intron_variant,non_coding_transcript_variant,coding_sequence_variant
rs757427533	missense_variant,genic_upstream_transcript_variant,intron_variant,non_coding_transcript_variant,coding_sequence_variant
rs757539632	coding_sequence_variant,missense_variant,genic_downstream_transcript_variant,non_coding_transcript_variant
rs757561412	intron_variant
rs757846126	genic_upstream_transcript_variant,intron_variant,non_coding_transcript_variant,coding_sequence_variant,stop_gained
rs757859772	3_prime_UTR_variant,genic_downstream_transcript_variant,non_coding_transcript_variant
rs757883355	missense_variant,genic_upstream_transcript_variant,intron_variant,non_coding_transcript_variant,coding_sequence_variant
rs757925979	coding_sequence_variant,missense_variant,genic_downstream_transcript_variant,non_coding_transcript_variant
rs758016814	missense_variant,genic_upstream_transcript_variant,intron_variant,non_coding_transcript_variant,coding_sequence_variant
rs758292223	coding_sequence_variant,missense_variant,genic_downstream_transcript_variant,non_coding_transcript_variant
rs758440368	coding_sequence_variant,missense_variant,genic_downstream_transcript_variant,non_coding_transcript_variant
rs758486336	coding_sequence_variant,missense_variant,genic_downstream_transcript_variant,non_coding_transcript_variant
rs758486777	coding_sequence_variant,missense_variant,genic_downstream_transcript_variant,non_coding_transcript_variant
rs758564871	missense_variant,genic_upstream_transcript_variant,intron_variant,non_coding_transcript_variant,coding_sequence_variant
rs758569834	missense_variant,genic_upstream_transcript_variant,intron_variant,non_coding_transcript_variant,coding_sequence_variant
rs758738756	non_coding_transcript_variant,genic_downstream_transcript_variant,coding_sequence_variant,missense_variant
rs758919662	genic_upstream_transcript_variant,intron_variant,non_coding_transcript_variant,coding_sequence_variant,missense_variant
rs759007523	intron_variant
rs759227622	genic_upstream_transcript_variant,intron_variant,non_coding_transcript_variant,coding_sequence_variant,missense_variant
rs759388741	genic_upstream_transcript_variant,intron_variant,non_coding_transcript_variant,coding_sequence_variant,missense_variant
rs759396158	non_coding_transcript_variant,synonymous_variant,coding_sequence_variant
rs759522148	non_coding_transcript_variant,genic_downstream_transcript_variant,coding_sequence_variant,missense_variant
rs760106468	genic_upstream_transcript_variant,intron_variant,non_coding_transcript_variant,coding_sequence_variant,missense_variant
rs760256871	non_coding_transcript_variant,synonymous_variant,coding_sequence_variant
rs760278126	genic_upstream_transcript_variant,intron_variant,synonymous_variant,non_coding_transcript_variant,coding_sequence_variant,missense_variant
rs760285693	non_coding_transcript_variant,genic_downstream_transcript_variant,coding_sequence_variant,missense_variant
rs760294359	non_coding_transcript_variant,synonymous_variant,coding_sequence_variant,missense_variant
rs760722037	non_coding_transcript_variant,synonymous_variant,coding_sequence_variant
rs761183186	non_coding_transcript_variant,coding_sequence_variant,missense_variant
rs761221409	genic_upstream_transcript_variant,intron_variant,non_coding_transcript_variant,coding_sequence_variant,missense_variant
rs761243391	non_coding_transcript_variant,coding_sequence_variant,missense_variant
rs761254993	5_prime_UTR_variant,non_coding_transcript_variant,coding_sequence_variant,missense_variant
rs761522694	non_coding_transcript_variant,genic_downstream_transcript_variant,coding_sequence_variant,missense_variant
rs761768345	genic_upstream_transcript_variant,intron_variant,non_coding_transcript_variant,coding_sequence_variant,missense_variant
rs761808213	non_coding_transcript_variant,genic_downstream_transcript_variant,coding_sequence_variant,missense_variant
rs761951444	non_coding_transcript_variant,coding_sequence_variant,missense_variant
rs762228405	non_coding_transcript_variant,coding_sequence_variant,missense_variant
rs762306750	intron_variant
rs762520351	non_coding_transcript_variant,synonymous_variant,coding_sequence_variant
rs762863861	non_coding_transcript_variant,coding_sequence_variant,missense_variant
rs762885066	genic_upstream_transcript_variant,intron_variant,synonymous_variant,non_coding_transcript_variant,coding_sequence_variant
rs762898756	non_coding_transcript_variant,synonymous_variant,coding_sequence_variant,missense_variant
rs763131600	non_coding_transcript_variant,genic_downstream_transcript_variant,synonymous_variant,coding_sequence_variant
rs763196530	genic_upstream_transcript_variant,intron_variant,synonymous_variant,non_coding_transcript_variant,coding_sequence_variant
rs763246917	intron_variant,genic_downstream_transcript_variant
rs763344951	genic_upstream_transcript_variant,intron_variant,non_coding_transcript_variant,coding_sequence_variant,missense_variant
rs763398740	non_coding_transcript_variant,genic_downstream_transcript_variant,coding_sequence_variant,missense_variant
rs763648816	intron_variant,genic_downstream_transcript_variant
rs763726060	non_coding_transcript_variant,synonymous_variant,coding_sequence_variant
rs763849125	non_coding_transcript_variant,coding_sequence_variant,missense_variant
rs763935791	genic_upstream_transcript_variant,intron_variant,non_coding_transcript_variant,coding_sequence_variant,missense_variant
rs763948315	intron_variant
rs763991073	non_coding_transcript_variant,coding_sequence_variant,missense_variant
rs764052874	genic_upstream_transcript_variant,intron_variant,non_coding_transcript_variant,coding_sequence_variant,missense_variant

rs764213589	non_coding_transcript_variant,genic_downstream_transcript_variant,coding_sequence_variant,missense_variant
rs764246939	genic_upstream_transcript_variant,intron_variant,stop_gained,non_coding_transcript_variant,coding_sequence_variant,missense_variant
rs764303128	non_coding_transcript_variant,genic_downstream_transcript_variant,coding_sequence_variant,missense_variant
rs764580322	5_prime_UTR_variant,non_coding_transcript_variant,coding_sequence_variant,missense_variant
rs764685570	genic_upstream_transcript_variant,intron_variant,synonymous_variant,non_coding_transcript_variant,coding_sequence_variant
rs764822445	non_coding_transcript_variant,genic_downstream_transcript_variant,synonymous_variant,coding_sequence_variant
rs764929802	non_coding_transcript_variant,genic_downstream_transcript_variant,3_prime_UTR_variant
rs764947837	non_coding_transcript_variant,genic_downstream_transcript_variant,coding_sequence_variant,missense_variant
rs764960693	non_coding_transcript_variant,coding_sequence_variant,missense_variant
rs765061513	genic_upstream_transcript_variant,intron_variant,non_coding_transcript_variant,coding_sequence_variant,missense_variant
rs765244598	non_coding_transcript_variant,coding_sequence_variant,missense_variant
rs765257726	genic_upstream_transcript_variant,intron_variant,non_coding_transcript_variant,coding_sequence_variant,missense_variant
rs765318603	non_coding_transcript_variant,synonymous_variant,coding_sequence_variant
rs765332671	non_coding_transcript_variant,genic_downstream_transcript_variant,coding_sequence_variant,missense_variant
rs765441927	non_coding_transcript_variant,coding_sequence_variant,missense_variant
rs765444467	genic_upstream_transcript_variant,intron_variant,non_coding_transcript_variant,coding_sequence_variant,missense_variant
rs765565377	non_coding_transcript_variant,genic_downstream_transcript_variant,synonymous_variant,coding_sequence_variant
rs765759353	genic_upstream_transcript_variant,intron_variant,non_coding_transcript_variant,coding_sequence_variant,missense_variant
rs766190061	non_coding_transcript_variant,genic_downstream_transcript_variant,coding_sequence_variant,missense_variant
rs766209435	non_coding_transcript_variant,coding_sequence_variant,missense_variant
rs766401668	non_coding_transcript_variant,synonymous_variant,coding_sequence_variant
rs766430458	non_coding_transcript_variant,coding_sequence_variant,missense_variant
rs766489685	non_coding_transcript_variant,genic_downstream_transcript_variant,coding_sequence_variant,missense_variant
rs766575983	intron_variant
rs766900241	intron_variant
rs766929091	non_coding_transcript_variant,initiator_codon_variant,coding_sequence_variant,missense_variant
rs767214353	stop_gained,synonymous_variant,non_coding_transcript_variant,genic_downstream_transcript_variant,coding_sequence_variant
rs767587065	non_coding_transcript_variant,genic_downstream_transcript_variant,coding_sequence_variant,missense_variant
rs767715328	missense_variant,non_coding_transcript_variant,stop_gained,coding_sequence_variant
rs767768257	intron_variant
rs767934760	non_coding_transcript_variant,genic_upstream_transcript_variant,coding_sequence_variant,missense_variant,synonymous_variant,intron_variant
rs768125521	genic_downstream_transcript_variant,non_coding_transcript_variant,missense_variant,coding_sequence_variant
rs768188910	genic_downstream_transcript_variant,non_coding_transcript_variant,missense_variant,coding_sequence_variant
rs768627036	genic_downstream_transcript_variant,synonymous_variant,non_coding_transcript_variant,coding_sequence_variant
rs768640780	non_coding_transcript_variant,genic_upstream_transcript_variant,coding_sequence_variant,missense_variant,intron_variant
rs768675699	non_coding_transcript_variant,genic_upstream_transcript_variant,coding_sequence_variant,missense_variant,intron_variant
rs768678989	genic_downstream_transcript_variant,non_coding_transcript_variant,missense_variant,coding_sequence_variant
rs769035394	synonymous_variant,non_coding_transcript_variant,coding_sequence_variant
rs769073123	missense_variant,non_coding_transcript_variant,coding_sequence_variant
rs769128478	non_coding_transcript_variant,genic_upstream_transcript_variant,coding_sequence_variant,missense_variant,intron_variant
rs769181927	non_coding_transcript_variant,genic_upstream_transcript_variant,coding_sequence_variant,missense_variant,intron_variant
rs769247886	intron_variant
rs769253547	missense_variant,non_coding_transcript_variant,coding_sequence_variant
rs769544894	non_coding_transcript_variant,genic_upstream_transcript_variant,coding_sequence_variant,missense_variant,intron_variant
rs769592729	missense_variant,non_coding_transcript_variant,coding_sequence_variant
rs769647929	intron_variant
rs770202229	non_coding_transcript_variant,genic_upstream_transcript_variant,coding_sequence_variant,synonymous_variant,intron_variant
rs770257335	non_coding_transcript_variant,genic_upstream_transcript_variant,coding_sequence_variant,synonymous_variant,intron_variant
rs770341307	non_coding_transcript_variant,genic_upstream_transcript_variant,coding_sequence_variant,missense_variant,intron_variant
rs770829669	non_coding_transcript_variant,genic_upstream_transcript_variant,coding_sequence_variant,synonymous_variant,intron_variant
rs770886012	genic_downstream_transcript_variant,synonymous_variant,non_coding_transcript_variant,coding_sequence_variant
rs770910579	genic_downstream_transcript_variant,non_coding_transcript_variant,3_prime_UTR_variant
rs771146873	non_coding_transcript_variant,genic_upstream_transcript_variant,coding_sequence_variant,missense_variant,intron_variant
rs771231484	missense_variant,intron_variant,coding_sequence_variant
rs771272439	missense_variant,non_coding_transcript_variant,coding_sequence_variant
rs771328219	genic_downstream_transcript_variant,non_coding_transcript_variant,missense_variant,coding_sequence_variant
rs771333219	missense_variant,intron_variant,coding_sequence_variant
rs771518812	genic_downstream_transcript_variant,non_coding_transcript_variant,3_prime_UTR_variant
rs771976817	missense_variant,intron_variant,coding_sequence_variant
rs772014416	non_coding_transcript_variant,genic_upstream_transcript_variant,coding_sequence_variant,missense_variant,synonymous_variant,intron_variant
rs772251895	non_coding_transcript_variant,genic_upstream_transcript_variant,coding_sequence_variant,synonymous_variant,intron_variant
rs772398152	non_coding_transcript_variant,genic_upstream_transcript_variant,coding_sequence_variant,missense_variant,intron_variant
rs772450441	synonymous_variant,non_coding_transcript_variant,coding_sequence_variant
rs772860611	genic_downstream_transcript_variant,synonymous_variant,non_coding_transcript_variant,coding_sequence_variant
rs773018125	non_coding_transcript_variant,genic_upstream_transcript_variant,coding_sequence_variant,missense_variant,intron_variant
rs773200558	non_coding_transcript_variant,genic_upstream_transcript_variant,coding_sequence_variant,missense_variant,intron_variant
rs773447798	genic_downstream_transcript_variant,non_coding_transcript_variant,missense_variant,coding_sequence_variant
rs773626056	missense_variant,non_coding_transcript_variant,5_prime_UTR_variant,coding_sequence_variant
rs773659883	non_coding_transcript_variant,genic_upstream_transcript_variant,coding_sequence_variant,missense_variant,intron_variant
rs773763852	non_coding_transcript_variant,coding_sequence_variant,genic_downstream_transcript_variant,synonymous_variant,missense_variant
rs773772398	genic_downstream_transcript_variant,non_coding_transcript_variant,missense_variant,coding_sequence_variant
rs773826297	missense_variant,non_coding_transcript_variant,coding_sequence_variant
rs773830746	non_coding_transcript_variant,genic_upstream_transcript_variant,coding_sequence_variant,missense_variant,intron_variant
rs773835391	intron_variant

rs773898036	genic_downstream_transcript_variant,non_coding_transcript_variant,missense_variant,coding_sequence_variant
rs774022115	non_coding_transcript_variant,genic_upstream_transcript_variant,coding_sequence_variant,missense_variant,intron_variant
rs774038166	non_coding_transcript_variant,genic_upstream_transcript_variant,coding_sequence_variant,synonymous_variant,intron_variant
rs774146015	non_coding_transcript_variant,genic_upstream_transcript_variant,coding_sequence_variant,missense_variant,intron_variant
rs774334135	genic_downstream_transcript_variant,synonymous_variant,non_coding_transcript_variant,coding_sequence_variant
rs774433287	genic_downstream_transcript_variant,non_coding_transcript_variant,missense_variant,coding_sequence_variant
rs774439479	synonymous_variant,non_coding_transcript_variant,coding_sequence_variant
rs774462373	missense_variant,non_coding_transcript_variant,coding_sequence_variant
rs774945178	missense_variant,non_coding_transcript_variant,coding_sequence_variant
rs775439897	non_coding_transcript_variant,genic_upstream_transcript_variant,coding_sequence_variant,missense_variant,intron_variant
rs775965879	missense_variant,non_coding_transcript_variant,coding_sequence_variant
rs775997318	genic_downstream_transcript_variant,non_coding_transcript_variant,missense_variant,coding_sequence_variant
rs776014448	non_coding_transcript_variant,genic_upstream_transcript_variant,coding_sequence_variant,missense_variant,intron_variant
rs776086430	genic_downstream_transcript_variant,non_coding_transcript_variant,missense_variant,coding_sequence_variant
rs776407390	non_coding_transcript_variant,genic_upstream_transcript_variant,coding_sequence_variant,missense_variant,intron_variant
rs776490266	synonymous_variant,non_coding_transcript_variant,coding_sequence_variant
rs776693512	non_coding_transcript_variant,genic_upstream_transcript_variant,coding_sequence_variant,missense_variant,synonymous_variant,intron_variant
rs776805376	coding_sequence_variant,non_coding_transcript_variant,missense_variant
rs777336138	intron_variant,missense_variant,non_coding_transcript_variant,genic_upstream_transcript_variant,coding_sequence_variant
rs777748634	coding_sequence_variant,genic_downstream_transcript_variant,non_coding_transcript_variant,missense_variant
rs778084120	coding_sequence_variant,genic_downstream_transcript_variant,non_coding_transcript_variant,missense_variant
rs778115147	coding_sequence_variant,non_coding_transcript_variant,missense_variant
rs778469367	intron_variant,missense_variant,non_coding_transcript_variant,genic_upstream_transcript_variant,coding_sequence_variant
rs778646229	coding_sequence_variant,genic_downstream_transcript_variant,non_coding_transcript_variant,missense_variant
rs779022887	coding_sequence_variant,non_coding_transcript_variant,missense_variant
rs779040487	intron_variant,missense_variant,non_coding_transcript_variant,genic_upstream_transcript_variant,coding_sequence_variant
rs779091695	synonymous_variant,coding_sequence_variant,non_coding_transcript_variant
rs779093896	intron_variant,non_coding_transcript_variant,genic_upstream_transcript_variant,stop_gained,coding_sequence_variant
rs779121848	coding_sequence_variant,genic_downstream_transcript_variant,non_coding_transcript_variant,missense_variant
rs779512046	intron_variant,non_coding_transcript_variant,genic_upstream_transcript_variant,synonymous_variant,coding_sequence_variant
rs779636840	coding_sequence_variant,genic_downstream_transcript_variant,non_coding_transcript_variant,missense_variant
rs779724665	intron_variant,missense_variant,non_coding_transcript_variant,genic_upstream_transcript_variant,coding_sequence_variant
rs779897466	intron_variant,missense_variant,non_coding_transcript_variant,genic_upstream_transcript_variant,coding_sequence_variant
rs779906308	coding_sequence_variant,genic_downstream_transcript_variant,non_coding_transcript_variant,missense_variant
rs780077898	coding_sequence_variant,non_coding_transcript_variant,missense_variant
rs780102059	coding_sequence_variant,genic_downstream_transcript_variant,non_coding_transcript_variant,missense_variant
rs780230492	intron_variant
rs780293990	coding_sequence_variant,non_coding_transcript_variant,missense_variant
rs780358093	intron_variant,missense_variant,non_coding_transcript_variant,genic_upstream_transcript_variant,coding_sequence_variant
rs780431412	coding_sequence_variant,genic_downstream_transcript_variant,non_coding_transcript_variant,missense_variant
rs780538636	coding_sequence_variant,non_coding_transcript_variant,missense_variant
rs780802614	intron_variant,missense_variant,non_coding_transcript_variant,genic_upstream_transcript_variant,coding_sequence_variant
rs781110968	coding_sequence_variant,non_coding_transcript_variant,missense_variant
rs781257386	intron_variant,missense_variant,non_coding_transcript_variant,genic_upstream_transcript_variant,synonymous_variant,coding_sequence_variant
rs781452657	coding_sequence_variant,non_coding_transcript_variant,missense_variant
rs781545528	coding_sequence_variant,non_coding_transcript_variant,missense_variant
rs781777052	intron_variant,missense_variant,non_coding_transcript_variant,genic_upstream_transcript_variant,coding_sequence_variant
rs786201612	missense_variant,non_coding_transcript_variant,genic_downstream_transcript_variant,synonymous_variant,coding_sequence_variant
rs786202191	intron_variant,coding_sequence_variant,missense_variant
rs786202310	intron_variant,missense_variant,non_coding_transcript_variant,genic_upstream_transcript_variant,coding_sequence_variant
rs786202724	coding_sequence_variant,genic_downstream_transcript_variant,non_coding_transcript_variant,missense_variant
rs786202727	intron_variant,missense_variant,non_coding_transcript_variant,genic_upstream_transcript_variant,coding_sequence_variant
rs786203065	intron_variant,missense_variant,non_coding_transcript_variant,genic_upstream_transcript_variant,coding_sequence_variant
rs786203471	intron_variant,non_coding_transcript_variant,genic_upstream_transcript_variant,synonymous_variant,coding_sequence_variant
rs786203672	coding_sequence_variant,non_coding_transcript_variant,missense_variant
rs786204044	coding_sequence_variant,genic_downstream_transcript_variant,non_coding_transcript_variant,missense_variant
rs786204102	coding_sequence_variant,genic_downstream_transcript_variant,non_coding_transcript_variant,missense_variant
rs786204142	intron_variant,coding_sequence_variant,missense_variant
rs794728016	coding_sequence_variant,non_coding_transcript_variant,missense_variant
rs863224376	intron_variant,non_coding_transcript_variant,5_prime_UTR_variant,genic_upstream_transcript_variant,synonymous_variant,coding_sequence_varia
rs863224377	intron_variant,synonymous_variant,coding_sequence_variant
rs863224378	synonymous_variant,coding_sequence_variant,non_coding_transcript_variant
rs863224379	intron_variant,genic_downstream_transcript_variant
rs863224380	intron_variant,non_coding_transcript_variant,genic_upstream_transcript_variant,synonymous_variant,coding_sequence_variant
rs863224692	intron_variant,missense_variant,non_coding_transcript_variant,genic_upstream_transcript_variant,coding_sequence_variant
rs863224693	intron_variant,missense_variant,non_coding_transcript_variant,genic_upstream_transcript_variant,coding_sequence_variant
rs863224694	coding_sequence_variant,non_coding_transcript_variant,missense_variant
rs863224695	intron_variant,missense_variant,non_coding_transcript_variant,genic_upstream_transcript_variant,coding_sequence_variant
rs863224696	intron_variant,missense_variant,non_coding_transcript_variant,genic_upstream_transcript_variant,synonymous_variant,coding_sequence_variant
rs864622222	intron_variant,non_coding_transcript_variant,genic_upstream_transcript_variant,synonymous_variant,coding_sequence_variant
rs864622521	intron_variant,genic_downstream_transcript_variant
rs866183824	intron_variant,missense_variant,non_coding_transcript_variant,genic_upstream_transcript_variant,coding_sequence_variant
rs866306366	synonymous_variant,coding_sequence_variant,non_coding_transcript_variant
rs866509744	5_prime_UTR_variant,intron_variant,genic_upstream_transcript_variant,non_coding_transcript_variant

rs868554436	3_prime_UTR_variant,genic_downstream_transcript_variant,non_coding_transcript_variant
rs869320706	genic_downstream_transcript_variant,coding_sequence_variant,intron_variant,non_coding_transcript_variant,splice_donor_variant
rs869320707	genic_downstream_transcript_variant,splice_donor_variant
rs879254328	synonymous_variant,coding_sequence_variant,genic_downstream_transcript_variant,non_coding_transcript_variant
rs879254330	genic_upstream_transcript_variant,missense_variant,coding_sequence_variant,intron_variant,non_coding_transcript_variant
rs879254331	genic_upstream_transcript_variant,intron_variant
rs879254332	coding_sequence_variant,missense_variant,non_coding_transcript_variant
rs879254333	coding_sequence_variant,missense_variant,non_coding_transcript_variant
rs879254334	coding_sequence_variant,intron_variant,missense_variant
rs879254335	coding_sequence_variant,genic_downstream_transcript_variant,missense_variant,non_coding_transcript_variant
rs879254336	synonymous_variant,genic_downstream_transcript_variant,missense_variant,stop_gained,coding_sequence_variant,non_coding_transcript_variant
rs879254337	genic_downstream_transcript_variant,splice_acceptor_variant
rs879254338	coding_sequence_variant,genic_downstream_transcript_variant,missense_variant,non_coding_transcript_variant
rs879254339	genic_upstream_transcript_variant,missense_variant,coding_sequence_variant,intron_variant,non_coding_transcript_variant
rs879254340	synonymous_variant,coding_sequence_variant,genic_downstream_transcript_variant,non_coding_transcript_variant
rs879254341	synonymous_variant,genic_upstream_transcript_variant,missense_variant,coding_sequence_variant,intron_variant,non_coding_transcript_variant
rs879254342	genic_upstream_transcript_variant,missense_variant,coding_sequence_variant,intron_variant,non_coding_transcript_variant
rs879254343	genic_upstream_transcript_variant,missense_variant,coding_sequence_variant,intron_variant,non_coding_transcript_variant
rs886038413	synonymous_variant,coding_sequence_variant,genic_downstream_transcript_variant,non_coding_transcript_variant
rs886042262	coding_sequence_variant,genic_downstream_transcript_variant,missense_variant,non_coding_transcript_variant
rs886061938	intron_variant,genic_upstream_transcript_variant,non_coding_transcript_variant,5_prime_UTR_variant
rs886061939	intron_variant,genic_upstream_transcript_variant,non_coding_transcript_variant,5_prime_UTR_variant
rs886061940	intron_variant,genic_upstream_transcript_variant,non_coding_transcript_variant,5_prime_UTR_variant
rs886061941	intron_variant,genic_upstream_transcript_variant,non_coding_transcript_variant,5_prime_UTR_variant
rs886061942	synonymous_variant,genic_upstream_transcript_variant,coding_sequence_variant,intron_variant,non_coding_transcript_variant
rs886061943	genic_upstream_transcript_variant,missense_variant,coding_sequence_variant,intron_variant,non_coding_transcript_variant
rs886061945	3_prime_UTR_variant,genic_downstream_transcript_variant,non_coding_transcript_variant
rs886061946	3_prime_UTR_variant,genic_downstream_transcript_variant,non_coding_transcript_variant
rs886061947	3_prime_UTR_variant,genic_downstream_transcript_variant,non_coding_transcript_variant
rs886061948	3_prime_UTR_variant,genic_downstream_transcript_variant,non_coding_transcript_variant
rs886061949	3_prime_UTR_variant,genic_downstream_transcript_variant,non_coding_transcript_variant
rs886061950	3_prime_UTR_variant,genic_downstream_transcript_variant,non_coding_transcript_variant
rs886061951	3_prime_UTR_variant,genic_downstream_transcript_variant,non_coding_transcript_variant
rs891908277	synonymous_variant,coding_sequence_variant,non_coding_transcript_variant
rs895820328	coding_sequence_variant,non_coding_transcript_variant,missense_variant
rs899151863	coding_sequence_variant,genic_upstream_transcript_variant,non_coding_transcript_variant,missense_variant,intron_variant
rs902884572	synonymous_variant,coding_sequence_variant,non_coding_transcript_variant
rs904162290	coding_sequence_variant,genic_upstream_transcript_variant,non_coding_transcript_variant,missense_variant,intron_variant
rs904275312	coding_sequence_variant,genic_upstream_transcript_variant,non_coding_transcript_variant,missense_variant,intron_variant
rs905891313	coding_sequence_variant,non_coding_transcript_variant,missense_variant
rs908653562	genic_downstream_transcript_variant,non_coding_transcript_variant,coding_sequence_variant,missense_variant
rs910873191	non_coding_transcript_variant,coding_sequence_variant,synonymous_variant
rs910937816	genic_downstream_transcript_variant,non_coding_transcript_variant,coding_sequence_variant,missense_variant
rs911380470	5_prime_UTR_variant,coding_sequence_variant,missense_variant,non_coding_transcript_variant
rs912927724	genic_downstream_transcript_variant,non_coding_transcript_variant,coding_sequence_variant,missense_variant
rs919828654	coding_sequence_variant,non_coding_transcript_variant,genic_upstream_transcript_variant,intron_variant,missense_variant
rs921083171	coding_sequence_variant,non_coding_transcript_variant,genic_upstream_transcript_variant,intron_variant,missense_variant
rs922296506	missense_variant,coding_sequence_variant,non_coding_transcript_variant,5_prime_UTR_variant
rs922442405	missense_variant,coding_sequence_variant,non_coding_transcript_variant,genic_downstream_transcript_variant
rs926365001	synonymous_variant,coding_sequence_variant,non_coding_transcript_variant,genic_downstream_transcript_variant
rs933744184	genic_downstream_transcript_variant,synonymous_variant,coding_sequence_variant,non_coding_transcript_variant
rs934426317	non_coding_transcript_variant,intron_variant,coding_sequence_variant,synonymous_variant,genic_upstream_transcript_variant
rs935854365	3_prime_UTR_variant,genic_downstream_transcript_variant,non_coding_transcript_variant
rs943753621	missense_variant,coding_sequence_variant,non_coding_transcript_variant
rs950642030	missense_variant,genic_downstream_transcript_variant,coding_sequence_variant,non_coding_transcript_variant
rs953588907	intron_variant
rs955990225	non_coding_transcript_variant,3_prime_UTR_variant,genic_downstream_transcript_variant
rs958023403	non_coding_transcript_variant,synonymous_variant,coding_sequence_variant
rs965135961	5_prime_UTR_variant,non_coding_transcript_variant,synonymous_variant,coding_sequence_variant
rs965319455	missense_variant,non_coding_transcript_variant,coding_sequence_variant
rs973796037	coding_sequence_variant,non_coding_transcript_variant,genic_upstream_transcript_variant,missense_variant,intron_variant
rs974352099	genic_downstream_transcript_variant,non_coding_transcript_variant,missense_variant,coding_sequence_variant
rs974448652	coding_sequence_variant,non_coding_transcript_variant,genic_upstream_transcript_variant,missense_variant,intron_variant
rs978734201	genic_downstream_transcript_variant,coding_sequence_variant,synonymous_variant,non_coding_transcript_variant
rs980467681	coding_sequence_variant,non_coding_transcript_variant,missense_variant
rs983457211	coding_sequence_variant,non_coding_transcript_variant,missense_variant,intron_variant,genic_upstream_transcript_variant
rs986139438	coding_sequence_variant,non_coding_transcript_variant,5_prime_UTR_variant,missense_variant
rs993773653	coding_sequence_variant,non_coding_transcript_variant,genic_downstream_transcript_variant,missense_variant
rs1001801655	3_prime_UTR_variant,genic_downstream_transcript_variant,non_coding_transcript_variant
rs1003586623	coding_sequence_variant,missense_variant,non_coding_transcript_variant
rs1004264326	coding_sequence_variant,missense_variant,non_coding_transcript_variant
rs1012909374	coding_sequence_variant,non_coding_transcript_variant,missense_variant
rs1013431413	splice_donor_variant
rs1027607387	synonymous_variant,intron_variant,coding_sequence_variant

rs1028165414	missense_variant,genic_upstream_transcript_variant,intron_variant,non_coding_transcript_variant,coding_sequence_variant
rs1032682239	missense_variant,genic_upstream_transcript_variant,intron_variant,non_coding_transcript_variant,coding_sequence_variant
rs1040920548	coding_sequence_variant,non_coding_transcript_variant,genic_upstream_transcript_variant,intron_variant,missense_variant
rs1041349799	coding_sequence_variant,non_coding_transcript_variant,missense_variant
rs1047795344	coding_sequence_variant,non_coding_transcript_variant,stop_gained,synonymous_variant,missense_variant
rs1052270861	missense_variant,non_coding_transcript_variant,coding_sequence_variant
rs1057519824	missense_variant,non_coding_transcript_variant,genic_downstream_transcript_variant,coding_sequence_variant
rs1057520030	missense_variant,non_coding_transcript_variant,genic_downstream_transcript_variant,coding_sequence_variant
rs1060503528	missense_variant,non_coding_transcript_variant,intron_variant,genic_upstream_transcript_variant,coding_sequence_variant
rs1060503529	missense_variant,non_coding_transcript_variant,intron_variant,genic_upstream_transcript_variant,coding_sequence_variant
rs1060503530	missense_variant,non_coding_transcript_variant,intron_variant,genic_upstream_transcript_variant,coding_sequence_variant
rs1060503531	missense_variant,non_coding_transcript_variant,coding_sequence_variant
rs1060503532	missense_variant,non_coding_transcript_variant,intron_variant,genic_upstream_transcript_variant,coding_sequence_variant
rs1060503533	missense_variant,non_coding_transcript_variant,genic_downstream_transcript_variant,coding_sequence_variant
rs1060503534	missense_variant,non_coding_transcript_variant,genic_downstream_transcript_variant,coding_sequence_variant
rs1060503535	inframe_deletion,non_coding_transcript_variant,coding_sequence_variant
rs1060503536	missense_variant,non_coding_transcript_variant,coding_sequence_variant
rs1060503537	missense_variant,non_coding_transcript_variant,intron_variant,genic_upstream_transcript_variant,coding_sequence_variant
rs1060503538	missense_variant,non_coding_transcript_variant,intron_variant,genic_upstream_transcript_variant,coding_sequence_variant
rs1060503539	missense_variant,non_coding_transcript_variant,coding_sequence_variant
rs1060503540	missense_variant,non_coding_transcript_variant,genic_downstream_transcript_variant,coding_sequence_variant
rs1060503541	missense_variant,intron_variant,coding_sequence_variant
rs1060503542	missense_variant,non_coding_transcript_variant,intron_variant,genic_upstream_transcript_variant,coding_sequence_variant
rs1060503543	missense_variant,non_coding_transcript_variant,genic_downstream_transcript_variant,coding_sequence_variant
rs1060503544	missense_variant,non_coding_transcript_variant,intron_variant,genic_upstream_transcript_variant,coding_sequence_variant
rs1060504939	non_coding_transcript_variant,synonymous_variant,intron_variant,genic_upstream_transcript_variant,coding_sequence_variant
rs1060504940	non_coding_transcript_variant,synonymous_variant,intron_variant,genic_upstream_transcript_variant,coding_sequence_variant
rs1060504941	non_coding_transcript_variant,synonymous_variant,intron_variant,genic_upstream_transcript_variant,coding_sequence_variant
rs1060504942	non_coding_transcript_variant,synonymous_variant,intron_variant,genic_upstream_transcript_variant,coding_sequence_variant
rs1060504943	intron_variant
rs1060504944	non_coding_transcript_variant,coding_sequence_variant,synonymous_variant
rs1060504945	intron_variant,genic_downstream_transcript_variant
rs1060504946	non_coding_transcript_variant,coding_sequence_variant,synonymous_variant
rs1156480798	non_coding_transcript_variant,coding_sequence_variant,synonymous_variant
rs1159812687	missense_variant,non_coding_transcript_variant,genic_downstream_transcript_variant,coding_sequence_variant
rs1160304097	intron_variant
rs1162352573	synonymous_variant,non_coding_transcript_variant,genic_downstream_transcript_variant,coding_sequence_variant
rs1162540199	genic_upstream_transcript_variant,non_coding_transcript_variant,intron_variant,missense_variant,coding_sequence_variant
rs1165913241	genic_upstream_transcript_variant,non_coding_transcript_variant,intron_variant,missense_variant,coding_sequence_variant
rs1169278904	genic_upstream_transcript_variant,non_coding_transcript_variant,intron_variant,missense_variant,coding_sequence_variant
rs1174194833	stop_gained,coding_sequence_variant,non_coding_transcript_variant,genic_downstream_transcript_variant
rs1177400289	missense_variant,coding_sequence_variant,non_coding_transcript_variant,genic_downstream_transcript_variant
rs1179812872	missense_variant,coding_sequence_variant,non_coding_transcript_variant,genic_downstream_transcript_variant
rs1180556775	coding_sequence_variant,intron_variant,missense_variant
rs1181927481	missense_variant,coding_sequence_variant,non_coding_transcript_variant
rs1186230260	missense_variant,coding_sequence_variant,non_coding_transcript_variant
rs1191752014	non_coding_transcript_variant,missense_variant,coding_sequence_variant,genic_downstream_transcript_variant
rs1193441628	non_coding_transcript_variant,stop_gained,coding_sequence_variant,genic_downstream_transcript_variant
rs1194542748	missense_variant,5_prime_UTR_variant,coding_sequence_variant,non_coding_transcript_variant
rs1197127761	missense_variant,non_coding_transcript_variant,coding_sequence_variant
rs1205392044	intron_variant,missense_variant,coding_sequence_variant
rs1207133647	3_prime_UTR_variant,genic_downstream_transcript_variant,non_coding_transcript_variant
rs1207381066	missense_variant,non_coding_transcript_variant,genic_upstream_transcript_variant,intron_variant,coding_sequence_variant
rs1209392687	non_coding_transcript_variant,coding_sequence_variant,missense_variant
rs1209881740	non_coding_transcript_variant,coding_sequence_variant,missense_variant
rs1210616219	genic_upstream_transcript_variant,non_coding_transcript_variant,missense_variant,coding_sequence_variant,intron_variant
rs1215872095	non_coding_transcript_variant,coding_sequence_variant,missense_variant
rs1216005099	non_coding_transcript_variant,coding_sequence_variant,missense_variant
rs1219703716	genic_downstream_transcript_variant,coding_sequence_variant,non_coding_transcript_variant,synonymous_variant
rs1221580502	intron_variant,synonymous_variant,genic_upstream_transcript_variant,coding_sequence_variant,non_coding_transcript_variant
rs1225303075	intron_variant,synonymous_variant,genic_upstream_transcript_variant,coding_sequence_variant,non_coding_transcript_variant
rs1226545782	missense_variant,intron_variant,genic_upstream_transcript_variant,coding_sequence_variant,non_coding_transcript_variant
rs1230741853	5_prime_UTR_variant,coding_sequence_variant,missense_variant,non_coding_transcript_variant
rs1232896056	missense_variant,intron_variant,genic_upstream_transcript_variant,coding_sequence_variant,non_coding_transcript_variant
rs1237033724	genic_downstream_transcript_variant,missense_variant,coding_sequence_variant,non_coding_transcript_variant
rs1237383281	genic_downstream_transcript_variant,3_prime_UTR_variant,non_coding_transcript_variant
rs1240673121	splice_acceptor_variant
rs1240760202	coding_sequence_variant,synonymous_variant,non_coding_transcript_variant
rs1246645896	non_coding_transcript_variant,intron_variant,genic_upstream_transcript_variant,missense_variant,coding_sequence_variant
rs1246936382	non_coding_transcript_variant,intron_variant,genic_upstream_transcript_variant,missense_variant,coding_sequence_variant,synonymous_variant
rs1247472138	non_coding_transcript_variant,missense_variant,genic_downstream_transcript_variant,coding_sequence_variant
rs1251215478	non_coding_transcript_variant,genic_downstream_transcript_variant,missense_variant,coding_sequence_variant,synonymous_variant
rs1253878709	non_coding_transcript_variant,missense_variant,coding_sequence_variant
rs1258377870	non_coding_transcript_variant,missense_variant,genic_downstream_transcript_variant,coding_sequence_variant

rs1258671501	non_coding_transcript_variant,missense_variant,genic_downstream_transcript_variant,coding_sequence_variant
rs1260635670	intron_variant,genic_upstream_transcript_variant,coding_sequence_variant,non_coding_transcript_variant,missense_variant
rs1261418076	non_coding_transcript_variant,missense_variant,coding_sequence_variant,genic_downstream_transcript_variant
rs1262601648	missense_variant,intron_variant,coding_sequence_variant
rs1263785859	non_coding_transcript_variant,missense_variant,coding_sequence_variant,genic_downstream_transcript_variant
rs1266863567	intron_variant,genic_upstream_transcript_variant,coding_sequence_variant,non_coding_transcript_variant,missense_variant
rs1278254320	non_coding_transcript_variant,missense_variant,genic_downstream_transcript_variant,coding_sequence_variant
rs1282444375	non_coding_transcript_variant,synonymous_variant,coding_sequence_variant
rs1282638781	missense_variant,non_coding_transcript_variant,coding_sequence_variant,intron_variant,genic_upstream_transcript_variant
rs1288860579	missense_variant,genic_downstream_transcript_variant,non_coding_transcript_variant,coding_sequence_variant
rs1289461217	missense_variant,5_prime_UTR_variant,non_coding_transcript_variant,coding_sequence_variant
rs1291977283	non_coding_transcript_variant,coding_sequence_variant,intron_variant,genic_upstream_transcript_variant,missense_variant
rs1292389793	missense_variant,genic_downstream_transcript_variant,non_coding_transcript_variant,coding_sequence_variant
rs1294910019	missense_variant,genic_downstream_transcript_variant,non_coding_transcript_variant,coding_sequence_variant
rs1294994065	non_coding_transcript_variant,coding_sequence_variant,intron_variant,genic_upstream_transcript_variant,missense_variant
rs1296330997	coding_sequence_variant,intron_variant,missense_variant,genic_upstream_transcript_variant,non_coding_transcript_variant
rs1301383613	intron_variant,genic_downstream_transcript_variant
rs1305399003	intron_variant
rs1306040798	coding_sequence_variant,intron_variant,missense_variant,genic_upstream_transcript_variant,non_coding_transcript_variant
rs1306740707	coding_sequence_variant,intron_variant,missense_variant,genic_upstream_transcript_variant,non_coding_transcript_variant
rs1307693651	coding_sequence_variant,non_coding_transcript_variant,missense_variant
rs1313347428	missense_variant,non_coding_transcript_variant,coding_sequence_variant
rs1318406399	missense_variant,non_coding_transcript_variant,coding_sequence_variant
rs1319458719	missense_variant,non_coding_transcript_variant,genic_downstream_transcript_variant,coding_sequence_variant
rs1320718414	non_coding_transcript_variant,intron_variant,genic_upstream_transcript_variant,coding_sequence_variant,missense_variant
rs1321841621	3_prime_UTR_variant,genic_downstream_transcript_variant,non_coding_transcript_variant
rs1323787899	genic_downstream_transcript_variant,non_coding_transcript_variant,coding_sequence_variant,synonymous_variant
rs1326405747	coding_sequence_variant,intron_variant,non_coding_transcript_variant,missense_variant,genic_upstream_transcript_variant
rs1328454755	missense_variant,non_coding_transcript_variant,coding_sequence_variant
rs1333930330	non_coding_transcript_variant,genic_downstream_transcript_variant,coding_sequence_variant,missense_variant
rs1336351205	splice_acceptor_variant
rs1337305891	intron_variant
rs1342295714	non_coding_transcript_variant,synonymous_variant,coding_sequence_variant,5_prime_UTR_variant
rs1344902873	non_coding_transcript_variant,coding_sequence_variant,missense_variant
rs1346572528	non_coding_transcript_variant,missense_variant,coding_sequence_variant
rs1348453406	non_coding_transcript_variant,missense_variant,genic_downstream_transcript_variant,coding_sequence_variant
rs1351634349	non_coding_transcript_variant,missense_variant,coding_sequence_variant
rs1354964885	synonymous_variant,non_coding_transcript_variant,stop_gained,coding_sequence_variant
rs1355716761	missense_variant,intron_variant,coding_sequence_variant
rs1355886011	missense_variant,genic_upstream_transcript_variant,non_coding_transcript_variant,intron_variant,coding_sequence_variant
rs1357266763	non_coding_transcript_variant,missense_variant,genic_downstream_transcript_variant,coding_sequence_variant
rs1358705636	non_coding_transcript_variant,synonymous_variant,5_prime_UTR_variant,coding_sequence_variant
rs1360184564	intron_variant,genic_downstream_transcript_variant
rs1361155132	non_coding_transcript_variant,coding_sequence_variant,genic_downstream_transcript_variant,missense_variant
rs1362532895	intron_variant
rs1363905218	synonymous_variant,intron_variant,non_coding_transcript_variant,coding_sequence_variant,genic_upstream_transcript_variant
rs1369230650	synonymous_variant,intron_variant,non_coding_transcript_variant,coding_sequence_variant,genic_upstream_transcript_variant
rs1370688649	splice_acceptor_variant
rs1375872823	coding_sequence_variant,genic_upstream_transcript_variant,missense_variant,non_coding_transcript_variant,intron_variant
rs1375932394	coding_sequence_variant,genic_upstream_transcript_variant,missense_variant,non_coding_transcript_variant,intron_variant
rs1376208948	synonymous_variant,coding_sequence_variant,non_coding_transcript_variant
rs1382410716	synonymous_variant,coding_sequence_variant,non_coding_transcript_variant
rs1384134548	missense_variant,coding_sequence_variant,non_coding_transcript_variant
rs1385709850	coding_sequence_variant,missense_variant,non_coding_transcript_variant
rs1386889641	synonymous_variant,coding_sequence_variant,genic_downstream_transcript_variant,non_coding_transcript_variant
rs1390590380	missense_variant,coding_sequence_variant,genic_downstream_transcript_variant,non_coding_transcript_variant
rs1390958903	coding_sequence_variant,missense_variant,non_coding_transcript_variant
rs1391853762	coding_sequence_variant,missense_variant,non_coding_transcript_variant
rs1394199299	missense_variant,coding_sequence_variant,genic_downstream_transcript_variant,non_coding_transcript_variant
rs1394626435	coding_sequence_variant,missense_variant,non_coding_transcript_variant
rs1395233386	intron_variant,missense_variant,genic_upstream_transcript_variant,coding_sequence_variant,non_coding_transcript_variant
rs1395763398	missense_variant,coding_sequence_variant,genic_downstream_transcript_variant,non_coding_transcript_variant
rs1395827839	intron_variant,missense_variant,genic_upstream_transcript_variant,coding_sequence_variant,non_coding_transcript_variant
rs1396083482	intron_variant,missense_variant,genic_upstream_transcript_variant,coding_sequence_variant,non_coding_transcript_variant
rs1397743069	genic_downstream_transcript_variant,coding_sequence_variant,missense_variant,non_coding_transcript_variant
rs1397775000	missense_variant,intron_variant,coding_sequence_variant,splice_acceptor_variant
rs1398243931	coding_sequence_variant,missense_variant,non_coding_transcript_variant
rs1398543726	genic_downstream_transcript_variant,coding_sequence_variant,missense_variant,non_coding_transcript_variant
rs1398648043	coding_sequence_variant,missense_variant,non_coding_transcript_variant
rs1402713307	coding_sequence_variant,missense_variant,non_coding_transcript_variant
rs1404824650	genic_downstream_transcript_variant,coding_sequence_variant,missense_variant,non_coding_transcript_variant
rs1408351682	coding_sequence_variant,missense_variant,non_coding_transcript_variant
rs1410705895	missense_variant,coding_sequence_variant,intron_variant,genic_upstream_transcript_variant,non_coding_transcript_variant
rs1410859976	missense_variant,non_coding_transcript_variant,coding_sequence_variant

rs1412870024	non_coding_transcript_variant,coding_sequence_variant,synonymous_variant
rs1416393044	missense_variant,coding_sequence_variant,intron_variant,genic_upstream_transcript_variant,non_coding_transcript_variant
rs1417054613	coding_sequence_variant,intron_variant,genic_upstream_transcript_variant,non_coding_transcript_variant,synonymous_variant
rs1420349380	missense_variant,non_coding_transcript_variant,genic_downstream_transcript_variant,coding_sequence_variant
rs1428685262	genic_upstream_transcript_variant,non_coding_transcript_variant,coding_sequence_variant,synonymous_variant,intron_variant
rs1429917144	coding_sequence_variant,missense_variant,non_coding_transcript_variant
rs1430652609	inframe_deletion,coding_sequence_variant,non_coding_transcript_variant
rs1431679847	intron_variant
rs1432247099	intron_variant
rs1432416607	coding_sequence_variant,synonymous_variant,missense_variant,non_coding_transcript_variant
rs1436537175	coding_sequence_variant,non_coding_transcript_variant,genic_downstream_transcript_variant,missense_variant
rs1436957498	intron_variant,non_coding_transcript_variant,coding_sequence_variant,genic_upstream_transcript_variant,missense_variant
rs1441045077	intron_variant,non_coding_transcript_variant,coding_sequence_variant,genic_upstream_transcript_variant,missense_variant
rs1442896663	coding_sequence_variant,non_coding_transcript_variant,genic_downstream_transcript_variant,missense_variant
rs1443741792	coding_sequence_variant,non_coding_transcript_variant,genic_downstream_transcript_variant,missense_variant
rs1444020594	intron_variant,non_coding_transcript_variant,coding_sequence_variant,genic_upstream_transcript_variant,synonymous_variant
rs1445578353	coding_sequence_variant,non_coding_transcript_variant,missense_variant
rs1446482547	coding_sequence_variant,non_coding_transcript_variant,missense_variant
rs1447240969	genic_downstream_transcript_variant,non_coding_transcript_variant,3_prime_UTR_variant
rs1447318689	genic_downstream_transcript_variant,coding_sequence_variant,synonymous_variant,non_coding_transcript_variant
rs1451799154	intron_variant,genic_upstream_transcript_variant,synonymous_variant,coding_sequence_variant,non_coding_transcript_variant,missense_variant
rs1453842331	genic_downstream_transcript_variant,coding_sequence_variant,non_coding_transcript_variant,missense_variant
rs1457756402	intron_variant,genic_upstream_transcript_variant,synonymous_variant,coding_sequence_variant,non_coding_transcript_variant
rs1458827441	coding_sequence_variant,synonymous_variant,non_coding_transcript_variant
rs1458912347	coding_sequence_variant,non_coding_transcript_variant,missense_variant
rs1460031754	coding_sequence_variant,genic_downstream_transcript_variant,missense_variant,non_coding_transcript_variant
rs1463245808	non_coding_transcript_variant,intron_variant,genic_upstream_transcript_variant,missense_variant,coding_sequence_variant
rs1463527073	synonymous_variant,coding_sequence_variant,missense_variant,non_coding_transcript_variant
rs1465559829	synonymous_variant,coding_sequence_variant,non_coding_transcript_variant
rs1470403316	splice_acceptor_variant
rs1472858680	missense_variant,non_coding_transcript_variant,intron_variant,genic_upstream_transcript_variant,coding_sequence_variant
rs1474436542	missense_variant,non_coding_transcript_variant,intron_variant,genic_upstream_transcript_variant,coding_sequence_variant
rs1477354447	intron_variant,genic_downstream_transcript_variant
rs1478309975	missense_variant,coding_sequence_variant,non_coding_transcript_variant
rs1484724510	missense_variant,coding_sequence_variant,non_coding_transcript_variant
rs1487106127	non_coding_transcript_variant,coding_sequence_variant,intron_variant,genic_upstream_transcript_variant,missense_variant
rs1489302008	missense_variant,coding_sequence_variant,intron_variant
rs1491102590	intron_variant
rs1554378225	non_coding_transcript_variant,coding_sequence_variant,intron_variant,genic_upstream_transcript_variant,missense_variant
rs1554378291	non_coding_transcript_variant,coding_sequence_variant,intron_variant,genic_upstream_transcript_variant,missense_variant
rs1554378297	non_coding_transcript_variant,coding_sequence_variant,intron_variant,genic_upstream_transcript_variant,missense_variant
rs1554378400	non_coding_transcript_variant,coding_sequence_variant,intron_variant,genic_upstream_transcript_variant,missense_variant
rs1554378412	non_coding_transcript_variant,coding_sequence_variant,intron_variant,genic_upstream_transcript_variant,missense_variant
rs1554378434	non_coding_transcript_variant,coding_sequence_variant,intron_variant,genic_upstream_transcript_variant,missense_variant
rs1554378477	non_coding_transcript_variant,coding_sequence_variant,intron_variant,genic_upstream_transcript_variant,missense_variant
rs1554378495	non_coding_transcript_variant,coding_sequence_variant,intron_variant,genic_upstream_transcript_variant,missense_variant
rs1554378645	non_coding_transcript_variant,coding_sequence_variant,intron_variant,genic_upstream_transcript_variant,frameshift_variant
rs1554378780	non_coding_transcript_variant,coding_sequence_variant,intron_variant,genic_upstream_transcript_variant,missense_variant
rs1554378983	non_coding_transcript_variant,coding_sequence_variant,intron_variant,genic_upstream_transcript_variant,missense_variant
rs1554379058	non_coding_transcript_variant,coding_sequence_variant,intron_variant,genic_upstream_transcript_variant,missense_variant
rs1554379127	non_coding_transcript_variant,coding_sequence_variant,intron_variant,synonymous_variant,genic_upstream_transcript_variant
rs1554379158	non_coding_transcript_variant,coding_sequence_variant,intron_variant,genic_upstream_transcript_variant,missense_variant
rs1554379180	non_coding_transcript_variant,coding_sequence_variant,intron_variant,genic_upstream_transcript_variant,missense_variant
rs1554379184	non_coding_transcript_variant,coding_sequence_variant,intron_variant,synonymous_variant,genic_upstream_transcript_variant
rs1554379254	non_coding_transcript_variant,coding_sequence_variant,intron_variant,synonymous_variant,genic_upstream_transcript_variant
rs1554388638	missense_variant,non_coding_transcript_variant,coding_sequence_variant,5_prime_UTR_variant
rs1554390902	missense_variant,non_coding_transcript_variant,coding_sequence_variant
rs1554390925	non_coding_transcript_variant,coding_sequence_variant,synonymous_variant
rs1554391208	intron_variant
rs1554391227	missense_variant,non_coding_transcript_variant,coding_sequence_variant
rs1554391232	non_coding_transcript_variant,coding_sequence_variant,synonymous_variant
rs1554391268	non_coding_transcript_variant,coding_sequence_variant,synonymous_variant
rs1554391313	non_coding_transcript_variant,coding_sequence_variant,synonymous_variant
rs1554391336	non_coding_transcript_variant,coding_sequence_variant,synonymous_variant
rs1554394916	intron_variant
rs1554394973	missense_variant,non_coding_transcript_variant,coding_sequence_variant
rs1554395371	missense_variant,non_coding_transcript_variant,coding_sequence_variant
rs1554395373	missense_variant,non_coding_transcript_variant,coding_sequence_variant
rs1554395374	missense_variant,non_coding_transcript_variant,coding_sequence_variant
rs1554395395	missense_variant,non_coding_transcript_variant,coding_sequence_variant
rs1554395399	missense_variant,non_coding_transcript_variant,coding_sequence_variant
rs1554395462	missense_variant,non_coding_transcript_variant,coding_sequence_variant
rs1554395656	non_coding_transcript_variant,coding_sequence_variant,synonymous_variant
rs1554395668	missense_variant,non_coding_transcript_variant,coding_sequence_variant

rs1554395680	missense_variant,non_coding_transcript_variant,coding_sequence_variant
rs1554395688	missense_variant,non_coding_transcript_variant,coding_sequence_variant
rs1554395720	non_coding_transcript_variant,coding_sequence_variant,synonymous_variant
rs1554395861	splice_acceptor_variant,intron_variant
rs1554395864	coding_sequence_variant,intron_variant,frameshift_variant
rs1554395868	missense_variant,coding_sequence_variant,intron_variant
rs1554395889	missense_variant,coding_sequence_variant,intron_variant
rs1554396554	missense_variant,non_coding_transcript_variant,coding_sequence_variant
rs1554396571	missense_variant,non_coding_transcript_variant,coding_sequence_variant
rs1554397896	intron_variant
rs1554398244	genic_downstream_transcript_variant,missense_variant,non_coding_transcript_variant,coding_sequence_variant
rs1554398258	genic_downstream_transcript_variant,non_coding_transcript_variant,coding_sequence_variant,synonymous_variant
rs1554398274	genic_downstream_transcript_variant,non_coding_transcript_variant,coding_sequence_variant,synonymous_variant
rs1554398288	genic_downstream_transcript_variant,missense_variant,non_coding_transcript_variant,coding_sequence_variant
rs1554398360	genic_downstream_transcript_variant,missense_variant,non_coding_transcript_variant,coding_sequence_variant
rs1554398378	genic_downstream_transcript_variant,missense_variant,non_coding_transcript_variant,coding_sequence_variant
rs1554398390	genic_downstream_transcript_variant,missense_variant,non_coding_transcript_variant,coding_sequence_variant
rs1554398397	genic_downstream_transcript_variant,non_coding_transcript_variant,coding_sequence_variant,stop_gained
rs1554398401	genic_downstream_transcript_variant,non_coding_transcript_variant,coding_sequence_variant,synonymous_variant
rs1554398418	genic_downstream_transcript_variant,missense_variant,non_coding_transcript_variant,coding_sequence_variant
rs1554398862	genic_downstream_transcript_variant,missense_variant,non_coding_transcript_variant,coding_sequence_variant
rs1554398889	genic_downstream_transcript_variant,non_coding_transcript_variant,coding_sequence_variant,synonymous_variant
rs1554398900	genic_downstream_transcript_variant,missense_variant,non_coding_transcript_variant,coding_sequence_variant
rs1554399341	genic_downstream_transcript_variant,non_coding_transcript_variant,coding_sequence_variant,synonymous_variant
rs1554399352	genic_downstream_transcript_variant,splice_donor_variant
rs1554399534	genic_downstream_transcript_variant,non_coding_transcript_variant,coding_sequence_variant,synonymous_variant
rs1554399556	genic_downstream_transcript_variant,missense_variant,non_coding_transcript_variant,coding_sequence_variant
rs1554399597	genic_downstream_transcript_variant,missense_variant,non_coding_transcript_variant,coding_sequence_variant
rs1554400069	genic_downstream_transcript_variant,intron_variant
rs1554400071	genic_downstream_transcript_variant,missense_variant,non_coding_transcript_variant,coding_sequence_variant
rs1554400099	genic_downstream_transcript_variant,missense_variant,non_coding_transcript_variant,coding_sequence_variant
rs1554400274	genic_downstream_transcript_variant,missense_variant,non_coding_transcript_variant,coding_sequence_variant
rs1554400286	genic_downstream_transcript_variant,missense_variant,non_coding_transcript_variant,coding_sequence_variant
rs1554402156	genic_downstream_transcript_variant,non_coding_transcript_variant,coding_sequence_variant,synonymous_variant
rs1554402170	genic_downstream_transcript_variant,non_coding_transcript_variant,coding_sequence_variant,synonymous_variant
rs1554402183	genic_downstream_transcript_variant,intron_variant
rs1554402266	genic_downstream_transcript_variant,missense_variant,non_coding_transcript_variant,coding_sequence_variant
rs1562882716	5_prime_UTR_variant,coding_sequence_variant,intron_variant,genic_upstream_transcript_variant,frameshift_variant,non_coding_transcript_variant
rs1562882876	coding_sequence_variant,intron_variant,genic_upstream_transcript_variant,non_coding_transcript_variant,missense_variant
rs1562883006	coding_sequence_variant,intron_variant,genic_upstream_transcript_variant,non_coding_transcript_variant,missense_variant
rs1562883214	coding_sequence_variant,intron_variant,genic_upstream_transcript_variant,non_coding_transcript_variant,missense_variant
rs1562883374	coding_sequence_variant,intron_variant,genic_upstream_transcript_variant,non_coding_transcript_variant,missense_variant
rs1562883575	coding_sequence_variant,intron_variant,genic_upstream_transcript_variant,non_coding_transcript_variant,missense_variant
rs1562883992	coding_sequence_variant,intron_variant,genic_upstream_transcript_variant,non_coding_transcript_variant,missense_variant
rs1562884016	coding_sequence_variant,intron_variant,genic_upstream_transcript_variant,non_coding_transcript_variant,missense_variant
rs1562884246	coding_sequence_variant,intron_variant,genic_upstream_transcript_variant,non_coding_transcript_variant,missense_variant
rs1562884601	coding_sequence_variant,intron_variant,genic_upstream_transcript_variant,non_coding_transcript_variant,missense_variant
rs1562908826	missense_variant,coding_sequence_variant,non_coding_transcript_variant
rs1562920414	missense_variant,coding_sequence_variant,non_coding_transcript_variant
rs1562921668	missense_variant,coding_sequence_variant,non_coding_transcript_variant
rs1562922167	missense_variant,coding_sequence_variant,non_coding_transcript_variant
rs1562923455	intron_variant
rs1562923583	missense_variant,coding_sequence_variant,intron_variant
rs1562925631	missense_variant,coding_sequence_variant,non_coding_transcript_variant
rs1562925732	missense_variant,coding_sequence_variant,non_coding_transcript_variant
rs1562925733	missense_variant,coding_sequence_variant,non_coding_transcript_variant
rs1562929174	missense_variant,coding_sequence_variant,non_coding_transcript_variant
rs1562930067	genic_downstream_transcript_variant,missense_variant,coding_sequence_variant,non_coding_transcript_variant
rs1562930263	genic_downstream_transcript_variant,intron_variant
rs1562931818	genic_downstream_transcript_variant,missense_variant,coding_sequence_variant,non_coding_transcript_variant
rs1562935204	genic_downstream_transcript_variant,frameshift_variant,coding_sequence_variant,non_coding_transcript_variant
rs1562935856	genic_downstream_transcript_variant,missense_variant,coding_sequence_variant,non_coding_transcript_variant
rs1562935903	genic_downstream_transcript_variant,missense_variant,coding_sequence_variant,non_coding_transcript_variant
rs1562941469	genic_downstream_transcript_variant,splice_donor_variant
rs1562941559	genic_downstream_transcript_variant,missense_variant,coding_sequence_variant,non_coding_transcript_variant
rs1562941622	genic_downstream_transcript_variant,missense_variant,coding_sequence_variant,non_coding_transcript_variant
rs1584875601	genic_upstream_transcript_variant,intron_variant,coding_sequence_variant,non_coding_transcript_variant,missense_variant
rs1584875653	genic_upstream_transcript_variant,intron_variant,coding_sequence_variant,non_coding_transcript_variant,synonymous_variant
rs1584875673	genic_upstream_transcript_variant,intron_variant,coding_sequence_variant,non_coding_transcript_variant,synonymous_variant
rs1584875719	genic_upstream_transcript_variant,intron_variant,coding_sequence_variant,non_coding_transcript_variant,missense_variant
rs1584875753	genic_upstream_transcript_variant,intron_variant,coding_sequence_variant,non_coding_transcript_variant,synonymous_variant
rs1584876240	genic_upstream_transcript_variant,intron_variant,coding_sequence_variant,non_coding_transcript_variant,missense_variant
rs1584876265	genic_upstream_transcript_variant,intron_variant,coding_sequence_variant,non_coding_transcript_variant,synonymous_variant
rs1584876383	genic_upstream_transcript_variant,intron_variant,coding_sequence_variant,non_coding_transcript_variant,synonymous_variant

rs1584876390	genic_upstream_transcript_variant,intron_variant,coding_sequence_variant,non_coding_transcript_variant,synonymous_variant
rs1584876615	genic_upstream_transcript_variant,intron_variant,coding_sequence_variant,non_coding_transcript_variant,missense_variant
rs1584876640	genic_upstream_transcript_variant,intron_variant,coding_sequence_variant,non_coding_transcript_variant,missense_variant
rs1584876662	genic_upstream_transcript_variant,intron_variant,coding_sequence_variant,non_coding_transcript_variant,synonymous_variant
rs1584876675	genic_upstream_transcript_variant,intron_variant,coding_sequence_variant,non_coding_transcript_variant,missense_variant
rs1584876716	genic_upstream_transcript_variant,intron_variant,coding_sequence_variant,non_coding_transcript_variant,missense_variant
rs1584876832	genic_upstream_transcript_variant,intron_variant,coding_sequence_variant,non_coding_transcript_variant,missense_variant
rs1584876842	genic_upstream_transcript_variant,intron_variant,coding_sequence_variant,non_coding_transcript_variant,synonymous_variant
rs1584876891	genic_upstream_transcript_variant,intron_variant,coding_sequence_variant,non_coding_transcript_variant,synonymous_variant
rs1584876898	genic_upstream_transcript_variant,intron_variant,coding_sequence_variant,non_coding_transcript_variant,missense_variant
rs1584876914	genic_upstream_transcript_variant,intron_variant,coding_sequence_variant,non_coding_transcript_variant,missense_variant
rs1584876986	genic_upstream_transcript_variant,intron_variant,coding_sequence_variant,non_coding_transcript_variant,missense_variant
rs1584877055	genic_upstream_transcript_variant,intron_variant,coding_sequence_variant,non_coding_transcript_variant,missense_variant
rs1584877113	genic_upstream_transcript_variant,intron_variant,coding_sequence_variant,synonymous_variant,non_coding_transcript_variant,missense_variant
rs1584877307	genic_upstream_transcript_variant,intron_variant,coding_sequence_variant,non_coding_transcript_variant,synonymous_variant
rs1584877533	genic_upstream_transcript_variant,intron_variant,frameshift_variant,coding_sequence_variant,non_coding_transcript_variant
rs1584877825	genic_upstream_transcript_variant,intron_variant,coding_sequence_variant,synonymous_variant,non_coding_transcript_variant,missense_variant
rs1584877845	genic_upstream_transcript_variant,intron_variant,coding_sequence_variant,non_coding_transcript_variant,missense_variant
rs1584877929	genic_upstream_transcript_variant,inframe_deletion,intron_variant,coding_sequence_variant,non_coding_transcript_variant
rs1584877972	genic_upstream_transcript_variant,intron_variant,coding_sequence_variant,non_coding_transcript_variant,missense_variant
rs1584878137	genic_upstream_transcript_variant,intron_variant,coding_sequence_variant,non_coding_transcript_variant,missense_variant
rs1584878246	genic_upstream_transcript_variant,intron_variant,coding_sequence_variant,non_coding_transcript_variant,missense_variant
rs1584878304	genic_upstream_transcript_variant,intron_variant,coding_sequence_variant,non_coding_transcript_variant,synonymous_variant
rs1584878345	genic_upstream_transcript_variant,intron_variant,coding_sequence_variant,non_coding_transcript_variant,synonymous_variant
rs1584878602	genic_upstream_transcript_variant,intron_variant,coding_sequence_variant,non_coding_transcript_variant,missense_variant
rs1584878668	genic_upstream_transcript_variant,intron_variant,coding_sequence_variant,non_coding_transcript_variant,missense_variant
rs1584914144	non_coding_transcript_variant,coding_sequence_variant,missense_variant
rs1584914185	non_coding_transcript_variant,coding_sequence_variant,missense_variant
rs1584914259	non_coding_transcript_variant,coding_sequence_variant,missense_variant
rs1584914261	non_coding_transcript_variant,coding_sequence_variant,missense_variant
rs1584914359	non_coding_transcript_variant,coding_sequence_variant,missense_variant
rs1584914382	non_coding_transcript_variant,coding_sequence_variant,missense_variant
rs1584914402	non_coding_transcript_variant,coding_sequence_variant,missense_variant
rs1584914409	splice_donor_variant
rs1584921898	splice_acceptor_variant
rs1584921909	splice_acceptor_variant
rs1584921949	non_coding_transcript_variant,synonymous_variant,coding_sequence_variant
rs1584922011	non_coding_transcript_variant,coding_sequence_variant,missense_variant
rs1584922045	non_coding_transcript_variant,coding_sequence_variant,missense_variant
rs1584922200	non_coding_transcript_variant,coding_sequence_variant,missense_variant
rs1584922241	splice_donor_variant
rs1584923073	non_coding_transcript_variant,coding_sequence_variant,missense_variant
rs1584923080	non_coding_transcript_variant,coding_sequence_variant,missense_variant
rs1584923096	non_coding_transcript_variant,synonymous_variant,coding_sequence_variant
rs1584923126	non_coding_transcript_variant,coding_sequence_variant,missense_variant
rs1584923132	non_coding_transcript_variant,coding_sequence_variant,stop_gained
rs1584923162	non_coding_transcript_variant,coding_sequence_variant,missense_variant
rs1584923176	non_coding_transcript_variant,coding_sequence_variant,missense_variant
rs1584923365	non_coding_transcript_variant,synonymous_variant,coding_sequence_variant
rs1584923423	non_coding_transcript_variant,coding_sequence_variant,missense_variant
rs1584923517	non_coding_transcript_variant,coding_sequence_variant,missense_variant
rs1584939128	non_coding_transcript_variant,coding_sequence_variant,missense_variant
rs1584939151	non_coding_transcript_variant,synonymous_variant,coding_sequence_variant
rs1584939204	non_coding_transcript_variant,coding_sequence_variant,missense_variant
rs1584941260	non_coding_transcript_variant,synonymous_variant,coding_sequence_variant
rs1584941533	non_coding_transcript_variant,coding_sequence_variant,missense_variant
rs1584941563	non_coding_transcript_variant,coding_sequence_variant,missense_variant
rs1584941649	non_coding_transcript_variant,synonymous_variant,coding_sequence_variant
rs1584941747	non_coding_transcript_variant,coding_sequence_variant,missense_variant
rs1584941772	non_coding_transcript_variant,coding_sequence_variant,missense_variant
rs1584941818	non_coding_transcript_variant,synonymous_variant,coding_sequence_variant
rs1584941830	non_coding_transcript_variant,coding_sequence_variant,missense_variant
rs1584941903	intron_variant
rs1584942689	non_coding_transcript_variant,synonymous_variant,coding_sequence_variant
rs1584942785	non_coding_transcript_variant,synonymous_variant,coding_sequence_variant
rs1584942863	non_coding_transcript_variant,coding_sequence_variant,missense_variant
rs1584943653	missense_variant,intron_variant,coding_sequence_variant
rs1584943719	synonymous_variant,intron_variant,coding_sequence_variant
rs1584946843	non_coding_transcript_variant,synonymous_variant,coding_sequence_variant
rs1584946860	non_coding_transcript_variant,synonymous_variant,coding_sequence_variant
rs1584946893	non_coding_transcript_variant,synonymous_variant,coding_sequence_variant
rs1584947043	non_coding_transcript_variant,coding_sequence_variant,missense_variant
rs1584947055	non_coding_transcript_variant,synonymous_variant,coding_sequence_variant
rs1584947097	non_coding_transcript_variant,coding_sequence_variant,missense_variant

rs1584953171	non_coding_transcript_variant,coding_sequence_variant,missense_variant
rs1584953249	non_coding_transcript_variant,coding_sequence_variant,missense_variant
rs1584953276	non_coding_transcript_variant,coding_sequence_variant,missense_variant
rs1584953336	non_coding_transcript_variant,synonymous_variant,coding_sequence_variant,missense_variant
rs1584955001	synonymous_variant,non_coding_transcript_variant,genic_downstream_transcript_variant,coding_sequence_variant
rs1584955059	genic_downstream_transcript_variant,intron_variant
rs1584955273	synonymous_variant,non_coding_transcript_variant,genic_downstream_transcript_variant,coding_sequence_variant
rs1584955330	non_coding_transcript_variant,genic_downstream_transcript_variant,coding_sequence_variant,missense_variant
rs1584955347	non_coding_transcript_variant,genic_downstream_transcript_variant,coding_sequence_variant,missense_variant
rs1584957663	non_coding_transcript_variant,genic_downstream_transcript_variant,coding_sequence_variant,missense_variant
rs1584957697	non_coding_transcript_variant,genic_downstream_transcript_variant,coding_sequence_variant,missense_variant
rs1584957724	non_coding_transcript_variant,genic_downstream_transcript_variant,coding_sequence_variant,missense_variant
rs1584957836	synonymous_variant,non_coding_transcript_variant,genic_downstream_transcript_variant,coding_sequence_variant
rs1584957841	non_coding_transcript_variant,genic_downstream_transcript_variant,coding_sequence_variant,missense_variant
rs1584957891	synonymous_variant,non_coding_transcript_variant,genic_downstream_transcript_variant,coding_sequence_variant
rs1584957937	non_coding_transcript_variant,genic_downstream_transcript_variant,coding_sequence_variant,missense_variant
rs1584957997	non_coding_transcript_variant,genic_downstream_transcript_variant,coding_sequence_variant,missense_variant
rs1584959967	synonymous_variant,non_coding_transcript_variant,genic_downstream_transcript_variant,coding_sequence_variant
rs1584959989	synonymous_variant,non_coding_transcript_variant,genic_downstream_transcript_variant,coding_sequence_variant
rs1584960002	non_coding_transcript_variant,genic_downstream_transcript_variant,coding_sequence_variant,missense_variant
rs1584961122	inframe_deletion,non_coding_transcript_variant,genic_downstream_transcript_variant,coding_sequence_variant
rs1584961300	non_coding_transcript_variant,genic_downstream_transcript_variant,coding_sequence_variant,missense_variant
rs1584964218	synonymous_variant,non_coding_transcript_variant,genic_downstream_transcript_variant,coding_sequence_variant
rs1584964221	non_coding_transcript_variant,genic_downstream_transcript_variant,coding_sequence_variant,missense_variant
rs1584964312	synonymous_variant,non_coding_transcript_variant,genic_downstream_transcript_variant,coding_sequence_variant
rs1584964366	genic_downstream_transcript_variant,intron_variant
rs1584965464	non_coding_transcript_variant,genic_downstream_transcript_variant,coding_sequence_variant,missense_variant
rs1584965488	synonymous_variant,non_coding_transcript_variant,genic_downstream_transcript_variant,coding_sequence_variant
rs1584965524	synonymous_variant,non_coding_transcript_variant,genic_downstream_transcript_variant,coding_sequence_variant
rs1584965536	non_coding_transcript_variant,genic_downstream_transcript_variant,coding_sequence_variant,missense_variant
rs1584965646	synonymous_variant,non_coding_transcript_variant,genic_downstream_transcript_variant,coding_sequence_variant
rs1584965685	synonymous_variant,non_coding_transcript_variant,genic_downstream_transcript_variant,coding_sequence_variant
rs1584977801	genic_downstream_transcript_variant,intron_variant
rs1584977880	synonymous_variant,non_coding_transcript_variant,genic_downstream_transcript_variant,coding_sequence_variant
rs1584977945	non_coding_transcript_variant,genic_downstream_transcript_variant,coding_sequence_variant,missense_variant
rs1584977952	non_coding_transcript_variant,genic_downstream_transcript_variant,coding_sequence_variant,missense_variant
rs1584977995	synonymous_variant,non_coding_transcript_variant,genic_downstream_transcript_variant,coding_sequence_variant
rs1584977997	synonymous_variant,non_coding_transcript_variant,genic_downstream_transcript_variant,coding_sequence_variant
rs1584978021	synonymous_variant,non_coding_transcript_variant,genic_downstream_transcript_variant,coding_sequence_variant
rs1584978029	non_coding_transcript_variant,genic_downstream_transcript_variant,coding_sequence_variant,missense_variant
rs1584978266	non_coding_transcript_variant,genic_downstream_transcript_variant,coding_sequence_variant,missense_variant
rs1584978394	non_coding_transcript_variant,genic_downstream_transcript_variant,frameshift_variant,coding_sequence_variant
rs1584978510	non_coding_transcript_variant,genic_downstream_transcript_variant,coding_sequence_variant,missense_variant
rs1584978594	non_coding_transcript_variant,genic_downstream_transcript_variant,coding_sequence_variant,missense_variant
rs1584978746	synonymous_variant,non_coding_transcript_variant,genic_downstream_transcript_variant,coding_sequence_variant
rs1791464303	coding_sequence_variant,missense_variant,intron_variant,non_coding_transcript_variant,genic_upstream_transcript_variant
rs1791466070	coding_sequence_variant,missense_variant,intron_variant,non_coding_transcript_variant,genic_upstream_transcript_variant
rs1791468662	coding_sequence_variant,missense_variant,intron_variant,non_coding_transcript_variant,genic_upstream_transcript_variant
rs1791471735	coding_sequence_variant,missense_variant,intron_variant,non_coding_transcript_variant,genic_upstream_transcript_variant
rs1791473262	coding_sequence_variant,missense_variant,intron_variant,non_coding_transcript_variant,genic_upstream_transcript_variant
rs1791473592	coding_sequence_variant,missense_variant,intron_variant,non_coding_transcript_variant,genic_upstream_transcript_variant
rs1791473684	coding_sequence_variant,missense_variant,intron_variant,non_coding_transcript_variant,genic_upstream_transcript_variant
rs1791474714	coding_sequence_variant,missense_variant,intron_variant,non_coding_transcript_variant,genic_upstream_transcript_variant
rs1791477416	coding_sequence_variant,missense_variant,intron_variant,non_coding_transcript_variant,genic_upstream_transcript_variant
rs1791478036	coding_sequence_variant,missense_variant,intron_variant,non_coding_transcript_variant,genic_upstream_transcript_variant
rs1791478119	coding_sequence_variant,missense_variant,intron_variant,non_coding_transcript_variant,genic_upstream_transcript_variant
rs1791478961	coding_sequence_variant,missense_variant,intron_variant,non_coding_transcript_variant,genic_upstream_transcript_variant
rs1791489495	coding_sequence_variant,missense_variant,intron_variant,non_coding_transcript_variant,genic_upstream_transcript_variant
rs1791489630	coding_sequence_variant,missense_variant,intron_variant,non_coding_transcript_variant,genic_upstream_transcript_variant
rs1791490183	coding_sequence_variant,missense_variant,intron_variant,non_coding_transcript_variant,genic_upstream_transcript_variant
rs1791492133	coding_sequence_variant,missense_variant,intron_variant,non_coding_transcript_variant,genic_upstream_transcript_variant
rs1791495307	coding_sequence_variant,missense_variant,intron_variant,non_coding_transcript_variant,genic_upstream_transcript_variant
rs1791500546	coding_sequence_variant,missense_variant,intron_variant,non_coding_transcript_variant,genic_upstream_transcript_variant
rs1791501640	coding_sequence_variant,inframe_deletion,intron_variant,non_coding_transcript_variant,genic_upstream_transcript_variant
rs1791501952	coding_sequence_variant,missense_variant,intron_variant,non_coding_transcript_variant,genic_upstream_transcript_variant
rs1791504987	coding_sequence_variant,missense_variant,intron_variant,non_coding_transcript_variant,genic_upstream_transcript_variant
rs1791505194	coding_sequence_variant,missense_variant,intron_variant,non_coding_transcript_variant,genic_upstream_transcript_variant
rs1791506647	coding_sequence_variant,missense_variant,intron_variant,non_coding_transcript_variant,genic_upstream_transcript_variant
rs1791509642	coding_sequence_variant,intron_variant,stop_gained,non_coding_transcript_variant,genic_upstream_transcript_variant
rs1791511003	coding_sequence_variant,missense_variant,intron_variant,non_coding_transcript_variant,genic_upstream_transcript_variant
rs1791511601	coding_sequence_variant,missense_variant,intron_variant,non_coding_transcript_variant,genic_upstream_transcript_variant
rs1791512225	coding_sequence_variant,missense_variant,intron_variant,non_coding_transcript_variant,genic_upstream_transcript_variant
rs1791513686	coding_sequence_variant,missense_variant,intron_variant,non_coding_transcript_variant,genic_upstream_transcript_variant
rs1791516755	coding_sequence_variant,missense_variant,intron_variant,non_coding_transcript_variant,genic_upstream_transcript_variant

rs1791519202	coding_sequence_variant,missense_variant,intron_variant,non_coding_transcript_variant,genic_upstream_transcript_variant
rs1791521654	coding_sequence_variant,missense_variant,intron_variant,non_coding_transcript_variant,genic_upstream_transcript_variant
rs1791522873	coding_sequence_variant,missense_variant,intron_variant,non_coding_transcript_variant,genic_upstream_transcript_variant
rs1791523699	coding_sequence_variant,missense_variant,intron_variant,non_coding_transcript_variant,genic_upstream_transcript_variant
rs1791523904	coding_sequence_variant,missense_variant,intron_variant,non_coding_transcript_variant,genic_upstream_transcript_variant
rs1791526414	coding_sequence_variant,intron_variant,synonymous_variant,non_coding_transcript_variant,genic_upstream_transcript_variant
rs1793009396	5_prime_UTR_variant,non_coding_transcript_variant,coding_sequence_variant,missense_variant
rs1793012257	5_prime_UTR_variant,non_coding_transcript_variant,coding_sequence_variant,missense_variant
rs1793014361	5_prime_UTR_variant,non_coding_transcript_variant,coding_sequence_variant,missense_variant
rs1793016615	non_coding_transcript_variant,coding_sequence_variant,missense_variant
rs1793017472	non_coding_transcript_variant,coding_sequence_variant,missense_variant
rs1793017605	non_coding_transcript_variant,coding_sequence_variant,missense_variant
rs1793018926	non_coding_transcript_variant,coding_sequence_variant,missense_variant
rs1793019219	non_coding_transcript_variant,coding_sequence_variant,missense_variant
rs1793019649	non_coding_transcript_variant,coding_sequence_variant,missense_variant
rs1793020405	non_coding_transcript_variant,coding_sequence_variant,missense_variant
rs1793020534	frameshift_variant,non_coding_transcript_variant,coding_sequence_variant
rs1793022563	intron_variant
rs1793378040	non_coding_transcript_variant,coding_sequence_variant,missense_variant
rs1793379533	non_coding_transcript_variant,coding_sequence_variant,missense_variant
rs1793380035	non_coding_transcript_variant,coding_sequence_variant,missense_variant
rs1793380587	non_coding_transcript_variant,coding_sequence_variant,missense_variant
rs1793383674	non_coding_transcript_variant,coding_sequence_variant,missense_variant
rs1793387054	intron_variant
rs1793387165	intron_variant
rs1793418567	non_coding_transcript_variant,coding_sequence_variant,missense_variant
rs1793420216	non_coding_transcript_variant,coding_sequence_variant,missense_variant
rs1793421596	non_coding_transcript_variant,coding_sequence_variant,missense_variant
rs1793422040	non_coding_transcript_variant,coding_sequence_variant,missense_variant
rs1793422444	non_coding_transcript_variant,coding_sequence_variant,missense_variant
rs1793422722	non_coding_transcript_variant,coding_sequence_variant,missense_variant
rs1793429265	non_coding_transcript_variant,coding_sequence_variant,missense_variant
rs1794140471	coding_sequence_variant,non_coding_transcript_variant,inframe_indel
rs1794141895	coding_sequence_variant,non_coding_transcript_variant,missense_variant
rs1794144964	coding_sequence_variant,non_coding_transcript_variant,missense_variant
rs1794145066	coding_sequence_variant,non_coding_transcript_variant,missense_variant
rs1794219866	coding_sequence_variant,non_coding_transcript_variant,synonymous_variant
rs1794220084	coding_sequence_variant,non_coding_transcript_variant,missense_variant
rs1794220294	coding_sequence_variant,non_coding_transcript_variant,missense_variant
rs1794236479	coding_sequence_variant,non_coding_transcript_variant,missense_variant
rs1794244388	coding_sequence_variant,non_coding_transcript_variant,missense_variant
rs1794245712	coding_sequence_variant,non_coding_transcript_variant,frameshift_variant
rs1794246284	coding_sequence_variant,non_coding_transcript_variant,missense_variant
rs1794246923	coding_sequence_variant,non_coding_transcript_variant,missense_variant
rs1794247110	intron_variant
rs1794272939	coding_sequence_variant,non_coding_transcript_variant,missense_variant
rs1794273981	coding_sequence_variant,non_coding_transcript_variant,missense_variant
rs1794276426	coding_sequence_variant,non_coding_transcript_variant,missense_variant
rs1794278950	coding_sequence_variant,non_coding_transcript_variant,missense_variant
rs1794279427	coding_sequence_variant,non_coding_transcript_variant,missense_variant
rs1794304820	intron_variant
rs1794309303	coding_sequence_variant,intron_variant,missense_variant
rs1794309783	coding_sequence_variant,intron_variant,missense_variant
rs1794310272	coding_sequence_variant,intron_variant,missense_variant
rs1794311390	coding_sequence_variant,intron_variant,missense_variant
rs1794313115	coding_sequence_variant,intron_variant,missense_variant
rs1794313226	coding_sequence_variant,intron_variant,missense_variant
rs1794461185	coding_sequence_variant,non_coding_transcript_variant,missense_variant
rs1794465668	coding_sequence_variant,non_coding_transcript_variant,missense_variant
rs1794468017	coding_sequence_variant,non_coding_transcript_variant,missense_variant
rs1794470895	coding_sequence_variant,non_coding_transcript_variant,missense_variant
rs1794471154	coding_sequence_variant,non_coding_transcript_variant,missense_variant
rs1794474122	coding_sequence_variant,non_coding_transcript_variant,missense_variant
rs1794474295	coding_sequence_variant,non_coding_transcript_variant,missense_variant
rs1794767589	coding_sequence_variant,non_coding_transcript_variant,missense_variant
rs1794830011	missense_variant,non_coding_transcript_variant,coding_sequence_variant,genic_downstream_transcript_variant
rs1794830159	missense_variant,non_coding_transcript_variant,coding_sequence_variant,genic_downstream_transcript_variant
rs1794832354	missense_variant,non_coding_transcript_variant,coding_sequence_variant,genic_downstream_transcript_variant
rs1794833108	missense_variant,non_coding_transcript_variant,coding_sequence_variant,genic_downstream_transcript_variant
rs1794833626	missense_variant,non_coding_transcript_variant,coding_sequence_variant,genic_downstream_transcript_variant
rs1794834628	missense_variant,non_coding_transcript_variant,coding_sequence_variant,genic_downstream_transcript_variant
rs1794836064	frameshift_variant,non_coding_transcript_variant,coding_sequence_variant,genic_downstream_transcript_variant
rs1794847178	missense_variant,non_coding_transcript_variant,coding_sequence_variant,genic_downstream_transcript_variant
rs1794847447	missense_variant,non_coding_transcript_variant,coding_sequence_variant,genic_downstream_transcript_variant

rs1794847963	missense_variant,non_coding_transcript_variant,coding_sequence_variant,genic_downstream_transcript_variant
rs1794848174	missense_variant,non_coding_transcript_variant,coding_sequence_variant,genic_downstream_transcript_variant
rs1794849768	missense_variant,non_coding_transcript_variant,coding_sequence_variant,genic_downstream_transcript_variant
rs1794850672	missense_variant,non_coding_transcript_variant,coding_sequence_variant,genic_downstream_transcript_variant
rs1794851181	missense_variant,non_coding_transcript_variant,coding_sequence_variant,genic_downstream_transcript_variant
rs1794851612	missense_variant,non_coding_transcript_variant,coding_sequence_variant,genic_downstream_transcript_variant
rs1794851763	splice_donor_variant,genic_downstream_transcript_variant
rs1794945602	missense_variant,non_coding_transcript_variant,coding_sequence_variant,genic_downstream_transcript_variant
rs1794946568	missense_variant,non_coding_transcript_variant,coding_sequence_variant,genic_downstream_transcript_variant
rs1794947477	missense_variant,non_coding_transcript_variant,coding_sequence_variant,genic_downstream_transcript_variant
rs1794948966	missense_variant,non_coding_transcript_variant,coding_sequence_variant,genic_downstream_transcript_variant
rs1794949125	missense_variant,non_coding_transcript_variant,coding_sequence_variant,genic_downstream_transcript_variant
rs1794950215	missense_variant,non_coding_transcript_variant,coding_sequence_variant,genic_downstream_transcript_variant
rs1794951633	missense_variant,non_coding_transcript_variant,coding_sequence_variant,genic_downstream_transcript_variant
rs1794953455	missense_variant,non_coding_transcript_variant,coding_sequence_variant,genic_downstream_transcript_variant
rs1794954978	missense_variant,non_coding_transcript_variant,coding_sequence_variant,genic_downstream_transcript_variant
rs1794955289	missense_variant,non_coding_transcript_variant,coding_sequence_variant,genic_downstream_transcript_variant
rs1794955378	missense_variant,non_coding_transcript_variant,coding_sequence_variant,genic_downstream_transcript_variant
rs1794955894	intron_variant,genic_downstream_transcript_variant
rs1795029105	missense_variant,non_coding_transcript_variant,coding_sequence_variant,genic_downstream_transcript_variant
rs1795029901	missense_variant,non_coding_transcript_variant,coding_sequence_variant,genic_downstream_transcript_variant
rs1795030662	missense_variant,non_coding_transcript_variant,coding_sequence_variant,genic_downstream_transcript_variant
rs1795065701	missense_variant,non_coding_transcript_variant,coding_sequence_variant,genic_downstream_transcript_variant
rs1795065976	missense_variant,non_coding_transcript_variant,coding_sequence_variant,genic_downstream_transcript_variant
rs1795069790	missense_variant,non_coding_transcript_variant,coding_sequence_variant,genic_downstream_transcript_variant
rs1795070394	missense_variant,non_coding_transcript_variant,coding_sequence_variant,genic_downstream_transcript_variant
rs1795070539	missense_variant,non_coding_transcript_variant,coding_sequence_variant,genic_downstream_transcript_variant
rs1795070664	missense_variant,non_coding_transcript_variant,coding_sequence_variant,genic_downstream_transcript_variant
rs1795071435	missense_variant,non_coding_transcript_variant,coding_sequence_variant,genic_downstream_transcript_variant
rs1795071897	missense_variant,non_coding_transcript_variant,coding_sequence_variant,genic_downstream_transcript_variant
rs1795073205	non_coding_transcript_variant,coding_sequence_variant,stop_gained,genic_downstream_transcript_variant
rs1795074297	missense_variant,non_coding_transcript_variant,coding_sequence_variant,genic_downstream_transcript_variant
rs1795171767	missense_variant,non_coding_transcript_variant,coding_sequence_variant,genic_downstream_transcript_variant
rs1795213262	non_coding_transcript_variant,coding_sequence_variant,stop_gained,genic_downstream_transcript_variant
rs1795214265	missense_variant,non_coding_transcript_variant,coding_sequence_variant,genic_downstream_transcript_variant
rs1795214615	splice_donor_variant,genic_downstream_transcript_variant
rs1795654625	splice_acceptor_variant,genic_downstream_transcript_variant
rs1795655626	missense_variant,non_coding_transcript_variant,coding_sequence_variant,genic_downstream_transcript_variant
rs1795657629	missense_variant,non_coding_transcript_variant,coding_sequence_variant,genic_downstream_transcript_variant
rs1795658722	missense_variant,non_coding_transcript_variant,coding_sequence_variant,genic_downstream_transcript_variant
rs1795661591	missense_variant,non_coding_transcript_variant,coding_sequence_variant,genic_downstream_transcript_variant
rs1795662723	missense_variant,non_coding_transcript_variant,coding_sequence_variant,genic_downstream_transcript_variant
rs1795668471	genic_downstream_transcript_variant,non_coding_transcript_variant,3_prime_UTR_variant
rs1795669540	genic_downstream_transcript_variant,non_coding_transcript_variant,3_prime_UTR_variant
rs1795682838	genic_downstream_transcript_variant,non_coding_transcript_variant,3_prime_UTR_variant
rs1795687373	genic_downstream_transcript_variant,non_coding_transcript_variant,3_prime_UTR_variant
rs1795712500	genic_downstream_transcript_variant,non_coding_transcript_variant,3_prime_UTR_variant
rs1795730503	genic_downstream_transcript_variant,non_coding_transcript_variant,3_prime_UTR_variant
rs1795737615	genic_downstream_transcript_variant,non_coding_transcript_variant,3_prime_UTR_variant
rs1796009308	5_prime_UTR_variant,genic_upstream_transcript_variant,non_coding_transcript_variant,intron_variant
rs1796012626	5_prime_UTR_variant,genic_upstream_transcript_variant,non_coding_transcript_variant,intron_variant
rs1621	non_coding_transcript_variant,3_prime_UTR_variant,genic_downstream_transcript_variant
rs1621	non_coding_transcript_variant,3_prime_UTR_variant,genic_downstream_transcript_variant
rs1621	non_coding_transcript_variant,3_prime_UTR_variant,genic_downstream_transcript_variant
rs1621	non_coding_transcript_variant,3_prime_UTR_variant,genic_downstream_transcript_variant
rs1621	non_coding_transcript_variant,3_prime_UTR_variant,genic_downstream_transcript_variant
rs6566	non_coding_transcript_variant,3_prime_UTR_variant,genic_downstream_transcript_variant
rs6566	non_coding_transcript_variant,3_prime_UTR_variant,genic_downstream_transcript_variant
rs6566	non_coding_transcript_variant,3_prime_UTR_variant,genic_downstream_transcript_variant
rs6566	non_coding_transcript_variant,3_prime_UTR_variant,genic_downstream_transcript_variant
rs6566	non_coding_transcript_variant,3_prime_UTR_variant,genic_downstream_transcript_variant
rs14456	non_coding_transcript_variant,3_prime_UTR_variant,genic_downstream_transcript_variant
rs14456	non_coding_transcript_variant,3_prime_UTR_variant,genic_downstream_transcript_variant
rs14456	non_coding_transcript_variant,3_prime_UTR_variant,genic_downstream_transcript_variant
rs41736	non_coding_transcript_variant,coding_sequence_variant,missense_variant,synonymous_variant,genic_downstream_transcript_variant
rs41736	non_coding_transcript_variant,coding_sequence_variant,missense_variant,synonymous_variant,genic_downstream_transcript_variant
rs41737	genic_downstream_transcript_variant,non_coding_transcript_variant,synonymous_variant,coding_sequence_variant
rs41737	genic_downstream_transcript_variant,non_coding_transcript_variant,synonymous_variant,coding_sequence_variant
rs41738	non_coding_transcript_variant,3_prime_UTR_variant,genic_downstream_transcript_variant
rs41738	non_coding_transcript_variant,3_prime_UTR_variant,genic_downstream_transcript_variant
rs41739	non_coding_transcript_variant,3_prime_UTR_variant,genic_downstream_transcript_variant
rs41739	non_coding_transcript_variant,3_prime_UTR_variant,genic_downstream_transcript_variant
rs41739	non_coding_transcript_variant,3_prime_UTR_variant,genic_downstream_transcript_variant
rs1129355	non_coding_transcript_variant,3_prime_UTR_variant,genic_downstream_transcript_variant

rs2023748	genic_downstream_transcript_variant,non_coding_transcript_variant,synonymous_variant,coding_sequence_variant
rs2023748	genic_downstream_transcript_variant,non_coding_transcript_variant,synonymous_variant,coding_sequence_variant
rs13223756	coding_sequence_variant,synonymous_variant,non_coding_transcript_variant
rs17138978	intron_variant
rs55985569	genic_upstream_transcript_variant,missense_variant,synonymous_variant,intron_variant,non_coding_transcript_variant,coding_sequence_variant
rs57349036	intron_variant
rs57349036	intron_variant
rs57349036	intron_variant
rs57349036	intron_variant
rs57349036	intron_variant
rs57349036	intron_variant
rs57349036	intron_variant
rs57349036	intron_variant
rs57349036	intron_variant
rs73469198	intron_variant
rs73469198	intron_variant
rs112241458	intron_variant
rs112241458	intron_variant
rs112241458	intron_variant
rs112241458	intron_variant
rs121913243	genic_downstream_transcript_variant,non_coding_transcript_variant,missense_variant,coding_sequence_variant
rs121913246	genic_downstream_transcript_variant,non_coding_transcript_variant,missense_variant,coding_sequence_variant
rs121913668	genic_downstream_transcript_variant,non_coding_transcript_variant,missense_variant,coding_sequence_variant
rs121913669	genic_downstream_transcript_variant,non_coding_transcript_variant,missense_variant,coding_sequence_variant
rs121913670	genic_downstream_transcript_variant,non_coding_transcript_variant,missense_variant,coding_sequence_variant
rs121913671	genic_downstream_transcript_variant,non_coding_transcript_variant,missense_variant,coding_sequence_variant
rs121913673	genic_downstream_transcript_variant,non_coding_transcript_variant,missense_variant,coding_sequence_variant
rs146651797	missense_variant,non_coding_transcript_variant,genic_downstream_transcript_variant,synonymous_variant,coding_sequence_variant
rs587780736	intron_variant
rs587780736	intron_variant
rs749738523	missense_variant,genic_upstream_transcript_variant,intron_variant,non_coding_transcript_variant,coding_sequence_variant
rs763344951	genic_upstream_transcript_variant,intron_variant,non_coding_transcript_variant,coding_sequence_variant,missense_variant
rs764960693	non_coding_transcript_variant,coding_sequence_variant,missense_variant
rs765244598	non_coding_transcript_variant,coding_sequence_variant,missense_variant
rs766900241	intron_variant
rs770202229	non_coding_transcript_variant,genic_upstream_transcript_variant,coding_sequence_variant,synonymous_variant,intron_variant
rs886061941	intron_variant,genic_upstream_transcript_variant,non_coding_transcript_variant,5_prime_UTR_variant
rs886061946	3_prime_UTR_variant,genic_downstream_transcript_variant,non_coding_transcript_variant
rs886061950	3_prime_UTR_variant,genic_downstream_transcript_variant,non_coding_transcript_variant
rs886061951	3_prime_UTR_variant,genic_downstream_transcript_variant,non_coding_transcript_variant
rs16945	downstream_transcript_variant,500B_downstream_variant
rs28841	intron_variant
rs38839	2KB_upstream_variant,intron_variant,upstream_transcript_variant,genic_upstream_transcript_variant
rs38840	genic_upstream_transcript_variant,intron_variant
rs38841	genic_upstream_transcript_variant,intron_variant
rs38842	genic_upstream_transcript_variant,intron_variant
rs38843	genic_upstream_transcript_variant,intron_variant
rs38844	genic_upstream_transcript_variant,intron_variant
rs38845	genic_upstream_transcript_variant,intron_variant
rs38846	genic_upstream_transcript_variant,intron_variant
rs38847	genic_upstream_transcript_variant,intron_variant
rs38848	genic_upstream_transcript_variant,intron_variant
rs38849	genic_upstream_transcript_variant,intron_variant
rs38850	genic_upstream_transcript_variant,intron_variant
rs38851	genic_upstream_transcript_variant,intron_variant
rs38852	genic_upstream_transcript_variant,intron_variant
rs38853	genic_upstream_transcript_variant,intron_variant
rs38854	genic_upstream_transcript_variant,intron_variant
rs38855	genic_upstream_transcript_variant,intron_variant
rs38856	genic_upstream_transcript_variant,intron_variant
rs38857	intron_variant
rs38858	intron_variant
rs38859	intron_variant
rs38860	intron_variant
rs39747	genic_upstream_transcript_variant,intron_variant
rs39748	genic_upstream_transcript_variant,intron_variant
rs39749	genic_upstream_transcript_variant,intron_variant
rs39750	upstream_transcript_variant,genic_upstream_transcript_variant,intron_variant
rs40238	genic_upstream_transcript_variant,intron_variant
rs40239	genic_upstream_transcript_variant,intron_variant
rs41735	genic_downstream_transcript_variant,intron_variant
rs41741	downstream_transcript_variant,500B_downstream_variant
rs168686	genic_downstream_transcript_variant,intron_variant
rs183642	genic_downstream_transcript_variant,intron_variant

rs184953	2KB_upstream_variant,intron_variant,upstream_transcript_variant,genic_upstream_transcript_variant
rs193686	genic_downstream_transcript_variant,intron_variant
rs193687	genic_downstream_transcript_variant,intron_variant
rs193688	genic_downstream_transcript_variant,intron_variant
rs714180	genic_upstream_transcript_variant,intron_variant
rs722134	genic_upstream_transcript_variant,intron_variant
rs763538	genic_upstream_transcript_variant,intron_variant
rs916941	genic_downstream_transcript_variant,intron_variant
rs1476454	genic_upstream_transcript_variant,intron_variant
rs1858829	genic_upstream_transcript_variant,intron_variant
rs2073560	genic_downstream_transcript_variant,intron_variant
rs2237708	genic_upstream_transcript_variant,intron_variant
rs2237709	genic_upstream_transcript_variant,intron_variant
rs2237710	genic_upstream_transcript_variant,intron_variant
rs2237711	genic_upstream_transcript_variant,intron_variant
rs2237712	genic_upstream_transcript_variant,intron_variant
rs2237713	genic_upstream_transcript_variant,intron_variant
rs2237714	genic_upstream_transcript_variant,intron_variant
rs2237715	intron_variant
rs2237716	intron_variant
rs2237717	intron_variant
rs2283051	genic_upstream_transcript_variant,intron_variant
rs2283052	genic_upstream_transcript_variant,intron_variant
rs2283053	genic_downstream_transcript_variant,intron_variant
rs2299433	genic_upstream_transcript_variant,intron_variant
rs2299434	genic_upstream_transcript_variant,intron_variant
rs2299435	genic_upstream_transcript_variant,intron_variant
rs2299436	genic_upstream_transcript_variant,intron_variant
rs2299437	genic_upstream_transcript_variant,intron_variant
rs2299438	genic_upstream_transcript_variant,intron_variant
rs2299439	genic_downstream_transcript_variant,intron_variant
rs2299440	genic_downstream_transcript_variant,intron_variant
rs2402118	genic_downstream_transcript_variant,intron_variant
rs2896191	genic_upstream_transcript_variant,intron_variant
rs3031564	2KB_upstream_variant,intron_variant,upstream_transcript_variant,genic_upstream_transcript_variant
rs3220187	intron_variant
rs3807996	intron_variant
rs3807997	genic_downstream_transcript_variant,intron_variant
rs3840635	genic_upstream_transcript_variant,intron_variant
rs3993905	intron_variant
rs4330631	genic_upstream_transcript_variant,intron_variant
rs4360233	intron_variant
rs4566974	genic_upstream_transcript_variant,intron_variant
rs4615490	genic_upstream_transcript_variant,intron_variant
rs4727836	intron_variant
rs5886835	intron_variant
rs6466592	intron_variant
rs6945273	intron_variant
rs6946538	intron_variant
rs6947629	genic_downstream_transcript_variant,intron_variant
rs6949201	intron_variant
rs6951311	genic_downstream_transcript_variant,intron_variant
rs6954316	genic_upstream_transcript_variant,intron_variant
rs6954327	intron_variant
rs6965570	genic_upstream_transcript_variant,intron_variant
rs6966012	genic_downstream_transcript_variant,intron_variant
rs6969853	intron_variant
rs6978135	genic_downstream_transcript_variant,intron_variant
rs6978257	genic_downstream_transcript_variant,intron_variant
rs7785933	genic_upstream_transcript_variant,intron_variant
rs7798983	genic_upstream_transcript_variant,intron_variant
rs7804148	intron_variant
rs7804632	intron_variant
rs7804778	intron_variant
rs7805089	genic_upstream_transcript_variant,intron_variant
rs7810164	genic_upstream_transcript_variant,intron_variant
rs9640772	genic_upstream_transcript_variant,intron_variant
rs9641562	genic_upstream_transcript_variant,intron_variant
rs9641563	genic_upstream_transcript_variant,intron_variant
rs9641564	genic_upstream_transcript_variant,intron_variant
rs9641565	genic_downstream_transcript_variant,intron_variant
rs9770051	genic_downstream_transcript_variant,intron_variant
rs10215153	intron_variant
rs10223961	genic_upstream_transcript_variant,intron_variant

rs10232307	intron_variant
rs10234854	genic_upstream_transcript_variant,intron_variant
rs10235944	intron_variant
rs10240033	genic_upstream_transcript_variant,intron_variant
rs10243024	genic_upstream_transcript_variant,intron_variant
rs10243964	genic_downstream_transcript_variant,intron_variant
rs10244333	intron_variant
rs10244916	intron_variant
rs10246585	genic_upstream_transcript_variant,intron_variant
rs10248537	genic_upstream_transcript_variant,intron_variant
rs10258960	genic_upstream_transcript_variant,intron_variant
rs10262620	intron_variant
rs10266869	intron_variant
rs10271561	genic_upstream_transcript_variant,intron_variant
rs10278415	intron_variant
rs10279782	intron_variant
rs10435378	genic_downstream_transcript_variant,intron_variant
rs10487353	genic_upstream_transcript_variant,intron_variant
rs10674901	intron_variant
rs10719237	intron_variant
rs11404514	genic_upstream_transcript_variant,intron_variant
rs11431559	genic_downstream_transcript_variant,intron_variant
rs11763782	intron_variant
rs11767567	intron_variant,genic_upstream_transcript_variant
rs11770163	intron_variant,genic_downstream_transcript_variant
rs11979548	intron_variant
rs12386593	intron_variant
rs12530841	intron_variant,genic_upstream_transcript_variant
rs12533223	intron_variant
rs12535996	intron_variant,genic_upstream_transcript_variant
rs12539654	2KB_upstream_variant,intron_variant,genic_upstream_transcript_variant,upstream_transcript_variant
rs12666656	intron_variant,genic_upstream_transcript_variant
rs13221751	intron_variant,genic_downstream_transcript_variant
rs13221767	intron_variant,genic_downstream_transcript_variant
rs13222452	non_coding_transcript_variant,genic_upstream_transcript_variant,intron_variant,5_prime_UTR_variant
rs13229115	intron_variant,genic_upstream_transcript_variant
rs13232154	2KB_upstream_variant,genic_upstream_transcript_variant,upstream_transcript_variant,intron_variant
rs13232252	2KB_upstream_variant,genic_upstream_transcript_variant,upstream_transcript_variant,intron_variant
rs13234971	intron_variant,genic_downstream_transcript_variant
rs13235174	non_coding_transcript_variant,genic_upstream_transcript_variant,intron_variant,5_prime_UTR_variant
rs13239139	intron_variant,genic_downstream_transcript_variant
rs13309274	intron_variant
rs13309275	intron_variant
rs13309280	intron_variant
rs17136160	intron_variant
rs17138937	intron_variant,genic_upstream_transcript_variant
rs17138943	intron_variant,genic_upstream_transcript_variant
rs17138945	intron_variant,genic_upstream_transcript_variant
rs17138948	intron_variant,genic_upstream_transcript_variant
rs17138950	intron_variant,genic_upstream_transcript_variant
rs17138971	intron_variant
rs17138980	intron_variant
rs17138983	intron_variant
rs17526983	intron_variant,genic_downstream_transcript_variant
rs28396520	intron_variant,genic_upstream_transcript_variant
rs28405781	intron_variant
rs28407345	intron_variant,genic_upstream_transcript_variant
rs28527400	intron_variant,genic_upstream_transcript_variant
rs28544234	intron_variant
rs28595367	intron_variant,genic_downstream_transcript_variant
rs28662325	intron_variant,genic_upstream_transcript_variant
rs28670381	intron_variant,genic_upstream_transcript_variant
rs28716070	intron_variant,genic_upstream_transcript_variant
rs28740948	intron_variant
rs34276124	intron_variant,genic_downstream_transcript_variant
rs34280975	intron_variant,genic_downstream_transcript_variant
rs34430832	intron_variant,genic_downstream_transcript_variant
rs34534013	intron_variant
rs34570320	intron_variant
rs34591721	intron_variant,genic_upstream_transcript_variant
rs34613418	intron_variant
rs34719004	intron_variant,genic_downstream_transcript_variant
rs34784937	intron_variant,genic_downstream_transcript_variant
rs34822187	intron_variant,splice_acceptor_variant

rs34830216	intron_variant,genic_downstream_transcript_variant
rs34849015	genic_upstream_transcript_variant,intron_variant,frameshift_variant,non_coding_transcript_variant,coding_sequence_variant
rs34858087	intron_variant,genic_downstream_transcript_variant
rs34878346	intron_variant
rs34906368	intron_variant,genic_downstream_transcript_variant
rs34939991	2KB_upstream_variant,genic_upstream_transcript_variant,upstream_transcript_variant,intron_variant
rs34986275	intron_variant
rs34993867	intron_variant,genic_downstream_transcript_variant
rs35017174	intron_variant
rs35027129	intron_variant,genic_downstream_transcript_variant
rs35060778	intron_variant,genic_downstream_transcript_variant
rs35189619	intron_variant
rs35199351	intron_variant,genic_downstream_transcript_variant
rs35212357	intron_variant,genic_upstream_transcript_variant
rs35260334	intron_variant,genic_upstream_transcript_variant
rs35307741	intron_variant,genic_downstream_transcript_variant
rs35309301	intron_variant
rs35314984	intron_variant,genic_downstream_transcript_variant
rs35400523	intron_variant
rs35404902	intron_variant,genic_upstream_transcript_variant
rs35422943	intron_variant,genic_upstream_transcript_variant
rs35448354	intron_variant,genic_downstream_transcript_variant
rs35487584	intron_variant,genic_upstream_transcript_variant
rs35514449	intron_variant,genic_downstream_transcript_variant
rs35523419	intron_variant
rs35548946	intron_variant,genic_downstream_transcript_variant
rs35624294	intron_variant,genic_downstream_transcript_variant
rs35626122	intron_variant,genic_upstream_transcript_variant
rs35650028	intron_variant
rs35731170	intron_variant,genic_downstream_transcript_variant
rs35759345	intron_variant
rs35939549	intron_variant,genic_downstream_transcript_variant
rs35955226	intron_variant,genic_downstream_transcript_variant
rs35990415	intron_variant,genic_downstream_transcript_variant
rs36013546	intron_variant
rs36031023	intron_variant
rs36076385	intron_variant,genic_upstream_transcript_variant
rs36115495	intron_variant
rs36198935	intron_variant
rs36222675	2KB_upstream_variant,genic_upstream_transcript_variant,upstream_transcript_variant,intron_variant
rs36222676	2KB_upstream_variant,genic_upstream_transcript_variant,upstream_transcript_variant,intron_variant
rs36222677	2KB_upstream_variant,genic_upstream_transcript_variant,upstream_transcript_variant,intron_variant
rs41281078	intron_variant,genic_downstream_transcript_variant
rs45450897	coding_sequence_variant,missense_variant,non_coding_transcript_variant
rs45454696	coding_sequence_variant,missense_variant,genic_downstream_transcript_variant,non_coding_transcript_variant
rs45471794	coding_sequence_variant,missense_variant,genic_downstream_transcript_variant,non_coding_transcript_variant
rs45484593	coding_sequence_variant,synonymous_variant,non_coding_transcript_variant
rs45489503	coding_sequence_variant,synonymous_variant,genic_downstream_transcript_variant,non_coding_transcript_variant
rs45495699	coding_sequence_variant,synonymous_variant,genic_downstream_transcript_variant,non_coding_transcript_variant
rs45496592	coding_sequence_variant,synonymous_variant,genic_downstream_transcript_variant,non_coding_transcript_variant
rs45516592	coding_sequence_variant,intron_variant,missense_variant
rs45531032	coding_sequence_variant,missense_variant,non_coding_transcript_variant
rs45532942	coding_sequence_variant,missense_variant,genic_downstream_transcript_variant,non_coding_transcript_variant
rs45541232	coding_sequence_variant,missense_variant,genic_downstream_transcript_variant,non_coding_transcript_variant
rs45571834	coding_sequence_variant,missense_variant,genic_downstream_transcript_variant,non_coding_transcript_variant
rs45583838	missense_variant,synonymous_variant,non_coding_transcript_variant,coding_sequence_variant,genic_downstream_transcript_variant
rs45592637	coding_sequence_variant,synonymous_variant,non_coding_transcript_variant
rs45595632	coding_sequence_variant,missense_variant,genic_downstream_transcript_variant,non_coding_transcript_variant
rs45604032	coding_sequence_variant,missense_variant,genic_downstream_transcript_variant,non_coding_transcript_variant
rs45605635	coding_sequence_variant,missense_variant,non_coding_transcript_variant
rs55830642	intron_variant,genic_upstream_transcript_variant
rs56197157	intron_variant
rs56212730	intron_variant,genic_downstream_transcript_variant
rs56258803	intron_variant
rs56339508	intron_variant,genic_downstream_transcript_variant
rs56361366	coding_sequence_variant,missense_variant,non_coding_transcript_variant
rs57013376	intron_variant,genic_upstream_transcript_variant
rs57028965	intron_variant,genic_upstream_transcript_variant
rs57097899	intron_variant
rs57132241	intron_variant,genic_upstream_transcript_variant
rs57149583	intron_variant,genic_upstream_transcript_variant
rs57407780	intron_variant,genic_upstream_transcript_variant
rs57669948	intron_variant
rs57693890	intron_variant,genic_upstream_transcript_variant

rs57713644	intron_variant,genic_upstream_transcript_variant,upstream_transcript_variant,2KB_upstream_variant
rs57789859	intron_variant,genic_upstream_transcript_variant
rs58086374	intron_variant
rs58131984	intron_variant,genic_upstream_transcript_variant,upstream_transcript_variant,2KB_upstream_variant
rs58444900	intron_variant,genic_upstream_transcript_variant
rs58668564	intron_variant,genic_upstream_transcript_variant
rs58950839	intron_variant
rs59088564	intron_variant
rs59291978	intron_variant
rs59375450	intron_variant
rs59578108	intron_variant,genic_upstream_transcript_variant
rs59578693	intron_variant
rs59673590	intron_variant,genic_upstream_transcript_variant
rs59745285	intron_variant
rs60046562	intron_variant
rs60405829	intron_variant
rs60600263	intron_variant,genic_upstream_transcript_variant
rs60604040	intron_variant,genic_downstream_transcript_variant
rs60649413	intron_variant
rs60730452	intron_variant
rs60935558	intron_variant
rs61038471	intron_variant,genic_upstream_transcript_variant
rs61248776	intron_variant
rs61293424	intron_variant,genic_upstream_transcript_variant
rs61513761	intron_variant,genic_upstream_transcript_variant
rs61651280	intron_variant
rs62469050	upstream_transcript_variant,genic_upstream_transcript_variant,intron_variant,2KB_upstream_variant
rs62469051	intron_variant,genic_upstream_transcript_variant
rs62469052	intron_variant,genic_upstream_transcript_variant
rs62469053	intron_variant,genic_upstream_transcript_variant
rs62469054	intron_variant,genic_upstream_transcript_variant
rs62469056	intron_variant,genic_upstream_transcript_variant
rs62470761	intron_variant
rs62470762	intron_variant
rs62470763	intron_variant
rs66712866	intron_variant,genic_upstream_transcript_variant,upstream_transcript_variant,2KB_upstream_variant
rs66849348	intron_variant
rs66904901	intron_variant,genic_downstream_transcript_variant
rs66908760	intron_variant,genic_upstream_transcript_variant
rs66996050	intron_variant
rs67297106	intron_variant
rs71148331	intron_variant
rs71314626	intron_variant
rs71528111	intron_variant,genic_upstream_transcript_variant
rs71529475	intron_variant,genic_downstream_transcript_variant
rs71564564	upstream_transcript_variant,genic_upstream_transcript_variant,intron_variant,2KB_upstream_variant
rs71564566	intron_variant
rs73208158	intron_variant,genic_upstream_transcript_variant
rs73208159	intron_variant,genic_upstream_transcript_variant
rs73208161	intron_variant,genic_upstream_transcript_variant
rs73208165	intron_variant
rs73208168	intron_variant
rs73469167	intron_variant,genic_upstream_transcript_variant
rs73469170	intron_variant,genic_upstream_transcript_variant
rs73469173	intron_variant,genic_upstream_transcript_variant
rs73469178	intron_variant,genic_upstream_transcript_variant
rs73469179	intron_variant,genic_upstream_transcript_variant
rs73469188	intron_variant,genic_upstream_transcript_variant
rs73469197	intron_variant
rs73471107	intron_variant
rs73471108	intron_variant
rs73471109	intron_variant
rs73471110	intron_variant
rs73471113	intron_variant
rs73471127	intron_variant,genic_downstream_transcript_variant
rs73471134	intron_variant,genic_downstream_transcript_variant
rs73471137	intron_variant,genic_downstream_transcript_variant
rs73471139	intron_variant,genic_downstream_transcript_variant
rs73716760	intron_variant,genic_upstream_transcript_variant,upstream_transcript_variant,2KB_upstream_variant
rs73716765	intron_variant
rs73716767	intron_variant
rs73716769	intron_variant,genic_downstream_transcript_variant
rs73716770	intron_variant,genic_downstream_transcript_variant
rs73716771	intron_variant,genic_downstream_transcript_variant

rs73716772	intron_variant,genic_downstream_transcript_variant
rs74217195	intron_variant
rs74367828	intron_variant
rs74368568	intron_variant,genic_upstream_transcript_variant,upstream_transcript_variant,2KB_upstream_variant
rs74463536	intron_variant
rs74513265	intron_variant
rs74621320	intron_variant,genic_upstream_transcript_variant
rs74779712	intron_variant
rs74867453	intron_variant,genic_upstream_transcript_variant
rs74957944	intron_variant,genic_downstream_transcript_variant
rs75081540	intron_variant,genic_upstream_transcript_variant
rs75159953	intron_variant,genic_upstream_transcript_variant
rs75195568	intron_variant,genic_upstream_transcript_variant
rs75217062	intron_variant,genic_downstream_transcript_variant
rs75218076	intron_variant,genic_downstream_transcript_variant
rs75261998	intron_variant,genic_upstream_transcript_variant
rs75311055	intron_variant,genic_downstream_transcript_variant
rs75311991	intron_variant,genic_upstream_transcript_variant
rs75318810	intron_variant,genic_downstream_transcript_variant
rs75347282	intron_variant,genic_upstream_transcript_variant
rs75361918	non_coding_transcript_variant,3_prime_UTR_variant,genic_downstream_transcript_variant
rs75444692	intron_variant,genic_upstream_transcript_variant
rs75459774	intron_variant,genic_upstream_transcript_variant
rs75483684	intron_variant,genic_upstream_transcript_variant
rs75577506	intron_variant,genic_upstream_transcript_variant
rs75598970	intron_variant
rs75760645	intron_variant
rs75770850	intron_variant,genic_upstream_transcript_variant
rs75808049	intron_variant,genic_upstream_transcript_variant
rs75841034	intron_variant,genic_upstream_transcript_variant,upstream_transcript_variant,2KB_upstream_variant
rs75852138	intron_variant
rs75855514	intron_variant
rs75887793	500B_downstream_variant,downstream_transcript_variant
rs75921106	intron_variant,genic_upstream_transcript_variant
rs75971075	intron_variant,genic_upstream_transcript_variant
rs75975306	intron_variant,genic_upstream_transcript_variant
rs76037678	intron_variant,genic_upstream_transcript_variant
rs76043381	intron_variant,genic_upstream_transcript_variant
rs76116343	intron_variant
rs76256933	intron_variant,genic_upstream_transcript_variant
rs76405901	intron_variant,genic_downstream_transcript_variant
rs76446249	intron_variant,genic_upstream_transcript_variant
rs76452897	intron_variant,genic_upstream_transcript_variant
rs76484229	intron_variant,genic_upstream_transcript_variant
rs76525083	intron_variant,genic_upstream_transcript_variant
rs76558705	intron_variant,genic_downstream_transcript_variant
rs76575533	intron_variant
rs76631208	intron_variant,genic_upstream_transcript_variant,non_coding_transcript_variant
rs76729878	intron_variant,genic_downstream_transcript_variant
rs76827029	intron_variant
rs76963637	intron_variant,genic_downstream_transcript_variant
rs76992406	intron_variant
rs77100113	intron_variant,genic_upstream_transcript_variant
rs77112188	intron_variant,genic_downstream_transcript_variant
rs77377485	intron_variant,genic_downstream_transcript_variant
rs77387230	intron_variant,genic_upstream_transcript_variant
rs77408757	intron_variant,genic_downstream_transcript_variant
rs77429602	intron_variant,genic_upstream_transcript_variant
rs77497856	intron_variant
rs77516370	intron_variant
rs77522444	intron_variant
rs77546020	intron_variant,genic_upstream_transcript_variant
rs77603709	intron_variant,genic_upstream_transcript_variant
rs77616768	intron_variant
rs77635956	intron_variant
rs77636459	intron_variant,genic_upstream_transcript_variant
rs77651398	non_coding_transcript_variant,intron_variant,coding_sequence_variant,missense_variant,genic_upstream_transcript_variant
rs77653812	intron_variant,genic_upstream_transcript_variant
rs77680491	intron_variant
rs77782453	intron_variant,genic_upstream_transcript_variant
rs77851542	intron_variant,genic_upstream_transcript_variant
rs77887158	intron_variant,genic_upstream_transcript_variant
rs77907624	intron_variant,genic_upstream_transcript_variant,upstream_transcript_variant,2KB_upstream_variant
rs77978685	intron_variant,genic_upstream_transcript_variant

rs77993315	intron_variant,genic_upstream_transcript_variant
rs78112475	intron_variant,genic_downstream_transcript_variant
rs78116323	intron_variant,genic_upstream_transcript_variant
rs78164004	intron_variant,genic_upstream_transcript_variant
rs78243835	intron_variant
rs78247334	intron_variant,genic_upstream_transcript_variant
rs78339146	intron_variant,genic_downstream_transcript_variant
rs78341555	intron_variant,genic_upstream_transcript_variant
rs78453517	intron_variant,genic_upstream_transcript_variant,upstream_transcript_variant,2KB_upstream_variant
rs78462472	intron_variant
rs78483586	intron_variant
rs78689264	intron_variant,genic_upstream_transcript_variant
rs78841592	intron_variant,genic_downstream_transcript_variant
rs78896909	intron_variant,genic_upstream_transcript_variant
rs79058672	intron_variant,genic_upstream_transcript_variant
rs79078086	intron_variant
rs79104788	intron_variant
rs79115875	intron_variant
rs79272363	intron_variant
rs79326908	intron_variant
rs79398475	intron_variant,genic_upstream_transcript_variant
rs79424781	intron_variant,genic_upstream_transcript_variant
rs79460379	intron_variant,genic_downstream_transcript_variant
rs79464413	intron_variant,genic_upstream_transcript_variant,upstream_transcript_variant,2KB_upstream_variant
rs79620817	intron_variant,genic_upstream_transcript_variant
rs79654553	intron_variant,genic_upstream_transcript_variant
rs79655174	intron_variant,genic_upstream_transcript_variant
rs79717477	intron_variant,genic_downstream_transcript_variant
rs79878822	intron_variant,genic_downstream_transcript_variant
rs79901542	2KB_upstream_variant,genic_upstream_transcript_variant,intron_variant,upstream_transcript_variant
rs80016017	intron_variant
rs80019847	intron_variant,genic_upstream_transcript_variant
rs80055619	intron_variant,genic_upstream_transcript_variant
rs80102316	intron_variant,genic_upstream_transcript_variant
rs80154660	intron_variant,genic_upstream_transcript_variant
rs80259898	intron_variant
rs80278428	intron_variant
rs111309351	intron_variant
rs111312176	intron_variant
rs111416989	intron_variant
rs111478642	intron_variant,genic_downstream_transcript_variant
rs111522192	intron_variant,genic_downstream_transcript_variant
rs111614112	intron_variant,genic_upstream_transcript_variant
rs111616529	intron_variant,genic_upstream_transcript_variant
rs111654103	intron_variant
rs111680932	intron_variant,genic_downstream_transcript_variant
rs111699200	intron_variant,genic_downstream_transcript_variant
rs111710970	intron_variant,genic_downstream_transcript_variant
rs111794917	intron_variant
rs111863186	intron_variant,genic_upstream_transcript_variant
rs111900049	intron_variant,genic_upstream_transcript_variant
rs111914725	intron_variant
rs111955405	intron_variant,genic_upstream_transcript_variant
rs111972263	intron_variant
rs112024074	intron_variant,genic_upstream_transcript_variant
rs112078932	intron_variant
rs112093839	intron_variant
rs112117974	intron_variant,genic_upstream_transcript_variant
rs112155944	intron_variant
rs112199560	intron_variant,genic_downstream_transcript_variant
rs112202696	intron_variant
rs112205913	intron_variant,genic_upstream_transcript_variant
rs112248112	intron_variant,genic_upstream_transcript_variant
rs112249479	genic_downstream_transcript_variant,3_prime_UTR_variant,non_coding_transcript_variant
rs112251285	intron_variant,genic_downstream_transcript_variant
rs112270031	intron_variant
rs112333710	intron_variant
rs112394333	intron_variant,genic_upstream_transcript_variant
rs112464343	intron_variant
rs112486776	intron_variant,genic_upstream_transcript_variant
rs112493416	2KB_upstream_variant,genic_upstream_transcript_variant,intron_variant,upstream_transcript_variant
rs112493453	2KB_upstream_variant,genic_upstream_transcript_variant,intron_variant,upstream_transcript_variant
rs112556889	intron_variant,genic_downstream_transcript_variant
rs112620858	intron_variant

rs112644632	intron_variant
rs112782799	intron_variant
rs112798793	intron_variant,genic_upstream_transcript_variant
rs112819631	intron_variant,genic_upstream_transcript_variant
rs112849331	intron_variant,genic_upstream_transcript_variant
rs112868493	intron_variant,genic_upstream_transcript_variant
rs112890288	intron_variant,genic_downstream_transcript_variant
rs112945585	intron_variant
rs113035202	intron_variant,genic_upstream_transcript_variant,upstream_transcript_variant
rs113076725	intron_variant
rs113080858	intron_variant,genic_upstream_transcript_variant
rs113130716	intron_variant
rs113205008	2KB_upstream_variant,genic_upstream_transcript_variant,intron_variant,upstream_transcript_variant
rs113255368	intron_variant,genic_upstream_transcript_variant
rs113261722	intron_variant
rs113467366	intron_variant,genic_upstream_transcript_variant
rs113515365	intron_variant,genic_downstream_transcript_variant
rs113522915	intron_variant,genic_downstream_transcript_variant
rs113650927	intron_variant,genic_upstream_transcript_variant
rs113656359	intron_variant
rs113681741	intron_variant
rs113698270	intron_variant
rs113754807	intron_variant,genic_upstream_transcript_variant
rs113763118	intron_variant,genic_downstream_transcript_variant
rs113806645	intron_variant,genic_downstream_transcript_variant
rs113841457	intron_variant,genic_upstream_transcript_variant
rs113974855	intron_variant,genic_upstream_transcript_variant
rs113992893	intron_variant
rs114079927	intron_variant,genic_downstream_transcript_variant
rs114202452	intron_variant,downstream_transcript_variant,genic_downstream_transcript_variant
rs114305562	intron_variant,genic_upstream_transcript_variant
rs114392631	intron_variant,genic_downstream_transcript_variant
rs114615446	intron_variant
rs114692368	intron_variant,genic_upstream_transcript_variant
rs114707545	intron_variant,genic_downstream_transcript_variant
rs114709616	intron_variant,genic_upstream_transcript_variant
rs114767833	intron_variant
rs114822722	intron_variant,genic_upstream_transcript_variant
rs114977543	intron_variant,genic_upstream_transcript_variant
rs115088619	intron_variant,genic_upstream_transcript_variant
rs115164400	intron_variant,genic_downstream_transcript_variant
rs115208619	intron_variant
rs115240747	intron_variant,genic_downstream_transcript_variant
rs115286439	intron_variant
rs115293079	intron_variant,genic_downstream_transcript_variant
rs115313396	intron_variant,genic_upstream_transcript_variant
rs115485522	intron_variant,genic_upstream_transcript_variant
rs115566611	intron_variant
rs115628473	intron_variant
rs115738760	intron_variant,genic_upstream_transcript_variant
rs115851641	intron_variant,genic_upstream_transcript_variant
rs115870111	intron_variant,genic_upstream_transcript_variant
rs115875256	intron_variant,genic_upstream_transcript_variant
rs115909573	intron_variant
rs115953870	intron_variant,genic_downstream_transcript_variant
rs116041574	intron_variant,genic_downstream_transcript_variant
rs116188641	intron_variant,genic_upstream_transcript_variant
rs116190704	intron_variant,genic_upstream_transcript_variant
rs116218483	intron_variant,genic_upstream_transcript_variant
rs116276647	intron_variant,genic_upstream_transcript_variant
rs116312019	intron_variant,genic_upstream_transcript_variant
rs116458171	intron_variant,genic_upstream_transcript_variant
rs116481715	intron_variant,genic_upstream_transcript_variant
rs116530062	intron_variant
rs116569774	intron_variant
rs116662833	intron_variant,genic_upstream_transcript_variant
rs116709476	intron_variant,genic_upstream_transcript_variant
rs116870479	intron_variant,genic_upstream_transcript_variant
rs116901588	intron_variant,genic_upstream_transcript_variant
rs116921956	genic_downstream_transcript_variant,intron_variant,downstream_transcript_variant
rs116952382	intron_variant,genic_upstream_transcript_variant
rs117028963	intron_variant,genic_upstream_transcript_variant
rs117029561	intron_variant
rs117062893	intron_variant,genic_upstream_transcript_variant

rs117066610	intron_variant,genic_upstream_transcript_variant
rs117120979	intron_variant
rs117181391	intron_variant
rs117274849	intron_variant
rs117350321	genic_downstream_transcript_variant,intron_variant
rs117369473	intron_variant,genic_upstream_transcript_variant
rs117459423	genic_downstream_transcript_variant,intron_variant
rs117585786	genic_downstream_transcript_variant,intron_variant
rs117602546	intron_variant,genic_upstream_transcript_variant
rs117610086	intron_variant,genic_upstream_transcript_variant
rs117674594	intron_variant,genic_upstream_transcript_variant
rs117780183	intron_variant,genic_upstream_transcript_variant
rs117805272	genic_downstream_transcript_variant,intron_variant
rs117877994	intron_variant
rs117927707	genic_downstream_transcript_variant,intron_variant
rs117930107	intron_variant,genic_upstream_transcript_variant
rs117965622	intron_variant
rs118043302	genic_downstream_transcript_variant,intron_variant
rs118052543	intron_variant
rs118133676	intron_variant
rs118157537	intron_variant,genic_upstream_transcript_variant
rs137856686	intron_variant,genic_upstream_transcript_variant
rs137920591	intron_variant
rs137929201	intron_variant,genic_upstream_transcript_variant
rs137975449	intron_variant
rs138041456	intron_variant
rs138083038	intron_variant,genic_upstream_transcript_variant
rs138132213	intron_variant
rs138177961	intron_variant,genic_upstream_transcript_variant
rs138190830	intron_variant,genic_upstream_transcript_variant
rs138198060	genic_downstream_transcript_variant,intron_variant
rs138237329	intron_variant,genic_upstream_transcript_variant
rs138238598	genic_downstream_transcript_variant,intron_variant
rs138248022	intron_variant,genic_upstream_transcript_variant
rs138302919	intron_variant,genic_upstream_transcript_variant
rs138369472	intron_variant
rs138488688	intron_variant
rs138528479	intron_variant
rs138536032	intron_variant,genic_upstream_transcript_variant
rs138575615	intron_variant
rs138598351	intron_variant,genic_upstream_transcript_variant
rs138624555	intron_variant
rs138637051	intron_variant
rs138649688	intron_variant,genic_upstream_transcript_variant
rs138784802	intron_variant
rs138845890	genic_downstream_transcript_variant,intron_variant
rs138854086	intron_variant,genic_upstream_transcript_variant
rs138854909	genic_downstream_transcript_variant,intron_variant
rs138858254	intron_variant
rs138860626	intron_variant,genic_upstream_transcript_variant
rs138906839	genic_downstream_transcript_variant,intron_variant
rs138907870	genic_downstream_transcript_variant,intron_variant
rs138917893	intron_variant,genic_upstream_transcript_variant
rs138981451	intron_variant
rs139045186	genic_downstream_transcript_variant,intron_variant
rs139081517	2KB_upstream_variant,intron_variant,genic_upstream_transcript_variant,upstream_transcript_variant
rs139220708	intron_variant
rs139254855	intron_variant,2KB_upstream_variant,genic_upstream_transcript_variant,upstream_transcript_variant
rs139261309	intron_variant,genic_upstream_transcript_variant
rs139317854	intron_variant,genic_upstream_transcript_variant
rs139333175	intron_variant,genic_upstream_transcript_variant
rs139335187	intron_variant
rs139360435	intron_variant
rs139384329	intron_variant,genic_upstream_transcript_variant
rs139396986	genic_downstream_transcript_variant,intron_variant
rs139400929	intron_variant,genic_upstream_transcript_variant
rs139463164	intron_variant
rs139529354	genic_downstream_transcript_variant,intron_variant
rs139540494	intron_variant,genic_upstream_transcript_variant
rs139591994	genic_downstream_transcript_variant,intron_variant
rs139706580	intron_variant
rs139744904	intron_variant
rs139814860	intron_variant,genic_upstream_transcript_variant
rs139824613	intron_variant,genic_upstream_transcript_variant

rs139923707	intron_variant
rs139956298	intron_variant
rs139963946	intron_variant
rs140027773	genic_downstream_transcript_variant,intron_variant
rs140100846	intron_variant,genic_upstream_transcript_variant
rs140137399	intron_variant,genic_upstream_transcript_variant
rs140166469	genic_downstream_transcript_variant,intron_variant
rs140234353	intron_variant,genic_upstream_transcript_variant
rs140239955	intron_variant
rs140308712	intron_variant
rs140308737	intron_variant,genic_upstream_transcript_variant
rs140369608	intron_variant
rs140372906	intron_variant
rs140390284	2KB_upstream_variant,intron_variant,genic_upstream_transcript_variant,upstream_transcript_variant
rs140435978	genic_downstream_transcript_variant,intron_variant
rs140439141	intron_variant
rs140446411	intron_variant,genic_upstream_transcript_variant
rs140481959	intron_variant,genic_upstream_transcript_variant
rs140508886	intron_variant
rs140508991	downstream_transcript_variant,500B_downstream_variant
rs140632871	intron_variant
rs140652385	intron_variant
rs140712080	intron_variant
rs140723017	intron_variant,genic_upstream_transcript_variant
rs140736731	intron_variant,genic_upstream_transcript_variant
rs140794452	intron_variant
rs140818026	intron_variant
rs140824245	genic_downstream_transcript_variant,intron_variant
rs140864161	genic_downstream_transcript_variant,intron_variant
rs140868029	intron_variant,genic_upstream_transcript_variant
rs140930646	intron_variant
rs140971436	intron_variant
rs141179034	intron_variant
rs141199678	intron_variant
rs141220474	intron_variant,genic_upstream_transcript_variant
rs141231432	intron_variant,2KB_upstream_variant,genic_upstream_transcript_variant,upstream_transcript_variant
rs141256603	intron_variant,genic_upstream_transcript_variant
rs141329901	genic_downstream_transcript_variant,intron_variant
rs141338324	intron_variant,genic_upstream_transcript_variant
rs141395129	genic_downstream_transcript_variant,intron_variant
rs141409396	genic_downstream_transcript_variant,intron_variant
rs141414046	intron_variant,genic_upstream_transcript_variant
rs141519069	intron_variant,genic_upstream_transcript_variant
rs141552422	intron_variant
rs141660864	intron_variant,genic_upstream_transcript_variant
rs141665829	intron_variant
rs141687946	intron_variant,genic_upstream_transcript_variant
rs141693647	intron_variant
rs141698670	intron_variant,genic_upstream_transcript_variant
rs141754088	intron_variant,genic_upstream_transcript_variant
rs141796362	intron_variant
rs141821960	intron_variant,genic_upstream_transcript_variant
rs141836842	intron_variant
rs141868901	genic_downstream_transcript_variant,intron_variant
rs141975249	genic_downstream_transcript_variant,intron_variant
rs141978615	2KB_upstream_variant,intron_variant,genic_upstream_transcript_variant,upstream_transcript_variant
rs141999552	intron_variant
rs142038039	intron_variant,genic_upstream_transcript_variant
rs142049996	intron_variant,genic_upstream_transcript_variant
rs142065796	intron_variant
rs142065996	genic_downstream_transcript_variant,intron_variant
rs142130317	genic_downstream_transcript_variant,intron_variant
rs142156056	intron_variant
rs142169934	intron_variant
rs142188792	genic_downstream_transcript_variant,intron_variant
rs142208803	intron_variant,genic_upstream_transcript_variant
rs142232873	intron_variant,genic_upstream_transcript_variant
rs142235447	intron_variant
rs142296370	intron_variant
rs142414409	intron_variant
rs142472407	intron_variant,genic_upstream_transcript_variant
rs142499284	genic_downstream_transcript_variant,intron_variant
rs142504410	intron_variant,genic_upstream_transcript_variant
rs142534912	intron_variant

rs142608712	intron_variant,genic_upstream_transcript_variant
rs142641212	intron_variant
rs142688112	genic_downstream_transcript_variant,intron_variant
rs142693440	2KB_upstream_variant,intron_variant,genic_upstream_transcript_variant,upstream_transcript_variant
rs142828636	intron_variant,genic_upstream_transcript_variant
rs142834198	downstream_transcript_variant,500B_downstream_variant
rs142857764	intron_variant
rs142859263	intron_variant,genic_upstream_transcript_variant
rs142880865	intron_variant
rs142881446	intron_variant,genic_upstream_transcript_variant
rs142913753	genic_downstream_transcript_variant,intron_variant
rs142917092	genic_downstream_transcript_variant,intron_variant
rs142947751	intron_variant
rs142954535	intron_variant,genic_upstream_transcript_variant
rs142988622	intron_variant,genic_upstream_transcript_variant
rs143084294	intron_variant,genic_upstream_transcript_variant
rs143117128	genic_downstream_transcript_variant,intron_variant
rs143159217	intron_variant,genic_upstream_transcript_variant
rs143224435	intron_variant
rs143326665	intron_variant
rs143338232	genic_downstream_transcript_variant,non_coding_transcript_variant,3_prime_UTR_variant
rs143345613	intron_variant,genic_upstream_transcript_variant
rs143363456	intron_variant,genic_upstream_transcript_variant
rs143406317	intron_variant
rs143423869	genic_downstream_transcript_variant,intron_variant
rs143431244	intron_variant
rs143433269	2KB_upstream_variant,intron_variant,genic_upstream_transcript_variant,upstream_transcript_variant
rs143499403	intron_variant
rs143561006	genic_downstream_transcript_variant,intron_variant
rs143572798	intron_variant
rs143608767	intron_variant,genic_upstream_transcript_variant
rs143639433	genic_downstream_transcript_variant,intron_variant
rs143759287	intron_variant,genic_upstream_transcript_variant
rs143784870	intron_variant
rs143785217	intron_variant
rs143789230	intron_variant,genic_upstream_transcript_variant
rs143820071	intron_variant
rs143823423	intron_variant
rs143849410	genic_downstream_transcript_variant,intron_variant
rs143851859	genic_downstream_transcript_variant,intron_variant
rs143912481	genic_downstream_transcript_variant,intron_variant
rs143992058	intron_variant,genic_upstream_transcript_variant
rs144076992	genic_upstream_transcript_variant,intron_variant
rs144121012	intron_variant
rs144142698	intron_variant
rs144159965	genic_upstream_transcript_variant,intron_variant
rs144248784	genic_downstream_transcript_variant,intron_variant
rs144259187	genic_upstream_transcript_variant,intron_variant
rs144286944	intron_variant
rs144325759	genic_upstream_transcript_variant,intron_variant
rs144389682	intron_variant
rs144456969	intron_variant
rs144461358	intron_variant
rs144526050	intron_variant
rs144545627	2KB_upstream_variant,upstream_transcript_variant,genic_upstream_transcript_variant,intron_variant
rs144589233	genic_downstream_transcript_variant,intron_variant
rs144625154	intron_variant
rs144672186	genic_upstream_transcript_variant,intron_variant
rs144731030	genic_downstream_transcript_variant,intron_variant
rs144737828	genic_upstream_transcript_variant,intron_variant
rs144803151	intron_variant
rs144830337	intron_variant
rs144899198	genic_upstream_transcript_variant,intron_variant
rs144939114	genic_downstream_transcript_variant,intron_variant
rs144943989	missense_variant,coding_sequence_variant,non_coding_transcript_variant
rs144944341	genic_upstream_transcript_variant,intron_variant
rs144969165	genic_upstream_transcript_variant,intron_variant
rs144978880	genic_downstream_transcript_variant,intron_variant
rs144982888	2KB_upstream_variant,upstream_transcript_variant,genic_upstream_transcript_variant,intron_variant
rs145019899	genic_downstream_transcript_variant,intron_variant
rs145025167	genic_upstream_transcript_variant,intron_variant
rs145053814	genic_downstream_transcript_variant,intron_variant
rs145080745	intron_variant
rs145145882	intron_variant

rs145212056	genic_downstream_transcript_variant,intron_variant
rs145219972	genic_upstream_transcript_variant,intron_variant
rs145290321	genic_upstream_transcript_variant,intron_variant
rs145366707	genic_upstream_transcript_variant,intron_variant
rs145396158	intron_variant
rs145400702	intron_variant
rs145435221	intron_variant
rs145507435	intron_variant
rs145545383	intron_variant
rs145626474	intron_variant
rs145680574	intron_variant
rs145688984	genic_upstream_transcript_variant,intron_variant
rs145762580	genic_downstream_transcript_variant,intron_variant
rs145989320	intron_variant
rs146047396	intron_variant
rs146094122	genic_upstream_transcript_variant,intron_variant
rs146111367	genic_downstream_transcript_variant,intron_variant
rs146143332	genic_downstream_transcript_variant,intron_variant
rs146174097	intron_variant
rs146215092	genic_upstream_transcript_variant,intron_variant
rs146233711	genic_upstream_transcript_variant,intron_variant
rs146252378	intron_variant
rs146270757	intron_variant
rs146334012	intron_variant
rs146338275	intron_variant
rs146352540	intron_variant
rs146373122	genic_downstream_transcript_variant,intron_variant
rs146410999	intron_variant
rs146429926	genic_upstream_transcript_variant,intron_variant
rs146443768	genic_upstream_transcript_variant,intron_variant
rs146514780	intron_variant
rs146553361	intron_variant
rs146652167	genic_upstream_transcript_variant,intron_variant
rs146671520	genic_downstream_transcript_variant,intron_variant
rs146723491	genic_upstream_transcript_variant,intron_variant
rs146735827	genic_upstream_transcript_variant,intron_variant
rs146755153	genic_upstream_transcript_variant,intron_variant
rs146812594	2KB_upstream_variant,upstream_transcript_variant,genic_upstream_transcript_variant,intron_variant
rs146851440	intron_variant
rs146856514	genic_upstream_transcript_variant,intron_variant
rs146856911	intron_variant
rs146905991	intron_variant
rs146977613	intron_variant
rs146994340	genic_downstream_transcript_variant,intron_variant
rs146994411	genic_upstream_transcript_variant,intron_variant
rs147010632	genic_downstream_transcript_variant,intron_variant
rs147081516	intron_variant
rs147131910	genic_upstream_transcript_variant,intron_variant
rs147148084	intron_variant
rs147193493	genic_upstream_transcript_variant,intron_variant
rs147309260	intron_variant
rs147327246	2KB_upstream_variant,upstream_transcript_variant,genic_upstream_transcript_variant,intron_variant
rs147358921	genic_downstream_transcript_variant,intron_variant
rs147361638	2KB_upstream_variant,upstream_transcript_variant,genic_upstream_transcript_variant,intron_variant
rs147431845	genic_upstream_transcript_variant,intron_variant
rs147449421	genic_upstream_transcript_variant,intron_variant
rs147603374	intron_variant
rs147637222	genic_downstream_transcript_variant,intron_variant
rs147691094	intron_variant
rs147707965	intron_variant
rs147794765	genic_downstream_transcript_variant,intron_variant
rs147797443	genic_upstream_transcript_variant,intron_variant
rs147811552	genic_downstream_transcript_variant,intron_variant
rs147814057	genic_upstream_transcript_variant,intron_variant
rs147919103	genic_upstream_transcript_variant,intron_variant
rs148002080	intron_variant
rs148073728	intron_variant
rs148090539	genic_downstream_transcript_variant,intron_variant
rs148143237	genic_downstream_transcript_variant,intron_variant
rs148145098	genic_upstream_transcript_variant,intron_variant
rs148164239	genic_upstream_transcript_variant,intron_variant
rs148215519	genic_upstream_transcript_variant,intron_variant
rs148234397	genic_upstream_transcript_variant,intron_variant
rs148287228	genic_upstream_transcript_variant,intron_variant

rs148303685	intron_variant
rs148354710	intron_variant
rs148370379	genic_downstream_transcript_variant,3_prime_UTR_variant,non_coding_transcript_variant
rs148372965	intron_variant
rs148456388	intron_variant
rs148475572	intron_variant
rs148526613	missense_variant,non_coding_transcript_variant,genic_upstream_transcript_variant,intron_variant,coding_sequence_variant
rs148544593	genic_downstream_transcript_variant,intron_variant
rs148595460	genic_downstream_transcript_variant,intron_variant
rs148612113	genic_downstream_transcript_variant,intron_variant
rs148663970	genic_downstream_transcript_variant,intron_variant
rs148666768	2KB_upstream_variant,upstream_transcript_variant,genic_upstream_transcript_variant,intron_variant
rs148685174	genic_upstream_transcript_variant,intron_variant
rs148755295	intron_variant
rs148755484	intron_variant
rs148807898	intron_variant
rs148858964	intron_variant
rs148859467	genic_downstream_transcript_variant,intron_variant
rs148861260	genic_upstream_transcript_variant,intron_variant
rs148910731	intron_variant
rs148915230	genic_downstream_transcript_variant,intron_variant
rs148930490	genic_upstream_transcript_variant,intron_variant
rs148997835	intron_variant
rs149050485	genic_upstream_transcript_variant,intron_variant
rs149066899	genic_downstream_transcript_variant,intron_variant
rs149118541	genic_downstream_transcript_variant,intron_variant
rs149121111	genic_upstream_transcript_variant,intron_variant
rs149138546	intron_variant
rs149242670	intron_variant
rs149251301	intron_variant
rs149313470	genic_upstream_transcript_variant,intron_variant
rs149364401	genic_downstream_transcript_variant,intron_variant
rs149385616	genic_upstream_transcript_variant,intron_variant
rs149402541	intron_variant
rs149456718	genic_upstream_transcript_variant,intron_variant
rs149508208	genic_upstream_transcript_variant,intron_variant
rs149524847	intron_variant
rs149577283	genic_upstream_transcript_variant,intron_variant
rs149593751	genic_downstream_transcript_variant,intron_variant
rs149645111	genic_downstream_transcript_variant,intron_variant
rs149678565	intron_variant
rs149697638	intron_variant
rs149704455	intron_variant
rs149838222	genic_upstream_transcript_variant,intron_variant
rs149857525	intron_variant
rs149889921	genic_downstream_transcript_variant,intron_variant
rs149927169	intron_variant
rs150031462	genic_upstream_transcript_variant,intron_variant
rs150047937	intron_variant
rs150081685	genic_upstream_transcript_variant,intron_variant
rs150133374	genic_upstream_transcript_variant,intron_variant
rs150149673	genic_downstream_transcript_variant,intron_variant
rs150169068	genic_downstream_transcript_variant,intron_variant
rs150289376	intron_variant
rs150310630	genic_upstream_transcript_variant,intron_variant
rs150341442	intron_variant
rs150379044	intron_variant
rs150431962	intron_variant
rs150432528	genic_upstream_transcript_variant,intron_variant
rs150485412	genic_upstream_transcript_variant,intron_variant
rs150536677	genic_upstream_transcript_variant,intron_variant
rs150606075	genic_upstream_transcript_variant,intron_variant
rs150624732	intron_variant
rs150673762	genic_downstream_transcript_variant,intron_variant
rs150675651	intron_variant
rs150691583	genic_downstream_transcript_variant,intron_variant
rs150744300	intron_variant
rs150814384	intron_variant
rs150832973	genic_downstream_transcript_variant,intron_variant
rs150835625	genic_upstream_transcript_variant,intron_variant
rs150888353	genic_upstream_transcript_variant,intron_variant
rs150904793	intron_variant
rs150918864	intron_variant
rs150976423	intron_variant

rs150989423	genic_downstream_transcript_variant,intron_variant
rs150991259	genic_upstream_transcript_variant,intron_variant
rs150992859	intron_variant
rs151058444	500B_downstream_variant,downstream_transcript_variant
rs151061160	genic_upstream_transcript_variant,intron_variant
rs151079868	genic_upstream_transcript_variant,intron_variant
rs151218135	genic_downstream_transcript_variant,intron_variant
rs151254852	genic_upstream_transcript_variant,intron_variant
rs151271433	intron_variant
rs151289561	intron_variant
rs151335286	genic_upstream_transcript_variant,intron_variant
rs180773571	genic_downstream_transcript_variant,intron_variant
rs180794948	intron_variant
rs180802565	genic_downstream_transcript_variant,intron_variant
rs180832548	genic_downstream_transcript_variant,intron_variant
rs180963374	genic_upstream_transcript_variant,intron_variant
rs181001374	genic_upstream_transcript_variant,intron_variant
rs181017470	genic_upstream_transcript_variant,intron_variant
rs181042500	genic_downstream_transcript_variant,intron_variant
rs181059104	genic_upstream_transcript_variant,intron_variant
rs181062044	intron_variant
rs181075269	intron_variant
rs181083017	genic_downstream_transcript_variant,intron_variant
rs181089956	intron_variant
rs181099511	intron_variant
rs181108979	genic_upstream_transcript_variant,intron_variant
rs181109101	genic_downstream_transcript_variant,intron_variant
rs181115606	genic_upstream_transcript_variant,intron_variant
rs181121462	intron_variant
rs181122307	genic_upstream_transcript_variant,intron_variant
rs181138331	genic_upstream_transcript_variant,intron_variant
rs181218108	genic_upstream_transcript_variant,intron_variant
rs181246833	genic_upstream_transcript_variant,intron_variant
rs181253089	genic_upstream_transcript_variant,intron_variant
rs181254131	genic_upstream_transcript_variant,intron_variant
rs181257423	intron_variant
rs181318630	genic_downstream_transcript_variant,intron_variant
rs181324784	intron_variant
rs181341965	genic_downstream_transcript_variant,intron_variant
rs181357178	genic_upstream_transcript_variant,intron_variant
rs181449499	intron_variant
rs181475273	genic_upstream_transcript_variant,intron_variant
rs181477949	intron_variant
rs181486234	intron_variant
rs181487844	genic_upstream_transcript_variant,intron_variant
rs181496887	genic_downstream_transcript_variant,intron_variant
rs181617254	intron_variant,genic_upstream_transcript_variant
rs181632403	intron_variant,genic_upstream_transcript_variant
rs181634052	intron_variant,genic_upstream_transcript_variant
rs181637225	intron_variant
rs181692338	intron_variant
rs181712796	genic_downstream_transcript_variant,intron_variant
rs181718280	genic_downstream_transcript_variant,intron_variant
rs181747548	intron_variant,genic_upstream_transcript_variant
rs181751458	genic_downstream_transcript_variant,intron_variant
rs181773926	intron_variant
rs181781631	intron_variant
rs181818408	genic_downstream_transcript_variant,non_coding_transcript_variant,3_prime_UTR_variant
rs181832192	genic_downstream_transcript_variant,intron_variant
rs181850913	intron_variant,genic_upstream_transcript_variant
rs181877385	intron_variant
rs181920009	intron_variant
rs181926764	intron_variant,genic_upstream_transcript_variant
rs181970953	intron_variant
rs182124415	intron_variant,genic_upstream_transcript_variant
rs182131737	intron_variant
rs182155621	genic_downstream_transcript_variant,intron_variant
rs182167415	intron_variant,genic_upstream_transcript_variant
rs182177565	intron_variant,genic_upstream_transcript_variant
rs182183650	genic_downstream_transcript_variant,intron_variant
rs182191423	intron_variant
rs182202951	intron_variant
rs182220935	genic_downstream_transcript_variant,intron_variant
rs182225931	intron_variant

rs182227689	intron_variant
rs182228951	intron_variant
rs182229643	intron_variant,genic_upstream_transcript_variant
rs182236537	genic_downstream_transcript_variant,intron_variant
rs182237211	intron_variant
rs182322573	intron_variant,genic_upstream_transcript_variant
rs182337634	intron_variant,genic_upstream_transcript_variant
rs182358752	intron_variant,genic_upstream_transcript_variant
rs182371288	intron_variant,genic_upstream_transcript_variant
rs182372249	intron_variant,genic_upstream_transcript_variant
rs182384081	intron_variant
rs182427854	intron_variant
rs182430340	intron_variant
rs182437939	genic_downstream_transcript_variant,intron_variant
rs182447735	5_prime_UTR_variant,intron_variant
rs182480559	intron_variant
rs182483696	intron_variant,genic_upstream_transcript_variant
rs182517414	intron_variant,genic_upstream_transcript_variant
rs182722031	intron_variant
rs182735227	intron_variant
rs182744138	upstream_transcript_variant,intron_variant,2KB_upstream_variant,genic_upstream_transcript_variant
rs182758855	intron_variant,genic_upstream_transcript_variant
rs182774494	intron_variant
rs182779243	intron_variant
rs182796812	intron_variant,genic_upstream_transcript_variant
rs182819705	intron_variant
rs182850010	genic_downstream_transcript_variant,intron_variant
rs182851669	intron_variant,genic_upstream_transcript_variant
rs182950182	intron_variant,genic_upstream_transcript_variant
rs182951757	intron_variant
rs182963802	intron_variant
rs183004460	genic_downstream_transcript_variant,intron_variant
rs183010787	intron_variant
rs183022486	genic_downstream_transcript_variant,intron_variant,downstream_transcript_variant
rs183035736	intron_variant,genic_upstream_transcript_variant
rs183047489	genic_downstream_transcript_variant,intron_variant
rs183060484	genic_downstream_transcript_variant,intron_variant
rs183129962	genic_downstream_transcript_variant,intron_variant
rs183153720	intron_variant,genic_upstream_transcript_variant
rs183162565	intron_variant
rs183169084	intron_variant,genic_upstream_transcript_variant
rs183181798	intron_variant
rs183186610	intron_variant
rs183216472	upstream_transcript_variant,intron_variant,2KB_upstream_variant,genic_upstream_transcript_variant
rs183224025	upstream_transcript_variant,intron_variant,2KB_upstream_variant,genic_upstream_transcript_variant
rs183224935	intron_variant,genic_upstream_transcript_variant
rs183273989	intron_variant
rs183333487	intron_variant,genic_upstream_transcript_variant
rs183338176	genic_downstream_transcript_variant,intron_variant
rs183360917	intron_variant,genic_upstream_transcript_variant
rs183375652	intron_variant
rs183417320	intron_variant,genic_upstream_transcript_variant
rs183451765	intron_variant,genic_upstream_transcript_variant
rs183452921	intron_variant,genic_upstream_transcript_variant
rs183481059	intron_variant
rs183559564	intron_variant,genic_upstream_transcript_variant
rs183562083	intron_variant,genic_upstream_transcript_variant
rs183564084	intron_variant
rs183573710	intron_variant
rs183578496	intron_variant,genic_upstream_transcript_variant
rs183590950	500B_downstream_variant,downstream_transcript_variant
rs183594343	intron_variant,genic_upstream_transcript_variant
rs183609864	intron_variant
rs183610924	intron_variant,genic_upstream_transcript_variant
rs183628119	intron_variant,genic_upstream_transcript_variant
rs183682632	intron_variant,genic_upstream_transcript_variant
rs183685952	genic_downstream_transcript_variant,intron_variant
rs183710100	intron_variant
rs183738503	intron_variant
rs183744635	genic_downstream_transcript_variant,intron_variant
rs183750961	genic_downstream_transcript_variant,intron_variant
rs183788694	intron_variant
rs183792760	intron_variant
rs183834974	intron_variant,genic_upstream_transcript_variant

rs183837517	intron_variant,genic_upstream_transcript_variant
rs183838246	intron_variant,genic_upstream_transcript_variant
rs183871547	genic_downstream_transcript_variant,intron_variant
rs183902418	upstream_transcript_variant,intron_variant,2KB_upstream_variant,genic_upstream_transcript_variant
rs183952788	intron_variant
rs184031477	intron_variant
rs184036796	intron_variant
rs184059396	intron_variant,genic_upstream_transcript_variant
rs184064938	intron_variant,genic_upstream_transcript_variant
rs184187476	genic_downstream_transcript_variant,intron_variant
rs184287586	intron_variant
rs184292390	genic_downstream_transcript_variant,intron_variant
rs184325106	intron_variant,genic_upstream_transcript_variant
rs184339266	intron_variant
rs184340036	genic_downstream_transcript_variant,intron_variant
rs184342241	intron_variant
rs184348578	intron_variant
rs184354416	genic_downstream_transcript_variant,intron_variant
rs184359541	genic_downstream_transcript_variant,intron_variant
rs184378419	intron_variant,genic_upstream_transcript_variant
rs184386794	intron_variant
rs184396677	genic_downstream_transcript_variant,intron_variant
rs184424020	genic_downstream_transcript_variant,intron_variant
rs184428583	intron_variant
rs184439568	intron_variant,genic_upstream_transcript_variant
rs184452073	intron_variant,genic_upstream_transcript_variant
rs184467506	intron_variant
rs184508593	genic_downstream_transcript_variant,intron_variant
rs184512697	intron_variant,genic_upstream_transcript_variant
rs184517571	intron_variant,genic_upstream_transcript_variant
rs184522192	intron_variant
rs184569469	intron_variant,genic_upstream_transcript_variant
rs184571259	intron_variant,genic_upstream_transcript_variant
rs184578449	intron_variant
rs184586067	genic_downstream_transcript_variant,intron_variant
rs184587931	intron_variant
rs184599757	intron_variant,genic_upstream_transcript_variant
rs184618579	intron_variant,genic_upstream_transcript_variant
rs184650918	upstream_transcript_variant,intron_variant,2KB_upstream_variant,genic_upstream_transcript_variant
rs184684467	intron_variant,genic_upstream_transcript_variant
rs184701203	intron_variant,genic_upstream_transcript_variant
rs184815676	intron_variant
rs184915956	genic_downstream_transcript_variant,intron_variant
rs184917798	intron_variant
rs184984861	genic_downstream_transcript_variant,intron_variant
rs185019444	intron_variant
rs185100233	upstream_transcript_variant,intron_variant,2KB_upstream_variant,genic_upstream_transcript_variant
rs185122189	genic_downstream_transcript_variant,intron_variant
rs185141870	intron_variant
rs185144153	genic_downstream_transcript_variant,intron_variant
rs185151194	intron_variant
rs185196751	intron_variant
rs185200665	intron_variant,genic_upstream_transcript_variant
rs185264958	intron_variant,genic_upstream_transcript_variant
rs185265967	intron_variant,genic_upstream_transcript_variant
rs185274324	intron_variant
rs185280303	intron_variant
rs185283273	intron_variant
rs185291452	intron_variant,genic_upstream_transcript_variant
rs185316200	intron_variant,genic_upstream_transcript_variant
rs185323698	intron_variant,genic_upstream_transcript_variant
rs185332853	intron_variant,genic_upstream_transcript_variant
rs185442020	intron_variant,genic_upstream_transcript_variant
rs185454881	intron_variant,genic_upstream_transcript_variant
rs185539839	intron_variant
rs185557707	intron_variant,genic_upstream_transcript_variant
rs185579541	intron_variant
rs185580081	intron_variant,genic_upstream_transcript_variant
rs185586013	intron_variant
rs185596181	genic_downstream_transcript_variant,intron_variant
rs185611591	intron_variant,genic_upstream_transcript_variant
rs185613765	intron_variant
rs185789054	intron_variant
rs185799332	genic_downstream_transcript_variant,intron_variant

rs185822587	intron_variant
rs185823960	genic_downstream_transcript_variant,intron_variant
rs185824789	intron_variant
rs185828988	intron_variant,genic_upstream_transcript_variant
rs185831140	intron_variant
rs185836885	genic_downstream_transcript_variant,intron_variant
rs185859315	intron_variant,genic_upstream_transcript_variant
rs185874674	intron_variant,genic_upstream_transcript_variant
rs185874979	intron_variant,genic_upstream_transcript_variant
rs185881048	intron_variant
rs185915651	intron_variant
rs185929204	intron_variant,genic_upstream_transcript_variant
rs185931949	intron_variant
rs185974584	intron_variant,genic_upstream_transcript_variant
rs186031932	intron_variant
rs186037588	upstream_transcript_variant,intron_variant,2KB_upstream_variant,genic_upstream_transcript_variant
rs186052051	genic_downstream_transcript_variant,intron_variant
rs186107741	intron_variant,genic_upstream_transcript_variant
rs186145433	intron_variant
rs186153186	genic_downstream_transcript_variant,intron_variant
rs186174347	intron_variant
rs186178894	genic_downstream_transcript_variant,intron_variant
rs186375389	intron_variant,genic_upstream_transcript_variant
rs186462523	intron_variant
rs186469831	intron_variant
rs186475635	genic_downstream_transcript_variant,intron_variant
rs186482548	intron_variant,genic_upstream_transcript_variant
rs186489621	intron_variant,genic_upstream_transcript_variant
rs186494385	intron_variant,genic_upstream_transcript_variant
rs186496275	intron_variant,genic_upstream_transcript_variant
rs186521391	intron_variant,genic_upstream_transcript_variant
rs186521516	intron_variant
rs186531870	intron_variant
rs186533526	intron_variant,genic_upstream_transcript_variant
rs186553329	intron_variant,genic_upstream_transcript_variant
rs186576712	genic_downstream_transcript_variant,intron_variant
rs186583408	genic_downstream_transcript_variant,intron_variant
rs186669569	intron_variant,genic_upstream_transcript_variant
rs186698354	intron_variant
rs186703671	intron_variant,genic_upstream_transcript_variant
rs186724809	intron_variant,genic_upstream_transcript_variant
rs186725197	intron_variant
rs186739631	intron_variant,genic_upstream_transcript_variant
rs186742019	intron_variant
rs186747779	intron_variant,genic_upstream_transcript_variant
rs186774701	intron_variant,genic_upstream_transcript_variant
rs186809804	intron_variant
rs186853335	intron_variant
rs186856609	intron_variant
rs186945227	intron_variant,genic_upstream_transcript_variant
rs186990910	intron_variant,genic_upstream_transcript_variant
rs187042844	intron_variant
rs187051097	intron_variant,genic_upstream_transcript_variant
rs187058025	genic_downstream_transcript_variant,intron_variant
rs187060407	intron_variant,genic_upstream_transcript_variant
rs187110456	intron_variant,genic_upstream_transcript_variant
rs187149837	intron_variant,genic_upstream_transcript_variant
rs187315452	genic_downstream_transcript_variant,intron_variant,downstream_transcript_variant
rs187317333	intron_variant
rs187338174	genic_downstream_transcript_variant,intron_variant
rs187340553	intron_variant
rs187342158	intron_variant
rs187350432	intron_variant
rs187352390	genic_downstream_transcript_variant,intron_variant
rs187353241	intron_variant
rs187367634	intron_variant
rs187380750	genic_downstream_transcript_variant,intron_variant
rs187384576	genic_downstream_transcript_variant,intron_variant
rs187389410	intron_variant,genic_upstream_transcript_variant
rs187390486	intron_variant,genic_upstream_transcript_variant
rs187390709	intron_variant
rs187481429	genic_downstream_transcript_variant,intron_variant
rs187560434	intron_variant
rs187576681	intron_variant

rs187584067	upstream_transcript_variant,intron_variant,2KB_upstream_variant,genic_upstream_transcript_variant
rs187598219	intron_variant,genic_upstream_transcript_variant
rs187598616	upstream_transcript_variant,intron_variant,2KB_upstream_variant,genic_upstream_transcript_variant
rs187607500	intron_variant,genic_upstream_transcript_variant
rs187628434	intron_variant
rs187658561	intron_variant
rs187779339	genic_downstream_transcript_variant,intron_variant
rs187911662	intron_variant
rs187917758	genic_downstream_transcript_variant,intron_variant
rs187921256	genic_downstream_transcript_variant,intron_variant
rs187922867	intron_variant,genic_upstream_transcript_variant
rs187950675	intron_variant
rs187952940	intron_variant,genic_upstream_transcript_variant
rs188000596	upstream_transcript_variant,intron_variant,2KB_upstream_variant,genic_upstream_transcript_variant
rs188092770	intron_variant,genic_upstream_transcript_variant
rs188098558	intron_variant,genic_upstream_transcript_variant
rs188109475	intron_variant,genic_upstream_transcript_variant
rs188149889	genic_downstream_transcript_variant,intron_variant
rs188154452	intron_variant
rs188169600	genic_downstream_transcript_variant,intron_variant
rs188174496	upstream_transcript_variant,intron_variant,genic_upstream_transcript_variant
rs188176286	intron_variant
rs188194441	intron_variant
rs188202245	intron_variant,genic_upstream_transcript_variant
rs188257776	genic_downstream_transcript_variant,intron_variant
rs188365840	upstream_transcript_variant,intron_variant,2KB_upstream_variant,genic_upstream_transcript_variant
rs188382413	intron_variant
rs188442041	intron_variant,genic_upstream_transcript_variant
rs188443459	intron_variant,genic_upstream_transcript_variant
rs188453969	genic_downstream_transcript_variant,intron_variant
rs188487065	intron_variant
rs188505702	intron_variant
rs188580437	intron_variant
rs188581602	intron_variant
rs188584574	intron_variant,genic_upstream_transcript_variant
rs188589350	genic_downstream_transcript_variant,intron_variant
rs188590680	genic_downstream_transcript_variant,intron_variant
rs188591194	genic_downstream_transcript_variant,intron_variant
rs188602755	intron_variant,genic_upstream_transcript_variant
rs188611134	intron_variant
rs188625229	intron_variant,genic_upstream_transcript_variant
rs188625460	intron_variant
rs188701834	intron_variant
rs188710120	intron_variant
rs188721542	intron_variant,genic_upstream_transcript_variant
rs188724450	genic_downstream_transcript_variant,intron_variant
rs188731635	intron_variant,genic_upstream_transcript_variant
rs188798273	intron_variant
rs188804282	intron_variant
rs188824621	intron_variant,genic_upstream_transcript_variant
rs188828405	intron_variant,genic_upstream_transcript_variant
rs188835223	genic_downstream_transcript_variant,intron_variant
rs188839163	intron_variant
rs188839217	genic_downstream_transcript_variant,intron_variant
rs188852840	intron_variant,genic_upstream_transcript_variant
rs188873275	intron_variant,genic_upstream_transcript_variant
rs188877694	intron_variant,genic_upstream_transcript_variant
rs188953277	intron_variant
rs188965473	intron_variant
rs188970221	genic_downstream_transcript_variant,intron_variant
rs188983319	intron_variant,genic_upstream_transcript_variant
rs188993740	intron_variant,genic_upstream_transcript_variant
rs188993773	intron_variant
rs188998817	intron_variant,genic_upstream_transcript_variant
rs189048908	intron_variant
rs189209962	genic_downstream_transcript_variant,intron_variant
rs189232404	intron_variant
rs189240181	intron_variant
rs189242797	genic_downstream_transcript_variant,intron_variant
rs189253889	genic_downstream_transcript_variant,intron_variant
rs189261959	intron_variant,genic_upstream_transcript_variant
rs189269316	intron_variant,genic_upstream_transcript_variant
rs189269601	intron_variant
rs189272965	intron_variant

rs189300335	intron_variant,genic_upstream_transcript_variant
rs189343414	genic_downstream_transcript_variant,intron_variant
rs189441404	intron_variant,genic_upstream_transcript_variant
rs189483394	intron_variant
rs189496481	intron_variant,genic_upstream_transcript_variant
rs189500122	intron_variant,genic_upstream_transcript_variant
rs189511032	intron_variant,genic_upstream_transcript_variant
rs189548541	intron_variant,genic_downstream_transcript_variant
rs189587519	intron_variant
rs189603147	intron_variant
rs189734950	intron_variant,genic_upstream_transcript_variant
rs189744686	intron_variant,genic_upstream_transcript_variant
rs189745349	intron_variant,genic_upstream_transcript_variant
rs189762447	intron_variant
rs189763247	intron_variant
rs189802564	intron_variant,genic_upstream_transcript_variant
rs189810227	coding_sequence_variant,non_coding_transcript_variant,genic_downstream_transcript_variant,missense_variant
rs189814561	intron_variant,genic_upstream_transcript_variant
rs189840637	intron_variant
rs189850806	intron_variant
rs189861057	intron_variant,genic_downstream_transcript_variant
rs189862274	intron_variant,genic_downstream_transcript_variant
rs189874389	intron_variant,genic_downstream_transcript_variant
rs189879523	intron_variant
rs190002206	intron_variant
rs190002377	intron_variant,genic_upstream_transcript_variant
rs190008103	intron_variant,genic_upstream_transcript_variant
rs190049498	intron_variant,genic_upstream_transcript_variant
rs190056562	intron_variant,genic_downstream_transcript_variant
rs190081449	intron_variant,genic_upstream_transcript_variant
rs190087766	intron_variant
rs190096557	intron_variant
rs190096796	intron_variant,genic_upstream_transcript_variant
rs190100786	intron_variant
rs190109578	intron_variant
rs190119214	intron_variant
rs190138665	intron_variant,genic_upstream_transcript_variant
rs190144620	intron_variant
rs190271021	intron_variant,genic_upstream_transcript_variant
rs190300865	intron_variant,genic_downstream_transcript_variant
rs190373292	intron_variant,genic_downstream_transcript_variant
rs190384648	intron_variant,genic_upstream_transcript_variant
rs190417625	intron_variant
rs190553891	intron_variant
rs190577791	intron_variant,genic_upstream_transcript_variant
rs190645639	intron_variant,genic_upstream_transcript_variant
rs190647436	intron_variant
rs190676713	intron_variant,genic_downstream_transcript_variant
rs190679682	intron_variant,genic_upstream_transcript_variant
rs190785973	intron_variant,genic_upstream_transcript_variant
rs190809443	intron_variant
rs190895070	intron_variant,genic_downstream_transcript_variant
rs190896373	intron_variant
rs190902809	intron_variant,genic_upstream_transcript_variant
rs190909279	intron_variant,genic_downstream_transcript_variant
rs190942459	intron_variant,genic_upstream_transcript_variant
rs190947831	intron_variant,genic_upstream_transcript_variant
rs190987423	intron_variant,genic_downstream_transcript_variant
rs191015255	intron_variant,genic_upstream_transcript_variant
rs191019243	intron_variant
rs191023329	intron_variant,genic_downstream_transcript_variant
rs191030851	intron_variant
rs191043034	intron_variant,genic_downstream_transcript_variant
rs191060445	intron_variant,genic_upstream_transcript_variant
rs191064598	2KB_upstream_variant,upstream_transcript_variant,intron_variant,genic_upstream_transcript_variant
rs191068881	genic_downstream_transcript_variant,non_coding_transcript_variant,3_prime_UTR_variant
rs191070842	2KB_upstream_variant,upstream_transcript_variant,intron_variant,genic_upstream_transcript_variant
rs191083829	intron_variant
rs191095511	intron_variant,genic_upstream_transcript_variant
rs191147850	intron_variant,genic_downstream_transcript_variant
rs191148707	intron_variant,genic_upstream_transcript_variant
rs191202336	2KB_upstream_variant,upstream_transcript_variant,intron_variant,genic_upstream_transcript_variant
rs191268392	intron_variant,genic_downstream_transcript_variant
rs191324212	intron_variant,genic_upstream_transcript_variant

rs191344711	intron_variant
rs191414839	intron_variant,genic_upstream_transcript_variant
rs191422903	intron_variant,genic_upstream_transcript_variant
rs191429758	intron_variant
rs191438754	intron_variant,genic_downstream_transcript_variant
rs191439857	intron_variant,genic_upstream_transcript_variant
rs191481021	intron_variant,genic_upstream_transcript_variant
rs191498755	intron_variant
rs191593694	intron_variant,genic_upstream_transcript_variant
rs191600071	intron_variant
rs191605951	intron_variant,genic_downstream_transcript_variant
rs191622610	intron_variant,genic_downstream_transcript_variant
rs191640048	intron_variant,genic_downstream_transcript_variant
rs191690926	intron_variant,genic_upstream_transcript_variant
rs191707300	intron_variant
rs191717411	intron_variant
rs191800706	intron_variant,genic_upstream_transcript_variant
rs191800900	intron_variant,genic_upstream_transcript_variant
rs191833020	intron_variant,genic_downstream_transcript_variant
rs191851036	intron_variant
rs191857662	intron_variant
rs191860708	intron_variant
rs191884083	intron_variant,genic_upstream_transcript_variant
rs191903304	intron_variant
rs191965531	intron_variant
rs191982888	intron_variant
rs192005782	2KB_upstream_variant,upstream_transcript_variant,intron_variant,genic_upstream_transcript_variant
rs192018711	intron_variant,genic_downstream_transcript_variant
rs192030136	intron_variant,genic_upstream_transcript_variant
rs192203349	intron_variant
rs192225324	intron_variant,genic_upstream_transcript_variant
rs192268529	intron_variant
rs192289298	intron_variant,genic_upstream_transcript_variant
rs192293739	intron_variant
rs192296541	intron_variant,genic_downstream_transcript_variant
rs192298985	intron_variant
rs192309281	intron_variant
rs192328198	intron_variant
rs192335540	intron_variant
rs192340739	intron_variant,genic_downstream_transcript_variant
rs192344311	intron_variant,genic_downstream_transcript_variant
rs192345064	intron_variant,genic_upstream_transcript_variant
rs192365606	intron_variant,genic_downstream_transcript_variant
rs192424541	intron_variant
rs192428094	intron_variant
rs192447663	intron_variant,genic_upstream_transcript_variant
rs192448217	intron_variant,genic_downstream_transcript_variant
rs192456832	intron_variant,genic_upstream_transcript_variant
rs192480608	intron_variant,genic_upstream_transcript_variant
rs192519414	intron_variant,genic_upstream_transcript_variant
rs192521576	intron_variant,genic_upstream_transcript_variant
rs192530910	intron_variant,genic_downstream_transcript_variant
rs192578133	intron_variant
rs192586871	intron_variant,genic_upstream_transcript_variant
rs192587052	intron_variant,genic_upstream_transcript_variant
rs192590250	intron_variant
rs192600901	intron_variant,genic_downstream_transcript_variant
rs192669437	intron_variant,genic_upstream_transcript_variant
rs192687135	intron_variant
rs192703787	2KB_upstream_variant,upstream_transcript_variant,intron_variant,genic_upstream_transcript_variant
rs192714031	intron_variant
rs192730265	intron_variant,genic_upstream_transcript_variant
rs192771581	intron_variant,genic_downstream_transcript_variant
rs192775415	intron_variant
rs192897975	intron_variant
rs192907924	intron_variant,genic_upstream_transcript_variant
rs192918959	intron_variant,genic_upstream_transcript_variant
rs192965555	intron_variant,genic_upstream_transcript_variant
rs192974833	intron_variant,genic_upstream_transcript_variant
rs193015869	intron_variant,genic_downstream_transcript_variant
rs193082291	intron_variant
rs193094079	intron_variant,genic_upstream_transcript_variant
rs193095029	intron_variant,genic_upstream_transcript_variant
rs193098192	intron_variant,genic_upstream_transcript_variant

rs193114393	intron_variant,genic_downstream_transcript_variant
rs193124340	intron_variant
rs193182346	intron_variant
rs193189509	intron_variant,genic_upstream_transcript_variant
rs193198366	intron_variant,genic_downstream_transcript_variant
rs193242530	intron_variant
rs193246342	intron_variant
rs193248311	intron_variant,genic_upstream_transcript_variant
rs193255597	intron_variant,genic_upstream_transcript_variant
rs193265846	intron_variant
rs199504586	intron_variant,splice_acceptor_variant
rs199549757	coding_sequence_variant,synonymous_variant,non_coding_transcript_variant
rs199583656	intron_variant
rs199595181	missense_variant,coding_sequence_variant,intron_variant,genic_upstream_transcript_variant,non_coding_transcript_variant
rs199599793	intron_variant
rs199599833	coding_sequence_variant,synonymous_variant,non_coding_transcript_variant,genic_downstream_transcript_variant
rs199609454	intron_variant
rs199661959	intron_variant,genic_upstream_transcript_variant
rs199685502	intron_variant
rs199701456	coding_sequence_variant,synonymous_variant,non_coding_transcript_variant,genic_downstream_transcript_variant
rs199705069	coding_sequence_variant,synonymous_variant,non_coding_transcript_variant
rs199718542	intron_variant,genic_upstream_transcript_variant
rs199760475	intron_variant
rs199761112	intron_variant
rs199808716	coding_sequence_variant,intron_variant,missense_variant
rs199810399	intron_variant,genic_upstream_transcript_variant
rs199812384	coding_sequence_variant,synonymous_variant,intron_variant,genic_upstream_transcript_variant,non_coding_transcript_variant
rs199846233	intron_variant,genic_upstream_transcript_variant
rs199867668	intron_variant,genic_downstream_transcript_variant
rs199897137	intron_variant,genic_upstream_transcript_variant
rs199898841	coding_sequence_variant,synonymous_variant,intron_variant,genic_upstream_transcript_variant,non_coding_transcript_variant
rs199899286	intron_variant
rs199918721	intron_variant
rs199930636	intron_variant,genic_upstream_transcript_variant
rs199941443	downstream_transcript_variant,intron_variant,genic_downstream_transcript_variant
rs199951038	intron_variant,genic_upstream_transcript_variant
rs199962199	intron_variant
rs200000704	coding_sequence_variant,synonymous_variant,non_coding_transcript_variant
rs200022810	intron_variant
rs200023903	missense_variant,coding_sequence_variant,intron_variant,genic_upstream_transcript_variant,non_coding_transcript_variant
rs200041888	intron_variant
rs200113917	intron_variant,genic_upstream_transcript_variant
rs200138825	coding_sequence_variant,synonymous_variant,non_coding_transcript_variant,genic_downstream_transcript_variant
rs200147283	intron_variant,genic_upstream_transcript_variant
rs200147795	intron_variant
rs200157445	intron_variant,genic_upstream_transcript_variant
rs200160046	intron_variant
rs200184140	coding_sequence_variant,synonymous_variant,non_coding_transcript_variant,genic_downstream_transcript_variant
rs200209903	intron_variant,genic_upstream_transcript_variant
rs200210428	coding_sequence_variant,synonymous_variant,non_coding_transcript_variant
rs200260902	intron_variant
rs200269358	coding_sequence_variant,non_coding_transcript_variant,missense_variant
rs200280369	intron_variant
rs200287865	intron_variant,genic_downstream_transcript_variant
rs200318158	intron_variant,genic_downstream_transcript_variant
rs200327980	intron_variant,genic_upstream_transcript_variant
rs200334747	intron_variant
rs200340066	coding_sequence_variant,synonymous_variant,non_coding_transcript_variant,genic_downstream_transcript_variant
rs200341323	coding_sequence_variant,synonymous_variant,non_coding_transcript_variant,missense_variant
rs200350112	upstream_transcript_variant,intron_variant,genic_upstream_transcript_variant
rs200397535	intron_variant,genic_downstream_transcript_variant
rs200400404	intron_variant,genic_upstream_transcript_variant
rs200403115	coding_sequence_variant,synonymous_variant,non_coding_transcript_variant,5_prime_UTR_variant
rs200456335	coding_sequence_variant,synonymous_variant,non_coding_transcript_variant,genic_downstream_transcript_variant
rs200457637	intron_variant
rs200472537	coding_sequence_variant,synonymous_variant,intron_variant,genic_upstream_transcript_variant,non_coding_transcript_variant
rs200487706	intron_variant,genic_upstream_transcript_variant
rs200573684	genic_downstream_transcript_variant,non_coding_transcript_variant,3_prime_UTR_variant
rs200576657	intron_variant
rs200582093	coding_sequence_variant,synonymous_variant,non_coding_transcript_variant
rs200584971	intron_variant
rs200592302	intron_variant,genic_upstream_transcript_variant
rs200599512	intron_variant
rs200635057	intron_variant

rs200641198	coding_sequence_variant,synonymous_variant,intron_variant,genic_upstream_transcript_variant,non_coding_transcript_variant
rs200644032	intron_variant,genic_upstream_transcript_variant
rs200666523	intron_variant
rs200692617	coding_sequence_variant,synonymous_variant,non_coding_transcript_variant,genic_downstream_transcript_variant
rs200725302	intron_variant,genic_downstream_transcript_variant
rs200739117	intron_variant,genic_upstream_transcript_variant
rs200752283	intron_variant
rs200806817	coding_sequence_variant,synonymous_variant,non_coding_transcript_variant
rs200862535	intron_variant,genic_downstream_transcript_variant
rs200865756	coding_sequence_variant,synonymous_variant,non_coding_transcript_variant,5_prime_UTR_variant
rs200911694	intron_variant,genic_upstream_transcript_variant
rs200939415	coding_sequence_variant,synonymous_variant,intron_variant,genic_upstream_transcript_variant,non_coding_transcript_variant
rs200945141	intron_variant,genic_upstream_transcript_variant
rs200992812	intron_variant,genic_upstream_transcript_variant
rs201027191	intron_variant,genic_upstream_transcript_variant
rs201044498	intron_variant,genic_downstream_transcript_variant
rs201079941	downstream_transcript_variant,intron_variant,genic_downstream_transcript_variant
rs201090828	coding_sequence_variant,non_coding_transcript_variant,missense_variant
rs201109546	coding_sequence_variant,synonymous_variant,intron_variant,genic_upstream_transcript_variant,non_coding_transcript_variant
rs201147646	coding_sequence_variant,synonymous_variant,non_coding_transcript_variant,genic_downstream_transcript_variant
rs201181441	coding_sequence_variant,synonymous_variant,non_coding_transcript_variant,5_prime_UTR_variant
rs201184739	intron_variant
rs201186420	intron_variant
rs201223702	intron_variant,genic_upstream_transcript_variant
rs201228499	intron_variant,genic_upstream_transcript_variant
rs201241099	intron_variant,genic_upstream_transcript_variant
rs201258917	coding_sequence_variant,synonymous_variant,intron_variant,genic_upstream_transcript_variant,non_coding_transcript_variant
rs201287489	intron_variant,genic_upstream_transcript_variant
rs201302437	intron_variant,genic_upstream_transcript_variant
rs201319905	intron_variant
rs201323678	intron_variant
rs201326561	coding_sequence_variant,synonymous_variant,non_coding_transcript_variant
rs201348724	intron_variant,genic_upstream_transcript_variant
rs201369312	intron_variant,genic_upstream_transcript_variant
rs201378674	coding_sequence_variant,synonymous_variant,non_coding_transcript_variant
rs201395418	intron_variant
rs201419562	intron_variant
rs201419910	coding_sequence_variant,synonymous_variant,non_coding_transcript_variant,genic_downstream_transcript_variant
rs201458828	intron_variant
rs201463173	intron_variant
rs201491944	intron_variant
rs201495740	intron_variant
rs201500347	intron_variant
rs201530909	intron_variant
rs201551341	intron_variant,genic_upstream_transcript_variant
rs201551961	coding_sequence_variant,synonymous_variant,non_coding_transcript_variant
rs201603404	intron_variant,genic_upstream_transcript_variant
rs201633919	intron_variant,genic_downstream_transcript_variant
rs201634868	intron_variant
rs201699105	intron_variant,genic_upstream_transcript_variant
rs201724557	intron_variant
rs201726372	intron_variant,genic_downstream_transcript_variant
rs201747580	coding_sequence_variant,synonymous_variant,non_coding_transcript_variant,genic_downstream_transcript_variant
rs201770887	intron_variant,genic_upstream_transcript_variant
rs201815666	coding_sequence_variant,synonymous_variant,non_coding_transcript_variant,genic_downstream_transcript_variant
rs201825443	intron_variant,genic_upstream_transcript_variant
rs201829409	intron_variant,genic_downstream_transcript_variant
rs201882845	coding_sequence_variant,synonymous_variant,non_coding_transcript_variant
rs201929141	intron_variant
rs201935135	intron_variant
rs201939285	intron_variant
rs201945798	coding_sequence_variant,synonymous_variant,intron_variant,genic_upstream_transcript_variant,non_coding_transcript_variant
rs201951536	intron_variant
rs201975734	intron_variant
rs201997422	coding_sequence_variant,synonymous_variant,non_coding_transcript_variant,genic_downstream_transcript_variant
rs202016698	intron_variant
rs202049553	intron_variant,genic_upstream_transcript_variant
rs202065067	coding_sequence_variant,synonymous_variant,non_coding_transcript_variant,genic_downstream_transcript_variant
rs202086515	intron_variant,genic_downstream_transcript_variant
rs202097954	intron_variant
rs202110450	missense_variant,coding_sequence_variant,synonymous_variant,non_coding_transcript_variant,genic_downstream_transcript_variant
rs202113885	intron_variant,genic_upstream_transcript_variant
rs202135726	coding_sequence_variant,non_coding_transcript_variant,missense_variant
rs202165096	intron_variant,genic_upstream_transcript_variant

rs202168939	intron_variant
rs202198336	coding_sequence_variant,synonymous_variant,non_coding_transcript_variant
rs202213085	intron_variant
rs202214034	intron_variant
rs202215501	intron_variant
rs202229900	intron_variant,genic_downstream_transcript_variant
rs202236031	5_prime_UTR_variant,missense_variant,coding_sequence_variant,synonymous_variant,non_coding_transcript_variant
rs207468445	intron_variant,genic_upstream_transcript_variant
rs207468446	intron_variant
rs267601244	coding_sequence_variant,non_coding_transcript_variant,genic_downstream_transcript_variant,missense_variant
rs367595475	intron_variant,genic_upstream_transcript_variant
rs367611023	intron_variant,genic_upstream_transcript_variant
rs367649400	coding_sequence_variant,intron_variant,missense_variant
rs367709280	intron_variant,genic_upstream_transcript_variant
rs367710867	upstream_transcript_variant,intron_variant,genic_upstream_transcript_variant
rs367711750	intron_variant,genic_downstream_transcript_variant
rs367722737	missense_variant,coding_sequence_variant,intron_variant,genic_upstream_transcript_variant,non_coding_transcript_variant
rs367723888	intron_variant
rs367734724	intron_variant
rs367751541	intron_variant
rs367772588	coding_sequence_variant,synonymous_variant,non_coding_transcript_variant,genic_downstream_transcript_variant
rs367981289	intron_variant
rs368000670	intron_variant,genic_upstream_transcript_variant
rs368003053	intron_variant,genic_upstream_transcript_variant
rs368017318	intron_variant,genic_downstream_transcript_variant
rs368023482	intron_variant
rs368038865	intron_variant
rs368105512	intron_variant,genic_downstream_transcript_variant
rs368140353	intron_variant
rs368150367	upstream_transcript_variant,intron_variant,genic_upstream_transcript_variant
rs368203119	intron_variant,genic_upstream_transcript_variant
rs368223040	intron_variant
rs368227467	intron_variant,genic_upstream_transcript_variant
rs368228150	intron_variant
rs368233103	intron_variant
rs368247435	intron_variant,genic_downstream_transcript_variant
rs368249740	coding_sequence_variant,synonymous_variant,intron_variant,genic_upstream_transcript_variant,non_coding_transcript_variant
rs368285058	intron_variant,genic_upstream_transcript_variant
rs368305676	intron_variant
rs368308893	intron_variant
rs368312290	coding_sequence_variant,non_coding_transcript_variant,genic_downstream_transcript_variant,missense_variant
rs368325045	intron_variant,genic_downstream_transcript_variant
rs368374512	intron_variant
rs368375848	intron_variant,genic_upstream_transcript_variant
rs368388054	intron_variant,genic_downstream_transcript_variant
rs368406490	intron_variant,genic_downstream_transcript_variant
rs368491756	intron_variant,genic_upstream_transcript_variant
rs368501036	intron_variant
rs368505502	intron_variant
rs368514774	intron_variant,genic_downstream_transcript_variant
rs368564717	intron_variant,genic_upstream_transcript_variant
rs368589212	intron_variant,genic_upstream_transcript_variant
rs368625804	intron_variant,genic_upstream_transcript_variant
rs368774874	intron_variant
rs368779177	genic_upstream_transcript_variant,intron_variant,upstream_transcript_variant
rs368798475	intron_variant,genic_upstream_transcript_variant
rs368822968	intron_variant
rs368852748	intron_variant
rs368855125	intron_variant
rs368863750	intron_variant,genic_upstream_transcript_variant
rs368863888	genic_upstream_transcript_variant,intron_variant,upstream_transcript_variant
rs368864111	intron_variant,genic_upstream_transcript_variant
rs368866025	intron_variant
rs369018334	intron_variant
rs369026988	intron_variant,genic_upstream_transcript_variant
rs369178779	intron_variant,genic_upstream_transcript_variant
rs369191825	genic_downstream_transcript_variant,intron_variant
rs369325536	genic_downstream_transcript_variant,intron_variant
rs369327837	genic_downstream_transcript_variant,intron_variant
rs369338451	intron_variant
rs369381534	intron_variant,genic_upstream_transcript_variant
rs369415260	genic_downstream_transcript_variant,intron_variant
rs369439819	upstream_transcript_variant,genic_upstream_transcript_variant,intron_variant,2KB_upstream_variant
rs369522996	intron_variant

rs369545465	intron_variant,genic_upstream_transcript_variant
rs369578950	intron_variant
rs369581100	intron_variant
rs369589827	genic_downstream_transcript_variant,intron_variant
rs369607133	intron_variant
rs369649529	intron_variant,genic_upstream_transcript_variant
rs369696160	intron_variant
rs369697508	genic_downstream_transcript_variant,intron_variant
rs369705803	5_prime_UTR_variant,stop_gained,coding_sequence_variant,synonymous_variant,non_coding_transcript_variant
rs369708852	intron_variant,genic_upstream_transcript_variant
rs369713005	intron_variant
rs369765654	genic_downstream_transcript_variant,non_coding_transcript_variant,3_prime_UTR_variant
rs369805886	intron_variant,genic_upstream_transcript_variant
rs369838736	intron_variant
rs369881174	intron_variant
rs369881530	intron_variant,genic_upstream_transcript_variant
rs369885235	intron_variant
rs369905546	genic_upstream_transcript_variant,intron_variant,upstream_transcript_variant
rs369954696	intron_variant
rs369958862	upstream_transcript_variant,genic_upstream_transcript_variant,intron_variant,2KB_upstream_variant
rs370000291	intron_variant
rs370019802	intron_variant
rs370051917	intron_variant,genic_upstream_transcript_variant
rs370073932	genic_downstream_transcript_variant,intron_variant
rs370080621	intron_variant,genic_upstream_transcript_variant
rs370083427	intron_variant
rs370087590	intron_variant
rs370139929	genic_downstream_transcript_variant,intron_variant
rs370195854	intron_variant
rs370267876	intron_variant,genic_upstream_transcript_variant
rs370271056	intron_variant,genic_upstream_transcript_variant
rs370274186	genic_downstream_transcript_variant,intron_variant
rs370278865	intron_variant
rs370292680	intron_variant,genic_upstream_transcript_variant
rs370359785	intron_variant,genic_upstream_transcript_variant
rs370374012	intron_variant
rs370379046	intron_variant
rs370381279	intron_variant
rs370397027	intron_variant
rs370444948	genic_downstream_transcript_variant,intron_variant
rs370473029	intron_variant
rs370482774	intron_variant,genic_upstream_transcript_variant
rs370492792	intron_variant,genic_upstream_transcript_variant
rs370593031	genic_downstream_transcript_variant,intron_variant
rs370598559	intron_variant
rs370644103	genic_downstream_transcript_variant,intron_variant
rs370784631	intron_variant
rs370798160	intron_variant,genic_upstream_transcript_variant
rs370815263	genic_downstream_transcript_variant,intron_variant
rs370850638	intron_variant,genic_upstream_transcript_variant
rs370865031	intron_variant
rs370874995	intron_variant,genic_upstream_transcript_variant
rs370914380	intron_variant
rs370980654	intron_variant,genic_upstream_transcript_variant
rs370995016	intron_variant
rs371020340	intron_variant
rs371036461	intron_variant,genic_upstream_transcript_variant
rs371066721	intron_variant,genic_upstream_transcript_variant
rs371079191	intron_variant
rs371083331	genic_downstream_transcript_variant,intron_variant
rs371091263	intron_variant
rs371128674	intron_variant,genic_upstream_transcript_variant
rs371130084	genic_upstream_transcript_variant,intron_variant,upstream_transcript_variant
rs371144483	intron_variant
rs371147842	intron_variant
rs371152827	genic_downstream_transcript_variant,intron_variant
rs371159003	intron_variant
rs371180027	intron_variant,genic_upstream_transcript_variant
rs371241828	intron_variant
rs371267972	intron_variant,genic_upstream_transcript_variant
rs371281495	genic_downstream_transcript_variant,intron_variant
rs371331202	intron_variant
rs371342329	intron_variant,genic_upstream_transcript_variant
rs371348187	genic_downstream_transcript_variant,intron_variant

rs371352675	genic_downstream_transcript_variant,intron_variant
rs371382301	intron_variant,genic_upstream_transcript_variant
rs371498759	genic_upstream_transcript_variant,intron_variant,upstream_transcript_variant
rs371512340	intron_variant
rs371519420	intron_variant,genic_upstream_transcript_variant
rs371530701	intron_variant
rs371551906	intron_variant
rs371556455	intron_variant,genic_upstream_transcript_variant
rs371570367	intron_variant
rs371575923	intron_variant,genic_upstream_transcript_variant
rs371592074	intron_variant,genic_upstream_transcript_variant
rs371600961	genic_downstream_transcript_variant,intron_variant
rs371602744	intron_variant,genic_upstream_transcript_variant
rs371609255	genic_downstream_transcript_variant,intron_variant
rs371610894	intron_variant
rs371627700	intron_variant
rs371636777	intron_variant
rs371656811	genic_downstream_transcript_variant,intron_variant
rs371676464	intron_variant,genic_upstream_transcript_variant
rs371723640	intron_variant,genic_upstream_transcript_variant
rs371725790	intron_variant,genic_upstream_transcript_variant
rs371766461	intron_variant,genic_upstream_transcript_variant
rs371772521	intron_variant,genic_upstream_transcript_variant
rs371773538	genic_downstream_transcript_variant,intron_variant
rs371820499	upstream_transcript_variant,2KB_upstream_variant,intron_variant,genic_upstream_transcript_variant
rs371841510	genic_downstream_transcript_variant,intron_variant
rs371843004	intron_variant
rs371927164	genic_downstream_transcript_variant,non_coding_transcript_variant,coding_sequence_variant,synonymous_variant
rs371941340	coding_sequence_variant,genic_upstream_transcript_variant,intron_variant,missense_variant,non_coding_transcript_variant
rs371954374	intron_variant
rs371968912	genic_downstream_transcript_variant,non_coding_transcript_variant,3_prime_UTR_variant
rs371983332	intron_variant
rs371997437	genic_downstream_transcript_variant,intron_variant
rs372127814	intron_variant,genic_upstream_transcript_variant
rs372233873	intron_variant,genic_upstream_transcript_variant
rs372243309	genic_downstream_transcript_variant,intron_variant
rs372258065	intron_variant
rs372275543	intron_variant,genic_upstream_transcript_variant
rs372314384	intron_variant
rs372320153	coding_sequence_variant,genic_upstream_transcript_variant,intron_variant,missense_variant,non_coding_transcript_variant
rs372320976	intron_variant
rs372355977	intron_variant
rs372394766	genic_upstream_transcript_variant,intron_variant,upstream_transcript_variant
rs372435180	intron_variant
rs372507091	intron_variant
rs372516956	genic_downstream_transcript_variant,intron_variant
rs372548129	genic_downstream_transcript_variant,intron_variant
rs372592914	upstream_transcript_variant,2KB_upstream_variant,intron_variant,genic_upstream_transcript_variant
rs372604482	genic_downstream_transcript_variant,intron_variant
rs372618546	intron_variant
rs372624042	intron_variant,genic_upstream_transcript_variant
rs372627025	intron_variant
rs372654692	upstream_transcript_variant,genic_upstream_transcript_variant,intron_variant,2KB_upstream_variant
rs372658159	intron_variant,genic_upstream_transcript_variant
rs372699296	non_coding_transcript_variant,coding_sequence_variant,missense_variant
rs372782270	genic_upstream_transcript_variant,intron_variant,upstream_transcript_variant
rs372784547	intron_variant
rs372801649	intron_variant
rs372830179	genic_downstream_transcript_variant,non_coding_transcript_variant,3_prime_UTR_variant
rs372968031	intron_variant
rs372974164	intron_variant,genic_upstream_transcript_variant
rs373030124	genic_downstream_transcript_variant,intron_variant
rs373041878	intron_variant
rs373075830	upstream_transcript_variant,2KB_upstream_variant,intron_variant,genic_upstream_transcript_variant
rs373083968	intron_variant,genic_upstream_transcript_variant
rs373087103	intron_variant,genic_upstream_transcript_variant
rs373101012	genic_downstream_transcript_variant,intron_variant
rs373105607	upstream_transcript_variant,genic_upstream_transcript_variant,intron_variant,2KB_upstream_variant
rs373107131	intron_variant
rs373136723	intron_variant
rs373195532	intron_variant,genic_upstream_transcript_variant
rs373221499	intron_variant
rs373253892	genic_downstream_transcript_variant,intron_variant
rs373265119	intron_variant

rs373340716	intron_variant
rs373343541	intron_variant,genic_upstream_transcript_variant
rs373346608	intron_variant,genic_upstream_transcript_variant
rs373352648	intron_variant,genic_upstream_transcript_variant
rs373369008	intron_variant
rs373377385	genic_downstream_transcript_variant,downstream_transcript_variant,intron_variant
rs373378325	intron_variant
rs373437580	coding_sequence_variant,genic_upstream_transcript_variant,intron_variant,synonymous_variant,non_coding_transcript_variant
rs373470921	genic_downstream_transcript_variant,intron_variant
rs373591857	intron_variant
rs373598739	intron_variant
rs373603482	intron_variant
rs373605293	intron_variant
rs373618465	intron_variant
rs373634891	genic_downstream_transcript_variant,intron_variant
rs373648323	intron_variant
rs373710761	genic_downstream_transcript_variant,intron_variant
rs373721078	genic_upstream_transcript_variant,intron_variant,upstream_transcript_variant
rs373721129	genic_downstream_transcript_variant,intron_variant
rs373802217	intron_variant,genic_upstream_transcript_variant
rs373806876	intron_variant,genic_upstream_transcript_variant
rs373809886	intron_variant
rs373819683	intron_variant,genic_upstream_transcript_variant
rs373824701	intron_variant
rs373837817	intron_variant
rs373842187	genic_downstream_transcript_variant,intron_variant
rs373854151	genic_downstream_transcript_variant,intron_variant
rs373857586	genic_downstream_transcript_variant,non_coding_transcript_variant,coding_sequence_variant,synonymous_variant
rs373914399	intron_variant,genic_upstream_transcript_variant
rs373940602	intron_variant
rs373972528	genic_downstream_transcript_variant,intron_variant
rs374060469	intron_variant
rs374292243	intron_variant,genic_upstream_transcript_variant
rs374329836	intron_variant
rs374348025	genic_upstream_transcript_variant,intron_variant,upstream_transcript_variant
rs374470641	intron_variant
rs374488841	genic_downstream_transcript_variant,intron_variant
rs374493155	genic_upstream_transcript_variant,intron_variant,upstream_transcript_variant
rs374551571	intron_variant,genic_upstream_transcript_variant
rs374557763	intron_variant,genic_upstream_transcript_variant
rs374561088	genic_downstream_transcript_variant,intron_variant
rs374567826	intron_variant
rs374619810	intron_variant
rs374641202	intron_variant,genic_upstream_transcript_variant
rs374661181	genic_downstream_transcript_variant,intron_variant
rs374693278	genic_downstream_transcript_variant,intron_variant
rs374759493	intron_variant
rs374793007	intron_variant,genic_upstream_transcript_variant
rs374803014	intron_variant
rs374809300	intron_variant,genic_upstream_transcript_variant
rs374811671	intron_variant
rs374906579	genic_downstream_transcript_variant,intron_variant
rs374960644	intron_variant,genic_upstream_transcript_variant
rs374967407	intron_variant
rs374993712	intron_variant,genic_upstream_transcript_variant
rs374996827	intron_variant,genic_upstream_transcript_variant
rs375002008	intron_variant
rs375075152	genic_downstream_transcript_variant,intron_variant
rs375127375	intron_variant,genic_upstream_transcript_variant
rs375235586	intron_variant,genic_upstream_transcript_variant
rs375266202	genic_downstream_transcript_variant,intron_variant
rs375272270	intron_variant
rs375285594	intron_variant
rs375298045	intron_variant,genic_upstream_transcript_variant
rs375315634	intron_variant,genic_upstream_transcript_variant
rs375317290	intron_variant,genic_upstream_transcript_variant
rs375415206	intron_variant,genic_upstream_transcript_variant
rs375461370	genic_downstream_transcript_variant,intron_variant
rs375476435	intron_variant
rs375500539	genic_downstream_transcript_variant,intron_variant
rs375531399	genic_downstream_transcript_variant,intron_variant
rs375535358	intron_variant,genic_upstream_transcript_variant
rs375566772	genic_downstream_transcript_variant,intron_variant
rs375569461	intron_variant,genic_upstream_transcript_variant

rs375573005	intron_variant,genic_upstream_transcript_variant
rs375621477	genic_downstream_transcript_variant,intron_variant
rs375637790	intron_variant
rs375648753	intron_variant,genic_upstream_transcript_variant
rs375651515	intron_variant,genic_upstream_transcript_variant
rs375674448	intron_variant
rs375694951	intron_variant
rs375700371	intron_variant
rs375733437	intron_variant
rs375756311	intron_variant,genic_upstream_transcript_variant
rs375772148	genic_downstream_transcript_variant,intron_variant
rs375787762	intron_variant
rs375806834	intron_variant
rs375835640	intron_variant,genic_upstream_transcript_variant
rs375843365	genic_downstream_transcript_variant,intron_variant
rs375959187	intron_variant,genic_upstream_transcript_variant
rs376040317	intron_variant,genic_upstream_transcript_variant
rs376045592	intron_variant,genic_upstream_transcript_variant
rs376058256	intron_variant
rs376060463	intron_variant
rs376127365	intron_variant
rs376137897	intron_variant
rs376177826	genic_upstream_transcript_variant,intron_variant,upstream_transcript_variant
rs376182957	intron_variant
rs376192509	intron_variant
rs376195363	genic_downstream_transcript_variant,intron_variant
rs376303518	intron_variant,genic_upstream_transcript_variant
rs376324433	intron_variant
rs376331419	intron_variant,genic_upstream_transcript_variant
rs376369236	intron_variant,genic_upstream_transcript_variant
rs376372586	intron_variant
rs376384548	intron_variant,genic_upstream_transcript_variant
rs376387602	intron_variant
rs376400600	genic_downstream_transcript_variant,intron_variant
rs376402061	intron_variant
rs376408065	intron_variant
rs376409399	intron_variant
rs376416146	genic_upstream_transcript_variant,intron_variant,upstream_transcript_variant
rs376433266	intron_variant
rs376444143	intron_variant,genic_upstream_transcript_variant
rs376572638	intron_variant,genic_upstream_transcript_variant
rs376584613	intron_variant
rs376585988	intron_variant
rs376669976	intron_variant
rs376673337	intron_variant,genic_upstream_transcript_variant
rs376674015	intron_variant
rs376678277	intron_variant,genic_upstream_transcript_variant
rs376686052	intron_variant
rs376711164	genic_downstream_transcript_variant,intron_variant
rs376745513	genic_downstream_transcript_variant,downstream_transcript_variant,intron_variant
rs376751279	genic_downstream_transcript_variant,non_coding_transcript_variant,coding_sequence_variant,missense_variant
rs376789821	intron_variant
rs376790354	intron_variant,genic_upstream_transcript_variant
rs376814199	genic_downstream_transcript_variant,intron_variant
rs376834205	intron_variant,genic_upstream_transcript_variant
rs376884212	intron_variant,genic_upstream_transcript_variant
rs376914803	genic_downstream_transcript_variant,intron_variant
rs376928397	non_coding_transcript_variant,coding_sequence_variant,missense_variant
rs376929051	intron_variant,genic_upstream_transcript_variant
rs377015915	intron_variant,genic_upstream_transcript_variant
rs377071518	upstream_transcript_variant,2KB_upstream_variant,intron_variant,genic_upstream_transcript_variant
rs377153244	intron_variant
rs377271832	intron_variant
rs377273708	intron_variant
rs377276879	intron_variant
rs377281773	intron_variant
rs377343977	intron_variant
rs377420134	genic_downstream_transcript_variant,intron_variant
rs377459941	intron_variant,genic_upstream_transcript_variant
rs377462977	genic_downstream_transcript_variant,downstream_transcript_variant,intron_variant
rs377463516	intron_variant,genic_upstream_transcript_variant
rs377469830	intron_variant
rs377476551	intron_variant,genic_upstream_transcript_variant
rs377522649	upstream_transcript_variant,2KB_upstream_variant,intron_variant,genic_upstream_transcript_variant

rs377523653	intron_variant
rs377526949	downstream_transcript_variant,500B_downstream_variant
rs377528988	intron_variant
rs377536391	intron_variant,genic_upstream_transcript_variant
rs377618312	intron_variant,genic_upstream_transcript_variant
rs377624981	intron_variant
rs377658190	intron_variant
rs377758389	genic_downstream_transcript_variant,intron_variant
rs386717097	intron_variant,genic_upstream_transcript_variant
rs386717098	intron_variant
rs386717099	intron_variant
rs397815915	intron_variant,genic_upstream_transcript_variant
rs527297841	intron_variant,genic_upstream_transcript_variant
rs527299112	intron_variant,genic_upstream_transcript_variant
rs527326849	genic_downstream_transcript_variant,intron_variant
rs527334474	intron_variant,genic_upstream_transcript_variant
rs527372963	upstream_transcript_variant,genic_upstream_transcript_variant,intron_variant,2KB_upstream_variant
rs527420953	intron_variant
rs527445368	upstream_transcript_variant,2KB_upstream_variant,intron_variant,genic_upstream_transcript_variant
rs527455120	intron_variant,genic_upstream_transcript_variant
rs527462682	genic_downstream_transcript_variant,intron_variant
rs527471177	intron_variant
rs527505347	intron_variant,genic_upstream_transcript_variant
rs527550963	upstream_transcript_variant,genic_upstream_transcript_variant,intron_variant,2KB_upstream_variant
rs527561428	intron_variant,genic_upstream_transcript_variant
rs527568562	genic_downstream_transcript_variant,intron_variant
rs527574159	genic_downstream_transcript_variant,intron_variant
rs527599112	intron_variant
rs527649236	genic_downstream_transcript_variant,intron_variant
rs527650469	intron_variant,genic_upstream_transcript_variant
rs527656939	intron_variant,genic_upstream_transcript_variant
rs527663775	intron_variant
rs527686191	genic_downstream_transcript_variant,intron_variant
rs527712364	intron_variant,genic_upstream_transcript_variant
rs527743235	intron_variant
rs527786414	intron_variant
rs527792763	intron_variant
rs527807128	intron_variant
rs527810273	genic_downstream_transcript_variant,intron_variant
rs527888516	intron_variant,genic_upstream_transcript_variant
rs527892794	intron_variant,genic_upstream_transcript_variant
rs527933016	intron_variant,genic_upstream_transcript_variant
rs527937695	intron_variant
rs527977246	intron_variant
rs527977384	genic_downstream_transcript_variant,intron_variant
rs527985565	intron_variant,genic_upstream_transcript_variant
rs528017332	intron_variant
rs528019768	intron_variant,genic_upstream_transcript_variant
rs528029309	intron_variant,genic_upstream_transcript_variant
rs528053401	intron_variant
rs528060896	genic_downstream_transcript_variant,intron_variant
rs528063210	intron_variant
rs528155409	intron_variant,genic_upstream_transcript_variant
rs528157054	intron_variant
rs528168908	intron_variant,genic_upstream_transcript_variant
rs528172363	genic_downstream_transcript_variant,intron_variant
rs528195279	intron_variant
rs528209488	genic_downstream_transcript_variant,intron_variant
rs528224773	intron_variant
rs528225840	genic_downstream_transcript_variant,intron_variant
rs528320542	intron_variant
rs528342908	intron_variant,genic_upstream_transcript_variant
rs528372333	genic_downstream_transcript_variant,intron_variant
rs528380080	intron_variant,genic_upstream_transcript_variant
rs528410037	genic_upstream_transcript_variant,intron_variant,upstream_transcript_variant
rs528423600	intron_variant
rs528470644	intron_variant,genic_upstream_transcript_variant
rs528509838	intron_variant,genic_upstream_transcript_variant
rs528512136	intron_variant
rs528518989	intron_variant,genic_upstream_transcript_variant
rs528531020	intron_variant,genic_upstream_transcript_variant
rs528627456	intron_variant,genic_upstream_transcript_variant
rs528636720	genic_downstream_transcript_variant,intron_variant
rs528639785	intron_variant,genic_upstream_transcript_variant

rs528649138	genic_upstream_transcript_variant,2KB_upstream_variant,intron_variant,upstream_transcript_variant
rs528662007	intron_variant
rs528695579	genic_downstream_transcript_variant,intron_variant
rs528712262	intron_variant,genic_upstream_transcript_variant
rs528747394	intron_variant
rs528788133	non_coding_transcript_variant,coding_sequence_variant,synonymous_variant
rs528791880	intron_variant,genic_upstream_transcript_variant
rs528802208	intron_variant
rs528806881	genic_downstream_transcript_variant,intron_variant
rs528808993	genic_downstream_transcript_variant,intron_variant
rs528850278	intron_variant
rs528884923	intron_variant,genic_upstream_transcript_variant
rs528885956	intron_variant
rs528898413	intron_variant,genic_upstream_transcript_variant
rs528922966	genic_downstream_transcript_variant,intron_variant
rs528940599	genic_downstream_transcript_variant,intron_variant
rs528946014	intron_variant,genic_upstream_transcript_variant
rs528951146	intron_variant
rs528974254	intron_variant
rs528987834	intron_variant
rs528997092	intron_variant
rs529009892	intron_variant,genic_upstream_transcript_variant
rs529035673	intron_variant,genic_upstream_transcript_variant
rs529055156	intron_variant
rs529061864	intron_variant,genic_upstream_transcript_variant
rs529074962	intron_variant
rs529104974	intron_variant,genic_upstream_transcript_variant
rs529146979	intron_variant,genic_upstream_transcript_variant
rs529152273	intron_variant
rs529178455	upstream_transcript_variant,2KB_upstream_variant,intron_variant,genic_upstream_transcript_variant
rs529190112	intron_variant
rs529241881	intron_variant,genic_upstream_transcript_variant
rs529266118	intron_variant,genic_upstream_transcript_variant
rs529292238	intron_variant,genic_upstream_transcript_variant
rs529325798	genic_downstream_transcript_variant,intron_variant
rs529356574	intron_variant,genic_upstream_transcript_variant
rs529373051	intron_variant,genic_upstream_transcript_variant
rs529401634	intron_variant,genic_upstream_transcript_variant
rs529441817	intron_variant
rs529446580	intron_variant
rs529447906	intron_variant,genic_upstream_transcript_variant
rs529499199	genic_downstream_transcript_variant,intron_variant
rs529501961	intron_variant
rs529524400	intron_variant,genic_upstream_transcript_variant
rs529541759	intron_variant
rs529559092	genic_downstream_transcript_variant,intron_variant
rs529584256	intron_variant,genic_upstream_transcript_variant
rs529640289	upstream_transcript_variant,2KB_upstream_variant,intron_variant,genic_upstream_transcript_variant
rs529641067	intron_variant,genic_upstream_transcript_variant
rs529671847	intron_variant
rs529685765	genic_downstream_transcript_variant,intron_variant
rs529701640	intron_variant
rs529724400	genic_downstream_transcript_variant,intron_variant
rs529730834	intron_variant,genic_upstream_transcript_variant
rs529840681	intron_variant,genic_upstream_transcript_variant
rs529874651	genic_downstream_transcript_variant,intron_variant
rs529906556	intron_variant
rs529945568	intron_variant
rs529958244	intron_variant
rs529965806	intron_variant,genic_upstream_transcript_variant
rs530088642	genic_downstream_transcript_variant,intron_variant
rs530088726	genic_downstream_transcript_variant,intron_variant
rs530112006	intron_variant,genic_upstream_transcript_variant
rs530117692	intron_variant,genic_upstream_transcript_variant
rs530126471	genic_downstream_transcript_variant,intron_variant
rs530172625	genic_downstream_transcript_variant,intron_variant
rs530175280	intron_variant,genic_upstream_transcript_variant
rs530205043	intron_variant,genic_upstream_transcript_variant
rs530218950	intron_variant,genic_upstream_transcript_variant
rs530239180	intron_variant
rs530299295	intron_variant,genic_upstream_transcript_variant
rs530304125	intron_variant
rs530318015	intron_variant,genic_upstream_transcript_variant
rs530336927	intron_variant

rs530359832	intron_variant,genic_upstream_transcript_variant
rs530401300	intron_variant,genic_upstream_transcript_variant
rs530406243	intron_variant,genic_upstream_transcript_variant
rs530424106	intron_variant
rs530460986	intron_variant
rs530487782	genic_downstream_transcript_variant,intron_variant
rs530501129	intron_variant,genic_upstream_transcript_variant
rs530502269	intron_variant
rs530526963	genic_downstream_transcript_variant,intron_variant
rs530540905	intron_variant
rs530586972	intron_variant,genic_upstream_transcript_variant
rs530589866	intron_variant
rs530601636	genic_upstream_transcript_variant,intron_variant,upstream_transcript_variant
rs530663950	downstream_transcript_variant,500B_downstream_variant
rs530688528	intron_variant
rs530731430	genic_downstream_transcript_variant,intron_variant
rs530753165	intron_variant,genic_upstream_transcript_variant
rs530772513	intron_variant
rs530803113	intron_variant,genic_upstream_transcript_variant
rs530805731	intron_variant,genic_upstream_transcript_variant
rs530808656	genic_downstream_transcript_variant,intron_variant
rs530813702	intron_variant,genic_upstream_transcript_variant
rs530815154	intron_variant,genic_upstream_transcript_variant
rs530815718	intron_variant,genic_upstream_transcript_variant
rs530835127	intron_variant,genic_upstream_transcript_variant
rs530859482	intron_variant
rs530859561	intron_variant
rs530961604	intron_variant,genic_upstream_transcript_variant
rs530978603	genic_downstream_transcript_variant,intron_variant
rs530989584	intron_variant
rs531001022	intron_variant,genic_upstream_transcript_variant
rs531018867	genic_downstream_transcript_variant,intron_variant
rs531024690	intron_variant,genic_upstream_transcript_variant
rs531059164	intron_variant,genic_upstream_transcript_variant
rs531085006	intron_variant,genic_upstream_transcript_variant
rs531104447	intron_variant
rs531127138	intron_variant
rs531166542	intron_variant
rs531167906	intron_variant
rs531239997	intron_variant,genic_upstream_transcript_variant
rs531265142	intron_variant,genic_upstream_transcript_variant
rs531290221	intron_variant
rs531291023	genic_downstream_transcript_variant,intron_variant
rs531337468	intron_variant,genic_upstream_transcript_variant
rs531364636	upstream_transcript_variant,2KB_upstream_variant,intron_variant,genic_upstream_transcript_variant
rs531420311	genic_downstream_transcript_variant,non_coding_transcript_variant,3_prime_UTR_variant
rs531420489	genic_downstream_transcript_variant,intron_variant
rs531426152	intron_variant
rs531426294	intron_variant,genic_upstream_transcript_variant
rs531448212	intron_variant
rs531466945	intron_variant,genic_upstream_transcript_variant
rs531479171	intron_variant,genic_upstream_transcript_variant
rs531529809	intron_variant
rs531555077	upstream_transcript_variant,2KB_upstream_variant,intron_variant,genic_upstream_transcript_variant
rs531565724	intron_variant
rs531569840	intron_variant,genic_upstream_transcript_variant
rs531578294	intron_variant
rs531613112	intron_variant,genic_upstream_transcript_variant
rs531618551	intron_variant,genic_upstream_transcript_variant
rs531653399	intron_variant
rs531680031	intron_variant,genic_upstream_transcript_variant
rs531681160	genic_downstream_transcript_variant,intron_variant
rs531689003	intron_variant,genic_upstream_transcript_variant
rs531714906	intron_variant,genic_upstream_transcript_variant
rs531720730	genic_downstream_transcript_variant,intron_variant
rs531721598	intron_variant
rs531739175	genic_downstream_transcript_variant,intron_variant
rs531765851	intron_variant,genic_upstream_transcript_variant
rs531794586	intron_variant,genic_upstream_transcript_variant
rs531877571	intron_variant,genic_upstream_transcript_variant
rs531891537	intron_variant
rs531898234	intron_variant
rs531944377	upstream_transcript_variant,2KB_upstream_variant,intron_variant,genic_upstream_transcript_variant
rs531957623	intron_variant,genic_upstream_transcript_variant

rs531965383	intron_variant,genic_upstream_transcript_variant
rs531986015	genic_upstream_transcript_variant,2KB_upstream_variant,intron_variant,upstream_transcript_variant
rs531987026	intron_variant
rs531994376	intron_variant
rs532014509	genic_downstream_transcript_variant,intron_variant
rs532016632	intron_variant,genic_upstream_transcript_variant
rs532025406	intron_variant
rs532061590	intron_variant,genic_upstream_transcript_variant
rs532097993	genic_downstream_transcript_variant,intron_variant
rs532124885	non_coding_transcript_variant,coding_sequence_variant,missense_variant
rs532136202	genic_downstream_transcript_variant,intron_variant
rs532182954	intron_variant,genic_upstream_transcript_variant
rs532234891	intron_variant
rs532240873	intron_variant
rs532243343	intron_variant,genic_upstream_transcript_variant
rs532482919	intron_variant,genic_upstream_transcript_variant
rs532566326	genic_downstream_transcript_variant,intron_variant
rs532582971	intron_variant,genic_upstream_transcript_variant
rs532631008	5_prime_UTR_variant,intron_variant,upstream_transcript_variant,genic_upstream_transcript_variant
rs532637960	intron_variant
rs532646177	intron_variant,genic_upstream_transcript_variant
rs532663407	intron_variant
rs532673266	intron_variant
rs532673717	intron_variant
rs532721417	intron_variant
rs532727158	intron_variant,genic_upstream_transcript_variant
rs532757054	intron_variant
rs532798220	intron_variant
rs532802993	intron_variant,genic_upstream_transcript_variant
rs532813357	intron_variant,genic_upstream_transcript_variant
rs532817576	intron_variant
rs532845702	genic_downstream_transcript_variant,intron_variant
rs532852079	intron_variant,genic_upstream_transcript_variant
rs532887056	genic_downstream_transcript_variant,intron_variant
rs532890285	genic_downstream_transcript_variant,intron_variant
rs532900254	intron_variant
rs532909300	intron_variant,genic_upstream_transcript_variant
rs532935054	genic_downstream_transcript_variant,intron_variant
rs532947757	intron_variant,genic_upstream_transcript_variant
rs532952903	intron_variant,genic_upstream_transcript_variant
rs532971193	genic_downstream_transcript_variant,intron_variant
rs532972240	downstream_transcript_variant,500B_downstream_variant
rs532985036	genic_downstream_transcript_variant,intron_variant
rs533061410	intron_variant
rs533068280	genic_downstream_transcript_variant,intron_variant
rs533070003	intron_variant
rs533100188	genic_downstream_transcript_variant,intron_variant
rs533120187	genic_downstream_transcript_variant,intron_variant
rs533130506	intron_variant,genic_upstream_transcript_variant
rs533134903	intron_variant
rs533203508	intron_variant
rs533210775	intron_variant,genic_upstream_transcript_variant
rs533222301	intron_variant
rs533237263	intron_variant
rs533237918	intron_variant,genic_upstream_transcript_variant
rs533239068	genic_downstream_transcript_variant,intron_variant
rs533285461	genic_downstream_transcript_variant,intron_variant
rs533308073	intron_variant,genic_upstream_transcript_variant
rs533319655	intron_variant
rs533365327	genic_downstream_transcript_variant,intron_variant
rs533373216	intron_variant,genic_upstream_transcript_variant
rs533382063	intron_variant,genic_upstream_transcript_variant
rs533419090	genic_upstream_transcript_variant,intron_variant,upstream_transcript_variant
rs533425751	intron_variant
rs533435832	intron_variant,genic_upstream_transcript_variant
rs533479506	intron_variant
rs533503139	intron_variant
rs533525932	intron_variant,genic_upstream_transcript_variant
rs533562645	intron_variant
rs533600061	intron_variant
rs533640524	intron_variant
rs533707752	intron_variant
rs533722293	intron_variant
rs533743403	intron_variant

rs533798699	genic_downstream_transcript_variant,intron_variant
rs533817358	intron_variant,genic_upstream_transcript_variant
rs533823901	intron_variant
rs533855630	intron_variant,genic_upstream_transcript_variant
rs533881098	genic_downstream_transcript_variant,intron_variant
rs533882295	genic_downstream_transcript_variant,intron_variant
rs533927960	intron_variant,genic_upstream_transcript_variant
rs533951282	intron_variant
rs533954179	intron_variant,genic_upstream_transcript_variant
rs533974682	intron_variant
rs533980825	intron_variant
rs534005596	intron_variant
rs534043695	genic_downstream_transcript_variant,intron_variant
rs534085049	intron_variant
rs534094322	intron_variant
rs534096195	intron_variant
rs534138970	intron_variant,genic_upstream_transcript_variant
rs534254489	intron_variant,genic_upstream_transcript_variant
rs534257160	intron_variant,genic_upstream_transcript_variant
rs534285822	genic_downstream_transcript_variant,intron_variant
rs534286739	intron_variant
rs534295642	intron_variant,genic_upstream_transcript_variant
rs534298235	intron_variant,genic_upstream_transcript_variant
rs534306270	intron_variant
rs534308640	intron_variant
rs534314962	genic_downstream_transcript_variant,intron_variant
rs534379730	intron_variant,genic_upstream_transcript_variant
rs534381931	intron_variant
rs534406308	intron_variant,genic_upstream_transcript_variant
rs534417009	intron_variant
rs534424133	intron_variant
rs534463648	intron_variant
rs534465126	intron_variant
rs534473251	genic_downstream_transcript_variant,non_coding_transcript_variant,3_prime_UTR_variant
rs534491220	intron_variant
rs534496555	intron_variant
rs534546602	intron_variant
rs534602528	genic_downstream_transcript_variant,intron_variant
rs534621938	genic_downstream_transcript_variant,intron_variant
rs534629631	intron_variant,genic_upstream_transcript_variant
rs534635383	intron_variant,genic_upstream_transcript_variant
rs534639807	intron_variant
rs534660573	intron_variant
rs534680121	intron_variant
rs534699983	intron_variant
rs534706980	genic_downstream_transcript_variant,intron_variant
rs534722049	genic_downstream_transcript_variant,intron_variant
rs534739750	intron_variant
rs534758557	genic_downstream_transcript_variant,intron_variant
rs534765843	intron_variant,genic_upstream_transcript_variant
rs534809311	intron_variant
rs534819031	upstream_transcript_variant,2KB_upstream_variant,intron_variant,genic_upstream_transcript_variant
rs534827373	intron_variant,genic_upstream_transcript_variant
rs534837239	intron_variant
rs534859339	intron_variant
rs534864019	genic_downstream_transcript_variant,intron_variant
rs534880861	intron_variant
rs534913663	genic_downstream_transcript_variant,intron_variant
rs534915068	intron_variant
rs534924166	intron_variant
rs534975019	intron_variant,genic_upstream_transcript_variant
rs535010118	intron_variant
rs535011703	intron_variant
rs535021820	genic_downstream_transcript_variant,intron_variant
rs535037880	intron_variant,genic_upstream_transcript_variant
rs535127764	genic_downstream_transcript_variant,non_coding_transcript_variant,3_prime_UTR_variant
rs535158148	intron_variant,genic_upstream_transcript_variant
rs535192701	intron_variant
rs535237691	genic_downstream_transcript_variant,intron_variant
rs535252636	genic_upstream_transcript_variant,2KB_upstream_variant,intron_variant,upstream_transcript_variant
rs535271213	genic_downstream_transcript_variant,intron_variant
rs535284043	intron_variant,genic_upstream_transcript_variant
rs535303102	intron_variant
rs535323183	intron_variant,genic_upstream_transcript_variant

rs535331027	intron_variant
rs535340888	genic_upstream_transcript_variant,intron_variant,upstream_transcript_variant
rs535343797	intron_variant,genic_upstream_transcript_variant
rs535349765	intron_variant,genic_upstream_transcript_variant
rs535365592	intron_variant,genic_upstream_transcript_variant
rs535419182	intron_variant
rs535422013	genic_downstream_transcript_variant,intron_variant
rs535426216	genic_downstream_transcript_variant,intron_variant
rs535434508	upstream_transcript_variant,2KB_upstream_variant,intron_variant,genic_upstream_transcript_variant
rs535507062	genic_upstream_transcript_variant,intron_variant,non_coding_transcript_variant,coding_sequence_variant,synonymous_variant
rs535518454	intron_variant
rs535535214	genic_downstream_transcript_variant,intron_variant
rs535544001	intron_variant
rs535552278	intron_variant,genic_upstream_transcript_variant
rs535570426	intron_variant,genic_upstream_transcript_variant
rs535592040	intron_variant,genic_upstream_transcript_variant
rs535608644	intron_variant,genic_upstream_transcript_variant
rs535621683	intron_variant,genic_upstream_transcript_variant
rs535624747	intron_variant
rs535674630	intron_variant,genic_upstream_transcript_variant
rs535680807	genic_downstream_transcript_variant,intron_variant
rs535685294	genic_downstream_transcript_variant,intron_variant
rs535714239	intron_variant
rs535728274	intron_variant
rs535732046	intron_variant,genic_upstream_transcript_variant
rs535733183	intron_variant,genic_upstream_transcript_variant
rs535744947	genic_downstream_transcript_variant,intron_variant
rs535751332	intron_variant
rs535781579	intron_variant,genic_upstream_transcript_variant
rs535917613	intron_variant
rs535919237	intron_variant,genic_upstream_transcript_variant
rs535919648	intron_variant,genic_upstream_transcript_variant
rs535932669	genic_upstream_transcript_variant,2KB_upstream_variant,intron_variant,upstream_transcript_variant
rs535950095	intron_variant,genic_upstream_transcript_variant
rs535979728	intron_variant,genic_upstream_transcript_variant
rs535992810	intron_variant,genic_upstream_transcript_variant
rs536040237	intron_variant,genic_upstream_transcript_variant
rs536063252	intron_variant,genic_upstream_transcript_variant
rs536068548	intron_variant,genic_upstream_transcript_variant
rs536074433	intron_variant,genic_upstream_transcript_variant
rs536104082	intron_variant,genic_upstream_transcript_variant
rs536106097	intron_variant,genic_upstream_transcript_variant
rs536116467	intron_variant,genic_upstream_transcript_variant
rs536164942	intron_variant
rs536168827	intron_variant,genic_upstream_transcript_variant
rs536169746	intron_variant
rs536174107	intron_variant,genic_upstream_transcript_variant
rs536209646	intron_variant
rs536222726	intron_variant
rs536249652	intron_variant,genic_upstream_transcript_variant
rs536296089	intron_variant,genic_upstream_transcript_variant
rs536296897	intron_variant
rs536309776	intron_variant
rs536311740	intron_variant
rs536317723	genic_downstream_transcript_variant,intron_variant
rs536333714	intron_variant
rs536348794	intron_variant
rs536364417	intron_variant
rs536395126	intron_variant,genic_upstream_transcript_variant
rs536399040	intron_variant
rs536471730	genic_downstream_transcript_variant,intron_variant
rs536475974	intron_variant
rs536487199	intron_variant
rs536505958	intron_variant,genic_upstream_transcript_variant
rs536565948	intron_variant
rs536609409	intron_variant,genic_upstream_transcript_variant
rs536646650	intron_variant
rs536656033	genic_downstream_transcript_variant,intron_variant
rs536685764	intron_variant
rs536741272	genic_downstream_transcript_variant,intron_variant
rs536771698	intron_variant
rs536778690	genic_downstream_transcript_variant,intron_variant
rs536788316	intron_variant,upstream_transcript_variant,genic_upstream_transcript_variant,2KB_upstream_variant
rs536805301	intron_variant

rs536901330	intron_variant,genic_upstream_transcript_variant
rs536927268	intron_variant
rs536927736	intron_variant
rs536957542	intron_variant,genic_upstream_transcript_variant
rs536965200	intron_variant
rs536990835	intron_variant,genic_upstream_transcript_variant
rs537040145	intron_variant
rs537057221	intron_variant,genic_upstream_transcript_variant
rs537071011	intron_variant,genic_downstream_transcript_variant
rs537081991	intron_variant,genic_upstream_transcript_variant
rs537085817	intron_variant,genic_upstream_transcript_variant
rs537120016	intron_variant,genic_upstream_transcript_variant
rs537177439	intron_variant,genic_downstream_transcript_variant
rs537226354	intron_variant,genic_upstream_transcript_variant
rs537241228	intron_variant,genic_upstream_transcript_variant
rs537251925	intron_variant,genic_downstream_transcript_variant
rs537293613	intron_variant
rs537301762	intron_variant
rs537310919	intron_variant,genic_upstream_transcript_variant
rs537329547	intron_variant,genic_upstream_transcript_variant
rs537344294	intron_variant,genic_downstream_transcript_variant
rs537381041	intron_variant,genic_downstream_transcript_variant
rs537411690	intron_variant
rs537470565	intron_variant,genic_downstream_transcript_variant
rs537544527	intron_variant
rs537568243	intron_variant,genic_upstream_transcript_variant
rs537568605	intron_variant,genic_upstream_transcript_variant
rs537629525	intron_variant,genic_upstream_transcript_variant
rs537641531	intron_variant,upstream_transcript_variant,genic_upstream_transcript_variant
rs537678258	intron_variant
rs537728288	intron_variant,upstream_transcript_variant,genic_upstream_transcript_variant,2KB_upstream_variant
rs537742050	intron_variant
rs537743356	intron_variant,genic_downstream_transcript_variant
rs537772162	intron_variant
rs537781991	3_prime_UTR_variant,genic_downstream_transcript_variant,non_coding_transcript_variant
rs537793579	5_prime_UTR_variant,intron_variant,genic_upstream_transcript_variant,non_coding_transcript_variant
rs537803270	intron_variant
rs537881614	intron_variant
rs537927743	intron_variant
rs537970103	intron_variant,genic_upstream_transcript_variant
rs537985831	intron_variant,genic_downstream_transcript_variant
rs538013359	intron_variant,genic_downstream_transcript_variant
rs538052613	intron_variant,genic_downstream_transcript_variant
rs538098468	intron_variant,genic_upstream_transcript_variant
rs538126385	intron_variant
rs538165357	intron_variant
rs538168047	intron_variant
rs538181845	intron_variant,genic_upstream_transcript_variant
rs538182014	intron_variant,genic_upstream_transcript_variant
rs538213748	intron_variant
rs538293176	intron_variant
rs538302158	intron_variant,genic_upstream_transcript_variant
rs538322092	intron_variant
rs538345241	intron_variant,genic_upstream_transcript_variant
rs538346198	intron_variant,genic_upstream_transcript_variant
rs538362083	intron_variant,genic_upstream_transcript_variant
rs538388519	intron_variant,genic_downstream_transcript_variant
rs538458601	intron_variant,genic_upstream_transcript_variant
rs538478134	intron_variant
rs538497590	3_prime_UTR_variant,genic_downstream_transcript_variant,non_coding_transcript_variant
rs538507410	intron_variant
rs538514683	intron_variant
rs538522890	intron_variant
rs538538299	intron_variant
rs538580541	intron_variant,genic_upstream_transcript_variant
rs538608249	intron_variant
rs538642016	intron_variant
rs538645065	intron_variant
rs538646878	intron_variant
rs538652757	intron_variant
rs538696118	intron_variant,upstream_transcript_variant,genic_upstream_transcript_variant
rs538713845	intron_variant,genic_downstream_transcript_variant
rs538734809	intron_variant
rs538762716	intron_variant

rs538766860	intron_variant
rs538783374	intron_variant
rs538828142	intron_variant
rs538978672	intron_variant,genic_downstream_transcript_variant,downstream_transcript_variant
rs538979718	intron_variant,genic_upstream_transcript_variant
rs539042187	intron_variant
rs539042514	intron_variant,genic_downstream_transcript_variant
rs539058009	intron_variant,genic_upstream_transcript_variant
rs539058016	intron_variant,genic_upstream_transcript_variant
rs539089132	intron_variant,genic_downstream_transcript_variant
rs539119346	intron_variant,genic_upstream_transcript_variant
rs539139195	intron_variant,genic_downstream_transcript_variant
rs539168733	intron_variant
rs539173271	intron_variant,genic_downstream_transcript_variant
rs539176417	intron_variant,genic_upstream_transcript_variant
rs539190782	intron_variant,genic_downstream_transcript_variant
rs539218108	intron_variant
rs539227343	intron_variant,genic_upstream_transcript_variant
rs539233340	intron_variant,genic_downstream_transcript_variant
rs539257702	upstream_transcript_variant,intron_variant,genic_upstream_transcript_variant,2KB_upstream_variant
rs539259338	intron_variant
rs539268263	intron_variant,genic_upstream_transcript_variant
rs539274678	intron_variant,genic_upstream_transcript_variant
rs539322291	intron_variant,genic_upstream_transcript_variant
rs539336123	intron_variant
rs539349209	intron_variant,genic_downstream_transcript_variant
rs539357778	intron_variant
rs539378584	intron_variant,genic_downstream_transcript_variant
rs539379996	intron_variant,genic_upstream_transcript_variant
rs539382836	intron_variant,genic_downstream_transcript_variant
rs539397126	intron_variant,genic_upstream_transcript_variant
rs539414123	intron_variant
rs539434582	intron_variant,genic_downstream_transcript_variant
rs539444702	intron_variant,genic_downstream_transcript_variant
rs539453317	intron_variant
rs539458298	intron_variant,genic_upstream_transcript_variant
rs539465146	intron_variant,genic_upstream_transcript_variant
rs539498686	intron_variant
rs539522825	intron_variant,upstream_transcript_variant,genic_upstream_transcript_variant,2KB_upstream_variant
rs539592745	intron_variant,genic_downstream_transcript_variant
rs539607054	intron_variant,genic_upstream_transcript_variant
rs539638181	intron_variant
rs539638302	intron_variant,genic_upstream_transcript_variant
rs539661581	intron_variant,genic_downstream_transcript_variant
rs539668979	intron_variant,genic_upstream_transcript_variant
rs539767695	intron_variant
rs539787058	intron_variant,genic_downstream_transcript_variant
rs539814782	intron_variant
rs539825890	intron_variant,genic_downstream_transcript_variant
rs539854084	intron_variant,genic_upstream_transcript_variant
rs539881126	intron_variant
rs539893711	intron_variant,genic_upstream_transcript_variant
rs539911090	intron_variant,genic_upstream_transcript_variant
rs540012349	upstream_transcript_variant,intron_variant,genic_upstream_transcript_variant,2KB_upstream_variant
rs540019272	intron_variant,genic_upstream_transcript_variant
rs540071984	intron_variant
rs540090411	intron_variant
rs540108460	intron_variant,genic_upstream_transcript_variant
rs540140668	intron_variant
rs540156351	intron_variant
rs540156802	intron_variant
rs540186017	intron_variant,genic_upstream_transcript_variant
rs540186612	intron_variant
rs540193128	intron_variant
rs540239295	intron_variant
rs540335032	intron_variant,genic_upstream_transcript_variant
rs540389033	intron_variant,genic_upstream_transcript_variant
rs540445584	upstream_transcript_variant,intron_variant,genic_upstream_transcript_variant,2KB_upstream_variant
rs540452918	intron_variant
rs540486654	intron_variant,genic_downstream_transcript_variant
rs540507995	intron_variant,genic_upstream_transcript_variant
rs540540827	5_prime_UTR_variant,coding_sequence_variant,missense_variant,non_coding_transcript_variant
rs540541578	intron_variant,genic_upstream_transcript_variant
rs540563103	intron_variant,genic_downstream_transcript_variant

rs540567863	intron_variant,genic_upstream_transcript_variant
rs540597455	intron_variant
rs540615069	intron_variant,genic_upstream_transcript_variant
rs540633686	intron_variant
rs540658374	intron_variant,upstream_transcript_variant,genic_upstream_transcript_variant,2KB_upstream_variant
rs540681294	intron_variant
rs540702149	intron_variant
rs540729227	intron_variant,genic_upstream_transcript_variant
rs540731785	intron_variant,genic_upstream_transcript_variant
rs540738780	intron_variant
rs540802344	intron_variant,genic_downstream_transcript_variant
rs540804012	intron_variant
rs540821657	intron_variant
rs540827687	upstream_transcript_variant,intron_variant,genic_upstream_transcript_variant,2KB_upstream_variant
rs540836980	intron_variant,genic_upstream_transcript_variant
rs540920940	intron_variant,genic_upstream_transcript_variant
rs540932687	intron_variant,genic_downstream_transcript_variant
rs540940807	intron_variant
rs540955782	intron_variant,genic_upstream_transcript_variant
rs540967590	intron_variant,genic_downstream_transcript_variant
rs541087395	intron_variant,genic_downstream_transcript_variant
rs541102523	intron_variant,genic_upstream_transcript_variant
rs541103845	intron_variant
rs541125654	intron_variant,genic_upstream_transcript_variant
rs541141654	intron_variant,genic_upstream_transcript_variant
rs541148448	intron_variant,genic_upstream_transcript_variant
rs541156628	intron_variant,genic_downstream_transcript_variant
rs541158141	intron_variant
rs541163928	intron_variant,genic_downstream_transcript_variant
rs541167749	intron_variant,genic_downstream_transcript_variant
rs541239627	intron_variant,genic_upstream_transcript_variant
rs541297306	intron_variant,genic_upstream_transcript_variant
rs541305999	intron_variant,genic_upstream_transcript_variant
rs541319710	intron_variant,genic_upstream_transcript_variant
rs541322928	3_prime_UTR_variant,genic_downstream_transcript_variant,downstream_transcript_variant,non_coding_transcript_variant
rs541325277	intron_variant
rs541332743	intron_variant,genic_upstream_transcript_variant
rs541351766	intron_variant,genic_upstream_transcript_variant
rs541428716	intron_variant,upstream_transcript_variant,genic_upstream_transcript_variant
rs541433797	intron_variant,genic_upstream_transcript_variant
rs541441794	3_prime_UTR_variant,genic_downstream_transcript_variant,non_coding_transcript_variant
rs541445179	upstream_transcript_variant,intron_variant,genic_upstream_transcript_variant,2KB_upstream_variant
rs541464724	intron_variant,genic_upstream_transcript_variant
rs541474191	intron_variant,genic_downstream_transcript_variant
rs541486130	intron_variant,genic_downstream_transcript_variant
rs541493133	intron_variant,genic_upstream_transcript_variant
rs541497034	intron_variant,genic_upstream_transcript_variant
rs541529889	intron_variant
rs541539083	intron_variant,genic_downstream_transcript_variant
rs541544135	intron_variant,genic_upstream_transcript_variant
rs541553735	intron_variant,genic_upstream_transcript_variant
rs541553801	intron_variant,genic_downstream_transcript_variant
rs541564187	intron_variant,genic_downstream_transcript_variant
rs541623514	intron_variant
rs541657543	intron_variant,genic_upstream_transcript_variant
rs541684690	intron_variant,genic_downstream_transcript_variant
rs541693563	intron_variant,genic_upstream_transcript_variant
rs541704711	intron_variant,genic_upstream_transcript_variant
rs541734329	intron_variant
rs541746515	intron_variant,genic_downstream_transcript_variant
rs541766261	intron_variant,genic_upstream_transcript_variant
rs541832747	intron_variant,upstream_transcript_variant,genic_upstream_transcript_variant,2KB_upstream_variant
rs541876545	intron_variant
rs541963413	intron_variant
rs541973573	intron_variant
rs542005099	intron_variant,genic_upstream_transcript_variant
rs542010513	intron_variant,genic_upstream_transcript_variant
rs542010602	intron_variant
rs542011488	intron_variant
rs542015591	intron_variant,genic_downstream_transcript_variant
rs542048185	intron_variant
rs542096684	intron_variant
rs542103167	intron_variant,non_coding_transcript_variant,genic_upstream_transcript_variant,coding_sequence_variant,missense_variant
rs542109808	intron_variant,genic_upstream_transcript_variant

rs542113335	intron_variant
rs542121884	intron_variant,genic_upstream_transcript_variant
rs542133506	intron_variant,genic_downstream_transcript_variant
rs542165170	intron_variant,upstream_transcript_variant,genic_upstream_transcript_variant,2KB_upstream_variant
rs542177937	intron_variant
rs542187443	intron_variant,genic_upstream_transcript_variant
rs542210593	intron_variant
rs542251070	intron_variant
rs542267057	intron_variant,non_coding_transcript_variant,genic_upstream_transcript_variant,coding_sequence_variant,missense_variant
rs542271812	3_prime_UTR_variant,genic_downstream_transcript_variant,non_coding_transcript_variant
rs542282757	intron_variant
rs542296514	intron_variant
rs542402158	5_prime_UTR_variant,intron_variant
rs542440838	intron_variant
rs542456294	3_prime_UTR_variant,genic_downstream_transcript_variant,non_coding_transcript_variant
rs542490724	intron_variant,genic_upstream_transcript_variant
rs542560043	intron_variant
rs542579914	upstream_transcript_variant,intron_variant,genic_upstream_transcript_variant,2KB_upstream_variant
rs542620288	intron_variant
rs542713188	intron_variant
rs542718741	intron_variant
rs542719315	intron_variant
rs542724954	intron_variant
rs542726958	intron_variant,genic_downstream_transcript_variant
rs542750781	intron_variant,genic_upstream_transcript_variant
rs542755535	intron_variant
rs542777524	intron_variant,genic_upstream_transcript_variant
rs542790265	intron_variant,genic_downstream_transcript_variant
rs542795808	intron_variant,genic_upstream_transcript_variant
rs542840134	intron_variant,genic_upstream_transcript_variant
rs542851962	intron_variant
rs542857662	intron_variant,genic_downstream_transcript_variant
rs542883005	intron_variant,genic_downstream_transcript_variant
rs542889141	intron_variant
rs542890757	intron_variant
rs542904678	intron_variant,genic_downstream_transcript_variant
rs542934651	intron_variant
rs542982913	intron_variant
rs542987892	intron_variant,genic_upstream_transcript_variant
rs543010238	intron_variant
rs543017759	intron_variant
rs543026979	intron_variant,genic_upstream_transcript_variant
rs543088074	intron_variant,genic_upstream_transcript_variant
rs543137641	intron_variant
rs543179640	intron_variant,genic_downstream_transcript_variant
rs543182899	intron_variant,genic_downstream_transcript_variant
rs543229800	intron_variant,genic_upstream_transcript_variant
rs543253156	intron_variant,genic_upstream_transcript_variant
rs543377449	intron_variant,genic_upstream_transcript_variant
rs543409064	intron_variant,genic_upstream_transcript_variant
rs543417900	intron_variant,genic_upstream_transcript_variant
rs543471172	intron_variant,genic_downstream_transcript_variant
rs543505292	intron_variant,genic_upstream_transcript_variant
rs543506413	intron_variant
rs543519144	intron_variant,genic_upstream_transcript_variant
rs543532836	intron_variant,genic_downstream_transcript_variant
rs543553819	intron_variant,genic_downstream_transcript_variant
rs543580693	intron_variant,genic_upstream_transcript_variant
rs543581054	intron_variant,genic_upstream_transcript_variant
rs543595746	intron_variant,genic_upstream_transcript_variant
rs543607954	intron_variant,genic_upstream_transcript_variant
rs543629579	intron_variant,genic_upstream_transcript_variant
rs543632409	intron_variant
rs543650055	5_prime_UTR_variant,intron_variant,genic_upstream_transcript_variant,non_coding_transcript_variant
rs543673515	3_prime_UTR_variant,genic_downstream_transcript_variant,non_coding_transcript_variant
rs543692626	intron_variant,genic_upstream_transcript_variant
rs543719365	intron_variant,genic_upstream_transcript_variant
rs543752526	intron_variant,genic_upstream_transcript_variant
rs543756448	intron_variant,genic_upstream_transcript_variant
rs543759686	intron_variant,genic_upstream_transcript_variant
rs543766277	intron_variant,genic_downstream_transcript_variant
rs543779397	downstream_transcript_variant,500B_downstream_variant
rs543788066	intron_variant,genic_downstream_transcript_variant
rs543804679	intron_variant,genic_upstream_transcript_variant

rs543805547	intron_variant,genic_upstream_transcript_variant
rs543819035	intron_variant,genic_upstream_transcript_variant
rs543833603	intron_variant
rs543860463	intron_variant,genic_downstream_transcript_variant
rs543863288	intron_variant
rs543864205	intron_variant,genic_downstream_transcript_variant
rs543865483	intron_variant
rs543866349	intron_variant,genic_upstream_transcript_variant
rs543884890	intron_variant,genic_downstream_transcript_variant
rs543915598	intron_variant,genic_downstream_transcript_variant
rs543948036	intron_variant,genic_downstream_transcript_variant
rs543948763	intron_variant
rs543980820	intron_variant,genic_upstream_transcript_variant
rs543995572	intron_variant,genic_upstream_transcript_variant
rs543997725	intron_variant
rs544075216	intron_variant,genic_upstream_transcript_variant
rs544084654	intron_variant,genic_downstream_transcript_variant
rs544092676	intron_variant,genic_upstream_transcript_variant
rs544113570	intron_variant
rs544137936	intron_variant,genic_upstream_transcript_variant
rs544219689	upstream_transcript_variant,intron_variant,genic_upstream_transcript_variant,2KB_upstream_variant
rs544233796	intron_variant
rs544237864	intron_variant
rs544251725	intron_variant,genic_upstream_transcript_variant
rs544259242	intron_variant,genic_upstream_transcript_variant
rs544272319	intron_variant
rs544290315	intron_variant
rs544310866	intron_variant
rs544313635	intron_variant,genic_upstream_transcript_variant
rs544360809	intron_variant
rs544389872	intron_variant,genic_downstream_transcript_variant
rs544431495	intron_variant,genic_upstream_transcript_variant
rs544488642	intron_variant,genic_downstream_transcript_variant
rs544519485	intron_variant
rs544570215	intron_variant,genic_upstream_transcript_variant
rs544577796	intron_variant
rs544583021	intron_variant,genic_upstream_transcript_variant
rs544657682	intron_variant,genic_upstream_transcript_variant
rs544686662	intron_variant
rs544695908	intron_variant
rs544720855	intron_variant,genic_upstream_transcript_variant
rs544773980	intron_variant
rs544791921	intron_variant,genic_downstream_transcript_variant
rs544840423	intron_variant,genic_downstream_transcript_variant
rs544880662	intron_variant
rs544915846	intron_variant,genic_upstream_transcript_variant,non_coding_transcript_variant
rs544923324	intron_variant,genic_downstream_transcript_variant
rs544959126	intron_variant
rs544969603	intron_variant
rs544969987	intron_variant,genic_downstream_transcript_variant
rs544970146	3_prime_UTR_variant,genic_downstream_transcript_variant,non_coding_transcript_variant
rs545006646	intron_variant
rs545026281	intron_variant,genic_upstream_transcript_variant
rs545028275	intron_variant
rs545051967	3_prime_UTR_variant,genic_downstream_transcript_variant,non_coding_transcript_variant
rs545075758	intron_variant
rs545140983	intron_variant,genic_downstream_transcript_variant
rs545157033	intron_variant
rs545226359	intron_variant,genic_downstream_transcript_variant
rs545265895	intron_variant,genic_upstream_transcript_variant
rs545274704	intron_variant
rs545280060	intron_variant,genic_downstream_transcript_variant
rs545282287	intron_variant
rs545285428	intron_variant
rs545365035	intron_variant,genic_upstream_transcript_variant
rs545381820	intron_variant,genic_downstream_transcript_variant
rs545415751	intron_variant,genic_downstream_transcript_variant
rs545419616	intron_variant,upstream_transcript_variant,genic_upstream_transcript_variant
rs545444524	intron_variant,genic_upstream_transcript_variant
rs545450460	intron_variant,genic_downstream_transcript_variant
rs545458374	intron_variant,genic_upstream_transcript_variant
rs545477729	intron_variant,genic_downstream_transcript_variant
rs545496806	intron_variant,genic_upstream_transcript_variant
rs545512167	intron_variant

rs545512490	intron_variant
rs545556735	intron_variant
rs545591604	intron_variant
rs545611355	intron_variant,genic_upstream_transcript_variant
rs545620910	intron_variant,genic_downstream_transcript_variant
rs545671919	intron_variant,genic_upstream_transcript_variant
rs545686303	intron_variant
rs545688660	intron_variant
rs545801291	intron_variant,genic_upstream_transcript_variant
rs545809899	intron_variant,genic_upstream_transcript_variant
rs545835271	intron_variant,genic_downstream_transcript_variant
rs545860655	3_prime_UTR_variant,genic_downstream_transcript_variant,non_coding_transcript_variant
rs545901249	intron_variant,genic_upstream_transcript_variant
rs545904481	intron_variant
rs545938651	3_prime_UTR_variant,genic_downstream_transcript_variant,non_coding_transcript_variant
rs545965765	intron_variant,genic_upstream_transcript_variant
rs546073297	intron_variant
rs546076294	genic_upstream_transcript_variant,intron_variant
rs546083703	intron_variant
rs546113994	genic_upstream_transcript_variant,intron_variant
rs546135716	genic_upstream_transcript_variant,intron_variant
rs546137148	genic_upstream_transcript_variant,intron_variant
rs546145995	genic_upstream_transcript_variant,upstream_transcript_variant,intron_variant
rs546164980	genic_upstream_transcript_variant,intron_variant
rs546177996	intron_variant
rs546199787	intron_variant
rs546223463	genic_downstream_transcript_variant,intron_variant
rs546264111	intron_variant
rs546306912	intron_variant
rs546322761	genic_upstream_transcript_variant,intron_variant
rs546336511	genic_downstream_transcript_variant,intron_variant
rs546362587	intron_variant
rs546373644	genic_upstream_transcript_variant,intron_variant
rs546382970	genic_downstream_transcript_variant,intron_variant
rs546433040	genic_upstream_transcript_variant,intron_variant
rs546444252	3_prime_UTR_variant,genic_downstream_transcript_variant,non_coding_transcript_variant
rs546445876	genic_upstream_transcript_variant,intron_variant
rs546472619	genic_upstream_transcript_variant,intron_variant
rs546476613	genic_downstream_transcript_variant,intron_variant
rs546492365	genic_upstream_transcript_variant,intron_variant
rs546494643	genic_upstream_transcript_variant,intron_variant
rs546510768	intron_variant
rs546514657	genic_upstream_transcript_variant,intron_variant
rs546520309	genic_upstream_transcript_variant,intron_variant
rs546531927	intron_variant
rs546534435	intron_variant
rs546559782	missense_variant,coding_sequence_variant,non_coding_transcript_variant
rs546594890	intron_variant
rs546600765	intron_variant
rs546631978	intron_variant
rs546657400	intron_variant
rs546681833	genic_downstream_transcript_variant,intron_variant
rs546688682	intron_variant
rs546733107	genic_upstream_transcript_variant,intron_variant
rs546747979	genic_upstream_transcript_variant,intron_variant
rs546754403	genic_upstream_transcript_variant,intron_variant
rs546772153	genic_downstream_transcript_variant,intron_variant
rs546851434	genic_upstream_transcript_variant,intron_variant
rs546857975	genic_downstream_transcript_variant,intron_variant
rs546859360	genic_downstream_transcript_variant,intron_variant
rs546889837	genic_upstream_transcript_variant,intron_variant
rs546922710	intron_variant
rs546945259	genic_downstream_transcript_variant,intron_variant
rs546959941	intron_variant
rs546962531	intron_variant
rs546965089	genic_downstream_transcript_variant,intron_variant
rs546997065	intron_variant
rs547006241	genic_upstream_transcript_variant,intron_variant
rs547008064	genic_upstream_transcript_variant,intron_variant
rs547050112	genic_downstream_transcript_variant,intron_variant
rs547071825	3_prime_UTR_variant,genic_downstream_transcript_variant,non_coding_transcript_variant
rs547103028	intron_variant
rs547143978	genic_upstream_transcript_variant,intron_variant
rs547151698	genic_downstream_transcript_variant,intron_variant

rs547167668	genic_downstream_transcript_variant,intron_variant
rs547181389	genic_upstream_transcript_variant,intron_variant
rs547182924	genic_upstream_transcript_variant,2KB_upstream_variant,upstream_transcript_variant,intron_variant
rs547209874	intron_variant
rs547218935	intron_variant
rs547245502	genic_upstream_transcript_variant,intron_variant
rs547248517	intron_variant
rs547255317	genic_downstream_transcript_variant,intron_variant
rs547255808	intron_variant
rs547284035	genic_upstream_transcript_variant,intron_variant
rs547297421	intron_variant
rs547320299	intron_variant
rs547348808	genic_upstream_transcript_variant,intron_variant
rs547400075	3_prime_UTR_variant,genic_downstream_transcript_variant,non_coding_transcript_variant
rs547412543	upstream_transcript_variant,genic_upstream_transcript_variant,2KB_upstream_variant,intron_variant
rs547428537	genic_upstream_transcript_variant,intron_variant
rs547457270	genic_upstream_transcript_variant,intron_variant
rs547531516	genic_downstream_transcript_variant,intron_variant
rs547532029	intron_variant
rs547571299	genic_upstream_transcript_variant,intron_variant
rs547580627	genic_upstream_transcript_variant,intron_variant
rs547615728	intron_variant
rs547627648	genic_upstream_transcript_variant,intron_variant
rs547644833	intron_variant
rs547668588	genic_downstream_transcript_variant,intron_variant
rs547746073	upstream_transcript_variant,genic_upstream_transcript_variant,2KB_upstream_variant,intron_variant
rs547777397	missense_variant,coding_sequence_variant,non_coding_transcript_variant
rs547794061	genic_upstream_transcript_variant,intron_variant
rs547795257	genic_upstream_transcript_variant,intron_variant
rs547856213	genic_upstream_transcript_variant,intron_variant
rs547862987	genic_upstream_transcript_variant,2KB_upstream_variant,upstream_transcript_variant,intron_variant
rs547903136	intron_variant
rs547911723	intron_variant
rs547919215	genic_upstream_transcript_variant,intron_variant
rs547920218	genic_downstream_transcript_variant,intron_variant
rs547928418	genic_upstream_transcript_variant,2KB_upstream_variant,upstream_transcript_variant,intron_variant
rs547930175	genic_upstream_transcript_variant,intron_variant
rs547975148	genic_upstream_transcript_variant,intron_variant
rs547981313	intron_variant
rs548018177	intron_variant
rs548086562	intron_variant
rs548105693	intron_variant
rs548108908	intron_variant
rs548134093	intron_variant
rs548145290	intron_variant
rs548154106	genic_upstream_transcript_variant,intron_variant
rs548197437	genic_upstream_transcript_variant,intron_variant
rs548199212	genic_upstream_transcript_variant,intron_variant
rs548214476	genic_upstream_transcript_variant,intron_variant
rs548224167	intron_variant
rs548251385	upstream_transcript_variant,genic_upstream_transcript_variant,2KB_upstream_variant,intron_variant
rs548311809	genic_upstream_transcript_variant,intron_variant
rs548339164	genic_upstream_transcript_variant,intron_variant
rs548358252	genic_downstream_transcript_variant,intron_variant
rs548390884	genic_upstream_transcript_variant,intron_variant
rs548403312	genic_upstream_transcript_variant,intron_variant
rs548489508	intron_variant
rs548502026	intron_variant
rs548503839	genic_upstream_transcript_variant,intron_variant
rs548525517	intron_variant
rs548580853	genic_downstream_transcript_variant,intron_variant
rs548683119	intron_variant
rs548700555	genic_upstream_transcript_variant,intron_variant
rs548704094	genic_upstream_transcript_variant,intron_variant
rs548773799	genic_upstream_transcript_variant,intron_variant
rs548785730	intron_variant
rs548789876	genic_upstream_transcript_variant,intron_variant
rs548794283	genic_upstream_transcript_variant,upstream_transcript_variant,intron_variant
rs548824068	intron_variant
rs548840877	genic_upstream_transcript_variant,intron_variant
rs548860509	genic_downstream_transcript_variant,intron_variant
rs548880210	intron_variant
rs548897187	genic_downstream_transcript_variant,intron_variant
rs548939428	genic_downstream_transcript_variant,intron_variant

rs548953381	genic_upstream_transcript_variant,intron_variant
rs548958127	genic_downstream_transcript_variant,intron_variant
rs548974533	genic_downstream_transcript_variant,intron_variant
rs549043010	intron_variant
rs549056527	intron_variant
rs549056620	intron_variant
rs549062554	genic_downstream_transcript_variant,intron_variant
rs549093290	intron_variant
rs549099286	genic_downstream_transcript_variant,intron_variant
rs549112422	intron_variant
rs549126385	genic_downstream_transcript_variant,intron_variant
rs549140685	genic_downstream_transcript_variant,intron_variant
rs549145824	genic_upstream_transcript_variant,intron_variant
rs549146218	intron_variant
rs549169361	genic_upstream_transcript_variant,intron_variant
rs549174351	intron_variant
rs549196468	genic_upstream_transcript_variant,intron_variant
rs549230483	genic_downstream_transcript_variant,intron_variant
rs549233899	genic_upstream_transcript_variant,intron_variant
rs549266450	genic_upstream_transcript_variant,intron_variant
rs549316062	genic_downstream_transcript_variant,intron_variant
rs549320063	genic_downstream_transcript_variant,intron_variant
rs549325630	intron_variant
rs549344138	genic_upstream_transcript_variant,intron_variant
rs549371468	intron_variant
rs549376289	genic_downstream_transcript_variant,intron_variant
rs549398651	intron_variant
rs549454527	intron_variant
rs549466632	genic_upstream_transcript_variant,intron_variant
rs549471394	genic_upstream_transcript_variant,intron_variant
rs549491658	intron_variant
rs549496549	genic_upstream_transcript_variant,intron_variant
rs549552612	genic_downstream_transcript_variant,intron_variant
rs549552643	genic_downstream_transcript_variant,intron_variant
rs549554011	intron_variant
rs549622055	genic_upstream_transcript_variant,intron_variant
rs549631830	intron_variant
rs549657766	genic_downstream_transcript_variant,intron_variant
rs549697617	intron_variant
rs549718057	intron_variant
rs549733487	genic_upstream_transcript_variant,intron_variant
rs549750541	intron_variant
rs549779706	genic_upstream_transcript_variant,intron_variant
rs549785530	genic_upstream_transcript_variant,intron_variant
rs549829223	genic_upstream_transcript_variant,intron_variant
rs549863508	genic_upstream_transcript_variant,intron_variant
rs549874584	intron_variant
rs549911000	genic_downstream_transcript_variant,intron_variant
rs549919945	intron_variant
rs549938487	upstream_transcript_variant,genic_upstream_transcript_variant,2KB_upstream_variant,intron_variant
rs549953371	intron_variant
rs549958646	genic_downstream_transcript_variant,intron_variant
rs549959989	genic_upstream_transcript_variant,intron_variant
rs549988101	intron_variant
rs549989941	genic_upstream_transcript_variant,intron_variant
rs550030690	genic_upstream_transcript_variant,intron_variant
rs550038852	intron_variant
rs550082986	upstream_transcript_variant,genic_upstream_transcript_variant,2KB_upstream_variant,intron_variant
rs550137929	genic_upstream_transcript_variant,intron_variant
rs550162551	genic_downstream_transcript_variant,intron_variant
rs550185871	genic_downstream_transcript_variant,intron_variant
rs550225195	genic_upstream_transcript_variant,intron_variant
rs550252261	intron_variant
rs550278856	genic_upstream_transcript_variant,intron_variant
rs550286635	genic_upstream_transcript_variant,intron_variant
rs550288023	genic_downstream_transcript_variant,intron_variant
rs550290947	genic_upstream_transcript_variant,intron_variant
rs550291205	genic_upstream_transcript_variant,intron_variant
rs550303394	genic_upstream_transcript_variant,intron_variant
rs550313513	intron_variant
rs550398988	genic_upstream_transcript_variant,intron_variant
rs550416576	genic_upstream_transcript_variant,upstream_transcript_variant,intron_variant
rs550462929	genic_upstream_transcript_variant,intron_variant
rs550476990	intron_variant

rs550513490	genic_upstream_transcript_variant,intron_variant
rs550514111	intron_variant
rs550519644	genic_upstream_transcript_variant,intron_variant
rs550599626	intron_variant
rs550630963	genic_downstream_transcript_variant,intron_variant
rs550645306	intron_variant
rs550682942	intron_variant
rs550689538	upstream_transcript_variant,genic_upstream_transcript_variant,2KB_upstream_variant,intron_variant
rs550696561	genic_downstream_transcript_variant,intron_variant
rs550703269	genic_upstream_transcript_variant,intron_variant
rs550718913	intron_variant
rs550729548	missense_variant,coding_sequence_variant,intron_variant
rs550734562	genic_upstream_transcript_variant,intron_variant
rs550738287	intron_variant
rs550757726	intron_variant
rs550777044	intron_variant
rs550777640	intron_variant
rs550791264	intron_variant
rs550857497	genic_upstream_transcript_variant,intron_variant
rs550884742	intron_variant
rs550909916	genic_downstream_transcript_variant,intron_variant
rs550934870	intron_variant
rs550972014	intron_variant
rs551007657	genic_upstream_transcript_variant,intron_variant
rs551059658	intron_variant
rs551063692	genic_downstream_transcript_variant,intron_variant
rs551105133	genic_downstream_transcript_variant,intron_variant
rs551126161	intron_variant
rs551180139	genic_upstream_transcript_variant,intron_variant
rs551181619	genic_upstream_transcript_variant,intron_variant
rs551206309	genic_upstream_transcript_variant,intron_variant
rs551261322	5_prime_UTR_variant,genic_upstream_transcript_variant,upstream_transcript_variant,intron_variant
rs551264352	genic_upstream_transcript_variant,intron_variant
rs551265020	genic_upstream_transcript_variant,intron_variant
rs551298214	genic_downstream_transcript_variant,intron_variant
rs551325522	intron_variant
rs551379728	genic_downstream_transcript_variant,intron_variant
rs551383880	genic_upstream_transcript_variant,intron_variant
rs551411613	genic_upstream_transcript_variant,intron_variant
rs551451678	intron_variant
rs551452197	intron_variant
rs551471351	genic_downstream_transcript_variant,intron_variant
rs551488643	intron_variant
rs551490256	intron_variant
rs551506116	genic_upstream_transcript_variant,intron_variant
rs551508074	3_prime_UTR_variant,genic_downstream_transcript_variant,non_coding_transcript_variant
rs551511079	genic_upstream_transcript_variant,intron_variant
rs551513741	genic_upstream_transcript_variant,intron_variant
rs551541415	genic_downstream_transcript_variant,intron_variant
rs551550460	genic_downstream_transcript_variant,intron_variant
rs551566673	genic_upstream_transcript_variant,intron_variant
rs551570313	genic_downstream_transcript_variant,intron_variant
rs551600216	intron_variant
rs551605356	genic_upstream_transcript_variant,intron_variant
rs551628216	genic_downstream_transcript_variant,intron_variant
rs551631168	genic_downstream_transcript_variant,intron_variant
rs551636614	genic_downstream_transcript_variant,intron_variant
rs551657817	intron_variant
rs551664843	genic_upstream_transcript_variant,intron_variant
rs551681261	intron_variant
rs551699477	genic_upstream_transcript_variant,intron_variant
rs551777558	genic_upstream_transcript_variant,intron_variant
rs551801700	genic_upstream_transcript_variant,intron_variant
rs551839201	genic_upstream_transcript_variant,intron_variant
rs551885186	genic_upstream_transcript_variant,intron_variant
rs551898360	genic_downstream_transcript_variant,intron_variant
rs551919023	intron_variant
rs551920140	genic_downstream_transcript_variant,intron_variant
rs551956067	intron_variant
rs551976686	intron_variant
rs552028655	intron_variant
rs552043744	intron_variant
rs552054530	intron_variant
rs552059644	intron_variant

rs552067474	genic_upstream_transcript_variant,intron_variant
rs552181100	genic_upstream_transcript_variant,intron_variant
rs552183471	intron_variant
rs552206217	genic_upstream_transcript_variant,intron_variant
rs552207024	intron_variant
rs552218749	genic_upstream_transcript_variant,intron_variant
rs552242554	genic_downstream_transcript_variant,intron_variant
rs552248968	genic_upstream_transcript_variant,intron_variant
rs552291696	genic_upstream_transcript_variant,intron_variant
rs552321692	genic_upstream_transcript_variant,intron_variant
rs552323933	intron_variant
rs552336950	intron_variant
rs552346808	genic_downstream_transcript_variant,intron_variant
rs552374823	intron_variant
rs552435121	genic_downstream_transcript_variant,intron_variant
rs552454472	genic_upstream_transcript_variant,intron_variant
rs552463156	genic_upstream_transcript_variant,intron_variant
rs552517211	intron_variant
rs552517794	genic_upstream_transcript_variant,intron_variant
rs552532940	intron_variant
rs552595360	genic_upstream_transcript_variant,intron_variant
rs552604784	genic_upstream_transcript_variant,intron_variant
rs552621661	genic_downstream_transcript_variant,intron_variant
rs552625914	intron_variant
rs552658735	intron_variant
rs552671634	genic_upstream_transcript_variant,intron_variant
rs552678010	intron_variant
rs552772997	intron_variant
rs552775944	genic_upstream_transcript_variant,intron_variant
rs552831805	genic_upstream_transcript_variant,intron_variant
rs552871115	intron_variant
rs552888738	genic_downstream_transcript_variant,intron_variant
rs552904563	genic_downstream_transcript_variant,intron_variant
rs552929190	genic_downstream_transcript_variant,intron_variant
rs552953455	missense_variant,coding_sequence_variant,intron_variant
rs552980762	genic_upstream_transcript_variant,intron_variant
rs552985441	intron_variant
rs553027074	intron_variant
rs553049743	intron_variant
rs553074721	genic_downstream_transcript_variant,intron_variant
rs553075022	intron_variant
rs553086613	intron_variant
rs553092655	genic_downstream_transcript_variant,intron_variant
rs553098188	genic_upstream_transcript_variant,intron_variant
rs553166868	genic_upstream_transcript_variant,intron_variant
rs553185323	genic_upstream_transcript_variant,intron_variant
rs553190334	intron_variant
rs553197725	genic_upstream_transcript_variant,intron_variant
rs553207008	genic_downstream_transcript_variant,downstream_transcript_variant,intron_variant
rs553210156	genic_upstream_transcript_variant,2KB_upstream_variant,upstream_transcript_variant,intron_variant
rs553278249	intron_variant
rs553304687	genic_upstream_transcript_variant,upstream_transcript_variant,intron_variant
rs553322810	genic_upstream_transcript_variant,intron_variant
rs553344089	genic_downstream_transcript_variant,intron_variant
rs553355112	genic_upstream_transcript_variant,2KB_upstream_variant,upstream_transcript_variant,intron_variant
rs553375578	intron_variant
rs553402575	genic_downstream_transcript_variant,intron_variant
rs553416664	genic_upstream_transcript_variant,intron_variant
rs553439408	genic_downstream_transcript_variant,intron_variant
rs553445059	genic_downstream_transcript_variant,intron_variant
rs553456683	genic_upstream_transcript_variant,intron_variant
rs553487134	intron_variant
rs553496094	intron_variant
rs553518087	genic_upstream_transcript_variant,intron_variant
rs553548043	intron_variant
rs553581931	intron_variant
rs553629628	intron_variant
rs553631537	genic_upstream_transcript_variant,intron_variant
rs553692920	genic_upstream_transcript_variant,intron_variant
rs553719252	genic_downstream_transcript_variant,intron_variant
rs553729006	genic_upstream_transcript_variant,intron_variant
rs553745206	3_prime_UTR_variant,genic_downstream_transcript_variant,non_coding_transcript_variant
rs553774616	intron_variant
rs553816126	genic_upstream_transcript_variant,intron_variant

rs553864000	intron_variant
rs553925927	genic_upstream_transcript_variant,intron_variant
rs553926099	genic_upstream_transcript_variant,intron_variant
rs553926132	genic_upstream_transcript_variant,intron_variant
rs553976691	genic_upstream_transcript_variant,intron_variant
rs554061265	downstream_transcript_variant,500B_downstream_variant
rs554072150	genic_upstream_transcript_variant,intron_variant
rs554081598	intron_variant
rs554091190	genic_upstream_transcript_variant,intron_variant
rs554167410	intron_variant
rs554170963	intron_variant
rs554196587	intron_variant
rs554220373	intron_variant
rs554286914	intron_variant
rs554313324	intron_variant
rs554314432	genic_upstream_transcript_variant,intron_variant
rs554374555	genic_downstream_transcript_variant,intron_variant
rs554438960	intron_variant
rs554475336	genic_downstream_transcript_variant,intron_variant
rs554477707	intron_variant
rs554499802	intron_variant
rs554542833	intron_variant
rs554542860	intron_variant
rs554585191	intron_variant
rs554586684	intron_variant
rs554628410	intron_variant
rs554643413	intron_variant
rs554666067	intron_variant
rs554690944	upstream_transcript_variant,genic_upstream_transcript_variant,2KB_upstream_variant,intron_variant
rs554699424	intron_variant
rs554748853	genic_upstream_transcript_variant,intron_variant
rs554810103	genic_upstream_transcript_variant,intron_variant
rs554833287	genic_downstream_transcript_variant,intron_variant
rs554844771	intron_variant
rs554900106	intron_variant
rs554917256	genic_upstream_transcript_variant,intron_variant
rs554965144	intron_variant
rs554970208	genic_upstream_transcript_variant,intron_variant
rs554984524	intron_variant
rs555010557	intron_variant
rs555011707	5_prime_UTR_variant,genic_upstream_transcript_variant,intron_variant,non_coding_transcript_variant
rs555019025	genic_downstream_transcript_variant,intron_variant
rs555026756	genic_upstream_transcript_variant,intron_variant
rs555066820	genic_downstream_transcript_variant,intron_variant
rs555073315	genic_upstream_transcript_variant,2KB_upstream_variant,upstream_transcript_variant,intron_variant
rs555103956	genic_downstream_transcript_variant,intron_variant
rs555110965	genic_upstream_transcript_variant,intron_variant
rs555165461	intron_variant
rs555165777	intron_variant
rs555170362	genic_upstream_transcript_variant,intron_variant
rs555185364	genic_upstream_transcript_variant,intron_variant
rs555288487	intron_variant
rs555428093	genic_upstream_transcript_variant,intron_variant
rs555443453	intron_variant
rs555466300	genic_downstream_transcript_variant,intron_variant
rs555469152	intron_variant
rs555487099	genic_upstream_transcript_variant,intron_variant
rs555582967	genic_upstream_transcript_variant,intron_variant
rs555583743	genic_upstream_transcript_variant,intron_variant
rs555612942	genic_downstream_transcript_variant,intron_variant
rs555640115	intron_variant
rs555644251	genic_upstream_transcript_variant,intron_variant
rs555649773	genic_downstream_transcript_variant,intron_variant
rs555677632	intron_variant
rs555726714	genic_upstream_transcript_variant,intron_variant
rs555729609	genic_downstream_transcript_variant,intron_variant
rs555736917	intron_variant
rs555753757	genic_upstream_transcript_variant,intron_variant
rs555765274	intron_variant
rs555824065	genic_upstream_transcript_variant,intron_variant
rs555835273	genic_upstream_transcript_variant,intron_variant
rs555843444	intron_variant
rs555844918	genic_downstream_transcript_variant,intron_variant
rs555859249	genic_upstream_transcript_variant,intron_variant

rs555864895	intron_variant
rs555887066	genic_upstream_transcript_variant,intron_variant
rs555892420	intron_variant
rs555898916	genic_upstream_transcript_variant,intron_variant
rs555913265	intron_variant
rs555945492	genic_upstream_transcript_variant,intron_variant
rs555950092	intron_variant
rs556011659	genic_upstream_transcript_variant,intron_variant
rs556013284	genic_upstream_transcript_variant,intron_variant
rs556038889	genic_upstream_transcript_variant,upstream_transcript_variant,intron_variant
rs556060143	genic_downstream_transcript_variant,intron_variant
rs556065804	downstream_transcript_variant,500B_downstream_variant
rs556070899	genic_upstream_transcript_variant,intron_variant
rs556075940	genic_upstream_transcript_variant,upstream_transcript_variant,intron_variant
rs556123759	genic_upstream_transcript_variant,intron_variant
rs556125651	genic_upstream_transcript_variant,intron_variant
rs556187071	genic_upstream_transcript_variant,intron_variant
rs556216308	intron_variant
rs556249753	intron_variant
rs556268027	intron_variant
rs556307717	intron_variant
rs556318484	genic_upstream_transcript_variant,intron_variant
rs556325842	genic_upstream_transcript_variant,intron_variant
rs556402208	genic_upstream_transcript_variant,intron_variant
rs556438585	intron_variant
rs556464511	genic_upstream_transcript_variant,intron_variant
rs556475794	genic_upstream_transcript_variant,intron_variant
rs556477309	intron_variant
rs556477349	intron_variant
rs556525894	genic_upstream_transcript_variant,intron_variant
rs556537526	intron_variant
rs556541308	2KB_upstream_variant,genic_upstream_transcript_variant,upstream_transcript_variant,intron_variant
rs556568244	genic_upstream_transcript_variant,intron_variant
rs556571788	intron_variant
rs556572301	intron_variant
rs556606506	intron_variant
rs556630746	intron_variant
rs556646620	intron_variant
rs556695962	intron_variant
rs556702600	intron_variant
rs556715633	genic_upstream_transcript_variant,intron_variant
rs556731087	genic_upstream_transcript_variant,intron_variant
rs556734218	intron_variant
rs556737816	intron_variant
rs556742174	genic_downstream_transcript_variant,3_prime_UTR_variant,non_coding_transcript_variant
rs556783464	genic_downstream_transcript_variant,intron_variant
rs556824187	genic_downstream_transcript_variant,intron_variant
rs556877595	genic_upstream_transcript_variant,intron_variant
rs556878090	intron_variant
rs556902471	intron_variant
rs556914824	intron_variant
rs557009907	intron_variant
rs557013295	intron_variant
rs557025386	intron_variant
rs557030739	genic_upstream_transcript_variant,intron_variant
rs557046660	intron_variant
rs557047501	intron_variant
rs557048475	intron_variant
rs557064660	genic_downstream_transcript_variant,intron_variant
rs557085805	genic_downstream_transcript_variant,intron_variant
rs557146277	intron_variant
rs557160157	genic_upstream_transcript_variant,intron_variant
rs557182947	intron_variant
rs557190488	genic_upstream_transcript_variant,intron_variant
rs557191629	genic_downstream_transcript_variant,intron_variant
rs557199935	genic_upstream_transcript_variant,intron_variant
rs557228730	genic_downstream_transcript_variant,intron_variant
rs557259711	intron_variant
rs557263221	genic_upstream_transcript_variant,intron_variant
rs557269117	intron_variant
rs557296950	genic_upstream_transcript_variant,intron_variant
rs557300996	downstream_transcript_variant,genic_downstream_transcript_variant,intron_variant
rs557307992	genic_downstream_transcript_variant,intron_variant
rs557364175	intron_variant

rs557380914	genic_upstream_transcript_variant,intron_variant
rs557401337	genic_downstream_transcript_variant,intron_variant
rs557401338	genic_downstream_transcript_variant,intron_variant
rs557407118	genic_downstream_transcript_variant,intron_variant
rs557408448	intron_variant
rs557437505	genic_upstream_transcript_variant,intron_variant
rs557501904	genic_downstream_transcript_variant,intron_variant
rs557507071	intron_variant
rs557507912	genic_upstream_transcript_variant,intron_variant
rs557569918	intron_variant
rs557573157	genic_upstream_transcript_variant,intron_variant
rs557578292	genic_downstream_transcript_variant,intron_variant
rs557636134	genic_upstream_transcript_variant,intron_variant
rs557646318	genic_upstream_transcript_variant,intron_variant
rs557671794	intron_variant
rs557699703	2KB_upstream_variant,genic_upstream_transcript_variant,upstream_transcript_variant,intron_variant
rs557718095	2KB_upstream_variant,genic_upstream_transcript_variant,upstream_transcript_variant,intron_variant
rs557719709	genic_downstream_transcript_variant,intron_variant
rs557765583	downstream_transcript_variant,500B_downstream_variant
rs557820994	intron_variant
rs557837727	genic_upstream_transcript_variant,intron_variant
rs557851750	genic_downstream_transcript_variant,3_prime_UTR_variant,non_coding_transcript_variant
rs557861718	intron_variant
rs557886701	genic_downstream_transcript_variant,intron_variant
rs557899935	2KB_upstream_variant,genic_upstream_transcript_variant,upstream_transcript_variant,intron_variant
rs557905262	genic_upstream_transcript_variant,intron_variant
rs557923392	intron_variant
rs557948939	genic_upstream_transcript_variant,intron_variant
rs557972178	genic_upstream_transcript_variant,intron_variant
rs557984934	genic_upstream_transcript_variant,intron_variant
rs557985061	genic_upstream_transcript_variant,intron_variant
rs558008526	genic_upstream_transcript_variant,intron_variant
rs558020397	genic_upstream_transcript_variant,intron_variant
rs558059449	intron_variant
rs558062180	genic_upstream_transcript_variant,intron_variant
rs558069187	genic_downstream_transcript_variant,intron_variant
rs558107770	genic_upstream_transcript_variant,intron_variant
rs558125492	intron_variant
rs558132414	genic_downstream_transcript_variant,intron_variant
rs558134898	intron_variant
rs558171313	2KB_upstream_variant,genic_upstream_transcript_variant,upstream_transcript_variant,intron_variant
rs558171992	genic_upstream_transcript_variant,intron_variant
rs558179437	genic_downstream_transcript_variant,intron_variant
rs558225070	intron_variant
rs558282289	genic_upstream_transcript_variant,intron_variant
rs558282945	genic_upstream_transcript_variant,intron_variant
rs558297687	genic_upstream_transcript_variant,intron_variant
rs558308666	genic_downstream_transcript_variant,intron_variant
rs558318149	genic_upstream_transcript_variant,intron_variant
rs558318982	genic_downstream_transcript_variant,intron_variant
rs558382003	genic_upstream_transcript_variant,intron_variant
rs558413610	intron_variant
rs558427109	intron_variant
rs558435638	genic_downstream_transcript_variant,intron_variant
rs558441244	genic_upstream_transcript_variant,intron_variant
rs558442118	intron_variant
rs558532849	2KB_upstream_variant,genic_upstream_transcript_variant,upstream_transcript_variant,intron_variant
rs558543477	genic_downstream_transcript_variant,intron_variant
rs558561366	genic_upstream_transcript_variant,intron_variant
rs558576370	genic_upstream_transcript_variant,intron_variant
rs558670539	intron_variant
rs558671734	genic_upstream_transcript_variant,intron_variant
rs558677066	intron_variant
rs558689684	genic_upstream_transcript_variant,intron_variant
rs558691310	intron_variant
rs558691321	intron_variant
rs558706578	2KB_upstream_variant,genic_upstream_transcript_variant,upstream_transcript_variant,intron_variant
rs558716467	genic_upstream_transcript_variant,upstream_transcript_variant,intron_variant
rs558788015	genic_downstream_transcript_variant,intron_variant
rs558811815	intron_variant
rs558839515	intron_variant
rs558870053	intron_variant
rs558889725	intron_variant
rs558907657	intron_variant

rs558928551	intron_variant
rs558933063	genic_downstream_transcript_variant,intron_variant
rs558979535	intron_variant
rs558989509	genic_upstream_transcript_variant,intron_variant
rs559010763	intron_variant
rs559014414	genic_upstream_transcript_variant,intron_variant
rs559015974	intron_variant
rs559046502	intron_variant
rs559104396	genic_downstream_transcript_variant,intron_variant
rs559109952	intron_variant
rs559171383	2KB_upstream_variant,genic_upstream_transcript_variant,upstream_transcript_variant,intron_variant
rs559173921	genic_upstream_transcript_variant,intron_variant
rs559208441	genic_downstream_transcript_variant,intron_variant
rs559223755	intron_variant
rs559248462	genic_upstream_transcript_variant,intron_variant
rs559269443	intron_variant
rs559301972	intron_variant
rs559340035	genic_downstream_transcript_variant,intron_variant
rs559345366	intron_variant
rs559354080	intron_variant
rs559388566	intron_variant
rs559392792	genic_upstream_transcript_variant,intron_variant
rs559442566	intron_variant
rs559443524	genic_upstream_transcript_variant,intron_variant
rs559495646	genic_downstream_transcript_variant,intron_variant
rs559504357	genic_upstream_transcript_variant,intron_variant
rs559520663	intron_variant
rs559532713	genic_downstream_transcript_variant,intron_variant
rs559537957	genic_upstream_transcript_variant,intron_variant
rs559547248	genic_upstream_transcript_variant,intron_variant
rs559591638	intron_variant
rs559629856	genic_upstream_transcript_variant,intron_variant
rs559653995	genic_upstream_transcript_variant,intron_variant
rs559660484	intron_variant
rs559712001	genic_downstream_transcript_variant,intron_variant
rs559713751	intron_variant
rs559736533	genic_upstream_transcript_variant,intron_variant
rs559747627	genic_downstream_transcript_variant,intron_variant
rs559748015	genic_downstream_transcript_variant,intron_variant
rs559778228	2KB_upstream_variant,genic_upstream_transcript_variant,upstream_transcript_variant,intron_variant
rs559797330	genic_upstream_transcript_variant,upstream_transcript_variant,intron_variant
rs559799281	genic_upstream_transcript_variant,intron_variant
rs559809381	genic_upstream_transcript_variant,intron_variant
rs559822726	genic_downstream_transcript_variant,intron_variant
rs559834327	intron_variant
rs559836184	genic_upstream_transcript_variant,intron_variant
rs559884260	genic_upstream_transcript_variant,intron_variant
rs560016183	genic_upstream_transcript_variant,intron_variant
rs560026608	intron_variant
rs560033098	genic_upstream_transcript_variant,intron_variant
rs560035458	intron_variant
rs560044400	intron_variant
rs560089517	intron_variant
rs560093559	genic_upstream_transcript_variant,intron_variant
rs560159298	intron_variant
rs560162629	genic_upstream_transcript_variant,intron_variant
rs560179368	genic_upstream_transcript_variant,intron_variant
rs560239873	intron_variant
rs560259454	genic_upstream_transcript_variant,intron_variant
rs560266467	genic_upstream_transcript_variant,intron_variant
rs560279523	intron_variant
rs560285479	genic_upstream_transcript_variant,intron_variant
rs560298133	genic_downstream_transcript_variant,3_prime_UTR_variant,non_coding_transcript_variant
rs560309592	intron_variant
rs560324112	genic_upstream_transcript_variant,intron_variant
rs560364895	intron_variant
rs560496764	intron_variant
rs560500537	intron_variant
rs560500958	intron_variant
rs560531035	genic_upstream_transcript_variant,intron_variant
rs560565791	intron_variant
rs560623890	genic_upstream_transcript_variant,intron_variant
rs560637490	genic_upstream_transcript_variant,intron_variant
rs560650260	genic_downstream_transcript_variant,intron_variant

rs560658903	genic_upstream_transcript_variant,intron_variant
rs560674910	genic_downstream_transcript_variant,intron_variant
rs560694099	genic_upstream_transcript_variant,intron_variant
rs560719624	genic_upstream_transcript_variant,intron_variant
rs560720032	intron_variant
rs560735696	genic_downstream_transcript_variant,intron_variant
rs560756723	5_prime_UTR_variant,intron_variant
rs560798338	genic_upstream_transcript_variant,intron_variant
rs560808755	genic_upstream_transcript_variant,intron_variant
rs560845270	genic_upstream_transcript_variant,upstream_transcript_variant,intron_variant
rs560845502	intron_variant
rs560860037	intron_variant
rs560864151	intron_variant
rs560877696	intron_variant
rs560878054	genic_downstream_transcript_variant,3_prime_UTR_variant,non_coding_transcript_variant
rs560882183	intron_variant
rs560970209	genic_upstream_transcript_variant,intron_variant
rs560981803	genic_downstream_transcript_variant,intron_variant
rs560996074	intron_variant
rs561050635	genic_upstream_transcript_variant,intron_variant
rs561060423	intron_variant
rs561091466	genic_upstream_transcript_variant,intron_variant
rs561098894	genic_downstream_transcript_variant,intron_variant
rs561148859	genic_upstream_transcript_variant,intron_variant
rs561178266	genic_downstream_transcript_variant,intron_variant
rs561185197	genic_downstream_transcript_variant,intron_variant
rs561186414	genic_upstream_transcript_variant,intron_variant
rs561187150	genic_downstream_transcript_variant,intron_variant
rs561202682	intron_variant
rs561252733	genic_upstream_transcript_variant,intron_variant
rs561311108	genic_upstream_transcript_variant,intron_variant
rs561318256	genic_downstream_transcript_variant,intron_variant
rs561347675	missense_variant,coding_sequence_variant,non_coding_transcript_variant
rs561355927	genic_downstream_transcript_variant,intron_variant
rs561420700	intron_variant
rs561442879	intron_variant
rs561500435	genic_downstream_transcript_variant,intron_variant
rs561529682	intron_variant
rs561553348	intron_variant
rs561567490	intron_variant
rs561569871	genic_upstream_transcript_variant,intron_variant
rs561591351	genic_downstream_transcript_variant,intron_variant
rs561631218	genic_upstream_transcript_variant,intron_variant
rs561639095	intron_variant
rs561650983	genic_downstream_transcript_variant,intron_variant
rs561661506	genic_upstream_transcript_variant,intron_variant
rs561696189	intron_variant
rs561710830	intron_variant
rs561717936	genic_upstream_transcript_variant,intron_variant
rs561745397	genic_upstream_transcript_variant,intron_variant
rs561770433	intron_variant
rs561779275	2KB_upstream_variant,genic_upstream_transcript_variant,upstream_transcript_variant,intron_variant
rs561791444	intron_variant
rs561802121	genic_upstream_transcript_variant,intron_variant
rs561809091	genic_upstream_transcript_variant,intron_variant
rs561830898	genic_downstream_transcript_variant,intron_variant
rs561877850	genic_downstream_transcript_variant,intron_variant
rs561888590	genic_upstream_transcript_variant,intron_variant
rs561903185	genic_upstream_transcript_variant,intron_variant
rs561908240	genic_upstream_transcript_variant,intron_variant
rs561913833	genic_upstream_transcript_variant,intron_variant
rs561932408	intron_variant
rs561940865	intron_variant
rs561972143	genic_upstream_transcript_variant,intron_variant
rs562002883	intron_variant
rs562072771	genic_upstream_transcript_variant,intron_variant
rs562111069	genic_downstream_transcript_variant,intron_variant
rs562140062	genic_upstream_transcript_variant,intron_variant
rs562142208	genic_upstream_transcript_variant,intron_variant
rs562168351	genic_upstream_transcript_variant,intron_variant
rs562170390	genic_downstream_transcript_variant,intron_variant
rs562175784	genic_upstream_transcript_variant,intron_variant
rs562189308	intron_variant
rs562201153	genic_upstream_transcript_variant,intron_variant

rs562228235	intron_variant
rs562234679	genic_upstream_transcript_variant,intron_variant
rs562244458	intron_variant
rs562259176	genic_downstream_transcript_variant,intron_variant
rs562270912	genic_upstream_transcript_variant,intron_variant
rs562311983	genic_upstream_transcript_variant,intron_variant
rs562334254	genic_downstream_transcript_variant,intron_variant
rs562337904	genic_upstream_transcript_variant,intron_variant
rs562339461	intron_variant
rs562396709	genic_downstream_transcript_variant,3_prime_UTR_variant,non_coding_transcript_variant
rs562463429	genic_upstream_transcript_variant,intron_variant
rs562472188	intron_variant
rs562474467	2KB_upstream_variant,genic_upstream_transcript_variant,upstream_transcript_variant,intron_variant
rs562509350	intron_variant
rs562531041	genic_downstream_transcript_variant,intron_variant
rs562541499	intron_variant
rs562564020	genic_downstream_transcript_variant,intron_variant
rs562564732	missense_variant,coding_sequence_variant,non_coding_transcript_variant
rs562619748	genic_upstream_transcript_variant,intron_variant
rs562652155	genic_downstream_transcript_variant,intron_variant
rs562657118	intron_variant
rs562659115	intron_variant
rs562676757	intron_variant
rs562714752	intron_variant
rs562721764	genic_upstream_transcript_variant,intron_variant,non_coding_transcript_variant
rs562748370	intron_variant
rs562825473	genic_downstream_transcript_variant,intron_variant
rs562979133	genic_upstream_transcript_variant,intron_variant
rs563007490	genic_upstream_transcript_variant,intron_variant
rs563045022	intron_variant
rs563045958	intron_variant
rs563055217	genic_upstream_transcript_variant,intron_variant
rs563064456	genic_downstream_transcript_variant,intron_variant
rs563064505	genic_upstream_transcript_variant,intron_variant
rs563094850	genic_upstream_transcript_variant,intron_variant
rs563146125	intron_variant
rs563185057	genic_upstream_transcript_variant,intron_variant
rs563189410	genic_downstream_transcript_variant,intron_variant
rs563191426	genic_downstream_transcript_variant,intron_variant
rs563220772	2KB_upstream_variant,genic_upstream_transcript_variant,upstream_transcript_variant,intron_variant
rs563225683	genic_downstream_transcript_variant,intron_variant
rs563233434	intron_variant
rs563250599	genic_upstream_transcript_variant,intron_variant
rs563278893	intron_variant
rs563279428	genic_upstream_transcript_variant,intron_variant
rs563312716	intron_variant
rs563319707	intron_variant
rs563337977	genic_downstream_transcript_variant,intron_variant
rs563343106	genic_upstream_transcript_variant,intron_variant
rs563360447	genic_upstream_transcript_variant,intron_variant
rs563409320	intron_variant
rs563413956	intron_variant
rs563434056	genic_upstream_transcript_variant,intron_variant
rs563453170	intron_variant
rs563463841	genic_upstream_transcript_variant,upstream_transcript_variant,intron_variant
rs563531071	intron_variant
rs563534182	genic_upstream_transcript_variant,intron_variant
rs563567855	genic_upstream_transcript_variant,intron_variant
rs563592976	intron_variant
rs563595596	genic_upstream_transcript_variant,intron_variant
rs563599359	genic_upstream_transcript_variant,intron_variant
rs563608993	5_prime_UTR_variant,intron_variant
rs563631358	genic_downstream_transcript_variant,intron_variant
rs563706962	genic_upstream_transcript_variant,intron_variant
rs563716794	genic_upstream_transcript_variant,intron_variant
rs563747292	intron_variant
rs563800150	genic_upstream_transcript_variant,intron_variant
rs563803328	genic_downstream_transcript_variant,intron_variant
rs563840110	genic_downstream_transcript_variant,intron_variant
rs563888661	intron_variant
rs563907136	intron_variant
rs563954693	intron_variant
rs563960993	intron_variant
rs564001592	genic_upstream_transcript_variant,intron_variant

rs564045805	genic_upstream_transcript_variant,intron_variant
rs564115135	genic_upstream_transcript_variant,intron_variant
rs564146639	genic_upstream_transcript_variant,intron_variant
rs564179426	genic_downstream_transcript_variant,intron_variant
rs564180465	genic_upstream_transcript_variant,intron_variant
rs564263321	intron_variant
rs564267389	genic_upstream_transcript_variant,intron_variant
rs564290425	intron_variant
rs564290838	intron_variant
rs564299007	genic_upstream_transcript_variant,intron_variant
rs564311728	genic_upstream_transcript_variant,intron_variant
rs564325037	intron_variant
rs564359745	genic_downstream_transcript_variant,intron_variant
rs564415838	genic_upstream_transcript_variant,intron_variant
rs564456203	genic_upstream_transcript_variant,intron_variant
rs564471390	intron_variant
rs564472559	intron_variant
rs564561731	intron_variant
rs564569484	genic_upstream_transcript_variant,intron_variant
rs564575523	genic_downstream_transcript_variant,intron_variant
rs564586978	intron_variant
rs564596798	intron_variant
rs564638188	genic_downstream_transcript_variant,intron_variant
rs564648642	intron_variant
rs564648735	intron_variant
rs564662260	genic_downstream_transcript_variant,intron_variant
rs564713000	genic_upstream_transcript_variant,intron_variant
rs564749735	genic_upstream_transcript_variant,intron_variant
rs564765380	intron_variant
rs564779395	genic_upstream_transcript_variant,intron_variant
rs564849565	genic_upstream_transcript_variant,intron_variant
rs564860523	intron_variant
rs564864821	genic_downstream_transcript_variant,intron_variant
rs564876950	genic_upstream_transcript_variant,intron_variant
rs564891216	intron_variant
rs564894613	genic_upstream_transcript_variant,intron_variant
rs564909330	genic_downstream_transcript_variant,intron_variant
rs564984562	upstream_transcript_variant,2KB_upstream_variant,genic_upstream_transcript_variant,intron_variant
rs565000347	intron_variant
rs565026414	genic_upstream_transcript_variant,intron_variant
rs565033466	intron_variant
rs565051937	genic_downstream_transcript_variant,intron_variant
rs565052258	intron_variant
rs565098516	genic_upstream_transcript_variant,intron_variant
rs565143020	genic_downstream_transcript_variant,intron_variant
rs565153629	intron_variant
rs565155913	intron_variant
rs565182900	genic_upstream_transcript_variant,intron_variant
rs565196848	genic_upstream_transcript_variant,intron_variant
rs565225885	intron_variant
rs565227835	intron_variant
rs565263933	intron_variant
rs565284912	genic_upstream_transcript_variant,intron_variant
rs565340536	genic_downstream_transcript_variant,intron_variant
rs565349390	genic_downstream_transcript_variant,intron_variant
rs565368885	genic_upstream_transcript_variant,intron_variant
rs565421203	intron_variant
rs565479601	intron_variant
rs565517972	genic_upstream_transcript_variant,intron_variant
rs565518326	genic_upstream_transcript_variant,intron_variant
rs565520032	genic_upstream_transcript_variant,intron_variant
rs565529338	genic_downstream_transcript_variant,intron_variant
rs565554780	intron_variant
rs565566853	genic_upstream_transcript_variant,intron_variant
rs565581078	genic_upstream_transcript_variant,intron_variant
rs565602135	intron_variant
rs565604638	intron_variant
rs565630171	genic_downstream_transcript_variant,intron_variant
rs565683451	intron_variant
rs565701148	intron_variant
rs565705941	upstream_transcript_variant,2KB_upstream_variant,genic_upstream_transcript_variant,intron_variant
rs565728956	genic_downstream_transcript_variant,3_prime_UTR_variant,non_coding_transcript_variant
rs565769652	genic_downstream_transcript_variant,intron_variant
rs565840108	intron_variant

rs565842370	genic_upstream_transcript_variant,intron_variant
rs565901515	genic_upstream_transcript_variant,intron_variant
rs565909763	genic_upstream_transcript_variant,intron_variant
rs565927958	genic_downstream_transcript_variant,intron_variant
rs565945762	genic_downstream_transcript_variant,intron_variant
rs565953301	intron_variant
rs565979428	genic_upstream_transcript_variant,intron_variant
rs565992619	intron_variant
rs566027684	genic_upstream_transcript_variant,intron_variant
rs566061763	intron_variant
rs566080892	genic_upstream_transcript_variant,intron_variant
rs566100307	genic_downstream_transcript_variant,intron_variant
rs566133571	intron_variant
rs566187439	genic_upstream_transcript_variant,intron_variant
rs566214192	genic_upstream_transcript_variant,intron_variant
rs566231589	genic_upstream_transcript_variant,intron_variant
rs566286225	intron_variant
rs566296175	genic_upstream_transcript_variant,intron_variant
rs566325516	genic_upstream_transcript_variant,intron_variant
rs566328036	intron_variant
rs566359835	genic_upstream_transcript_variant,intron_variant
rs566359904	genic_upstream_transcript_variant,intron_variant
rs566359915	genic_upstream_transcript_variant,intron_variant
rs566371663	genic_downstream_transcript_variant,intron_variant
rs566394943	upstream_transcript_variant,2KB_upstream_variant,genic_upstream_transcript_variant,intron_variant
rs566413192	intron_variant
rs566413912	intron_variant
rs566416598	genic_upstream_transcript_variant,intron_variant
rs566450176	genic_downstream_transcript_variant,intron_variant
rs566454800	intron_variant
rs566534022	intron_variant
rs566550417	genic_upstream_transcript_variant,intron_variant
rs566551459	intron_variant
rs566566006	intron_variant
rs566617956	non_coding_transcript_variant,genic_downstream_transcript_variant,coding_sequence_variant,synonymous_variant
rs566704837	intron_variant
rs566711407	intron_variant
rs566713361	genic_downstream_transcript_variant,intron_variant
rs566737506	genic_upstream_transcript_variant,intron_variant
rs566744493	intron_variant
rs566762877	genic_downstream_transcript_variant,intron_variant
rs566790058	genic_upstream_transcript_variant,intron_variant
rs566792422	5_prime_UTR_variant,non_coding_transcript_variant,genic_upstream_transcript_variant,intron_variant
rs566813792	intron_variant
rs566843188	genic_downstream_transcript_variant,intron_variant
rs566896765	genic_upstream_transcript_variant,intron_variant
rs566916755	intron_variant
rs566943801	intron_variant
rs566945822	genic_upstream_transcript_variant,intron_variant
rs566977547	genic_downstream_transcript_variant,intron_variant
rs566982278	intron_variant
rs566993048	genic_downstream_transcript_variant,intron_variant
rs567033632	intron_variant
rs567055239	intron_variant
rs567063919	intron_variant
rs567088819	genic_upstream_transcript_variant,intron_variant
rs567116463	genic_downstream_transcript_variant,intron_variant
rs567120617	intron_variant
rs567122904	intron_variant
rs567128834	genic_downstream_transcript_variant,intron_variant
rs567136590	genic_upstream_transcript_variant,intron_variant
rs567175972	genic_downstream_transcript_variant,intron_variant
rs567207379	intron_variant
rs567228644	genic_downstream_transcript_variant,intron_variant
rs567229868	genic_downstream_transcript_variant,intron_variant
rs567246729	genic_downstream_transcript_variant,intron_variant
rs567256878	genic_upstream_transcript_variant,intron_variant
rs567281353	genic_downstream_transcript_variant,intron_variant
rs567294559	genic_upstream_transcript_variant,intron_variant
rs567312869	genic_downstream_transcript_variant,intron_variant
rs567358450	genic_upstream_transcript_variant,intron_variant
rs567366255	genic_upstream_transcript_variant,intron_variant
rs567380575	intron_variant
rs567390429	genic_downstream_transcript_variant,intron_variant

rs567393232	upstream_transcript_variant,2KB_upstream_variant,genic_upstream_transcript_variant,intron_variant
rs567394566	intron_variant
rs567442689	intron_variant
rs567471769	genic_upstream_transcript_variant,intron_variant
rs567511555	genic_downstream_transcript_variant,intron_variant
rs567530345	genic_upstream_transcript_variant,intron_variant
rs567559667	genic_upstream_transcript_variant,intron_variant
rs567581042	genic_downstream_transcript_variant,intron_variant
rs567636506	intron_variant
rs567639477	genic_downstream_transcript_variant,intron_variant
rs567685524	intron_variant
rs567686687	genic_downstream_transcript_variant,intron_variant
rs567698515	genic_downstream_transcript_variant,intron_variant
rs567705129	intron_variant
rs567705718	genic_upstream_transcript_variant,intron_variant
rs567777778	intron_variant
rs567846166	intron_variant
rs567866465	genic_upstream_transcript_variant,intron_variant
rs567881883	intron_variant
rs567935138	upstream_transcript_variant,2KB_upstream_variant,genic_upstream_transcript_variant,intron_variant
rs567950150	genic_upstream_transcript_variant,intron_variant
rs567994951	genic_downstream_transcript_variant,intron_variant
rs567999341	intron_variant
rs568119445	intron_variant
rs568120123	genic_downstream_transcript_variant,intron_variant
rs568152978	genic_downstream_transcript_variant,intron_variant
rs568157672	genic_upstream_transcript_variant,intron_variant
rs568180587	genic_upstream_transcript_variant,intron_variant
rs568260358	intron_variant
rs568293455	genic_upstream_transcript_variant,intron_variant
rs568351783	upstream_transcript_variant,2KB_upstream_variant,genic_upstream_transcript_variant,intron_variant
rs568387876	intron_variant
rs568409295	intron_variant
rs568452408	intron_variant
rs568458534	intron_variant
rs568470646	intron_variant
rs568481618	intron_variant
rs568495386	genic_upstream_transcript_variant,intron_variant
rs568561864	genic_downstream_transcript_variant,intron_variant
rs568600369	intron_variant
rs568626761	intron_variant
rs568652122	genic_upstream_transcript_variant,intron_variant
rs568734054	genic_downstream_transcript_variant,3_prime_UTR_variant,non_coding_transcript_variant
rs568760092	intron_variant
rs568776842	intron_variant
rs568796085	genic_downstream_transcript_variant,downstream_transcript_variant,intron_variant
rs568805040	genic_upstream_transcript_variant,intron_variant
rs568853289	intron_variant
rs568873990	genic_downstream_transcript_variant,intron_variant
rs568930922	intron_variant
rs568944744	intron_variant
rs568964246	upstream_transcript_variant,genic_upstream_transcript_variant,intron_variant
rs568966871	intron_variant
rs568969775	intron_variant
rs569003930	intron_variant
rs569047642	intron_variant
rs569074363	intron_variant
rs569104133	intron_variant
rs569147645	intron_variant
rs569222113	intron_variant
rs569225474	intron_variant
rs569243564	intron_variant
rs569260414	upstream_transcript_variant,2KB_upstream_variant,genic_upstream_transcript_variant,intron_variant
rs569283948	genic_upstream_transcript_variant,intron_variant
rs569286078	genic_downstream_transcript_variant,intron_variant
rs569292290	intron_variant
rs569298077	intron_variant
rs569314925	genic_downstream_transcript_variant,intron_variant
rs569326210	intron_variant
rs569352632	genic_downstream_transcript_variant,intron_variant
rs569365762	intron_variant
rs569370314	genic_upstream_transcript_variant,intron_variant
rs569429258	genic_upstream_transcript_variant,intron_variant
rs569440962	genic_upstream_transcript_variant,intron_variant

rs569467794	genic_downstream_transcript_variant,intron_variant
rs569472551	genic_downstream_transcript_variant,intron_variant
rs569493936	genic_upstream_transcript_variant,intron_variant
rs569512412	intron_variant
rs569551511	intron_variant
rs569582074	genic_upstream_transcript_variant,intron_variant
rs569599303	genic_upstream_transcript_variant,intron_variant
rs569650197	genic_upstream_transcript_variant,intron_variant
rs569651880	genic_upstream_transcript_variant,intron_variant
rs569743981	500B_downstream_variant,downstream_transcript_variant
rs569744962	upstream_transcript_variant,2KB_upstream_variant,genic_upstream_transcript_variant,intron_variant
rs569756075	genic_upstream_transcript_variant,intron_variant
rs569759238	intron_variant
rs569773563	intron_variant
rs569786918	genic_downstream_transcript_variant,intron_variant
rs569853349	genic_upstream_transcript_variant,intron_variant
rs569889379	genic_upstream_transcript_variant,intron_variant
rs569897707	genic_upstream_transcript_variant,intron_variant
rs569925845	genic_upstream_transcript_variant,intron_variant
rs569934255	genic_upstream_transcript_variant,intron_variant
rs569935290	intron_variant
rs569954666	genic_downstream_transcript_variant,intron_variant
rs570017283	intron_variant
rs570036852	genic_upstream_transcript_variant,intron_variant
rs570043311	intron_variant
rs570141585	genic_upstream_transcript_variant,intron_variant
rs570191239	genic_downstream_transcript_variant,intron_variant
rs570259313	genic_upstream_transcript_variant,intron_variant
rs570263538	genic_downstream_transcript_variant,intron_variant
rs570280645	intron_variant
rs570300329	intron_variant
rs570301178	genic_downstream_transcript_variant,intron_variant
rs570321143	intron_variant
rs570324539	genic_upstream_transcript_variant,intron_variant
rs570326644	genic_upstream_transcript_variant,intron_variant
rs570328815	intron_variant
rs570341142	genic_upstream_transcript_variant,intron_variant
rs570351099	genic_downstream_transcript_variant,intron_variant
rs570361275	upstream_transcript_variant,2KB_upstream_variant,genic_upstream_transcript_variant,intron_variant
rs570386997	genic_upstream_transcript_variant,intron_variant
rs570404894	upstream_transcript_variant,2KB_upstream_variant,genic_upstream_transcript_variant,intron_variant
rs570416991	intron_variant
rs570436523	genic_upstream_transcript_variant,intron_variant
rs570470680	genic_upstream_transcript_variant,intron_variant
rs570472090	genic_upstream_transcript_variant,intron_variant
rs570678032	intron_variant
rs570714333	intron_variant
rs570802138	intron_variant
rs570805373	genic_upstream_transcript_variant,intron_variant
rs570828437	genic_upstream_transcript_variant,intron_variant
rs570855669	intron_variant
rs570860788	genic_downstream_transcript_variant,intron_variant
rs570871843	genic_upstream_transcript_variant,intron_variant
rs570915736	intron_variant
rs570936347	genic_downstream_transcript_variant,intron_variant
rs570981871	genic_downstream_transcript_variant,intron_variant
rs570993098	genic_upstream_transcript_variant,intron_variant
rs570999335	genic_upstream_transcript_variant,intron_variant
rs571001808	intron_variant
rs571014763	genic_upstream_transcript_variant,intron_variant
rs571020696	genic_downstream_transcript_variant,3_prime_UTR_variant,non_coding_transcript_variant
rs571021707	intron_variant
rs571027570	intron_variant
rs571122923	intron_variant
rs571145677	genic_upstream_transcript_variant,intron_variant
rs571156532	upstream_transcript_variant,genic_upstream_transcript_variant,intron_variant
rs571198784	genic_upstream_transcript_variant,intron_variant
rs571208359	genic_upstream_transcript_variant,intron_variant
rs571208389	genic_upstream_transcript_variant,intron_variant
rs571243169	intron_variant
rs571253866	intron_variant
rs571262132	genic_upstream_transcript_variant,intron_variant
rs571277516	genic_downstream_transcript_variant,intron_variant
rs571290716	genic_downstream_transcript_variant,intron_variant

rs571347127	intron_variant
rs571363146	intron_variant
rs571369878	intron_variant
rs571397768	genic_upstream_transcript_variant,intron_variant
rs571403009	genic_downstream_transcript_variant,intron_variant
rs571419819	genic_downstream_transcript_variant,intron_variant
rs571453992	missense_variant,non_coding_transcript_variant,genic_upstream_transcript_variant,coding_sequence_variant,intron_variant
rs571496258	intron_variant
rs571496301	genic_downstream_transcript_variant,intron_variant
rs571501976	intron_variant
rs571503749	genic_upstream_transcript_variant,intron_variant
rs571513386	genic_downstream_transcript_variant,intron_variant
rs571519188	intron_variant
rs571532416	genic_downstream_transcript_variant,intron_variant
rs571566952	missense_variant,coding_sequence_variant,intron_variant
rs571659316	genic_downstream_transcript_variant,intron_variant
rs571698885	intron_variant
rs571717363	genic_downstream_transcript_variant,intron_variant
rs571746962	genic_downstream_transcript_variant,intron_variant
rs571793113	5_prime_UTR_variant,upstream_transcript_variant,genic_upstream_transcript_variant,intron_variant
rs571794724	genic_downstream_transcript_variant,intron_variant
rs571813771	intron_variant
rs571838067	intron_variant
rs571846974	intron_variant
rs571856829	upstream_transcript_variant,genic_upstream_transcript_variant,intron_variant
rs571870925	genic_downstream_transcript_variant,intron_variant
rs571874671	intron_variant
rs571881076	genic_upstream_transcript_variant,intron_variant
rs571938291	genic_upstream_transcript_variant,intron_variant
rs572035958	genic_upstream_transcript_variant,intron_variant
rs572038412	genic_upstream_transcript_variant,intron_variant
rs572058092	intron_variant
rs572061828	genic_upstream_transcript_variant,intron_variant
rs572075442	genic_upstream_transcript_variant,intron_variant
rs572084726	intron_variant
rs572088455	intron_variant
rs572161353	upstream_transcript_variant,2KB_upstream_variant,genic_upstream_transcript_variant,intron_variant
rs572172614	genic_upstream_transcript_variant,intron_variant
rs572172818	intron_variant
rs572233520	intron_variant
rs572253987	genic_downstream_transcript_variant,intron_variant
rs572266600	intron_variant
rs572293126	genic_upstream_transcript_variant,intron_variant
rs572315099	genic_downstream_transcript_variant,intron_variant
rs572333379	genic_upstream_transcript_variant,intron_variant
rs572342218	intron_variant
rs572350205	genic_downstream_transcript_variant,intron_variant
rs572358873	genic_upstream_transcript_variant,intron_variant
rs572366356	upstream_transcript_variant,2KB_upstream_variant,genic_upstream_transcript_variant,intron_variant
rs572379502	genic_downstream_transcript_variant,intron_variant
rs572384348	genic_upstream_transcript_variant,intron_variant
rs572396298	genic_upstream_transcript_variant,intron_variant
rs572447489	intron_variant
rs572458221	genic_upstream_transcript_variant,intron_variant
rs572499967	genic_upstream_transcript_variant,intron_variant
rs572519162	genic_upstream_transcript_variant,intron_variant
rs572535159	intron_variant
rs572566741	intron_variant
rs572572112	intron_variant
rs572599245	intron_variant
rs572637047	genic_downstream_transcript_variant,intron_variant
rs572709519	genic_upstream_transcript_variant,intron_variant
rs572713479	intron_variant
rs572724690	genic_downstream_transcript_variant,intron_variant
rs572793775	genic_upstream_transcript_variant,intron_variant
rs572808693	genic_upstream_transcript_variant,intron_variant
rs572854237	intron_variant
rs572890551	genic_upstream_transcript_variant,intron_variant
rs572939708	intron_variant
rs572941913	intron_variant
rs572961673	genic_upstream_transcript_variant,intron_variant
rs572971674	intron_variant
rs572973569	genic_upstream_transcript_variant,intron_variant
rs573007894	intron_variant

rs573027298	genic_downstream_transcript_variant,intron_variant
rs573050256	genic_upstream_transcript_variant,intron_variant
rs573071639	intron_variant
rs573081576	genic_upstream_transcript_variant,intron_variant
rs573122315	genic_downstream_transcript_variant,intron_variant
rs573147485	intron_variant
rs573157234	genic_upstream_transcript_variant,intron_variant
rs573181235	intron_variant
rs573202121	genic_downstream_transcript_variant,intron_variant
rs573205008	genic_downstream_transcript_variant,intron_variant
rs573206724	intron_variant
rs573289588	intron_variant
rs573291461	upstream_transcript_variant,2KB_upstream_variant,genic_upstream_transcript_variant,intron_variant
rs573331347	genic_downstream_transcript_variant,intron_variant
rs573358625	genic_downstream_transcript_variant,3_prime_UTR_variant,non_coding_transcript_variant
rs573362356	genic_upstream_transcript_variant,intron_variant
rs573389176	genic_downstream_transcript_variant,intron_variant
rs573427683	genic_upstream_transcript_variant,intron_variant
rs573427729	genic_upstream_transcript_variant,intron_variant
rs573444859	genic_downstream_transcript_variant,intron_variant
rs573486887	intron_variant
rs573497782	genic_upstream_transcript_variant,intron_variant
rs573518389	genic_upstream_transcript_variant,intron_variant
rs573571083	genic_downstream_transcript_variant,intron_variant
rs573601571	intron_variant
rs573612778	intron_variant
rs573652957	genic_downstream_transcript_variant,intron_variant
rs573673657	intron_variant,genic_downstream_transcript_variant
rs573735145	intron_variant,genic_downstream_transcript_variant
rs573744428	genic_upstream_transcript_variant,intron_variant
rs573744962	intron_variant,genic_downstream_transcript_variant
rs573754983	intron_variant
rs573757917	intron_variant
rs573806094	intron_variant
rs573857966	intron_variant
rs573871866	genic_upstream_transcript_variant,intron_variant
rs573892433	genic_upstream_transcript_variant,intron_variant
rs573919186	genic_upstream_transcript_variant,intron_variant
rs573968419	intron_variant,genic_downstream_transcript_variant
rs574005170	genic_upstream_transcript_variant,intron_variant
rs574033435	intron_variant,genic_downstream_transcript_variant
rs574048051	intron_variant
rs574073708	intron_variant
rs574118941	intron_variant
rs574139717	intron_variant,genic_downstream_transcript_variant
rs574146572	intron_variant
rs574156340	intron_variant
rs574162070	intron_variant,genic_downstream_transcript_variant
rs574174512	genic_upstream_transcript_variant,intron_variant
rs574181371	intron_variant
rs574187374	genic_upstream_transcript_variant,intron_variant
rs574207921	genic_upstream_transcript_variant,intron_variant
rs574286701	genic_upstream_transcript_variant,intron_variant
rs574292626	intron_variant
rs574309830	intron_variant
rs574339494	genic_upstream_transcript_variant,intron_variant
rs574340806	genic_upstream_transcript_variant,intron_variant
rs574345243	intron_variant
rs574345948	genic_upstream_transcript_variant,intron_variant,upstream_transcript_variant
rs574350881	downstream_transcript_variant,500B_downstream_variant
rs574372059	intron_variant,genic_downstream_transcript_variant
rs574377673	genic_upstream_transcript_variant,intron_variant
rs574393486	genic_upstream_transcript_variant,intron_variant
rs574416910	intron_variant
rs574434721	genic_upstream_transcript_variant,intron_variant,upstream_transcript_variant
rs574459492	intron_variant
rs574470929	intron_variant
rs574508877	genic_upstream_transcript_variant,intron_variant
rs574532537	intron_variant
rs574549350	genic_upstream_transcript_variant,intron_variant
rs574558797	intron_variant
rs574571138	genic_upstream_transcript_variant,intron_variant
rs574588938	intron_variant
rs574603106	intron_variant

rs574604644	genic_upstream_transcript_variant,intron_variant
rs574661581	intron_variant,genic_downstream_transcript_variant
rs574678876	intron_variant
rs574721805	genic_upstream_transcript_variant,intron_variant
rs574732421	genic_upstream_transcript_variant,intron_variant,upstream_transcript_variant,2KB_upstream_variant
rs574738526	intron_variant
rs574744049	intron_variant
rs574748666	3_prime_UTR_variant,non_coding_transcript_variant,genic_downstream_transcript_variant
rs574769607	intron_variant
rs574784356	genic_upstream_transcript_variant,intron_variant
rs574832774	intron_variant
rs574900373	genic_upstream_transcript_variant,intron_variant
rs574936356	missense_variant,non_coding_transcript_variant,genic_downstream_transcript_variant,coding_sequence_variant
rs574975510	genic_upstream_transcript_variant,intron_variant
rs575003180	genic_upstream_transcript_variant,intron_variant
rs575007643	intron_variant
rs575043475	genic_upstream_transcript_variant,intron_variant
rs575049652	intron_variant
rs575062595	intron_variant,genic_downstream_transcript_variant
rs575066344	intron_variant
rs575070113	genic_upstream_transcript_variant,intron_variant,upstream_transcript_variant,2KB_upstream_variant
rs575079293	intron_variant
rs575109043	genic_upstream_transcript_variant,intron_variant
rs575130596	genic_upstream_transcript_variant,intron_variant
rs575131608	intron_variant,genic_downstream_transcript_variant
rs575179735	genic_upstream_transcript_variant,intron_variant
rs575196693	intron_variant
rs575213827	intron_variant
rs575220693	genic_upstream_transcript_variant,intron_variant
rs575237530	intron_variant
rs575292866	intron_variant
rs575311252	intron_variant
rs575343932	intron_variant
rs575380260	intron_variant
rs575380383	intron_variant,5_prime_UTR_variant
rs575399236	intron_variant
rs575419504	intron_variant
rs575446186	genic_upstream_transcript_variant,intron_variant
rs575484022	genic_upstream_transcript_variant,intron_variant
rs575485755	intron_variant
rs575489173	intron_variant
rs575507752	genic_upstream_transcript_variant,intron_variant
rs575514101	intron_variant,genic_downstream_transcript_variant
rs575552625	intron_variant,genic_downstream_transcript_variant
rs575554044	intron_variant
rs575554130	intron_variant
rs575594451	intron_variant,genic_downstream_transcript_variant
rs575596679	intron_variant
rs575629314	intron_variant,genic_downstream_transcript_variant
rs575667775	intron_variant,genic_downstream_transcript_variant
rs575824930	intron_variant
rs575862671	genic_upstream_transcript_variant,intron_variant
rs575953588	intron_variant
rs575989051	intron_variant
rs575989614	genic_upstream_transcript_variant,intron_variant
rs576032137	intron_variant,genic_downstream_transcript_variant
rs576035513	intron_variant,genic_downstream_transcript_variant
rs576043573	intron_variant
rs576063144	genic_upstream_transcript_variant,intron_variant
rs576081949	genic_upstream_transcript_variant,intron_variant
rs576101694	intron_variant
rs576115847	genic_upstream_transcript_variant,intron_variant
rs576122628	intron_variant,genic_downstream_transcript_variant
rs576184179	intron_variant
rs576211472	genic_upstream_transcript_variant,intron_variant
rs576220662	intron_variant
rs576278455	genic_upstream_transcript_variant,intron_variant
rs576289256	genic_upstream_transcript_variant,intron_variant
rs576339696	genic_upstream_transcript_variant,intron_variant
rs576392373	genic_upstream_transcript_variant,intron_variant
rs576448782	genic_upstream_transcript_variant,intron_variant
rs576453691	downstream_transcript_variant,500B_downstream_variant
rs576461271	genic_upstream_transcript_variant,intron_variant
rs576504013	intron_variant

rs576518957	intron_variant,genic_downstream_transcript_variant
rs576527146	intron_variant
rs576564280	intron_variant
rs576568770	intron_variant
rs576575889	intron_variant
rs576581350	intron_variant
rs576585070	genic_upstream_transcript_variant,intron_variant
rs576606062	genic_upstream_transcript_variant,intron_variant
rs576634644	genic_upstream_transcript_variant,intron_variant
rs576673110	genic_upstream_transcript_variant,intron_variant
rs576757793	intron_variant
rs576767989	intron_variant
rs576782990	genic_upstream_transcript_variant,intron_variant
rs576785477	genic_upstream_transcript_variant,intron_variant
rs576821839	intron_variant,genic_downstream_transcript_variant
rs576834589	genic_upstream_transcript_variant,intron_variant
rs576836414	intron_variant
rs576847635	genic_upstream_transcript_variant,intron_variant
rs576870616	genic_upstream_transcript_variant,intron_variant
rs576911053	genic_upstream_transcript_variant,intron_variant
rs576949323	genic_upstream_transcript_variant,intron_variant
rs576981888	genic_upstream_transcript_variant,intron_variant
rs576983482	intron_variant
rs577002609	intron_variant
rs577006830	intron_variant
rs577020158	intron_variant
rs577023937	intron_variant
rs577093432	intron_variant
rs577157545	intron_variant,genic_downstream_transcript_variant
rs577165620	intron_variant
rs577166417	intron_variant
rs577166923	intron_variant
rs577177874	genic_upstream_transcript_variant,intron_variant
rs577179703	genic_upstream_transcript_variant,intron_variant
rs577201178	intron_variant
rs577202790	intron_variant
rs577241923	genic_upstream_transcript_variant,intron_variant
rs577263709	genic_upstream_transcript_variant,intron_variant
rs577297062	genic_upstream_transcript_variant,intron_variant
rs577303897	genic_upstream_transcript_variant,intron_variant
rs577306447	genic_upstream_transcript_variant,intron_variant
rs577341637	genic_upstream_transcript_variant,intron_variant,upstream_transcript_variant,2KB_upstream_variant
rs577346409	genic_upstream_transcript_variant,intron_variant
rs577397101	intron_variant
rs577416382	3_prime_UTR_variant,non_coding_transcript_variant,genic_downstream_transcript_variant
rs577438837	intron_variant
rs577476417	intron_variant
rs577477223	intron_variant,genic_downstream_transcript_variant
rs577487381	genic_upstream_transcript_variant,intron_variant
rs577527271	genic_upstream_transcript_variant,intron_variant
rs577554043	intron_variant
rs577555622	intron_variant
rs577557245	intron_variant
rs577583673	intron_variant
rs577594470	intron_variant
rs577605241	3_prime_UTR_variant,non_coding_transcript_variant,genic_downstream_transcript_variant
rs577711272	genic_upstream_transcript_variant,intron_variant
rs577740223	genic_upstream_transcript_variant,intron_variant
rs577748502	intron_variant
rs577758019	intron_variant
rs577806852	intron_variant,genic_downstream_transcript_variant
rs577825127	intron_variant
rs577860341	intron_variant
rs577875975	intron_variant,genic_downstream_transcript_variant
rs577882321	genic_upstream_transcript_variant,intron_variant
rs577968756	genic_upstream_transcript_variant,intron_variant
rs577972915	genic_upstream_transcript_variant,intron_variant,upstream_transcript_variant
rs577979091	intron_variant
rs577980412	genic_upstream_transcript_variant,intron_variant
rs577996339	intron_variant,genic_downstream_transcript_variant
rs578000565	intron_variant
rs578015809	intron_variant,genic_downstream_transcript_variant
rs578015883	intron_variant,genic_downstream_transcript_variant
rs578049095	genic_upstream_transcript_variant,intron_variant

rs578056540	genic_upstream_transcript_variant,intron_variant
rs578078389	intron_variant
rs578082111	genic_upstream_transcript_variant,intron_variant
rs578103472	intron_variant
rs578150977	genic_upstream_transcript_variant,intron_variant
rs578199996	genic_upstream_transcript_variant,intron_variant,upstream_transcript_variant,2KB_upstream_variant
rs578217943	intron_variant
rs578230532	genic_upstream_transcript_variant,intron_variant
rs578245798	genic_upstream_transcript_variant,intron_variant
rs578246193	genic_upstream_transcript_variant,intron_variant
rs745324745	intron_variant,genic_downstream_transcript_variant
rs745366999	intron_variant,genic_downstream_transcript_variant
rs745368078	genic_upstream_transcript_variant,intron_variant
rs745391152	genic_upstream_transcript_variant,intron_variant,upstream_transcript_variant,2KB_upstream_variant
rs745423991	non_coding_transcript_variant,intron_variant,genic_upstream_transcript_variant,missense_variant,coding_sequence_variant
rs745448709	intron_variant
rs745492071	genic_upstream_transcript_variant,intron_variant
rs745514624	intron_variant,genic_downstream_transcript_variant
rs745516511	genic_upstream_transcript_variant,intron_variant
rs745524785	intron_variant,genic_downstream_transcript_variant
rs745531681	missense_variant,non_coding_transcript_variant,coding_sequence_variant
rs745538300	intron_variant,genic_downstream_transcript_variant
rs745549295	intron_variant,genic_downstream_transcript_variant
rs745564729	intron_variant,genic_downstream_transcript_variant
rs745616260	intron_variant
rs745643473	intron_variant
rs745696924	intron_variant
rs745718484	synonymous_variant,non_coding_transcript_variant,coding_sequence_variant
rs745726656	synonymous_variant,5_prime_UTR_variant,non_coding_transcript_variant,coding_sequence_variant
rs745731122	intron_variant
rs745751463	intron_variant,genic_downstream_transcript_variant
rs745768685	genic_upstream_transcript_variant,intron_variant
rs745810421	intron_variant,genic_downstream_transcript_variant
rs745818057	genic_upstream_transcript_variant,intron_variant
rs745861246	intron_variant
rs745880601	genic_upstream_transcript_variant,intron_variant
rs745940688	genic_upstream_transcript_variant,intron_variant
rs745950412	intron_variant
rs745954717	intron_variant
rs745968500	intron_variant
rs745995913	genic_upstream_transcript_variant,intron_variant
rs746000857	intron_variant
rs746013159	genic_upstream_transcript_variant,intron_variant
rs746016639	genic_upstream_transcript_variant,intron_variant
rs746056678	genic_upstream_transcript_variant,intron_variant
rs746091644	intron_variant,genic_downstream_transcript_variant
rs746093579	intron_variant
rs746131555	intron_variant
rs746146071	intron_variant
rs746172777	intron_variant
rs746249527	intron_variant
rs746251950	intron_variant,genic_downstream_transcript_variant
rs746273927	genic_upstream_transcript_variant,intron_variant
rs746281781	intron_variant
rs746291237	missense_variant,non_coding_transcript_variant,genic_downstream_transcript_variant,coding_sequence_variant
rs746291919	genic_upstream_transcript_variant,intron_variant
rs746295363	non_coding_transcript_variant,intron_variant,genic_upstream_transcript_variant,missense_variant,coding_sequence_variant
rs746334800	missense_variant,non_coding_transcript_variant,coding_sequence_variant
rs746338869	intron_variant
rs746353083	missense_variant,non_coding_transcript_variant,genic_downstream_transcript_variant,coding_sequence_variant
rs746360487	intron_variant,genic_downstream_transcript_variant
rs746379233	genic_upstream_transcript_variant,intron_variant
rs746388251	missense_variant,non_coding_transcript_variant,coding_sequence_variant
rs746413882	3_prime_UTR_variant,non_coding_transcript_variant,genic_downstream_transcript_variant
rs746437327	intron_variant
rs746450854	intron_variant
rs746470357	intron_variant
rs746494770	non_coding_transcript_variant,intron_variant,genic_upstream_transcript_variant,synonymous_variant,coding_sequence_variant
rs746501922	intron_variant
rs746516047	genic_upstream_transcript_variant,intron_variant
rs746545921	genic_upstream_transcript_variant,intron_variant
rs746546046	synonymous_variant,non_coding_transcript_variant,coding_sequence_variant
rs746595540	genic_upstream_transcript_variant,intron_variant
rs746606050	genic_upstream_transcript_variant,intron_variant

rs746669230	intron_variant
rs746684452	genic_upstream_transcript_variant,intron_variant,upstream_transcript_variant,2KB_upstream_variant
rs746689980	intron_variant,genic_downstream_transcript_variant
rs746734953	missense_variant,non_coding_transcript_variant,coding_sequence_variant
rs746760646	intron_variant
rs746761310	intron_variant,genic_downstream_transcript_variant
rs746798601	intron_variant
rs746816156	intron_variant,genic_downstream_transcript_variant
rs746835458	intron_variant,genic_downstream_transcript_variant
rs746854646	genic_upstream_transcript_variant,intron_variant
rs746855633	intron_variant
rs746864274	genic_upstream_transcript_variant,intron_variant
rs746865243	missense_variant,non_coding_transcript_variant,genic_downstream_transcript_variant,coding_sequence_variant
rs746901685	intron_variant,genic_downstream_transcript_variant
rs746906141	intron_variant
rs746930982	genic_upstream_transcript_variant,intron_variant
rs746970798	intron_variant
rs747003224	intron_variant,genic_downstream_transcript_variant
rs747015667	genic_upstream_transcript_variant,intron_variant,upstream_transcript_variant,2KB_upstream_variant
rs747055891	intron_variant,genic_downstream_transcript_variant
rs747089056	intron_variant
rs747107225	intron_variant,genic_downstream_transcript_variant
rs747138607	non_coding_transcript_variant,intron_variant,genic_upstream_transcript_variant,missense_variant,coding_sequence_variant
rs747141434	genic_upstream_transcript_variant,intron_variant
rs747142105	intron_variant
rs747146480	genic_upstream_transcript_variant,intron_variant
rs747153060	intron_variant
rs747190582	genic_upstream_transcript_variant,intron_variant
rs747235459	genic_upstream_transcript_variant,intron_variant
rs747258269	genic_upstream_transcript_variant,intron_variant
rs747301810	genic_upstream_transcript_variant,intron_variant
rs747316358	intron_variant,genic_downstream_transcript_variant
rs747331344	intron_variant
rs747391719	non_coding_transcript_variant,intron_variant,genic_upstream_transcript_variant,missense_variant,coding_sequence_variant
rs747447086	intron_variant
rs747450670	intron_variant
rs747464458	intron_variant
rs747473463	intron_variant
rs747516278	genic_upstream_transcript_variant,intron_variant
rs747528521	synonymous_variant,non_coding_transcript_variant,coding_sequence_variant
rs747538243	3_prime_UTR_variant,non_coding_transcript_variant,genic_downstream_transcript_variant
rs747555073	genic_upstream_transcript_variant,intron_variant
rs747572262	intron_variant,genic_downstream_transcript_variant
rs747606954	genic_upstream_transcript_variant,intron_variant
rs747649577	intron_variant
rs747665246	intron_variant
rs747729085	intron_variant,genic_downstream_transcript_variant
rs747729860	intron_variant
rs747761472	intron_variant
rs747791360	intron_variant
rs747878642	intron_variant
rs747887276	intron_variant,genic_downstream_transcript_variant
rs747891294	genic_upstream_transcript_variant,intron_variant
rs747926452	intron_variant,genic_downstream_transcript_variant
rs747936472	intron_variant
rs747963694	genic_upstream_transcript_variant,intron_variant
rs747982314	missense_variant,non_coding_transcript_variant,coding_sequence_variant
rs748010112	intron_variant,genic_downstream_transcript_variant
rs748013016	intron_variant
rs748035435	intron_variant
rs748042018	genic_upstream_transcript_variant,intron_variant
rs748074774	intron_variant,genic_downstream_transcript_variant
rs748117822	intron_variant
rs748178409	intron_variant,genic_downstream_transcript_variant
rs748207507	intron_variant,genic_downstream_transcript_variant
rs748249800	intron_variant
rs748262827	genic_upstream_transcript_variant,intron_variant
rs748269416	genic_upstream_transcript_variant,intron_variant
rs748277452	genic_upstream_transcript_variant,intron_variant
rs748279701	intron_variant
rs748305839	intron_variant,genic_downstream_transcript_variant
rs748314633	intron_variant
rs748315409	intron_variant
rs748325092	genic_upstream_transcript_variant,intron_variant

rs748328299	genic_upstream_transcript_variant,intron_variant
rs748411634	intron_variant,genic_downstream_transcript_variant
rs748415091	genic_upstream_transcript_variant,intron_variant,upstream_transcript_variant,2KB_upstream_variant
rs748430872	genic_upstream_transcript_variant,intron_variant
rs748436276	intron_variant
rs748447008	intron_variant
rs748494471	intron_variant
rs748547585	intron_variant
rs748628043	intron_variant
rs748629100	genic_upstream_transcript_variant,intron_variant
rs748636241	intron_variant
rs748661147	synonymous_variant,non_coding_transcript_variant,genic_downstream_transcript_variant,coding_sequence_variant
rs748742882	intron_variant,genic_downstream_transcript_variant
rs748756462	intron_variant,genic_downstream_transcript_variant
rs748773382	intron_variant
rs748776466	non_coding_transcript_variant,intron_variant,genic_upstream_transcript_variant,missense_variant,coding_sequence_variant
rs748818807	missense_variant,synonymous_variant,intron_variant,coding_sequence_variant
rs748829868	genic_upstream_transcript_variant,intron_variant
rs748833988	genic_upstream_transcript_variant,intron_variant
rs748841205	genic_upstream_transcript_variant,intron_variant
rs748868626	genic_upstream_transcript_variant,intron_variant
rs748906576	downstream_transcript_variant,500B_downstream_variant
rs748917309	genic_upstream_transcript_variant,intron_variant
rs748922103	intron_variant
rs748923998	genic_upstream_transcript_variant,intron_variant
rs748954119	genic_upstream_transcript_variant,intron_variant
rs748978073	intron_variant,genic_downstream_transcript_variant
rs749013392	intron_variant
rs749022411	genic_upstream_transcript_variant,intron_variant
rs749064648	intron_variant
rs749069628	intron_variant
rs749082043	non_coding_transcript_variant,intron_variant,genic_upstream_transcript_variant,missense_variant,coding_sequence_variant
rs749082857	intron_variant,genic_downstream_transcript_variant
rs749086707	intron_variant,genic_downstream_transcript_variant
rs749099626	genic_upstream_transcript_variant,intron_variant,upstream_transcript_variant,2KB_upstream_variant
rs749120760	intron_variant
rs749121133	intron_variant
rs749122405	non_coding_transcript_variant,intron_variant,genic_upstream_transcript_variant,synonymous_variant,coding_sequence_variant
rs749134628	intron_variant
rs749152789	intron_variant
rs749156725	intron_variant
rs749177488	genic_upstream_transcript_variant,intron_variant
rs749200333	genic_upstream_transcript_variant,intron_variant
rs749253844	intron_variant,genic_downstream_transcript_variant
rs749259118	genic_upstream_transcript_variant,intron_variant
rs749302092	intron_variant
rs749313721	intron_variant
rs749359098	intron_variant
rs749364285	intron_variant
rs749374553	genic_upstream_transcript_variant,intron_variant
rs749395525	intron_variant
rs749405158	genic_upstream_transcript_variant,intron_variant
rs749426056	intron_variant,genic_downstream_transcript_variant
rs749434465	genic_upstream_transcript_variant,intron_variant
rs749435032	genic_upstream_transcript_variant,intron_variant
rs749438942	synonymous_variant,non_coding_transcript_variant,genic_downstream_transcript_variant,coding_sequence_variant
rs749452784	genic_upstream_transcript_variant,intron_variant
rs749472993	genic_upstream_transcript_variant,intron_variant
rs749481056	intron_variant,genic_downstream_transcript_variant
rs749533070	intron_variant
rs749537778	intron_variant
rs749577114	genic_upstream_transcript_variant,intron_variant
rs749580508	intron_variant
rs749588045	intron_variant,genic_downstream_transcript_variant
rs749598612	intron_variant
rs749625939	intron_variant,genic_downstream_transcript_variant
rs749648392	intron_variant
rs749650711	intron_variant,genic_upstream_transcript_variant
rs749702072	intron_variant,genic_upstream_transcript_variant,2KB_upstream_variant,upstream_transcript_variant
rs749736139	coding_sequence_variant,missense_variant,non_coding_transcript_variant
rs749744230	intron_variant,genic_upstream_transcript_variant
rs749749352	intron_variant,genic_upstream_transcript_variant
rs749766519	intron_variant,genic_downstream_transcript_variant
rs749777197	intron_variant,genic_downstream_transcript_variant

rs749821599	3_prime_UTR_variant,genic_downstream_transcript_variant,non_coding_transcript_variant
rs749848254	intron_variant,genic_downstream_transcript_variant
rs749848531	intron_variant,genic_upstream_transcript_variant
rs749876472	intron_variant
rs749903262	intron_variant,genic_downstream_transcript_variant
rs749927665	intron_variant,genic_downstream_transcript_variant
rs749934142	intron_variant
rs750025736	intron_variant
rs750055538	intron_variant,genic_downstream_transcript_variant
rs750085561	intron_variant
rs750144857	intron_variant,genic_upstream_transcript_variant
rs750155301	intron_variant
rs750161206	intron_variant,genic_downstream_transcript_variant
rs750162418	intron_variant,genic_upstream_transcript_variant
rs750172603	intron_variant,genic_upstream_transcript_variant
rs750188400	genic_upstream_transcript_variant,synonymous_variant,intron_variant,non_coding_transcript_variant,coding_sequence_variant
rs750198737	intron_variant,genic_downstream_transcript_variant
rs750200033	intron_variant,genic_upstream_transcript_variant
rs750225996	intron_variant,genic_upstream_transcript_variant
rs750238911	intron_variant
rs750246464	intron_variant,genic_upstream_transcript_variant
rs750249902	intron_variant,genic_downstream_transcript_variant
rs750272033	intron_variant,genic_upstream_transcript_variant
rs750276956	coding_sequence_variant,missense_variant,non_coding_transcript_variant
rs750318380	intron_variant
rs750386119	intron_variant
rs750407774	intron_variant,genic_upstream_transcript_variant,2KB_upstream_variant,upstream_transcript_variant
rs750421436	intron_variant,genic_downstream_transcript_variant
rs750427793	intron_variant,genic_downstream_transcript_variant
rs750431672	intron_variant,genic_upstream_transcript_variant
rs750451385	intron_variant,genic_downstream_transcript_variant
rs750463659	intron_variant
rs750481539	intron_variant,genic_upstream_transcript_variant
rs750486793	intron_variant,genic_upstream_transcript_variant
rs750499702	intron_variant,genic_downstream_transcript_variant
rs750506239	intron_variant,genic_downstream_transcript_variant
rs750511986	intron_variant,genic_downstream_transcript_variant
rs750514432	intron_variant,genic_upstream_transcript_variant
rs750520213	intron_variant
rs750565007	intron_variant,genic_downstream_transcript_variant
rs750584357	intron_variant
rs750597048	intron_variant
rs750629072	intron_variant,genic_upstream_transcript_variant
rs750665552	intron_variant,genic_upstream_transcript_variant
rs750763063	intron_variant
rs750763620	intron_variant
rs750792697	coding_sequence_variant,missense_variant,non_coding_transcript_variant
rs750811600	3_prime_UTR_variant,genic_downstream_transcript_variant,non_coding_transcript_variant
rs750813608	downstream_transcript_variant,500B_downstream_variant
rs750820405	genic_upstream_transcript_variant,synonymous_variant,intron_variant,non_coding_transcript_variant,coding_sequence_variant
rs750821229	intron_variant
rs750858911	coding_sequence_variant,genic_downstream_transcript_variant,non_coding_transcript_variant,synonymous_variant
rs750873058	intron_variant,genic_upstream_transcript_variant
rs750874023	intron_variant
rs750895431	intron_variant,genic_upstream_transcript_variant
rs750927198	intron_variant,genic_upstream_transcript_variant
rs750951259	intron_variant,genic_downstream_transcript_variant
rs750956494	intron_variant,genic_downstream_transcript_variant
rs750973154	intron_variant
rs751041644	intron_variant
rs751057241	intron_variant,genic_downstream_transcript_variant
rs751079775	intron_variant
rs751090845	intron_variant,genic_downstream_transcript_variant
rs751101285	coding_sequence_variant,intron_variant,missense_variant
rs751118933	intron_variant
rs751138529	intron_variant,genic_upstream_transcript_variant
rs751139564	intron_variant,genic_upstream_transcript_variant
rs751140286	intron_variant,genic_upstream_transcript_variant
rs751159881	intron_variant,genic_upstream_transcript_variant
rs751232626	intron_variant,genic_upstream_transcript_variant
rs751239059	missense_variant,genic_upstream_transcript_variant,intron_variant,non_coding_transcript_variant,coding_sequence_variant
rs751258225	intron_variant
rs751262367	intron_variant,genic_upstream_transcript_variant
rs751267613	intron_variant,genic_upstream_transcript_variant,2KB_upstream_variant,upstream_transcript_variant

rs751276578	intron_variant,genic_downstream_transcript_variant
rs751291529	intron_variant,genic_downstream_transcript_variant
rs751325832	intron_variant,genic_downstream_transcript_variant
rs751368503	intron_variant,genic_upstream_transcript_variant
rs751375580	intron_variant,genic_downstream_transcript_variant,downstream_transcript_variant
rs751389705	intron_variant
rs751421114	intron_variant
rs751423676	intron_variant,genic_upstream_transcript_variant
rs751484088	intron_variant,genic_upstream_transcript_variant
rs751493573	intron_variant
rs751495627	intron_variant
rs751521116	intron_variant,genic_upstream_transcript_variant
rs751541593	intron_variant,genic_upstream_transcript_variant
rs751555216	intron_variant,genic_upstream_transcript_variant
rs751570611	intron_variant,genic_downstream_transcript_variant
rs751572663	missense_variant,genic_upstream_transcript_variant,intron_variant,non_coding_transcript_variant,coding_sequence_variant,stop_gained
rs751586503	intron_variant,genic_downstream_transcript_variant
rs751613983	intron_variant
rs751721014	intron_variant
rs751721710	intron_variant,genic_upstream_transcript_variant
rs751729200	coding_sequence_variant,missense_variant,genic_downstream_transcript_variant,non_coding_transcript_variant
rs751742182	intron_variant
rs751758734	intron_variant,genic_upstream_transcript_variant
rs751781411	intron_variant,genic_upstream_transcript_variant
rs751784236	3_prime_UTR_variant,genic_downstream_transcript_variant,non_coding_transcript_variant
rs751785708	intron_variant,genic_downstream_transcript_variant
rs751829190	intron_variant
rs751881668	intron_variant
rs751907602	coding_sequence_variant,missense_variant,non_coding_transcript_variant
rs751909561	intron_variant
rs751909630	coding_sequence_variant,missense_variant,genic_downstream_transcript_variant,non_coding_transcript_variant
rs751942482	intron_variant
rs751945581	intron_variant
rs751955874	intron_variant,genic_downstream_transcript_variant
rs751959253	intron_variant,genic_downstream_transcript_variant
rs751962456	intron_variant
rs751962539	intron_variant,genic_downstream_transcript_variant,downstream_transcript_variant
rs751972395	intron_variant
rs751983533	intron_variant
rs752021129	intron_variant,genic_upstream_transcript_variant
rs752042664	intron_variant,genic_upstream_transcript_variant
rs752055485	missense_variant,genic_upstream_transcript_variant,intron_variant,non_coding_transcript_variant,coding_sequence_variant
rs752080939	intron_variant
rs752105100	intron_variant,genic_upstream_transcript_variant
rs752121634	intron_variant
rs752179199	intron_variant,genic_downstream_transcript_variant
rs752219165	intron_variant,genic_downstream_transcript_variant
rs752229925	intron_variant
rs752234308	intron_variant
rs752235438	intron_variant
rs752243890	intron_variant,genic_downstream_transcript_variant
rs752244121	intron_variant
rs752246483	intron_variant
rs752269312	intron_variant,genic_upstream_transcript_variant
rs752281264	coding_sequence_variant,missense_variant,non_coding_transcript_variant,5_prime_UTR_variant
rs752290576	intron_variant
rs752299863	intron_variant,genic_upstream_transcript_variant
rs752327398	intron_variant
rs752349336	genic_upstream_transcript_variant,intron_variant,non_coding_transcript_variant,coding_sequence_variant,frameshift_variant
rs752436091	intron_variant,genic_downstream_transcript_variant
rs752446675	intron_variant,genic_upstream_transcript_variant
rs752448982	intron_variant,genic_downstream_transcript_variant
rs752452201	intron_variant,genic_downstream_transcript_variant
rs752480046	intron_variant
rs752484484	intron_variant
rs752495532	missense_variant,genic_upstream_transcript_variant,intron_variant,non_coding_transcript_variant,coding_sequence_variant
rs752515251	intron_variant
rs752516881	intron_variant
rs752531321	intron_variant
rs752535640	coding_sequence_variant,non_coding_transcript_variant,synonymous_variant
rs752576180	intron_variant,genic_upstream_transcript_variant
rs752593587	coding_sequence_variant,genic_downstream_transcript_variant,non_coding_transcript_variant,synonymous_variant
rs752610969	intron_variant,genic_upstream_transcript_variant
rs752618630	intron_variant,genic_downstream_transcript_variant,downstream_transcript_variant

rs752631943	intron_variant,genic_upstream_transcript_variant
rs752678356	intron_variant,genic_upstream_transcript_variant
rs752683210	intron_variant,genic_upstream_transcript_variant
rs752686328	intron_variant,genic_upstream_transcript_variant
rs752740791	coding_sequence_variant,missense_variant,non_coding_transcript_variant
rs752778502	intron_variant,genic_downstream_transcript_variant
rs752779566	intron_variant,genic_upstream_transcript_variant
rs752823191	3_prime_UTR_variant,genic_downstream_transcript_variant,non_coding_transcript_variant
rs752837137	intron_variant,genic_downstream_transcript_variant
rs752850047	intron_variant,genic_downstream_transcript_variant,downstream_transcript_variant
rs752866538	intron_variant,genic_upstream_transcript_variant
rs752898916	intron_variant
rs752921667	intron_variant,genic_upstream_transcript_variant
rs752937307	intron_variant
rs752948862	intron_variant,genic_upstream_transcript_variant
rs752977785	intron_variant,genic_upstream_transcript_variant
rs753000586	intron_variant
rs753009009	intron_variant
rs753027254	intron_variant,genic_upstream_transcript_variant
rs753036415	intron_variant,genic_downstream_transcript_variant
rs753091019	coding_sequence_variant,missense_variant,non_coding_transcript_variant
rs753102751	intron_variant,genic_upstream_transcript_variant
rs753125607	intron_variant,genic_upstream_transcript_variant
rs753135733	intron_variant,genic_downstream_transcript_variant
rs753136699	intron_variant
rs753190193	intron_variant
rs753203317	intron_variant,genic_downstream_transcript_variant
rs753267370	intron_variant,genic_downstream_transcript_variant
rs753271673	intron_variant,genic_upstream_transcript_variant
rs753291080	intron_variant,genic_upstream_transcript_variant,2KB_upstream_variant,upstream_transcript_variant
rs753309112	genic_upstream_transcript_variant,synonymous_variant,intron_variant,non_coding_transcript_variant,coding_sequence_variant
rs753311682	intron_variant
rs753347670	intron_variant,genic_downstream_transcript_variant
rs753349495	intron_variant,genic_upstream_transcript_variant
rs753356451	intron_variant
rs753362371	intron_variant
rs753400437	intron_variant,genic_upstream_transcript_variant
rs753409176	intron_variant,genic_upstream_transcript_variant
rs753426615	intron_variant
rs753557906	intron_variant,genic_upstream_transcript_variant
rs753583632	intron_variant,genic_downstream_transcript_variant
rs753588663	intron_variant,genic_upstream_transcript_variant,2KB_upstream_variant,upstream_transcript_variant
rs753615961	intron_variant
rs753621571	intron_variant
rs753654815	intron_variant
rs753693782	intron_variant,genic_upstream_transcript_variant
rs753694421	intron_variant,genic_upstream_transcript_variant
rs753713629	coding_sequence_variant,missense_variant,non_coding_transcript_variant
rs753722970	intron_variant,genic_downstream_transcript_variant
rs753752972	intron_variant,genic_downstream_transcript_variant
rs753759661	intron_variant,genic_upstream_transcript_variant
rs753762177	missense_variant,genic_upstream_transcript_variant,intron_variant,non_coding_transcript_variant,coding_sequence_variant,stop_gained
rs753783838	intron_variant,genic_downstream_transcript_variant
rs753836275	intron_variant,genic_downstream_transcript_variant
rs753877026	intron_variant,genic_downstream_transcript_variant
rs753889604	intron_variant
rs753911898	coding_sequence_variant,missense_variant,non_coding_transcript_variant
rs753934337	intron_variant,genic_upstream_transcript_variant
rs753944287	3_prime_UTR_variant,genic_downstream_transcript_variant,non_coding_transcript_variant
rs753979601	intron_variant,genic_downstream_transcript_variant
rs753987432	intron_variant,genic_upstream_transcript_variant
rs754011564	intron_variant
rs754017864	intron_variant
rs754047456	intron_variant
rs754083112	intron_variant
rs754103262	intron_variant
rs754106906	coding_sequence_variant,missense_variant,genic_downstream_transcript_variant,non_coding_transcript_variant
rs754148023	intron_variant,genic_upstream_transcript_variant
rs754179332	3_prime_UTR_variant,genic_downstream_transcript_variant,non_coding_transcript_variant
rs754198809	intron_variant
rs754229793	intron_variant
rs754240211	intron_variant
rs754247965	intron_variant
rs754251596	intron_variant,genic_upstream_transcript_variant

rs754272780	intron_variant,genic_upstream_transcript_variant
rs754276218	intron_variant,genic_upstream_transcript_variant
rs754321742	intron_variant,genic_upstream_transcript_variant
rs754383158	intron_variant,genic_downstream_transcript_variant
rs754389367	intron_variant
rs754415763	intron_variant
rs754418396	intron_variant
rs754469552	intron_variant,genic_downstream_transcript_variant
rs754474067	intron_variant,genic_upstream_transcript_variant
rs754505927	intron_variant
rs754512854	intron_variant
rs754534808	intron_variant,genic_downstream_transcript_variant
rs754586331	intron_variant
rs754643525	intron_variant,genic_downstream_transcript_variant
rs754648829	intron_variant
rs754680405	intron_variant
rs754700435	intron_variant,genic_downstream_transcript_variant
rs754706203	intron_variant,genic_upstream_transcript_variant
rs754719339	intron_variant,genic_upstream_transcript_variant
rs754720043	intron_variant
rs754762636	intron_variant,genic_upstream_transcript_variant
rs754775087	intron_variant
rs754818565	intron_variant,genic_upstream_transcript_variant
rs754820465	genic_upstream_transcript_variant,synonymous_variant,intron_variant,non_coding_transcript_variant,coding_sequence_variant
rs754825675	intron_variant,genic_upstream_transcript_variant
rs754880183	intron_variant,genic_downstream_transcript_variant
rs754938238	intron_variant,genic_upstream_transcript_variant
rs754950066	intron_variant
rs754953870	intron_variant,genic_downstream_transcript_variant
rs755000162	3_prime_UTR_variant,genic_downstream_transcript_variant,non_coding_transcript_variant
rs755059519	intron_variant
rs755109343	intron_variant,genic_downstream_transcript_variant
rs755115906	intron_variant
rs755129209	intron_variant
rs755150048	intron_variant,genic_upstream_transcript_variant
rs755150738	intron_variant,genic_downstream_transcript_variant
rs755194593	coding_sequence_variant,missense_variant,non_coding_transcript_variant
rs755210486	intron_variant,genic_upstream_transcript_variant
rs755216488	intron_variant
rs755234289	intron_variant,genic_downstream_transcript_variant
rs755260834	intron_variant,genic_upstream_transcript_variant
rs755323728	intron_variant,genic_upstream_transcript_variant
rs755336603	intron_variant,genic_upstream_transcript_variant
rs755350357	intron_variant
rs755353954	intron_variant,genic_upstream_transcript_variant
rs755359106	intron_variant,genic_downstream_transcript_variant
rs755382741	intron_variant
rs755409392	intron_variant,genic_upstream_transcript_variant
rs755411892	intron_variant,genic_upstream_transcript_variant
rs755438694	intron_variant
rs755467912	intron_variant,genic_upstream_transcript_variant
rs755470995	coding_sequence_variant,non_coding_transcript_variant,synonymous_variant
rs755481360	intron_variant,genic_upstream_transcript_variant
rs755487203	coding_sequence_variant,missense_variant,non_coding_transcript_variant
rs755506358	intron_variant
rs755533373	intron_variant
rs755535168	intron_variant,genic_downstream_transcript_variant
rs755557635	intron_variant
rs755576622	intron_variant,genic_upstream_transcript_variant
rs755627167	intron_variant,genic_downstream_transcript_variant
rs755641157	intron_variant
rs755641668	intron_variant
rs755694349	intron_variant
rs755709342	intron_variant,genic_upstream_transcript_variant
rs755734829	intron_variant,genic_upstream_transcript_variant
rs755765275	intron_variant
rs755802076	intron_variant,genic_upstream_transcript_variant
rs755816605	coding_sequence_variant,intron_variant,synonymous_variant
rs755821605	intron_variant,genic_downstream_transcript_variant
rs755822176	intron_variant
rs755827594	intron_variant,genic_upstream_transcript_variant
rs755838189	intron_variant,genic_upstream_transcript_variant
rs755838370	intron_variant,genic_downstream_transcript_variant
rs755852535	intron_variant,genic_upstream_transcript_variant

rs755897024	intron_variant
rs755972463	intron_variant,genic_upstream_transcript_variant
rs756000510	intron_variant,genic_upstream_transcript_variant
rs756005576	intron_variant,genic_downstream_transcript_variant,downstream_transcript_variant
rs756036364	intron_variant,genic_downstream_transcript_variant
rs756042406	intron_variant
rs756043598	intron_variant
rs756086234	3_prime_UTR_variant,genic_downstream_transcript_variant,non_coding_transcript_variant
rs756156682	intron_variant,genic_downstream_transcript_variant
rs756158949	intron_variant,genic_downstream_transcript_variant
rs756179880	intron_variant,genic_upstream_transcript_variant
rs756185039	intron_variant,genic_upstream_transcript_variant
rs756189116	intron_variant,genic_upstream_transcript_variant
rs756198003	intron_variant,genic_upstream_transcript_variant
rs756207703	intron_variant
rs756209410	coding_sequence_variant,missense_variant,non_coding_transcript_variant
rs756225840	intron_variant
rs756253170	intron_variant,genic_downstream_transcript_variant
rs756277216	intron_variant
rs756318657	intron_variant,genic_downstream_transcript_variant
rs756364068	intron_variant,genic_downstream_transcript_variant
rs756396862	intron_variant,genic_upstream_transcript_variant
rs756403758	intron_variant
rs756436546	intron_variant,genic_downstream_transcript_variant
rs756440095	intron_variant,genic_upstream_transcript_variant
rs756484908	intron_variant,genic_upstream_transcript_variant
rs756536563	intron_variant
rs756538415	intron_variant,genic_upstream_transcript_variant
rs756554970	intron_variant
rs756600973	intron_variant
rs756603223	intron_variant
rs756610496	intron_variant
rs756703407	intron_variant
rs756703567	intron_variant,genic_downstream_transcript_variant
rs756758022	intron_variant,genic_upstream_transcript_variant
rs756777779	intron_variant
rs756828607	intron_variant
rs756856284	missense_variant,genic_upstream_transcript_variant,intron_variant,non_coding_transcript_variant,coding_sequence_variant
rs756911444	genic_upstream_transcript_variant,synonymous_variant,intron_variant,non_coding_transcript_variant,coding_sequence_variant
rs756918159	intron_variant
rs756923140	intron_variant,genic_upstream_transcript_variant
rs756957004	coding_sequence_variant,genic_downstream_transcript_variant,non_coding_transcript_variant,synonymous_variant
rs756965131	intron_variant,genic_downstream_transcript_variant
rs756971766	intron_variant,genic_upstream_transcript_variant,2KB_upstream_variant,upstream_transcript_variant
rs756986876	intron_variant,genic_upstream_transcript_variant
rs757001661	intron_variant
rs757006484	intron_variant
rs757022316	intron_variant,genic_downstream_transcript_variant
rs757100386	intron_variant,genic_upstream_transcript_variant,2KB_upstream_variant,upstream_transcript_variant
rs757111476	coding_sequence_variant,missense_variant,non_coding_transcript_variant
rs757166437	splice_donor_variant
rs757210861	intron_variant
rs757217266	intron_variant,genic_upstream_transcript_variant
rs757217661	intron_variant,genic_upstream_transcript_variant
rs757250888	intron_variant,genic_upstream_transcript_variant
rs757294942	intron_variant
rs757296777	coding_sequence_variant,non_coding_transcript_variant,synonymous_variant
rs757307335	intron_variant
rs757319358	intron_variant
rs757333718	intron_variant,genic_upstream_transcript_variant
rs757365058	intron_variant,genic_upstream_transcript_variant
rs757405950	intron_variant,genic_upstream_transcript_variant
rs757413061	intron_variant
rs757424541	intron_variant,genic_upstream_transcript_variant
rs757448894	intron_variant
rs757455983	intron_variant
rs757520538	intron_variant,genic_downstream_transcript_variant
rs757521023	3_prime_UTR_variant,genic_downstream_transcript_variant,non_coding_transcript_variant
rs757526105	intron_variant,genic_upstream_transcript_variant
rs757592643	3_prime_UTR_variant,genic_downstream_transcript_variant,non_coding_transcript_variant
rs757614947	intron_variant
rs757665813	intron_variant
rs757722408	intron_variant,genic_upstream_transcript_variant
rs757740450	intron_variant

rs757750209	3_prime_UTR_variant,genic_downstream_transcript_variant,non_coding_transcript_variant
rs757753572	intron_variant,genic_downstream_transcript_variant
rs757759704	coding_sequence_variant,missense_variant,non_coding_transcript_variant
rs757778158	intron_variant
rs757781542	intron_variant
rs757811101	coding_sequence_variant,missense_variant,non_coding_transcript_variant
rs757837264	intron_variant,genic_upstream_transcript_variant
rs757839238	intron_variant
rs757867751	intron_variant,genic_downstream_transcript_variant
rs757883444	intron_variant,genic_upstream_transcript_variant,2KB_upstream_variant,upstream_transcript_variant
rs757919874	intron_variant
rs757978409	intron_variant,genic_upstream_transcript_variant
rs758018541	intron_variant,genic_downstream_transcript_variant
rs758046215	intron_variant,5_prime_UTR_variant
rs758048261	intron_variant
rs758065083	intron_variant,genic_upstream_transcript_variant,2KB_upstream_variant,upstream_transcript_variant
rs758123151	coding_sequence_variant,missense_variant,genic_downstream_transcript_variant,non_coding_transcript_variant
rs758137419	intron_variant,genic_upstream_transcript_variant
rs758153966	intron_variant
rs758184735	intron_variant
rs758208761	intron_variant,genic_upstream_transcript_variant,2KB_upstream_variant,upstream_transcript_variant
rs758209474	intron_variant,genic_upstream_transcript_variant
rs758230781	intron_variant,genic_downstream_transcript_variant
rs758236038	intron_variant,genic_upstream_transcript_variant
rs758242168	intron_variant,genic_downstream_transcript_variant
rs758246482	intron_variant
rs758298344	intron_variant
rs758299724	coding_sequence_variant,missense_variant,non_coding_transcript_variant
rs758369474	intron_variant,genic_upstream_transcript_variant,2KB_upstream_variant,upstream_transcript_variant
rs758381357	intron_variant
rs758477366	intron_variant,genic_upstream_transcript_variant
rs758499914	intron_variant,genic_upstream_transcript_variant
rs758511772	intron_variant,genic_upstream_transcript_variant
rs758565332	coding_sequence_variant,missense_variant,non_coding_transcript_variant
rs758603017	intron_variant,genic_upstream_transcript_variant
rs758616831	coding_sequence_variant,missense_variant,non_coding_transcript_variant
rs758691199	intron_variant
rs758706999	intron_variant
rs758711808	intron_variant
rs758717390	genic_upstream_transcript_variant,intron_variant
rs758765878	genic_upstream_transcript_variant,intron_variant
rs758810596	genic_upstream_transcript_variant,intron_variant
rs758831759	genic_upstream_transcript_variant,intron_variant
rs758862874	genic_upstream_transcript_variant,intron_variant,non_coding_transcript_variant,coding_sequence_variant,missense_variant
rs758868205	genic_upstream_transcript_variant,intron_variant,splice_acceptor_variant
rs758889512	genic_upstream_transcript_variant,intron_variant
rs758892892	intron_variant,genic_downstream_transcript_variant
rs758900174	intron_variant
rs758902071	non_coding_transcript_variant,genic_downstream_transcript_variant,synonymous_variant,coding_sequence_variant
rs758912327	genic_upstream_transcript_variant,intron_variant
rs758949656	non_coding_transcript_variant,genic_downstream_transcript_variant,3_prime_UTR_variant
rs759006869	genic_upstream_transcript_variant,intron_variant
rs759020611	intron_variant
rs759034645	genic_upstream_transcript_variant,intron_variant
rs759050445	intron_variant,genic_downstream_transcript_variant
rs759074353	genic_upstream_transcript_variant,intron_variant
rs759146083	intron_variant,genic_downstream_transcript_variant
rs759151928	intron_variant
rs759196676	genic_upstream_transcript_variant,intron_variant
rs759224383	genic_upstream_transcript_variant,intron_variant
rs759248192	intron_variant
rs759259560	intron_variant
rs759326927	genic_upstream_transcript_variant,intron_variant
rs759372620	intron_variant
rs759415024	genic_upstream_transcript_variant,intron_variant
rs759429097	intron_variant
rs759467172	intron_variant
rs759492209	intron_variant,genic_downstream_transcript_variant
rs759512297	non_coding_transcript_variant,genic_downstream_transcript_variant,3_prime_UTR_variant
rs759577935	genic_upstream_transcript_variant,intron_variant
rs759578562	genic_upstream_transcript_variant,intron_variant
rs759580025	intron_variant,genic_downstream_transcript_variant
rs759592653	intron_variant
rs759601762	intron_variant

rs759602956	genic_upstream_transcript_variant,intron_variant,synonymous_variant,non_coding_transcript_variant,coding_sequence_variant
rs759603561	intron_variant
rs759644071	genic_upstream_transcript_variant,intron_variant
rs759653192	non_coding_transcript_variant,genic_downstream_transcript_variant,3_prime_UTR_variant
rs759725554	intron_variant,genic_downstream_transcript_variant
rs759726534	intron_variant,genic_downstream_transcript_variant
rs759733340	genic_upstream_transcript_variant,intron_variant
rs759814382	intron_variant,genic_downstream_transcript_variant
rs759815968	intron_variant
rs759816115	intron_variant
rs759821959	intron_variant
rs759849443	intron_variant
rs759857869	intron_variant
rs759884389	intron_variant
rs759930796	intron_variant
rs759960697	intron_variant
rs759987500	genic_upstream_transcript_variant,intron_variant
rs760008942	genic_upstream_transcript_variant,intron_variant
rs760010863	genic_upstream_transcript_variant,intron_variant
rs760013316	intron_variant
rs760014990	intron_variant,genic_downstream_transcript_variant
rs760040735	genic_upstream_transcript_variant,intron_variant
rs760052842	intron_variant
rs760091049	genic_upstream_transcript_variant,intron_variant
rs760098507	intron_variant
rs760098700	intron_variant,genic_downstream_transcript_variant
rs760146587	intron_variant
rs760153701	non_coding_transcript_variant,genic_downstream_transcript_variant,coding_sequence_variant,missense_variant
rs760191152	genic_upstream_transcript_variant,intron_variant,upstream_transcript_variant,2KB_upstream_variant
rs760195972	intron_variant
rs760224921	genic_upstream_transcript_variant,intron_variant,upstream_transcript_variant,2KB_upstream_variant
rs760238138	intron_variant,genic_downstream_transcript_variant
rs760253336	genic_upstream_transcript_variant,intron_variant
rs760284924	intron_variant,genic_downstream_transcript_variant
rs760339018	non_coding_transcript_variant,genic_downstream_transcript_variant,coding_sequence_variant,missense_variant
rs760350427	intron_variant,genic_downstream_transcript_variant
rs760373751	intron_variant
rs760375783	genic_upstream_transcript_variant,intron_variant
rs760413666	intron_variant
rs760519841	genic_upstream_transcript_variant,intron_variant
rs760581237	genic_upstream_transcript_variant,intron_variant
rs760581284	genic_upstream_transcript_variant,intron_variant
rs760582864	intron_variant
rs760609715	non_coding_transcript_variant,synonymous_variant,coding_sequence_variant
rs760617164	intron_variant
rs760646861	intron_variant
rs760663148	intron_variant,coding_sequence_variant,missense_variant
rs760664725	intron_variant
rs760706250	genic_upstream_transcript_variant,intron_variant
rs760718962	intron_variant,genic_downstream_transcript_variant
rs760727137	genic_upstream_transcript_variant,intron_variant,synonymous_variant,non_coding_transcript_variant,coding_sequence_variant,missense_variant
rs760727499	intron_variant,genic_downstream_transcript_variant
rs760728977	intron_variant
rs760784337	intron_variant
rs760833700	intron_variant
rs760838791	intron_variant
rs760843853	intron_variant
rs760868842	intron_variant
rs760898456	genic_upstream_transcript_variant,intron_variant
rs760935013	intron_variant,genic_downstream_transcript_variant
rs760943254	intron_variant,genic_downstream_transcript_variant
rs760953555	intron_variant
rs760960304	intron_variant,genic_downstream_transcript_variant
rs760984193	genic_upstream_transcript_variant,intron_variant,upstream_transcript_variant,2KB_upstream_variant
rs761022373	genic_upstream_transcript_variant,intron_variant
rs761043122	5_prime_UTR_variant,non_coding_transcript_variant,coding_sequence_variant,missense_variant
rs761045329	genic_upstream_transcript_variant,intron_variant
rs761059407	intron_variant,genic_downstream_transcript_variant
rs761076497	intron_variant
rs761110329	intron_variant
rs761114112	intron_variant
rs761132934	intron_variant
rs761141143	genic_upstream_transcript_variant,intron_variant
rs761144580	intron_variant

rs761144864	genic_upstream_transcript_variant,intron_variant
rs761162957	non_coding_transcript_variant,genic_downstream_transcript_variant,synonymous_variant,coding_sequence_variant
rs761164322	genic_upstream_transcript_variant,intron_variant
rs761218900	intron_variant,genic_downstream_transcript_variant
rs761227260	intron_variant
rs761237537	non_coding_transcript_variant,genic_downstream_transcript_variant,synonymous_variant,coding_sequence_variant
rs761256009	intron_variant
rs761295320	genic_upstream_transcript_variant,intron_variant
rs761305394	intron_variant,frameshift_variant,coding_sequence_variant
rs761323026	genic_upstream_transcript_variant,intron_variant
rs761336829	intron_variant
rs761346772	genic_upstream_transcript_variant,intron_variant
rs761354855	genic_upstream_transcript_variant,intron_variant,non_coding_transcript_variant,coding_sequence_variant,missense_variant
rs761366196	intron_variant,genic_downstream_transcript_variant
rs761404310	intron_variant
rs761419338	intron_variant,genic_downstream_transcript_variant
rs761529350	intron_variant
rs761551984	genic_upstream_transcript_variant,intron_variant
rs761558546	genic_upstream_transcript_variant,intron_variant
rs761679634	genic_upstream_transcript_variant,intron_variant
rs761725546	genic_upstream_transcript_variant,intron_variant
rs761727097	non_coding_transcript_variant,coding_sequence_variant,missense_variant
rs761753816	genic_upstream_transcript_variant,intron_variant
rs761807652	intron_variant
rs761812322	intron_variant
rs761830410	intron_variant,genic_downstream_transcript_variant
rs761841081	intron_variant
rs761858361	intron_variant
rs761863145	intron_variant,genic_downstream_transcript_variant
rs761863605	intron_variant
rs761865581	genic_upstream_transcript_variant,intron_variant
rs761877064	intron_variant
rs761884841	intron_variant,genic_downstream_transcript_variant
rs761916998	genic_upstream_transcript_variant,intron_variant
rs761951558	intron_variant
rs762004763	non_coding_transcript_variant,coding_sequence_variant,missense_variant
rs762010930	intron_variant,genic_downstream_transcript_variant
rs762033653	genic_upstream_transcript_variant,intron_variant
rs762035743	genic_upstream_transcript_variant,intron_variant
rs762037673	intron_variant
rs762057806	intron_variant
rs762059386	genic_upstream_transcript_variant,intron_variant,non_coding_transcript_variant,coding_sequence_variant,missense_variant
rs762069973	intron_variant
rs762101350	intron_variant,genic_downstream_transcript_variant
rs762147603	intron_variant,genic_downstream_transcript_variant
rs762160906	genic_upstream_transcript_variant,intron_variant
rs762167152	genic_upstream_transcript_variant,intron_variant
rs762170557	intron_variant,genic_downstream_transcript_variant
rs762175278	intron_variant
rs762202581	intron_variant
rs762218595	non_coding_transcript_variant,genic_downstream_transcript_variant,synonymous_variant,coding_sequence_variant
rs762239246	genic_upstream_transcript_variant,intron_variant
rs762253815	genic_upstream_transcript_variant,intron_variant,non_coding_transcript_variant,coding_sequence_variant,missense_variant
rs762256425	intron_variant
rs762270997	intron_variant
rs762317059	genic_upstream_transcript_variant,intron_variant,upstream_transcript_variant,2KB_upstream_variant
rs762389147	non_coding_transcript_variant,coding_sequence_variant,missense_variant
rs762397963	intron_variant,genic_downstream_transcript_variant
rs762434656	genic_upstream_transcript_variant,intron_variant
rs762447442	genic_upstream_transcript_variant,intron_variant
rs762463522	genic_upstream_transcript_variant,intron_variant
rs762464302	intron_variant
rs762466945	intron_variant,genic_downstream_transcript_variant
rs762468061	intron_variant
rs762487154	intron_variant
rs762496200	intron_variant,genic_downstream_transcript_variant
rs762511653	genic_upstream_transcript_variant,intron_variant
rs762514890	intron_variant
rs762538423	intron_variant
rs762545251	intron_variant
rs762559626	intron_variant
rs762590681	intron_variant
rs762596260	intron_variant,genic_downstream_transcript_variant
rs762601307	intron_variant,genic_downstream_transcript_variant

rs762601535	intron_variant
rs762607082	genic_upstream_transcript_variant,intron_variant
rs762633614	intron_variant,genic_downstream_transcript_variant,downstream_transcript_variant
rs762649571	genic_upstream_transcript_variant,intron_variant
rs762679588	intron_variant
rs762806877	intron_variant
rs762818113	genic_upstream_transcript_variant,intron_variant
rs762883310	genic_upstream_transcript_variant,intron_variant
rs762895764	intron_variant,genic_downstream_transcript_variant
rs762908812	genic_upstream_transcript_variant,intron_variant,upstream_transcript_variant,2KB_upstream_variant
rs762916873	intron_variant,genic_downstream_transcript_variant
rs762940258	genic_upstream_transcript_variant,intron_variant
rs762988090	genic_upstream_transcript_variant,intron_variant
rs762991378	genic_upstream_transcript_variant,intron_variant
rs762997935	genic_upstream_transcript_variant,intron_variant
rs763003602	intron_variant
rs763045980	intron_variant
rs763047195	intron_variant
rs763200862	non_coding_transcript_variant,genic_downstream_transcript_variant,frameshift_variant,coding_sequence_variant
rs763220648	intron_variant
rs763228563	genic_upstream_transcript_variant,intron_variant,upstream_transcript_variant,2KB_upstream_variant
rs763229134	intron_variant
rs763250326	genic_upstream_transcript_variant,intron_variant
rs763254967	intron_variant
rs763290694	intron_variant,genic_downstream_transcript_variant
rs763336926	intron_variant,genic_downstream_transcript_variant
rs763349755	intron_variant
rs763365486	intron_variant,genic_downstream_transcript_variant
rs763371973	genic_upstream_transcript_variant,intron_variant
rs763388965	intron_variant
rs763442680	intron_variant
rs763481809	non_coding_transcript_variant,synonymous_variant,coding_sequence_variant
rs763503217	intron_variant
rs763521162	intron_variant
rs763532683	intron_variant
rs763566579	intron_variant
rs763567278	genic_upstream_transcript_variant,intron_variant,upstream_transcript_variant,2KB_upstream_variant
rs763587498	intron_variant
rs763602886	genic_upstream_transcript_variant,intron_variant
rs763613982	genic_upstream_transcript_variant,intron_variant
rs763750999	intron_variant
rs763766124	intron_variant,genic_downstream_transcript_variant
rs763805848	genic_upstream_transcript_variant,intron_variant
rs763809275	intron_variant
rs763829971	intron_variant
rs763839257	intron_variant
rs763894522	genic_upstream_transcript_variant,intron_variant,upstream_transcript_variant,2KB_upstream_variant
rs763901969	intron_variant
rs763921739	intron_variant
rs763937238	genic_upstream_transcript_variant,intron_variant
rs763982333	genic_upstream_transcript_variant,intron_variant
rs764001968	genic_upstream_transcript_variant,intron_variant
rs764098545	intron_variant,genic_downstream_transcript_variant
rs764105241	genic_upstream_transcript_variant,intron_variant
rs764114444	genic_upstream_transcript_variant,intron_variant
rs764173758	intron_variant
rs764197623	intron_variant
rs764206203	intron_variant,genic_downstream_transcript_variant
rs764250962	genic_upstream_transcript_variant,intron_variant
rs764255635	intron_variant,genic_downstream_transcript_variant
rs764256174	non_coding_transcript_variant,genic_downstream_transcript_variant,3_prime_UTR_variant
rs764271159	intron_variant,genic_downstream_transcript_variant
rs764297742	intron_variant
rs764298326	intron_variant
rs764327245	intron_variant,genic_downstream_transcript_variant
rs764341826	genic_upstream_transcript_variant,intron_variant
rs764368218	non_coding_transcript_variant,genic_downstream_transcript_variant,coding_sequence_variant,missense_variant
rs764387480	intron_variant
rs764400972	intron_variant
rs764421234	intron_variant,genic_downstream_transcript_variant,downstream_transcript_variant
rs764450961	genic_upstream_transcript_variant,intron_variant
rs764455004	genic_upstream_transcript_variant,intron_variant,non_coding_transcript_variant,coding_sequence_variant,missense_variant
rs764479450	intron_variant
rs764487179	genic_upstream_transcript_variant,intron_variant

rs764549271	non_coding_transcript_variant,genic_downstream_transcript_variant,coding_sequence_variant,missense_variant
rs764560452	intron_variant
rs764564850	genic_upstream_transcript_variant,intron_variant
rs764594692	genic_upstream_transcript_variant,intron_variant
rs764595037	intron_variant,genic_downstream_transcript_variant
rs764613944	genic_upstream_transcript_variant,intron_variant
rs764653966	genic_upstream_transcript_variant,intron_variant
rs764655154	genic_upstream_transcript_variant,intron_variant
rs764662911	intron_variant
rs764680429	genic_upstream_transcript_variant,intron_variant
rs764682992	intron_variant
rs764687022	genic_upstream_transcript_variant,intron_variant
rs764706563	intron_variant,genic_downstream_transcript_variant
rs764715522	genic_upstream_transcript_variant,intron_variant
rs764716245	intron_variant
rs764718816	intron_variant
rs764720791	intron_variant
rs764729191	genic_upstream_transcript_variant,intron_variant
rs764750373	intron_variant
rs764811491	genic_upstream_transcript_variant,intron_variant,inframe_deletion,non_coding_transcript_variant,coding_sequence_variant
rs764814223	non_coding_transcript_variant,genic_downstream_transcript_variant,coding_sequence_variant,missense_variant
rs764856420	intron_variant
rs764875423	non_coding_transcript_variant,genic_downstream_transcript_variant,coding_sequence_variant,missense_variant
rs764877929	intron_variant
rs764901343	intron_variant
rs764931963	intron_variant
rs765004631	intron_variant,genic_downstream_transcript_variant
rs765045451	genic_upstream_transcript_variant,intron_variant
rs765057097	genic_upstream_transcript_variant,intron_variant
rs765058024	non_coding_transcript_variant,genic_downstream_transcript_variant,3_prime_UTR_variant
rs765082989	genic_upstream_transcript_variant,intron_variant
rs765122275	intron_variant,genic_downstream_transcript_variant
rs765133207	genic_upstream_transcript_variant,intron_variant
rs765135279	genic_upstream_transcript_variant,intron_variant
rs765135956	intron_variant
rs765246117	genic_upstream_transcript_variant,intron_variant,non_coding_transcript_variant,coding_sequence_variant,missense_variant
rs765259685	intron_variant
rs765292036	genic_upstream_transcript_variant,intron_variant
rs765311042	non_coding_transcript_variant,coding_sequence_variant,missense_variant
rs765318458	genic_upstream_transcript_variant,intron_variant
rs765346543	intron_variant
rs765356988	intron_variant
rs765358638	intron_variant
rs765362467	genic_upstream_transcript_variant,intron_variant
rs765371714	intron_variant,genic_downstream_transcript_variant,downstream_transcript_variant
rs765396951	intron_variant,genic_downstream_transcript_variant
rs765413500	intron_variant
rs765423511	genic_upstream_transcript_variant,intron_variant
rs765451597	intron_variant,genic_downstream_transcript_variant
rs765470362	intron_variant,genic_downstream_transcript_variant
rs765480608	intron_variant
rs765501240	intron_variant
rs765512275	intron_variant,genic_downstream_transcript_variant
rs765518753	intron_variant
rs765537944	genic_upstream_transcript_variant,intron_variant
rs765540755	intron_variant
rs765604809	genic_upstream_transcript_variant,intron_variant,upstream_transcript_variant,2KB_upstream_variant
rs765643487	intron_variant
rs765646981	intron_variant
rs765659351	intron_variant,genic_downstream_transcript_variant
rs765690911	intron_variant,genic_downstream_transcript_variant
rs765711111	intron_variant
rs765748859	genic_upstream_transcript_variant,intron_variant
rs765749466	intron_variant,genic_downstream_transcript_variant
rs765771575	synonymous_variant,non_coding_transcript_variant,genic_downstream_transcript_variant,coding_sequence_variant,missense_variant
rs765880662	genic_upstream_transcript_variant,intron_variant
rs765971182	intron_variant
rs765976081	intron_variant,genic_downstream_transcript_variant
rs766024538	intron_variant,genic_downstream_transcript_variant
rs766068826	genic_upstream_transcript_variant,intron_variant
rs766127293	intron_variant
rs766136369	non_coding_transcript_variant,genic_downstream_transcript_variant,synonymous_variant,coding_sequence_variant
rs766141870	intron_variant
rs766161277	genic_upstream_transcript_variant,intron_variant,upstream_transcript_variant,2KB_upstream_variant

rs766220619	intron_variant
rs766228630	intron_variant,genic_downstream_transcript_variant
rs766388196	intron_variant
rs766399523	intron_variant
rs766405300	intron_variant
rs766452749	intron_variant
rs766503170	genic_upstream_transcript_variant,intron_variant
rs766558855	intron_variant
rs766566436	intron_variant
rs766574369	genic_upstream_transcript_variant,intron_variant
rs766611865	intron_variant
rs766626042	genic_upstream_transcript_variant,intron_variant
rs766639008	intron_variant,genic_downstream_transcript_variant
rs766655052	genic_upstream_transcript_variant,intron_variant
rs766678471	intron_variant
rs766696956	intron_variant,genic_downstream_transcript_variant
rs766722248	intron_variant
rs766797459	intron_variant
rs766798203	intron_variant,genic_downstream_transcript_variant
rs766802875	genic_upstream_transcript_variant,intron_variant
rs766815155	genic_upstream_transcript_variant,intron_variant,non_coding_transcript_variant,coding_sequence_variant,missense_variant
rs766835086	intron_variant
rs766849054	genic_upstream_transcript_variant,intron_variant
rs766865557	stop_gained,synonymous_variant,non_coding_transcript_variant,genic_downstream_transcript_variant,coding_sequence_variant
rs766874293	genic_upstream_transcript_variant,intron_variant,non_coding_transcript_variant,coding_sequence_variant,missense_variant
rs766890763	intron_variant
rs766901114	genic_upstream_transcript_variant,intron_variant,non_coding_transcript_variant
rs766923199	intron_variant
rs766944441	genic_upstream_transcript_variant,intron_variant,non_coding_transcript_variant,coding_sequence_variant,missense_variant
rs766944509	intron_variant
rs766977461	genic_upstream_transcript_variant,intron_variant
rs766983799	intron_variant
rs767033171	intron_variant
rs767063578	intron_variant,genic_downstream_transcript_variant
rs767126190	genic_upstream_transcript_variant,intron_variant
rs767177456	intron_variant
rs767181465	intron_variant,genic_downstream_transcript_variant
rs767200663	intron_variant,genic_downstream_transcript_variant
rs767232472	non_coding_transcript_variant,coding_sequence_variant,missense_variant
rs767250103	genic_upstream_transcript_variant,intron_variant
rs767256435	intron_variant,genic_downstream_transcript_variant
rs767267441	synonymous_variant,non_coding_transcript_variant,genic_downstream_transcript_variant,coding_sequence_variant,missense_variant
rs767290443	intron_variant,genic_downstream_transcript_variant
rs767305539	intron_variant
rs767348234	intron_variant,genic_downstream_transcript_variant
rs767357051	intron_variant,genic_downstream_transcript_variant,downstream_transcript_variant
rs767362061	genic_upstream_transcript_variant,intron_variant
rs767375583	genic_upstream_transcript_variant,intron_variant,synonymous_variant,non_coding_transcript_variant,coding_sequence_variant
rs767406683	intron_variant
rs767442805	intron_variant
rs767454912	genic_upstream_transcript_variant,intron_variant
rs767491349	intron_variant,genic_downstream_transcript_variant
rs767527057	genic_upstream_transcript_variant,intron_variant
rs767537903	intron_variant
rs767585305	genic_upstream_transcript_variant,intron_variant
rs767585693	intron_variant,genic_downstream_transcript_variant
rs767593124	genic_upstream_transcript_variant,intron_variant
rs767636605	intron_variant
rs767654490	intron_variant
rs767679159	genic_downstream_transcript_variant,intron_variant
rs767687478	genic_downstream_transcript_variant,non_coding_transcript_variant,missense_variant,coding_sequence_variant
rs767710588	intron_variant
rs767712825	genic_upstream_transcript_variant,intron_variant
rs767719555	genic_downstream_transcript_variant,intron_variant
rs767720971	genic_downstream_transcript_variant,intron_variant
rs767728710	genic_downstream_transcript_variant,intron_variant
rs767736065	genic_downstream_transcript_variant,non_coding_transcript_variant,missense_variant,coding_sequence_variant
rs767737226	genic_downstream_transcript_variant,intron_variant
rs767770443	missense_variant,non_coding_transcript_variant,coding_sequence_variant
rs767781666	genic_downstream_transcript_variant,intron_variant
rs767803237	intron_variant
rs767810987	genic_downstream_transcript_variant,intron_variant
rs767816035	genic_upstream_transcript_variant,intron_variant
rs767825474	intron_variant

rs767931554	genic_downstream_transcript_variant,non_coding_transcript_variant,missense_variant,coding_sequence_variant
rs767943914	2KB_upstream_variant,upstream_transcript_variant,genic_upstream_transcript_variant,intron_variant
rs768000278	genic_upstream_transcript_variant,intron_variant
rs768039929	intron_variant
rs768053811	intron_variant
rs768074029	intron_variant
rs768095750	intron_variant
rs768133322	intron_variant
rs768140276	intron_variant
rs768149852	synonymous_variant,non_coding_transcript_variant,coding_sequence_variant
rs768164011	intron_variant
rs768186336	genic_upstream_transcript_variant,intron_variant
rs768207662	genic_upstream_transcript_variant,intron_variant
rs768237016	2KB_upstream_variant,upstream_transcript_variant,genic_upstream_transcript_variant,intron_variant
rs768247839	genic_upstream_transcript_variant,intron_variant
rs768262313	intron_variant
rs768265505	genic_upstream_transcript_variant,intron_variant
rs768279546	intron_variant
rs768282705	genic_downstream_transcript_variant,intron_variant
rs768302930	genic_upstream_transcript_variant,intron_variant
rs768338757	genic_downstream_transcript_variant,intron_variant
rs768342842	genic_upstream_transcript_variant,intron_variant
rs768392752	genic_downstream_transcript_variant,intron_variant
rs768401013	genic_upstream_transcript_variant,intron_variant
rs768416838	genic_upstream_transcript_variant,intron_variant
rs768452050	genic_downstream_transcript_variant,non_coding_transcript_variant,3_prime_UTR_variant
rs768452749	genic_upstream_transcript_variant,intron_variant
rs768478510	genic_upstream_transcript_variant,intron_variant
rs768481976	genic_upstream_transcript_variant,intron_variant
rs768510364	intron_variant
rs768514788	intron_variant
rs768521017	genic_downstream_transcript_variant,synonymous_variant,non_coding_transcript_variant,coding_sequence_variant
rs768536142	2KB_upstream_variant,upstream_transcript_variant,genic_upstream_transcript_variant,intron_variant
rs768567386	intron_variant
rs768568721	intron_variant
rs768619871	intron_variant
rs768631933	intron_variant
rs768638354	intron_variant
rs768648038	genic_upstream_transcript_variant,intron_variant
rs768666216	genic_downstream_transcript_variant,intron_variant
rs768666703	genic_downstream_transcript_variant,intron_variant
rs768681640	intron_variant
rs768705021	intron_variant
rs768719446	genic_downstream_transcript_variant,intron_variant
rs768723704	genic_upstream_transcript_variant,intron_variant
rs768785167	genic_downstream_transcript_variant,intron_variant
rs768789322	genic_downstream_transcript_variant,intron_variant
rs768789594	intron_variant
rs768791457	intron_variant
rs768842447	intron_variant
rs768845843	genic_upstream_transcript_variant,intron_variant
rs768873133	genic_downstream_transcript_variant,intron_variant
rs768875023	genic_downstream_transcript_variant,intron_variant
rs768957279	intron_variant
rs768986107	genic_downstream_transcript_variant,intron_variant
rs768994279	intron_variant
rs769023801	intron_variant
rs769062753	genic_upstream_transcript_variant,intron_variant
rs769077231	intron_variant
rs769080501	genic_downstream_transcript_variant,intron_variant
rs769085111	genic_downstream_transcript_variant,intron_variant
rs769149301	intron_variant
rs769193197	intron_variant
rs769240915	genic_downstream_transcript_variant,intron_variant
rs769298832	intron_variant
rs769300115	genic_upstream_transcript_variant,intron_variant
rs769320137	intron_variant
rs769349525	genic_downstream_transcript_variant,intron_variant
rs769366576	intron_variant
rs769390271	genic_downstream_transcript_variant,non_coding_transcript_variant,3_prime_UTR_variant
rs769397942	genic_upstream_transcript_variant,intron_variant
rs769409893	intron_variant
rs769410849	genic_downstream_transcript_variant,intron_variant
rs769412597	genic_upstream_transcript_variant,intron_variant

rs769436388	intron_variant
rs769441540	genic_downstream_transcript_variant,intron_variant
rs769454967	genic_upstream_transcript_variant,intron_variant
rs769474791	genic_downstream_transcript_variant,synonymous_variant,non_coding_transcript_variant,coding_sequence_variant
rs769507421	non_coding_transcript_variant,genic_upstream_transcript_variant,coding_sequence_variant,synonymous_variant,intron_variant
rs769523896	genic_upstream_transcript_variant,intron_variant
rs769529815	intron_variant
rs769552513	intron_variant
rs769559836	intron_variant
rs769585920	intron_variant
rs769590991	genic_upstream_transcript_variant,intron_variant
rs769635709	genic_upstream_transcript_variant,intron_variant
rs769642577	intron_variant
rs769649681	missense_variant,non_coding_transcript_variant,coding_sequence_variant
rs769653743	intron_variant
rs769696690	genic_upstream_transcript_variant,intron_variant
rs769706345	intron_variant
rs769721494	genic_downstream_transcript_variant,non_coding_transcript_variant,missense_variant,coding_sequence_variant
rs769757694	synonymous_variant,non_coding_transcript_variant,coding_sequence_variant
rs769773230	intron_variant
rs769776433	genic_upstream_transcript_variant,intron_variant
rs769799488	intron_variant
rs769808985	genic_downstream_transcript_variant,intron_variant
rs769814035	genic_downstream_transcript_variant,intron_variant
rs769827488	genic_downstream_transcript_variant,intron_variant
rs769830885	genic_downstream_transcript_variant,intron_variant
rs769859867	intron_variant
rs769881952	genic_downstream_transcript_variant,intron_variant
rs769931527	genic_upstream_transcript_variant,intron_variant
rs769988668	genic_upstream_transcript_variant,intron_variant
rs769993835	genic_upstream_transcript_variant,intron_variant
rs770022379	genic_upstream_transcript_variant,intron_variant
rs770025886	genic_upstream_transcript_variant,intron_variant
rs770028047	intron_variant
rs770065936	genic_downstream_transcript_variant,synonymous_variant,non_coding_transcript_variant,coding_sequence_variant
rs770127842	intron_variant
rs770136314	intron_variant
rs770136415	intron_variant
rs770139854	intron_variant
rs770140466	intron_variant
rs770168163	genic_downstream_transcript_variant,intron_variant
rs770193543	intron_variant
rs770215323	genic_upstream_transcript_variant,intron_variant
rs770226367	intron_variant
rs770235320	intron_variant
rs770244862	intron_variant
rs770248442	synonymous_variant,intron_variant,coding_sequence_variant
rs770257200	intron_variant
rs770262391	genic_downstream_transcript_variant,intron_variant
rs770292593	genic_upstream_transcript_variant,intron_variant
rs770343484	genic_upstream_transcript_variant,intron_variant
rs770384154	synonymous_variant,non_coding_transcript_variant,coding_sequence_variant
rs770445407	genic_downstream_transcript_variant,intron_variant
rs770486997	intron_variant
rs770492395	intron_variant
rs770528254	non_coding_transcript_variant,genic_upstream_transcript_variant,coding_sequence_variant,missense_variant,intron_variant
rs770538411	genic_downstream_transcript_variant,intron_variant
rs770545490	genic_downstream_transcript_variant,intron_variant
rs770547332	genic_upstream_transcript_variant,intron_variant
rs770562344	intron_variant
rs770598900	genic_downstream_transcript_variant,intron_variant
rs770600770	intron_variant
rs770600906	intron_variant
rs770648346	genic_upstream_transcript_variant,intron_variant
rs770687297	genic_downstream_transcript_variant,intron_variant
rs770726789	intron_variant
rs770782239	intron_variant
rs770811693	3_prime_UTR_variant,intron_variant
rs770812567	intron_variant
rs770827456	intron_variant
rs770829642	genic_downstream_transcript_variant,intron_variant
rs770848331	intron_variant
rs770869284	genic_downstream_transcript_variant,intron_variant
rs770877257	genic_upstream_transcript_variant,intron_variant

rs770883074	genic_upstream_transcript_variant,intron_variant
rs770944275	intron_variant
rs770981650	genic_downstream_transcript_variant,intron_variant
rs771010969	genic_upstream_transcript_variant,intron_variant
rs771092101	intron_variant
rs771093041	genic_upstream_transcript_variant,intron_variant
rs771150095	genic_upstream_transcript_variant,intron_variant
rs771160906	genic_upstream_transcript_variant,intron_variant
rs771162921	genic_upstream_transcript_variant,intron_variant
rs771175019	intron_variant
rs771180044	intron_variant
rs771266283	intron_variant
rs771272738	intron_variant
rs771274474	genic_downstream_transcript_variant,intron_variant
rs771306637	genic_upstream_transcript_variant,intron_variant
rs771311966	genic_upstream_transcript_variant,intron_variant
rs771327980	genic_upstream_transcript_variant,intron_variant
rs771375062	intron_variant
rs771382902	genic_downstream_transcript_variant,intron_variant
rs771383250	genic_downstream_transcript_variant,intron_variant
rs771435910	intron_variant
rs771436236	genic_downstream_transcript_variant,non_coding_transcript_variant,missense_variant,coding_sequence_variant
rs771477900	intron_variant
rs771500235	intron_variant
rs771505942	genic_downstream_transcript_variant,intron_variant
rs771569093	genic_downstream_transcript_variant,intron_variant
rs771571698	genic_downstream_transcript_variant,synonymous_variant,non_coding_transcript_variant,coding_sequence_variant
rs771574814	genic_upstream_transcript_variant,intron_variant
rs771627874	2KB_upstream_variant,upstream_transcript_variant,genic_upstream_transcript_variant,intron_variant
rs771636506	genic_downstream_transcript_variant,intron_variant
rs771668305	missense_variant,non_coding_transcript_variant,coding_sequence_variant
rs771679296	genic_downstream_transcript_variant,non_coding_transcript_variant,frameshift_variant,coding_sequence_variant
rs771685073	genic_upstream_transcript_variant,intron_variant
rs771738253	genic_downstream_transcript_variant,intron_variant
rs771739170	genic_upstream_transcript_variant,intron_variant
rs771748687	intron_variant
rs771762363	genic_upstream_transcript_variant,intron_variant
rs771791385	genic_downstream_transcript_variant,intron_variant
rs771831786	genic_downstream_transcript_variant,intron_variant
rs771842911	intron_variant
rs771843017	genic_upstream_transcript_variant,intron_variant
rs771846514	intron_variant
rs771872531	intron_variant
rs771908927	non_coding_transcript_variant,genic_upstream_transcript_variant,coding_sequence_variant,missense_variant,intron_variant
rs771957466	synonymous_variant,intron_variant,coding_sequence_variant
rs771985905	genic_upstream_transcript_variant,intron_variant
rs772022370	intron_variant
rs772036258	genic_upstream_transcript_variant,intron_variant
rs772060041	genic_upstream_transcript_variant,intron_variant
rs772067081	2KB_upstream_variant,upstream_transcript_variant,genic_upstream_transcript_variant,intron_variant
rs772070414	intron_variant
rs772089603	intron_variant
rs772099331	genic_downstream_transcript_variant,intron_variant
rs772174989	genic_downstream_transcript_variant,intron_variant
rs772185290	genic_downstream_transcript_variant,intron_variant
rs772188778	genic_upstream_transcript_variant,intron_variant
rs772191921	intron_variant
rs772210094	intron_variant
rs772251732	genic_upstream_transcript_variant,intron_variant
rs772264642	intron_variant
rs772273569	genic_upstream_transcript_variant,intron_variant
rs772324616	genic_downstream_transcript_variant,intron_variant
rs772355754	genic_upstream_transcript_variant,intron_variant
rs772367628	intron_variant
rs772368072	intron_variant
rs772380192	intron_variant
rs772395904	intron_variant
rs772411950	genic_downstream_transcript_variant,non_coding_transcript_variant,3_prime_UTR_variant
rs772423139	intron_variant
rs772429193	intron_variant
rs772435465	genic_downstream_transcript_variant,synonymous_variant,non_coding_transcript_variant,coding_sequence_variant
rs772478562	genic_downstream_transcript_variant,intron_variant
rs772483236	genic_upstream_transcript_variant,intron_variant
rs772517822	genic_upstream_transcript_variant,intron_variant

rs772597264	missense_variant,non_coding_transcript_variant,coding_sequence_variant
rs772601338	intron_variant
rs772603428	genic_upstream_transcript_variant,intron_variant
rs772628248	intron_variant
rs772700988	genic_downstream_transcript_variant,intron_variant
rs772735574	genic_downstream_transcript_variant,intron_variant
rs772771000	splice_acceptor_variant,intron_variant
rs772778479	genic_upstream_transcript_variant,intron_variant
rs772792840	genic_downstream_transcript_variant,intron_variant
rs772802861	intron_variant
rs772808573	genic_downstream_transcript_variant,intron_variant
rs772814117	genic_upstream_transcript_variant,intron_variant
rs772825451	genic_downstream_transcript_variant,intron_variant
rs772880631	intron_variant
rs772881779	2KB_upstream_variant,upstream_transcript_variant,genic_upstream_transcript_variant,intron_variant
rs772900732	genic_upstream_transcript_variant,intron_variant
rs772912065	intron_variant
rs772960515	genic_downstream_transcript_variant,intron_variant
rs772960961	intron_variant
rs772971653	genic_downstream_transcript_variant,non_coding_transcript_variant,3_prime_UTR_variant
rs773019048	genic_downstream_transcript_variant,non_coding_transcript_variant,3_prime_UTR_variant
rs773033518	genic_upstream_transcript_variant,intron_variant
rs773044670	genic_upstream_transcript_variant,intron_variant
rs773066983	2KB_upstream_variant,upstream_transcript_variant,genic_upstream_transcript_variant,intron_variant
rs773102768	genic_upstream_transcript_variant,intron_variant
rs773107496	synonymous_variant,non_coding_transcript_variant,coding_sequence_variant
rs773138058	intron_variant
rs773160188	missense_variant,intron_variant,coding_sequence_variant
rs773227861	intron_variant
rs773246977	intron_variant
rs773249836	intron_variant
rs773267197	genic_upstream_transcript_variant,intron_variant
rs773274779	genic_upstream_transcript_variant,intron_variant
rs773276671	intron_variant
rs773294911	intron_variant
rs773296243	genic_upstream_transcript_variant,intron_variant
rs773307293	intron_variant
rs773322216	intron_variant
rs773365033	intron_variant
rs773370210	missense_variant,intron_variant,coding_sequence_variant
rs773419186	genic_downstream_transcript_variant,intron_variant
rs773457266	genic_downstream_transcript_variant,intron_variant
rs773472204	genic_downstream_transcript_variant,intron_variant
rs773505925	genic_upstream_transcript_variant,intron_variant
rs773507438	genic_upstream_transcript_variant,intron_variant
rs773537252	intron_variant
rs773604455	non_coding_transcript_variant,genic_upstream_transcript_variant,coding_sequence_variant,synonymous_variant,intron_variant
rs773645721	genic_downstream_transcript_variant,intron_variant
rs773690724	intron_variant
rs773704375	intron_variant
rs773719648	genic_downstream_transcript_variant,synonymous_variant,non_coding_transcript_variant,coding_sequence_variant
rs773725143	genic_upstream_transcript_variant,intron_variant
rs773767160	genic_downstream_transcript_variant,intron_variant
rs773774215	intron_variant
rs773786664	intron_variant
rs773799431	genic_upstream_transcript_variant,intron_variant
rs773819002	genic_downstream_transcript_variant,intron_variant
rs773830736	missense_variant,non_coding_transcript_variant,coding_sequence_variant
rs773834696	genic_downstream_transcript_variant,intron_variant
rs773835378	genic_upstream_transcript_variant,intron_variant
rs773860147	intron_variant
rs773882064	intron_variant
rs773886979	genic_upstream_transcript_variant,intron_variant
rs773904282	intron_variant
rs773923004	genic_downstream_transcript_variant,intron_variant
rs773983076	genic_downstream_transcript_variant,intron_variant
rs774028977	genic_downstream_transcript_variant,intron_variant
rs774043814	genic_upstream_transcript_variant,intron_variant
rs774093686	intron_variant
rs774106710	intron_variant
rs774186517	genic_upstream_transcript_variant,intron_variant
rs774207320	intron_variant
rs774213720	genic_downstream_transcript_variant,intron_variant
rs774216118	intron_variant

rs774231385	genic_upstream_transcript_variant,intron_variant
rs774388148	intron_variant
rs774456424	genic_downstream_transcript_variant,intron_variant
rs774473696	intron_variant
rs774490932	genic_downstream_transcript_variant,non_coding_transcript_variant,3_prime_UTR_variant
rs774531064	genic_upstream_transcript_variant,intron_variant
rs774534149	genic_upstream_transcript_variant,intron_variant
rs774556111	intron_variant
rs774561552	intron_variant
rs774562505	genic_downstream_transcript_variant,intron_variant
rs774585404	non_coding_transcript_variant,genic_upstream_transcript_variant,coding_sequence_variant,missense_variant,intron_variant
rs774593771	genic_downstream_transcript_variant,intron_variant
rs774621792	intron_variant
rs774639922	intron_variant
rs774685524	genic_upstream_transcript_variant,intron_variant
rs774779741	missense_variant,non_coding_transcript_variant,5_prime_UTR_variant,coding_sequence_variant
rs774807315	intron_variant
rs774817618	genic_downstream_transcript_variant,synonymous_variant,non_coding_transcript_variant,coding_sequence_variant
rs774828552	genic_downstream_transcript_variant,intron_variant
rs774832964	intron_variant
rs774869768	intron_variant
rs774874334	genic_upstream_transcript_variant,intron_variant
rs774883255	intron_variant
rs774885631	synonymous_variant,non_coding_transcript_variant,coding_sequence_variant
rs774901623	genic_upstream_transcript_variant,intron_variant
rs774914492	genic_upstream_transcript_variant,intron_variant
rs774936388	intron_variant
rs774952015	intron_variant
rs774952152	missense_variant,non_coding_transcript_variant,coding_sequence_variant
rs774972566	intron_variant
rs775011117	missense_variant,non_coding_transcript_variant,coding_sequence_variant
rs775043661	genic_downstream_transcript_variant,non_coding_transcript_variant,missense_variant,coding_sequence_variant
rs775105219	genic_upstream_transcript_variant,intron_variant
rs775150626	intron_variant
rs775159771	genic_downstream_transcript_variant,intron_variant
rs775176900	genic_upstream_transcript_variant,intron_variant
rs775178778	genic_downstream_transcript_variant,intron_variant
rs775183859	missense_variant,non_coding_transcript_variant,coding_sequence_variant
rs775200178	genic_downstream_transcript_variant,intron_variant
rs775205260	genic_upstream_transcript_variant,intron_variant
rs775215349	genic_downstream_transcript_variant,intron_variant
rs775235138	synonymous_variant,non_coding_transcript_variant,coding_sequence_variant
rs775241410	intron_variant
rs775248095	genic_upstream_transcript_variant,intron_variant
rs775286634	intron_variant
rs775290780	intron_variant
rs775309451	genic_upstream_transcript_variant,intron_variant
rs775328951	genic_downstream_transcript_variant,intron_variant
rs775340340	genic_downstream_transcript_variant,intron_variant
rs775350202	genic_downstream_transcript_variant,intron_variant
rs775351710	genic_downstream_transcript_variant,intron_variant
rs775380286	genic_downstream_transcript_variant,intron_variant
rs775399904	intron_variant
rs775435029	genic_upstream_transcript_variant,intron_variant
rs775443447	intron_variant
rs775458347	genic_downstream_transcript_variant,intron_variant
rs775541395	genic_downstream_transcript_variant,intron_variant
rs775629036	genic_downstream_transcript_variant,intron_variant
rs775631651	genic_downstream_transcript_variant,intron_variant
rs775635934	intron_variant
rs775636278	genic_upstream_transcript_variant,intron_variant
rs775643960	intron_variant
rs775684515	intron_variant
rs775694331	genic_upstream_transcript_variant,intron_variant
rs775729503	intron_variant
rs775734997	2KB_upstream_variant,upstream_transcript_variant,genic_upstream_transcript_variant,intron_variant
rs775763251	genic_upstream_transcript_variant,intron_variant
rs775781308	genic_upstream_transcript_variant,intron_variant
rs775784977	intron_variant
rs775837022	genic_upstream_transcript_variant,intron_variant
rs775848964	genic_upstream_transcript_variant,intron_variant
rs775859892	genic_downstream_transcript_variant,intron_variant
rs775889973	genic_upstream_transcript_variant,intron_variant
rs775899680	genic_upstream_transcript_variant,intron_variant

rs775916576	intron_variant
rs775969203	intron_variant
rs775986918	intron_variant
rs776021887	intron_variant
rs776036761	genic_downstream_transcript_variant,intron_variant
rs776054822	intron_variant
rs776067879	genic_upstream_transcript_variant,intron_variant
rs776070341	genic_upstream_transcript_variant,intron_variant
rs776074043	intron_variant
rs776075302	genic_upstream_transcript_variant,intron_variant
rs776086522	intron_variant
rs776086725	intron_variant
rs776103715	genic_upstream_transcript_variant,intron_variant
rs776137927	synonymous_variant,non_coding_transcript_variant,coding_sequence_variant
rs776142243	genic_downstream_transcript_variant,intron_variant
rs776179393	intron_variant
rs776202512	genic_downstream_transcript_variant,intron_variant
rs776228225	genic_downstream_transcript_variant,intron_variant
rs776267715	intron_variant
rs776396109	intron_variant
rs776398619	genic_upstream_transcript_variant,intron_variant
rs776418678	genic_upstream_transcript_variant,intron_variant
rs776426414	non_coding_transcript_variant,genic_upstream_transcript_variant,coding_sequence_variant,missense_variant,intron_variant
rs776449230	genic_upstream_transcript_variant,intron_variant
rs776453842	genic_upstream_transcript_variant,intron_variant
rs776515946	genic_upstream_transcript_variant,intron_variant
rs776541753	intron_variant
rs776559516	genic_upstream_transcript_variant,intron_variant
rs776562646	intron_variant
rs776564008	genic_downstream_transcript_variant,intron_variant
rs776571309	genic_downstream_transcript_variant,intron_variant
rs776573546	genic_upstream_transcript_variant,intron_variant
rs776589175	genic_upstream_transcript_variant,intron_variant
rs776640090	non_coding_transcript_variant,genic_upstream_transcript_variant,coding_sequence_variant,5_prime_UTR_variant,missense_variant,intron_variant
rs776651937	synonymous_variant,non_coding_transcript_variant,coding_sequence_variant
rs776653997	genic_downstream_transcript_variant,intron_variant
rs776654237	genic_downstream_transcript_variant,intron_variant
rs776671844	intron_variant
rs776673450	intron_variant
rs776714088	genic_downstream_transcript_variant,intron_variant
rs776730375	intron_variant
rs776735081	genic_upstream_transcript_variant,intron_variant
rs776754079	intron_variant,genic_upstream_transcript_variant
rs776756331	intron_variant
rs776766309	intron_variant,genic_downstream_transcript_variant,downstream_transcript_variant
rs776773955	intron_variant
rs776798807	intron_variant,genic_upstream_transcript_variant
rs776809301	intron_variant
rs776829098	intron_variant,synonymous_variant,coding_sequence_variant
rs776840873	intron_variant,genic_upstream_transcript_variant
rs776895034	intron_variant,genic_downstream_transcript_variant
rs776928564	intron_variant
rs776957271	intron_variant,upstream_transcript_variant,genic_upstream_transcript_variant,2KB_upstream_variant
rs776983934	intron_variant,genic_upstream_transcript_variant
rs777057689	intron_variant,genic_downstream_transcript_variant
rs777095825	intron_variant
rs777147946	intron_variant,genic_upstream_transcript_variant
rs777152817	5_prime_UTR_variant,intron_variant
rs777181515	intron_variant
rs777182688	intron_variant
rs777188029	intron_variant,genic_upstream_transcript_variant
rs777200215	intron_variant,genic_downstream_transcript_variant
rs777212789	intron_variant,genic_upstream_transcript_variant
rs777214043	coding_sequence_variant,genic_downstream_transcript_variant,non_coding_transcript_variant,missense_variant
rs777220198	intron_variant
rs777280338	intron_variant,genic_upstream_transcript_variant
rs777292117	intron_variant,genic_upstream_transcript_variant
rs777309408	intron_variant,genic_downstream_transcript_variant
rs777311092	genic_downstream_transcript_variant,non_coding_transcript_variant,3_prime_UTR_variant
rs777323971	intron_variant,genic_upstream_transcript_variant
rs777366204	intron_variant,genic_upstream_transcript_variant
rs777367882	synonymous_variant,coding_sequence_variant,non_coding_transcript_variant
rs777380968	intron_variant
rs777385331	intron_variant,genic_upstream_transcript_variant

rs777402379	synonymous_variant,coding_sequence_variant,genic_downstream_transcript_variant,non_coding_transcript_variant
rs777414377	intron_variant,genic_downstream_transcript_variant
rs777423152	intron_variant
rs777423234	intron_variant,genic_upstream_transcript_variant
rs777425331	intron_variant
rs777436282	intron_variant,genic_upstream_transcript_variant
rs777473183	intron_variant,missense_variant,non_coding_transcript_variant,genic_upstream_transcript_variant,coding_sequence_variant
rs777490511	intron_variant,upstream_transcript_variant,genic_upstream_transcript_variant,2KB_upstream_variant
rs777553893	intron_variant,genic_upstream_transcript_variant
rs777556288	intron_variant
rs777608334	intron_variant,genic_downstream_transcript_variant
rs777609309	intron_variant
rs777610887	intron_variant
rs777624815	intron_variant,genic_downstream_transcript_variant
rs777651966	intron_variant,genic_downstream_transcript_variant
rs777670783	intron_variant,genic_upstream_transcript_variant
rs777691235	intron_variant
rs777753098	intron_variant,genic_downstream_transcript_variant
rs777763851	intron_variant
rs777779283	intron_variant,genic_upstream_transcript_variant
rs777810342	intron_variant,genic_upstream_transcript_variant
rs777833095	coding_sequence_variant,non_coding_transcript_variant,missense_variant
rs777843595	intron_variant,genic_upstream_transcript_variant
rs777883495	intron_variant
rs777959426	intron_variant,genic_upstream_transcript_variant
rs777961116	upstream_transcript_variant,intron_variant,2KB_upstream_variant,genic_upstream_transcript_variant
rs777985196	intron_variant,genic_downstream_transcript_variant
rs777995744	intron_variant,genic_downstream_transcript_variant
rs778002788	intron_variant
rs778067527	intron_variant,genic_upstream_transcript_variant
rs778121111	intron_variant
rs778150393	intron_variant
rs778158901	intron_variant
rs778165612	intron_variant
rs778238570	intron_variant
rs778261185	intron_variant
rs778264060	intron_variant,genic_downstream_transcript_variant
rs778282062	coding_sequence_variant,genic_downstream_transcript_variant,non_coding_transcript_variant,missense_variant
rs778298921	intron_variant,genic_downstream_transcript_variant
rs778309347	coding_sequence_variant,non_coding_transcript_variant,missense_variant
rs778318661	intron_variant,genic_upstream_transcript_variant
rs778359072	intron_variant,genic_downstream_transcript_variant
rs778375178	coding_sequence_variant,intron_variant,missense_variant
rs778415960	intron_variant,non_coding_transcript_variant,genic_upstream_transcript_variant,synonymous_variant,coding_sequence_variant
rs778425096	intron_variant,genic_upstream_transcript_variant
rs778434987	intron_variant,genic_upstream_transcript_variant
rs778448140	intron_variant,genic_downstream_transcript_variant
rs778450148	intron_variant
rs778467568	intron_variant
rs778484201	intron_variant,genic_downstream_transcript_variant
rs778503876	intron_variant,genic_upstream_transcript_variant
rs778505186	upstream_transcript_variant,intron_variant,2KB_upstream_variant,genic_upstream_transcript_variant
rs778520785	intron_variant,genic_downstream_transcript_variant
rs778528018	intron_variant,genic_upstream_transcript_variant
rs778573027	intron_variant,genic_upstream_transcript_variant
rs778587768	intron_variant,genic_downstream_transcript_variant
rs778609534	intron_variant,genic_upstream_transcript_variant
rs778614607	intron_variant
rs778626291	coding_sequence_variant,non_coding_transcript_variant,missense_variant
rs778629511	intron_variant
rs778664530	intron_variant
rs778681345	intron_variant
rs778681378	intron_variant
rs778736289	intron_variant,genic_downstream_transcript_variant
rs778759130	intron_variant,genic_downstream_transcript_variant
rs778761793	intron_variant
rs778787496	intron_variant
rs778814556	intron_variant,genic_upstream_transcript_variant
rs778884266	synonymous_variant,coding_sequence_variant,genic_downstream_transcript_variant,non_coding_transcript_variant
rs778904798	intron_variant
rs778914669	intron_variant,genic_upstream_transcript_variant
rs778970616	coding_sequence_variant,genic_downstream_transcript_variant,non_coding_transcript_variant,missense_variant
rs778987291	intron_variant,genic_upstream_transcript_variant
rs779012697	intron_variant

rs779038672	upstream_transcript_variant,intron_variant,2KB_upstream_variant,genic_upstream_transcript_variant
rs779067011	intron_variant,genic_upstream_transcript_variant
rs779070975	intron_variant,genic_upstream_transcript_variant
rs779093243	intron_variant,genic_downstream_transcript_variant,downstream_transcript_variant
rs779104334	intron_variant,genic_upstream_transcript_variant
rs779124983	intron_variant,genic_upstream_transcript_variant
rs779146959	intron_variant,genic_downstream_transcript_variant
rs779157918	intron_variant,genic_upstream_transcript_variant
rs779165473	intron_variant
rs779172881	intron_variant
rs779218607	frameshift_variant,coding_sequence_variant,non_coding_transcript_variant
rs779234527	intron_variant,genic_downstream_transcript_variant
rs779284022	intron_variant,genic_upstream_transcript_variant
rs779289387	intron_variant
rs779328011	synonymous_variant,coding_sequence_variant,non_coding_transcript_variant
rs779339728	coding_sequence_variant,genic_downstream_transcript_variant,non_coding_transcript_variant,missense_variant
rs779346485	intron_variant,upstream_transcript_variant,genic_upstream_transcript_variant,2KB_upstream_variant
rs779353566	intron_variant,genic_upstream_transcript_variant
rs779356180	intron_variant,genic_downstream_transcript_variant
rs779395066	intron_variant
rs779395507	intron_variant
rs779441001	intron_variant,genic_upstream_transcript_variant
rs779545151	intron_variant
rs779571629	intron_variant,upstream_transcript_variant,genic_upstream_transcript_variant,2KB_upstream_variant
rs779579721	intron_variant
rs779587544	synonymous_variant,coding_sequence_variant,intron_variant
rs779604155	intron_variant,genic_downstream_transcript_variant
rs779636079	intron_variant,genic_upstream_transcript_variant
rs779661923	intron_variant
rs779666633	intron_variant,genic_upstream_transcript_variant
rs779671766	intron_variant,genic_upstream_transcript_variant
rs779675074	intron_variant,genic_upstream_transcript_variant
rs779688649	genic_downstream_transcript_variant,non_coding_transcript_variant,3_prime_UTR_variant
rs779694924	intron_variant,synonymous_variant,coding_sequence_variant
rs779763150	intron_variant,genic_upstream_transcript_variant
rs779786138	intron_variant,genic_downstream_transcript_variant
rs779832602	intron_variant
rs779850530	intron_variant,genic_downstream_transcript_variant
rs779880717	intron_variant
rs779931654	intron_variant
rs779950475	5_prime_UTR_variant,synonymous_variant,coding_sequence_variant,non_coding_transcript_variant
rs779971558	intron_variant
rs779985742	intron_variant
rs780021124	intron_variant,genic_downstream_transcript_variant
rs780044012	intron_variant
rs780054879	intron_variant
rs780136309	intron_variant,genic_downstream_transcript_variant
rs780141946	intron_variant
rs780154942	intron_variant,genic_downstream_transcript_variant
rs780236216	intron_variant
rs780285360	intron_variant
rs780287516	coding_sequence_variant,non_coding_transcript_variant,missense_variant
rs780294514	intron_variant,genic_upstream_transcript_variant
rs780296617	intron_variant,genic_upstream_transcript_variant
rs780317042	intron_variant,genic_downstream_transcript_variant
rs780355204	genic_downstream_transcript_variant,non_coding_transcript_variant,3_prime_UTR_variant
rs780385471	intron_variant,genic_downstream_transcript_variant
rs780411452	intron_variant,missense_variant,non_coding_transcript_variant,genic_upstream_transcript_variant,coding_sequence_variant
rs780465335	upstream_transcript_variant,intron_variant,2KB_upstream_variant,genic_upstream_transcript_variant
rs780485505	intron_variant
rs780550641	intron_variant
rs780589650	intron_variant,genic_downstream_transcript_variant
rs780607606	intron_variant
rs780616626	intron_variant
rs780652026	intron_variant,genic_downstream_transcript_variant
rs780686331	intron_variant,genic_upstream_transcript_variant
rs780704997	intron_variant,genic_upstream_transcript_variant
rs780707791	intron_variant,genic_downstream_transcript_variant
rs780766198	coding_sequence_variant,non_coding_transcript_variant,missense_variant
rs780792079	intron_variant,genic_upstream_transcript_variant
rs780792766	intron_variant,genic_upstream_transcript_variant
rs780799092	intron_variant,genic_upstream_transcript_variant
rs780802188	intron_variant
rs780813482	intron_variant

rs780815764	intron_variant,upstream_transcript_variant,genic_upstream_transcript_variant,2KB_upstream_variant
rs780819058	coding_sequence_variant,genic_downstream_transcript_variant,non_coding_transcript_variant,missense_variant
rs780855618	synonymous_variant,coding_sequence_variant,non_coding_transcript_variant
rs780875548	intron_variant,genic_upstream_transcript_variant
rs780928472	intron_variant,non_coding_transcript_variant,genic_upstream_transcript_variant,synonymous_variant,coding_sequence_variant
rs780930361	intron_variant
rs780949820	intron_variant,genic_downstream_transcript_variant
rs780969642	intron_variant,genic_upstream_transcript_variant
rs781051400	intron_variant,genic_downstream_transcript_variant
rs781062382	intron_variant
rs781069869	intron_variant
rs781124848	intron_variant
rs781129270	intron_variant,genic_downstream_transcript_variant
rs781129767	intron_variant,genic_downstream_transcript_variant
rs781180945	intron_variant,genic_downstream_transcript_variant
rs781197995	intron_variant
rs781206797	intron_variant
rs781207398	intron_variant
rs781221151	intron_variant,genic_upstream_transcript_variant
rs781257931	coding_sequence_variant,non_coding_transcript_variant,missense_variant
rs781264989	intron_variant
rs781298100	intron_variant,genic_downstream_transcript_variant
rs781308852	intron_variant,genic_upstream_transcript_variant
rs781344739	intron_variant,genic_upstream_transcript_variant
rs781381710	intron_variant,genic_upstream_transcript_variant
rs781381929	intron_variant,genic_downstream_transcript_variant
rs781383911	intron_variant
rs781401324	intron_variant
rs781433659	missense_variant,non_coding_transcript_variant,genic_downstream_transcript_variant,stop_gained,coding_sequence_variant
rs781469951	intron_variant,genic_upstream_transcript_variant
rs781486731	intron_variant,genic_downstream_transcript_variant
rs781488136	intron_variant
rs781505735	intron_variant,genic_downstream_transcript_variant
rs781521245	intron_variant
rs781524190	intron_variant
rs781579372	intron_variant
rs781581028	intron_variant
rs781604780	intron_variant,genic_upstream_transcript_variant
rs781609446	intron_variant,genic_downstream_transcript_variant
rs781614353	genic_downstream_transcript_variant,non_coding_transcript_variant,3_prime_UTR_variant
rs781625430	intron_variant,genic_downstream_transcript_variant
rs781644862	intron_variant
rs781650156	intron_variant,genic_upstream_transcript_variant
rs781680431	intron_variant
rs781686908	coding_sequence_variant,genic_downstream_transcript_variant,non_coding_transcript_variant,missense_variant
rs781735854	intron_variant
rs781763089	intron_variant,genic_upstream_transcript_variant
rs781774783	intron_variant,genic_upstream_transcript_variant
rs796071410	intron_variant,genic_upstream_transcript_variant
rs796127593	intron_variant,genic_upstream_transcript_variant
rs796135284	intron_variant,genic_downstream_transcript_variant
rs796135481	intron_variant,genic_upstream_transcript_variant
rs796184995	intron_variant,genic_upstream_transcript_variant
rs796186474	intron_variant,genic_upstream_transcript_variant
rs796292241	intron_variant
rs796323371	intron_variant,genic_upstream_transcript_variant
rs796447036	intron_variant,genic_upstream_transcript_variant
rs796448506	intron_variant,genic_upstream_transcript_variant
rs796471380	5_prime_UTR_variant,intron_variant
rs796498668	intron_variant,genic_downstream_transcript_variant
rs796579144	intron_variant
rs796590489	upstream_transcript_variant,intron_variant,genic_upstream_transcript_variant
rs796794767	intron_variant,genic_upstream_transcript_variant
rs796823658	intron_variant,genic_downstream_transcript_variant
rs796839041	intron_variant
rs796872180	intron_variant
rs796941186	intron_variant,genic_upstream_transcript_variant
rs865868534	intron_variant
rs865895969	intron_variant,genic_upstream_transcript_variant
rs865996178	intron_variant
rs866021466	intron_variant,genic_upstream_transcript_variant
rs866072933	synonymous_variant,coding_sequence_variant,non_coding_transcript_variant
rs866116019	intron_variant
rs866117463	intron_variant

rs866129732	intron_variant,genic_upstream_transcript_variant
rs866135295	intron_variant
rs866139419	intron_variant
rs866147016	upstream_transcript_variant,intron_variant,2KB_upstream_variant,genic_upstream_transcript_variant
rs866156213	intron_variant,genic_upstream_transcript_variant
rs866158270	intron_variant,genic_upstream_transcript_variant
rs866163516	intron_variant
rs866255765	intron_variant
rs866282792	intron_variant,genic_downstream_transcript_variant
rs866285134	intron_variant
rs866377933	intron_variant,genic_downstream_transcript_variant
rs866411626	intron_variant,genic_downstream_transcript_variant
rs866413148	intron_variant,genic_downstream_transcript_variant
rs866464968	coding_sequence_variant,genic_downstream_transcript_variant,non_coding_transcript_variant,missense_variant
rs866517595	intron_variant,genic_upstream_transcript_variant
rs866559477	intron_variant,genic_upstream_transcript_variant
rs866561655	intron_variant,genic_downstream_transcript_variant
rs866567825	intron_variant
rs866596003	intron_variant,genic_upstream_transcript_variant
rs866596302	intron_variant,genic_upstream_transcript_variant
rs866600031	intron_variant,genic_upstream_transcript_variant
rs866630185	intron_variant,genic_downstream_transcript_variant
rs866717048	intron_variant,genic_downstream_transcript_variant
rs866717311	intron_variant
rs866726960	intron_variant,genic_upstream_transcript_variant
rs866758369	intron_variant
rs866780626	intron_variant,genic_upstream_transcript_variant
rs866844720	intron_variant
rs866866444	intron_variant,genic_upstream_transcript_variant
rs866866604	intron_variant,genic_upstream_transcript_variant
rs866873279	intron_variant,missense_variant,non_coding_transcript_variant,genic_upstream_transcript_variant,coding_sequence_variant
rs866903795	intron_variant
rs866909935	intron_variant
rs866913843	intron_variant,genic_upstream_transcript_variant
rs866962614	intron_variant
rs866974270	intron_variant,genic_downstream_transcript_variant
rs866976422	intron_variant,genic_upstream_transcript_variant
rs866980293	intron_variant
rs867004471	intron_variant,genic_upstream_transcript_variant
rs867043640	intron_variant
rs867051450	intron_variant
rs867059626	intron_variant,genic_upstream_transcript_variant
rs867064468	coding_sequence_variant,genic_downstream_transcript_variant,non_coding_transcript_variant,missense_variant
rs867075700	intron_variant,upstream_transcript_variant,genic_upstream_transcript_variant,2KB_upstream_variant
rs867128229	intron_variant,genic_upstream_transcript_variant
rs867189432	intron_variant
rs867225791	intron_variant,genic_upstream_transcript_variant
rs867246020	intron_variant,genic_upstream_transcript_variant
rs867317441	intron_variant
rs867324138	intron_variant,genic_downstream_transcript_variant
rs867330835	intron_variant,genic_upstream_transcript_variant
rs867336692	intron_variant,upstream_transcript_variant,genic_upstream_transcript_variant,2KB_upstream_variant
rs867395176	intron_variant,genic_downstream_transcript_variant
rs867401932	intron_variant
rs867409805	intron_variant,genic_upstream_transcript_variant
rs867418929	intron_variant,genic_upstream_transcript_variant
rs867419146	intron_variant,genic_downstream_transcript_variant
rs867422334	intron_variant,genic_upstream_transcript_variant
rs867429752	intron_variant,genic_downstream_transcript_variant
rs867538349	intron_variant
rs867560373	intron_variant,upstream_transcript_variant,genic_upstream_transcript_variant,2KB_upstream_variant
rs867569589	intron_variant
rs867650544	intron_variant
rs867656845	intron_variant,genic_downstream_transcript_variant
rs867667440	intron_variant,genic_downstream_transcript_variant
rs867673904	intron_variant
rs867708964	intron_variant,genic_upstream_transcript_variant
rs867709212	intron_variant
rs867718119	intron_variant,genic_upstream_transcript_variant
rs867769502	coding_sequence_variant,genic_downstream_transcript_variant,non_coding_transcript_variant,missense_variant
rs867816790	intron_variant,genic_downstream_transcript_variant
rs867827441	intron_variant,genic_upstream_transcript_variant
rs867828084	intron_variant
rs867831902	intron_variant,genic_upstream_transcript_variant

rs867883956	intron_variant,genic_downstream_transcript_variant
rs867933645	intron_variant,genic_downstream_transcript_variant
rs867943410	intron_variant
rs867943897	intron_variant,genic_upstream_transcript_variant
rs867956036	genic_upstream_transcript_variant,intron_variant
rs867960474	genic_upstream_transcript_variant,intron_variant
rs867962356	upstream_transcript_variant,intron_variant,genic_upstream_transcript_variant
rs867967813	genic_upstream_transcript_variant,intron_variant
rs867969566	genic_upstream_transcript_variant,intron_variant,upstream_transcript_variant,2KB_upstream_variant
rs868003416	genic_upstream_transcript_variant,missense_variant,coding_sequence_variant,intron_variant,non_coding_transcript_variant
rs868027278	intron_variant
rs868034735	genic_upstream_transcript_variant,intron_variant
rs868076820	intron_variant
rs868084131	intron_variant,genic_downstream_transcript_variant
rs868094939	genic_upstream_transcript_variant,intron_variant
rs868098829	genic_upstream_transcript_variant,intron_variant
rs868108321	genic_upstream_transcript_variant,intron_variant
rs868142960	3_prime_UTR_variant,genic_downstream_transcript_variant,non_coding_transcript_variant
rs868155049	genic_upstream_transcript_variant,intron_variant
rs868166944	genic_upstream_transcript_variant,intron_variant
rs868171636	genic_upstream_transcript_variant,intron_variant
rs868272311	genic_upstream_transcript_variant,intron_variant
rs868280487	intron_variant
rs868282379	genic_upstream_transcript_variant,intron_variant
rs868294475	genic_upstream_transcript_variant,intron_variant,upstream_transcript_variant,2KB_upstream_variant
rs868395705	intron_variant
rs868403794	intron_variant
rs868419564	intron_variant
rs868431935	genic_upstream_transcript_variant,intron_variant
rs868433277	intron_variant
rs868437104	coding_sequence_variant,missense_variant,non_coding_transcript_variant
rs868440205	intron_variant,genic_downstream_transcript_variant
rs868540974	genic_upstream_transcript_variant,intron_variant
rs868575976	intron_variant
rs868665823	genic_upstream_transcript_variant,intron_variant
rs868681924	genic_upstream_transcript_variant,intron_variant
rs868720067	intron_variant
rs868813295	intron_variant,genic_downstream_transcript_variant
rs868820030	genic_upstream_transcript_variant,intron_variant
rs869054681	genic_upstream_transcript_variant,frameshift_variant,coding_sequence_variant,intron_variant,non_coding_transcript_variant
rs869072784	intron_variant,genic_downstream_transcript_variant
rs869074523	intron_variant
rs869084827	genic_upstream_transcript_variant,frameshift_variant,coding_sequence_variant,intron_variant,non_coding_transcript_variant
rs869087957	intron_variant,genic_upstream_transcript_variant,non_coding_transcript_variant,5_prime_UTR_variant
rs869105634	3_prime_UTR_variant,genic_downstream_transcript_variant,non_coding_transcript_variant
rs869108679	intron_variant,genic_downstream_transcript_variant
rs869116751	genic_upstream_transcript_variant,intron_variant
rs869117608	intron_variant
rs869130595	intron_variant
rs869136712	intron_variant,genic_downstream_transcript_variant
rs869144331	intron_variant
rs869157577	genic_upstream_transcript_variant,intron_variant
rs869159251	intron_variant
rs869193290	intron_variant
rs869198656	intron_variant
rs869203467	intron_variant
rs869216735	genic_upstream_transcript_variant,intron_variant
rs869225402	intron_variant,genic_downstream_transcript_variant
rs869226067	frameshift_variant,coding_sequence_variant,genic_downstream_transcript_variant,non_coding_transcript_variant
rs869258875	intron_variant,genic_downstream_transcript_variant
rs869286835	frameshift_variant,coding_sequence_variant,genic_downstream_transcript_variant,non_coding_transcript_variant
rs879027402	genic_upstream_transcript_variant,intron_variant
rs879035111	intron_variant
rs879123272	intron_variant
rs879171843	genic_upstream_transcript_variant,intron_variant
rs879208715	intron_variant
rs879221608	genic_upstream_transcript_variant,intron_variant
rs879269110	intron_variant
rs879274787	intron_variant,genic_downstream_transcript_variant
rs879319935	intron_variant
rs879335257	genic_upstream_transcript_variant,intron_variant
rs879377675	intron_variant
rs879396963	intron_variant
rs879467954	intron_variant

rs879471092	intron_variant
rs879478588	genic_upstream_transcript_variant,intron_variant
rs879517306	genic_upstream_transcript_variant,intron_variant
rs879544290	intron_variant
rs879564380	intron_variant
rs879592861	intron_variant
rs879619622	intron_variant,genic_downstream_transcript_variant
rs879630821	genic_upstream_transcript_variant,intron_variant
rs879657325	genic_upstream_transcript_variant,intron_variant
rs879675122	intron_variant
rs879712674	intron_variant
rs879738827	intron_variant,genic_downstream_transcript_variant
rs879744409	intron_variant
rs879790658	genic_upstream_transcript_variant,intron_variant
rs879825420	genic_upstream_transcript_variant,intron_variant
rs879866507	genic_upstream_transcript_variant,intron_variant
rs879888437	genic_upstream_transcript_variant,intron_variant
rs879896121	intron_variant
rs879919011	intron_variant
rs879925029	intron_variant
rs879938776	genic_upstream_transcript_variant,intron_variant
rs886072095	intron_variant,genic_downstream_transcript_variant
rs886074868	intron_variant
rs886080901	genic_upstream_transcript_variant,intron_variant
rs886123579	genic_upstream_transcript_variant,intron_variant
rs886176993	intron_variant,genic_downstream_transcript_variant
rs886181716	intron_variant
rs886215714	genic_upstream_transcript_variant,intron_variant
rs886229149	intron_variant
rs886250431	genic_upstream_transcript_variant,intron_variant
rs886295354	intron_variant
rs886303991	genic_upstream_transcript_variant,intron_variant
rs886312940	intron_variant,genic_downstream_transcript_variant
rs886317880	intron_variant
rs886342773	genic_upstream_transcript_variant,intron_variant
rs886356161	genic_upstream_transcript_variant,intron_variant
rs886361595	genic_upstream_transcript_variant,intron_variant
rs886371784	intron_variant
rs886437993	intron_variant
rs886439423	intron_variant,genic_downstream_transcript_variant
rs886446484	intron_variant
rs886455405	genic_upstream_transcript_variant,intron_variant
rs886546761	intron_variant,genic_downstream_transcript_variant
rs886566300	intron_variant
rs886567640	intron_variant
rs886576742	intron_variant
rs886581050	genic_upstream_transcript_variant,intron_variant
rs886588667	3_prime_UTR_variant,genic_downstream_transcript_variant,non_coding_transcript_variant
rs886620822	intron_variant
rs886655993	genic_upstream_transcript_variant,intron_variant
rs886671812	intron_variant
rs886692105	intron_variant,genic_downstream_transcript_variant
rs886692947	intron_variant,genic_downstream_transcript_variant
rs886791647	genic_upstream_transcript_variant,intron_variant
rs886823911	genic_upstream_transcript_variant,intron_variant
rs886843093	genic_upstream_transcript_variant,intron_variant
rs886903536	genic_upstream_transcript_variant,intron_variant
rs886912063	intron_variant,genic_downstream_transcript_variant
rs886924048	intron_variant
rs886992750	genic_upstream_transcript_variant,intron_variant
rs887040547	intron_variant,genic_downstream_transcript_variant
rs887052875	intron_variant,genic_downstream_transcript_variant
rs887057655	intron_variant
rs887075884	genic_upstream_transcript_variant,intron_variant
rs887096404	genic_upstream_transcript_variant,intron_variant
rs887101028	intron_variant
rs887110929	intron_variant,genic_downstream_transcript_variant
rs887165629	intron_variant
rs887176980	genic_upstream_transcript_variant,intron_variant
rs887177657	genic_upstream_transcript_variant,intron_variant
rs887185927	intron_variant,genic_downstream_transcript_variant
rs887209390	genic_upstream_transcript_variant,intron_variant
rs887210701	genic_upstream_transcript_variant,intron_variant
rs887243379	intron_variant,genic_downstream_transcript_variant

rs887253567	intron_variant
rs887272521	genic_upstream_transcript_variant,intron_variant
rs887300951	genic_upstream_transcript_variant,intron_variant
rs887333144	intron_variant
rs887387928	genic_upstream_transcript_variant,intron_variant
rs887445250	intron_variant,genic_downstream_transcript_variant
rs887456020	genic_upstream_transcript_variant,intron_variant
rs887482366	intron_variant
rs887508558	genic_upstream_transcript_variant,intron_variant
rs887545245	genic_upstream_transcript_variant,intron_variant
rs887580738	intron_variant
rs887587790	3_prime_UTR_variant,genic_downstream_transcript_variant,non_coding_transcript_variant
rs887620764	intron_variant
rs887648898	genic_upstream_transcript_variant,intron_variant,upstream_transcript_variant,2KB_upstream_variant
rs887665984	intron_variant
rs887668431	genic_upstream_transcript_variant,intron_variant,upstream_transcript_variant,2KB_upstream_variant
rs887677809	intron_variant
rs887683613	intron_variant,genic_downstream_transcript_variant
rs887691771	genic_upstream_transcript_variant,intron_variant
rs887752586	genic_upstream_transcript_variant,intron_variant
rs887798532	intron_variant
rs887837147	intron_variant,genic_downstream_transcript_variant
rs887838194	intron_variant
rs887845906	genic_upstream_transcript_variant,intron_variant
rs887868112	intron_variant,genic_downstream_transcript_variant
rs887869085	intron_variant
rs887897981	genic_upstream_transcript_variant,intron_variant
rs887899506	500B_downstream_variant,downstream_transcript_variant
rs887907114	genic_upstream_transcript_variant,intron_variant
rs887913165	intron_variant,upstream_transcript_variant,genic_upstream_transcript_variant,2KB_upstream_variant
rs887935416	genic_upstream_transcript_variant,intron_variant
rs887964113	intron_variant
rs887976119	genic_upstream_transcript_variant,intron_variant
rs887977560	intron_variant,upstream_transcript_variant,genic_upstream_transcript_variant,2KB_upstream_variant
rs887999896	intron_variant
rs888054716	genic_upstream_transcript_variant,intron_variant
rs888054855	intron_variant,upstream_transcript_variant,genic_upstream_transcript_variant,2KB_upstream_variant
rs888077879	intron_variant,genic_upstream_transcript_variant,non_coding_transcript_variant,5_prime_UTR_variant
rs888088498	genic_upstream_transcript_variant,intron_variant
rs888105602	downstream_transcript_variant,intron_variant,genic_downstream_transcript_variant
rs888108138	intron_variant
rs888147695	intron_variant,genic_downstream_transcript_variant
rs888179479	genic_upstream_transcript_variant,intron_variant
rs888272251	genic_upstream_transcript_variant,intron_variant
rs888287405	intron_variant
rs888303496	upstream_transcript_variant,intron_variant,genic_upstream_transcript_variant
rs888370177	intron_variant
rs888382312	intron_variant,upstream_transcript_variant,genic_upstream_transcript_variant,5_prime_UTR_variant
rs888387477	genic_upstream_transcript_variant,intron_variant
rs888419274	intron_variant,genic_downstream_transcript_variant
rs888420076	intron_variant
rs888450154	intron_variant,genic_downstream_transcript_variant
rs888466820	genic_upstream_transcript_variant,intron_variant
rs888467672	genic_upstream_transcript_variant,intron_variant
rs888498754	genic_upstream_transcript_variant,intron_variant
rs888516366	intron_variant,genic_downstream_transcript_variant
rs888532449	genic_upstream_transcript_variant,intron_variant,upstream_transcript_variant,2KB_upstream_variant
rs888563497	genic_upstream_transcript_variant,intron_variant,upstream_transcript_variant,2KB_upstream_variant
rs888573945	genic_upstream_transcript_variant,intron_variant
rs888600207	genic_upstream_transcript_variant,intron_variant
rs888625138	genic_upstream_transcript_variant,intron_variant
rs888636227	intron_variant
rs888651810	intron_variant,genic_downstream_transcript_variant
rs888677055	intron_variant,genic_downstream_transcript_variant
rs888683781	genic_upstream_transcript_variant,intron_variant
rs888700380	intron_variant,genic_downstream_transcript_variant
rs888736626	intron_variant
rs888746872	intron_variant
rs888759986	intron_variant,genic_downstream_transcript_variant
rs888797855	intron_variant
rs888799811	genic_upstream_transcript_variant,intron_variant
rs888849165	intron_variant
rs888853473	genic_upstream_transcript_variant,intron_variant
rs888854599	genic_upstream_transcript_variant,intron_variant

rs888857399	intron_variant
rs888858174	genic_upstream_transcript_variant,intron_variant
rs888882642	intron_variant,upstream_transcript_variant,genic_upstream_transcript_variant,2KB_upstream_variant
rs888912740	intron_variant,genic_downstream_transcript_variant
rs888926684	genic_upstream_transcript_variant,intron_variant
rs888940958	intron_variant
rs888994407	intron_variant
rs888997986	intron_variant
rs888999092	genic_upstream_transcript_variant,intron_variant,upstream_transcript_variant,2KB_upstream_variant
rs889017806	intron_variant,genic_downstream_transcript_variant
rs889062723	intron_variant,genic_downstream_transcript_variant
rs889073790	intron_variant,upstream_transcript_variant,genic_upstream_transcript_variant,2KB_upstream_variant
rs889109680	genic_upstream_transcript_variant,intron_variant
rs889118120	intron_variant
rs889152877	intron_variant,genic_downstream_transcript_variant
rs889210494	intron_variant,upstream_transcript_variant,genic_upstream_transcript_variant,2KB_upstream_variant
rs889222433	genic_upstream_transcript_variant,intron_variant
rs889231475	genic_upstream_transcript_variant,intron_variant
rs889256868	genic_upstream_transcript_variant,intron_variant
rs889266699	intron_variant
rs889337368	intron_variant
rs889399285	genic_upstream_transcript_variant,intron_variant
rs889411033	intron_variant
rs889431980	genic_upstream_transcript_variant,intron_variant
rs889443564	intron_variant
rs889451402	intron_variant
rs889452052	genic_upstream_transcript_variant,intron_variant
rs889502414	intron_variant
rs889506790	intron_variant
rs889559159	genic_upstream_transcript_variant,intron_variant
rs889570819	intron_variant
rs889583106	genic_upstream_transcript_variant,intron_variant
rs889595920	genic_upstream_transcript_variant,intron_variant
rs889613773	intron_variant
rs889669736	intron_variant
rs889698256	genic_upstream_transcript_variant,intron_variant
rs889734250	genic_upstream_transcript_variant,intron_variant
rs889765925	genic_upstream_transcript_variant,intron_variant
rs889784391	intron_variant,genic_downstream_transcript_variant
rs889797752	genic_upstream_transcript_variant,intron_variant,upstream_transcript_variant,2KB_upstream_variant
rs889822185	intron_variant
rs889827718	genic_upstream_transcript_variant,intron_variant,upstream_transcript_variant,2KB_upstream_variant
rs889843839	intron_variant
rs889852846	intron_variant
rs889864266	genic_upstream_transcript_variant,intron_variant
rs889894529	intron_variant,genic_downstream_transcript_variant
rs889896751	genic_upstream_transcript_variant,intron_variant
rs889915354	intron_variant
rs889919439	genic_upstream_transcript_variant,intron_variant
rs889933816	genic_upstream_transcript_variant,intron_variant
rs889943721	intron_variant,genic_downstream_transcript_variant
rs889959005	genic_upstream_transcript_variant,intron_variant
rs889991572	intron_variant
rs889992223	intron_variant,genic_downstream_transcript_variant
rs890045963	intron_variant,genic_downstream_transcript_variant
rs890088057	genic_upstream_transcript_variant,intron_variant
rs890113099	genic_upstream_transcript_variant,intron_variant
rs890117771	intron_variant,genic_downstream_transcript_variant
rs890171858	intron_variant
rs890173280	genic_upstream_transcript_variant,intron_variant
rs890175917	intron_variant
rs890178943	genic_upstream_transcript_variant,intron_variant
rs890221652	intron_variant,genic_downstream_transcript_variant
rs890222039	genic_upstream_transcript_variant,intron_variant
rs890252723	intron_variant
rs890278002	intron_variant
rs890289845	genic_upstream_transcript_variant,intron_variant
rs890347972	3_prime_UTR_variant,genic_downstream_transcript_variant,non_coding_transcript_variant
rs890403289	intron_variant
rs890404541	genic_upstream_transcript_variant,intron_variant
rs890429843	intron_variant,genic_downstream_transcript_variant
rs890432270	intron_variant
rs890478435	upstream_transcript_variant,intron_variant,genic_upstream_transcript_variant
rs890488793	genic_upstream_transcript_variant,intron_variant

rs890516324	genic_upstream_transcript_variant,intron_variant
rs890519904	intron_variant,genic_downstream_transcript_variant
rs890541365	genic_upstream_transcript_variant,intron_variant
rs890555557	intron_variant,genic_upstream_transcript_variant,non_coding_transcript_variant,5_prime_UTR_variant
rs890569259	genic_upstream_transcript_variant,intron_variant
rs890595991	intron_variant,genic_downstream_transcript_variant
rs890643346	intron_variant
rs890656470	intron_variant,genic_downstream_transcript_variant
rs890662930	intron_variant
rs890671010	intron_variant
rs890677955	intron_variant
rs890683176	genic_upstream_transcript_variant,intron_variant
rs890684616	intron_variant,genic_downstream_transcript_variant
rs890690376	genic_upstream_transcript_variant,intron_variant
rs890747495	intron_variant,genic_downstream_transcript_variant
rs890798182	intron_variant
rs890810332	genic_upstream_transcript_variant,intron_variant
rs890833905	genic_upstream_transcript_variant,intron_variant
rs890905093	genic_upstream_transcript_variant,intron_variant
rs890925095	genic_upstream_transcript_variant,intron_variant
rs890942044	intron_variant
rs890962514	intron_variant
rs890992365	intron_variant
rs890999910	intron_variant,genic_downstream_transcript_variant
rs891013667	genic_upstream_transcript_variant,intron_variant
rs891014955	intron_variant,genic_downstream_transcript_variant
rs891016314	intron_variant
rs891016495	intron_variant
rs891025262	intron_variant,genic_downstream_transcript_variant
rs891026993	genic_upstream_transcript_variant,intron_variant
rs891088493	genic_upstream_transcript_variant,intron_variant
rs891099852	genic_upstream_transcript_variant,intron_variant
rs891147991	genic_upstream_transcript_variant,intron_variant
rs891182363	intron_variant
rs891196809	genic_upstream_transcript_variant,intron_variant
rs891198886	intron_variant
rs891250779	genic_upstream_transcript_variant,intron_variant
rs891266803	intron_variant
rs891327100	genic_upstream_transcript_variant,intron_variant
rs891333383	genic_upstream_transcript_variant,intron_variant
rs891334937	intron_variant
rs891405668	intron_variant,genic_downstream_transcript_variant
rs891435131	intron_variant,genic_downstream_transcript_variant
rs891436599	intron_variant
rs891459978	genic_upstream_transcript_variant,intron_variant
rs891471383	genic_upstream_transcript_variant,intron_variant
rs891473588	intron_variant
rs891521372	intron_variant,genic_downstream_transcript_variant
rs891523396	intron_variant
rs891549159	genic_upstream_transcript_variant,intron_variant
rs891588063	genic_upstream_transcript_variant,intron_variant
rs891610964	genic_upstream_transcript_variant,intron_variant
rs891623260	intron_variant
rs891634935	coding_sequence_variant,missense_variant,non_coding_transcript_variant
rs891641030	genic_upstream_transcript_variant,intron_variant
rs891679501	genic_upstream_transcript_variant,intron_variant
rs891701280	genic_upstream_transcript_variant,intron_variant
rs891730580	intron_variant
rs891737446	intron_variant,genic_downstream_transcript_variant
rs891755369	genic_upstream_transcript_variant,intron_variant
rs891769620	genic_upstream_transcript_variant,intron_variant
rs891805785	intron_variant
rs891838671	intron_variant
rs891844831	intron_variant
rs891867356	genic_upstream_transcript_variant,intron_variant
rs891880304	genic_upstream_transcript_variant,intron_variant
rs891898944	intron_variant
rs891933826	intron_variant,genic_downstream_transcript_variant
rs891935155	genic_upstream_transcript_variant,intron_variant
rs891947642	intron_variant
rs891952782	intron_variant
rs892008629	genic_upstream_transcript_variant,intron_variant
rs892108517	genic_upstream_transcript_variant,intron_variant
rs892169924	intron_variant

rs892211396	genic_upstream_transcript_variant,intron_variant
rs892266201	intron_variant,genic_downstream_transcript_variant
rs892285217	intron_variant,5_prime_UTR_variant
rs892285465	intron_variant
rs892289772	coding_sequence_variant,intron_variant,missense_variant
rs892331789	upstream_transcript_variant,intron_variant,genic_upstream_transcript_variant
rs892377399	downstream_transcript_variant,intron_variant,genic_downstream_transcript_variant
rs892407154	intron_variant,genic_downstream_transcript_variant
rs892425911	intron_variant
rs892441506	3_prime_UTR_variant,genic_downstream_transcript_variant,non_coding_transcript_variant
rs892473594	intron_variant
rs892474045	3_prime_UTR_variant,genic_downstream_transcript_variant,non_coding_transcript_variant
rs892515817	genic_upstream_transcript_variant,intron_variant
rs892532055	intron_variant
rs892542970	intron_variant,genic_downstream_transcript_variant
rs892605220	genic_upstream_transcript_variant,intron_variant
rs892691547	intron_variant
rs892711157	intron_variant,genic_downstream_transcript_variant
rs892742402	intron_variant,genic_downstream_transcript_variant
rs892749172	genic_upstream_transcript_variant,intron_variant,upstream_transcript_variant,2KB_upstream_variant
rs892802075	genic_upstream_transcript_variant,intron_variant
rs892858706	intron_variant
rs892861856	3_prime_UTR_variant,genic_downstream_transcript_variant,non_coding_transcript_variant
rs892871218	intron_variant,genic_downstream_transcript_variant
rs892910259	genic_upstream_transcript_variant,intron_variant
rs892911514	intron_variant
rs892921324	intron_variant
rs892943505	genic_upstream_transcript_variant,intron_variant
rs892946152	genic_upstream_transcript_variant,intron_variant
rs892977948	genic_upstream_transcript_variant,intron_variant
rs893001173	intron_variant
rs893025943	genic_upstream_transcript_variant,intron_variant
rs893026003	intron_variant
rs893030103	intron_variant,genic_downstream_transcript_variant
rs893093238	genic_upstream_transcript_variant,intron_variant
rs893111523	intron_variant
rs893138184	genic_upstream_transcript_variant,intron_variant
rs893196174	intron_variant
rs893201827	intron_variant,genic_downstream_transcript_variant
rs893205485	intron_variant
rs893207545	intron_variant,genic_downstream_transcript_variant
rs893209864	genic_upstream_transcript_variant,intron_variant
rs893249038	intron_variant,genic_downstream_transcript_variant
rs893256157	intron_variant
rs893281106	genic_upstream_transcript_variant,intron_variant
rs893303218	intron_variant,genic_downstream_transcript_variant
rs893349124	genic_upstream_transcript_variant,intron_variant
rs893390307	genic_upstream_transcript_variant,intron_variant
rs893409118	intron_variant,genic_downstream_transcript_variant
rs893410600	intron_variant,genic_downstream_transcript_variant
rs893414311	intron_variant
rs893419769	genic_upstream_transcript_variant,intron_variant
rs893423433	intron_variant
rs893478593	500B_downstream_variant,downstream_transcript_variant
rs893484454	intron_variant
rs893539368	genic_upstream_transcript_variant,intron_variant
rs893540344	intron_variant
rs893551535	intron_variant,genic_downstream_transcript_variant
rs893559509	genic_upstream_transcript_variant,intron_variant
rs893627786	intron_variant,genic_downstream_transcript_variant
rs893631480	genic_upstream_transcript_variant,intron_variant
rs893724651	intron_variant
rs893737485	intron_variant
rs893754828	genic_upstream_transcript_variant,intron_variant,upstream_transcript_variant,2KB_upstream_variant
rs893784444	intron_variant,genic_downstream_transcript_variant
rs893800782	genic_upstream_transcript_variant,intron_variant
rs893806462	intron_variant
rs893807592	intron_variant
rs893808973	genic_upstream_transcript_variant,intron_variant
rs893831772	genic_upstream_transcript_variant,intron_variant
rs893842563	intron_variant,upstream_transcript_variant,genic_upstream_transcript_variant,2KB_upstream_variant
rs893842717	genic_upstream_transcript_variant,intron_variant
rs893884267	genic_upstream_transcript_variant,intron_variant
rs893888476	intron_variant

rs893925429	genic_upstream_transcript_variant,intron_variant
rs893937972	intron_variant,genic_downstream_transcript_variant
rs893967059	intron_variant
rs893997368	intron_variant
rs894007064	intron_variant
rs894016433	intron_variant
rs894017077	genic_upstream_transcript_variant,intron_variant
rs894046551	genic_upstream_transcript_variant,intron_variant
rs894047114	intron_variant,genic_downstream_transcript_variant
rs894098921	genic_upstream_transcript_variant,intron_variant
rs894131911	intron_variant
rs894134423	genic_upstream_transcript_variant,intron_variant
rs894150896	intron_variant
rs894167708	genic_upstream_transcript_variant,intron_variant
rs894191835	intron_variant
rs894208993	genic_upstream_transcript_variant,intron_variant
rs894239676	intron_variant,genic_downstream_transcript_variant
rs894272365	genic_upstream_transcript_variant,intron_variant
rs894285626	intron_variant
rs894346103	genic_upstream_transcript_variant,intron_variant
rs894348881	intron_variant
rs894381885	intron_variant
rs894422930	genic_upstream_transcript_variant,intron_variant
rs894445685	intron_variant,genic_downstream_transcript_variant
rs894455729	intron_variant
rs894485658	intron_variant,genic_downstream_transcript_variant
rs894520288	genic_upstream_transcript_variant,intron_variant
rs894555822	intron_variant,genic_downstream_transcript_variant
rs894564102	intron_variant
rs894576616	intron_variant
rs894584478	genic_upstream_transcript_variant,intron_variant
rs894608084	intron_variant,genic_downstream_transcript_variant
rs894644335	intron_variant
rs894655844	intron_variant
rs894694888	intron_variant
rs894705079	intron_variant
rs894714927	intron_variant,genic_downstream_transcript_variant
rs894724273	genic_upstream_transcript_variant,intron_variant
rs894736877	intron_variant
rs894748151	intron_variant,genic_downstream_transcript_variant
rs894756897	genic_upstream_transcript_variant,intron_variant
rs894788991	intron_variant
rs894790011	genic_upstream_transcript_variant,intron_variant
rs894796748	intron_variant
rs894814577	intron_variant,genic_downstream_transcript_variant
rs894857863	intron_variant
rs894863976	genic_upstream_transcript_variant,intron_variant
rs894885981	intron_variant
rs894892909	intron_variant,genic_downstream_transcript_variant
rs894903119	intron_variant
rs894911498	intron_variant
rs894929695	genic_upstream_transcript_variant,intron_variant
rs894943535	intron_variant,genic_downstream_transcript_variant
rs895025402	genic_upstream_transcript_variant,intron_variant,upstream_transcript_variant,2KB_upstream_variant
rs895077470	genic_upstream_transcript_variant,intron_variant
rs895079900	intron_variant,genic_downstream_transcript_variant
rs895087563	intron_variant
rs895114198	intron_variant,upstream_transcript_variant,genic_upstream_transcript_variant,2KB_upstream_variant
rs895136433	intron_variant
rs895166228	intron_variant
rs895215214	genic_upstream_transcript_variant,intron_variant
rs895219814	intron_variant,upstream_transcript_variant,genic_upstream_transcript_variant,2KB_upstream_variant
rs895259251	intron_variant
rs895285218	intron_variant,genic_upstream_transcript_variant
rs895331717	intron_variant
rs895350231	intron_variant,genic_upstream_transcript_variant
rs895376991	intron_variant,genic_upstream_transcript_variant
rs895386147	intron_variant
rs895391980	intron_variant,genic_upstream_transcript_variant
rs895395329	intron_variant,genic_upstream_transcript_variant
rs895429755	intron_variant,genic_upstream_transcript_variant,2KB_upstream_variant,upstream_transcript_variant
rs895430655	intron_variant
rs895450021	intron_variant
rs895458479	intron_variant,genic_downstream_transcript_variant

rs895487842	intron_variant,genic_downstream_transcript_variant
rs895514304	intron_variant,genic_upstream_transcript_variant
rs895518432	intron_variant,genic_upstream_transcript_variant
rs895518635	intron_variant
rs895557219	intron_variant,genic_upstream_transcript_variant,2KB_upstream_variant,upstream_transcript_variant
rs895558647	intron_variant,genic_downstream_transcript_variant
rs895566383	intron_variant,genic_upstream_transcript_variant
rs895578036	intron_variant
rs895601156	intron_variant
rs895601318	intron_variant,genic_upstream_transcript_variant
rs895636369	intron_variant
rs895683840	intron_variant,genic_upstream_transcript_variant
rs895688266	intron_variant,genic_upstream_transcript_variant,upstream_transcript_variant
rs895688428	intron_variant
rs895714991	intron_variant,genic_downstream_transcript_variant
rs895729345	intron_variant
rs895730756	intron_variant,genic_upstream_transcript_variant
rs895749698	intron_variant,genic_upstream_transcript_variant,upstream_transcript_variant
rs895751445	intron_variant
rs895761828	intron_variant,genic_upstream_transcript_variant
rs895804602	intron_variant,genic_downstream_transcript_variant
rs895827393	intron_variant,genic_upstream_transcript_variant
rs895832805	intron_variant
rs895838237	intron_variant,genic_upstream_transcript_variant
rs895957282	intron_variant
rs895962097	intron_variant
rs895989726	intron_variant
rs896001999	intron_variant,genic_upstream_transcript_variant
rs896056241	intron_variant
rs896056683	intron_variant
rs896118975	intron_variant,genic_upstream_transcript_variant
rs896122967	intron_variant
rs896152858	intron_variant,genic_upstream_transcript_variant
rs896160892	intron_variant
rs896162725	intron_variant,genic_upstream_transcript_variant
rs896171399	intron_variant,genic_upstream_transcript_variant
rs896288143	intron_variant,genic_upstream_transcript_variant
rs896292491	intron_variant
rs896294593	intron_variant,genic_upstream_transcript_variant
rs896302251	intron_variant
rs896330128	intron_variant,genic_upstream_transcript_variant
rs896377607	intron_variant,genic_upstream_transcript_variant
rs896391898	intron_variant,genic_upstream_transcript_variant
rs896444215	intron_variant,genic_upstream_transcript_variant
rs896464611	intron_variant
rs896473526	intron_variant
rs896530720	intron_variant,genic_downstream_transcript_variant
rs896532175	intron_variant,genic_downstream_transcript_variant
rs896533829	intron_variant,genic_upstream_transcript_variant
rs896570514	intron_variant,genic_downstream_transcript_variant
rs896577259	intron_variant,genic_upstream_transcript_variant
rs896630434	intron_variant,genic_upstream_transcript_variant
rs896632067	intron_variant,genic_upstream_transcript_variant
rs896669386	intron_variant,genic_downstream_transcript_variant
rs896689895	intron_variant,genic_upstream_transcript_variant
rs896697588	intron_variant,genic_upstream_transcript_variant
rs896701995	intron_variant
rs896704960	intron_variant,genic_upstream_transcript_variant
rs896788906	intron_variant,genic_upstream_transcript_variant
rs896888530	intron_variant,genic_downstream_transcript_variant
rs896888575	intron_variant,genic_upstream_transcript_variant
rs896935661	intron_variant,genic_upstream_transcript_variant
rs896936526	intron_variant,genic_upstream_transcript_variant
rs896965610	intron_variant,genic_upstream_transcript_variant
rs896985799	intron_variant,genic_upstream_transcript_variant
rs897018734	intron_variant
rs897063794	non_coding_transcript_variant,genic_upstream_transcript_variant,intron_variant,5_prime_UTR_variant
rs897088643	intron_variant,genic_upstream_transcript_variant
rs897090701	intron_variant
rs897137527	intron_variant,genic_upstream_transcript_variant
rs897162448	intron_variant,genic_upstream_transcript_variant
rs897183475	intron_variant
rs897327219	intron_variant
rs897341364	intron_variant

rs897391460	intron_variant
rs897400319	intron_variant,genic_downstream_transcript_variant
rs897431331	intron_variant
rs897435995	intron_variant,genic_downstream_transcript_variant
rs897443690	intron_variant
rs897455077	intron_variant
rs897470722	intron_variant,genic_upstream_transcript_variant
rs897488227	intron_variant,genic_downstream_transcript_variant
rs897490087	intron_variant
rs897507479	intron_variant
rs897509622	intron_variant,genic_upstream_transcript_variant
rs897610938	intron_variant
rs897618119	intron_variant,genic_upstream_transcript_variant
rs897626316	intron_variant,genic_upstream_transcript_variant
rs897627566	intron_variant,genic_upstream_transcript_variant
rs897715832	intron_variant
rs897744319	intron_variant,genic_downstream_transcript_variant
rs897751212	intron_variant
rs897768864	intron_variant,genic_downstream_transcript_variant
rs897806829	intron_variant
rs897812678	intron_variant
rs897820211	intron_variant,genic_downstream_transcript_variant
rs897860647	intron_variant
rs897867962	intron_variant,genic_upstream_transcript_variant
rs897872000	intron_variant,genic_upstream_transcript_variant,upstream_transcript_variant
rs897891127	intron_variant,genic_upstream_transcript_variant
rs897929540	intron_variant,genic_upstream_transcript_variant,upstream_transcript_variant
rs897931699	intron_variant,genic_upstream_transcript_variant
rs897976413	intron_variant
rs897999558	intron_variant
rs898004083	intron_variant,genic_upstream_transcript_variant
rs898021431	intron_variant,genic_upstream_transcript_variant,2KB_upstream_variant,upstream_transcript_variant
rs898054287	intron_variant
rs898147486	intron_variant
rs898158515	intron_variant,genic_downstream_transcript_variant
rs898175177	intron_variant,genic_upstream_transcript_variant
rs898267104	intron_variant,genic_upstream_transcript_variant
rs898279202	intron_variant
rs898283368	intron_variant,genic_upstream_transcript_variant
rs898294039	intron_variant,genic_upstream_transcript_variant
rs898299642	intron_variant,genic_downstream_transcript_variant
rs898323747	intron_variant
rs898371833	intron_variant,genic_downstream_transcript_variant
rs898399998	intron_variant
rs898414133	intron_variant,genic_upstream_transcript_variant
rs898433248	intron_variant,genic_downstream_transcript_variant
rs898506034	intron_variant,genic_upstream_transcript_variant
rs898531139	intron_variant
rs898537401	intron_variant,genic_downstream_transcript_variant
rs898554650	intron_variant
rs898557340	intron_variant,genic_upstream_transcript_variant
rs898588297	intron_variant,genic_downstream_transcript_variant
rs898625183	intron_variant
rs898628477	intron_variant,genic_upstream_transcript_variant
rs898663810	intron_variant
rs898666331	intron_variant,genic_upstream_transcript_variant
rs898698321	intron_variant,genic_upstream_transcript_variant
rs898719692	intron_variant
rs898764682	intron_variant,genic_upstream_transcript_variant
rs898772854	intron_variant
rs898808142	intron_variant,genic_upstream_transcript_variant
rs898856273	intron_variant,genic_downstream_transcript_variant
rs898932029	intron_variant,genic_upstream_transcript_variant
rs898999778	intron_variant,genic_upstream_transcript_variant
rs899029898	intron_variant,genic_upstream_transcript_variant
rs899055574	intron_variant
rs899082939	intron_variant,genic_upstream_transcript_variant
rs899088190	intron_variant
rs899105485	intron_variant,genic_upstream_transcript_variant
rs899128677	intron_variant
rs899183061	intron_variant,genic_upstream_transcript_variant
rs899240957	intron_variant,genic_downstream_transcript_variant
rs899271927	intron_variant,genic_downstream_transcript_variant
rs899278962	intron_variant,genic_upstream_transcript_variant

rs899291261	intron_variant,genic_upstream_transcript_variant
rs899302009	intron_variant
rs899307459	intron_variant,genic_upstream_transcript_variant
rs899339823	intron_variant,genic_upstream_transcript_variant
rs899363561	intron_variant
rs899364998	intron_variant,genic_downstream_transcript_variant
rs899418615	intron_variant,genic_downstream_transcript_variant
rs899424650	3_prime_UTR_variant,non_coding_transcript_variant,genic_downstream_transcript_variant
rs899501593	intron_variant
rs899507118	intron_variant,genic_downstream_transcript_variant,downstream_transcript_variant
rs899518261	intron_variant,genic_upstream_transcript_variant
rs899569508	synonymous_variant,coding_sequence_variant,genic_upstream_transcript_variant,non_coding_transcript_variant,intron_variant
rs899571662	intron_variant,genic_upstream_transcript_variant
rs899576456	intron_variant
rs899610698	intron_variant,genic_upstream_transcript_variant
rs899641729	intron_variant,genic_downstream_transcript_variant
rs899652025	intron_variant
rs899656436	intron_variant,genic_upstream_transcript_variant
rs899662107	intron_variant,genic_upstream_transcript_variant
rs899668490	intron_variant,genic_downstream_transcript_variant
rs899693263	intron_variant,genic_upstream_transcript_variant
rs899713873	intron_variant,genic_downstream_transcript_variant
rs899742969	intron_variant
rs899754998	intron_variant
rs899773615	intron_variant,genic_downstream_transcript_variant
rs899790345	intron_variant,genic_upstream_transcript_variant
rs899794247	intron_variant,genic_upstream_transcript_variant
rs899860623	intron_variant
rs899910016	intron_variant
rs899935863	intron_variant,genic_upstream_transcript_variant
rs899964049	intron_variant
rs900006049	intron_variant,genic_upstream_transcript_variant
rs900015366	intron_variant
rs900017008	intron_variant
rs900048387	intron_variant
rs900125386	intron_variant,genic_upstream_transcript_variant
rs900163055	intron_variant
rs900170028	intron_variant,genic_upstream_transcript_variant
rs900192245	intron_variant
rs900210693	3_prime_UTR_variant,non_coding_transcript_variant,genic_downstream_transcript_variant
rs900290843	intron_variant,genic_upstream_transcript_variant
rs900324263	intron_variant
rs900348407	intron_variant,genic_upstream_transcript_variant
rs900355310	intron_variant
rs900401385	intron_variant,genic_upstream_transcript_variant
rs900440143	intron_variant,genic_upstream_transcript_variant,2KB_upstream_variant,upstream_transcript_variant
rs900440967	intron_variant
rs900451980	intron_variant,genic_upstream_transcript_variant
rs900452351	intron_variant
rs900481885	intron_variant,genic_upstream_transcript_variant
rs900514028	intron_variant
rs900524496	intron_variant,genic_downstream_transcript_variant
rs900572051	intron_variant,genic_downstream_transcript_variant
rs900602771	intron_variant,genic_downstream_transcript_variant
rs900606479	intron_variant,genic_upstream_transcript_variant
rs900608849	intron_variant
rs900654174	intron_variant
rs900709168	intron_variant
rs900716689	intron_variant,genic_downstream_transcript_variant
rs900772316	intron_variant,genic_downstream_transcript_variant
rs900776907	intron_variant
rs900817628	intron_variant,genic_upstream_transcript_variant
rs900825728	intron_variant,genic_downstream_transcript_variant
rs900833348	intron_variant
rs900849971	intron_variant,genic_upstream_transcript_variant
rs900858371	intron_variant,genic_downstream_transcript_variant
rs900859752	intron_variant,genic_downstream_transcript_variant
rs900885021	intron_variant
rs900894305	intron_variant,genic_upstream_transcript_variant
rs900979731	intron_variant,genic_upstream_transcript_variant
rs901007056	intron_variant,genic_upstream_transcript_variant
rs901022467	intron_variant
rs901031881	intron_variant,genic_upstream_transcript_variant
rs901043345	intron_variant,genic_upstream_transcript_variant,2KB_upstream_variant,upstream_transcript_variant

rs901089773	intron_variant,genic_downstream_transcript_variant
rs901124250	intron_variant
rs901183200	intron_variant,genic_downstream_transcript_variant
rs901189268	intron_variant,genic_upstream_transcript_variant,2KB_upstream_variant,upstream_transcript_variant
rs901198338	intron_variant
rs901207191	intron_variant
rs901242451	intron_variant,genic_downstream_transcript_variant
rs901245956	intron_variant,genic_upstream_transcript_variant
rs901246074	intron_variant,genic_upstream_transcript_variant
rs901261281	intron_variant
rs901261630	intron_variant,genic_upstream_transcript_variant
rs901277205	intron_variant,genic_upstream_transcript_variant
rs901317043	intron_variant,genic_upstream_transcript_variant
rs901336465	intron_variant,genic_upstream_transcript_variant,2KB_upstream_variant,upstream_transcript_variant
rs901348543	intron_variant,genic_upstream_transcript_variant
rs901366158	intron_variant,genic_upstream_transcript_variant,2KB_upstream_variant,upstream_transcript_variant
rs901378121	intron_variant
rs901382244	intron_variant,genic_downstream_transcript_variant
rs901391802	intron_variant,genic_downstream_transcript_variant
rs901421211	intron_variant,genic_upstream_transcript_variant
rs901428552	intron_variant,genic_upstream_transcript_variant
rs901437377	intron_variant
rs901480936	intron_variant,genic_downstream_transcript_variant
rs901501300	intron_variant
rs901505996	intron_variant,genic_downstream_transcript_variant
rs901536116	intron_variant
rs901556439	intron_variant,genic_upstream_transcript_variant
rs901572810	intron_variant,genic_downstream_transcript_variant
rs901586899	intron_variant
rs901589784	intron_variant,genic_upstream_transcript_variant
rs901607487	intron_variant,genic_upstream_transcript_variant
rs901609817	intron_variant
rs901619953	intron_variant,genic_upstream_transcript_variant,2KB_upstream_variant,upstream_transcript_variant
rs901647513	intron_variant,genic_upstream_transcript_variant
rs901661095	intron_variant
rs901676247	intron_variant
rs901678598	intron_variant,genic_upstream_transcript_variant
rs901711970	intron_variant
rs901717326	intron_variant,genic_downstream_transcript_variant
rs901725755	intron_variant,genic_downstream_transcript_variant
rs901736812	intron_variant,genic_upstream_transcript_variant
rs901765923	intron_variant,genic_upstream_transcript_variant
rs901778019	intron_variant,genic_downstream_transcript_variant
rs901844506	intron_variant
rs901865131	intron_variant,genic_upstream_transcript_variant
rs901913069	intron_variant,genic_upstream_transcript_variant
rs901922058	intron_variant
rs901963499	3_prime_UTR_variant,non_coding_transcript_variant,genic_downstream_transcript_variant
rs901963783	intron_variant,genic_upstream_transcript_variant
rs901964224	intron_variant,genic_upstream_transcript_variant
rs901975889	intron_variant
rs902001994	intron_variant,genic_upstream_transcript_variant
rs902013283	3_prime_UTR_variant,non_coding_transcript_variant,genic_downstream_transcript_variant
rs902085081	intron_variant,genic_upstream_transcript_variant,2KB_upstream_variant,upstream_transcript_variant
rs902086015	intron_variant,genic_downstream_transcript_variant
rs902092978	intron_variant
rs902103215	intron_variant
rs902115606	intron_variant,genic_upstream_transcript_variant,2KB_upstream_variant,upstream_transcript_variant
rs902118572	intron_variant,genic_downstream_transcript_variant
rs902129100	intron_variant
rs902138043	intron_variant,genic_upstream_transcript_variant
rs902173545	intron_variant,genic_upstream_transcript_variant,2KB_upstream_variant,upstream_transcript_variant
rs902179662	intron_variant
rs902186200	intron_variant,genic_upstream_transcript_variant
rs902201596	intron_variant
rs902273826	intron_variant,genic_upstream_transcript_variant
rs902309427	intron_variant,genic_upstream_transcript_variant
rs902316258	intron_variant
rs902341182	intron_variant,genic_upstream_transcript_variant
rs902376341	intron_variant,genic_downstream_transcript_variant
rs902473210	intron_variant
rs902532363	intron_variant,genic_upstream_transcript_variant
rs902533713	intron_variant
rs902539970	intron_variant,genic_upstream_transcript_variant

rs902550966	intron_variant,genic_downstream_transcript_variant
rs902559359	intron_variant
rs902570217	intron_variant,genic_downstream_transcript_variant
rs902580518	intron_variant,genic_upstream_transcript_variant
rs902606551	intron_variant,genic_downstream_transcript_variant
rs902611764	intron_variant,genic_upstream_transcript_variant
rs902621951	intron_variant,genic_upstream_transcript_variant,2KB_upstream_variant,upstream_transcript_variant
rs902715676	intron_variant
rs902734745	intron_variant
rs902743840	intron_variant,genic_upstream_transcript_variant
rs902753986	intron_variant,genic_downstream_transcript_variant
rs902780925	intron_variant,genic_upstream_transcript_variant
rs902783024	intron_variant,genic_upstream_transcript_variant
rs902799906	intron_variant
rs902862430	intron_variant,genic_upstream_transcript_variant
rs902884302	intron_variant,genic_downstream_transcript_variant
rs902888340	intron_variant,genic_upstream_transcript_variant,upstream_transcript_variant
rs902900948	intron_variant,genic_downstream_transcript_variant
rs902935683	intron_variant,genic_upstream_transcript_variant,2KB_upstream_variant,upstream_transcript_variant
rs902936792	intron_variant,genic_downstream_transcript_variant
rs902940348	intron_variant,genic_upstream_transcript_variant
rs902967673	intron_variant
rs902996863	intron_variant
rs903029231	intron_variant,genic_downstream_transcript_variant
rs903039204	intron_variant,genic_downstream_transcript_variant
rs903053147	intron_variant
rs903056150	intron_variant,genic_upstream_transcript_variant
rs903056437	intron_variant,genic_upstream_transcript_variant
rs903086609	intron_variant,genic_upstream_transcript_variant
rs903120297	non_coding_transcript_variant,genic_upstream_transcript_variant,intron_variant,5_prime_UTR_variant
rs903127125	intron_variant,genic_upstream_transcript_variant,upstream_transcript_variant
rs903157711	intron_variant,genic_upstream_transcript_variant
rs903168256	intron_variant,genic_downstream_transcript_variant
rs903231460	intron_variant,genic_upstream_transcript_variant
rs903256594	intron_variant
rs903307026	intron_variant,genic_downstream_transcript_variant
rs903318335	intron_variant,genic_downstream_transcript_variant
rs903331035	intron_variant
rs903331587	intron_variant
rs903367021	intron_variant,genic_upstream_transcript_variant
rs903422061	intron_variant,genic_upstream_transcript_variant
rs903426131	intron_variant
rs903442983	intron_variant,genic_upstream_transcript_variant
rs903447741	intron_variant
rs903454693	intron_variant,genic_downstream_transcript_variant
rs903478953	intron_variant
rs903484078	intron_variant,genic_downstream_transcript_variant
rs903530192	intron_variant,genic_upstream_transcript_variant,2KB_upstream_variant,upstream_transcript_variant
rs903568106	intron_variant
rs903579560	intron_variant,genic_downstream_transcript_variant
rs903594554	intron_variant
rs903598316	intron_variant,genic_upstream_transcript_variant
rs903612109	intron_variant,genic_downstream_transcript_variant
rs903619786	intron_variant,genic_downstream_transcript_variant
rs903625267	intron_variant,genic_upstream_transcript_variant
rs903645501	intron_variant
rs903657975	intron_variant,genic_upstream_transcript_variant
rs903691765	intron_variant,genic_upstream_transcript_variant
rs903709523	intron_variant
rs903721711	intron_variant,genic_downstream_transcript_variant
rs903736538	intron_variant
rs903750104	intron_variant,genic_upstream_transcript_variant
rs903752804	intron_variant,genic_upstream_transcript_variant
rs903756538	intron_variant
rs903808434	intron_variant
rs903821612	intron_variant,genic_upstream_transcript_variant
rs903826950	intron_variant
rs903846931	intron_variant
rs903852843	intron_variant,genic_upstream_transcript_variant
rs903883640	intron_variant,genic_upstream_transcript_variant
rs903887373	intron_variant,genic_downstream_transcript_variant
rs903915262	intron_variant,genic_upstream_transcript_variant
rs903945350	intron_variant,genic_upstream_transcript_variant
rs903983996	intron_variant

rs904015346	intron_variant,genic_upstream_transcript_variant
rs904031307	intron_variant
rs904048222	intron_variant,genic_upstream_transcript_variant
rs904096948	intron_variant
rs904123077	intron_variant,genic_downstream_transcript_variant
rs904149010	intron_variant
rs904191799	intron_variant
rs904246726	intron_variant,genic_upstream_transcript_variant
rs904280419	intron_variant,genic_upstream_transcript_variant
rs904320711	intron_variant,genic_downstream_transcript_variant
rs904338459	intron_variant
rs904353109	intron_variant,genic_upstream_transcript_variant
rs904369089	intron_variant
rs904384409	intron_variant,genic_downstream_transcript_variant
rs904430680	intron_variant
rs904453227	intron_variant
rs904454221	intron_variant,genic_downstream_transcript_variant
rs904457791	intron_variant,genic_downstream_transcript_variant
rs904463381	intron_variant
rs904498286	intron_variant
rs904514538	intron_variant,genic_upstream_transcript_variant
rs904524372	intron_variant,5_prime_UTR_variant
rs904554523	intron_variant
rs904559594	synonymous_variant,coding_sequence_variant,non_coding_transcript_variant
rs904598041	intron_variant,genic_upstream_transcript_variant
rs904607621	intron_variant,genic_upstream_transcript_variant
rs904633047	intron_variant,genic_upstream_transcript_variant
rs904637920	intron_variant,genic_upstream_transcript_variant
rs904654047	intron_variant,genic_upstream_transcript_variant
rs904672331	intron_variant,genic_downstream_transcript_variant
rs904680049	intron_variant
rs904738215	intron_variant,genic_downstream_transcript_variant
rs904777214	intron_variant,genic_upstream_transcript_variant
rs904872509	intron_variant,genic_upstream_transcript_variant
rs904879619	intron_variant,genic_upstream_transcript_variant
rs904896510	intron_variant
rs904899025	intron_variant,genic_downstream_transcript_variant
rs904915397	intron_variant
rs904922695	intron_variant,genic_upstream_transcript_variant
rs904951445	intron_variant,genic_downstream_transcript_variant
rs904955909	intron_variant,genic_downstream_transcript_variant
rs904974488	intron_variant,genic_upstream_transcript_variant
rs904995215	intron_variant,genic_upstream_transcript_variant
rs905031520	intron_variant
rs905055278	intron_variant,genic_downstream_transcript_variant,downstream_transcript_variant
rs905056587	intron_variant,genic_upstream_transcript_variant
rs905084944	intron_variant
rs905131578	intron_variant,genic_upstream_transcript_variant
rs905132293	intron_variant,genic_upstream_transcript_variant
rs905167763	intron_variant
rs905175292	intron_variant,genic_downstream_transcript_variant
rs905192814	3_prime_UTR_variant,non_coding_transcript_variant,genic_downstream_transcript_variant
rs905227349	intron_variant,genic_upstream_transcript_variant
rs905262666	intron_variant,genic_downstream_transcript_variant
rs905280191	intron_variant,genic_upstream_transcript_variant
rs905288814	intron_variant,genic_upstream_transcript_variant,upstream_transcript_variant
rs905311101	intron_variant,genic_downstream_transcript_variant
rs905374456	intron_variant,genic_upstream_transcript_variant,2KB_upstream_variant,upstream_transcript_variant
rs905396043	intron_variant
rs905409321	intron_variant
rs905430304	intron_variant
rs905443260	intron_variant,genic_upstream_transcript_variant
rs905460095	intron_variant,genic_downstream_transcript_variant
rs905466828	intron_variant,genic_upstream_transcript_variant,2KB_upstream_variant,upstream_transcript_variant
rs905506485	intron_variant,genic_upstream_transcript_variant
rs905508852	intron_variant,genic_downstream_transcript_variant
rs905567236	intron_variant
rs905574715	intron_variant
rs905599491	intron_variant,genic_downstream_transcript_variant
rs905615798	intron_variant
rs905643748	intron_variant,genic_upstream_transcript_variant
rs905644783	intron_variant,genic_downstream_transcript_variant
rs905658873	intron_variant,genic_downstream_transcript_variant
rs905695443	intron_variant,genic_downstream_transcript_variant

rs905707871	intron_variant,genic_upstream_transcript_variant
rs905733602	intron_variant
rs905762881	intron_variant
rs905782294	intron_variant,genic_upstream_transcript_variant
rs905790118	intron_variant,genic_downstream_transcript_variant
rs905808630	intron_variant
rs905830681	intron_variant,genic_upstream_transcript_variant
rs905831706	intron_variant,genic_upstream_transcript_variant
rs905985177	intron_variant
rs906041033	intron_variant
rs906054424	intron_variant
rs906107221	intron_variant
rs906119396	intron_variant,genic_upstream_transcript_variant
rs906148961	intron_variant,genic_downstream_transcript_variant
rs906151787	intron_variant
rs906173881	intron_variant,genic_upstream_transcript_variant
rs906227434	intron_variant,genic_upstream_transcript_variant
rs906245346	intron_variant,genic_upstream_transcript_variant,2KB_upstream_variant,upstream_transcript_variant
rs906283761	intron_variant
rs906287897	intron_variant,genic_upstream_transcript_variant
rs906310174	intron_variant
rs906324485	intron_variant,genic_downstream_transcript_variant
rs906402014	intron_variant
rs906404083	intron_variant
rs906407678	intron_variant,genic_upstream_transcript_variant
rs906440721	intron_variant,genic_downstream_transcript_variant
rs906460571	intron_variant,genic_upstream_transcript_variant
rs906469324	intron_variant,genic_upstream_transcript_variant
rs906477896	intron_variant
rs906538432	intron_variant,genic_upstream_transcript_variant
rs906631536	intron_variant,genic_downstream_transcript_variant
rs906651609	intron_variant
rs906657752	intron_variant,genic_upstream_transcript_variant
rs906666484	intron_variant,genic_upstream_transcript_variant
rs906688783	intron_variant,genic_upstream_transcript_variant
rs906824291	intron_variant,genic_upstream_transcript_variant
rs906824515	intron_variant
rs906876420	intron_variant,genic_upstream_transcript_variant
rs906888167	intron_variant
rs906892018	intron_variant
rs906975930	intron_variant
rs906980961	intron_variant
rs906995755	genic_upstream_transcript_variant,intron_variant
rs906999846	intron_variant
rs907019694	genic_upstream_transcript_variant,intron_variant
rs907106214	intron_variant
rs907146102	3_prime_UTR_variant,non_coding_transcript_variant,genic_downstream_transcript_variant
rs907153129	500B_downstream_variant,downstream_transcript_variant
rs907156890	intron_variant
rs907204455	genic_upstream_transcript_variant,intron_variant
rs907235903	intron_variant
rs907237269	genic_upstream_transcript_variant,intron_variant
rs907249941	intron_variant
rs907262223	genic_upstream_transcript_variant,intron_variant
rs907266328	genic_downstream_transcript_variant,intron_variant
rs907286172	genic_downstream_transcript_variant,intron_variant
rs907302279	intron_variant
rs907338341	upstream_transcript_variant,genic_upstream_transcript_variant,intron_variant,2KB_upstream_variant
rs907378294	genic_upstream_transcript_variant,intron_variant
rs907409362	genic_upstream_transcript_variant,intron_variant
rs907414490	genic_upstream_transcript_variant,intron_variant
rs907424573	genic_upstream_transcript_variant,intron_variant
rs907426975	intron_variant
rs907474208	upstream_transcript_variant,genic_upstream_transcript_variant,intron_variant,2KB_upstream_variant
rs907515073	genic_upstream_transcript_variant,intron_variant
rs907598998	intron_variant
rs907601369	genic_downstream_transcript_variant,intron_variant
rs907603008	genic_upstream_transcript_variant,intron_variant
rs907615485	genic_upstream_transcript_variant,intron_variant
rs907618252	intron_variant
rs907629560	intron_variant
rs907678968	genic_upstream_transcript_variant,intron_variant
rs907688326	upstream_transcript_variant,2KB_upstream_variant,genic_upstream_transcript_variant,intron_variant
rs907719341	non_coding_transcript_variant,genic_upstream_transcript_variant,intron_variant

rs907743396	intron_variant
rs907779413	genic_upstream_transcript_variant,intron_variant
rs907795502	intron_variant
rs907801427	genic_upstream_transcript_variant,intron_variant
rs907804404	genic_downstream_transcript_variant,intron_variant
rs907812535	genic_upstream_transcript_variant,intron_variant
rs907816733	intron_variant
rs907856338	intron_variant
rs907886371	genic_upstream_transcript_variant,intron_variant
rs907886741	genic_upstream_transcript_variant,intron_variant
rs907892456	upstream_transcript_variant,genic_upstream_transcript_variant,intron_variant,2KB_upstream_variant
rs907897276	genic_downstream_transcript_variant,intron_variant
rs907935348	genic_upstream_transcript_variant,intron_variant
rs907977889	genic_downstream_transcript_variant,intron_variant
rs908027202	intron_variant
rs908102068	intron_variant
rs908124464	3_prime_UTR_variant,non_coding_transcript_variant,genic_downstream_transcript_variant
rs908138585	genic_upstream_transcript_variant,intron_variant
rs908151184	intron_variant
rs908159868	genic_upstream_transcript_variant,intron_variant
rs908168624	genic_upstream_transcript_variant,intron_variant
rs908190254	intron_variant
rs908208664	genic_upstream_transcript_variant,intron_variant
rs908219533	intron_variant
rs908226400	genic_upstream_transcript_variant,intron_variant
rs908232411	genic_downstream_transcript_variant,intron_variant
rs908268728	genic_upstream_transcript_variant,intron_variant
rs908317365	intron_variant
rs908327609	genic_upstream_transcript_variant,intron_variant
rs908361874	intron_variant
rs908399541	genic_upstream_transcript_variant,intron_variant
rs908436555	intron_variant
rs908458153	genic_downstream_transcript_variant,intron_variant
rs908488802	intron_variant
rs908492781	genic_upstream_transcript_variant,intron_variant
rs908511861	genic_downstream_transcript_variant,intron_variant
rs908530457	intron_variant
rs908541205	genic_downstream_transcript_variant,intron_variant
rs908574113	genic_upstream_transcript_variant,intron_variant
rs908584018	intron_variant
rs908587790	genic_downstream_transcript_variant,intron_variant
rs908618872	genic_downstream_transcript_variant,intron_variant
rs908639492	intron_variant
rs908710074	upstream_transcript_variant,genic_upstream_transcript_variant,intron_variant
rs908710942	genic_upstream_transcript_variant,intron_variant
rs908713154	genic_upstream_transcript_variant,intron_variant
rs908725175	intron_variant
rs908786110	genic_downstream_transcript_variant,intron_variant
rs908797866	upstream_transcript_variant,2KB_upstream_variant,genic_upstream_transcript_variant,intron_variant
rs908886592	intron_variant
rs908892041	genic_upstream_transcript_variant,intron_variant
rs908894472	genic_upstream_transcript_variant,intron_variant
rs908945533	genic_upstream_transcript_variant,intron_variant
rs908970471	genic_upstream_transcript_variant,intron_variant
rs908991222	genic_upstream_transcript_variant,intron_variant
rs909050251	intron_variant
rs909088409	genic_downstream_transcript_variant,intron_variant
rs909115462	intron_variant
rs909125563	genic_upstream_transcript_variant,intron_variant
rs909127758	500B_downstream_variant,downstream_transcript_variant
rs909148540	genic_downstream_transcript_variant,intron_variant
rs909205753	intron_variant
rs909206616	genic_upstream_transcript_variant,intron_variant
rs909224645	intron_variant
rs909269103	intron_variant
rs909279414	genic_upstream_transcript_variant,intron_variant
rs909379316	intron_variant
rs909387765	genic_downstream_transcript_variant,intron_variant
rs909401416	intron_variant
rs909407453	genic_downstream_transcript_variant,intron_variant
rs909419666	genic_upstream_transcript_variant,intron_variant
rs909421271	genic_upstream_transcript_variant,intron_variant
rs909468318	upstream_transcript_variant,genic_upstream_transcript_variant,intron_variant,2KB_upstream_variant
rs909470004	genic_upstream_transcript_variant,intron_variant

rs909593064	intron_variant
rs909601201	intron_variant
rs909607387	genic_upstream_transcript_variant,intron_variant
rs909631412	genic_upstream_transcript_variant,intron_variant
rs909668625	genic_upstream_transcript_variant,intron_variant
rs909679918	genic_upstream_transcript_variant,intron_variant
rs909691977	genic_downstream_transcript_variant,intron_variant
rs909726407	genic_downstream_transcript_variant,intron_variant
rs909791844	upstream_transcript_variant,genic_upstream_transcript_variant,intron_variant
rs909803554	upstream_transcript_variant,genic_upstream_transcript_variant,intron_variant
rs909814578	upstream_transcript_variant,genic_upstream_transcript_variant,intron_variant,2KB_upstream_variant
rs909816686	intron_variant
rs909865764	genic_upstream_transcript_variant,intron_variant
rs909902096	genic_downstream_transcript_variant,intron_variant
rs909916113	genic_upstream_transcript_variant,intron_variant
rs909954312	genic_downstream_transcript_variant,intron_variant
rs909961986	intron_variant
rs909974334	genic_downstream_transcript_variant,intron_variant
rs909985530	genic_downstream_transcript_variant,intron_variant
rs910029798	upstream_transcript_variant,2KB_upstream_variant,genic_upstream_transcript_variant,intron_variant
rs910061089	upstream_transcript_variant,2KB_upstream_variant,genic_upstream_transcript_variant,intron_variant
rs910063617	genic_upstream_transcript_variant,intron_variant
rs910091075	intron_variant
rs910120814	genic_downstream_transcript_variant,intron_variant
rs910141339	intron_variant
rs910147857	intron_variant
rs910150512	genic_downstream_transcript_variant,intron_variant
rs910184196	genic_upstream_transcript_variant,intron_variant
rs910236555	genic_upstream_transcript_variant,intron_variant
rs910238782	intron_variant
rs910251230	genic_upstream_transcript_variant,intron_variant
rs910291546	genic_upstream_transcript_variant,intron_variant
rs910305969	upstream_transcript_variant,genic_upstream_transcript_variant,intron_variant
rs910307059	genic_upstream_transcript_variant,intron_variant
rs910329334	intron_variant
rs910356166	genic_upstream_transcript_variant,intron_variant
rs910361745	intron_variant
rs910402297	genic_upstream_transcript_variant,intron_variant
rs910435047	intron_variant
rs910465948	genic_downstream_transcript_variant,intron_variant
rs910466521	genic_upstream_transcript_variant,intron_variant
rs910472502	genic_upstream_transcript_variant,intron_variant
rs910477933	genic_upstream_transcript_variant,intron_variant
rs910520684	genic_upstream_transcript_variant,intron_variant
rs910534422	genic_downstream_transcript_variant,intron_variant
rs910537104	intron_variant
rs910541035	intron_variant
rs910558384	intron_variant
rs910568990	genic_upstream_transcript_variant,intron_variant
rs910576687	genic_upstream_transcript_variant,intron_variant
rs910598704	genic_downstream_transcript_variant,intron_variant
rs910662313	genic_upstream_transcript_variant,intron_variant
rs910706547	intron_variant
rs910716594	genic_upstream_transcript_variant,intron_variant
rs910720381	genic_upstream_transcript_variant,intron_variant
rs910728761	intron_variant
rs910737487	intron_variant
rs910751480	5_prime_UTR_variant,genic_upstream_transcript_variant,intron_variant,non_coding_transcript_variant
rs910834974	upstream_transcript_variant,genic_upstream_transcript_variant,intron_variant,2KB_upstream_variant
rs910837025	genic_upstream_transcript_variant,intron_variant
rs910922607	genic_upstream_transcript_variant,intron_variant
rs910999643	intron_variant
rs911013717	genic_upstream_transcript_variant,intron_variant
rs911014108	genic_upstream_transcript_variant,intron_variant
rs911016293	genic_upstream_transcript_variant,intron_variant
rs911031777	intron_variant
rs911046325	upstream_transcript_variant,genic_upstream_transcript_variant,intron_variant,2KB_upstream_variant
rs911073360	genic_upstream_transcript_variant,intron_variant
rs911081861	intron_variant
rs911172164	genic_downstream_transcript_variant,intron_variant
rs911176396	intron_variant
rs911180773	intron_variant
rs911208250	genic_downstream_transcript_variant,intron_variant
rs911231477	intron_variant

rs911233995	genic_upstream_transcript_variant,intron_variant
rs911270597	intron_variant
rs911289515	genic_upstream_transcript_variant,intron_variant
rs911290722	genic_downstream_transcript_variant,intron_variant
rs911309410	genic_downstream_transcript_variant,intron_variant
rs911323680	genic_downstream_transcript_variant,intron_variant
rs911330211	intron_variant
rs911366276	genic_upstream_transcript_variant,intron_variant
rs911373024	intron_variant
rs911408438	intron_variant
rs911414222	genic_upstream_transcript_variant,intron_variant
rs911426773	genic_downstream_transcript_variant,intron_variant
rs911459560	genic_upstream_transcript_variant,intron_variant
rs911494024	genic_upstream_transcript_variant,intron_variant
rs911503091	intron_variant
rs911515578	genic_upstream_transcript_variant,intron_variant
rs911540603	intron_variant
rs911557796	intron_variant
rs911571952	genic_upstream_transcript_variant,intron_variant
rs911593151	intron_variant
rs911602682	genic_upstream_transcript_variant,intron_variant
rs911623760	intron_variant
rs911657177	genic_downstream_transcript_variant,intron_variant
rs911722528	genic_upstream_transcript_variant,intron_variant
rs911731413	genic_upstream_transcript_variant,intron_variant
rs911743932	intron_variant
rs911756630	genic_upstream_transcript_variant,intron_variant
rs911801861	3_prime_UTR_variant,non_coding_transcript_variant,genic_downstream_transcript_variant
rs911838696	intron_variant
rs911841351	genic_upstream_transcript_variant,intron_variant
rs911853905	intron_variant
rs911860339	genic_downstream_transcript_variant,intron_variant
rs911968865	genic_downstream_transcript_variant,intron_variant
rs911969874	intron_variant
rs911987691	intron_variant
rs911991641	genic_upstream_transcript_variant,intron_variant
rs912039256	genic_upstream_transcript_variant,intron_variant
rs912040209	genic_upstream_transcript_variant,intron_variant
rs912072085	genic_upstream_transcript_variant,intron_variant
rs912078923	genic_downstream_transcript_variant,intron_variant
rs912087589	genic_upstream_transcript_variant,intron_variant
rs912113125	intron_variant
rs912122552	genic_downstream_transcript_variant,intron_variant
rs912126773	genic_downstream_transcript_variant,intron_variant
rs912146150	intron_variant
rs912150573	genic_upstream_transcript_variant,intron_variant
rs912162795	intron_variant
rs912213855	intron_variant
rs912256083	genic_upstream_transcript_variant,intron_variant
rs912277596	genic_downstream_transcript_variant,intron_variant
rs912307205	genic_upstream_transcript_variant,intron_variant
rs912324497	genic_downstream_transcript_variant,intron_variant
rs912326828	genic_upstream_transcript_variant,intron_variant
rs912334486	intron_variant
rs912371745	genic_upstream_transcript_variant,intron_variant
rs912396495	genic_upstream_transcript_variant,intron_variant
rs912401002	intron_variant
rs912443447	intron_variant
rs912448029	genic_downstream_transcript_variant,intron_variant
rs912470041	genic_upstream_transcript_variant,intron_variant
rs912522776	intron_variant
rs912534040	genic_upstream_transcript_variant,intron_variant
rs912542748	genic_upstream_transcript_variant,intron_variant
rs912587844	genic_upstream_transcript_variant,intron_variant
rs912637691	genic_upstream_transcript_variant,intron_variant
rs912640636	intron_variant
rs912685270	genic_upstream_transcript_variant,intron_variant
rs912689025	intron_variant
rs912698292	genic_upstream_transcript_variant,intron_variant
rs912804802	genic_upstream_transcript_variant,intron_variant
rs912810247	genic_downstream_transcript_variant,intron_variant
rs912810753	genic_upstream_transcript_variant,intron_variant
rs912813090	genic_upstream_transcript_variant,intron_variant
rs912835653	genic_upstream_transcript_variant,intron_variant

rs912854113	intron_variant
rs912864910	genic_downstream_transcript_variant,intron_variant
rs912889714	intron_variant
rs912890076	intron_variant
rs912895149	genic_downstream_transcript_variant,intron_variant
rs912929812	genic_upstream_transcript_variant,intron_variant
rs912941387	genic_downstream_transcript_variant,intron_variant
rs912947008	intron_variant
rs912953490	intron_variant
rs912972448	genic_downstream_transcript_variant,intron_variant
rs912988012	upstream_transcript_variant,genic_upstream_transcript_variant,intron_variant,2KB_upstream_variant
rs913063432	3_prime_UTR_variant,non_coding_transcript_variant,genic_downstream_transcript_variant
rs913075838	intron_variant
rs913090996	upstream_transcript_variant,genic_upstream_transcript_variant,intron_variant,2KB_upstream_variant
rs913128597	genic_upstream_transcript_variant,intron_variant
rs913195866	genic_upstream_transcript_variant,intron_variant
rs913201272	genic_upstream_transcript_variant,intron_variant
rs913214053	genic_upstream_transcript_variant,intron_variant
rs913245228	genic_upstream_transcript_variant,intron_variant
rs913250114	intron_variant
rs913254515	intron_variant
rs913261113	intron_variant
rs913273279	genic_downstream_transcript_variant,intron_variant
rs913282552	intron_variant
rs913303699	genic_upstream_transcript_variant,intron_variant
rs913304911	intron_variant
rs913325524	genic_downstream_transcript_variant,intron_variant
rs913379643	genic_downstream_transcript_variant,intron_variant
rs913383047	intron_variant
rs913391698	genic_downstream_transcript_variant,intron_variant
rs913430621	genic_upstream_transcript_variant,intron_variant
rs913434884	intron_variant
rs913442602	genic_upstream_transcript_variant,intron_variant
rs913503649	genic_upstream_transcript_variant,intron_variant
rs913548092	upstream_transcript_variant,2KB_upstream_variant,genic_upstream_transcript_variant,intron_variant
rs913577559	upstream_transcript_variant,2KB_upstream_variant,genic_upstream_transcript_variant,intron_variant
rs913584058	genic_downstream_transcript_variant,intron_variant
rs913584602	intron_variant
rs913625620	genic_upstream_transcript_variant,intron_variant
rs913640719	intron_variant
rs913660804	genic_downstream_transcript_variant,intron_variant
rs913667986	genic_upstream_transcript_variant,intron_variant
rs913684822	intron_variant
rs913727331	genic_downstream_transcript_variant,intron_variant
rs913770250	intron_variant
rs913777830	genic_upstream_transcript_variant,intron_variant
rs913802173	genic_downstream_transcript_variant,intron_variant
rs913807283	genic_upstream_transcript_variant,intron_variant
rs913878075	genic_upstream_transcript_variant,intron_variant
rs913983341	3_prime_UTR_variant,non_coding_transcript_variant,genic_downstream_transcript_variant
rs913993371	genic_upstream_transcript_variant,intron_variant
rs913994505	3_prime_UTR_variant,non_coding_transcript_variant,genic_downstream_transcript_variant
rs914006228	intron_variant
rs914015459	genic_upstream_transcript_variant,intron_variant
rs914040788	intron_variant
rs914124687	intron_variant
rs914156447	intron_variant
rs914174561	genic_upstream_transcript_variant,intron_variant
rs914198197	intron_variant
rs914216401	upstream_transcript_variant,genic_upstream_transcript_variant,intron_variant,2KB_upstream_variant
rs914227349	intron_variant
rs914250784	genic_upstream_transcript_variant,intron_variant
rs914251471	intron_variant
rs914340384	genic_downstream_transcript_variant,intron_variant
rs914344550	intron_variant
rs914346922	genic_upstream_transcript_variant,intron_variant
rs914414650	genic_upstream_transcript_variant,intron_variant
rs914474458	genic_upstream_transcript_variant,intron_variant
rs914487550	upstream_transcript_variant,genic_upstream_transcript_variant,intron_variant,2KB_upstream_variant
rs914502628	genic_downstream_transcript_variant,intron_variant
rs914523321	genic_downstream_transcript_variant,intron_variant
rs914530730	genic_upstream_transcript_variant,intron_variant
rs914532756	intron_variant
rs914547754	genic_upstream_transcript_variant,intron_variant

rs914583629	intron_variant
rs914614864	genic_downstream_transcript_variant,intron_variant
rs914645757	genic_downstream_transcript_variant,intron_variant
rs914662174	genic_upstream_transcript_variant,intron_variant
rs914668316	intron_variant
rs914671397	genic_upstream_transcript_variant,intron_variant
rs914715101	intron_variant
rs914741312	500B_downstream_variant,downstream_transcript_variant
rs914757416	intron_variant
rs914764641	genic_upstream_transcript_variant,intron_variant
rs914823305	genic_downstream_transcript_variant,intron_variant
rs914831340	intron_variant
rs914832094	genic_downstream_transcript_variant,intron_variant
rs914839688	genic_upstream_transcript_variant,intron_variant
rs914919708	genic_upstream_transcript_variant,intron_variant
rs914957605	intron_variant
rs914995459	genic_upstream_transcript_variant,intron_variant
rs915031123	intron_variant
rs915101691	genic_upstream_transcript_variant,intron_variant
rs915128276	genic_downstream_transcript_variant,intron_variant
rs915146258	genic_downstream_transcript_variant,intron_variant
rs915155828	genic_upstream_transcript_variant,intron_variant
rs915164401	intron_variant
rs915185928	intron_variant
rs915193160	genic_upstream_transcript_variant,intron_variant
rs915238202	genic_downstream_transcript_variant,intron_variant
rs915248019	genic_downstream_transcript_variant,intron_variant
rs915253843	intron_variant
rs915269222	genic_downstream_transcript_variant,intron_variant
rs915280256	intron_variant
rs915280989	genic_upstream_transcript_variant,intron_variant
rs915322174	genic_downstream_transcript_variant,intron_variant
rs915326933	genic_upstream_transcript_variant,intron_variant
rs915342068	genic_upstream_transcript_variant,intron_variant
rs915342452	intron_variant
rs915343529	genic_upstream_transcript_variant,intron_variant
rs915386806	upstream_transcript_variant,2KB_upstream_variant,genic_upstream_transcript_variant,intron_variant
rs915419449	genic_upstream_transcript_variant,intron_variant
rs915421978	genic_upstream_transcript_variant,intron_variant
rs915435216	upstream_transcript_variant,2KB_upstream_variant,genic_upstream_transcript_variant,intron_variant
rs915454137	intron_variant
rs915499788	genic_upstream_transcript_variant,intron_variant
rs915525826	intron_variant
rs915534389	intron_variant
rs915544481	genic_upstream_transcript_variant,intron_variant
rs915614772	intron_variant
rs915692777	intron_variant
rs915694613	genic_upstream_transcript_variant,intron_variant
rs915705016	genic_upstream_transcript_variant,intron_variant
rs915725083	intron_variant
rs915776471	genic_downstream_transcript_variant,intron_variant
rs915870536	genic_upstream_transcript_variant,intron_variant
rs915874823	genic_downstream_transcript_variant,intron_variant
rs915934010	non_coding_transcript_variant,genic_upstream_transcript_variant,intron_variant
rs915951822	intron_variant
rs915963287	intron_variant
rs915978772	coding_sequence_variant,intron_variant,genic_upstream_transcript_variant,synonymous_variant,non_coding_transcript_variant
rs916004644	coding_sequence_variant,intron_variant,5_prime_UTR_variant,genic_upstream_transcript_variant,non_coding_transcript_variant,missense_variant
rs916056075	intron_variant
rs916095164	intron_variant
rs916095395	genic_upstream_transcript_variant,intron_variant
rs916116152	intron_variant
rs916126282	intron_variant
rs916143248	genic_downstream_transcript_variant,intron_variant
rs916177472	genic_downstream_transcript_variant,intron_variant
rs916184599	intron_variant
rs916208536	genic_downstream_transcript_variant,intron_variant
rs916272656	upstream_transcript_variant,2KB_upstream_variant,genic_upstream_transcript_variant,intron_variant
rs916305057	upstream_transcript_variant,2KB_upstream_variant,genic_upstream_transcript_variant,intron_variant
rs916321248	genic_downstream_transcript_variant,intron_variant
rs916325723	genic_upstream_transcript_variant,intron_variant
rs916331630	genic_upstream_transcript_variant,intron_variant
rs916340923	intron_variant
rs916407046	genic_downstream_transcript_variant,intron_variant

rs916414320	genic_upstream_transcript_variant,intron_variant
rs916462837	genic_upstream_transcript_variant,intron_variant
rs916464530	intron_variant
rs916480017	genic_downstream_transcript_variant,intron_variant
rs916497601	genic_downstream_transcript_variant,intron_variant
rs916502190	intron_variant
rs916510960	genic_downstream_transcript_variant,intron_variant
rs916524845	genic_upstream_transcript_variant,intron_variant
rs916551290	intron_variant
rs916698951	genic_upstream_transcript_variant,intron_variant
rs916700097	upstream_transcript_variant,genic_upstream_transcript_variant,intron_variant,2KB_upstream_variant
rs916702214	intron_variant
rs916713398	genic_downstream_transcript_variant,intron_variant
rs916718833	intron_variant
rs916752805	genic_upstream_transcript_variant,intron_variant
rs916778287	intron_variant
rs916799997	genic_upstream_transcript_variant,intron_variant
rs916829339	intron_variant
rs916870888	genic_upstream_transcript_variant,intron_variant
rs916887872	genic_upstream_transcript_variant,intron_variant
rs916927812	upstream_transcript_variant,genic_upstream_transcript_variant,intron_variant
rs916928244	intron_variant
rs916982498	intron_variant
rs916983613	3_prime_UTR_variant,non_coding_transcript_variant,genic_downstream_transcript_variant
rs917003024	genic_upstream_transcript_variant,intron_variant
rs917008172	5_prime_UTR_variant,genic_upstream_transcript_variant,intron_variant,non_coding_transcript_variant
rs917008760	genic_upstream_transcript_variant,intron_variant
rs917056724	genic_downstream_transcript_variant,intron_variant
rs917090670	genic_downstream_transcript_variant,intron_variant
rs917105176	genic_upstream_transcript_variant,intron_variant
rs917109442	intron_variant
rs917145826	genic_downstream_transcript_variant,intron_variant
rs917151876	genic_downstream_transcript_variant,intron_variant
rs917166604	intron_variant
rs917180203	genic_upstream_transcript_variant,intron_variant
rs917184495	genic_downstream_transcript_variant,intron_variant
rs917191940	genic_upstream_transcript_variant,intron_variant
rs917221635	genic_downstream_transcript_variant,intron_variant
rs917224861	genic_upstream_transcript_variant,intron_variant
rs917263857	genic_downstream_transcript_variant,downstream_transcript_variant,intron_variant
rs917266638	intron_variant
rs917282326	genic_upstream_transcript_variant,intron_variant
rs917332351	genic_downstream_transcript_variant,intron_variant
rs917349025	intron_variant
rs917353750	intron_variant
rs917363288	genic_upstream_transcript_variant,intron_variant
rs917476864	genic_upstream_transcript_variant,intron_variant
rs917480721	intron_variant
rs917494772	intron_variant
rs917527270	intron_variant
rs917539056	genic_upstream_transcript_variant,intron_variant
rs917545902	genic_upstream_transcript_variant,intron_variant
rs917571337	intron_variant
rs917667676	genic_downstream_transcript_variant,intron_variant
rs917699456	genic_upstream_transcript_variant,intron_variant
rs917701958	intron_variant
rs917728272	genic_upstream_transcript_variant,intron_variant
rs917774485	genic_upstream_transcript_variant,intron_variant
rs917833316	intron_variant
rs917875206	genic_upstream_transcript_variant,intron_variant
rs917893346	genic_upstream_transcript_variant,intron_variant
rs917922985	genic_upstream_transcript_variant,intron_variant
rs917949465	intron_variant
rs917964618	genic_downstream_transcript_variant,intron_variant
rs917966721	intron_variant
rs917987322	genic_downstream_transcript_variant,intron_variant
rs918004285	genic_downstream_transcript_variant,intron_variant
rs918014783	intron_variant
rs918052587	genic_upstream_transcript_variant,intron_variant
rs918074194	intron_variant
rs918078044	intron_variant
rs918081676	genic_downstream_transcript_variant,intron_variant
rs918086337	genic_upstream_transcript_variant,intron_variant
rs918106759	genic_upstream_transcript_variant,intron_variant

rs918145681	intron_variant
rs918149604	genic_upstream_transcript_variant,intron_variant
rs918234707	genic_upstream_transcript_variant,intron_variant
rs918269867	genic_upstream_transcript_variant,intron_variant
rs918313066	intron_variant
rs918327997	genic_upstream_transcript_variant,intron_variant
rs918337574	intron_variant
rs918434673	intron_variant
rs918435800	genic_upstream_transcript_variant,intron_variant
rs918454726	genic_downstream_transcript_variant,intron_variant
rs918458866	intron_variant
rs918469239	intron_variant
rs918476124	genic_downstream_transcript_variant,intron_variant
rs918488343	genic_upstream_transcript_variant,intron_variant
rs918499092	genic_upstream_transcript_variant,intron_variant
rs918510845	intron_variant
rs918518955	genic_downstream_transcript_variant,intron_variant
rs918541399	intron_variant
rs918554987	intron_variant
rs918574380	intron_variant
rs918582413	genic_upstream_transcript_variant,intron_variant
rs918588728	genic_downstream_transcript_variant,intron_variant
rs918608179	intron_variant
rs918619944	genic_downstream_transcript_variant,intron_variant
rs918643502	genic_upstream_transcript_variant,intron_variant
rs918698222	intron_variant
rs918708751	intron_variant
rs918723979	intron_variant
rs918728162	genic_upstream_transcript_variant,intron_variant
rs918744668	intron_variant
rs918764980	genic_upstream_transcript_variant,intron_variant
rs918866159	genic_upstream_transcript_variant,intron_variant
rs918869085	intron_variant
rs918916843	genic_upstream_transcript_variant,intron_variant
rs918925719	3_prime_UTR_variant,non_coding_transcript_variant,genic_downstream_transcript_variant
rs918928864	intron_variant
rs918956919	3_prime_UTR_variant,non_coding_transcript_variant,genic_downstream_transcript_variant
rs919031461	intron_variant
rs919032220	genic_downstream_transcript_variant,intron_variant
rs919047306	genic_upstream_transcript_variant,intron_variant
rs919050790	intron_variant
rs919061704	500B_downstream_variant,downstream_transcript_variant
rs919080129	intron_variant
rs919112304	genic_upstream_transcript_variant,intron_variant
rs919152271	intron_variant
rs919170347	genic_upstream_transcript_variant,intron_variant
rs919199760	genic_upstream_transcript_variant,intron_variant
rs919208026	intron_variant
rs919208770	500B_downstream_variant,downstream_transcript_variant
rs919222827	genic_upstream_transcript_variant,intron_variant
rs919285204	genic_downstream_transcript_variant,intron_variant
rs919293448	intron_variant
rs919298676	intron_variant
rs919303283	intron_variant
rs919335453	genic_upstream_transcript_variant,intron_variant
rs919415389	2KB_upstream_variant,genic_upstream_transcript_variant,upstream_transcript_variant,intron_variant
rs919420042	genic_upstream_transcript_variant,intron_variant
rs919427044	intron_variant
rs919431318	genic_upstream_transcript_variant,intron_variant
rs919472633	intron_variant
rs919515421	intron_variant,non_coding_transcript_variant,genic_upstream_transcript_variant,5_prime_UTR_variant
rs919542193	intron_variant
rs919556031	genic_downstream_transcript_variant,intron_variant
rs919573498	intron_variant
rs919578761	intron_variant
rs919622387	intron_variant
rs919640764	genic_upstream_transcript_variant,intron_variant
rs919722927	genic_upstream_transcript_variant,intron_variant
rs919729848	genic_upstream_transcript_variant,intron_variant
rs919730536	intron_variant
rs919735808	genic_downstream_transcript_variant,intron_variant
rs919751471	intron_variant
rs919826117	intron_variant
rs919828985	genic_downstream_transcript_variant,intron_variant

rs919881814	intron_variant
rs919896979	genic_upstream_transcript_variant,intron_variant
rs919939712	2KB_upstream_variant,upstream_transcript_variant,genic_upstream_transcript_variant,intron_variant
rs919957711	intron_variant
rs919968749	intron_variant
rs919974203	intron_variant
rs919982616	intron_variant
rs919984435	genic_downstream_transcript_variant,intron_variant
rs919987914	genic_upstream_transcript_variant,intron_variant
rs919994891	genic_upstream_transcript_variant,intron_variant
rs920016453	genic_upstream_transcript_variant,intron_variant
rs920051085	genic_upstream_transcript_variant,intron_variant
rs920057591	genic_upstream_transcript_variant,intron_variant
rs920062693	genic_downstream_transcript_variant,intron_variant
rs920082797	genic_upstream_transcript_variant,intron_variant
rs920113030	intron_variant
rs920139811	intron_variant
rs920198928	genic_upstream_transcript_variant,intron_variant
rs920201575	genic_upstream_transcript_variant,intron_variant
rs920206176	genic_upstream_transcript_variant,intron_variant
rs920231551	genic_upstream_transcript_variant,intron_variant
rs920256194	genic_upstream_transcript_variant,intron_variant
rs920272373	intron_variant
rs920300950	intron_variant
rs920317979	genic_downstream_transcript_variant,intron_variant
rs920326133	genic_upstream_transcript_variant,intron_variant
rs920326927	genic_upstream_transcript_variant,intron_variant
rs920370314	genic_upstream_transcript_variant,intron_variant
rs920415889	genic_downstream_transcript_variant,intron_variant
rs920432619	intron_variant
rs920447018	intron_variant
rs920495205	genic_upstream_transcript_variant,intron_variant
rs920510243	genic_downstream_transcript_variant,intron_variant
rs920518217	genic_downstream_transcript_variant,intron_variant
rs920524879	genic_upstream_transcript_variant,intron_variant
rs920556863	genic_downstream_transcript_variant,intron_variant
rs920569453	intron_variant
rs920585471	genic_upstream_transcript_variant,intron_variant
rs920624124	3_prime_UTR_variant,non_coding_transcript_variant,genic_downstream_transcript_variant
rs920681675	intron_variant
rs920694371	genic_downstream_transcript_variant,intron_variant
rs920701199	intron_variant
rs920771440	genic_upstream_transcript_variant,intron_variant
rs920796955	intron_variant
rs920800479	genic_downstream_transcript_variant,intron_variant
rs920813492	intron_variant
rs920820231	intron_variant
rs920879647	intron_variant
rs920936580	intron_variant
rs920972078	genic_upstream_transcript_variant,intron_variant
rs921005909	intron_variant
rs921016438	intron_variant
rs921022512	genic_downstream_transcript_variant,intron_variant
rs921047335	intron_variant
rs921066767	genic_downstream_transcript_variant,intron_variant
rs921069794	genic_downstream_transcript_variant,intron_variant
rs921087397	intron_variant
rs921156067	genic_upstream_transcript_variant,intron_variant
rs921187083	genic_upstream_transcript_variant,intron_variant
rs921189227	coding_sequence_variant,non_coding_transcript_variant,genic_upstream_transcript_variant,intron_variant,synonymous_variant
rs921212455	genic_downstream_transcript_variant,intron_variant
rs921226256	genic_upstream_transcript_variant,intron_variant
rs921259951	intron_variant
rs921278710	genic_upstream_transcript_variant,intron_variant
rs921286355	genic_downstream_transcript_variant,intron_variant
rs921304940	genic_upstream_transcript_variant,intron_variant
rs921308683	synonymous_variant,coding_sequence_variant,non_coding_transcript_variant
rs921310635	intron_variant
rs921322399	5_prime_UTR_variant,intron_variant
rs921454521	downstream_transcript_variant,genic_downstream_transcript_variant,intron_variant
rs921460883	genic_upstream_transcript_variant,intron_variant
rs921554392	intron_variant
rs921579402	intron_variant
rs921601805	intron_variant

rs921667765	genic_upstream_transcript_variant,intron_variant
rs921669580	intron_variant
rs921709092	genic_upstream_transcript_variant,intron_variant
rs921738226	genic_upstream_transcript_variant,intron_variant
rs921788374	genic_upstream_transcript_variant,intron_variant
rs921807110	intron_variant
rs921809935	genic_upstream_transcript_variant,intron_variant
rs921879528	genic_upstream_transcript_variant,intron_variant
rs921880275	intron_variant
rs921891195	genic_downstream_transcript_variant,intron_variant
rs921949099	genic_upstream_transcript_variant,intron_variant
rs921951210	intron_variant
rs921953439	genic_downstream_transcript_variant,intron_variant
rs921982287	intron_variant
rs921990959	intron_variant
rs922022359	genic_downstream_transcript_variant,intron_variant
rs922031581	intron_variant
rs922044024	genic_upstream_transcript_variant,intron_variant
rs922068583	intron_variant
rs922074233	3_prime_UTR_variant,non_coding_transcript_variant,genic_downstream_transcript_variant
rs922090178	genic_upstream_transcript_variant,intron_variant
rs922132444	intron_variant
rs922165292	genic_downstream_transcript_variant,intron_variant
rs922177645	500B_downstream_variant,downstream_transcript_variant
rs922179075	genic_downstream_transcript_variant,intron_variant
rs922260812	intron_variant
rs922260960	genic_upstream_transcript_variant,intron_variant
rs922268420	genic_upstream_transcript_variant,intron_variant
rs922300636	genic_upstream_transcript_variant,intron_variant
rs922306353	genic_upstream_transcript_variant,intron_variant
rs922334820	genic_downstream_transcript_variant,intron_variant
rs922393106	genic_upstream_transcript_variant,intron_variant
rs922406011	intron_variant
rs922457335	genic_upstream_transcript_variant,intron_variant
rs922476976	genic_upstream_transcript_variant,intron_variant
rs922540644	genic_upstream_transcript_variant,intron_variant
rs922551308	genic_upstream_transcript_variant,intron_variant
rs922563967	genic_downstream_transcript_variant,intron_variant
rs922583789	genic_downstream_transcript_variant,intron_variant
rs922626528	genic_upstream_transcript_variant,intron_variant
rs922630107	genic_upstream_transcript_variant,intron_variant
rs922675980	genic_downstream_transcript_variant,intron_variant
rs922705007	intron_variant
rs922705451	2KB_upstream_variant,upstream_transcript_variant,genic_upstream_transcript_variant,intron_variant
rs922720391	genic_downstream_transcript_variant,intron_variant
rs922751954	intron_variant
rs922769384	genic_upstream_transcript_variant,intron_variant
rs922788826	genic_upstream_transcript_variant,intron_variant
rs922827735	genic_downstream_transcript_variant,intron_variant
rs922827882	genic_downstream_transcript_variant,intron_variant
rs922834704	genic_downstream_transcript_variant,intron_variant
rs922839547	intron_variant
rs922853141	2KB_upstream_variant,upstream_transcript_variant,genic_upstream_transcript_variant,intron_variant
rs922910720	genic_downstream_transcript_variant,intron_variant
rs922954878	genic_upstream_transcript_variant,intron_variant
rs922958823	intron_variant
rs922985827	intron_variant
rs923006469	2KB_upstream_variant,upstream_transcript_variant,genic_upstream_transcript_variant,intron_variant
rs923027877	genic_downstream_transcript_variant,intron_variant
rs923035312	genic_downstream_transcript_variant,intron_variant
rs923038187	intron_variant
rs923064685	intron_variant
rs923090337	intron_variant
rs923097564	genic_downstream_transcript_variant,intron_variant
rs923129190	genic_downstream_transcript_variant,intron_variant
rs923139107	intron_variant
rs923169099	genic_downstream_transcript_variant,intron_variant
rs923184253	intron_variant
rs923223329	genic_downstream_transcript_variant,intron_variant
rs923242112	genic_upstream_transcript_variant,intron_variant
rs923267515	intron_variant
rs923268465	intron_variant
rs923279269	intron_variant
rs923320510	intron_variant

rs923387824	genic_upstream_transcript_variant,intron_variant
rs923409372	genic_upstream_transcript_variant,intron_variant
rs923436472	3_prime_UTR_variant,non_coding_transcript_variant,genic_downstream_transcript_variant
rs923480989	intron_variant
rs923486647	genic_upstream_transcript_variant,intron_variant
rs923492230	genic_upstream_transcript_variant,intron_variant
rs923514626	genic_upstream_transcript_variant,upstream_transcript_variant,intron_variant
rs923523897	genic_upstream_transcript_variant,intron_variant
rs923549688	intron_variant
rs923552713	genic_upstream_transcript_variant,intron_variant
rs923564494	genic_upstream_transcript_variant,intron_variant
rs923593178	genic_upstream_transcript_variant,intron_variant
rs923603015	intron_variant
rs923606360	intron_variant
rs923658536	intron_variant
rs923668358	2KB_upstream_variant,upstream_transcript_variant,genic_upstream_transcript_variant,intron_variant
rs923699353	2KB_upstream_variant,upstream_transcript_variant,genic_upstream_transcript_variant,intron_variant
rs923730264	intron_variant
rs923736003	genic_upstream_transcript_variant,intron_variant
rs923756566	intron_variant
rs923774410	genic_downstream_transcript_variant,intron_variant
rs923808076	intron_variant
rs923920109	intron_variant
rs923920860	genic_upstream_transcript_variant,intron_variant
rs923953189	genic_downstream_transcript_variant,intron_variant
rs923968295	intron_variant
rs923982426	intron_variant
rs923996498	genic_downstream_transcript_variant,intron_variant
rs924015604	genic_upstream_transcript_variant,intron_variant
rs924019460	genic_upstream_transcript_variant,intron_variant
rs924029879	genic_upstream_transcript_variant,intron_variant
rs924047709	genic_upstream_transcript_variant,intron_variant
rs924050385	genic_upstream_transcript_variant,intron_variant
rs924059939	intron_variant
rs924062304	intron_variant
rs924084968	intron_variant
rs924116195	intron_variant
rs924136556	intron_variant
rs924150891	genic_downstream_transcript_variant,intron_variant
rs924155302	intron_variant
rs924158629	intron_variant
rs924202583	intron_variant
rs924206123	genic_upstream_transcript_variant,intron_variant
rs924254686	genic_upstream_transcript_variant,intron_variant
rs924328629	genic_downstream_transcript_variant,intron_variant
rs924338967	genic_downstream_transcript_variant,intron_variant
rs924343847	intron_variant
rs924352886	genic_upstream_transcript_variant,intron_variant
rs924354091	genic_upstream_transcript_variant,upstream_transcript_variant,intron_variant
rs924371282	genic_upstream_transcript_variant,intron_variant
rs924393170	intron_variant
rs924421404	intron_variant
rs924432259	genic_upstream_transcript_variant,intron_variant
rs924463940	genic_downstream_transcript_variant,intron_variant
rs924493368	intron_variant
rs924502271	intron_variant
rs924540146	genic_upstream_transcript_variant,intron_variant
rs924544589	genic_downstream_transcript_variant,intron_variant
rs924548134	genic_downstream_transcript_variant,intron_variant
rs924568051	genic_upstream_transcript_variant,intron_variant
rs924584903	intron_variant
rs924608376	genic_upstream_transcript_variant,intron_variant
rs924616165	intron_variant
rs924649132	genic_upstream_transcript_variant,intron_variant
rs924672361	intron_variant
rs924682814	genic_upstream_transcript_variant,intron_variant
rs924750366	genic_downstream_transcript_variant,intron_variant
rs924756623	2KB_upstream_variant,genic_upstream_transcript_variant,upstream_transcript_variant,intron_variant
rs924758599	genic_upstream_transcript_variant,intron_variant
rs924856510	intron_variant
rs924885354	genic_upstream_transcript_variant,intron_variant
rs924903596	genic_upstream_transcript_variant,intron_variant
rs924927810	synonymous_variant,coding_sequence_variant,non_coding_transcript_variant
rs924930566	intron_variant

rs924933913	genic_downstream_transcript_variant,intron_variant
rs924952572	genic_upstream_transcript_variant,intron_variant
rs924977777	intron_variant
rs925000327	intron_variant
rs925008196	genic_downstream_transcript_variant,intron_variant
rs925023828	intron_variant
rs925056402	2KB_upstream_variant,upstream_transcript_variant,genic_upstream_transcript_variant,intron_variant
rs925142579	genic_downstream_transcript_variant,intron_variant
rs925167176	intron_variant
rs925198992	genic_downstream_transcript_variant,intron_variant
rs925273640	genic_downstream_transcript_variant,intron_variant
rs925278724	genic_upstream_transcript_variant,intron_variant
rs925281081	intron_variant
rs925285894	genic_upstream_transcript_variant,intron_variant
rs925290877	intron_variant
rs925313384	genic_downstream_transcript_variant,intron_variant
rs925327245	intron_variant
rs925342998	intron_variant
rs925369946	genic_upstream_transcript_variant,intron_variant
rs925444992	genic_upstream_transcript_variant,intron_variant
rs925448141	intron_variant
rs925450383	genic_downstream_transcript_variant,intron_variant
rs925457252	intron_variant
rs925465969	genic_upstream_transcript_variant,intron_variant
rs925481557	genic_downstream_transcript_variant,intron_variant
rs925516841	genic_upstream_transcript_variant,intron_variant
rs925519668	genic_downstream_transcript_variant,intron_variant
rs925535398	intron_variant
rs925569878	genic_upstream_transcript_variant,intron_variant
rs925600985	coding_sequence_variant,non_coding_transcript_variant,genic_upstream_transcript_variant,intron_variant,synonymous_variant
rs925603654	genic_downstream_transcript_variant,intron_variant
rs925606318	intron_variant
rs925616952	intron_variant
rs925633329	genic_downstream_transcript_variant,intron_variant
rs925669416	intron_variant
rs925752133	intron_variant
rs925800078	genic_upstream_transcript_variant,intron_variant
rs925805779	intron_variant
rs925809690	genic_upstream_transcript_variant,intron_variant
rs925879253	genic_downstream_transcript_variant,intron_variant
rs925906618	genic_upstream_transcript_variant,intron_variant
rs925923114	genic_upstream_transcript_variant,intron_variant
rs925951580	downstream_transcript_variant,genic_downstream_transcript_variant,intron_variant
rs925976994	genic_upstream_transcript_variant,intron_variant
rs926003759	downstream_transcript_variant,genic_downstream_transcript_variant,intron_variant
rs926053303	intron_variant
rs926065995	intron_variant
rs926131179	intron_variant
rs926134896	genic_upstream_transcript_variant,intron_variant
rs926144902	genic_downstream_transcript_variant,intron_variant
rs926166084	intron_variant
rs926204812	genic_upstream_transcript_variant,intron_variant
rs926236468	intron_variant
rs926245421	intron_variant
rs926293635	intron_variant
rs926303119	genic_upstream_transcript_variant,intron_variant
rs926330054	intron_variant
rs926341970	genic_downstream_transcript_variant,intron_variant
rs926368109	intron_variant
rs926437293	intron_variant
rs926438171	genic_upstream_transcript_variant,intron_variant
rs926454984	intron_variant
rs926473109	genic_downstream_transcript_variant,intron_variant
rs926534593	intron_variant
rs926537388	intron_variant
rs926545347	genic_upstream_transcript_variant,intron_variant
rs926553157	intron_variant
rs926581715	genic_upstream_transcript_variant,intron_variant
rs926587655	intron_variant
rs926610325	genic_downstream_transcript_variant,intron_variant
rs926620065	genic_upstream_transcript_variant,intron_variant
rs926666909	intron_variant
rs926683171	genic_upstream_transcript_variant,intron_variant
rs926699162	intron_variant

rs926701098	genic_downstream_transcript_variant,intron_variant
rs926714469	genic_upstream_transcript_variant,intron_variant
rs926758874	genic_upstream_transcript_variant,intron_variant
rs926789863	genic_upstream_transcript_variant,intron_variant
rs926790715	intron_variant
rs926808396	intron_variant
rs926817290	genic_downstream_transcript_variant,intron_variant
rs926841782	intron_variant
rs926842757	intron_variant
rs926891078	genic_downstream_transcript_variant,intron_variant
rs926911853	genic_downstream_transcript_variant,intron_variant
rs926920720	intron_variant
rs926931415	genic_upstream_transcript_variant,intron_variant
rs926949147	3_prime_UTR_variant,non_coding_transcript_variant,genic_downstream_transcript_variant
rs927012268	genic_downstream_transcript_variant,intron_variant
rs927015969	genic_upstream_transcript_variant,intron_variant
rs927036342	intron_variant
rs927037596	genic_downstream_transcript_variant,intron_variant
rs927046782	genic_upstream_transcript_variant,intron_variant
rs927060608	intron_variant
rs927118475	intron_variant
rs927150828	genic_downstream_transcript_variant,intron_variant
rs927158129	intron_variant
rs927192113	genic_upstream_transcript_variant,intron_variant
rs927215348	genic_downstream_transcript_variant,intron_variant
rs927283322	intron_variant
rs927304946	downstream_transcript_variant,genic_downstream_transcript_variant,intron_variant
rs927345571	intron_variant
rs927372677	2KB_upstream_variant,genic_upstream_transcript_variant,upstream_transcript_variant,intron_variant
rs927383889	genic_upstream_transcript_variant,intron_variant
rs927392520	genic_upstream_transcript_variant,intron_variant
rs927397966	genic_upstream_transcript_variant,intron_variant
rs927404705	genic_upstream_transcript_variant,intron_variant
rs927417354	intron_variant
rs927420616	intron_variant
rs927432176	genic_upstream_transcript_variant,intron_variant
rs927457584	intron_variant
rs927479921	intron_variant
rs927534086	intron_variant
rs927546781	genic_upstream_transcript_variant,intron_variant
rs927547431	genic_upstream_transcript_variant,intron_variant
rs927557154	genic_upstream_transcript_variant,intron_variant
rs927590177	genic_upstream_transcript_variant,intron_variant
rs927663542	genic_downstream_transcript_variant,intron_variant
rs927692772	genic_upstream_transcript_variant,intron_variant
rs927700399	genic_upstream_transcript_variant,intron_variant
rs927709732	genic_downstream_transcript_variant,intron_variant
rs927733936	intron_variant
rs927741929	genic_upstream_transcript_variant,intron_variant
rs927757514	intron_variant
rs927830043	intron_variant
rs927858957	genic_upstream_transcript_variant,intron_variant
rs927893511	genic_downstream_transcript_variant,intron_variant
rs927928194	genic_upstream_transcript_variant,intron_variant
rs927948152	genic_upstream_transcript_variant,intron_variant
rs927948686	intron_variant
rs927985338	genic_upstream_transcript_variant,intron_variant
rs928006673	genic_upstream_transcript_variant,intron_variant
rs928007892	3_prime_UTR_variant,non_coding_transcript_variant,genic_downstream_transcript_variant
rs928062812	intron_variant
rs928075396	3_prime_UTR_variant,non_coding_transcript_variant,genic_downstream_transcript_variant
rs928085918	intron_variant
rs928102594	genic_upstream_transcript_variant,intron_variant
rs928156361	genic_upstream_transcript_variant,intron_variant
rs928178624	intron_variant
rs928190382	intron_variant
rs928208607	genic_upstream_transcript_variant,intron_variant
rs928227616	intron_variant
rs928241775	intron_variant
rs928261285	genic_upstream_transcript_variant,intron_variant
rs928283513	2KB_upstream_variant,genic_upstream_transcript_variant,upstream_transcript_variant,intron_variant
rs928284514	genic_upstream_transcript_variant,intron_variant
rs928287177	intron_variant
rs928304839	intron_variant

rs928307784	genic_upstream_transcript_variant,intron_variant
rs928316199	2KB_upstream_variant,genic_upstream_transcript_variant,upstream_transcript_variant,intron_variant
rs928423686	intron_variant
rs928460602	2KB_upstream_variant,genic_upstream_transcript_variant,upstream_transcript_variant,intron_variant
rs928490758	genic_upstream_transcript_variant,intron_variant
rs928522259	intron_variant
rs928541105	intron_variant
rs928542592	intron_variant
rs928597761	genic_upstream_transcript_variant,intron_variant
rs928611804	genic_downstream_transcript_variant,intron_variant
rs928620871	genic_upstream_transcript_variant,intron_variant
rs928625687	intron_variant
rs928663332	intron_variant
rs928673569	intron_variant
rs928674072	genic_downstream_transcript_variant,intron_variant
rs928694319	genic_upstream_transcript_variant,intron_variant
rs928708977	genic_upstream_transcript_variant,intron_variant
rs928750441	intron_variant
rs928773864	intron_variant
rs928783802	genic_upstream_transcript_variant,intron_variant
rs928793603	genic_upstream_transcript_variant,intron_variant
rs928804674	intron_variant
rs928810149	genic_downstream_transcript_variant,intron_variant
rs928810617	genic_downstream_transcript_variant,intron_variant
rs928830398	genic_downstream_transcript_variant,intron_variant
rs928839079	genic_downstream_transcript_variant,intron_variant
rs928910154	genic_downstream_transcript_variant,intron_variant
rs928925693	genic_upstream_transcript_variant,intron_variant
rs928946706	genic_downstream_transcript_variant,intron_variant
rs928947941	intron_variant
rs929007225	genic_downstream_transcript_variant,intron_variant
rs929061044	genic_downstream_transcript_variant,intron_variant
rs929062349	intron_variant
rs929104113	genic_downstream_transcript_variant,intron_variant
rs929158643	intron_variant
rs929212431	intron_variant
rs929223281	genic_upstream_transcript_variant,intron_variant
rs929233874	3_prime_UTR_variant,non_coding_transcript_variant,genic_downstream_transcript_variant
rs929297936	genic_downstream_transcript_variant,intron_variant
rs929303688	genic_upstream_transcript_variant,intron_variant
rs929304210	genic_downstream_transcript_variant,intron_variant
rs929304849	intron_variant
rs929314743	intron_variant
rs929353907	genic_upstream_transcript_variant,intron_variant
rs929386021	genic_upstream_transcript_variant,intron_variant
rs929428563	intron_variant
rs929433558	genic_upstream_transcript_variant,intron_variant
rs929439525	genic_downstream_transcript_variant,intron_variant
rs929550389	genic_downstream_transcript_variant,intron_variant
rs929555798	intron_variant
rs929582729	genic_upstream_transcript_variant,intron_variant
rs929583634	intron_variant
rs929607430	intron_variant
rs929608828	genic_downstream_transcript_variant,intron_variant
rs929638343	genic_downstream_transcript_variant,intron_variant
rs929658424	intron_variant
rs929663031	genic_upstream_transcript_variant,intron_variant
rs929676740	genic_upstream_transcript_variant,intron_variant
rs929676819	intron_variant
rs929686930	genic_upstream_transcript_variant,intron_variant
rs929700340	intron_variant
rs929745507	genic_upstream_transcript_variant,intron_variant
rs929748934	genic_downstream_transcript_variant,intron_variant
rs929779047	genic_upstream_transcript_variant,intron_variant
rs929829081	genic_downstream_transcript_variant,intron_variant
rs929864380	genic_downstream_transcript_variant,intron_variant
rs929879598	intron_variant
rs929905398	genic_downstream_transcript_variant,intron_variant
rs929913263	genic_upstream_transcript_variant,intron_variant
rs929924906	genic_downstream_transcript_variant,intron_variant
rs929940189	intron_variant
rs929945796	genic_upstream_transcript_variant,intron_variant
rs929986481	intron_variant
rs929990973	intron_variant

rs929999641	genic_upstream_transcript_variant,intron_variant
rs930055699	intron_variant
rs930095992	genic_upstream_transcript_variant,intron_variant
rs930126270	intron_variant
rs930144218	genic_upstream_transcript_variant,intron_variant
rs930152434	intron_variant
rs930161646	downstream_transcript_variant,genic_downstream_transcript_variant,intron_variant
rs930166195	intron_variant
rs930169564	genic_upstream_transcript_variant,intron_variant
rs930188016	genic_upstream_transcript_variant,intron_variant
rs930188876	genic_upstream_transcript_variant,intron_variant
rs930244833	genic_upstream_transcript_variant,intron_variant
rs930247826	intron_variant
rs930284423	genic_upstream_transcript_variant,intron_variant
rs930297676	intron_variant
rs930339633	genic_upstream_transcript_variant,intron_variant
rs930356306	genic_upstream_transcript_variant,intron_variant
rs930403954	genic_downstream_transcript_variant,intron_variant
rs930411127	intron_variant
rs930436947	genic_downstream_transcript_variant,intron_variant
rs930441899	intron_variant
rs930474300	intron_variant
rs930521755	genic_upstream_transcript_variant,intron_variant
rs930559570	genic_downstream_transcript_variant,intron_variant
rs930597378	intron_variant
rs930614450	genic_upstream_transcript_variant,intron_variant
rs930636193	genic_upstream_transcript_variant,2KB_upstream_variant,upstream_transcript_variant,intron_variant
rs930685638	intron_variant
rs930701717	intron_variant
rs930705957	genic_downstream_transcript_variant,intron_variant
rs930724085	intron_variant
rs930736533	intron_variant
rs930755581	intron_variant
rs930850673	intron_variant
rs930874808	3_prime_UTR_variant,genic_downstream_transcript_variant,non_coding_transcript_variant
rs930902737	genic_upstream_transcript_variant,intron_variant
rs930916473	genic_upstream_transcript_variant,intron_variant
rs931008116	genic_upstream_transcript_variant,intron_variant
rs931015077	intron_variant
rs931039749	genic_upstream_transcript_variant,intron_variant
rs931042367	intron_variant
rs931050535	intron_variant
rs931060865	genic_upstream_transcript_variant,intron_variant
rs931146625	genic_upstream_transcript_variant,intron_variant
rs931177843	genic_downstream_transcript_variant,intron_variant
rs931180758	intron_variant
rs931224167	genic_upstream_transcript_variant,intron_variant
rs931233987	genic_upstream_transcript_variant,intron_variant
rs931261306	genic_downstream_transcript_variant,intron_variant
rs931275405	intron_variant
rs931276300	intron_variant
rs931315477	non_coding_transcript_variant,genic_upstream_transcript_variant,intron_variant
rs931334300	genic_upstream_transcript_variant,intron_variant
rs931399662	intron_variant
rs931424228	genic_upstream_transcript_variant,intron_variant
rs931442823	genic_upstream_transcript_variant,intron_variant
rs931445238	intron_variant
rs931505079	intron_variant
rs931525393	genic_upstream_transcript_variant,intron_variant
rs931542321	genic_downstream_transcript_variant,intron_variant
rs931557788	intron_variant
rs931565339	intron_variant
rs931573351	genic_downstream_transcript_variant,intron_variant
rs931612764	genic_downstream_transcript_variant,intron_variant
rs931649534	intron_variant
rs931681802	intron_variant
rs931751070	genic_downstream_transcript_variant,intron_variant
rs931753609	intron_variant
rs931814632	genic_upstream_transcript_variant,intron_variant
rs931820727	genic_upstream_transcript_variant,intron_variant
rs931839052	genic_downstream_transcript_variant,intron_variant
rs931841283	genic_upstream_transcript_variant,intron_variant
rs931841521	genic_upstream_transcript_variant,intron_variant
rs931843458	intron_variant

rs931893021	genic_upstream_transcript_variant,2KB_upstream_variant,upstream_transcript_variant,intron_variant
rs931895559	genic_downstream_transcript_variant,intron_variant
rs931898196	genic_upstream_transcript_variant,2KB_upstream_variant,upstream_transcript_variant,intron_variant
rs931961210	genic_upstream_transcript_variant,intron_variant
rs931971062	genic_upstream_transcript_variant,intron_variant
rs931981882	genic_upstream_transcript_variant,intron_variant
rs932004630	intron_variant
rs932081782	genic_upstream_transcript_variant,intron_variant
rs932126167	genic_upstream_transcript_variant,intron_variant
rs932145410	intron_variant
rs932168124	500B_downstream_variant,downstream_transcript_variant
rs932192796	intron_variant
rs932214516	genic_upstream_transcript_variant,intron_variant
rs932226792	intron_variant
rs932232554	genic_upstream_transcript_variant,intron_variant
rs932241914	intron_variant
rs932249785	intron_variant
rs932266259	genic_downstream_transcript_variant,intron_variant
rs932291966	genic_downstream_transcript_variant,intron_variant
rs932300035	genic_upstream_transcript_variant,intron_variant
rs932309917	genic_upstream_transcript_variant,intron_variant
rs932425120	genic_downstream_transcript_variant,intron_variant
rs932434369	genic_upstream_transcript_variant,intron_variant
rs932467081	genic_downstream_transcript_variant,intron_variant
rs932492107	non_coding_transcript_variant,intron_variant,coding_sequence_variant,synonymous_variant,genic_upstream_transcript_variant
rs932520666	genic_downstream_transcript_variant,intron_variant
rs932521391	genic_downstream_transcript_variant,intron_variant
rs932546762	genic_upstream_transcript_variant,intron_variant
rs932577827	genic_upstream_transcript_variant,intron_variant
rs932602774	genic_upstream_transcript_variant,intron_variant
rs932676491	genic_upstream_transcript_variant,intron_variant
rs932714970	genic_downstream_transcript_variant,downstream_transcript_variant,intron_variant
rs932746300	genic_downstream_transcript_variant,intron_variant
rs932792031	genic_upstream_transcript_variant,intron_variant
rs932846927	genic_downstream_transcript_variant,intron_variant
rs932847552	intron_variant
rs932898933	intron_variant
rs932959601	genic_upstream_transcript_variant,intron_variant
rs932996544	genic_downstream_transcript_variant,intron_variant
rs933007749	genic_downstream_transcript_variant,intron_variant
rs933016142	genic_upstream_transcript_variant,intron_variant
rs933027761	intron_variant
rs933062096	genic_upstream_transcript_variant,intron_variant
rs933141285	genic_upstream_transcript_variant,intron_variant
rs933160531	intron_variant
rs933191347	500B_downstream_variant,downstream_transcript_variant
rs933247145	genic_downstream_transcript_variant,intron_variant
rs933270183	genic_upstream_transcript_variant,intron_variant
rs933292372	intron_variant
rs933332894	genic_upstream_transcript_variant,intron_variant
rs933343464	intron_variant
rs933368358	intron_variant
rs933388905	genic_downstream_transcript_variant,intron_variant
rs933421721	genic_upstream_transcript_variant,intron_variant
rs933423905	intron_variant
rs933438402	genic_downstream_transcript_variant,intron_variant
rs933443843	intron_variant
rs933465618	genic_upstream_transcript_variant,intron_variant
rs933467447	genic_upstream_transcript_variant,intron_variant
rs933492530	genic_upstream_transcript_variant,2KB_upstream_variant,upstream_transcript_variant,intron_variant
rs933513895	genic_upstream_transcript_variant,intron_variant
rs933514257	intron_variant
rs933517686	genic_upstream_transcript_variant,intron_variant
rs933539491	genic_upstream_transcript_variant,upstream_transcript_variant,intron_variant
rs933539789	genic_upstream_transcript_variant,intron_variant
rs933568182	intron_variant
rs933576324	genic_upstream_transcript_variant,intron_variant
rs933745986	genic_upstream_transcript_variant,intron_variant
rs933751514	genic_downstream_transcript_variant,intron_variant
rs933755340	intron_variant
rs933777285	intron_variant
rs933781617	genic_upstream_transcript_variant,intron_variant
rs933797198	genic_upstream_transcript_variant,2KB_upstream_variant,upstream_transcript_variant,intron_variant
rs933816809	genic_upstream_transcript_variant,intron_variant

rs933822890	genic_upstream_transcript_variant,intron_variant
rs933824215	genic_upstream_transcript_variant,upstream_transcript_variant,intron_variant
rs933828539	genic_upstream_transcript_variant,2KB_upstream_variant,upstream_transcript_variant,intron_variant
rs933852473	intron_variant
rs933854651	intron_variant
rs933909284	genic_upstream_transcript_variant,intron_variant
rs933965608	genic_upstream_transcript_variant,intron_variant
rs933993017	genic_downstream_transcript_variant,intron_variant
rs934003531	intron_variant
rs934051850	intron_variant
rs934059211	genic_upstream_transcript_variant,intron_variant
rs934061888	genic_upstream_transcript_variant,intron_variant
rs934069079	upstream_transcript_variant,2KB_upstream_variant,genic_upstream_transcript_variant,intron_variant
rs934103428	genic_downstream_transcript_variant,intron_variant
rs934104516	intron_variant
rs934113913	genic_upstream_transcript_variant,intron_variant
rs934125452	genic_downstream_transcript_variant,intron_variant
rs934223870	genic_downstream_transcript_variant,intron_variant
rs934243821	intron_variant
rs934244345	genic_upstream_transcript_variant,intron_variant
rs934254138	genic_upstream_transcript_variant,intron_variant
rs934255184	genic_downstream_transcript_variant,intron_variant
rs934263232	genic_upstream_transcript_variant,intron_variant
rs934272226	genic_downstream_transcript_variant,intron_variant
rs934304491	genic_upstream_transcript_variant,upstream_transcript_variant,intron_variant
rs934337156	genic_upstream_transcript_variant,intron_variant
rs934350535	genic_upstream_transcript_variant,upstream_transcript_variant,intron_variant
rs934361821	intron_variant
rs934373350	genic_downstream_transcript_variant,intron_variant
rs934393044	intron_variant
rs934461758	intron_variant
rs934469506	genic_upstream_transcript_variant,intron_variant
rs934486624	intron_variant
rs934490293	genic_downstream_transcript_variant,intron_variant
rs934518588	genic_downstream_transcript_variant,intron_variant
rs934538816	intron_variant
rs934599734	genic_upstream_transcript_variant,intron_variant
rs934657966	genic_upstream_transcript_variant,intron_variant
rs934693162	genic_downstream_transcript_variant,downstream_transcript_variant,intron_variant
rs934731633	genic_upstream_transcript_variant,intron_variant
rs934735885	intron_variant
rs934750443	genic_downstream_transcript_variant,downstream_transcript_variant,intron_variant
rs934767210	intron_variant
rs934771568	genic_downstream_transcript_variant,intron_variant
rs934846443	intron_variant
rs934850476	intron_variant
rs934856191	5_prime_UTR_variant,upstream_transcript_variant,genic_upstream_transcript_variant,intron_variant
rs934871407	genic_upstream_transcript_variant,intron_variant
rs934993837	genic_upstream_transcript_variant,intron_variant
rs935031783	intron_variant
rs935038749	genic_upstream_transcript_variant,intron_variant
rs935047265	intron_variant
rs935048361	genic_upstream_transcript_variant,intron_variant
rs935065561	genic_upstream_transcript_variant,intron_variant
rs935075329	genic_upstream_transcript_variant,intron_variant
rs935084359	intron_variant
rs935152176	intron_variant
rs935197511	intron_variant
rs935202940	intron_variant
rs935203345	genic_upstream_transcript_variant,intron_variant
rs935240523	genic_upstream_transcript_variant,intron_variant
rs935264626	genic_upstream_transcript_variant,intron_variant
rs935288630	genic_downstream_transcript_variant,intron_variant
rs935305319	intron_variant
rs935315713	genic_upstream_transcript_variant,intron_variant
rs935347885	intron_variant
rs935373937	genic_upstream_transcript_variant,intron_variant
rs935381717	genic_downstream_transcript_variant,intron_variant
rs935424233	genic_downstream_transcript_variant,intron_variant
rs935436857	intron_variant
rs935451509	genic_upstream_transcript_variant,intron_variant
rs935452895	genic_upstream_transcript_variant,intron_variant
rs935458171	intron_variant
rs935526911	genic_upstream_transcript_variant,intron_variant

rs935560154	genic_upstream_transcript_variant,intron_variant
rs935573796	genic_upstream_transcript_variant,intron_variant
rs935574055	intron_variant
rs935574732	intron_variant
rs935608683	genic_downstream_transcript_variant,intron_variant
rs935626351	intron_variant
rs935640771	genic_upstream_transcript_variant,2KB_upstream_variant,upstream_transcript_variant,intron_variant
rs935660112	genic_upstream_transcript_variant,intron_variant
rs935715389	genic_downstream_transcript_variant,intron_variant
rs935745156	genic_downstream_transcript_variant,intron_variant
rs935749669	genic_upstream_transcript_variant,2KB_upstream_variant,upstream_transcript_variant,intron_variant
rs935784541	intron_variant
rs935818777	genic_upstream_transcript_variant,intron_variant
rs935854719	intron_variant
rs935862137	intron_variant
rs935869523	genic_upstream_transcript_variant,2KB_upstream_variant,upstream_transcript_variant,intron_variant
rs935873033	intron_variant
rs935922573	intron_variant
rs935958775	intron_variant
rs935964036	genic_downstream_transcript_variant,intron_variant
rs935985332	genic_upstream_transcript_variant,intron_variant
rs935991339	genic_upstream_transcript_variant,intron_variant
rs936007944	intron_variant
rs936013625	intron_variant
rs936052248	genic_upstream_transcript_variant,intron_variant
rs936130561	genic_downstream_transcript_variant,intron_variant
rs936170881	genic_upstream_transcript_variant,intron_variant
rs936203188	genic_upstream_transcript_variant,intron_variant
rs936204635	genic_downstream_transcript_variant,intron_variant
rs936241207	genic_downstream_transcript_variant,intron_variant
rs936263002	genic_downstream_transcript_variant,intron_variant
rs936281679	genic_downstream_transcript_variant,intron_variant
rs936300686	genic_downstream_transcript_variant,intron_variant
rs936312565	intron_variant
rs936327777	upstream_transcript_variant,2KB_upstream_variant,genic_upstream_transcript_variant,intron_variant
rs936331420	genic_upstream_transcript_variant,intron_variant
rs936339140	intron_variant
rs936358635	intron_variant
rs936376615	genic_upstream_transcript_variant,intron_variant
rs936401139	genic_upstream_transcript_variant,intron_variant
rs936431047	genic_upstream_transcript_variant,intron_variant
rs936435144	genic_downstream_transcript_variant,intron_variant
rs936454205	intron_variant
rs936461199	intron_variant
rs936463653	upstream_transcript_variant,2KB_upstream_variant,genic_upstream_transcript_variant,intron_variant
rs936478068	genic_downstream_transcript_variant,intron_variant
rs936510159	genic_upstream_transcript_variant,intron_variant
rs936513194	intron_variant
rs936570295	genic_upstream_transcript_variant,intron_variant
rs936598431	5_prime_UTR_variant,intron_variant
rs936652013	genic_upstream_transcript_variant,intron_variant
rs936666863	intron_variant
rs936670886	genic_upstream_transcript_variant,intron_variant
rs936723909	3_prime_UTR_variant,genic_downstream_transcript_variant,non_coding_transcript_variant
rs936739142	intron_variant
rs936746255	genic_upstream_transcript_variant,intron_variant
rs936780067	intron_variant
rs936782478	3_prime_UTR_variant,genic_downstream_transcript_variant,non_coding_transcript_variant
rs936803610	intron_variant
rs936822958	genic_downstream_transcript_variant,intron_variant
rs936823327	genic_upstream_transcript_variant,intron_variant
rs936833179	intron_variant
rs936850416	genic_upstream_transcript_variant,intron_variant
rs936857150	intron_variant
rs936859381	3_prime_UTR_variant,genic_downstream_transcript_variant,non_coding_transcript_variant
rs936925051	genic_upstream_transcript_variant,intron_variant
rs936948928	intron_variant
rs936968640	intron_variant
rs936993871	genic_upstream_transcript_variant,2KB_upstream_variant,upstream_transcript_variant,intron_variant
rs936999033	genic_downstream_transcript_variant,intron_variant
rs937050407	intron_variant
rs937055013	genic_upstream_transcript_variant,intron_variant
rs937057080	genic_upstream_transcript_variant,intron_variant
rs937065345	genic_upstream_transcript_variant,intron_variant

rs937101455	intron_variant
rs937144514	intron_variant
rs937176316	genic_upstream_transcript_variant,intron_variant
rs937223581	3_prime_UTR_variant,genic_downstream_transcript_variant,non_coding_transcript_variant
rs937291155	intron_variant
rs937296625	genic_upstream_transcript_variant,intron_variant
rs937297140	genic_upstream_transcript_variant,intron_variant
rs937299753	genic_downstream_transcript_variant,intron_variant
rs937323671	genic_upstream_transcript_variant,intron_variant
rs937383806	genic_upstream_transcript_variant,intron_variant
rs937401600	genic_upstream_transcript_variant,intron_variant
rs937435487	genic_downstream_transcript_variant,intron_variant
rs937444740	genic_upstream_transcript_variant,intron_variant
rs937452278	intron_variant
rs937470438	genic_downstream_transcript_variant,intron_variant
rs937502443	intron_variant
rs937515485	intron_variant
rs937532828	genic_upstream_transcript_variant,intron_variant
rs937547672	genic_upstream_transcript_variant,intron_variant
rs937580981	genic_upstream_transcript_variant,intron_variant
rs937623524	genic_downstream_transcript_variant,intron_variant
rs937624021	genic_downstream_transcript_variant,intron_variant
rs937661532	intron_variant
rs937721111	intron_variant
rs937750774	intron_variant
rs937759983	intron_variant
rs937777073	genic_upstream_transcript_variant,intron_variant
rs937828223	intron_variant
rs937849987	genic_upstream_transcript_variant,intron_variant
rs937859073	intron_variant
rs937889207	genic_upstream_transcript_variant,intron_variant
rs937893779	genic_downstream_transcript_variant,intron_variant
rs937910225	genic_upstream_transcript_variant,intron_variant
rs937922752	intron_variant
rs937943440	genic_downstream_transcript_variant,intron_variant
rs937959400	genic_downstream_transcript_variant,intron_variant
rs937991681	genic_upstream_transcript_variant,intron_variant
rs937998109	intron_variant
rs938017988	intron_variant
rs938046205	3_prime_UTR_variant,genic_downstream_transcript_variant,non_coding_transcript_variant
rs938061303	3_prime_UTR_variant,genic_downstream_transcript_variant,non_coding_transcript_variant
rs938110913	genic_upstream_transcript_variant,intron_variant
rs938134988	intron_variant
rs938141546	intron_variant
rs938189181	genic_downstream_transcript_variant,intron_variant
rs938193388	intron_variant
rs938258857	intron_variant
rs938280490	genic_upstream_transcript_variant,intron_variant
rs938299267	intron_variant
rs938303503	genic_downstream_transcript_variant,intron_variant
rs938303829	genic_downstream_transcript_variant,intron_variant
rs938304724	upstream_transcript_variant,2KB_upstream_variant,genic_upstream_transcript_variant,intron_variant
rs938339237	intron_variant
rs938348784	genic_downstream_transcript_variant,intron_variant
rs938403137	genic_upstream_transcript_variant,intron_variant
rs938436871	intron_variant
rs938441174	genic_downstream_transcript_variant,intron_variant
rs938469147	genic_upstream_transcript_variant,intron_variant
rs938472264	genic_downstream_transcript_variant,intron_variant
rs938497101	genic_downstream_transcript_variant,intron_variant
rs938520332	intron_variant
rs938525231	genic_upstream_transcript_variant,intron_variant
rs938542753	genic_upstream_transcript_variant,intron_variant
rs938569312	genic_upstream_transcript_variant,2KB_upstream_variant,upstream_transcript_variant,intron_variant
rs938572106	genic_upstream_transcript_variant,intron_variant
rs938573873	genic_upstream_transcript_variant,intron_variant
rs938595021	intron_variant
rs938598021	intron_variant
rs938651757	intron_variant
rs938678247	intron_variant
rs938723512	genic_downstream_transcript_variant,intron_variant
rs938730378	genic_downstream_transcript_variant,intron_variant
rs938754405	genic_downstream_transcript_variant,intron_variant
rs938815885	intron_variant

rs938839759	intron_variant
rs938862857	genic_upstream_transcript_variant,intron_variant
rs938867164	genic_downstream_transcript_variant,intron_variant
rs938868857	genic_downstream_transcript_variant,intron_variant
rs938917966	intron_variant
rs938934187	genic_upstream_transcript_variant,intron_variant
rs938958370	genic_upstream_transcript_variant,intron_variant
rs938973651	intron_variant
rs938976885	intron_variant
rs939004246	intron_variant
rs939043131	genic_upstream_transcript_variant,intron_variant
rs939049796	intron_variant
rs939059793	genic_downstream_transcript_variant,intron_variant
rs939091174	genic_downstream_transcript_variant,intron_variant
rs939115438	genic_upstream_transcript_variant,intron_variant
rs939122938	intron_variant
rs939140460	upstream_transcript_variant,2KB_upstream_variant,genic_upstream_transcript_variant,intron_variant
rs939155786	intron_variant
rs939172061	genic_upstream_transcript_variant,intron_variant
rs939204138	genic_downstream_transcript_variant,intron_variant
rs939216567	intron_variant
rs939283763	genic_upstream_transcript_variant,intron_variant
rs939288109	genic_upstream_transcript_variant,intron_variant
rs939318211	genic_downstream_transcript_variant,intron_variant
rs939324967	genic_upstream_transcript_variant,intron_variant
rs939336247	genic_upstream_transcript_variant,intron_variant
rs939350717	genic_upstream_transcript_variant,intron_variant
rs939356027	genic_upstream_transcript_variant,intron_variant
rs939382956	genic_upstream_transcript_variant,intron_variant
rs939392539	intron_variant
rs939392609	intron_variant
rs939414657	genic_upstream_transcript_variant,intron_variant
rs939425857	intron_variant
rs939444909	intron_variant
rs939517824	genic_upstream_transcript_variant,intron_variant
rs939525476	genic_upstream_transcript_variant,intron_variant
rs939526510	genic_upstream_transcript_variant,intron_variant
rs939541474	intron_variant
rs939557226	intron_variant
rs939578264	genic_upstream_transcript_variant,intron_variant
rs939621662	genic_upstream_transcript_variant,intron_variant
rs939641148	genic_downstream_transcript_variant,intron_variant
rs939678861	genic_downstream_transcript_variant,intron_variant
rs939680786	intron_variant
rs939709876	genic_downstream_transcript_variant,intron_variant
rs939788470	intron_variant
rs939799168	genic_downstream_transcript_variant,intron_variant
rs939802797	genic_upstream_transcript_variant,intron_variant
rs939827002	genic_upstream_transcript_variant,2KB_upstream_variant,upstream_transcript_variant,intron_variant
rs939868348	genic_downstream_transcript_variant,intron_variant
rs939887784	genic_upstream_transcript_variant,intron_variant
rs939937790	genic_upstream_transcript_variant,intron_variant
rs939938310	intron_variant
rs939940706	genic_upstream_transcript_variant,2KB_upstream_variant,upstream_transcript_variant,intron_variant
rs939970405	intron_variant
rs940013276	intron_variant
rs940017518	genic_downstream_transcript_variant,intron_variant
rs940028464	intron_variant
rs940053396	genic_upstream_transcript_variant,intron_variant
rs940060335	upstream_transcript_variant,2KB_upstream_variant,genic_upstream_transcript_variant,intron_variant
rs940081872	intron_variant
rs940106409	intron_variant
rs940109529	intron_variant
rs940134190	intron_variant
rs940156517	genic_upstream_transcript_variant,intron_variant
rs940156877	upstream_transcript_variant,2KB_upstream_variant,genic_upstream_transcript_variant,intron_variant
rs940188755	upstream_transcript_variant,2KB_upstream_variant,genic_upstream_transcript_variant,intron_variant
rs940232843	genic_upstream_transcript_variant,intron_variant
rs940260537	intron_variant
rs940276053	genic_downstream_transcript_variant,intron_variant
rs940307815	genic_downstream_transcript_variant,intron_variant
rs940313868	intron_variant
rs940330993	genic_downstream_transcript_variant,intron_variant
rs940361468	genic_upstream_transcript_variant,intron_variant

rs940380581	genic_upstream_transcript_variant,intron_variant
rs940384331	intron_variant
rs940411801	genic_upstream_transcript_variant,intron_variant
rs940419521	intron_variant
rs940461813	intron_variant
rs940485001	intron_variant
rs940489444	upstream_transcript_variant,2KB_upstream_variant,genic_upstream_transcript_variant,intron_variant
rs940508284	genic_downstream_transcript_variant,intron_variant
rs940601132	genic_downstream_transcript_variant,intron_variant
rs940609502	intron_variant
rs940618370	genic_downstream_transcript_variant,intron_variant
rs940689317	intron_variant
rs940699078	intron_variant
rs940719470	genic_upstream_transcript_variant,intron_variant
rs940725075	genic_upstream_transcript_variant,intron_variant
rs940740275	intron_variant
rs940765294	genic_upstream_transcript_variant,intron_variant
rs940799198	genic_upstream_transcript_variant,intron_variant
rs940851158	intron_variant
rs940861258	genic_upstream_transcript_variant,intron_variant
rs940885371	genic_upstream_transcript_variant,intron_variant
rs940886110	3_prime_UTR_variant,genic_downstream_transcript_variant,non_coding_transcript_variant
rs940897418	genic_upstream_transcript_variant,intron_variant
rs940922335	genic_upstream_transcript_variant,2KB_upstream_variant,upstream_transcript_variant,intron_variant
rs940930842	genic_upstream_transcript_variant,intron_variant
rs940974480	genic_upstream_transcript_variant,intron_variant
rs940981549	intron_variant
rs940995413	intron_variant
rs940997893	genic_upstream_transcript_variant,intron_variant
rs941008420	intron_variant
rs941012514	genic_downstream_transcript_variant,intron_variant
rs941033299	intron_variant
rs941038623	genic_upstream_transcript_variant,intron_variant
rs941039631	genic_upstream_transcript_variant,intron_variant
rs941074853	genic_upstream_transcript_variant,intron_variant
rs941129606	genic_upstream_transcript_variant,intron_variant
rs941134920	genic_downstream_transcript_variant,intron_variant
rs941161667	genic_downstream_transcript_variant,intron_variant
rs941161867	intron_variant
rs941175328	genic_downstream_transcript_variant,intron_variant
rs941228338	intron_variant
rs941260929	5_prime_UTR_variant,intron_variant,genic_upstream_transcript_variant,non_coding_transcript_variant
rs941263849	intron_variant
rs941267546	genic_downstream_transcript_variant,intron_variant
rs941313459	genic_upstream_transcript_variant,intron_variant
rs941321234	intron_variant
rs941333355	genic_upstream_transcript_variant,intron_variant
rs941339780	genic_upstream_transcript_variant,intron_variant
rs941382732	genic_downstream_transcript_variant,intron_variant
rs941386557	genic_downstream_transcript_variant,intron_variant
rs941425004	genic_upstream_transcript_variant,intron_variant
rs941427380	intron_variant
rs941458029	intron_variant
rs941480972	genic_downstream_transcript_variant,intron_variant
rs941524871	genic_upstream_transcript_variant,intron_variant
rs941557918	genic_upstream_transcript_variant,intron_variant
rs941568588	intron_variant
rs941591353	intron_variant
rs941600853	intron_variant
rs941691862	intron_variant
rs941694127	genic_upstream_transcript_variant,intron_variant
rs941714005	intron_variant
rs941742595	intron_variant
rs941757308	3_prime_UTR_variant,genic_downstream_transcript_variant,non_coding_transcript_variant
rs941776285	intron_variant
rs941826424	genic_downstream_transcript_variant,intron_variant
rs941835625	3_prime_UTR_variant,genic_downstream_transcript_variant,non_coding_transcript_variant
rs941865663	genic_upstream_transcript_variant,intron_variant
rs941887833	500B_downstream_variant,downstream_transcript_variant
rs941938398	intron_variant
rs941972508	intron_variant
rs941980220	genic_upstream_transcript_variant,intron_variant
rs941998316	intron_variant
rs942002767	genic_downstream_transcript_variant,intron_variant

rs942033780	genic_downstream_transcript_variant,intron_variant
rs942046258	genic_upstream_transcript_variant,intron_variant
rs942055633	genic_upstream_transcript_variant,intron_variant
rs942077373	genic_downstream_transcript_variant,intron_variant
rs942115831	genic_upstream_transcript_variant,intron_variant
rs942117211	intron_variant
rs942118136	intron_variant
rs942182192	intron_variant
rs942188747	genic_upstream_transcript_variant,2KB_upstream_variant,upstream_transcript_variant,intron_variant
rs942266229	intron_variant
rs942277007	intron_variant
rs942278716	genic_downstream_transcript_variant,intron_variant
rs942317838	genic_downstream_transcript_variant,intron_variant
rs942322578	synonymous_variant,coding_sequence_variant,non_coding_transcript_variant
rs942323644	intron_variant
rs942392921	genic_upstream_transcript_variant,intron_variant
rs942424476	intron_variant
rs942424628	genic_upstream_transcript_variant,intron_variant
rs942453578	genic_downstream_transcript_variant,intron_variant
rs942465958	genic_upstream_transcript_variant,intron_variant
rs942489223	intron_variant
rs942522674	genic_downstream_transcript_variant,intron_variant
rs942580751	intron_variant
rs942583041	genic_upstream_transcript_variant,intron_variant
rs942594446	genic_upstream_transcript_variant,intron_variant
rs942595743	genic_upstream_transcript_variant,intron_variant
rs942617207	genic_downstream_transcript_variant,intron_variant
rs942639009	genic_downstream_transcript_variant,intron_variant
rs942665095	intron_variant
rs942700438	intron_variant
rs942704772	intron_variant
rs942737540	genic_downstream_transcript_variant,intron_variant
rs942764424	intron_variant
rs942830301	genic_upstream_transcript_variant,intron_variant
rs942848558	genic_downstream_transcript_variant,intron_variant
rs942856825	intron_variant
rs942978803	genic_upstream_transcript_variant,intron_variant
rs942981328	intron_variant
rs943030870	intron_variant
rs943044931	intron_variant
rs943094982	intron_variant
rs943116591	genic_downstream_transcript_variant,intron_variant
rs943124976	genic_upstream_transcript_variant,intron_variant
rs943157486	genic_upstream_transcript_variant,intron_variant
rs943188103	genic_upstream_transcript_variant,intron_variant
rs943197266	intron_variant
rs943205876	genic_downstream_transcript_variant,intron_variant
rs943260373	genic_upstream_transcript_variant,intron_variant
rs943284159	intron_variant
rs943315087	intron_variant
rs943318664	genic_upstream_transcript_variant,intron_variant
rs943339570	genic_downstream_transcript_variant,intron_variant
rs943364911	intron_variant
rs943369262	genic_downstream_transcript_variant,intron_variant
rs943394737	intron_variant
rs943424172	intron_variant
rs943426543	intron_variant
rs943454637	intron_variant
rs943476864	intron_variant
rs943544812	genic_downstream_transcript_variant,intron_variant
rs943574535	intron_variant
rs943578232	intron_variant
rs943587627	genic_upstream_transcript_variant,intron_variant
rs943639027	genic_downstream_transcript_variant,intron_variant
rs943655920	intron_variant
rs943668353	genic_upstream_transcript_variant,intron_variant
rs943701294	intron_variant
rs943726770	intron_variant
rs943801476	downstream_transcript_variant,genic_downstream_transcript_variant,intron_variant
rs943854424	genic_upstream_transcript_variant,intron_variant
rs943856313	genic_upstream_transcript_variant,intron_variant
rs943882027	intron_variant
rs943898732	genic_downstream_transcript_variant,intron_variant
rs943965270	genic_downstream_transcript_variant,intron_variant

rs943972301	genic_upstream_transcript_variant,intron_variant
rs944041229	genic_upstream_transcript_variant,intron_variant
rs944044768	intron_variant
rs944049570	genic_upstream_transcript_variant,intron_variant
rs944068241	intron_variant
rs944132411	intron_variant
rs944169248	genic_upstream_transcript_variant,intron_variant
rs944188070	downstream_transcript_variant,genic_downstream_transcript_variant,intron_variant
rs944229161	intron_variant
rs944236271	genic_upstream_transcript_variant,intron_variant
rs944239713	genic_upstream_transcript_variant,upstream_transcript_variant,intron_variant
rs944243856	genic_upstream_transcript_variant,intron_variant
rs944335837	genic_downstream_transcript_variant,intron_variant
rs944404510	genic_upstream_transcript_variant,intron_variant
rs944423793	intron_variant
rs944452999	genic_upstream_transcript_variant,intron_variant
rs944503531	genic_downstream_transcript_variant,intron_variant
rs944507261	genic_upstream_transcript_variant,intron_variant
rs944564418	genic_upstream_transcript_variant,intron_variant
rs944581997	genic_upstream_transcript_variant,intron_variant
rs944589788	genic_upstream_transcript_variant,intron_variant
rs944592557	intron_variant
rs944595130	intron_variant
rs944694811	genic_upstream_transcript_variant,intron_variant
rs944703586	genic_downstream_transcript_variant,intron_variant
rs944708862	genic_downstream_transcript_variant,intron_variant
rs944742832	genic_upstream_transcript_variant,intron_variant
rs944746591	genic_upstream_transcript_variant,intron_variant
rs944797974	intron_variant
rs944810191	intron_variant
rs944813290	intron_variant
rs944836166	genic_upstream_transcript_variant,intron_variant
rs944849603	intron_variant
rs944895498	3_prime_UTR_variant,genic_downstream_transcript_variant,non_coding_transcript_variant
rs944899941	genic_upstream_transcript_variant,intron_variant
rs944955422	intron_variant
rs944957093	intron_variant
rs944972544	genic_downstream_transcript_variant,intron_variant
rs945035708	intron_variant
rs945045960	intron_variant
rs945058733	genic_upstream_transcript_variant,intron_variant
rs945070903	intron_variant
rs945125037	genic_upstream_transcript_variant,intron_variant
rs945178267	intron_variant
rs945192841	genic_downstream_transcript_variant,intron_variant
rs945193793	genic_upstream_transcript_variant,intron_variant
rs945202324	genic_downstream_transcript_variant,intron_variant
rs945211485	genic_upstream_transcript_variant,intron_variant
rs945220313	genic_upstream_transcript_variant,intron_variant
rs945232322	intron_variant
rs945240770	genic_downstream_transcript_variant,intron_variant
rs945272269	intron_variant
rs945297815	genic_upstream_transcript_variant,intron_variant
rs945313994	genic_downstream_transcript_variant,intron_variant
rs945390529	genic_upstream_transcript_variant,intron_variant
rs945406056	5_prime_UTR_variant,genic_upstream_transcript_variant,intron_variant,non_coding_transcript_variant
rs945430184	genic_upstream_transcript_variant,intron_variant
rs945441204	genic_upstream_transcript_variant,intron_variant
rs945454632	3_prime_UTR_variant,genic_downstream_transcript_variant,non_coding_transcript_variant
rs945480908	genic_upstream_transcript_variant,intron_variant
rs945519525	intron_variant
rs945534705	genic_upstream_transcript_variant,intron_variant
rs945552393	intron_variant
rs945576045	genic_upstream_transcript_variant,intron_variant
rs945601619	genic_upstream_transcript_variant,intron_variant
rs945616058	genic_upstream_transcript_variant,intron_variant
rs945636938	synonymous_variant,coding_sequence_variant,non_coding_transcript_variant
rs945703702	2KB_upstream_variant,genic_upstream_transcript_variant,upstream_transcript_variant,intron_variant
rs945711160	2KB_upstream_variant,genic_upstream_transcript_variant,upstream_transcript_variant,intron_variant
rs945752443	genic_upstream_transcript_variant,intron_variant
rs945761764	intron_variant
rs945772377	intron_variant
rs945774532	genic_upstream_transcript_variant,intron_variant
rs945795163	downstream_transcript_variant,500B_downstream_variant

rs945803382	intron_variant
rs945818084	genic_upstream_transcript_variant,intron_variant
rs945868643	genic_upstream_transcript_variant,intron_variant
rs945888704	genic_upstream_transcript_variant,intron_variant
rs945910574	genic_upstream_transcript_variant,intron_variant
rs945915429	missense_variant,coding_sequence_variant,non_coding_transcript_variant
rs945916202	2KB_upstream_variant,genic_upstream_transcript_variant,upstream_transcript_variant,intron_variant
rs945922307	genic_upstream_transcript_variant,intron_variant
rs946026927	intron_variant
rs946031282	genic_downstream_transcript_variant,intron_variant
rs946038990	genic_downstream_transcript_variant,intron_variant
rs946109856	intron_variant
rs946114246	genic_upstream_transcript_variant,intron_variant
rs946155692	genic_downstream_transcript_variant,intron_variant
rs946158093	genic_upstream_transcript_variant,intron_variant
rs946197190	intron_variant
rs946246878	genic_downstream_transcript_variant,intron_variant
rs946271316	genic_downstream_transcript_variant,intron_variant
rs946306400	intron_variant
rs946315162	intron_variant
rs946360554	genic_upstream_transcript_variant,intron_variant
rs946375618	intron_variant
rs946377474	genic_upstream_transcript_variant,intron_variant
rs946383022	genic_upstream_transcript_variant,intron_variant
rs946414613	genic_upstream_transcript_variant,intron_variant
rs946441846	intron_variant
rs946459211	intron_variant
rs946485581	intron_variant
rs946523448	intron_variant
rs946530565	intron_variant
rs946557530	genic_upstream_transcript_variant,intron_variant
rs946582639	genic_upstream_transcript_variant,intron_variant
rs946621099	genic_downstream_transcript_variant,intron_variant
rs946674471	genic_downstream_transcript_variant,intron_variant
rs946706901	genic_downstream_transcript_variant,intron_variant
rs946723610	intron_variant
rs946762573	intron_variant
rs946768626	genic_upstream_transcript_variant,intron_variant
rs946769070	genic_upstream_transcript_variant,intron_variant
rs946776716	genic_upstream_transcript_variant,intron_variant
rs946787264	genic_downstream_transcript_variant,intron_variant
rs946804892	intron_variant
rs946845607	genic_downstream_transcript_variant,intron_variant
rs946860788	upstream_transcript_variant,2KB_upstream_variant,genic_upstream_transcript_variant,intron_variant
rs946894955	genic_downstream_transcript_variant,intron_variant
rs946896919	genic_downstream_transcript_variant,intron_variant
rs946918437	genic_upstream_transcript_variant,intron_variant
rs946948014	genic_upstream_transcript_variant,intron_variant
rs946979178	genic_upstream_transcript_variant,intron_variant
rs946979783	intron_variant
rs946984191	genic_downstream_transcript_variant,intron_variant
rs947001461	intron_variant
rs947031445	genic_upstream_transcript_variant,intron_variant
rs947046038	genic_downstream_transcript_variant,intron_variant
rs947070176	genic_upstream_transcript_variant,intron_variant
rs947084711	intron_variant
rs947088393	genic_upstream_transcript_variant,intron_variant
rs947104715	intron_variant
rs947104858	intron_variant
rs947115031	genic_upstream_transcript_variant,upstream_transcript_variant,intron_variant
rs947133557	genic_upstream_transcript_variant,intron_variant
rs947148276	genic_downstream_transcript_variant,intron_variant
rs947162358	intron_variant
rs947195727	genic_upstream_transcript_variant,intron_variant
rs947252044	genic_upstream_transcript_variant,intron_variant
rs947285516	genic_downstream_transcript_variant,intron_variant
rs947286606	genic_upstream_transcript_variant,intron_variant
rs947315567	intron_variant
rs947317611	genic_upstream_transcript_variant,intron_variant
rs947321313	genic_downstream_transcript_variant,intron_variant
rs947328336	intron_variant
rs947363050	genic_upstream_transcript_variant,intron_variant
rs947386583	intron_variant
rs947406252	intron_variant

rs947409267	intron_variant
rs947449627	genic_upstream_transcript_variant,intron_variant
rs947485270	genic_upstream_transcript_variant,intron_variant
rs947504808	genic_upstream_transcript_variant,intron_variant
rs947546883	genic_upstream_transcript_variant,upstream_transcript_variant,intron_variant
rs947550679	intron_variant
rs947558298	intron_variant
rs947594679	genic_upstream_transcript_variant,intron_variant
rs947613549	genic_upstream_transcript_variant,intron_variant
rs947623206	2KB_upstream_variant,genic_upstream_transcript_variant,upstream_transcript_variant,intron_variant
rs947646118	genic_upstream_transcript_variant,intron_variant
rs947700132	upstream_transcript_variant,2KB_upstream_variant,genic_upstream_transcript_variant,intron_variant
rs947739047	genic_downstream_transcript_variant,intron_variant
rs947764394	genic_upstream_transcript_variant,intron_variant
rs947766576	upstream_transcript_variant,2KB_upstream_variant,genic_upstream_transcript_variant,intron_variant
rs947771129	genic_upstream_transcript_variant,intron_variant
rs947784095	intron_variant
rs947847297	genic_upstream_transcript_variant,intron_variant
rs947880495	genic_upstream_transcript_variant,intron_variant
rs947882403	intron_variant
rs947949854	genic_downstream_transcript_variant,intron_variant
rs948019304	intron_variant
rs948053275	genic_downstream_transcript_variant,intron_variant
rs948053852	genic_downstream_transcript_variant,intron_variant
rs948100918	genic_upstream_transcript_variant,intron_variant
rs948101299	genic_upstream_transcript_variant,intron_variant
rs948118556	intron_variant
rs948124260	2KB_upstream_variant,genic_upstream_transcript_variant,upstream_transcript_variant,intron_variant
rs948130216	intron_variant
rs948144686	intron_variant
rs948168300	genic_downstream_transcript_variant,intron_variant
rs948244961	genic_upstream_transcript_variant,intron_variant
rs948245874	intron_variant
rs948263962	genic_upstream_transcript_variant,intron_variant
rs948279578	genic_upstream_transcript_variant,intron_variant
rs948297002	genic_upstream_transcript_variant,intron_variant
rs948350190	genic_upstream_transcript_variant,upstream_transcript_variant,intron_variant
rs948354471	genic_upstream_transcript_variant,intron_variant
rs948360059	intron_variant
rs948432113	intron_variant
rs948434486	genic_downstream_transcript_variant,intron_variant
rs948456455	genic_upstream_transcript_variant,intron_variant
rs948459169	5_prime_UTR_variant,genic_upstream_transcript_variant,intron_variant,non_coding_transcript_variant
rs948459755	intron_variant
rs948464844	intron_variant
rs948470900	genic_upstream_transcript_variant,intron_variant
rs948473205	5_prime_UTR_variant,genic_upstream_transcript_variant,intron_variant,non_coding_transcript_variant
rs948499579	intron_variant
rs948500064	genic_downstream_transcript_variant,intron_variant
rs948500570	genic_upstream_transcript_variant,intron_variant
rs948561730	genic_downstream_transcript_variant,intron_variant
rs948581524	genic_upstream_transcript_variant,intron_variant
rs948612309	genic_upstream_transcript_variant,upstream_transcript_variant,intron_variant
rs948638373	genic_upstream_transcript_variant,intron_variant
rs948646656	genic_downstream_transcript_variant,intron_variant
rs948659431	genic_upstream_transcript_variant,intron_variant
rs948664287	intron_variant
rs948666333	intron_variant
rs948693196	intron_variant
rs948720943	genic_upstream_transcript_variant,intron_variant
rs948734115	genic_downstream_transcript_variant,intron_variant
rs948761716	genic_upstream_transcript_variant,intron_variant
rs948777033	intron_variant
rs948795817	genic_downstream_transcript_variant,intron_variant
rs948853507	genic_upstream_transcript_variant,intron_variant
rs948862756	intron_variant
rs948863634	genic_downstream_transcript_variant,intron_variant
rs948896472	genic_downstream_transcript_variant,intron_variant
rs948952260	genic_upstream_transcript_variant,intron_variant
rs948955158	intron_variant
rs949000945	intron_variant
rs949001695	genic_upstream_transcript_variant,intron_variant
rs949030256	intron_variant
rs949044011	genic_downstream_transcript_variant,intron_variant

rs949084427	genic_downstream_transcript_variant,intron_variant
rs949102878	genic_upstream_transcript_variant,intron_variant
rs949107252	intron_variant
rs949115024	genic_downstream_transcript_variant,intron_variant
rs949121860	intron_variant
rs949154878	intron_variant
rs949166349	genic_downstream_transcript_variant,intron_variant
rs949268377	genic_upstream_transcript_variant,intron_variant
rs949295930	intron_variant
rs949317105	downstream_transcript_variant,genic_downstream_transcript_variant,intron_variant
rs949318941	genic_upstream_transcript_variant,intron_variant
rs949334018	genic_upstream_transcript_variant,intron_variant
rs949361084	intron_variant
rs949381369	intron_variant
rs949414460	genic_upstream_transcript_variant,intron_variant
rs949417684	genic_upstream_transcript_variant,intron_variant
rs949421010	intron_variant
rs949457882	3_prime_UTR_variant,genic_downstream_transcript_variant,non_coding_transcript_variant
rs949527353	intron_variant
rs949534791	genic_upstream_transcript_variant,intron_variant
rs949539298	genic_downstream_transcript_variant,intron_variant
rs949547891	intron_variant
rs949562610	genic_upstream_transcript_variant,intron_variant
rs949570419	genic_downstream_transcript_variant,intron_variant
rs949601874	genic_upstream_transcript_variant,intron_variant
rs949628131	intron_variant
rs949638688	upstream_transcript_variant,2KB_upstream_variant,genic_upstream_transcript_variant,intron_variant
rs949638974	upstream_transcript_variant,2KB_upstream_variant,genic_upstream_transcript_variant,intron_variant
rs949691189	genic_upstream_transcript_variant,intron_variant
rs949694045	intron_variant
rs949753775	genic_downstream_transcript_variant,intron_variant
rs949765512	missense_variant,genic_upstream_transcript_variant,intron_variant,coding_sequence_variant,non_coding_transcript_variant
rs949810826	intron_variant
rs949869171	genic_downstream_transcript_variant,intron_variant
rs949885756	genic_upstream_transcript_variant,intron_variant
rs949901709	genic_downstream_transcript_variant,intron_variant
rs949941126	intron_variant
rs949958462	intron_variant
rs949969012	genic_upstream_transcript_variant,intron_variant
rs950010800	genic_upstream_transcript_variant,intron_variant
rs950023253	genic_upstream_transcript_variant,intron_variant
rs950077902	genic_upstream_transcript_variant,intron_variant
rs950100784	genic_upstream_transcript_variant,intron_variant
rs950135613	genic_upstream_transcript_variant,intron_variant
rs950143102	intron_variant
rs950211327	genic_upstream_transcript_variant,intron_variant
rs950242886	genic_upstream_transcript_variant,intron_variant
rs950260100	intron_variant
rs950293741	intron_variant
rs950412565	intron_variant
rs950501441	intron_variant
rs950518668	genic_upstream_transcript_variant,intron_variant
rs950567473	intron_variant
rs950583053	intron_variant
rs950590012	genic_upstream_transcript_variant,intron_variant
rs950597106	intron_variant
rs950624770	genic_upstream_transcript_variant,intron_variant
rs950657237	genic_upstream_transcript_variant,intron_variant
rs950659484	genic_upstream_transcript_variant,intron_variant
rs950664695	genic_downstream_transcript_variant,intron_variant
rs950737958	intron_variant
rs950739799	intron_variant
rs950741227	genic_downstream_transcript_variant,intron_variant
rs950778489	intron_variant
rs950817172	genic_downstream_transcript_variant,intron_variant
rs950879791	intron_variant
rs950885374	missense_variant,genic_upstream_transcript_variant,intron_variant,coding_sequence_variant,non_coding_transcript_variant
rs950886444	intron_variant
rs950910799	genic_upstream_transcript_variant,intron_variant
rs950967895	genic_downstream_transcript_variant,intron_variant
rs950977522	intron_variant
rs951053003	intron_variant
rs951061949	intron_variant
rs951080604	intron_variant

rs951142751	genic_downstream_transcript_variant,intron_variant
rs951160206	intron_variant
rs951172371	genic_upstream_transcript_variant,intron_variant
rs951196432	genic_downstream_transcript_variant,intron_variant
rs951203761	intron_variant
rs951234605	genic_downstream_transcript_variant,intron_variant
rs951239111	genic_upstream_transcript_variant,intron_variant
rs951284699	intron_variant
rs951328901	genic_downstream_transcript_variant,intron_variant
rs951352029	genic_downstream_transcript_variant,intron_variant
rs951364963	intron_variant
rs951397048	genic_upstream_transcript_variant,intron_variant
rs951400556	intron_variant
rs951408085	genic_upstream_transcript_variant,intron_variant
rs951421329	genic_upstream_transcript_variant,intron_variant
rs951424321	genic_upstream_transcript_variant,intron_variant
rs951451504	genic_upstream_transcript_variant,intron_variant
rs951483751	genic_upstream_transcript_variant,intron_variant
rs951501402	genic_upstream_transcript_variant,intron_variant
rs951505014	intron_variant
rs951536797	genic_upstream_transcript_variant,intron_variant
rs951542472	intron_variant
rs951574893	genic_upstream_transcript_variant,intron_variant
rs951610837	genic_upstream_transcript_variant,intron_variant
rs951664973	intron_variant
rs951676513	genic_upstream_transcript_variant,intron_variant
rs951676943	3_prime_UTR_variant,genic_downstream_transcript_variant,non_coding_transcript_variant
rs951688640	genic_upstream_transcript_variant,intron_variant
rs951691629	intron_variant
rs951733308	genic_upstream_transcript_variant,intron_variant
rs951734313	intron_variant
rs951784331	genic_upstream_transcript_variant,intron_variant
rs951790254	intron_variant
rs951793364	intron_variant
rs951813236	3_prime_UTR_variant,genic_downstream_transcript_variant,non_coding_transcript_variant
rs951818323	intron_variant
rs951833683	genic_upstream_transcript_variant,intron_variant
rs951852904	intron_variant
rs951875499	genic_downstream_transcript_variant,intron_variant
rs951910729	intron_variant
rs951910998	upstream_transcript_variant,2KB_upstream_variant,genic_upstream_transcript_variant,intron_variant
rs951927034	synonymous_variant,genic_downstream_transcript_variant,coding_sequence_variant,non_coding_transcript_variant
rs951938308	genic_upstream_transcript_variant,intron_variant
rs951949650	genic_downstream_transcript_variant,intron_variant
rs951955453	genic_downstream_transcript_variant,intron_variant
rs951974857	intron_variant
rs951977896	intron_variant
rs952022664	genic_upstream_transcript_variant,intron_variant
rs952035125	intron_variant
rs952058097	intron_variant
rs952061154	genic_upstream_transcript_variant,intron_variant
rs952075414	downstream_transcript_variant,500B_downstream_variant
rs952080437	genic_upstream_transcript_variant,intron_variant
rs952092063	2KB_upstream_variant,genic_upstream_transcript_variant,upstream_transcript_variant,intron_variant
rs952096283	genic_upstream_transcript_variant,intron_variant
rs952113592	genic_upstream_transcript_variant,intron_variant
rs952188821	genic_upstream_transcript_variant,intron_variant
rs952191427	genic_upstream_transcript_variant,intron_variant
rs952204490	intron_variant
rs952207512	genic_upstream_transcript_variant,intron_variant
rs952217925	intron_variant
rs952267811	2KB_upstream_variant,genic_upstream_transcript_variant,upstream_transcript_variant,intron_variant
rs952276854	intron_variant
rs952355045	genic_upstream_transcript_variant,intron_variant
rs952356006	genic_upstream_transcript_variant,intron_variant
rs952394205	genic_upstream_transcript_variant,intron_variant
rs952505203	intron_variant
rs952533058	genic_upstream_transcript_variant,intron_variant
rs952592263	2KB_upstream_variant,genic_upstream_transcript_variant,upstream_transcript_variant,intron_variant
rs952628533	intron_variant
rs952650358	genic_upstream_transcript_variant,intron_variant
rs952662891	intron_variant
rs952665403	intron_variant
rs952709715	upstream_transcript_variant,2KB_upstream_variant,genic_upstream_transcript_variant,intron_variant

rs952820114	intron_variant
rs952871672	genic_upstream_transcript_variant,intron_variant
rs952888824	genic_downstream_transcript_variant,intron_variant
rs952907639	intron_variant
rs952944258	genic_downstream_transcript_variant,intron_variant
rs952960371	genic_upstream_transcript_variant,upstream_transcript_variant,intron_variant
rs952968244	genic_downstream_transcript_variant,intron_variant
rs952978826	genic_upstream_transcript_variant,intron_variant
rs953038424	genic_upstream_transcript_variant,intron_variant
rs953066010	intron_variant
rs953086220	3_prime_UTR_variant,genic_downstream_transcript_variant,non_coding_transcript_variant
rs953112329	2KB_upstream_variant,genic_upstream_transcript_variant,upstream_transcript_variant,intron_variant
rs953123032	intron_variant
rs953137438	intron_variant
rs953139413	intron_variant
rs953142424	genic_downstream_transcript_variant,intron_variant
rs953168404	intron_variant
rs953194200	genic_downstream_transcript_variant,intron_variant
rs953218728	upstream_transcript_variant,2KB_upstream_variant,genic_upstream_transcript_variant,intron_variant
rs953234197	2KB_upstream_variant,genic_upstream_transcript_variant,upstream_transcript_variant,intron_variant
rs953237572	genic_upstream_transcript_variant,intron_variant
rs953238108	genic_upstream_transcript_variant,intron_variant
rs953250144	genic_downstream_transcript_variant,intron_variant
rs953279995	genic_upstream_transcript_variant,intron_variant
rs953306848	genic_downstream_transcript_variant,intron_variant
rs953308814	genic_upstream_transcript_variant,intron_variant
rs953326774	genic_upstream_transcript_variant,intron_variant
rs953347085	genic_downstream_transcript_variant,intron_variant
rs953352465	intron_variant
rs953387489	intron_variant
rs953407799	genic_upstream_transcript_variant,intron_variant
rs953411428	2KB_upstream_variant,genic_upstream_transcript_variant,upstream_transcript_variant,intron_variant
rs953451655	upstream_transcript_variant,2KB_upstream_variant,genic_upstream_transcript_variant,intron_variant
rs953465169	genic_upstream_transcript_variant,intron_variant
rs953539461	genic_upstream_transcript_variant,intron_variant
rs953551447	intron_variant
rs953561418	genic_downstream_transcript_variant,intron_variant
rs953686961	genic_upstream_transcript_variant,intron_variant
rs953750463	intron_variant
rs953801529	genic_upstream_transcript_variant,intron_variant
rs953803645	intron_variant
rs953816066	intron_variant
rs953844543	genic_upstream_transcript_variant,intron_variant
rs953958858	intron_variant
rs953969205	genic_upstream_transcript_variant,intron_variant
rs954009308	upstream_transcript_variant,intron_variant,genic_upstream_transcript_variant,2KB_upstream_variant
rs954031858	intron_variant
rs954038473	intron_variant,genic_upstream_transcript_variant
rs954067534	intron_variant,genic_downstream_transcript_variant
rs954097741	intron_variant
rs954116065	intron_variant,genic_upstream_transcript_variant
rs954146778	intron_variant,genic_downstream_transcript_variant
rs954147188	intron_variant,genic_upstream_transcript_variant
rs954212754	intron_variant,genic_downstream_transcript_variant
rs954244475	intron_variant
rs954256692	intron_variant
rs954275822	intron_variant,downstream_transcript_variant,genic_downstream_transcript_variant
rs954290109	intron_variant
rs954338953	intron_variant,genic_downstream_transcript_variant
rs954340307	intron_variant,genic_upstream_transcript_variant,upstream_transcript_variant,2KB_upstream_variant
rs954341341	intron_variant,genic_upstream_transcript_variant
rs954360561	intron_variant,genic_upstream_transcript_variant
rs954375089	intron_variant,genic_downstream_transcript_variant
rs954407259	intron_variant,genic_downstream_transcript_variant
rs954419715	intron_variant,genic_upstream_transcript_variant
rs954448042	intron_variant,genic_downstream_transcript_variant
rs954448635	intron_variant
rs954505450	intron_variant,genic_downstream_transcript_variant
rs954543426	intron_variant
rs954551250	intron_variant,genic_downstream_transcript_variant
rs954564323	intron_variant,genic_upstream_transcript_variant
rs954654953	intron_variant
rs954668667	intron_variant
rs954670676	intron_variant,genic_upstream_transcript_variant

rs954678195	intron_variant,genic_downstream_transcript_variant
rs954714202	intron_variant
rs954757282	intron_variant
rs954805577	upstream_transcript_variant,5_prime_UTR_variant,intron_variant,genic_upstream_transcript_variant
rs954819857	intron_variant,genic_upstream_transcript_variant
rs954844471	intron_variant
rs954901593	intron_variant
rs954913522	intron_variant
rs954915540	intron_variant,genic_upstream_transcript_variant,upstream_transcript_variant,2KB_upstream_variant
rs954926811	intron_variant
rs954929033	intron_variant,genic_upstream_transcript_variant
rs954947537	intron_variant,genic_upstream_transcript_variant
rs954948813	intron_variant,genic_upstream_transcript_variant
rs954997919	intron_variant,upstream_transcript_variant,genic_upstream_transcript_variant
rs955008706	intron_variant,genic_downstream_transcript_variant
rs955040287	intron_variant,genic_upstream_transcript_variant
rs955054240	intron_variant,genic_upstream_transcript_variant
rs955074604	intron_variant,genic_upstream_transcript_variant
rs955086606	intron_variant
rs955090791	intron_variant,upstream_transcript_variant,genic_upstream_transcript_variant
rs955108480	intron_variant,genic_upstream_transcript_variant
rs955131483	intron_variant,genic_downstream_transcript_variant
rs955144079	intron_variant,genic_downstream_transcript_variant
rs955229338	intron_variant,genic_upstream_transcript_variant
rs955242591	intron_variant
rs955243579	intron_variant
rs955261936	intron_variant,genic_upstream_transcript_variant
rs955262359	intron_variant
rs955272941	intron_variant,genic_upstream_transcript_variant,upstream_transcript_variant,2KB_upstream_variant
rs955280877	intron_variant
rs955282331	intron_variant,genic_upstream_transcript_variant
rs955290653	intron_variant,genic_upstream_transcript_variant
rs955300938	intron_variant,genic_upstream_transcript_variant
rs955326362	intron_variant,genic_downstream_transcript_variant
rs955353017	intron_variant,genic_upstream_transcript_variant
rs955365648	intron_variant
rs955375563	intron_variant
rs955392204	intron_variant
rs955443398	intron_variant,genic_upstream_transcript_variant
rs955443870	intron_variant,genic_upstream_transcript_variant
rs955503771	intron_variant
rs955540411	intron_variant,genic_downstream_transcript_variant
rs955567094	intron_variant,genic_upstream_transcript_variant
rs955597392	intron_variant,genic_upstream_transcript_variant
rs955628663	intron_variant
rs955670792	intron_variant
rs955681263	intron_variant
rs955730089	intron_variant,genic_upstream_transcript_variant
rs955753124	intron_variant,genic_upstream_transcript_variant
rs955765721	intron_variant,genic_downstream_transcript_variant
rs955806532	intron_variant
rs955811841	intron_variant,genic_upstream_transcript_variant
rs955821328	intron_variant,genic_upstream_transcript_variant
rs955836782	intron_variant,genic_downstream_transcript_variant
rs955855423	non_coding_transcript_variant,3_prime_UTR_variant,genic_downstream_transcript_variant
rs955858324	intron_variant,genic_downstream_transcript_variant
rs955873518	intron_variant,genic_upstream_transcript_variant
rs955878524	intron_variant,genic_downstream_transcript_variant
rs955894517	intron_variant,genic_upstream_transcript_variant
rs955912675	intron_variant
rs955928599	intron_variant,genic_upstream_transcript_variant
rs955943264	intron_variant
rs955957142	intron_variant,upstream_transcript_variant,genic_upstream_transcript_variant
rs955999291	intron_variant,genic_upstream_transcript_variant
rs956023923	intron_variant
rs956034601	intron_variant
rs956048117	intron_variant
rs956096507	intron_variant,genic_downstream_transcript_variant
rs956105088	intron_variant
rs956110498	intron_variant,genic_upstream_transcript_variant
rs956142707	intron_variant
rs956179665	intron_variant
rs956208850	intron_variant
rs956218214	intron_variant,upstream_transcript_variant,genic_upstream_transcript_variant

rs956223063	intron_variant
rs956233399	intron_variant,genic_downstream_transcript_variant
rs956256379	intron_variant,genic_upstream_transcript_variant
rs956284675	intron_variant
rs956328465	intron_variant,genic_upstream_transcript_variant
rs956385253	intron_variant
rs956403076	intron_variant
rs956430569	intron_variant,genic_upstream_transcript_variant
rs956432937	intron_variant,genic_downstream_transcript_variant
rs956448237	intron_variant,genic_upstream_transcript_variant
rs956484167	intron_variant
rs956490139	intron_variant,genic_upstream_transcript_variant
rs956502956	intron_variant,genic_upstream_transcript_variant
rs956524122	intron_variant,genic_upstream_transcript_variant
rs956590814	intron_variant,genic_upstream_transcript_variant
rs956597576	intron_variant,genic_upstream_transcript_variant
rs956612804	intron_variant
rs956640223	intron_variant
rs956655362	intron_variant,genic_upstream_transcript_variant
rs956672615	intron_variant
rs956716543	intron_variant,genic_upstream_transcript_variant
rs956723530	intron_variant
rs956740304	intron_variant
rs956752638	intron_variant,genic_upstream_transcript_variant
rs956775547	intron_variant,genic_downstream_transcript_variant
rs956827205	missense_variant,non_coding_transcript_variant,genic_downstream_transcript_variant,coding_sequence_variant
rs956842708	intron_variant,genic_downstream_transcript_variant
rs956858347	intron_variant
rs956893669	intron_variant,genic_upstream_transcript_variant
rs956975159	intron_variant,genic_downstream_transcript_variant
rs956988171	intron_variant
rs957003429	intron_variant,genic_upstream_transcript_variant
rs957006381	intron_variant
rs957018595	intron_variant
rs957048930	intron_variant
rs957090321	intron_variant,genic_downstream_transcript_variant
rs957096455	intron_variant,genic_downstream_transcript_variant
rs957098381	intron_variant
rs957112748	intron_variant
rs957164450	intron_variant,genic_upstream_transcript_variant
rs957206460	intron_variant,genic_upstream_transcript_variant
rs957239330	intron_variant,genic_downstream_transcript_variant
rs957286760	intron_variant
rs957299889	intron_variant
rs957321826	non_coding_transcript_variant,3_prime_UTR_variant,genic_downstream_transcript_variant
rs957331150	intron_variant
rs957353501	intron_variant,genic_downstream_transcript_variant
rs957360240	intron_variant
rs957364729	intron_variant,genic_upstream_transcript_variant
rs957369120	intron_variant
rs957398810	intron_variant,genic_downstream_transcript_variant
rs957399845	intron_variant,genic_upstream_transcript_variant
rs957417995	intron_variant,genic_upstream_transcript_variant
rs957419248	intron_variant,genic_upstream_transcript_variant
rs957448661	intron_variant,genic_upstream_transcript_variant
rs957452788	intron_variant
rs957491171	intron_variant,genic_upstream_transcript_variant
rs957500862	intron_variant,genic_upstream_transcript_variant,upstream_transcript_variant,2KB_upstream_variant
rs957519994	intron_variant,genic_upstream_transcript_variant
rs957524344	intron_variant,genic_downstream_transcript_variant
rs957531308	intron_variant
rs957551179	intron_variant,genic_upstream_transcript_variant
rs957556005	intron_variant,genic_upstream_transcript_variant
rs957569916	intron_variant
rs957576419	intron_variant,genic_downstream_transcript_variant
rs957600992	intron_variant,genic_upstream_transcript_variant
rs957608977	intron_variant
rs957635634	intron_variant,genic_downstream_transcript_variant
rs957660407	intron_variant
rs957666091	intron_variant
rs957726590	intron_variant,genic_upstream_transcript_variant
rs957750045	upstream_transcript_variant,intron_variant,genic_upstream_transcript_variant,2KB_upstream_variant
rs957795484	intron_variant
rs957854291	intron_variant,genic_upstream_transcript_variant

rs957882896	upstream_transcript_variant,intron_variant,genic_upstream_transcript_variant,2KB_upstream_variant
rs957884483	intron_variant,genic_upstream_transcript_variant
rs957904649	intron_variant,genic_downstream_transcript_variant
rs957908824	intron_variant
rs957914699	intron_variant,genic_upstream_transcript_variant
rs957932359	intron_variant
rs958006275	intron_variant
rs958007364	intron_variant,genic_upstream_transcript_variant
rs958041666	intron_variant
rs958057365	intron_variant,genic_downstream_transcript_variant
rs958077211	intron_variant
rs958141288	intron_variant,genic_upstream_transcript_variant
rs958171282	intron_variant,genic_downstream_transcript_variant
rs958186057	non_coding_transcript_variant,3_prime_UTR_variant,genic_downstream_transcript_variant
rs958190197	intron_variant,genic_upstream_transcript_variant
rs958202578	intron_variant,genic_downstream_transcript_variant
rs958207744	intron_variant,genic_upstream_transcript_variant
rs958223072	intron_variant
rs958249902	intron_variant,genic_upstream_transcript_variant
rs958294696	intron_variant,genic_upstream_transcript_variant
rs958298442	intron_variant,genic_upstream_transcript_variant
rs958319884	non_coding_transcript_variant,3_prime_UTR_variant,genic_downstream_transcript_variant
rs958331591	intron_variant,genic_upstream_transcript_variant,upstream_transcript_variant,2KB_upstream_variant
rs958386295	intron_variant,genic_upstream_transcript_variant
rs958398830	intron_variant
rs958421392	intron_variant
rs958425362	intron_variant,genic_upstream_transcript_variant,upstream_transcript_variant,2KB_upstream_variant
rs958447975	intron_variant
rs958553879	intron_variant
rs958596529	intron_variant,genic_upstream_transcript_variant
rs958623193	intron_variant,genic_downstream_transcript_variant
rs958634375	intron_variant,genic_upstream_transcript_variant
rs958673682	intron_variant
rs958742211	intron_variant,genic_upstream_transcript_variant
rs958757850	intron_variant,genic_upstream_transcript_variant
rs958775220	intron_variant,genic_downstream_transcript_variant
rs958781890	intron_variant
rs958785228	intron_variant,genic_upstream_transcript_variant
rs958893416	intron_variant
rs958910809	intron_variant
rs958928340	intron_variant,genic_downstream_transcript_variant
rs958932854	intron_variant
rs958980696	intron_variant,genic_upstream_transcript_variant
rs958995697	intron_variant,genic_downstream_transcript_variant
rs959007774	intron_variant
rs959055914	intron_variant,genic_upstream_transcript_variant
rs959112855	intron_variant,genic_upstream_transcript_variant
rs959140310	intron_variant,genic_downstream_transcript_variant
rs959142856	intron_variant
rs959197596	intron_variant,genic_downstream_transcript_variant
rs959210215	upstream_transcript_variant,intron_variant,genic_upstream_transcript_variant,2KB_upstream_variant
rs959211105	intron_variant,genic_upstream_transcript_variant
rs959235796	intron_variant
rs959238978	intron_variant,genic_upstream_transcript_variant
rs959294309	intron_variant,genic_downstream_transcript_variant
rs959323092	intron_variant,genic_upstream_transcript_variant
rs959353480	intron_variant
rs959360621	intron_variant,genic_upstream_transcript_variant
rs959424835	intron_variant,genic_upstream_transcript_variant
rs959432467	intron_variant,genic_downstream_transcript_variant
rs959468785	intron_variant
rs959506329	intron_variant
rs959508015	intron_variant,genic_downstream_transcript_variant
rs959615656	intron_variant,genic_upstream_transcript_variant
rs959625002	500B_downstream_variant,downstream_transcript_variant
rs959677295	intron_variant
rs959749472	intron_variant
rs959775515	intron_variant,genic_downstream_transcript_variant
rs959825983	intron_variant,genic_upstream_transcript_variant
rs959828891	intron_variant,genic_downstream_transcript_variant
rs959831909	intron_variant,genic_upstream_transcript_variant
rs959832841	intron_variant
rs959841200	intron_variant
rs959908104	intron_variant

rs959918754	intron_variant,genic_downstream_transcript_variant
rs959925049	intron_variant,upstream_transcript_variant,genic_upstream_transcript_variant
rs959940426	intron_variant,genic_upstream_transcript_variant
rs959957077	intron_variant,genic_upstream_transcript_variant
rs959971398	intron_variant,genic_downstream_transcript_variant
rs959979677	intron_variant
rs960005386	intron_variant,genic_upstream_transcript_variant
rs960010988	intron_variant
rs960050267	intron_variant
rs960068507	intron_variant,genic_upstream_transcript_variant
rs960068655	intron_variant,genic_upstream_transcript_variant
rs960145378	intron_variant
rs960154393	intron_variant,genic_downstream_transcript_variant
rs960173052	intron_variant
rs960202065	intron_variant
rs960220357	intron_variant,genic_upstream_transcript_variant
rs960243191	intron_variant,genic_downstream_transcript_variant
rs960256756	intron_variant
rs960269559	intron_variant
rs960313441	intron_variant,genic_downstream_transcript_variant
rs960314842	intron_variant,genic_downstream_transcript_variant
rs960318632	intron_variant,genic_upstream_transcript_variant
rs960356463	intron_variant
rs960371921	intron_variant,genic_downstream_transcript_variant
rs960381670	intron_variant
rs960496419	intron_variant,genic_upstream_transcript_variant
rs960505947	intron_variant,genic_upstream_transcript_variant
rs960516478	intron_variant,genic_upstream_transcript_variant
rs960551179	intron_variant,genic_upstream_transcript_variant
rs960555627	intron_variant
rs960600659	intron_variant
rs960601416	intron_variant,genic_upstream_transcript_variant
rs960643054	intron_variant,genic_downstream_transcript_variant
rs960647405	intron_variant,genic_downstream_transcript_variant
rs960665404	intron_variant,genic_upstream_transcript_variant
rs960695652	intron_variant
rs960734673	intron_variant
rs960739036	intron_variant,genic_downstream_transcript_variant
rs960761852	intron_variant,genic_upstream_transcript_variant
rs960762932	intron_variant,genic_upstream_transcript_variant
rs960783016	intron_variant,genic_upstream_transcript_variant
rs960786149	intron_variant
rs960853613	intron_variant,genic_upstream_transcript_variant
rs960875934	intron_variant,genic_upstream_transcript_variant
rs960884219	intron_variant
rs960920696	intron_variant,genic_upstream_transcript_variant
rs960981516	intron_variant,genic_upstream_transcript_variant
rs960994325	intron_variant,genic_downstream_transcript_variant
rs961031153	intron_variant,genic_downstream_transcript_variant
rs961048835	intron_variant,genic_upstream_transcript_variant
rs961090362	intron_variant,genic_upstream_transcript_variant
rs961093661	intron_variant,genic_downstream_transcript_variant
rs961097886	intron_variant,genic_upstream_transcript_variant
rs961149186	intron_variant,genic_downstream_transcript_variant
rs961149529	intron_variant,genic_upstream_transcript_variant
rs961150281	intron_variant,genic_upstream_transcript_variant
rs961172742	intron_variant,genic_upstream_transcript_variant
rs961248187	intron_variant
rs961286211	intron_variant,genic_downstream_transcript_variant
rs961324016	intron_variant,genic_upstream_transcript_variant
rs961373985	intron_variant,genic_upstream_transcript_variant
rs961399228	intron_variant,genic_downstream_transcript_variant
rs961407562	intron_variant
rs961461768	intron_variant,genic_downstream_transcript_variant
rs961464136	upstream_transcript_variant,5_prime_UTR_variant,intron_variant,genic_upstream_transcript_variant
rs961498929	intron_variant
rs961520938	intron_variant,genic_upstream_transcript_variant
rs961570762	intron_variant,genic_upstream_transcript_variant
rs961575767	intron_variant,genic_downstream_transcript_variant
rs961605471	intron_variant,genic_downstream_transcript_variant
rs961608317	intron_variant
rs961651356	intron_variant
rs961655247	intron_variant,genic_upstream_transcript_variant
rs961659390	intron_variant,genic_upstream_transcript_variant

rs961666135	intron_variant
rs961687032	intron_variant
rs961687972	non_coding_transcript_variant,3_prime_UTR_variant,genic_downstream_transcript_variant
rs961703436	intron_variant
rs961705412	intron_variant,genic_upstream_transcript_variant
rs961721103	non_coding_transcript_variant,3_prime_UTR_variant,genic_downstream_transcript_variant
rs961723831	intron_variant,genic_upstream_transcript_variant
rs961734858	intron_variant
rs961778582	intron_variant
rs961794488	intron_variant,genic_upstream_transcript_variant
rs961820546	intron_variant,genic_downstream_transcript_variant
rs961847040	intron_variant
rs961850779	intron_variant
rs961860641	intron_variant,genic_downstream_transcript_variant
rs961900170	intron_variant,genic_downstream_transcript_variant
rs961913134	intron_variant
rs961917117	upstream_transcript_variant,intron_variant,genic_upstream_transcript_variant,2KB_upstream_variant
rs961925839	intron_variant,genic_downstream_transcript_variant
rs961937622	intron_variant
rs961988580	intron_variant
rs962012523	intron_variant
rs962027324	intron_variant,genic_upstream_transcript_variant
rs962036419	intron_variant,genic_upstream_transcript_variant,upstream_transcript_variant,2KB_upstream_variant
rs962075222	intron_variant,genic_upstream_transcript_variant
rs962107253	intron_variant,upstream_transcript_variant,genic_upstream_transcript_variant
rs962166816	intron_variant
rs962183175	intron_variant,genic_upstream_transcript_variant
rs962188753	intron_variant,genic_downstream_transcript_variant
rs962231860	intron_variant,genic_upstream_transcript_variant
rs962276391	intron_variant
rs962279628	intron_variant,genic_downstream_transcript_variant
rs962300514	intron_variant,genic_downstream_transcript_variant
rs962315738	intron_variant,genic_upstream_transcript_variant
rs962339536	intron_variant,upstream_transcript_variant,genic_upstream_transcript_variant
rs962343831	intron_variant
rs962369975	intron_variant,genic_upstream_transcript_variant
rs962393881	intron_variant,genic_upstream_transcript_variant,upstream_transcript_variant,2KB_upstream_variant
rs962411186	intron_variant
rs962462996	intron_variant
rs962467155	intron_variant,genic_upstream_transcript_variant
rs962476637	intron_variant
rs962499004	intron_variant
rs962511525	intron_variant,genic_upstream_transcript_variant
rs962513988	intron_variant,genic_upstream_transcript_variant
rs962527664	intron_variant,genic_upstream_transcript_variant
rs962548379	intron_variant
rs962571275	intron_variant,genic_downstream_transcript_variant
rs962587300	intron_variant,genic_upstream_transcript_variant
rs962629185	intron_variant,genic_downstream_transcript_variant
rs962641686	non_coding_transcript_variant,synonymous_variant,coding_sequence_variant
rs962682869	intron_variant,genic_upstream_transcript_variant
rs962705201	intron_variant
rs962711920	intron_variant
rs962736958	intron_variant,genic_upstream_transcript_variant
rs962850351	intron_variant
rs962854252	intron_variant
rs962872462	intron_variant,genic_downstream_transcript_variant
rs962958150	intron_variant,genic_upstream_transcript_variant
rs962964191	intron_variant
rs963028788	intron_variant,genic_upstream_transcript_variant
rs963037610	intron_variant,genic_upstream_transcript_variant
rs963119814	intron_variant
rs963125161	intron_variant
rs963192588	intron_variant
rs963251827	intron_variant,genic_upstream_transcript_variant
rs963266550	intron_variant,genic_upstream_transcript_variant
rs963268857	intron_variant,genic_upstream_transcript_variant
rs963338663	intron_variant,genic_upstream_transcript_variant
rs963359406	intron_variant
rs963430823	intron_variant
rs963477772	intron_variant,genic_downstream_transcript_variant
rs963496991	non_coding_transcript_variant,3_prime_UTR_variant,genic_downstream_transcript_variant
rs963525945	intron_variant,genic_upstream_transcript_variant
rs963547880	intron_variant,genic_upstream_transcript_variant

rs963595693	intron_variant,genic_downstream_transcript_variant
rs963605171	intron_variant,genic_downstream_transcript_variant
rs963627764	intron_variant,genic_downstream_transcript_variant
rs963655789	intron_variant
rs963663507	5_prime_UTR_variant,non_coding_transcript_variant,missense_variant,coding_sequence_variant,intron_variant,genic_upstream_transcript_variant
rs963695930	intron_variant
rs963733322	intron_variant,genic_upstream_transcript_variant
rs963787409	intron_variant
rs963793863	intron_variant,genic_upstream_transcript_variant
rs963809486	intron_variant,genic_downstream_transcript_variant
rs963845321	intron_variant,genic_downstream_transcript_variant
rs963858287	intron_variant
rs963906537	intron_variant,genic_upstream_transcript_variant
rs963936254	intron_variant,genic_upstream_transcript_variant
rs963943694	intron_variant
rs963948948	intron_variant
rs963954054	intron_variant
rs963985352	intron_variant
rs964037056	intron_variant
rs964071324	intron_variant,genic_upstream_transcript_variant
rs964090669	intron_variant
rs964131832	intron_variant,genic_downstream_transcript_variant
rs964220306	intron_variant,genic_upstream_transcript_variant
rs964229260	intron_variant
rs964246724	intron_variant,genic_upstream_transcript_variant
rs964266757	intron_variant
rs964291044	intron_variant
rs964307818	intron_variant,genic_upstream_transcript_variant
rs964320180	intron_variant
rs964356368	non_coding_transcript_variant,missense_variant,coding_sequence_variant,intron_variant,genic_upstream_transcript_variant
rs964376121	intron_variant,genic_downstream_transcript_variant
rs964396014	non_coding_transcript_variant,3_prime_UTR_variant,genic_downstream_transcript_variant
rs964410497	intron_variant,genic_upstream_transcript_variant
rs964422803	intron_variant
rs964500408	intron_variant,genic_upstream_transcript_variant
rs964576679	intron_variant
rs964592234	missense_variant,non_coding_transcript_variant,coding_sequence_variant
rs964679589	intron_variant,genic_upstream_transcript_variant
rs964691004	intron_variant,genic_upstream_transcript_variant
rs964721144	intron_variant,genic_upstream_transcript_variant
rs964750194	intron_variant,genic_downstream_transcript_variant
rs964752167	intron_variant,genic_upstream_transcript_variant
rs964780861	intron_variant,genic_downstream_transcript_variant
rs964783870	intron_variant,genic_downstream_transcript_variant
rs964784290	intron_variant,genic_upstream_transcript_variant
rs964829546	intron_variant,genic_upstream_transcript_variant
rs964894130	intron_variant
rs964902579	intron_variant,genic_upstream_transcript_variant
rs964908511	intron_variant,genic_upstream_transcript_variant
rs964911687	intron_variant,genic_upstream_transcript_variant
rs964973693	intron_variant,genic_upstream_transcript_variant
rs964994696	intron_variant,genic_downstream_transcript_variant
rs965026667	non_coding_transcript_variant,3_prime_UTR_variant,genic_downstream_transcript_variant
rs965030027	intron_variant,genic_upstream_transcript_variant
rs965036276	intron_variant,genic_downstream_transcript_variant
rs965052772	intron_variant,genic_upstream_transcript_variant
rs965065925	intron_variant,genic_downstream_transcript_variant
rs965066206	intron_variant
rs965079629	intron_variant,genic_upstream_transcript_variant
rs965087858	intron_variant
rs965123564	intron_variant
rs965198848	intron_variant
rs965206394	intron_variant
rs965212785	intron_variant,genic_upstream_transcript_variant
rs965310303	intron_variant
rs965356945	intron_variant,genic_upstream_transcript_variant
rs965365559	intron_variant,genic_upstream_transcript_variant
rs965377383	intron_variant
rs965428008	intron_variant
rs965450990	intron_variant
rs965455003	intron_variant,genic_upstream_transcript_variant
rs965471260	intron_variant,genic_downstream_transcript_variant
rs965514340	intron_variant,genic_upstream_transcript_variant
rs965519617	intron_variant,genic_upstream_transcript_variant

rs965586185	intron_variant,genic_upstream_transcript_variant
rs965589390	intron_variant,genic_upstream_transcript_variant
rs965620890	intron_variant,downstream_transcript_variant,genic_downstream_transcript_variant
rs965646302	intron_variant,genic_downstream_transcript_variant
rs965677344	intron_variant,genic_downstream_transcript_variant
rs965725998	intron_variant,genic_upstream_transcript_variant
rs965755299	genic_downstream_transcript_variant,intron_variant
rs965776877	genic_upstream_transcript_variant,intron_variant
rs965788932	intron_variant
rs965804758	genic_upstream_transcript_variant,intron_variant
rs965842534	genic_upstream_transcript_variant,intron_variant
rs965865729	genic_downstream_transcript_variant,intron_variant
rs965876208	genic_upstream_transcript_variant,intron_variant
rs965898767	genic_upstream_transcript_variant,intron_variant
rs965907913	genic_upstream_transcript_variant,intron_variant
rs965917698	genic_upstream_transcript_variant,intron_variant
rs965919829	genic_upstream_transcript_variant,intron_variant
rs965935592	genic_upstream_transcript_variant,intron_variant
rs965967242	intron_variant
rs965978359	intron_variant
rs965984717	genic_downstream_transcript_variant,downstream_transcript_variant,intron_variant
rs965992664	intron_variant
rs966090421	genic_upstream_transcript_variant,intron_variant
rs966090566	intron_variant
rs966140372	genic_upstream_transcript_variant,intron_variant
rs966140717	intron_variant
rs966155550	genic_upstream_transcript_variant,intron_variant
rs966170540	genic_downstream_transcript_variant,intron_variant
rs966198893	upstream_transcript_variant,2KB_upstream_variant,genic_upstream_transcript_variant,intron_variant
rs966204599	genic_downstream_transcript_variant,intron_variant
rs966210004	genic_upstream_transcript_variant,intron_variant
rs966216953	intron_variant
rs966228823	genic_upstream_transcript_variant,intron_variant
rs966272521	intron_variant
rs966283224	genic_upstream_transcript_variant,intron_variant
rs966315347	genic_upstream_transcript_variant,intron_variant
rs966328932	intron_variant
rs966336201	genic_downstream_transcript_variant,intron_variant
rs966366668	intron_variant
rs966382443	intron_variant
rs966382822	genic_downstream_transcript_variant,intron_variant
rs966396190	genic_upstream_transcript_variant,intron_variant
rs966413887	genic_downstream_transcript_variant,intron_variant
rs966427299	genic_upstream_transcript_variant,intron_variant
rs966452795	500B_downstream_variant,downstream_transcript_variant
rs966496613	genic_upstream_transcript_variant,intron_variant
rs966533708	genic_downstream_transcript_variant,intron_variant
rs966564308	intron_variant
rs966632916	genic_downstream_transcript_variant,intron_variant
rs966643140	intron_variant
rs966674559	genic_upstream_transcript_variant,intron_variant
rs966687202	genic_downstream_transcript_variant,intron_variant
rs966706550	genic_upstream_transcript_variant,intron_variant
rs966707038	genic_upstream_transcript_variant,intron_variant
rs966717668	genic_downstream_transcript_variant,intron_variant
rs966759299	intron_variant
rs966784756	intron_variant
rs966789607	intron_variant
rs966805394	genic_upstream_transcript_variant,intron_variant
rs966871873	intron_variant
rs966888472	genic_upstream_transcript_variant,intron_variant
rs966889790	genic_upstream_transcript_variant,intron_variant
rs966890564	genic_upstream_transcript_variant,intron_variant
rs966896581	genic_upstream_transcript_variant,intron_variant
rs966927509	intron_variant
rs966944416	genic_upstream_transcript_variant,intron_variant
rs967049060	intron_variant
rs967060702	intron_variant
rs967066235	genic_upstream_transcript_variant,intron_variant
rs967069466	intron_variant
rs967078214	genic_upstream_transcript_variant,intron_variant
rs967125763	genic_upstream_transcript_variant,intron_variant
rs967134117	intron_variant
rs967163037	intron_variant

rs967179088	intron_variant
rs967204791	intron_variant
rs967224872	genic_upstream_transcript_variant,intron_variant
rs967234663	genic_upstream_transcript_variant,intron_variant
rs967242812	genic_upstream_transcript_variant,intron_variant
rs967243840	upstream_transcript_variant,2KB_upstream_variant,genic_upstream_transcript_variant,intron_variant
rs967276114	genic_upstream_transcript_variant,intron_variant
rs967283463	intron_variant
rs967319288	genic_upstream_transcript_variant,intron_variant
rs967341433	genic_upstream_transcript_variant,intron_variant
rs967357267	genic_upstream_transcript_variant,intron_variant
rs967360574	genic_downstream_transcript_variant,intron_variant
rs967384716	genic_upstream_transcript_variant,intron_variant
rs967427260	genic_upstream_transcript_variant,intron_variant
rs967455974	genic_downstream_transcript_variant,intron_variant
rs967479578	genic_upstream_transcript_variant,intron_variant
rs967482888	genic_downstream_transcript_variant,intron_variant
rs967520595	upstream_transcript_variant,2KB_upstream_variant,genic_upstream_transcript_variant,intron_variant
rs967526627	genic_upstream_transcript_variant,intron_variant
rs967602884	genic_downstream_transcript_variant,intron_variant
rs967642703	intron_variant
rs967659230	intron_variant
rs967690264	genic_downstream_transcript_variant,intron_variant
rs967693286	genic_upstream_transcript_variant,intron_variant
rs967712593	genic_upstream_transcript_variant,intron_variant
rs967728187	genic_downstream_transcript_variant,intron_variant
rs967738968	genic_upstream_transcript_variant,intron_variant
rs967762924	genic_downstream_transcript_variant,intron_variant
rs967775142	genic_downstream_transcript_variant,intron_variant
rs967810838	genic_upstream_transcript_variant,intron_variant
rs967835858	intron_variant
rs967845001	genic_upstream_transcript_variant,intron_variant
rs967859019	intron_variant
rs967895310	intron_variant
rs967896963	genic_downstream_transcript_variant,intron_variant
rs967922773	intron_variant
rs967934975	intron_variant
rs967945824	genic_upstream_transcript_variant,intron_variant
rs967968395	genic_upstream_transcript_variant,intron_variant
rs968028473	genic_upstream_transcript_variant,intron_variant
rs968034125	intron_variant
rs968039946	intron_variant
rs968072005	genic_upstream_transcript_variant,intron_variant
rs968079371	intron_variant
rs968098622	genic_upstream_transcript_variant,intron_variant
rs968122219	genic_downstream_transcript_variant,intron_variant
rs968142708	intron_variant
rs968168784	genic_upstream_transcript_variant,intron_variant
rs968177856	intron_variant
rs968187976	genic_upstream_transcript_variant,intron_variant
rs968292360	intron_variant
rs968340669	genic_upstream_transcript_variant,intron_variant
rs968342603	genic_downstream_transcript_variant,intron_variant
rs968364867	genic_downstream_transcript_variant,intron_variant
rs968368048	intron_variant
rs968375515	genic_downstream_transcript_variant,intron_variant
rs968389438	genic_downstream_transcript_variant,intron_variant
rs968414429	genic_downstream_transcript_variant,intron_variant
rs968478091	genic_upstream_transcript_variant,intron_variant
rs968508484	intron_variant
rs968512516	intron_variant
rs968551537	intron_variant
rs968580306	intron_variant
rs968626758	intron_variant
rs968631069	genic_upstream_transcript_variant,intron_variant
rs968636277	genic_downstream_transcript_variant,intron_variant
rs968669313	genic_upstream_transcript_variant,2KB_upstream_variant,upstream_transcript_variant,intron_variant
rs968716357	intron_variant
rs968727568	genic_upstream_transcript_variant,non_coding_transcript_variant,5_prime_UTR_variant,intron_variant
rs968736147	intron_variant
rs968750041	genic_upstream_transcript_variant,intron_variant
rs968813177	genic_upstream_transcript_variant,intron_variant
rs968878413	genic_upstream_transcript_variant,intron_variant
rs968899321	upstream_transcript_variant,genic_upstream_transcript_variant,intron_variant

rs968901060	upstream_transcript_variant,genic_upstream_transcript_variant,intron_variant
rs968958135	genic_upstream_transcript_variant,upstream_transcript_variant,5_prime_UTR_variant,intron_variant
rs969022420	genic_downstream_transcript_variant,intron_variant
rs969024933	upstream_transcript_variant,genic_upstream_transcript_variant,intron_variant
rs969032915	genic_upstream_transcript_variant,intron_variant
rs969034216	genic_upstream_transcript_variant,intron_variant
rs969066386	genic_upstream_transcript_variant,intron_variant
rs969085066	genic_upstream_transcript_variant,intron_variant
rs969175681	intron_variant
rs969183806	intron_variant
rs969188845	intron_variant
rs969218735	intron_variant
rs969235431	genic_downstream_transcript_variant,intron_variant
rs969253537	genic_upstream_transcript_variant,intron_variant
rs969294088	intron_variant
rs969334735	genic_upstream_transcript_variant,intron_variant
rs969365355	genic_downstream_transcript_variant,intron_variant
rs969394405	genic_upstream_transcript_variant,intron_variant
rs969405192	intron_variant
rs969409897	genic_upstream_transcript_variant,intron_variant
rs969417141	genic_downstream_transcript_variant,intron_variant
rs969419688	upstream_transcript_variant,genic_upstream_transcript_variant,intron_variant
rs969430279	genic_upstream_transcript_variant,intron_variant
rs969445321	intron_variant
rs969455022	intron_variant
rs969496997	intron_variant
rs969509895	genic_upstream_transcript_variant,intron_variant
rs969532034	genic_upstream_transcript_variant,2KB_upstream_variant,upstream_transcript_variant,intron_variant
rs969543202	genic_upstream_transcript_variant,intron_variant
rs969557020	genic_upstream_transcript_variant,intron_variant
rs969582246	genic_upstream_transcript_variant,intron_variant
rs969621405	genic_upstream_transcript_variant,intron_variant
rs969637390	genic_upstream_transcript_variant,intron_variant
rs969646436	genic_downstream_transcript_variant,intron_variant
rs969646477	genic_downstream_transcript_variant,intron_variant
rs969685999	intron_variant
rs969769228	genic_upstream_transcript_variant,intron_variant
rs969793803	genic_upstream_transcript_variant,intron_variant
rs969864705	intron_variant
rs969865175	intron_variant
rs969872681	genic_upstream_transcript_variant,intron_variant
rs969909698	genic_downstream_transcript_variant,intron_variant
rs969938880	intron_variant
rs969956432	genic_downstream_transcript_variant,intron_variant
rs969972446	genic_upstream_transcript_variant,intron_variant
rs969980154	genic_downstream_transcript_variant,intron_variant
rs969989466	intron_variant
rs970042969	genic_upstream_transcript_variant,intron_variant
rs970101097	intron_variant
rs970101127	intron_variant
rs970153847	intron_variant
rs970200705	genic_upstream_transcript_variant,intron_variant
rs970206545	genic_downstream_transcript_variant,non_coding_transcript_variant,missense_variant,coding_sequence_variant
rs970229176	intron_variant
rs970241068	genic_upstream_transcript_variant,intron_variant
rs970277664	genic_upstream_transcript_variant,intron_variant
rs970279084	intron_variant
rs970280415	genic_downstream_transcript_variant,intron_variant
rs970310147	genic_downstream_transcript_variant,intron_variant
rs970337794	genic_upstream_transcript_variant,intron_variant
rs970338725	genic_upstream_transcript_variant,intron_variant
rs970371015	intron_variant
rs970402078	genic_upstream_transcript_variant,2KB_upstream_variant,upstream_transcript_variant,intron_variant
rs970512328	intron_variant
rs970607296	genic_downstream_transcript_variant,intron_variant
rs970615851	intron_variant
rs970621616	genic_downstream_transcript_variant,intron_variant
rs970621858	genic_upstream_transcript_variant,intron_variant
rs970643807	genic_upstream_transcript_variant,intron_variant
rs970652902	genic_downstream_transcript_variant,intron_variant
rs970663779	genic_upstream_transcript_variant,intron_variant
rs970686059	genic_downstream_transcript_variant,intron_variant
rs970708679	genic_upstream_transcript_variant,intron_variant
rs970711078	genic_upstream_transcript_variant,intron_variant

rs970759579	genic_downstream_transcript_variant,intron_variant
rs970767995	intron_variant
rs970815265	intron_variant
rs970860543	intron_variant
rs970867744	genic_upstream_transcript_variant,intron_variant
rs970873399	genic_downstream_transcript_variant,intron_variant
rs970898566	genic_upstream_transcript_variant,intron_variant
rs970953696	intron_variant
rs970985685	genic_downstream_transcript_variant,intron_variant
rs970989724	genic_upstream_transcript_variant,intron_variant
rs970990427	genic_downstream_transcript_variant,intron_variant
rs970991062	genic_upstream_transcript_variant,intron_variant
rs971044366	intron_variant
rs971046044	genic_upstream_transcript_variant,intron_variant
rs971067673	intron_variant
rs971163612	intron_variant
rs971173000	genic_downstream_transcript_variant,intron_variant
rs971197453	intron_variant
rs971266153	genic_downstream_transcript_variant,non_coding_transcript_variant,3_prime_UTR_variant
rs971273713	genic_upstream_transcript_variant,intron_variant
rs971276001	genic_upstream_transcript_variant,intron_variant
rs971286467	500B_downstream_variant,downstream_transcript_variant
rs971304753	genic_upstream_transcript_variant,intron_variant
rs971323607	intron_variant
rs971356949	genic_downstream_transcript_variant,intron_variant
rs971362676	coding_sequence_variant,non_coding_transcript_variant,genic_upstream_transcript_variant,missense_variant,intron_variant
rs971376334	intron_variant
rs971424003	intron_variant
rs971438384	intron_variant
rs971457482	intron_variant
rs971462607	genic_downstream_transcript_variant,intron_variant
rs971462742	intron_variant
rs971530860	intron_variant
rs971563069	non_coding_transcript_variant,missense_variant,coding_sequence_variant
rs971585559	genic_upstream_transcript_variant,intron_variant
rs971645196	genic_upstream_transcript_variant,intron_variant
rs971658126	genic_downstream_transcript_variant,intron_variant
rs971684124	genic_upstream_transcript_variant,intron_variant
rs971749562	genic_upstream_transcript_variant,intron_variant
rs971759152	intron_variant
rs971766951	genic_upstream_transcript_variant,intron_variant
rs971806579	genic_upstream_transcript_variant,intron_variant
rs971853548	genic_upstream_transcript_variant,intron_variant
rs971865008	genic_upstream_transcript_variant,intron_variant
rs971875449	genic_downstream_transcript_variant,downstream_transcript_variant,intron_variant
rs971896496	genic_downstream_transcript_variant,intron_variant
rs971922030	genic_upstream_transcript_variant,intron_variant
rs971951665	genic_upstream_transcript_variant,intron_variant
rs971955089	genic_upstream_transcript_variant,intron_variant
rs976726671	genic_upstream_transcript_variant,2KB_upstream_variant,upstream_transcript_variant,intron_variant
rs976784816	5_prime_UTR_variant,intron_variant
rs976846972	intron_variant
rs976867068	genic_downstream_transcript_variant,intron_variant
rs976895506	intron_variant
rs976903215	intron_variant
rs976929352	genic_upstream_transcript_variant,intron_variant
rs976957397	intron_variant
rs976963520	genic_downstream_transcript_variant,intron_variant
rs976994252	intron_variant
rs977000663	intron_variant
rs977031248	intron_variant
rs977031550	genic_upstream_transcript_variant,2KB_upstream_variant,upstream_transcript_variant,intron_variant
rs977048779	genic_downstream_transcript_variant,intron_variant
rs977054798	upstream_transcript_variant,2KB_upstream_variant,genic_upstream_transcript_variant,intron_variant
rs977060393	genic_downstream_transcript_variant,downstream_transcript_variant,intron_variant
rs977075258	genic_upstream_transcript_variant,intron_variant
rs977080204	intron_variant
rs977129833	genic_upstream_transcript_variant,intron_variant
rs977138902	genic_downstream_transcript_variant,intron_variant
rs977151376	genic_upstream_transcript_variant,intron_variant
rs977214022	genic_upstream_transcript_variant,intron_variant
rs977239438	genic_downstream_transcript_variant,intron_variant
rs977246967	intron_variant
rs977247684	genic_upstream_transcript_variant,intron_variant

rs977252320	intron_variant
rs977274965	genic_upstream_transcript_variant,intron_variant
rs977275643	upstream_transcript_variant,2KB_upstream_variant,genic_upstream_transcript_variant,intron_variant
rs977279381	genic_upstream_transcript_variant,intron_variant
rs977293915	genic_upstream_transcript_variant,intron_variant
rs977300734	genic_downstream_transcript_variant,downstream_transcript_variant,intron_variant
rs977323028	intron_variant
rs977335240	genic_upstream_transcript_variant,intron_variant
rs977360679	genic_upstream_transcript_variant,intron_variant
rs977416323	intron_variant
rs977476360	intron_variant
rs977485458	genic_downstream_transcript_variant,intron_variant
rs977507141	intron_variant
rs977569495	intron_variant
rs977574164	genic_upstream_transcript_variant,intron_variant
rs977616151	genic_downstream_transcript_variant,intron_variant
rs977639063	genic_downstream_transcript_variant,intron_variant
rs977648531	genic_downstream_transcript_variant,intron_variant
rs977659511	genic_upstream_transcript_variant,intron_variant
rs977664470	intron_variant
rs977671689	genic_downstream_transcript_variant,intron_variant
rs977678396	intron_variant
rs977760267	intron_variant
rs977761497	genic_upstream_transcript_variant,intron_variant
rs977815199	intron_variant
rs977850836	intron_variant
rs977889410	genic_upstream_transcript_variant,intron_variant
rs977892325	genic_downstream_transcript_variant,intron_variant
rs977909337	genic_upstream_transcript_variant,intron_variant
rs977928622	genic_upstream_transcript_variant,intron_variant
rs977934257	intron_variant
rs978091867	intron_variant
rs978097598	genic_downstream_transcript_variant,intron_variant
rs978214925	genic_upstream_transcript_variant,intron_variant
rs978233070	intron_variant
rs978305178	genic_downstream_transcript_variant,intron_variant
rs978328375	genic_upstream_transcript_variant,intron_variant
rs978351908	intron_variant
rs978356552	intron_variant
rs978364235	genic_upstream_transcript_variant,intron_variant
rs978365089	intron_variant
rs978388325	intron_variant
rs978393558	genic_upstream_transcript_variant,intron_variant
rs978415600	intron_variant
rs978454207	genic_upstream_transcript_variant,intron_variant
rs978477015	intron_variant
rs978506758	genic_upstream_transcript_variant,upstream_transcript_variant,intron_variant
rs978524232	genic_upstream_transcript_variant,intron_variant
rs978565323	intron_variant
rs978581375	genic_upstream_transcript_variant,intron_variant
rs978585660	genic_upstream_transcript_variant,intron_variant
rs978604201	genic_upstream_transcript_variant,intron_variant
rs978606456	intron_variant
rs978615276	genic_upstream_transcript_variant,intron_variant
rs978661931	intron_variant
rs978696269	genic_upstream_transcript_variant,intron_variant
rs978720790	intron_variant
rs978768802	intron_variant
rs978802718	intron_variant
rs978843023	intron_variant
rs978853765	intron_variant
rs978921032	intron_variant
rs978951351	genic_upstream_transcript_variant,upstream_transcript_variant,5_prime_UTR_variant,intron_variant
rs979008288	genic_upstream_transcript_variant,intron_variant
rs979016496	intron_variant
rs979023618	intron_variant
rs979116240	genic_upstream_transcript_variant,intron_variant
rs979130768	genic_downstream_transcript_variant,intron_variant
rs979143316	genic_upstream_transcript_variant,intron_variant
rs979154714	genic_upstream_transcript_variant,intron_variant
rs979180275	intron_variant
rs979184482	intron_variant
rs979209403	genic_upstream_transcript_variant,intron_variant
rs979237333	genic_upstream_transcript_variant,intron_variant

rs979276486	genic_upstream_transcript_variant,intron_variant
rs979278536	genic_upstream_transcript_variant,intron_variant
rs979297260	genic_upstream_transcript_variant,intron_variant
rs979310895	intron_variant
rs979315998	genic_upstream_transcript_variant,intron_variant
rs979325888	genic_upstream_transcript_variant,intron_variant
rs979363298	intron_variant
rs979387297	intron_variant
rs979399728	intron_variant
rs979477645	genic_downstream_transcript_variant,intron_variant
rs979575234	genic_downstream_transcript_variant,intron_variant
rs979576435	genic_upstream_transcript_variant,intron_variant
rs979621541	intron_variant
rs979650542	genic_upstream_transcript_variant,intron_variant
rs979665140	intron_variant
rs979729056	genic_upstream_transcript_variant,upstream_transcript_variant,intron_variant
rs979740993	intron_variant
rs979776687	genic_upstream_transcript_variant,intron_variant
rs979784314	5_prime_UTR_variant,intron_variant
rs979785375	genic_upstream_transcript_variant,intron_variant
rs979804757	genic_upstream_transcript_variant,intron_variant
rs979808981	genic_upstream_transcript_variant,non_coding_transcript_variant,5_prime_UTR_variant,intron_variant
rs979809621	genic_upstream_transcript_variant,intron_variant
rs979851863	intron_variant
rs979858317	intron_variant
rs979863389	genic_downstream_transcript_variant,intron_variant
rs979897217	genic_upstream_transcript_variant,intron_variant
rs979905219	genic_downstream_transcript_variant,intron_variant
rs979932580	intron_variant
rs979939236	intron_variant
rs979957551	genic_downstream_transcript_variant,intron_variant
rs979969441	genic_upstream_transcript_variant,intron_variant
rs979973046	intron_variant
rs979998267	genic_downstream_transcript_variant,intron_variant
rs980048758	genic_downstream_transcript_variant,intron_variant
rs980049383	genic_upstream_transcript_variant,intron_variant
rs980057274	genic_upstream_transcript_variant,intron_variant
rs980072343	genic_downstream_transcript_variant,intron_variant
rs980078114	intron_variant
rs980129692	intron_variant
rs980154011	intron_variant
rs980201638	intron_variant
rs980202629	intron_variant
rs980206044	intron_variant
rs980208831	genic_upstream_transcript_variant,intron_variant
rs980232820	genic_upstream_transcript_variant,intron_variant
rs980258164	genic_upstream_transcript_variant,intron_variant
rs980266955	3_prime_UTR_variant,non_coding_transcript_variant,genic_downstream_transcript_variant
rs980284598	genic_upstream_transcript_variant,intron_variant
rs980297714	genic_upstream_transcript_variant,intron_variant
rs980305036	genic_upstream_transcript_variant,intron_variant
rs980356821	intron_variant
rs980387264	genic_upstream_transcript_variant,intron_variant
rs980409941	genic_upstream_transcript_variant,upstream_transcript_variant,intron_variant
rs980431892	intron_variant
rs980479988	intron_variant
rs980556323	genic_upstream_transcript_variant,intron_variant
rs980602576	genic_upstream_transcript_variant,intron_variant
rs980677568	genic_upstream_transcript_variant,intron_variant
rs980734134	intron_variant
rs980743994	intron_variant
rs980783723	genic_downstream_transcript_variant,intron_variant
rs980801337	genic_upstream_transcript_variant,intron_variant
rs980816174	intron_variant
rs980822794	genic_upstream_transcript_variant,intron_variant
rs980895613	genic_upstream_transcript_variant,intron_variant
rs980907795	genic_downstream_transcript_variant,intron_variant
rs980915605	intron_variant
rs980938493	genic_downstream_transcript_variant,intron_variant
rs980940721	3_prime_UTR_variant,non_coding_transcript_variant,genic_downstream_transcript_variant
rs980945881	genic_upstream_transcript_variant,intron_variant
rs980976906	intron_variant
rs981007641	intron_variant
rs981038325	genic_upstream_transcript_variant,intron_variant

rs981040661	intron_variant
rs981075382	intron_variant
rs981081156	intron_variant
rs981147597	intron_variant
rs981149783	genic_upstream_transcript_variant,2KB_upstream_variant,upstream_transcript_variant,intron_variant
rs981209679	intron_variant
rs981214348	genic_upstream_transcript_variant,intron_variant
rs981214510	genic_upstream_transcript_variant,intron_variant
rs981224871	genic_upstream_transcript_variant,2KB_upstream_variant,upstream_transcript_variant,intron_variant
rs981251985	genic_downstream_transcript_variant,intron_variant
rs981254877	intron_variant
rs981283339	genic_upstream_transcript_variant,intron_variant
rs981303623	genic_downstream_transcript_variant,intron_variant
rs981319605	genic_downstream_transcript_variant,intron_variant
rs981395599	genic_upstream_transcript_variant,intron_variant
rs981397946	intron_variant
rs981401332	coding_sequence_variant,non_coding_transcript_variant,missense_variant
rs981408928	intron_variant
rs981450401	genic_upstream_transcript_variant,intron_variant
rs981460007	genic_downstream_transcript_variant,intron_variant
rs981461485	intron_variant
rs981487430	genic_upstream_transcript_variant,intron_variant
rs981488639	genic_downstream_transcript_variant,intron_variant
rs981496176	genic_upstream_transcript_variant,intron_variant
rs981550480	genic_upstream_transcript_variant,intron_variant
rs981563889	genic_downstream_transcript_variant,intron_variant
rs981566883	genic_upstream_transcript_variant,intron_variant
rs981592293	genic_downstream_transcript_variant,intron_variant
rs981595494	genic_downstream_transcript_variant,intron_variant
rs981599530	genic_upstream_transcript_variant,intron_variant
rs981623239	genic_downstream_transcript_variant,intron_variant
rs981653351	intron_variant
rs981664259	genic_downstream_transcript_variant,intron_variant
rs981678972	intron_variant
rs981697277	intron_variant
rs981732952	intron_variant
rs981743946	intron_variant
rs981753425	intron_variant
rs981785248	intron_variant
rs981798654	genic_upstream_transcript_variant,2KB_upstream_variant,upstream_transcript_variant,intron_variant
rs981808954	genic_upstream_transcript_variant,intron_variant
rs981866256	genic_downstream_transcript_variant,intron_variant
rs981871464	genic_upstream_transcript_variant,splice_donor_variant,intron_variant
rs981893345	genic_upstream_transcript_variant,intron_variant
rs981897939	genic_upstream_transcript_variant,intron_variant
rs981914762	genic_downstream_transcript_variant,intron_variant
rs981921630	genic_downstream_transcript_variant,intron_variant
rs981945812	genic_downstream_transcript_variant,intron_variant
rs981984090	intron_variant
rs981988350	genic_downstream_transcript_variant,intron_variant
rs982050267	genic_upstream_transcript_variant,intron_variant
rs982062864	genic_upstream_transcript_variant,intron_variant
rs982153478	intron_variant
rs982156423	genic_upstream_transcript_variant,intron_variant
rs982188188	genic_upstream_transcript_variant,intron_variant
rs982205700	intron_variant
rs982215091	genic_upstream_transcript_variant,2KB_upstream_variant,upstream_transcript_variant,intron_variant
rs982228909	3_prime_UTR_variant,non_coding_transcript_variant,genic_downstream_transcript_variant
rs982241089	genic_downstream_transcript_variant,intron_variant
rs982243002	genic_downstream_transcript_variant,intron_variant
rs982293372	genic_downstream_transcript_variant,intron_variant
rs982302923	genic_upstream_transcript_variant,intron_variant
rs982359583	intron_variant
rs982365238	intron_variant
rs982429223	genic_downstream_transcript_variant,intron_variant
rs982476629	genic_upstream_transcript_variant,intron_variant
rs982504312	genic_upstream_transcript_variant,intron_variant
rs982535102	intron_variant
rs982542585	genic_upstream_transcript_variant,intron_variant
rs982546066	genic_upstream_transcript_variant,intron_variant
rs982591873	genic_downstream_transcript_variant,intron_variant
rs982657623	genic_downstream_transcript_variant,intron_variant
rs982676702	genic_upstream_transcript_variant,intron_variant
rs982677865	genic_upstream_transcript_variant,intron_variant

rs982688862	genic_downstream_transcript_variant,intron_variant
rs982706112	genic_upstream_transcript_variant,intron_variant
rs982738721	genic_upstream_transcript_variant,intron_variant
rs982758836	genic_downstream_transcript_variant,intron_variant
rs982771186	genic_upstream_transcript_variant,intron_variant
rs982785757	genic_upstream_transcript_variant,intron_variant
rs982785999	genic_upstream_transcript_variant,intron_variant
rs982814384	intron_variant
rs982856694	genic_upstream_transcript_variant,intron_variant
rs982868433	intron_variant
rs982904721	genic_downstream_transcript_variant,intron_variant
rs982917490	genic_upstream_transcript_variant,intron_variant
rs982943219	genic_upstream_transcript_variant,intron_variant
rs982956990	intron_variant
rs982984020	intron_variant
rs983029555	genic_downstream_transcript_variant,downstream_transcript_variant,intron_variant
rs983062105	coding_sequence_variant,non_coding_transcript_variant,missense_variant
rs983092606	genic_downstream_transcript_variant,intron_variant
rs983104186	intron_variant
rs983108059	genic_upstream_transcript_variant,intron_variant
rs983119377	genic_upstream_transcript_variant,intron_variant
rs983136026	genic_upstream_transcript_variant,intron_variant
rs983187368	intron_variant
rs983204340	intron_variant
rs983205344	intron_variant
rs983230288	genic_upstream_transcript_variant,intron_variant
rs983266661	intron_variant
rs983346380	genic_downstream_transcript_variant,coding_sequence_variant,synonymous_variant,non_coding_transcript_variant
rs983365262	genic_downstream_transcript_variant,intron_variant
rs983367297	genic_upstream_transcript_variant,intron_variant
rs983367625	intron_variant
rs983375839	genic_downstream_transcript_variant,intron_variant
rs983389492	genic_upstream_transcript_variant,intron_variant
rs983393795	intron_variant
rs983442351	genic_upstream_transcript_variant,upstream_transcript_variant,intron_variant
rs983456405	intron_variant
rs983485541	500B_downstream_variant,downstream_transcript_variant
rs983502490	genic_upstream_transcript_variant,intron_variant
rs983509193	intron_variant
rs983511699	intron_variant
rs983539400	genic_downstream_transcript_variant,intron_variant
rs983548802	intron_variant
rs983556684	genic_upstream_transcript_variant,intron_variant
rs983580721	genic_upstream_transcript_variant,intron_variant
rs983615156	intron_variant
rs983622411	intron_variant
rs983626668	intron_variant
rs983632505	genic_downstream_transcript_variant,intron_variant
rs983633422	genic_downstream_transcript_variant,intron_variant
rs983670669	genic_upstream_transcript_variant,intron_variant
rs983672340	genic_upstream_transcript_variant,intron_variant
rs983704657	intron_variant
rs983740463	genic_upstream_transcript_variant,intron_variant
rs983766848	genic_upstream_transcript_variant,intron_variant
rs983771549	genic_downstream_transcript_variant,intron_variant
rs983790241	intron_variant
rs983836319	intron_variant
rs983854121	genic_upstream_transcript_variant,intron_variant
rs983859953	intron_variant
rs983882657	intron_variant
rs983888612	intron_variant
rs983894994	genic_upstream_transcript_variant,intron_variant
rs983907199	genic_upstream_transcript_variant,intron_variant
rs983910802	genic_upstream_transcript_variant,intron_variant
rs983920878	genic_upstream_transcript_variant,intron_variant
rs983944622	genic_upstream_transcript_variant,2KB_upstream_variant,upstream_transcript_variant,intron_variant
rs983954517	intron_variant
rs983979332	genic_upstream_transcript_variant,intron_variant
rs984043025	genic_upstream_transcript_variant,intron_variant
rs984051672	genic_downstream_transcript_variant,intron_variant
rs984060325	genic_downstream_transcript_variant,intron_variant
rs984093176	intron_variant
rs984127679	intron_variant
rs984138307	intron_variant

rs984156001	intron_variant
rs984156651	500B_downstream_variant,downstream_transcript_variant
rs984181799	intron_variant
rs984187930	genic_downstream_transcript_variant,intron_variant
rs984207832	500B_downstream_variant,downstream_transcript_variant
rs984209707	intron_variant
rs984233093	intron_variant
rs984296386	genic_downstream_transcript_variant,intron_variant
rs984296847	genic_upstream_transcript_variant,intron_variant
rs984297769	intron_variant
rs984303861	genic_downstream_transcript_variant,intron_variant
rs984310200	genic_downstream_transcript_variant,intron_variant
rs984310666	genic_upstream_transcript_variant,intron_variant
rs984324874	intron_variant
rs984387603	genic_upstream_transcript_variant,2KB_upstream_variant,upstream_transcript_variant,intron_variant
rs984461811	genic_upstream_transcript_variant,intron_variant
rs984491304	intron_variant
rs984512189	intron_variant
rs984513517	intron_variant
rs984538353	genic_upstream_transcript_variant,intron_variant
rs984544465	3_prime_UTR_variant,non_coding_transcript_variant,genic_downstream_transcript_variant
rs984568706	intron_variant
rs984588935	genic_downstream_transcript_variant,intron_variant
rs984662707	genic_downstream_transcript_variant,intron_variant
rs984717910	genic_downstream_transcript_variant,intron_variant
rs984720689	genic_upstream_transcript_variant,intron_variant
rs984732913	intron_variant
rs984751094	intron_variant
rs984766441	genic_upstream_transcript_variant,intron_variant
rs984805752	genic_upstream_transcript_variant,upstream_transcript_variant,intron_variant
rs984807765	intron_variant
rs984825765	genic_upstream_transcript_variant,2KB_upstream_variant,upstream_transcript_variant,intron_variant
rs984829473	genic_upstream_transcript_variant,2KB_upstream_variant,upstream_transcript_variant,intron_variant
rs984833025	genic_upstream_transcript_variant,2KB_upstream_variant,upstream_transcript_variant,intron_variant
rs984850525	intron_variant
rs984878998	genic_upstream_transcript_variant,intron_variant
rs984908068	intron_variant
rs984933304	genic_upstream_transcript_variant,intron_variant
rs984944560	genic_downstream_transcript_variant,intron_variant
rs984959449	intron_variant
rs984974349	genic_upstream_transcript_variant,intron_variant
rs985011629	genic_downstream_transcript_variant,intron_variant
rs985020066	genic_downstream_transcript_variant,intron_variant
rs985042800	genic_downstream_transcript_variant,intron_variant
rs985063541	genic_downstream_transcript_variant,intron_variant
rs985064204	genic_upstream_transcript_variant,intron_variant
rs985111649	intron_variant
rs985157132	intron_variant
rs985164120	intron_variant
rs985169263	genic_upstream_transcript_variant,intron_variant
rs985193426	genic_upstream_transcript_variant,intron_variant
rs985225244	intron_variant
rs985277397	genic_upstream_transcript_variant,intron_variant
rs985278429	genic_upstream_transcript_variant,2KB_upstream_variant,upstream_transcript_variant,intron_variant
rs985327431	intron_variant
rs985355743	intron_variant
rs985359405	genic_upstream_transcript_variant,intron_variant
rs985363632	intron_variant
rs985370405	genic_upstream_transcript_variant,2KB_upstream_variant,upstream_transcript_variant,intron_variant
rs985387018	intron_variant
rs985402205	genic_upstream_transcript_variant,intron_variant
rs985429214	genic_upstream_transcript_variant,intron_variant
rs985467106	genic_upstream_transcript_variant,intron_variant
rs985480005	genic_downstream_transcript_variant,intron_variant
rs985584769	genic_upstream_transcript_variant,intron_variant
rs985598257	intron_variant
rs985617469	intron_variant
rs985627295	intron_variant
rs985628201	intron_variant
rs985632681	genic_downstream_transcript_variant,intron_variant
rs985647164	genic_downstream_transcript_variant,intron_variant
rs985714336	intron_variant
rs985720581	genic_upstream_transcript_variant,intron_variant
rs985739376	intron_variant

rs985758632	genic_upstream_transcript_variant,2KB_upstream_variant,upstream_transcript_variant,intron_variant
rs985778943	genic_upstream_transcript_variant,intron_variant
rs985823701	genic_downstream_transcript_variant,intron_variant
rs985847645	genic_downstream_transcript_variant,intron_variant
rs985880501	intron_variant
rs985896772	intron_variant
rs985915572	genic_upstream_transcript_variant,intron_variant
rs985966056	intron_variant
rs985979245	genic_downstream_transcript_variant,intron_variant
rs986021572	genic_upstream_transcript_variant,intron_variant
rs986041806	genic_upstream_transcript_variant,intron_variant
rs986046831	intron_variant
rs986049671	genic_downstream_transcript_variant,intron_variant
rs986054862	genic_upstream_transcript_variant,intron_variant
rs986087961	genic_upstream_transcript_variant,2KB_upstream_variant,upstream_transcript_variant,intron_variant
rs986090866	intron_variant
rs986091685	intron_variant
rs986099847	genic_downstream_transcript_variant,intron_variant
rs986113526	genic_upstream_transcript_variant,2KB_upstream_variant,upstream_transcript_variant,intron_variant
rs986120623	genic_upstream_transcript_variant,upstream_transcript_variant,5_prime_UTR_variant,intron_variant
rs986147447	genic_upstream_transcript_variant,intron_variant
rs986158033	genic_upstream_transcript_variant,intron_variant
rs986228826	genic_upstream_transcript_variant,upstream_transcript_variant,intron_variant
rs986248335	genic_upstream_transcript_variant,intron_variant
rs986281116	genic_upstream_transcript_variant,upstream_transcript_variant,intron_variant
rs986303962	intron_variant
rs986353414	genic_upstream_transcript_variant,intron_variant
rs986356422	intron_variant
rs986395349	genic_upstream_transcript_variant,2KB_upstream_variant,upstream_transcript_variant,intron_variant
rs986414591	genic_downstream_transcript_variant,intron_variant
rs986461754	genic_downstream_transcript_variant,intron_variant
rs986466722	genic_upstream_transcript_variant,intron_variant
rs986481251	intron_variant
rs986494432	intron_variant
rs986499245	genic_upstream_transcript_variant,intron_variant
rs986517162	intron_variant
rs986599862	genic_upstream_transcript_variant,intron_variant
rs986623896	intron_variant
rs986667524	genic_downstream_transcript_variant,intron_variant
rs986687913	genic_upstream_transcript_variant,intron_variant
rs986692396	intron_variant
rs986692608	genic_upstream_transcript_variant,intron_variant
rs986724774	genic_upstream_transcript_variant,intron_variant
rs986745488	intron_variant
rs986758299	intron_variant
rs986800524	intron_variant
rs986821204	genic_upstream_transcript_variant,intron_variant
rs986828891	genic_downstream_transcript_variant,intron_variant
rs986864727	genic_upstream_transcript_variant,intron_variant
rs986898199	genic_upstream_transcript_variant,intron_variant
rs986914324	intron_variant
rs986976255	genic_upstream_transcript_variant,intron_variant
rs987020406	intron_variant
rs987027105	genic_upstream_transcript_variant,intron_variant
rs987027755	intron_variant
rs987069860	genic_upstream_transcript_variant,intron_variant
rs987113308	3_prime_UTR_variant,non_coding_transcript_variant,genic_downstream_transcript_variant
rs987144308	3_prime_UTR_variant,non_coding_transcript_variant,genic_downstream_transcript_variant
rs987157549	genic_upstream_transcript_variant,intron_variant
rs987181894	intron_variant
rs987184423	genic_downstream_transcript_variant,intron_variant
rs987225984	genic_upstream_transcript_variant,intron_variant
rs987231220	genic_upstream_transcript_variant,intron_variant
rs987266854	genic_downstream_transcript_variant,intron_variant
rs987275827	genic_upstream_transcript_variant,intron_variant
rs987305948	genic_upstream_transcript_variant,intron_variant
rs987361341	intron_variant
rs987418073	genic_downstream_transcript_variant,intron_variant
rs987437769	genic_downstream_transcript_variant,intron_variant
rs987466238	genic_upstream_transcript_variant,intron_variant
rs987496487	intron_variant
rs987506288	intron_variant
rs987508441	intron_variant
rs987548752	intron_variant

rs987553968	genic_downstream_transcript_variant,intron_variant
rs987611282	genic_downstream_transcript_variant,intron_variant
rs987647463	intron_variant
rs987656402	genic_downstream_transcript_variant,intron_variant
rs987718318	genic_upstream_transcript_variant,intron_variant
rs987720028	genic_downstream_transcript_variant,intron_variant
rs987720467	intron_variant
rs987726650	genic_upstream_transcript_variant,2KB_upstream_variant,upstream_transcript_variant,intron_variant
rs987730503	genic_upstream_transcript_variant,intron_variant
rs987747265	genic_upstream_transcript_variant,intron_variant
rs987752856	genic_upstream_transcript_variant,intron_variant
rs987773786	intron_variant
rs987833441	genic_upstream_transcript_variant,intron_variant
rs987843104	intron_variant
rs987862665	genic_upstream_transcript_variant,2KB_upstream_variant,upstream_transcript_variant,intron_variant
rs987889236	intron_variant
rs987939302	intron_variant
rs987939626	genic_upstream_transcript_variant,intron_variant
rs987977911	genic_downstream_transcript_variant,intron_variant
rs987991819	genic_upstream_transcript_variant,intron_variant
rs988011806	genic_upstream_transcript_variant,intron_variant
rs988029193	genic_upstream_transcript_variant,intron_variant
rs988051715	genic_downstream_transcript_variant,intron_variant
rs988080681	genic_upstream_transcript_variant,intron_variant
rs988091939	intron_variant
rs988163911	genic_downstream_transcript_variant,intron_variant
rs988183383	genic_upstream_transcript_variant,intron_variant
rs988206652	intron_variant
rs988237974	genic_downstream_transcript_variant,intron_variant
rs988296665	genic_upstream_transcript_variant,intron_variant
rs988326618	intron_variant
rs988327106	intron_variant
rs988349846	genic_upstream_transcript_variant,intron_variant
rs988361797	intron_variant
rs988406582	intron_variant
rs988431995	genic_upstream_transcript_variant,intron_variant
rs988499793	genic_upstream_transcript_variant,intron_variant
rs988503943	genic_downstream_transcript_variant,intron_variant
rs988513498	intron_variant
rs988531077	genic_upstream_transcript_variant,intron_variant
rs988565673	intron_variant
rs988575764	genic_upstream_transcript_variant,intron_variant
rs988583716	genic_downstream_transcript_variant,intron_variant
rs988668429	3_prime_UTR_variant,non_coding_transcript_variant,genic_downstream_transcript_variant
rs988697351	intron_variant
rs988753781	intron_variant
rs988776588	genic_upstream_transcript_variant,intron_variant
rs988791784	genic_upstream_transcript_variant,intron_variant
rs988815418	genic_downstream_transcript_variant,intron_variant
rs988849951	genic_upstream_transcript_variant,intron_variant
rs988886123	genic_downstream_transcript_variant,intron_variant
rs988894084	genic_upstream_transcript_variant,upstream_transcript_variant,5_prime_UTR_variant,intron_variant
rs988915282	intron_variant
rs988926131	genic_upstream_transcript_variant,intron_variant
rs988927727	genic_upstream_transcript_variant,intron_variant
rs988964281	genic_upstream_transcript_variant,intron_variant
rs988977811	intron_variant
rs989048729	intron_variant
rs989087729	intron_variant
rs989095276	intron_variant
rs989099156	genic_downstream_transcript_variant,intron_variant
rs989103431	genic_upstream_transcript_variant,intron_variant
rs989168407	genic_downstream_transcript_variant,intron_variant
rs989176315	intron_variant
rs989179135	intron_variant
rs989190168	intron_variant
rs989194073	genic_upstream_transcript_variant,intron_variant
rs989198149	genic_downstream_transcript_variant,intron_variant
rs989232737	intron_variant
rs989324791	genic_downstream_transcript_variant,intron_variant
rs989348912	intron_variant
rs989369657	intron_variant
rs989406007	genic_upstream_transcript_variant,intron_variant
rs989408663	genic_downstream_transcript_variant,intron_variant

rs989427485	genic_upstream_transcript_variant,intron_variant
rs989434404	intron_variant
rs989462044	genic_upstream_transcript_variant,intron_variant
rs989470725	genic_upstream_transcript_variant,intron_variant
rs989505963	genic_downstream_transcript_variant,intron_variant
rs989601976	intron_variant
rs989683659	genic_upstream_transcript_variant,intron_variant
rs989705330	genic_upstream_transcript_variant,intron_variant
rs989714104	intron_variant
rs989718727	genic_upstream_transcript_variant,intron_variant
rs989758811	intron_variant
rs989773239	genic_downstream_transcript_variant,intron_variant
rs989795579	genic_upstream_transcript_variant,intron_variant
rs989797000	2KB_upstream_variant,genic_upstream_transcript_variant,upstream_transcript_variant,intron_variant
rs989806139	intron_variant
rs989849573	intron_variant
rs989849784	intron_variant
rs989881732	genic_downstream_transcript_variant,intron_variant
rs989895818	genic_upstream_transcript_variant,intron_variant
rs989896685	genic_upstream_transcript_variant,intron_variant
rs989917810	genic_upstream_transcript_variant,intron_variant
rs989938278	genic_upstream_transcript_variant,intron_variant
rs990017039	intron_variant
rs990049323	intron_variant
rs990053641	genic_upstream_transcript_variant,intron_variant
rs990065840	genic_upstream_transcript_variant,intron_variant
rs990070124	genic_upstream_transcript_variant,intron_variant
rs990081136	genic_upstream_transcript_variant,intron_variant
rs990134645	genic_upstream_transcript_variant,intron_variant
rs990154098	intron_variant
rs990176631	genic_upstream_transcript_variant,intron_variant
rs990189238	genic_downstream_transcript_variant,intron_variant
rs990207732	genic_upstream_transcript_variant,intron_variant
rs990235861	downstream_transcript_variant,500B_downstream_variant
rs990252958	intron_variant
rs990256435	genic_downstream_transcript_variant,intron_variant
rs990314661	downstream_transcript_variant,500B_downstream_variant
rs990326581	genic_upstream_transcript_variant,intron_variant
rs990343551	intron_variant
rs990371186	genic_upstream_transcript_variant,intron_variant
rs990409276	2KB_upstream_variant,genic_upstream_transcript_variant,upstream_transcript_variant,intron_variant
rs990487563	intron_variant
rs990517508	genic_upstream_transcript_variant,intron_variant
rs990522367	intron_variant
rs990534696	genic_upstream_transcript_variant,intron_variant
rs990541339	genic_upstream_transcript_variant,intron_variant
rs990552853	intron_variant
rs990584028	genic_upstream_transcript_variant,intron_variant
rs990655739	genic_upstream_transcript_variant,intron_variant
rs990664513	genic_downstream_transcript_variant,intron_variant
rs990678243	genic_upstream_transcript_variant,intron_variant
rs990685314	intron_variant
rs990699363	genic_downstream_transcript_variant,intron_variant
rs990710128	intron_variant
rs990711829	genic_downstream_transcript_variant,intron_variant
rs990722629	intron_variant
rs990725522	intron_variant
rs990769847	genic_upstream_transcript_variant,intron_variant
rs990775058	intron_variant
rs990810039	genic_upstream_transcript_variant,intron_variant
rs990835856	genic_upstream_transcript_variant,intron_variant
rs990882775	genic_upstream_transcript_variant,intron_variant
rs990887610	intron_variant
rs990930106	genic_upstream_transcript_variant,intron_variant
rs990930546	genic_upstream_transcript_variant,intron_variant
rs990933387	intron_variant
rs990954161	genic_upstream_transcript_variant,intron_variant
rs990986455	intron_variant
rs991006414	genic_upstream_transcript_variant,intron_variant
rs991006697	genic_upstream_transcript_variant,intron_variant
rs991052338	intron_variant
rs991059198	2KB_upstream_variant,genic_upstream_transcript_variant,upstream_transcript_variant,intron_variant
rs991065157	genic_upstream_transcript_variant,intron_variant
rs991129711	intron_variant

rs991130335	genic_upstream_transcript_variant,intron_variant
rs991153114	intron_variant
rs991186035	genic_upstream_transcript_variant,intron_variant
rs991205896	genic_upstream_transcript_variant,intron_variant
rs991206513	genic_upstream_transcript_variant,intron_variant
rs991206925	intron_variant
rs991267818	genic_upstream_transcript_variant,intron_variant
rs991281941	2KB_upstream_variant,genic_upstream_transcript_variant,upstream_transcript_variant,intron_variant
rs991291109	genic_upstream_transcript_variant,intron_variant
rs991329093	genic_upstream_transcript_variant,intron_variant
rs991337030	genic_upstream_transcript_variant,intron_variant
rs991441243	genic_upstream_transcript_variant,intron_variant
rs991442086	intron_variant
rs991469471	genic_downstream_transcript_variant,intron_variant
rs991491791	genic_upstream_transcript_variant,intron_variant
rs991515989	genic_upstream_transcript_variant,intron_variant
rs991525012	genic_downstream_transcript_variant,intron_variant
rs991548906	genic_upstream_transcript_variant,intron_variant
rs991559473	intron_variant
rs991581488	2KB_upstream_variant,genic_upstream_transcript_variant,upstream_transcript_variant,intron_variant
rs991606505	genic_downstream_transcript_variant,intron_variant
rs991622405	genic_downstream_transcript_variant,intron_variant
rs991631759	intron_variant
rs991658716	intron_variant
rs991661249	genic_upstream_transcript_variant,intron_variant
rs991700463	genic_downstream_transcript_variant,intron_variant
rs991705596	intron_variant
rs991715197	intron_variant
rs991734264	genic_downstream_transcript_variant,intron_variant
rs991739492	genic_upstream_transcript_variant,intron_variant
rs991763134	genic_upstream_transcript_variant,intron_variant
rs991771212	genic_upstream_transcript_variant,intron_variant
rs991808143	genic_upstream_transcript_variant,intron_variant
rs991869722	2KB_upstream_variant,genic_upstream_transcript_variant,upstream_transcript_variant,intron_variant
rs991897273	intron_variant
rs991943492	genic_upstream_transcript_variant,intron_variant
rs991973659	intron_variant
rs991986446	intron_variant
rs991988641	genic_upstream_transcript_variant,intron_variant
rs992033274	genic_downstream_transcript_variant,intron_variant
rs992049401	genic_upstream_transcript_variant,intron_variant
rs992070374	genic_downstream_transcript_variant,intron_variant
rs992117060	genic_downstream_transcript_variant,intron_variant
rs992118366	genic_upstream_transcript_variant,intron_variant
rs992127980	intron_variant
rs992142681	intron_variant
rs992165999	genic_downstream_transcript_variant,intron_variant
rs992187660	genic_upstream_transcript_variant,intron_variant
rs992196815	genic_downstream_transcript_variant,intron_variant
rs992207730	genic_downstream_transcript_variant,intron_variant
rs992207872	genic_upstream_transcript_variant,intron_variant
rs992253090	genic_downstream_transcript_variant,intron_variant
rs992286735	genic_upstream_transcript_variant,intron_variant
rs992296182	intron_variant
rs992310986	genic_downstream_transcript_variant,intron_variant
rs992311181	intron_variant
rs992318612	intron_variant
rs992318885	genic_upstream_transcript_variant,intron_variant
rs992332467	genic_upstream_transcript_variant,intron_variant
rs992436511	intron_variant
rs992451383	intron_variant
rs992453949	genic_upstream_transcript_variant,intron_variant
rs992457555	intron_variant
rs992460190	genic_downstream_transcript_variant,intron_variant
rs992514559	intron_variant
rs992545867	intron_variant
rs992600347	genic_upstream_transcript_variant,intron_variant
rs992612842	genic_downstream_transcript_variant,intron_variant
rs992620810	intron_variant
rs992676718	genic_downstream_transcript_variant,intron_variant
rs992680197	genic_downstream_transcript_variant,intron_variant
rs992688421	intron_variant
rs992721848	genic_downstream_transcript_variant,intron_variant
rs992733809	intron_variant

rs992737239	genic_downstream_transcript_variant,intron_variant
rs992741818	genic_upstream_transcript_variant,intron_variant
rs992776395	intron_variant
rs992791698	genic_downstream_transcript_variant,intron_variant
rs992797905	genic_upstream_transcript_variant,intron_variant
rs992798021	genic_downstream_transcript_variant,intron_variant
rs992824518	genic_upstream_transcript_variant,intron_variant
rs992830734	intron_variant
rs992889106	intron_variant
rs992899466	intron_variant
rs992920676	intron_variant
rs992957608	genic_upstream_transcript_variant,intron_variant
rs992997056	intron_variant,upstream_transcript_variant,genic_upstream_transcript_variant,5_prime_UTR_variant
rs993008015	intron_variant
rs993013195	intron_variant
rs993039930	intron_variant
rs993056851	genic_upstream_transcript_variant,intron_variant
rs993178028	genic_downstream_transcript_variant,intron_variant
rs993178168	genic_upstream_transcript_variant,intron_variant
rs993191651	genic_downstream_transcript_variant,intron_variant
rs993207007	genic_upstream_transcript_variant,intron_variant
rs993246408	genic_downstream_transcript_variant,intron_variant
rs993256566	genic_upstream_transcript_variant,intron_variant
rs993259196	intron_variant
rs993303089	genic_downstream_transcript_variant,intron_variant
rs993334324	genic_downstream_transcript_variant,intron_variant
rs993379881	genic_upstream_transcript_variant,intron_variant
rs993402209	intron_variant
rs993440131	intron_variant
rs993441251	genic_upstream_transcript_variant,intron_variant
rs993463693	coding_sequence_variant,non_coding_transcript_variant,missense_variant
rs993471987	genic_upstream_transcript_variant,intron_variant
rs993491152	genic_upstream_transcript_variant,intron_variant
rs993505280	genic_upstream_transcript_variant,intron_variant
rs993523915	intron_variant
rs993604360	genic_downstream_transcript_variant,intron_variant
rs993613857	genic_downstream_transcript_variant,intron_variant
rs993637163	genic_downstream_transcript_variant,intron_variant
rs993642679	intron_variant
rs993656177	intron_variant
rs993670583	genic_upstream_transcript_variant,intron_variant
rs993685645	genic_upstream_transcript_variant,intron_variant
rs993706328	intron_variant
rs993739199	intron_variant
rs993770244	intron_variant
rs993774563	genic_upstream_transcript_variant,intron_variant
rs993797898	intron_variant
rs993805774	genic_upstream_transcript_variant,intron_variant
rs993826820	intron_variant
rs993829568	genic_downstream_transcript_variant,intron_variant
rs993846364	genic_downstream_transcript_variant,intron_variant
rs993855215	intron_variant
rs993863440	intron_variant
rs993917587	genic_upstream_transcript_variant,intron_variant
rs993917843	intron_variant
rs993930730	intron_variant
rs993939813	genic_downstream_transcript_variant,intron_variant
rs993958690	intron_variant
rs993960836	genic_upstream_transcript_variant,intron_variant
rs993960848	upstream_transcript_variant,genic_upstream_transcript_variant,intron_variant
rs993969559	genic_downstream_transcript_variant,intron_variant
rs993979083	intron_variant
rs994038131	intron_variant
rs994072372	intron_variant
rs994078101	genic_downstream_transcript_variant,intron_variant
rs994098897	genic_downstream_transcript_variant,intron_variant
rs994102053	intron_variant
rs994130367	genic_upstream_transcript_variant,intron_variant
rs994131939	genic_upstream_transcript_variant,intron_variant
rs994133135	intron_variant
rs994147235	genic_downstream_transcript_variant,intron_variant
rs994165900	intron_variant
rs994168985	genic_upstream_transcript_variant,intron_variant
rs994171070	genic_upstream_transcript_variant,intron_variant

rs994188177	intron_variant
rs994221243	genic_upstream_transcript_variant,intron_variant
rs994243395	intron_variant
rs994253237	genic_downstream_transcript_variant,intron_variant
rs994342540	intron_variant
rs994362958	genic_downstream_transcript_variant,intron_variant
rs994368430	genic_upstream_transcript_variant,intron_variant
rs994408280	genic_upstream_transcript_variant,intron_variant
rs994433716	intron_variant
rs994449189	genic_upstream_transcript_variant,intron_variant
rs994449938	genic_downstream_transcript_variant,intron_variant
rs994481142	genic_downstream_transcript_variant,intron_variant
rs994502877	genic_upstream_transcript_variant,intron_variant
rs994504555	intron_variant
rs994563287	genic_upstream_transcript_variant,intron_variant
rs994563692	genic_upstream_transcript_variant,intron_variant
rs994569872	intron_variant
rs994572662	genic_upstream_transcript_variant,intron_variant
rs994657746	genic_downstream_transcript_variant,intron_variant
rs994668730	genic_upstream_transcript_variant,intron_variant
rs994715212	intron_variant
rs994747518	genic_downstream_transcript_variant,intron_variant
rs994748444	intron_variant
rs994816846	intron_variant
rs994836131	genic_upstream_transcript_variant,intron_variant
rs994914510	genic_downstream_transcript_variant,intron_variant
rs994916914	intron_variant
rs994945808	genic_upstream_transcript_variant,intron_variant
rs994984192	intron_variant
rs995002483	genic_upstream_transcript_variant,intron_variant
rs995008616	3_prime_UTR_variant,genic_downstream_transcript_variant,non_coding_transcript_variant
rs995013621	intron_variant
rs995040442	genic_downstream_transcript_variant,intron_variant
rs995087774	genic_downstream_transcript_variant,intron_variant
rs995097064	intron_variant
rs995108743	genic_upstream_transcript_variant,intron_variant
rs995119569	genic_upstream_transcript_variant,intron_variant
rs995170419	genic_upstream_transcript_variant,intron_variant
rs995201080	genic_downstream_transcript_variant,intron_variant
rs995271235	intron_variant
rs995310318	downstream_transcript_variant,500B_downstream_variant
rs995327283	genic_upstream_transcript_variant,intron_variant
rs995336445	intron_variant
rs995347729	intron_variant
rs995358106	genic_upstream_transcript_variant,intron_variant
rs995370095	intron_variant
rs995382671	intron_variant
rs995441347	genic_upstream_transcript_variant,intron_variant
rs995443711	intron_variant
rs995454502	genic_upstream_transcript_variant,intron_variant
rs995586811	genic_upstream_transcript_variant,intron_variant
rs995685139	intron_variant
rs995696658	genic_upstream_transcript_variant,intron_variant
rs995699477	intron_variant
rs995750018	intron_variant
rs995768923	genic_downstream_transcript_variant,intron_variant
rs995775115	genic_downstream_transcript_variant,intron_variant
rs995778162	intron_variant
rs995789751	genic_upstream_transcript_variant,intron_variant
rs995802187	intron_variant
rs995813209	genic_upstream_transcript_variant,intron_variant
rs995830924	genic_downstream_transcript_variant,intron_variant
rs995831878	intron_variant
rs995841732	intron_variant
rs995853014	intron_variant
rs995858972	genic_downstream_transcript_variant,intron_variant
rs995876187	genic_downstream_transcript_variant,intron_variant
rs995928644	genic_downstream_transcript_variant,intron_variant
rs996007740	intron_variant
rs996089755	intron_variant
rs996116969	intron_variant
rs996122563	genic_upstream_transcript_variant,intron_variant
rs996130950	genic_downstream_transcript_variant,intron_variant
rs996151223	intron_variant

rs996183018	genic_upstream_transcript_variant,intron_variant
rs996198269	genic_upstream_transcript_variant,intron_variant
rs996232306	2KB_upstream_variant,genic_upstream_transcript_variant,upstream_transcript_variant,intron_variant
rs996243551	3_prime_UTR_variant,genic_downstream_transcript_variant,non_coding_transcript_variant
rs996251973	genic_downstream_transcript_variant,intron_variant
rs996330199	genic_upstream_transcript_variant,intron_variant
rs996352712	intron_variant
rs996381912	intron_variant
rs996421480	genic_upstream_transcript_variant,intron_variant
rs996447836	intron_variant
rs996482112	genic_downstream_transcript_variant,intron_variant
rs996484697	intron_variant
rs996492493	genic_upstream_transcript_variant,intron_variant
rs996523401	intron_variant
rs996524422	genic_downstream_transcript_variant,intron_variant
rs996529473	genic_upstream_transcript_variant,intron_variant
rs996567812	genic_downstream_transcript_variant,intron_variant
rs996580734	3_prime_UTR_variant,genic_downstream_transcript_variant,non_coding_transcript_variant
rs996584253	genic_upstream_transcript_variant,intron_variant
rs996606183	genic_upstream_transcript_variant,intron_variant
rs996643070	intron_variant
rs996661930	genic_upstream_transcript_variant,intron_variant
rs996754212	intron_variant
rs996807036	genic_downstream_transcript_variant,intron_variant
rs996828941	genic_upstream_transcript_variant,intron_variant
rs996857540	genic_upstream_transcript_variant,intron_variant
rs996861682	genic_upstream_transcript_variant,intron_variant
rs996866475	genic_upstream_transcript_variant,intron_variant
rs996893026	genic_upstream_transcript_variant,intron_variant
rs996914753	intron_variant
rs996960750	genic_upstream_transcript_variant,intron_variant
rs996964651	genic_downstream_transcript_variant,intron_variant
rs996968225	genic_downstream_transcript_variant,intron_variant
rs996982807	intron_variant
rs996990653	intron_variant
rs996999672	genic_upstream_transcript_variant,intron_variant
rs997042787	genic_upstream_transcript_variant,intron_variant
rs997075615	intron_variant
rs997118055	intron_variant
rs997122000	genic_upstream_transcript_variant,intron_variant
rs997123488	intron_variant
rs997165841	genic_upstream_transcript_variant,intron_variant
rs997184113	genic_upstream_transcript_variant,intron_variant
rs997216554	intron_variant
rs997246245	genic_upstream_transcript_variant,intron_variant
rs997315113	genic_upstream_transcript_variant,intron_variant
rs997315169	intron_variant
rs997317328	genic_upstream_transcript_variant,intron_variant
rs997343275	genic_downstream_transcript_variant,intron_variant
rs997362224	intron_variant
rs997382443	intron_variant
rs997395862	genic_downstream_transcript_variant,intron_variant
rs997447943	genic_downstream_transcript_variant,intron_variant
rs997454064	intron_variant
rs997472553	genic_upstream_transcript_variant,intron_variant
rs997476737	genic_upstream_transcript_variant,intron_variant
rs997506424	intron_variant
rs997551849	genic_upstream_transcript_variant,intron_variant
rs997578626	2KB_upstream_variant,genic_upstream_transcript_variant,upstream_transcript_variant,intron_variant
rs997587482	intron_variant
rs997591983	genic_upstream_transcript_variant,intron_variant
rs997625508	genic_upstream_transcript_variant,intron_variant
rs997716772	genic_upstream_transcript_variant,intron_variant
rs997755332	intron_variant
rs997767223	intron_variant
rs997826108	genic_downstream_transcript_variant,intron_variant
rs997843036	intron_variant
rs997892243	genic_upstream_transcript_variant,intron_variant
rs997892556	genic_downstream_transcript_variant,intron_variant
rs997923925	intron_variant
rs998002711	genic_upstream_transcript_variant,intron_variant
rs998004041	genic_upstream_transcript_variant,intron_variant
rs998023570	intron_variant
rs998027346	intron_variant,upstream_transcript_variant,genic_upstream_transcript_variant,5_prime_UTR_variant

rs998068389	intron_variant
rs998089560	intron_variant
rs998090462	2KB_upstream_variant,genic_upstream_transcript_variant,upstream_transcript_variant,intron_variant
rs998124540	genic_upstream_transcript_variant,intron_variant
rs998132952	intron_variant
rs998187776	2KB_upstream_variant,genic_upstream_transcript_variant,upstream_transcript_variant,intron_variant
rs998212506	genic_downstream_transcript_variant,intron_variant
rs998262576	genic_downstream_transcript_variant,intron_variant
rs998273280	genic_upstream_transcript_variant,intron_variant
rs998294531	2KB_upstream_variant,genic_upstream_transcript_variant,upstream_transcript_variant,intron_variant
rs998314916	genic_downstream_transcript_variant,intron_variant
rs998341570	intron_variant
rs998353995	genic_upstream_transcript_variant,intron_variant
rs998373028	genic_downstream_transcript_variant,intron_variant
rs998383872	intron_variant
rs998393632	intron_variant
rs998440362	intron_variant
rs998442672	intron_variant
rs998448005	genic_upstream_transcript_variant,intron_variant
rs998456907	intron_variant,non_coding_transcript_variant,genic_upstream_transcript_variant,5_prime_UTR_variant
rs998467231	genic_downstream_transcript_variant,intron_variant
rs998485529	genic_downstream_transcript_variant,intron_variant
rs998496104	intron_variant
rs998498355	genic_downstream_transcript_variant,intron_variant
rs998512918	genic_upstream_transcript_variant,intron_variant
rs998529969	intron_variant
rs998575479	genic_downstream_transcript_variant,intron_variant
rs998593126	intron_variant
rs998615785	2KB_upstream_variant,genic_upstream_transcript_variant,upstream_transcript_variant,intron_variant
rs998666387	intron_variant
rs998696664	genic_downstream_transcript_variant,intron_variant
rs998700394	genic_upstream_transcript_variant,intron_variant
rs998722895	genic_upstream_transcript_variant,intron_variant
rs998731382	genic_upstream_transcript_variant,intron_variant
rs998746785	intron_variant
rs998755745	upstream_transcript_variant,genic_upstream_transcript_variant,intron_variant
rs998768435	intron_variant
rs998813064	genic_downstream_transcript_variant,intron_variant
rs998815828	genic_downstream_transcript_variant,intron_variant
rs998839828	genic_downstream_transcript_variant,intron_variant
rs998840021	genic_upstream_transcript_variant,intron_variant
rs998874309	genic_upstream_transcript_variant,intron_variant
rs998894755	intron_variant
rs998902975	intron_variant
rs998904013	intron_variant
rs998912061	genic_upstream_transcript_variant,intron_variant
rs998917673	genic_upstream_transcript_variant,intron_variant
rs998964185	intron_variant
rs998990154	2KB_upstream_variant,genic_upstream_transcript_variant,upstream_transcript_variant,intron_variant
rs999016283	intron_variant
rs999022239	genic_upstream_transcript_variant,intron_variant
rs999048739	intron_variant
rs999061741	intron_variant
rs999092620	intron_variant
rs999098207	genic_upstream_transcript_variant,intron_variant
rs999107986	genic_downstream_transcript_variant,intron_variant
rs999109183	genic_upstream_transcript_variant,intron_variant
rs999151222	genic_downstream_transcript_variant,intron_variant
rs999248584	genic_upstream_transcript_variant,intron_variant
rs999286870	intron_variant
rs999342085	intron_variant
rs999362705	intron_variant
rs999385495	intron_variant
rs999406265	genic_upstream_transcript_variant,intron_variant
rs999436100	intron_variant
rs999468563	genic_upstream_transcript_variant,intron_variant
rs999471313	genic_upstream_transcript_variant,intron_variant
rs999496385	intron_variant
rs999514613	genic_downstream_transcript_variant,intron_variant
rs999527755	genic_upstream_transcript_variant,intron_variant
rs999606436	intron_variant
rs999617615	intron_variant
rs999636479	intron_variant
rs999661433	genic_upstream_transcript_variant,intron_variant

rs999693978	genic_upstream_transcript_variant,intron_variant
rs999719585	intron_variant
rs999752887	genic_upstream_transcript_variant,intron_variant
rs999758797	genic_upstream_transcript_variant,intron_variant
rs999796429	genic_upstream_transcript_variant,intron_variant
rs999801684	genic_upstream_transcript_variant,intron_variant
rs999807091	genic_upstream_transcript_variant,intron_variant
rs999841620	genic_downstream_transcript_variant,intron_variant
rs999848531	genic_upstream_transcript_variant,intron_variant
rs999914600	genic_upstream_transcript_variant,intron_variant
rs999944341	genic_downstream_transcript_variant,intron_variant
rs999986628	genic_upstream_transcript_variant,intron_variant
rs1000009944	intron_variant
rs1000044357	genic_downstream_transcript_variant,intron_variant
rs1000047140	genic_downstream_transcript_variant,intron_variant
rs1000053326	intron_variant
rs1000070601	genic_upstream_transcript_variant,intron_variant
rs1000114390	genic_downstream_transcript_variant,intron_variant
rs1000183177	genic_upstream_transcript_variant,intron_variant
rs1000206092	genic_downstream_transcript_variant,intron_variant
rs1000211667	genic_upstream_transcript_variant,intron_variant
rs1000216862	intron_variant
rs1000238085	intron_variant
rs1000278836	genic_downstream_transcript_variant,intron_variant
rs1000279027	genic_downstream_transcript_variant,intron_variant
rs1000302339	intron_variant
rs1000317282	genic_upstream_transcript_variant,intron_variant
rs1000337119	upstream_transcript_variant,genic_upstream_transcript_variant,intron_variant
rs1000499252	intron_variant
rs1000499771	genic_downstream_transcript_variant,downstream_transcript_variant,intron_variant
rs1000523804	genic_upstream_transcript_variant,intron_variant
rs1000543705	genic_downstream_transcript_variant,intron_variant
rs1000562570	genic_downstream_transcript_variant,intron_variant
rs1000596234	intron_variant
rs1000679280	genic_upstream_transcript_variant,intron_variant
rs1000711011	genic_downstream_transcript_variant,intron_variant
rs1000721472	genic_upstream_transcript_variant,intron_variant
rs1000748102	intron_variant
rs1000754117	genic_upstream_transcript_variant,intron_variant
rs1000816776	genic_upstream_transcript_variant,intron_variant
rs1000824515	genic_upstream_transcript_variant,intron_variant
rs1000874165	genic_upstream_transcript_variant,intron_variant
rs1000883062	intron_variant
rs1000920418	genic_upstream_transcript_variant,intron_variant
rs1000933222	intron_variant
rs1000949734	genic_upstream_transcript_variant,intron_variant
rs1000953077	intron_variant
rs1000973009	genic_upstream_transcript_variant,intron_variant
rs1000981999	genic_upstream_transcript_variant,intron_variant
rs1001071336	genic_upstream_transcript_variant,intron_variant
rs1001140887	intron_variant,genic_upstream_transcript_variant
rs1001163976	intron_variant
rs1001191474	intron_variant,genic_upstream_transcript_variant
rs1001193997	genic_downstream_transcript_variant,intron_variant
rs1001197590	upstream_transcript_variant,intron_variant,genic_upstream_transcript_variant
rs1001299939	intron_variant
rs1001306594	intron_variant
rs1001317171	genic_downstream_transcript_variant,intron_variant
rs1001349628	intron_variant
rs1001362196	genic_downstream_transcript_variant,intron_variant
rs1001371751	3_prime_UTR_variant,genic_downstream_transcript_variant,non_coding_transcript_variant
rs1001378562	intron_variant,genic_upstream_transcript_variant
rs1001401932	intron_variant
rs1001442050	intron_variant
rs1001444582	intron_variant,genic_upstream_transcript_variant
rs1001466539	intron_variant,genic_upstream_transcript_variant
rs1001529661	intron_variant,genic_upstream_transcript_variant
rs1001541938	genic_downstream_transcript_variant,intron_variant
rs1001569367	intron_variant
rs1001610722	intron_variant
rs1001620551	intron_variant
rs1001624251	genic_downstream_transcript_variant,intron_variant
rs1001657793	intron_variant,genic_upstream_transcript_variant
rs1001662026	intron_variant,genic_upstream_transcript_variant

rs1001700956	intron_variant
rs1001730845	intron_variant
rs1001759760	intron_variant,genic_upstream_transcript_variant
rs1001760551	intron_variant
rs1001791621	intron_variant
rs1001811893	upstream_transcript_variant,intron_variant,2KB_upstream_variant,genic_upstream_transcript_variant
rs1001834380	genic_downstream_transcript_variant,intron_variant
rs1001871525	intron_variant,genic_upstream_transcript_variant
rs1001949722	intron_variant
rs1001952714	intron_variant
rs1001958761	intron_variant,genic_upstream_transcript_variant
rs1001974556	downstream_transcript_variant,intron_variant,genic_downstream_transcript_variant
rs1002015914	intron_variant
rs1002027875	intron_variant
rs1002041305	genic_downstream_transcript_variant,intron_variant
rs1002046814	intron_variant
rs1002054002	intron_variant,genic_upstream_transcript_variant
rs1002071725	intron_variant
rs1002074309	intron_variant,genic_upstream_transcript_variant
rs1002079430	intron_variant
rs1002180885	genic_downstream_transcript_variant,intron_variant
rs1002211508	intron_variant
rs1002211935	genic_downstream_transcript_variant,intron_variant
rs1002248049	intron_variant
rs1002286461	non_coding_transcript_variant,intron_variant,genic_upstream_transcript_variant,missense_variant,coding_sequence_variant
rs1002299810	upstream_transcript_variant,intron_variant,2KB_upstream_variant,genic_upstream_transcript_variant
rs1002318757	intron_variant
rs1002319778	intron_variant,genic_upstream_transcript_variant
rs1002344800	downstream_transcript_variant,intron_variant,genic_downstream_transcript_variant
rs1002378785	intron_variant,genic_upstream_transcript_variant
rs1002399843	intron_variant,genic_upstream_transcript_variant
rs1002428573	intron_variant,genic_upstream_transcript_variant
rs1002449966	genic_downstream_transcript_variant,intron_variant
rs1002455394	3_prime_UTR_variant,genic_downstream_transcript_variant,non_coding_transcript_variant
rs1002492806	intron_variant
rs1002507339	intron_variant,genic_upstream_transcript_variant
rs1002527375	intron_variant,genic_upstream_transcript_variant
rs1002546705	genic_downstream_transcript_variant,intron_variant
rs1002549466	intron_variant,genic_upstream_transcript_variant
rs1002580125	intron_variant
rs1002609864	intron_variant
rs1002620381	intron_variant
rs1002677132	upstream_transcript_variant,intron_variant,2KB_upstream_variant,genic_upstream_transcript_variant
rs1002721456	upstream_transcript_variant,intron_variant,2KB_upstream_variant,genic_upstream_transcript_variant
rs1002731933	intron_variant,genic_upstream_transcript_variant
rs1002741309	intron_variant
rs1002758853	genic_downstream_transcript_variant,intron_variant
rs1002793749	3_prime_UTR_variant,genic_downstream_transcript_variant,non_coding_transcript_variant
rs1002822713	genic_downstream_transcript_variant,intron_variant
rs1002835268	intron_variant
rs1002850794	intron_variant
rs1002869137	genic_downstream_transcript_variant,intron_variant
rs1002881270	intron_variant
rs1002884026	intron_variant,genic_upstream_transcript_variant
rs1002940599	intron_variant
rs1002969531	intron_variant,genic_upstream_transcript_variant
rs1002978484	intron_variant
rs1003013474	intron_variant
rs1003071421	intron_variant,genic_upstream_transcript_variant
rs1003101252	genic_downstream_transcript_variant,intron_variant
rs1003118216	intron_variant,genic_upstream_transcript_variant
rs1003131579	upstream_transcript_variant,intron_variant,2KB_upstream_variant,genic_upstream_transcript_variant
rs1003165324	genic_downstream_transcript_variant,intron_variant
rs1003168327	intron_variant
rs1003202294	downstream_transcript_variant,500B_downstream_variant
rs1003213873	intron_variant,genic_upstream_transcript_variant
rs1003217071	intron_variant,genic_upstream_transcript_variant
rs1003272984	intron_variant,genic_upstream_transcript_variant
rs1003344393	intron_variant
rs1003376924	downstream_transcript_variant,intron_variant,genic_downstream_transcript_variant
rs1003393893	genic_downstream_transcript_variant,intron_variant
rs1003435890	genic_downstream_transcript_variant,intron_variant
rs1003438588	genic_downstream_transcript_variant,intron_variant
rs1003451205	intron_variant

rs1003454294	intron_variant,genic_upstream_transcript_variant
rs1003483466	intron_variant,genic_upstream_transcript_variant
rs1003499444	genic_downstream_transcript_variant,intron_variant
rs1003541452	intron_variant,genic_upstream_transcript_variant
rs1003608007	upstream_transcript_variant,intron_variant,2KB_upstream_variant,genic_upstream_transcript_variant
rs1003623437	intron_variant
rs1003696060	intron_variant
rs1003706270	intron_variant,genic_upstream_transcript_variant
rs1003715756	intron_variant,genic_upstream_transcript_variant
rs1003727088	intron_variant,genic_upstream_transcript_variant
rs1003739705	intron_variant,genic_upstream_transcript_variant
rs1003745298	downstream_transcript_variant,500B_downstream_variant
rs1003746064	intron_variant,genic_upstream_transcript_variant
rs1003831301	genic_downstream_transcript_variant,intron_variant
rs1003841607	downstream_transcript_variant,intron_variant,genic_downstream_transcript_variant
rs1003853735	intron_variant,genic_upstream_transcript_variant
rs1003879784	genic_downstream_transcript_variant,intron_variant
rs1003903164	intron_variant
rs1003954947	intron_variant
rs1003971285	intron_variant
rs1003981633	upstream_transcript_variant,intron_variant,2KB_upstream_variant,genic_upstream_transcript_variant
rs1004022625	intron_variant
rs1004043921	intron_variant
rs1004077253	intron_variant,genic_upstream_transcript_variant
rs1004120708	intron_variant,genic_upstream_transcript_variant
rs1004131526	intron_variant,genic_upstream_transcript_variant
rs1004149775	intron_variant,genic_upstream_transcript_variant
rs1004149789	genic_downstream_transcript_variant,intron_variant
rs1004173170	intron_variant,genic_upstream_transcript_variant
rs1004182013	intron_variant
rs1004208029	upstream_transcript_variant,intron_variant,2KB_upstream_variant,genic_upstream_transcript_variant
rs1004216014	genic_downstream_transcript_variant,intron_variant
rs1004260803	intron_variant,genic_upstream_transcript_variant
rs1004260943	intron_variant
rs1004273183	intron_variant,genic_upstream_transcript_variant
rs1004304246	intron_variant
rs1004304562	upstream_transcript_variant,intron_variant,2KB_upstream_variant,genic_upstream_transcript_variant
rs1004381109	intron_variant,genic_upstream_transcript_variant
rs1004428230	intron_variant
rs1004457665	intron_variant,genic_upstream_transcript_variant
rs1004463767	intron_variant
rs1004488036	intron_variant
rs1004503190	intron_variant,genic_upstream_transcript_variant
rs1004532112	genic_downstream_transcript_variant,intron_variant
rs1004541337	intron_variant,genic_upstream_transcript_variant
rs1004574017	genic_downstream_transcript_variant,intron_variant
rs1004606812	intron_variant,genic_upstream_transcript_variant
rs1004633827	genic_downstream_transcript_variant,intron_variant
rs1004649513	intron_variant
rs1004653463	intron_variant
rs1004673037	intron_variant
rs1004675623	genic_downstream_transcript_variant,intron_variant
rs1004690545	intron_variant
rs1004699006	intron_variant,genic_upstream_transcript_variant
rs1004708799	genic_downstream_transcript_variant,intron_variant
rs1004722383	intron_variant
rs1004743627	intron_variant
rs1004764037	intron_variant
rs1004771606	intron_variant,genic_upstream_transcript_variant
rs1004774947	genic_downstream_transcript_variant,intron_variant
rs1004826950	intron_variant
rs1004831614	genic_downstream_transcript_variant,intron_variant
rs1004880414	intron_variant,genic_upstream_transcript_variant
rs1004890065	intron_variant,genic_upstream_transcript_variant
rs1004894408	intron_variant
rs1000044357	genic_downstream_transcript_variant,intron_variant
rs1000047140	genic_downstream_transcript_variant,intron_variant
rs1000053326	intron_variant
rs1000070601	genic_upstream_transcript_variant,intron_variant
rs1000114390	genic_downstream_transcript_variant,intron_variant
rs1000183177	genic_upstream_transcript_variant,intron_variant
rs1000206092	genic_downstream_transcript_variant,intron_variant
rs1000211667	genic_upstream_transcript_variant,intron_variant
rs1000216862	intron_variant

rs1000238085	intron_variant
rs1000278836	genic_downstream_transcript_variant,intron_variant
rs1000279027	genic_downstream_transcript_variant,intron_variant
rs1000302339	intron_variant
rs1000317282	genic_upstream_transcript_variant,intron_variant
rs1000337119	upstream_transcript_variant,genic_upstream_transcript_variant,intron_variant
rs1000499252	intron_variant
rs1000499771	genic_downstream_transcript_variant,downstream_transcript_variant,intron_variant
rs1000523804	genic_upstream_transcript_variant,intron_variant
rs1000543705	genic_downstream_transcript_variant,intron_variant
rs1000562570	genic_downstream_transcript_variant,intron_variant
rs1000596234	intron_variant
rs1000679280	genic_upstream_transcript_variant,intron_variant
rs1000711011	genic_downstream_transcript_variant,intron_variant
rs1000721472	genic_upstream_transcript_variant,intron_variant
rs1000748102	intron_variant
rs1000754117	genic_upstream_transcript_variant,intron_variant
rs1000816776	genic_upstream_transcript_variant,intron_variant
rs1000824515	genic_upstream_transcript_variant,intron_variant
rs1000874165	genic_upstream_transcript_variant,intron_variant
rs1000883062	intron_variant
rs1000920418	genic_upstream_transcript_variant,intron_variant
rs1000933222	intron_variant
rs1000949734	genic_upstream_transcript_variant,intron_variant
rs1000953077	intron_variant
rs1000973009	genic_upstream_transcript_variant,intron_variant
rs1000981999	genic_upstream_transcript_variant,intron_variant
rs1001071336	genic_upstream_transcript_variant,intron_variant
rs1001140887	intron_variant,genic_upstream_transcript_variant
rs1001163976	intron_variant
rs1001191474	intron_variant,genic_upstream_transcript_variant
rs1001193997	genic_downstream_transcript_variant,intron_variant
rs1001197590	upstream_transcript_variant,intron_variant,genic_upstream_transcript_variant
rs1001299939	intron_variant
rs1001306594	intron_variant
rs1001317171	genic_downstream_transcript_variant,intron_variant
rs1001349628	intron_variant
rs1001362196	genic_downstream_transcript_variant,intron_variant
rs1001371751	3_prime_UTR_variant,genic_downstream_transcript_variant,non_coding_transcript_variant
rs1001378562	intron_variant,genic_upstream_transcript_variant
rs1001401932	intron_variant
rs1001442050	intron_variant
rs1001444582	intron_variant,genic_upstream_transcript_variant
rs1001466539	intron_variant,genic_upstream_transcript_variant
rs1001529661	intron_variant,genic_upstream_transcript_variant
rs1001541938	genic_downstream_transcript_variant,intron_variant
rs1001569367	intron_variant
rs1001610722	intron_variant
rs1001620551	intron_variant
rs1001624251	genic_downstream_transcript_variant,intron_variant
rs1001657793	intron_variant,genic_upstream_transcript_variant
rs1001662026	intron_variant,genic_upstream_transcript_variant
rs1001700956	intron_variant
rs1001730845	intron_variant
rs1001759760	intron_variant,genic_upstream_transcript_variant
rs1001760551	intron_variant
rs1001791621	intron_variant
rs1001811893	upstream_transcript_variant,intron_variant,2KB_upstream_variant,genic_upstream_transcript_variant
rs1001834380	genic_downstream_transcript_variant,intron_variant
rs1001871525	intron_variant,genic_upstream_transcript_variant
rs1001949722	intron_variant
rs1001952714	intron_variant
rs1001958761	intron_variant,genic_upstream_transcript_variant
rs1001974556	downstream_transcript_variant,intron_variant,genic_downstream_transcript_variant
rs1002015914	intron_variant
rs1002027875	intron_variant
rs1002041305	genic_downstream_transcript_variant,intron_variant
rs1002046814	intron_variant
rs1002054002	intron_variant,genic_upstream_transcript_variant
rs1002071725	intron_variant
rs1002074309	intron_variant,genic_upstream_transcript_variant
rs1002079430	intron_variant
rs1002180885	genic_downstream_transcript_variant,intron_variant
rs1002211508	intron_variant

rs1002211935	genic_downstream_transcript_variant,intron_variant
rs1002248049	intron_variant
rs1002286461	non_coding_transcript_variant,intron_variant,genic_upstream_transcript_variant,missense_variant,coding_sequence_variant
rs1002299810	upstream_transcript_variant,intron_variant,2KB_upstream_variant,genic_upstream_transcript_variant
rs1002318757	intron_variant
rs1002319778	intron_variant,genic_upstream_transcript_variant
rs1002344800	downstream_transcript_variant,intron_variant,genic_downstream_transcript_variant
rs1002378785	intron_variant,genic_upstream_transcript_variant
rs1002399843	intron_variant,genic_upstream_transcript_variant
rs1002428573	intron_variant,genic_upstream_transcript_variant
rs1002449966	genic_downstream_transcript_variant,intron_variant
rs1002455394	3_prime_UTR_variant,genic_downstream_transcript_variant,non_coding_transcript_variant
rs1002492806	intron_variant
rs1002507339	intron_variant,genic_upstream_transcript_variant
rs1002527375	intron_variant,genic_upstream_transcript_variant
rs1002546705	genic_downstream_transcript_variant,intron_variant
rs1002549466	intron_variant,genic_upstream_transcript_variant
rs1002580125	intron_variant
rs1002609864	intron_variant
rs1002620381	intron_variant
rs1002677132	upstream_transcript_variant,intron_variant,2KB_upstream_variant,genic_upstream_transcript_variant
rs1002721456	upstream_transcript_variant,intron_variant,2KB_upstream_variant,genic_upstream_transcript_variant
rs1002731933	intron_variant,genic_upstream_transcript_variant
rs1002741309	intron_variant
rs1002758853	genic_downstream_transcript_variant,intron_variant
rs1002793749	3_prime_UTR_variant,genic_downstream_transcript_variant,non_coding_transcript_variant
rs1002822713	genic_downstream_transcript_variant,intron_variant
rs1002835268	intron_variant
rs1002850794	intron_variant
rs1002869137	genic_downstream_transcript_variant,intron_variant
rs1002881270	intron_variant
rs1002884026	intron_variant,genic_upstream_transcript_variant
rs1002940599	intron_variant
rs1002969531	intron_variant,genic_upstream_transcript_variant
rs1002978484	intron_variant
rs1003013474	intron_variant
rs1003071421	intron_variant,genic_upstream_transcript_variant
rs1003101252	genic_downstream_transcript_variant,intron_variant
rs1003118216	intron_variant,genic_upstream_transcript_variant
rs1003131579	upstream_transcript_variant,intron_variant,2KB_upstream_variant,genic_upstream_transcript_variant
rs1003165324	genic_downstream_transcript_variant,intron_variant
rs1003168327	intron_variant
rs1003202294	downstream_transcript_variant,500B_downstream_variant
rs1003213873	intron_variant,genic_upstream_transcript_variant
rs1003217071	intron_variant,genic_upstream_transcript_variant
rs1003272984	intron_variant,genic_upstream_transcript_variant
rs1003344393	intron_variant
rs1003376924	downstream_transcript_variant,intron_variant,genic_downstream_transcript_variant
rs1003393893	genic_downstream_transcript_variant,intron_variant
rs1003435890	genic_downstream_transcript_variant,intron_variant
rs1003438588	genic_downstream_transcript_variant,intron_variant
rs1003451205	intron_variant
rs1003454294	intron_variant,genic_upstream_transcript_variant
rs1003483466	intron_variant,genic_upstream_transcript_variant
rs1003499444	genic_downstream_transcript_variant,intron_variant
rs1003541452	intron_variant,genic_upstream_transcript_variant
rs1003608007	upstream_transcript_variant,intron_variant,2KB_upstream_variant,genic_upstream_transcript_variant
rs1003623437	intron_variant
rs1003696060	intron_variant
rs1003706270	intron_variant,genic_upstream_transcript_variant
rs1003715756	intron_variant,genic_upstream_transcript_variant
rs1003727088	intron_variant,genic_upstream_transcript_variant
rs1003739705	intron_variant,genic_upstream_transcript_variant
rs1003745298	downstream_transcript_variant,500B_downstream_variant
rs1003746064	intron_variant,genic_upstream_transcript_variant
rs1003831301	genic_downstream_transcript_variant,intron_variant
rs1003841607	downstream_transcript_variant,intron_variant,genic_downstream_transcript_variant
rs1003853735	intron_variant,genic_upstream_transcript_variant
rs1003879784	genic_downstream_transcript_variant,intron_variant
rs1003903164	intron_variant
rs1003954947	intron_variant
rs1003971285	intron_variant
rs1003981633	upstream_transcript_variant,intron_variant,2KB_upstream_variant,genic_upstream_transcript_variant
rs1004022625	intron_variant

rs1004043921	intron_variant
rs1004077253	intron_variant,genic_upstream_transcript_variant
rs1004120708	intron_variant,genic_upstream_transcript_variant
rs1004131526	intron_variant,genic_upstream_transcript_variant
rs1004149775	intron_variant,genic_upstream_transcript_variant
rs1004149789	genic_downstream_transcript_variant,intron_variant
rs1004173170	intron_variant,genic_upstream_transcript_variant
rs1004182013	intron_variant
rs1004208029	upstream_transcript_variant,intron_variant,2KB_upstream_variant,genic_upstream_transcript_variant
rs1004216014	genic_downstream_transcript_variant,intron_variant
rs1004260803	intron_variant,genic_upstream_transcript_variant
rs1004260943	intron_variant
rs1004273183	intron_variant,genic_upstream_transcript_variant
rs1004304246	intron_variant
rs1004304562	upstream_transcript_variant,intron_variant,2KB_upstream_variant,genic_upstream_transcript_variant
rs1004381109	intron_variant,genic_upstream_transcript_variant
rs1004428230	intron_variant
rs1004457665	intron_variant,genic_upstream_transcript_variant
rs1004463767	intron_variant
rs1004488036	intron_variant
rs1004503190	intron_variant,genic_upstream_transcript_variant
rs1004532112	genic_downstream_transcript_variant,intron_variant
rs1004541337	intron_variant,genic_upstream_transcript_variant
rs1004574017	genic_downstream_transcript_variant,intron_variant
rs1004606812	intron_variant,genic_upstream_transcript_variant
rs1004633827	genic_downstream_transcript_variant,intron_variant
rs1004649513	intron_variant
rs1004653463	intron_variant
rs1004673037	intron_variant
rs1004675623	genic_downstream_transcript_variant,intron_variant
rs1004690545	intron_variant
rs1004699006	intron_variant,genic_upstream_transcript_variant
rs1004708799	genic_downstream_transcript_variant,intron_variant
rs1004722383	intron_variant
rs1004743627	intron_variant
rs1004764037	intron_variant
rs1004771606	intron_variant,genic_upstream_transcript_variant
rs1004774947	genic_downstream_transcript_variant,intron_variant
rs1004826950	intron_variant
rs1004831614	genic_downstream_transcript_variant,intron_variant
rs1004880414	intron_variant,genic_upstream_transcript_variant
rs1004890065	intron_variant,genic_upstream_transcript_variant
rs1004894408	intron_variant
rs1004902398	genic_downstream_transcript_variant,intron_variant
rs1004923221	intron_variant
rs1004951980	downstream_transcript_variant,500B_downstream_variant
rs1004985867	intron_variant
rs1004998523	intron_variant,genic_upstream_transcript_variant
rs1005015974	intron_variant,genic_upstream_transcript_variant
rs1005050156	intron_variant,genic_upstream_transcript_variant
rs1005075293	intron_variant
rs1005101174	genic_downstream_transcript_variant,intron_variant
rs1005114126	intron_variant,genic_upstream_transcript_variant
rs1005147642	intron_variant,genic_upstream_transcript_variant
rs1005164438	intron_variant
rs1005165364	genic_downstream_transcript_variant,intron_variant
rs1005186384	genic_downstream_transcript_variant,intron_variant
rs1005218402	intron_variant,genic_upstream_transcript_variant
rs1005237724	genic_downstream_transcript_variant,intron_variant
rs1005299629	genic_downstream_transcript_variant,intron_variant
rs1005305956	intron_variant
rs1005311855	intron_variant,genic_upstream_transcript_variant
rs1005326196	intron_variant
rs1005340200	intron_variant
rs1005347455	intron_variant
rs1005352986	intron_variant
rs1005370380	intron_variant,genic_upstream_transcript_variant
rs1005411331	upstream_transcript_variant,intron_variant,2KB_upstream_variant,genic_upstream_transcript_variant
rs1005422014	intron_variant
rs1005443808	upstream_transcript_variant,intron_variant,5_prime_UTR_variant,genic_upstream_transcript_variant
rs1005490088	genic_downstream_transcript_variant,intron_variant
rs1005495774	intron_variant,genic_upstream_transcript_variant
rs1005508459	genic_downstream_transcript_variant,intron_variant
rs1005526171	intron_variant

rs1005531673	intron_variant
rs1005589987	intron_variant,genic_upstream_transcript_variant
rs1005619742	intron_variant,genic_upstream_transcript_variant
rs1005623053	intron_variant
rs1005633745	intron_variant,genic_upstream_transcript_variant
rs1005660390	upstream_transcript_variant,intron_variant,2KB_upstream_variant,genic_upstream_transcript_variant
rs1005689946	intron_variant,genic_upstream_transcript_variant
rs1005704149	intron_variant
rs1005732608	intron_variant
rs1005767028	genic_downstream_transcript_variant,intron_variant
rs1005777701	intron_variant
rs1005792628	genic_downstream_transcript_variant,intron_variant
rs1005806983	intron_variant
rs1005854219	intron_variant,genic_upstream_transcript_variant
rs1005864465	intron_variant
rs1005868316	intron_variant
rs1005902166	intron_variant,genic_upstream_transcript_variant
rs1005947737	3_prime_UTR_variant,genic_downstream_transcript_variant,non_coding_transcript_variant
rs1005948074	intron_variant
rs1005969221	intron_variant,genic_upstream_transcript_variant
rs1006015195	intron_variant,genic_upstream_transcript_variant
rs1006016023	intron_variant,genic_upstream_transcript_variant
rs1006028717	intron_variant
rs1006058983	intron_variant
rs1006088662	intron_variant
rs1006097274	genic_downstream_transcript_variant,intron_variant
rs1006128633	intron_variant,genic_upstream_transcript_variant
rs1006165985	genic_downstream_transcript_variant,intron_variant
rs1006182765	genic_downstream_transcript_variant,intron_variant
rs1006183787	intron_variant
rs1006198410	intron_variant
rs1006199277	intron_variant,genic_upstream_transcript_variant
rs1006213675	intron_variant,genic_upstream_transcript_variant
rs1006239899	genic_downstream_transcript_variant,intron_variant
rs1006304745	upstream_transcript_variant,intron_variant,2KB_upstream_variant,genic_upstream_transcript_variant
rs1006305997	intron_variant,genic_upstream_transcript_variant
rs1006317147	intron_variant
rs1006320975	genic_downstream_transcript_variant,intron_variant
rs1006337812	intron_variant,genic_upstream_transcript_variant
rs1006338708	upstream_transcript_variant,intron_variant,genic_upstream_transcript_variant
rs1006365524	intron_variant,genic_upstream_transcript_variant
rs1006399079	intron_variant
rs1006437066	intron_variant,genic_upstream_transcript_variant
rs1006469219	intron_variant,genic_upstream_transcript_variant
rs1006475498	intron_variant
rs1006549423	genic_downstream_transcript_variant,intron_variant
rs1006550291	intron_variant,genic_upstream_transcript_variant
rs1006564393	genic_downstream_transcript_variant,intron_variant
rs1006576286	intron_variant
rs1006620854	genic_downstream_transcript_variant,intron_variant
rs1006639540	genic_downstream_transcript_variant,intron_variant
rs1006656986	intron_variant
rs1006675246	intron_variant
rs1006684509	intron_variant,genic_upstream_transcript_variant
rs1006731611	intron_variant
rs1006768061	intron_variant
rs1006777000	intron_variant
rs1006780676	genic_downstream_transcript_variant,intron_variant
rs1006809830	upstream_transcript_variant,intron_variant,2KB_upstream_variant,genic_upstream_transcript_variant
rs1006822600	genic_downstream_transcript_variant,intron_variant
rs1006893041	intron_variant,genic_upstream_transcript_variant
rs1006899382	upstream_transcript_variant,intron_variant,2KB_upstream_variant,genic_upstream_transcript_variant
rs1006927925	intron_variant
rs1006943076	genic_downstream_transcript_variant,intron_variant
rs1006964028	intron_variant
rs1006976200	intron_variant,genic_upstream_transcript_variant
rs1007025034	intron_variant,genic_upstream_transcript_variant
rs1007045515	intron_variant,genic_upstream_transcript_variant
rs1007045733	genic_downstream_transcript_variant,intron_variant
rs1007063343	intron_variant
rs1007080391	genic_downstream_transcript_variant,intron_variant
rs1007093696	intron_variant,genic_upstream_transcript_variant
rs1007096431	genic_downstream_transcript_variant,intron_variant
rs1007151410	intron_variant,genic_upstream_transcript_variant

rs1007170430	intron_variant
rs1007175765	downstream_transcript_variant,500B_downstream_variant
rs1007188272	genic_downstream_transcript_variant,intron_variant
rs1007205533	intron_variant,genic_upstream_transcript_variant
rs1007246817	intron_variant,genic_upstream_transcript_variant
rs1007280889	intron_variant
rs1007282466	genic_downstream_transcript_variant,intron_variant
rs1007288564	intron_variant,genic_upstream_transcript_variant
rs1007296084	intron_variant
rs1007314554	intron_variant,genic_upstream_transcript_variant
rs1007347570	intron_variant
rs1007356441	genic_downstream_transcript_variant,intron_variant
rs1007359541	intron_variant
rs1007429124	intron_variant,genic_upstream_transcript_variant
rs1007459515	intron_variant,genic_upstream_transcript_variant
rs1007469103	intron_variant,genic_upstream_transcript_variant
rs1007514809	upstream_transcript_variant,intron_variant,genic_upstream_transcript_variant
rs1007519277	intron_variant
rs1007521229	upstream_transcript_variant,intron_variant,2KB_upstream_variant,genic_upstream_transcript_variant
rs1007522195	genic_downstream_transcript_variant,intron_variant
rs1007544555	intron_variant
rs1007572081	intron_variant,5_prime_UTR_variant,genic_upstream_transcript_variant,non_coding_transcript_variant
rs1007592174	genic_downstream_transcript_variant,intron_variant
rs1007598961	intron_variant
rs1007604481	intron_variant,genic_upstream_transcript_variant
rs1007619839	genic_downstream_transcript_variant,intron_variant
rs1007628450	intron_variant
rs1007640573	intron_variant,genic_upstream_transcript_variant
rs1007647379	intron_variant,genic_upstream_transcript_variant
rs1007674490	coding_sequence_variant,missense_variant,genic_downstream_transcript_variant,non_coding_transcript_variant
rs1007685362	intron_variant
rs1007716883	intron_variant,genic_upstream_transcript_variant
rs1007723869	intron_variant
rs1007749382	intron_variant,genic_upstream_transcript_variant
rs1007765666	intron_variant,genic_upstream_transcript_variant
rs1007767096	intron_variant
rs1007782313	intron_variant,genic_upstream_transcript_variant
rs1007825631	genic_downstream_transcript_variant,intron_variant
rs1007825793	intron_variant
rs1007839691	intron_variant,splice_acceptor_variant
rs1007875691	intron_variant
rs1007897070	intron_variant,genic_upstream_transcript_variant
rs1007897370	upstream_transcript_variant,intron_variant,2KB_upstream_variant,genic_upstream_transcript_variant
rs1007913995	intron_variant
rs1007946866	genic_downstream_transcript_variant,intron_variant
rs1007954344	intron_variant,genic_upstream_transcript_variant
rs1007967880	intron_variant,genic_upstream_transcript_variant
rs1008007147	intron_variant
rs1008061773	intron_variant,genic_upstream_transcript_variant
rs1008069096	intron_variant,genic_upstream_transcript_variant
rs1008083083	intron_variant
rs1008107821	genic_downstream_transcript_variant,intron_variant
rs1008195382	intron_variant,genic_upstream_transcript_variant
rs1008226325	intron_variant
rs1008278918	intron_variant,genic_upstream_transcript_variant
rs1008280497	intron_variant
rs1008305534	intron_variant
rs1008305909	intron_variant,genic_upstream_transcript_variant
rs1008336231	intron_variant
rs1008409555	intron_variant
rs1008434652	intron_variant,genic_upstream_transcript_variant
rs1008435047	intron_variant
rs1008445015	intron_variant
rs1008446120	intron_variant,genic_upstream_transcript_variant
rs1008484844	intron_variant
rs1008531717	intron_variant,genic_upstream_transcript_variant
rs1008564654	intron_variant
rs1008584552	intron_variant,genic_upstream_transcript_variant
rs1008659247	intron_variant,genic_upstream_transcript_variant
rs1008671931	intron_variant
rs1008699593	intron_variant
rs1008729647	intron_variant
rs1008766776	intron_variant,genic_upstream_transcript_variant
rs1008822572	intron_variant

rs1008830512	intron_variant,genic_upstream_transcript_variant
rs1008894100	intron_variant,genic_upstream_transcript_variant
rs1008903515	intron_variant
rs1008954443	intron_variant
rs1008974858	intron_variant,genic_upstream_transcript_variant
rs1008987633	intron_variant,genic_upstream_transcript_variant
rs1008998127	intron_variant
rs1009027458	genic_downstream_transcript_variant,intron_variant
rs1009043634	genic_downstream_transcript_variant,intron_variant
rs1009186700	intron_variant
rs1009187351	intron_variant
rs1009189369	intron_variant,genic_upstream_transcript_variant
rs1009223423	intron_variant,genic_upstream_transcript_variant
rs1009302062	intron_variant
rs1009331746	intron_variant,genic_upstream_transcript_variant
rs1009385009	intron_variant,genic_upstream_transcript_variant
rs1009385386	downstream_transcript_variant,intron_variant,genic_downstream_transcript_variant
rs1009407580	intron_variant
rs1009436489	intron_variant
rs1009473472	intron_variant
rs1009525172	intron_variant
rs1009552161	intron_variant,genic_upstream_transcript_variant
rs1009590181	genic_downstream_transcript_variant,intron_variant
rs1009604581	intron_variant
rs1009613235	intron_variant
rs1009644404	intron_variant,genic_upstream_transcript_variant
rs1009658335	intron_variant
rs1009690200	genic_downstream_transcript_variant,intron_variant
rs1009713083	intron_variant
rs1009725204	intron_variant
rs1009727787	intron_variant,genic_upstream_transcript_variant
rs1009743962	intron_variant
rs1009774931	3_prime_UTR_variant,genic_downstream_transcript_variant,non_coding_transcript_variant
rs1009778972	intron_variant
rs1009801217	intron_variant,genic_upstream_transcript_variant
rs1009808267	intron_variant
rs1009812855	intron_variant,genic_upstream_transcript_variant
rs1009826853	3_prime_UTR_variant,genic_downstream_transcript_variant,non_coding_transcript_variant
rs1009865381	intron_variant,genic_upstream_transcript_variant
rs1009937544	intron_variant
rs1009939602	intron_variant
rs1009950778	intron_variant
rs1009982159	genic_downstream_transcript_variant,intron_variant
rs1010018621	intron_variant
rs1010060953	genic_downstream_transcript_variant,intron_variant
rs1010077737	intron_variant
rs1010078383	3_prime_UTR_variant,genic_downstream_transcript_variant,non_coding_transcript_variant
rs1010119236	intron_variant
rs1010129081	intron_variant
rs1010136649	intron_variant,genic_upstream_transcript_variant
rs1010210019	intron_variant
rs1010215350	intron_variant
rs1010238751	intron_variant,genic_upstream_transcript_variant
rs1010246691	genic_downstream_transcript_variant,intron_variant
rs1010259602	intron_variant
rs1010285734	intron_variant,genic_upstream_transcript_variant
rs1010298466	intron_variant,genic_upstream_transcript_variant
rs1010327477	genic_downstream_transcript_variant,intron_variant
rs1010330435	genic_downstream_transcript_variant,intron_variant
rs1010405928	intron_variant,genic_upstream_transcript_variant
rs1010477285	upstream_transcript_variant,intron_variant,2KB_upstream_variant,genic_upstream_transcript_variant
rs1010525134	genic_downstream_transcript_variant,intron_variant
rs1010542688	upstream_transcript_variant,intron_variant,2KB_upstream_variant,genic_upstream_transcript_variant
rs1010587853	intron_variant
rs1010604562	intron_variant
rs1010622850	intron_variant,genic_upstream_transcript_variant
rs1010624481	genic_downstream_transcript_variant,intron_variant
rs1010631352	intron_variant
rs1010662415	intron_variant
rs1010714001	genic_downstream_transcript_variant,intron_variant
rs1010729365	intron_variant,genic_upstream_transcript_variant
rs1010782918	intron_variant
rs1010786777	genic_downstream_transcript_variant,intron_variant
rs1010810717	intron_variant

rs1010859067	intron_variant,genic_upstream_transcript_variant
rs1010867326	intron_variant,genic_upstream_transcript_variant
rs1010890664	intron_variant
rs1010926765	upstream_transcript_variant,intron_variant,2KB_upstream_variant,genic_upstream_transcript_variant
rs1010948901	upstream_transcript_variant,intron_variant,2KB_upstream_variant,genic_upstream_transcript_variant
rs1010969420	intron_variant,genic_upstream_transcript_variant
rs1010993047	genic_downstream_transcript_variant,intron_variant
rs1011038016	intron_variant
rs1011045998	intron_variant
rs1011067899	intron_variant,genic_upstream_transcript_variant
rs1011074196	genic_downstream_transcript_variant,intron_variant
rs1011093720	intron_variant,genic_upstream_transcript_variant
rs1011112531	intron_variant,genic_upstream_transcript_variant
rs1011122380	intron_variant,genic_upstream_transcript_variant
rs1011153901	intron_variant,genic_upstream_transcript_variant
rs1011205779	upstream_transcript_variant,intron_variant,genic_upstream_transcript_variant
rs1011208699	intron_variant
rs1011211075	genic_downstream_transcript_variant,intron_variant
rs1011237585	intron_variant,genic_upstream_transcript_variant
rs1011253323	upstream_transcript_variant,intron_variant,2KB_upstream_variant,genic_upstream_transcript_variant
rs1011272926	genic_downstream_transcript_variant,intron_variant
rs1011284318	upstream_transcript_variant,intron_variant,2KB_upstream_variant,genic_upstream_transcript_variant
rs1011295144	genic_downstream_transcript_variant,intron_variant
rs1011298601	intron_variant,genic_upstream_transcript_variant
rs1011309216	intron_variant
rs1011324394	intron_variant,genic_upstream_transcript_variant
rs1011325367	genic_downstream_transcript_variant,intron_variant
rs1011371258	intron_variant,genic_upstream_transcript_variant
rs1011390092	intron_variant
rs1011420021	intron_variant
rs1011442851	intron_variant,genic_upstream_transcript_variant
rs1011523237	upstream_transcript_variant,intron_variant,2KB_upstream_variant,genic_upstream_transcript_variant
rs1011530980	intron_variant
rs1011555181	intron_variant
rs1011614502	intron_variant
rs1011621519	genic_downstream_transcript_variant,intron_variant
rs1011647384	intron_variant
rs1011683805	genic_downstream_transcript_variant,intron_variant
rs1011711629	intron_variant
rs1011725698	intron_variant,genic_upstream_transcript_variant
rs1011731926	genic_downstream_transcript_variant,intron_variant
rs1011734549	intron_variant,genic_upstream_transcript_variant
rs1011754674	intron_variant
rs1011797103	intron_variant
rs1011874013	intron_variant,genic_upstream_transcript_variant
rs1011875969	genic_downstream_transcript_variant,intron_variant
rs1011908053	upstream_transcript_variant,intron_variant,2KB_upstream_variant,genic_upstream_transcript_variant
rs1011909157	intron_variant
rs1011946106	genic_downstream_transcript_variant,intron_variant
rs1011976405	intron_variant
rs1011983544	genic_downstream_transcript_variant,intron_variant
rs1012018203	genic_downstream_transcript_variant,intron_variant
rs1012135004	genic_downstream_transcript_variant,intron_variant
rs1012138243	genic_downstream_transcript_variant,intron_variant
rs1012176880	intron_variant
rs1012207294	upstream_transcript_variant,intron_variant,2KB_upstream_variant,genic_upstream_transcript_variant
rs1012209640	intron_variant
rs1012228300	intron_variant
rs1012287470	intron_variant,genic_upstream_transcript_variant
rs1012295473	intron_variant,genic_upstream_transcript_variant
rs1012328532	intron_variant,genic_upstream_transcript_variant
rs1012342113	intron_variant,genic_upstream_transcript_variant
rs1012345356	intron_variant
rs1012417084	intron_variant,genic_upstream_transcript_variant
rs1012428580	intron_variant,genic_upstream_transcript_variant
rs1012440618	intron_variant
rs1012441677	intron_variant,genic_upstream_transcript_variant
rs1012460652	intron_variant,genic_upstream_transcript_variant
rs1012480550	intron_variant
rs1012523074	intron_variant
rs1012551357	genic_downstream_transcript_variant,intron_variant
rs1012578963	upstream_transcript_variant,intron_variant,2KB_upstream_variant,genic_upstream_transcript_variant
rs1012579923	genic_downstream_transcript_variant,intron_variant
rs1012601175	intron_variant,genic_upstream_transcript_variant

rs1012669276	intron_variant,genic_upstream_transcript_variant
rs1012701223	intron_variant
rs1012732027	intron_variant
rs1012750996	intron_variant,genic_upstream_transcript_variant
rs1012754969	intron_variant
rs1012758720	intron_variant,genic_upstream_transcript_variant
rs1012770452	intron_variant,genic_upstream_transcript_variant
rs1012786753	intron_variant,genic_upstream_transcript_variant
rs1012844792	intron_variant,genic_upstream_transcript_variant
rs1012862828	intron_variant
rs1012918002	2KB_upstream_variant,intron_variant,genic_upstream_transcript_variant,upstream_transcript_variant
rs1012918847	intron_variant,genic_upstream_transcript_variant
rs1012948048	intron_variant
rs1012965243	intron_variant
rs1012976656	intron_variant,genic_upstream_transcript_variant
rs1012993594	genic_downstream_transcript_variant,intron_variant
rs1013015234	intron_variant,genic_upstream_transcript_variant
rs1013058082	genic_downstream_transcript_variant,intron_variant
rs1013077332	intron_variant,genic_upstream_transcript_variant
rs1013149889	intron_variant
rs1013155391	intron_variant,genic_upstream_transcript_variant,upstream_transcript_variant
rs1013178489	intron_variant,genic_upstream_transcript_variant
rs1013226549	genic_downstream_transcript_variant,intron_variant
rs1013283562	intron_variant
rs1013300617	genic_downstream_transcript_variant,intron_variant
rs1013302959	intron_variant,genic_upstream_transcript_variant
rs1013329027	intron_variant,genic_upstream_transcript_variant,upstream_transcript_variant
rs1013349382	genic_downstream_transcript_variant,intron_variant
rs1013371212	genic_downstream_transcript_variant,intron_variant
rs1013400368	intron_variant
rs1013437649	intron_variant,genic_upstream_transcript_variant
rs1013443959	intron_variant
rs1013445762	intron_variant,genic_upstream_transcript_variant
rs1013484268	genic_downstream_transcript_variant,intron_variant
rs1013501515	intron_variant,genic_upstream_transcript_variant
rs1013511279	intron_variant,genic_upstream_transcript_variant
rs1013528843	intron_variant,genic_upstream_transcript_variant
rs1013543871	intron_variant
rs1013546347	intron_variant,genic_upstream_transcript_variant
rs1013610820	genic_downstream_transcript_variant,intron_variant
rs1013651208	intron_variant
rs1013653440	intron_variant,genic_upstream_transcript_variant
rs1013707866	intron_variant
rs1013744219	genic_downstream_transcript_variant,intron_variant
rs1013790639	genic_downstream_transcript_variant,intron_variant
rs1013805784	3_prime_UTR_variant,intron_variant
rs1013810355	intron_variant,genic_upstream_transcript_variant
rs1013831374	intron_variant,genic_upstream_transcript_variant
rs1013850503	genic_downstream_transcript_variant,intron_variant
rs1013861503	intron_variant,5_prime_UTR_variant
rs1013896285	intron_variant
rs1013933649	intron_variant
rs1013940744	genic_downstream_transcript_variant,intron_variant
rs1013997008	genic_downstream_transcript_variant,intron_variant
rs1013997730	genic_downstream_transcript_variant,intron_variant
rs1014002619	intron_variant,genic_upstream_transcript_variant
rs1014002694	intron_variant
rs1014023504	intron_variant,genic_upstream_transcript_variant
rs1014026471	non_coding_transcript_variant,genic_downstream_transcript_variant,3_prime_UTR_variant
rs1014098522	intron_variant,genic_upstream_transcript_variant
rs1014117262	intron_variant,genic_upstream_transcript_variant
rs1014142840	genic_downstream_transcript_variant,intron_variant
rs1014209126	genic_downstream_transcript_variant,intron_variant
rs1014223811	intron_variant
rs1014287842	genic_downstream_transcript_variant,intron_variant
rs1014293126	intron_variant,genic_upstream_transcript_variant
rs1014295864	intron_variant
rs1014417199	intron_variant,5_prime_UTR_variant
rs1014428346	genic_downstream_transcript_variant,intron_variant
rs1014451744	intron_variant
rs1014473192	intron_variant,genic_upstream_transcript_variant
rs1014488051	intron_variant
rs1014553838	intron_variant,genic_upstream_transcript_variant
rs1014567062	intron_variant

rs1014639507	intron_variant,genic_upstream_transcript_variant
rs1014648144	intron_variant
rs1014656720	intron_variant,genic_upstream_transcript_variant
rs1014668446	intron_variant,genic_upstream_transcript_variant
rs1014671187	intron_variant,genic_upstream_transcript_variant
rs1014709254	intron_variant
rs1014724224	intron_variant,genic_upstream_transcript_variant
rs1014726135	genic_downstream_transcript_variant,intron_variant
rs1014729224	intron_variant
rs1014766607	intron_variant,genic_upstream_transcript_variant
rs1014790136	intron_variant,genic_upstream_transcript_variant
rs1014791960	intron_variant
rs1014797297	genic_downstream_transcript_variant,intron_variant
rs1014804709	intron_variant
rs1014837441	intron_variant
rs1014838425	intron_variant
rs1014877307	genic_downstream_transcript_variant,intron_variant
rs1014885675	genic_downstream_transcript_variant,intron_variant
rs1014903042	intron_variant,genic_upstream_transcript_variant
rs1014919569	2KB_upstream_variant,intron_variant,genic_upstream_transcript_variant,upstream_transcript_variant
rs1014950000	genic_downstream_transcript_variant,intron_variant
rs1015049531	intron_variant,genic_upstream_transcript_variant
rs1015050666	intron_variant
rs1015066987	intron_variant,genic_upstream_transcript_variant
rs1015075695	2KB_upstream_variant,intron_variant,genic_upstream_transcript_variant,upstream_transcript_variant
rs1015091709	intron_variant
rs1015113445	intron_variant,genic_upstream_transcript_variant
rs1015119673	intron_variant,genic_upstream_transcript_variant
rs1015127069	intron_variant
rs1015155326	intron_variant
rs1015157989	intron_variant
rs1015215204	intron_variant,genic_upstream_transcript_variant
rs1015217708	genic_downstream_transcript_variant,intron_variant
rs1015238192	intron_variant,genic_upstream_transcript_variant
rs1015269878	intron_variant
rs1015286930	genic_downstream_transcript_variant,intron_variant,downstream_transcript_variant
rs1015332898	intron_variant,genic_upstream_transcript_variant
rs1015342126	intron_variant,genic_upstream_transcript_variant
rs1015369120	intron_variant
rs1015385973	intron_variant,genic_upstream_transcript_variant
rs1015412889	2KB_upstream_variant,intron_variant,genic_upstream_transcript_variant,upstream_transcript_variant
rs1015425751	2KB_upstream_variant,intron_variant,genic_upstream_transcript_variant,upstream_transcript_variant
rs1015435163	intron_variant
rs1015443914	upstream_transcript_variant,intron_variant,genic_upstream_transcript_variant,5_prime_UTR_variant
rs1015464050	intron_variant
rs1015468031	intron_variant,genic_upstream_transcript_variant
rs1015496542	intron_variant
rs1015512446	intron_variant
rs1015513836	2KB_upstream_variant,intron_variant,genic_upstream_transcript_variant,upstream_transcript_variant
rs1015518298	intron_variant
rs1015544062	genic_downstream_transcript_variant,intron_variant
rs1015575369	genic_downstream_transcript_variant,intron_variant
rs1015577199	intron_variant
rs1015592350	intron_variant,genic_upstream_transcript_variant
rs1015600362	genic_downstream_transcript_variant,intron_variant
rs1015642082	2KB_upstream_variant,intron_variant,genic_upstream_transcript_variant,upstream_transcript_variant
rs1015715830	intron_variant
rs1015758869	genic_downstream_transcript_variant,intron_variant
rs1015768696	intron_variant
rs1015791819	intron_variant,genic_upstream_transcript_variant
rs1015796520	intron_variant,genic_upstream_transcript_variant
rs1015800142	intron_variant
rs1015805812	intron_variant,genic_upstream_transcript_variant
rs1015831550	intron_variant,genic_upstream_transcript_variant
rs1015862695	intron_variant,genic_upstream_transcript_variant
rs1015888778	genic_downstream_transcript_variant,intron_variant,downstream_transcript_variant
rs1015906422	intron_variant,genic_upstream_transcript_variant
rs1015985700	genic_downstream_transcript_variant,intron_variant
rs1015990447	intron_variant
rs1016016129	intron_variant,genic_upstream_transcript_variant
rs1016078413	intron_variant,genic_upstream_transcript_variant
rs1016098351	intron_variant
rs1016111321	intron_variant
rs1016111783	intron_variant,genic_upstream_transcript_variant

rs1016128674	intron_variant
rs1016167230	intron_variant,genic_upstream_transcript_variant
rs1016184879	intron_variant,genic_upstream_transcript_variant
rs1016227377	intron_variant,genic_upstream_transcript_variant
rs1016244343	intron_variant,genic_upstream_transcript_variant,upstream_transcript_variant
rs1016251516	genic_downstream_transcript_variant,intron_variant
rs1016255081	intron_variant
rs1016277577	intron_variant
rs1016335395	2KB_upstream_variant,intron_variant,genic_upstream_transcript_variant,upstream_transcript_variant
rs1016339088	intron_variant,genic_upstream_transcript_variant,upstream_transcript_variant
rs1016390434	intron_variant,genic_upstream_transcript_variant,upstream_transcript_variant
rs1016392528	intron_variant
rs1016412526	genic_downstream_transcript_variant,intron_variant
rs1016432368	genic_downstream_transcript_variant,intron_variant
rs1016464829	intron_variant
rs1016468450	intron_variant,genic_upstream_transcript_variant
rs1016480988	intron_variant,genic_upstream_transcript_variant
rs1016493359	genic_downstream_transcript_variant,intron_variant
rs1016515883	intron_variant
rs1016522847	genic_downstream_transcript_variant,intron_variant
rs1016552377	genic_downstream_transcript_variant,intron_variant
rs1016591861	genic_downstream_transcript_variant,intron_variant
rs1016611181	intron_variant,genic_upstream_transcript_variant
rs1016642609	genic_downstream_transcript_variant,intron_variant
rs1016664819	intron_variant,genic_upstream_transcript_variant
rs1016675174	intron_variant
rs1016723908	intron_variant
rs1016731598	intron_variant
rs1016745629	intron_variant,genic_upstream_transcript_variant
rs1016753913	intron_variant,genic_upstream_transcript_variant
rs1016786554	intron_variant,genic_upstream_transcript_variant
rs1016795790	genic_downstream_transcript_variant,intron_variant
rs1016802710	intron_variant
rs1016858824	genic_downstream_transcript_variant,intron_variant
rs1016892011	intron_variant,genic_upstream_transcript_variant
rs1016913058	intron_variant,genic_upstream_transcript_variant
rs1016956440	intron_variant
rs1016970788	intron_variant,genic_upstream_transcript_variant
rs1016983216	intron_variant,genic_upstream_transcript_variant
rs1017024385	intron_variant
rs1017062541	intron_variant
rs1017123880	intron_variant,genic_upstream_transcript_variant
rs1017141910	intron_variant
rs1017150372	genic_downstream_transcript_variant,intron_variant
rs1017176587	genic_downstream_transcript_variant,intron_variant
rs1017187747	intron_variant,genic_upstream_transcript_variant
rs1017212598	intron_variant,genic_upstream_transcript_variant
rs1017215136	intron_variant
rs1017241732	intron_variant,genic_upstream_transcript_variant
rs1017261560	intron_variant
rs1017275256	intron_variant
rs1017309811	intron_variant
rs1017344874	intron_variant
rs1017346121	intron_variant,genic_upstream_transcript_variant
rs1017356179	intron_variant
rs1017389964	intron_variant
rs1017402995	intron_variant,genic_upstream_transcript_variant
rs1017426391	intron_variant
rs1017482688	intron_variant,genic_upstream_transcript_variant
rs1017493599	intron_variant,genic_upstream_transcript_variant
rs1017536922	upstream_transcript_variant,intron_variant,genic_upstream_transcript_variant,5_prime_UTR_variant
rs1017548698	intron_variant
rs1017581079	genic_downstream_transcript_variant,intron_variant
rs1017583272	intron_variant,genic_upstream_transcript_variant
rs1017594681	genic_downstream_transcript_variant,intron_variant
rs1017662310	intron_variant
rs1017698232	genic_downstream_transcript_variant,intron_variant
rs1017707061	genic_downstream_transcript_variant,intron_variant
rs1017727184	intron_variant,genic_upstream_transcript_variant
rs1017738621	genic_downstream_transcript_variant,intron_variant
rs1017748152	genic_downstream_transcript_variant,intron_variant
rs1017778524	intron_variant
rs1017795889	intron_variant,genic_upstream_transcript_variant,upstream_transcript_variant
rs1017801714	intron_variant,genic_upstream_transcript_variant

rs1017820376	intron_variant,genic_upstream_transcript_variant
rs1017848093	intron_variant,genic_upstream_transcript_variant,upstream_transcript_variant
rs1017865638	intron_variant,genic_upstream_transcript_variant
rs1017877653	intron_variant,genic_upstream_transcript_variant
rs1017896853	intron_variant,genic_upstream_transcript_variant
rs1017901909	intron_variant,genic_upstream_transcript_variant
rs1017944490	intron_variant,genic_upstream_transcript_variant
rs1017950011	intron_variant
rs1017959550	intron_variant
rs1018036240	intron_variant,genic_upstream_transcript_variant
rs1018043705	genic_downstream_transcript_variant,intron_variant
rs1018049582	intron_variant,genic_upstream_transcript_variant
rs1018057290	intron_variant
rs1018082972	genic_downstream_transcript_variant,intron_variant
rs1018101333	intron_variant
rs1018142861	intron_variant
rs1018143299	intron_variant,genic_upstream_transcript_variant
rs1018182216	intron_variant,genic_upstream_transcript_variant
rs1018224017	intron_variant
rs1018226445	intron_variant
rs1018239517	intron_variant
rs1018263301	intron_variant,genic_upstream_transcript_variant
rs1018273568	intron_variant,genic_upstream_transcript_variant
rs1018283471	intron_variant
rs1018297828	genic_downstream_transcript_variant,intron_variant
rs1018312371	intron_variant
rs1018334455	intron_variant
rs1018357996	genic_downstream_transcript_variant,intron_variant
rs1018371620	genic_downstream_transcript_variant,intron_variant
rs1018371815	intron_variant,genic_upstream_transcript_variant
rs1018392429	intron_variant,genic_upstream_transcript_variant
rs1018421827	intron_variant,genic_upstream_transcript_variant
rs1018478118	intron_variant,genic_upstream_transcript_variant
rs1018481785	intron_variant
rs1018562270	genic_downstream_transcript_variant,intron_variant
rs1018565359	intron_variant
rs1018590660	intron_variant,genic_upstream_transcript_variant
rs1018619569	intron_variant
rs1018736053	intron_variant
rs1018753719	intron_variant,genic_upstream_transcript_variant
rs1018792490	intron_variant,genic_upstream_transcript_variant
rs1018792989	genic_downstream_transcript_variant,intron_variant
rs1018807799	intron_variant,genic_upstream_transcript_variant
rs1018824734	intron_variant,genic_upstream_transcript_variant
rs1018844529	intron_variant,genic_upstream_transcript_variant
rs1018892619	intron_variant,genic_upstream_transcript_variant
rs1018909299	intron_variant
rs1018999843	intron_variant,non_coding_transcript_variant,genic_upstream_transcript_variant,missense_variant,coding_sequence_variant
rs1019006787	intron_variant
rs1019008057	genic_downstream_transcript_variant,intron_variant
rs1019021493	intron_variant,genic_upstream_transcript_variant
rs1019031678	intron_variant
rs1019082959	genic_downstream_transcript_variant,intron_variant
rs1019119070	intron_variant,genic_upstream_transcript_variant
rs1019145623	intron_variant
rs1019165385	genic_downstream_transcript_variant,intron_variant
rs1019193356	intron_variant,genic_upstream_transcript_variant
rs1019199577	intron_variant,genic_upstream_transcript_variant
rs1019218013	intron_variant
rs1019265687	intron_variant,genic_upstream_transcript_variant
rs1019321897	intron_variant
rs1019327022	intron_variant
rs1019332741	intron_variant
rs1019338968	intron_variant,genic_upstream_transcript_variant
rs1019347156	genic_downstream_transcript_variant,intron_variant
rs1019347841	genic_downstream_transcript_variant,intron_variant
rs1019393038	genic_downstream_transcript_variant,intron_variant
rs1019409744	intron_variant
rs1019427156	genic_downstream_transcript_variant,intron_variant
rs1019441430	intron_variant,genic_upstream_transcript_variant
rs1019482662	intron_variant
rs1019499355	genic_downstream_transcript_variant,intron_variant
rs1019507641	intron_variant,genic_upstream_transcript_variant
rs1019553113	genic_downstream_transcript_variant,intron_variant

rs1019573048	genic_downstream_transcript_variant,intron_variant
rs1019587877	intron_variant,genic_upstream_transcript_variant
rs1019679179	intron_variant
rs1019697265	intron_variant,genic_upstream_transcript_variant
rs1019705469	genic_downstream_transcript_variant,intron_variant
rs1019718599	intron_variant
rs1019736277	intron_variant,genic_upstream_transcript_variant
rs1019756093	intron_variant,genic_upstream_transcript_variant
rs1019803047	intron_variant
rs1019803902	intron_variant
rs1019833265	intron_variant
rs1019835097	intron_variant
rs1019867927	intron_variant
rs1019891911	intron_variant,genic_upstream_transcript_variant
rs1019924096	intron_variant,genic_upstream_transcript_variant
rs1019939145	intron_variant
rs1019956192	intron_variant,genic_upstream_transcript_variant
rs1019956621	genic_downstream_transcript_variant,intron_variant
rs1020033459	intron_variant,genic_upstream_transcript_variant
rs1020059818	intron_variant,genic_upstream_transcript_variant
rs1020100182	non_coding_transcript_variant,genic_downstream_transcript_variant,3_prime_UTR_variant
rs1020106969	intron_variant,genic_upstream_transcript_variant
rs1020132519	genic_downstream_transcript_variant,intron_variant
rs1020133459	intron_variant,genic_upstream_transcript_variant
rs1020173809	non_coding_transcript_variant,genic_downstream_transcript_variant,3_prime_UTR_variant
rs1020223984	intron_variant
rs1020266851	intron_variant
rs1020293984	intron_variant,genic_upstream_transcript_variant
rs1020314429	intron_variant,genic_upstream_transcript_variant
rs1020319819	intron_variant
rs1020321551	genic_downstream_transcript_variant,intron_variant
rs1020355619	genic_downstream_transcript_variant,intron_variant
rs1020377724	intron_variant
rs1020378372	intron_variant,genic_upstream_transcript_variant
rs1020398460	genic_downstream_transcript_variant,intron_variant
rs1020408336	genic_downstream_transcript_variant,intron_variant
rs1020418470	intron_variant,genic_upstream_transcript_variant
rs1020501108	genic_downstream_transcript_variant,intron_variant
rs1020508714	genic_downstream_transcript_variant,intron_variant
rs1020525889	intron_variant
rs1020531083	2KB_upstream_variant,intron_variant,genic_upstream_transcript_variant,upstream_transcript_variant
rs1020549305	intron_variant
rs1020561765	intron_variant
rs1020593559	genic_downstream_transcript_variant,intron_variant
rs1020603722	intron_variant
rs1020652423	2KB_upstream_variant,intron_variant,genic_upstream_transcript_variant,upstream_transcript_variant
rs1020661903	intron_variant
rs1020666227	genic_downstream_transcript_variant,intron_variant
rs1020668739	intron_variant,genic_upstream_transcript_variant
rs1020730410	intron_variant,genic_upstream_transcript_variant
rs1020767289	intron_variant
rs1020774340	genic_downstream_transcript_variant,intron_variant
rs1020783824	2KB_upstream_variant,intron_variant,genic_upstream_transcript_variant,upstream_transcript_variant
rs1020801171	genic_downstream_transcript_variant,intron_variant
rs1020814462	genic_downstream_transcript_variant,intron_variant
rs1020866228	genic_downstream_transcript_variant,intron_variant
rs1020890008	intron_variant,genic_upstream_transcript_variant
rs1020903608	intron_variant
rs1020944432	intron_variant
rs1020979562	genic_downstream_transcript_variant,intron_variant
rs1021027951	intron_variant
rs1021034173	2KB_upstream_variant,intron_variant,genic_upstream_transcript_variant,upstream_transcript_variant
rs1021038709	intron_variant,genic_upstream_transcript_variant
rs1021044350	intron_variant,genic_upstream_transcript_variant
rs1021046465	genic_downstream_transcript_variant,intron_variant
rs1021064672	intron_variant,genic_upstream_transcript_variant
rs1021100343	genic_downstream_transcript_variant,intron_variant
rs1021143894	intron_variant,genic_upstream_transcript_variant
rs1021158996	intron_variant,genic_upstream_transcript_variant
rs1021175524	intron_variant,genic_upstream_transcript_variant
rs1021196224	intron_variant,genic_upstream_transcript_variant
rs1021231886	intron_variant,genic_upstream_transcript_variant
rs1021241041	intron_variant
rs1021255153	2KB_upstream_variant,intron_variant,genic_upstream_transcript_variant,upstream_transcript_variant

rs1021274644	intron_variant
rs1021274868	intron_variant
rs1021309404	2KB_upstream_variant,intron_variant,genic_upstream_transcript_variant,upstream_transcript_variant
rs1021320595	intron_variant,genic_upstream_transcript_variant
rs1021360436	intron_variant
rs1021388112	intron_variant,genic_upstream_transcript_variant
rs1021391808	intron_variant
rs1021495272	intron_variant
rs1021512157	intron_variant
rs1021522920	intron_variant,genic_upstream_transcript_variant
rs1021574004	intron_variant
rs1021579711	intron_variant
rs1021585880	non_coding_transcript_variant,intron_variant,genic_upstream_transcript_variant,5_prime_UTR_variant
rs1021607286	intron_variant,genic_upstream_transcript_variant
rs1021607765	intron_variant
rs1021665656	intron_variant
rs1021670765	genic_downstream_transcript_variant,intron_variant
rs1021742179	intron_variant
rs1021750680	intron_variant,genic_upstream_transcript_variant
rs1021827033	intron_variant
rs1021846020	intron_variant,genic_upstream_transcript_variant
rs1021852710	intron_variant
rs1021892901	intron_variant
rs1021924709	intron_variant
rs1021944753	intron_variant
rs1021985034	intron_variant,genic_upstream_transcript_variant
rs1022059664	genic_downstream_transcript_variant,intron_variant
rs1022080072	intron_variant
rs1022102008	intron_variant
rs1022113954	genic_downstream_transcript_variant,intron_variant
rs1022129005	intron_variant,genic_upstream_transcript_variant
rs1022160287	intron_variant,genic_upstream_transcript_variant
rs1022160614	intron_variant
rs1022174314	intron_variant
rs1022226566	intron_variant
rs1022304429	genic_downstream_transcript_variant,intron_variant
rs1022324467	genic_downstream_transcript_variant,intron_variant
rs1022335309	intron_variant
rs1022363387	genic_downstream_transcript_variant,intron_variant
rs1022378844	intron_variant,genic_upstream_transcript_variant
rs1022402432	intron_variant,genic_upstream_transcript_variant
rs1022406518	intron_variant
rs1022409363	intron_variant,genic_upstream_transcript_variant
rs1022434579	genic_downstream_transcript_variant,intron_variant
rs1022439911	genic_downstream_transcript_variant,intron_variant
rs1022443102	intron_variant,genic_upstream_transcript_variant
rs1022506386	genic_downstream_transcript_variant,intron_variant
rs1022536623	intron_variant,genic_upstream_transcript_variant
rs1022574513	intron_variant
rs1022583134	intron_variant
rs1022617901	genic_downstream_transcript_variant,intron_variant
rs1022649801	intron_variant
rs1022669595	intron_variant
rs1022671692	genic_downstream_transcript_variant,intron_variant
rs1022692471	genic_downstream_transcript_variant,intron_variant
rs1022695914	intron_variant,genic_upstream_transcript_variant
rs1022715198	intron_variant
rs1022778639	intron_variant,genic_upstream_transcript_variant,upstream_transcript_variant
rs1022788036	intron_variant
rs1022800185	intron_variant
rs1022818610	coding_sequence_variant,non_coding_transcript_variant,genic_downstream_transcript_variant,synonymous_variant
rs1022869188	intron_variant,genic_upstream_transcript_variant
rs1022870621	intron_variant
rs1022873938	intron_variant
rs1022896086	intron_variant,genic_upstream_transcript_variant
rs1022920930	2KB_upstream_variant,intron_variant,genic_upstream_transcript_variant,upstream_transcript_variant
rs1022939447	2KB_upstream_variant,intron_variant,genic_upstream_transcript_variant,upstream_transcript_variant
rs1022956475	genic_downstream_transcript_variant,intron_variant
rs1022963538	intron_variant,genic_upstream_transcript_variant
rs1022972035	intron_variant,genic_upstream_transcript_variant
rs1022985983	genic_downstream_transcript_variant,intron_variant
rs1023012040	intron_variant,genic_upstream_transcript_variant
rs1023025129	intron_variant
rs1023040748	intron_variant

rs1023060962	intron_variant
rs1023216992	intron_variant,genic_upstream_transcript_variant
rs1023220530	genic_downstream_transcript_variant,intron_variant
rs1023236159	intron_variant,genic_upstream_transcript_variant
rs1023242949	intron_variant
rs1023255093	intron_variant,genic_upstream_transcript_variant
rs1023340526	intron_variant
rs1023341782	intron_variant,genic_upstream_transcript_variant
rs1023351017	intron_variant,genic_upstream_transcript_variant
rs1023359328	intron_variant
rs1023430723	genic_downstream_transcript_variant,intron_variant
rs1023480123	intron_variant,genic_upstream_transcript_variant
rs1023501288	intron_variant
rs1023514985	genic_downstream_transcript_variant,intron_variant
rs1023535758	intron_variant
rs1023548602	intron_variant,genic_upstream_transcript_variant
rs1023557508	intron_variant,genic_upstream_transcript_variant
rs1023564119	intron_variant,genic_upstream_transcript_variant
rs1023564610	genic_downstream_transcript_variant,intron_variant
rs1023569732	intron_variant,genic_upstream_transcript_variant
rs1023576548	intron_variant
rs1023621074	intron_variant,genic_upstream_transcript_variant
rs1023635171	intron_variant,genic_upstream_transcript_variant
rs1023653319	genic_downstream_transcript_variant,intron_variant
rs1023654209	genic_downstream_transcript_variant,intron_variant
rs1023715576	intron_variant,genic_upstream_transcript_variant
rs1023731875	intron_variant,genic_upstream_transcript_variant,upstream_transcript_variant
rs1023751733	intron_variant,genic_upstream_transcript_variant
rs1023769275	intron_variant,genic_upstream_transcript_variant
rs1023771936	intron_variant
rs1023825323	intron_variant,genic_upstream_transcript_variant
rs1023836942	intron_variant
rs1023854899	intron_variant,genic_upstream_transcript_variant
rs1023919125	intron_variant,genic_upstream_transcript_variant
rs1023923371	genic_downstream_transcript_variant,intron_variant
rs1023932777	intron_variant
rs1023947279	intron_variant,genic_upstream_transcript_variant
rs1024001694	intron_variant
rs1024040421	intron_variant
rs1024054024	intron_variant,genic_upstream_transcript_variant
rs1024091351	intron_variant,genic_upstream_transcript_variant
rs1024108081	non_coding_transcript_variant,genic_downstream_transcript_variant,3_prime_UTR_variant
rs1024112887	intron_variant
rs1024130218	intron_variant,genic_upstream_transcript_variant
rs1024149574	intron_variant,genic_upstream_transcript_variant
rs1024224075	intron_variant,genic_upstream_transcript_variant
rs1024240925	intron_variant,genic_upstream_transcript_variant
rs1024249903	intron_variant
rs1024335209	intron_variant
rs1024344425	intron_variant,genic_upstream_transcript_variant
rs1024370645	intron_variant,genic_upstream_transcript_variant
rs1024398461	intron_variant
rs1024403762	intron_variant,genic_upstream_transcript_variant
rs1024405857	intron_variant
rs1024426747	intron_variant
rs1024461805	2KB_upstream_variant,intron_variant,genic_upstream_transcript_variant,upstream_transcript_variant
rs1024496839	intron_variant,genic_upstream_transcript_variant
rs1024506023	coding_sequence_variant,non_coding_transcript_variant,missense_variant
rs1024554098	intron_variant,genic_upstream_transcript_variant
rs1024596023	intron_variant
rs1024627767	intron_variant,genic_upstream_transcript_variant
rs1024644361	intron_variant,genic_upstream_transcript_variant
rs1024646460	intron_variant
rs1024659888	non_coding_transcript_variant,genic_downstream_transcript_variant,3_prime_UTR_variant
rs1024667425	intron_variant
rs1024727952	intron_variant
rs1024740436	intron_variant
rs1024744238	genic_upstream_transcript_variant,intron_variant
rs1024746681	intron_variant,genic_downstream_transcript_variant,downstream_transcript_variant
rs1024758521	genic_upstream_transcript_variant,intron_variant,upstream_transcript_variant
rs1024814400	intron_variant,3_prime_UTR_variant
rs1024831931	intron_variant
rs1024835463	genic_upstream_transcript_variant,intron_variant
rs1024838641	intron_variant

rs1024894550	intron_variant
rs1024898247	intron_variant,genic_downstream_transcript_variant
rs1024932344	genic_upstream_transcript_variant,intron_variant
rs1024933536	intron_variant
rs1024970154	genic_upstream_transcript_variant,intron_variant
rs1024971214	intron_variant,genic_downstream_transcript_variant
rs1024977874	intron_variant
rs1025018256	genic_upstream_transcript_variant,intron_variant
rs1025025147	intron_variant,genic_downstream_transcript_variant
rs1025133646	intron_variant
rs1025152194	genic_upstream_transcript_variant,intron_variant
rs1025152757	intron_variant
rs1025178872	genic_upstream_transcript_variant,intron_variant,2KB_upstream_variant,upstream_transcript_variant
rs1025181706	intron_variant
rs1025191276	intron_variant,genic_downstream_transcript_variant
rs1025196653	genic_upstream_transcript_variant,intron_variant
rs1025200724	genic_upstream_transcript_variant,intron_variant
rs1025204532	genic_upstream_transcript_variant,intron_variant
rs1025228687	intron_variant
rs1025238479	genic_upstream_transcript_variant,intron_variant
rs1025360362	genic_upstream_transcript_variant,intron_variant
rs1025419708	intron_variant
rs1025423248	genic_upstream_transcript_variant,intron_variant
rs1025432258	genic_upstream_transcript_variant,intron_variant
rs1025440831	genic_upstream_transcript_variant,intron_variant
rs1025453048	intron_variant
rs1025467259	genic_upstream_transcript_variant,intron_variant,2KB_upstream_variant,upstream_transcript_variant
rs1025502766	intron_variant
rs1025521964	intron_variant,genic_downstream_transcript_variant
rs1025528705	intron_variant
rs1025543139	genic_upstream_transcript_variant,intron_variant
rs1025548892	genic_upstream_transcript_variant,intron_variant
rs1025593394	intron_variant,genic_downstream_transcript_variant
rs1025635076	intron_variant
rs1025655839	genic_upstream_transcript_variant,intron_variant
rs1025668759	intron_variant
rs1025713656	genic_upstream_transcript_variant,intron_variant
rs1025775703	intron_variant
rs1025791891	genic_upstream_transcript_variant,intron_variant
rs1025807773	intron_variant
rs1025820467	intron_variant
rs1025862032	intron_variant
rs1025938195	genic_upstream_transcript_variant,intron_variant
rs1026050263	genic_upstream_transcript_variant,intron_variant
rs1026089493	non_coding_transcript_variant,genic_downstream_transcript_variant,3_prime_UTR_variant
rs1026108780	intron_variant
rs1026121853	genic_upstream_transcript_variant,intron_variant
rs1026168519	intron_variant
rs1026197638	intron_variant
rs1026207273	genic_upstream_transcript_variant,intron_variant
rs1026222878	genic_upstream_transcript_variant,intron_variant
rs1026280249	intron_variant,genic_downstream_transcript_variant
rs1026290900	intron_variant,genic_downstream_transcript_variant
rs1026307052	genic_upstream_transcript_variant,intron_variant
rs1026319741	intron_variant,genic_downstream_transcript_variant
rs1026369431	intron_variant
rs1026420297	genic_upstream_transcript_variant,intron_variant
rs1026429907	intron_variant
rs1026451594	non_coding_transcript_variant,genic_downstream_transcript_variant,3_prime_UTR_variant
rs1026476215	intron_variant
rs1026488974	intron_variant
rs1026507881	intron_variant
rs1026532144	intron_variant,genic_downstream_transcript_variant
rs1026555704	intron_variant
rs1026576414	intron_variant
rs1026613486	intron_variant
rs1026641397	genic_upstream_transcript_variant,intron_variant
rs1026644109	intron_variant,genic_downstream_transcript_variant
rs1026665649	genic_upstream_transcript_variant,intron_variant,upstream_transcript_variant
rs1026676193	genic_upstream_transcript_variant,intron_variant
rs1026690760	intron_variant
rs1026703523	genic_upstream_transcript_variant,intron_variant
rs1026714165	intron_variant
rs1026775333	intron_variant,genic_downstream_transcript_variant,downstream_transcript_variant

rs1026792094	genic_upstream_transcript_variant,intron_variant
rs1026822474	intron_variant
rs1026832778	genic_upstream_transcript_variant,intron_variant
rs1026850747	genic_upstream_transcript_variant,intron_variant
rs1026878880	intron_variant
rs1026881332	intron_variant,genic_downstream_transcript_variant
rs1026918336	intron_variant,genic_downstream_transcript_variant
rs1026927243	genic_upstream_transcript_variant,intron_variant
rs1027001627	intron_variant
rs1027004349	intron_variant,genic_downstream_transcript_variant
rs1027054369	genic_upstream_transcript_variant,intron_variant,2KB_upstream_variant,upstream_transcript_variant
rs1027068758	genic_upstream_transcript_variant,intron_variant
rs1027111564	intron_variant
rs1027140628	intron_variant
rs1027153638	intron_variant,genic_downstream_transcript_variant
rs1027160520	intron_variant,genic_downstream_transcript_variant
rs1027206441	intron_variant,genic_downstream_transcript_variant
rs1027272067	intron_variant,genic_downstream_transcript_variant
rs1027295353	genic_upstream_transcript_variant,intron_variant
rs1027309488	intron_variant
rs1027311640	intron_variant
rs1027333045	intron_variant,genic_downstream_transcript_variant
rs1027373411	genic_upstream_transcript_variant,intron_variant,2KB_upstream_variant,upstream_transcript_variant
rs1027429538	genic_upstream_transcript_variant,intron_variant
rs1027442439	genic_upstream_transcript_variant,intron_variant
rs1027443630	intron_variant
rs1027479697	genic_upstream_transcript_variant,intron_variant
rs1027508629	genic_upstream_transcript_variant,intron_variant
rs1027526121	genic_upstream_transcript_variant,intron_variant,2KB_upstream_variant,upstream_transcript_variant
rs1027540070	genic_upstream_transcript_variant,intron_variant
rs1027557988	intron_variant
rs1027612312	genic_upstream_transcript_variant,intron_variant
rs1027646670	intron_variant,genic_downstream_transcript_variant
rs1027738818	missense_variant,genic_upstream_transcript_variant,intron_variant,non_coding_transcript_variant,coding_sequence_variant
rs1027786262	genic_upstream_transcript_variant,intron_variant
rs1027796512	intron_variant
rs1027808795	intron_variant
rs1027833640	genic_upstream_transcript_variant,2KB_upstream_variant,intron_variant,upstream_transcript_variant
rs1027854017	intron_variant
rs1027873185	intron_variant
rs1027929392	genic_upstream_transcript_variant,intron_variant
rs1027930146	genic_upstream_transcript_variant,intron_variant
rs1027951071	intron_variant,genic_downstream_transcript_variant
rs1027953962	genic_upstream_transcript_variant,intron_variant
rs1027964777	intron_variant
rs1028065950	genic_upstream_transcript_variant,intron_variant
rs1028083886	intron_variant
rs1028134766	genic_upstream_transcript_variant,intron_variant
rs1028207880	genic_upstream_transcript_variant,intron_variant
rs1028208514	intron_variant
rs1028249112	genic_upstream_transcript_variant,intron_variant
rs1028249608	genic_upstream_transcript_variant,intron_variant
rs1028312735	genic_upstream_transcript_variant,intron_variant
rs1028324917	intron_variant,genic_downstream_transcript_variant
rs1028340516	intron_variant
rs1028355068	intron_variant,genic_downstream_transcript_variant
rs1028381199	intron_variant
rs1028421413	intron_variant
rs1028427864	genic_upstream_transcript_variant,intron_variant
rs1028460880	intron_variant,genic_downstream_transcript_variant
rs1028485983	intron_variant
rs1028514982	intron_variant
rs1028528698	genic_upstream_transcript_variant,intron_variant
rs1028560520	intron_variant
rs1028608450	genic_upstream_transcript_variant,intron_variant
rs1028622524	genic_upstream_transcript_variant,intron_variant
rs1028630144	genic_upstream_transcript_variant,intron_variant
rs1028634509	genic_upstream_transcript_variant,intron_variant
rs1028654157	intron_variant
rs1028726517	intron_variant
rs1028745568	genic_upstream_transcript_variant,intron_variant
rs1028759335	genic_upstream_transcript_variant,intron_variant
rs1028870865	genic_upstream_transcript_variant,intron_variant
rs1028894168	genic_upstream_transcript_variant,intron_variant

rs1028948413	intron_variant
rs1028958269	genic_upstream_transcript_variant,intron_variant
rs1028966184	intron_variant
rs1028979439	intron_variant
rs1029010488	genic_upstream_transcript_variant,intron_variant
rs1029045424	intron_variant
rs1029062272	intron_variant,genic_downstream_transcript_variant
rs1029097834	intron_variant,genic_downstream_transcript_variant
rs1029129632	genic_upstream_transcript_variant,intron_variant
rs1029156431	intron_variant
rs1029186303	intron_variant
rs1029216979	intron_variant,genic_downstream_transcript_variant
rs1029224555	intron_variant
rs1029233710	genic_upstream_transcript_variant,intron_variant
rs1029313604	intron_variant
rs1029333528	genic_upstream_transcript_variant,intron_variant
rs1029430722	intron_variant,genic_downstream_transcript_variant
rs1029436231	genic_upstream_transcript_variant,intron_variant
rs1029449613	intron_variant
rs1029468298	genic_upstream_transcript_variant,intron_variant
rs1029481577	non_coding_transcript_variant,5_prime_UTR_variant,intron_variant,genic_upstream_transcript_variant
rs1029525680	genic_upstream_transcript_variant,intron_variant
rs1029557150	genic_upstream_transcript_variant,2KB_upstream_variant,intron_variant,upstream_transcript_variant
rs1029564320	intron_variant
rs1029564438	intron_variant
rs1029586342	genic_upstream_transcript_variant,intron_variant
rs1029588779	intron_variant
rs1029616129	intron_variant
rs1029618378	intron_variant,genic_downstream_transcript_variant
rs1029643456	intron_variant,genic_downstream_transcript_variant
rs1029664052	genic_upstream_transcript_variant,intron_variant
rs1029693386	intron_variant,genic_downstream_transcript_variant
rs1029707067	genic_upstream_transcript_variant,intron_variant,2KB_upstream_variant,upstream_transcript_variant
rs1029716326	genic_upstream_transcript_variant,intron_variant
rs1029739918	intron_variant,genic_downstream_transcript_variant
rs1029770146	intron_variant,genic_downstream_transcript_variant
rs1029797658	intron_variant
rs1029802073	intron_variant,genic_downstream_transcript_variant
rs1029821893	intron_variant
rs1029875574	intron_variant
rs1029878661	intron_variant,genic_downstream_transcript_variant
rs1029891407	intron_variant
rs1029899403	intron_variant
rs1029905895	genic_upstream_transcript_variant,intron_variant
rs1029955797	intron_variant,genic_downstream_transcript_variant
rs1030000033	genic_upstream_transcript_variant,intron_variant
rs1030022257	genic_upstream_transcript_variant,intron_variant
rs1030034254	intron_variant
rs1030039956	genic_upstream_transcript_variant,intron_variant
rs1030102539	genic_upstream_transcript_variant,intron_variant
rs1030102892	genic_upstream_transcript_variant,intron_variant
rs1030172300	intron_variant
rs1030179620	genic_upstream_transcript_variant,intron_variant
rs1030210171	intron_variant,genic_downstream_transcript_variant
rs1030214913	intron_variant
rs1030223102	intron_variant
rs1030233666	genic_upstream_transcript_variant,intron_variant,2KB_upstream_variant,upstream_transcript_variant
rs1030235572	intron_variant,genic_downstream_transcript_variant
rs1030265438	genic_upstream_transcript_variant,intron_variant
rs1030266080	intron_variant
rs1030269221	genic_upstream_transcript_variant,intron_variant
rs1030275194	genic_upstream_transcript_variant,2KB_upstream_variant,intron_variant,upstream_transcript_variant
rs1030306599	intron_variant
rs1030313623	genic_upstream_transcript_variant,intron_variant
rs1030316795	intron_variant,genic_downstream_transcript_variant
rs1030372302	non_coding_transcript_variant,genic_downstream_transcript_variant,3_prime_UTR_variant
rs1030388726	intron_variant,genic_downstream_transcript_variant
rs1030418561	intron_variant
rs1030480151	genic_upstream_transcript_variant,intron_variant
rs1030485541	intron_variant
rs1030531848	intron_variant
rs1030554153	genic_upstream_transcript_variant,intron_variant,2KB_upstream_variant,upstream_transcript_variant
rs1030568134	genic_upstream_transcript_variant,intron_variant
rs1030575569	non_coding_transcript_variant,genic_downstream_transcript_variant,3_prime_UTR_variant

rs1030587758	intron_variant,genic_downstream_transcript_variant
rs1030593873	intron_variant
rs1030628137	genic_upstream_transcript_variant,intron_variant
rs1030657521	intron_variant
rs1030672974	intron_variant
rs1030693572	intron_variant,genic_downstream_transcript_variant
rs1030716104	genic_upstream_transcript_variant,intron_variant
rs1030771245	genic_upstream_transcript_variant,intron_variant
rs1030777499	intron_variant
rs1030793481	genic_upstream_transcript_variant,intron_variant,upstream_transcript_variant
rs1030829199	genic_upstream_transcript_variant,intron_variant
rs1030837751	intron_variant
rs1030856165	genic_upstream_transcript_variant,intron_variant
rs1030866678	5_prime_UTR_variant,intron_variant,upstream_transcript_variant,genic_upstream_transcript_variant
rs1030879760	genic_upstream_transcript_variant,intron_variant
rs1030995179	genic_upstream_transcript_variant,intron_variant
rs1031010611	intron_variant
rs1031030746	intron_variant
rs1031058149	intron_variant
rs1031098520	genic_upstream_transcript_variant,intron_variant,2KB_upstream_variant,upstream_transcript_variant
rs1031113005	intron_variant
rs1031119318	genic_upstream_transcript_variant,intron_variant
rs1031144960	intron_variant,genic_downstream_transcript_variant
rs1031155122	intron_variant,genic_downstream_transcript_variant
rs1031170535	genic_upstream_transcript_variant,intron_variant,2KB_upstream_variant,upstream_transcript_variant
rs1031201502	genic_upstream_transcript_variant,intron_variant
rs1031231512	intron_variant,genic_downstream_transcript_variant
rs1031263408	genic_upstream_transcript_variant,intron_variant
rs1031263507	non_coding_transcript_variant,missense_variant,genic_downstream_transcript_variant,coding_sequence_variant
rs1031283204	intron_variant,genic_downstream_transcript_variant
rs1031316112	intron_variant,genic_downstream_transcript_variant
rs1031346143	intron_variant
rs1031373195	intron_variant
rs1031395118	intron_variant,genic_downstream_transcript_variant
rs1031434598	genic_upstream_transcript_variant,intron_variant
rs1031445859	intron_variant,genic_downstream_transcript_variant
rs1031483349	intron_variant
rs1031487261	intron_variant,genic_downstream_transcript_variant
rs1031500566	genic_upstream_transcript_variant,intron_variant
rs1031574250	intron_variant
rs1031587640	intron_variant,genic_downstream_transcript_variant
rs1031644246	genic_upstream_transcript_variant,intron_variant
rs1031646843	genic_upstream_transcript_variant,intron_variant
rs1031650077	non_coding_transcript_variant,missense_variant,coding_sequence_variant
rs1031735122	intron_variant
rs1031750115	genic_upstream_transcript_variant,intron_variant
rs1031757866	intron_variant
rs1031760756	genic_upstream_transcript_variant,intron_variant
rs1031790401	intron_variant
rs1031806731	genic_upstream_transcript_variant,intron_variant
rs1031820555	intron_variant
rs1031834689	intron_variant,genic_downstream_transcript_variant
rs1031869637	intron_variant,genic_downstream_transcript_variant
rs1031905651	intron_variant,genic_downstream_transcript_variant
rs1031916783	intron_variant
rs1031922585	genic_upstream_transcript_variant,intron_variant
rs1031954191	intron_variant
rs1032016537	intron_variant
rs1032068467	intron_variant
rs1032074450	intron_variant,genic_downstream_transcript_variant
rs1032076507	intron_variant
rs1032099049	intron_variant
rs1032101638	intron_variant
rs1032113840	genic_upstream_transcript_variant,intron_variant
rs1032146739	5_prime_UTR_variant,intron_variant
rs1032152380	intron_variant
rs1032224964	genic_upstream_transcript_variant,intron_variant,upstream_transcript_variant
rs1032249086	genic_upstream_transcript_variant,intron_variant
rs1032251922	genic_upstream_transcript_variant,intron_variant
rs1032263485	intron_variant,genic_downstream_transcript_variant
rs1032342047	intron_variant
rs1032401004	genic_upstream_transcript_variant,intron_variant
rs1032423065	intron_variant
rs1032438169	genic_upstream_transcript_variant,intron_variant

rs1032453528	genic_upstream_transcript_variant,intron_variant
rs1032481283	intron_variant
rs1032522458	intron_variant,genic_downstream_transcript_variant
rs1032539388	intron_variant
rs1032565691	genic_upstream_transcript_variant,intron_variant
rs1032573039	intron_variant,genic_downstream_transcript_variant
rs1032579261	non_coding_transcript_variant,genic_downstream_transcript_variant,3_prime_UTR_variant
rs1032592106	intron_variant
rs1032603208	genic_upstream_transcript_variant,intron_variant
rs1032630282	non_coding_transcript_variant,genic_downstream_transcript_variant,3_prime_UTR_variant
rs1032630644	intron_variant
rs1032678553	genic_upstream_transcript_variant,intron_variant
rs1032698519	intron_variant
rs1032751404	intron_variant
rs1032763257	genic_upstream_transcript_variant,intron_variant
rs1032789767	intron_variant
rs1032818382	genic_upstream_transcript_variant,intron_variant
rs1032840318	intron_variant
rs1032878990	genic_upstream_transcript_variant,intron_variant
rs1032893138	non_coding_transcript_variant,genic_downstream_transcript_variant,3_prime_UTR_variant
rs1032910944	non_coding_transcript_variant,genic_downstream_transcript_variant,3_prime_UTR_variant
rs1032914660	genic_upstream_transcript_variant,intron_variant
rs1032917885	intron_variant
rs1032983864	intron_variant,genic_downstream_transcript_variant
rs1033012361	intron_variant,genic_downstream_transcript_variant
rs1033019977	intron_variant
rs1033046853	genic_upstream_transcript_variant,intron_variant
rs1033093453	intron_variant
rs1033093988	intron_variant,genic_downstream_transcript_variant
rs1033109857	genic_upstream_transcript_variant,intron_variant
rs1033125769	intron_variant
rs1033129190	intron_variant,genic_downstream_transcript_variant
rs1033161787	intron_variant,genic_downstream_transcript_variant
rs1033166423	genic_upstream_transcript_variant,intron_variant
rs1033193067	genic_upstream_transcript_variant,intron_variant
rs1033201340	genic_upstream_transcript_variant,intron_variant
rs1033220658	genic_upstream_transcript_variant,intron_variant
rs1033232940	intron_variant
rs1033245540	intron_variant,genic_downstream_transcript_variant
rs1033274278	genic_upstream_transcript_variant,intron_variant
rs1033293858	intron_variant
rs1033326295	genic_upstream_transcript_variant,intron_variant
rs1033339393	intron_variant,genic_downstream_transcript_variant
rs1033389656	genic_upstream_transcript_variant,intron_variant
rs1033434044	intron_variant,genic_downstream_transcript_variant
rs1033439242	genic_upstream_transcript_variant,intron_variant
rs1033482028	genic_upstream_transcript_variant,intron_variant
rs1033507868	genic_upstream_transcript_variant,intron_variant
rs1033529036	intron_variant
rs1033556559	intron_variant
rs1033586067	genic_upstream_transcript_variant,intron_variant,non_coding_transcript_variant,coding_sequence_variant,synonymous_variant
rs1033618759	genic_upstream_transcript_variant,intron_variant
rs1033649894	genic_upstream_transcript_variant,2KB_upstream_variant,intron_variant,upstream_transcript_variant
rs1033741869	intron_variant
rs1033763374	intron_variant
rs1033764040	genic_upstream_transcript_variant,intron_variant
rs1033773677	genic_upstream_transcript_variant,intron_variant
rs1033829824	genic_upstream_transcript_variant,intron_variant
rs1033845243	intron_variant
rs1033847172	non_coding_transcript_variant,genic_downstream_transcript_variant,3_prime_UTR_variant
rs1033847643	intron_variant,genic_downstream_transcript_variant
rs1033874166	intron_variant,genic_downstream_transcript_variant
rs1033878007	intron_variant
rs1033891123	genic_upstream_transcript_variant,intron_variant
rs1033948381	genic_upstream_transcript_variant,intron_variant
rs1033954520	genic_upstream_transcript_variant,intron_variant
rs1034007893	intron_variant
rs1034010001	intron_variant,genic_downstream_transcript_variant
rs1034080480	intron_variant
rs1034081710	intron_variant
rs1034092517	non_coding_transcript_variant,genic_downstream_transcript_variant,3_prime_UTR_variant
rs1034111781	genic_upstream_transcript_variant,intron_variant
rs1034173206	500B_downstream_variant,downstream_transcript_variant
rs1034186286	genic_upstream_transcript_variant,intron_variant

rs1034209838	intron_variant,genic_downstream_transcript_variant
rs1034212276	intron_variant
rs1034259745	genic_upstream_transcript_variant,intron_variant
rs1034260218	genic_upstream_transcript_variant,intron_variant
rs1034287073	intron_variant
rs1034313337	genic_upstream_transcript_variant,intron_variant
rs1034364917	intron_variant
rs1034411939	genic_upstream_transcript_variant,2KB_upstream_variant,intron_variant,upstream_transcript_variant
rs1034429722	intron_variant
rs1034453482	intron_variant
rs1034484459	intron_variant
rs1034491548	intron_variant,genic_downstream_transcript_variant
rs1034493969	intron_variant,genic_downstream_transcript_variant
rs1034506068	genic_upstream_transcript_variant,2KB_upstream_variant,intron_variant,upstream_transcript_variant
rs1034512052	genic_upstream_transcript_variant,intron_variant
rs1034552945	genic_upstream_transcript_variant,intron_variant
rs1034591613	non_coding_transcript_variant,genic_downstream_transcript_variant,3_prime_UTR_variant
rs1034599668	intron_variant,genic_downstream_transcript_variant
rs1034648942	intron_variant
rs1034767860	intron_variant
rs1034782473	genic_upstream_transcript_variant,intron_variant
rs1034798455	intron_variant
rs1034812133	intron_variant,genic_downstream_transcript_variant
rs1034855737	genic_upstream_transcript_variant,intron_variant,2KB_upstream_variant,upstream_transcript_variant
rs1034881262	intron_variant,genic_downstream_transcript_variant
rs1034885462	genic_upstream_transcript_variant,intron_variant,2KB_upstream_variant,upstream_transcript_variant
rs1034894536	genic_upstream_transcript_variant,intron_variant
rs1034897941	genic_upstream_transcript_variant,intron_variant
rs1034927469	intron_variant,genic_downstream_transcript_variant
rs1034950470	genic_upstream_transcript_variant,intron_variant
rs1034957774	intron_variant
rs1035000028	genic_upstream_transcript_variant,intron_variant
rs1035010240	genic_upstream_transcript_variant,2KB_upstream_variant,intron_variant,upstream_transcript_variant
rs1035020677	intron_variant
rs1035030065	intron_variant
rs1035050733	intron_variant
rs1035067685	genic_upstream_transcript_variant,intron_variant
rs1035075983	intron_variant
rs1035077217	genic_upstream_transcript_variant,intron_variant
rs1035108474	genic_upstream_transcript_variant,intron_variant
rs1035134879	intron_variant
rs1035141881	intron_variant,genic_downstream_transcript_variant
rs1035152966	intron_variant
rs1035163860	genic_upstream_transcript_variant,intron_variant
rs1035172009	genic_upstream_transcript_variant,intron_variant
rs1035176160	genic_upstream_transcript_variant,intron_variant
rs1035193531	genic_upstream_transcript_variant,intron_variant
rs1035203710	intron_variant
rs1035221677	genic_upstream_transcript_variant,intron_variant
rs1035242854	intron_variant,genic_downstream_transcript_variant
rs1035266703	genic_upstream_transcript_variant,intron_variant
rs1035269753	intron_variant
rs1035285721	genic_upstream_transcript_variant,intron_variant
rs1035338828	intron_variant
rs1035359221	intron_variant
rs1035416475	genic_upstream_transcript_variant,2KB_upstream_variant,intron_variant,upstream_transcript_variant
rs1035428773	genic_upstream_transcript_variant,intron_variant
rs1035439339	intron_variant
rs1035446734	intron_variant,genic_downstream_transcript_variant
rs1035456694	intron_variant
rs1035473466	genic_upstream_transcript_variant,intron_variant
rs1035481584	intron_variant,genic_downstream_transcript_variant
rs1035487883	intron_variant
rs1035496340	genic_upstream_transcript_variant,intron_variant
rs1035496938	genic_upstream_transcript_variant,intron_variant
rs1035509794	genic_upstream_transcript_variant,intron_variant
rs1035529833	genic_upstream_transcript_variant,intron_variant
rs1035541992	intron_variant
rs1035573539	genic_upstream_transcript_variant,intron_variant
rs1035648150	genic_upstream_transcript_variant,2KB_upstream_variant,intron_variant,upstream_transcript_variant
rs1035676006	genic_upstream_transcript_variant,intron_variant
rs1035699869	genic_upstream_transcript_variant,intron_variant
rs1035709000	intron_variant
rs1035744630	intron_variant,genic_downstream_transcript_variant

rs1035747846	genic_upstream_transcript_variant,2KB_upstream_variant,intron_variant,upstream_transcript_variant
rs1035765584	genic_upstream_transcript_variant,intron_variant
rs1035786903	intron_variant,genic_downstream_transcript_variant
rs1035857913	genic_upstream_transcript_variant,intron_variant
rs1035871824	intron_variant
rs1035899318	intron_variant
rs1035950860	intron_variant
rs1035964851	intron_variant
rs1036005283	genic_upstream_transcript_variant,intron_variant,upstream_transcript_variant
rs1036008496	intron_variant
rs1036031688	genic_upstream_transcript_variant,intron_variant
rs1036059471	intron_variant
rs1036077780	intron_variant,genic_downstream_transcript_variant
rs1036086324	intron_variant
rs1036091704	intron_variant
rs1036106957	intron_variant
rs1036164388	genic_upstream_transcript_variant,intron_variant,2KB_upstream_variant,upstream_transcript_variant
rs1036232217	intron_variant,genic_downstream_transcript_variant
rs1036242526	intron_variant
rs1036257863	intron_variant,genic_downstream_transcript_variant
rs1036262060	genic_upstream_transcript_variant,intron_variant,2KB_upstream_variant,upstream_transcript_variant
rs1036264842	intron_variant,genic_downstream_transcript_variant
rs1036311216	genic_upstream_transcript_variant,intron_variant
rs1036326563	intron_variant
rs1036362708	intron_variant
rs1036406092	genic_upstream_transcript_variant,intron_variant
rs1036427504	intron_variant
rs1036462229	genic_upstream_transcript_variant,intron_variant
rs1036490359	genic_upstream_transcript_variant,intron_variant
rs1036504133	genic_upstream_transcript_variant,intron_variant
rs1036506753	intron_variant
rs1036606502	genic_upstream_transcript_variant,intron_variant
rs1036618644	genic_upstream_transcript_variant,intron_variant
rs1036649236	genic_upstream_transcript_variant,intron_variant
rs1036653866	2KB_upstream_variant,genic_upstream_transcript_variant,upstream_transcript_variant,intron_variant
rs1036688328	genic_upstream_transcript_variant,intron_variant
rs1036692417	genic_upstream_transcript_variant,intron_variant
rs1036716154	genic_downstream_transcript_variant,intron_variant
rs1036757961	2KB_upstream_variant,genic_upstream_transcript_variant,upstream_transcript_variant,intron_variant
rs1036790423	genic_downstream_transcript_variant,intron_variant
rs1036800474	intron_variant
rs1036849719	2KB_upstream_variant,genic_upstream_transcript_variant,upstream_transcript_variant,intron_variant
rs1036850647	intron_variant
rs1036917823	2KB_upstream_variant,genic_upstream_transcript_variant,upstream_transcript_variant,intron_variant
rs1036950595	genic_upstream_transcript_variant,intron_variant
rs1036951032	genic_upstream_transcript_variant,intron_variant
rs1036977643	genic_upstream_transcript_variant,intron_variant
rs1036990064	5_prime_UTR_variant,non_coding_transcript_variant,genic_upstream_transcript_variant,intron_variant
rs1037001310	2KB_upstream_variant,genic_upstream_transcript_variant,upstream_transcript_variant,intron_variant
rs1037035129	genic_upstream_transcript_variant,intron_variant
rs1037048542	intron_variant
rs1037051057	genic_downstream_transcript_variant,intron_variant
rs1037064829	intron_variant
rs1037160741	intron_variant
rs1037209874	intron_variant
rs1037251078	intron_variant
rs1037256692	genic_upstream_transcript_variant,intron_variant
rs1037320468	genic_upstream_transcript_variant,intron_variant
rs1037333149	genic_downstream_transcript_variant,intron_variant
rs1037349391	genic_upstream_transcript_variant,intron_variant
rs1037393215	genic_downstream_transcript_variant,intron_variant
rs1037394144	genic_upstream_transcript_variant,intron_variant
rs1037471526	intron_variant
rs1037482399	genic_upstream_transcript_variant,intron_variant
rs1037500697	intron_variant
rs1037502939	intron_variant
rs1037512411	genic_upstream_transcript_variant,intron_variant
rs1037520287	genic_upstream_transcript_variant,intron_variant
rs1037566894	genic_upstream_transcript_variant,intron_variant
rs1037649219	intron_variant
rs1037653419	genic_downstream_transcript_variant,intron_variant
rs1037660316	genic_upstream_transcript_variant,intron_variant
rs1037721615	genic_downstream_transcript_variant,intron_variant
rs1037737076	intron_variant

rs1037745449	intron_variant
rs1037760424	genic_upstream_transcript_variant,intron_variant
rs1037826507	genic_upstream_transcript_variant,intron_variant
rs1037855193	genic_downstream_transcript_variant,intron_variant
rs1037864879	intron_variant
rs1037882420	intron_variant
rs1037905388	genic_upstream_transcript_variant,intron_variant
rs1037946581	intron_variant
rs1037946700	genic_downstream_transcript_variant,intron_variant
rs1037961345	genic_upstream_transcript_variant,intron_variant
rs1037970707	genic_upstream_transcript_variant,intron_variant
rs1038009299	genic_upstream_transcript_variant,intron_variant
rs1038057921	genic_downstream_transcript_variant,intron_variant
rs1038075119	genic_downstream_transcript_variant,intron_variant
rs1038080366	intron_variant
rs1038093717	genic_downstream_transcript_variant,intron_variant
rs1038136314	genic_downstream_transcript_variant,intron_variant
rs1038158300	genic_downstream_transcript_variant,intron_variant
rs1038165255	intron_variant
rs1038191047	genic_downstream_transcript_variant,intron_variant
rs1038238179	genic_upstream_transcript_variant,intron_variant
rs1038247061	genic_downstream_transcript_variant,intron_variant
rs1038247997	intron_variant
rs1038280819	intron_variant
rs1038296740	intron_variant
rs1038326478	intron_variant
rs1038327109	genic_downstream_transcript_variant,intron_variant
rs1038339616	genic_upstream_transcript_variant,intron_variant
rs1038358197	intron_variant
rs1038414674	genic_upstream_transcript_variant,intron_variant
rs1038419114	intron_variant
rs1038432955	intron_variant
rs1038446960	genic_upstream_transcript_variant,intron_variant
rs1038484467	genic_upstream_transcript_variant,intron_variant
rs1038505560	genic_upstream_transcript_variant,intron_variant
rs1038549138	genic_upstream_transcript_variant,intron_variant
rs1038595675	intron_variant
rs1038640033	genic_upstream_transcript_variant,intron_variant
rs1038647344	genic_upstream_transcript_variant,intron_variant
rs1038676535	genic_upstream_transcript_variant,intron_variant
rs1038680326	genic_downstream_transcript_variant,intron_variant
rs1038683390	genic_downstream_transcript_variant,intron_variant
rs1038704235	genic_upstream_transcript_variant,intron_variant
rs1038732024	genic_upstream_transcript_variant,intron_variant
rs1038782604	genic_downstream_transcript_variant,intron_variant
rs1038802243	intron_variant
rs1038804061	intron_variant
rs1038809715	intron_variant
rs1038825040	genic_downstream_transcript_variant,non_coding_transcript_variant,3_prime_UTR_variant
rs1038845936	genic_upstream_transcript_variant,intron_variant
rs1038856847	intron_variant
rs1038900924	genic_upstream_transcript_variant,intron_variant
rs1038933093	genic_upstream_transcript_variant,intron_variant
rs1039009466	genic_downstream_transcript_variant,intron_variant
rs1039016616	intron_variant
rs1039111271	intron_variant
rs1039123266	intron_variant
rs1039127338	genic_upstream_transcript_variant,intron_variant
rs1039176355	genic_upstream_transcript_variant,intron_variant
rs1039176965	intron_variant
rs1039181500	genic_upstream_transcript_variant,intron_variant
rs1039187814	intron_variant
rs1039216160	genic_downstream_transcript_variant,intron_variant
rs1039219244	genic_upstream_transcript_variant,intron_variant
rs1039221415	genic_downstream_transcript_variant,intron_variant
rs1039223728	intron_variant
rs1039233939	genic_upstream_transcript_variant,intron_variant
rs1039293658	genic_downstream_transcript_variant,intron_variant
rs1039312371	intron_variant
rs1039312993	genic_upstream_transcript_variant,intron_variant
rs1039313707	genic_downstream_transcript_variant,non_coding_transcript_variant,3_prime_UTR_variant
rs1039349451	genic_upstream_transcript_variant,intron_variant
rs1039379981	genic_upstream_transcript_variant,intron_variant
rs1039383510	intron_variant

rs1039387100	genic_downstream_transcript_variant,intron_variant
rs1039410000	genic_downstream_transcript_variant,intron_variant
rs1039414818	intron_variant
rs1039424369	genic_downstream_transcript_variant,intron_variant
rs1039499440	intron_variant
rs1039500672	intron_variant
rs1039548882	genic_upstream_transcript_variant,intron_variant
rs1039561429	intron_variant
rs1039568338	genic_upstream_transcript_variant,intron_variant
rs1039616723	intron_variant
rs1039629132	genic_downstream_transcript_variant,intron_variant
rs1039630031	genic_downstream_transcript_variant,intron_variant
rs1039637619	genic_upstream_transcript_variant,intron_variant
rs1039642009	genic_upstream_transcript_variant,intron_variant
rs1039651764	genic_upstream_transcript_variant,intron_variant
rs1039667815	intron_variant
rs1039688347	intron_variant
rs1039739519	intron_variant
rs1039739932	intron_variant
rs1039753743	genic_upstream_transcript_variant,intron_variant
rs1039756141	genic_upstream_transcript_variant,intron_variant
rs1039786048	genic_upstream_transcript_variant,intron_variant
rs1039829767	genic_upstream_transcript_variant,intron_variant
rs1039836444	intron_variant
rs1039840986	genic_upstream_transcript_variant,intron_variant
rs1039885513	intron_variant
rs1039890932	intron_variant
rs1039917557	genic_upstream_transcript_variant,intron_variant
rs1039967914	intron_variant
rs1040051638	intron_variant
rs1040084559	genic_downstream_transcript_variant,intron_variant
rs1040089268	genic_downstream_transcript_variant,intron_variant
rs1040099445	intron_variant
rs1040121697	intron_variant
rs1040142298	intron_variant
rs1040167967	2KB_upstream_variant,genic_upstream_transcript_variant,upstream_transcript_variant,intron_variant
rs1040175213	intron_variant
rs1040193130	intron_variant
rs1040203556	genic_upstream_transcript_variant,intron_variant
rs1040223731	intron_variant
rs1040226197	genic_upstream_transcript_variant,intron_variant
rs1040233509	intron_variant
rs1040260756	2KB_upstream_variant,genic_upstream_transcript_variant,upstream_transcript_variant,intron_variant
rs1040290269	intron_variant
rs1040361641	genic_downstream_transcript_variant,intron_variant
rs1040398598	genic_upstream_transcript_variant,intron_variant
rs1040401415	genic_upstream_transcript_variant,intron_variant
rs1040454040	intron_variant
rs1040484267	genic_upstream_transcript_variant,intron_variant
rs1040553171	genic_upstream_transcript_variant,intron_variant
rs1040577560	genic_upstream_transcript_variant,intron_variant
rs1040585823	genic_downstream_transcript_variant,non_coding_transcript_variant,3_prime_UTR_variant
rs1040611641	genic_upstream_transcript_variant,intron_variant
rs1040612180	intron_variant
rs1040616355	genic_upstream_transcript_variant,intron_variant
rs1040619810	intron_variant
rs1040707623	genic_downstream_transcript_variant,intron_variant
rs1040758056	genic_upstream_transcript_variant,intron_variant
rs1040775732	intron_variant
rs1040847937	intron_variant
rs1040858792	genic_downstream_transcript_variant,intron_variant
rs1040901649	genic_upstream_transcript_variant,intron_variant
rs1040926195	genic_upstream_transcript_variant,intron_variant
rs1040953314	genic_downstream_transcript_variant,intron_variant
rs1040953976	genic_upstream_transcript_variant,intron_variant
rs1040998158	intron_variant
rs1041001234	intron_variant
rs1041018209	genic_upstream_transcript_variant,intron_variant
rs1041035286	genic_downstream_transcript_variant,intron_variant
rs1041035833	genic_downstream_transcript_variant,non_coding_transcript_variant,3_prime_UTR_variant
rs1041039478	intron_variant
rs1041063070	intron_variant
rs1041077971	intron_variant
rs1041154057	genic_upstream_transcript_variant,intron_variant

rs1041194087	intron_variant
rs1041240263	intron_variant
rs1041241392	genic_downstream_transcript_variant,intron_variant
rs1041316843	genic_downstream_transcript_variant,intron_variant
rs1041317169	genic_downstream_transcript_variant,intron_variant
rs1041320020	intron_variant
rs1041400087	genic_downstream_transcript_variant,intron_variant
rs1041415665	genic_upstream_transcript_variant,intron_variant
rs1041423745	500B_downstream_variant,downstream_transcript_variant
rs1041452734	genic_downstream_transcript_variant,intron_variant
rs1041497799	genic_downstream_transcript_variant,intron_variant
rs1041538717	intron_variant
rs1041566754	genic_upstream_transcript_variant,intron_variant
rs1041571619	intron_variant
rs1041597488	genic_upstream_transcript_variant,intron_variant
rs1041599369	genic_upstream_transcript_variant,intron_variant
rs1041686512	intron_variant
rs1041769196	intron_variant
rs1041775939	intron_variant
rs1041801500	intron_variant
rs1041813771	intron_variant
rs1041836036	genic_upstream_transcript_variant,intron_variant
rs1041852335	intron_variant
rs1041875366	genic_downstream_transcript_variant,intron_variant
rs1041926507	genic_downstream_transcript_variant,intron_variant
rs1041927454	intron_variant
rs1041936242	genic_upstream_transcript_variant,intron_variant
rs1041978884	genic_downstream_transcript_variant,intron_variant
rs1041997097	5_prime_UTR_variant,non_coding_transcript_variant,genic_upstream_transcript_variant,intron_variant
rs1041999943	genic_downstream_transcript_variant,intron_variant
rs1042049674	genic_downstream_transcript_variant,intron_variant
rs1042056480	genic_upstream_transcript_variant,intron_variant
rs1042096538	genic_downstream_transcript_variant,intron_variant
rs1042151073	2KB_upstream_variant,genic_upstream_transcript_variant,upstream_transcript_variant,intron_variant
rs1042157413	genic_downstream_transcript_variant,intron_variant
rs1042158891	intron_variant
rs1042160259	intron_variant
rs1042168499	intron_variant
rs1042183831	intron_variant
rs1042184480	genic_downstream_transcript_variant,intron_variant
rs1042188830	intron_variant
rs1042192416	genic_upstream_transcript_variant,intron_variant
rs1042252412	genic_downstream_transcript_variant,intron_variant
rs1042256787	intron_variant
rs1042313258	intron_variant
rs1042321479	intron_variant
rs1042328620	genic_upstream_transcript_variant,intron_variant
rs1042343576	intron_variant
rs1042364001	genic_upstream_transcript_variant,intron_variant
rs1042381614	genic_upstream_transcript_variant,intron_variant
rs1042393855	genic_upstream_transcript_variant,intron_variant
rs1042394876	genic_upstream_transcript_variant,intron_variant
rs1042512220	2KB_upstream_variant,genic_upstream_transcript_variant,upstream_transcript_variant,intron_variant
rs1042540370	genic_upstream_transcript_variant,intron_variant
rs1042546144	intron_variant
rs1042558664	genic_downstream_transcript_variant,intron_variant
rs1042582075	genic_downstream_transcript_variant,intron_variant
rs1042609132	genic_downstream_transcript_variant,intron_variant
rs1042664105	upstream_transcript_variant,genic_upstream_transcript_variant,intron_variant
rs1042674323	genic_upstream_transcript_variant,intron_variant
rs1042716568	intron_variant
rs1042717998	genic_upstream_transcript_variant,intron_variant
rs1042719370	upstream_transcript_variant,genic_upstream_transcript_variant,intron_variant
rs1042753565	intron_variant
rs1042763323	intron_variant
rs1042804993	intron_variant
rs1042838814	intron_variant
rs1042844471	intron_variant
rs1042862349	intron_variant
rs1042871389	genic_upstream_transcript_variant,intron_variant
rs1042922584	genic_downstream_transcript_variant,intron_variant
rs1042965118	intron_variant
rs1042993146	genic_downstream_transcript_variant,intron_variant
rs1043013675	intron_variant

rs1043054385	genic_upstream_transcript_variant,intron_variant
rs1043059956	genic_upstream_transcript_variant,intron_variant
rs1043063898	intron_variant
rs1043088645	genic_upstream_transcript_variant,intron_variant
rs1043116150	genic_upstream_transcript_variant,intron_variant
rs1043210829	intron_variant
rs1043267261	intron_variant
rs1043327639	genic_upstream_transcript_variant,intron_variant
rs1043338874	genic_upstream_transcript_variant,intron_variant
rs1043376078	intron_variant
rs1043411902	genic_upstream_transcript_variant,intron_variant
rs1043423174	genic_upstream_transcript_variant,intron_variant
rs1043432005	intron_variant
rs1043456596	intron_variant
rs1043521749	genic_upstream_transcript_variant,intron_variant
rs1043624334	genic_upstream_transcript_variant,intron_variant
rs1043676662	genic_upstream_transcript_variant,intron_variant
rs1043717215	genic_downstream_transcript_variant,intron_variant
rs1043777124	genic_downstream_transcript_variant,intron_variant
rs1043791312	intron_variant
rs1043839541	intron_variant
rs1043859824	genic_downstream_transcript_variant,intron_variant
rs1043866412	intron_variant
rs1043907705	genic_upstream_transcript_variant,intron_variant
rs1043914584	genic_upstream_transcript_variant,intron_variant
rs1043926428	genic_upstream_transcript_variant,intron_variant
rs1043931745	genic_downstream_transcript_variant,intron_variant
rs1043940149	genic_upstream_transcript_variant,intron_variant
rs1043941272	genic_upstream_transcript_variant,intron_variant
rs1043958781	genic_upstream_transcript_variant,intron_variant
rs1043977438	genic_upstream_transcript_variant,intron_variant
rs1044031827	genic_upstream_transcript_variant,intron_variant
rs1044064691	intron_variant
rs1044065855	2KB_upstream_variant,genic_upstream_transcript_variant,upstream_transcript_variant,intron_variant
rs1044096407	5_prime_UTR_variant,non_coding_transcript_variant,genic_upstream_transcript_variant,intron_variant
rs1044102479	genic_downstream_transcript_variant,intron_variant
rs1044112121	intron_variant
rs1044114913	genic_downstream_transcript_variant,intron_variant
rs1044151782	genic_upstream_transcript_variant,intron_variant
rs1044203064	genic_upstream_transcript_variant,intron_variant
rs1044216331	genic_upstream_transcript_variant,intron_variant
rs1044246534	genic_downstream_transcript_variant,intron_variant
rs1044263405	intron_variant
rs1044287384	genic_downstream_transcript_variant,intron_variant
rs1044323253	upstream_transcript_variant,genic_upstream_transcript_variant,intron_variant
rs1044331420	intron_variant
rs1044335345	intron_variant
rs1044380628	genic_upstream_transcript_variant,intron_variant
rs1044405350	intron_variant
rs1044441427	genic_upstream_transcript_variant,intron_variant
rs1044455261	genic_upstream_transcript_variant,intron_variant
rs1044455778	intron_variant
rs1044490663	genic_upstream_transcript_variant,intron_variant
rs1044541563	genic_upstream_transcript_variant,intron_variant
rs1044549495	genic_upstream_transcript_variant,intron_variant
rs1044566601	intron_variant
rs1044600148	intron_variant
rs1044616410	coding_sequence_variant,non_coding_transcript_variant,synonymous_variant,genic_upstream_transcript_variant,intron_variant
rs1044636210	intron_variant
rs1044654231	intron_variant
rs1044673640	intron_variant
rs1044710199	intron_variant
rs1044724117	genic_upstream_transcript_variant,intron_variant
rs1044741459	intron_variant
rs1044777916	genic_upstream_transcript_variant,intron_variant
rs1044791827	genic_upstream_transcript_variant,intron_variant
rs1044794191	intron_variant
rs1044802714	intron_variant
rs1044823415	genic_downstream_transcript_variant,intron_variant
rs1044841579	genic_upstream_transcript_variant,intron_variant
rs1044855801	intron_variant
rs1044872868	genic_upstream_transcript_variant,intron_variant
rs1044901844	genic_upstream_transcript_variant,intron_variant
rs1044938124	intron_variant

rs1044966393	intron_variant
rs1044967809	genic_downstream_transcript_variant,intron_variant
rs1044969976	intron_variant
rs1045025593	genic_downstream_transcript_variant,intron_variant
rs1045066002	genic_downstream_transcript_variant,intron_variant
rs1045066985	intron_variant
rs1045100213	genic_upstream_transcript_variant,intron_variant
rs1045126981	genic_upstream_transcript_variant,intron_variant
rs1045133464	genic_downstream_transcript_variant,intron_variant
rs1045133691	genic_upstream_transcript_variant,intron_variant
rs1045148901	intron_variant
rs1045188143	genic_downstream_transcript_variant,intron_variant
rs1045204938	intron_variant
rs1045207259	genic_downstream_transcript_variant,intron_variant
rs1045207332	genic_downstream_transcript_variant,intron_variant
rs1045217828	intron_variant
rs1045222640	genic_upstream_transcript_variant,intron_variant
rs1045304873	intron_variant
rs1045307158	genic_upstream_transcript_variant,intron_variant
rs1045342179	genic_downstream_transcript_variant,intron_variant
rs1045350796	intron_variant
rs1045381851	intron_variant
rs1045408849	2KB_upstream_variant,genic_upstream_transcript_variant,upstream_transcript_variant,intron_variant
rs1045419307	genic_downstream_transcript_variant,intron_variant
rs1045448822	genic_upstream_transcript_variant,intron_variant
rs1045470343	genic_downstream_transcript_variant,intron_variant
rs1045483135	genic_upstream_transcript_variant,intron_variant
rs1045483910	genic_downstream_transcript_variant,intron_variant
rs1045485187	intron_variant
rs1045520126	genic_downstream_transcript_variant,intron_variant
rs1045541260	genic_downstream_transcript_variant,intron_variant
rs1045636106	genic_downstream_transcript_variant,non_coding_transcript_variant,3_prime_UTR_variant
rs1045667680	genic_upstream_transcript_variant,intron_variant
rs1045674282	intron_variant
rs1045723046	intron_variant
rs1045739680	genic_upstream_transcript_variant,intron_variant
rs1045743701	genic_upstream_transcript_variant,intron_variant
rs1045746059	genic_downstream_transcript_variant,intron_variant
rs1045750572	genic_upstream_transcript_variant,intron_variant
rs1045791936	genic_upstream_transcript_variant,intron_variant
rs1045806858	genic_upstream_transcript_variant,intron_variant
rs1045809520	genic_downstream_transcript_variant,intron_variant
rs1045887472	genic_downstream_transcript_variant,intron_variant
rs1045906455	genic_upstream_transcript_variant,intron_variant
rs1045939518	intron_variant
rs1045954622	intron_variant
rs1045962858	genic_upstream_transcript_variant,intron_variant
rs1045986309	intron_variant
rs1046021524	genic_upstream_transcript_variant,intron_variant
rs1046050593	genic_upstream_transcript_variant,intron_variant
rs1046058900	genic_upstream_transcript_variant,intron_variant
rs1046082065	genic_downstream_transcript_variant,intron_variant
rs1046089920	genic_downstream_transcript_variant,intron_variant
rs1046170388	intron_variant
rs1046213948	genic_upstream_transcript_variant,intron_variant
rs1046214518	genic_downstream_transcript_variant,intron_variant
rs1046230978	intron_variant
rs1046264946	intron_variant
rs1046301026	intron_variant
rs1046334826	genic_downstream_transcript_variant,intron_variant
rs1046356799	genic_downstream_transcript_variant,non_coding_transcript_variant,3_prime_UTR_variant
rs1046377635	intron_variant
rs1046379351	intron_variant
rs1046407556	genic_upstream_transcript_variant,intron_variant
rs1046415765	genic_upstream_transcript_variant,intron_variant
rs1046422663	intron_variant
rs1046435889	intron_variant
rs1046442829	genic_downstream_transcript_variant,intron_variant
rs1046457433	intron_variant
rs1046477775	intron_variant
rs1046486968	intron_variant
rs1046503177	genic_upstream_transcript_variant,intron_variant
rs1046533701	intron_variant
rs1046546917	genic_upstream_transcript_variant,intron_variant

rs1046556653	genic_downstream_transcript_variant,intron_variant
rs1046595846	intron_variant
rs1046629975	genic_downstream_transcript_variant,downstream_transcript_variant,intron_variant
rs1046732561	genic_upstream_transcript_variant,intron_variant
rs1046770380	genic_upstream_transcript_variant,intron_variant
rs1046784372	intron_variant
rs1046785066	genic_downstream_transcript_variant,intron_variant
rs1046792710	genic_upstream_transcript_variant,intron_variant
rs1046843203	intron_variant
rs1046869285	genic_downstream_transcript_variant,non_coding_transcript_variant,3_prime_UTR_variant
rs1046884772	genic_downstream_transcript_variant,intron_variant
rs1046912313	genic_upstream_transcript_variant,intron_variant
rs1046922089	intron_variant
rs1046976549	intron_variant
rs1046990957	genic_downstream_transcript_variant,intron_variant
rs1047015800	intron_variant
rs1047021156	genic_downstream_transcript_variant,intron_variant
rs1047052531	genic_downstream_transcript_variant,intron_variant
rs1047062186	genic_upstream_transcript_variant,intron_variant
rs1047067876	intron_variant
rs1047076947	genic_downstream_transcript_variant,intron_variant
rs1047105967	genic_upstream_transcript_variant,intron_variant
rs1047136628	genic_upstream_transcript_variant,intron_variant
rs1047230756	intron_variant
rs1047233791	genic_upstream_transcript_variant,intron_variant
rs1047239673	intron_variant
rs1047264194	genic_upstream_transcript_variant,intron_variant
rs1047270424	intron_variant
rs1047273629	genic_upstream_transcript_variant,intron_variant
rs1047348367	genic_upstream_transcript_variant,intron_variant
rs1047374745	genic_downstream_transcript_variant,intron_variant
rs1047405334	intron_variant
rs1047413498	intron_variant
rs1047420659	genic_upstream_transcript_variant,intron_variant
rs1047437236	intron_variant
rs1047442060	genic_upstream_transcript_variant,intron_variant
rs1047448835	genic_downstream_transcript_variant,intron_variant
rs1047474188	genic_upstream_transcript_variant,intron_variant
rs1047485150	intron_variant
rs1047532844	genic_upstream_transcript_variant,intron_variant
rs1047573355	genic_upstream_transcript_variant,intron_variant
rs1047573757	intron_variant
rs1047583590	genic_downstream_transcript_variant,intron_variant
rs1047605159	intron_variant
rs1047610625	genic_upstream_transcript_variant,intron_variant
rs1047639092	intron_variant
rs1047678961	genic_upstream_transcript_variant,intron_variant
rs1047701962	genic_upstream_transcript_variant,intron_variant
rs1047760118	intron_variant
rs1047795499	intron_variant
rs1047843178	genic_upstream_transcript_variant,intron_variant
rs1047866755	genic_upstream_transcript_variant,intron_variant
rs1047868970	genic_downstream_transcript_variant,intron_variant
rs1047881239	genic_downstream_transcript_variant,intron_variant
rs1047887902	intron_variant
rs1047910117	genic_downstream_transcript_variant,intron_variant
rs1047934070	genic_downstream_transcript_variant,intron_variant
rs1047949848	intron_variant
rs1047967416	genic_upstream_transcript_variant,intron_variant
rs1047990709	genic_upstream_transcript_variant,intron_variant
rs1047996544	genic_downstream_transcript_variant,non_coding_transcript_variant,3_prime_UTR_variant
rs1048024020	genic_upstream_transcript_variant,intron_variant
rs1048065386	intron_variant
rs1048078133	intron_variant
rs1048127071	intron_variant
rs1048137433	intron_variant
rs1048223830	genic_upstream_transcript_variant,intron_variant
rs1048228391	genic_downstream_transcript_variant,intron_variant
rs1048230704	intron_variant
rs1048234173	genic_upstream_transcript_variant,intron_variant
rs1048281308	intron_variant,genic_downstream_transcript_variant
rs1048289634	intron_variant
rs1048328305	intron_variant,genic_upstream_transcript_variant
rs1048345289	intron_variant,genic_upstream_transcript_variant

rs1048357161	intron_variant
rs1048370601	intron_variant
rs1048375922	intron_variant,genic_upstream_transcript_variant
rs1048415804	intron_variant
rs1048429175	intron_variant,genic_upstream_transcript_variant
rs1048445609	intron_variant,genic_downstream_transcript_variant
rs1048455667	intron_variant
rs1048484266	intron_variant,genic_upstream_transcript_variant
rs1048517150	intron_variant,genic_downstream_transcript_variant
rs1048552423	intron_variant
rs1048556984	upstream_transcript_variant,intron_variant,genic_upstream_transcript_variant,2KB_upstream_variant
rs1048567921	intron_variant,genic_downstream_transcript_variant
rs1048601295	intron_variant,genic_downstream_transcript_variant
rs1048620823	intron_variant,genic_upstream_transcript_variant
rs1048627583	intron_variant,genic_upstream_transcript_variant
rs1048636319	intron_variant
rs1048698840	intron_variant
rs1048723230	intron_variant,genic_upstream_transcript_variant
rs1048739580	intron_variant,genic_downstream_transcript_variant
rs1048756393	upstream_transcript_variant,intron_variant,genic_upstream_transcript_variant,2KB_upstream_variant
rs1048764438	intron_variant
rs1048796964	intron_variant
rs1048810074	intron_variant,genic_downstream_transcript_variant
rs1048814462	intron_variant
rs1048851644	intron_variant,genic_downstream_transcript_variant
rs1048874220	intron_variant,genic_downstream_transcript_variant
rs1048885544	intron_variant,genic_upstream_transcript_variant
rs1048905890	intron_variant,genic_downstream_transcript_variant
rs1048916632	intron_variant
rs1048957308	intron_variant,genic_upstream_transcript_variant
rs1048959391	intron_variant,genic_upstream_transcript_variant
rs1048963907	intron_variant
rs1048972052	intron_variant
rs1049045633	intron_variant
rs1049070333	intron_variant
rs1049073147	upstream_transcript_variant,intron_variant,genic_upstream_transcript_variant,2KB_upstream_variant
rs1049079343	upstream_transcript_variant,intron_variant,genic_upstream_transcript_variant,2KB_upstream_variant
rs1049118224	intron_variant
rs1049149315	intron_variant
rs1049175151	intron_variant,genic_upstream_transcript_variant
rs1049273278	intron_variant,genic_upstream_transcript_variant
rs1049322936	intron_variant,genic_upstream_transcript_variant
rs1049351189	intron_variant
rs1049403365	intron_variant
rs1049423608	intron_variant,genic_downstream_transcript_variant
rs1049434320	upstream_transcript_variant,intron_variant,genic_upstream_transcript_variant,2KB_upstream_variant
rs1049479719	intron_variant,genic_upstream_transcript_variant
rs1049506782	upstream_transcript_variant,intron_variant,genic_upstream_transcript_variant,2KB_upstream_variant
rs1049588083	intron_variant
rs1049611678	intron_variant,genic_upstream_transcript_variant
rs1049615580	intron_variant,genic_downstream_transcript_variant
rs1049669148	intron_variant,genic_upstream_transcript_variant
rs1049690470	intron_variant
rs1049698292	intron_variant,genic_downstream_transcript_variant
rs1049715691	upstream_transcript_variant,intron_variant,genic_upstream_transcript_variant,2KB_upstream_variant
rs1049723970	intron_variant
rs1049735599	intron_variant,genic_upstream_transcript_variant
rs1049740748	intron_variant
rs1049754538	intron_variant
rs1049786435	intron_variant,genic_upstream_transcript_variant
rs1049838936	intron_variant,genic_upstream_transcript_variant
rs1049847675	intron_variant,genic_upstream_transcript_variant
rs1049862443	intron_variant,genic_downstream_transcript_variant
rs1049865012	intron_variant,genic_downstream_transcript_variant
rs1049916748	intron_variant
rs1049987046	intron_variant,genic_upstream_transcript_variant
rs1050002265	intron_variant,genic_upstream_transcript_variant
rs1050005124	intron_variant,genic_upstream_transcript_variant
rs1050030554	intron_variant,genic_downstream_transcript_variant
rs1050084807	intron_variant
rs1050132994	upstream_transcript_variant,intron_variant,genic_upstream_transcript_variant,2KB_upstream_variant
rs1050142433	intron_variant
rs1050172567	intron_variant,genic_upstream_transcript_variant
rs1050178210	intron_variant

rs1050190073	intron_variant,genic_downstream_transcript_variant
rs1050197733	intron_variant,genic_downstream_transcript_variant
rs1050218370	intron_variant,genic_upstream_transcript_variant
rs1050224844	intron_variant,genic_upstream_transcript_variant
rs1050248741	intron_variant,genic_upstream_transcript_variant
rs1050274370	intron_variant,genic_upstream_transcript_variant
rs1050299214	intron_variant
rs1050334732	intron_variant,genic_downstream_transcript_variant
rs1050351221	intron_variant
rs1050396275	intron_variant,genic_upstream_transcript_variant
rs1050436532	intron_variant
rs1050438286	intron_variant
rs1050474893	intron_variant,genic_upstream_transcript_variant
rs1050476709	upstream_transcript_variant,intron_variant,genic_upstream_transcript_variant,2KB_upstream_variant
rs1050516424	non_coding_transcript_variant,genic_downstream_transcript_variant,3_prime_UTR_variant
rs1050550558	intron_variant,genic_upstream_transcript_variant
rs1050567203	intron_variant,genic_downstream_transcript_variant
rs1050585218	intron_variant,genic_upstream_transcript_variant
rs1050604164	intron_variant,genic_upstream_transcript_variant
rs1050668693	intron_variant,genic_downstream_transcript_variant
rs1050678120	intron_variant
rs1050680071	intron_variant
rs1050696282	intron_variant,genic_upstream_transcript_variant
rs1050733636	intron_variant
rs1050750751	intron_variant,genic_upstream_transcript_variant
rs1050772007	intron_variant,genic_upstream_transcript_variant
rs1050781383	intron_variant
rs1050804515	intron_variant
rs1050805255	intron_variant,genic_downstream_transcript_variant
rs1050822862	intron_variant,genic_upstream_transcript_variant
rs1050898988	intron_variant,genic_downstream_transcript_variant
rs1050908063	intron_variant
rs1050914392	intron_variant,genic_upstream_transcript_variant
rs1050917546	intron_variant
rs1050935677	intron_variant,genic_upstream_transcript_variant
rs1050949595	intron_variant
rs1050966360	intron_variant
rs1050988473	intron_variant,genic_upstream_transcript_variant
rs1050999999	intron_variant,genic_downstream_transcript_variant
rs1051037610	intron_variant,genic_upstream_transcript_variant
rs1051055293	intron_variant
rs1051066910	non_coding_transcript_variant,genic_downstream_transcript_variant,3_prime_UTR_variant
rs1051075183	intron_variant
rs1051118510	intron_variant
rs1051127966	intron_variant
rs1051129105	intron_variant
rs1051167963	intron_variant,genic_upstream_transcript_variant
rs1051186173	intron_variant,genic_upstream_transcript_variant
rs1051199410	upstream_transcript_variant,intron_variant,genic_upstream_transcript_variant,2KB_upstream_variant
rs1051203584	intron_variant,genic_upstream_transcript_variant
rs1051239214	intron_variant,genic_upstream_transcript_variant
rs1051323364	intron_variant,genic_downstream_transcript_variant
rs1051336410	intron_variant,genic_downstream_transcript_variant
rs1051363064	intron_variant
rs1051409211	intron_variant
rs1051429032	intron_variant,genic_upstream_transcript_variant
rs1051460780	intron_variant
rs1051484424	intron_variant,genic_upstream_transcript_variant
rs1051529416	intron_variant,genic_upstream_transcript_variant
rs1051545066	intron_variant,genic_downstream_transcript_variant
rs1051576079	intron_variant,genic_upstream_transcript_variant
rs1051604454	intron_variant,genic_upstream_transcript_variant
rs1051646130	intron_variant
rs1051665582	non_coding_transcript_variant,genic_downstream_transcript_variant,3_prime_UTR_variant
rs1051674774	intron_variant
rs1051684442	intron_variant
rs1051693864	intron_variant,genic_upstream_transcript_variant
rs1051701590	intron_variant
rs1051722054	intron_variant,genic_upstream_transcript_variant
rs1051726763	intron_variant
rs1051746273	intron_variant
rs1051753885	intron_variant,genic_upstream_transcript_variant
rs1051761067	upstream_transcript_variant,intron_variant,genic_upstream_transcript_variant,2KB_upstream_variant
rs1051836897	intron_variant

rs1051843897	intron_variant,genic_upstream_transcript_variant,upstream_transcript_variant
rs1051901766	intron_variant,genic_upstream_transcript_variant
rs1051906055	intron_variant,genic_upstream_transcript_variant
rs1051910873	intron_variant
rs1051937694	downstream_transcript_variant,500B_downstream_variant
rs1051947815	intron_variant,genic_downstream_transcript_variant
rs1051964666	intron_variant,genic_upstream_transcript_variant
rs1051967877	intron_variant
rs1052009692	intron_variant,genic_upstream_transcript_variant
rs1052025658	intron_variant,genic_upstream_transcript_variant
rs1052066154	intron_variant
rs1052089014	intron_variant
rs1052120618	intron_variant,genic_downstream_transcript_variant
rs1052125114	intron_variant,genic_downstream_transcript_variant
rs1052134793	intron_variant,genic_upstream_transcript_variant
rs1052161955	intron_variant
rs1052171459	intron_variant,genic_downstream_transcript_variant
rs1052182877	intron_variant,genic_downstream_transcript_variant
rs1052214303	intron_variant,genic_upstream_transcript_variant
rs1052259775	intron_variant,genic_upstream_transcript_variant
rs1052301130	intron_variant,genic_upstream_transcript_variant
rs1052339679	missense_variant,intron_variant,coding_sequence_variant
rs1052344186	intron_variant
rs1052354725	intron_variant,genic_upstream_transcript_variant
rs1052449918	intron_variant,genic_downstream_transcript_variant
rs1052469429	intron_variant
rs1052482860	intron_variant,genic_downstream_transcript_variant
rs1052538729	intron_variant
rs1052542229	intron_variant,genic_upstream_transcript_variant
rs1052559367	intron_variant,genic_upstream_transcript_variant
rs1052588101	intron_variant
rs1052618052	upstream_transcript_variant,intron_variant,genic_upstream_transcript_variant,2KB_upstream_variant
rs1052628449	intron_variant,genic_upstream_transcript_variant
rs1052652560	intron_variant,genic_downstream_transcript_variant
rs1052679732	intron_variant
rs1052753393	intron_variant,genic_upstream_transcript_variant
rs1052775364	intron_variant,genic_downstream_transcript_variant
rs1052865716	intron_variant
rs1052873444	intron_variant
rs1052918561	intron_variant
rs1052922424	intron_variant
rs1052954313	intron_variant,genic_downstream_transcript_variant
rs1052964457	intron_variant
rs1052993344	intron_variant,genic_downstream_transcript_variant
rs1052998107	intron_variant,genic_upstream_transcript_variant
rs1053032890	intron_variant
rs1053033948	intron_variant,genic_upstream_transcript_variant
rs1053041007	intron_variant,genic_downstream_transcript_variant
rs1053079909	intron_variant,genic_upstream_transcript_variant
rs1053085626	intron_variant
rs1053136956	intron_variant,genic_upstream_transcript_variant
rs1053205284	intron_variant
rs1053222304	intron_variant
rs1053227376	intron_variant
rs1053244860	intron_variant
rs1053257260	intron_variant
rs1053330555	intron_variant,genic_upstream_transcript_variant
rs1053339183	intron_variant,genic_downstream_transcript_variant
rs1053344678	downstream_transcript_variant,500B_downstream_variant
rs1053372287	intron_variant,genic_downstream_transcript_variant
rs1053383145	intron_variant
rs1053384463	intron_variant,genic_upstream_transcript_variant
rs1053416348	intron_variant
rs1053492753	intron_variant,genic_upstream_transcript_variant
rs1053539137	intron_variant
rs1053547626	intron_variant
rs1053553454	intron_variant,genic_upstream_transcript_variant
rs1053558058	intron_variant
rs1053594594	intron_variant
rs1053614129	upstream_transcript_variant,intron_variant,genic_upstream_transcript_variant,2KB_upstream_variant
rs1053677874	intron_variant,genic_upstream_transcript_variant
rs1053709928	intron_variant,genic_downstream_transcript_variant
rs1053717328	intron_variant,genic_downstream_transcript_variant
rs1053744046	intron_variant

rs1053795641	intron_variant
rs1053819647	intron_variant,genic_upstream_transcript_variant
rs1053825912	intron_variant
rs1053834502	intron_variant,genic_upstream_transcript_variant
rs1053874864	intron_variant,genic_upstream_transcript_variant
rs1053890103	intron_variant
rs1053993399	intron_variant,genic_upstream_transcript_variant
rs1054021893	intron_variant,genic_upstream_transcript_variant
rs1054053289	intron_variant
rs1054056652	intron_variant
rs1054064442	intron_variant,genic_downstream_transcript_variant
rs1054086318	intron_variant
rs1054107323	upstream_transcript_variant,intron_variant,genic_upstream_transcript_variant,2KB_upstream_variant
rs1054136485	intron_variant,genic_upstream_transcript_variant
rs1054182885	intron_variant,genic_upstream_transcript_variant
rs1054184097	intron_variant,genic_upstream_transcript_variant
rs1054186353	upstream_transcript_variant,intron_variant,genic_upstream_transcript_variant,2KB_upstream_variant
rs1054187045	intron_variant
rs1054228455	upstream_transcript_variant,intron_variant,genic_upstream_transcript_variant,2KB_upstream_variant
rs1054255212	intron_variant
rs1054261308	upstream_transcript_variant,intron_variant,genic_upstream_transcript_variant,2KB_upstream_variant
rs1054289040	intron_variant
rs1054338312	intron_variant,genic_upstream_transcript_variant
rs1054359147	intron_variant
rs1054409722	intron_variant,genic_upstream_transcript_variant
rs1054418241	intron_variant,genic_upstream_transcript_variant
rs1054420847	intron_variant,genic_upstream_transcript_variant
rs1054420982	intron_variant,genic_upstream_transcript_variant
rs1054461545	intron_variant,genic_downstream_transcript_variant
rs1054467358	intron_variant
rs1054484503	intron_variant,genic_downstream_transcript_variant
rs1054488209	intron_variant
rs1054536581	intron_variant
rs1054551997	intron_variant
rs1054556630	intron_variant,genic_upstream_transcript_variant
rs1054573748	intron_variant
rs1054590164	intron_variant
rs1054603843	intron_variant
rs1054609836	intron_variant
rs1054617374	intron_variant,genic_upstream_transcript_variant
rs1054646057	upstream_transcript_variant,intron_variant,genic_upstream_transcript_variant,2KB_upstream_variant
rs1054674159	intron_variant,genic_downstream_transcript_variant
rs1054694333	intron_variant,genic_downstream_transcript_variant
rs1054712577	intron_variant
rs1054713562	intron_variant,genic_upstream_transcript_variant
rs1054749449	intron_variant
rs1054779569	intron_variant,genic_upstream_transcript_variant
rs1054784283	intron_variant,genic_upstream_transcript_variant
rs1054792843	intron_variant,genic_upstream_transcript_variant
rs1054809891	intron_variant,genic_downstream_transcript_variant
rs1054834161	intron_variant,genic_upstream_transcript_variant
rs1054893049	intron_variant
rs1054917969	intron_variant
rs1054944377	intron_variant
rs1054946730	intron_variant,genic_downstream_transcript_variant
rs1054966427	intron_variant,genic_downstream_transcript_variant
rs1054976834	intron_variant,genic_downstream_transcript_variant
rs1054983801	intron_variant,genic_upstream_transcript_variant
rs1055004292	intron_variant,genic_upstream_transcript_variant
rs1055068754	intron_variant,genic_downstream_transcript_variant
rs1055113478	intron_variant,genic_downstream_transcript_variant
rs1055118876	intron_variant
rs1055133588	intron_variant,genic_upstream_transcript_variant
rs1055136932	intron_variant
rs1055162438	intron_variant,genic_upstream_transcript_variant
rs1055172794	intron_variant
rs1055195554	intron_variant
rs1055220159	intron_variant,genic_upstream_transcript_variant
rs1055238997	intron_variant
rs1055307219	intron_variant,genic_upstream_transcript_variant
rs1055329382	intron_variant,genic_upstream_transcript_variant
rs1055362374	intron_variant
rs1055398159	intron_variant,genic_downstream_transcript_variant
rs1055398641	intron_variant,genic_upstream_transcript_variant

rs1055404992	intron_variant
rs1055451166	intron_variant,genic_downstream_transcript_variant
rs1055480538	intron_variant,genic_upstream_transcript_variant
rs1055498170	non_coding_transcript_variant,genic_downstream_transcript_variant,3_prime_UTR_variant
rs1055505715	intron_variant,genic_upstream_transcript_variant
rs1055549155	intron_variant,genic_upstream_transcript_variant
rs1055562088	intron_variant
rs1055591245	intron_variant
rs1055662378	intron_variant
rs1055662873	intron_variant,genic_upstream_transcript_variant
rs1055682499	intron_variant
rs1055690952	intron_variant,genic_upstream_transcript_variant
rs1055698527	intron_variant
rs1055736429	non_coding_transcript_variant,genic_downstream_transcript_variant,3_prime_UTR_variant
rs1055804865	intron_variant
rs1055815048	intron_variant,genic_downstream_transcript_variant
rs1055815225	intron_variant,genic_downstream_transcript_variant
rs1055825686	intron_variant
rs1055866056	intron_variant,genic_downstream_transcript_variant
rs1055892368	intron_variant,genic_upstream_transcript_variant
rs1055923200	intron_variant
rs1055967819	intron_variant,genic_downstream_transcript_variant
rs1055989862	intron_variant,genic_upstream_transcript_variant
rs1055990878	intron_variant
rs1056021222	upstream_transcript_variant,intron_variant,genic_upstream_transcript_variant,2KB_upstream_variant
rs1056023521	upstream_transcript_variant,intron_variant,genic_upstream_transcript_variant,2KB_upstream_variant
rs1056027998	intron_variant,genic_upstream_transcript_variant
rs1056033574	intron_variant,genic_upstream_transcript_variant
rs1056087456	intron_variant,genic_upstream_transcript_variant
rs1056099425	upstream_transcript_variant,intron_variant,genic_upstream_transcript_variant,2KB_upstream_variant
rs1056131781	intron_variant,genic_upstream_transcript_variant
rs1056134141	intron_variant,genic_upstream_transcript_variant
rs1056135900	intron_variant
rs1056154204	intron_variant,genic_downstream_transcript_variant
rs1056207802	intron_variant
rs1056262733	intron_variant
rs1056273991	intron_variant
rs1056323289	intron_variant,genic_upstream_transcript_variant
rs1056370439	intron_variant,genic_upstream_transcript_variant
rs1056420173	intron_variant,genic_upstream_transcript_variant
rs1056434556	intron_variant,genic_upstream_transcript_variant
rs1056435196	intron_variant,genic_upstream_transcript_variant
rs1056435276	intron_variant,genic_upstream_transcript_variant
rs1056470566	intron_variant
rs1056473248	intron_variant,genic_downstream_transcript_variant
rs1056495021	intron_variant,genic_downstream_transcript_variant
rs1056500661	intron_variant,genic_upstream_transcript_variant
rs1056513304	upstream_transcript_variant,intron_variant,genic_upstream_transcript_variant,2KB_upstream_variant
rs1056558517	intron_variant
rs1056647701	intron_variant,genic_upstream_transcript_variant
rs1056651403	intron_variant,genic_downstream_transcript_variant
rs1056667134	intron_variant
rs1056680599	intron_variant
rs1056691373	intron_variant,genic_downstream_transcript_variant
rs1056704977	intron_variant,genic_upstream_transcript_variant
rs1056773744	intron_variant,genic_upstream_transcript_variant
rs1056789824	intron_variant
rs1056810652	intron_variant
rs1056815382	upstream_transcript_variant,intron_variant,genic_upstream_transcript_variant,2KB_upstream_variant
rs1056831602	intron_variant,genic_downstream_transcript_variant
rs1056832662	intron_variant,genic_upstream_transcript_variant
rs1056858110	intron_variant,genic_upstream_transcript_variant
rs1056866527	intron_variant
rs1056880982	upstream_transcript_variant,intron_variant,genic_upstream_transcript_variant,2KB_upstream_variant
rs1056891764	intron_variant
rs1056924867	intron_variant
rs1056954782	intron_variant
rs1056968216	intron_variant
rs1056986535	intron_variant,genic_downstream_transcript_variant
rs1057000500	intron_variant
rs1057035787	intron_variant,genic_upstream_transcript_variant
rs1057037700	intron_variant,genic_upstream_transcript_variant
rs1057058159	intron_variant,genic_upstream_transcript_variant
rs1057089790	intron_variant,genic_upstream_transcript_variant,upstream_transcript_variant

rs1057097764	intron_variant,genic_upstream_transcript_variant
rs1057109191	intron_variant,genic_upstream_transcript_variant
rs1057136692	intron_variant,genic_upstream_transcript_variant
rs1057155138	intron_variant,genic_downstream_transcript_variant
rs1057159493	intron_variant,genic_upstream_transcript_variant
rs1057161088	intron_variant,genic_upstream_transcript_variant,upstream_transcript_variant
rs1057189642	intron_variant,genic_upstream_transcript_variant
rs1057189829	intron_variant,genic_upstream_transcript_variant
rs1057202757	intron_variant
rs1057263101	intron_variant
rs1057304569	intron_variant,genic_upstream_transcript_variant
rs1057321793	upstream_transcript_variant,intron_variant,genic_upstream_transcript_variant,2KB_upstream_variant
rs1057330863	intron_variant,genic_downstream_transcript_variant
rs1057332042	intron_variant,genic_upstream_transcript_variant
rs1057344286	intron_variant,genic_upstream_transcript_variant
rs1057402061	intron_variant,genic_downstream_transcript_variant
rs1057430424	intron_variant,genic_upstream_transcript_variant
rs1057454981	intron_variant
rs1057465803	intron_variant
rs1057482679	intron_variant,genic_downstream_transcript_variant
rs1057493280	intron_variant,genic_upstream_transcript_variant
rs1057497531	intron_variant,genic_upstream_transcript_variant
rs1156238219	intron_variant,genic_downstream_transcript_variant
rs1156263180	intron_variant,genic_downstream_transcript_variant
rs1156265004	intron_variant,genic_upstream_transcript_variant
rs1156270600	intron_variant,genic_upstream_transcript_variant
rs1156314582	intron_variant,genic_downstream_transcript_variant
rs1156316060	intron_variant,genic_downstream_transcript_variant
rs1156331544	intron_variant,genic_downstream_transcript_variant
rs1156368119	intron_variant,genic_upstream_transcript_variant
rs1156375618	intron_variant
rs1156430932	intron_variant
rs1156431640	non_coding_transcript_variant,genic_downstream_transcript_variant,3_prime_UTR_variant
rs1156447103	intron_variant
rs1156449068	intron_variant,genic_upstream_transcript_variant
rs1156472670	intron_variant,genic_upstream_transcript_variant
rs1156499984	intron_variant,genic_upstream_transcript_variant
rs1156532089	intron_variant
rs1156554766	intron_variant,genic_upstream_transcript_variant
rs1156562216	intron_variant
rs1156633663	intron_variant,genic_upstream_transcript_variant
rs1156722075	intron_variant,genic_upstream_transcript_variant
rs1156748589	upstream_transcript_variant,intron_variant,genic_upstream_transcript_variant,2KB_upstream_variant
rs1156759729	intron_variant,genic_upstream_transcript_variant
rs1156786664	intron_variant
rs1156790048	intron_variant,genic_downstream_transcript_variant
rs1156795390	intron_variant
rs1156833196	intron_variant,genic_upstream_transcript_variant
rs1156839096	intron_variant,genic_upstream_transcript_variant
rs1156842060	intron_variant,genic_downstream_transcript_variant
rs1156860506	intron_variant,genic_upstream_transcript_variant
rs1156887354	intron_variant
rs1156914826	intron_variant
rs1156986130	intron_variant
rs1156988845	intron_variant,genic_upstream_transcript_variant,upstream_transcript_variant
rs1157046106	intron_variant,genic_upstream_transcript_variant
rs1157087250	intron_variant,genic_upstream_transcript_variant
rs1157087962	intron_variant,genic_downstream_transcript_variant
rs1157092020	intron_variant
rs1157114624	intron_variant,genic_upstream_transcript_variant
rs1157140040	intron_variant,genic_upstream_transcript_variant
rs1157157520	intron_variant,genic_upstream_transcript_variant
rs1157169853	intron_variant,genic_upstream_transcript_variant
rs1157251036	missense_variant,non_coding_transcript_variant,intron_variant,genic_upstream_transcript_variant,coding_sequence_variant
rs1157252951	intron_variant,genic_upstream_transcript_variant
rs1157261740	intron_variant
rs1157270088	intron_variant
rs1157309729	non_coding_transcript_variant,genic_downstream_transcript_variant,3_prime_UTR_variant
rs1157318663	intron_variant
rs1157320844	intron_variant,genic_downstream_transcript_variant
rs1157330646	intron_variant
rs1157355622	intron_variant,genic_upstream_transcript_variant
rs1157386531	intron_variant
rs1157416855	intron_variant

rs1157423377	non_coding_transcript_variant,genic_downstream_transcript_variant,3_prime_UTR_variant
rs1157510767	intron_variant,genic_upstream_transcript_variant,upstream_transcript_variant,5_prime_UTR_variant
rs1157512892	intron_variant
rs1157644794	intron_variant
rs1157738518	intron_variant
rs1157755668	intron_variant,genic_upstream_transcript_variant
rs1157759789	intron_variant
rs1157778365	intron_variant
rs1157793713	intron_variant,genic_upstream_transcript_variant,upstream_transcript_variant
rs1157809611	intron_variant
rs1157813214	intron_variant
rs1157841876	intron_variant,genic_downstream_transcript_variant
rs1157872878	intron_variant,genic_upstream_transcript_variant
rs1157884524	intron_variant,genic_upstream_transcript_variant,upstream_transcript_variant
rs1157886665	intron_variant,genic_downstream_transcript_variant
rs1157966283	intron_variant
rs1157997355	intron_variant,genic_downstream_transcript_variant
rs1158002068	intron_variant,genic_downstream_transcript_variant
rs1158021252	intron_variant,genic_downstream_transcript_variant
rs1158021755	missense_variant,non_coding_transcript_variant,genic_downstream_transcript_variant,coding_sequence_variant
rs1158036966	intron_variant,genic_upstream_transcript_variant
rs1158051781	intron_variant,genic_downstream_transcript_variant
rs1158107700	intron_variant,genic_upstream_transcript_variant
rs1158166362	intron_variant,genic_upstream_transcript_variant
rs1158188435	intron_variant,genic_upstream_transcript_variant,upstream_transcript_variant
rs1158201382	intron_variant
rs1158240013	intron_variant,genic_downstream_transcript_variant
rs1158248439	upstream_transcript_variant,intron_variant,genic_upstream_transcript_variant,2KB_upstream_variant
rs1158309588	intron_variant,genic_downstream_transcript_variant
rs1158324706	intron_variant,genic_upstream_transcript_variant
rs1158352400	intron_variant,genic_upstream_transcript_variant
rs1158414429	intron_variant,genic_upstream_transcript_variant
rs1158450342	intron_variant,genic_downstream_transcript_variant
rs1158464756	intron_variant,genic_downstream_transcript_variant
rs1158469759	intron_variant
rs1158471126	intron_variant,genic_upstream_transcript_variant
rs1158491142	intron_variant,genic_upstream_transcript_variant
rs1158492040	intron_variant,genic_upstream_transcript_variant
rs1158497354	intron_variant,genic_upstream_transcript_variant
rs1158541592	intron_variant,genic_upstream_transcript_variant
rs1158565250	intron_variant
rs1158573005	intron_variant,genic_downstream_transcript_variant
rs1158589650	intron_variant
rs1158607204	intron_variant,genic_upstream_transcript_variant
rs1158614455	intron_variant,genic_upstream_transcript_variant
rs1158623789	intron_variant,genic_downstream_transcript_variant
rs1158646059	intron_variant
rs1158647241	intron_variant,genic_downstream_transcript_variant
rs1158666409	intron_variant,genic_upstream_transcript_variant
rs1158674673	intron_variant,genic_downstream_transcript_variant
rs1158678854	intron_variant
rs1158715302	upstream_transcript_variant,intron_variant,genic_upstream_transcript_variant,2KB_upstream_variant
rs1158735034	missense_variant,non_coding_transcript_variant,genic_downstream_transcript_variant,coding_sequence_variant
rs1158740280	intron_variant
rs1158744600	intron_variant
rs1158751400	intron_variant
rs1158763978	intron_variant
rs1158766750	intron_variant,genic_upstream_transcript_variant
rs1158767214	intron_variant,genic_upstream_transcript_variant
rs1158837658	intron_variant,genic_downstream_transcript_variant
rs1158842973	intron_variant
rs1158855385	intron_variant,genic_upstream_transcript_variant
rs1158877865	intron_variant
rs1158929224	intron_variant,genic_upstream_transcript_variant
rs1158929330	intron_variant,genic_upstream_transcript_variant
rs1159023521	intron_variant
rs1159200071	intron_variant
rs1159264019	intron_variant
rs1159298491	genic_upstream_transcript_variant,intron_variant,5_prime_UTR_variant,non_coding_transcript_variant
rs1159300101	intron_variant,genic_downstream_transcript_variant
rs1159308129	intron_variant,genic_upstream_transcript_variant
rs1159412295	intron_variant,genic_upstream_transcript_variant
rs1159529716	intron_variant,genic_upstream_transcript_variant
rs1159545087	intron_variant,genic_upstream_transcript_variant,2KB_upstream_variant,upstream_transcript_variant

rs1159554633	synonymous_variant,non_coding_transcript_variant,coding_sequence_variant
rs1159565847	intron_variant,genic_upstream_transcript_variant
rs1159570309	intron_variant,genic_downstream_transcript_variant
rs1159582836	intron_variant
rs1159594769	intron_variant,genic_upstream_transcript_variant,upstream_transcript_variant
rs1159613194	intron_variant,genic_upstream_transcript_variant
rs1159614215	intron_variant
rs1159657196	intron_variant,genic_upstream_transcript_variant
rs1159662516	intron_variant,genic_downstream_transcript_variant
rs1159670216	intron_variant
rs1159753967	intron_variant,genic_upstream_transcript_variant
rs1159797317	intron_variant,genic_upstream_transcript_variant
rs1159847811	intron_variant,genic_downstream_transcript_variant
rs1159875470	intron_variant,genic_upstream_transcript_variant
rs1159879243	intron_variant,genic_upstream_transcript_variant
rs1159946417	intron_variant,genic_upstream_transcript_variant
rs1159957778	intron_variant
rs1159997008	intron_variant
rs1160014764	intron_variant
rs1160047047	synonymous_variant,non_coding_transcript_variant,coding_sequence_variant
rs1160061759	intron_variant,genic_upstream_transcript_variant,2KB_upstream_variant,upstream_transcript_variant
rs1160065525	intron_variant,genic_upstream_transcript_variant
rs1160080048	intron_variant,genic_upstream_transcript_variant
rs1160090283	intron_variant,genic_upstream_transcript_variant
rs1160137069	intron_variant,genic_upstream_transcript_variant
rs1160143509	intron_variant
rs1160155853	intron_variant,genic_upstream_transcript_variant
rs1160214491	intron_variant
rs1160286553	intron_variant,genic_upstream_transcript_variant
rs1160307326	intron_variant
rs1160344447	intron_variant
rs1160367169	intron_variant
rs1160371361	intron_variant,genic_upstream_transcript_variant
rs1160391862	intron_variant,genic_upstream_transcript_variant
rs1160393200	intron_variant,genic_downstream_transcript_variant
rs1160401756	intron_variant,genic_downstream_transcript_variant
rs1160439856	intron_variant
rs1160455701	intron_variant,genic_downstream_transcript_variant
rs1160465483	intron_variant
rs1160490211	genic_upstream_transcript_variant,intron_variant,2KB_upstream_variant,upstream_transcript_variant
rs1160494141	intron_variant
rs1160517226	intron_variant
rs1160520306	genic_upstream_transcript_variant,intron_variant,2KB_upstream_variant,upstream_transcript_variant
rs1160532363	intron_variant
rs1160549795	intron_variant
rs1160570568	intron_variant,genic_upstream_transcript_variant
rs1160590573	intron_variant,genic_downstream_transcript_variant
rs1160640871	intron_variant
rs1160652485	intron_variant
rs1160708482	intron_variant,genic_upstream_transcript_variant
rs1160803030	genic_upstream_transcript_variant,intron_variant,2KB_upstream_variant,upstream_transcript_variant
rs1160816487	intron_variant,genic_downstream_transcript_variant
rs1160821810	missense_variant,non_coding_transcript_variant,coding_sequence_variant
rs1160850346	intron_variant
rs1160890305	intron_variant
rs1160892136	intron_variant
rs1160927623	intron_variant,genic_upstream_transcript_variant
rs1160984039	intron_variant,genic_upstream_transcript_variant
rs1160991896	intron_variant
rs1161056019	non_coding_transcript_variant,genic_downstream_transcript_variant,3_prime_UTR_variant
rs1161062339	intron_variant,genic_upstream_transcript_variant
rs1161081035	intron_variant
rs1161112192	intron_variant,genic_downstream_transcript_variant
rs1161141504	intron_variant,genic_upstream_transcript_variant
rs1161148949	intron_variant,genic_upstream_transcript_variant
rs1161163868	intron_variant,genic_upstream_transcript_variant
rs1161189760	intron_variant
rs1161225498	intron_variant,genic_upstream_transcript_variant
rs1161240429	intron_variant,genic_upstream_transcript_variant,upstream_transcript_variant
rs1161252489	intron_variant,genic_upstream_transcript_variant
rs1161255319	intron_variant
rs1161269175	intron_variant,genic_upstream_transcript_variant
rs1161281761	intron_variant,genic_upstream_transcript_variant
rs1161302101	non_coding_transcript_variant,genic_downstream_transcript_variant,3_prime_UTR_variant

rs1161341747	intron_variant
rs1161425732	genic_upstream_transcript_variant,intron_variant,upstream_transcript_variant,5_prime_UTR_variant
rs1161435399	intron_variant,genic_downstream_transcript_variant
rs1161437762	intron_variant,genic_upstream_transcript_variant
rs1161474789	intron_variant,genic_upstream_transcript_variant
rs1161522847	intron_variant,genic_upstream_transcript_variant
rs1161539896	intron_variant,genic_upstream_transcript_variant
rs1161549710	intron_variant,genic_downstream_transcript_variant
rs1161590652	intron_variant,genic_upstream_transcript_variant
rs1161620619	intron_variant
rs1161649916	intron_variant,genic_upstream_transcript_variant
rs1161677915	intron_variant
rs1161702517	intron_variant
rs1161703269	non_coding_transcript_variant,genic_downstream_transcript_variant,3_prime_UTR_variant
rs1161704161	intron_variant,genic_upstream_transcript_variant
rs1161726560	intron_variant,genic_upstream_transcript_variant
rs1161745729	intron_variant
rs1161774092	intron_variant,genic_upstream_transcript_variant
rs1161780938	intron_variant,genic_upstream_transcript_variant
rs1161781865	intron_variant
rs1161805452	intron_variant
rs1161815786	intron_variant,genic_upstream_transcript_variant
rs1161860934	intron_variant,genic_upstream_transcript_variant
rs1161887725	intron_variant
rs1161946530	intron_variant,genic_upstream_transcript_variant
rs1161957215	intron_variant,genic_downstream_transcript_variant
rs1161961634	intron_variant
rs1161975783	intron_variant,genic_downstream_transcript_variant
rs1162012536	intron_variant,genic_upstream_transcript_variant
rs1162014535	intron_variant,genic_upstream_transcript_variant
rs1162057656	intron_variant,genic_upstream_transcript_variant,2KB_upstream_variant,upstream_transcript_variant
rs1162111244	intron_variant,genic_upstream_transcript_variant
rs1162116332	intron_variant
rs1162131277	intron_variant,genic_downstream_transcript_variant
rs1162135733	intron_variant,missense_variant,coding_sequence_variant
rs1162136618	intron_variant,genic_upstream_transcript_variant
rs1162174940	intron_variant,genic_downstream_transcript_variant
rs1162238827	intron_variant,genic_upstream_transcript_variant
rs1162243103	missense_variant,non_coding_transcript_variant,genic_downstream_transcript_variant,coding_sequence_variant
rs1162254003	intron_variant,genic_upstream_transcript_variant
rs1162255898	intron_variant
rs1162259085	intron_variant,genic_upstream_transcript_variant
rs1162302732	intron_variant,downstream_transcript_variant,genic_downstream_transcript_variant
rs1162325910	intron_variant,genic_downstream_transcript_variant
rs1162329543	intron_variant
rs1162399303	intron_variant,genic_downstream_transcript_variant
rs1162426350	intron_variant,genic_downstream_transcript_variant
rs1162438221	intron_variant,genic_downstream_transcript_variant
rs1162449876	intron_variant
rs1162452895	intron_variant,genic_upstream_transcript_variant
rs1162453053	intron_variant
rs1162527833	intron_variant,genic_upstream_transcript_variant
rs1162551251	intron_variant,genic_downstream_transcript_variant
rs1162551792	intron_variant
rs1162562378	intron_variant,genic_upstream_transcript_variant
rs1162570264	intron_variant,genic_downstream_transcript_variant
rs1162656180	intron_variant,genic_downstream_transcript_variant
rs1162659665	intron_variant
rs1162667777	intron_variant,genic_downstream_transcript_variant
rs1162691497	intron_variant
rs1162737036	intron_variant,genic_upstream_transcript_variant
rs1162751638	intron_variant,genic_upstream_transcript_variant
rs1162774981	synonymous_variant,non_coding_transcript_variant,coding_sequence_variant
rs1162811410	intron_variant
rs1162836630	intron_variant
rs1162869994	intron_variant
rs1162878755	intron_variant,genic_upstream_transcript_variant
rs1162893477	intron_variant
rs1162894470	intron_variant
rs1162899176	intron_variant,genic_upstream_transcript_variant
rs1162912353	intron_variant,genic_upstream_transcript_variant
rs1162943809	intron_variant,genic_upstream_transcript_variant
rs1162959071	intron_variant
rs1162960924	intron_variant

rs1162982170	intron_variant,genic_downstream_transcript_variant
rs1163002178	intron_variant,genic_upstream_transcript_variant
rs1163016730	intron_variant,genic_upstream_transcript_variant
rs1163026316	intron_variant
rs1163073886	intron_variant
rs1163121852	intron_variant,genic_downstream_transcript_variant
rs1163165818	intron_variant
rs1163166935	intron_variant
rs1163188018	intron_variant
rs1163198028	intron_variant,genic_upstream_transcript_variant
rs1163220097	intron_variant,genic_upstream_transcript_variant
rs1163315198	intron_variant,genic_downstream_transcript_variant
rs1163321111	intron_variant,genic_upstream_transcript_variant
rs1163328620	intron_variant,genic_upstream_transcript_variant
rs1163350845	intron_variant,genic_upstream_transcript_variant
rs1163374298	intron_variant,genic_upstream_transcript_variant
rs1163389741	intron_variant,genic_upstream_transcript_variant
rs1163395460	intron_variant,genic_upstream_transcript_variant
rs1163426074	intron_variant
rs1163518685	intron_variant,5_prime_UTR_variant
rs1163519736	intron_variant,genic_downstream_transcript_variant
rs1163526478	intron_variant,genic_upstream_transcript_variant
rs1163577342	non_coding_transcript_variant,genic_downstream_transcript_variant,3_prime_UTR_variant
rs1163592164	intron_variant
rs1163641776	intron_variant,genic_upstream_transcript_variant
rs1163731416	intron_variant,genic_upstream_transcript_variant,upstream_transcript_variant
rs1163772828	intron_variant,genic_upstream_transcript_variant
rs1163837953	intron_variant,genic_downstream_transcript_variant
rs1163883118	intron_variant,genic_upstream_transcript_variant
rs1163884679	genic_upstream_transcript_variant,intron_variant,2KB_upstream_variant,upstream_transcript_variant
rs1163888908	intron_variant,genic_upstream_transcript_variant
rs1163891306	intron_variant,genic_upstream_transcript_variant
rs1163953942	intron_variant
rs1163954354	intron_variant,genic_upstream_transcript_variant
rs1163959348	intron_variant
rs1164031065	intron_variant
rs1164040863	intron_variant,genic_downstream_transcript_variant
rs1164049554	intron_variant,genic_upstream_transcript_variant
rs1164113330	intron_variant,genic_downstream_transcript_variant
rs1164118777	intron_variant,genic_upstream_transcript_variant
rs1164207553	intron_variant,genic_upstream_transcript_variant
rs1164220422	intron_variant,genic_upstream_transcript_variant
rs1164236798	intron_variant
rs1164275548	intron_variant,genic_downstream_transcript_variant
rs1164297438	intron_variant
rs1164307753	intron_variant,genic_upstream_transcript_variant
rs1164314982	intron_variant,genic_upstream_transcript_variant
rs1164396339	intron_variant,genic_upstream_transcript_variant
rs1164465610	intron_variant
rs1164474296	intron_variant,genic_upstream_transcript_variant
rs1164505924	intron_variant,genic_upstream_transcript_variant
rs1164530077	intron_variant,genic_downstream_transcript_variant
rs1164546569	missense_variant,non_coding_transcript_variant,coding_sequence_variant
rs1164564549	intron_variant
rs1164587328	intron_variant
rs1164616242	intron_variant,genic_upstream_transcript_variant
rs1164657018	intron_variant,genic_upstream_transcript_variant
rs1164657688	intron_variant,genic_upstream_transcript_variant
rs1164662571	intron_variant,genic_downstream_transcript_variant
rs1164705504	intron_variant
rs1164729147	intron_variant
rs1164767094	intron_variant,genic_upstream_transcript_variant
rs1164771938	intron_variant,genic_upstream_transcript_variant
rs1164805994	intron_variant
rs1164819895	intron_variant,genic_downstream_transcript_variant
rs1164863045	intron_variant
rs1164890885	non_coding_transcript_variant,genic_downstream_transcript_variant,3_prime_UTR_variant
rs1164945772	intron_variant
rs1164957767	intron_variant,genic_upstream_transcript_variant
rs1164982538	intron_variant,genic_upstream_transcript_variant
rs1164988105	intron_variant,genic_downstream_transcript_variant
rs1164990430	genic_upstream_transcript_variant,intron_variant,2KB_upstream_variant,upstream_transcript_variant
rs1164997515	intron_variant,genic_upstream_transcript_variant
rs1165005682	intron_variant,genic_upstream_transcript_variant

rs1165063151	intron_variant
rs1165064390	intron_variant
rs1165113385	intron_variant,genic_upstream_transcript_variant
rs1165116650	intron_variant
rs1165121714	intron_variant
rs1165125144	intron_variant
rs1165132351	intron_variant,genic_upstream_transcript_variant
rs1165146285	intron_variant,genic_upstream_transcript_variant
rs1165148403	intron_variant,genic_upstream_transcript_variant,upstream_transcript_variant
rs1165157994	intron_variant
rs1165292737	intron_variant
rs1165302861	intron_variant,genic_upstream_transcript_variant,upstream_transcript_variant
rs1165313375	genic_upstream_transcript_variant,intron_variant,2KB_upstream_variant,upstream_transcript_variant
rs1165402999	intron_variant
rs1165423544	intron_variant,genic_upstream_transcript_variant,upstream_transcript_variant
rs1165504004	intron_variant,genic_upstream_transcript_variant,upstream_transcript_variant
rs1165539754	intron_variant
rs1165565227	intron_variant
rs1165567562	intron_variant,genic_upstream_transcript_variant
rs1165578867	intron_variant,genic_upstream_transcript_variant
rs1165606252	intron_variant,genic_downstream_transcript_variant
rs1165631200	intron_variant
rs1165675906	intron_variant,downstream_transcript_variant,genic_downstream_transcript_variant
rs1165686183	intron_variant
rs1165713069	intron_variant,genic_downstream_transcript_variant
rs1165754188	intron_variant,genic_downstream_transcript_variant
rs1165765854	intron_variant,genic_upstream_transcript_variant,upstream_transcript_variant
rs1165776302	intron_variant,genic_upstream_transcript_variant
rs1165786130	intron_variant
rs1165804438	intron_variant
rs1165815861	intron_variant,genic_upstream_transcript_variant
rs1165826797	intron_variant
rs1165938208	intron_variant
rs1165948294	intron_variant
rs1165953762	intron_variant
rs1165971256	intron_variant,genic_downstream_transcript_variant
rs1165981528	intron_variant,genic_downstream_transcript_variant
rs1165985876	intron_variant,genic_downstream_transcript_variant
rs1165988604	intron_variant
rs1166017128	intron_variant
rs1166019588	intron_variant,genic_downstream_transcript_variant
rs1166033796	intron_variant
rs1166097706	intron_variant,genic_upstream_transcript_variant
rs1166111910	intron_variant,genic_upstream_transcript_variant
rs1166144699	intron_variant
rs1166176802	intron_variant,genic_upstream_transcript_variant
rs1166204866	intron_variant
rs1166259596	intron_variant,genic_upstream_transcript_variant
rs1166285645	intron_variant,genic_upstream_transcript_variant
rs1166289922	intron_variant,genic_upstream_transcript_variant
rs1166301044	intron_variant,genic_downstream_transcript_variant
rs1166311950	intron_variant,genic_upstream_transcript_variant
rs1166344170	synonymous_variant,non_coding_transcript_variant,coding_sequence_variant,missense_variant,genic_downstream_transcript_variant
rs1166368854	intron_variant,genic_downstream_transcript_variant
rs1166376725	intron_variant
rs1166447545	intron_variant,genic_downstream_transcript_variant
rs1166459036	intron_variant,genic_upstream_transcript_variant
rs1166503011	intron_variant,genic_upstream_transcript_variant
rs1166531812	intron_variant
rs1166537321	intron_variant
rs1166573602	intron_variant
rs1166581039	intron_variant
rs1166588783	intron_variant
rs1166603580	intron_variant
rs1166612551	intron_variant,genic_downstream_transcript_variant
rs1166652916	missense_variant,non_coding_transcript_variant,genic_downstream_transcript_variant,coding_sequence_variant
rs1166667915	intron_variant,genic_upstream_transcript_variant
rs1166681331	intron_variant
rs1166681910	intron_variant,genic_upstream_transcript_variant
rs1166701935	intron_variant,genic_downstream_transcript_variant
rs1166708597	intron_variant,genic_upstream_transcript_variant
rs1166748457	intron_variant,genic_upstream_transcript_variant,upstream_transcript_variant
rs1166750404	intron_variant
rs1166791252	intron_variant

rs1166794481	intron_variant
rs1166801810	intron_variant,genic_upstream_transcript_variant
rs1166828541	intron_variant,genic_downstream_transcript_variant
rs1166831331	intron_variant,genic_downstream_transcript_variant
rs1166889661	intron_variant
rs1166898798	intron_variant,genic_upstream_transcript_variant
rs1166911788	intron_variant
rs1166967081	intron_variant,genic_upstream_transcript_variant
rs1166984006	intron_variant
rs1167001399	intron_variant
rs1167049299	intron_variant,genic_downstream_transcript_variant
rs1167081672	intron_variant,genic_downstream_transcript_variant
rs1167093562	genic_upstream_transcript_variant,non_coding_transcript_variant,intron_variant,missense_variant,coding_sequence_variant
rs1167116930	synonymous_variant,non_coding_transcript_variant,coding_sequence_variant
rs1167142583	intron_variant,genic_upstream_transcript_variant
rs1167148208	intron_variant,genic_upstream_transcript_variant
rs1167174037	intron_variant
rs1167198668	intron_variant
rs1167231127	intron_variant
rs1167273965	intron_variant,genic_upstream_transcript_variant
rs1167290669	500B_downstream_variant,downstream_transcript_variant
rs1167296853	intron_variant,genic_upstream_transcript_variant,upstream_transcript_variant
rs1167447816	intron_variant,genic_upstream_transcript_variant,2KB_upstream_variant,upstream_transcript_variant
rs1167449430	intron_variant,genic_upstream_transcript_variant
rs1167462636	intron_variant,genic_downstream_transcript_variant
rs1167477303	intron_variant
rs1167486031	intron_variant,genic_downstream_transcript_variant
rs1167502615	genic_upstream_transcript_variant,intron_variant,2KB_upstream_variant,upstream_transcript_variant
rs1167511848	intron_variant,genic_upstream_transcript_variant
rs1167554347	intron_variant,genic_upstream_transcript_variant
rs1167572256	non_coding_transcript_variant,genic_downstream_transcript_variant,3_prime_UTR_variant
rs1167603693	intron_variant,genic_upstream_transcript_variant
rs1167632895	intron_variant,genic_upstream_transcript_variant,upstream_transcript_variant
rs1167763643	intron_variant,genic_downstream_transcript_variant
rs1167775250	intron_variant,genic_upstream_transcript_variant
rs1167804286	intron_variant,genic_upstream_transcript_variant
rs1167820986	intron_variant,genic_upstream_transcript_variant
rs1167848353	intron_variant,genic_upstream_transcript_variant
rs1167851500	genic_upstream_transcript_variant,synonymous_variant,non_coding_transcript_variant,intron_variant,missense_variant,coding_sequence_variant
rs1167857818	intron_variant,genic_downstream_transcript_variant
rs1167875033	intron_variant,genic_downstream_transcript_variant
rs1167912597	intron_variant,genic_upstream_transcript_variant
rs1167927177	intron_variant
rs1167930286	intron_variant,genic_upstream_transcript_variant
rs1167978353	intron_variant,genic_downstream_transcript_variant
rs1168002403	intron_variant,genic_upstream_transcript_variant
rs1168066116	intron_variant
rs1168091854	genic_upstream_transcript_variant,non_coding_transcript_variant,intron_variant,missense_variant,coding_sequence_variant
rs1168117990	genic_upstream_transcript_variant,intron_variant,2KB_upstream_variant,upstream_transcript_variant
rs1168123302	intron_variant
rs1168131760	intron_variant,genic_upstream_transcript_variant
rs1168159294	intron_variant
rs1168161261	intron_variant,genic_downstream_transcript_variant
rs1168220004	intron_variant
rs1168238440	intron_variant,genic_upstream_transcript_variant
rs1168293105	intron_variant,genic_upstream_transcript_variant
rs1168322724	intron_variant,genic_upstream_transcript_variant
rs1168348760	intron_variant,genic_upstream_transcript_variant
rs1168374891	intron_variant,genic_upstream_transcript_variant
rs1168408802	intron_variant
rs1168429884	intron_variant,genic_downstream_transcript_variant
rs1168455925	intron_variant
rs1168520580	intron_variant
rs1168527840	intron_variant
rs1168551672	intron_variant,genic_upstream_transcript_variant,2KB_upstream_variant,upstream_transcript_variant
rs1168587164	intron_variant,genic_upstream_transcript_variant,upstream_transcript_variant
rs1168592008	intron_variant,genic_upstream_transcript_variant
rs1168705329	intron_variant,genic_upstream_transcript_variant
rs1168713178	intron_variant,genic_upstream_transcript_variant
rs1168713978	intron_variant,genic_upstream_transcript_variant
rs1168734224	intron_variant,genic_upstream_transcript_variant
rs1168739007	intron_variant,genic_upstream_transcript_variant
rs1168750141	intron_variant,genic_downstream_transcript_variant
rs1168862161	missense_variant,non_coding_transcript_variant,genic_downstream_transcript_variant,coding_sequence_variant

rs1168902391	genic_upstream_transcript_variant,intron_variant,2KB_upstream_variant,upstream_transcript_variant
rs1168919833	intron_variant,genic_upstream_transcript_variant
rs1168929642	intron_variant,genic_upstream_transcript_variant
rs1168935845	intron_variant
rs1168937110	intron_variant,genic_upstream_transcript_variant,2KB_upstream_variant,upstream_transcript_variant
rs1168978989	intron_variant,genic_upstream_transcript_variant
rs1168986019	intron_variant
rs1169031386	intron_variant,genic_upstream_transcript_variant
rs1169036700	intron_variant,genic_downstream_transcript_variant
rs1169080179	genic_upstream_transcript_variant,intron_variant,5_prime_UTR_variant,non_coding_transcript_variant
rs1169112723	intron_variant
rs1169143601	intron_variant
rs1169157960	intron_variant,genic_upstream_transcript_variant
rs1169170943	intron_variant,genic_upstream_transcript_variant,2KB_upstream_variant,upstream_transcript_variant
rs1169179255	intron_variant
rs1169179300	non_coding_transcript_variant,genic_downstream_transcript_variant,3_prime_UTR_variant
rs1169229138	intron_variant,genic_upstream_transcript_variant
rs1169260312	intron_variant,genic_upstream_transcript_variant
rs1169306917	intron_variant
rs1169320835	intron_variant
rs1169334989	intron_variant,genic_upstream_transcript_variant
rs1169384064	intron_variant
rs1169399730	intron_variant,genic_upstream_transcript_variant
rs1169406106	intron_variant
rs1169469478	intron_variant
rs1169469820	intron_variant
rs1169475877	intron_variant,genic_upstream_transcript_variant
rs1169484546	intron_variant,genic_upstream_transcript_variant
rs1169522914	intron_variant,genic_downstream_transcript_variant
rs1169601704	intron_variant,genic_upstream_transcript_variant
rs1169602037	intron_variant,genic_downstream_transcript_variant
rs1169629750	missense_variant,non_coding_transcript_variant,genic_downstream_transcript_variant,coding_sequence_variant
rs1169639570	intron_variant
rs1169699790	500B_downstream_variant,downstream_transcript_variant
rs1169704975	intron_variant
rs1169711073	intron_variant,genic_downstream_transcript_variant
rs1169715802	intron_variant,genic_upstream_transcript_variant
rs1169763656	non_coding_transcript_variant,genic_downstream_transcript_variant,3_prime_UTR_variant
rs1169855481	intron_variant,genic_upstream_transcript_variant
rs1169862200	intron_variant,genic_downstream_transcript_variant
rs1169874294	intron_variant
rs1169896187	intron_variant,genic_downstream_transcript_variant
rs1170064943	intron_variant
rs1170081826	intron_variant
rs1170096703	intron_variant
rs1170099567	intron_variant,genic_upstream_transcript_variant
rs1170135408	intron_variant,genic_downstream_transcript_variant
rs1170146791	intron_variant
rs1170163526	intron_variant
rs1170192752	intron_variant,genic_upstream_transcript_variant
rs1170222568	intron_variant,genic_upstream_transcript_variant
rs1170229319	intron_variant,genic_upstream_transcript_variant
rs1170238205	intron_variant,genic_downstream_transcript_variant
rs1170247308	genic_upstream_transcript_variant,intron_variant,2KB_upstream_variant,upstream_transcript_variant
rs1170252410	intron_variant,genic_downstream_transcript_variant
rs1170280617	genic_upstream_transcript_variant,intron_variant,2KB_upstream_variant,upstream_transcript_variant
rs1170316245	intron_variant
rs1170327786	intron_variant,genic_upstream_transcript_variant
rs1170350487	intron_variant
rs1170390091	intron_variant,genic_upstream_transcript_variant
rs1170405392	intron_variant
rs1170429295	intron_variant
rs1170442625	intron_variant,genic_upstream_transcript_variant
rs1170496578	intron_variant
rs1170508478	intron_variant
rs1170511553	non_coding_transcript_variant,genic_downstream_transcript_variant,3_prime_UTR_variant
rs1170534401	intron_variant,genic_downstream_transcript_variant
rs1170544966	intron_variant,genic_upstream_transcript_variant
rs1170604838	intron_variant,genic_downstream_transcript_variant
rs1170609772	intron_variant
rs1170659481	intron_variant,genic_upstream_transcript_variant,2KB_upstream_variant,upstream_transcript_variant
rs1170672686	intron_variant,genic_downstream_transcript_variant
rs1170717502	genic_upstream_transcript_variant,intron_variant,2KB_upstream_variant,upstream_transcript_variant
rs1170726921	intron_variant,genic_upstream_transcript_variant

rs1170736301	intron_variant,genic_upstream_transcript_variant,upstream_transcript_variant
rs1170739382	intron_variant,genic_upstream_transcript_variant
rs1170770898	intron_variant,genic_upstream_transcript_variant,upstream_transcript_variant
rs1170777166	intron_variant,genic_upstream_transcript_variant
rs1170936390	intron_variant,genic_upstream_transcript_variant
rs1170977619	genic_upstream_transcript_variant,intron_variant,2KB_upstream_variant,upstream_transcript_variant
rs1171000027	intron_variant,genic_upstream_transcript_variant
rs1171004836	intron_variant,genic_downstream_transcript_variant
rs1171019557	intron_variant,genic_upstream_transcript_variant
rs1171052016	intron_variant,genic_downstream_transcript_variant
rs1171097970	intron_variant,genic_upstream_transcript_variant
rs1171139476	intron_variant,genic_upstream_transcript_variant
rs1171214311	intron_variant,genic_downstream_transcript_variant
rs1171296287	genic_upstream_transcript_variant,intron_variant
rs1171302198	intron_variant
rs1171330017	intron_variant
rs1171406746	intron_variant,genic_downstream_transcript_variant
rs1171455840	genic_upstream_transcript_variant,intron_variant
rs1171481048	genic_upstream_transcript_variant,upstream_transcript_variant,intron_variant
rs1171489063	intron_variant
rs1171529030	genic_upstream_transcript_variant,intron_variant
rs1171533030	intron_variant,genic_downstream_transcript_variant
rs1171576050	genic_upstream_transcript_variant,intron_variant
rs1171579825	intron_variant
rs1171599552	intron_variant
rs1171613576	genic_upstream_transcript_variant,intron_variant
rs1171616127	intron_variant
rs1171638535	intron_variant,genic_downstream_transcript_variant
rs1171697944	genic_upstream_transcript_variant,intron_variant
rs1171747170	intron_variant,genic_downstream_transcript_variant
rs1171756972	intron_variant
rs1171761931	intron_variant,genic_downstream_transcript_variant
rs1171778468	genic_upstream_transcript_variant,intron_variant
rs1171785561	intron_variant
rs1171808750	intron_variant,genic_downstream_transcript_variant
rs1171825076	intron_variant
rs1171832510	genic_upstream_transcript_variant,intron_variant
rs1171833631	intron_variant
rs1171850900	genic_upstream_transcript_variant,intron_variant
rs1171866537	intron_variant,genic_downstream_transcript_variant
rs1171870831	intron_variant
rs1171900072	genic_upstream_transcript_variant,upstream_transcript_variant,2KB_upstream_variant,intron_variant
rs1171942845	intron_variant
rs1172019171	intron_variant
rs1172036825	intron_variant
rs1172093834	intron_variant
rs1172120822	intron_variant
rs1172127122	2KB_upstream_variant,genic_upstream_transcript_variant,upstream_transcript_variant,intron_variant
rs1172136124	downstream_transcript_variant,500B_downstream_variant
rs1172138511	non_coding_transcript_variant,3_prime_UTR_variant,genic_downstream_transcript_variant
rs1172244823	intron_variant,genic_downstream_transcript_variant
rs1172256472	intron_variant
rs1172272386	genic_upstream_transcript_variant,intron_variant
rs1172297155	intron_variant
rs1172302902	intron_variant
rs1172309642	intron_variant,genic_downstream_transcript_variant
rs1172345273	intron_variant,genic_downstream_transcript_variant
rs1172357278	intron_variant
rs1172369826	genic_upstream_transcript_variant,intron_variant
rs1172396460	genic_upstream_transcript_variant,intron_variant
rs1172402433	intron_variant
rs1172437377	intron_variant
rs1172454283	intron_variant
rs1172459787	intron_variant
rs1172482469	intron_variant,genic_downstream_transcript_variant
rs1172490289	intron_variant
rs1172501723	intron_variant
rs1172539769	5_prime_UTR_variant,upstream_transcript_variant,genic_upstream_transcript_variant,intron_variant
rs1172544218	genic_upstream_transcript_variant,intron_variant
rs1172566930	intron_variant
rs1172696888	intron_variant
rs1172714209	intron_variant
rs1172723667	genic_upstream_transcript_variant,intron_variant
rs1172748233	intron_variant,genic_downstream_transcript_variant

rs1172791645	genic_upstream_transcript_variant,intron_variant
rs1172793685	genic_upstream_transcript_variant,intron_variant
rs1172825624	genic_upstream_transcript_variant,intron_variant
rs1172827335	genic_upstream_transcript_variant,upstream_transcript_variant,intron_variant
rs1172856971	intron_variant,genic_downstream_transcript_variant
rs1172857366	genic_upstream_transcript_variant,intron_variant
rs1172889878	genic_upstream_transcript_variant,intron_variant
rs1172902830	genic_upstream_transcript_variant,intron_variant
rs1172919110	genic_upstream_transcript_variant,intron_variant
rs1172928754	genic_upstream_transcript_variant,intron_variant
rs1172939527	genic_upstream_transcript_variant,intron_variant
rs1172958763	intron_variant
rs1172984423	intron_variant
rs1172986259	2KB_upstream_variant,genic_upstream_transcript_variant,upstream_transcript_variant,intron_variant
rs1172996895	genic_upstream_transcript_variant,intron_variant
rs1173022438	intron_variant,genic_downstream_transcript_variant
rs1173051857	genic_upstream_transcript_variant,intron_variant
rs1173065088	genic_upstream_transcript_variant,intron_variant
rs1173096135	intron_variant
rs1173114634	genic_upstream_transcript_variant,intron_variant
rs1173144279	genic_upstream_transcript_variant,intron_variant
rs1173147623	intron_variant
rs1173150821	genic_upstream_transcript_variant,intron_variant
rs1173162969	genic_upstream_transcript_variant,intron_variant
rs1173220065	genic_upstream_transcript_variant,intron_variant
rs1173234054	intron_variant
rs1173285707	intron_variant
rs1173290757	genic_upstream_transcript_variant,intron_variant
rs1173320622	genic_upstream_transcript_variant,intron_variant
rs1173328514	intron_variant
rs1173357280	intron_variant
rs1173372099	2KB_upstream_variant,genic_upstream_transcript_variant,upstream_transcript_variant,intron_variant
rs1173372713	intron_variant
rs1173396753	intron_variant
rs1173424922	non_coding_transcript_variant,3_prime_UTR_variant,genic_downstream_transcript_variant
rs1173453804	intron_variant
rs1173460463	intron_variant,genic_downstream_transcript_variant
rs1173464083	genic_upstream_transcript_variant,intron_variant
rs1173491884	intron_variant
rs1173504924	intron_variant,genic_downstream_transcript_variant
rs1173505549	intron_variant
rs1173529820	5_prime_UTR_variant,intron_variant
rs1173571927	intron_variant
rs1173589736	intron_variant
rs1173605554	genic_upstream_transcript_variant,intron_variant
rs1173648832	genic_upstream_transcript_variant,intron_variant
rs1173658238	non_coding_transcript_variant,3_prime_UTR_variant,genic_downstream_transcript_variant
rs1173670558	intron_variant,genic_downstream_transcript_variant
rs1173687099	intron_variant,genic_downstream_transcript_variant
rs1173717037	genic_upstream_transcript_variant,intron_variant
rs1173766446	genic_upstream_transcript_variant,intron_variant
rs1173767792	intron_variant
rs1173795668	intron_variant
rs1173826747	intron_variant,genic_downstream_transcript_variant
rs1173837016	intron_variant
rs1173852292	intron_variant
rs1173853702	intron_variant
rs1173898040	genic_upstream_transcript_variant,intron_variant
rs1174007468	intron_variant
rs1174024378	intron_variant
rs1174026356	genic_upstream_transcript_variant,intron_variant
rs1174049713	intron_variant,genic_downstream_transcript_variant
rs1174053984	genic_upstream_transcript_variant,intron_variant
rs1174057879	intron_variant
rs1174071856	genic_upstream_transcript_variant,intron_variant
rs1174143074	intron_variant,genic_downstream_transcript_variant
rs1174161333	genic_upstream_transcript_variant,intron_variant
rs1174164548	intron_variant
rs1174171134	intron_variant,genic_downstream_transcript_variant
rs1174193787	synonymous_variant,coding_sequence_variant,non_coding_transcript_variant,genic_downstream_transcript_variant
rs1174196900	intron_variant,genic_downstream_transcript_variant
rs1174214230	genic_upstream_transcript_variant,intron_variant
rs1174225221	intron_variant,genic_downstream_transcript_variant
rs1174234952	intron_variant

rs1174260412	genic_upstream_transcript_variant,upstream_transcript_variant,intron_variant
rs1174266989	intron_variant,genic_downstream_transcript_variant
rs1174284061	intron_variant,genic_downstream_transcript_variant
rs1174294996	intron_variant,genic_downstream_transcript_variant
rs1174413040	intron_variant
rs1174423550	genic_upstream_transcript_variant,intron_variant
rs1174445292	intron_variant
rs1174471966	genic_upstream_transcript_variant,intron_variant
rs1174487675	genic_upstream_transcript_variant,intron_variant
rs1174500379	downstream_transcript_variant,500B_downstream_variant
rs1174529675	genic_upstream_transcript_variant,intron_variant
rs1174543569	genic_upstream_transcript_variant,intron_variant
rs1174580584	intron_variant
rs1174656971	intron_variant
rs1174673826	genic_upstream_transcript_variant,intron_variant
rs1174704566	intron_variant,genic_downstream_transcript_variant
rs1174759526	intron_variant
rs1174786318	intron_variant,genic_downstream_transcript_variant
rs1174795250	genic_upstream_transcript_variant,intron_variant
rs1174803860	intron_variant
rs1174812703	genic_upstream_transcript_variant,intron_variant
rs1174825269	intron_variant
rs1174841180	genic_upstream_transcript_variant,intron_variant
rs1174843209	intron_variant,genic_downstream_transcript_variant
rs1174848658	genic_upstream_transcript_variant,intron_variant
rs1174868885	genic_upstream_transcript_variant,intron_variant
rs1174873642	genic_upstream_transcript_variant,intron_variant
rs1174912744	intron_variant
rs1174982308	genic_upstream_transcript_variant,intron_variant
rs1174996552	intron_variant,genic_downstream_transcript_variant
rs1174998011	genic_upstream_transcript_variant,intron_variant
rs1175062495	genic_upstream_transcript_variant,intron_variant
rs1175076505	intron_variant,genic_downstream_transcript_variant
rs1175149486	intron_variant
rs1175196911	genic_upstream_transcript_variant,intron_variant
rs1175251199	intron_variant
rs1175338336	genic_upstream_transcript_variant,intron_variant
rs1175339091	genic_upstream_transcript_variant,intron_variant
rs1175359817	intron_variant
rs1175384041	intron_variant
rs1175407954	genic_upstream_transcript_variant,intron_variant
rs1175430246	intron_variant
rs1175458192	genic_upstream_transcript_variant,intron_variant
rs1175466489	intron_variant
rs1175490299	genic_upstream_transcript_variant,intron_variant
rs1175522218	genic_upstream_transcript_variant,intron_variant
rs1175526362	intron_variant
rs1175540600	genic_upstream_transcript_variant,intron_variant
rs1175546855	intron_variant,genic_downstream_transcript_variant
rs1175558385	genic_upstream_transcript_variant,intron_variant
rs1175558648	genic_upstream_transcript_variant,intron_variant
rs1175606749	genic_upstream_transcript_variant,intron_variant
rs1175627333	intron_variant
rs1175638209	intron_variant,genic_downstream_transcript_variant
rs1175669250	intron_variant
rs1175669415	intron_variant
rs1175728440	intron_variant
rs1175737629	intron_variant
rs1175743942	genic_upstream_transcript_variant,intron_variant
rs1175776183	genic_upstream_transcript_variant,intron_variant
rs1175792179	intron_variant
rs1175801596	genic_upstream_transcript_variant,intron_variant
rs1175843978	genic_upstream_transcript_variant,intron_variant
rs1175857919	intron_variant
rs1175872018	intron_variant
rs1175879164	genic_upstream_transcript_variant,intron_variant
rs1175900253	intron_variant
rs1175987399	intron_variant
rs1175990734	genic_upstream_transcript_variant,upstream_transcript_variant,intron_variant
rs1176030083	genic_upstream_transcript_variant,intron_variant
rs1176051274	genic_upstream_transcript_variant,intron_variant
rs1176088481	genic_upstream_transcript_variant,intron_variant
rs1176101100	intron_variant,genic_downstream_transcript_variant
rs1176112251	missense_variant,coding_sequence_variant,non_coding_transcript_variant

rs1176165765	intron_variant,genic_downstream_transcript_variant
rs1176169561	intron_variant,genic_downstream_transcript_variant
rs1176194393	genic_upstream_transcript_variant,intron_variant
rs1176194687	genic_upstream_transcript_variant,intron_variant
rs1176206312	intron_variant,genic_downstream_transcript_variant
rs1176251872	genic_upstream_transcript_variant,intron_variant
rs1176297406	genic_upstream_transcript_variant,upstream_transcript_variant,intron_variant
rs1176324312	intron_variant
rs1176339618	intron_variant
rs1176346304	intron_variant
rs1176352060	intron_variant
rs1176356843	non_coding_transcript_variant,3_prime_UTR_variant,genic_downstream_transcript_variant
rs1176363163	genic_upstream_transcript_variant,intron_variant
rs1176376514	genic_upstream_transcript_variant,intron_variant
rs1176398078	intron_variant
rs1176403422	genic_upstream_transcript_variant,upstream_transcript_variant,intron_variant
rs1176425508	intron_variant
rs1176443675	intron_variant
rs1176471216	intron_variant
rs1176483649	intron_variant,genic_downstream_transcript_variant
rs1176486564	intron_variant,genic_downstream_transcript_variant
rs1176490159	intron_variant
rs1176548903	intron_variant,genic_downstream_transcript_variant
rs1176556418	intron_variant
rs1176573271	intron_variant,genic_downstream_transcript_variant
rs1176615440	genic_upstream_transcript_variant,intron_variant
rs1176619648	intron_variant
rs1176627671	intron_variant
rs1176639725	genic_upstream_transcript_variant,intron_variant
rs1176656422	intron_variant,genic_downstream_transcript_variant
rs1176659582	intron_variant
rs1176666682	intron_variant,genic_downstream_transcript_variant
rs1176675389	genic_upstream_transcript_variant,intron_variant
rs1176684983	intron_variant
rs1176703445	intron_variant,genic_downstream_transcript_variant
rs1176732224	genic_upstream_transcript_variant,intron_variant
rs1176750613	intron_variant,genic_downstream_transcript_variant
rs1176753773	genic_upstream_transcript_variant,intron_variant
rs1176757777	intron_variant,genic_downstream_transcript_variant
rs1176780606	intron_variant
rs1176786648	intron_variant,genic_downstream_transcript_variant
rs1176794214	intron_variant,genic_downstream_transcript_variant
rs1176801475	genic_upstream_transcript_variant,intron_variant
rs1176809820	genic_upstream_transcript_variant,upstream_transcript_variant,intron_variant
rs1176817945	intron_variant
rs1176880139	intron_variant
rs1176890278	intron_variant
rs1176895443	genic_upstream_transcript_variant,intron_variant
rs1177016159	intron_variant
rs1177018607	intron_variant,genic_downstream_transcript_variant
rs1177033389	intron_variant
rs1177078744	intron_variant
rs1177112883	genic_upstream_transcript_variant,intron_variant
rs1177154202	intron_variant
rs1177189009	intron_variant
rs1177232712	intron_variant
rs1177266873	intron_variant,genic_downstream_transcript_variant
rs1177317352	intron_variant
rs1177334260	intron_variant
rs1177340131	genic_upstream_transcript_variant,intron_variant
rs1177388774	genic_upstream_transcript_variant,intron_variant
rs1177464546	genic_upstream_transcript_variant,upstream_transcript_variant,intron_variant
rs1177509178	genic_upstream_transcript_variant,intron_variant
rs1177577753	genic_upstream_transcript_variant,upstream_transcript_variant,2KB_upstream_variant,intron_variant
rs1177591481	genic_upstream_transcript_variant,intron_variant
rs1177617568	intron_variant,genic_downstream_transcript_variant
rs1177677516	intron_variant
rs1177735297	genic_upstream_transcript_variant,intron_variant
rs1177845605	intron_variant
rs1177895895	genic_upstream_transcript_variant,intron_variant
rs1177909636	genic_upstream_transcript_variant,intron_variant
rs1177931657	intron_variant,genic_downstream_transcript_variant
rs1177948363	intron_variant,genic_downstream_transcript_variant
rs1177964111	intron_variant

rs1177986500	genic_upstream_transcript_variant,intron_variant
rs1177991410	intron_variant,genic_downstream_transcript_variant
rs1178019868	intron_variant,genic_downstream_transcript_variant
rs1178038516	intron_variant,genic_downstream_transcript_variant
rs1178057688	2KB_upstream_variant,genic_upstream_transcript_variant,upstream_transcript_variant,intron_variant
rs1178071993	intron_variant
rs1178118813	genic_upstream_transcript_variant,intron_variant
rs1178150031	genic_upstream_transcript_variant,intron_variant,coding_sequence_variant,non_coding_transcript_variant,synonymous_variant
rs1178159448	genic_upstream_transcript_variant,intron_variant
rs1178169917	genic_upstream_transcript_variant,intron_variant
rs1178172919	intron_variant
rs1178238693	2KB_upstream_variant,genic_upstream_transcript_variant,upstream_transcript_variant,intron_variant
rs1178244474	genic_upstream_transcript_variant,intron_variant
rs1178266474	genic_upstream_transcript_variant,upstream_transcript_variant,intron_variant
rs1178278280	intron_variant
rs1178321797	intron_variant
rs1178328105	intron_variant
rs1178355837	intron_variant,genic_downstream_transcript_variant
rs1178384187	intron_variant
rs1178399278	genic_upstream_transcript_variant,intron_variant
rs1178430025	intron_variant
rs1178507322	intron_variant,genic_downstream_transcript_variant
rs1178518112	intron_variant
rs1178659147	2KB_upstream_variant,genic_upstream_transcript_variant,upstream_transcript_variant,intron_variant
rs1178690804	intron_variant
rs1178717003	intron_variant,genic_downstream_transcript_variant
rs1178736227	intron_variant
rs1178747223	intron_variant,genic_downstream_transcript_variant
rs1178762351	genic_upstream_transcript_variant,intron_variant
rs1178763062	genic_upstream_transcript_variant,intron_variant
rs1178791647	intron_variant,genic_downstream_transcript_variant
rs1178798795	intron_variant
rs1178832992	intron_variant
rs1178846049	genic_upstream_transcript_variant,intron_variant
rs1178852623	intron_variant
rs1178871078	genic_upstream_transcript_variant,intron_variant
rs1178899349	intron_variant,genic_downstream_transcript_variant
rs1178903165	genic_upstream_transcript_variant,intron_variant
rs1178932986	genic_upstream_transcript_variant,intron_variant
rs1178986556	genic_upstream_transcript_variant,intron_variant
rs1179018154	genic_upstream_transcript_variant,intron_variant
rs1179038016	intron_variant
rs1179049270	intron_variant
rs1179097828	intron_variant
rs1179110414	intron_variant
rs1179117084	genic_upstream_transcript_variant,intron_variant
rs1179136922	intron_variant
rs1179161218	intron_variant,genic_downstream_transcript_variant
rs1179166455	intron_variant
rs1179192792	intron_variant,genic_downstream_transcript_variant
rs1179201081	intron_variant
rs1179228324	genic_upstream_transcript_variant,intron_variant
rs1179286304	genic_upstream_transcript_variant,intron_variant
rs1179289288	intron_variant
rs1179324298	genic_upstream_transcript_variant,intron_variant
rs1179408376	genic_upstream_transcript_variant,intron_variant
rs1179411664	genic_upstream_transcript_variant,intron_variant
rs1179422816	genic_upstream_transcript_variant,intron_variant
rs1179466519	intron_variant
rs1179513136	genic_upstream_transcript_variant,intron_variant
rs1179528378	intron_variant
rs1179552467	intron_variant
rs1179555616	intron_variant
rs1179608733	intron_variant
rs1179612756	intron_variant
rs1179630871	genic_upstream_transcript_variant,intron_variant,non_coding_transcript_variant
rs1179632454	intron_variant
rs1179633030	genic_upstream_transcript_variant,intron_variant
rs1179649860	intron_variant,genic_downstream_transcript_variant
rs1179678316	genic_upstream_transcript_variant,intron_variant
rs1179693447	intron_variant,genic_downstream_transcript_variant
rs1179696513	intron_variant
rs1179715527	genic_upstream_transcript_variant,intron_variant
rs1179719039	genic_upstream_transcript_variant,upstream_transcript_variant,intron_variant

rs1179719284	intron_variant
rs1179721785	genic_upstream_transcript_variant,intron_variant
rs1179727686	intron_variant
rs1179760074	intron_variant
rs1179774327	intron_variant
rs1179780006	genic_upstream_transcript_variant,intron_variant
rs1179812235	intron_variant
rs1179841598	missense_variant,coding_sequence_variant,non_coding_transcript_variant
rs1179900472	genic_upstream_transcript_variant,intron_variant
rs1179986381	intron_variant
rs1179991463	genic_upstream_transcript_variant,intron_variant
rs1179996123	intron_variant
rs1180042521	intron_variant,genic_downstream_transcript_variant
rs1180052502	genic_upstream_transcript_variant,intron_variant
rs1180112565	genic_upstream_transcript_variant,intron_variant
rs1180126235	genic_upstream_transcript_variant,intron_variant
rs1180238758	genic_upstream_transcript_variant,intron_variant
rs1180319732	intron_variant,genic_downstream_transcript_variant
rs1180346486	intron_variant
rs1180352228	genic_upstream_transcript_variant,intron_variant
rs1180415202	missense_variant,coding_sequence_variant,non_coding_transcript_variant,genic_downstream_transcript_variant
rs1180424743	intron_variant,genic_downstream_transcript_variant
rs1180434116	intron_variant
rs1180443794	intron_variant
rs1180445951	genic_upstream_transcript_variant,intron_variant
rs1180541370	intron_variant
rs1180572828	intron_variant
rs1180648118	downstream_transcript_variant,intron_variant,genic_downstream_transcript_variant
rs1180658080	genic_upstream_transcript_variant,intron_variant
rs1180714899	synonymous_variant,coding_sequence_variant,non_coding_transcript_variant
rs1180735012	intron_variant
rs1180802650	genic_upstream_transcript_variant,intron_variant
rs1180891326	intron_variant
rs1180950151	intron_variant,genic_downstream_transcript_variant
rs1180987132	genic_upstream_transcript_variant,intron_variant
rs1181016795	genic_upstream_transcript_variant,intron_variant
rs1181026243	intron_variant
rs1181043895	genic_upstream_transcript_variant,intron_variant
rs1181080387	intron_variant
rs1181093632	intron_variant,genic_downstream_transcript_variant
rs1181123450	genic_upstream_transcript_variant,intron_variant
rs1181221478	intron_variant
rs1181250959	5_prime_UTR_variant,intron_variant
rs1181329720	intron_variant,genic_downstream_transcript_variant
rs1181335899	genic_upstream_transcript_variant,intron_variant
rs1181347874	genic_upstream_transcript_variant,intron_variant
rs1181371607	genic_upstream_transcript_variant,intron_variant
rs1181386804	genic_upstream_transcript_variant,intron_variant
rs1181399527	genic_upstream_transcript_variant,intron_variant
rs1181428551	intron_variant
rs1181576580	genic_upstream_transcript_variant,intron_variant
rs1181623164	intron_variant
rs1181625003	genic_upstream_transcript_variant,intron_variant
rs1181629035	genic_upstream_transcript_variant,intron_variant
rs1181647445	intron_variant
rs1181677006	genic_upstream_transcript_variant,intron_variant
rs1181699594	genic_upstream_transcript_variant,intron_variant
rs1181711739	genic_upstream_transcript_variant,intron_variant
rs1181733200	genic_upstream_transcript_variant,intron_variant
rs1181735770	intron_variant
rs1181749563	intron_variant,genic_downstream_transcript_variant
rs1181783501	genic_upstream_transcript_variant,intron_variant
rs1181862737	intron_variant
rs1181902482	intron_variant
rs1181912381	genic_upstream_transcript_variant,intron_variant
rs1181917342	intron_variant
rs1181933414	genic_upstream_transcript_variant,intron_variant
rs1181937573	genic_upstream_transcript_variant,intron_variant
rs1181937823	intron_variant
rs1181970929	intron_variant
rs1181971857	genic_upstream_transcript_variant,intron_variant
rs1181992246	2KB_upstream_variant,genic_upstream_transcript_variant,upstream_transcript_variant,intron_variant
rs1182042097	genic_upstream_transcript_variant,intron_variant
rs1182047442	intron_variant

rs1182074623	intron_variant
rs1182095571	intron_variant
rs1182107281	genic_upstream_transcript_variant,intron_variant
rs1182144251	genic_upstream_transcript_variant,intron_variant
rs1182167119	genic_upstream_transcript_variant,intron_variant
rs1182171972	intron_variant
rs1182195735	genic_upstream_transcript_variant,intron_variant,missense_variant,coding_sequence_variant,non_coding_transcript_variant
rs1182199807	intron_variant
rs1182222404	intron_variant
rs1182235649	genic_upstream_transcript_variant,intron_variant
rs1182270418	intron_variant,genic_downstream_transcript_variant
rs1182278082	genic_upstream_transcript_variant,intron_variant
rs1182296115	intron_variant
rs1182370361	genic_upstream_transcript_variant,intron_variant
rs1182374291	genic_upstream_transcript_variant,intron_variant
rs1182377188	intron_variant
rs1182382916	genic_upstream_transcript_variant,intron_variant
rs1182392002	genic_upstream_transcript_variant,intron_variant
rs1182416668	intron_variant
rs1182444042	intron_variant
rs1182456503	genic_upstream_transcript_variant,intron_variant
rs1182477142	genic_upstream_transcript_variant,upstream_transcript_variant,intron_variant
rs1182519145	genic_upstream_transcript_variant,intron_variant
rs1182535311	intron_variant,genic_downstream_transcript_variant
rs1182536342	genic_upstream_transcript_variant,intron_variant
rs1182567563	intron_variant
rs1182585858	genic_upstream_transcript_variant,intron_variant
rs1182616540	intron_variant,genic_downstream_transcript_variant
rs1182673514	2KB_upstream_variant,genic_upstream_transcript_variant,upstream_transcript_variant,intron_variant
rs1182727638	non_coding_transcript_variant,3_prime_UTR_variant,genic_downstream_transcript_variant
rs1182792913	intron_variant
rs1182813340	intron_variant
rs1182815939	intron_variant
rs1182842382	genic_upstream_transcript_variant,intron_variant
rs1182854061	intron_variant
rs1182902863	intron_variant,genic_downstream_transcript_variant
rs1182975356	intron_variant,genic_downstream_transcript_variant
rs1182979020	genic_upstream_transcript_variant,intron_variant
rs1182994959	missense_variant,coding_sequence_variant,non_coding_transcript_variant
rs1183001020	intron_variant
rs1183061126	intron_variant
rs1183061745	genic_upstream_transcript_variant,intron_variant
rs1183073399	genic_upstream_transcript_variant,intron_variant
rs1183077121	intron_variant
rs1183108894	genic_upstream_transcript_variant,intron_variant
rs1183149433	intron_variant,genic_downstream_transcript_variant
rs1183188173	intron_variant
rs1183188357	2KB_upstream_variant,genic_upstream_transcript_variant,upstream_transcript_variant,intron_variant
rs1183196417	intron_variant,genic_downstream_transcript_variant
rs1183266937	intron_variant
rs1183274203	intron_variant,genic_downstream_transcript_variant
rs1183312827	intron_variant,genic_downstream_transcript_variant
rs1183357897	genic_upstream_transcript_variant,intron_variant
rs1183432579	missense_variant,coding_sequence_variant,non_coding_transcript_variant
rs1183439365	genic_upstream_transcript_variant,intron_variant
rs1183452889	genic_upstream_transcript_variant,intron_variant
rs1183469757	genic_upstream_transcript_variant,intron_variant
rs1183521618	intron_variant
rs1183530779	genic_upstream_transcript_variant,intron_variant
rs1183546727	genic_upstream_transcript_variant,intron_variant
rs1183551764	genic_upstream_transcript_variant,intron_variant
rs1183562906	genic_upstream_transcript_variant,intron_variant
rs1183572881	genic_upstream_transcript_variant,intron_variant
rs1183675814	intron_variant
rs1183733283	genic_upstream_transcript_variant,intron_variant
rs1183772785	intron_variant,genic_downstream_transcript_variant
rs1183803972	intron_variant
rs1183914142	non_coding_transcript_variant,genic_downstream_transcript_variant,3_prime_UTR_variant
rs1183914509	intron_variant,genic_downstream_transcript_variant
rs1183918482	genic_upstream_transcript_variant,intron_variant
rs1183993896	genic_upstream_transcript_variant,intron_variant
rs1183996780	genic_upstream_transcript_variant,intron_variant
rs1184016460	genic_upstream_transcript_variant,intron_variant
rs1184039081	intron_variant,synonymous_variant,coding_sequence_variant

rs1184050498	intron_variant
rs1184108013	genic_upstream_transcript_variant,intron_variant
rs1184164857	intron_variant
rs1184180022	intron_variant,genic_downstream_transcript_variant
rs1184213247	genic_upstream_transcript_variant,intron_variant
rs1184234109	intron_variant
rs1184278682	non_coding_transcript_variant,genic_downstream_transcript_variant,3_prime_UTR_variant
rs1184287283	genic_upstream_transcript_variant,intron_variant,non_coding_transcript_variant,5_prime_UTR_variant
rs1184411865	genic_upstream_transcript_variant,intron_variant
rs1184439616	genic_upstream_transcript_variant,intron_variant
rs1184446203	genic_upstream_transcript_variant,intron_variant
rs1184447026	genic_upstream_transcript_variant,intron_variant
rs1184480106	intron_variant
rs1184518741	genic_upstream_transcript_variant,intron_variant
rs1184519275	genic_upstream_transcript_variant,intron_variant,upstream_transcript_variant,2KB_upstream_variant
rs1184543757	genic_upstream_transcript_variant,intron_variant
rs1184751557	genic_upstream_transcript_variant,intron_variant,upstream_transcript_variant,2KB_upstream_variant
rs1184777056	genic_upstream_transcript_variant,intron_variant
rs1184810400	intron_variant,genic_downstream_transcript_variant
rs1184821629	genic_upstream_transcript_variant,intron_variant
rs1184861283	genic_upstream_transcript_variant,intron_variant
rs1184867871	genic_upstream_transcript_variant,intron_variant
rs1184876759	genic_upstream_transcript_variant,intron_variant
rs1184886521	intron_variant,genic_downstream_transcript_variant
rs1184897454	genic_upstream_transcript_variant,intron_variant
rs1184908762	genic_upstream_transcript_variant,intron_variant
rs1184913993	intron_variant
rs1184920952	genic_upstream_transcript_variant,intron_variant
rs1184968324	intron_variant,genic_downstream_transcript_variant
rs1184978892	genic_upstream_transcript_variant,intron_variant
rs1184992839	genic_upstream_transcript_variant,intron_variant,upstream_transcript_variant,2KB_upstream_variant
rs1185038228	genic_upstream_transcript_variant,intron_variant
rs1185094780	genic_upstream_transcript_variant,intron_variant,upstream_transcript_variant
rs1185113109	genic_upstream_transcript_variant,intron_variant
rs1185122218	intron_variant,genic_downstream_transcript_variant
rs1185124782	intron_variant
rs1185136442	genic_upstream_transcript_variant,intron_variant,upstream_transcript_variant,2KB_upstream_variant
rs1185137783	intron_variant
rs1185159155	genic_upstream_transcript_variant,intron_variant
rs1185255167	genic_upstream_transcript_variant,intron_variant
rs1185302327	genic_upstream_transcript_variant,intron_variant
rs1185329274	intron_variant,genic_downstream_transcript_variant
rs1185332869	non_coding_transcript_variant,missense_variant,coding_sequence_variant,genic_downstream_transcript_variant
rs1185350191	genic_upstream_transcript_variant,intron_variant,upstream_transcript_variant,2KB_upstream_variant
rs1185364519	genic_upstream_transcript_variant,intron_variant
rs1185376042	non_coding_transcript_variant,genic_downstream_transcript_variant,3_prime_UTR_variant
rs1185378046	intron_variant
rs1185405023	genic_upstream_transcript_variant,intron_variant
rs1185411980	intron_variant,genic_downstream_transcript_variant
rs1185427296	intron_variant,genic_downstream_transcript_variant
rs1185463944	intron_variant
rs1185474446	intron_variant,genic_downstream_transcript_variant
rs1185475324	non_coding_transcript_variant,synonymous_variant,coding_sequence_variant,genic_downstream_transcript_variant
rs1185486891	intron_variant
rs1185512794	genic_upstream_transcript_variant,intron_variant
rs1185542918	intron_variant
rs1185569741	genic_upstream_transcript_variant,intron_variant
rs1185584399	intron_variant,genic_downstream_transcript_variant
rs1185600595	genic_upstream_transcript_variant,intron_variant
rs1185613967	genic_upstream_transcript_variant,intron_variant,5_prime_UTR_variant,upstream_transcript_variant
rs1185633833	genic_upstream_transcript_variant,intron_variant
rs1185673160	intron_variant,genic_downstream_transcript_variant
rs1185697520	intron_variant
rs1185748076	genic_upstream_transcript_variant,intron_variant,upstream_transcript_variant
rs1185749827	genic_upstream_transcript_variant,intron_variant
rs1185766732	genic_upstream_transcript_variant,intron_variant
rs1185809498	intron_variant
rs1185819293	genic_upstream_transcript_variant,intron_variant
rs1185848010	intron_variant,genic_downstream_transcript_variant
rs1185873097	genic_upstream_transcript_variant,intron_variant
rs1185928520	genic_upstream_transcript_variant,intron_variant
rs1185930107	genic_upstream_transcript_variant,intron_variant
rs1185942285	intron_variant,genic_downstream_transcript_variant,downstream_transcript_variant
rs1185955047	intron_variant

rs1185989542	genic_upstream_transcript_variant,intron_variant
rs1185993226	intron_variant
rs1186001279	intron_variant
rs1186004212	intron_variant
rs1186019769	intron_variant
rs1186030251	intron_variant
rs1186035834	intron_variant
rs1186038432	intron_variant
rs1186106153	intron_variant
rs1186162203	intron_variant
rs1186178731	genic_upstream_transcript_variant,intron_variant,upstream_transcript_variant
rs1186233516	genic_upstream_transcript_variant,intron_variant
rs1186244987	intron_variant,genic_downstream_transcript_variant,downstream_transcript_variant
rs1186277583	intron_variant
rs1186283555	intron_variant
rs1186293841	intron_variant,genic_downstream_transcript_variant
rs1186299119	genic_upstream_transcript_variant,intron_variant
rs1186329287	genic_upstream_transcript_variant,intron_variant
rs1186348281	intron_variant,genic_downstream_transcript_variant
rs1186351244	genic_upstream_transcript_variant,intron_variant
rs1186383397	intron_variant
rs1186391190	intron_variant
rs1186404464	genic_upstream_transcript_variant,intron_variant
rs1186419363	intron_variant,genic_downstream_transcript_variant
rs1186500182	intron_variant
rs1186503486	genic_upstream_transcript_variant,intron_variant
rs1186534812	genic_upstream_transcript_variant,intron_variant
rs1186539590	genic_upstream_transcript_variant,intron_variant
rs1186545504	genic_upstream_transcript_variant,intron_variant
rs1186556354	non_coding_transcript_variant,genic_downstream_transcript_variant,3_prime_UTR_variant
rs1186617133	genic_upstream_transcript_variant,intron_variant
rs1186643105	intron_variant,genic_downstream_transcript_variant
rs1186662704	intron_variant,genic_downstream_transcript_variant
rs1186664981	genic_upstream_transcript_variant,intron_variant
rs1186781402	non_coding_transcript_variant,synonymous_variant,coding_sequence_variant,genic_downstream_transcript_variant
rs1186797358	intron_variant
rs1186805665	intron_variant
rs1186884591	genic_upstream_transcript_variant,intron_variant
rs1186911329	synonymous_variant,coding_sequence_variant,non_coding_transcript_variant
rs1186969699	intron_variant
rs1186978034	coding_sequence_variant,intron_variant,genic_upstream_transcript_variant,missense_variant,non_coding_transcript_variant
rs1186982648	genic_upstream_transcript_variant,intron_variant
rs1187028971	intron_variant,genic_downstream_transcript_variant
rs1187052314	genic_upstream_transcript_variant,intron_variant
rs1187064095	intron_variant,genic_downstream_transcript_variant
rs1187080461	intron_variant
rs1187102621	intron_variant
rs1187113396	intron_variant
rs1187134285	genic_upstream_transcript_variant,intron_variant
rs1187180018	intron_variant
rs1187258289	intron_variant
rs1187275743	intron_variant
rs1187285584	genic_upstream_transcript_variant,intron_variant
rs1187287184	intron_variant,genic_downstream_transcript_variant
rs1187316785	intron_variant
rs1187364611	intron_variant
rs1187389058	genic_upstream_transcript_variant,intron_variant
rs1187400392	intron_variant
rs1187407134	intron_variant,genic_downstream_transcript_variant
rs1187413182	intron_variant,genic_downstream_transcript_variant
rs1187413403	genic_upstream_transcript_variant,intron_variant
rs1187458149	intron_variant,genic_downstream_transcript_variant
rs1187524970	genic_upstream_transcript_variant,intron_variant
rs1187530086	intron_variant,genic_downstream_transcript_variant
rs1187547063	genic_upstream_transcript_variant,intron_variant
rs1187552730	intron_variant
rs1187627139	intron_variant,genic_downstream_transcript_variant
rs1187640312	genic_upstream_transcript_variant,intron_variant
rs1187683666	intron_variant,genic_downstream_transcript_variant
rs1187706154	intron_variant
rs1187783281	genic_upstream_transcript_variant,intron_variant,upstream_transcript_variant,2KB_upstream_variant
rs1187788796	genic_upstream_transcript_variant,intron_variant
rs1187789939	intron_variant
rs1187866189	genic_upstream_transcript_variant,intron_variant

rs1187891585	intron_variant
rs1187906951	intron_variant
rs1187910563	genic_upstream_transcript_variant,intron_variant
rs1187910936	genic_upstream_transcript_variant,intron_variant
rs1187920771	genic_upstream_transcript_variant,intron_variant
rs1187924542	genic_upstream_transcript_variant,intron_variant
rs1187952229	intron_variant
rs1187977848	intron_variant
rs1188098995	intron_variant
rs1188105869	intron_variant
rs1188135173	intron_variant
rs1188173431	non_coding_transcript_variant,genic_downstream_transcript_variant,3_prime_UTR_variant
rs1188192255	genic_upstream_transcript_variant,intron_variant
rs1188199660	intron_variant
rs1188238914	genic_upstream_transcript_variant,intron_variant
rs1188280003	intron_variant,genic_downstream_transcript_variant
rs1188318770	genic_upstream_transcript_variant,intron_variant,upstream_transcript_variant
rs1188411615	genic_upstream_transcript_variant,intron_variant
rs1188416984	intron_variant
rs1188418376	intron_variant
rs1188426370	genic_upstream_transcript_variant,intron_variant
rs1188432095	non_coding_transcript_variant,synonymous_variant,coding_sequence_variant,genic_downstream_transcript_variant
rs1188492067	genic_upstream_transcript_variant,intron_variant
rs1188495304	intron_variant
rs1188499050	genic_upstream_transcript_variant,intron_variant
rs1188522406	intron_variant
rs1188552152	genic_upstream_transcript_variant,intron_variant
rs1188556392	genic_upstream_transcript_variant,intron_variant
rs1188598291	intron_variant,genic_downstream_transcript_variant
rs1188616642	genic_upstream_transcript_variant,intron_variant
rs1188670893	intron_variant,genic_downstream_transcript_variant
rs1188683004	intron_variant
rs1188692557	genic_upstream_transcript_variant,intron_variant
rs1188701260	genic_upstream_transcript_variant,intron_variant
rs1188703906	intron_variant,genic_downstream_transcript_variant
rs1188713421	intron_variant
rs1188749918	intron_variant
rs1188824082	non_coding_transcript_variant,genic_downstream_transcript_variant,3_prime_UTR_variant
rs1188855432	intron_variant
rs1188882005	genic_upstream_transcript_variant,intron_variant
rs1188903987	intron_variant,genic_downstream_transcript_variant
rs1188924741	non_coding_transcript_variant,genic_downstream_transcript_variant,3_prime_UTR_variant
rs1188931794	intron_variant,genic_downstream_transcript_variant
rs1188940748	intron_variant,genic_downstream_transcript_variant
rs1188963992	intron_variant,genic_downstream_transcript_variant
rs1188974766	intron_variant,genic_downstream_transcript_variant
rs1188991544	intron_variant
rs1188994543	intron_variant,genic_downstream_transcript_variant
rs1189027178	genic_upstream_transcript_variant,intron_variant
rs1189042271	genic_upstream_transcript_variant,intron_variant
rs1189071750	intron_variant
rs1189093834	genic_upstream_transcript_variant,intron_variant
rs1189153016	intron_variant,genic_downstream_transcript_variant
rs1189239268	intron_variant,genic_downstream_transcript_variant
rs1189271616	intron_variant
rs1189287335	intron_variant
rs1189309346	genic_upstream_transcript_variant,intron_variant
rs1189321026	genic_upstream_transcript_variant,intron_variant
rs1189329783	intron_variant
rs1189351867	intron_variant,genic_downstream_transcript_variant
rs1189359898	intron_variant
rs1189364030	intron_variant,genic_downstream_transcript_variant
rs1189410832	intron_variant,genic_downstream_transcript_variant
rs1189432573	intron_variant
rs1189434811	intron_variant
rs1189442726	genic_upstream_transcript_variant,intron_variant
rs1189450252	genic_upstream_transcript_variant,intron_variant
rs1189478343	genic_upstream_transcript_variant,intron_variant
rs1189489197	genic_upstream_transcript_variant,intron_variant
rs1189518657	intron_variant
rs1189521067	intron_variant
rs1189527607	intron_variant
rs1189533257	intron_variant
rs1189539887	genic_upstream_transcript_variant,intron_variant,upstream_transcript_variant

rs1189573292	intron_variant
rs1189614710	genic_upstream_transcript_variant,intron_variant
rs1189614815	intron_variant,genic_downstream_transcript_variant
rs1189682261	genic_upstream_transcript_variant,intron_variant
rs1189706970	genic_upstream_transcript_variant,intron_variant
rs1189773940	genic_upstream_transcript_variant,intron_variant
rs1189819895	intron_variant,genic_downstream_transcript_variant
rs1189827328	intron_variant,genic_downstream_transcript_variant
rs1189842704	genic_upstream_transcript_variant,intron_variant
rs1189843223	intron_variant,genic_downstream_transcript_variant
rs1189855432	intron_variant
rs1189872940	genic_upstream_transcript_variant,intron_variant
rs1189878919	intron_variant
rs1189900234	intron_variant
rs1189967753	non_coding_transcript_variant,genic_downstream_transcript_variant,3_prime_UTR_variant
rs1189997744	intron_variant
rs1190041274	genic_upstream_transcript_variant,intron_variant,upstream_transcript_variant
rs1190056549	genic_upstream_transcript_variant,intron_variant,upstream_transcript_variant,2KB_upstream_variant
rs1190099358	intron_variant
rs1190102177	intron_variant
rs1190125463	genic_upstream_transcript_variant,intron_variant
rs1190129568	intron_variant
rs1190151541	intron_variant
rs1190165468	intron_variant
rs1190211329	intron_variant
rs1190214640	intron_variant,genic_downstream_transcript_variant
rs1190215128	genic_upstream_transcript_variant,intron_variant
rs1190215773	genic_upstream_transcript_variant,intron_variant,upstream_transcript_variant
rs1190224150	genic_upstream_transcript_variant,intron_variant
rs1190232962	intron_variant
rs1190248948	intron_variant
rs1190269987	genic_upstream_transcript_variant,intron_variant
rs1190275940	genic_upstream_transcript_variant,intron_variant,upstream_transcript_variant
rs1190293995	genic_upstream_transcript_variant,intron_variant,5_prime_UTR_variant,non_coding_transcript_variant
rs1190302422	genic_upstream_transcript_variant,intron_variant
rs1190312920	missense_variant,intron_variant,coding_sequence_variant
rs1190322356	intron_variant
rs1190345221	genic_upstream_transcript_variant,intron_variant
rs1190350092	genic_upstream_transcript_variant,intron_variant
rs1190369853	intron_variant
rs1190375756	genic_upstream_transcript_variant,intron_variant
rs1190383990	genic_upstream_transcript_variant,intron_variant
rs1190394192	genic_upstream_transcript_variant,intron_variant,upstream_transcript_variant,2KB_upstream_variant
rs1190419241	genic_upstream_transcript_variant,intron_variant
rs1190433463	intron_variant
rs1190542558	genic_upstream_transcript_variant,intron_variant
rs1190594506	genic_upstream_transcript_variant,intron_variant
rs1190660146	intron_variant
rs1190662710	genic_upstream_transcript_variant,intron_variant
rs1190722053	intron_variant,genic_downstream_transcript_variant
rs1190746319	intron_variant
rs1190753468	genic_upstream_transcript_variant,intron_variant
rs1190758534	non_coding_transcript_variant,genic_downstream_transcript_variant,3_prime_UTR_variant
rs1190767833	genic_upstream_transcript_variant,intron_variant
rs1190783798	genic_upstream_transcript_variant,intron_variant
rs1190821770	intron_variant
rs1190849945	genic_upstream_transcript_variant,intron_variant
rs1190870151	intron_variant
rs1190898645	intron_variant,genic_downstream_transcript_variant
rs1190913769	genic_upstream_transcript_variant,intron_variant
rs1190917231	genic_upstream_transcript_variant,intron_variant
rs1190932576	genic_upstream_transcript_variant,intron_variant,upstream_transcript_variant
rs1190983579	genic_upstream_transcript_variant,intron_variant
rs1190992162	genic_upstream_transcript_variant,intron_variant
rs1191002994	intron_variant
rs1191034725	genic_upstream_transcript_variant,intron_variant
rs1191077226	intron_variant
rs1191157440	intron_variant,genic_downstream_transcript_variant
rs1191212638	genic_upstream_transcript_variant,intron_variant
rs1191215964	intron_variant
rs1191251944	genic_upstream_transcript_variant,intron_variant
rs1191268181	genic_upstream_transcript_variant,intron_variant
rs1191288875	intron_variant
rs1191329800	genic_upstream_transcript_variant,intron_variant

rs1191332920	intron_variant
rs1191342063	genic_upstream_transcript_variant,intron_variant
rs1191375188	intron_variant
rs1191381216	non_coding_transcript_variant,missense_variant,coding_sequence_variant,genic_downstream_transcript_variant
rs1191390259	non_coding_transcript_variant,genic_downstream_transcript_variant,3_prime_UTR_variant
rs1191430646	intron_variant,genic_downstream_transcript_variant
rs1191452162	intron_variant
rs1191491473	genic_upstream_transcript_variant,intron_variant
rs1191500434	genic_upstream_transcript_variant,intron_variant
rs1191560881	intron_variant
rs1191561099	genic_upstream_transcript_variant,intron_variant
rs1191569963	genic_upstream_transcript_variant,intron_variant,upstream_transcript_variant
rs1191573394	intron_variant
rs1191579792	genic_upstream_transcript_variant,intron_variant
rs1191592629	non_coding_transcript_variant,missense_variant,coding_sequence_variant,genic_downstream_transcript_variant
rs1191660181	genic_upstream_transcript_variant,intron_variant
rs1191675784	genic_upstream_transcript_variant,intron_variant,upstream_transcript_variant
rs1191685647	intron_variant
rs1191690392	intron_variant
rs1191742801	intron_variant,genic_downstream_transcript_variant
rs1191751281	intron_variant,genic_downstream_transcript_variant
rs1191752543	genic_upstream_transcript_variant,intron_variant
rs1191764562	genic_upstream_transcript_variant,intron_variant
rs1191770065	intron_variant,genic_downstream_transcript_variant
rs1191775765	genic_upstream_transcript_variant,intron_variant,upstream_transcript_variant
rs1191833507	genic_upstream_transcript_variant,intron_variant
rs1191847561	intron_variant,5_prime_UTR_variant
rs1191916384	intron_variant
rs1191935030	intron_variant
rs1191956704	synonymous_variant,coding_sequence_variant,non_coding_transcript_variant
rs1191975404	genic_upstream_transcript_variant,intron_variant,upstream_transcript_variant,2KB_upstream_variant
rs1191980170	genic_upstream_transcript_variant,intron_variant
rs1192022152	genic_upstream_transcript_variant,intron_variant
rs1192060925	intron_variant
rs1192103124	intron_variant
rs1192157862	genic_upstream_transcript_variant,intron_variant,5_prime_UTR_variant,non_coding_transcript_variant
rs1192191666	intron_variant
rs1192194220	intron_variant,genic_downstream_transcript_variant
rs1192207235	intron_variant
rs1192214422	genic_upstream_transcript_variant,intron_variant
rs1192224668	intron_variant,genic_downstream_transcript_variant
rs1192233044	intron_variant
rs1192291588	intron_variant
rs1192295423	genic_upstream_transcript_variant,intron_variant
rs1192350534	intron_variant
rs1192367512	non_coding_transcript_variant,coding_sequence_variant,frameshift_variant,genic_downstream_transcript_variant
rs1192411510	genic_upstream_transcript_variant,intron_variant
rs1192457673	genic_upstream_transcript_variant,intron_variant
rs1192473941	intron_variant
rs1192501752	intron_variant,genic_downstream_transcript_variant
rs1192531098	genic_upstream_transcript_variant,intron_variant
rs1192547389	intron_variant,genic_downstream_transcript_variant
rs1192551660	genic_upstream_transcript_variant,intron_variant
rs1192572475	intron_variant,genic_downstream_transcript_variant
rs1192576002	genic_upstream_transcript_variant,intron_variant
rs1192601141	genic_upstream_transcript_variant,intron_variant,upstream_transcript_variant,2KB_upstream_variant
rs1192606771	intron_variant
rs1192618097	genic_upstream_transcript_variant,intron_variant
rs1192639240	intron_variant
rs1192679835	intron_variant,genic_downstream_transcript_variant
rs1192717671	genic_upstream_transcript_variant,intron_variant
rs1192724270	intron_variant
rs1192749887	genic_upstream_transcript_variant,intron_variant
rs1192764854	500B_downstream_variant,downstream_transcript_variant
rs1192812083	genic_upstream_transcript_variant,intron_variant
rs1192840592	genic_upstream_transcript_variant,intron_variant
rs1192904318	intron_variant,genic_downstream_transcript_variant
rs1192914888	intron_variant
rs1192949955	genic_upstream_transcript_variant,intron_variant
rs1192983002	genic_upstream_transcript_variant,intron_variant,upstream_transcript_variant,2KB_upstream_variant
rs1192989958	intron_variant,genic_downstream_transcript_variant
rs1193003334	intron_variant,genic_downstream_transcript_variant
rs1193007848	genic_upstream_transcript_variant,intron_variant,upstream_transcript_variant,2KB_upstream_variant
rs1193014160	intron_variant,genic_downstream_transcript_variant

rs1193039018	genic_upstream_transcript_variant,intron_variant
rs1193040287	intron_variant,genic_downstream_transcript_variant
rs1193129411	genic_upstream_transcript_variant,intron_variant
rs1193138320	intron_variant,genic_downstream_transcript_variant
rs1193153581	genic_upstream_transcript_variant,intron_variant
rs1193158158	genic_upstream_transcript_variant,intron_variant
rs1193187089	intron_variant,genic_downstream_transcript_variant
rs1193267375	genic_upstream_transcript_variant,intron_variant,5_prime_UTR_variant,non_coding_transcript_variant
rs1193304569	intron_variant,genic_downstream_transcript_variant
rs1193349558	intron_variant
rs1193349886	genic_upstream_transcript_variant,intron_variant,upstream_transcript_variant,2KB_upstream_variant
rs1193360661	genic_upstream_transcript_variant,intron_variant
rs1193365228	genic_upstream_transcript_variant,intron_variant
rs1193393702	genic_upstream_transcript_variant,intron_variant
rs1193395384	intron_variant,genic_downstream_transcript_variant
rs1193416712	intron_variant
rs1193437287	genic_upstream_transcript_variant,intron_variant
rs1193477750	intron_variant
rs1193482743	intron_variant
rs1193509014	intron_variant
rs1193534551	genic_upstream_transcript_variant,intron_variant
rs1193542215	non_coding_transcript_variant,missense_variant,coding_sequence_variant,genic_downstream_transcript_variant
rs1193546764	intron_variant
rs1193612115	intron_variant
rs1193621896	genic_upstream_transcript_variant,intron_variant
rs1193625461	intron_variant,genic_downstream_transcript_variant
rs1193628982	genic_upstream_transcript_variant,intron_variant
rs1193630157	intron_variant
rs1193634474	intron_variant
rs1193679059	genic_upstream_transcript_variant,intron_variant
rs1193685626	intron_variant,genic_downstream_transcript_variant
rs1193687414	genic_upstream_transcript_variant,intron_variant
rs1193700346	intron_variant
rs1193734746	intron_variant
rs1193737975	genic_upstream_transcript_variant,intron_variant
rs1193747923	genic_upstream_transcript_variant,intron_variant
rs1193748212	intron_variant
rs1193777218	intron_variant
rs1193822708	genic_upstream_transcript_variant,intron_variant,upstream_transcript_variant
rs1193828928	non_coding_transcript_variant,genic_downstream_transcript_variant,3_prime_UTR_variant
rs1193869366	genic_upstream_transcript_variant,intron_variant
rs1193890866	intron_variant,genic_downstream_transcript_variant
rs1193904596	genic_upstream_transcript_variant,intron_variant
rs1193904916	non_coding_transcript_variant,missense_variant,coding_sequence_variant,genic_downstream_transcript_variant
rs1193934282	intron_variant,genic_downstream_transcript_variant
rs1193960073	intron_variant
rs1193980038	intron_variant
rs1193999836	intron_variant,genic_downstream_transcript_variant
rs1194000273	intron_variant,genic_downstream_transcript_variant
rs1194006543	genic_upstream_transcript_variant,intron_variant
rs1194055017	intron_variant,genic_downstream_transcript_variant
rs1194104749	intron_variant
rs1194128558	genic_upstream_transcript_variant,intron_variant
rs1194132634	intron_variant
rs1194151800	intron_variant,genic_downstream_transcript_variant
rs1194177006	genic_upstream_transcript_variant,intron_variant
rs1194226239	genic_upstream_transcript_variant,intron_variant
rs1194235444	genic_upstream_transcript_variant,intron_variant
rs1194243308	intron_variant
rs1194251734	intron_variant,genic_downstream_transcript_variant
rs1194254943	intron_variant,genic_downstream_transcript_variant
rs1194286952	intron_variant,genic_downstream_transcript_variant
rs1194291417	genic_upstream_transcript_variant,intron_variant
rs1194312782	intron_variant
rs1194340293	intron_variant,genic_downstream_transcript_variant
rs1194342773	intron_variant
rs1194400079	genic_upstream_transcript_variant,intron_variant
rs1194465754	intron_variant,genic_downstream_transcript_variant
rs1194481505	intron_variant
rs1194504438	intron_variant,genic_downstream_transcript_variant
rs1194512343	intron_variant,genic_downstream_transcript_variant
rs1194538892	intron_variant,genic_downstream_transcript_variant
rs1194547373	intron_variant
rs1194606703	genic_upstream_transcript_variant,intron_variant

rs1194620846	genic_upstream_transcript_variant,intron_variant
rs1194630876	intron_variant
rs1194709506	intron_variant,genic_downstream_transcript_variant,downstream_transcript_variant
rs1194715008	intron_variant
rs1194721704	intron_variant
rs1194774036	genic_upstream_transcript_variant,intron_variant
rs1194825719	intron_variant
rs1194851948	genic_upstream_transcript_variant,intron_variant,upstream_transcript_variant,2KB_upstream_variant
rs1194860722	genic_upstream_transcript_variant,intron_variant
rs1194930026	intron_variant
rs1194949325	intron_variant
rs1194949802	genic_upstream_transcript_variant,intron_variant
rs1195000182	genic_upstream_transcript_variant,intron_variant
rs1195092347	intron_variant
rs1195103668	genic_upstream_transcript_variant,intron_variant
rs1195146507	genic_upstream_transcript_variant,intron_variant
rs1195186990	intron_variant
rs1195218785	genic_upstream_transcript_variant,intron_variant,upstream_transcript_variant
rs1195250265	genic_upstream_transcript_variant,intron_variant
rs1195259259	intron_variant
rs1195285784	intron_variant
rs1195304926	genic_upstream_transcript_variant,intron_variant,upstream_transcript_variant,2KB_upstream_variant
rs1195330592	genic_upstream_transcript_variant,intron_variant,upstream_transcript_variant,2KB_upstream_variant
rs1195357465	non_coding_transcript_variant,genic_downstream_transcript_variant,3_prime_UTR_variant
rs1195364667	intron_variant
rs1195366434	genic_upstream_transcript_variant,intron_variant
rs1195406147	intron_variant,genic_downstream_transcript_variant
rs1195447344	intron_variant
rs1195521453	intron_variant
rs1195561194	genic_upstream_transcript_variant,intron_variant,upstream_transcript_variant,2KB_upstream_variant
rs1195612306	genic_upstream_transcript_variant,intron_variant
rs1195621341	genic_upstream_transcript_variant,intron_variant
rs1195649847	genic_upstream_transcript_variant,intron_variant
rs1195725631	genic_upstream_transcript_variant,intron_variant,upstream_transcript_variant,2KB_upstream_variant
rs1195727512	genic_upstream_transcript_variant,intron_variant
rs1195758666	genic_upstream_transcript_variant,intron_variant
rs1195791926	genic_upstream_transcript_variant,intron_variant,upstream_transcript_variant,2KB_upstream_variant
rs1195806242	intron_variant
rs1195819987	genic_upstream_transcript_variant,intron_variant
rs1195856179	intron_variant
rs1195866207	genic_upstream_transcript_variant,intron_variant
rs1195889928	intron_variant
rs1195925217	intron_variant,genic_downstream_transcript_variant
rs1195955839	genic_upstream_transcript_variant,intron_variant
rs1195959999	genic_upstream_transcript_variant,intron_variant
rs1195995476	non_coding_transcript_variant,genic_downstream_transcript_variant,3_prime_UTR_variant
rs1196006546	genic_upstream_transcript_variant,intron_variant
rs1196017220	intron_variant,genic_downstream_transcript_variant
rs1196049497	genic_upstream_transcript_variant,intron_variant
rs1196054321	genic_upstream_transcript_variant,intron_variant,upstream_transcript_variant
rs1196066128	intron_variant
rs1196110884	genic_upstream_transcript_variant,intron_variant,upstream_transcript_variant
rs1196119414	intron_variant,genic_downstream_transcript_variant
rs1196132800	intron_variant
rs1196138098	intron_variant,genic_downstream_transcript_variant
rs1196149751	genic_upstream_transcript_variant,intron_variant,5_prime_UTR_variant,non_coding_transcript_variant
rs1196163495	genic_upstream_transcript_variant,intron_variant,upstream_transcript_variant
rs1196165469	intron_variant
rs1196168142	non_coding_transcript_variant,missense_variant,coding_sequence_variant,genic_downstream_transcript_variant
rs1196169572	genic_upstream_transcript_variant,intron_variant,upstream_transcript_variant,2KB_upstream_variant
rs1196187276	intron_variant
rs1196188378	genic_upstream_transcript_variant,intron_variant
rs1196217305	genic_downstream_transcript_variant,intron_variant
rs1196218927	genic_upstream_transcript_variant,intron_variant
rs1196238382	genic_upstream_transcript_variant,intron_variant
rs1196302670	intron_variant
rs1196323009	genic_downstream_transcript_variant,intron_variant
rs1196325488	genic_upstream_transcript_variant,intron_variant
rs1196347819	genic_upstream_transcript_variant,intron_variant
rs1196351061	genic_downstream_transcript_variant,intron_variant
rs1196381520	intron_variant
rs1196381704	intron_variant
rs1196391249	genic_upstream_transcript_variant,intron_variant
rs1196399036	synonymous_variant,non_coding_transcript_variant,coding_sequence_variant

rs1196440713	intron_variant
rs1196510883	intron_variant
rs1196516677	intron_variant
rs1196543273	intron_variant,missense_variant,coding_sequence_variant
rs1196556720	genic_upstream_transcript_variant,intron_variant
rs1196567962	genic_upstream_transcript_variant,2KB_upstream_variant,intron_variant,upstream_transcript_variant
rs1196581212	intron_variant
rs1196611711	intron_variant
rs1196622812	intron_variant
rs1196674795	genic_downstream_transcript_variant,intron_variant
rs1196676183	intron_variant
rs1196678036	genic_upstream_transcript_variant,intron_variant
rs1196726849	intron_variant
rs1196740251	3_prime_UTR_variant,genic_downstream_transcript_variant,non_coding_transcript_variant
rs1196754108	intron_variant
rs1196804729	genic_upstream_transcript_variant,intron_variant
rs1196809163	genic_upstream_transcript_variant,intron_variant
rs1196826445	intron_variant
rs1196837257	intron_variant
rs1196884309	genic_upstream_transcript_variant,intron_variant
rs1196895479	genic_upstream_transcript_variant,intron_variant
rs1196895647	genic_upstream_transcript_variant,intron_variant
rs1196922083	genic_upstream_transcript_variant,intron_variant
rs1196956233	intron_variant
rs1196960071	intron_variant
rs1197032056	genic_downstream_transcript_variant,intron_variant
rs1197063691	genic_upstream_transcript_variant,2KB_upstream_variant,intron_variant,upstream_transcript_variant
rs1197081060	genic_upstream_transcript_variant,intron_variant
rs1197175970	intron_variant
rs1197190961	genic_downstream_transcript_variant,intron_variant
rs1197204514	genic_upstream_transcript_variant,intron_variant
rs1197207766	genic_downstream_transcript_variant,missense_variant,non_coding_transcript_variant,coding_sequence_variant
rs1197227100	genic_upstream_transcript_variant,intron_variant
rs1197296975	intron_variant
rs1197303180	genic_downstream_transcript_variant,intron_variant
rs1197308040	genic_downstream_transcript_variant,intron_variant
rs1197347647	genic_upstream_transcript_variant,intron_variant
rs1197371608	genic_upstream_transcript_variant,intron_variant
rs1197407327	genic_downstream_transcript_variant,intron_variant,downstream_transcript_variant
rs1197451596	intron_variant
rs1197465232	intron_variant
rs1197468171	intron_variant
rs1197527470	genic_upstream_transcript_variant,intron_variant
rs1197564531	genic_upstream_transcript_variant,intron_variant
rs1197609739	genic_downstream_transcript_variant,intron_variant
rs1197612327	genic_downstream_transcript_variant,intron_variant
rs1197647926	genic_upstream_transcript_variant,intron_variant,upstream_transcript_variant,5_prime_UTR_variant
rs1197679254	intron_variant
rs1197734792	intron_variant
rs1197760361	intron_variant
rs1197771094	genic_downstream_transcript_variant,intron_variant
rs1197806118	genic_upstream_transcript_variant,intron_variant
rs1197875060	genic_upstream_transcript_variant,intron_variant
rs1197924272	genic_downstream_transcript_variant,intron_variant,downstream_transcript_variant
rs1197932364	genic_downstream_transcript_variant,intron_variant
rs1197950961	intron_variant
rs1197969978	genic_upstream_transcript_variant,2KB_upstream_variant,intron_variant,upstream_transcript_variant
rs1197998795	intron_variant
rs1198024271	genic_upstream_transcript_variant,intron_variant
rs1198037711	genic_upstream_transcript_variant,intron_variant,upstream_transcript_variant
rs1198038217	genic_downstream_transcript_variant,intron_variant,downstream_transcript_variant
rs1198045160	genic_upstream_transcript_variant,intron_variant
rs1198139331	genic_upstream_transcript_variant,intron_variant
rs1198182203	3_prime_UTR_variant,genic_downstream_transcript_variant,downstream_transcript_variant,non_coding_transcript_variant
rs1198220547	500B_downstream_variant,downstream_transcript_variant
rs1198224832	genic_downstream_transcript_variant,intron_variant
rs1198231654	genic_downstream_transcript_variant,intron_variant
rs1198238475	intron_variant
rs1198246861	genic_upstream_transcript_variant,intron_variant
rs1198255084	genic_upstream_transcript_variant,intron_variant
rs1198259103	genic_upstream_transcript_variant,intron_variant,upstream_transcript_variant
rs1198280806	intron_variant
rs1198359690	genic_upstream_transcript_variant,intron_variant
rs1198414243	genic_upstream_transcript_variant,intron_variant

rs1198446430	intron_variant
rs1198470248	intron_variant
rs1198473740	genic_upstream_transcript_variant,intron_variant,upstream_transcript_variant
rs1198476899	intron_variant
rs1198583456	intron_variant
rs1198599842	genic_upstream_transcript_variant,intron_variant
rs1198623523	genic_upstream_transcript_variant,intron_variant
rs1198653975	genic_downstream_transcript_variant,intron_variant
rs1198656512	intron_variant
rs1198687410	genic_upstream_transcript_variant,intron_variant
rs1198700958	genic_upstream_transcript_variant,intron_variant,upstream_transcript_variant
rs1198719289	genic_downstream_transcript_variant,intron_variant
rs1198741631	genic_upstream_transcript_variant,intron_variant
rs1198767258	genic_downstream_transcript_variant,intron_variant
rs1198768041	genic_upstream_transcript_variant,intron_variant
rs1198780945	3_prime_UTR_variant,genic_downstream_transcript_variant,non_coding_transcript_variant
rs1198789002	genic_upstream_transcript_variant,intron_variant,upstream_transcript_variant,5_prime_UTR_variant
rs1198793925	intron_variant
rs1198808105	genic_upstream_transcript_variant,intron_variant
rs1198823208	intron_variant
rs1198840037	intron_variant
rs1198872722	intron_variant
rs1198904741	genic_upstream_transcript_variant,intron_variant
rs1198913524	genic_upstream_transcript_variant,intron_variant
rs1198937338	genic_downstream_transcript_variant,intron_variant
rs1198976464	genic_upstream_transcript_variant,intron_variant
rs1199025915	genic_upstream_transcript_variant,intron_variant
rs1199069315	genic_upstream_transcript_variant,intron_variant
rs1199085979	genic_downstream_transcript_variant,intron_variant
rs1199090063	genic_downstream_transcript_variant,intron_variant
rs1199090098	intron_variant
rs1199097857	genic_downstream_transcript_variant,intron_variant
rs1199104872	genic_upstream_transcript_variant,2KB_upstream_variant,intron_variant,upstream_transcript_variant
rs1199164973	genic_upstream_transcript_variant,intron_variant
rs1199249051	genic_upstream_transcript_variant,intron_variant
rs1199252791	genic_upstream_transcript_variant,2KB_upstream_variant,intron_variant,upstream_transcript_variant
rs1199322178	intron_variant
rs1199327890	genic_upstream_transcript_variant,intron_variant
rs1199333382	genic_downstream_transcript_variant,intron_variant
rs1199341480	3_prime_UTR_variant,genic_downstream_transcript_variant,non_coding_transcript_variant
rs1199356214	genic_upstream_transcript_variant,intron_variant
rs1199417696	intron_variant
rs1199433608	genic_upstream_transcript_variant,intron_variant
rs1199440222	genic_upstream_transcript_variant,intron_variant,upstream_transcript_variant
rs1199447864	intron_variant
rs1199499357	genic_downstream_transcript_variant,intron_variant
rs1199539113	genic_downstream_transcript_variant,intron_variant
rs1199542261	genic_upstream_transcript_variant,intron_variant
rs1199612436	genic_downstream_transcript_variant,intron_variant
rs1199615955	3_prime_UTR_variant,genic_downstream_transcript_variant,non_coding_transcript_variant
rs1199620507	intron_variant
rs1199644578	genic_upstream_transcript_variant,intron_variant
rs1199686934	intron_variant
rs1199706120	intron_variant
rs1199723513	genic_downstream_transcript_variant,missense_variant,non_coding_transcript_variant,coding_sequence_variant
rs1199757361	genic_downstream_transcript_variant,intron_variant
rs1199757803	intron_variant
rs1199781638	genic_upstream_transcript_variant,intron_variant
rs1199798023	intron_variant
rs1199814249	intron_variant
rs1199836609	intron_variant
rs1199870701	intron_variant
rs1199898520	intron_variant
rs1199925989	genic_upstream_transcript_variant,intron_variant
rs1200005623	intron_variant
rs1200067402	genic_upstream_transcript_variant,intron_variant
rs1200077170	intron_variant
rs1200077547	genic_downstream_transcript_variant,intron_variant
rs1200134436	intron_variant
rs1200175556	intron_variant
rs1200178074	genic_downstream_transcript_variant,intron_variant
rs1200192033	genic_upstream_transcript_variant,intron_variant
rs1200204105	genic_upstream_transcript_variant,intron_variant
rs1200229918	genic_upstream_transcript_variant,intron_variant

rs1200273632	intron_variant
rs1200283033	3_prime_UTR_variant,genic_downstream_transcript_variant,non_coding_transcript_variant
rs1200326893	intron_variant
rs1200347824	genic_upstream_transcript_variant,intron_variant
rs1200409365	intron_variant
rs1200410468	intron_variant
rs1200423161	missense_variant,non_coding_transcript_variant,genic_upstream_transcript_variant,intron_variant,coding_sequence_variant
rs1200430980	genic_upstream_transcript_variant,intron_variant
rs1200472808	genic_upstream_transcript_variant,intron_variant
rs1200590288	3_prime_UTR_variant,genic_downstream_transcript_variant,non_coding_transcript_variant
rs1200681030	genic_upstream_transcript_variant,intron_variant,upstream_transcript_variant
rs1200683299	intron_variant
rs1200713854	genic_downstream_transcript_variant,intron_variant
rs1200716934	intron_variant
rs1200749425	intron_variant
rs1200759385	genic_downstream_transcript_variant,intron_variant
rs1200763446	genic_downstream_transcript_variant,intron_variant
rs1200808228	genic_downstream_transcript_variant,intron_variant
rs1200845181	genic_upstream_transcript_variant,intron_variant
rs1200898159	intron_variant
rs1200913489	genic_upstream_transcript_variant,intron_variant
rs1200926886	genic_downstream_transcript_variant,intron_variant
rs1200928416	intron_variant
rs1200935984	intron_variant
rs1200965661	3_prime_UTR_variant,genic_downstream_transcript_variant,non_coding_transcript_variant
rs1200987388	intron_variant
rs1200994299	frameshift_variant,non_coding_transcript_variant,coding_sequence_variant
rs1200997897	genic_upstream_transcript_variant,intron_variant
rs1201003869	intron_variant
rs1201004667	intron_variant
rs1201010637	genic_upstream_transcript_variant,2KB_upstream_variant,intron_variant,upstream_transcript_variant
rs1201032934	genic_upstream_transcript_variant,intron_variant
rs1201094283	genic_upstream_transcript_variant,intron_variant
rs1201130135	genic_upstream_transcript_variant,intron_variant
rs1201200555	genic_upstream_transcript_variant,intron_variant
rs1201226455	genic_upstream_transcript_variant,intron_variant
rs1201239692	genic_upstream_transcript_variant,intron_variant
rs1201276838	genic_upstream_transcript_variant,intron_variant
rs1201279795	intron_variant
rs1201282511	intron_variant
rs1201282824	genic_downstream_transcript_variant,intron_variant
rs1201332424	intron_variant
rs1201335023	intron_variant
rs1201337782	intron_variant
rs1201402460	genic_upstream_transcript_variant,intron_variant
rs1201414304	intron_variant
rs1201415013	intron_variant
rs1201515303	genic_upstream_transcript_variant,intron_variant
rs1201573173	intron_variant
rs1201598003	stop_lost,intron_variant,terminator_codon_variant
rs1201620008	genic_upstream_transcript_variant,intron_variant
rs1201632993	genic_downstream_transcript_variant,intron_variant
rs1201635852	genic_upstream_transcript_variant,intron_variant
rs1201688647	genic_upstream_transcript_variant,intron_variant
rs1201702333	genic_downstream_transcript_variant,intron_variant
rs1201717315	genic_upstream_transcript_variant,intron_variant
rs1201735189	genic_upstream_transcript_variant,intron_variant
rs1201744431	intron_variant
rs1201747416	genic_downstream_transcript_variant,intron_variant
rs1201764777	genic_upstream_transcript_variant,intron_variant
rs1201771940	genic_downstream_transcript_variant,intron_variant,downstream_transcript_variant
rs1201775842	genic_downstream_transcript_variant,intron_variant
rs1201810654	genic_upstream_transcript_variant,intron_variant
rs1201889385	intron_variant
rs1201907362	genic_upstream_transcript_variant,intron_variant
rs1201911394	intron_variant
rs1201911964	genic_downstream_transcript_variant,intron_variant
rs1201989602	genic_downstream_transcript_variant,missense_variant,non_coding_transcript_variant,coding_sequence_variant
rs1202001389	genic_upstream_transcript_variant,intron_variant
rs1202009195	genic_upstream_transcript_variant,intron_variant
rs1202020424	genic_upstream_transcript_variant,intron_variant
rs1202035096	genic_upstream_transcript_variant,intron_variant
rs1202074208	intron_variant
rs1202112298	genic_upstream_transcript_variant,intron_variant

rs1202114321	intron_variant
rs1202147608	genic_downstream_transcript_variant,intron_variant
rs1202164246	intron_variant
rs1202185273	genic_upstream_transcript_variant,intron_variant
rs1202196416	intron_variant
rs1202205978	intron_variant
rs1202263102	intron_variant
rs1202270909	intron_variant
rs1202298146	intron_variant
rs1202326206	genic_upstream_transcript_variant,intron_variant
rs1202350959	intron_variant
rs1202351259	intron_variant
rs1202354861	genic_upstream_transcript_variant,intron_variant
rs1202406416	genic_downstream_transcript_variant,intron_variant
rs1202422467	genic_upstream_transcript_variant,intron_variant
rs1202452648	intron_variant
rs1202483224	intron_variant
rs1202502254	intron_variant
rs1202519074	intron_variant
rs1202524241	intron_variant
rs1202655454	intron_variant
rs1202688437	genic_upstream_transcript_variant,intron_variant
rs1202727430	genic_downstream_transcript_variant,intron_variant
rs1202744082	genic_upstream_transcript_variant,intron_variant
rs1202750189	intron_variant
rs1202759964	intron_variant
rs1202789345	intron_variant
rs1202794615	genic_downstream_transcript_variant,intron_variant
rs1202801453	genic_upstream_transcript_variant,intron_variant
rs1202802521	genic_upstream_transcript_variant,intron_variant
rs1202838081	genic_upstream_transcript_variant,intron_variant
rs1202887966	intron_variant
rs1202894637	intron_variant
rs1202932972	genic_upstream_transcript_variant,intron_variant
rs1202956897	genic_downstream_transcript_variant,intron_variant
rs1202957409	intron_variant
rs1202975894	genic_upstream_transcript_variant,intron_variant
rs1203025434	genic_upstream_transcript_variant,intron_variant,upstream_transcript_variant
rs1203028428	genic_downstream_transcript_variant,intron_variant
rs1203041185	3_prime_UTR_variant,genic_downstream_transcript_variant,non_coding_transcript_variant
rs1203067406	500B_downstream_variant,downstream_transcript_variant
rs1203086695	genic_upstream_transcript_variant,intron_variant
rs1203093337	genic_downstream_transcript_variant,intron_variant
rs1203117590	intron_variant
rs1203162796	genic_upstream_transcript_variant,2KB_upstream_variant,intron_variant,upstream_transcript_variant
rs1203165307	genic_downstream_transcript_variant,intron_variant
rs1203175913	genic_upstream_transcript_variant,intron_variant
rs1203209935	intron_variant
rs1203246387	genic_upstream_transcript_variant,intron_variant
rs1203250277	intron_variant
rs1203271681	intron_variant
rs1203330972	genic_upstream_transcript_variant,intron_variant
rs1203331281	intron_variant
rs1203345372	genic_downstream_transcript_variant,intron_variant
rs1203386342	genic_upstream_transcript_variant,intron_variant
rs1203392526	intron_variant
rs1203398258	genic_upstream_transcript_variant,intron_variant
rs1203410917	missense_variant,non_coding_transcript_variant,genic_upstream_transcript_variant,intron_variant,coding_sequence_variant
rs1203460996	genic_upstream_transcript_variant,intron_variant
rs1203461624	genic_downstream_transcript_variant,intron_variant
rs1203469981	intron_variant,missense_variant,coding_sequence_variant
rs1203476400	missense_variant,non_coding_transcript_variant,genic_upstream_transcript_variant,intron_variant,coding_sequence_variant
rs1203500976	intron_variant
rs1203522613	intron_variant
rs1203554387	intron_variant
rs1203589394	genic_upstream_transcript_variant,intron_variant
rs1203592713	intron_variant
rs1203593225	genic_downstream_transcript_variant,intron_variant
rs1203609401	genic_upstream_transcript_variant,intron_variant
rs1203610182	genic_downstream_transcript_variant,missense_variant,non_coding_transcript_variant,coding_sequence_variant
rs1203616054	genic_upstream_transcript_variant,intron_variant
rs1203616586	genic_downstream_transcript_variant,intron_variant
rs1203634586	3_prime_UTR_variant,genic_downstream_transcript_variant,non_coding_transcript_variant
rs1203635437	genic_upstream_transcript_variant,intron_variant

rs1203646458	intron_variant
rs1203663703	genic_downstream_transcript_variant,intron_variant
rs1203671676	genic_downstream_transcript_variant,intron_variant
rs1203700462	genic_downstream_transcript_variant,intron_variant
rs1203709326	intron_variant
rs1203711097	intron_variant
rs1203760423	intron_variant
rs1203763140	intron_variant
rs1203775666	intron_variant
rs1203803458	intron_variant
rs1203922784	genic_downstream_transcript_variant,intron_variant
rs1203950919	intron_variant
rs1203983199	genic_downstream_transcript_variant,intron_variant
rs1203996501	intron_variant
rs1204007272	genic_upstream_transcript_variant,intron_variant
rs1204008241	intron_variant
rs1204121216	intron_variant
rs1204145623	intron_variant
rs1204151443	genic_upstream_transcript_variant,intron_variant
rs1204248942	genic_upstream_transcript_variant,intron_variant
rs1204305969	genic_downstream_transcript_variant,intron_variant
rs1204343680	genic_upstream_transcript_variant,intron_variant
rs1204369989	genic_upstream_transcript_variant,intron_variant
rs1204386242	genic_upstream_transcript_variant,intron_variant
rs1204394272	intron_variant
rs1204449001	intron_variant
rs1204449979	genic_upstream_transcript_variant,intron_variant
rs1204461579	intron_variant
rs1204472220	genic_downstream_transcript_variant,missense_variant,non_coding_transcript_variant,coding_sequence_variant
rs1204499611	intron_variant
rs1204507428	genic_upstream_transcript_variant,intron_variant
rs1204560446	intron_variant
rs1204581312	genic_downstream_transcript_variant,intron_variant
rs1204604476	genic_upstream_transcript_variant,intron_variant
rs1204608351	intron_variant
rs1204617931	genic_upstream_transcript_variant,intron_variant
rs1204643629	intron_variant
rs1204656590	genic_downstream_transcript_variant,intron_variant
rs1204681687	genic_downstream_transcript_variant,intron_variant
rs1204706635	intron_variant
rs1204728771	missense_variant,non_coding_transcript_variant,coding_sequence_variant
rs1204731573	genic_upstream_transcript_variant,intron_variant
rs1204874658	intron_variant
rs1204914840	genic_upstream_transcript_variant,intron_variant
rs1204969842	genic_upstream_transcript_variant,intron_variant
rs1204974833	genic_downstream_transcript_variant,intron_variant
rs1205026700	genic_upstream_transcript_variant,intron_variant
rs1205031728	genic_downstream_transcript_variant,intron_variant
rs1205043948	genic_downstream_transcript_variant,intron_variant
rs1205063845	intron_variant
rs1205077210	genic_upstream_transcript_variant,intron_variant
rs1205095378	intron_variant
rs1205098866	genic_upstream_transcript_variant,intron_variant
rs1205151807	intron_variant
rs1205154151	intron_variant
rs1205180082	intron_variant
rs1205183327	genic_upstream_transcript_variant,intron_variant
rs1205186994	genic_downstream_transcript_variant,intron_variant
rs1205253882	genic_upstream_transcript_variant,intron_variant
rs1205286105	intron_variant
rs1205329178	genic_upstream_transcript_variant,intron_variant
rs1205370103	intron_variant
rs1205386628	genic_upstream_transcript_variant,intron_variant
rs1205394229	genic_upstream_transcript_variant,intron_variant
rs1205484398	genic_upstream_transcript_variant,intron_variant
rs1205490084	genic_downstream_transcript_variant,intron_variant
rs1205512929	genic_downstream_transcript_variant,intron_variant
rs1205514582	intron_variant
rs1205533270	intron_variant
rs1205587236	intron_variant
rs1205599999	intron_variant
rs1205604402	intron_variant
rs1205620651	genic_downstream_transcript_variant,intron_variant
rs1205625016	intron_variant

rs1205661199	genic_upstream_transcript_variant,intron_variant
rs1205685589	genic_downstream_transcript_variant,intron_variant
rs1205693447	intron_variant
rs1205720857	genic_upstream_transcript_variant,intron_variant
rs1205731011	genic_upstream_transcript_variant,intron_variant
rs1205768235	intron_variant
rs1205812208	genic_upstream_transcript_variant,intron_variant
rs1205822468	genic_upstream_transcript_variant,intron_variant
rs1205823537	intron_variant
rs1205880919	intron_variant
rs1205947635	intron_variant
rs1205951070	intron_variant
rs1206008008	intron_variant
rs1206012846	intron_variant
rs1206014127	intron_variant
rs1206015285	3_prime_UTR_variant,genic_downstream_transcript_variant,non_coding_transcript_variant
rs1206017189	genic_downstream_transcript_variant,intron_variant
rs1206025453	intron_variant
rs1206037023	intron_variant
rs1206070562	intron_variant
rs1206110829	genic_upstream_transcript_variant,intron_variant
rs1206175065	intron_variant
rs1206177202	intron_variant
rs1206219649	genic_upstream_transcript_variant,intron_variant
rs1206254695	genic_upstream_transcript_variant,intron_variant
rs1206276045	genic_upstream_transcript_variant,intron_variant
rs1206286841	genic_upstream_transcript_variant,intron_variant
rs1206305486	genic_upstream_transcript_variant,intron_variant
rs1206306034	genic_downstream_transcript_variant,intron_variant
rs1206324743	genic_upstream_transcript_variant,intron_variant
rs1206389388	genic_downstream_transcript_variant,intron_variant
rs1206444130	intron_variant
rs1206476409	intron_variant
rs1206531099	intron_variant
rs1206540495	genic_upstream_transcript_variant,intron_variant
rs1206542386	genic_upstream_transcript_variant,intron_variant
rs1206554517	genic_upstream_transcript_variant,intron_variant
rs1206592835	genic_upstream_transcript_variant,intron_variant,upstream_transcript_variant
rs1206633371	intron_variant
rs1206633447	intron_variant
rs1206650139	genic_downstream_transcript_variant,intron_variant
rs1206691932	intron_variant
rs1206822415	genic_upstream_transcript_variant,intron_variant
rs1206826015	genic_upstream_transcript_variant,intron_variant
rs1206834725	genic_upstream_transcript_variant,intron_variant,upstream_transcript_variant
rs1206871139	genic_upstream_transcript_variant,intron_variant
rs1206893882	genic_upstream_transcript_variant,intron_variant
rs1206930645	genic_downstream_transcript_variant,intron_variant
rs1206941415	genic_downstream_transcript_variant,intron_variant
rs1207008573	intron_variant,5_prime_UTR_variant
rs1207014634	genic_downstream_transcript_variant,intron_variant
rs1207145111	genic_downstream_transcript_variant,intron_variant
rs1207180536	genic_upstream_transcript_variant,intron_variant
rs1207197546	genic_upstream_transcript_variant,intron_variant
rs1207259682	genic_upstream_transcript_variant,intron_variant
rs1207261309	genic_upstream_transcript_variant,intron_variant
rs1207285150	genic_upstream_transcript_variant,intron_variant
rs1207327782	intron_variant
rs1207338822	intron_variant
rs1207346693	genic_downstream_transcript_variant,intron_variant
rs1207377119	genic_downstream_transcript_variant,intron_variant
rs1207446597	genic_upstream_transcript_variant,intron_variant
rs1207473531	genic_upstream_transcript_variant,intron_variant
rs1207523555	genic_upstream_transcript_variant,intron_variant
rs1207571615	genic_upstream_transcript_variant,intron_variant
rs1207585132	intron_variant
rs1207632648	genic_upstream_transcript_variant,intron_variant
rs1207633659	intron_variant
rs1207634832	genic_downstream_transcript_variant,intron_variant
rs1207672264	intron_variant
rs1207673037	genic_upstream_transcript_variant,intron_variant
rs1207698595	genic_upstream_transcript_variant,intron_variant
rs1207715563	missense_variant,non_coding_transcript_variant,coding_sequence_variant
rs1207721175	intron_variant

rs1207763636	genic_upstream_transcript_variant,intron_variant
rs1207766075	genic_upstream_transcript_variant,intron_variant
rs1207781386	intron_variant
rs1207857178	intron_variant
rs1207890929	genic_upstream_transcript_variant,intron_variant
rs1207918474	genic_upstream_transcript_variant,intron_variant
rs1207954497	intron_variant
rs1207984877	genic_upstream_transcript_variant,intron_variant
rs1208004319	intron_variant
rs1208009683	genic_upstream_transcript_variant,intron_variant
rs1208036798	intron_variant
rs1208043070	genic_upstream_transcript_variant,intron_variant
rs1208069537	genic_upstream_transcript_variant,intron_variant
rs1208081371	intron_variant
rs1208085344	genic_upstream_transcript_variant,intron_variant
rs1208096025	intron_variant,5_prime_UTR_variant
rs1208134252	genic_upstream_transcript_variant,intron_variant
rs1208168478	genic_upstream_transcript_variant,intron_variant
rs1208196359	intron_variant
rs1208201073	genic_downstream_transcript_variant,intron_variant
rs1208214985	intron_variant
rs1208260703	genic_upstream_transcript_variant,intron_variant
rs1208272553	genic_upstream_transcript_variant,intron_variant
rs1208284007	genic_upstream_transcript_variant,intron_variant,upstream_transcript_variant
rs1208332434	intron_variant
rs1208419577	intron_variant
rs1208436687	genic_upstream_transcript_variant,intron_variant
rs1208439651	intron_variant
rs1208449243	intron_variant
rs1208457980	genic_upstream_transcript_variant,intron_variant
rs1208537624	intron_variant
rs1208554913	genic_downstream_transcript_variant,synonymous_variant,non_coding_transcript_variant,coding_sequence_variant
rs1208561175	intron_variant
rs1208561495	genic_upstream_transcript_variant,intron_variant
rs1208605975	intron_variant
rs1208665247	intron_variant
rs1208695083	genic_downstream_transcript_variant,intron_variant
rs1208757432	genic_downstream_transcript_variant,intron_variant
rs1208758704	genic_downstream_transcript_variant,intron_variant
rs1208779807	intron_variant
rs1208808330	genic_upstream_transcript_variant,intron_variant
rs1208818911	genic_upstream_transcript_variant,intron_variant
rs1208830118	genic_upstream_transcript_variant,intron_variant
rs1208853386	genic_upstream_transcript_variant,intron_variant
rs1208861264	genic_downstream_transcript_variant,intron_variant
rs1208881531	non_coding_transcript_variant,coding_sequence_variant,missense_variant
rs1208889143	intron_variant
rs1208896661	intron_variant
rs1208908752	genic_downstream_transcript_variant,intron_variant
rs1208995664	genic_upstream_transcript_variant,upstream_transcript_variant,2KB_upstream_variant,intron_variant
rs1209003689	genic_downstream_transcript_variant,intron_variant
rs1209063164	genic_upstream_transcript_variant,intron_variant
rs1209108640	genic_upstream_transcript_variant,intron_variant
rs1209117351	genic_upstream_transcript_variant,intron_variant
rs1209123853	genic_upstream_transcript_variant,intron_variant
rs1209150356	intron_variant
rs1209151497	genic_upstream_transcript_variant,intron_variant
rs1209152972	genic_downstream_transcript_variant,intron_variant
rs1209154203	intron_variant
rs1209170944	genic_upstream_transcript_variant,intron_variant
rs1209189614	2KB_upstream_variant,upstream_transcript_variant,genic_upstream_transcript_variant,intron_variant
rs1209204260	genic_upstream_transcript_variant,intron_variant
rs1209208843	genic_downstream_transcript_variant,intron_variant
rs1209209305	genic_downstream_transcript_variant,intron_variant
rs1209245083	genic_downstream_transcript_variant,intron_variant
rs1209259949	intron_variant
rs1209266849	genic_upstream_transcript_variant,intron_variant
rs1209387556	upstream_transcript_variant,genic_upstream_transcript_variant,intron_variant
rs1209412899	intron_variant
rs1209429198	genic_upstream_transcript_variant,intron_variant
rs1209477355	intron_variant
rs1209502038	downstream_transcript_variant,500B_downstream_variant
rs1209527272	genic_upstream_transcript_variant,intron_variant
rs1209537403	genic_upstream_transcript_variant,intron_variant

rs1209586598	genic_downstream_transcript_variant,non_coding_transcript_variant,3_prime_UTR_variant
rs1209593152	genic_downstream_transcript_variant,intron_variant
rs1209676950	intron_variant
rs1209690432	intron_variant
rs1209717213	intron_variant
rs1209725856	genic_upstream_transcript_variant,intron_variant
rs1209780574	genic_upstream_transcript_variant,intron_variant
rs1209786998	intron_variant
rs1209816075	genic_upstream_transcript_variant,intron_variant
rs1209856153	genic_upstream_transcript_variant,intron_variant
rs1209871985	genic_upstream_transcript_variant,intron_variant
rs1209876782	intron_variant
rs1209894072	upstream_transcript_variant,genic_upstream_transcript_variant,intron_variant
rs1209924245	genic_upstream_transcript_variant,intron_variant
rs1209937129	genic_downstream_transcript_variant,non_coding_transcript_variant,3_prime_UTR_variant
rs1209948347	genic_upstream_transcript_variant,intron_variant
rs1209958299	intron_variant
rs1209964980	intron_variant
rs1210002767	genic_upstream_transcript_variant,intron_variant
rs1210025765	genic_upstream_transcript_variant,intron_variant
rs1210058129	genic_downstream_transcript_variant,intron_variant
rs1210080595	genic_upstream_transcript_variant,upstream_transcript_variant,2KB_upstream_variant,intron_variant
rs1210082700	genic_upstream_transcript_variant,intron_variant
rs1210094777	genic_downstream_transcript_variant,intron_variant
rs1210116420	genic_upstream_transcript_variant,intron_variant
rs1210161335	upstream_transcript_variant,genic_upstream_transcript_variant,intron_variant
rs1210179390	intron_variant
rs1210193238	intron_variant
rs1210196447	genic_upstream_transcript_variant,intron_variant
rs1210329889	genic_upstream_transcript_variant,intron_variant
rs1210331350	genic_upstream_transcript_variant,intron_variant
rs1210332315	upstream_transcript_variant,genic_upstream_transcript_variant,5_prime_UTR_variant,intron_variant
rs1210334373	genic_upstream_transcript_variant,upstream_transcript_variant,2KB_upstream_variant,intron_variant
rs1210366998	genic_upstream_transcript_variant,intron_variant
rs1210431619	genic_downstream_transcript_variant,intron_variant
rs1210442457	genic_upstream_transcript_variant,intron_variant
rs1210453691	intron_variant
rs1210485570	genic_upstream_transcript_variant,intron_variant
rs1210525291	intron_variant
rs1210532772	intron_variant
rs1210548139	intron_variant
rs1210548788	genic_upstream_transcript_variant,intron_variant
rs1210591533	intron_variant
rs1210602767	intron_variant
rs1210632005	intron_variant
rs1210643427	genic_upstream_transcript_variant,intron_variant
rs1210678192	genic_upstream_transcript_variant,intron_variant
rs1210681259	intron_variant
rs1210682948	genic_upstream_transcript_variant,intron_variant
rs1210741718	genic_downstream_transcript_variant,intron_variant
rs1210765179	genic_upstream_transcript_variant,intron_variant
rs1210778783	genic_upstream_transcript_variant,intron_variant
rs1210826383	genic_upstream_transcript_variant,intron_variant
rs1210829440	genic_downstream_transcript_variant,intron_variant
rs1210834839	genic_upstream_transcript_variant,intron_variant
rs1210891084	intron_variant
rs1210932614	genic_upstream_transcript_variant,intron_variant
rs1210969120	upstream_transcript_variant,genic_upstream_transcript_variant,intron_variant
rs1210977485	genic_downstream_transcript_variant,non_coding_transcript_variant,3_prime_UTR_variant
rs1210982553	intron_variant
rs1210999585	5_prime_UTR_variant,intron_variant
rs1211000491	genic_upstream_transcript_variant,upstream_transcript_variant,2KB_upstream_variant,intron_variant
rs1211023131	genic_upstream_transcript_variant,intron_variant
rs1211031341	genic_downstream_transcript_variant,intron_variant
rs1211040959	genic_upstream_transcript_variant,intron_variant
rs1211050455	intron_variant
rs1211077639	genic_downstream_transcript_variant,intron_variant
rs1211097680	intron_variant
rs1211118607	intron_variant
rs1211174938	genic_upstream_transcript_variant,intron_variant
rs1211190351	genic_upstream_transcript_variant,intron_variant
rs1211247302	genic_upstream_transcript_variant,intron_variant
rs1211286578	intron_variant
rs1211316338	genic_upstream_transcript_variant,intron_variant

rs1211335106	genic_downstream_transcript_variant,intron_variant
rs1211366871	genic_upstream_transcript_variant,intron_variant
rs1211377109	genic_upstream_transcript_variant,intron_variant
rs1211391891	intron_variant
rs1211401785	2KB_upstream_variant,upstream_transcript_variant,genic_upstream_transcript_variant,intron_variant
rs1211426360	intron_variant
rs1211429498	intron_variant
rs1211466502	upstream_transcript_variant,genic_upstream_transcript_variant,intron_variant
rs1211517175	genic_upstream_transcript_variant,intron_variant
rs1211521585	intron_variant
rs1211582818	genic_upstream_transcript_variant,intron_variant
rs1211634426	genic_upstream_transcript_variant,intron_variant
rs1211639393	genic_downstream_transcript_variant,non_coding_transcript_variant,3_prime_UTR_variant
rs1211657383	genic_upstream_transcript_variant,intron_variant
rs1211731923	genic_upstream_transcript_variant,intron_variant
rs1211740492	genic_downstream_transcript_variant,intron_variant
rs1211753124	genic_downstream_transcript_variant,intron_variant
rs1211759210	intron_variant
rs1211761117	genic_upstream_transcript_variant,intron_variant
rs1211769235	genic_upstream_transcript_variant,intron_variant
rs1211776065	2KB_upstream_variant,upstream_transcript_variant,genic_upstream_transcript_variant,intron_variant
rs1211843066	genic_upstream_transcript_variant,intron_variant
rs1211857489	genic_upstream_transcript_variant,intron_variant
rs1211880808	intron_variant
rs1211894369	genic_upstream_transcript_variant,intron_variant
rs1211916104	genic_downstream_transcript_variant,intron_variant
rs1211919519	2KB_upstream_variant,upstream_transcript_variant,genic_upstream_transcript_variant,intron_variant
rs1211938992	intron_variant
rs1211978382	genic_upstream_transcript_variant,intron_variant
rs1212007539	genic_upstream_transcript_variant,intron_variant
rs1212017798	intron_variant
rs1212030329	intron_variant
rs1212067371	genic_upstream_transcript_variant,intron_variant
rs1212088892	genic_upstream_transcript_variant,intron_variant
rs1212131316	genic_upstream_transcript_variant,intron_variant
rs1212160399	genic_upstream_transcript_variant,intron_variant
rs1212161024	intron_variant
rs1212170699	intron_variant
rs1212264775	genic_upstream_transcript_variant,intron_variant
rs1212278437	intron_variant
rs1212300955	intron_variant
rs1212331896	genic_upstream_transcript_variant,intron_variant
rs1212332214	genic_upstream_transcript_variant,intron_variant
rs1212353759	genic_upstream_transcript_variant,intron_variant
rs1212367251	intron_variant
rs1212404012	genic_upstream_transcript_variant,intron_variant
rs1212432376	genic_downstream_transcript_variant,intron_variant
rs1212444700	genic_downstream_transcript_variant,intron_variant
rs1212524160	genic_upstream_transcript_variant,intron_variant
rs1212623836	genic_upstream_transcript_variant,intron_variant
rs1212626518	genic_upstream_transcript_variant,intron_variant
rs1212678632	genic_downstream_transcript_variant,intron_variant
rs1212712705	genic_upstream_transcript_variant,intron_variant
rs1212713407	genic_upstream_transcript_variant,intron_variant
rs1212737241	genic_downstream_transcript_variant,intron_variant
rs1212758299	genic_upstream_transcript_variant,intron_variant
rs1212794811	intron_variant
rs1212829355	genic_upstream_transcript_variant,intron_variant
rs1212858866	genic_downstream_transcript_variant,non_coding_transcript_variant,3_prime_UTR_variant
rs1212916439	genic_downstream_transcript_variant,intron_variant
rs1212950991	genic_downstream_transcript_variant,non_coding_transcript_variant,3_prime_UTR_variant
rs1212952877	genic_upstream_transcript_variant,intron_variant
rs1212972544	intron_variant
rs1212996109	genic_upstream_transcript_variant,intron_variant
rs1213012774	genic_upstream_transcript_variant,intron_variant
rs1213126000	genic_upstream_transcript_variant,intron_variant
rs1213136041	intron_variant
rs1213147990	genic_upstream_transcript_variant,intron_variant
rs1213148790	intron_variant
rs1213161026	genic_upstream_transcript_variant,intron_variant
rs1213225485	genic_upstream_transcript_variant,intron_variant
rs1213241942	genic_upstream_transcript_variant,intron_variant
rs1213285514	genic_upstream_transcript_variant,intron_variant
rs1213295164	genic_downstream_transcript_variant,intron_variant

rs1213306008	genic_upstream_transcript_variant,intron_variant
rs1213348202	intron_variant
rs1213351920	genic_upstream_transcript_variant,intron_variant
rs1213371501	intron_variant
rs1213379562	intron_variant
rs1213384633	genic_upstream_transcript_variant,intron_variant
rs1213439168	intron_variant
rs1213538795	intron_variant
rs1213545724	intron_variant
rs1213576258	genic_upstream_transcript_variant,intron_variant
rs1213593403	intron_variant
rs1213595530	intron_variant
rs1213598358	intron_variant
rs1213652522	genic_upstream_transcript_variant,intron_variant
rs1213754829	genic_upstream_transcript_variant,intron_variant
rs1213756333	intron_variant
rs1213780293	genic_upstream_transcript_variant,intron_variant
rs1213812053	genic_upstream_transcript_variant,intron_variant
rs1213835400	intron_variant
rs1213842740	intron_variant
rs1213870492	intron_variant
rs1213887634	genic_downstream_transcript_variant,intron_variant
rs1213892973	intron_variant
rs1213894549	genic_downstream_transcript_variant,intron_variant
rs1213907373	genic_downstream_transcript_variant,intron_variant
rs1213983523	intron_variant
rs1214032894	genic_downstream_transcript_variant,intron_variant
rs1214044515	genic_downstream_transcript_variant,intron_variant
rs1214058558	genic_upstream_transcript_variant,intron_variant
rs1214061891	genic_upstream_transcript_variant,intron_variant
rs1214069939	genic_downstream_transcript_variant,intron_variant
rs1214116690	intron_variant
rs1214144183	upstream_transcript_variant,genic_upstream_transcript_variant,intron_variant
rs1214183349	genic_downstream_transcript_variant,intron_variant
rs1214217581	genic_upstream_transcript_variant,intron_variant
rs1214282117	genic_upstream_transcript_variant,intron_variant
rs1214291712	genic_upstream_transcript_variant,intron_variant
rs1214323810	genic_upstream_transcript_variant,splice_acceptor_variant,non_coding_transcript_variant,5_prime_UTR_variant,intron_variant
rs1214336919	intron_variant
rs1214345369	genic_downstream_transcript_variant,intron_variant
rs1214382967	genic_downstream_transcript_variant,intron_variant
rs1214435284	genic_upstream_transcript_variant,intron_variant
rs1214437689	genic_downstream_transcript_variant,intron_variant
rs1214458686	downstream_transcript_variant,500B_downstream_variant
rs1214466956	intron_variant
rs1214480544	genic_upstream_transcript_variant,intron_variant
rs1214489838	genic_downstream_transcript_variant,intron_variant
rs1214495034	genic_upstream_transcript_variant,intron_variant
rs1214533769	genic_downstream_transcript_variant,intron_variant
rs1214595204	intron_variant
rs1214615074	genic_upstream_transcript_variant,intron_variant
rs1214629015	genic_downstream_transcript_variant,coding_sequence_variant,non_coding_transcript_variant,synonymous_variant
rs1214651018	genic_downstream_transcript_variant,coding_sequence_variant,non_coding_transcript_variant,synonymous_variant
rs1214661333	intron_variant
rs1214672674	intron_variant
rs1214697791	genic_upstream_transcript_variant,intron_variant
rs1214706525	intron_variant
rs1214763053	intron_variant
rs1214771086	genic_upstream_transcript_variant,intron_variant
rs1214864480	genic_downstream_transcript_variant,intron_variant
rs1214869117	intron_variant
rs1214874796	intron_variant
rs1214919132	genic_upstream_transcript_variant,intron_variant
rs1214940547	genic_downstream_transcript_variant,intron_variant
rs1215002304	genic_upstream_transcript_variant,upstream_transcript_variant,2KB_upstream_variant,intron_variant
rs1215069290	genic_upstream_transcript_variant,intron_variant
rs1215092339	upstream_transcript_variant,genic_upstream_transcript_variant,intron_variant
rs1215093301	intron_variant
rs1215159649	intron_variant
rs1215172960	intron_variant
rs1215212193	genic_upstream_transcript_variant,intron_variant
rs1215276759	genic_upstream_transcript_variant,intron_variant
rs1215352770	upstream_transcript_variant,genic_upstream_transcript_variant,intron_variant
rs1215391711	genic_upstream_transcript_variant,intron_variant

rs1215410089	intron_variant
rs1215438121	intron_variant
rs1215479478	intron_variant
rs1215504173	genic_downstream_transcript_variant,intron_variant
rs1215518337	intron_variant
rs1215532441	genic_upstream_transcript_variant,intron_variant
rs1215560654	intron_variant
rs1215578357	genic_upstream_transcript_variant,intron_variant
rs1215580408	genic_downstream_transcript_variant,intron_variant
rs1215589880	upstream_transcript_variant,genic_upstream_transcript_variant,5_prime_UTR_variant,intron_variant
rs1215593992	genic_downstream_transcript_variant,intron_variant
rs1215597155	genic_downstream_transcript_variant,intron_variant
rs1215600480	intron_variant
rs1215621530	intron_variant
rs1215643280	genic_upstream_transcript_variant,intron_variant
rs1215649030	intron_variant
rs1215715680	genic_downstream_transcript_variant,intron_variant
rs1215721337	intron_variant
rs1215725527	genic_downstream_transcript_variant,intron_variant
rs1215793236	genic_upstream_transcript_variant,intron_variant
rs1215798406	intron_variant
rs1215819797	intron_variant
rs1215821599	intron_variant
rs1215821934	genic_upstream_transcript_variant,intron_variant
rs1215887064	intron_variant
rs1215904394	intron_variant
rs1215910345	intron_variant
rs1215916275	genic_upstream_transcript_variant,intron_variant
rs1215968260	genic_upstream_transcript_variant,intron_variant
rs1215968927	genic_upstream_transcript_variant,intron_variant
rs1216020976	genic_upstream_transcript_variant,intron_variant
rs1216022895	intron_variant
rs1216027886	genic_upstream_transcript_variant,intron_variant
rs1216035158	genic_upstream_transcript_variant,intron_variant
rs1216061305	genic_downstream_transcript_variant,intron_variant
rs1216074038	genic_upstream_transcript_variant,intron_variant
rs1216129920	genic_upstream_transcript_variant,intron_variant
rs1216159165	2KB_upstream_variant,upstream_transcript_variant,genic_upstream_transcript_variant,intron_variant
rs1216209700	genic_upstream_transcript_variant,intron_variant
rs1216210728	genic_downstream_transcript_variant,intron_variant
rs1216214375	intron_variant
rs1216230885	genic_upstream_transcript_variant,intron_variant
rs1216243229	genic_upstream_transcript_variant,intron_variant
rs1216304845	intron_variant
rs1216307191	upstream_transcript_variant,genic_upstream_transcript_variant,intron_variant
rs1216364792	intron_variant
rs1216365724	genic_upstream_transcript_variant,intron_variant
rs1216405041	genic_upstream_transcript_variant,intron_variant
rs1216442573	genic_upstream_transcript_variant,intron_variant
rs1216515288	2KB_upstream_variant,upstream_transcript_variant,genic_upstream_transcript_variant,intron_variant
rs1216606981	intron_variant
rs1216619781	genic_downstream_transcript_variant,non_coding_transcript_variant,3_prime_UTR_variant
rs1216624347	intron_variant
rs1216666466	genic_upstream_transcript_variant,upstream_transcript_variant,2KB_upstream_variant,intron_variant
rs1216682962	genic_upstream_transcript_variant,intron_variant
rs1216685767	genic_upstream_transcript_variant,intron_variant
rs1216706835	intron_variant
rs1216717757	intron_variant
rs1216730134	intron_variant
rs1216733215	intron_variant
rs1216794136	genic_upstream_transcript_variant,intron_variant
rs1216816717	intron_variant
rs1216839882	intron_variant
rs1216854737	genic_upstream_transcript_variant,intron_variant
rs1216868539	genic_downstream_transcript_variant,intron_variant
rs1216885275	intron_variant
rs1216911284	genic_upstream_transcript_variant,intron_variant
rs1216931638	genic_downstream_transcript_variant,synonymous_variant,non_coding_transcript_variant,missense_variant,coding_sequence_variant
rs1216964684	intron_variant
rs1216968896	intron_variant
rs1216984631	intron_variant
rs1217017258	genic_upstream_transcript_variant,intron_variant
rs1217053924	genic_downstream_transcript_variant,intron_variant
rs1217077605	genic_upstream_transcript_variant,intron_variant

rs1217087416	genic_downstream_transcript_variant,intron_variant
rs1217118002	intron_variant
rs1217154545	intron_variant
rs1217158089	genic_upstream_transcript_variant,intron_variant
rs1217179005	genic_upstream_transcript_variant,intron_variant
rs1217213931	genic_upstream_transcript_variant,intron_variant
rs1217229328	genic_downstream_transcript_variant,intron_variant
rs1217234677	intron_variant
rs1217240021	genic_upstream_transcript_variant,intron_variant
rs1217247389	upstream_transcript_variant,genic_upstream_transcript_variant,intron_variant
rs1217264494	genic_upstream_transcript_variant,intron_variant
rs1217292407	genic_downstream_transcript_variant,intron_variant
rs1217296763	genic_upstream_transcript_variant,intron_variant
rs1217378290	intron_variant
rs1217459159	genic_upstream_transcript_variant,intron_variant
rs1217459359	genic_downstream_transcript_variant,intron_variant
rs1217477142	genic_upstream_transcript_variant,upstream_transcript_variant,2KB_upstream_variant,intron_variant
rs1217498767	genic_upstream_transcript_variant,intron_variant
rs1217564612	genic_upstream_transcript_variant,intron_variant
rs1217565423	genic_upstream_transcript_variant,intron_variant
rs1217634591	intron_variant
rs1217645734	intron_variant
rs1217657808	genic_upstream_transcript_variant,intron_variant
rs1217665419	intron_variant
rs1217682541	genic_upstream_transcript_variant,intron_variant
rs1217690442	genic_upstream_transcript_variant,intron_variant
rs1217756818	genic_upstream_transcript_variant,intron_variant
rs1217816070	genic_upstream_transcript_variant,intron_variant
rs1217876205	intron_variant
rs1217887756	intron_variant
rs1217893157	genic_upstream_transcript_variant,intron_variant
rs1217942331	intron_variant
rs1217950288	intron_variant
rs1217968266	genic_downstream_transcript_variant,intron_variant
rs1217974140	intron_variant
rs1217985027	genic_downstream_transcript_variant,intron_variant
rs1217990676	genic_upstream_transcript_variant,intron_variant
rs1217998648	intron_variant
rs1218003906	non_coding_transcript_variant,coding_sequence_variant,missense_variant
rs1218041925	intron_variant
rs1218043975	5_prime_UTR_variant,intron_variant
rs1218086070	upstream_transcript_variant,genic_upstream_transcript_variant,intron_variant
rs1218090193	intron_variant
rs1218100612	intron_variant
rs1218108482	upstream_transcript_variant,genic_upstream_transcript_variant,intron_variant
rs1218118118	intron_variant
rs1218133562	genic_upstream_transcript_variant,upstream_transcript_variant,2KB_upstream_variant,intron_variant
rs1218137304	upstream_transcript_variant,genic_upstream_transcript_variant,intron_variant
rs1218140083	genic_upstream_transcript_variant,intron_variant
rs1218232880	genic_upstream_transcript_variant,intron_variant
rs1218241359	genic_upstream_transcript_variant,intron_variant
rs1218251768	genic_upstream_transcript_variant,intron_variant
rs1218264520	genic_upstream_transcript_variant,intron_variant
rs1218278185	genic_upstream_transcript_variant,intron_variant
rs1218348180	intron_variant
rs1218359091	genic_upstream_transcript_variant,intron_variant
rs1218373146	genic_downstream_transcript_variant,intron_variant
rs1218374112	genic_upstream_transcript_variant,upstream_transcript_variant,2KB_upstream_variant,intron_variant
rs1218376235	intron_variant
rs1218396634	intron_variant
rs1218430563	intron_variant
rs1218461653	genic_upstream_transcript_variant,intron_variant
rs1218466398	genic_upstream_transcript_variant,intron_variant
rs1218474741	genic_upstream_transcript_variant,intron_variant
rs1218478527	intron_variant
rs1218514479	intron_variant
rs1218517674	genic_upstream_transcript_variant,intron_variant
rs1218521600	genic_upstream_transcript_variant,intron_variant
rs1218528592	genic_upstream_transcript_variant,intron_variant
rs1218537665	intron_variant
rs1218538929	2KB_upstream_variant,upstream_transcript_variant,genic_upstream_transcript_variant,intron_variant
rs1218560740	genic_upstream_transcript_variant,intron_variant
rs1218563207	2KB_upstream_variant,upstream_transcript_variant,genic_upstream_transcript_variant,intron_variant
rs1218565132	intron_variant

rs1218582530	genic_upstream_transcript_variant,intron_variant
rs1218585805	intron_variant
rs1218618057	genic_upstream_transcript_variant,intron_variant
rs1218625511	intron_variant
rs1218669045	intron_variant
rs1218673336	genic_downstream_transcript_variant,intron_variant
rs1218701804	genic_upstream_transcript_variant,intron_variant
rs1218765177	intron_variant
rs1218786145	genic_upstream_transcript_variant,intron_variant
rs1218801177	intron_variant
rs1218802139	genic_downstream_transcript_variant,non_coding_transcript_variant,3_prime_UTR_variant
rs1218816835	intron_variant
rs1218840974	intron_variant
rs1218847989	genic_downstream_transcript_variant,intron_variant
rs1218876536	non_coding_transcript_variant,genic_upstream_transcript_variant,5_prime_UTR_variant,intron_variant
rs1218930306	genic_upstream_transcript_variant,intron_variant
rs1218936621	genic_downstream_transcript_variant,non_coding_transcript_variant,3_prime_UTR_variant
rs1218942279	genic_upstream_transcript_variant,intron_variant
rs1218949047	genic_downstream_transcript_variant,intron_variant
rs1218953889	genic_downstream_transcript_variant,intron_variant
rs1218954381	intron_variant
rs1218964250	genic_upstream_transcript_variant,intron_variant
rs1218977476	genic_upstream_transcript_variant,intron_variant
rs1219042592	genic_upstream_transcript_variant,intron_variant
rs1219061648	upstream_transcript_variant,genic_upstream_transcript_variant,intron_variant
rs1219072645	intron_variant
rs1219079130	intron_variant
rs1219127604	intron_variant
rs1219128253	genic_upstream_transcript_variant,intron_variant
rs1219154876	genic_downstream_transcript_variant,intron_variant
rs1219156328	genic_upstream_transcript_variant,intron_variant
rs1219182965	upstream_transcript_variant,genic_upstream_transcript_variant,intron_variant
rs1219188801	genic_upstream_transcript_variant,intron_variant
rs1219221108	genic_downstream_transcript_variant,intron_variant
rs1219223787	genic_upstream_transcript_variant,intron_variant
rs1219227607	intron_variant
rs1219259039	genic_upstream_transcript_variant,intron_variant
rs1219264141	genic_downstream_transcript_variant,intron_variant
rs1219291944	genic_upstream_transcript_variant,intron_variant
rs1219300147	genic_upstream_transcript_variant,intron_variant
rs1219303467	genic_upstream_transcript_variant,intron_variant
rs1219318008	genic_upstream_transcript_variant,upstream_transcript_variant,2KB_upstream_variant,intron_variant
rs1219322267	upstream_transcript_variant,genic_upstream_transcript_variant,intron_variant
rs1219352205	genic_downstream_transcript_variant,intron_variant
rs1219383236	intron_variant
rs1219418868	genic_downstream_transcript_variant,intron_variant
rs1219438538	upstream_transcript_variant,genic_upstream_transcript_variant,intron_variant
rs1219463067	genic_downstream_transcript_variant,intron_variant
rs1219464462	genic_upstream_transcript_variant,intron_variant
rs1219473635	intron_variant
rs1219492091	genic_upstream_transcript_variant,intron_variant
rs1219517050	genic_upstream_transcript_variant,intron_variant
rs1219561715	downstream_transcript_variant,500B_downstream_variant
rs1219578408	intron_variant
rs1219579840	intron_variant
rs1219592208	genic_downstream_transcript_variant,non_coding_transcript_variant,3_prime_UTR_variant
rs1219600638	intron_variant
rs1219656045	intron_variant
rs1219692925	intron_variant
rs1219707301	intron_variant
rs1219732643	intron_variant
rs1219780465	intron_variant
rs1219789485	upstream_transcript_variant,genic_upstream_transcript_variant,intron_variant
rs1219794645	intron_variant
rs1219796168	intron_variant
rs1219801720	genic_upstream_transcript_variant,intron_variant
rs1219840736	intron_variant
rs1219843125	intron_variant
rs1219864210	intron_variant
rs1219879836	upstream_transcript_variant,genic_upstream_transcript_variant,intron_variant
rs1219924034	genic_upstream_transcript_variant,intron_variant
rs1219934595	intron_variant
rs1219970078	intron_variant
rs1219975946	genic_upstream_transcript_variant,intron_variant

rs1219979845	non_coding_transcript_variant,coding_sequence_variant,missense_variant
rs1220008281	genic_upstream_transcript_variant,intron_variant
rs1220008503	genic_downstream_transcript_variant,intron_variant
rs1220053493	genic_upstream_transcript_variant,upstream_transcript_variant,2KB_upstream_variant,intron_variant
rs1220132786	genic_upstream_transcript_variant,intron_variant
rs1220133688	2KB_upstream_variant,upstream_transcript_variant,genic_upstream_transcript_variant,intron_variant
rs1220163497	genic_upstream_transcript_variant,intron_variant
rs1220243533	intron_variant
rs1220252989	genic_downstream_transcript_variant,intron_variant
rs1220274849	genic_downstream_transcript_variant,downstream_transcript_variant,intron_variant
rs1220275126	genic_upstream_transcript_variant,intron_variant
rs1220332878	genic_upstream_transcript_variant,intron_variant
rs1220337646	genic_upstream_transcript_variant,intron_variant
rs1220401962	genic_upstream_transcript_variant,intron_variant
rs1220413698	genic_downstream_transcript_variant,intron_variant
rs1220441014	intron_variant
rs1220497421	intron_variant
rs1220510213	intron_variant
rs1220625114	intron_variant
rs1220636610	genic_upstream_transcript_variant,intron_variant
rs1220696247	genic_upstream_transcript_variant,intron_variant
rs1220707166	genic_upstream_transcript_variant,intron_variant
rs1220736985	genic_upstream_transcript_variant,intron_variant
rs1220741034	genic_downstream_transcript_variant,intron_variant
rs1220749635	intron_variant
rs1220756470	intron_variant
rs1220790097	intron_variant
rs1220855951	genic_upstream_transcript_variant,intron_variant
rs1220859289	genic_downstream_transcript_variant,intron_variant
rs1220881862	genic_upstream_transcript_variant,intron_variant
rs1220990290	genic_downstream_transcript_variant,non_coding_transcript_variant,3_prime_UTR_variant
rs1220998769	intron_variant
rs1221055168	genic_upstream_transcript_variant,intron_variant
rs1221137453	genic_upstream_transcript_variant,intron_variant
rs1221139844	intron_variant
rs1221158616	genic_upstream_transcript_variant,intron_variant
rs1221167966	genic_upstream_transcript_variant,intron_variant
rs1221168542	genic_upstream_transcript_variant,intron_variant
rs1221322641	intron_variant
rs1221330236	genic_upstream_transcript_variant,intron_variant
rs1221358445	genic_upstream_transcript_variant,intron_variant
rs1221362186	genic_upstream_transcript_variant,upstream_transcript_variant,intron_variant
rs1221407039	genic_upstream_transcript_variant,intron_variant
rs1221464156	genic_downstream_transcript_variant,intron_variant
rs1221476287	genic_upstream_transcript_variant,intron_variant
rs1221498435	genic_upstream_transcript_variant,intron_variant
rs1221502920	intron_variant
rs1221519846	genic_upstream_transcript_variant,intron_variant
rs1221576031	intron_variant
rs1221599495	2KB_upstream_variant,genic_upstream_transcript_variant,upstream_transcript_variant,intron_variant
rs1221637466	missense_variant,intron_variant,genic_upstream_transcript_variant,5_prime_UTR_variant,coding_sequence_variant,non_coding_transcript_variant
rs1221654406	genic_upstream_transcript_variant,intron_variant
rs1221660653	intron_variant
rs1221671630	genic_upstream_transcript_variant,intron_variant
rs1221674339	genic_upstream_transcript_variant,intron_variant
rs1221702844	genic_upstream_transcript_variant,intron_variant
rs1221721610	intron_variant
rs1221735312	intron_variant
rs1221759893	genic_upstream_transcript_variant,upstream_transcript_variant,intron_variant
rs1221760044	genic_upstream_transcript_variant,intron_variant
rs1221763212	genic_downstream_transcript_variant,synonymous_variant,coding_sequence_variant,non_coding_transcript_variant
rs1221781601	intron_variant
rs1221787363	genic_upstream_transcript_variant,upstream_transcript_variant,intron_variant
rs1221819207	genic_upstream_transcript_variant,intron_variant
rs1221832301	intron_variant
rs1221865571	intron_variant
rs1221883668	genic_upstream_transcript_variant,intron_variant
rs1221926046	genic_downstream_transcript_variant,3_prime_UTR_variant,non_coding_transcript_variant
rs1221927980	intron_variant
rs1221939796	genic_upstream_transcript_variant,intron_variant
rs1221954630	genic_upstream_transcript_variant,intron_variant
rs1221995647	intron_variant
rs1222044946	genic_upstream_transcript_variant,intron_variant
rs1222096100	genic_upstream_transcript_variant,intron_variant

rs1222119998	genic_upstream_transcript_variant,intron_variant
rs1222144296	intron_variant
rs1222168005	genic_downstream_transcript_variant,intron_variant
rs1222201717	2KB_upstream_variant,upstream_transcript_variant,intron_variant,genic_upstream_transcript_variant
rs1222216538	genic_downstream_transcript_variant,intron_variant
rs1222227505	genic_upstream_transcript_variant,intron_variant
rs1222286641	genic_upstream_transcript_variant,intron_variant
rs1222326369	intron_variant
rs1222334053	intron_variant
rs1222395521	genic_upstream_transcript_variant,intron_variant
rs1222405001	genic_downstream_transcript_variant,intron_variant
rs1222417976	genic_upstream_transcript_variant,intron_variant
rs1222504860	genic_downstream_transcript_variant,intron_variant
rs1222524518	intron_variant
rs1222543450	genic_upstream_transcript_variant,intron_variant
rs1222565653	genic_downstream_transcript_variant,intron_variant
rs1222579313	genic_upstream_transcript_variant,upstream_transcript_variant,intron_variant
rs1222627308	genic_upstream_transcript_variant,intron_variant
rs1222647993	genic_upstream_transcript_variant,upstream_transcript_variant,intron_variant
rs1222648684	genic_downstream_transcript_variant,3_prime_UTR_variant,non_coding_transcript_variant
rs1222649039	genic_upstream_transcript_variant,intron_variant
rs1222669382	2KB_upstream_variant,genic_upstream_transcript_variant,upstream_transcript_variant,intron_variant
rs1222717069	intron_variant
rs1222727434	genic_downstream_transcript_variant,intron_variant
rs1222742220	2KB_upstream_variant,genic_upstream_transcript_variant,upstream_transcript_variant,intron_variant
rs1222754697	intron_variant
rs1222758859	genic_downstream_transcript_variant,3_prime_UTR_variant,non_coding_transcript_variant
rs1222777675	intron_variant
rs1222786222	intron_variant
rs1222794139	genic_upstream_transcript_variant,intron_variant
rs1222843315	genic_upstream_transcript_variant,upstream_transcript_variant,intron_variant
rs1222870130	genic_upstream_transcript_variant,intron_variant
rs1222878777	genic_downstream_transcript_variant,intron_variant
rs1222905741	genic_upstream_transcript_variant,intron_variant
rs1222915717	intron_variant
rs1222927447	intron_variant
rs1222938520	genic_upstream_transcript_variant,intron_variant
rs1222947035	genic_upstream_transcript_variant,intron_variant
rs1222986496	intron_variant
rs1223092751	intron_variant
rs1223132365	genic_upstream_transcript_variant,intron_variant
rs1223134241	genic_downstream_transcript_variant,intron_variant
rs1223137322	intron_variant
rs1223174669	genic_downstream_transcript_variant,intron_variant
rs1223201251	intron_variant
rs1223210994	intron_variant
rs1223222010	intron_variant
rs1223234832	genic_upstream_transcript_variant,intron_variant
rs1223250156	genic_downstream_transcript_variant,intron_variant
rs1223301394	intron_variant
rs1223318804	intron_variant
rs1223350367	genic_upstream_transcript_variant,intron_variant
rs1223369694	genic_downstream_transcript_variant,intron_variant
rs1223414655	genic_downstream_transcript_variant,intron_variant
rs1223460200	downstream_transcript_variant,500B_downstream_variant
rs1223495569	genic_upstream_transcript_variant,intron_variant
rs1223558287	genic_upstream_transcript_variant,intron_variant
rs1223567432	intron_variant
rs1223568666	intron_variant
rs1223591160	genic_upstream_transcript_variant,intron_variant
rs1223603530	intron_variant
rs1223610189	genic_downstream_transcript_variant,intron_variant
rs1223636171	genic_upstream_transcript_variant,intron_variant
rs1223689433	genic_downstream_transcript_variant,intron_variant
rs1223749319	genic_upstream_transcript_variant,intron_variant
rs1223802785	genic_upstream_transcript_variant,intron_variant
rs1223810503	genic_downstream_transcript_variant,intron_variant
rs1223845891	genic_upstream_transcript_variant,intron_variant
rs1223905368	2KB_upstream_variant,upstream_transcript_variant,intron_variant,genic_upstream_transcript_variant
rs1223929172	intron_variant
rs1223953978	genic_upstream_transcript_variant,intron_variant
rs1224003582	genic_downstream_transcript_variant,intron_variant
rs1224011647	genic_upstream_transcript_variant,intron_variant
rs1224033408	genic_downstream_transcript_variant,intron_variant

rs1224043223	genic_upstream_transcript_variant,intron_variant
rs1224084714	genic_downstream_transcript_variant,intron_variant
rs1224099937	intron_variant
rs1224106871	intron_variant
rs1224107635	genic_downstream_transcript_variant,intron_variant
rs1224155624	intron_variant
rs1224163032	intron_variant
rs1224167187	genic_downstream_transcript_variant,intron_variant
rs1224186647	2KB_upstream_variant,genic_upstream_transcript_variant,upstream_transcript_variant,intron_variant
rs1224204381	genic_upstream_transcript_variant,intron_variant
rs1224255852	intron_variant
rs1224288989	intron_variant
rs1224331975	genic_downstream_transcript_variant,intron_variant
rs1224332252	5_prime_UTR_variant,upstream_transcript_variant,intron_variant,genic_upstream_transcript_variant
rs1224376930	intron_variant
rs1224382769	intron_variant
rs1224464741	genic_upstream_transcript_variant,intron_variant
rs1224476144	intron_variant
rs1224497987	genic_upstream_transcript_variant,intron_variant
rs1224511467	intron_variant
rs1224545132	intron_variant
rs1224597864	intron_variant
rs1224642756	genic_downstream_transcript_variant,intron_variant
rs1224662715	genic_upstream_transcript_variant,intron_variant
rs1224675146	intron_variant
rs1224680436	missense_variant,intron_variant,synonymous_variant,genic_upstream_transcript_variant,coding_sequence_variant,non_coding_transcript_variant
rs1224689436	intron_variant
rs1224698236	genic_upstream_transcript_variant,intron_variant
rs1224701388	intron_variant
rs1224705929	intron_variant
rs1224720832	intron_variant
rs1224767478	genic_upstream_transcript_variant,intron_variant
rs1224833848	genic_upstream_transcript_variant,intron_variant
rs1224883191	genic_upstream_transcript_variant,intron_variant
rs1224927716	intron_variant
rs1224944892	genic_downstream_transcript_variant,intron_variant
rs1224954264	genic_downstream_transcript_variant,intron_variant
rs1224964269	genic_upstream_transcript_variant,intron_variant
rs1225003038	genic_downstream_transcript_variant,intron_variant
rs1225012831	genic_downstream_transcript_variant,intron_variant
rs1225060575	intron_variant
rs1225060760	intron_variant
rs1225062422	intron_variant
rs1225064569	genic_downstream_transcript_variant,intron_variant
rs1225103516	genic_upstream_transcript_variant,intron_variant
rs1225105688	genic_downstream_transcript_variant,intron_variant
rs1225146848	genic_upstream_transcript_variant,intron_variant
rs1225149337	intron_variant
rs1225170663	genic_upstream_transcript_variant,intron_variant
rs1225180946	intron_variant
rs1225209076	genic_upstream_transcript_variant,intron_variant
rs1225209376	genic_downstream_transcript_variant,intron_variant
rs1225219480	intron_variant
rs1225222038	genic_downstream_transcript_variant,intron_variant
rs1225226071	intron_variant
rs1225238848	genic_downstream_transcript_variant,intron_variant
rs1225346727	intron_variant
rs1225367781	intron_variant
rs1225387817	intron_variant
rs1225408406	intron_variant
rs1225408683	intron_variant
rs1225427331	genic_upstream_transcript_variant,intron_variant
rs1225458521	genic_downstream_transcript_variant,intron_variant
rs1225490440	genic_upstream_transcript_variant,intron_variant
rs1225522515	intron_variant
rs1225539426	genic_upstream_transcript_variant,intron_variant
rs1225582482	genic_upstream_transcript_variant,intron_variant
rs1225588592	intron_variant
rs1225594603	missense_variant,intron_variant,genic_upstream_transcript_variant,coding_sequence_variant,non_coding_transcript_variant
rs1225597759	intron_variant
rs1225638395	genic_upstream_transcript_variant,intron_variant
rs1225646520	intron_variant
rs1225678316	genic_upstream_transcript_variant,intron_variant
rs1225681469	genic_upstream_transcript_variant,intron_variant

rs1225696530	intron_variant
rs1225699255	genic_upstream_transcript_variant,intron_variant
rs1225707377	genic_downstream_transcript_variant,intron_variant
rs1225713805	intron_variant
rs1225737207	intron_variant
rs1225747315	genic_downstream_transcript_variant,intron_variant
rs1225808498	genic_downstream_transcript_variant,intron_variant
rs1225893232	intron_variant
rs1225895154	genic_upstream_transcript_variant,intron_variant
rs1225899421	genic_upstream_transcript_variant,intron_variant
rs1225923801	intron_variant
rs1225961153	genic_downstream_transcript_variant,intron_variant
rs1225978138	genic_upstream_transcript_variant,upstream_transcript_variant,intron_variant
rs1225986200	genic_upstream_transcript_variant,upstream_transcript_variant,intron_variant
rs1226000281	genic_upstream_transcript_variant,intron_variant
rs1226016140	intron_variant
rs1226064884	genic_downstream_transcript_variant,intron_variant
rs1226083454	intron_variant
rs1226094191	genic_upstream_transcript_variant,intron_variant
rs1226179537	intron_variant
rs1226255571	intron_variant
rs1226273140	genic_downstream_transcript_variant,intron_variant
rs1226291241	genic_downstream_transcript_variant,intron_variant
rs1226309216	genic_upstream_transcript_variant,intron_variant
rs1226309949	intron_variant
rs1226311988	intron_variant
rs1226362262	genic_downstream_transcript_variant,intron_variant
rs1226431517	genic_upstream_transcript_variant,intron_variant
rs1226433059	genic_upstream_transcript_variant,intron_variant
rs1226437895	downstream_transcript_variant,500B_downstream_variant
rs1226462336	intron_variant
rs1226470165	genic_upstream_transcript_variant,intron_variant
rs1226507710	intron_variant
rs1226515243	genic_upstream_transcript_variant,upstream_transcript_variant,intron_variant
rs1226551595	genic_upstream_transcript_variant,intron_variant
rs1226552031	genic_downstream_transcript_variant,intron_variant
rs1226559510	intron_variant
rs1226627452	genic_upstream_transcript_variant,intron_variant
rs1226670445	genic_downstream_transcript_variant,intron_variant
rs1226689670	genic_upstream_transcript_variant,intron_variant
rs1226720935	intron_variant
rs1226729961	genic_upstream_transcript_variant,intron_variant
rs1226806378	genic_upstream_transcript_variant,intron_variant
rs1226818300	genic_upstream_transcript_variant,intron_variant
rs1226841387	genic_upstream_transcript_variant,intron_variant
rs1226845463	genic_downstream_transcript_variant,intron_variant
rs1226861890	genic_upstream_transcript_variant,intron_variant
rs1226939060	intron_variant
rs1226959863	intron_variant
rs1226964890	genic_upstream_transcript_variant,intron_variant
rs1226966968	intron_variant
rs1226991663	genic_upstream_transcript_variant,intron_variant
rs1227046289	genic_downstream_transcript_variant,intron_variant
rs1227079159	genic_downstream_transcript_variant,intron_variant
rs1227091238	missense_variant,intron_variant,genic_upstream_transcript_variant,coding_sequence_variant,non_coding_transcript_variant
rs1227153363	intron_variant
rs1227156192	intron_variant
rs1227167695	intron_variant
rs1227179836	genic_upstream_transcript_variant,intron_variant
rs1227197476	intron_variant
rs1227205972	genic_upstream_transcript_variant,intron_variant
rs1227206570	5_prime_UTR_variant,non_coding_transcript_variant,intron_variant,genic_upstream_transcript_variant
rs1227223433	intron_variant
rs1227280545	genic_upstream_transcript_variant,intron_variant
rs1227311436	genic_upstream_transcript_variant,intron_variant
rs1227335311	genic_upstream_transcript_variant,intron_variant
rs1227355036	intron_variant
rs1227408112	intron_variant
rs1227505469	genic_upstream_transcript_variant,intron_variant
rs1227530022	intron_variant
rs1227566865	intron_variant
rs1227569042	genic_downstream_transcript_variant,intron_variant
rs1227582782	intron_variant
rs1227626613	genic_upstream_transcript_variant,intron_variant

rs1227639879	genic_upstream_transcript_variant,intron_variant
rs1227663377	intron_variant
rs1227671284	intron_variant
rs1227695316	genic_upstream_transcript_variant,intron_variant
rs1227716087	intron_variant
rs1227795260	genic_upstream_transcript_variant,intron_variant
rs1227818910	genic_upstream_transcript_variant,intron_variant
rs1227829336	genic_upstream_transcript_variant,intron_variant
rs1227851611	2KB_upstream_variant,upstream_transcript_variant,intron_variant,genic_upstream_transcript_variant
rs1227854767	intron_variant
rs1227924930	genic_upstream_transcript_variant,intron_variant
rs1227939852	genic_upstream_transcript_variant,intron_variant
rs1227946309	genic_upstream_transcript_variant,intron_variant
rs1228006009	genic_upstream_transcript_variant,intron_variant
rs1228030872	genic_downstream_transcript_variant,intron_variant
rs1228066468	genic_upstream_transcript_variant,intron_variant
rs1228085611	intron_variant
rs1228100842	genic_downstream_transcript_variant,intron_variant
rs1228112656	genic_upstream_transcript_variant,intron_variant
rs1228137550	intron_variant
rs1228138895	intron_variant
rs1228167684	genic_downstream_transcript_variant,intron_variant
rs1228182400	intron_variant
rs1228211068	genic_upstream_transcript_variant,intron_variant
rs1228229618	genic_upstream_transcript_variant,intron_variant
rs1228294519	genic_upstream_transcript_variant,intron_variant
rs1228304526	genic_upstream_transcript_variant,intron_variant
rs1228307500	genic_downstream_transcript_variant,intron_variant
rs1228320548	genic_upstream_transcript_variant,intron_variant
rs1228338878	genic_upstream_transcript_variant,upstream_transcript_variant,intron_variant
rs1228361885	genic_upstream_transcript_variant,upstream_transcript_variant,intron_variant
rs1228362771	genic_upstream_transcript_variant,intron_variant
rs1228381644	intron_variant
rs1228402271	2KB_upstream_variant,genic_upstream_transcript_variant,upstream_transcript_variant,intron_variant
rs1228438123	intron_variant
rs1228474374	genic_downstream_transcript_variant,intron_variant
rs1228479669	genic_upstream_transcript_variant,intron_variant
rs1228483034	genic_upstream_transcript_variant,intron_variant
rs1228488755	genic_upstream_transcript_variant,intron_variant
rs1228495466	intron_variant
rs1228514890	intron_variant
rs1228544283	intron_variant
rs1228587901	genic_upstream_transcript_variant,intron_variant
rs1228641008	genic_upstream_transcript_variant,intron_variant
rs1228643710	intron_variant
rs1228683429	genic_upstream_transcript_variant,intron_variant
rs1228683712	genic_upstream_transcript_variant,intron_variant
rs1228709099	genic_upstream_transcript_variant,intron_variant
rs1228808955	genic_upstream_transcript_variant,intron_variant
rs1228869887	intron_variant
rs1228899986	intron_variant
rs1228930555	genic_upstream_transcript_variant,intron_variant
rs1228951837	genic_downstream_transcript_variant,3_prime_UTR_variant,non_coding_transcript_variant
rs1228957883	genic_downstream_transcript_variant,intron_variant
rs1228968710	genic_downstream_transcript_variant,intron_variant
rs1229061935	genic_upstream_transcript_variant,intron_variant
rs1229092747	genic_upstream_transcript_variant,intron_variant
rs1229113386	genic_upstream_transcript_variant,intron_variant
rs1229128530	genic_upstream_transcript_variant,intron_variant
rs1229130114	genic_upstream_transcript_variant,intron_variant
rs1229135891	genic_upstream_transcript_variant,intron_variant
rs1229151724	genic_upstream_transcript_variant,upstream_transcript_variant,intron_variant
rs1229160446	genic_upstream_transcript_variant,intron_variant
rs1229220149	intron_variant
rs1229271317	genic_upstream_transcript_variant,intron_variant
rs1229295007	genic_upstream_transcript_variant,intron_variant
rs1229299167	intron_variant
rs1229307530	genic_downstream_transcript_variant,downstream_transcript_variant,intron_variant
rs1229310577	intron_variant
rs1229312045	intron_variant
rs1229367145	genic_downstream_transcript_variant,3_prime_UTR_variant,non_coding_transcript_variant
rs1229381803	intron_variant
rs1229397624	genic_upstream_transcript_variant,intron_variant
rs1229402220	genic_upstream_transcript_variant,intron_variant

rs1229408685	genic_upstream_transcript_variant,intron_variant
rs1229421695	intron_variant
rs1229447635	genic_upstream_transcript_variant,intron_variant
rs1229487223	genic_downstream_transcript_variant,3_prime_UTR_variant,non_coding_transcript_variant
rs1229493852	genic_upstream_transcript_variant,intron_variant
rs1229506400	genic_upstream_transcript_variant,intron_variant
rs1229511041	intron_variant
rs1229514890	genic_upstream_transcript_variant,intron_variant
rs1229571629	intron_variant
rs1229572726	intron_variant
rs1229591207	genic_upstream_transcript_variant,intron_variant
rs1229593922	missense_variant,intron_variant,genic_upstream_transcript_variant,coding_sequence_variant,non_coding_transcript_variant
rs1229607462	genic_upstream_transcript_variant,intron_variant
rs1229658669	genic_upstream_transcript_variant,intron_variant
rs1229688054	genic_upstream_transcript_variant,intron_variant
rs1229707662	genic_upstream_transcript_variant,intron_variant
rs1229728910	genic_downstream_transcript_variant,intron_variant
rs1229733734	genic_upstream_transcript_variant,intron_variant
rs1229737039	genic_downstream_transcript_variant,intron_variant
rs1229774845	genic_downstream_transcript_variant,intron_variant
rs1229803564	genic_upstream_transcript_variant,intron_variant
rs1229808090	genic_downstream_transcript_variant,intron_variant
rs1229870432	genic_downstream_transcript_variant,3_prime_UTR_variant,non_coding_transcript_variant
rs1229882987	genic_downstream_transcript_variant,intron_variant
rs1229918278	genic_upstream_transcript_variant,intron_variant
rs1229954521	intron_variant
rs1229971370	genic_downstream_transcript_variant,intron_variant
rs1230014614	intron_variant
rs1230065602	genic_upstream_transcript_variant,intron_variant
rs1230138417	genic_downstream_transcript_variant,intron_variant
rs1230160796	intron_variant
rs1230195272	5_prime_UTR_variant,intron_variant
rs1230201999	intron_variant
rs1230228661	genic_downstream_transcript_variant,intron_variant
rs1230305243	genic_upstream_transcript_variant,intron_variant
rs1230381311	genic_upstream_transcript_variant,intron_variant
rs1230386206	intron_variant
rs1230424308	intron_variant
rs1230452615	genic_upstream_transcript_variant,intron_variant
rs1230456059	2KB_upstream_variant,upstream_transcript_variant,intron_variant,genic_upstream_transcript_variant
rs1230481832	intron_variant
rs1230499713	genic_upstream_transcript_variant,intron_variant
rs1230512852	genic_downstream_transcript_variant,intron_variant
rs1230513660	genic_upstream_transcript_variant,intron_variant
rs1230563599	genic_upstream_transcript_variant,intron_variant
rs1230618834	intron_variant
rs1230625246	genic_downstream_transcript_variant,intron_variant
rs1230663213	genic_upstream_transcript_variant,intron_variant
rs1230681907	genic_downstream_transcript_variant,intron_variant
rs1230715421	intron_variant
rs1230731097	genic_downstream_transcript_variant,intron_variant
rs1230819446	genic_upstream_transcript_variant,intron_variant
rs1230825633	genic_upstream_transcript_variant,intron_variant
rs1230840605	genic_upstream_transcript_variant,intron_variant
rs1230894448	intron_variant
rs1230897683	intron_variant
rs1230898937	intron_variant
rs1230937035	genic_upstream_transcript_variant,intron_variant
rs1230951143	genic_upstream_transcript_variant,intron_variant
rs1230964289	intron_variant
rs1230985748	intron_variant
rs1231003085	intron_variant
rs1231007612	intron_variant
rs1231012742	genic_upstream_transcript_variant,intron_variant
rs1231115092	intron_variant
rs1231117505	intron_variant
rs1231149653	genic_upstream_transcript_variant,intron_variant
rs1231158668	genic_upstream_transcript_variant,intron_variant
rs1231165337	genic_downstream_transcript_variant,3_prime_UTR_variant,non_coding_transcript_variant
rs1231201110	genic_upstream_transcript_variant,intron_variant
rs1231267202	intron_variant
rs1231272435	genic_upstream_transcript_variant,intron_variant
rs1231324273	intron_variant
rs1231341397	intron_variant

rs1231354604	genic_downstream_transcript_variant,intron_variant
rs1231374786	genic_upstream_transcript_variant,intron_variant
rs1231398922	2KB_upstream_variant,upstream_transcript_variant,intron_variant,genic_upstream_transcript_variant
rs1231402228	genic_upstream_transcript_variant,intron_variant
rs1231458805	intron_variant
rs1231479862	5_prime_UTR_variant,intron_variant
rs1231503107	5_prime_UTR_variant,intron_variant
rs1231507570	genic_downstream_transcript_variant,downstream_transcript_variant,intron_variant
rs1231529408	genic_upstream_transcript_variant,intron_variant
rs1231575293	genic_upstream_transcript_variant,intron_variant
rs1231611391	genic_upstream_transcript_variant,intron_variant
rs1231626589	intron_variant
rs1231690050	genic_downstream_transcript_variant,intron_variant
rs1231735175	intron_variant
rs1231742323	intron_variant
rs1231772512	genic_upstream_transcript_variant,intron_variant
rs1231781773	intron_variant
rs1231805025	genic_upstream_transcript_variant,intron_variant
rs1231852458	intron_variant
rs1231852743	intron_variant
rs1231856319	intron_variant
rs1231875851	genic_upstream_transcript_variant,intron_variant
rs1231883271	genic_downstream_transcript_variant,3_prime_UTR_variant,non_coding_transcript_variant
rs1231956990	intron_variant
rs1231966438	genic_upstream_transcript_variant,intron_variant
rs1232016975	2KB_upstream_variant,upstream_transcript_variant,intron_variant,genic_upstream_transcript_variant
rs1232057735	intron_variant
rs1232061145	genic_downstream_transcript_variant,intron_variant
rs1232064433	intron_variant
rs1232077999	genic_upstream_transcript_variant,intron_variant
rs1232093375	genic_upstream_transcript_variant,intron_variant
rs1232096092	intron_variant
rs1232105599	intron_variant
rs1232139763	intron_variant
rs1232160041	genic_upstream_transcript_variant,intron_variant
rs1232172495	genic_downstream_transcript_variant,intron_variant
rs1232245105	genic_downstream_transcript_variant,intron_variant
rs1232324523	2KB_upstream_variant,upstream_transcript_variant,intron_variant,genic_upstream_transcript_variant
rs1232331496	genic_upstream_transcript_variant,intron_variant
rs1232370917	intron_variant
rs1232375563	intron_variant
rs1232420803	intron_variant
rs1232436183	genic_upstream_transcript_variant,upstream_transcript_variant,intron_variant
rs1232458237	intron_variant
rs1232486656	genic_upstream_transcript_variant,intron_variant
rs1232496532	genic_upstream_transcript_variant,intron_variant
rs1232534816	genic_downstream_transcript_variant,intron_variant
rs1232582516	genic_downstream_transcript_variant,intron_variant
rs1232614145	intron_variant
rs1232615249	genic_upstream_transcript_variant,intron_variant
rs1232623795	intron_variant
rs1232623846	genic_upstream_transcript_variant,intron_variant
rs1232637074	genic_upstream_transcript_variant,intron_variant
rs1232641857	intron_variant
rs1232673465	genic_upstream_transcript_variant,intron_variant
rs1232687474	intron_variant
rs1232709115	genic_upstream_transcript_variant,upstream_transcript_variant,intron_variant
rs1232731197	genic_upstream_transcript_variant,intron_variant
rs1232738322	genic_downstream_transcript_variant,intron_variant
rs1232795077	intron_variant
rs1232807514	genic_upstream_transcript_variant,intron_variant
rs1232863608	intron_variant
rs1232879423	genic_downstream_transcript_variant,intron_variant
rs1232892081	genic_upstream_transcript_variant,intron_variant
rs1232933533	intron_variant
rs1232947155	genic_downstream_transcript_variant,intron_variant
rs1232959517	intron_variant
rs1232960075	genic_upstream_transcript_variant,intron_variant
rs1232988004	intron_variant
rs1233026792	genic_downstream_transcript_variant,intron_variant
rs1233043791	genic_downstream_transcript_variant,intron_variant
rs1233062399	genic_upstream_transcript_variant,intron_variant
rs1233105584	genic_upstream_transcript_variant,intron_variant
rs1233130248	intron_variant

rs1233143350	intron_variant
rs1233166956	genic_downstream_transcript_variant,intron_variant
rs1233171888	genic_upstream_transcript_variant,intron_variant
rs1233186975	genic_upstream_transcript_variant,intron_variant
rs1233219875	genic_downstream_transcript_variant,3_prime_UTR_variant,non_coding_transcript_variant
rs1233277641	intron_variant
rs1233295872	genic_downstream_transcript_variant,3_prime_UTR_variant,non_coding_transcript_variant
rs1233298554	intron_variant
rs1233307049	genic_upstream_transcript_variant,intron_variant
rs1233396766	genic_upstream_transcript_variant,intron_variant
rs1233443866	genic_upstream_transcript_variant,intron_variant
rs1233475279	intron_variant
rs1233500550	genic_upstream_transcript_variant,intron_variant
rs1233566360	intron_variant
rs1233566622	genic_upstream_transcript_variant,intron_variant
rs1233590044	genic_upstream_transcript_variant,intron_variant
rs1233620992	intron_variant,genic_upstream_transcript_variant
rs1233621908	upstream_transcript_variant,intron_variant,2KB_upstream_variant,genic_upstream_transcript_variant
rs1233687622	intron_variant
rs1233780711	intron_variant,genic_upstream_transcript_variant
rs1233802560	intron_variant,genic_downstream_transcript_variant
rs1233811822	intron_variant,genic_upstream_transcript_variant
rs1233812726	intron_variant,genic_downstream_transcript_variant
rs1233836654	intron_variant
rs1233839296	intron_variant,genic_upstream_transcript_variant
rs1233852533	intron_variant,genic_upstream_transcript_variant
rs1233864818	intron_variant,genic_upstream_transcript_variant
rs1233869210	intron_variant,genic_upstream_transcript_variant
rs1233871234	intron_variant,genic_upstream_transcript_variant
rs1233881489	intron_variant
rs1233893999	intron_variant,genic_upstream_transcript_variant
rs1233896418	intron_variant,genic_upstream_transcript_variant
rs1233902050	intron_variant,genic_upstream_transcript_variant
rs1233941546	intron_variant,genic_upstream_transcript_variant
rs1233951560	intron_variant
rs1233988990	intron_variant
rs1233991294	intron_variant,genic_upstream_transcript_variant
rs1234062083	intron_variant
rs1234063504	intron_variant,genic_upstream_transcript_variant
rs1234095876	intron_variant,genic_downstream_transcript_variant
rs1234140847	intron_variant,genic_upstream_transcript_variant
rs1234216281	intron_variant,genic_downstream_transcript_variant
rs1234232688	intron_variant
rs1234274580	intron_variant
rs1234304094	intron_variant
rs1234306365	intron_variant,genic_downstream_transcript_variant
rs1234339598	intron_variant,genic_upstream_transcript_variant
rs1234386547	intron_variant
rs1234410632	intron_variant
rs1234454292	intron_variant,genic_upstream_transcript_variant
rs1234572151	intron_variant
rs1234576771	intron_variant
rs1234578029	intron_variant
rs1234649278	intron_variant
rs1234659408	intron_variant
rs1234737057	intron_variant
rs1234783494	intron_variant,genic_upstream_transcript_variant
rs1234838177	intron_variant,genic_upstream_transcript_variant
rs1234871797	intron_variant
rs1234876925	intron_variant,genic_upstream_transcript_variant
rs1234884854	intron_variant
rs1234954636	intron_variant,genic_downstream_transcript_variant
rs1234963974	intron_variant
rs1234966074	intron_variant,genic_upstream_transcript_variant
rs1235023827	intron_variant,genic_upstream_transcript_variant
rs1235076049	intron_variant,genic_upstream_transcript_variant
rs1235118376	intron_variant,genic_upstream_transcript_variant
rs1235120468	intron_variant,genic_downstream_transcript_variant
rs1235157276	intron_variant,genic_downstream_transcript_variant
rs1235194599	intron_variant,genic_upstream_transcript_variant
rs1235224028	intron_variant,genic_upstream_transcript_variant
rs1235227024	intron_variant,genic_downstream_transcript_variant
rs1235242184	intron_variant,genic_upstream_transcript_variant
rs1235254882	intron_variant,genic_upstream_transcript_variant

rs1235256081	intron_variant
rs1235268752	intron_variant
rs1235270217	intron_variant,genic_upstream_transcript_variant
rs1235281419	intron_variant,genic_upstream_transcript_variant
rs1235283594	intron_variant,genic_downstream_transcript_variant
rs1235341211	intron_variant,genic_upstream_transcript_variant
rs1235380576	intron_variant,genic_upstream_transcript_variant
rs1235395044	intron_variant,genic_upstream_transcript_variant
rs1235451226	intron_variant,genic_downstream_transcript_variant
rs1235460050	intron_variant
rs1235489358	intron_variant
rs1235496768	intron_variant
rs1235537330	intron_variant
rs1235593869	intron_variant
rs1235692400	intron_variant
rs1235696699	intron_variant,genic_upstream_transcript_variant
rs1235714248	intron_variant,genic_upstream_transcript_variant
rs1235750624	intron_variant,genic_upstream_transcript_variant
rs1235755029	genic_downstream_transcript_variant,missense_variant,coding_sequence_variant,non_coding_transcript_variant
rs1235811955	intron_variant
rs1235856921	intron_variant,genic_upstream_transcript_variant
rs1235876336	intron_variant
rs1235882637	intron_variant,genic_upstream_transcript_variant
rs1235885634	intron_variant,genic_downstream_transcript_variant
rs1235902821	intron_variant,genic_downstream_transcript_variant
rs1235911682	intron_variant
rs1235958960	intron_variant
rs1235967434	intron_variant
rs1235977945	intron_variant
rs1236023632	intron_variant,genic_upstream_transcript_variant
rs1236023633	intron_variant
rs1236051656	intron_variant,genic_upstream_transcript_variant
rs1236072091	intron_variant,genic_upstream_transcript_variant
rs1236077524	intron_variant,genic_upstream_transcript_variant
rs1236128864	intron_variant,upstream_transcript_variant,genic_upstream_transcript_variant
rs1236158827	intron_variant,genic_upstream_transcript_variant
rs1236159398	upstream_transcript_variant,5_prime_UTR_variant,intron_variant,genic_upstream_transcript_variant
rs1236177974	intron_variant,genic_upstream_transcript_variant
rs1236192811	intron_variant
rs1236214906	intron_variant,genic_upstream_transcript_variant
rs1236215887	intron_variant
rs1236226209	intron_variant
rs1236231025	downstream_transcript_variant,500B_downstream_variant
rs1236233056	intron_variant,upstream_transcript_variant,2KB_upstream_variant,genic_upstream_transcript_variant
rs1236317966	intron_variant,genic_upstream_transcript_variant
rs1236406125	intron_variant,genic_downstream_transcript_variant
rs1236438776	intron_variant,genic_downstream_transcript_variant
rs1236447067	intron_variant
rs1236472509	intron_variant
rs1236506742	intron_variant,genic_upstream_transcript_variant
rs1236508845	intron_variant
rs1236519089	intron_variant,genic_upstream_transcript_variant
rs1236548414	intron_variant,genic_upstream_transcript_variant
rs1236649959	intron_variant
rs1236658863	intron_variant
rs1236672662	intron_variant
rs1236700359	intron_variant,genic_upstream_transcript_variant
rs1236701900	genic_downstream_transcript_variant,3_prime_UTR_variant,non_coding_transcript_variant
rs1236792774	intron_variant,genic_downstream_transcript_variant
rs1236821779	intron_variant,genic_downstream_transcript_variant
rs1236822695	intron_variant,genic_upstream_transcript_variant
rs1236869672	intron_variant,genic_upstream_transcript_variant
rs1236915456	intron_variant
rs1236933109	intron_variant
rs1237006751	intron_variant,genic_upstream_transcript_variant
rs1237046425	intron_variant,genic_downstream_transcript_variant
rs1237051749	intron_variant,genic_upstream_transcript_variant
rs1237061041	intron_variant
rs1237107479	intron_variant
rs1237114831	upstream_transcript_variant,intron_variant,2KB_upstream_variant,genic_upstream_transcript_variant
rs1237130006	intron_variant
rs1237135188	intron_variant
rs1237166350	intron_variant,genic_downstream_transcript_variant
rs1237229876	intron_variant,upstream_transcript_variant,genic_upstream_transcript_variant

rs1237234167	intron_variant,genic_upstream_transcript_variant
rs1237243858	intron_variant,genic_downstream_transcript_variant
rs1237267853	intron_variant
rs1237289073	intron_variant
rs1237410192	intron_variant
rs1237443951	intron_variant,genic_downstream_transcript_variant
rs1237453745	intron_variant,genic_downstream_transcript_variant
rs1237475770	intron_variant,genic_downstream_transcript_variant
rs1237533548	intron_variant,genic_upstream_transcript_variant
rs1237542066	intron_variant,genic_downstream_transcript_variant
rs1237564902	frameshift_variant,genic_downstream_transcript_variant,coding_sequence_variant,non_coding_transcript_variant
rs1237633030	intron_variant
rs1237636980	intron_variant
rs1237682517	upstream_transcript_variant,intron_variant,2KB_upstream_variant,genic_upstream_transcript_variant
rs1237686591	intron_variant
rs1237698399	intron_variant
rs1237743341	intron_variant
rs1237798032	intron_variant,genic_upstream_transcript_variant
rs1237800472	intron_variant
rs1237842304	intron_variant
rs1237843704	intron_variant,genic_upstream_transcript_variant
rs1237888339	intron_variant,genic_upstream_transcript_variant
rs1237905483	coding_sequence_variant,synonymous_variant,non_coding_transcript_variant
rs1237945961	intron_variant,genic_upstream_transcript_variant
rs1238007014	intron_variant
rs1238009134	intron_variant,genic_upstream_transcript_variant
rs1238046931	upstream_transcript_variant,intron_variant,2KB_upstream_variant,genic_upstream_transcript_variant
rs1238113666	intron_variant,genic_upstream_transcript_variant
rs1238138436	intron_variant
rs1238176721	intron_variant,genic_upstream_transcript_variant
rs1238225343	intron_variant,genic_upstream_transcript_variant
rs1238233483	intron_variant,genic_upstream_transcript_variant
rs1238252542	intron_variant,genic_upstream_transcript_variant
rs1238282285	intron_variant
rs1238305409	intron_variant,genic_upstream_transcript_variant
rs1238359909	intron_variant,genic_downstream_transcript_variant
rs1238394741	intron_variant,genic_downstream_transcript_variant
rs1238410605	intron_variant,genic_upstream_transcript_variant
rs1238424246	intron_variant,genic_upstream_transcript_variant
rs1238427947	intron_variant
rs1238457112	intron_variant
rs1238515894	intron_variant,genic_upstream_transcript_variant
rs1238516911	intron_variant,genic_downstream_transcript_variant
rs1238562865	intron_variant
rs1238589856	intron_variant
rs1238668438	intron_variant,genic_upstream_transcript_variant
rs1238726284	intron_variant,genic_upstream_transcript_variant
rs1238772696	intron_variant
rs1238790150	intron_variant
rs1238803216	intron_variant
rs1238825926	intron_variant,genic_upstream_transcript_variant
rs1238828612	intron_variant,genic_upstream_transcript_variant
rs1238834195	intron_variant
rs1238853911	intron_variant,genic_upstream_transcript_variant
rs1238877632	intron_variant,genic_downstream_transcript_variant
rs1238901545	intron_variant,genic_upstream_transcript_variant
rs1238907796	intron_variant
rs1238929514	intron_variant,genic_upstream_transcript_variant
rs1238960135	intron_variant,upstream_transcript_variant,genic_upstream_transcript_variant
rs1238960867	intron_variant
rs1238974806	intron_variant,genic_upstream_transcript_variant
rs1238980622	intron_variant
rs1238983700	genic_downstream_transcript_variant,missense_variant,coding_sequence_variant,non_coding_transcript_variant
rs1238985908	genic_downstream_transcript_variant,3_prime_UTR_variant,non_coding_transcript_variant
rs1239045246	intron_variant,genic_upstream_transcript_variant
rs1239045696	intron_variant,upstream_transcript_variant,2KB_upstream_variant,genic_upstream_transcript_variant
rs1239054240	intron_variant,upstream_transcript_variant,genic_upstream_transcript_variant
rs1239064267	intron_variant,genic_upstream_transcript_variant
rs1239067028	intron_variant
rs1239162925	intron_variant
rs1239166904	missense_variant,non_coding_transcript_variant,coding_sequence_variant,genic_upstream_transcript_variant,intron_variant
rs1239195789	intron_variant
rs1239202459	intron_variant
rs1239211431	intron_variant

rs1239269198	intron_variant,upstream_transcript_variant,genic_upstream_transcript_variant
rs1239285418	intron_variant
rs1239315770	intron_variant
rs1239326673	intron_variant,genic_upstream_transcript_variant
rs1239338337	intron_variant,genic_upstream_transcript_variant
rs1239345327	intron_variant
rs1239364505	intron_variant,genic_upstream_transcript_variant
rs1239407310	intron_variant,genic_upstream_transcript_variant
rs1239443054	intron_variant,genic_upstream_transcript_variant
rs1239443930	intron_variant
rs1239464800	missense_variant,coding_sequence_variant,non_coding_transcript_variant
rs1239501260	intron_variant,genic_upstream_transcript_variant
rs1239559354	intron_variant
rs1239640523	intron_variant,genic_upstream_transcript_variant
rs1239641665	intron_variant
rs1239643960	intron_variant,genic_downstream_transcript_variant
rs1239658974	intron_variant
rs1239659071	intron_variant,genic_downstream_transcript_variant
rs1239661289	intron_variant,genic_upstream_transcript_variant
rs1239692748	intron_variant
rs1239701531	intron_variant,genic_upstream_transcript_variant
rs1239705581	intron_variant
rs1239751029	intron_variant,genic_upstream_transcript_variant
rs1239772560	intron_variant,genic_upstream_transcript_variant
rs1239783995	intron_variant,genic_upstream_transcript_variant
rs1239827390	intron_variant
rs1239837205	intron_variant
rs1239843462	intron_variant,genic_downstream_transcript_variant
rs1239876936	intron_variant,genic_upstream_transcript_variant
rs1239961935	intron_variant,genic_downstream_transcript_variant
rs1239967692	intron_variant,genic_upstream_transcript_variant
rs1239970077	intron_variant
rs1239995564	intron_variant
rs1240041843	genic_downstream_transcript_variant,3_prime_UTR_variant,non_coding_transcript_variant
rs1240044110	intron_variant,genic_upstream_transcript_variant
rs1240050099	intron_variant,genic_upstream_transcript_variant
rs1240054071	intron_variant,genic_downstream_transcript_variant
rs1240055274	intron_variant
rs1240062915	intron_variant,genic_downstream_transcript_variant
rs1240073622	upstream_transcript_variant,intron_variant,2KB_upstream_variant,genic_upstream_transcript_variant
rs1240124152	intron_variant,upstream_transcript_variant,genic_upstream_transcript_variant
rs1240159233	intron_variant,genic_upstream_transcript_variant
rs1240183703	intron_variant
rs1240207858	intron_variant,genic_upstream_transcript_variant
rs1240213404	intron_variant
rs1240239236	intron_variant,genic_upstream_transcript_variant
rs1240324571	intron_variant,upstream_transcript_variant,genic_upstream_transcript_variant
rs1240336700	intron_variant,genic_upstream_transcript_variant
rs1240353665	intron_variant
rs1240401454	intron_variant
rs1240402954	intron_variant,genic_upstream_transcript_variant
rs1240416935	intron_variant,genic_upstream_transcript_variant
rs1240429691	intron_variant,genic_downstream_transcript_variant
rs1240472114	intron_variant,genic_upstream_transcript_variant
rs1240492636	intron_variant,genic_upstream_transcript_variant
rs1240528334	intron_variant,genic_downstream_transcript_variant
rs1240546343	intron_variant,upstream_transcript_variant,genic_upstream_transcript_variant
rs1240591603	intron_variant,genic_upstream_transcript_variant
rs1240610557	intron_variant,genic_upstream_transcript_variant
rs1240641815	upstream_transcript_variant,intron_variant,2KB_upstream_variant,genic_upstream_transcript_variant
rs1240659674	intron_variant
rs1240694645	intron_variant,genic_downstream_transcript_variant
rs1240712172	intron_variant,genic_downstream_transcript_variant
rs1240757549	intron_variant,genic_upstream_transcript_variant
rs1240763462	intron_variant,genic_upstream_transcript_variant
rs1240769804	intron_variant,genic_upstream_transcript_variant
rs1240805675	intron_variant
rs1240840582	intron_variant,genic_upstream_transcript_variant
rs1240899031	intron_variant
rs1240951833	intron_variant,genic_downstream_transcript_variant
rs1240962919	intron_variant
rs1240996344	intron_variant
rs1241024131	intron_variant,genic_downstream_transcript_variant
rs1241032768	intron_variant,genic_upstream_transcript_variant

rs1241038941	intron_variant,genic_upstream_transcript_variant
rs1241046010	intron_variant,genic_upstream_transcript_variant
rs1241051905	intron_variant
rs1241068747	intron_variant
rs1241081246	intron_variant,genic_upstream_transcript_variant
rs1241101284	intron_variant,genic_downstream_transcript_variant
rs1241137350	intron_variant,genic_upstream_transcript_variant
rs1241159792	intron_variant,genic_upstream_transcript_variant
rs1241171384	intron_variant,genic_upstream_transcript_variant
rs1241211751	intron_variant,genic_upstream_transcript_variant
rs1241215493	intron_variant,genic_downstream_transcript_variant
rs1241216726	intron_variant,upstream_transcript_variant,2KB_upstream_variant,genic_upstream_transcript_variant
rs1241266420	intron_variant,genic_upstream_transcript_variant
rs1241277246	intron_variant
rs1241326221	intron_variant
rs1241338160	intron_variant,genic_downstream_transcript_variant
rs1241342716	intron_variant,genic_upstream_transcript_variant
rs1241357107	intron_variant
rs1241364129	intron_variant
rs1241366172	intron_variant,genic_upstream_transcript_variant
rs1241452106	intron_variant
rs1241465049	intron_variant,genic_downstream_transcript_variant
rs1241473498	intron_variant
rs1241544907	intron_variant,genic_downstream_transcript_variant
rs1241548162	intron_variant
rs1241559449	intron_variant
rs1241589791	intron_variant,genic_upstream_transcript_variant
rs1241624156	intron_variant,genic_upstream_transcript_variant
rs1241649184	upstream_transcript_variant,intron_variant,2KB_upstream_variant,genic_upstream_transcript_variant
rs1241666014	intron_variant
rs1241667511	intron_variant,upstream_transcript_variant,genic_upstream_transcript_variant
rs1241686709	intron_variant,genic_upstream_transcript_variant
rs1241698955	intron_variant,genic_downstream_transcript_variant
rs1241747730	intron_variant
rs1241765208	upstream_transcript_variant,intron_variant,2KB_upstream_variant,genic_upstream_transcript_variant
rs1241804169	intron_variant
rs1241875488	intron_variant
rs1241895940	intron_variant,genic_upstream_transcript_variant
rs1241908649	upstream_transcript_variant,intron_variant,2KB_upstream_variant,genic_upstream_transcript_variant
rs1241965643	intron_variant,genic_upstream_transcript_variant
rs1242002306	intron_variant,genic_upstream_transcript_variant
rs1242004149	intron_variant
rs1242039940	intron_variant,genic_downstream_transcript_variant
rs1242079368	intron_variant
rs1242094186	intron_variant,upstream_transcript_variant,2KB_upstream_variant,genic_upstream_transcript_variant
rs1242122235	intron_variant,genic_upstream_transcript_variant
rs1242190172	intron_variant,genic_upstream_transcript_variant
rs1242191088	upstream_transcript_variant,intron_variant,2KB_upstream_variant,genic_upstream_transcript_variant
rs1242221055	intron_variant
rs1242252663	intron_variant,genic_upstream_transcript_variant
rs1242253316	intron_variant,genic_upstream_transcript_variant
rs1242268921	intron_variant
rs1242336178	intron_variant,genic_upstream_transcript_variant
rs1242350412	intron_variant,genic_upstream_transcript_variant
rs1242355593	intron_variant,genic_upstream_transcript_variant
rs1242472268	intron_variant
rs1242504420	intron_variant,genic_upstream_transcript_variant
rs1242530690	intron_variant,genic_upstream_transcript_variant
rs1242535875	intron_variant,genic_upstream_transcript_variant
rs1242550608	intron_variant,genic_upstream_transcript_variant
rs1242634687	intron_variant,upstream_transcript_variant,genic_upstream_transcript_variant
rs1242757118	intron_variant,genic_downstream_transcript_variant
rs1242758089	intron_variant,genic_upstream_transcript_variant
rs1242778605	intron_variant,genic_upstream_transcript_variant
rs1242779381	intron_variant,upstream_transcript_variant,genic_upstream_transcript_variant
rs1242792234	intron_variant,genic_downstream_transcript_variant
rs1242795641	intron_variant,genic_upstream_transcript_variant
rs1242802428	intron_variant,genic_upstream_transcript_variant
rs1242811908	intron_variant,genic_downstream_transcript_variant
rs1242814365	intron_variant,upstream_transcript_variant,genic_upstream_transcript_variant
rs1242842266	intron_variant
rs1242927603	intron_variant,upstream_transcript_variant,2KB_upstream_variant,genic_upstream_transcript_variant
rs1242942858	intron_variant,genic_downstream_transcript_variant
rs1242953645	intron_variant

rs1242974814	intron_variant,genic_upstream_transcript_variant
rs1242990387	intron_variant,genic_upstream_transcript_variant
rs1242999855	intron_variant,genic_downstream_transcript_variant
rs1243091254	intron_variant
rs1243179913	intron_variant,genic_upstream_transcript_variant
rs1243190210	intron_variant
rs1243233537	intron_variant
rs1243246671	intron_variant,genic_upstream_transcript_variant
rs1243269626	intron_variant,genic_upstream_transcript_variant
rs1243304925	intron_variant
rs1243310779	intron_variant,genic_upstream_transcript_variant
rs1243331052	upstream_transcript_variant,5_prime_UTR_variant,intron_variant,genic_upstream_transcript_variant
rs1243340837	intron_variant
rs1243374825	intron_variant,upstream_transcript_variant,genic_upstream_transcript_variant
rs1243408136	intron_variant,genic_upstream_transcript_variant
rs1243440729	intron_variant,genic_downstream_transcript_variant
rs1243454877	intron_variant,genic_downstream_transcript_variant
rs1243466614	intron_variant,genic_downstream_transcript_variant
rs1243515171	intron_variant,genic_upstream_transcript_variant
rs1243538013	intron_variant
rs1243543318	genic_downstream_transcript_variant,terminator_codon_variant,synonymous_variant,non_coding_transcript_variant
rs1243589042	intron_variant,genic_downstream_transcript_variant
rs1243592867	intron_variant
rs1243648135	intron_variant
rs1243669987	intron_variant,genic_upstream_transcript_variant
rs1243671557	intron_variant
rs1243679064	intron_variant,genic_downstream_transcript_variant
rs1243706414	intron_variant
rs1243708887	intron_variant,genic_downstream_transcript_variant
rs1243742599	intron_variant
rs1243757538	intron_variant,genic_downstream_transcript_variant
rs1243809749	intron_variant
rs1243812465	intron_variant
rs1243814393	intron_variant,genic_upstream_transcript_variant
rs1243841297	intron_variant,genic_upstream_transcript_variant
rs1243877215	intron_variant,genic_downstream_transcript_variant
rs1243879172	intron_variant
rs1243920420	intron_variant,genic_upstream_transcript_variant
rs1243933251	intron_variant,genic_upstream_transcript_variant
rs1243950308	intron_variant,genic_downstream_transcript_variant,downstream_transcript_variant
rs1243960466	intron_variant,genic_downstream_transcript_variant
rs1243994445	intron_variant,genic_downstream_transcript_variant
rs1243994799	intron_variant,genic_upstream_transcript_variant
rs1244031166	intron_variant,genic_upstream_transcript_variant
rs1244033869	intron_variant,genic_downstream_transcript_variant
rs1244052789	intron_variant,genic_upstream_transcript_variant
rs1244096375	intron_variant,upstream_transcript_variant,2KB_upstream_variant,genic_upstream_transcript_variant
rs1244120743	intron_variant,genic_upstream_transcript_variant
rs1244143614	intron_variant
rs1244147672	intron_variant,genic_downstream_transcript_variant,downstream_transcript_variant
rs1244156630	intron_variant
rs1244156923	intron_variant
rs1244207504	intron_variant
rs1244244280	intron_variant
rs1244244368	intron_variant,genic_downstream_transcript_variant
rs1244257057	downstream_transcript_variant,500B_downstream_variant
rs1244316167	intron_variant,genic_upstream_transcript_variant
rs1244359459	intron_variant,genic_downstream_transcript_variant
rs1244367394	intron_variant
rs1244406240	intron_variant
rs1244472435	intron_variant,genic_downstream_transcript_variant
rs1244472990	intron_variant,genic_upstream_transcript_variant
rs1244484211	intron_variant
rs1244518059	intron_variant
rs1244574188	downstream_transcript_variant,500B_downstream_variant
rs1244594882	intron_variant,genic_upstream_transcript_variant
rs1244674641	intron_variant,genic_upstream_transcript_variant
rs1244721967	intron_variant,genic_downstream_transcript_variant
rs1244761688	intron_variant
rs1244818772	intron_variant
rs1244819755	intron_variant,genic_upstream_transcript_variant
rs1244833624	intron_variant
rs1244859141	intron_variant
rs1244867478	intron_variant

rs1244956292	intron_variant,genic_upstream_transcript_variant
rs1244972425	intron_variant,genic_upstream_transcript_variant
rs1245039374	intron_variant,genic_upstream_transcript_variant
rs1245060818	intron_variant
rs1245062029	intron_variant,genic_downstream_transcript_variant
rs1245127838	intron_variant
rs1245142390	intron_variant,genic_upstream_transcript_variant
rs1245167046	intron_variant
rs1245240891	intron_variant,upstream_transcript_variant,genic_upstream_transcript_variant
rs1245247836	intron_variant,genic_upstream_transcript_variant
rs1245252905	intron_variant,genic_upstream_transcript_variant
rs1245289872	intron_variant,genic_upstream_transcript_variant
rs1245405703	intron_variant,genic_downstream_transcript_variant
rs1245417180	intron_variant,genic_upstream_transcript_variant,non_coding_transcript_variant
rs1245439491	intron_variant,genic_downstream_transcript_variant
rs1245495020	intron_variant,genic_downstream_transcript_variant
rs1245527574	intron_variant
rs1245578550	intron_variant,upstream_transcript_variant,genic_upstream_transcript_variant
rs1245608959	intron_variant,genic_upstream_transcript_variant
rs1245751303	intron_variant
rs1245763439	intron_variant,genic_upstream_transcript_variant
rs1245785485	intron_variant
rs1245804407	intron_variant
rs1245841003	intron_variant,genic_downstream_transcript_variant
rs1245856644	intron_variant,genic_upstream_transcript_variant
rs1245898130	intron_variant,genic_upstream_transcript_variant
rs1245899289	intron_variant,upstream_transcript_variant,genic_upstream_transcript_variant
rs1245903856	intron_variant
rs1245964675	intron_variant,upstream_transcript_variant,2KB_upstream_variant,genic_upstream_transcript_variant
rs1246056172	intron_variant
rs1246059627	intron_variant,genic_downstream_transcript_variant
rs1246085531	intron_variant
rs1246094460	missense_variant,coding_sequence_variant,non_coding_transcript_variant
rs1246096770	intron_variant,upstream_transcript_variant,genic_upstream_transcript_variant
rs1246148312	genic_upstream_transcript_variant,intron_variant
rs1246155653	intron_variant
rs1246169468	intron_variant
rs1246189397	genic_upstream_transcript_variant,intron_variant
rs1246232272	genic_upstream_transcript_variant,intron_variant
rs1246243243	intron_variant
rs1246257547	genic_downstream_transcript_variant,intron_variant
rs1246258794	intron_variant
rs1246268986	genic_upstream_transcript_variant,intron_variant
rs1246281148	non_coding_transcript_variant,intron_variant,genic_upstream_transcript_variant,missense_variant,coding_sequence_variant
rs1246335812	intron_variant
rs1246338971	genic_upstream_transcript_variant,intron_variant
rs1246364158	genic_upstream_transcript_variant,intron_variant
rs1246387116	intron_variant
rs1246406545	genic_downstream_transcript_variant,intron_variant
rs1246430010	intron_variant
rs1246437165	genic_upstream_transcript_variant,intron_variant
rs1246447307	5_prime_UTR_variant,intron_variant
rs1246474413	genic_downstream_transcript_variant,intron_variant
rs1246477666	genic_downstream_transcript_variant,intron_variant
rs1246506337	genic_downstream_transcript_variant,intron_variant
rs1246535788	genic_upstream_transcript_variant,intron_variant
rs1246542966	intron_variant
rs1246550980	intron_variant
rs1246574762	intron_variant
rs1246578196	intron_variant
rs1246659131	genic_upstream_transcript_variant,intron_variant
rs1246668877	genic_upstream_transcript_variant,intron_variant
rs1246700027	intron_variant
rs1246723457	non_coding_transcript_variant,3_prime_UTR_variant,genic_downstream_transcript_variant
rs1246737295	upstream_transcript_variant,genic_upstream_transcript_variant,intron_variant
rs1246738341	genic_upstream_transcript_variant,intron_variant
rs1246740315	genic_upstream_transcript_variant,intron_variant
rs1246841572	genic_downstream_transcript_variant,intron_variant
rs1246856635	intron_variant
rs1246861763	non_coding_transcript_variant,intron_variant,genic_upstream_transcript_variant,missense_variant,coding_sequence_variant
rs1246863396	downstream_transcript_variant,genic_downstream_transcript_variant,intron_variant
rs1246922287	non_coding_transcript_variant,3_prime_UTR_variant,genic_downstream_transcript_variant
rs1246968996	intron_variant
rs1247000152	non_coding_transcript_variant,3_prime_UTR_variant,genic_downstream_transcript_variant

rs1247049705	intron_variant
rs1247050595	intron_variant
rs1247053898	genic_upstream_transcript_variant,intron_variant
rs1247059282	genic_upstream_transcript_variant,intron_variant
rs1247082215	genic_downstream_transcript_variant,intron_variant
rs1247095935	genic_upstream_transcript_variant,intron_variant
rs1247100626	downstream_transcript_variant,genic_downstream_transcript_variant,intron_variant
rs1247108719	intron_variant
rs1247118703	genic_downstream_transcript_variant,intron_variant
rs1247168154	genic_upstream_transcript_variant,intron_variant
rs1247188385	genic_upstream_transcript_variant,intron_variant
rs1247191290	genic_upstream_transcript_variant,intron_variant
rs1247227893	upstream_transcript_variant,2KB_upstream_variant,genic_upstream_transcript_variant,intron_variant
rs1247248031	upstream_transcript_variant,genic_upstream_transcript_variant,intron_variant
rs1247257946	intron_variant
rs1247271392	genic_upstream_transcript_variant,intron_variant
rs1247326540	intron_variant
rs1247345002	downstream_transcript_variant,genic_downstream_transcript_variant,intron_variant
rs1247380258	intron_variant
rs1247434919	genic_upstream_transcript_variant,intron_variant
rs1247437750	intron_variant
rs1247449885	intron_variant
rs1247453898	intron_variant
rs1247463386	genic_upstream_transcript_variant,intron_variant
rs1247527345	genic_upstream_transcript_variant,intron_variant
rs1247563458	intron_variant
rs1247587739	genic_upstream_transcript_variant,intron_variant
rs1247607392	genic_upstream_transcript_variant,intron_variant
rs1247623978	intron_variant
rs1247646572	genic_upstream_transcript_variant,intron_variant
rs1247695567	genic_upstream_transcript_variant,intron_variant
rs1247715144	intron_variant
rs1247718119	intron_variant
rs1247750099	non_coding_transcript_variant,intron_variant,genic_upstream_transcript_variant,missense_variant,coding_sequence_variant
rs1247779257	genic_downstream_transcript_variant,intron_variant
rs1247873497	genic_upstream_transcript_variant,intron_variant
rs1247897232	genic_downstream_transcript_variant,intron_variant
rs1247971041	intron_variant
rs1247976272	genic_downstream_transcript_variant,intron_variant
rs1247978535	genic_upstream_transcript_variant,intron_variant
rs1247989872	genic_downstream_transcript_variant,intron_variant
rs1248004024	non_coding_transcript_variant,synonymous_variant,coding_sequence_variant
rs1248026146	genic_upstream_transcript_variant,intron_variant
rs1248128378	upstream_transcript_variant,2KB_upstream_variant,genic_upstream_transcript_variant,intron_variant
rs1248129894	genic_upstream_transcript_variant,intron_variant
rs1248160728	intron_variant
rs1248176452	intron_variant
rs1248182985	intron_variant
rs1248202249	genic_upstream_transcript_variant,intron_variant
rs1248209070	frameshift_variant,coding_sequence_variant,intron_variant
rs1248223065	genic_upstream_transcript_variant,intron_variant
rs1248285770	intron_variant
rs1248304212	genic_upstream_transcript_variant,intron_variant
rs1248322106	genic_upstream_transcript_variant,intron_variant
rs1248335861	genic_upstream_transcript_variant,intron_variant
rs1248342501	intron_variant
rs1248385547	genic_upstream_transcript_variant,intron_variant
rs1248428905	genic_upstream_transcript_variant,intron_variant
rs1248504576	genic_downstream_transcript_variant,intron_variant
rs1248526746	genic_upstream_transcript_variant,intron_variant
rs1248624760	upstream_transcript_variant,genic_upstream_transcript_variant,intron_variant
rs1248645608	genic_upstream_transcript_variant,intron_variant
rs1248651171	genic_downstream_transcript_variant,intron_variant
rs1248664255	genic_upstream_transcript_variant,intron_variant
rs1248685739	intron_variant
rs1248757632	genic_upstream_transcript_variant,intron_variant
rs1248781445	intron_variant
rs1248815067	intron_variant
rs1248848645	genic_downstream_transcript_variant,intron_variant
rs1248851448	genic_upstream_transcript_variant,intron_variant
rs1248891973	genic_upstream_transcript_variant,intron_variant
rs1248906887	intron_variant
rs1248907991	intron_variant
rs1248931356	genic_upstream_transcript_variant,intron_variant

rs1248942555	genic_upstream_transcript_variant,intron_variant
rs1248945324	intron_variant
rs1248965058	intron_variant
rs1249017576	non_coding_transcript_variant,3_prime_UTR_variant,genic_downstream_transcript_variant
rs1249031278	intron_variant
rs1249038754	intron_variant
rs1249049357	intron_variant
rs1249066509	upstream_transcript_variant,genic_upstream_transcript_variant,intron_variant
rs1249067598	genic_upstream_transcript_variant,intron_variant
rs1249122151	intron_variant
rs1249127459	genic_upstream_transcript_variant,intron_variant
rs1249129656	genic_upstream_transcript_variant,intron_variant
rs1249130635	genic_downstream_transcript_variant,intron_variant
rs1249131139	intron_variant
rs1249151756	genic_upstream_transcript_variant,intron_variant
rs1249158950	intron_variant
rs1249186892	genic_upstream_transcript_variant,intron_variant
rs1249241492	genic_upstream_transcript_variant,intron_variant
rs1249253912	genic_upstream_transcript_variant,intron_variant
rs1249266190	intron_variant
rs1249279188	genic_upstream_transcript_variant,intron_variant
rs1249287547	genic_upstream_transcript_variant,intron_variant
rs1249355694	genic_upstream_transcript_variant,intron_variant
rs1249371062	non_coding_transcript_variant,intron_variant,genic_upstream_transcript_variant,missense_variant,coding_sequence_variant
rs1249386826	intron_variant
rs1249389374	genic_upstream_transcript_variant,intron_variant
rs1249452450	genic_upstream_transcript_variant,intron_variant
rs1249460956	genic_upstream_transcript_variant,intron_variant
rs1249480381	genic_upstream_transcript_variant,intron_variant
rs1249561527	genic_upstream_transcript_variant,intron_variant
rs1249619506	intron_variant
rs1249634253	genic_downstream_transcript_variant,intron_variant
rs1249644070	intron_variant
rs1249703413	genic_upstream_transcript_variant,intron_variant
rs1249706800	genic_downstream_transcript_variant,intron_variant
rs1249710422	genic_upstream_transcript_variant,intron_variant
rs1249739995	non_coding_transcript_variant,terminator_codon_variant,stop_lost,genic_downstream_transcript_variant
rs1249826648	genic_upstream_transcript_variant,intron_variant
rs1249828435	genic_downstream_transcript_variant,intron_variant
rs1249835679	genic_downstream_transcript_variant,intron_variant
rs1249842113	genic_downstream_transcript_variant,intron_variant
rs1249846868	genic_downstream_transcript_variant,intron_variant
rs1249900741	genic_upstream_transcript_variant,intron_variant
rs1249911409	genic_downstream_transcript_variant,intron_variant
rs1249917723	genic_upstream_transcript_variant,intron_variant
rs1249921143	intron_variant
rs1249958152	intron_variant
rs1249974533	genic_upstream_transcript_variant,intron_variant
rs1249995422	intron_variant
rs1250003744	intron_variant
rs1250011901	genic_upstream_transcript_variant,intron_variant
rs1250033877	5_prime_UTR_variant,intron_variant
rs1250057707	genic_upstream_transcript_variant,intron_variant
rs1250111869	intron_variant,2KB_upstream_variant,genic_upstream_transcript_variant,upstream_transcript_variant
rs1250199038	genic_upstream_transcript_variant,intron_variant
rs1250227693	intron_variant
rs1250231374	genic_upstream_transcript_variant,intron_variant
rs1250268061	intron_variant
rs1250344565	genic_downstream_transcript_variant,intron_variant
rs1250378386	intron_variant
rs1250424137	genic_upstream_transcript_variant,intron_variant
rs1250495610	intron_variant
rs1250529984	intron_variant
rs1250577192	genic_upstream_transcript_variant,intron_variant
rs1250596944	intron_variant
rs1250608741	genic_upstream_transcript_variant,intron_variant
rs1250623222	genic_upstream_transcript_variant,intron_variant
rs1250623264	intron_variant
rs1250638401	genic_upstream_transcript_variant,intron_variant
rs1250699243	genic_upstream_transcript_variant,intron_variant
rs1250715249	genic_upstream_transcript_variant,intron_variant
rs1250717856	genic_downstream_transcript_variant,intron_variant
rs1250779402	genic_downstream_transcript_variant,intron_variant
rs1250782030	genic_upstream_transcript_variant,intron_variant

rs1250817714	intron_variant
rs1250868411	non_coding_transcript_variant,synonymous_variant,coding_sequence_variant
rs1250876544	intron_variant
rs1250883463	genic_downstream_transcript_variant,intron_variant
rs1250885143	genic_upstream_transcript_variant,intron_variant
rs1250937145	intron_variant
rs1250956651	genic_upstream_transcript_variant,intron_variant
rs1251033507	intron_variant,2KB_upstream_variant,genic_upstream_transcript_variant,upstream_transcript_variant
rs1251052402	non_coding_transcript_variant,3_prime_UTR_variant,genic_downstream_transcript_variant
rs1251127060	genic_downstream_transcript_variant,intron_variant
rs1251172893	genic_downstream_transcript_variant,intron_variant
rs1251204601	genic_downstream_transcript_variant,intron_variant
rs1251206482	downstream_transcript_variant,500B_downstream_variant
rs1251217396	intron_variant
rs1251221777	genic_upstream_transcript_variant,intron_variant
rs1251315558	intron_variant
rs1251327113	intron_variant
rs1251433924	genic_upstream_transcript_variant,intron_variant
rs1251521242	intron_variant
rs1251532738	intron_variant
rs1251616628	genic_upstream_transcript_variant,intron_variant
rs1251634173	genic_upstream_transcript_variant,intron_variant
rs1251647299	genic_upstream_transcript_variant,intron_variant
rs1251665860	intron_variant
rs1251701514	genic_downstream_transcript_variant,intron_variant
rs1251728273	genic_downstream_transcript_variant,intron_variant
rs1251746339	genic_upstream_transcript_variant,intron_variant
rs1251814057	genic_downstream_transcript_variant,intron_variant
rs1251830845	genic_downstream_transcript_variant,intron_variant
rs1251896821	genic_upstream_transcript_variant,intron_variant
rs1251920329	genic_downstream_transcript_variant,intron_variant
rs1251924604	intron_variant
rs1251928541	genic_upstream_transcript_variant,intron_variant
rs1251972255	non_coding_transcript_variant,3_prime_UTR_variant,genic_downstream_transcript_variant
rs1251984060	genic_upstream_transcript_variant,intron_variant
rs1252018592	intron_variant
rs1252048626	intron_variant
rs1252082066	genic_upstream_transcript_variant,intron_variant
rs1252132617	genic_upstream_transcript_variant,intron_variant
rs1252150202	genic_downstream_transcript_variant,intron_variant
rs1252161749	intron_variant
rs1252217722	intron_variant
rs1252220390	genic_upstream_transcript_variant,intron_variant
rs1252220941	genic_upstream_transcript_variant,intron_variant
rs1252223710	intron_variant
rs1252292829	intron_variant
rs1252304350	genic_upstream_transcript_variant,intron_variant
rs1252334252	genic_downstream_transcript_variant,intron_variant
rs1252335077	intron_variant
rs1252350977	intron_variant
rs1252391684	genic_upstream_transcript_variant,intron_variant
rs1252405183	intron_variant
rs1252430332	genic_upstream_transcript_variant,intron_variant
rs1252493910	genic_upstream_transcript_variant,intron_variant
rs1252495356	intron_variant
rs1252510349	genic_upstream_transcript_variant,intron_variant
rs1252519002	intron_variant
rs1252534978	intron_variant
rs1252547178	downstream_transcript_variant,500B_downstream_variant
rs1252548856	genic_downstream_transcript_variant,intron_variant
rs1252550524	intron_variant
rs1252558021	genic_upstream_transcript_variant,intron_variant
rs1252597448	genic_downstream_transcript_variant,intron_variant
rs1252603861	intron_variant
rs1252644328	genic_upstream_transcript_variant,intron_variant
rs1252649634	non_coding_transcript_variant,3_prime_UTR_variant,genic_downstream_transcript_variant
rs1252650433	intron_variant
rs1252666974	genic_upstream_transcript_variant,intron_variant
rs1252669700	downstream_transcript_variant,genic_downstream_transcript_variant,intron_variant
rs1252670221	genic_upstream_transcript_variant,intron_variant
rs1252673021	intron_variant,2KB_upstream_variant,genic_upstream_transcript_variant,upstream_transcript_variant
rs1252706945	genic_downstream_transcript_variant,intron_variant
rs1252721086	upstream_transcript_variant,genic_upstream_transcript_variant,intron_variant
rs1252728850	intron_variant

rs1252750802	upstream_transcript_variant,2KB_upstream_variant,genic_upstream_transcript_variant,intron_variant
rs1252758173	genic_upstream_transcript_variant,intron_variant
rs1252791293	genic_upstream_transcript_variant,intron_variant
rs1252807478	genic_upstream_transcript_variant,intron_variant
rs1252947198	genic_upstream_transcript_variant,intron_variant
rs1252957974	intron_variant
rs1252959557	intron_variant
rs1252968703	intron_variant
rs1252970686	intron_variant
rs1252973748	genic_upstream_transcript_variant,intron_variant
rs1252999025	genic_upstream_transcript_variant,intron_variant
rs1252999648	intron_variant,2KB_upstream_variant,genic_upstream_transcript_variant,upstream_transcript_variant
rs1253007078	genic_upstream_transcript_variant,intron_variant
rs1253024374	genic_downstream_transcript_variant,intron_variant
rs1253077547	genic_upstream_transcript_variant,intron_variant
rs1253077816	intron_variant
rs1253092794	upstream_transcript_variant,genic_upstream_transcript_variant,intron_variant
rs1253139441	genic_upstream_transcript_variant,intron_variant
rs1253171578	intron_variant
rs1253186200	upstream_transcript_variant,genic_upstream_transcript_variant,intron_variant
rs1253200284	intron_variant
rs1253211360	intron_variant
rs1253229197	intron_variant
rs1253244386	upstream_transcript_variant,genic_upstream_transcript_variant,intron_variant
rs1253262900	genic_downstream_transcript_variant,intron_variant
rs1253278690	intron_variant
rs1253302545	genic_downstream_transcript_variant,intron_variant
rs1253336512	intron_variant
rs1253403984	genic_upstream_transcript_variant,intron_variant
rs1253411803	intron_variant
rs1253413251	intron_variant
rs1253425235	intron_variant
rs1253443305	intron_variant
rs1253465718	genic_upstream_transcript_variant,intron_variant
rs1253468643	intron_variant
rs1253474228	intron_variant
rs1253485771	genic_downstream_transcript_variant,intron_variant
rs1253526590	genic_downstream_transcript_variant,intron_variant
rs1253559144	genic_upstream_transcript_variant,intron_variant
rs1253603006	intron_variant
rs1253613792	genic_upstream_transcript_variant,intron_variant
rs1253614566	intron_variant
rs1253640290	genic_upstream_transcript_variant,intron_variant
rs1253653570	intron_variant
rs1253675900	genic_downstream_transcript_variant,intron_variant
rs1253722469	intron_variant
rs1253747931	intron_variant
rs1253799789	genic_downstream_transcript_variant,intron_variant
rs1253819490	intron_variant
rs1253860544	intron_variant
rs1253868847	intron_variant
rs1253894496	non_coding_transcript_variant,missense_variant,coding_sequence_variant
rs1253933109	genic_upstream_transcript_variant,intron_variant
rs1253934855	genic_upstream_transcript_variant,intron_variant
rs1253945981	genic_downstream_transcript_variant,intron_variant
rs1253962831	genic_upstream_transcript_variant,intron_variant
rs1253974728	intron_variant
rs1253985996	intron_variant
rs1253990061	intron_variant
rs1254025252	genic_downstream_transcript_variant,intron_variant
rs1254056436	genic_upstream_transcript_variant,intron_variant
rs1254071485	intron_variant
rs1254090807	genic_upstream_transcript_variant,intron_variant
rs1254171826	genic_upstream_transcript_variant,intron_variant
rs1254215342	intron_variant
rs1254248620	intron_variant
rs1254259667	intron_variant
rs1254268734	genic_upstream_transcript_variant,intron_variant
rs1254288799	intron_variant
rs1254296852	genic_upstream_transcript_variant,intron_variant
rs1254313884	genic_downstream_transcript_variant,intron_variant
rs1254395342	intron_variant
rs1254407302	intron_variant
rs1254439890	intron_variant

rs1254539419	genic_upstream_transcript_variant,intron_variant
rs1254566448	intron_variant
rs1254604697	intron_variant
rs1254638714	non_coding_transcript_variant,intron_variant,genic_upstream_transcript_variant,coding_sequence_variant,synonymous_variant
rs1254672049	genic_upstream_transcript_variant,intron_variant
rs1254697440	genic_downstream_transcript_variant,intron_variant
rs1254707965	non_coding_transcript_variant,intron_variant,genic_upstream_transcript_variant,missense_variant,coding_sequence_variant
rs1254719560	non_coding_transcript_variant,intron_variant,genic_upstream_transcript_variant,missense_variant,coding_sequence_variant
rs1254723357	intron_variant
rs1254781638	genic_upstream_transcript_variant,intron_variant
rs1254792647	intron_variant
rs1254835503	genic_downstream_transcript_variant,intron_variant
rs1254883315	genic_upstream_transcript_variant,intron_variant
rs1254893347	genic_upstream_transcript_variant,intron_variant
rs1254906707	intron_variant
rs1254932832	intron_variant
rs1254992922	genic_upstream_transcript_variant,intron_variant
rs1255004413	intron_variant
rs1255010679	genic_upstream_transcript_variant,intron_variant
rs1255035188	genic_upstream_transcript_variant,intron_variant
rs1255052661	genic_upstream_transcript_variant,intron_variant
rs1255087036	intron_variant
rs1255093222	genic_upstream_transcript_variant,intron_variant
rs1255096350	genic_upstream_transcript_variant,intron_variant
rs1255125045	genic_downstream_transcript_variant,intron_variant
rs1255171543	genic_downstream_transcript_variant,intron_variant
rs1255179807	genic_upstream_transcript_variant,intron_variant
rs1255185058	non_coding_transcript_variant,missense_variant,genic_downstream_transcript_variant,coding_sequence_variant
rs1255233104	intron_variant
rs1255243283	intron_variant
rs1255299009	intron_variant
rs1255306147	genic_downstream_transcript_variant,intron_variant
rs1255309756	intron_variant
rs1255445254	genic_downstream_transcript_variant,intron_variant
rs1255471934	genic_upstream_transcript_variant,intron_variant
rs1255497343	genic_upstream_transcript_variant,intron_variant
rs1255558682	genic_upstream_transcript_variant,intron_variant
rs1255637586	intron_variant
rs1255652323	genic_upstream_transcript_variant,intron_variant
rs1255666549	genic_downstream_transcript_variant,intron_variant
rs1255713239	intron_variant
rs1255757299	genic_upstream_transcript_variant,intron_variant
rs1255776297	genic_downstream_transcript_variant,intron_variant
rs1255800063	genic_upstream_transcript_variant,intron_variant
rs1255812296	genic_upstream_transcript_variant,intron_variant
rs1255832364	genic_upstream_transcript_variant,intron_variant
rs1255858403	genic_upstream_transcript_variant,intron_variant
rs1255883868	intron_variant
rs1255904443	upstream_transcript_variant,2KB_upstream_variant,genic_upstream_transcript_variant,intron_variant
rs1255952729	upstream_transcript_variant,genic_upstream_transcript_variant,intron_variant
rs1255958255	genic_upstream_transcript_variant,intron_variant
rs1255959599	genic_upstream_transcript_variant,intron_variant
rs1255991317	genic_upstream_transcript_variant,intron_variant
rs1256017810	genic_downstream_transcript_variant,intron_variant
rs1256040061	genic_upstream_transcript_variant,intron_variant
rs1256063601	genic_upstream_transcript_variant,intron_variant
rs1256114756	intron_variant
rs1256119820	genic_downstream_transcript_variant,intron_variant
rs1256121742	genic_upstream_transcript_variant,intron_variant
rs1256150784	genic_upstream_transcript_variant,intron_variant
rs1256176208	genic_upstream_transcript_variant,intron_variant
rs1256190349	genic_downstream_transcript_variant,intron_variant
rs1256226583	upstream_transcript_variant,genic_upstream_transcript_variant,intron_variant
rs1256233370	intron_variant
rs1256274602	intron_variant
rs1256337863	intron_variant,2KB_upstream_variant,genic_upstream_transcript_variant,upstream_transcript_variant
rs1256344522	intron_variant
rs1256391739	genic_upstream_transcript_variant,intron_variant
rs1256425988	non_coding_transcript_variant,missense_variant,genic_downstream_transcript_variant,coding_sequence_variant
rs1256449467	upstream_transcript_variant,genic_upstream_transcript_variant,intron_variant
rs1256472069	intron_variant
rs1256537145	genic_downstream_transcript_variant,intron_variant
rs1256540242	intron_variant
rs1256586537	genic_upstream_transcript_variant,intron_variant

rs1256607494	intron_variant
rs1256638571	intron_variant
rs1256645640	genic_upstream_transcript_variant,intron_variant
rs1256668487	upstream_transcript_variant,genic_upstream_transcript_variant,intron_variant
rs1256673892	genic_upstream_transcript_variant,intron_variant
rs1256682723	intron_variant,2KB_upstream_variant,genic_upstream_transcript_variant,upstream_transcript_variant
rs1256693498	genic_upstream_transcript_variant,intron_variant
rs1256710095	intron_variant
rs1256711038	non_coding_transcript_variant,intron_variant,genic_upstream_transcript_variant,missense_variant,coding_sequence_variant
rs1256720573	genic_upstream_transcript_variant,intron_variant
rs1256728074	intron_variant
rs1256729427	genic_upstream_transcript_variant,intron_variant
rs1256761667	genic_downstream_transcript_variant,intron_variant
rs1256777568	intron_variant
rs1256817092	upstream_transcript_variant,2KB_upstream_variant,genic_upstream_transcript_variant,intron_variant
rs1256889016	genic_upstream_transcript_variant,intron_variant
rs1256920012	genic_upstream_transcript_variant,intron_variant
rs1256949694	genic_upstream_transcript_variant,intron_variant
rs1256991986	genic_upstream_transcript_variant,intron_variant
rs1256995654	intron_variant
rs1257003785	genic_upstream_transcript_variant,intron_variant
rs1257061539	genic_downstream_transcript_variant,intron_variant
rs1257156444	intron_variant
rs1257179733	genic_upstream_transcript_variant,intron_variant
rs1257190383	genic_upstream_transcript_variant,intron_variant
rs1257213734	genic_downstream_transcript_variant,intron_variant
rs1257243306	genic_downstream_transcript_variant,intron_variant
rs1257264066	genic_upstream_transcript_variant,intron_variant
rs1257277589	upstream_transcript_variant,2KB_upstream_variant,genic_upstream_transcript_variant,intron_variant
rs1257347653	intron_variant
rs1257362501	intron_variant
rs1257371417	intron_variant
rs1257393817	intron_variant
rs1257414540	intron_variant
rs1257460478	intron_variant
rs1257517606	genic_upstream_transcript_variant,intron_variant
rs1257522365	genic_downstream_transcript_variant,intron_variant
rs1257543439	genic_downstream_transcript_variant,intron_variant
rs1257544932	intron_variant
rs1257556323	genic_downstream_transcript_variant,intron_variant
rs1257573318	intron_variant
rs1257594734	genic_upstream_transcript_variant,intron_variant
rs1257606547	intron_variant
rs1257622227	genic_downstream_transcript_variant,intron_variant
rs1257643147	upstream_transcript_variant,genic_upstream_transcript_variant,intron_variant
rs1257647207	missense_variant,coding_sequence_variant,intron_variant
rs1257681798	genic_upstream_transcript_variant,intron_variant
rs1257719210	genic_upstream_transcript_variant,intron_variant
rs1257724789	genic_upstream_transcript_variant,intron_variant
rs1257799607	intron_variant
rs1257811340	intron_variant
rs1257823647	intron_variant
rs1257844148	intron_variant,2KB_upstream_variant,genic_upstream_transcript_variant,upstream_transcript_variant
rs1257852670	genic_upstream_transcript_variant,intron_variant
rs1257853630	genic_downstream_transcript_variant,intron_variant
rs1257857935	genic_downstream_transcript_variant,intron_variant
rs1257889335	genic_upstream_transcript_variant,intron_variant
rs1257915454	intron_variant
rs1257935758	intron_variant
rs1257959133	genic_upstream_transcript_variant,intron_variant
rs1257997212	intron_variant
rs1258031674	genic_upstream_transcript_variant,intron_variant
rs1258066110	genic_upstream_transcript_variant,intron_variant
rs1258088041	non_coding_transcript_variant,missense_variant,coding_sequence_variant
rs1258119991	genic_downstream_transcript_variant,intron_variant
rs1258140541	genic_upstream_transcript_variant,intron_variant
rs1258141410	intron_variant
rs1258162164	genic_upstream_transcript_variant,intron_variant
rs1258181216	intron_variant
rs1258207877	genic_downstream_transcript_variant,intron_variant
rs1258207909	genic_downstream_transcript_variant,intron_variant
rs1258223955	genic_upstream_transcript_variant,intron_variant
rs1258284337	genic_upstream_transcript_variant,intron_variant
rs1258303518	intron_variant

rs1258329043	intron_variant
rs1258398427	genic_upstream_transcript_variant,intron_variant
rs1258411721	genic_upstream_transcript_variant,intron_variant
rs1258454596	intron_variant
rs1258470863	intron_variant,2KB_upstream_variant,genic_upstream_transcript_variant,upstream_transcript_variant
rs1258497687	intron_variant
rs1258543962	genic_downstream_transcript_variant,intron_variant
rs1258549372	intron_variant
rs1258618103	genic_downstream_transcript_variant,intron_variant
rs1258621300	intron_variant
rs1258628318	genic_upstream_transcript_variant,intron_variant
rs1258733340	intron_variant,genic_upstream_transcript_variant
rs1258752950	intron_variant,genic_downstream_transcript_variant
rs1258771719	intron_variant,genic_upstream_transcript_variant,upstream_transcript_variant
rs1258840872	intron_variant
rs1258842336	intron_variant,genic_upstream_transcript_variant
rs1258869980	intron_variant,genic_upstream_transcript_variant
rs1258923143	intron_variant,genic_downstream_transcript_variant
rs1258957308	intron_variant
rs1258958446	2KB_upstream_variant,genic_upstream_transcript_variant,intron_variant,upstream_transcript_variant
rs1258984144	intron_variant
rs1259009141	intron_variant,genic_upstream_transcript_variant
rs1259013135	intron_variant,genic_upstream_transcript_variant
rs1259022775	intron_variant,genic_downstream_transcript_variant
rs1259038686	intron_variant,genic_upstream_transcript_variant
rs1259058383	intron_variant
rs1259076595	5_prime_UTR_variant,intron_variant
rs1259168791	intron_variant,genic_upstream_transcript_variant
rs1259204167	intron_variant,genic_upstream_transcript_variant
rs1259220607	intron_variant
rs1259247533	2KB_upstream_variant,intron_variant,upstream_transcript_variant,genic_upstream_transcript_variant
rs1259266907	intron_variant
rs1259294863	intron_variant,genic_upstream_transcript_variant
rs1259318185	intron_variant,genic_upstream_transcript_variant
rs1259377256	intron_variant,genic_upstream_transcript_variant
rs1259422583	intron_variant,genic_upstream_transcript_variant
rs1259423757	intron_variant
rs1259469540	intron_variant,genic_upstream_transcript_variant
rs1259496902	intron_variant,genic_upstream_transcript_variant
rs1259523949	intron_variant,genic_upstream_transcript_variant
rs1259524001	intron_variant,genic_downstream_transcript_variant
rs1259528093	intron_variant
rs1259567971	intron_variant,genic_upstream_transcript_variant
rs1259595147	intron_variant
rs1259607542	intron_variant,genic_downstream_transcript_variant
rs1259647785	intron_variant,genic_downstream_transcript_variant
rs1259672070	intron_variant,genic_upstream_transcript_variant
rs1259699848	intron_variant,genic_upstream_transcript_variant
rs1259709770	intron_variant,genic_downstream_transcript_variant
rs1259747556	intron_variant
rs1259762463	intron_variant,genic_upstream_transcript_variant
rs1259779984	intron_variant,genic_upstream_transcript_variant
rs1259816980	intron_variant,genic_upstream_transcript_variant
rs1259824078	intron_variant,genic_upstream_transcript_variant
rs1259833437	intron_variant
rs1259941083	intron_variant
rs1259946939	intron_variant,genic_upstream_transcript_variant
rs1259949645	intron_variant,genic_downstream_transcript_variant
rs1259950066	non_coding_transcript_variant,3_prime_UTR_variant,genic_downstream_transcript_variant
rs1259956013	intron_variant,genic_upstream_transcript_variant
rs1259985584	non_coding_transcript_variant,3_prime_UTR_variant,genic_downstream_transcript_variant
rs1260001540	intron_variant,genic_upstream_transcript_variant,coding_sequence_variant,non_coding_transcript_variant,missense_variant
rs1260007248	intron_variant,genic_upstream_transcript_variant
rs1260038194	intron_variant,genic_upstream_transcript_variant
rs1260070533	intron_variant,genic_upstream_transcript_variant
rs1260104319	intron_variant,genic_downstream_transcript_variant
rs1260124092	intron_variant,genic_upstream_transcript_variant
rs1260159912	2KB_upstream_variant,genic_upstream_transcript_variant,intron_variant,upstream_transcript_variant
rs1260208879	intron_variant,genic_downstream_transcript_variant
rs1260243834	intron_variant
rs1260312711	intron_variant,genic_upstream_transcript_variant
rs1260325129	intron_variant,genic_downstream_transcript_variant
rs1260339214	non_coding_transcript_variant,3_prime_UTR_variant,genic_downstream_transcript_variant
rs1260416556	downstream_transcript_variant,500B_downstream_variant

rs1260440133	intron_variant
rs1260484288	intron_variant
rs1260495798	intron_variant,genic_downstream_transcript_variant
rs1260495920	intron_variant
rs1260507122	intron_variant,genic_downstream_transcript_variant
rs1260519586	intron_variant,genic_downstream_transcript_variant
rs1260566494	intron_variant
rs1260566934	2KB_upstream_variant,genic_upstream_transcript_variant,intron_variant,upstream_transcript_variant
rs1260607781	intron_variant,genic_upstream_transcript_variant
rs1260611824	intron_variant,genic_upstream_transcript_variant
rs1260659719	intron_variant,genic_downstream_transcript_variant
rs1260660345	intron_variant
rs1260673035	intron_variant
rs1260794052	intron_variant,genic_downstream_transcript_variant
rs1260846553	intron_variant
rs1260848441	intron_variant,genic_upstream_transcript_variant
rs1260856431	downstream_transcript_variant,500B_downstream_variant
rs1260859495	intron_variant
rs1260914298	intron_variant,genic_downstream_transcript_variant
rs1261027459	intron_variant,genic_upstream_transcript_variant
rs1261051921	intron_variant
rs1261079833	non_coding_transcript_variant,3_prime_UTR_variant,genic_downstream_transcript_variant
rs1261110624	intron_variant,genic_upstream_transcript_variant
rs1261119813	intron_variant
rs1261142067	intron_variant
rs1261169204	intron_variant
rs1261183013	intron_variant,genic_upstream_transcript_variant
rs1261233119	intron_variant,genic_downstream_transcript_variant
rs1261258771	intron_variant
rs1261268865	intron_variant
rs1261381978	intron_variant,genic_downstream_transcript_variant
rs1261456064	intron_variant,genic_downstream_transcript_variant
rs1261466999	intron_variant
rs1261468112	intron_variant,genic_upstream_transcript_variant
rs1261479001	intron_variant,genic_upstream_transcript_variant
rs1261479123	intron_variant,genic_downstream_transcript_variant
rs1261537196	intron_variant,genic_upstream_transcript_variant
rs1261567748	intron_variant,genic_upstream_transcript_variant
rs1261613474	intron_variant,genic_downstream_transcript_variant
rs1261620956	intron_variant,genic_downstream_transcript_variant
rs1261632170	intron_variant,genic_upstream_transcript_variant
rs1261636019	intron_variant,genic_upstream_transcript_variant
rs1261685417	intron_variant,genic_upstream_transcript_variant
rs1261704425	intron_variant
rs1261704732	intron_variant,genic_upstream_transcript_variant,upstream_transcript_variant
rs1261761288	intron_variant,genic_upstream_transcript_variant
rs1261871479	intron_variant,genic_downstream_transcript_variant
rs1261881230	intron_variant,genic_upstream_transcript_variant
rs1261907989	intron_variant,genic_downstream_transcript_variant
rs1261951023	intron_variant,genic_downstream_transcript_variant
rs1262084868	intron_variant
rs1262121210	intron_variant
rs1262129956	intron_variant,genic_upstream_transcript_variant
rs1262139394	intron_variant,genic_upstream_transcript_variant,upstream_transcript_variant
rs1262151508	intron_variant
rs1262195411	intron_variant
rs1262201349	intron_variant,genic_upstream_transcript_variant
rs1262206087	intron_variant
rs1262213420	intron_variant,genic_upstream_transcript_variant
rs1262271583	intron_variant,genic_upstream_transcript_variant
rs1262279105	intron_variant
rs1262283132	intron_variant
rs1262285676	intron_variant
rs1262303556	2KB_upstream_variant,genic_upstream_transcript_variant,intron_variant,upstream_transcript_variant
rs1262304409	intron_variant,genic_upstream_transcript_variant
rs1262362922	intron_variant
rs1262363010	intron_variant,genic_downstream_transcript_variant
rs1262369585	intron_variant,genic_upstream_transcript_variant
rs1262373900	intron_variant,genic_upstream_transcript_variant
rs1262462179	intron_variant,genic_downstream_transcript_variant
rs1262483970	intron_variant
rs1262486155	intron_variant
rs1262496580	intron_variant,genic_upstream_transcript_variant
rs1262523341	intron_variant,genic_upstream_transcript_variant

rs1262567390	intron_variant
rs1262626879	intron_variant
rs1262648433	intron_variant,genic_upstream_transcript_variant
rs1262663317	intron_variant
rs1262685478	2KB_upstream_variant,intron_variant,upstream_transcript_variant,genic_upstream_transcript_variant
rs1262707583	intron_variant
rs1262725876	intron_variant,genic_upstream_transcript_variant
rs1262743711	intron_variant,genic_downstream_transcript_variant
rs1262768593	non_coding_transcript_variant,synonymous_variant,coding_sequence_variant,genic_downstream_transcript_variant
rs1262818212	intron_variant
rs1262879559	intron_variant,genic_upstream_transcript_variant
rs1262897461	intron_variant,genic_upstream_transcript_variant
rs1262910300	2KB_upstream_variant,intron_variant,upstream_transcript_variant,genic_upstream_transcript_variant
rs1262919853	intron_variant
rs1262925397	intron_variant
rs1262935003	intron_variant
rs1262960080	intron_variant
rs1262980117	intron_variant,genic_upstream_transcript_variant
rs1262993424	intron_variant,genic_upstream_transcript_variant
rs1263013047	intron_variant
rs1263050872	non_coding_transcript_variant,5_prime_UTR_variant,genic_upstream_transcript_variant,intron_variant
rs1263051771	intron_variant
rs1263202629	intron_variant,genic_upstream_transcript_variant
rs1263212131	intron_variant
rs1263225671	intron_variant
rs1263251532	intron_variant,genic_upstream_transcript_variant,upstream_transcript_variant
rs1263357401	intron_variant,genic_upstream_transcript_variant,upstream_transcript_variant
rs1263404612	intron_variant,genic_downstream_transcript_variant
rs1263420949	intron_variant
rs1263428214	intron_variant
rs1263448012	intron_variant
rs1263485653	intron_variant,genic_upstream_transcript_variant
rs1263506226	downstream_transcript_variant,500B_downstream_variant
rs1263552463	intron_variant
rs1263573717	intron_variant,genic_upstream_transcript_variant
rs1263584679	intron_variant,genic_upstream_transcript_variant
rs1263588153	intron_variant,genic_downstream_transcript_variant
rs1263619196	intron_variant
rs1263628356	intron_variant,genic_upstream_transcript_variant
rs1263668056	intron_variant,genic_upstream_transcript_variant
rs1263686802	intron_variant
rs1263693625	intron_variant,genic_upstream_transcript_variant
rs1263694313	intron_variant,genic_downstream_transcript_variant
rs1263697629	intron_variant,genic_upstream_transcript_variant
rs1263702747	intron_variant,genic_upstream_transcript_variant
rs1263711748	intron_variant,genic_upstream_transcript_variant
rs1263760186	intron_variant,genic_upstream_transcript_variant
rs1263766148	intron_variant
rs1263769989	intron_variant,genic_upstream_transcript_variant
rs1263780898	intron_variant
rs1263817135	intron_variant,genic_downstream_transcript_variant
rs1263833705	intron_variant
rs1263855745	intron_variant,genic_upstream_transcript_variant
rs1263855843	intron_variant
rs1263857667	intron_variant,genic_upstream_transcript_variant
rs1263864839	intron_variant
rs1263869350	intron_variant
rs1263913910	intron_variant,genic_downstream_transcript_variant
rs1263924065	intron_variant,genic_upstream_transcript_variant
rs1263935801	intron_variant
rs1264001884	intron_variant
rs1264006612	intron_variant
rs1264014006	intron_variant,genic_upstream_transcript_variant
rs1264020929	intron_variant
rs1264044255	intron_variant,genic_downstream_transcript_variant
rs1264071222	intron_variant,genic_downstream_transcript_variant
rs1264113576	intron_variant
rs1264139877	intron_variant,genic_upstream_transcript_variant
rs1264146174	2KB_upstream_variant,genic_upstream_transcript_variant,intron_variant,upstream_transcript_variant
rs1264247778	2KB_upstream_variant,intron_variant,upstream_transcript_variant,genic_upstream_transcript_variant
rs1264264681	intron_variant
rs1264283247	intron_variant,genic_upstream_transcript_variant
rs1264329868	intron_variant,genic_upstream_transcript_variant
rs1264345366	intron_variant

rs1264371589	intron_variant,genic_upstream_transcript_variant
rs1264418745	intron_variant,genic_downstream_transcript_variant
rs1264467440	intron_variant
rs1264492455	2KB_upstream_variant,genic_upstream_transcript_variant,intron_variant,upstream_transcript_variant
rs1264492986	intron_variant,genic_upstream_transcript_variant,upstream_transcript_variant
rs1264523457	intron_variant
rs1264531949	intron_variant
rs1264571435	intron_variant
rs1264609812	intron_variant,genic_upstream_transcript_variant
rs1264616517	intron_variant
rs1264706400	intron_variant
rs1264706454	non_coding_transcript_variant,3_prime_UTR_variant,genic_downstream_transcript_variant
rs1264726211	intron_variant
rs1264739065	intron_variant,genic_upstream_transcript_variant
rs1264756387	intron_variant,genic_upstream_transcript_variant
rs1264776318	intron_variant,genic_upstream_transcript_variant
rs1264798179	intron_variant
rs1264844178	intron_variant,genic_upstream_transcript_variant
rs1264899368	intron_variant,genic_upstream_transcript_variant
rs1264955778	intron_variant,genic_downstream_transcript_variant
rs1264989567	intron_variant,genic_downstream_transcript_variant
rs1265041111	intron_variant,genic_downstream_transcript_variant
rs1265068248	intron_variant,genic_downstream_transcript_variant
rs1265071852	intron_variant,genic_upstream_transcript_variant
rs1265111752	intron_variant
rs1265120061	intron_variant,genic_downstream_transcript_variant
rs1265133807	intron_variant,genic_upstream_transcript_variant
rs1265160652	intron_variant,genic_downstream_transcript_variant
rs1265174847	intron_variant
rs1265177629	intron_variant,genic_upstream_transcript_variant,upstream_transcript_variant
rs1265277471	intron_variant,genic_upstream_transcript_variant
rs1265281832	intron_variant,genic_upstream_transcript_variant
rs1265295558	intron_variant,genic_downstream_transcript_variant
rs1265387204	intron_variant,genic_upstream_transcript_variant
rs1265389228	intron_variant
rs1265410295	intron_variant,genic_upstream_transcript_variant
rs1265448922	intron_variant,genic_downstream_transcript_variant
rs1265456287	intron_variant
rs1265466188	intron_variant,genic_upstream_transcript_variant
rs1265502933	intron_variant
rs1265506197	intron_variant,genic_downstream_transcript_variant
rs1265523956	intron_variant
rs1265589397	intron_variant,genic_upstream_transcript_variant
rs1265596965	intron_variant
rs1265671115	intron_variant,genic_upstream_transcript_variant,upstream_transcript_variant
rs1265677027	intron_variant
rs1265719293	intron_variant,genic_upstream_transcript_variant
rs1265733679	intron_variant
rs1265763417	intron_variant,genic_downstream_transcript_variant
rs1265810319	non_coding_transcript_variant,5_prime_UTR_variant,intron_variant,genic_upstream_transcript_variant
rs1265813578	intron_variant
rs1265898838	intron_variant
rs1265898927	intron_variant,genic_upstream_transcript_variant
rs1265911623	intron_variant
rs1265915068	intron_variant,genic_upstream_transcript_variant
rs1265925864	intron_variant
rs1265928842	intron_variant
rs1265966589	intron_variant,genic_upstream_transcript_variant
rs1265997685	intron_variant,genic_upstream_transcript_variant
rs1266064482	intron_variant,genic_downstream_transcript_variant
rs1266082683	intron_variant,genic_downstream_transcript_variant
rs1266134740	intron_variant,genic_upstream_transcript_variant
rs1266141030	intron_variant
rs1266157358	intron_variant,genic_upstream_transcript_variant
rs1266208625	intron_variant
rs1266212951	intron_variant,genic_upstream_transcript_variant
rs1266234864	intron_variant
rs1266240357	intron_variant,genic_upstream_transcript_variant
rs1266319879	intron_variant,coding_sequence_variant,synonymous_variant,non_coding_transcript_variant,genic_upstream_transcript_variant
rs1266366684	intron_variant,genic_downstream_transcript_variant
rs1266379680	intron_variant,genic_upstream_transcript_variant
rs1266394693	intron_variant,genic_upstream_transcript_variant,upstream_transcript_variant
rs1266415296	intron_variant,genic_upstream_transcript_variant
rs1266421569	intron_variant,genic_upstream_transcript_variant

rs1266425510	intron_variant,genic_upstream_transcript_variant
rs1266429947	intron_variant,genic_upstream_transcript_variant
rs1266472527	intron_variant,genic_downstream_transcript_variant
rs1266481010	intron_variant
rs1266531077	intron_variant
rs1266545007	intron_variant,genic_upstream_transcript_variant
rs1266561592	intron_variant,genic_downstream_transcript_variant
rs1266565086	intron_variant
rs1266567934	intron_variant
rs1266570238	intron_variant,genic_upstream_transcript_variant
rs1266570718	intron_variant,genic_upstream_transcript_variant
rs1266571099	intron_variant,genic_upstream_transcript_variant
rs1266590447	intron_variant
rs1266596960	intron_variant
rs1266663868	2KB_upstream_variant,intron_variant,upstream_transcript_variant,genic_upstream_transcript_variant
rs1266680120	intron_variant,genic_upstream_transcript_variant
rs1266741708	intron_variant,genic_upstream_transcript_variant
rs1266743285	intron_variant,genic_upstream_transcript_variant
rs1266763017	intron_variant
rs1266779693	intron_variant
rs1266790730	intron_variant,genic_downstream_transcript_variant
rs1266800457	intron_variant,genic_upstream_transcript_variant
rs1266810947	intron_variant,genic_upstream_transcript_variant
rs1266833135	2KB_upstream_variant,genic_upstream_transcript_variant,intron_variant,upstream_transcript_variant
rs1266867499	intron_variant,genic_upstream_transcript_variant
rs1266871309	intron_variant
rs1266967070	intron_variant,genic_downstream_transcript_variant
rs1266977012	intron_variant,genic_upstream_transcript_variant
rs1267041840	intron_variant,genic_upstream_transcript_variant
rs1267096603	intron_variant,genic_downstream_transcript_variant
rs1267096845	intron_variant,genic_upstream_transcript_variant
rs1267099111	2KB_upstream_variant,genic_upstream_transcript_variant,intron_variant,upstream_transcript_variant
rs1267104861	intron_variant
rs1267121730	intron_variant,genic_upstream_transcript_variant
rs1267171884	intron_variant,genic_upstream_transcript_variant
rs1267190613	downstream_transcript_variant,500B_downstream_variant
rs1267269082	intron_variant,genic_upstream_transcript_variant
rs1267276106	intron_variant,genic_downstream_transcript_variant
rs1267289866	intron_variant,genic_upstream_transcript_variant
rs1267341909	intron_variant,genic_upstream_transcript_variant
rs1267342507	intron_variant,genic_upstream_transcript_variant,upstream_transcript_variant
rs1267352914	intron_variant,genic_upstream_transcript_variant
rs1267363125	intron_variant,genic_upstream_transcript_variant
rs1267408813	intron_variant
rs1267419620	non_coding_transcript_variant,missense_variant,coding_sequence_variant,genic_downstream_transcript_variant
rs1267429223	intron_variant
rs1267457825	intron_variant
rs1267496294	intron_variant,genic_downstream_transcript_variant
rs1267521691	intron_variant,genic_upstream_transcript_variant
rs1267549275	intron_variant,genic_downstream_transcript_variant
rs1267554631	intron_variant,genic_upstream_transcript_variant,upstream_transcript_variant
rs1267597048	intron_variant
rs1267597200	non_coding_transcript_variant,3_prime_UTR_variant,genic_downstream_transcript_variant
rs1267602049	intron_variant,genic_upstream_transcript_variant,upstream_transcript_variant
rs1267624824	intron_variant,genic_upstream_transcript_variant
rs1267631828	intron_variant,genic_downstream_transcript_variant
rs1267649933	intron_variant,genic_upstream_transcript_variant
rs1267698626	intron_variant
rs1267805730	intron_variant,genic_downstream_transcript_variant
rs1267911481	intron_variant
rs1267924842	non_coding_transcript_variant,3_prime_UTR_variant,genic_downstream_transcript_variant
rs1267937498	intron_variant,genic_upstream_transcript_variant
rs1267947909	intron_variant
rs1267952693	intron_variant,genic_downstream_transcript_variant
rs1267955231	intron_variant,genic_downstream_transcript_variant
rs1267969037	intron_variant
rs1267999665	intron_variant,genic_upstream_transcript_variant
rs1268007339	intron_variant,genic_upstream_transcript_variant
rs1268014059	intron_variant,genic_downstream_transcript_variant
rs1268014365	intron_variant,genic_downstream_transcript_variant
rs1268030382	2KB_upstream_variant,genic_upstream_transcript_variant,intron_variant,upstream_transcript_variant
rs1268037087	intron_variant,genic_downstream_transcript_variant
rs1268044324	intron_variant,genic_upstream_transcript_variant
rs1268054380	intron_variant

rs1268077800	intron_variant
rs1268093312	intron_variant
rs1268119349	intron_variant,genic_upstream_transcript_variant
rs1268186645	intron_variant,genic_upstream_transcript_variant
rs1268187927	intron_variant
rs1268280839	intron_variant,downstream_transcript_variant,genic_downstream_transcript_variant
rs1268283013	intron_variant,genic_downstream_transcript_variant
rs1268301263	intron_variant
rs1268314646	intron_variant
rs1268316680	intron_variant
rs1268322924	intron_variant
rs1268397494	intron_variant,genic_downstream_transcript_variant
rs1268409776	intron_variant,genic_upstream_transcript_variant
rs1268414692	2KB_upstream_variant,genic_upstream_transcript_variant,intron_variant,upstream_transcript_variant
rs1268448540	intron_variant,genic_upstream_transcript_variant
rs1268460422	intron_variant,genic_upstream_transcript_variant
rs1268477184	intron_variant,genic_upstream_transcript_variant
rs1268491538	intron_variant,genic_downstream_transcript_variant
rs1268500087	intron_variant,genic_upstream_transcript_variant
rs1268518192	intron_variant,genic_upstream_transcript_variant
rs1268539188	intron_variant,genic_upstream_transcript_variant
rs1268543138	intron_variant,genic_upstream_transcript_variant
rs1268551338	intron_variant
rs1268567717	intron_variant,genic_upstream_transcript_variant,upstream_transcript_variant
rs1268568333	intron_variant
rs1268580953	intron_variant
rs1268589933	intron_variant,genic_upstream_transcript_variant
rs1268611842	intron_variant
rs1268642668	intron_variant,genic_downstream_transcript_variant
rs1268674634	intron_variant,genic_downstream_transcript_variant
rs1268692132	intron_variant,genic_upstream_transcript_variant,upstream_transcript_variant
rs1268711657	intron_variant
rs1268718333	intron_variant,genic_upstream_transcript_variant,upstream_transcript_variant
rs1268724874	5_prime_UTR_variant,intron_variant
rs1268753458	intron_variant
rs1268773094	intron_variant
rs1268792821	intron_variant,genic_upstream_transcript_variant
rs1268823581	intron_variant,genic_upstream_transcript_variant
rs1268849644	intron_variant
rs1268875887	intron_variant,genic_downstream_transcript_variant
rs1268890696	intron_variant
rs1268920478	intron_variant,genic_upstream_transcript_variant
rs1268940780	intron_variant
rs1268947712	intron_variant,genic_upstream_transcript_variant
rs1268971643	intron_variant,genic_downstream_transcript_variant
rs1269007797	intron_variant,genic_upstream_transcript_variant
rs1269083785	intron_variant
rs1269084226	intron_variant,genic_upstream_transcript_variant
rs1269149486	intron_variant,genic_upstream_transcript_variant
rs1269191859	intron_variant,coding_sequence_variant,synonymous_variant,non_coding_transcript_variant,genic_upstream_transcript_variant
rs1269248713	intron_variant,genic_upstream_transcript_variant
rs1269319758	intron_variant
rs1269328602	intron_variant,genic_upstream_transcript_variant
rs1269391733	intron_variant,genic_downstream_transcript_variant
rs1269394190	intron_variant,genic_upstream_transcript_variant
rs1269411388	intron_variant
rs1269420897	intron_variant,genic_upstream_transcript_variant
rs1269444000	2KB_upstream_variant,intron_variant,upstream_transcript_variant,genic_upstream_transcript_variant
rs1269459319	intron_variant,genic_upstream_transcript_variant
rs1269460192	intron_variant,genic_upstream_transcript_variant
rs1269475572	intron_variant,genic_upstream_transcript_variant
rs1269529639	intron_variant
rs1269535609	intron_variant,genic_upstream_transcript_variant
rs1269549520	intron_variant,genic_upstream_transcript_variant
rs1269574918	intron_variant,genic_upstream_transcript_variant
rs1269588659	intron_variant,genic_downstream_transcript_variant
rs1269602807	non_coding_transcript_variant,3_prime_UTR_variant,genic_downstream_transcript_variant
rs1269621250	intron_variant,genic_downstream_transcript_variant
rs1269657341	intron_variant
rs1269659047	intron_variant
rs1269706080	intron_variant
rs1269730743	intron_variant
rs1269743926	intron_variant
rs1269775940	intron_variant,genic_downstream_transcript_variant

rs1269814098	non_coding_transcript_variant,3_prime_UTR_variant,genic_downstream_transcript_variant
rs1269864381	intron_variant,genic_upstream_transcript_variant
rs1269915986	intron_variant,genic_upstream_transcript_variant
rs1269924078	intron_variant,genic_downstream_transcript_variant
rs1269995706	intron_variant,genic_upstream_transcript_variant
rs1270015773	intron_variant,genic_downstream_transcript_variant
rs1270111196	intron_variant,genic_upstream_transcript_variant
rs1270140640	intron_variant,genic_upstream_transcript_variant,upstream_transcript_variant
rs1270202780	intron_variant
rs1270203029	intron_variant
rs1270211245	intron_variant,genic_upstream_transcript_variant
rs1270211422	intron_variant,genic_upstream_transcript_variant
rs1270213566	intron_variant,genic_downstream_transcript_variant
rs1270260986	intron_variant
rs1270283992	intron_variant
rs1270332386	non_coding_transcript_variant,3_prime_UTR_variant,genic_downstream_transcript_variant
rs1270343851	intron_variant,genic_upstream_transcript_variant
rs1270347996	intron_variant,genic_downstream_transcript_variant
rs1270383335	intron_variant
rs1270383848	intron_variant,genic_upstream_transcript_variant
rs1270397095	intron_variant,genic_downstream_transcript_variant
rs1270409168	intron_variant,genic_downstream_transcript_variant
rs1270434765	intron_variant,genic_downstream_transcript_variant
rs1270488345	intron_variant,genic_downstream_transcript_variant
rs1270491965	intron_variant
rs1270493821	intron_variant,genic_upstream_transcript_variant,upstream_transcript_variant
rs1270496136	intron_variant,genic_downstream_transcript_variant
rs1270587436	intron_variant,genic_upstream_transcript_variant
rs1270595949	intron_variant,genic_downstream_transcript_variant
rs1270597191	intron_variant,genic_upstream_transcript_variant
rs1270638173	intron_variant,genic_upstream_transcript_variant
rs1270681698	intron_variant
rs1270687423	intron_variant
rs1270700890	intron_variant,genic_upstream_transcript_variant,upstream_transcript_variant
rs1270724588	intron_variant
rs1270731720	intron_variant
rs1270763345	intron_variant
rs1270764547	intron_variant
rs1270801929	intron_variant,genic_upstream_transcript_variant
rs1270820631	intron_variant
rs1270820853	intron_variant
rs1270863054	intron_variant,genic_downstream_transcript_variant
rs1270879627	intron_variant
rs1270906901	intron_variant,genic_upstream_transcript_variant
rs1270960929	intron_variant,genic_upstream_transcript_variant
rs1270971153	intron_variant,genic_upstream_transcript_variant
rs1271023596	intron_variant,genic_upstream_transcript_variant
rs1271024528	5_prime_UTR_variant,intron_variant
rs1271067098	2KB_upstream_variant,genic_upstream_transcript_variant,intron_variant,upstream_transcript_variant
rs1271082117	intron_variant,genic_upstream_transcript_variant
rs1271095289	intron_variant,genic_upstream_transcript_variant
rs1271102748	intron_variant
rs1271176407	intron_variant,genic_upstream_transcript_variant
rs1271211591	intron_variant
rs1271223115	intron_variant
rs1271305036	intron_variant,genic_upstream_transcript_variant
rs1271322855	genic_downstream_transcript_variant,intron_variant
rs1271328491	genic_downstream_transcript_variant,intron_variant
rs1271382339	intron_variant,genic_upstream_transcript_variant
rs1271450346	genic_downstream_transcript_variant,intron_variant,downstream_transcript_variant
rs1271461975	genic_downstream_transcript_variant,intron_variant
rs1271476358	genic_downstream_transcript_variant,intron_variant
rs1271487393	intron_variant,genic_upstream_transcript_variant
rs1271507677	intron_variant,genic_upstream_transcript_variant
rs1271562395	intron_variant,genic_upstream_transcript_variant
rs1271566452	genic_downstream_transcript_variant,intron_variant
rs1271633342	intron_variant,genic_upstream_transcript_variant
rs1271645541	intron_variant
rs1271673163	intron_variant
rs1271697724	intron_variant
rs1271733182	intron_variant,genic_upstream_transcript_variant
rs1271748852	upstream_transcript_variant,intron_variant,genic_upstream_transcript_variant,2KB_upstream_variant
rs1271761738	genic_downstream_transcript_variant,intron_variant
rs1271778906	non_coding_transcript_variant,genic_downstream_transcript_variant,3_prime_UTR_variant

rs1271782836	genic_downstream_transcript_variant,intron_variant
rs1271865735	intron_variant
rs1271902332	intron_variant
rs1271905478	intron_variant,genic_upstream_transcript_variant
rs1271922364	intron_variant
rs1271932503	genic_downstream_transcript_variant,intron_variant
rs1271984909	intron_variant,genic_upstream_transcript_variant
rs1272057679	intron_variant,genic_upstream_transcript_variant
rs1272074290	genic_downstream_transcript_variant,intron_variant
rs1272117015	intron_variant
rs1272175558	intron_variant
rs1272186417	genic_downstream_transcript_variant,intron_variant
rs1272196499	intron_variant
rs1272278388	intron_variant,genic_upstream_transcript_variant
rs1272312513	genic_downstream_transcript_variant,intron_variant
rs1272320534	non_coding_transcript_variant,genic_downstream_transcript_variant,3_prime_UTR_variant
rs1272340467	genic_downstream_transcript_variant,intron_variant
rs1272396944	intron_variant,genic_upstream_transcript_variant
rs1272404368	non_coding_transcript_variant,genic_downstream_transcript_variant,3_prime_UTR_variant
rs1272420960	intron_variant
rs1272537718	intron_variant,genic_upstream_transcript_variant
rs1272557212	intron_variant
rs1272587756	intron_variant,genic_upstream_transcript_variant
rs1272592193	intron_variant
rs1272603873	intron_variant,genic_upstream_transcript_variant
rs1272617326	intron_variant
rs1272631805	intron_variant,genic_upstream_transcript_variant
rs1272696846	intron_variant,genic_upstream_transcript_variant
rs1272698128	intron_variant,genic_upstream_transcript_variant
rs1272722489	intron_variant,genic_upstream_transcript_variant
rs1272733884	intron_variant,genic_upstream_transcript_variant
rs1272734421	intron_variant
rs1272782141	intron_variant,genic_upstream_transcript_variant
rs1272823749	intron_variant,genic_upstream_transcript_variant
rs1272828855	intron_variant,genic_upstream_transcript_variant
rs1272850760	genic_downstream_transcript_variant,intron_variant
rs1272868492	upstream_transcript_variant,intron_variant,genic_upstream_transcript_variant
rs1272870635	intron_variant,genic_upstream_transcript_variant
rs1272899215	genic_downstream_transcript_variant,intron_variant
rs1272918935	intron_variant,genic_upstream_transcript_variant
rs1272929122	intron_variant
rs1272933112	intron_variant,genic_upstream_transcript_variant
rs1272945226	non_coding_transcript_variant,intron_variant,genic_upstream_transcript_variant
rs1272979769	upstream_transcript_variant,intron_variant,genic_upstream_transcript_variant,2KB_upstream_variant
rs1273052250	intron_variant
rs1273056732	intron_variant,genic_upstream_transcript_variant
rs1273062258	upstream_transcript_variant,intron_variant,genic_upstream_transcript_variant,2KB_upstream_variant
rs1273084503	intron_variant,genic_upstream_transcript_variant
rs1273084687	intron_variant,genic_upstream_transcript_variant
rs1273114533	non_coding_transcript_variant,genic_downstream_transcript_variant,3_prime_UTR_variant
rs1273178275	intron_variant,genic_upstream_transcript_variant
rs1273199339	intron_variant
rs1273223489	intron_variant,genic_upstream_transcript_variant
rs1273314690	intron_variant,genic_upstream_transcript_variant
rs1273316276	intron_variant
rs1273353680	genic_downstream_transcript_variant,intron_variant
rs1273358157	intron_variant,genic_upstream_transcript_variant
rs1273370956	intron_variant
rs1273388859	intron_variant,genic_upstream_transcript_variant
rs1273414936	intron_variant
rs1273435114	genic_downstream_transcript_variant,intron_variant
rs1273442882	intron_variant
rs1273457063	intron_variant
rs1273460822	intron_variant,genic_upstream_transcript_variant
rs1273464579	intron_variant
rs1273474181	intron_variant
rs1273474248	genic_downstream_transcript_variant,intron_variant
rs1273483201	upstream_transcript_variant,2KB_upstream_variant,intron_variant,genic_upstream_transcript_variant
rs1273495779	intron_variant,genic_upstream_transcript_variant
rs1273505543	intron_variant,genic_upstream_transcript_variant
rs1273535888	intron_variant
rs1273580423	intron_variant,genic_upstream_transcript_variant
rs1273724771	intron_variant,genic_upstream_transcript_variant
rs1273727722	genic_downstream_transcript_variant,intron_variant

rs1273733576	intron_variant
rs1273739875	intron_variant
rs1273751798	intron_variant,genic_upstream_transcript_variant
rs1273752363	intron_variant,genic_upstream_transcript_variant
rs1273784620	intron_variant,genic_upstream_transcript_variant
rs1273810906	intron_variant
rs1273819384	intron_variant,genic_upstream_transcript_variant
rs1273868095	intron_variant
rs1273879606	genic_downstream_transcript_variant,intron_variant
rs1273926620	500B_downstream_variant,downstream_transcript_variant
rs1273970296	intron_variant,genic_upstream_transcript_variant
rs1273988011	missense_variant,non_coding_transcript_variant,coding_sequence_variant,intron_variant,genic_upstream_transcript_variant
rs1273997256	intron_variant,genic_upstream_transcript_variant
rs1274000718	genic_downstream_transcript_variant,intron_variant
rs1274027255	genic_downstream_transcript_variant,intron_variant
rs1274043788	intron_variant
rs1274081296	intron_variant,genic_upstream_transcript_variant
rs1274090937	5_prime_UTR_variant,intron_variant
rs1274120505	intron_variant
rs1274123833	intron_variant,genic_upstream_transcript_variant
rs1274143620	genic_downstream_transcript_variant,intron_variant
rs1274163180	upstream_transcript_variant,intron_variant,genic_upstream_transcript_variant
rs1274178540	intron_variant,genic_upstream_transcript_variant
rs1274214074	intron_variant
rs1274239740	genic_downstream_transcript_variant,intron_variant
rs1274295488	intron_variant,genic_upstream_transcript_variant
rs1274338412	intron_variant
rs1274339810	intron_variant,genic_upstream_transcript_variant
rs1274362652	intron_variant
rs1274377295	intron_variant
rs1274386081	intron_variant
rs1274471462	intron_variant,genic_upstream_transcript_variant
rs1274479297	intron_variant
rs1274496968	intron_variant,genic_upstream_transcript_variant
rs1274536123	intron_variant,genic_upstream_transcript_variant
rs1274547369	intron_variant,genic_upstream_transcript_variant
rs1274568469	intron_variant,genic_upstream_transcript_variant
rs1274587254	intron_variant
rs1274600374	genic_downstream_transcript_variant,intron_variant
rs1274644399	intron_variant
rs1274645069	intron_variant
rs1274675873	intron_variant
rs1274714567	genic_downstream_transcript_variant,intron_variant
rs1274719337	genic_downstream_transcript_variant,intron_variant
rs1274741509	genic_downstream_transcript_variant,intron_variant
rs1274769203	intron_variant,genic_upstream_transcript_variant
rs1274773656	intron_variant
rs1274827408	intron_variant,genic_upstream_transcript_variant
rs1274834695	intron_variant,genic_upstream_transcript_variant
rs1274873645	intron_variant
rs1274950946	intron_variant,genic_upstream_transcript_variant
rs1275001923	intron_variant,genic_upstream_transcript_variant
rs1275017631	intron_variant,genic_upstream_transcript_variant
rs1275029173	genic_downstream_transcript_variant,intron_variant
rs1275087522	missense_variant,non_coding_transcript_variant,coding_sequence_variant,intron_variant,genic_upstream_transcript_variant
rs1275124807	non_coding_transcript_variant,missense_variant,genic_downstream_transcript_variant,coding_sequence_variant
rs1275152897	genic_downstream_transcript_variant,intron_variant
rs1275186748	intron_variant,genic_upstream_transcript_variant
rs1275251026	intron_variant,genic_upstream_transcript_variant
rs1275314985	intron_variant,genic_upstream_transcript_variant
rs1275328045	intron_variant
rs1275357431	non_coding_transcript_variant,genic_downstream_transcript_variant,3_prime_UTR_variant
rs1275385094	genic_downstream_transcript_variant,intron_variant
rs1275486227	genic_downstream_transcript_variant,intron_variant
rs1275517445	intron_variant,genic_upstream_transcript_variant
rs1275596895	upstream_transcript_variant,intron_variant,genic_upstream_transcript_variant
rs1275609972	intron_variant,genic_upstream_transcript_variant
rs1275647547	intron_variant
rs1275651632	intron_variant
rs1275654333	genic_downstream_transcript_variant,intron_variant
rs1275661752	synonymous_variant,non_coding_transcript_variant,coding_sequence_variant,intron_variant,genic_upstream_transcript_variant
rs1275718228	intron_variant
rs1275723601	intron_variant
rs1275725329	intron_variant,genic_upstream_transcript_variant

rs1275754018	intron_variant,genic_upstream_transcript_variant
rs1275781723	genic_downstream_transcript_variant,intron_variant
rs1275809659	intron_variant,genic_upstream_transcript_variant
rs1275893989	intron_variant,genic_upstream_transcript_variant
rs1275907943	intron_variant
rs1275911637	intron_variant,genic_upstream_transcript_variant
rs1275914492	intron_variant,genic_upstream_transcript_variant
rs1275978331	intron_variant,genic_upstream_transcript_variant
rs1276035998	intron_variant
rs1276037404	genic_downstream_transcript_variant,intron_variant
rs1276108526	intron_variant,genic_upstream_transcript_variant
rs1276157296	intron_variant,genic_upstream_transcript_variant
rs1276203604	intron_variant
rs1276215470	intron_variant
rs1276221106	upstream_transcript_variant,intron_variant,genic_upstream_transcript_variant
rs1276267801	intron_variant
rs1276294981	intron_variant
rs1276324192	intron_variant,genic_upstream_transcript_variant
rs1276351734	upstream_transcript_variant,intron_variant,genic_upstream_transcript_variant,2KB_upstream_variant
rs1276397391	intron_variant
rs1276415135	intron_variant,genic_upstream_transcript_variant
rs1276446010	intron_variant
rs1276489401	intron_variant,genic_upstream_transcript_variant
rs1276511153	intron_variant
rs1276512434	intron_variant,genic_upstream_transcript_variant
rs1276523357	intron_variant
rs1276564264	intron_variant
rs1276610201	intron_variant,genic_upstream_transcript_variant
rs1276638136	intron_variant,genic_upstream_transcript_variant
rs1276638458	non_coding_transcript_variant,genic_downstream_transcript_variant,3_prime_UTR_variant
rs1276658520	intron_variant,genic_upstream_transcript_variant
rs1276700722	intron_variant
rs1276739865	intron_variant
rs1276765917	intron_variant
rs1276779762	intron_variant,genic_upstream_transcript_variant
rs1276815349	intron_variant
rs1276841913	500B_downstream_variant,downstream_transcript_variant
rs1276846232	intron_variant
rs1276852337	intron_variant
rs1276896980	intron_variant
rs1276930490	upstream_transcript_variant,2KB_upstream_variant,intron_variant,genic_upstream_transcript_variant
rs1276959222	intron_variant,genic_upstream_transcript_variant
rs1276965726	intron_variant
rs1276992647	intron_variant,genic_upstream_transcript_variant
rs1277001581	intron_variant
rs1277010675	upstream_transcript_variant,2KB_upstream_variant,intron_variant,genic_upstream_transcript_variant
rs1277023162	intron_variant
rs1277039666	intron_variant
rs1277052613	upstream_transcript_variant,intron_variant,genic_upstream_transcript_variant
rs1277060801	upstream_transcript_variant,intron_variant,genic_upstream_transcript_variant
rs1277067723	intron_variant,genic_upstream_transcript_variant
rs1277078629	intron_variant,genic_upstream_transcript_variant
rs1277104433	genic_downstream_transcript_variant,intron_variant
rs1277111038	intron_variant,genic_upstream_transcript_variant
rs1277119952	intron_variant,genic_upstream_transcript_variant
rs1277143361	genic_downstream_transcript_variant,intron_variant
rs1277144210	upstream_transcript_variant,intron_variant,genic_upstream_transcript_variant
rs1277153504	genic_downstream_transcript_variant,intron_variant
rs1277209546	intron_variant,genic_upstream_transcript_variant
rs1277209638	genic_downstream_transcript_variant,intron_variant
rs1277226606	intron_variant
rs1277269145	intron_variant
rs1277293299	genic_downstream_transcript_variant,intron_variant
rs1277297003	intron_variant
rs1277298967	intron_variant
rs1277331731	upstream_transcript_variant,intron_variant,genic_upstream_transcript_variant,2KB_upstream_variant
rs1277365079	genic_downstream_transcript_variant,intron_variant
rs1277377886	intron_variant,genic_upstream_transcript_variant
rs1277390214	upstream_transcript_variant,intron_variant,genic_upstream_transcript_variant,2KB_upstream_variant
rs1277411587	genic_downstream_transcript_variant,intron_variant
rs1277417440	intron_variant
rs1277427459	genic_downstream_transcript_variant,intron_variant
rs1277442958	intron_variant,genic_upstream_transcript_variant
rs1277462520	intron_variant

rs1277472557	intron_variant
rs1277491107	genic_downstream_transcript_variant,intron_variant
rs1277522171	intron_variant,genic_upstream_transcript_variant
rs1277559477	intron_variant
rs1277579784	intron_variant,genic_upstream_transcript_variant
rs1277599833	genic_downstream_transcript_variant,intron_variant
rs1277612411	genic_downstream_transcript_variant,intron_variant
rs1277649454	genic_downstream_transcript_variant,intron_variant
rs1277651598	intron_variant
rs1277656752	intron_variant,genic_upstream_transcript_variant
rs1277720860	intron_variant
rs1277723870	intron_variant
rs1277741585	genic_downstream_transcript_variant,intron_variant
rs1277772381	intron_variant
rs1277775525	intron_variant
rs1277795347	intron_variant,genic_upstream_transcript_variant
rs1277831483	intron_variant
rs1277921231	intron_variant,genic_upstream_transcript_variant
rs1277934472	genic_downstream_transcript_variant,intron_variant
rs1277944693	intron_variant,genic_upstream_transcript_variant
rs1277970331	intron_variant
rs1277972584	intron_variant,genic_upstream_transcript_variant
rs1277997460	intron_variant
rs1277998285	genic_downstream_transcript_variant,intron_variant
rs1278009708	genic_downstream_transcript_variant,intron_variant
rs1278027483	intron_variant,genic_upstream_transcript_variant
rs1278084939	intron_variant
rs1278136729	intron_variant
rs1278149539	intron_variant,genic_upstream_transcript_variant
rs1278161224	intron_variant
rs1278162995	intron_variant
rs1278170000	intron_variant,genic_upstream_transcript_variant
rs1278181669	genic_downstream_transcript_variant,intron_variant
rs1278188090	intron_variant
rs1278191312	genic_downstream_transcript_variant,intron_variant
rs1278246902	intron_variant,genic_upstream_transcript_variant
rs1278247606	upstream_transcript_variant,5_prime_UTR_variant,intron_variant,genic_upstream_transcript_variant
rs1278266970	intron_variant
rs1278299668	genic_downstream_transcript_variant,intron_variant
rs1278327101	intron_variant,genic_upstream_transcript_variant
rs1278355739	intron_variant
rs1278381639	genic_downstream_transcript_variant,intron_variant
rs1278480721	intron_variant,genic_upstream_transcript_variant
rs1278509873	upstream_transcript_variant,intron_variant,genic_upstream_transcript_variant,2KB_upstream_variant
rs1278519265	non_coding_transcript_variant,genic_downstream_transcript_variant,3_prime_UTR_variant
rs1278572217	intron_variant,genic_upstream_transcript_variant
rs1278582969	intron_variant,genic_upstream_transcript_variant
rs1278589356	intron_variant
rs1278607916	intron_variant,genic_upstream_transcript_variant
rs1278621285	genic_downstream_transcript_variant,intron_variant
rs1278662383	intron_variant,genic_upstream_transcript_variant
rs1278683289	intron_variant,genic_upstream_transcript_variant
rs1278690740	intron_variant
rs1278699328	intron_variant,genic_upstream_transcript_variant
rs1278700449	intron_variant,genic_upstream_transcript_variant
rs1278730439	upstream_transcript_variant,intron_variant,genic_upstream_transcript_variant,2KB_upstream_variant
rs1278777062	intron_variant
rs1278807556	upstream_transcript_variant,intron_variant,genic_upstream_transcript_variant
rs1278837822	intron_variant
rs1278862804	intron_variant
rs1278901075	genic_downstream_transcript_variant,intron_variant
rs1278914912	upstream_transcript_variant,intron_variant,genic_upstream_transcript_variant,2KB_upstream_variant
rs1278974934	intron_variant,genic_upstream_transcript_variant
rs1278990580	upstream_transcript_variant,intron_variant,genic_upstream_transcript_variant
rs1278995056	intron_variant,genic_upstream_transcript_variant
rs1279008898	intron_variant
rs1279012305	intron_variant
rs1279078055	intron_variant,genic_upstream_transcript_variant
rs1279083186	intron_variant
rs1279168775	intron_variant,genic_upstream_transcript_variant
rs1279178324	intron_variant,genic_upstream_transcript_variant
rs1279183241	intron_variant,genic_upstream_transcript_variant
rs1279309129	intron_variant,genic_upstream_transcript_variant
rs1279314106	intron_variant,genic_upstream_transcript_variant

rs1279345802	intron_variant
rs1279375870	intron_variant
rs1279385571	intron_variant,genic_upstream_transcript_variant
rs1279427840	intron_variant,genic_upstream_transcript_variant
rs1279501309	intron_variant
rs1279526015	intron_variant
rs1279603875	intron_variant,genic_upstream_transcript_variant
rs1279614924	intron_variant
rs1279616540	intron_variant
rs1279628429	intron_variant,genic_upstream_transcript_variant
rs1279634913	intron_variant
rs1279655452	genic_downstream_transcript_variant,intron_variant
rs1279681494	intron_variant,genic_upstream_transcript_variant
rs1279685851	intron_variant
rs1279686630	intron_variant,genic_upstream_transcript_variant
rs1279695290	intron_variant
rs1279722189	genic_downstream_transcript_variant,intron_variant
rs1279728514	intron_variant,genic_upstream_transcript_variant
rs1279731627	genic_downstream_transcript_variant,intron_variant
rs1279760943	upstream_transcript_variant,intron_variant,genic_upstream_transcript_variant,2KB_upstream_variant
rs1279766176	intron_variant
rs1279791207	intron_variant
rs1279813380	intron_variant,genic_upstream_transcript_variant
rs1279834765	genic_downstream_transcript_variant,intron_variant
rs1279840256	intron_variant
rs1279850324	intron_variant
rs1279874180	intron_variant,genic_upstream_transcript_variant
rs1279881447	intron_variant,genic_upstream_transcript_variant
rs1279889665	intron_variant,genic_upstream_transcript_variant
rs1279908242	intron_variant
rs1279915286	genic_downstream_transcript_variant,intron_variant
rs1279957983	intron_variant
rs1279984734	intron_variant
rs1279990295	genic_downstream_transcript_variant,intron_variant
rs1280005196	intron_variant,genic_upstream_transcript_variant
rs1280013458	intron_variant
rs1280037748	intron_variant
rs1280080393	intron_variant
rs1280091054	intron_variant
rs1280109095	genic_downstream_transcript_variant,intron_variant
rs1280127517	intron_variant,genic_upstream_transcript_variant
rs1280146709	intron_variant
rs1280163081	intron_variant
rs1280164718	upstream_transcript_variant,2KB_upstream_variant,intron_variant,genic_upstream_transcript_variant
rs1280179947	intron_variant,genic_upstream_transcript_variant
rs1280182293	intron_variant,genic_upstream_transcript_variant
rs1280185295	intron_variant,genic_upstream_transcript_variant
rs1280200435	intron_variant
rs1280202392	upstream_transcript_variant,intron_variant,genic_upstream_transcript_variant
rs1280234542	intron_variant,genic_upstream_transcript_variant
rs1280253255	intron_variant
rs1280283658	genic_downstream_transcript_variant,intron_variant
rs1280315927	intron_variant
rs1280323416	intron_variant,genic_upstream_transcript_variant
rs1280329877	genic_downstream_transcript_variant,intron_variant
rs1280353352	genic_downstream_transcript_variant,intron_variant
rs1280365415	intron_variant,genic_upstream_transcript_variant
rs1280368838	intron_variant,genic_upstream_transcript_variant
rs1280392223	intron_variant,genic_upstream_transcript_variant
rs1280403460	genic_downstream_transcript_variant,intron_variant
rs1280404179	intron_variant
rs1280413678	intron_variant
rs1280439431	intron_variant
rs1280439917	intron_variant,genic_upstream_transcript_variant
rs1280451611	500B_downstream_variant,downstream_transcript_variant
rs1280469555	intron_variant
rs1280477599	intron_variant
rs1280499789	genic_downstream_transcript_variant,intron_variant
rs1280507718	intron_variant,genic_upstream_transcript_variant
rs1280533370	intron_variant
rs1280608441	intron_variant,genic_upstream_transcript_variant
rs1280621026	intron_variant
rs1280625230	genic_downstream_transcript_variant,intron_variant
rs1280677004	intron_variant,genic_upstream_transcript_variant

rs1280722934	intron_variant
rs1280782616	intron_variant
rs1280813136	intron_variant
rs1280848117	intron_variant
rs1280865187	intron_variant,genic_upstream_transcript_variant
rs1280876929	intron_variant
rs1280927746	intron_variant,genic_upstream_transcript_variant
rs1280997418	intron_variant,genic_upstream_transcript_variant
rs1281008331	intron_variant
rs1281024827	intron_variant,genic_upstream_transcript_variant
rs1281050708	non_coding_transcript_variant,5_prime_UTR_variant,intron_variant,genic_upstream_transcript_variant
rs1281054035	intron_variant
rs1281099493	genic_downstream_transcript_variant,intron_variant
rs1281101051	intron_variant,genic_upstream_transcript_variant
rs1281107245	intron_variant
rs1281119923	intron_variant
rs1281121858	upstream_transcript_variant,2KB_upstream_variant,intron_variant,genic_upstream_transcript_variant
rs1281163852	genic_downstream_transcript_variant,intron_variant
rs1281185321	intron_variant
rs1281246327	intron_variant,genic_upstream_transcript_variant
rs1281264563	upstream_transcript_variant,intron_variant,genic_upstream_transcript_variant
rs1281277752	intron_variant
rs1281299481	intron_variant,genic_upstream_transcript_variant
rs1281306051	intron_variant
rs1281319411	intron_variant,genic_upstream_transcript_variant
rs1281327418	intron_variant,genic_upstream_transcript_variant
rs1281330823	intron_variant
rs1281370096	intron_variant,genic_upstream_transcript_variant
rs1281373178	intron_variant
rs1281387954	genic_downstream_transcript_variant,intron_variant
rs1281418934	intron_variant,genic_upstream_transcript_variant
rs1281432191	intron_variant,genic_upstream_transcript_variant
rs1281438118	upstream_transcript_variant,intron_variant,genic_upstream_transcript_variant,2KB_upstream_variant
rs1281446375	intron_variant
rs1281454176	intron_variant,genic_upstream_transcript_variant
rs1281480342	intron_variant
rs1281512769	intron_variant,genic_upstream_transcript_variant
rs1281538374	intron_variant
rs1281541939	genic_downstream_transcript_variant,intron_variant
rs1281565512	intron_variant
rs1281595146	intron_variant,genic_upstream_transcript_variant
rs1281598502	intron_variant
rs1281654180	genic_downstream_transcript_variant,intron_variant
rs1281663962	genic_downstream_transcript_variant,intron_variant
rs1281669691	intron_variant
rs1281733180	intron_variant,genic_upstream_transcript_variant
rs1281739381	genic_downstream_transcript_variant,intron_variant
rs1281748256	intron_variant,genic_upstream_transcript_variant
rs1281772042	frameshift_variant,non_coding_transcript_variant,coding_sequence_variant,intron_variant,genic_upstream_transcript_variant
rs1281774087	intron_variant
rs1281799148	genic_downstream_transcript_variant,intron_variant
rs1281860482	non_coding_transcript_variant,synonymous_variant,coding_sequence_variant
rs1281879668	intron_variant,genic_upstream_transcript_variant
rs1281891321	genic_downstream_transcript_variant,intron_variant
rs1281897645	genic_downstream_transcript_variant,intron_variant,downstream_transcript_variant
rs1281936082	upstream_transcript_variant,intron_variant,genic_upstream_transcript_variant
rs1281956324	intron_variant
rs1282015697	intron_variant,genic_upstream_transcript_variant
rs1282019505	intron_variant,genic_upstream_transcript_variant
rs1282050234	intron_variant
rs1282104503	intron_variant
rs1282108738	intron_variant,genic_upstream_transcript_variant
rs1282140178	genic_downstream_transcript_variant,intron_variant
rs1282168201	intron_variant,genic_upstream_transcript_variant
rs1282168364	genic_downstream_transcript_variant,intron_variant
rs1282238947	intron_variant,genic_upstream_transcript_variant
rs1282242060	intron_variant
rs1282244470	genic_downstream_transcript_variant,intron_variant
rs1282251378	intron_variant,genic_upstream_transcript_variant
rs1282257537	intron_variant,genic_upstream_transcript_variant
rs1282271380	upstream_transcript_variant,intron_variant,genic_upstream_transcript_variant
rs1282321478	intron_variant
rs1282338832	intron_variant,genic_upstream_transcript_variant
rs1282343552	intron_variant,genic_upstream_transcript_variant

rs1282379409	upstream_transcript_variant,2KB_upstream_variant,intron_variant,genic_upstream_transcript_variant
rs1282393033	intron_variant,genic_upstream_transcript_variant
rs1282428483	intron_variant
rs1282447446	intron_variant
rs1282449283	intron_variant,genic_upstream_transcript_variant
rs1282492265	genic_downstream_transcript_variant,intron_variant
rs1282501409	genic_downstream_transcript_variant,intron_variant
rs1282535840	intron_variant,genic_upstream_transcript_variant
rs1282577549	intron_variant
rs1282580231	intron_variant,genic_upstream_transcript_variant
rs1282622468	intron_variant
rs1282654244	intron_variant
rs1282698340	intron_variant
rs1282701050	genic_downstream_transcript_variant,intron_variant
rs1282703470	genic_downstream_transcript_variant,intron_variant
rs1282709062	intron_variant,genic_upstream_transcript_variant
rs1282716584	missense_variant,non_coding_transcript_variant,coding_sequence_variant,intron_variant,genic_upstream_transcript_variant
rs1282745722	intron_variant,genic_upstream_transcript_variant
rs1282795450	intron_variant,genic_upstream_transcript_variant
rs1282813582	genic_downstream_transcript_variant,intron_variant
rs1282843185	intron_variant
rs1282845389	genic_downstream_transcript_variant,intron_variant
rs1282865339	intron_variant
rs1282926304	intron_variant,genic_upstream_transcript_variant
rs1282980380	intron_variant,genic_upstream_transcript_variant
rs1282984968	intron_variant,genic_upstream_transcript_variant
rs1282994326	intron_variant
rs1283007648	intron_variant,genic_upstream_transcript_variant
rs1283022158	intron_variant
rs1283026928	intron_variant
rs1283080503	intron_variant
rs1283089076	intron_variant
rs1283118901	intron_variant
rs1283124915	intron_variant
rs1283168380	intron_variant
rs1283193919	synonymous_variant,non_coding_transcript_variant,coding_sequence_variant,intron_variant,genic_upstream_transcript_variant
rs1283205419	non_coding_transcript_variant,missense_variant,genic_downstream_transcript_variant,coding_sequence_variant
rs1283260041	intron_variant
rs1283286175	intron_variant
rs1283312333	intron_variant
rs1283394226	intron_variant,genic_upstream_transcript_variant
rs1283410383	genic_downstream_transcript_variant,intron_variant
rs1283452666	intron_variant,genic_upstream_transcript_variant
rs1283503003	intron_variant
rs1283503776	genic_downstream_transcript_variant,intron_variant
rs1283510023	intron_variant,genic_upstream_transcript_variant
rs1283518529	intron_variant
rs1283530638	genic_downstream_transcript_variant,intron_variant
rs1283546203	intron_variant,genic_upstream_transcript_variant
rs1283567679	intron_variant
rs1283575333	intron_variant
rs1283602430	upstream_transcript_variant,intron_variant,genic_upstream_transcript_variant
rs1283610015	genic_downstream_transcript_variant,intron_variant
rs1283618504	intron_variant,genic_upstream_transcript_variant
rs1283673714	genic_downstream_transcript_variant,intron_variant
rs1283721322	intron_variant
rs1283768274	intron_variant
rs1283808683	2KB_upstream_variant,upstream_transcript_variant,intron_variant,genic_upstream_transcript_variant
rs1283841195	genic_downstream_transcript_variant,intron_variant
rs1283855654	upstream_transcript_variant,intron_variant,genic_upstream_transcript_variant
rs1283865297	intron_variant,genic_upstream_transcript_variant
rs1283891978	intron_variant,genic_upstream_transcript_variant
rs1283937259	intron_variant,genic_upstream_transcript_variant
rs1283943833	intron_variant
rs1283971109	intron_variant,genic_upstream_transcript_variant
rs1283985912	genic_downstream_transcript_variant,intron_variant
rs1284047306	intron_variant
rs1284068993	genic_downstream_transcript_variant,intron_variant
rs1284084342	genic_downstream_transcript_variant,intron_variant
rs1284142413	intron_variant
rs1284168468	intron_variant
rs1284192243	intron_variant,genic_upstream_transcript_variant
rs1284246792	genic_downstream_transcript_variant,intron_variant
rs1284312324	intron_variant,genic_upstream_transcript_variant

rs1284355125	intron_variant
rs1284388875	intron_variant,genic_upstream_transcript_variant
rs1284408966	genic_downstream_transcript_variant,intron_variant
rs1284415718	upstream_transcript_variant,intron_variant,genic_upstream_transcript_variant
rs1284434363	intron_variant,genic_upstream_transcript_variant
rs1284441190	intron_variant,genic_upstream_transcript_variant
rs1284446344	intron_variant
rs1284507952	intron_variant
rs1284539964	genic_downstream_transcript_variant,non_coding_transcript_variant,synonymous_variant,coding_sequence_variant
rs1284548430	intron_variant,genic_upstream_transcript_variant
rs1284548526	intron_variant,genic_upstream_transcript_variant
rs1284565671	intron_variant
rs1284578089	intron_variant,genic_upstream_transcript_variant
rs1284593490	genic_downstream_transcript_variant,intron_variant
rs1284597044	genic_downstream_transcript_variant,intron_variant
rs1284672128	intron_variant,genic_upstream_transcript_variant
rs1284689183	genic_downstream_transcript_variant,intron_variant
rs1284703751	genic_downstream_transcript_variant,intron_variant
rs1284734136	intron_variant,genic_upstream_transcript_variant
rs1284750228	intron_variant,genic_upstream_transcript_variant
rs1284751940	genic_downstream_transcript_variant,intron_variant
rs1284768160	intron_variant
rs1284770690	intron_variant,genic_upstream_transcript_variant
rs1284774153	intron_variant
rs1284783062	intron_variant,genic_upstream_transcript_variant
rs1284819948	intron_variant
rs1284835826	intron_variant
rs1284852036	intron_variant,genic_upstream_transcript_variant
rs1284872223	intron_variant,genic_upstream_transcript_variant
rs1284886370	intron_variant,genic_upstream_transcript_variant
rs1284899517	genic_downstream_transcript_variant,intron_variant
rs1284910416	intron_variant,genic_upstream_transcript_variant
rs1284979559	genic_downstream_transcript_variant,intron_variant
rs1285023238	intron_variant,genic_upstream_transcript_variant
rs1285027278	intron_variant,genic_upstream_transcript_variant
rs1285050439	2KB_upstream_variant,upstream_transcript_variant,intron_variant,genic_upstream_transcript_variant
rs1285107313	genic_downstream_transcript_variant,3_prime_UTR_variant,non_coding_transcript_variant
rs1285107403	intron_variant
rs1285120914	intron_variant,genic_upstream_transcript_variant
rs1285136432	intron_variant
rs1285143621	intron_variant
rs1285208468	intron_variant,genic_upstream_transcript_variant
rs1285271578	intron_variant,genic_upstream_transcript_variant
rs1285272487	intron_variant,genic_upstream_transcript_variant
rs1285331586	intron_variant
rs1285344428	intron_variant,genic_upstream_transcript_variant
rs1285419473	upstream_transcript_variant,intron_variant,genic_upstream_transcript_variant
rs1285436453	genic_downstream_transcript_variant,intron_variant
rs1285504541	intron_variant,genic_upstream_transcript_variant
rs1285508873	intron_variant,genic_upstream_transcript_variant
rs1285517144	intron_variant,genic_upstream_transcript_variant
rs1285535822	intron_variant,genic_upstream_transcript_variant
rs1285597857	genic_downstream_transcript_variant,intron_variant
rs1285635871	intron_variant
rs1285659894	intron_variant,genic_upstream_transcript_variant
rs1285703107	intron_variant
rs1285707255	intron_variant,genic_upstream_transcript_variant
rs1285728876	upstream_transcript_variant,intron_variant,genic_upstream_transcript_variant
rs1285730150	genic_downstream_transcript_variant,intron_variant
rs1285731743	genic_downstream_transcript_variant,intron_variant
rs1285762910	intron_variant,genic_upstream_transcript_variant
rs1285805044	intron_variant
rs1285808715	2KB_upstream_variant,upstream_transcript_variant,intron_variant,genic_upstream_transcript_variant
rs1285814811	non_coding_transcript_variant,coding_sequence_variant,intron_variant,synonymous_variant,genic_upstream_transcript_variant
rs1285834272	intron_variant
rs1285861148	intron_variant,genic_upstream_transcript_variant
rs1285882072	genic_downstream_transcript_variant,non_coding_transcript_variant,synonymous_variant,coding_sequence_variant
rs1285885083	intron_variant,genic_upstream_transcript_variant
rs1285886859	genic_downstream_transcript_variant,intron_variant
rs1285898840	intron_variant,genic_upstream_transcript_variant
rs1285934494	intron_variant,genic_upstream_transcript_variant
rs1285948685	intron_variant
rs1285969030	genic_downstream_transcript_variant,intron_variant
rs1285969294	intron_variant

rs1286032387	intron_variant,genic_upstream_transcript_variant
rs1286049811	intron_variant,genic_upstream_transcript_variant
rs1286056026	genic_downstream_transcript_variant,intron_variant
rs1286089686	intron_variant,genic_upstream_transcript_variant
rs1286108139	genic_downstream_transcript_variant,intron_variant
rs1286114427	intron_variant,genic_upstream_transcript_variant
rs1286148199	intron_variant,genic_upstream_transcript_variant
rs1286168412	intron_variant,genic_upstream_transcript_variant
rs1286280181	intron_variant
rs1286295576	intron_variant,genic_upstream_transcript_variant
rs1286303343	intron_variant,genic_upstream_transcript_variant
rs1286307221	intron_variant
rs1286336446	intron_variant
rs1286383884	intron_variant,genic_upstream_transcript_variant
rs1286392674	intron_variant
rs1286410665	intron_variant
rs1286425611	intron_variant,genic_upstream_transcript_variant
rs1286429727	genic_downstream_transcript_variant,intron_variant
rs1286463225	genic_downstream_transcript_variant,intron_variant
rs1286470576	genic_downstream_transcript_variant,intron_variant
rs1286515581	intron_variant
rs1286558982	intron_variant
rs1286579416	intron_variant,genic_upstream_transcript_variant
rs1286615677	intron_variant,genic_upstream_transcript_variant
rs1286621135	intron_variant
rs1286623650	intron_variant
rs1286628046	intron_variant,genic_upstream_transcript_variant
rs1286648579	intron_variant,genic_upstream_transcript_variant
rs1286675349	intron_variant
rs1286690548	intron_variant
rs1286699503	2KB_upstream_variant,upstream_transcript_variant,intron_variant,genic_upstream_transcript_variant
rs1286727983	intron_variant
rs1286750638	intron_variant,genic_upstream_transcript_variant
rs1286754741	missense_variant,genic_downstream_transcript_variant,non_coding_transcript_variant,coding_sequence_variant
rs1286810875	intron_variant,genic_upstream_transcript_variant
rs1286844084	2KB_upstream_variant,upstream_transcript_variant,intron_variant,genic_upstream_transcript_variant
rs1286855905	intron_variant,genic_upstream_transcript_variant
rs1286864691	intron_variant,genic_upstream_transcript_variant
rs1286865994	intron_variant,genic_upstream_transcript_variant
rs1286867856	2KB_upstream_variant,upstream_transcript_variant,intron_variant,genic_upstream_transcript_variant
rs1286873438	intron_variant
rs1286921734	missense_variant,genic_downstream_transcript_variant,non_coding_transcript_variant,coding_sequence_variant
rs1286956397	intron_variant,genic_upstream_transcript_variant
rs1287005793	intron_variant,genic_upstream_transcript_variant
rs1287045050	intron_variant
rs1287077050	intron_variant,genic_upstream_transcript_variant
rs1287086268	intron_variant
rs1287177207	2KB_upstream_variant,upstream_transcript_variant,intron_variant,genic_upstream_transcript_variant
rs1287293358	intron_variant
rs1287311800	intron_variant
rs1287314329	intron_variant,genic_upstream_transcript_variant
rs1287340050	intron_variant,genic_upstream_transcript_variant
rs1287359148	intron_variant
rs1287430006	2KB_upstream_variant,upstream_transcript_variant,intron_variant,genic_upstream_transcript_variant
rs1287434452	2KB_upstream_variant,upstream_transcript_variant,intron_variant,genic_upstream_transcript_variant
rs1287445972	genic_downstream_transcript_variant,intron_variant
rs1287449571	intron_variant,genic_upstream_transcript_variant
rs1287467579	intron_variant,genic_upstream_transcript_variant
rs1287471560	intron_variant,genic_upstream_transcript_variant
rs1287523485	intron_variant,genic_upstream_transcript_variant
rs1287543820	upstream_transcript_variant,intron_variant,genic_upstream_transcript_variant
rs1287580062	intron_variant,genic_upstream_transcript_variant
rs1287589229	intron_variant,genic_upstream_transcript_variant
rs1287611749	intron_variant,genic_upstream_transcript_variant
rs1287619412	intron_variant
rs1287623350	intron_variant,genic_upstream_transcript_variant
rs1287669352	intron_variant,genic_upstream_transcript_variant
rs1287702840	intron_variant,genic_upstream_transcript_variant
rs1287705881	intron_variant
rs1287713346	missense_variant,non_coding_transcript_variant,coding_sequence_variant
rs1287728252	intron_variant
rs1287746166	intron_variant
rs1287756510	genic_downstream_transcript_variant,intron_variant
rs1287767258	intron_variant,genic_upstream_transcript_variant

rs1287798056	genic_downstream_transcript_variant,intron_variant
rs1287804526	intron_variant
rs1287844092	intron_variant,genic_upstream_transcript_variant
rs1287863113	genic_downstream_transcript_variant,intron_variant
rs1287870131	intron_variant,genic_upstream_transcript_variant
rs1287884896	intron_variant,genic_upstream_transcript_variant
rs1287889627	genic_downstream_transcript_variant,intron_variant
rs1287897336	genic_downstream_transcript_variant,3_prime_UTR_variant,non_coding_transcript_variant
rs1287941977	intron_variant
rs1287955166	intron_variant,genic_upstream_transcript_variant
rs1288025184	genic_downstream_transcript_variant,intron_variant
rs1288060370	intron_variant,genic_upstream_transcript_variant
rs1288074173	intron_variant
rs1288117967	upstream_transcript_variant,intron_variant,genic_upstream_transcript_variant
rs1288127047	intron_variant,genic_upstream_transcript_variant
rs1288130005	genic_downstream_transcript_variant,intron_variant
rs1288133932	intron_variant,genic_upstream_transcript_variant
rs1288152479	genic_downstream_transcript_variant,intron_variant
rs1288173120	intron_variant,genic_upstream_transcript_variant
rs1288173385	genic_downstream_transcript_variant,intron_variant
rs1288210811	2KB_upstream_variant,upstream_transcript_variant,intron_variant,genic_upstream_transcript_variant
rs1288273663	genic_downstream_transcript_variant,intron_variant
rs1288300392	genic_downstream_transcript_variant,intron_variant
rs1288324061	genic_downstream_transcript_variant,intron_variant
rs1288329194	intron_variant
rs1288361160	genic_downstream_transcript_variant,intron_variant
rs1288415206	intron_variant,genic_upstream_transcript_variant
rs1288420616	intron_variant
rs1288425843	intron_variant,genic_upstream_transcript_variant
rs1288461236	intron_variant
rs1288477621	missense_variant,non_coding_transcript_variant,coding_sequence_variant
rs1288497665	genic_downstream_transcript_variant,intron_variant
rs1288509480	intron_variant,genic_upstream_transcript_variant
rs1288511499	intron_variant
rs1288541247	upstream_transcript_variant,intron_variant,genic_upstream_transcript_variant
rs1288544058	intron_variant
rs1288574667	intron_variant,genic_upstream_transcript_variant
rs1288576355	intron_variant
rs1288625626	intron_variant
rs1288678213	intron_variant
rs1288686098	genic_downstream_transcript_variant,3_prime_UTR_variant,non_coding_transcript_variant
rs1288719074	intron_variant,genic_upstream_transcript_variant
rs1288736024	genic_downstream_transcript_variant,intron_variant
rs1288796369	intron_variant,genic_upstream_transcript_variant
rs1288798752	intron_variant
rs1288804179	missense_variant,non_coding_transcript_variant,coding_sequence_variant
rs1288843237	missense_variant,5_prime_UTR_variant,non_coding_transcript_variant,coding_sequence_variant
rs1288850635	upstream_transcript_variant,intron_variant,genic_upstream_transcript_variant
rs1288921729	genic_downstream_transcript_variant,intron_variant
rs1288941934	intron_variant
rs1288970736	intron_variant,genic_upstream_transcript_variant
rs1288993104	5_prime_UTR_variant,upstream_transcript_variant,intron_variant,genic_upstream_transcript_variant
rs1289025289	genic_downstream_transcript_variant,intron_variant
rs1289109150	genic_downstream_transcript_variant,intron_variant
rs1289122927	2KB_upstream_variant,upstream_transcript_variant,intron_variant,genic_upstream_transcript_variant
rs1289141363	genic_downstream_transcript_variant,intron_variant
rs1289151667	genic_downstream_transcript_variant,intron_variant
rs1289170197	genic_downstream_transcript_variant,intron_variant
rs1289225276	intron_variant,genic_upstream_transcript_variant
rs1289301201	intron_variant
rs1289304740	intron_variant,genic_upstream_transcript_variant
rs1289375343	intron_variant,genic_upstream_transcript_variant
rs1289426113	intron_variant
rs1289433626	2KB_upstream_variant,upstream_transcript_variant,intron_variant,genic_upstream_transcript_variant
rs1289435740	intron_variant
rs1289441925	intron_variant
rs1289453370	intron_variant
rs1289491672	intron_variant
rs1289519995	intron_variant
rs1289545627	intron_variant
rs1289548347	genic_downstream_transcript_variant,intron_variant
rs1289570763	intron_variant,genic_upstream_transcript_variant
rs1289581175	intron_variant
rs1289601470	intron_variant,genic_upstream_transcript_variant

rs1289610537	intron_variant
rs1289667027	upstream_transcript_variant,intron_variant,genic_upstream_transcript_variant
rs1289672936	intron_variant,genic_upstream_transcript_variant
rs1289689030	intron_variant,genic_upstream_transcript_variant
rs1289692037	intron_variant
rs1289697844	missense_variant,non_coding_transcript_variant,coding_sequence_variant
rs1289703955	intron_variant
rs1289733281	genic_downstream_transcript_variant,intron_variant
rs1289734599	intron_variant
rs1289741596	intron_variant,genic_upstream_transcript_variant
rs1289752709	intron_variant,genic_upstream_transcript_variant
rs1289767590	intron_variant
rs1289787138	intron_variant,genic_upstream_transcript_variant
rs1289795258	genic_downstream_transcript_variant,intron_variant
rs1289831406	intron_variant
rs1289836269	intron_variant
rs1289838678	intron_variant
rs1289865204	2KB_upstream_variant,upstream_transcript_variant,intron_variant,genic_upstream_transcript_variant
rs1289959970	intron_variant
rs1289960557	genic_downstream_transcript_variant,intron_variant
rs1289962387	intron_variant
rs1289965025	intron_variant,genic_upstream_transcript_variant
rs1289983407	intron_variant,genic_upstream_transcript_variant
rs1290020505	intron_variant
rs1290026340	intron_variant,genic_upstream_transcript_variant
rs1290039988	non_coding_transcript_variant,coding_sequence_variant,intron_variant,synonymous_variant,genic_upstream_transcript_variant
rs1290067267	intron_variant,genic_upstream_transcript_variant
rs1290103534	intron_variant
rs1290133060	intron_variant,genic_upstream_transcript_variant
rs1290139738	genic_downstream_transcript_variant,3_prime_UTR_variant,non_coding_transcript_variant
rs1290191273	intron_variant
rs1290266741	intron_variant,genic_upstream_transcript_variant
rs1290296325	genic_downstream_transcript_variant,intron_variant
rs1290308562	intron_variant
rs1290346315	genic_downstream_transcript_variant,intron_variant
rs1290346524	missense_variant,non_coding_transcript_variant,coding_sequence_variant
rs1290348214	intron_variant
rs1290357124	genic_downstream_transcript_variant,downstream_transcript_variant,intron_variant
rs1290435208	genic_downstream_transcript_variant,intron_variant
rs1290445484	intron_variant
rs1290449854	genic_downstream_transcript_variant,intron_variant
rs1290518006	intron_variant
rs1290556224	intron_variant,genic_upstream_transcript_variant
rs1290577233	intron_variant,genic_upstream_transcript_variant
rs1290589440	genic_downstream_transcript_variant,intron_variant
rs1290614302	intron_variant
rs1290627957	intron_variant,genic_upstream_transcript_variant
rs1290743233	intron_variant,genic_upstream_transcript_variant
rs1290746165	intron_variant,genic_upstream_transcript_variant
rs1290808051	intron_variant
rs1290831460	intron_variant,genic_upstream_transcript_variant
rs1290879909	intron_variant,genic_upstream_transcript_variant
rs1290891402	intron_variant,genic_upstream_transcript_variant
rs1290913078	intron_variant,genic_upstream_transcript_variant
rs1290917552	missense_variant,genic_downstream_transcript_variant,non_coding_transcript_variant,coding_sequence_variant
rs1290919717	intron_variant
rs1290942818	intron_variant,genic_upstream_transcript_variant
rs1290981278	upstream_transcript_variant,intron_variant,genic_upstream_transcript_variant
rs1291023487	genic_downstream_transcript_variant,intron_variant
rs1291141844	intron_variant,genic_upstream_transcript_variant
rs1291142722	intron_variant,genic_upstream_transcript_variant
rs1291146785	intron_variant,genic_upstream_transcript_variant
rs1291174323	intron_variant,genic_upstream_transcript_variant
rs1291192893	genic_downstream_transcript_variant,intron_variant
rs1291195326	intron_variant
rs1291245134	intron_variant,genic_upstream_transcript_variant
rs1291249936	intron_variant,genic_upstream_transcript_variant
rs1291274502	intron_variant,genic_upstream_transcript_variant
rs1291296342	intron_variant,genic_upstream_transcript_variant
rs1291326178	intron_variant
rs1291329266	intron_variant
rs1291339327	genic_downstream_transcript_variant,intron_variant
rs1291375024	intron_variant,genic_upstream_transcript_variant
rs1291386679	genic_downstream_transcript_variant,intron_variant

rs1291424376	intron_variant
rs1291428820	intron_variant
rs1291490691	genic_downstream_transcript_variant,intron_variant
rs1291541736	genic_downstream_transcript_variant,intron_variant
rs1291544396	intron_variant
rs1291590272	intron_variant
rs1291594245	genic_downstream_transcript_variant,intron_variant
rs1291600493	intron_variant
rs1291603995	genic_downstream_transcript_variant,intron_variant
rs1291673362	intron_variant
rs1291695510	intron_variant
rs1291695835	intron_variant,genic_upstream_transcript_variant
rs1291706633	intron_variant
rs1291709499	intron_variant
rs1291789951	intron_variant
rs1291837610	intron_variant,genic_upstream_transcript_variant
rs1291860796	intron_variant
rs1291899646	intron_variant,genic_upstream_transcript_variant
rs1291984722	intron_variant
rs1291995584	intron_variant,genic_upstream_transcript_variant
rs1291999515	intron_variant
rs1292030283	upstream_transcript_variant,intron_variant,genic_upstream_transcript_variant
rs1292031212	intron_variant,genic_upstream_transcript_variant
rs1292034686	intron_variant,genic_upstream_transcript_variant
rs1292039375	intron_variant,genic_upstream_transcript_variant
rs1292067972	intron_variant
rs1292186674	intron_variant
rs1292218089	intron_variant,genic_upstream_transcript_variant
rs1292218982	intron_variant
rs1292243824	intron_variant
rs1292263943	intron_variant,genic_upstream_transcript_variant
rs1292286021	intron_variant,genic_upstream_transcript_variant
rs1292299145	intron_variant,genic_upstream_transcript_variant
rs1292299439	upstream_transcript_variant,intron_variant,genic_upstream_transcript_variant
rs1292324988	intron_variant
rs1292326082	intron_variant
rs1292384674	genic_downstream_transcript_variant,intron_variant
rs1292387014	intron_variant,genic_upstream_transcript_variant
rs1292398565	intron_variant
rs1292421465	intron_variant
rs1292479477	genic_downstream_transcript_variant,intron_variant
rs1292484159	intron_variant
rs1292497762	intron_variant,genic_upstream_transcript_variant
rs1292511225	intron_variant,genic_upstream_transcript_variant
rs1292520469	intron_variant
rs1292545280	intron_variant
rs1292577093	genic_downstream_transcript_variant,intron_variant
rs1292605611	intron_variant,genic_upstream_transcript_variant
rs1292663972	genic_downstream_transcript_variant,intron_variant
rs1292666609	intron_variant
rs1292726852	intron_variant
rs1292757932	intron_variant,genic_upstream_transcript_variant
rs1292761203	intron_variant,genic_upstream_transcript_variant
rs1292790122	intron_variant
rs1292808791	genic_downstream_transcript_variant,intron_variant
rs1292816383	intron_variant,genic_upstream_transcript_variant
rs1292835360	intron_variant,genic_upstream_transcript_variant
rs1292843318	intron_variant
rs1292888872	intron_variant
rs1292911013	intron_variant
rs1292920682	intron_variant,genic_upstream_transcript_variant
rs1292990794	intron_variant,genic_upstream_transcript_variant
rs1293001086	2KB_upstream_variant,upstream_transcript_variant,intron_variant,genic_upstream_transcript_variant
rs1293111305	intron_variant,genic_upstream_transcript_variant
rs1293115994	intron_variant,genic_upstream_transcript_variant
rs1293167747	intron_variant
rs1293175896	intron_variant,genic_upstream_transcript_variant
rs1293182814	intron_variant,genic_upstream_transcript_variant
rs1293201092	intron_variant,genic_upstream_transcript_variant
rs1293229973	intron_variant,genic_upstream_transcript_variant
rs1293254971	intron_variant
rs1293297101	intron_variant,genic_upstream_transcript_variant
rs1293302386	intron_variant,genic_upstream_transcript_variant
rs1293314079	intron_variant,genic_upstream_transcript_variant

rs1293347552	intron_variant
rs1293352539	intron_variant,genic_upstream_transcript_variant
rs1293376532	intron_variant,genic_upstream_transcript_variant
rs1293393029	intron_variant,genic_upstream_transcript_variant
rs1293555456	genic_downstream_transcript_variant,intron_variant
rs1293558094	intron_variant
rs1293558177	intron_variant
rs1293570179	intron_variant,genic_upstream_transcript_variant
rs1293619689	missense_variant,non_coding_transcript_variant,coding_sequence_variant
rs1293629156	intron_variant
rs1293662097	intron_variant,genic_upstream_transcript_variant
rs1293710243	intron_variant,genic_upstream_transcript_variant
rs1293724373	intron_variant
rs1293760543	non_coding_transcript_variant,coding_sequence_variant,intron_variant,genic_upstream_transcript_variant,missense_variant
rs1293761372	genic_downstream_transcript_variant,intron_variant
rs1293766090	intron_variant,genic_upstream_transcript_variant
rs1293787714	intron_variant
rs1293796802	intron_variant
rs1293802982	intron_variant
rs1293825986	intron_variant
rs1293873029	upstream_transcript_variant,intron_variant,genic_upstream_transcript_variant
rs1293932716	intron_variant
rs1293961262	intron_variant
rs1293962930	intron_variant,genic_upstream_transcript_variant
rs1293964085	genic_downstream_transcript_variant,3_prime_UTR_variant,non_coding_transcript_variant
rs1293974802	intron_variant
rs1293981682	intron_variant,genic_upstream_transcript_variant
rs1294013018	intron_variant
rs1294015220	intron_variant,genic_upstream_transcript_variant
rs1294048353	intron_variant
rs1294057952	intron_variant
rs1294065862	intron_variant
rs1294067298	intron_variant,genic_upstream_transcript_variant
rs1294129869	splice_donor_variant,intron_variant
rs1294137335	intron_variant
rs1294164501	upstream_transcript_variant,intron_variant,genic_upstream_transcript_variant
rs1294171619	genic_downstream_transcript_variant,intron_variant
rs1294180567	intron_variant
rs1294182538	intron_variant,genic_upstream_transcript_variant
rs1294251330	intron_variant,genic_upstream_transcript_variant
rs1294256173	genic_downstream_transcript_variant,intron_variant
rs1294296941	intron_variant,genic_upstream_transcript_variant
rs1294306253	intron_variant,genic_upstream_transcript_variant
rs1294343125	intron_variant
rs1294357532	intron_variant,genic_upstream_transcript_variant
rs1294358256	genic_downstream_transcript_variant,intron_variant
rs1294361435	2KB_upstream_variant,upstream_transcript_variant,intron_variant,genic_upstream_transcript_variant
rs1294408368	genic_downstream_transcript_variant,intron_variant
rs1294420483	intron_variant,genic_upstream_transcript_variant
rs1294429779	missense_variant,non_coding_transcript_variant,coding_sequence_variant
rs1294436710	intron_variant,genic_upstream_transcript_variant
rs1294477697	intron_variant,genic_upstream_transcript_variant
rs1294521418	intron_variant,genic_upstream_transcript_variant
rs1294552230	intron_variant,genic_upstream_transcript_variant
rs1294553706	genic_downstream_transcript_variant,intron_variant
rs1294565381	intron_variant
rs1294565627	genic_downstream_transcript_variant,intron_variant
rs1294575200	2KB_upstream_variant,upstream_transcript_variant,intron_variant,genic_upstream_transcript_variant
rs1294577229	intron_variant,genic_upstream_transcript_variant
rs1294577809	genic_downstream_transcript_variant,intron_variant
rs1294632372	intron_variant,genic_upstream_transcript_variant
rs1294642856	intron_variant,genic_upstream_transcript_variant
rs1294643520	2KB_upstream_variant,upstream_transcript_variant,intron_variant,genic_upstream_transcript_variant
rs1294659481	intron_variant,genic_upstream_transcript_variant
rs1294667743	intron_variant,genic_upstream_transcript_variant
rs1294700303	intron_variant,genic_upstream_transcript_variant
rs1294717831	genic_downstream_transcript_variant,intron_variant
rs1294722137	genic_downstream_transcript_variant,intron_variant
rs1294769391	non_coding_transcript_variant,coding_sequence_variant,intron_variant,genic_upstream_transcript_variant,missense_variant
rs1294783432	intron_variant,genic_upstream_transcript_variant
rs1294784258	intron_variant
rs1294797673	intron_variant,genic_upstream_transcript_variant
rs1294821030	intron_variant,genic_upstream_transcript_variant
rs1294853112	intron_variant

rs1294924754	non_coding_transcript_variant,coding_sequence_variant,intron_variant,genic_upstream_transcript_variant,missense_variant
rs1294932681	genic_downstream_transcript_variant,intron_variant
rs1295011733	genic_downstream_transcript_variant,intron_variant
rs1295055412	intron_variant,genic_upstream_transcript_variant
rs1295070149	intron_variant
rs1295075433	intron_variant
rs1295083446	intron_variant,genic_upstream_transcript_variant
rs1295105383	intron_variant
rs1295110452	intron_variant,genic_upstream_transcript_variant
rs1295127328	intron_variant
rs1295164696	intron_variant
rs1295166292	intron_variant
rs1295185291	intron_variant,genic_upstream_transcript_variant
rs1295219530	intron_variant,genic_upstream_transcript_variant
rs1295247757	non_coding_transcript_variant,synonymous_variant,coding_sequence_variant
rs1295253357	intron_variant,genic_upstream_transcript_variant
rs1295455580	genic_downstream_transcript_variant,3_prime_UTR_variant,non_coding_transcript_variant
rs1295548832	intron_variant
rs1295555188	2KB_upstream_variant,upstream_transcript_variant,intron_variant,genic_upstream_transcript_variant
rs1295577027	genic_downstream_transcript_variant,intron_variant
rs1295588443	intron_variant,genic_upstream_transcript_variant
rs1295642266	intron_variant
rs1295658411	intron_variant,genic_upstream_transcript_variant
rs1295665070	intron_variant
rs1295732671	genic_downstream_transcript_variant,downstream_transcript_variant,intron_variant
rs1295738778	2KB_upstream_variant,upstream_transcript_variant,intron_variant,genic_upstream_transcript_variant
rs1295748290	intron_variant,genic_upstream_transcript_variant
rs1295790703	5_prime_UTR_variant,intron_variant
rs1295905915	intron_variant
rs1295906264	intron_variant
rs1295936923	intron_variant,genic_upstream_transcript_variant
rs1295937311	intron_variant
rs1295946866	intron_variant,genic_upstream_transcript_variant
rs1295955085	intron_variant
rs1296028280	intron_variant,genic_upstream_transcript_variant
rs1296171493	intron_variant
rs1296186667	intron_variant,genic_upstream_transcript_variant
rs1296209507	intron_variant,genic_upstream_transcript_variant
rs1296237612	genic_downstream_transcript_variant,intron_variant
rs1296266788	intron_variant
rs1296280927	intron_variant
rs1296289277	intron_variant,genic_upstream_transcript_variant
rs1296318268	genic_upstream_transcript_variant,intron_variant
rs1296336828	genic_upstream_transcript_variant,intron_variant
rs1296397597	intron_variant
rs1296452113	genic_upstream_transcript_variant,intron_variant
rs1296452439	genic_upstream_transcript_variant,intron_variant
rs1296498488	genic_upstream_transcript_variant,intron_variant,upstream_transcript_variant
rs1296525889	intron_variant,genic_downstream_transcript_variant
rs1296543685	genic_upstream_transcript_variant,intron_variant
rs1296586686	genic_upstream_transcript_variant,intron_variant
rs1296592140	intron_variant
rs1296603624	intron_variant
rs1296634139	intron_variant,genic_downstream_transcript_variant
rs1296682431	intron_variant,genic_downstream_transcript_variant
rs1296707879	intron_variant
rs1296722918	intron_variant
rs1296809253	genic_upstream_transcript_variant,intron_variant
rs1296811173	genic_upstream_transcript_variant,intron_variant
rs1296820564	genic_upstream_transcript_variant,intron_variant
rs1296821487	genic_upstream_transcript_variant,intron_variant
rs1296882636	intron_variant,genic_downstream_transcript_variant
rs1296884412	genic_upstream_transcript_variant,intron_variant
rs1296893038	intron_variant
rs1296908519	genic_upstream_transcript_variant,intron_variant
rs1296920761	genic_upstream_transcript_variant,intron_variant
rs1296957946	genic_upstream_transcript_variant,intron_variant,upstream_transcript_variant,2KB_upstream_variant
rs1296969482	coding_sequence_variant,intron_variant,missense_variant
rs1296978574	genic_upstream_transcript_variant,intron_variant
rs1296993780	intron_variant
rs1297029465	genic_upstream_transcript_variant,intron_variant
rs1297040241	genic_upstream_transcript_variant,intron_variant
rs1297049474	500B_downstream_variant,downstream_transcript_variant
rs1297053108	genic_upstream_transcript_variant,intron_variant

rs1297054056	genic_upstream_transcript_variant,intron_variant
rs1297111310	intron_variant
rs1297134988	intron_variant,genic_downstream_transcript_variant
rs1297151502	genic_upstream_transcript_variant,intron_variant
rs1297172129	intron_variant
rs1297178607	genic_upstream_transcript_variant,intron_variant,upstream_transcript_variant
rs1297187515	intron_variant,genic_downstream_transcript_variant
rs1297237758	genic_upstream_transcript_variant,intron_variant
rs1297284987	genic_upstream_transcript_variant,intron_variant
rs1297289430	genic_upstream_transcript_variant,intron_variant
rs1297363533	intron_variant
rs1297421252	intron_variant,genic_downstream_transcript_variant
rs1297424041	genic_upstream_transcript_variant,intron_variant,upstream_transcript_variant,2KB_upstream_variant
rs1297427786	intron_variant
rs1297472107	coding_sequence_variant,non_coding_transcript_variant,genic_downstream_transcript_variant,missense_variant
rs1297527450	non_coding_transcript_variant,genic_downstream_transcript_variant,3_prime_UTR_variant
rs1297535819	intron_variant,genic_downstream_transcript_variant
rs1297541506	intron_variant
rs1297574817	intron_variant
rs1297575857	intron_variant
rs1297603367	intron_variant,genic_downstream_transcript_variant
rs1297624711	intron_variant
rs1297734010	intron_variant
rs1297742097	intron_variant
rs1297816187	intron_variant
rs1297817088	genic_upstream_transcript_variant,intron_variant
rs1297858282	intron_variant,downstream_transcript_variant,genic_downstream_transcript_variant
rs1297909232	genic_upstream_transcript_variant,intron_variant
rs1297910164	genic_upstream_transcript_variant,intron_variant
rs1297937826	intron_variant
rs1297976293	genic_upstream_transcript_variant,intron_variant
rs1297979004	intron_variant
rs1298035495	genic_upstream_transcript_variant,intron_variant
rs1298052510	intron_variant,genic_downstream_transcript_variant
rs1298053401	intron_variant
rs1298055514	intron_variant,genic_downstream_transcript_variant
rs1298089355	intron_variant,genic_downstream_transcript_variant
rs1298091568	genic_upstream_transcript_variant,intron_variant
rs1298092527	intron_variant,genic_downstream_transcript_variant
rs1298094251	intron_variant
rs1298095012	intron_variant
rs1298096753	genic_upstream_transcript_variant,intron_variant
rs1298112298	intron_variant
rs1298146897	genic_upstream_transcript_variant,intron_variant
rs1298174423	intron_variant,genic_downstream_transcript_variant
rs1298188904	intron_variant,genic_downstream_transcript_variant
rs1298193450	intron_variant
rs1298243665	genic_upstream_transcript_variant,intron_variant,upstream_transcript_variant,2KB_upstream_variant
rs1298297408	intron_variant
rs1298322992	genic_upstream_transcript_variant,intron_variant
rs1298334169	genic_upstream_transcript_variant,intron_variant
rs1298337884	genic_upstream_transcript_variant,intron_variant,upstream_transcript_variant,2KB_upstream_variant
rs1298476736	intron_variant,genic_downstream_transcript_variant
rs1298495458	genic_upstream_transcript_variant,intron_variant
rs1298508111	genic_upstream_transcript_variant,intron_variant
rs1298526779	genic_upstream_transcript_variant,intron_variant
rs1298590534	non_coding_transcript_variant,genic_downstream_transcript_variant,3_prime_UTR_variant
rs1298591677	genic_upstream_transcript_variant,intron_variant
rs1298611584	genic_upstream_transcript_variant,intron_variant,upstream_transcript_variant
rs1298644628	genic_upstream_transcript_variant,intron_variant
rs1298650856	genic_upstream_transcript_variant,intron_variant
rs1298672205	intron_variant,genic_downstream_transcript_variant
rs1298692173	genic_upstream_transcript_variant,intron_variant
rs1298727386	intron_variant
rs1298757306	genic_upstream_transcript_variant,intron_variant
rs1298778672	genic_upstream_transcript_variant,intron_variant,upstream_transcript_variant,2KB_upstream_variant
rs1298815843	genic_upstream_transcript_variant,intron_variant
rs1298884227	intron_variant,genic_downstream_transcript_variant
rs1298918119	intron_variant
rs1298921406	intron_variant,genic_downstream_transcript_variant
rs1298941365	genic_upstream_transcript_variant,intron_variant
rs1298950610	non_coding_transcript_variant,genic_downstream_transcript_variant,3_prime_UTR_variant
rs1298963318	genic_upstream_transcript_variant,intron_variant
rs1298998515	intron_variant

rs1298999049	intron_variant
rs1299041988	intron_variant,genic_downstream_transcript_variant
rs1299121703	genic_upstream_transcript_variant,intron_variant
rs1299130122	coding_sequence_variant,non_coding_transcript_variant,missense_variant
rs1299157175	intron_variant
rs1299175587	intron_variant
rs1299199370	intron_variant
rs1299231404	genic_upstream_transcript_variant,intron_variant
rs1299248664	intron_variant
rs1299259688	genic_upstream_transcript_variant,intron_variant
rs1299260910	genic_upstream_transcript_variant,intron_variant
rs1299264174	intron_variant
rs1299278209	genic_upstream_transcript_variant,intron_variant
rs1299287391	intron_variant
rs1299336980	intron_variant
rs1299352528	intron_variant
rs1299352731	genic_upstream_transcript_variant,intron_variant
rs1299364568	genic_upstream_transcript_variant,intron_variant,upstream_transcript_variant
rs1299446291	genic_upstream_transcript_variant,intron_variant
rs1299454234	genic_upstream_transcript_variant,intron_variant
rs1299468610	intron_variant
rs1299503920	genic_upstream_transcript_variant,intron_variant
rs1299568369	intron_variant,genic_downstream_transcript_variant
rs1299597332	500B_downstream_variant,downstream_transcript_variant
rs1299627198	intron_variant
rs1299730672	genic_upstream_transcript_variant,intron_variant
rs1299734798	genic_upstream_transcript_variant,intron_variant
rs1299745767	intron_variant
rs1299784649	intron_variant
rs1299865692	intron_variant
rs1299900374	intron_variant
rs1299926681	intron_variant
rs1299952832	intron_variant
rs1299974297	genic_upstream_transcript_variant,intron_variant
rs1300019852	intron_variant
rs1300119557	genic_upstream_transcript_variant,intron_variant
rs1300129575	genic_upstream_transcript_variant,intron_variant
rs1300134171	intron_variant
rs1300168011	intron_variant
rs1300169869	genic_upstream_transcript_variant,intron_variant,upstream_transcript_variant,2KB_upstream_variant
rs1300169905	genic_upstream_transcript_variant,intron_variant,upstream_transcript_variant,2KB_upstream_variant
rs1300172131	genic_upstream_transcript_variant,intron_variant
rs1300181890	genic_upstream_transcript_variant,intron_variant
rs1300213412	genic_upstream_transcript_variant,intron_variant
rs1300235374	genic_upstream_transcript_variant,intron_variant
rs1300239116	genic_upstream_transcript_variant,intron_variant
rs1300264803	intron_variant,genic_downstream_transcript_variant
rs1300275398	genic_upstream_transcript_variant,intron_variant,upstream_transcript_variant,2KB_upstream_variant
rs1300316498	intron_variant
rs1300328824	intron_variant
rs1300371827	genic_upstream_transcript_variant,intron_variant
rs1300390885	intron_variant
rs1300411958	genic_upstream_transcript_variant,intron_variant
rs1300431535	intron_variant,genic_downstream_transcript_variant
rs1300449590	intron_variant
rs1300468749	intron_variant,genic_downstream_transcript_variant
rs1300476012	genic_upstream_transcript_variant,intron_variant
rs1300538176	intron_variant
rs1300547026	genic_upstream_transcript_variant,intron_variant,upstream_transcript_variant
rs1300630793	genic_upstream_transcript_variant,intron_variant,upstream_transcript_variant,2KB_upstream_variant
rs1300665336	intron_variant
rs1300671802	intron_variant
rs1300689038	genic_upstream_transcript_variant,intron_variant
rs1300705414	intron_variant
rs1300749335	intron_variant,genic_downstream_transcript_variant
rs1300783422	genic_upstream_transcript_variant,intron_variant
rs1300795206	intron_variant
rs1300799286	intron_variant
rs1300864006	genic_upstream_transcript_variant,intron_variant
rs1300895037	genic_upstream_transcript_variant,intron_variant
rs1300912120	genic_upstream_transcript_variant,intron_variant
rs1300951436	intron_variant
rs1300961648	coding_sequence_variant,intron_variant,missense_variant,genic_upstream_transcript_variant,non_coding_transcript_variant
rs1301004112	genic_upstream_transcript_variant,intron_variant

rs1301005022	genic_upstream_transcript_variant,intron_variant
rs1301025144	genic_upstream_transcript_variant,intron_variant
rs1301028854	intron_variant
rs1301078079	genic_upstream_transcript_variant,intron_variant,upstream_transcript_variant,2KB_upstream_variant
rs1301098090	intron_variant
rs1301135165	intron_variant,genic_downstream_transcript_variant
rs1301135389	intron_variant
rs1301160434	intron_variant,genic_downstream_transcript_variant
rs1301188696	genic_upstream_transcript_variant,intron_variant,upstream_transcript_variant,2KB_upstream_variant
rs1301250920	intron_variant,genic_downstream_transcript_variant
rs1301278281	intron_variant
rs1301299167	intron_variant
rs1301312767	intron_variant
rs1301375929	intron_variant,genic_downstream_transcript_variant
rs1301381855	intron_variant
rs1301400156	genic_upstream_transcript_variant,intron_variant,upstream_transcript_variant,2KB_upstream_variant
rs1301439144	genic_upstream_transcript_variant,intron_variant,upstream_transcript_variant
rs1301476969	intron_variant
rs1301491730	genic_upstream_transcript_variant,intron_variant,upstream_transcript_variant
rs1301503021	non_coding_transcript_variant,genic_downstream_transcript_variant,3_prime_UTR_variant
rs1301537010	genic_upstream_transcript_variant,intron_variant
rs1301567749	intron_variant
rs1301609205	genic_upstream_transcript_variant,intron_variant
rs1301648576	intron_variant
rs1301654793	genic_upstream_transcript_variant,intron_variant
rs1301682613	genic_upstream_transcript_variant,intron_variant
rs1301726121	genic_upstream_transcript_variant,intron_variant
rs1301733471	genic_upstream_transcript_variant,intron_variant
rs1301750241	coding_sequence_variant,non_coding_transcript_variant,missense_variant
rs1301774834	intron_variant
rs1301775134	intron_variant
rs1301784900	genic_upstream_transcript_variant,intron_variant
rs1301785827	intron_variant,genic_downstream_transcript_variant
rs1301787596	intron_variant
rs1301801700	intron_variant
rs1301871408	genic_upstream_transcript_variant,intron_variant
rs1301872536	genic_upstream_transcript_variant,intron_variant
rs1301883404	intron_variant,genic_downstream_transcript_variant
rs1301887137	intron_variant
rs1301919237	intron_variant
rs1301986324	genic_upstream_transcript_variant,intron_variant
rs1302014964	genic_upstream_transcript_variant,intron_variant
rs1302028245	intron_variant
rs1302033618	intron_variant
rs1302038623	intron_variant
rs1302052221	intron_variant
rs1302076455	intron_variant
rs1302084910	genic_upstream_transcript_variant,intron_variant
rs1302088477	intron_variant
rs1302088589	genic_upstream_transcript_variant,intron_variant,upstream_transcript_variant,2KB_upstream_variant
rs1302090619	intron_variant,genic_downstream_transcript_variant
rs1302133512	genic_upstream_transcript_variant,intron_variant
rs1302143378	genic_upstream_transcript_variant,intron_variant
rs1302148918	genic_upstream_transcript_variant,intron_variant
rs1302153704	intron_variant,genic_downstream_transcript_variant
rs1302192513	intron_variant,genic_downstream_transcript_variant
rs1302249211	intron_variant
rs1302260541	genic_upstream_transcript_variant,intron_variant
rs1302260616	genic_upstream_transcript_variant,intron_variant
rs1302329593	intron_variant
rs1302368858	genic_upstream_transcript_variant,intron_variant
rs1302371805	intron_variant
rs1302379818	intron_variant,genic_downstream_transcript_variant
rs1302398779	genic_upstream_transcript_variant,intron_variant,upstream_transcript_variant
rs1302427603	genic_upstream_transcript_variant,intron_variant
rs1302438869	intron_variant
rs1302499371	intron_variant,genic_downstream_transcript_variant
rs1302507340	intron_variant
rs1302512681	intron_variant
rs1302513833	genic_upstream_transcript_variant,intron_variant
rs1302558720	genic_upstream_transcript_variant,intron_variant,upstream_transcript_variant,2KB_upstream_variant
rs1302587458	genic_upstream_transcript_variant,intron_variant
rs1302594027	genic_upstream_transcript_variant,intron_variant,upstream_transcript_variant,2KB_upstream_variant
rs1302713204	non_coding_transcript_variant,genic_downstream_transcript_variant,3_prime_UTR_variant

rs1302743882	genic_upstream_transcript_variant,intron_variant,upstream_transcript_variant
rs1302746288	genic_upstream_transcript_variant,intron_variant
rs1302768282	intron_variant,genic_downstream_transcript_variant
rs1302786361	genic_upstream_transcript_variant,intron_variant
rs1302798523	genic_upstream_transcript_variant,intron_variant
rs1302838145	genic_upstream_transcript_variant,intron_variant
rs1302913460	genic_upstream_transcript_variant,intron_variant
rs1302914221	intron_variant
rs1302943389	intron_variant
rs1302982272	genic_upstream_transcript_variant,intron_variant
rs1303026120	intron_variant
rs1303042681	genic_upstream_transcript_variant,intron_variant
rs1303051316	intron_variant
rs1303054061	genic_upstream_transcript_variant,intron_variant
rs1303063845	genic_upstream_transcript_variant,intron_variant
rs1303163189	intron_variant
rs1303177815	genic_upstream_transcript_variant,intron_variant
rs1303181366	intron_variant,genic_downstream_transcript_variant
rs1303201906	intron_variant
rs1303212008	genic_upstream_transcript_variant,intron_variant
rs1303216504	genic_upstream_transcript_variant,intron_variant
rs1303255651	intron_variant
rs1303257051	intron_variant,genic_downstream_transcript_variant
rs1303287027	intron_variant
rs1303332788	intron_variant
rs1303347578	genic_upstream_transcript_variant,intron_variant
rs1303357710	intron_variant
rs1303395409	intron_variant
rs1303404657	intron_variant,genic_downstream_transcript_variant
rs1303496010	intron_variant
rs1303514988	genic_upstream_transcript_variant,intron_variant
rs1303531946	genic_upstream_transcript_variant,intron_variant
rs1303554896	intron_variant
rs1303568851	genic_upstream_transcript_variant,intron_variant
rs1303621696	genic_upstream_transcript_variant,intron_variant,upstream_transcript_variant,2KB_upstream_variant
rs1303704009	genic_upstream_transcript_variant,intron_variant
rs1303708620	genic_upstream_transcript_variant,intron_variant
rs1303752575	intron_variant
rs1303755887	genic_upstream_transcript_variant,intron_variant
rs1303869254	intron_variant
rs1303881810	genic_upstream_transcript_variant,intron_variant,upstream_transcript_variant,2KB_upstream_variant
rs1303903534	non_coding_transcript_variant,genic_downstream_transcript_variant,3_prime_UTR_variant
rs1303933628	intron_variant,genic_downstream_transcript_variant
rs1303960816	intron_variant
rs1303977348	intron_variant
rs1303985986	intron_variant,genic_downstream_transcript_variant
rs1304094423	intron_variant,genic_downstream_transcript_variant
rs1304162063	intron_variant
rs1304166225	genic_upstream_transcript_variant,intron_variant,upstream_transcript_variant,2KB_upstream_variant
rs1304207319	genic_upstream_transcript_variant,intron_variant
rs1304217978	genic_upstream_transcript_variant,intron_variant
rs1304235575	intron_variant
rs1304260049	genic_upstream_transcript_variant,intron_variant,upstream_transcript_variant,2KB_upstream_variant
rs1304271896	intron_variant,genic_downstream_transcript_variant
rs1304317163	genic_upstream_transcript_variant,intron_variant
rs1304331130	intron_variant
rs1304382571	intron_variant
rs1304433160	intron_variant
rs1304460497	coding_sequence_variant,non_coding_transcript_variant,missense_variant
rs1304475236	intron_variant
rs1304595463	genic_upstream_transcript_variant,intron_variant
rs1304601935	intron_variant
rs1304603842	genic_upstream_transcript_variant,intron_variant
rs1304607970	intron_variant,genic_downstream_transcript_variant
rs1304662662	genic_upstream_transcript_variant,intron_variant
rs1304672600	genic_upstream_transcript_variant,intron_variant
rs1304696380	intron_variant,genic_downstream_transcript_variant
rs1304741783	intron_variant
rs1304762731	genic_upstream_transcript_variant,intron_variant
rs1304765887	coding_sequence_variant,genic_downstream_transcript_variant,missense_variant,synonymous_variant,non_coding_transcript_variant
rs1304765992	genic_upstream_transcript_variant,intron_variant
rs1304818904	intron_variant,genic_downstream_transcript_variant
rs1304820471	genic_upstream_transcript_variant,intron_variant
rs1304837412	intron_variant

rs1304854937	genic_upstream_transcript_variant,intron_variant
rs1304940256	genic_upstream_transcript_variant,intron_variant
rs1304943665	intron_variant,genic_downstream_transcript_variant
rs1304951695	genic_upstream_transcript_variant,intron_variant,upstream_transcript_variant,2KB_upstream_variant
rs1304993591	intron_variant
rs1305071497	intron_variant
rs1305088304	intron_variant
rs1305168527	genic_upstream_transcript_variant,intron_variant
rs1305174716	coding_sequence_variant,intron_variant,missense_variant,synonymous_variant,genic_upstream_transcript_variant,non_coding_transcript_variant
rs1305212965	genic_upstream_transcript_variant,intron_variant
rs1305253671	genic_upstream_transcript_variant,intron_variant
rs1305282357	genic_upstream_transcript_variant,intron_variant
rs1305286405	genic_upstream_transcript_variant,intron_variant
rs1305329009	intron_variant
rs1305339099	intron_variant
rs1305369264	genic_upstream_transcript_variant,intron_variant
rs1305383482	intron_variant
rs1305397162	intron_variant
rs1305411309	genic_upstream_transcript_variant,intron_variant
rs1305437290	genic_upstream_transcript_variant,intron_variant
rs1305448757	intron_variant
rs1305467807	genic_upstream_transcript_variant,intron_variant
rs1305528287	genic_upstream_transcript_variant,intron_variant
rs1305535640	intron_variant
rs1305553070	intron_variant
rs1305582926	intron_variant
rs1305594217	genic_upstream_transcript_variant,intron_variant
rs1305628153	genic_upstream_transcript_variant,intron_variant
rs1305691952	intron_variant
rs1305708815	intron_variant
rs1305709349	genic_upstream_transcript_variant,intron_variant
rs1305710880	intron_variant
rs1305714796	genic_upstream_transcript_variant,intron_variant
rs1305750674	genic_upstream_transcript_variant,intron_variant
rs1305757836	intron_variant,genic_downstream_transcript_variant
rs1305764767	intron_variant
rs1305767571	intron_variant
rs1305791402	intron_variant
rs1305794529	genic_upstream_transcript_variant,intron_variant
rs1305836795	intron_variant
rs1305852593	genic_upstream_transcript_variant,intron_variant
rs1305855213	genic_upstream_transcript_variant,intron_variant,upstream_transcript_variant
rs1305943122	intron_variant
rs1305958071	intron_variant
rs1305966614	genic_upstream_transcript_variant,intron_variant
rs1305999315	intron_variant
rs1306050081	intron_variant
rs1306054463	non_coding_transcript_variant,genic_downstream_transcript_variant,3_prime_UTR_variant
rs1306081840	coding_sequence_variant,non_coding_transcript_variant,missense_variant
rs1306125971	intron_variant
rs1306187277	intron_variant,genic_downstream_transcript_variant
rs1306197496	intron_variant
rs1306257882	genic_upstream_transcript_variant,intron_variant,upstream_transcript_variant,2KB_upstream_variant
rs1306317694	genic_upstream_transcript_variant,intron_variant
rs1306385360	intron_variant
rs1306430151	intron_variant
rs1306435940	intron_variant,genic_downstream_transcript_variant
rs1306455130	genic_upstream_transcript_variant,intron_variant
rs1306457178	genic_upstream_transcript_variant,intron_variant,upstream_transcript_variant,2KB_upstream_variant
rs1306503770	genic_upstream_transcript_variant,intron_variant
rs1306513029	intron_variant
rs1306539083	intron_variant,genic_downstream_transcript_variant
rs1306554431	intron_variant
rs1306596278	intron_variant
rs1306648033	genic_upstream_transcript_variant,intron_variant
rs1306702084	intron_variant
rs1306743022	non_coding_transcript_variant,genic_downstream_transcript_variant,3_prime_UTR_variant
rs1306790234	intron_variant,genic_downstream_transcript_variant
rs1306834857	intron_variant
rs1306867252	intron_variant
rs1306895692	genic_upstream_transcript_variant,intron_variant
rs1306906825	genic_upstream_transcript_variant,intron_variant
rs1306934322	intron_variant
rs1306976105	intron_variant

rs1307003274	genic_upstream_transcript_variant,intron_variant
rs1307026003	genic_upstream_transcript_variant,intron_variant,upstream_transcript_variant
rs1307037078	genic_upstream_transcript_variant,intron_variant
rs1307040952	500B_downstream_variant,downstream_transcript_variant
rs1307042860	genic_upstream_transcript_variant,intron_variant
rs1307047293	intron_variant
rs1307076159	genic_upstream_transcript_variant,intron_variant
rs1307081926	intron_variant
rs1307094547	intron_variant,genic_downstream_transcript_variant
rs1307112964	genic_upstream_transcript_variant,intron_variant
rs1307141797	genic_upstream_transcript_variant,intron_variant
rs1307144196	genic_upstream_transcript_variant,intron_variant
rs1307177608	intron_variant
rs1307235391	intron_variant
rs1307248519	genic_upstream_transcript_variant,intron_variant
rs1307253611	genic_upstream_transcript_variant,intron_variant
rs1307260687	genic_upstream_transcript_variant,intron_variant,upstream_transcript_variant,2KB_upstream_variant
rs1307264647	genic_upstream_transcript_variant,intron_variant,upstream_transcript_variant
rs1307376941	intron_variant
rs1307414526	genic_upstream_transcript_variant,intron_variant
rs1307418443	intron_variant,genic_downstream_transcript_variant
rs1307465012	intron_variant
rs1307492478	genic_upstream_transcript_variant,intron_variant,upstream_transcript_variant,2KB_upstream_variant
rs1307494294	genic_upstream_transcript_variant,intron_variant
rs1307542495	genic_upstream_transcript_variant,intron_variant
rs1307547811	genic_upstream_transcript_variant,intron_variant
rs1307584609	coding_sequence_variant,non_coding_transcript_variant,missense_variant
rs1307625015	genic_upstream_transcript_variant,intron_variant
rs1307690544	intron_variant
rs1307753319	genic_upstream_transcript_variant,intron_variant
rs1307755063	genic_upstream_transcript_variant,intron_variant
rs1307757992	intron_variant
rs1307806710	intron_variant
rs1307819444	intron_variant
rs1307824723	intron_variant
rs1307859660	genic_upstream_transcript_variant,intron_variant
rs1307883185	intron_variant
rs1307909222	intron_variant
rs1307937187	genic_upstream_transcript_variant,intron_variant
rs1307976629	intron_variant
rs1308048296	intron_variant
rs1308048791	genic_upstream_transcript_variant,intron_variant
rs1308060081	genic_upstream_transcript_variant,intron_variant
rs1308104382	intron_variant,genic_downstream_transcript_variant
rs1308111433	intron_variant
rs1308148206	intron_variant
rs1308154994	genic_upstream_transcript_variant,intron_variant
rs1308162642	non_coding_transcript_variant,genic_downstream_transcript_variant,3_prime_UTR_variant
rs1308210171	intron_variant,genic_downstream_transcript_variant
rs1308227428	genic_upstream_transcript_variant,intron_variant,upstream_transcript_variant
rs1308281877	genic_upstream_transcript_variant,intron_variant
rs1308293195	genic_upstream_transcript_variant,intron_variant
rs1308331627	intron_variant
rs1308335923	genic_upstream_transcript_variant,intron_variant
rs1308356152	genic_upstream_transcript_variant,intron_variant
rs1308374006	intron_variant,genic_downstream_transcript_variant
rs1308414121	coding_sequence_variant,non_coding_transcript_variant,inframe_indel,stop_gained
rs1308423158	genic_upstream_transcript_variant,intron_variant
rs1308429703	intron_variant,genic_downstream_transcript_variant
rs1308457568	intron_variant
rs1308494335	intron_variant,5_prime_UTR_variant
rs1308499090	genic_upstream_transcript_variant,intron_variant
rs1308532501	intron_variant
rs1308549163	intron_variant,genic_downstream_transcript_variant
rs1308563209	genic_upstream_transcript_variant,intron_variant,upstream_transcript_variant
rs1308584251	intron_variant
rs1308600899	intron_variant,genic_downstream_transcript_variant
rs1308626323	genic_upstream_transcript_variant,intron_variant,upstream_transcript_variant,5_prime_UTR_variant
rs1308637291	genic_upstream_transcript_variant,intron_variant
rs1308660021	intron_variant
rs1308720709	genic_upstream_transcript_variant,intron_variant
rs1308726301	intron_variant
rs1308749435	genic_upstream_transcript_variant,intron_variant
rs1308773055	genic_upstream_transcript_variant,intron_variant,upstream_transcript_variant,2KB_upstream_variant

rs1308826106	genic_upstream_transcript_variant,intron_variant
rs1308844944	genic_upstream_transcript_variant,intron_variant
rs1308849438	genic_upstream_transcript_variant,intron_variant
rs1308865312	intron_variant,genic_upstream_transcript_variant
rs1308911093	intron_variant,genic_upstream_transcript_variant
rs1308931094	intron_variant,genic_upstream_transcript_variant
rs1309051082	intron_variant
rs1309099009	intron_variant
rs1309145893	intron_variant,genic_upstream_transcript_variant
rs1309165130	intron_variant,non_coding_transcript_variant,genic_upstream_transcript_variant,5_prime_UTR_variant
rs1309183570	intron_variant,genic_upstream_transcript_variant
rs1309236031	non_coding_transcript_variant,intron_variant,genic_upstream_transcript_variant,coding_sequence_variant,missense_variant
rs1309292692	intron_variant,genic_upstream_transcript_variant
rs1309304637	intron_variant,genic_upstream_transcript_variant
rs1309343067	intron_variant
rs1309379022	intron_variant,genic_upstream_transcript_variant
rs1309380958	intron_variant,genic_upstream_transcript_variant
rs1309429273	intron_variant,genic_downstream_transcript_variant
rs1309447259	intron_variant,upstream_transcript_variant,genic_upstream_transcript_variant
rs1309479864	intron_variant
rs1309496490	intron_variant,genic_downstream_transcript_variant
rs1309565491	intron_variant,genic_downstream_transcript_variant
rs1309611040	intron_variant,genic_upstream_transcript_variant
rs1309616793	intron_variant,genic_downstream_transcript_variant
rs1309638201	non_coding_transcript_variant,synonymous_variant,genic_downstream_transcript_variant,coding_sequence_variant
rs1309650693	intron_variant,genic_upstream_transcript_variant
rs1309656283	intron_variant
rs1309668249	intron_variant,genic_upstream_transcript_variant
rs1309668523	intron_variant
rs1309693976	intron_variant,upstream_transcript_variant,genic_upstream_transcript_variant
rs1309713208	intron_variant
rs1309716165	intron_variant,genic_upstream_transcript_variant
rs1309737976	non_coding_transcript_variant,3_prime_UTR_variant,genic_downstream_transcript_variant
rs1309746473	intron_variant,genic_upstream_transcript_variant
rs1309754764	intron_variant
rs1309768143	intron_variant
rs1309786469	intron_variant,genic_upstream_transcript_variant
rs1309786924	intron_variant
rs1309836638	intron_variant,genic_downstream_transcript_variant
rs1309863765	intron_variant
rs1309886126	intron_variant,genic_downstream_transcript_variant
rs1309922132	intron_variant
rs1309976931	intron_variant,genic_upstream_transcript_variant
rs1309999371	intron_variant,2KB_upstream_variant,upstream_transcript_variant,genic_upstream_transcript_variant
rs1310082688	intron_variant,upstream_transcript_variant,genic_upstream_transcript_variant
rs1310102033	intron_variant,genic_upstream_transcript_variant
rs1310132990	intron_variant,genic_upstream_transcript_variant
rs1310173093	intron_variant,genic_upstream_transcript_variant
rs1310174152	intron_variant,upstream_transcript_variant,genic_upstream_transcript_variant
rs1310174699	intron_variant,genic_upstream_transcript_variant
rs1310190422	intron_variant,genic_downstream_transcript_variant
rs1310197846	intron_variant,genic_upstream_transcript_variant
rs1310198865	intron_variant,genic_upstream_transcript_variant
rs1310238376	intron_variant
rs1310260084	intron_variant,genic_upstream_transcript_variant
rs1310261782	500B_downstream_variant,downstream_transcript_variant
rs1310273754	intron_variant
rs1310321706	intron_variant,genic_upstream_transcript_variant
rs1310363757	intron_variant,genic_downstream_transcript_variant
rs1310372387	intron_variant,genic_downstream_transcript_variant
rs1310385514	intron_variant
rs1310393391	intron_variant
rs1310409843	intron_variant,genic_upstream_transcript_variant
rs1310473164	intron_variant
rs1310487412	intron_variant
rs1310503223	intron_variant,genic_upstream_transcript_variant
rs1310591058	intron_variant
rs1310598349	intron_variant,genic_upstream_transcript_variant
rs1310613010	non_coding_transcript_variant,3_prime_UTR_variant,genic_downstream_transcript_variant
rs1310619482	intron_variant,missense_variant,coding_sequence_variant
rs1310643465	intron_variant,genic_downstream_transcript_variant
rs1310651658	intron_variant,non_coding_transcript_variant,genic_upstream_transcript_variant,5_prime_UTR_variant
rs1310710739	intron_variant,genic_upstream_transcript_variant
rs1310741467	intron_variant,genic_downstream_transcript_variant

rs1310747650	intron_variant,genic_upstream_transcript_variant
rs1310786944	intron_variant,genic_upstream_transcript_variant
rs1310800288	intron_variant
rs1310803113	intron_variant,genic_upstream_transcript_variant
rs1310832219	non_coding_transcript_variant,synonymous_variant,genic_downstream_transcript_variant,coding_sequence_variant
rs1310887358	intron_variant,genic_upstream_transcript_variant
rs1310887943	intron_variant,genic_upstream_transcript_variant
rs1310903240	intron_variant
rs1310908391	intron_variant
rs1310910925	intron_variant,genic_upstream_transcript_variant
rs1310964979	non_coding_transcript_variant,3_prime_UTR_variant,genic_downstream_transcript_variant
rs1310987823	intron_variant,genic_upstream_transcript_variant
rs1311030513	intron_variant
rs1311050999	intron_variant,genic_upstream_transcript_variant
rs1311091263	intron_variant
rs1311094713	intron_variant,genic_upstream_transcript_variant
rs1311145277	intron_variant,genic_upstream_transcript_variant
rs1311158792	intron_variant,upstream_transcript_variant,genic_upstream_transcript_variant
rs1311171795	intron_variant
rs1311172244	intron_variant
rs1311187997	intron_variant
rs1311241742	intron_variant,genic_upstream_transcript_variant
rs1311257743	intron_variant,genic_downstream_transcript_variant
rs1311260141	intron_variant,genic_upstream_transcript_variant
rs1311298215	intron_variant,genic_downstream_transcript_variant
rs1311308159	intron_variant
rs1311389149	intron_variant
rs1311442112	intron_variant,genic_upstream_transcript_variant
rs1311461295	intron_variant,genic_downstream_transcript_variant
rs1311467716	intron_variant
rs1311477661	intron_variant
rs1311521382	intron_variant
rs1311569481	intron_variant
rs1311605781	intron_variant,genic_upstream_transcript_variant
rs1311644064	intron_variant
rs1311693340	intron_variant
rs1311728402	intron_variant
rs1311742622	intron_variant,genic_upstream_transcript_variant
rs1311768807	intron_variant,genic_downstream_transcript_variant
rs1311773989	intron_variant,2KB_upstream_variant,upstream_transcript_variant,genic_upstream_transcript_variant
rs1311836650	intron_variant
rs1311850323	intron_variant,genic_upstream_transcript_variant
rs1311922319	intron_variant
rs1311936526	intron_variant,genic_upstream_transcript_variant
rs1311972922	intron_variant
rs1311983597	missense_variant,non_coding_transcript_variant,genic_downstream_transcript_variant,coding_sequence_variant
rs1311998419	intron_variant,genic_downstream_transcript_variant
rs1312007874	intron_variant
rs1312042851	intron_variant,genic_upstream_transcript_variant
rs1312106881	intron_variant
rs1312139095	intron_variant,genic_downstream_transcript_variant
rs1312144662	intron_variant,genic_upstream_transcript_variant
rs1312156280	intron_variant,genic_upstream_transcript_variant
rs1312159802	intron_variant,genic_upstream_transcript_variant
rs1312170986	intron_variant,genic_downstream_transcript_variant
rs1312171732	intron_variant,genic_downstream_transcript_variant
rs1312252459	intron_variant,genic_upstream_transcript_variant
rs1312286749	intron_variant,genic_downstream_transcript_variant
rs1312300932	intron_variant
rs1312322680	intron_variant,genic_downstream_transcript_variant
rs1312392351	intron_variant,genic_downstream_transcript_variant
rs1312439984	intron_variant,genic_downstream_transcript_variant
rs1312503717	intron_variant,genic_upstream_transcript_variant
rs1312515497	intron_variant
rs1312527529	intron_variant,genic_upstream_transcript_variant
rs1312532343	intron_variant,upstream_transcript_variant,genic_upstream_transcript_variant
rs1312593517	intron_variant,2KB_upstream_variant,upstream_transcript_variant,genic_upstream_transcript_variant
rs1312596041	intron_variant,genic_upstream_transcript_variant
rs1312641375	intron_variant,genic_upstream_transcript_variant
rs1312699411	intron_variant,genic_downstream_transcript_variant
rs1312719048	intron_variant
rs1312761234	intron_variant
rs1312781407	intron_variant
rs1312798093	intron_variant,genic_upstream_transcript_variant

rs1312816921	intron_variant,genic_upstream_transcript_variant
rs1312838552	intron_variant,genic_downstream_transcript_variant
rs1312860654	intron_variant
rs1312910217	intron_variant,genic_upstream_transcript_variant
rs1312923839	intron_variant,genic_upstream_transcript_variant
rs1313008707	intron_variant
rs1313032767	intron_variant
rs1313040813	intron_variant,genic_upstream_transcript_variant
rs1313096882	intron_variant
rs1313121708	intron_variant,genic_upstream_transcript_variant
rs1313136198	intron_variant,genic_downstream_transcript_variant
rs1313150532	intron_variant
rs1313161693	intron_variant
rs1313162007	intron_variant,genic_upstream_transcript_variant
rs1313206098	intron_variant,genic_downstream_transcript_variant
rs1313227253	intron_variant,genic_upstream_transcript_variant
rs1313241609	intron_variant,genic_upstream_transcript_variant
rs1313257892	intron_variant,genic_upstream_transcript_variant
rs1313290480	intron_variant,genic_upstream_transcript_variant
rs1313293093	intron_variant,genic_upstream_transcript_variant
rs1313361854	intron_variant
rs1313395317	intron_variant,genic_downstream_transcript_variant
rs1313408176	intron_variant,genic_upstream_transcript_variant
rs1313420103	intron_variant,genic_upstream_transcript_variant
rs1313449372	intron_variant,genic_downstream_transcript_variant
rs1313499311	intron_variant
rs1313512551	intron_variant
rs1313568976	intron_variant,genic_downstream_transcript_variant
rs1313596143	intron_variant,genic_upstream_transcript_variant
rs1313606827	intron_variant,genic_upstream_transcript_variant
rs1313679643	intron_variant
rs1313700078	intron_variant
rs1313706663	intron_variant,genic_upstream_transcript_variant
rs1313768228	intron_variant
rs1313787614	intron_variant,genic_upstream_transcript_variant
rs1313841562	intron_variant,genic_upstream_transcript_variant
rs1313939591	intron_variant,genic_upstream_transcript_variant
rs1313942631	intron_variant
rs1313948364	intron_variant,genic_upstream_transcript_variant
rs1313955104	intron_variant
rs1313961511	intron_variant
rs1313972395	intron_variant
rs1314014455	intron_variant,genic_upstream_transcript_variant
rs1314045268	intron_variant,genic_upstream_transcript_variant
rs1314066690	intron_variant,genic_upstream_transcript_variant
rs1314080905	intron_variant
rs1314084671	intron_variant,genic_upstream_transcript_variant
rs1314101981	intron_variant
rs1314103016	intron_variant,genic_downstream_transcript_variant
rs1314210311	intron_variant,genic_downstream_transcript_variant
rs1314210830	intron_variant,genic_downstream_transcript_variant
rs1314212790	intron_variant,genic_upstream_transcript_variant
rs1314242184	intron_variant,genic_upstream_transcript_variant
rs1314244685	intron_variant
rs1314246978	intron_variant,genic_upstream_transcript_variant
rs1314253250	intron_variant,genic_downstream_transcript_variant
rs1314254870	intron_variant,genic_downstream_transcript_variant
rs1314265312	intron_variant
rs1314299624	intron_variant,2KB_upstream_variant,upstream_transcript_variant,genic_upstream_transcript_variant
rs1314316832	intron_variant
rs1314349950	intron_variant,genic_downstream_transcript_variant
rs1314351657	intron_variant,genic_upstream_transcript_variant
rs1314362625	intron_variant,2KB_upstream_variant,upstream_transcript_variant,genic_upstream_transcript_variant
rs1314389070	intron_variant
rs1314433862	intron_variant,genic_upstream_transcript_variant
rs1314460059	intron_variant
rs1314471932	intron_variant
rs1314485150	intron_variant
rs1314499806	intron_variant,genic_upstream_transcript_variant
rs1314501420	intron_variant,genic_upstream_transcript_variant
rs1314566498	intron_variant,genic_downstream_transcript_variant
rs1314580840	intron_variant,genic_upstream_transcript_variant
rs1314596653	intron_variant,genic_downstream_transcript_variant
rs1314633310	intron_variant,genic_upstream_transcript_variant

rs1314660916	intron_variant
rs1314673705	intron_variant,genic_downstream_transcript_variant
rs1314714445	intron_variant,genic_upstream_transcript_variant
rs1314723459	intron_variant,upstream_transcript_variant,genic_upstream_transcript_variant
rs1314733877	intron_variant
rs1314775000	intron_variant,genic_upstream_transcript_variant
rs1314812346	intron_variant,genic_upstream_transcript_variant
rs1314827480	intron_variant,2KB_upstream_variant,upstream_transcript_variant,genic_upstream_transcript_variant
rs1314836783	intron_variant,genic_downstream_transcript_variant
rs1314847440	intron_variant,genic_upstream_transcript_variant
rs1314882291	intron_variant,genic_upstream_transcript_variant
rs1314886088	intron_variant,genic_downstream_transcript_variant
rs1314890057	intron_variant
rs1314904039	intron_variant
rs1314918749	intron_variant
rs1314949728	intron_variant
rs1314952457	intron_variant,genic_downstream_transcript_variant
rs1314965037	intron_variant
rs1314967077	intron_variant,genic_downstream_transcript_variant
rs1314969875	intron_variant,genic_upstream_transcript_variant
rs1314996451	intron_variant,genic_upstream_transcript_variant
rs1315052534	intron_variant
rs1315070148	intron_variant,genic_upstream_transcript_variant
rs1315101846	intron_variant,genic_upstream_transcript_variant
rs1315105938	intron_variant
rs1315122312	intron_variant,genic_upstream_transcript_variant
rs1315135764	intron_variant
rs1315193738	intron_variant,genic_upstream_transcript_variant
rs1315217323	intron_variant,genic_upstream_transcript_variant
rs1315301481	intron_variant
rs1315302912	intron_variant
rs1315323526	intron_variant,genic_upstream_transcript_variant
rs1315366426	intron_variant
rs1315373642	intron_variant
rs1315407112	intron_variant,genic_downstream_transcript_variant
rs1315454302	intron_variant
rs1315460255	intron_variant,2KB_upstream_variant,upstream_transcript_variant,genic_upstream_transcript_variant
rs1315468146	intron_variant,genic_upstream_transcript_variant
rs1315473226	intron_variant,2KB_upstream_variant,upstream_transcript_variant,genic_upstream_transcript_variant
rs1315475370	intron_variant,upstream_transcript_variant,genic_upstream_transcript_variant
rs1315485265	intron_variant,genic_downstream_transcript_variant
rs1315489413	intron_variant,genic_upstream_transcript_variant
rs1315499829	intron_variant,genic_downstream_transcript_variant
rs1315508035	intron_variant,genic_upstream_transcript_variant
rs1315520596	non_coding_transcript_variant,3_prime_UTR_variant,genic_downstream_transcript_variant
rs1315533938	intron_variant,upstream_transcript_variant,genic_upstream_transcript_variant
rs1315607296	intron_variant,genic_upstream_transcript_variant
rs1315611464	intron_variant,genic_downstream_transcript_variant
rs1315676476	intron_variant,2KB_upstream_variant,upstream_transcript_variant,genic_upstream_transcript_variant
rs1315681743	intron_variant
rs1315718744	intron_variant,genic_downstream_transcript_variant
rs1315732511	intron_variant
rs1315737119	intron_variant
rs1315749816	intron_variant,genic_upstream_transcript_variant
rs1315774152	intron_variant,genic_upstream_transcript_variant
rs1315793874	intron_variant
rs1315794875	intron_variant
rs1315824987	intron_variant,upstream_transcript_variant,genic_upstream_transcript_variant
rs1315845555	intron_variant,genic_upstream_transcript_variant
rs1315893381	intron_variant,genic_upstream_transcript_variant
rs1315973226	intron_variant,genic_downstream_transcript_variant
rs1316028931	intron_variant,genic_upstream_transcript_variant
rs1316047252	intron_variant
rs1316069752	intron_variant,genic_downstream_transcript_variant
rs1316113803	intron_variant,genic_upstream_transcript_variant
rs1316145404	intron_variant
rs1316168791	intron_variant,genic_upstream_transcript_variant
rs1316168915	intron_variant
rs1316188443	intron_variant,genic_downstream_transcript_variant
rs1316298233	intron_variant,genic_upstream_transcript_variant
rs1316323838	intron_variant,genic_downstream_transcript_variant
rs1316330750	intron_variant
rs1316338721	intron_variant
rs1316353233	intron_variant,genic_upstream_transcript_variant

rs1316362726	intron_variant,genic_upstream_transcript_variant
rs1316404138	intron_variant,genic_upstream_transcript_variant
rs1316481945	intron_variant,genic_upstream_transcript_variant
rs1316497333	intron_variant
rs1316500207	intron_variant,genic_downstream_transcript_variant
rs1316548396	intron_variant
rs1316581869	non_coding_transcript_variant,intron_variant,synonymous_variant,genic_upstream_transcript_variant,coding_sequence_variant
rs1316660881	intron_variant,genic_upstream_transcript_variant
rs1316707712	intron_variant,genic_upstream_transcript_variant
rs1316755781	intron_variant,genic_downstream_transcript_variant
rs1316758352	intron_variant,genic_upstream_transcript_variant
rs1316763701	intron_variant,genic_upstream_transcript_variant
rs1316770425	intron_variant
rs1316772781	intron_variant,genic_upstream_transcript_variant
rs1316825870	intron_variant,genic_upstream_transcript_variant
rs1316833722	intron_variant,genic_upstream_transcript_variant
rs1316846123	intron_variant,genic_upstream_transcript_variant
rs1316854950	intron_variant
rs1316926135	intron_variant
rs1316931320	intron_variant,genic_upstream_transcript_variant
rs1317014866	intron_variant,genic_downstream_transcript_variant
rs1317049899	intron_variant
rs1317069464	intron_variant,genic_upstream_transcript_variant
rs1317085466	intron_variant
rs1317096267	intron_variant,genic_downstream_transcript_variant
rs1317100437	intron_variant,genic_upstream_transcript_variant
rs1317141254	intron_variant,genic_downstream_transcript_variant
rs1317143188	intron_variant
rs1317148095	intron_variant,genic_upstream_transcript_variant
rs1317158856	intron_variant,genic_upstream_transcript_variant
rs1317223702	intron_variant,genic_upstream_transcript_variant
rs1317226279	intron_variant,genic_upstream_transcript_variant
rs1317252459	intron_variant
rs1317268165	intron_variant,genic_downstream_transcript_variant
rs1317289343	intron_variant
rs1317304202	intron_variant
rs1317385076	intron_variant
rs1317401028	intron_variant,genic_upstream_transcript_variant
rs1317402858	intron_variant
rs1317434153	intron_variant
rs1317507594	intron_variant,genic_upstream_transcript_variant
rs1317528549	intron_variant
rs1317539640	intron_variant,genic_upstream_transcript_variant
rs1317620369	intron_variant
rs1317631627	intron_variant
rs1317645768	intron_variant,2KB_upstream_variant,upstream_transcript_variant,genic_upstream_transcript_variant
rs1317670956	intron_variant,genic_upstream_transcript_variant
rs1317693557	intron_variant,genic_upstream_transcript_variant
rs1317729018	intron_variant,genic_upstream_transcript_variant
rs1317744056	intron_variant,genic_upstream_transcript_variant
rs1317780444	intron_variant,genic_upstream_transcript_variant
rs1317792157	intron_variant,genic_upstream_transcript_variant
rs1317802984	intron_variant,upstream_transcript_variant,genic_upstream_transcript_variant
rs1317820599	intron_variant
rs1317861730	intron_variant
rs1317867672	intron_variant,genic_upstream_transcript_variant
rs1317895159	non_coding_transcript_variant,3_prime_UTR_variant,genic_downstream_transcript_variant
rs1317898349	intron_variant,genic_upstream_transcript_variant
rs1317917702	intron_variant
rs1317919095	intron_variant,genic_upstream_transcript_variant
rs1317932193	intron_variant
rs1317971041	intron_variant,genic_upstream_transcript_variant
rs1317971935	intron_variant,genic_downstream_transcript_variant
rs1317988356	intron_variant,genic_upstream_transcript_variant
rs1317999170	intron_variant,genic_upstream_transcript_variant
rs1318015893	intron_variant,genic_downstream_transcript_variant
rs1318018459	intron_variant
rs1318041627	intron_variant,genic_upstream_transcript_variant
rs1318044138	intron_variant,genic_upstream_transcript_variant
rs1318093823	intron_variant,genic_downstream_transcript_variant
rs1318101371	intron_variant,genic_upstream_transcript_variant
rs1318112013	intron_variant,genic_upstream_transcript_variant
rs1318129903	intron_variant
rs1318134586	intron_variant

rs1318152785	intron_variant,genic_upstream_transcript_variant
rs1318169583	intron_variant
rs1318190870	intron_variant,genic_upstream_transcript_variant
rs1318193394	intron_variant,upstream_transcript_variant,genic_upstream_transcript_variant
rs1318218096	intron_variant,genic_downstream_transcript_variant
rs1318238016	intron_variant
rs1318238025	intron_variant,upstream_transcript_variant,genic_upstream_transcript_variant
rs1318264169	intron_variant,genic_upstream_transcript_variant
rs1318307677	intron_variant,genic_downstream_transcript_variant
rs1318309834	intron_variant,genic_upstream_transcript_variant
rs1318314904	intron_variant
rs1318317624	intron_variant,genic_upstream_transcript_variant
rs1318335190	intron_variant,genic_upstream_transcript_variant
rs1318361513	intron_variant
rs1318399253	intron_variant,genic_upstream_transcript_variant
rs1318418086	intron_variant
rs1318422004	intron_variant
rs1318484930	intron_variant
rs1318488047	intron_variant
rs1318492672	non_coding_transcript_variant,3_prime_UTR_variant,genic_downstream_transcript_variant
rs1318500804	intron_variant,genic_upstream_transcript_variant
rs1318551704	intron_variant
rs1318611404	synonymous_variant,non_coding_transcript_variant,coding_sequence_variant
rs1318622991	intron_variant,genic_downstream_transcript_variant
rs1318638076	missense_variant,non_coding_transcript_variant,genic_downstream_transcript_variant,coding_sequence_variant
rs1318656483	intron_variant,genic_upstream_transcript_variant
rs1318721401	intron_variant,genic_upstream_transcript_variant
rs1318732623	intron_variant
rs1318759264	intron_variant,genic_downstream_transcript_variant
rs1318767968	intron_variant
rs1318829346	intron_variant
rs1318839357	intron_variant
rs1318848496	intron_variant
rs1318878799	intron_variant
rs1318889211	intron_variant,upstream_transcript_variant,genic_upstream_transcript_variant
rs1318963633	intron_variant,genic_downstream_transcript_variant
rs1318963676	intron_variant
rs1318989606	missense_variant,non_coding_transcript_variant,coding_sequence_variant
rs1319044097	intron_variant,genic_upstream_transcript_variant
rs1319050655	intron_variant,genic_downstream_transcript_variant
rs1319063223	intron_variant,genic_downstream_transcript_variant
rs1319076081	intron_variant,genic_upstream_transcript_variant
rs1319077915	intron_variant
rs1319078911	intron_variant,genic_downstream_transcript_variant
rs1319115034	intron_variant,genic_upstream_transcript_variant
rs1319124374	intron_variant,genic_upstream_transcript_variant
rs1319194264	intron_variant,genic_upstream_transcript_variant
rs1319330211	intron_variant,2KB_upstream_variant,upstream_transcript_variant,genic_upstream_transcript_variant
rs1319336075	intron_variant,genic_upstream_transcript_variant
rs1319341405	intron_variant,genic_upstream_transcript_variant
rs1319363254	intron_variant
rs1319396999	intron_variant
rs1319450660	intron_variant
rs1319462130	intron_variant,genic_upstream_transcript_variant
rs1319473127	intron_variant,genic_upstream_transcript_variant
rs1319496434	intron_variant,genic_upstream_transcript_variant
rs1319545780	intron_variant,genic_upstream_transcript_variant
rs1319555960	synonymous_variant,non_coding_transcript_variant,coding_sequence_variant
rs1319584109	intron_variant
rs1319595534	intron_variant,genic_downstream_transcript_variant
rs1319617654	intron_variant,genic_upstream_transcript_variant
rs1319621529	intron_variant,genic_upstream_transcript_variant
rs1319633623	intron_variant,genic_upstream_transcript_variant
rs1319645354	intron_variant,genic_upstream_transcript_variant
rs1319682630	intron_variant
rs1319711175	intron_variant,genic_downstream_transcript_variant
rs1319799684	intron_variant
rs1319841199	intron_variant,2KB_upstream_variant,upstream_transcript_variant,genic_upstream_transcript_variant
rs1319852252	intron_variant,genic_upstream_transcript_variant
rs1319855597	intron_variant,genic_upstream_transcript_variant
rs1319885933	intron_variant
rs1319913612	intron_variant
rs1319938110	intron_variant,genic_upstream_transcript_variant
rs1319968419	intron_variant

rs1319986428	intron_variant,genic_upstream_transcript_variant
rs1320018937	intron_variant,genic_upstream_transcript_variant
rs1320027830	intron_variant,genic_upstream_transcript_variant
rs1320074432	intron_variant
rs1320099793	intron_variant,genic_upstream_transcript_variant
rs1320163570	intron_variant,genic_upstream_transcript_variant
rs1320167135	intron_variant,genic_upstream_transcript_variant
rs1320178418	intron_variant
rs1320179292	intron_variant,genic_upstream_transcript_variant
rs1320196271	intron_variant
rs1320227357	intron_variant
rs1320255340	intron_variant
rs1320271311	intron_variant,genic_upstream_transcript_variant
rs1320300297	intron_variant
rs1320314120	intron_variant,genic_upstream_transcript_variant
rs1320317425	intron_variant
rs1320331688	intron_variant
rs1320345314	intron_variant,upstream_transcript_variant,genic_upstream_transcript_variant
rs1320372231	intron_variant,genic_upstream_transcript_variant
rs1320390013	intron_variant,genic_upstream_transcript_variant
rs1320412256	intron_variant,genic_upstream_transcript_variant
rs1320458607	intron_variant,genic_upstream_transcript_variant
rs1320468228	intron_variant
rs1320468925	intron_variant
rs1320479541	intron_variant,genic_downstream_transcript_variant
rs1320496007	intron_variant
rs1320527721	intron_variant,genic_upstream_transcript_variant
rs1320536749	intron_variant
rs1320541463	intron_variant,genic_downstream_transcript_variant
rs1320570570	intron_variant,2KB_upstream_variant,upstream_transcript_variant,genic_upstream_transcript_variant
rs1320597331	intron_variant,genic_downstream_transcript_variant
rs1320601559	intron_variant
rs1320682989	intron_variant,genic_upstream_transcript_variant
rs1320690274	intron_variant,genic_upstream_transcript_variant
rs1320692896	intron_variant,genic_downstream_transcript_variant
rs1320714016	intron_variant,upstream_transcript_variant,genic_upstream_transcript_variant
rs1320741403	intron_variant,genic_upstream_transcript_variant
rs1320742117	intron_variant
rs1320744670	intron_variant
rs1320801769	intron_variant
rs1320813311	intron_variant
rs1320814164	intron_variant,genic_upstream_transcript_variant
rs1320818173	intron_variant
rs1320868707	intron_variant,genic_upstream_transcript_variant
rs1320874990	intron_variant,genic_upstream_transcript_variant
rs1320900784	intron_variant,genic_downstream_transcript_variant
rs1320918996	intron_variant
rs1320942111	intron_variant,genic_upstream_transcript_variant
rs1320995226	intron_variant
rs1321043011	intron_variant
rs1321072964	intron_variant,genic_upstream_transcript_variant
rs1321081598	intron_variant,genic_upstream_transcript_variant
rs1321085240	intron_variant,genic_upstream_transcript_variant
rs1321148900	intron_variant,genic_upstream_transcript_variant
rs1321166532	intron_variant
rs1321185662	intron_variant,genic_upstream_transcript_variant
rs1321268752	intron_variant,genic_downstream_transcript_variant
rs1321269120	intron_variant,upstream_transcript_variant,genic_upstream_transcript_variant
rs1321270978	intron_variant,genic_upstream_transcript_variant
rs1321272696	intron_variant,genic_upstream_transcript_variant
rs1321373360	non_coding_transcript_variant,3_prime_UTR_variant,genic_downstream_transcript_variant
rs1321397765	intron_variant
rs1321435511	intron_variant,genic_downstream_transcript_variant
rs1321441526	intron_variant,upstream_transcript_variant,5_prime_UTR_variant,genic_upstream_transcript_variant
rs1321451850	intron_variant,genic_downstream_transcript_variant
rs1321453622	intron_variant,genic_downstream_transcript_variant
rs1321500965	intron_variant,genic_upstream_transcript_variant
rs1321528450	intron_variant,genic_downstream_transcript_variant
rs1321540103	intron_variant,genic_upstream_transcript_variant
rs1321554895	intron_variant,genic_upstream_transcript_variant
rs1321563617	intron_variant
rs1321586526	intron_variant,genic_upstream_transcript_variant
rs1321617672	intron_variant,genic_downstream_transcript_variant
rs1321619225	intron_variant

rs1321792768	intron_variant
rs1321794883	intron_variant,genic_upstream_transcript_variant
rs1321795295	intron_variant,genic_upstream_transcript_variant
rs1321808230	intron_variant
rs1321830924	intron_variant,genic_downstream_transcript_variant
rs1321841155	intron_variant,genic_upstream_transcript_variant
rs1321872678	intron_variant,genic_upstream_transcript_variant
rs1321921325	intron_variant,genic_downstream_transcript_variant
rs1321931532	intron_variant
rs1321933627	intron_variant,genic_upstream_transcript_variant
rs1321959949	intron_variant,5_prime_UTR_variant
rs1321966340	intron_variant,genic_upstream_transcript_variant
rs1322057499	intron_variant,genic_upstream_transcript_variant
rs1322059884	intron_variant,upstream_transcript_variant,genic_upstream_transcript_variant
rs1322067458	missense_variant,non_coding_transcript_variant,coding_sequence_variant
rs1322077881	intron_variant,genic_upstream_transcript_variant
rs1322084786	intron_variant,genic_upstream_transcript_variant
rs1322183460	intron_variant,genic_upstream_transcript_variant
rs1322186460	intron_variant,genic_upstream_transcript_variant
rs1322202293	non_coding_transcript_variant,coding_sequence_variant,synonymous_variant
rs1322223442	intron_variant
rs1322245914	intron_variant
rs1322344567	3_prime_UTR_variant,genic_downstream_transcript_variant,non_coding_transcript_variant
rs1322362224	intron_variant,genic_upstream_transcript_variant
rs1322364607	intron_variant,genic_downstream_transcript_variant
rs1322412267	intron_variant
rs1322422810	intron_variant,genic_downstream_transcript_variant
rs1322431113	intron_variant,genic_upstream_transcript_variant
rs1322527165	2KB_upstream_variant,intron_variant,upstream_transcript_variant,genic_upstream_transcript_variant
rs1322528151	intron_variant,genic_upstream_transcript_variant
rs1322551971	intron_variant
rs1322579389	intron_variant,genic_upstream_transcript_variant
rs1322579863	intron_variant,genic_upstream_transcript_variant
rs1322602172	2KB_upstream_variant,intron_variant,upstream_transcript_variant,genic_upstream_transcript_variant
rs1322657600	intron_variant
rs1322662483	intron_variant,genic_downstream_transcript_variant
rs1322702194	intron_variant
rs1322742025	intron_variant
rs1322743378	intron_variant,genic_upstream_transcript_variant
rs1322770743	intron_variant
rs1322805200	intron_variant,genic_upstream_transcript_variant
rs1322856265	intron_variant,genic_upstream_transcript_variant
rs1322880807	intron_variant,genic_downstream_transcript_variant
rs1322893269	genic_downstream_transcript_variant,non_coding_transcript_variant,coding_sequence_variant,synonymous_variant
rs1322910262	intron_variant
rs1322940581	intron_variant,genic_upstream_transcript_variant
rs1322961078	intron_variant
rs1322968009	intron_variant,genic_upstream_transcript_variant
rs1322973213	intron_variant,genic_downstream_transcript_variant
rs1322992542	intron_variant
rs1323023024	intron_variant
rs1323083590	intron_variant
rs1323087416	intron_variant,genic_upstream_transcript_variant
rs1323109627	intron_variant,genic_downstream_transcript_variant
rs1323158874	intron_variant,genic_downstream_transcript_variant
rs1323170004	intron_variant,genic_upstream_transcript_variant
rs1323185444	3_prime_UTR_variant,genic_downstream_transcript_variant,non_coding_transcript_variant
rs1323185967	intron_variant,genic_upstream_transcript_variant
rs1323192827	intron_variant,genic_upstream_transcript_variant
rs1323211679	intron_variant
rs1323223900	intron_variant
rs1323244351	intron_variant
rs1323247697	intron_variant,genic_upstream_transcript_variant
rs1323251142	intron_variant,genic_downstream_transcript_variant
rs1323259112	intron_variant
rs1323259162	intron_variant,genic_downstream_transcript_variant
rs1323268249	intron_variant
rs1323269142	intron_variant
rs1323286905	intron_variant,genic_downstream_transcript_variant
rs1323314530	downstream_transcript_variant,intron_variant,genic_downstream_transcript_variant
rs1323315146	intron_variant,upstream_transcript_variant,genic_upstream_transcript_variant
rs1323338287	intron_variant,genic_upstream_transcript_variant
rs1323381081	intron_variant,upstream_transcript_variant,genic_upstream_transcript_variant
rs1323410809	intron_variant,genic_downstream_transcript_variant

rs1323439654	intron_variant
rs1323467002	intron_variant
rs1323480004	intron_variant
rs1323651888	intron_variant,genic_upstream_transcript_variant
rs1323682977	intron_variant
rs1323687144	intron_variant,genic_downstream_transcript_variant
rs1323714356	intron_variant,genic_upstream_transcript_variant
rs1323727086	intron_variant,genic_downstream_transcript_variant
rs1323736658	intron_variant
rs1323754044	intron_variant,genic_downstream_transcript_variant
rs1323774260	intron_variant,genic_upstream_transcript_variant
rs1323791991	intron_variant
rs1323814035	intron_variant
rs1323893725	intron_variant
rs1323903784	intron_variant,genic_upstream_transcript_variant
rs1323904766	intron_variant,genic_upstream_transcript_variant
rs1323910168	2KB_upstream_variant,intron_variant,upstream_transcript_variant,genic_upstream_transcript_variant
rs1323910749	3_prime_UTR_variant,genic_downstream_transcript_variant,non_coding_transcript_variant
rs1323939997	intron_variant
rs1324076277	intron_variant
rs1324079234	intron_variant
rs1324089908	intron_variant,genic_upstream_transcript_variant
rs1324090725	intron_variant,genic_upstream_transcript_variant
rs1324119623	intron_variant,genic_upstream_transcript_variant
rs1324148232	intron_variant,genic_downstream_transcript_variant
rs1324205447	intron_variant,genic_upstream_transcript_variant
rs1324228638	intron_variant,genic_upstream_transcript_variant
rs1324233317	intron_variant
rs1324236890	intron_variant,genic_downstream_transcript_variant
rs1324253449	intron_variant,genic_upstream_transcript_variant
rs1324314215	intron_variant,non_coding_transcript_variant,5_prime_UTR_variant,genic_upstream_transcript_variant
rs1324359680	intron_variant
rs1324380384	3_prime_UTR_variant,genic_downstream_transcript_variant,non_coding_transcript_variant
rs1324428942	intron_variant
rs1324480588	intron_variant
rs1324527155	intron_variant,genic_downstream_transcript_variant
rs1324572782	coding_sequence_variant,intron_variant,non_coding_transcript_variant,synonymous_variant,genic_upstream_transcript_variant
rs1324576950	non_coding_transcript_variant,coding_sequence_variant,synonymous_variant
rs1324579666	intron_variant,genic_downstream_transcript_variant
rs1324604905	intron_variant
rs1324628450	intron_variant,genic_upstream_transcript_variant
rs1324643073	intron_variant,genic_upstream_transcript_variant
rs1324690686	intron_variant,genic_downstream_transcript_variant
rs1324705743	intron_variant
rs1324778341	intron_variant,genic_upstream_transcript_variant
rs1324818519	intron_variant
rs1324873050	intron_variant,genic_upstream_transcript_variant
rs1324888690	intron_variant
rs1324913999	intron_variant,genic_upstream_transcript_variant
rs1324921567	intron_variant,genic_upstream_transcript_variant
rs1324939009	intron_variant,genic_downstream_transcript_variant
rs1324987266	intron_variant,genic_downstream_transcript_variant
rs1325036336	2KB_upstream_variant,intron_variant,upstream_transcript_variant,genic_upstream_transcript_variant
rs1325054995	intron_variant,genic_downstream_transcript_variant
rs1325059737	intron_variant
rs1325105583	intron_variant,genic_upstream_transcript_variant
rs1325112056	intron_variant,genic_upstream_transcript_variant
rs1325122929	intron_variant,genic_upstream_transcript_variant
rs1325130386	2KB_upstream_variant,intron_variant,upstream_transcript_variant,genic_upstream_transcript_variant
rs1325131038	intron_variant
rs1325163779	intron_variant,genic_upstream_transcript_variant
rs1325192240	intron_variant
rs1325226416	intron_variant,genic_downstream_transcript_variant
rs1325229995	intron_variant,genic_downstream_transcript_variant
rs1325285418	intron_variant,genic_downstream_transcript_variant
rs1325312662	intron_variant,genic_downstream_transcript_variant
rs1325318175	intron_variant,genic_upstream_transcript_variant
rs1325409189	intron_variant
rs1325429134	intron_variant,genic_upstream_transcript_variant
rs1325429382	intron_variant,genic_downstream_transcript_variant
rs1325441629	intron_variant
rs1325481654	intron_variant
rs1325503042	intron_variant,genic_upstream_transcript_variant
rs1325516471	intron_variant,genic_upstream_transcript_variant

rs1325544646	intron_variant,genic_upstream_transcript_variant
rs1325553076	intron_variant,genic_downstream_transcript_variant
rs1325555482	intron_variant,genic_upstream_transcript_variant
rs1325580866	intron_variant
rs1325581534	intron_variant,genic_downstream_transcript_variant
rs1325608145	intron_variant,genic_upstream_transcript_variant
rs1325624514	intron_variant
rs1325627557	intron_variant,genic_downstream_transcript_variant
rs1325697374	intron_variant
rs1325708558	intron_variant,genic_upstream_transcript_variant
rs1325710754	intron_variant
rs1325751868	intron_variant,genic_downstream_transcript_variant
rs1325756140	intron_variant,genic_downstream_transcript_variant
rs1325762263	intron_variant
rs1325795587	intron_variant,genic_upstream_transcript_variant
rs1325814882	intron_variant,genic_upstream_transcript_variant
rs1325854769	intron_variant,genic_upstream_transcript_variant
rs1325895008	intron_variant,genic_downstream_transcript_variant
rs1325911075	intron_variant,upstream_transcript_variant,genic_upstream_transcript_variant
rs1325929837	intron_variant
rs1325982409	intron_variant
rs1326011916	intron_variant,genic_upstream_transcript_variant
rs1326033869	intron_variant
rs1326047091	intron_variant,genic_upstream_transcript_variant
rs1326051668	intron_variant,genic_downstream_transcript_variant
rs1326096172	intron_variant
rs1326099975	intron_variant,genic_upstream_transcript_variant
rs1326113892	intron_variant,genic_upstream_transcript_variant
rs1326180691	intron_variant,genic_upstream_transcript_variant
rs1326194326	intron_variant,genic_upstream_transcript_variant
rs1326212416	intron_variant
rs1326225557	intron_variant,genic_upstream_transcript_variant
rs1326230165	intron_variant
rs1326242186	intron_variant,genic_downstream_transcript_variant
rs1326261152	intron_variant
rs1326314138	intron_variant
rs1326316698	intron_variant,genic_upstream_transcript_variant
rs1326367979	2KB_upstream_variant,intron_variant,upstream_transcript_variant,genic_upstream_transcript_variant
rs1326378950	2KB_upstream_variant,intron_variant,upstream_transcript_variant,genic_upstream_transcript_variant
rs1326397874	intron_variant,genic_downstream_transcript_variant
rs1326427443	intron_variant,genic_upstream_transcript_variant
rs1326440243	intron_variant
rs1326448636	intron_variant
rs1326482497	intron_variant
rs1326490414	intron_variant,genic_upstream_transcript_variant
rs1326490421	intron_variant
rs1326506815	intron_variant,genic_upstream_transcript_variant
rs1326615827	intron_variant,genic_upstream_transcript_variant
rs1326688135	intron_variant,genic_downstream_transcript_variant
rs1326722551	intron_variant,genic_upstream_transcript_variant
rs1326727211	intron_variant
rs1326757604	intron_variant
rs1326835730	intron_variant
rs1326883459	intron_variant
rs1326905804	intron_variant,genic_upstream_transcript_variant
rs1326906719	intron_variant,genic_upstream_transcript_variant
rs1326918752	intron_variant
rs1326939449	intron_variant,genic_upstream_transcript_variant
rs1326940730	intron_variant,genic_upstream_transcript_variant
rs1326972153	intron_variant,genic_downstream_transcript_variant
rs1326985412	intron_variant
rs1326989480	intron_variant
rs1326990929	intron_variant,genic_upstream_transcript_variant
rs1326998718	intron_variant,genic_upstream_transcript_variant
rs1327033566	intron_variant,genic_upstream_transcript_variant
rs1327107416	intron_variant,genic_downstream_transcript_variant
rs1327124995	intron_variant,genic_upstream_transcript_variant
rs1327137995	intron_variant,genic_upstream_transcript_variant
rs1327211585	intron_variant,genic_downstream_transcript_variant
rs1327212854	intron_variant
rs1327228661	intron_variant,genic_downstream_transcript_variant
rs1327236305	intron_variant
rs1327307574	intron_variant
rs1327367196	intron_variant,genic_upstream_transcript_variant

rs1327404104	intron_variant
rs1327408036	intron_variant
rs1327431626	intron_variant,genic_upstream_transcript_variant
rs1327462031	intron_variant,genic_downstream_transcript_variant
rs1327479611	intron_variant,genic_downstream_transcript_variant
rs1327492198	splice_acceptor_variant
rs1327497989	intron_variant
rs1327533649	intron_variant
rs1327548980	intron_variant
rs1327552622	intron_variant,genic_downstream_transcript_variant
rs1327584450	intron_variant
rs1327644331	intron_variant
rs1327718918	intron_variant
rs1327756937	intron_variant
rs1327785446	intron_variant
rs1327796740	intron_variant,genic_upstream_transcript_variant
rs1327806663	intron_variant,genic_upstream_transcript_variant
rs1327816329	intron_variant
rs1327851511	intron_variant,genic_upstream_transcript_variant
rs1327880753	intron_variant
rs1327940479	intron_variant,genic_downstream_transcript_variant
rs1327946446	coding_sequence_variant,intron_variant,non_coding_transcript_variant,synonymous_variant,genic_upstream_transcript_variant
rs1327962009	intron_variant
rs1327990004	intron_variant
rs1328043569	intron_variant,genic_upstream_transcript_variant
rs1328048562	intron_variant
rs1328062272	intron_variant,genic_upstream_transcript_variant
rs1328076020	intron_variant,upstream_transcript_variant,genic_upstream_transcript_variant
rs1328104080	intron_variant,genic_upstream_transcript_variant
rs1328110342	coding_sequence_variant,intron_variant,non_coding_transcript_variant,missense_variant,genic_upstream_transcript_variant
rs1328133734	intron_variant
rs1328177768	intron_variant
rs1328178605	intron_variant,genic_upstream_transcript_variant
rs1328188238	intron_variant,genic_upstream_transcript_variant
rs1328202839	intron_variant,genic_upstream_transcript_variant
rs1328226625	intron_variant
rs1328266523	intron_variant,genic_upstream_transcript_variant
rs1328270242	2KB_upstream_variant,intron_variant,upstream_transcript_variant,genic_upstream_transcript_variant
rs1328305260	intron_variant,upstream_transcript_variant,genic_upstream_transcript_variant
rs1328365659	intron_variant
rs1328369786	intron_variant,genic_downstream_transcript_variant
rs1328396840	intron_variant
rs1328482883	intron_variant
rs1328487624	intron_variant
rs1328562316	intron_variant,genic_upstream_transcript_variant
rs1328583345	intron_variant
rs1328629675	intron_variant
rs1328670612	intron_variant,genic_upstream_transcript_variant
rs1328687532	intron_variant,genic_upstream_transcript_variant
rs1328693429	intron_variant
rs1328711850	intron_variant
rs1328738544	intron_variant
rs1328807237	2KB_upstream_variant,intron_variant,upstream_transcript_variant,genic_upstream_transcript_variant
rs1328836205	intron_variant
rs1328963556	intron_variant,genic_upstream_transcript_variant
rs1328984807	intron_variant
rs1328992609	intron_variant
rs1329008493	intron_variant,genic_upstream_transcript_variant
rs1329012117	intron_variant
rs1329045527	intron_variant
rs1329065140	intron_variant
rs1329145390	intron_variant
rs1329161301	intron_variant,genic_upstream_transcript_variant
rs1329169055	intron_variant
rs1329203662	intron_variant,genic_upstream_transcript_variant
rs1329246037	intron_variant
rs1329249195	intron_variant,genic_upstream_transcript_variant
rs1329269227	coding_sequence_variant,intron_variant,non_coding_transcript_variant,missense_variant,genic_upstream_transcript_variant
rs1329270699	intron_variant,genic_upstream_transcript_variant
rs1329272976	intron_variant,genic_downstream_transcript_variant
rs1329310210	intron_variant,genic_downstream_transcript_variant
rs1329331730	intron_variant,genic_upstream_transcript_variant
rs1329337431	intron_variant,genic_downstream_transcript_variant
rs1329342578	intron_variant

rs1329428078	intron_variant,genic_downstream_transcript_variant
rs1329454199	intron_variant,genic_downstream_transcript_variant
rs1329455141	intron_variant
rs1329459348	intron_variant,genic_upstream_transcript_variant
rs1329464737	intron_variant,genic_upstream_transcript_variant
rs1329468294	intron_variant,genic_downstream_transcript_variant
rs1329584745	intron_variant,upstream_transcript_variant,genic_upstream_transcript_variant
rs1329623383	intron_variant,genic_downstream_transcript_variant
rs1329705563	intron_variant,genic_upstream_transcript_variant
rs1329709083	intron_variant,genic_upstream_transcript_variant
rs1329735263	intron_variant
rs1329741031	intron_variant,genic_downstream_transcript_variant
rs1329754023	intron_variant
rs1329754951	missense_variant,non_coding_transcript_variant,coding_sequence_variant
rs1329809230	downstream_transcript_variant,intron_variant,genic_downstream_transcript_variant
rs1329812885	intron_variant,genic_upstream_transcript_variant
rs1329825598	intron_variant
rs1329833092	intron_variant,genic_downstream_transcript_variant
rs1329839811	intron_variant
rs1329862044	intron_variant,genic_downstream_transcript_variant
rs1329886933	missense_variant,non_coding_transcript_variant,coding_sequence_variant,synonymous_variant
rs1329907432	intron_variant,genic_downstream_transcript_variant
rs1329909009	intron_variant,genic_upstream_transcript_variant
rs1329947981	2KB_upstream_variant,intron_variant,upstream_transcript_variant,genic_upstream_transcript_variant
rs1329990605	intron_variant
rs1330062336	intron_variant,genic_downstream_transcript_variant
rs1330072532	intron_variant,genic_upstream_transcript_variant
rs1330125175	intron_variant
rs1330140020	intron_variant
rs1330187995	intron_variant,genic_upstream_transcript_variant
rs1330198104	intron_variant,genic_upstream_transcript_variant
rs1330230732	intron_variant,genic_upstream_transcript_variant
rs1330269617	intron_variant
rs1330331613	intron_variant
rs1330356892	intron_variant
rs1330361986	intron_variant
rs1330382403	intron_variant,genic_upstream_transcript_variant
rs1330388336	intron_variant,genic_upstream_transcript_variant
rs1330412416	intron_variant,genic_upstream_transcript_variant
rs1330421176	intron_variant,genic_upstream_transcript_variant
rs1330456407	intron_variant
rs1330530750	intron_variant
rs1330537117	non_coding_transcript_variant,coding_sequence_variant,synonymous_variant
rs1330538771	intron_variant,genic_downstream_transcript_variant
rs1330565170	intron_variant
rs1330566980	missense_variant,genic_downstream_transcript_variant,non_coding_transcript_variant,coding_sequence_variant
rs1330586121	intron_variant,genic_upstream_transcript_variant
rs1330604825	intron_variant,genic_downstream_transcript_variant
rs1330604948	intron_variant
rs1330605624	intron_variant,genic_upstream_transcript_variant
rs1330646563	intron_variant,genic_upstream_transcript_variant
rs1330648228	missense_variant,non_coding_transcript_variant,coding_sequence_variant
rs1330675420	intron_variant,genic_upstream_transcript_variant
rs1330737886	intron_variant
rs1330738577	intron_variant,genic_upstream_transcript_variant
rs1330820652	intron_variant
rs1330841060	intron_variant,genic_upstream_transcript_variant
rs1330852166	2KB_upstream_variant,intron_variant,upstream_transcript_variant,genic_upstream_transcript_variant
rs1330870101	intron_variant,genic_upstream_transcript_variant
rs1330883296	intron_variant,genic_upstream_transcript_variant
rs1330893335	intron_variant,upstream_transcript_variant,genic_upstream_transcript_variant
rs1330905122	intron_variant
rs1330906260	intron_variant,genic_downstream_transcript_variant
rs1330916042	intron_variant
rs1330922830	intron_variant,genic_downstream_transcript_variant
rs1330983822	intron_variant,upstream_transcript_variant,genic_upstream_transcript_variant
rs1331010047	intron_variant
rs1331054382	intron_variant
rs1331075578	downstream_transcript_variant,500B_downstream_variant
rs1331149506	intron_variant,genic_downstream_transcript_variant
rs1331150318	intron_variant,genic_upstream_transcript_variant
rs1331159520	intron_variant,genic_downstream_transcript_variant
rs1331184024	intron_variant,genic_upstream_transcript_variant
rs1331223041	intron_variant,genic_upstream_transcript_variant

rs1331307137	intron_variant,genic_upstream_transcript_variant
rs1331345039	intron_variant,genic_upstream_transcript_variant
rs1331357889	2KB_upstream_variant,intron_variant,upstream_transcript_variant,genic_upstream_transcript_variant
rs1331381396	intron_variant
rs1331381634	intron_variant
rs1331396936	intron_variant,genic_upstream_transcript_variant
rs1331410213	intron_variant,genic_upstream_transcript_variant
rs1331441848	3_prime_UTR_variant,genic_downstream_transcript_variant,non_coding_transcript_variant
rs1331442207	intron_variant
rs1331449833	intron_variant,genic_upstream_transcript_variant
rs1331473056	intron_variant,genic_downstream_transcript_variant
rs1331515187	intron_variant,genic_downstream_transcript_variant
rs1331516834	intron_variant,genic_upstream_transcript_variant
rs1331521895	intron_variant
rs1331534280	intron_variant,genic_upstream_transcript_variant
rs1331541903	intron_variant,genic_upstream_transcript_variant
rs1331610298	intron_variant
rs1331631723	intron_variant,genic_upstream_transcript_variant
rs1331639031	intron_variant,upstream_transcript_variant,genic_upstream_transcript_variant
rs1331657499	intron_variant
rs1331713647	intron_variant,genic_upstream_transcript_variant
rs1331718048	intron_variant
rs1331724479	intron_variant,genic_upstream_transcript_variant
rs1331747904	intron_variant,genic_upstream_transcript_variant
rs1331762345	intron_variant,genic_upstream_transcript_variant
rs1331767631	intron_variant,genic_downstream_transcript_variant
rs1331775128	intron_variant,upstream_transcript_variant,genic_upstream_transcript_variant
rs1331788631	intron_variant
rs1331819889	intron_variant,genic_upstream_transcript_variant
rs1331903155	intron_variant,genic_downstream_transcript_variant
rs1331924184	intron_variant,genic_upstream_transcript_variant
rs1331945062	intron_variant,genic_downstream_transcript_variant
rs1332004880	intron_variant,genic_upstream_transcript_variant
rs1332005755	intron_variant,genic_upstream_transcript_variant
rs1332049203	genic_downstream_transcript_variant,non_coding_transcript_variant,coding_sequence_variant,synonymous_variant
rs1332067642	intron_variant
rs1332129021	intron_variant,genic_upstream_transcript_variant
rs1332182169	intron_variant,genic_upstream_transcript_variant
rs1332205384	intron_variant,genic_upstream_transcript_variant
rs1332209545	intron_variant,genic_upstream_transcript_variant
rs1332226306	intron_variant,genic_downstream_transcript_variant
rs1332254769	intron_variant,genic_downstream_transcript_variant
rs1332264691	2KB_upstream_variant,intron_variant,upstream_transcript_variant,genic_upstream_transcript_variant
rs1332295534	intron_variant,genic_upstream_transcript_variant
rs1332322470	intron_variant,genic_upstream_transcript_variant
rs1332335317	intron_variant,genic_upstream_transcript_variant
rs1332371053	intron_variant
rs1332440164	intron_variant,genic_upstream_transcript_variant
rs1332461919	intron_variant,genic_upstream_transcript_variant
rs1332466514	intron_variant
rs1332469617	intron_variant,5_prime_UTR_variant
rs1332512223	intron_variant,genic_downstream_transcript_variant
rs1332528920	intron_variant
rs1332538895	intron_variant,genic_upstream_transcript_variant
rs1332542620	intron_variant,genic_downstream_transcript_variant
rs1332549572	intron_variant
rs1332559833	intron_variant,genic_downstream_transcript_variant
rs1332608617	intron_variant,genic_upstream_transcript_variant
rs1332611671	intron_variant
rs1332625147	intron_variant,genic_upstream_transcript_variant
rs1332657678	intron_variant,genic_upstream_transcript_variant
rs1332661739	intron_variant,genic_downstream_transcript_variant
rs1332681477	intron_variant
rs1332714152	intron_variant,genic_upstream_transcript_variant
rs1332751242	intron_variant,genic_upstream_transcript_variant
rs1332855371	intron_variant,genic_upstream_transcript_variant
rs1332864923	intron_variant,genic_downstream_transcript_variant
rs1332866600	intron_variant
rs1332904083	intron_variant,genic_downstream_transcript_variant
rs1332913528	intron_variant,genic_upstream_transcript_variant
rs1332923354	intron_variant,genic_downstream_transcript_variant
rs1332938160	intron_variant
rs1332966461	intron_variant,genic_downstream_transcript_variant
rs1333052320	intron_variant

rs1333074057	intron_variant,genic_downstream_transcript_variant
rs1333075112	intron_variant,genic_upstream_transcript_variant
rs1333125207	2KB_upstream_variant,intron_variant,upstream_transcript_variant,genic_upstream_transcript_variant
rs1333130780	intron_variant,genic_downstream_transcript_variant
rs1333133391	intron_variant
rs1333171640	intron_variant
rs1333179468	2KB_upstream_variant,intron_variant,upstream_transcript_variant,genic_upstream_transcript_variant
rs1333186598	intron_variant,genic_upstream_transcript_variant
rs1333192203	intron_variant,genic_upstream_transcript_variant
rs1333196976	downstream_transcript_variant,500B_downstream_variant
rs1333198689	intron_variant,upstream_transcript_variant,genic_upstream_transcript_variant
rs1333220649	intron_variant,genic_downstream_transcript_variant
rs1333303237	intron_variant
rs1333347470	intron_variant
rs1333372034	2KB_upstream_variant,intron_variant,upstream_transcript_variant,genic_upstream_transcript_variant
rs1333446970	intron_variant,genic_upstream_transcript_variant
rs1333491821	intron_variant,genic_downstream_transcript_variant
rs1333501976	intron_variant,genic_upstream_transcript_variant
rs1333556847	intron_variant
rs1333565307	intron_variant,upstream_transcript_variant,genic_upstream_transcript_variant
rs1333579078	intron_variant,genic_upstream_transcript_variant
rs1333592944	intron_variant
rs1333602835	intron_variant
rs1333636235	intron_variant,genic_upstream_transcript_variant
rs1333674220	intron_variant,genic_upstream_transcript_variant
rs1333700242	intron_variant
rs1333731030	intron_variant,genic_upstream_transcript_variant
rs1333742949	intron_variant,genic_downstream_transcript_variant
rs1333788480	intron_variant
rs1333788633	intron_variant,genic_downstream_transcript_variant
rs1333882720	intron_variant,genic_upstream_transcript_variant
rs1333887574	intron_variant
rs1333908313	genic_downstream_transcript_variant,intron_variant
rs1333926072	intron_variant
rs1333967976	genic_downstream_transcript_variant,intron_variant
rs1334005270	intron_variant,genic_upstream_transcript_variant
rs1334031738	intron_variant,genic_upstream_transcript_variant
rs1334042408	intron_variant
rs1334096043	intron_variant,genic_upstream_transcript_variant
rs1334105614	intron_variant
rs1334111816	intron_variant
rs1334173817	intron_variant
rs1334178056	intron_variant,genic_upstream_transcript_variant
rs1334216052	intron_variant,genic_upstream_transcript_variant
rs1334218300	intron_variant
rs1334221824	intron_variant
rs1334283549	genic_downstream_transcript_variant,intron_variant
rs1334302251	intron_variant,genic_upstream_transcript_variant
rs1334318956	intron_variant,genic_upstream_transcript_variant
rs1334373241	intron_variant,genic_upstream_transcript_variant
rs1334391118	intron_variant
rs1334394662	intron_variant,genic_upstream_transcript_variant
rs1334398721	genic_downstream_transcript_variant,intron_variant
rs1334473920	genic_downstream_transcript_variant,intron_variant
rs1334504424	non_coding_transcript_variant,3_prime_UTR_variant,genic_downstream_transcript_variant
rs1334512628	intron_variant,genic_upstream_transcript_variant
rs1334515278	intron_variant,upstream_transcript_variant,genic_upstream_transcript_variant
rs1334578917	non_coding_transcript_variant,3_prime_UTR_variant,genic_downstream_transcript_variant
rs1334586292	intron_variant
rs1334589198	intron_variant
rs1334605114	intron_variant,genic_upstream_transcript_variant
rs1334637956	intron_variant
rs1334654331	intron_variant
rs1334660522	intron_variant,genic_upstream_transcript_variant
rs1334665947	genic_downstream_transcript_variant,intron_variant
rs1334668737	genic_upstream_transcript_variant,intron_variant,upstream_transcript_variant,2KB_upstream_variant
rs1334685859	intron_variant,genic_upstream_transcript_variant
rs1334687964	intron_variant
rs1334766793	intron_variant
rs1334774166	intron_variant,genic_upstream_transcript_variant
rs1334802639	intron_variant,genic_upstream_transcript_variant
rs1334820552	genic_downstream_transcript_variant,intron_variant
rs1334824833	intron_variant,genic_upstream_transcript_variant
rs1334843137	intron_variant

rs1334911583	intron_variant,genic_upstream_transcript_variant
rs1335055945	intron_variant
rs1335059449	genic_downstream_transcript_variant,intron_variant
rs1335128622	genic_downstream_transcript_variant,intron_variant
rs1335148121	genic_downstream_transcript_variant,intron_variant
rs1335160840	intron_variant,genic_upstream_transcript_variant
rs1335265318	intron_variant
rs1335265522	intron_variant
rs1335285177	intron_variant,genic_upstream_transcript_variant
rs1335293676	genic_downstream_transcript_variant,intron_variant
rs1335325125	intron_variant,upstream_transcript_variant,genic_upstream_transcript_variant
rs1335326423	intron_variant,genic_upstream_transcript_variant
rs1335406924	intron_variant,genic_upstream_transcript_variant
rs1335435278	non_coding_transcript_variant,3_prime_UTR_variant,genic_downstream_transcript_variant
rs1335458513	genic_downstream_transcript_variant,intron_variant
rs1335465711	intron_variant,genic_upstream_transcript_variant
rs1335479498	genic_downstream_transcript_variant,intron_variant
rs1335489909	genic_downstream_transcript_variant,intron_variant
rs1335535004	intron_variant
rs1335583090	intron_variant,genic_upstream_transcript_variant
rs1335587346	intron_variant
rs1335600340	genic_downstream_transcript_variant,intron_variant
rs1335627507	genic_downstream_transcript_variant,intron_variant
rs1335687644	intron_variant,upstream_transcript_variant,genic_upstream_transcript_variant
rs1335715095	genic_downstream_transcript_variant,intron_variant
rs1335762575	genic_downstream_transcript_variant,intron_variant
rs1335781404	intron_variant,genic_upstream_transcript_variant
rs1335785982	intron_variant
rs1335813441	intron_variant
rs1335891240	genic_downstream_transcript_variant,intron_variant
rs1335895455	intron_variant,genic_upstream_transcript_variant
rs1335909469	intron_variant,genic_upstream_transcript_variant
rs1335962325	intron_variant
rs1336000420	intron_variant
rs1336021644	intron_variant,genic_upstream_transcript_variant
rs1336050894	intron_variant
rs1336081345	genic_downstream_transcript_variant,intron_variant
rs1336103802	intron_variant,genic_upstream_transcript_variant
rs1336145038	intron_variant
rs1336162289	intron_variant,genic_upstream_transcript_variant
rs1336169119	intron_variant
rs1336185650	intron_variant,genic_upstream_transcript_variant
rs1336189515	intron_variant,genic_upstream_transcript_variant
rs1336252627	intron_variant
rs1336254364	intron_variant
rs1336309126	intron_variant
rs1336329666	genic_downstream_transcript_variant,intron_variant
rs1336369496	intron_variant,genic_upstream_transcript_variant
rs1336422435	genic_downstream_transcript_variant,intron_variant
rs1336436142	intron_variant,genic_upstream_transcript_variant
rs1336444482	non_coding_transcript_variant,3_prime_UTR_variant,genic_downstream_transcript_variant
rs1336448873	intron_variant
rs1336567107	intron_variant
rs1336627749	intron_variant
rs1336652491	intron_variant,genic_upstream_transcript_variant
rs1336695801	intron_variant
rs1336719624	intron_variant,genic_upstream_transcript_variant
rs1336723120	intron_variant
rs1336733236	genic_downstream_transcript_variant,intron_variant
rs1336739355	intron_variant,upstream_transcript_variant,genic_upstream_transcript_variant
rs1336744786	intron_variant
rs1336752197	genic_downstream_transcript_variant,intron_variant
rs1336774980	intron_variant
rs1336788497	genic_downstream_transcript_variant,intron_variant
rs1336792208	intron_variant
rs1336844061	genic_downstream_transcript_variant,intron_variant
rs1336904826	intron_variant
rs1336906568	genic_downstream_transcript_variant,intron_variant
rs1336956622	intron_variant,genic_upstream_transcript_variant
rs1337012787	intron_variant
rs1337042061	intron_variant,genic_upstream_transcript_variant
rs1337089814	intron_variant,genic_upstream_transcript_variant
rs1337114602	intron_variant,genic_upstream_transcript_variant
rs1337148236	intron_variant

rs1337161917	intron_variant
rs1337171565	intron_variant,genic_upstream_transcript_variant
rs1337191257	intron_variant,genic_upstream_transcript_variant
rs1337200258	2KB_upstream_variant,intron_variant,upstream_transcript_variant,genic_upstream_transcript_variant
rs1337216785	2KB_upstream_variant,intron_variant,upstream_transcript_variant,genic_upstream_transcript_variant
rs1337246792	intron_variant
rs1337299680	intron_variant,genic_upstream_transcript_variant
rs1337329589	intron_variant,genic_upstream_transcript_variant
rs1337357109	intron_variant,genic_upstream_transcript_variant
rs1337375831	genic_downstream_transcript_variant,intron_variant
rs1337400253	intron_variant,genic_upstream_transcript_variant
rs1337403980	genic_downstream_transcript_variant,intron_variant
rs1337458094	intron_variant
rs1337559243	intron_variant,genic_upstream_transcript_variant
rs1337643347	genic_downstream_transcript_variant,intron_variant
rs1337664635	intron_variant,genic_upstream_transcript_variant
rs1337673781	intron_variant
rs1337679365	intron_variant
rs1337704130	genic_downstream_transcript_variant,intron_variant
rs1337704667	genic_downstream_transcript_variant,intron_variant
rs1337720615	intron_variant,genic_upstream_transcript_variant
rs1337735122	intron_variant,genic_upstream_transcript_variant
rs1337772896	intron_variant,genic_upstream_transcript_variant
rs1337780484	genic_upstream_transcript_variant,intron_variant,upstream_transcript_variant,2KB_upstream_variant
rs1337791530	intron_variant,genic_upstream_transcript_variant
rs1337846579	intron_variant,genic_upstream_transcript_variant
rs1337901970	genic_upstream_transcript_variant,intron_variant,upstream_transcript_variant,2KB_upstream_variant
rs1337933606	genic_downstream_transcript_variant,intron_variant
rs1337943565	intron_variant
rs1337947187	intron_variant,genic_upstream_transcript_variant
rs1337979787	intron_variant
rs1337981924	intron_variant,genic_upstream_transcript_variant
rs1337999042	intron_variant
rs1338007523	intron_variant
rs1338026569	upstream_transcript_variant,intron_variant,5_prime_UTR_variant,genic_upstream_transcript_variant
rs1338036886	intron_variant,genic_upstream_transcript_variant
rs1338062630	intron_variant,genic_upstream_transcript_variant
rs1338081416	intron_variant,genic_upstream_transcript_variant
rs1338086616	intron_variant,genic_upstream_transcript_variant
rs1338104840	intron_variant
rs1338123803	genic_downstream_transcript_variant,intron_variant
rs1338136655	intron_variant
rs1338171336	intron_variant
rs1338193883	non_coding_transcript_variant,3_prime_UTR_variant,genic_downstream_transcript_variant
rs1338210486	genic_downstream_transcript_variant,intron_variant
rs1338236595	intron_variant,genic_upstream_transcript_variant
rs1338247576	genic_downstream_transcript_variant,intron_variant
rs1338278307	intron_variant,genic_upstream_transcript_variant
rs1338321338	intron_variant,upstream_transcript_variant,genic_upstream_transcript_variant
rs1338333346	intron_variant
rs1338341659	intron_variant,genic_upstream_transcript_variant
rs1338346162	intron_variant
rs1338353503	intron_variant
rs1338363631	intron_variant
rs1338364898	intron_variant
rs1338398772	intron_variant,genic_upstream_transcript_variant
rs1338438743	intron_variant
rs1338449474	intron_variant
rs1338454862	genic_downstream_transcript_variant,intron_variant
rs1338455902	downstream_transcript_variant,genic_downstream_transcript_variant,intron_variant
rs1338476724	intron_variant
rs1338487585	genic_downstream_transcript_variant,intron_variant
rs1338494451	genic_downstream_transcript_variant,intron_variant
rs1338526059	intron_variant
rs1338551931	intron_variant
rs1338601605	2KB_upstream_variant,intron_variant,upstream_transcript_variant,genic_upstream_transcript_variant
rs1338625159	2KB_upstream_variant,intron_variant,upstream_transcript_variant,genic_upstream_transcript_variant
rs1338635676	intron_variant
rs1338670172	genic_downstream_transcript_variant,intron_variant
rs1338717969	intron_variant,genic_upstream_transcript_variant
rs1338718160	intron_variant
rs1338737794	genic_downstream_transcript_variant,intron_variant
rs1338758996	intron_variant,genic_upstream_transcript_variant
rs1338775108	intron_variant

rs1338780448	genic_downstream_transcript_variant,intron_variant
rs1338801347	genic_downstream_transcript_variant,intron_variant
rs1338807065	intron_variant
rs1338860471	intron_variant,genic_upstream_transcript_variant
rs1338864751	intron_variant
rs1338912107	intron_variant
rs1338941729	intron_variant,genic_upstream_transcript_variant
rs1338977990	intron_variant,genic_upstream_transcript_variant
rs1338990448	intron_variant,genic_upstream_transcript_variant
rs1338995167	intron_variant
rs1338997667	intron_variant
rs1339042090	intron_variant,upstream_transcript_variant,genic_upstream_transcript_variant
rs1339050243	intron_variant
rs1339077062	genic_downstream_transcript_variant,intron_variant
rs1339119523	intron_variant
rs1339122014	intron_variant
rs1339160368	intron_variant,upstream_transcript_variant,genic_upstream_transcript_variant
rs1339176911	genic_downstream_transcript_variant,intron_variant
rs1339198054	intron_variant
rs1339256078	intron_variant
rs1339260212	intron_variant
rs1339282614	intron_variant
rs1339295749	intron_variant,genic_upstream_transcript_variant
rs1339307877	intron_variant
rs1339324747	intron_variant
rs1339335868	intron_variant,genic_upstream_transcript_variant
rs1339337936	intron_variant
rs1339376504	intron_variant
rs1339398823	intron_variant,genic_upstream_transcript_variant
rs1339431244	intron_variant,genic_upstream_transcript_variant
rs1339437070	intron_variant
rs1339451128	intron_variant,genic_upstream_transcript_variant
rs1339454028	intron_variant,genic_upstream_transcript_variant
rs1339494768	intron_variant
rs1339518012	genic_downstream_transcript_variant,intron_variant
rs1339523180	genic_downstream_transcript_variant,intron_variant
rs1339526225	intron_variant,genic_upstream_transcript_variant
rs1339532638	intron_variant,genic_upstream_transcript_variant
rs1339542490	intron_variant
rs1339576742	genic_downstream_transcript_variant,intron_variant
rs1339577807	intron_variant
rs1339639385	intron_variant,genic_upstream_transcript_variant
rs1339639703	genic_downstream_transcript_variant,intron_variant
rs1339645147	genic_downstream_transcript_variant,intron_variant
rs1339658904	intron_variant,genic_upstream_transcript_variant
rs1339678565	intron_variant
rs1339694523	intron_variant,genic_upstream_transcript_variant
rs1339714248	genic_downstream_transcript_variant,intron_variant
rs1339743887	intron_variant
rs1339765906	intron_variant,upstream_transcript_variant,genic_upstream_transcript_variant
rs1339784162	intron_variant
rs1339806853	intron_variant,genic_upstream_transcript_variant
rs1339819232	intron_variant
rs1339840999	intron_variant,genic_upstream_transcript_variant
rs1339880410	genic_upstream_transcript_variant,intron_variant,upstream_transcript_variant,2KB_upstream_variant
rs1339883122	intron_variant,genic_upstream_transcript_variant
rs1339884859	genic_downstream_transcript_variant,intron_variant
rs1339895615	intron_variant,genic_upstream_transcript_variant
rs1339963831	intron_variant,genic_upstream_transcript_variant
rs1339987740	intron_variant,genic_upstream_transcript_variant
rs1339998364	intron_variant,genic_upstream_transcript_variant
rs1340011745	intron_variant,genic_upstream_transcript_variant
rs1340015462	intron_variant,genic_upstream_transcript_variant
rs1340017497	intron_variant
rs1340023680	intron_variant,genic_upstream_transcript_variant
rs1340033870	2KB_upstream_variant,intron_variant,upstream_transcript_variant,genic_upstream_transcript_variant
rs1340103393	genic_downstream_transcript_variant,intron_variant
rs1340133585	genic_downstream_transcript_variant,intron_variant
rs1340143336	intron_variant,genic_upstream_transcript_variant
rs1340253277	intron_variant
rs1340272586	intron_variant,genic_upstream_transcript_variant
rs1340309726	intron_variant
rs1340353892	intron_variant
rs1340355638	genic_downstream_transcript_variant,intron_variant

rs1340360044	intron_variant,genic_upstream_transcript_variant
rs1340391174	intron_variant,genic_upstream_transcript_variant
rs1340395461	500B_downstream_variant,downstream_transcript_variant
rs1340400978	genic_downstream_transcript_variant,intron_variant
rs1340416297	intron_variant
rs1340431060	intron_variant,genic_upstream_transcript_variant
rs1340437049	intron_variant,genic_upstream_transcript_variant
rs1340483700	genic_downstream_transcript_variant,intron_variant
rs1340598485	intron_variant
rs1340606631	intron_variant,upstream_transcript_variant,genic_upstream_transcript_variant
rs1340633472	intron_variant
rs1340639067	intron_variant
rs1340643010	intron_variant,genic_upstream_transcript_variant
rs1340647558	intron_variant,genic_upstream_transcript_variant
rs1340678133	intron_variant
rs1340719967	intron_variant
rs1340727607	intron_variant
rs1340829345	intron_variant,genic_upstream_transcript_variant
rs1340843346	intron_variant,genic_upstream_transcript_variant
rs1340861449	2KB_upstream_variant,intron_variant,upstream_transcript_variant,genic_upstream_transcript_variant
rs1340885531	intron_variant,genic_upstream_transcript_variant
rs1340912420	intron_variant
rs1340956559	genic_downstream_transcript_variant,intron_variant
rs1340998104	intron_variant
rs1341010618	genic_upstream_transcript_variant,intron_variant,upstream_transcript_variant,2KB_upstream_variant
rs1341057509	non_coding_transcript_variant,3_prime_UTR_variant,genic_downstream_transcript_variant
rs1341057555	intron_variant,genic_upstream_transcript_variant
rs1341075076	genic_upstream_transcript_variant,intron_variant,upstream_transcript_variant,2KB_upstream_variant
rs1341097476	intron_variant
rs1341109555	intron_variant
rs1341125882	intron_variant
rs1341131498	intron_variant
rs1341144290	intron_variant,genic_upstream_transcript_variant
rs1341180148	intron_variant
rs1341186535	intron_variant,genic_upstream_transcript_variant
rs1341234379	intron_variant
rs1341241469	intron_variant
rs1341294888	genic_downstream_transcript_variant,intron_variant
rs1341295607	intron_variant
rs1341305791	intron_variant,genic_upstream_transcript_variant
rs1341306746	intron_variant,genic_upstream_transcript_variant
rs1341327488	intron_variant
rs1341334839	intron_variant
rs1341353915	intron_variant,upstream_transcript_variant,genic_upstream_transcript_variant
rs1341391355	genic_downstream_transcript_variant,intron_variant
rs1341405465	intron_variant
rs1341414146	genic_downstream_transcript_variant,intron_variant
rs1341442063	intron_variant
rs1341453381	intron_variant,genic_upstream_transcript_variant
rs1341464366	intron_variant,genic_upstream_transcript_variant
rs1341501832	intron_variant
rs1341528088	intron_variant,upstream_transcript_variant,genic_upstream_transcript_variant
rs1341536807	2KB_upstream_variant,intron_variant,upstream_transcript_variant,genic_upstream_transcript_variant
rs1341606363	intron_variant
rs1341623403	intron_variant,genic_upstream_transcript_variant
rs1341699599	intron_variant,5_prime_UTR_variant
rs1341722094	genic_downstream_transcript_variant,intron_variant
rs1341728679	non_coding_transcript_variant,intron_variant,coding_sequence_variant,synonymous_variant,genic_upstream_transcript_variant
rs1341735923	genic_downstream_transcript_variant,intron_variant
rs1341746030	intron_variant
rs1341752714	intron_variant
rs1341770513	intron_variant,genic_upstream_transcript_variant
rs1341850825	intron_variant
rs1341883889	intron_variant
rs1341923625	genic_downstream_transcript_variant,intron_variant
rs1341952484	intron_variant,genic_upstream_transcript_variant
rs1341991399	intron_variant,genic_upstream_transcript_variant
rs1342047696	genic_downstream_transcript_variant,intron_variant
rs1342062456	intron_variant
rs1342124111	intron_variant
rs1342142633	intron_variant,genic_upstream_transcript_variant
rs1342203050	intron_variant
rs1342223243	non_coding_transcript_variant,3_prime_UTR_variant,genic_downstream_transcript_variant
rs1342265486	intron_variant,genic_upstream_transcript_variant

rs1342292659	intron_variant,upstream_transcript_variant,genic_upstream_transcript_variant
rs1342354302	intron_variant,upstream_transcript_variant,genic_upstream_transcript_variant
rs1342386401	intron_variant
rs1342428691	intron_variant
rs1342456081	intron_variant
rs1342458876	intron_variant,upstream_transcript_variant,genic_upstream_transcript_variant
rs1342529891	intron_variant
rs1342535646	genic_downstream_transcript_variant,intron_variant
rs1342574948	non_coding_transcript_variant,intron_variant,coding_sequence_variant,missense_variant,genic_upstream_transcript_variant
rs1342594643	intron_variant
rs1342598916	intron_variant
rs1342602156	intron_variant,genic_upstream_transcript_variant
rs1342637597	genic_downstream_transcript_variant,intron_variant
rs1342637662	intron_variant
rs1342654767	intron_variant
rs1342706236	intron_variant,genic_upstream_transcript_variant
rs1342725448	non_coding_transcript_variant,3_prime_UTR_variant,genic_downstream_transcript_variant
rs1342734617	non_coding_transcript_variant,splice_donor_variant,intron_variant,genic_upstream_transcript_variant
rs1342743446	genic_downstream_transcript_variant,intron_variant
rs1342756381	intron_variant,5_prime_UTR_variant
rs1342806465	intron_variant,genic_upstream_transcript_variant
rs1342854839	intron_variant,genic_upstream_transcript_variant
rs1342894980	genic_downstream_transcript_variant,intron_variant
rs1342917690	intron_variant,genic_upstream_transcript_variant
rs1342934942	intron_variant,genic_upstream_transcript_variant
rs1342987227	intron_variant
rs1342990510	intron_variant,genic_upstream_transcript_variant
rs1343015346	downstream_transcript_variant,genic_downstream_transcript_variant,intron_variant
rs1343017179	intron_variant,genic_upstream_transcript_variant
rs1343020085	intron_variant
rs1343023828	intron_variant
rs1343027955	downstream_transcript_variant,genic_downstream_transcript_variant,intron_variant
rs1343065146	genic_downstream_transcript_variant,intron_variant
rs1343092185	intron_variant
rs1343106958	intron_variant
rs1343108868	intron_variant
rs1343122346	intron_variant
rs1343134723	intron_variant,genic_upstream_transcript_variant
rs1343137707	intron_variant,genic_upstream_transcript_variant
rs1343175330	intron_variant
rs1343180032	intron_variant
rs1343194599	intron_variant,genic_upstream_transcript_variant
rs1343257232	intron_variant
rs1343303134	intron_variant
rs1343320758	genic_downstream_transcript_variant,intron_variant
rs1343323331	intron_variant
rs1343335601	intron_variant
rs1343346143	genic_downstream_transcript_variant,intron_variant
rs1343399129	genic_downstream_transcript_variant,intron_variant
rs1343401962	genic_downstream_transcript_variant,intron_variant
rs1343409141	genic_downstream_transcript_variant,intron_variant
rs1343421107	intron_variant,genic_upstream_transcript_variant
rs1343440072	intron_variant,genic_upstream_transcript_variant
rs1343460085	intron_variant
rs1343474952	intron_variant
rs1343493697	intron_variant
rs1343500564	intron_variant
rs1343509937	intron_variant,genic_upstream_transcript_variant
rs1343525846	intron_variant,genic_upstream_transcript_variant
rs1343529498	non_coding_transcript_variant,genic_downstream_transcript_variant,coding_sequence_variant,missense_variant
rs1343567888	genic_downstream_transcript_variant,intron_variant
rs1343588073	intron_variant
rs1343656664	intron_variant,genic_upstream_transcript_variant
rs1343674412	non_coding_transcript_variant,intron_variant,coding_sequence_variant,missense_variant,genic_upstream_transcript_variant
rs1343676664	genic_downstream_transcript_variant,intron_variant
rs1343762424	intron_variant,genic_upstream_transcript_variant
rs1343779719	intron_variant
rs1343787790	intron_variant
rs1343804330	intron_variant
rs1343823149	intron_variant
rs1343864308	genic_downstream_transcript_variant,intron_variant
rs1343918198	intron_variant,genic_upstream_transcript_variant
rs1343957950	genic_downstream_transcript_variant,intron_variant
rs1343980167	intron_variant

rs1344007292	intron_variant,genic_upstream_transcript_variant
rs1344108273	intron_variant,genic_upstream_transcript_variant
rs1344147526	intron_variant,genic_upstream_transcript_variant
rs1344153277	genic_downstream_transcript_variant,intron_variant
rs1344248489	intron_variant,genic_upstream_transcript_variant
rs1344279266	intron_variant
rs1344280412	intron_variant
rs1344298211	intron_variant,genic_upstream_transcript_variant
rs1344323380	intron_variant,genic_upstream_transcript_variant
rs1344391274	intron_variant,genic_upstream_transcript_variant
rs1344417174	intron_variant
rs1344426944	non_coding_transcript_variant,3_prime_UTR_variant,genic_downstream_transcript_variant
rs1344439719	intron_variant,upstream_transcript_variant,genic_upstream_transcript_variant
rs1344485271	intron_variant,genic_upstream_transcript_variant
rs1344563062	genic_downstream_transcript_variant,intron_variant
rs1344642921	intron_variant
rs1344648064	genic_downstream_transcript_variant,intron_variant
rs1344651260	intron_variant,genic_upstream_transcript_variant
rs1344717938	genic_downstream_transcript_variant,intron_variant
rs1344737127	intron_variant
rs1344761436	genic_downstream_transcript_variant,intron_variant
rs1344782183	intron_variant,genic_upstream_transcript_variant
rs1344872497	intron_variant,genic_upstream_transcript_variant
rs1344876785	intron_variant,genic_upstream_transcript_variant
rs1344889163	intron_variant
rs1344962806	intron_variant,upstream_transcript_variant,genic_upstream_transcript_variant
rs1344969402	non_coding_transcript_variant,3_prime_UTR_variant,genic_downstream_transcript_variant
rs1344980497	intron_variant
rs1344989128	intron_variant,upstream_transcript_variant,genic_upstream_transcript_variant
rs1344991350	intron_variant
rs1345001793	intron_variant,genic_upstream_transcript_variant
rs1345059696	intron_variant
rs1345075643	non_coding_transcript_variant,3_prime_UTR_variant,genic_downstream_transcript_variant
rs1345085602	intron_variant
rs1345107152	intron_variant,genic_upstream_transcript_variant
rs1345162038	intron_variant
rs1345212101	genic_downstream_transcript_variant,intron_variant
rs1345244655	intron_variant,genic_upstream_transcript_variant
rs1345247311	genic_downstream_transcript_variant,intron_variant
rs1345256864	intron_variant,genic_upstream_transcript_variant
rs1345272234	intron_variant
rs1345273237	intron_variant,genic_upstream_transcript_variant
rs1345302567	intron_variant,genic_upstream_transcript_variant
rs1345346817	intron_variant,genic_upstream_transcript_variant
rs1345348634	intron_variant
rs1345365382	intron_variant,genic_upstream_transcript_variant
rs1345377535	non_coding_transcript_variant,3_prime_UTR_variant,genic_downstream_transcript_variant
rs1345380298	genic_downstream_transcript_variant,intron_variant
rs1345380388	intron_variant,genic_upstream_transcript_variant
rs1345398908	intron_variant
rs1345403558	intron_variant
rs1345450709	intron_variant,genic_upstream_transcript_variant
rs1345451155	intron_variant
rs1345472532	genic_downstream_transcript_variant,intron_variant
rs1345488283	intron_variant
rs1345494226	intron_variant
rs1345510115	genic_downstream_transcript_variant,intron_variant
rs1345511794	intron_variant,genic_upstream_transcript_variant
rs1345522425	intron_variant
rs1345545341	intron_variant,genic_upstream_transcript_variant
rs1345557081	genic_downstream_transcript_variant,intron_variant
rs1345591332	intron_variant
rs1345607283	2KB_upstream_variant,intron_variant,upstream_transcript_variant,genic_upstream_transcript_variant
rs1345608539	intron_variant
rs1345617396	2KB_upstream_variant,intron_variant,upstream_transcript_variant,genic_upstream_transcript_variant
rs1345664285	intron_variant
rs1345667396	intron_variant,genic_upstream_transcript_variant
rs1345672182	intron_variant,genic_upstream_transcript_variant
rs1345673956	intron_variant,genic_upstream_transcript_variant
rs1345681864	intron_variant
rs1345699239	intron_variant
rs1345769310	intron_variant,upstream_transcript_variant,genic_upstream_transcript_variant
rs1345774417	intron_variant,genic_upstream_transcript_variant
rs1345776014	genic_downstream_transcript_variant,intron_variant

rs1345795109	genic_downstream_transcript_variant,intron_variant
rs1345802169	genic_downstream_transcript_variant,intron_variant
rs1345850536	intron_variant,genic_upstream_transcript_variant
rs1345851722	intron_variant,genic_upstream_transcript_variant
rs1345857520	non_coding_transcript_variant,intron_variant,coding_sequence_variant,missense_variant,genic_upstream_transcript_variant
rs1345932692	intron_variant,genic_upstream_transcript_variant
rs1345947614	intron_variant,genic_upstream_transcript_variant
rs1345978560	intron_variant
rs1346002112	intron_variant,genic_upstream_transcript_variant
rs1346005111	intron_variant,genic_upstream_transcript_variant
rs1346018097	intron_variant
rs1346031676	intron_variant,genic_upstream_transcript_variant
rs1346033934	genic_downstream_transcript_variant,intron_variant
rs1346042634	intron_variant,genic_upstream_transcript_variant
rs1346139588	intron_variant
rs1346253737	intron_variant,genic_upstream_transcript_variant
rs1346256946	genic_downstream_transcript_variant,intron_variant
rs1346277635	intron_variant
rs1346366082	intron_variant,genic_upstream_transcript_variant
rs1346372147	genic_downstream_transcript_variant,intron_variant
rs1346382926	intron_variant,genic_upstream_transcript_variant
rs1346391573	intron_variant,genic_upstream_transcript_variant
rs1346406065	genic_upstream_transcript_variant,intron_variant,upstream_transcript_variant,2KB_upstream_variant
rs1346438165	intron_variant,genic_upstream_transcript_variant
rs1346445675	intron_variant
rs1346459780	genic_upstream_transcript_variant,intron_variant
rs1346491930	intron_variant
rs1346525560	genic_upstream_transcript_variant,upstream_transcript_variant,intron_variant
rs1346537002	genic_downstream_transcript_variant,intron_variant
rs1346546327	genic_downstream_transcript_variant,intron_variant
rs1346563051	genic_upstream_transcript_variant,intron_variant
rs1346572945	genic_upstream_transcript_variant,intron_variant
rs1346631305	intron_variant
rs1346671591	genic_upstream_transcript_variant,2KB_upstream_variant,intron_variant,upstream_transcript_variant
rs1346753195	genic_upstream_transcript_variant,intron_variant
rs1346773991	intron_variant
rs1346799202	genic_downstream_transcript_variant,intron_variant
rs1346800125	genic_upstream_transcript_variant,intron_variant
rs1346863327	intron_variant
rs1346868420	genic_upstream_transcript_variant,intron_variant
rs1346868648	genic_downstream_transcript_variant,intron_variant
rs1346914646	intron_variant
rs1346916475	genic_downstream_transcript_variant,intron_variant
rs1346922386	genic_upstream_transcript_variant,intron_variant
rs1346963309	intron_variant
rs1346985134	genic_upstream_transcript_variant,2KB_upstream_variant,intron_variant,upstream_transcript_variant
rs1347014302	genic_upstream_transcript_variant,intron_variant
rs1347024955	genic_upstream_transcript_variant,intron_variant
rs1347029293	intron_variant
rs1347031392	genic_upstream_transcript_variant,intron_variant
rs1347035704	intron_variant
rs1347090299	genic_upstream_transcript_variant,intron_variant
rs1347098677	intron_variant
rs1347135941	non_coding_transcript_variant,3_prime_UTR_variant,genic_downstream_transcript_variant
rs1347217377	intron_variant
rs1347254562	genic_upstream_transcript_variant,intron_variant
rs1347282546	genic_downstream_transcript_variant,intron_variant
rs1347299309	genic_upstream_transcript_variant,intron_variant
rs1347317504	genic_downstream_transcript_variant,intron_variant
rs1347343321	genic_downstream_transcript_variant,intron_variant
rs1347359907	genic_upstream_transcript_variant,intron_variant
rs1347387694	genic_upstream_transcript_variant,intron_variant
rs1347424934	genic_downstream_transcript_variant,intron_variant
rs1347429977	genic_downstream_transcript_variant,intron_variant
rs1347453693	intron_variant
rs1347483123	genic_downstream_transcript_variant,intron_variant
rs1347507639	intron_variant
rs1347538005	intron_variant
rs1347605036	genic_upstream_transcript_variant,intron_variant
rs1347619289	genic_upstream_transcript_variant,intron_variant
rs1347654408	intron_variant
rs1347660993	genic_downstream_transcript_variant,intron_variant
rs1347670168	genic_upstream_transcript_variant,intron_variant
rs1347687514	genic_upstream_transcript_variant,intron_variant

rs1347689589	genic_downstream_transcript_variant,intron_variant
rs1347736125	intron_variant
rs1347737002	non_coding_transcript_variant,missense_variant,genic_downstream_transcript_variant,coding_sequence_variant
rs1347751396	genic_upstream_transcript_variant,intron_variant
rs1347753004	genic_upstream_transcript_variant,2KB_upstream_variant,intron_variant,upstream_transcript_variant
rs1347821032	genic_upstream_transcript_variant,intron_variant
rs1347886925	genic_upstream_transcript_variant,intron_variant
rs1347961257	genic_upstream_transcript_variant,intron_variant
rs1347997990	genic_upstream_transcript_variant,intron_variant
rs1348001734	genic_downstream_transcript_variant,intron_variant
rs1348034286	genic_upstream_transcript_variant,intron_variant
rs1348081015	genic_upstream_transcript_variant,intron_variant
rs1348081272	intron_variant
rs1348096668	genic_upstream_transcript_variant,intron_variant
rs1348123448	intron_variant
rs1348202567	genic_upstream_transcript_variant,2KB_upstream_variant,upstream_transcript_variant,intron_variant
rs1348218284	genic_upstream_transcript_variant,intron_variant
rs1348232582	genic_upstream_transcript_variant,intron_variant
rs1348251462	non_coding_transcript_variant,synonymous_variant,coding_sequence_variant
rs1348308327	genic_upstream_transcript_variant,upstream_transcript_variant,intron_variant
rs1348351473	genic_upstream_transcript_variant,intron_variant
rs1348354147	genic_upstream_transcript_variant,intron_variant
rs1348454209	genic_upstream_transcript_variant,intron_variant
rs1348479090	intron_variant
rs1348514662	genic_downstream_transcript_variant,intron_variant
rs1348537909	genic_downstream_transcript_variant,intron_variant
rs1348559967	genic_upstream_transcript_variant,intron_variant
rs1348582788	intron_variant
rs1348591008	genic_upstream_transcript_variant,intron_variant
rs1348602093	genic_upstream_transcript_variant,intron_variant
rs1348679801	genic_upstream_transcript_variant,intron_variant
rs1348697579	genic_upstream_transcript_variant,upstream_transcript_variant,intron_variant
rs1348720324	genic_upstream_transcript_variant,intron_variant
rs1348770354	intron_variant
rs1348804266	intron_variant
rs1348809709	intron_variant
rs1348826248	genic_upstream_transcript_variant,intron_variant
rs1348841679	non_coding_transcript_variant,missense_variant,coding_sequence_variant
rs1348933541	genic_upstream_transcript_variant,intron_variant
rs1348952127	genic_downstream_transcript_variant,intron_variant
rs1348973580	intron_variant
rs1348978879	genic_downstream_transcript_variant,intron_variant
rs1349029495	genic_downstream_transcript_variant,intron_variant
rs1349031337	genic_upstream_transcript_variant,intron_variant
rs1349044989	genic_upstream_transcript_variant,intron_variant
rs1349081796	genic_upstream_transcript_variant,intron_variant
rs1349114861	genic_upstream_transcript_variant,intron_variant,5_prime_UTR_variant,upstream_transcript_variant
rs1349150963	non_coding_transcript_variant,3_prime_UTR_variant,genic_downstream_transcript_variant
rs1349192656	intron_variant
rs1349224260	genic_upstream_transcript_variant,intron_variant
rs1349242213	genic_downstream_transcript_variant,intron_variant
rs1349267470	genic_downstream_transcript_variant,intron_variant
rs1349269997	genic_downstream_transcript_variant,intron_variant
rs1349287659	intron_variant
rs1349370738	intron_variant
rs1349386552	intron_variant
rs1349401266	genic_downstream_transcript_variant,intron_variant
rs1349412983	genic_upstream_transcript_variant,2KB_upstream_variant,intron_variant,upstream_transcript_variant
rs1349416293	genic_downstream_transcript_variant,intron_variant
rs1349437539	genic_downstream_transcript_variant,intron_variant
rs1349443727	genic_upstream_transcript_variant,intron_variant
rs1349447437	genic_upstream_transcript_variant,2KB_upstream_variant,intron_variant,upstream_transcript_variant
rs1349457728	genic_downstream_transcript_variant,intron_variant
rs1349460819	genic_downstream_transcript_variant,intron_variant
rs1349560405	intron_variant
rs1349561470	genic_upstream_transcript_variant,intron_variant
rs1349613502	intron_variant
rs1349631995	intron_variant
rs1349632232	genic_downstream_transcript_variant,intron_variant
rs1349710915	intron_variant
rs1349731282	intron_variant
rs1349736401	genic_downstream_transcript_variant,intron_variant
rs1349791092	genic_upstream_transcript_variant,intron_variant
rs1349793907	genic_upstream_transcript_variant,intron_variant

rs1349820824	genic_downstream_transcript_variant,intron_variant
rs1349827644	intron_variant
rs1349833588	genic_downstream_transcript_variant,intron_variant
rs1349835800	genic_upstream_transcript_variant,intron_variant
rs1349912980	intron_variant
rs1349945025	intron_variant
rs1349947834	genic_upstream_transcript_variant,intron_variant
rs1350038140	genic_upstream_transcript_variant,intron_variant
rs1350055738	genic_upstream_transcript_variant,intron_variant
rs1350069995	intron_variant
rs1350070170	genic_downstream_transcript_variant,intron_variant
rs1350111216	genic_upstream_transcript_variant,intron_variant
rs1350164030	intron_variant
rs1350192437	genic_downstream_transcript_variant,intron_variant
rs1350264736	genic_upstream_transcript_variant,intron_variant
rs1350282779	genic_upstream_transcript_variant,2KB_upstream_variant,intron_variant,upstream_transcript_variant
rs1350293447	genic_upstream_transcript_variant,intron_variant
rs1350311480	intron_variant
rs1350342239	genic_upstream_transcript_variant,intron_variant
rs1350402208	intron_variant
rs1350423153	genic_downstream_transcript_variant,intron_variant
rs1350442422	genic_upstream_transcript_variant,intron_variant
rs1350459796	intron_variant
rs1350473304	intron_variant
rs1350496151	genic_downstream_transcript_variant,intron_variant
rs1350511700	genic_downstream_transcript_variant,intron_variant
rs1350538518	genic_upstream_transcript_variant,intron_variant
rs1350645135	genic_upstream_transcript_variant,intron_variant
rs1350649711	intron_variant
rs1350670891	intron_variant
rs1350675858	genic_upstream_transcript_variant,intron_variant
rs1350685826	intron_variant
rs1350728182	genic_upstream_transcript_variant,intron_variant
rs1350750087	genic_downstream_transcript_variant,intron_variant
rs1350755691	non_coding_transcript_variant,3_prime_UTR_variant,genic_downstream_transcript_variant
rs1350775209	intron_variant
rs1350808964	intron_variant
rs1350848955	intron_variant
rs1350858633	genic_upstream_transcript_variant,2KB_upstream_variant,intron_variant,upstream_transcript_variant
rs1350860464	genic_downstream_transcript_variant,intron_variant
rs1350874568	genic_upstream_transcript_variant,2KB_upstream_variant,intron_variant,upstream_transcript_variant
rs1350889917	intron_variant
rs1350930754	genic_downstream_transcript_variant,intron_variant
rs1350962296	intron_variant
rs1350967660	downstream_transcript_variant,genic_downstream_transcript_variant,intron_variant
rs1350969026	genic_upstream_transcript_variant,intron_variant
rs1351036076	intron_variant
rs1351043132	intron_variant
rs1351070608	genic_downstream_transcript_variant,intron_variant
rs1351083834	downstream_transcript_variant,genic_downstream_transcript_variant,intron_variant
rs1351132332	genic_upstream_transcript_variant,2KB_upstream_variant,upstream_transcript_variant,intron_variant
rs1351165181	intron_variant
rs1351171595	intron_variant
rs1351182810	intron_variant
rs1351192155	genic_upstream_transcript_variant,intron_variant
rs1351192699	genic_upstream_transcript_variant,intron_variant
rs1351210098	genic_downstream_transcript_variant,intron_variant
rs1351264833	non_coding_transcript_variant,missense_variant,coding_sequence_variant
rs1351295846	genic_upstream_transcript_variant,intron_variant
rs1351385255	genic_upstream_transcript_variant,intron_variant
rs1351387400	genic_downstream_transcript_variant,intron_variant
rs1351394598	genic_upstream_transcript_variant,intron_variant
rs1351402141	intron_variant
rs1351442838	intron_variant
rs1351448764	genic_upstream_transcript_variant,upstream_transcript_variant,intron_variant
rs1351475769	genic_downstream_transcript_variant,intron_variant
rs1351511652	genic_upstream_transcript_variant,intron_variant
rs1351525859	intron_variant
rs1351527149	genic_downstream_transcript_variant,intron_variant
rs1351541087	genic_downstream_transcript_variant,intron_variant
rs1351583207	intron_variant
rs1351650393	intron_variant
rs1351668958	genic_upstream_transcript_variant,2KB_upstream_variant,upstream_transcript_variant,intron_variant
rs1351672109	intron_variant

rs1351678757	genic_upstream_transcript_variant,intron_variant
rs1351704846	genic_upstream_transcript_variant,intron_variant
rs1351737025	intron_variant
rs1351742428	genic_downstream_transcript_variant,intron_variant
rs1351778229	genic_downstream_transcript_variant,intron_variant
rs1351783172	genic_upstream_transcript_variant,upstream_transcript_variant,intron_variant
rs1351792123	genic_upstream_transcript_variant,intron_variant
rs1351811919	genic_downstream_transcript_variant,intron_variant
rs1351827833	genic_downstream_transcript_variant,intron_variant
rs1351854488	intron_variant
rs1351880689	genic_upstream_transcript_variant,intron_variant
rs1351911707	genic_upstream_transcript_variant,intron_variant
rs1351915320	genic_downstream_transcript_variant,intron_variant
rs1351935080	intron_variant
rs1351982820	intron_variant
rs1352017920	genic_upstream_transcript_variant,intron_variant
rs1352046770	genic_downstream_transcript_variant,intron_variant
rs1352074410	genic_downstream_transcript_variant,intron_variant
rs1352089193	genic_upstream_transcript_variant,intron_variant
rs1352126032	intron_variant
rs1352136857	intron_variant
rs1352168002	genic_upstream_transcript_variant,intron_variant
rs1352202377	intron_variant
rs1352212645	genic_upstream_transcript_variant,intron_variant
rs1352263140	genic_upstream_transcript_variant,upstream_transcript_variant,intron_variant
rs1352282130	intron_variant
rs1352292316	genic_upstream_transcript_variant,intron_variant
rs1352302316	intron_variant
rs1352337226	genic_downstream_transcript_variant,intron_variant
rs1352338219	intron_variant
rs1352339046	intron_variant
rs1352343997	genic_upstream_transcript_variant,intron_variant
rs1352353948	genic_downstream_transcript_variant,intron_variant
rs1352435675	genic_upstream_transcript_variant,intron_variant
rs1352470359	genic_upstream_transcript_variant,intron_variant
rs1352478752	genic_downstream_transcript_variant,intron_variant
rs1352487633	genic_downstream_transcript_variant,intron_variant
rs1352555756	genic_downstream_transcript_variant,intron_variant
rs1352560909	genic_upstream_transcript_variant,intron_variant
rs1352564413	genic_downstream_transcript_variant,intron_variant
rs1352566238	genic_upstream_transcript_variant,intron_variant
rs1352578573	genic_upstream_transcript_variant,2KB_upstream_variant,upstream_transcript_variant,intron_variant
rs1352602498	intron_variant
rs1352606563	genic_downstream_transcript_variant,intron_variant
rs1352641926	genic_upstream_transcript_variant,intron_variant
rs1352691383	genic_downstream_transcript_variant,intron_variant
rs1352729221	genic_upstream_transcript_variant,upstream_transcript_variant,intron_variant
rs1352735958	intron_variant
rs1352758140	genic_upstream_transcript_variant,intron_variant
rs1352761667	genic_upstream_transcript_variant,non_coding_transcript_variant,intron_variant,synonymous_variant,coding_sequence_variant
rs1352772871	genic_upstream_transcript_variant,intron_variant
rs1352779547	intron_variant
rs1352830409	genic_upstream_transcript_variant,upstream_transcript_variant,intron_variant
rs1352837438	genic_downstream_transcript_variant,intron_variant
rs1352857140	intron_variant
rs1352860038	genic_upstream_transcript_variant,intron_variant
rs1352863206	intron_variant
rs1352874187	intron_variant
rs1352892055	intron_variant
rs1352916958	intron_variant
rs1352941807	intron_variant
rs1352971438	genic_upstream_transcript_variant,intron_variant
rs1353007586	genic_upstream_transcript_variant,intron_variant
rs1353015135	intron_variant
rs1353070074	genic_downstream_transcript_variant,intron_variant
rs1353095486	genic_downstream_transcript_variant,intron_variant
rs1353118536	genic_downstream_transcript_variant,intron_variant
rs1353133879	genic_downstream_transcript_variant,intron_variant
rs1353170333	genic_upstream_transcript_variant,intron_variant
rs1353178670	genic_upstream_transcript_variant,intron_variant
rs1353198512	intron_variant
rs1353249876	genic_upstream_transcript_variant,intron_variant
rs1353259142	intron_variant
rs1353279424	intron_variant

rs1353329465	genic_upstream_transcript_variant,2KB_upstream_variant,upstream_transcript_variant,intron_variant
rs1353397392	intron_variant
rs1353448417	genic_upstream_transcript_variant,intron_variant
rs1353557106	genic_upstream_transcript_variant,intron_variant
rs1353611697	intron_variant
rs1353612134	intron_variant
rs1353630836	genic_upstream_transcript_variant,intron_variant
rs1353645459	genic_downstream_transcript_variant,intron_variant
rs1353712617	genic_upstream_transcript_variant,intron_variant
rs1353716199	genic_downstream_transcript_variant,intron_variant
rs1353731578	genic_downstream_transcript_variant,intron_variant
rs1353818791	genic_upstream_transcript_variant,intron_variant
rs1353976564	genic_downstream_transcript_variant,intron_variant
rs1354019290	genic_downstream_transcript_variant,intron_variant
rs1354121610	intron_variant
rs1354201129	intron_variant
rs1354227374	genic_upstream_transcript_variant,intron_variant
rs1354278877	genic_upstream_transcript_variant,upstream_transcript_variant,intron_variant
rs1354283359	genic_upstream_transcript_variant,intron_variant
rs1354290053	genic_upstream_transcript_variant,intron_variant
rs1354293833	genic_downstream_transcript_variant,intron_variant
rs1354307983	intron_variant
rs1354332314	genic_upstream_transcript_variant,intron_variant
rs1354348320	genic_upstream_transcript_variant,intron_variant
rs1354354733	intron_variant
rs1354391169	genic_upstream_transcript_variant,intron_variant
rs1354391607	genic_upstream_transcript_variant,2KB_upstream_variant,intron_variant,upstream_transcript_variant
rs1354417151	genic_upstream_transcript_variant,intron_variant
rs1354424778	genic_upstream_transcript_variant,intron_variant
rs1354435204	intron_variant
rs1354498245	genic_upstream_transcript_variant,intron_variant
rs1354500837	genic_downstream_transcript_variant,intron_variant
rs1354520865	genic_upstream_transcript_variant,intron_variant
rs1354524052	genic_upstream_transcript_variant,intron_variant
rs1354541551	intron_variant
rs1354543692	genic_upstream_transcript_variant,intron_variant
rs1354554397	intron_variant
rs1354568968	genic_upstream_transcript_variant,intron_variant
rs1354570519	genic_upstream_transcript_variant,intron_variant
rs1354591573	intron_variant
rs1354636079	intron_variant
rs1354639614	genic_upstream_transcript_variant,intron_variant
rs1354651683	intron_variant
rs1354651917	genic_downstream_transcript_variant,intron_variant
rs1354653058	genic_upstream_transcript_variant,intron_variant
rs1354686397	genic_upstream_transcript_variant,intron_variant
rs1354714508	intron_variant
rs1354740511	genic_downstream_transcript_variant,intron_variant
rs1354775439	intron_variant
rs1354775472	non_coding_transcript_variant,3_prime_UTR_variant,genic_downstream_transcript_variant
rs1354779493	intron_variant
rs1354782688	genic_upstream_transcript_variant,intron_variant
rs1354785720	intron_variant
rs1354862909	genic_upstream_transcript_variant,intron_variant
rs1354881701	genic_upstream_transcript_variant,upstream_transcript_variant,intron_variant
rs1354895139	genic_downstream_transcript_variant,intron_variant
rs1354911496	intron_variant
rs1354925620	intron_variant
rs1354964108	intron_variant
rs1354984944	genic_upstream_transcript_variant,intron_variant
rs1355028102	genic_upstream_transcript_variant,intron_variant
rs1355058670	genic_downstream_transcript_variant,intron_variant
rs1355075163	genic_downstream_transcript_variant,intron_variant
rs1355100822	non_coding_transcript_variant,synonymous_variant,genic_downstream_transcript_variant,coding_sequence_variant
rs1355101551	intron_variant
rs1355122579	intron_variant
rs1355136867	genic_upstream_transcript_variant,intron_variant
rs1355144622	genic_upstream_transcript_variant,intron_variant
rs1355144759	intron_variant
rs1355165187	genic_upstream_transcript_variant,2KB_upstream_variant,upstream_transcript_variant,intron_variant
rs1355180273	intron_variant
rs1355198989	intron_variant
rs1355218012	genic_downstream_transcript_variant,intron_variant
rs1355239358	genic_upstream_transcript_variant,intron_variant

rs1355280937	intron_variant
rs1355299660	genic_upstream_transcript_variant,intron_variant
rs1355306497	intron_variant
rs1355320235	genic_upstream_transcript_variant,2KB_upstream_variant,upstream_transcript_variant,intron_variant
rs1355332286	intron_variant
rs1355374889	genic_upstream_transcript_variant,upstream_transcript_variant,intron_variant
rs1355391983	genic_upstream_transcript_variant,intron_variant
rs1355428376	intron_variant
rs1355440320	genic_upstream_transcript_variant,upstream_transcript_variant,intron_variant
rs1355484626	intron_variant
rs1355544533	intron_variant
rs1355663759	genic_downstream_transcript_variant,intron_variant
rs1355684997	genic_upstream_transcript_variant,intron_variant
rs1355694178	intron_variant
rs1355709450	intron_variant
rs1355767116	genic_upstream_transcript_variant,2KB_upstream_variant,upstream_transcript_variant,intron_variant
rs1355798175	intron_variant
rs1355820389	genic_downstream_transcript_variant,intron_variant
rs1355873895	genic_upstream_transcript_variant,2KB_upstream_variant,intron_variant,upstream_transcript_variant
rs1355906176	genic_downstream_transcript_variant,intron_variant
rs1355924895	intron_variant
rs1355949775	genic_downstream_transcript_variant,intron_variant
rs1355964684	genic_upstream_transcript_variant,intron_variant
rs1355987904	genic_upstream_transcript_variant,non_coding_transcript_variant,5_prime_UTR_variant,intron_variant
rs1355990511	genic_downstream_transcript_variant,intron_variant
rs1355998204	genic_upstream_transcript_variant,intron_variant
rs1356022788	intron_variant
rs1356043766	genic_upstream_transcript_variant,intron_variant
rs1356052241	genic_upstream_transcript_variant,intron_variant
rs1356055623	intron_variant
rs1356107249	genic_upstream_transcript_variant,intron_variant
rs1356112435	intron_variant
rs1356140735	genic_upstream_transcript_variant,intron_variant
rs1356159933	intron_variant
rs1356171633	genic_upstream_transcript_variant,2KB_upstream_variant,upstream_transcript_variant,intron_variant
rs1356239940	genic_upstream_transcript_variant,intron_variant,5_prime_UTR_variant,upstream_transcript_variant
rs1356255068	genic_upstream_transcript_variant,intron_variant
rs1356282089	genic_downstream_transcript_variant,intron_variant
rs1356364729	intron_variant
rs1356376883	genic_downstream_transcript_variant,intron_variant
rs1356416799	genic_downstream_transcript_variant,intron_variant
rs1356424482	intron_variant
rs1356427557	intron_variant
rs1356445659	genic_upstream_transcript_variant,intron_variant
rs1356480828	intron_variant
rs1356500675	intron_variant
rs1356528138	genic_upstream_transcript_variant,intron_variant
rs1356562963	genic_upstream_transcript_variant,intron_variant
rs1356588179	intron_variant
rs1356646352	genic_upstream_transcript_variant,intron_variant
rs1356699546	genic_upstream_transcript_variant,intron_variant
rs1356701074	intron_variant
rs1356710358	genic_upstream_transcript_variant,intron_variant
rs1356749515	non_coding_transcript_variant,3_prime_UTR_variant,genic_downstream_transcript_variant
rs1356767689	intron_variant
rs1356807231	intron_variant
rs1356868153	genic_upstream_transcript_variant,intron_variant
rs1356903297	intron_variant
rs1356905174	genic_upstream_transcript_variant,intron_variant
rs1356911511	genic_upstream_transcript_variant,intron_variant
rs1356928659	genic_upstream_transcript_variant,intron_variant
rs1356938801	intron_variant
rs1356955157	genic_upstream_transcript_variant,2KB_upstream_variant,intron_variant,upstream_transcript_variant
rs1356960569	genic_downstream_transcript_variant,intron_variant
rs1357041710	genic_upstream_transcript_variant,non_coding_transcript_variant,5_prime_UTR_variant,intron_variant
rs1357043145	genic_upstream_transcript_variant,intron_variant
rs1357056117	genic_upstream_transcript_variant,intron_variant
rs1357128457	genic_downstream_transcript_variant,intron_variant
rs1357143582	genic_upstream_transcript_variant,intron_variant
rs1357151176	intron_variant
rs1357153890	intron_variant
rs1357265123	genic_upstream_transcript_variant,intron_variant
rs1357279459	genic_upstream_transcript_variant,upstream_transcript_variant,intron_variant
rs1357310865	genic_upstream_transcript_variant,intron_variant

rs1357313956	genic_downstream_transcript_variant,intron_variant
rs1357350458	non_coding_transcript_variant,3_prime_UTR_variant,genic_downstream_transcript_variant
rs1357352399	genic_upstream_transcript_variant,intron_variant
rs1357379878	genic_downstream_transcript_variant,intron_variant
rs1357385277	genic_upstream_transcript_variant,intron_variant
rs1357412101	genic_upstream_transcript_variant,upstream_transcript_variant,intron_variant
rs1357412698	genic_upstream_transcript_variant,intron_variant
rs1357428950	intron_variant
rs1357435143	intron_variant
rs1357453854	missense_variant,genic_upstream_transcript_variant,non_coding_transcript_variant,intron_variant,coding_sequence_variant
rs1357478725	intron_variant
rs1357487657	genic_upstream_transcript_variant,intron_variant
rs1357502290	intron_variant
rs1357512697	genic_upstream_transcript_variant,2KB_upstream_variant,intron_variant,upstream_transcript_variant
rs1357514230	genic_upstream_transcript_variant,intron_variant
rs1357552046	intron_variant
rs1357581184	intron_variant
rs1357604473	intron_variant
rs1357624089	intron_variant
rs1357628123	genic_upstream_transcript_variant,intron_variant
rs1357660311	genic_upstream_transcript_variant,intron_variant
rs1357674788	intron_variant
rs1357701027	intron_variant
rs1357715815	genic_upstream_transcript_variant,intron_variant
rs1357743613	genic_upstream_transcript_variant,intron_variant
rs1357764260	genic_upstream_transcript_variant,2KB_upstream_variant,upstream_transcript_variant,intron_variant
rs1357770062	genic_upstream_transcript_variant,intron_variant
rs1357777139	genic_upstream_transcript_variant,intron_variant
rs1357784591	genic_downstream_transcript_variant,intron_variant
rs1357798038	intron_variant
rs1357852540	intron_variant
rs1357878521	genic_upstream_transcript_variant,intron_variant
rs1357892881	genic_downstream_transcript_variant,intron_variant
rs1357966325	genic_upstream_transcript_variant,intron_variant
rs1357998666	genic_upstream_transcript_variant,intron_variant
rs1357998676	genic_upstream_transcript_variant,intron_variant
rs1358056439	genic_upstream_transcript_variant,intron_variant
rs1358064569	intron_variant
rs1358079693	genic_upstream_transcript_variant,intron_variant
rs1358080640	genic_upstream_transcript_variant,upstream_transcript_variant,intron_variant
rs1358106817	intron_variant
rs1358118948	genic_upstream_transcript_variant,intron_variant
rs1358133031	intron_variant
rs1358133149	genic_upstream_transcript_variant,intron_variant
rs1358142623	genic_upstream_transcript_variant,intron_variant
rs1358197090	genic_downstream_transcript_variant,intron_variant
rs1358242907	intron_variant
rs1358269357	intron_variant
rs1358300299	genic_downstream_transcript_variant,intron_variant
rs1358351135	genic_upstream_transcript_variant,intron_variant
rs1358364228	genic_upstream_transcript_variant,intron_variant
rs1358398757	genic_downstream_transcript_variant,intron_variant
rs1358403500	intron_variant
rs1358409191	intron_variant
rs1358456501	genic_upstream_transcript_variant,intron_variant
rs1358459698	genic_upstream_transcript_variant,upstream_transcript_variant,intron_variant
rs1358471387	non_coding_transcript_variant,3_prime_UTR_variant,genic_downstream_transcript_variant
rs1358482892	genic_downstream_transcript_variant,intron_variant
rs1358505053	genic_downstream_transcript_variant,intron_variant
rs1358519925	genic_upstream_transcript_variant,2KB_upstream_variant,upstream_transcript_variant,intron_variant
rs1358595037	genic_downstream_transcript_variant,intron_variant
rs1358699390	intron_variant
rs1358701641	intron_variant
rs1358742985	intron_variant
rs1358780675	intron_variant
rs1358786712	genic_upstream_transcript_variant,2KB_upstream_variant,upstream_transcript_variant,intron_variant
rs1358816923	500B_downstream_variant,downstream_transcript_variant
rs1358836653	genic_downstream_transcript_variant,intron_variant
rs1358882208	intron_variant
rs1358892766	intron_variant
rs1358895258	genic_upstream_transcript_variant,intron_variant
rs1358896755	genic_upstream_transcript_variant,intron_variant
rs1358929536	genic_upstream_transcript_variant,2KB_upstream_variant,upstream_transcript_variant,intron_variant
rs1358931345	intron_variant

rs1358938551	intron_variant
rs1358961027	intron_variant
rs1358961870	genic_downstream_transcript_variant,intron_variant
rs1358997308	genic_downstream_transcript_variant,intron_variant
rs1359019914	genic_upstream_transcript_variant,intron_variant
rs1359046151	genic_downstream_transcript_variant,intron_variant
rs1359105842	intron_variant
rs1359115935	intron_variant
rs1359163244	2KB_upstream_variant,intron_variant,upstream_transcript_variant,genic_upstream_transcript_variant
rs1359200595	intron_variant,genic_upstream_transcript_variant
rs1359208244	intron_variant
rs1359316654	intron_variant
rs1359341222	intron_variant,genic_upstream_transcript_variant
rs1359366276	intron_variant,genic_downstream_transcript_variant
rs1359368382	intron_variant,genic_upstream_transcript_variant
rs1359388735	intron_variant
rs1359391569	intron_variant,genic_downstream_transcript_variant
rs1359407422	intron_variant
rs1359451522	intron_variant
rs1359474280	intron_variant,genic_downstream_transcript_variant
rs1359491678	intron_variant
rs1359579263	intron_variant
rs1359626495	intron_variant,genic_downstream_transcript_variant
rs1359644270	intron_variant,genic_downstream_transcript_variant
rs1359647855	intron_variant,genic_upstream_transcript_variant
rs1359669586	intron_variant
rs1359695332	intron_variant,genic_upstream_transcript_variant
rs1359709779	intron_variant,genic_downstream_transcript_variant
rs1359736726	intron_variant,genic_downstream_transcript_variant
rs1359771462	intron_variant
rs1359777694	intron_variant,genic_downstream_transcript_variant
rs1359809768	intron_variant
rs1359826498	intron_variant,genic_downstream_transcript_variant
rs1359849508	intron_variant,genic_upstream_transcript_variant
rs1359882487	intron_variant,genic_downstream_transcript_variant
rs1359940129	intron_variant,genic_upstream_transcript_variant
rs1360035784	intron_variant,genic_upstream_transcript_variant
rs1360043532	intron_variant
rs1360048751	intron_variant
rs1360065658	intron_variant,genic_upstream_transcript_variant
rs1360092682	intron_variant
rs1360109575	intron_variant,genic_downstream_transcript_variant
rs1360112268	intron_variant,genic_upstream_transcript_variant
rs1360154886	intron_variant,genic_upstream_transcript_variant
rs1360163644	intron_variant,genic_upstream_transcript_variant
rs1360174387	intron_variant,5_prime_UTR_variant,upstream_transcript_variant,genic_upstream_transcript_variant
rs1360199227	intron_variant,genic_downstream_transcript_variant
rs1360225756	intron_variant
rs1360287480	intron_variant,genic_upstream_transcript_variant
rs1360364776	intron_variant,genic_upstream_transcript_variant
rs1360374243	2KB_upstream_variant,intron_variant,upstream_transcript_variant,genic_upstream_transcript_variant
rs1360378472	intron_variant,genic_upstream_transcript_variant
rs1360397403	intron_variant
rs1360428765	2KB_upstream_variant,intron_variant,upstream_transcript_variant,genic_upstream_transcript_variant
rs1360446030	intron_variant,genic_upstream_transcript_variant
rs1360454642	intron_variant,genic_downstream_transcript_variant
rs1360458852	intron_variant,genic_downstream_transcript_variant
rs1360463500	intron_variant,genic_upstream_transcript_variant
rs1360485915	intron_variant
rs1360490339	intron_variant,genic_downstream_transcript_variant
rs1360515898	intron_variant,genic_upstream_transcript_variant
rs1360583011	intron_variant
rs1360631133	intron_variant,genic_downstream_transcript_variant
rs1360640194	intron_variant,genic_upstream_transcript_variant
rs1360685627	intron_variant
rs1360688918	intron_variant
rs1360743509	intron_variant,genic_upstream_transcript_variant
rs1360751951	intron_variant
rs1360762307	intron_variant,genic_downstream_transcript_variant
rs1360763384	intron_variant
rs1360778929	intron_variant
rs1360929240	intron_variant,upstream_transcript_variant,genic_upstream_transcript_variant
rs1360947173	intron_variant,genic_upstream_transcript_variant
rs1360951820	intron_variant,upstream_transcript_variant,genic_upstream_transcript_variant

rs1361027837	intron_variant,genic_upstream_transcript_variant
rs1361035455	intron_variant
rs1361066921	intron_variant
rs1361089276	intron_variant,genic_upstream_transcript_variant
rs1361091037	intron_variant
rs1361110969	intron_variant,genic_upstream_transcript_variant
rs1361150862	intron_variant,genic_upstream_transcript_variant
rs1361219309	intron_variant
rs1361248622	intron_variant,genic_upstream_transcript_variant
rs1361269530	intron_variant,genic_upstream_transcript_variant
rs1361270771	intron_variant,genic_upstream_transcript_variant
rs1361278684	intron_variant
rs1361296945	intron_variant,genic_upstream_transcript_variant
rs1361308704	intron_variant
rs1361330351	intron_variant,genic_upstream_transcript_variant
rs1361337132	intron_variant,upstream_transcript_variant,genic_upstream_transcript_variant
rs1361337787	2KB_upstream_variant,intron_variant,upstream_transcript_variant,genic_upstream_transcript_variant
rs1361419745	intron_variant,genic_upstream_transcript_variant
rs1361444629	intron_variant
rs1361472281	intron_variant
rs1361475306	intron_variant,genic_upstream_transcript_variant
rs1361492274	intron_variant
rs1361506826	intron_variant,genic_upstream_transcript_variant
rs1361517822	intron_variant,genic_upstream_transcript_variant
rs1361532494	intron_variant,genic_upstream_transcript_variant
rs1361535658	intron_variant,genic_upstream_transcript_variant
rs1361629762	intron_variant,genic_upstream_transcript_variant
rs1361647753	intron_variant,genic_upstream_transcript_variant
rs1361706135	intron_variant,genic_upstream_transcript_variant
rs1361706649	intron_variant,genic_upstream_transcript_variant
rs1361708302	2KB_upstream_variant,intron_variant,upstream_transcript_variant,genic_upstream_transcript_variant
rs1361719900	intron_variant,genic_downstream_transcript_variant
rs1361734777	intron_variant,genic_downstream_transcript_variant
rs1361736609	intron_variant
rs1361765607	intron_variant,genic_upstream_transcript_variant
rs1361780063	intron_variant,genic_upstream_transcript_variant
rs1361822975	intron_variant,genic_upstream_transcript_variant
rs1361892164	downstream_transcript_variant,intron_variant,genic_downstream_transcript_variant
rs1361892520	intron_variant
rs1361897994	intron_variant
rs1361918117	intron_variant,genic_upstream_transcript_variant
rs1361920501	non_coding_transcript_variant,intron_variant,5_prime_UTR_variant,genic_upstream_transcript_variant
rs1361945217	intron_variant
rs1361989102	intron_variant,genic_upstream_transcript_variant
rs1362041289	intron_variant
rs1362047178	intron_variant,genic_upstream_transcript_variant
rs1362061922	intron_variant,genic_upstream_transcript_variant
rs1362073137	intron_variant
rs1362093141	intron_variant
rs1362151522	intron_variant
rs1362156913	intron_variant
rs1362173994	intron_variant,genic_downstream_transcript_variant
rs1362193937	intron_variant
rs1362194917	intron_variant
rs1362222837	intron_variant,genic_upstream_transcript_variant
rs1362238421	intron_variant,genic_upstream_transcript_variant
rs1362260104	intron_variant,genic_upstream_transcript_variant
rs1362261120	intron_variant
rs1362282691	intron_variant
rs1362302823	intron_variant
rs1362329709	intron_variant,genic_upstream_transcript_variant
rs1362396753	intron_variant,genic_upstream_transcript_variant
rs1362397390	intron_variant,genic_downstream_transcript_variant
rs1362399017	intron_variant,genic_upstream_transcript_variant
rs1362413243	intron_variant
rs1362430363	intron_variant
rs1362450923	intron_variant,genic_upstream_transcript_variant
rs1362455776	intron_variant,genic_downstream_transcript_variant
rs1362487462	intron_variant,genic_upstream_transcript_variant
rs1362506145	intron_variant
rs1362553076	intron_variant
rs1362573189	non_coding_transcript_variant,3_prime_UTR_variant,genic_downstream_transcript_variant
rs1362634590	intron_variant,genic_upstream_transcript_variant
rs1362696590	intron_variant,genic_upstream_transcript_variant

rs1362733840	non_coding_transcript_variant,coding_sequence_variant,genic_downstream_transcript_variant,missense_variant
rs1362749650	intron_variant,genic_downstream_transcript_variant
rs1362753843	intron_variant
rs1362755614	intron_variant,genic_upstream_transcript_variant
rs1362760469	intron_variant,genic_upstream_transcript_variant
rs1362772417	intron_variant
rs1362808843	intron_variant
rs1362826904	intron_variant,upstream_transcript_variant,genic_upstream_transcript_variant
rs1362833635	intron_variant,genic_downstream_transcript_variant
rs1362837000	intron_variant,genic_downstream_transcript_variant
rs1362840337	intron_variant,genic_upstream_transcript_variant
rs1362855388	intron_variant,genic_upstream_transcript_variant
rs1362871634	intron_variant,genic_upstream_transcript_variant
rs1362892500	intron_variant,genic_downstream_transcript_variant
rs1362894661	intron_variant,genic_upstream_transcript_variant
rs1362921039	intron_variant,genic_downstream_transcript_variant
rs1362957984	intron_variant,genic_upstream_transcript_variant
rs1362961325	intron_variant,upstream_transcript_variant,genic_upstream_transcript_variant
rs1362971667	intron_variant
rs1362975055	intron_variant
rs1362991416	intron_variant
rs1363059462	intron_variant
rs1363079810	intron_variant,genic_downstream_transcript_variant
rs1363094471	intron_variant,genic_upstream_transcript_variant
rs1363132403	intron_variant
rs1363167838	intron_variant,genic_upstream_transcript_variant
rs1363174219	non_coding_transcript_variant,3_prime_UTR_variant,genic_downstream_transcript_variant
rs1363187970	intron_variant,upstream_transcript_variant,genic_upstream_transcript_variant
rs1363228387	intron_variant
rs1363270849	intron_variant,genic_downstream_transcript_variant
rs1363285731	intron_variant,genic_upstream_transcript_variant
rs1363298049	intron_variant,genic_downstream_transcript_variant
rs1363305196	intron_variant
rs1363317955	intron_variant,genic_downstream_transcript_variant
rs1363338722	intron_variant
rs1363381487	intron_variant
rs1363404466	intron_variant,genic_upstream_transcript_variant
rs1363407146	intron_variant
rs1363407857	intron_variant,genic_downstream_transcript_variant
rs1363415748	intron_variant,genic_upstream_transcript_variant
rs1363453444	intron_variant,genic_downstream_transcript_variant
rs1363459579	intron_variant,genic_downstream_transcript_variant
rs1363480238	intron_variant,genic_downstream_transcript_variant
rs1363488734	intron_variant,genic_upstream_transcript_variant
rs1363551867	non_coding_transcript_variant,coding_sequence_variant,genic_downstream_transcript_variant,missense_variant
rs1363555277	2KB_upstream_variant,intron_variant,upstream_transcript_variant,genic_upstream_transcript_variant
rs1363607564	intron_variant
rs1363615406	non_coding_transcript_variant,3_prime_UTR_variant,genic_downstream_transcript_variant
rs1363622189	intron_variant
rs1363630057	intron_variant
rs1363657408	intron_variant,genic_upstream_transcript_variant
rs1363674169	intron_variant,genic_downstream_transcript_variant
rs1363740793	intron_variant
rs1363745227	intron_variant,genic_upstream_transcript_variant
rs1363746282	intron_variant
rs1363829911	intron_variant,genic_upstream_transcript_variant
rs1363836105	intron_variant
rs1363846362	intron_variant,genic_downstream_transcript_variant
rs1363879382	intron_variant,genic_upstream_transcript_variant
rs1363903287	intron_variant
rs1363904084	intron_variant,genic_upstream_transcript_variant
rs1363907450	intron_variant,genic_downstream_transcript_variant
rs1363908236	intron_variant,genic_upstream_transcript_variant
rs1363928898	intron_variant,genic_upstream_transcript_variant
rs1364013265	non_coding_transcript_variant,3_prime_UTR_variant,genic_downstream_transcript_variant
rs1364044303	intron_variant
rs1364078897	non_coding_transcript_variant,intron_variant,5_prime_UTR_variant,genic_upstream_transcript_variant
rs1364087347	intron_variant,genic_downstream_transcript_variant
rs1364105757	intron_variant,genic_downstream_transcript_variant
rs1364107048	intron_variant
rs1364138054	2KB_upstream_variant,intron_variant,upstream_transcript_variant,genic_upstream_transcript_variant
rs1364158056	intron_variant,genic_downstream_transcript_variant
rs1364167676	intron_variant,genic_upstream_transcript_variant
rs1364174663	intron_variant,genic_upstream_transcript_variant

rs1364186082	intron_variant,genic_upstream_transcript_variant
rs1364195847	intron_variant,genic_downstream_transcript_variant
rs1364233542	intron_variant
rs1364262437	intron_variant
rs1364374480	intron_variant,genic_upstream_transcript_variant
rs1364383095	intron_variant
rs1364401199	intron_variant,genic_upstream_transcript_variant
rs1364445964	intron_variant
rs1364452469	intron_variant
rs1364466436	intron_variant,genic_downstream_transcript_variant
rs1364512912	intron_variant,genic_upstream_transcript_variant
rs1364514718	intron_variant
rs1364575009	intron_variant,genic_upstream_transcript_variant
rs1364604054	intron_variant
rs1364632573	intron_variant
rs1364657964	intron_variant
rs1364660416	intron_variant
rs1364670796	intron_variant,genic_downstream_transcript_variant
rs1364673162	intron_variant,genic_upstream_transcript_variant
rs1364704304	intron_variant,genic_downstream_transcript_variant
rs1364738119	intron_variant
rs1364840261	intron_variant,genic_upstream_transcript_variant
rs1364840315	intron_variant
rs1364853579	intron_variant,genic_upstream_transcript_variant
rs1364874358	intron_variant,genic_downstream_transcript_variant
rs1364881839	intron_variant,genic_upstream_transcript_variant
rs1364892164	intron_variant
rs1364901697	intron_variant,upstream_transcript_variant,genic_upstream_transcript_variant
rs1364911262	intron_variant,upstream_transcript_variant,genic_upstream_transcript_variant
rs1364984780	intron_variant
rs1365027826	downstream_transcript_variant,500B_downstream_variant
rs1365041543	intron_variant
rs1365145003	intron_variant
rs1365147395	intron_variant
rs1365181482	intron_variant
rs1365199940	intron_variant,genic_upstream_transcript_variant
rs1365232016	intron_variant,upstream_transcript_variant,genic_upstream_transcript_variant
rs1365247381	non_coding_transcript_variant,3_prime_UTR_variant,genic_downstream_transcript_variant
rs1365257046	intron_variant
rs1365316645	intron_variant,genic_upstream_transcript_variant
rs1365318926	intron_variant,genic_upstream_transcript_variant
rs1365355100	intron_variant,genic_upstream_transcript_variant
rs1365429263	intron_variant
rs1365463670	intron_variant,5_prime_UTR_variant,upstream_transcript_variant,genic_upstream_transcript_variant
rs1365483013	intron_variant,genic_upstream_transcript_variant
rs1365527871	non_coding_transcript_variant,3_prime_UTR_variant,genic_downstream_transcript_variant
rs1365553431	intron_variant,genic_upstream_transcript_variant
rs1365575797	intron_variant,genic_downstream_transcript_variant
rs1365594274	intron_variant,genic_upstream_transcript_variant
rs1365620989	intron_variant
rs1365626671	intron_variant,genic_upstream_transcript_variant
rs1365670296	intron_variant,genic_upstream_transcript_variant
rs1365704453	intron_variant
rs1365713264	intron_variant,genic_upstream_transcript_variant
rs1365722517	intron_variant
rs1365824239	intron_variant
rs1365842286	intron_variant,genic_upstream_transcript_variant
rs1365852417	intron_variant,genic_upstream_transcript_variant
rs1365862418	intron_variant
rs1365888853	intron_variant,genic_upstream_transcript_variant
rs1365892586	intron_variant
rs1365900739	intron_variant
rs1365903813	intron_variant,genic_downstream_transcript_variant
rs1366013346	intron_variant
rs1366019118	intron_variant,genic_upstream_transcript_variant
rs1366023349	intron_variant,genic_upstream_transcript_variant
rs1366037692	intron_variant,genic_upstream_transcript_variant
rs1366089678	intron_variant,genic_upstream_transcript_variant
rs1366142852	intron_variant,genic_downstream_transcript_variant
rs1366143829	intron_variant,genic_downstream_transcript_variant
rs1366206974	intron_variant,genic_upstream_transcript_variant
rs1366217435	intron_variant
rs1366233668	intron_variant,genic_upstream_transcript_variant
rs1366261191	intron_variant

rs1366271957	intron_variant,genic_upstream_transcript_variant
rs1366299317	intron_variant
rs1366339845	intron_variant,genic_upstream_transcript_variant
rs1366371634	intron_variant
rs1366382102	intron_variant,genic_downstream_transcript_variant
rs1366386319	non_coding_transcript_variant,3_prime_UTR_variant,genic_downstream_transcript_variant
rs1366412643	intron_variant,genic_downstream_transcript_variant
rs1366434391	intron_variant
rs1366439375	intron_variant,upstream_transcript_variant,genic_upstream_transcript_variant
rs1366491336	intron_variant,genic_downstream_transcript_variant
rs1366504099	intron_variant
rs1366532563	intron_variant
rs1366572835	intron_variant,genic_downstream_transcript_variant
rs1366610729	intron_variant
rs1366651413	non_coding_transcript_variant,intron_variant,genic_upstream_transcript_variant
rs1366709635	intron_variant
rs1366758311	intron_variant,genic_downstream_transcript_variant
rs1366772847	intron_variant
rs1366786920	intron_variant,genic_downstream_transcript_variant
rs1366786989	intron_variant,genic_upstream_transcript_variant
rs1366787704	2KB_upstream_variant,intron_variant,upstream_transcript_variant,genic_upstream_transcript_variant
rs1366859625	intron_variant
rs1366869336	intron_variant,genic_upstream_transcript_variant
rs1366869622	intron_variant
rs1366884873	intron_variant
rs1366911287	intron_variant,genic_upstream_transcript_variant
rs1366983833	intron_variant
rs1366989926	intron_variant
rs1367014839	intron_variant
rs1367023592	intron_variant,genic_downstream_transcript_variant
rs1367025713	intron_variant
rs1367057073	intron_variant,genic_upstream_transcript_variant
rs1367146285	intron_variant
rs1367179408	intron_variant,genic_downstream_transcript_variant
rs1367181978	intron_variant,upstream_transcript_variant,genic_upstream_transcript_variant
rs1367193572	intron_variant
rs1367265974	intron_variant,genic_upstream_transcript_variant
rs1367341975	non_coding_transcript_variant,coding_sequence_variant,missense_variant
rs1367355932	intron_variant
rs1367361175	intron_variant,genic_upstream_transcript_variant
rs1367400410	intron_variant,genic_downstream_transcript_variant
rs1367439751	intron_variant
rs1367445940	non_coding_transcript_variant,coding_sequence_variant,genic_downstream_transcript_variant,missense_variant
rs1367476221	intron_variant,upstream_transcript_variant,genic_upstream_transcript_variant
rs1367545990	intron_variant,upstream_transcript_variant,genic_upstream_transcript_variant
rs1367602352	intron_variant
rs1367610633	intron_variant,genic_downstream_transcript_variant
rs1367611089	intron_variant
rs1367732239	intron_variant
rs1367898321	intron_variant,genic_downstream_transcript_variant
rs1367907241	intron_variant,genic_upstream_transcript_variant
rs1367940410	intron_variant
rs1367967180	2KB_upstream_variant,intron_variant,upstream_transcript_variant,genic_upstream_transcript_variant
rs1367973414	intron_variant,genic_downstream_transcript_variant
rs1367989115	intron_variant,genic_upstream_transcript_variant
rs1368074439	intron_variant,genic_upstream_transcript_variant
rs1368081463	intron_variant,genic_upstream_transcript_variant
rs1368125590	intron_variant,genic_downstream_transcript_variant
rs1368132939	intron_variant,upstream_transcript_variant,genic_upstream_transcript_variant
rs1368158199	intron_variant,genic_upstream_transcript_variant
rs1368247515	intron_variant,genic_upstream_transcript_variant
rs1368256648	intron_variant,genic_downstream_transcript_variant
rs1368257141	intron_variant
rs1368314422	intron_variant
rs1368320405	intron_variant,genic_upstream_transcript_variant
rs1368363729	intron_variant,genic_downstream_transcript_variant
rs1368384947	intron_variant
rs1368385665	intron_variant
rs1368400781	non_coding_transcript_variant,3_prime_UTR_variant,genic_downstream_transcript_variant
rs1368427645	intron_variant,genic_upstream_transcript_variant
rs1368457474	intron_variant,genic_upstream_transcript_variant
rs1368457713	intron_variant
rs1368494690	intron_variant,genic_upstream_transcript_variant
rs1368574297	intron_variant

rs1368645027	intron_variant,genic_upstream_transcript_variant
rs1368661115	non_coding_transcript_variant,3_prime_UTR_variant,genic_downstream_transcript_variant
rs1368680418	non_coding_transcript_variant,inframe_deletion,coding_sequence_variant
rs1368690531	intron_variant
rs1368694040	intron_variant,genic_upstream_transcript_variant
rs1368727869	intron_variant,upstream_transcript_variant,genic_upstream_transcript_variant
rs1368751471	intron_variant
rs1368769976	intron_variant
rs1368842975	intron_variant,upstream_transcript_variant,genic_upstream_transcript_variant
rs1368869286	intron_variant,genic_upstream_transcript_variant
rs1368943546	intron_variant
rs1368960165	intron_variant
rs1368962307	intron_variant,5_prime_UTR_variant
rs1368982280	intron_variant,genic_upstream_transcript_variant
rs1368999898	intron_variant,genic_upstream_transcript_variant
rs1369012275	intron_variant
rs1369064578	intron_variant
rs1369103898	intron_variant,genic_upstream_transcript_variant
rs1369128339	intron_variant,genic_upstream_transcript_variant
rs1369143697	intron_variant,genic_upstream_transcript_variant
rs1369148680	intron_variant,genic_upstream_transcript_variant
rs1369255277	intron_variant,genic_downstream_transcript_variant
rs1369283076	intron_variant,genic_downstream_transcript_variant
rs1369291785	intron_variant
rs1369342547	intron_variant,genic_upstream_transcript_variant
rs1369356488	intron_variant,genic_upstream_transcript_variant
rs1369374871	2KB_upstream_variant,intron_variant,upstream_transcript_variant,genic_upstream_transcript_variant
rs1369390754	intron_variant
rs1369400081	intron_variant,genic_upstream_transcript_variant
rs1369409711	intron_variant
rs1369412980	intron_variant
rs1369423040	intron_variant,genic_upstream_transcript_variant
rs1369434773	intron_variant
rs1369443353	intron_variant,genic_upstream_transcript_variant
rs1369469600	intron_variant,genic_upstream_transcript_variant
rs1369508214	intron_variant
rs1369520786	intron_variant
rs1369556062	intron_variant,genic_upstream_transcript_variant
rs1369599475	intron_variant,genic_upstream_transcript_variant
rs1369656655	intron_variant,genic_downstream_transcript_variant
rs1369664635	intron_variant,genic_downstream_transcript_variant
rs1369677018	intron_variant,genic_upstream_transcript_variant
rs1369681283	intron_variant
rs1369682603	intron_variant
rs1369697945	intron_variant,genic_downstream_transcript_variant
rs1369708675	intron_variant
rs1369763148	intron_variant,genic_downstream_transcript_variant
rs1369783203	intron_variant
rs1369815398	intron_variant,genic_downstream_transcript_variant
rs1369847550	intron_variant,genic_upstream_transcript_variant
rs1369868054	intron_variant,genic_upstream_transcript_variant
rs1369880264	intron_variant
rs1369908914	intron_variant
rs1369919389	non_coding_transcript_variant,3_prime_UTR_variant,genic_downstream_transcript_variant
rs1369923464	intron_variant
rs1369933377	intron_variant,genic_upstream_transcript_variant
rs1369943321	intron_variant,genic_upstream_transcript_variant
rs1369967347	intron_variant,upstream_transcript_variant,genic_upstream_transcript_variant
rs1369974663	intron_variant,genic_downstream_transcript_variant
rs1369989994	intron_variant
rs1370003550	intron_variant
rs1370010879	intron_variant,genic_upstream_transcript_variant
rs1370013844	intron_variant,genic_upstream_transcript_variant
rs1370049185	intron_variant
rs1370049783	intron_variant
rs1370053350	intron_variant,genic_upstream_transcript_variant
rs1370069359	intron_variant,genic_upstream_transcript_variant
rs1370085403	intron_variant,genic_upstream_transcript_variant
rs1370114971	intron_variant,genic_upstream_transcript_variant
rs1370119775	intron_variant,genic_upstream_transcript_variant
rs1370194133	intron_variant,genic_downstream_transcript_variant
rs1370216511	intron_variant,genic_upstream_transcript_variant
rs1370236717	intron_variant
rs1370263448	intron_variant,genic_downstream_transcript_variant

rs1370281596	intron_variant
rs1370291128	intron_variant
rs1370293957	intron_variant,genic_upstream_transcript_variant
rs1370311784	intron_variant,genic_upstream_transcript_variant
rs1370379159	intron_variant,genic_downstream_transcript_variant
rs1370406622	intron_variant,genic_downstream_transcript_variant
rs1370425021	intron_variant,genic_upstream_transcript_variant
rs1370427039	intron_variant
rs1370468279	intron_variant,genic_upstream_transcript_variant
rs1370504408	intron_variant
rs1370511341	intron_variant,genic_downstream_transcript_variant
rs1370520372	intron_variant,genic_downstream_transcript_variant
rs1370532745	intron_variant,genic_upstream_transcript_variant
rs1370541728	intron_variant
rs1370551641	intron_variant
rs1370566545	intron_variant
rs1370588027	intron_variant,genic_upstream_transcript_variant
rs1370609151	non_coding_transcript_variant,3_prime_UTR_variant,genic_downstream_transcript_variant
rs1370635970	intron_variant
rs1370646414	intron_variant
rs1370682898	intron_variant
rs1370706402	downstream_transcript_variant,intron_variant,genic_downstream_transcript_variant
rs1370748602	intron_variant,genic_downstream_transcript_variant
rs1370789845	intron_variant,genic_upstream_transcript_variant
rs1370790729	intron_variant,upstream_transcript_variant,genic_upstream_transcript_variant
rs1370807718	intron_variant,genic_downstream_transcript_variant
rs1370834057	intron_variant,genic_upstream_transcript_variant
rs1370840781	intron_variant
rs1370855258	non_coding_transcript_variant,coding_sequence_variant,synonymous_variant,genic_downstream_transcript_variant
rs1370891482	intron_variant,genic_downstream_transcript_variant
rs1370899692	intron_variant
rs1370923210	intron_variant,genic_downstream_transcript_variant
rs1371026058	intron_variant
rs1371090559	intron_variant
rs1371095616	intron_variant
rs1371139971	intron_variant,genic_upstream_transcript_variant
rs1371205146	intron_variant,genic_downstream_transcript_variant
rs1371206531	intron_variant
rs1371207580	intron_variant
rs1371218739	intron_variant,genic_upstream_transcript_variant
rs1371253610	intron_variant,genic_upstream_transcript_variant
rs1371280427	intron_variant
rs1371350150	intron_variant,genic_upstream_transcript_variant
rs1371352384	intron_variant
rs1371381262	intron_variant,genic_upstream_transcript_variant
rs1371390093	intron_variant,genic_upstream_transcript_variant
rs1371411761	intron_variant,genic_upstream_transcript_variant
rs1371450346	intron_variant
rs1371460318	intron_variant,genic_downstream_transcript_variant
rs1371468240	intron_variant,genic_upstream_transcript_variant
rs1371490564	intron_variant,genic_upstream_transcript_variant
rs1371491181	intron_variant
rs1371492877	intron_variant,genic_downstream_transcript_variant
rs1371552809	intron_variant
rs1371557285	intron_variant,genic_upstream_transcript_variant
rs1371585799	intron_variant,genic_upstream_transcript_variant
rs1371628015	intron_variant,genic_upstream_transcript_variant
rs1371648494	intron_variant
rs1371665284	intron_variant,genic_upstream_transcript_variant
rs1371676232	genic_downstream_transcript_variant,intron_variant
rs1371678871	intron_variant,genic_upstream_transcript_variant
rs1371685556	intron_variant
rs1371722654	intron_variant
rs1371729816	upstream_transcript_variant,intron_variant,genic_upstream_transcript_variant
rs1371779101	intron_variant,genic_upstream_transcript_variant
rs1371807236	genic_downstream_transcript_variant,intron_variant
rs1371807876	genic_downstream_transcript_variant,intron_variant
rs1371821200	intron_variant
rs1371851556	intron_variant,genic_upstream_transcript_variant
rs1371861635	intron_variant,genic_upstream_transcript_variant
rs1371876837	intron_variant,genic_upstream_transcript_variant
rs1371920139	upstream_transcript_variant,intron_variant,genic_upstream_transcript_variant
rs1371935134	intron_variant,genic_upstream_transcript_variant
rs1371943770	genic_downstream_transcript_variant,downstream_transcript_variant,intron_variant

rs1371951530	intron_variant,genic_upstream_transcript_variant
rs1371978397	genic_downstream_transcript_variant,intron_variant
rs1371979702	intron_variant
rs1371988996	genic_downstream_transcript_variant,intron_variant
rs1372038331	intron_variant,genic_upstream_transcript_variant
rs1372085194	downstream_transcript_variant,500B_downstream_variant
rs1372126450	intron_variant
rs1372130338	intron_variant,genic_upstream_transcript_variant
rs1372155627	intron_variant
rs1372172783	genic_downstream_transcript_variant,intron_variant
rs1372217838	intron_variant
rs1372219444	intron_variant,genic_upstream_transcript_variant
rs1372228539	intron_variant,genic_upstream_transcript_variant
rs1372250842	intron_variant,genic_upstream_transcript_variant
rs1372289422	genic_downstream_transcript_variant,intron_variant
rs1372346877	intron_variant,genic_upstream_transcript_variant
rs1372414421	intron_variant
rs1372418348	intron_variant,genic_upstream_transcript_variant
rs1372432962	intron_variant,genic_upstream_transcript_variant
rs1372436116	intron_variant
rs1372459548	genic_downstream_transcript_variant,intron_variant
rs1372532963	genic_downstream_transcript_variant,intron_variant
rs1372552457	intron_variant
rs1372559700	genic_downstream_transcript_variant,intron_variant
rs1372615127	genic_downstream_transcript_variant,intron_variant
rs1372696396	missense_variant,coding_sequence_variant,non_coding_transcript_variant
rs1372788928	intron_variant
rs1372910112	intron_variant,genic_upstream_transcript_variant
rs1372951429	intron_variant
rs1372959330	intron_variant
rs1372972001	intron_variant
rs1373024154	intron_variant,genic_upstream_transcript_variant
rs1373034124	intron_variant,genic_upstream_transcript_variant
rs1373042634	2KB_upstream_variant,upstream_transcript_variant,intron_variant,genic_upstream_transcript_variant
rs1373068448	intron_variant
rs1373087651	genic_downstream_transcript_variant,intron_variant
rs1373108302	2KB_upstream_variant,upstream_transcript_variant,intron_variant,genic_upstream_transcript_variant
rs1373190244	2KB_upstream_variant,upstream_transcript_variant,intron_variant,genic_upstream_transcript_variant
rs1373257360	intron_variant,genic_upstream_transcript_variant
rs1373269653	genic_downstream_transcript_variant,intron_variant
rs1373280142	genic_downstream_transcript_variant,intron_variant
rs1373306317	intron_variant,genic_upstream_transcript_variant
rs1373348674	intron_variant
rs1373351272	intron_variant
rs1373363424	genic_downstream_transcript_variant,intron_variant
rs1373369191	intron_variant,genic_upstream_transcript_variant
rs1373404177	intron_variant
rs1373410581	intron_variant,genic_upstream_transcript_variant
rs1373477924	intron_variant
rs1373483244	intron_variant,genic_upstream_transcript_variant
rs1373500417	genic_downstream_transcript_variant,intron_variant
rs1373517938	intron_variant
rs1373518888	intron_variant,genic_upstream_transcript_variant
rs1373570802	intron_variant
rs1373572705	intron_variant,genic_upstream_transcript_variant
rs1373597317	intron_variant
rs1373617852	intron_variant,genic_upstream_transcript_variant
rs1373708712	intron_variant
rs1373744613	genic_downstream_transcript_variant,intron_variant
rs1373756273	intron_variant,genic_upstream_transcript_variant
rs1373890647	intron_variant,genic_upstream_transcript_variant
rs1373906478	intron_variant,genic_upstream_transcript_variant
rs1373932887	genic_downstream_transcript_variant,intron_variant
rs1373992313	intron_variant,genic_upstream_transcript_variant
rs1374003952	intron_variant,genic_upstream_transcript_variant
rs1374030949	intron_variant
rs1374048736	intron_variant,genic_upstream_transcript_variant
rs1374071771	genic_downstream_transcript_variant,intron_variant
rs1374098076	intron_variant
rs1374151019	upstream_transcript_variant,intron_variant,genic_upstream_transcript_variant
rs1374214693	intron_variant,genic_upstream_transcript_variant
rs1374222095	intron_variant,genic_upstream_transcript_variant
rs1374232333	intron_variant,genic_upstream_transcript_variant
rs1374252907	intron_variant

rs1374261878	intron_variant,genic_upstream_transcript_variant
rs1374264056	genic_downstream_transcript_variant,intron_variant
rs1374266810	intron_variant
rs1374285814	intron_variant,genic_upstream_transcript_variant
rs1374289416	intron_variant,genic_upstream_transcript_variant
rs1374300579	genic_downstream_transcript_variant,intron_variant
rs1374388546	intron_variant,genic_upstream_transcript_variant
rs1374398138	intron_variant
rs1374413099	synonymous_variant,coding_sequence_variant,non_coding_transcript_variant
rs1374417266	2KB_upstream_variant,upstream_transcript_variant,intron_variant,genic_upstream_transcript_variant
rs1374448254	intron_variant
rs1374480042	intron_variant
rs1374486100	genic_downstream_transcript_variant,intron_variant
rs1374515462	genic_downstream_transcript_variant,intron_variant
rs1374557011	intron_variant
rs1374578858	intron_variant
rs1374587949	intron_variant,genic_upstream_transcript_variant
rs1374608036	intron_variant,genic_upstream_transcript_variant
rs1374612216	intron_variant
rs1374647966	intron_variant,genic_upstream_transcript_variant
rs1374650199	intron_variant,genic_upstream_transcript_variant
rs1374724128	missense_variant,coding_sequence_variant,non_coding_transcript_variant
rs1374731264	intron_variant,genic_upstream_transcript_variant
rs1374760604	genic_downstream_transcript_variant,intron_variant
rs1374794377	intron_variant,genic_upstream_transcript_variant
rs1374864020	intron_variant,genic_upstream_transcript_variant
rs1374906726	intron_variant,genic_upstream_transcript_variant
rs1374942869	2KB_upstream_variant,upstream_transcript_variant,intron_variant,genic_upstream_transcript_variant
rs1374949048	intron_variant
rs1374980331	intron_variant
rs1374984200	intron_variant
rs1375005774	intron_variant,genic_upstream_transcript_variant
rs1375020631	intron_variant,genic_upstream_transcript_variant
rs1375021820	intron_variant,genic_upstream_transcript_variant
rs1375036015	2KB_upstream_variant,upstream_transcript_variant,intron_variant,genic_upstream_transcript_variant
rs1375077277	intron_variant,genic_upstream_transcript_variant
rs1375094044	intron_variant
rs1375097448	intron_variant
rs1375104668	intron_variant,genic_upstream_transcript_variant
rs1375143791	intron_variant,genic_upstream_transcript_variant
rs1375151564	upstream_transcript_variant,5_prime_UTR_variant,intron_variant,genic_upstream_transcript_variant
rs1375243367	intron_variant,genic_upstream_transcript_variant
rs1375245061	intron_variant,genic_upstream_transcript_variant
rs1375263433	intron_variant
rs1375285399	intron_variant
rs1375312481	intron_variant
rs1375321543	intron_variant
rs1375324383	intron_variant,genic_upstream_transcript_variant
rs1375324943	genic_downstream_transcript_variant,intron_variant
rs1375326249	genic_downstream_transcript_variant,intron_variant
rs1375386318	intron_variant
rs1375410603	intron_variant,genic_upstream_transcript_variant
rs1375414969	genic_downstream_transcript_variant,intron_variant
rs1375431225	genic_downstream_transcript_variant,intron_variant
rs1375447901	intron_variant
rs1375470594	intron_variant,genic_upstream_transcript_variant
rs1375517431	intron_variant
rs1375546924	intron_variant,genic_upstream_transcript_variant
rs1375557409	intron_variant
rs1375598587	intron_variant
rs1375601004	genic_downstream_transcript_variant,intron_variant
rs1375626272	2KB_upstream_variant,upstream_transcript_variant,intron_variant,genic_upstream_transcript_variant
rs1375630353	intron_variant
rs1375697888	genic_downstream_transcript_variant,intron_variant
rs1375756677	genic_downstream_transcript_variant,intron_variant
rs1375779234	2KB_upstream_variant,upstream_transcript_variant,intron_variant,genic_upstream_transcript_variant
rs1375784135	intron_variant
rs1375877141	intron_variant,genic_upstream_transcript_variant
rs1375898640	intron_variant
rs1375953145	intron_variant
rs1376082003	intron_variant,genic_upstream_transcript_variant
rs1376106698	intron_variant
rs1376154338	2KB_upstream_variant,upstream_transcript_variant,intron_variant,genic_upstream_transcript_variant
rs1376167889	genic_downstream_transcript_variant,intron_variant

rs1376175981	intron_variant,genic_upstream_transcript_variant
rs1376177102	intron_variant
rs1376194619	genic_downstream_transcript_variant,intron_variant
rs1376206586	intron_variant
rs1376215900	2KB_upstream_variant,upstream_transcript_variant,intron_variant,genic_upstream_transcript_variant
rs1376220905	intron_variant
rs1376270659	intron_variant,genic_upstream_transcript_variant
rs1376295165	intron_variant,genic_upstream_transcript_variant
rs1376309242	intron_variant,genic_upstream_transcript_variant
rs1376356507	intron_variant,genic_upstream_transcript_variant
rs1376390374	upstream_transcript_variant,intron_variant,genic_upstream_transcript_variant
rs1376499542	genic_downstream_transcript_variant,intron_variant
rs1376504059	intron_variant,genic_upstream_transcript_variant
rs1376517293	intron_variant,genic_upstream_transcript_variant
rs1376518172	intron_variant
rs1376606159	2KB_upstream_variant,upstream_transcript_variant,intron_variant,genic_upstream_transcript_variant
rs1376607159	intron_variant
rs1376608086	intron_variant,genic_upstream_transcript_variant
rs1376647769	intron_variant
rs1376666962	intron_variant
rs1376678961	intron_variant,genic_upstream_transcript_variant
rs1376687119	intron_variant,genic_upstream_transcript_variant
rs1376691488	intron_variant
rs1376726227	intron_variant,genic_upstream_transcript_variant
rs1376782616	intron_variant
rs1376824178	intron_variant,genic_upstream_transcript_variant
rs1376879216	intron_variant
rs1376885201	intron_variant,genic_upstream_transcript_variant
rs1376897894	intron_variant,genic_upstream_transcript_variant
rs1376904686	intron_variant
rs1376924625	intron_variant,genic_upstream_transcript_variant
rs1376966388	intron_variant
rs1377039968	genic_downstream_transcript_variant,intron_variant
rs1377043772	genic_downstream_transcript_variant,intron_variant
rs1377051452	intron_variant,genic_upstream_transcript_variant
rs1377051775	genic_downstream_transcript_variant,intron_variant
rs1377099824	genic_downstream_transcript_variant,intron_variant
rs1377190110	intron_variant
rs1377214126	genic_downstream_transcript_variant,intron_variant
rs1377225979	intron_variant
rs1377317045	intron_variant,genic_upstream_transcript_variant
rs1377333532	intron_variant,genic_upstream_transcript_variant
rs1377340739	intron_variant,genic_upstream_transcript_variant
rs1377357363	intron_variant
rs1377373568	genic_downstream_transcript_variant,intron_variant
rs1377385831	2KB_upstream_variant,upstream_transcript_variant,intron_variant,genic_upstream_transcript_variant
rs1377400281	intron_variant,genic_upstream_transcript_variant
rs1377449058	intron_variant
rs1377472821	intron_variant,genic_upstream_transcript_variant
rs1377507739	2KB_upstream_variant,upstream_transcript_variant,intron_variant,genic_upstream_transcript_variant
rs1377556179	intron_variant,genic_upstream_transcript_variant
rs1377559420	intron_variant
rs1377575152	intron_variant,genic_upstream_transcript_variant
rs1377629643	intron_variant,genic_upstream_transcript_variant
rs1377675540	intron_variant,genic_upstream_transcript_variant
rs1377691467	intron_variant
rs1377697417	genic_downstream_transcript_variant,intron_variant
rs1377735815	genic_downstream_transcript_variant,intron_variant
rs1377741501	upstream_transcript_variant,intron_variant,genic_upstream_transcript_variant
rs1377751294	genic_downstream_transcript_variant,intron_variant
rs1377758118	coding_sequence_variant,genic_upstream_transcript_variant,missense_variant,non_coding_transcript_variant,intron_variant
rs1377784179	intron_variant,genic_upstream_transcript_variant
rs1377800110	intron_variant
rs1377804283	genic_downstream_transcript_variant,intron_variant
rs1377823554	intron_variant,genic_upstream_transcript_variant
rs1377872978	genic_downstream_transcript_variant,intron_variant
rs1377935711	intron_variant
rs1377953941	genic_downstream_transcript_variant,intron_variant
rs1378002025	intron_variant,genic_upstream_transcript_variant
rs1378011207	intron_variant
rs1378031908	intron_variant,genic_upstream_transcript_variant
rs1378061865	genic_downstream_transcript_variant,intron_variant
rs1378120910	intron_variant,genic_upstream_transcript_variant
rs1378129046	intron_variant

rs1378132900	intron_variant
rs1378149050	intron_variant,genic_upstream_transcript_variant
rs1378154283	intron_variant,genic_upstream_transcript_variant
rs1378190821	genic_downstream_transcript_variant,intron_variant
rs1378200304	intron_variant
rs1378229618	intron_variant,genic_upstream_transcript_variant
rs1378251166	intron_variant
rs1378326043	intron_variant,genic_upstream_transcript_variant
rs1378330960	intron_variant
rs1378359211	intron_variant
rs1378368059	intron_variant,genic_upstream_transcript_variant
rs1378396865	intron_variant
rs1378402747	intron_variant,genic_upstream_transcript_variant
rs1378415815	intron_variant,genic_upstream_transcript_variant
rs1378418693	intron_variant
rs1378419134	intron_variant,genic_upstream_transcript_variant
rs1378433521	intron_variant
rs1378435496	intron_variant,genic_upstream_transcript_variant
rs1378454960	genic_downstream_transcript_variant,intron_variant
rs1378490506	intron_variant
rs1378520496	intron_variant,genic_upstream_transcript_variant
rs1378599992	intron_variant
rs1378603779	upstream_transcript_variant,intron_variant,genic_upstream_transcript_variant
rs1378626919	intron_variant,genic_upstream_transcript_variant
rs1378627162	intron_variant,genic_upstream_transcript_variant
rs1378660561	intron_variant
rs1378666021	intron_variant
rs1378666843	upstream_transcript_variant,intron_variant,genic_upstream_transcript_variant
rs1378713605	intron_variant,genic_upstream_transcript_variant
rs1378721171	intron_variant,genic_upstream_transcript_variant
rs1378740898	intron_variant
rs1378754922	intron_variant,genic_upstream_transcript_variant
rs1378759032	genic_downstream_transcript_variant,intron_variant
rs1378764768	2KB_upstream_variant,upstream_transcript_variant,intron_variant,genic_upstream_transcript_variant
rs1378772766	intron_variant
rs1378806303	intron_variant,genic_upstream_transcript_variant
rs1378865173	genic_downstream_transcript_variant,intron_variant
rs1378925174	intron_variant
rs1378947517	intron_variant,genic_upstream_transcript_variant
rs1378963674	genic_downstream_transcript_variant,intron_variant
rs1378996595	intron_variant
rs1379007709	intron_variant,genic_upstream_transcript_variant
rs1379012001	intron_variant
rs1379048138	genic_downstream_transcript_variant,intron_variant
rs1379078529	genic_downstream_transcript_variant,missense_variant,coding_sequence_variant,non_coding_transcript_variant
rs1379099372	intron_variant
rs1379133384	intron_variant
rs1379146765	genic_downstream_transcript_variant,intron_variant
rs1379200579	genic_downstream_transcript_variant,intron_variant
rs1379218158	genic_downstream_transcript_variant,intron_variant
rs1379245003	upstream_transcript_variant,intron_variant,genic_upstream_transcript_variant
rs1379288765	intron_variant
rs1379319941	intron_variant,genic_upstream_transcript_variant
rs1379320051	intron_variant
rs1379374195	genic_downstream_transcript_variant,intron_variant
rs1379386057	downstream_transcript_variant,500B_downstream_variant
rs1379443877	genic_downstream_transcript_variant,intron_variant
rs1379462485	genic_downstream_transcript_variant,intron_variant
rs1379476494	intron_variant,genic_upstream_transcript_variant
rs1379482761	genic_downstream_transcript_variant,3_prime_UTR_variant,non_coding_transcript_variant
rs1379507846	genic_downstream_transcript_variant,intron_variant
rs1379517827	intron_variant
rs1379540373	intron_variant,genic_upstream_transcript_variant
rs1379574810	genic_downstream_transcript_variant,intron_variant
rs1379623853	genic_downstream_transcript_variant,intron_variant
rs1379633658	intron_variant
rs1379654139	intron_variant,genic_upstream_transcript_variant
rs1379669221	intron_variant,genic_upstream_transcript_variant
rs1379723005	intron_variant,genic_upstream_transcript_variant
rs1379752268	intron_variant
rs1379815125	intron_variant,genic_upstream_transcript_variant
rs1379818206	genic_downstream_transcript_variant,intron_variant
rs1379856977	intron_variant
rs1379882081	genic_downstream_transcript_variant,intron_variant

rs1379886233	intron_variant
rs1379917310	intron_variant,genic_upstream_transcript_variant
rs1379934608	intron_variant
rs1379949689	intron_variant
rs1379950354	intron_variant
rs1380012385	intron_variant
rs1380019258	intron_variant
rs1380064475	genic_downstream_transcript_variant,intron_variant
rs1380097645	intron_variant
rs1380141941	intron_variant,genic_upstream_transcript_variant
rs1380155580	intron_variant
rs1380201461	genic_downstream_transcript_variant,intron_variant
rs1380218442	intron_variant,genic_upstream_transcript_variant
rs1380220737	intron_variant,genic_upstream_transcript_variant
rs1380257341	intron_variant
rs1380300273	intron_variant
rs1380329809	genic_downstream_transcript_variant,intron_variant
rs1380341309	genic_downstream_transcript_variant,intron_variant
rs1380406180	intron_variant
rs1380456478	genic_downstream_transcript_variant,intron_variant
rs1380465485	genic_downstream_transcript_variant,intron_variant
rs1380501001	intron_variant
rs1380521515	intron_variant
rs1380568693	genic_downstream_transcript_variant,intron_variant
rs1380593829	intron_variant,genic_upstream_transcript_variant
rs1380636538	intron_variant,genic_upstream_transcript_variant
rs1380639845	intron_variant
rs1380643241	genic_downstream_transcript_variant,intron_variant
rs1380660880	intron_variant,genic_upstream_transcript_variant
rs1380686960	intron_variant
rs1380704259	intron_variant
rs1380716538	genic_downstream_transcript_variant,intron_variant
rs1380739073	intron_variant
rs1380753371	intron_variant
rs1380786322	upstream_transcript_variant,intron_variant,genic_upstream_transcript_variant
rs1380799661	intron_variant,genic_upstream_transcript_variant
rs1380809268	intron_variant
rs1380855156	genic_downstream_transcript_variant,intron_variant
rs1380889320	intron_variant,genic_upstream_transcript_variant
rs1380896518	upstream_transcript_variant,intron_variant,genic_upstream_transcript_variant
rs1380897374	intron_variant,genic_upstream_transcript_variant
rs1380913802	intron_variant,genic_upstream_transcript_variant
rs1380934533	intron_variant,genic_upstream_transcript_variant
rs1380981363	intron_variant
rs1381009467	intron_variant
rs1381048424	intron_variant,genic_upstream_transcript_variant
rs1381062235	intron_variant,genic_upstream_transcript_variant
rs1381092078	upstream_transcript_variant,intron_variant,genic_upstream_transcript_variant
rs1381118238	genic_downstream_transcript_variant,intron_variant
rs1381129384	intron_variant,genic_upstream_transcript_variant
rs1381157659	2KB_upstream_variant,upstream_transcript_variant,intron_variant,genic_upstream_transcript_variant
rs1381178160	intron_variant
rs1381185879	intron_variant
rs1381225360	intron_variant,genic_upstream_transcript_variant
rs1381298662	intron_variant
rs1381412567	intron_variant
rs1381420521	genic_downstream_transcript_variant,intron_variant
rs1381450027	intron_variant,genic_upstream_transcript_variant
rs1381473114	intron_variant,genic_upstream_transcript_variant
rs1381495695	intron_variant
rs1381523825	intron_variant,genic_upstream_transcript_variant
rs1381548414	intron_variant
rs1381554784	coding_sequence_variant,genic_upstream_transcript_variant,missense_variant,non_coding_transcript_variant,intron_variant
rs1381571819	intron_variant
rs1381622779	intron_variant
rs1381623174	intron_variant
rs1381649287	intron_variant,genic_upstream_transcript_variant
rs1381660941	intron_variant
rs1381694848	synonymous_variant,coding_sequence_variant,non_coding_transcript_variant
rs1381725732	genic_downstream_transcript_variant,intron_variant
rs1381743748	upstream_transcript_variant,5_prime_UTR_variant,intron_variant,genic_upstream_transcript_variant
rs1381748037	intron_variant
rs1381789805	intron_variant,genic_upstream_transcript_variant
rs1381813156	intron_variant,genic_upstream_transcript_variant

rs1381822111	upstream_transcript_variant,intron_variant,genic_upstream_transcript_variant
rs1381833846	intron_variant
rs1381834234	upstream_transcript_variant,intron_variant,genic_upstream_transcript_variant
rs1381847410	intron_variant,genic_upstream_transcript_variant
rs1381852348	intron_variant,genic_upstream_transcript_variant
rs1381894921	2KB_upstream_variant,upstream_transcript_variant,intron_variant,genic_upstream_transcript_variant
rs1381914996	intron_variant,genic_upstream_transcript_variant
rs1381946914	intron_variant
rs1381975427	intron_variant
rs1382005025	genic_downstream_transcript_variant,intron_variant
rs1382062652	intron_variant,genic_upstream_transcript_variant
rs1382088629	intron_variant,genic_upstream_transcript_variant
rs1382141349	intron_variant,genic_upstream_transcript_variant
rs1382156424	intron_variant,genic_upstream_transcript_variant
rs1382199100	intron_variant,genic_upstream_transcript_variant
rs1382212168	genic_downstream_transcript_variant,intron_variant
rs1382245126	intron_variant,genic_upstream_transcript_variant
rs1382263718	intron_variant
rs1382267511	intron_variant,genic_upstream_transcript_variant
rs1382275871	intron_variant
rs1382293477	intron_variant,genic_upstream_transcript_variant
rs1382340136	intron_variant
rs1382340697	intron_variant,genic_upstream_transcript_variant
rs1382342598	intron_variant
rs1382402201	genic_downstream_transcript_variant,intron_variant
rs1382404525	intron_variant,genic_upstream_transcript_variant
rs1382422522	genic_downstream_transcript_variant,intron_variant
rs1382426549	intron_variant,genic_upstream_transcript_variant
rs1382440266	intron_variant,genic_upstream_transcript_variant
rs1382441321	intron_variant,genic_upstream_transcript_variant
rs1382443390	intron_variant,genic_upstream_transcript_variant
rs1382446348	genic_downstream_transcript_variant,intron_variant
rs1382531041	2KB_upstream_variant,upstream_transcript_variant,intron_variant,genic_upstream_transcript_variant
rs1382540447	intron_variant
rs1382567133	downstream_transcript_variant,500B_downstream_variant
rs1382579634	intron_variant
rs1382583306	genic_downstream_transcript_variant,intron_variant
rs1382608429	genic_downstream_transcript_variant,intron_variant
rs1382635259	intron_variant,genic_upstream_transcript_variant
rs1382664164	intron_variant,genic_upstream_transcript_variant
rs1382669338	genic_downstream_transcript_variant,intron_variant
rs1382674774	intron_variant,genic_upstream_transcript_variant
rs1382681519	intron_variant,genic_upstream_transcript_variant
rs1382714094	genic_downstream_transcript_variant,intron_variant
rs1382752445	upstream_transcript_variant,intron_variant,genic_upstream_transcript_variant
rs1382772997	upstream_transcript_variant,intron_variant,genic_upstream_transcript_variant
rs1382787042	genic_downstream_transcript_variant,downstream_transcript_variant,intron_variant
rs1382811393	intron_variant
rs1382821546	genic_downstream_transcript_variant,intron_variant
rs1382844845	intron_variant,genic_upstream_transcript_variant
rs1382871882	intron_variant,genic_upstream_transcript_variant
rs1382903588	intron_variant,genic_upstream_transcript_variant
rs1382905617	intron_variant
rs1382910566	intron_variant,genic_upstream_transcript_variant
rs1382957259	intron_variant,genic_upstream_transcript_variant
rs1382983235	intron_variant,genic_upstream_transcript_variant
rs1382983595	genic_downstream_transcript_variant,3_prime_UTR_variant,non_coding_transcript_variant
rs1382993558	genic_downstream_transcript_variant,intron_variant
rs1383010384	intron_variant
rs1383011276	intron_variant
rs1383012850	intron_variant
rs1383013952	2KB_upstream_variant,upstream_transcript_variant,intron_variant,genic_upstream_transcript_variant
rs1383030592	2KB_upstream_variant,upstream_transcript_variant,intron_variant,genic_upstream_transcript_variant
rs1383044067	intron_variant,genic_upstream_transcript_variant
rs1383104743	intron_variant
rs1383115382	intron_variant,genic_upstream_transcript_variant
rs1383138195	intron_variant,genic_upstream_transcript_variant
rs1383141686	intron_variant,genic_upstream_transcript_variant
rs1383178470	intron_variant
rs1383184292	intron_variant
rs1383207917	intron_variant
rs1383230541	intron_variant
rs1383256668	intron_variant
rs1383276866	intron_variant

rs1383295696	intron_variant
rs1383331755	intron_variant,genic_upstream_transcript_variant
rs1383374613	intron_variant
rs1383383155	intron_variant,genic_upstream_transcript_variant
rs1383386383	intron_variant,genic_upstream_transcript_variant
rs1383389845	genic_downstream_transcript_variant,intron_variant
rs1383431312	intron_variant
rs1383446554	intron_variant,genic_upstream_transcript_variant
rs1383476358	genic_downstream_transcript_variant,intron_variant
rs1383516843	intron_variant,genic_upstream_transcript_variant
rs1383543664	intron_variant
rs1383563784	intron_variant
rs1383568140	genic_downstream_transcript_variant,intron_variant
rs1383601767	intron_variant,genic_upstream_transcript_variant
rs1383610420	intron_variant
rs1383621711	intron_variant,genic_upstream_transcript_variant
rs1383683727	intron_variant
rs1383724768	intron_variant
rs1383755312	genic_downstream_transcript_variant,intron_variant
rs1383828081	intron_variant,genic_upstream_transcript_variant
rs1383836032	genic_downstream_transcript_variant,intron_variant
rs1383867175	intron_variant,genic_upstream_transcript_variant
rs1383892805	2KB_upstream_variant,upstream_transcript_variant,intron_variant,genic_upstream_transcript_variant
rs1383934902	intron_variant,genic_upstream_transcript_variant
rs1383968940	genic_downstream_transcript_variant,intron_variant
rs1384009356	genic_downstream_transcript_variant,intron_variant
rs1384095355	genic_downstream_transcript_variant,intron_variant
rs1384111159	intron_variant,genic_upstream_transcript_variant
rs1384137257	intron_variant
rs1384170447	intron_variant,genic_upstream_transcript_variant
rs1384271703	intron_variant,genic_downstream_transcript_variant
rs1384325687	intron_variant,genic_upstream_transcript_variant
rs1384329313	intron_variant
rs1384345609	intron_variant,genic_downstream_transcript_variant
rs1384366777	intron_variant
rs1384409373	intron_variant,genic_upstream_transcript_variant
rs1384428538	intron_variant
rs1384454859	intron_variant,genic_downstream_transcript_variant
rs1384463304	intron_variant,genic_downstream_transcript_variant
rs1384500331	intron_variant
rs1384544481	intron_variant,genic_upstream_transcript_variant
rs1384565004	intron_variant
rs1384577017	intron_variant
rs1384662132	intron_variant,genic_upstream_transcript_variant
rs1384719734	intron_variant,genic_downstream_transcript_variant
rs1384745840	intron_variant,genic_upstream_transcript_variant
rs1384803382	intron_variant,genic_upstream_transcript_variant,upstream_transcript_variant
rs1384804925	genic_downstream_transcript_variant,3_prime_UTR_variant,non_coding_transcript_variant
rs1384826178	intron_variant
rs1384884873	intron_variant
rs1384923387	intron_variant
rs1385026432	intron_variant
rs1385030644	intron_variant,genic_downstream_transcript_variant
rs1385064216	intron_variant,genic_upstream_transcript_variant
rs1385105421	intron_variant,genic_upstream_transcript_variant
rs1385167713	intron_variant,genic_downstream_transcript_variant
rs1385182317	downstream_transcript_variant,intron_variant,genic_downstream_transcript_variant
rs1385193389	intron_variant,genic_upstream_transcript_variant
rs1385220808	intron_variant
rs1385232647	intron_variant,genic_upstream_transcript_variant
rs1385263789	intron_variant,genic_upstream_transcript_variant
rs1385266262	intron_variant,genic_downstream_transcript_variant
rs1385270673	intron_variant
rs1385275180	intron_variant
rs1385295918	intron_variant
rs1385303140	intron_variant
rs1385324980	genic_upstream_transcript_variant,intron_variant,5_prime_UTR_variant,upstream_transcript_variant
rs1385371022	upstream_transcript_variant,intron_variant,genic_upstream_transcript_variant,2KB_upstream_variant
rs1385417926	upstream_transcript_variant,intron_variant,genic_upstream_transcript_variant,2KB_upstream_variant
rs1385429940	intron_variant,genic_upstream_transcript_variant
rs1385437846	intron_variant,genic_upstream_transcript_variant
rs1385533983	intron_variant,genic_downstream_transcript_variant
rs1385567719	upstream_transcript_variant,intron_variant,genic_upstream_transcript_variant,2KB_upstream_variant
rs1385570605	intron_variant,genic_downstream_transcript_variant

rs1385616331	intron_variant,genic_upstream_transcript_variant
rs1385626914	intron_variant,genic_upstream_transcript_variant
rs1385628689	intron_variant,genic_upstream_transcript_variant
rs1385635394	intron_variant
rs1385696742	intron_variant,genic_downstream_transcript_variant
rs1385718256	intron_variant
rs1385783526	intron_variant,genic_downstream_transcript_variant
rs1385834236	intron_variant
rs1385866035	coding_sequence_variant,frameshift_variant,non_coding_transcript_variant
rs1385889225	intron_variant,genic_upstream_transcript_variant
rs1385939015	intron_variant,genic_upstream_transcript_variant
rs1385958001	intron_variant,genic_upstream_transcript_variant,upstream_transcript_variant
rs1385990783	intron_variant,genic_downstream_transcript_variant
rs1386004773	intron_variant
rs1386015604	intron_variant,genic_upstream_transcript_variant
rs1386072717	intron_variant,genic_downstream_transcript_variant
rs1386082247	intron_variant
rs1386101687	intron_variant,genic_downstream_transcript_variant
rs1386101878	intron_variant
rs1386153686	intron_variant
rs1386162623	intron_variant,genic_upstream_transcript_variant
rs1386215990	intron_variant,genic_upstream_transcript_variant
rs1386225386	intron_variant,genic_downstream_transcript_variant
rs1386232755	intron_variant,genic_downstream_transcript_variant
rs1386245037	intron_variant,genic_upstream_transcript_variant,upstream_transcript_variant
rs1386257299	intron_variant,genic_downstream_transcript_variant
rs1386260763	intron_variant
rs1386295688	intron_variant,genic_upstream_transcript_variant
rs1386307606	intron_variant,genic_upstream_transcript_variant
rs1386339445	intron_variant,genic_upstream_transcript_variant
rs1386343651	intron_variant
rs1386355015	intron_variant,genic_downstream_transcript_variant
rs1386387759	intron_variant
rs1386402335	intron_variant,genic_upstream_transcript_variant
rs1386418718	intron_variant,genic_downstream_transcript_variant
rs1386434810	genic_downstream_transcript_variant,3_prime_UTR_variant,non_coding_transcript_variant
rs1386475587	intron_variant
rs1386489358	intron_variant,genic_upstream_transcript_variant
rs1386493397	intron_variant
rs1386496023	intron_variant,genic_upstream_transcript_variant
rs1386508426	intron_variant
rs1386553574	intron_variant
rs1386556933	intron_variant,genic_upstream_transcript_variant
rs1386580690	coding_sequence_variant,missense_variant,non_coding_transcript_variant
rs1386591021	intron_variant
rs1386637354	intron_variant,genic_downstream_transcript_variant
rs1386650557	downstream_transcript_variant,intron_variant,genic_downstream_transcript_variant
rs1386718651	intron_variant,genic_upstream_transcript_variant
rs1386756691	intron_variant,genic_downstream_transcript_variant
rs1386758364	intron_variant,genic_upstream_transcript_variant
rs1386763508	intron_variant,genic_downstream_transcript_variant
rs1386763556	intron_variant
rs1386845287	intron_variant,genic_upstream_transcript_variant
rs1386853491	intron_variant,genic_downstream_transcript_variant
rs1386875834	intron_variant,genic_upstream_transcript_variant
rs1386894508	intron_variant,genic_downstream_transcript_variant
rs1386941951	intron_variant
rs1386954566	intron_variant,genic_upstream_transcript_variant
rs1386998509	intron_variant
rs1387008126	intron_variant,genic_upstream_transcript_variant
rs1387014911	genic_downstream_transcript_variant,3_prime_UTR_variant,non_coding_transcript_variant
rs1387043446	intron_variant
rs1387050963	intron_variant
rs1387051194	intron_variant,genic_downstream_transcript_variant
rs1387065415	intron_variant,missense_variant,genic_upstream_transcript_variant,coding_sequence_variant,non_coding_transcript_variant
rs1387070309	intron_variant
rs1387138694	intron_variant
rs1387162764	intron_variant,genic_upstream_transcript_variant
rs1387168803	intron_variant,genic_upstream_transcript_variant
rs1387243275	intron_variant,genic_upstream_transcript_variant
rs1387267958	intron_variant,genic_upstream_transcript_variant
rs1387337481	intron_variant,genic_upstream_transcript_variant,upstream_transcript_variant
rs1387337919	intron_variant,genic_upstream_transcript_variant
rs1387354897	intron_variant

rs1387443154	intron_variant
rs1387497913	intron_variant
rs1387513545	intron_variant,genic_upstream_transcript_variant
rs1387531681	intron_variant,genic_downstream_transcript_variant
rs1387543965	intron_variant,genic_downstream_transcript_variant
rs1387564266	intron_variant,genic_upstream_transcript_variant
rs1387573950	intron_variant,genic_downstream_transcript_variant
rs1387610193	intron_variant
rs1387663766	intron_variant,genic_downstream_transcript_variant
rs1387731685	intron_variant,genic_upstream_transcript_variant
rs1387739761	intron_variant,genic_upstream_transcript_variant
rs1387742918	intron_variant
rs1387766820	intron_variant
rs1387785357	intron_variant,genic_upstream_transcript_variant
rs1387788005	intron_variant
rs1387855378	intron_variant,genic_upstream_transcript_variant
rs1387856163	intron_variant,genic_upstream_transcript_variant
rs1387905198	intron_variant
rs1387983077	intron_variant,genic_upstream_transcript_variant
rs1387985036	intron_variant,genic_downstream_transcript_variant
rs1388012901	intron_variant
rs1388021068	intron_variant,genic_downstream_transcript_variant
rs1388036369	intron_variant,genic_upstream_transcript_variant
rs1388106244	intron_variant
rs1388129177	intron_variant,genic_downstream_transcript_variant
rs1388150627	intron_variant,genic_upstream_transcript_variant
rs1388200051	intron_variant,genic_upstream_transcript_variant
rs1388267642	intron_variant
rs1388293391	intron_variant,genic_downstream_transcript_variant
rs1388333532	intron_variant
rs1388342618	intron_variant,genic_upstream_transcript_variant
rs1388374044	intron_variant,genic_upstream_transcript_variant
rs1388386566	intron_variant,genic_upstream_transcript_variant
rs1388391125	intron_variant,genic_downstream_transcript_variant
rs1388419803	intron_variant,genic_downstream_transcript_variant
rs1388437607	intron_variant,genic_downstream_transcript_variant
rs1388450525	intron_variant,genic_upstream_transcript_variant
rs1388482012	intron_variant,genic_upstream_transcript_variant
rs1388487349	intron_variant,genic_upstream_transcript_variant,upstream_transcript_variant
rs1388495072	intron_variant,genic_upstream_transcript_variant
rs1388505904	intron_variant,genic_upstream_transcript_variant,upstream_transcript_variant
rs1388545214	intron_variant,genic_upstream_transcript_variant
rs1388552126	intron_variant
rs1388559658	intron_variant
rs1388579075	intron_variant,genic_upstream_transcript_variant
rs1388611075	intron_variant
rs1388616839	intron_variant
rs1388652724	intron_variant,genic_downstream_transcript_variant
rs1388656041	intron_variant,genic_upstream_transcript_variant
rs1388680871	intron_variant,5_prime_UTR_variant
rs1388703641	intron_variant,genic_downstream_transcript_variant
rs1388744855	intron_variant,genic_upstream_transcript_variant
rs1388750913	intron_variant,genic_upstream_transcript_variant
rs1388756033	intron_variant
rs1388762352	intron_variant,genic_upstream_transcript_variant
rs1388815449	intron_variant
rs1388841010	intron_variant,genic_upstream_transcript_variant
rs1388926847	intron_variant
rs1388947864	intron_variant,genic_upstream_transcript_variant
rs1388968418	intron_variant
rs1388977265	intron_variant,genic_upstream_transcript_variant
rs1389010919	intron_variant,genic_downstream_transcript_variant
rs1389038590	intron_variant
rs1389042686	intron_variant,genic_upstream_transcript_variant
rs1389050816	intron_variant,genic_upstream_transcript_variant
rs1389128699	intron_variant,genic_downstream_transcript_variant
rs1389142138	intron_variant,genic_upstream_transcript_variant
rs1389151480	intron_variant
rs1389168534	intron_variant,genic_downstream_transcript_variant
rs1389168849	intron_variant,genic_upstream_transcript_variant,stop_gained,coding_sequence_variant,non_coding_transcript_variant
rs1389172325	intron_variant
rs1389235979	intron_variant
rs1389241703	intron_variant
rs1389408592	intron_variant

rs1389434885	intron_variant,genic_upstream_transcript_variant
rs1389455158	intron_variant
rs1389520279	intron_variant
rs1389527381	intron_variant,genic_downstream_transcript_variant
rs1389557371	intron_variant,genic_upstream_transcript_variant
rs1389641872	intron_variant
rs1389643930	intron_variant
rs1389698759	intron_variant
rs1389753853	intron_variant,genic_upstream_transcript_variant
rs1389811761	intron_variant
rs1389840688	intron_variant
rs1389869505	intron_variant,genic_downstream_transcript_variant
rs1389884382	intron_variant,genic_upstream_transcript_variant
rs1389977134	intron_variant
rs1390002568	intron_variant,genic_upstream_transcript_variant
rs1390003037	intron_variant
rs1390016918	genic_downstream_transcript_variant,3_prime_UTR_variant,non_coding_transcript_variant
rs1390034180	intron_variant,genic_upstream_transcript_variant
rs1390036063	intron_variant,genic_downstream_transcript_variant
rs1390039369	coding_sequence_variant,missense_variant,non_coding_transcript_variant
rs1390101748	intron_variant
rs1390107936	intron_variant,genic_downstream_transcript_variant
rs1390117090	intron_variant,genic_upstream_transcript_variant
rs1390181967	intron_variant,genic_upstream_transcript_variant
rs1390183727	intron_variant,genic_downstream_transcript_variant
rs1390229875	downstream_transcript_variant,intron_variant,genic_downstream_transcript_variant
rs1390238580	intron_variant,genic_upstream_transcript_variant,upstream_transcript_variant
rs1390276030	intron_variant,genic_upstream_transcript_variant
rs1390298808	intron_variant
rs1390326041	intron_variant
rs1390348104	intron_variant,genic_upstream_transcript_variant
rs1390355629	intron_variant,genic_downstream_transcript_variant
rs1390415667	intron_variant,genic_upstream_transcript_variant
rs1390452240	intron_variant,genic_upstream_transcript_variant
rs1390473288	intron_variant,genic_upstream_transcript_variant
rs1390517780	intron_variant,genic_upstream_transcript_variant
rs1390555366	intron_variant,genic_upstream_transcript_variant
rs1390567365	intron_variant
rs1390588460	intron_variant,genic_upstream_transcript_variant
rs1390596457	intron_variant
rs1390656474	intron_variant
rs1390659298	intron_variant,genic_upstream_transcript_variant
rs1390690848	intron_variant,genic_downstream_transcript_variant
rs1390691749	intron_variant,genic_upstream_transcript_variant
rs1390759798	intron_variant
rs1390768627	intron_variant,genic_upstream_transcript_variant
rs1390813768	intron_variant
rs1390825507	intron_variant,genic_upstream_transcript_variant
rs1390845450	intron_variant
rs1390880579	intron_variant
rs1390885683	intron_variant
rs1390896432	intron_variant,genic_downstream_transcript_variant
rs1390926163	intron_variant,genic_upstream_transcript_variant
rs1390946091	intron_variant,genic_upstream_transcript_variant
rs1390948619	intron_variant,genic_upstream_transcript_variant
rs1390948791	intron_variant,genic_upstream_transcript_variant,upstream_transcript_variant
rs1390963367	intron_variant,genic_upstream_transcript_variant
rs1390972971	intron_variant,genic_upstream_transcript_variant
rs1390976676	intron_variant,genic_downstream_transcript_variant
rs1390999860	intron_variant,genic_downstream_transcript_variant
rs1391009634	intron_variant,genic_upstream_transcript_variant
rs1391057922	intron_variant,genic_upstream_transcript_variant
rs1391079652	intron_variant
rs1391110775	intron_variant,genic_upstream_transcript_variant
rs1391113855	intron_variant,genic_upstream_transcript_variant
rs1391140756	intron_variant,genic_upstream_transcript_variant
rs1391159415	intron_variant,genic_upstream_transcript_variant
rs1391172129	genic_upstream_transcript_variant,intron_variant,5_prime_UTR_variant,upstream_transcript_variant
rs1391183781	intron_variant,genic_upstream_transcript_variant
rs1391198809	intron_variant,genic_downstream_transcript_variant
rs1391242302	intron_variant,genic_downstream_transcript_variant
rs1391268690	intron_variant,genic_downstream_transcript_variant
rs1391387615	intron_variant,genic_upstream_transcript_variant
rs1391401423	intron_variant

rs1391418372	intron_variant,genic_upstream_transcript_variant
rs1391469707	upstream_transcript_variant,intron_variant,genic_upstream_transcript_variant,2KB_upstream_variant
rs1391491424	intron_variant,genic_upstream_transcript_variant
rs1391519514	intron_variant,genic_upstream_transcript_variant
rs1391539830	intron_variant
rs1391540049	intron_variant,genic_downstream_transcript_variant
rs1391553931	intron_variant
rs1391557859	intron_variant,genic_upstream_transcript_variant
rs1391563299	intron_variant
rs1391586038	intron_variant
rs1391590759	intron_variant,genic_upstream_transcript_variant,upstream_transcript_variant
rs1391603220	intron_variant
rs1391603972	intron_variant,genic_downstream_transcript_variant
rs1391607156	intron_variant,genic_upstream_transcript_variant
rs1391626998	intron_variant
rs1391636683	intron_variant,genic_upstream_transcript_variant,upstream_transcript_variant
rs1391680687	intron_variant,missense_variant,genic_upstream_transcript_variant,coding_sequence_variant,non_coding_transcript_variant
rs1391694378	intron_variant
rs1391715912	intron_variant,genic_downstream_transcript_variant
rs1391742795	downstream_transcript_variant,500B_downstream_variant
rs1391774518	intron_variant,genic_upstream_transcript_variant
rs1391790850	intron_variant,genic_upstream_transcript_variant
rs1391841634	intron_variant,genic_upstream_transcript_variant
rs1391842538	intron_variant
rs1391865462	intron_variant,genic_upstream_transcript_variant
rs1391904010	intron_variant,genic_upstream_transcript_variant
rs1391923217	intron_variant,genic_upstream_transcript_variant
rs1391943001	intron_variant,genic_upstream_transcript_variant
rs1392001441	intron_variant
rs1392027282	intron_variant
rs1392046754	intron_variant
rs1392060595	intron_variant
rs1392065314	intron_variant,genic_upstream_transcript_variant
rs1392076416	intron_variant,genic_downstream_transcript_variant
rs1392076852	intron_variant,genic_upstream_transcript_variant
rs1392083114	intron_variant,genic_upstream_transcript_variant
rs1392135008	upstream_transcript_variant,intron_variant,genic_upstream_transcript_variant,2KB_upstream_variant
rs1392176735	intron_variant,genic_upstream_transcript_variant
rs1392207037	intron_variant,genic_upstream_transcript_variant
rs1392251633	intron_variant
rs1392252263	intron_variant,genic_upstream_transcript_variant
rs1392271299	intron_variant,genic_upstream_transcript_variant
rs1392284200	intron_variant,genic_upstream_transcript_variant
rs1392321895	intron_variant,genic_upstream_transcript_variant
rs1392358198	intron_variant,genic_upstream_transcript_variant
rs1392368017	intron_variant,genic_downstream_transcript_variant
rs1392417542	intron_variant
rs1392437791	intron_variant,genic_upstream_transcript_variant
rs1392449523	intron_variant,genic_upstream_transcript_variant
rs1392468798	intron_variant,genic_upstream_transcript_variant,upstream_transcript_variant
rs1392471361	intron_variant
rs1392491294	intron_variant,genic_upstream_transcript_variant,upstream_transcript_variant
rs1392501177	intron_variant
rs1392534201	intron_variant,genic_upstream_transcript_variant
rs1392538796	intron_variant
rs1392605251	intron_variant,genic_upstream_transcript_variant
rs1392658166	intron_variant
rs1392697024	intron_variant,genic_upstream_transcript_variant
rs1392717082	intron_variant,genic_upstream_transcript_variant
rs1392717772	intron_variant,genic_downstream_transcript_variant
rs1392720195	intron_variant,genic_upstream_transcript_variant
rs1392735114	intron_variant,genic_upstream_transcript_variant
rs1392743285	intron_variant,genic_downstream_transcript_variant
rs1392773719	intron_variant,genic_downstream_transcript_variant
rs1392786339	intron_variant
rs1392825301	intron_variant,genic_upstream_transcript_variant
rs1392829448	synonymous_variant,intron_variant,missense_variant,genic_upstream_transcript_variant,coding_sequence_variant,non_coding_transcript_variant
rs1392866293	intron_variant,genic_upstream_transcript_variant,upstream_transcript_variant
rs1392879999	upstream_transcript_variant,intron_variant,genic_upstream_transcript_variant,2KB_upstream_variant
rs1392983126	intron_variant,genic_downstream_transcript_variant
rs1393007534	intron_variant,genic_upstream_transcript_variant
rs1393020819	intron_variant,genic_upstream_transcript_variant
rs1393087394	intron_variant,genic_downstream_transcript_variant
rs1393097964	intron_variant,genic_downstream_transcript_variant

rs1393154492	intron_variant
rs1393154617	intron_variant
rs1393190201	intron_variant
rs1393197238	intron_variant,genic_downstream_transcript_variant
rs1393311156	intron_variant,genic_downstream_transcript_variant
rs1393324757	intron_variant,genic_upstream_transcript_variant
rs1393343136	intron_variant,genic_upstream_transcript_variant
rs1393360554	upstream_transcript_variant,intron_variant,genic_upstream_transcript_variant,2KB_upstream_variant
rs1393396497	intron_variant
rs1393412131	intron_variant
rs1393448723	intron_variant,genic_upstream_transcript_variant
rs1393520091	intron_variant,genic_downstream_transcript_variant
rs1393535176	intron_variant,genic_upstream_transcript_variant
rs1393637625	intron_variant,genic_downstream_transcript_variant
rs1393653506	intron_variant,genic_downstream_transcript_variant
rs1393681568	intron_variant,genic_downstream_transcript_variant
rs1393727634	intron_variant,genic_upstream_transcript_variant
rs1393728777	upstream_transcript_variant,intron_variant,genic_upstream_transcript_variant,2KB_upstream_variant
rs1393739006	intron_variant,genic_downstream_transcript_variant
rs1393764968	intron_variant,genic_upstream_transcript_variant
rs1393778508	intron_variant,genic_upstream_transcript_variant
rs1393822256	intron_variant,genic_downstream_transcript_variant
rs1393846996	intron_variant,genic_upstream_transcript_variant
rs1393938014	intron_variant,genic_upstream_transcript_variant
rs1393939095	genic_downstream_transcript_variant,3_prime_UTR_variant,non_coding_transcript_variant
rs1393984038	intron_variant,genic_upstream_transcript_variant
rs1394019228	intron_variant,genic_upstream_transcript_variant
rs1394050458	intron_variant
rs1394061739	intron_variant,genic_upstream_transcript_variant
rs1394124974	intron_variant
rs1394127304	intron_variant,genic_upstream_transcript_variant
rs1394183208	intron_variant
rs1394231349	intron_variant,genic_upstream_transcript_variant
rs1394240816	intron_variant
rs1394284673	intron_variant,genic_downstream_transcript_variant
rs1394299674	intron_variant,genic_upstream_transcript_variant
rs1394303425	intron_variant
rs1394308434	intron_variant,genic_upstream_transcript_variant
rs1394311352	intron_variant
rs1394329370	intron_variant,genic_upstream_transcript_variant
rs1394382625	intron_variant
rs1394383257	intron_variant
rs1394402012	intron_variant,genic_upstream_transcript_variant,upstream_transcript_variant
rs1394427951	intron_variant
rs1394428259	intron_variant,genic_upstream_transcript_variant
rs1394440250	intron_variant,genic_downstream_transcript_variant
rs1394544879	intron_variant,genic_upstream_transcript_variant
rs1394556471	intron_variant
rs1394562472	intron_variant,genic_upstream_transcript_variant
rs1394565500	intron_variant,genic_downstream_transcript_variant
rs1394571385	upstream_transcript_variant,intron_variant,genic_upstream_transcript_variant,2KB_upstream_variant
rs1394580514	intron_variant,genic_downstream_transcript_variant
rs1394583519	intron_variant,genic_downstream_transcript_variant
rs1394618775	intron_variant,genic_upstream_transcript_variant
rs1394629404	intron_variant,genic_downstream_transcript_variant
rs1394633587	intron_variant,genic_upstream_transcript_variant
rs1394636708	intron_variant,genic_downstream_transcript_variant
rs1394640404	intron_variant
rs1394735019	intron_variant,genic_upstream_transcript_variant
rs1394769739	intron_variant,genic_upstream_transcript_variant
rs1394809365	intron_variant,genic_upstream_transcript_variant
rs1394815797	intron_variant,genic_upstream_transcript_variant
rs1394896281	intron_variant,genic_downstream_transcript_variant
rs1394912220	intron_variant
rs1394921073	intron_variant,genic_downstream_transcript_variant
rs1394940728	genic_downstream_transcript_variant,3_prime_UTR_variant,non_coding_transcript_variant
rs1394942238	intron_variant,genic_upstream_transcript_variant
rs1394949637	intron_variant,genic_downstream_transcript_variant
rs1394956840	intron_variant,genic_downstream_transcript_variant
rs1394963810	intron_variant
rs1394970351	intron_variant,genic_downstream_transcript_variant
rs1395014018	intron_variant
rs1395027799	intron_variant
rs1395028454	intron_variant

rs1395039176	intron_variant,genic_upstream_transcript_variant
rs1395060625	intron_variant,genic_downstream_transcript_variant
rs1395064472	intron_variant,genic_upstream_transcript_variant
rs1395082543	upstream_transcript_variant,intron_variant,genic_upstream_transcript_variant,2KB_upstream_variant
rs1395113889	intron_variant
rs1395139124	intron_variant,genic_upstream_transcript_variant
rs1395181621	intron_variant,genic_downstream_transcript_variant
rs1395206293	intron_variant,genic_upstream_transcript_variant
rs1395265340	intron_variant
rs1395279142	intron_variant
rs1395299343	intron_variant,genic_upstream_transcript_variant
rs1395333171	intron_variant
rs1395357475	intron_variant,genic_downstream_transcript_variant
rs1395357537	intron_variant
rs1395517273	intron_variant
rs1395519589	intron_variant,genic_upstream_transcript_variant
rs1395523674	intron_variant,genic_upstream_transcript_variant
rs1395581151	intron_variant,genic_upstream_transcript_variant
rs1395592378	intron_variant,genic_downstream_transcript_variant
rs1395642953	intron_variant,genic_upstream_transcript_variant
rs1395687041	intron_variant
rs1395692890	intron_variant
rs1395760932	upstream_transcript_variant,intron_variant,genic_upstream_transcript_variant,2KB_upstream_variant
rs1395775084	intron_variant
rs1395804138	intron_variant,genic_upstream_transcript_variant
rs1395862494	intron_variant,genic_upstream_transcript_variant
rs1395897044	intron_variant,genic_upstream_transcript_variant
rs1395901955	intron_variant
rs1395921645	intron_variant
rs1395922943	intron_variant,genic_downstream_transcript_variant
rs1395924398	intron_variant
rs1395984612	intron_variant
rs1395995307	intron_variant,genic_upstream_transcript_variant
rs1396075295	upstream_transcript_variant,intron_variant,genic_upstream_transcript_variant,2KB_upstream_variant
rs1396143056	intron_variant,genic_downstream_transcript_variant
rs1396170556	intron_variant
rs1396223664	intron_variant
rs1396274963	upstream_transcript_variant,intron_variant,genic_upstream_transcript_variant,2KB_upstream_variant
rs1396297602	intron_variant,genic_upstream_transcript_variant
rs1396299962	intron_variant,genic_downstream_transcript_variant
rs1396323039	intron_variant
rs1396363918	intron_variant,genic_upstream_transcript_variant
rs1396385102	intron_variant
rs1396423002	upstream_transcript_variant,intron_variant,genic_upstream_transcript_variant,2KB_upstream_variant
rs1396435945	intron_variant,genic_upstream_transcript_variant
rs1396436522	intron_variant
rs1396493554	intron_variant,genic_upstream_transcript_variant
rs1396527808	intron_variant,genic_upstream_transcript_variant
rs1396576070	intron_variant
rs1396664478	intron_variant,genic_downstream_transcript_variant
rs1396683493	intron_variant
rs1396701351	intron_variant,genic_upstream_transcript_variant
rs1396740952	intron_variant
rs1396745115	intron_variant,genic_downstream_transcript_variant
rs1396752765	intron_variant,genic_downstream_transcript_variant
rs1396776719	genic_upstream_transcript_variant,intron_variant
rs1396794558	genic_upstream_transcript_variant,intron_variant
rs1396875725	intron_variant
rs1396897770	genic_upstream_transcript_variant,intron_variant
rs1396993395	genic_downstream_transcript_variant,intron_variant
rs1396999546	genic_downstream_transcript_variant,intron_variant
rs1397038390	genic_upstream_transcript_variant,intron_variant
rs1397043697	genic_upstream_transcript_variant,intron_variant
rs1397073927	genic_upstream_transcript_variant,intron_variant
rs1397081075	intron_variant
rs1397106571	genic_downstream_transcript_variant,intron_variant
rs1397114258	genic_downstream_transcript_variant,intron_variant
rs1397140271	genic_upstream_transcript_variant,intron_variant
rs1397190387	genic_upstream_transcript_variant,intron_variant,2KB_upstream_variant,upstream_transcript_variant
rs1397219098	intron_variant
rs1397237824	genic_downstream_transcript_variant,intron_variant
rs1397246785	genic_upstream_transcript_variant,intron_variant
rs1397283936	intron_variant
rs1397364142	genic_downstream_transcript_variant,intron_variant

rs1397370375	genic_downstream_transcript_variant,intron_variant
rs1397379383	splice_acceptor_variant
rs1397433742	intron_variant
rs1397454094	genic_upstream_transcript_variant,intron_variant
rs1397463668	genic_upstream_transcript_variant,intron_variant,2KB_upstream_variant,upstream_transcript_variant
rs1397481642	genic_upstream_transcript_variant,intron_variant
rs1397486422	genic_downstream_transcript_variant,intron_variant
rs1397525688	intron_variant
rs1397525801	genic_downstream_transcript_variant,3_prime_UTR_variant,non_coding_transcript_variant
rs1397539101	intron_variant
rs1397560319	intron_variant
rs1397583022	genic_downstream_transcript_variant,intron_variant
rs1397666697	genic_downstream_transcript_variant,intron_variant
rs1397709571	genic_upstream_transcript_variant,intron_variant,2KB_upstream_variant,upstream_transcript_variant
rs1397732742	genic_upstream_transcript_variant,intron_variant
rs1397739698	genic_downstream_transcript_variant,intron_variant
rs1397743685	genic_upstream_transcript_variant,intron_variant,upstream_transcript_variant
rs1397796844	genic_upstream_transcript_variant,intron_variant,upstream_transcript_variant
rs1397844602	intron_variant
rs1397851383	genic_upstream_transcript_variant,intron_variant
rs1397861032	genic_upstream_transcript_variant,intron_variant
rs1397888194	genic_upstream_transcript_variant,intron_variant
rs1397898796	intron_variant
rs1397937296	genic_upstream_transcript_variant,intron_variant
rs1397945397	genic_downstream_transcript_variant,intron_variant
rs1397971431	genic_upstream_transcript_variant,intron_variant,2KB_upstream_variant,upstream_transcript_variant
rs1397981108	genic_upstream_transcript_variant,intron_variant,2KB_upstream_variant,upstream_transcript_variant
rs1397996024	genic_downstream_transcript_variant,intron_variant
rs1398002795	genic_upstream_transcript_variant,intron_variant,upstream_transcript_variant
rs1398026932	genic_upstream_transcript_variant,intron_variant
rs1398056510	intron_variant
rs1398063583	genic_upstream_transcript_variant,intron_variant,upstream_transcript_variant
rs1398098105	genic_downstream_transcript_variant,3_prime_UTR_variant,non_coding_transcript_variant
rs1398104958	intron_variant
rs1398127915	genic_downstream_transcript_variant,intron_variant
rs1398180577	genic_downstream_transcript_variant,intron_variant
rs1398215122	intron_variant
rs1398224126	intron_variant
rs1398255358	genic_upstream_transcript_variant,intron_variant
rs1398282604	genic_upstream_transcript_variant,intron_variant
rs1398289052	genic_upstream_transcript_variant,intron_variant,2KB_upstream_variant,upstream_transcript_variant
rs1398292232	intron_variant
rs1398293033	intron_variant
rs1398339494	genic_upstream_transcript_variant,intron_variant
rs1398418585	intron_variant
rs1398420227	genic_upstream_transcript_variant,intron_variant
rs1398452089	intron_variant
rs1398457521	genic_upstream_transcript_variant,intron_variant
rs1398473599	intron_variant
rs1398484254	genic_upstream_transcript_variant,intron_variant
rs1398575273	intron_variant
rs1398582671	genic_upstream_transcript_variant,intron_variant
rs1398589576	genic_upstream_transcript_variant,intron_variant
rs1398612320	genic_downstream_transcript_variant,intron_variant
rs1398685464	intron_variant
rs1398709370	genic_downstream_transcript_variant,intron_variant
rs1398713358	genic_upstream_transcript_variant,intron_variant
rs1398785484	genic_downstream_transcript_variant,intron_variant
rs1398816615	genic_upstream_transcript_variant,intron_variant
rs1398819393	genic_upstream_transcript_variant,intron_variant
rs1398845800	genic_upstream_transcript_variant,intron_variant
rs1398896443	genic_upstream_transcript_variant,intron_variant
rs1398903280	genic_upstream_transcript_variant,intron_variant
rs1398938962	genic_upstream_transcript_variant,intron_variant
rs1398957115	genic_downstream_transcript_variant,intron_variant
rs1398963779	genic_upstream_transcript_variant,intron_variant
rs1399040652	genic_upstream_transcript_variant,intron_variant
rs1399041725	genic_upstream_transcript_variant,intron_variant
rs1399047858	genic_upstream_transcript_variant,intron_variant
rs1399068291	genic_upstream_transcript_variant,intron_variant,2KB_upstream_variant,upstream_transcript_variant
rs1399090336	intron_variant
rs1399171071	intron_variant
rs1399210476	genic_upstream_transcript_variant,intron_variant
rs1399225087	intron_variant

rs1399247454	genic_upstream_transcript_variant,intron_variant
rs1399262460	intron_variant
rs1399264024	intron_variant
rs1399270343	intron_variant
rs1399276258	genic_downstream_transcript_variant,intron_variant
rs1399308676	genic_downstream_transcript_variant,3_prime_UTR_variant,non_coding_transcript_variant
rs1399336597	intron_variant
rs1399349778	genic_upstream_transcript_variant,intron_variant
rs1399350541	intron_variant
rs1399463760	genic_downstream_transcript_variant,3_prime_UTR_variant,non_coding_transcript_variant
rs1399541508	genic_downstream_transcript_variant,intron_variant
rs1399542264	genic_upstream_transcript_variant,intron_variant
rs1399555312	intron_variant
rs1399556261	intron_variant
rs1399601631	genic_upstream_transcript_variant,intron_variant
rs1399610063	genic_downstream_transcript_variant,intron_variant
rs1399630402	intron_variant
rs1399651611	genic_upstream_transcript_variant,intron_variant
rs1399691058	genic_upstream_transcript_variant,intron_variant
rs1399734837	intron_variant
rs1399744800	genic_downstream_transcript_variant,intron_variant,downstream_transcript_variant
rs1399779759	genic_downstream_transcript_variant,intron_variant
rs1399798369	coding_sequence_variant,missense_variant,non_coding_transcript_variant
rs1399804096	intron_variant
rs1399815677	genic_upstream_transcript_variant,intron_variant
rs1399845684	genic_upstream_transcript_variant,intron_variant
rs1399920328	genic_downstream_transcript_variant,intron_variant
rs1399923800	intron_variant
rs1400006951	intron_variant
rs1400011582	genic_upstream_transcript_variant,intron_variant
rs1400049256	genic_upstream_transcript_variant,intron_variant
rs1400054224	genic_downstream_transcript_variant,intron_variant
rs1400061840	genic_upstream_transcript_variant,intron_variant
rs1400078960	intron_variant
rs1400115104	intron_variant
rs1400146578	intron_variant
rs1400152335	genic_upstream_transcript_variant,intron_variant,2KB_upstream_variant,upstream_transcript_variant
rs1400165846	intron_variant
rs1400265010	intron_variant
rs1400314541	intron_variant
rs1400319231	intron_variant
rs1400494495	genic_upstream_transcript_variant,intron_variant
rs1400528124	intron_variant
rs1400579016	genic_downstream_transcript_variant,intron_variant
rs1400601138	intron_variant
rs1400697668	genic_downstream_transcript_variant,intron_variant
rs1400704152	intron_variant
rs1400718885	genic_upstream_transcript_variant,intron_variant
rs1400768221	genic_upstream_transcript_variant,intron_variant
rs1400771555	genic_downstream_transcript_variant,intron_variant
rs1400781732	genic_upstream_transcript_variant,intron_variant,upstream_transcript_variant
rs1400782170	genic_upstream_transcript_variant,intron_variant
rs1400784733	intron_variant
rs1400850827	genic_upstream_transcript_variant,intron_variant,upstream_transcript_variant
rs1400860408	genic_upstream_transcript_variant,intron_variant
rs1400902854	intron_variant
rs1400907482	intron_variant
rs1400940130	intron_variant
rs1400971832	genic_downstream_transcript_variant,intron_variant
rs1400978209	intron_variant
rs1400988119	intron_variant
rs1401024274	genic_upstream_transcript_variant,intron_variant
rs1401030161	intron_variant
rs1401089255	intron_variant
rs1401138460	coding_sequence_variant,missense_variant,non_coding_transcript_variant
rs1401166174	genic_downstream_transcript_variant,intron_variant
rs1401253924	genic_upstream_transcript_variant,intron_variant
rs1401290558	intron_variant
rs1401296933	genic_upstream_transcript_variant,intron_variant
rs1401299513	intron_variant
rs1401320870	genic_upstream_transcript_variant,intron_variant,upstream_transcript_variant
rs1401348506	genic_upstream_transcript_variant,intron_variant
rs1401357145	genic_upstream_transcript_variant,intron_variant
rs1401393203	genic_upstream_transcript_variant,intron_variant,2KB_upstream_variant,upstream_transcript_variant

rs1401415452	intron_variant
rs1401438772	intron_variant
rs1401474245	intron_variant
rs1401474552	genic_downstream_transcript_variant,intron_variant
rs1401513640	genic_upstream_transcript_variant,intron_variant
rs1401620046	genic_downstream_transcript_variant,intron_variant
rs1401629190	genic_upstream_transcript_variant,intron_variant
rs1401636710	genic_upstream_transcript_variant,intron_variant
rs1401653678	genic_upstream_transcript_variant,intron_variant
rs1401671481	genic_downstream_transcript_variant,intron_variant
rs1401700111	genic_downstream_transcript_variant,intron_variant
rs1401728449	genic_downstream_transcript_variant,3_prime_UTR_variant,non_coding_transcript_variant
rs1401756364	genic_upstream_transcript_variant,intron_variant,upstream_transcript_variant
rs1401765810	genic_upstream_transcript_variant,intron_variant
rs1401842053	genic_upstream_transcript_variant,intron_variant
rs1401842100	intron_variant
rs1401848948	genic_upstream_transcript_variant,intron_variant,2KB_upstream_variant,upstream_transcript_variant
rs1401890456	genic_upstream_transcript_variant,intron_variant
rs1401917880	genic_upstream_transcript_variant,intron_variant
rs1401931585	genic_upstream_transcript_variant,intron_variant
rs1401942989	intron_variant
rs1402062262	genic_upstream_transcript_variant,intron_variant
rs1402078503	genic_upstream_transcript_variant,intron_variant,upstream_transcript_variant
rs1402089605	genic_upstream_transcript_variant,intron_variant
rs1402126322	intron_variant
rs1402168154	intron_variant
rs1402177081	genic_upstream_transcript_variant,intron_variant
rs1402213895	intron_variant
rs1402228351	genic_upstream_transcript_variant,intron_variant
rs1402247015	genic_upstream_transcript_variant,intron_variant
rs1402255562	genic_downstream_transcript_variant,intron_variant
rs1402344381	genic_upstream_transcript_variant,intron_variant
rs1402358414	genic_downstream_transcript_variant,intron_variant
rs1402363842	genic_upstream_transcript_variant,intron_variant
rs1402366432	genic_upstream_transcript_variant,intron_variant
rs1402419587	intron_variant
rs1402419977	intron_variant
rs1402426825	genic_upstream_transcript_variant,intron_variant
rs1402442649	intron_variant
rs1402451619	genic_upstream_transcript_variant,intron_variant
rs1402485042	genic_upstream_transcript_variant,intron_variant
rs1402503967	genic_downstream_transcript_variant,coding_sequence_variant,missense_variant,non_coding_transcript_variant
rs1402504699	genic_upstream_transcript_variant,intron_variant
rs1402518966	genic_upstream_transcript_variant,intron_variant
rs1402527598	genic_upstream_transcript_variant,intron_variant
rs1402529434	genic_downstream_transcript_variant,intron_variant
rs1402555312	intron_variant
rs1402584222	genic_upstream_transcript_variant,intron_variant
rs1402597855	intron_variant
rs1402599741	intron_variant
rs1402653699	genic_upstream_transcript_variant,intron_variant
rs1402661125	intron_variant
rs1402662402	genic_downstream_transcript_variant,intron_variant
rs1402663467	intron_variant
rs1402663741	intron_variant
rs1402663951	intron_variant
rs1402679515	genic_upstream_transcript_variant,intron_variant
rs1402699936	genic_downstream_transcript_variant,intron_variant
rs1402714522	genic_upstream_transcript_variant,intron_variant
rs1402746532	genic_downstream_transcript_variant,3_prime_UTR_variant,non_coding_transcript_variant
rs1402754217	genic_upstream_transcript_variant,intron_variant,upstream_transcript_variant
rs1402788042	genic_downstream_transcript_variant,intron_variant
rs1402818786	intron_variant
rs1402828262	intron_variant
rs1402831493	intron_variant
rs1402875941	genic_upstream_transcript_variant,intron_variant
rs1402898507	genic_upstream_transcript_variant,intron_variant
rs1402909043	genic_upstream_transcript_variant,intron_variant
rs1402910964	intron_variant
rs1402960587	intron_variant
rs1402999757	genic_upstream_transcript_variant,intron_variant
rs1403002682	genic_upstream_transcript_variant,intron_variant,upstream_transcript_variant
rs1403063008	genic_upstream_transcript_variant,intron_variant
rs1403096549	intron_variant

rs1403121735	genic_downstream_transcript_variant,intron_variant
rs1403127976	genic_upstream_transcript_variant,intron_variant
rs1403151290	genic_upstream_transcript_variant,intron_variant,2KB_upstream_variant,upstream_transcript_variant
rs1403156551	intron_variant
rs1403177208	genic_downstream_transcript_variant,intron_variant
rs1403190223	genic_upstream_transcript_variant,intron_variant
rs1403223634	intron_variant
rs1403237125	genic_downstream_transcript_variant,intron_variant
rs1403246310	intron_variant
rs1403261908	intron_variant
rs1403289841	intron_variant
rs1403316374	intron_variant
rs1403329924	genic_upstream_transcript_variant,intron_variant
rs1403354032	genic_downstream_transcript_variant,intron_variant
rs1403372645	genic_downstream_transcript_variant,intron_variant
rs1403388670	genic_upstream_transcript_variant,intron_variant
rs1403393822	intron_variant
rs1403422565	intron_variant
rs1403424653	intron_variant
rs1403468253	genic_upstream_transcript_variant,intron_variant
rs1403495982	intron_variant
rs1403508414	genic_upstream_transcript_variant,intron_variant
rs1403635061	genic_upstream_transcript_variant,intron_variant
rs1403657655	intron_variant
rs1403719773	intron_variant
rs1403741537	intron_variant
rs1403793431	genic_downstream_transcript_variant,intron_variant
rs1403795037	intron_variant
rs1403840707	genic_upstream_transcript_variant,intron_variant
rs1403856425	intron_variant
rs1403864326	genic_upstream_transcript_variant,intron_variant
rs1403887434	intron_variant
rs1403901306	genic_downstream_transcript_variant,intron_variant
rs1403933327	genic_upstream_transcript_variant,intron_variant
rs1403950811	intron_variant
rs1403978051	intron_variant
rs1403996664	genic_upstream_transcript_variant,intron_variant
rs1403999086	genic_downstream_transcript_variant,intron_variant
rs1404048688	genic_upstream_transcript_variant,intron_variant
rs1404073539	genic_upstream_transcript_variant,intron_variant
rs1404119190	intron_variant
rs1404140529	genic_downstream_transcript_variant,intron_variant
rs1404149659	genic_upstream_transcript_variant,intron_variant
rs1404157832	coding_sequence_variant,missense_variant,non_coding_transcript_variant
rs1404205667	genic_upstream_transcript_variant,intron_variant
rs1404211585	intron_variant
rs1404222846	genic_upstream_transcript_variant,intron_variant
rs1404228616	genic_upstream_transcript_variant,intron_variant
rs1404249213	genic_upstream_transcript_variant,intron_variant
rs1404270987	genic_downstream_transcript_variant,intron_variant
rs1404358656	genic_upstream_transcript_variant,intron_variant
rs1404368926	genic_downstream_transcript_variant,intron_variant
rs1404382281	intron_variant
rs1404385206	intron_variant
rs1404391420	intron_variant
rs1404400198	intron_variant
rs1404448573	genic_upstream_transcript_variant,intron_variant
rs1404481890	genic_upstream_transcript_variant,intron_variant
rs1404502692	genic_downstream_transcript_variant,intron_variant
rs1404521953	intron_variant
rs1404524774	genic_upstream_transcript_variant,intron_variant
rs1404557562	genic_downstream_transcript_variant,intron_variant
rs1404557904	genic_upstream_transcript_variant,intron_variant
rs1404589968	genic_upstream_transcript_variant,intron_variant
rs1404610035	genic_downstream_transcript_variant,intron_variant
rs1404611923	genic_downstream_transcript_variant,intron_variant
rs1404642938	intron_variant
rs1404644873	intron_variant
rs1404664878	coding_sequence_variant,missense_variant,non_coding_transcript_variant
rs1404669620	genic_upstream_transcript_variant,intron_variant
rs1404701545	genic_upstream_transcript_variant,intron_variant
rs1404754489	genic_downstream_transcript_variant,intron_variant
rs1404769803	genic_upstream_transcript_variant,intron_variant,upstream_transcript_variant
rs1404770724	genic_upstream_transcript_variant,intron_variant

rs1404868702	genic_upstream_transcript_variant,intron_variant,2KB_upstream_variant,upstream_transcript_variant
rs1404944327	intron_variant
rs1404976765	intron_variant
rs1405065544	intron_variant
rs1405133922	genic_upstream_transcript_variant,intron_variant
rs1405135998	genic_upstream_transcript_variant,intron_variant
rs1405145346	genic_upstream_transcript_variant,intron_variant
rs1405198664	genic_upstream_transcript_variant,intron_variant
rs1405217794	genic_upstream_transcript_variant,intron_variant,upstream_transcript_variant
rs1405241317	intron_variant
rs1405275048	intron_variant
rs1405368883	genic_downstream_transcript_variant,intron_variant
rs1405398824	genic_downstream_transcript_variant,intron_variant
rs1405405272	genic_downstream_transcript_variant,intron_variant
rs1405445793	genic_upstream_transcript_variant,intron_variant
rs1405470971	intron_variant
rs1405530230	genic_downstream_transcript_variant,3_prime_UTR_variant,non_coding_transcript_variant
rs1405646437	genic_upstream_transcript_variant,intron_variant
rs1405684941	genic_downstream_transcript_variant,intron_variant
rs1405687873	genic_upstream_transcript_variant,intron_variant
rs1405717731	genic_downstream_transcript_variant,intron_variant
rs1405720332	intron_variant
rs1405723345	genic_upstream_transcript_variant,intron_variant
rs1405793633	genic_downstream_transcript_variant,intron_variant
rs1405799810	genic_downstream_transcript_variant,intron_variant
rs1405802049	intron_variant
rs1405848769	genic_upstream_transcript_variant,intron_variant
rs1405892315	intron_variant
rs1405913973	intron_variant
rs1405988381	genic_upstream_transcript_variant,intron_variant
rs1406010807	genic_upstream_transcript_variant,intron_variant
rs1406015175	genic_upstream_transcript_variant,intron_variant
rs1406051361	intron_variant
rs1406081347	genic_upstream_transcript_variant,intron_variant
rs1406084651	intron_variant
rs1406099770	intron_variant
rs1406119507	genic_upstream_transcript_variant,5_prime_UTR_variant,intron_variant,upstream_transcript_variant
rs1406160567	genic_downstream_transcript_variant,intron_variant
rs1406165600	intron_variant
rs1406254208	genic_downstream_transcript_variant,intron_variant
rs1406266599	intron_variant
rs1406300427	genic_upstream_transcript_variant,intron_variant,upstream_transcript_variant
rs1406315678	intron_variant
rs1406323885	intron_variant
rs1406345426	intron_variant
rs1406352796	genic_upstream_transcript_variant,intron_variant,upstream_transcript_variant
rs1406399745	intron_variant
rs1406412610	genic_upstream_transcript_variant,intron_variant
rs1406435985	intron_variant
rs1406444235	genic_upstream_transcript_variant,intron_variant
rs1406512018	genic_upstream_transcript_variant,intron_variant
rs1406551909	genic_downstream_transcript_variant,intron_variant
rs1406571582	intron_variant
rs1406593098	intron_variant
rs1406593131	genic_upstream_transcript_variant,intron_variant
rs1406618410	genic_upstream_transcript_variant,intron_variant
rs1406629732	genic_upstream_transcript_variant,intron_variant
rs1406817029	genic_downstream_transcript_variant,3_prime_UTR_variant,non_coding_transcript_variant
rs1406831574	intron_variant
rs1406889695	genic_upstream_transcript_variant,intron_variant
rs1406901812	genic_upstream_transcript_variant,intron_variant
rs1406927755	genic_upstream_transcript_variant,intron_variant
rs1406951205	genic_downstream_transcript_variant,intron_variant
rs1407032044	intron_variant
rs1407064292	intron_variant
rs1407076118	intron_variant
rs1407087270	genic_upstream_transcript_variant,intron_variant
rs1407091744	intron_variant
rs1407199518	genic_upstream_transcript_variant,intron_variant
rs1407216678	genic_downstream_transcript_variant,intron_variant
rs1407252678	genic_downstream_transcript_variant,intron_variant
rs1407336963	genic_upstream_transcript_variant,intron_variant,2KB_upstream_variant,upstream_transcript_variant
rs1407358466	genic_upstream_transcript_variant,intron_variant
rs1407368886	intron_variant

rs1407383609	genic_downstream_transcript_variant,intron_variant
rs1407399030	genic_upstream_transcript_variant,intron_variant,upstream_transcript_variant
rs1407428091	genic_upstream_transcript_variant,intron_variant
rs1407456891	genic_upstream_transcript_variant,intron_variant
rs1407523075	genic_upstream_transcript_variant,intron_variant
rs1407538714	genic_upstream_transcript_variant,intron_variant
rs1407577477	genic_downstream_transcript_variant,intron_variant
rs1407586096	genic_downstream_transcript_variant,intron_variant
rs1407617054	genic_upstream_transcript_variant,intron_variant
rs1407641271	genic_downstream_transcript_variant,intron_variant
rs1407682776	intron_variant
rs1407683359	intron_variant
rs1407695373	genic_upstream_transcript_variant,intron_variant
rs1407702066	intron_variant
rs1407763259	intron_variant
rs1407769251	intron_variant
rs1407801698	genic_upstream_transcript_variant,intron_variant
rs1407855004	genic_downstream_transcript_variant,intron_variant
rs1407868841	genic_upstream_transcript_variant,intron_variant
rs1407874753	intron_variant
rs1407905265	genic_upstream_transcript_variant,intron_variant
rs1407912510	genic_upstream_transcript_variant,intron_variant
rs1407944805	genic_downstream_transcript_variant,intron_variant
rs1407970053	5_prime_UTR_variant,coding_sequence_variant,synonymous_variant,non_coding_transcript_variant
rs1407973213	intron_variant
rs1407986000	genic_upstream_transcript_variant,intron_variant
rs1408010919	intron_variant
rs1408020593	genic_upstream_transcript_variant,intron_variant
rs1408040941	intron_variant
rs1408052284	intron_variant
rs1408156878	genic_upstream_transcript_variant,intron_variant
rs1408178237	genic_upstream_transcript_variant,intron_variant,upstream_transcript_variant
rs1408191707	genic_upstream_transcript_variant,intron_variant
rs1408230169	genic_upstream_transcript_variant,intron_variant
rs1408247934	genic_upstream_transcript_variant,intron_variant
rs1408288276	genic_downstream_transcript_variant,intron_variant
rs1408336470	intron_variant
rs1408341452	genic_upstream_transcript_variant,intron_variant,2KB_upstream_variant,upstream_transcript_variant
rs1408406328	genic_upstream_transcript_variant,intron_variant
rs1408411636	genic_upstream_transcript_variant,intron_variant
rs1408419351	genic_downstream_transcript_variant,intron_variant
rs1408477450	genic_downstream_transcript_variant,intron_variant
rs1408481977	intron_variant
rs1408515246	genic_upstream_transcript_variant,intron_variant
rs1408528857	intron_variant
rs1408539401	genic_upstream_transcript_variant,intron_variant
rs1408546040	intron_variant
rs1408546075	genic_upstream_transcript_variant,intron_variant
rs1408603216	intron_variant
rs1408615767	intron_variant
rs1408631711	genic_upstream_transcript_variant,intron_variant,2KB_upstream_variant,upstream_transcript_variant
rs1408668493	genic_downstream_transcript_variant,intron_variant
rs1408691505	genic_upstream_transcript_variant,intron_variant
rs1408711856	genic_upstream_transcript_variant,intron_variant
rs1408734401	intron_variant
rs1408755668	genic_upstream_transcript_variant,intron_variant
rs1408773691	genic_upstream_transcript_variant,intron_variant
rs1408776595	genic_downstream_transcript_variant,3_prime_UTR_variant,non_coding_transcript_variant
rs1408806649	genic_upstream_transcript_variant,intron_variant
rs1408817208	genic_upstream_transcript_variant,intron_variant
rs1408828933	intron_variant
rs1408855733	intron_variant
rs1408874725	genic_upstream_transcript_variant,intron_variant
rs1408907462	genic_downstream_transcript_variant,intron_variant
rs1408956787	intron_variant
rs1408974200	genic_upstream_transcript_variant,intron_variant
rs1409018322	intron_variant
rs1409022997	genic_upstream_transcript_variant,intron_variant
rs1409052054	intron_variant
rs1409176560	genic_upstream_transcript_variant,intron_variant
rs1409226578	genic_downstream_transcript_variant,intron_variant
rs1409253185	intron_variant
rs1409256334	genic_upstream_transcript_variant,intron_variant
rs1409286948	genic_upstream_transcript_variant,intron_variant

rs1409336181	intron_variant
rs1409375032	intron_variant,genic_upstream_transcript_variant
rs1409380881	intron_variant
rs1409472143	intron_variant
rs1409479274	intron_variant
rs1409505956	intron_variant,genic_upstream_transcript_variant
rs1409556438	intron_variant,genic_upstream_transcript_variant
rs1409566679	intron_variant,genic_upstream_transcript_variant
rs1409620584	intron_variant
rs1409672945	intron_variant
rs1409677171	intron_variant,genic_upstream_transcript_variant,upstream_transcript_variant
rs1409683864	intron_variant,genic_downstream_transcript_variant
rs1409742491	intron_variant
rs1409750676	intron_variant
rs1409781866	intron_variant,genic_upstream_transcript_variant
rs1409813341	intron_variant,genic_downstream_transcript_variant
rs1409945534	intron_variant,genic_upstream_transcript_variant
rs1410009532	intron_variant,genic_upstream_transcript_variant
rs1410013407	intron_variant
rs1410037401	intron_variant,genic_downstream_transcript_variant
rs1410099782	intron_variant,genic_upstream_transcript_variant
rs1410100135	intron_variant,genic_downstream_transcript_variant
rs1410131871	intron_variant,genic_upstream_transcript_variant
rs1410155882	intron_variant,genic_upstream_transcript_variant
rs1410215863	intron_variant
rs1410251305	intron_variant,genic_upstream_transcript_variant
rs1410268441	intron_variant
rs1410278679	intron_variant,genic_upstream_transcript_variant
rs1410341568	intron_variant,genic_upstream_transcript_variant
rs1410373280	intron_variant,genic_upstream_transcript_variant,5_prime_UTR_variant,upstream_transcript_variant
rs1410405814	intron_variant,genic_upstream_transcript_variant
rs1410433139	intron_variant,genic_downstream_transcript_variant
rs1410463772	intron_variant,genic_upstream_transcript_variant
rs1410476910	intron_variant,genic_upstream_transcript_variant
rs1410506607	genic_downstream_transcript_variant,non_coding_transcript_variant,3_prime_UTR_variant
rs1410528382	intron_variant
rs1410556602	intron_variant,genic_downstream_transcript_variant
rs1410621901	intron_variant,genic_downstream_transcript_variant
rs1410628296	intron_variant
rs1410639381	intron_variant
rs1410664971	intron_variant,genic_downstream_transcript_variant
rs1410677667	intron_variant,genic_downstream_transcript_variant
rs1410744156	intron_variant,genic_downstream_transcript_variant
rs1410752181	intron_variant
rs1410780061	intron_variant,genic_upstream_transcript_variant
rs1410805066	intron_variant,genic_upstream_transcript_variant
rs1410829427	intron_variant,genic_upstream_transcript_variant
rs1410862430	intron_variant,genic_downstream_transcript_variant
rs1410882045	intron_variant,genic_upstream_transcript_variant
rs1410882577	intron_variant,genic_upstream_transcript_variant
rs1410974324	intron_variant,genic_upstream_transcript_variant
rs1411011874	intron_variant,genic_upstream_transcript_variant
rs1411048094	non_coding_transcript_variant,genic_downstream_transcript_variant,coding_sequence_variant,synonymous_variant
rs1411048617	intron_variant,genic_upstream_transcript_variant
rs1411050715	intron_variant
rs1411127306	intron_variant,genic_upstream_transcript_variant
rs1411174360	intron_variant,genic_upstream_transcript_variant
rs1411179958	intron_variant,genic_upstream_transcript_variant
rs1411228867	intron_variant,genic_upstream_transcript_variant
rs1411241079	intron_variant
rs1411249020	2KB_upstream_variant,intron_variant,genic_upstream_transcript_variant,upstream_transcript_variant
rs1411256225	intron_variant,genic_upstream_transcript_variant
rs1411270124	intron_variant,genic_downstream_transcript_variant
rs1411291039	intron_variant,genic_upstream_transcript_variant
rs1411291766	intron_variant
rs1411293192	intron_variant
rs1411310411	intron_variant
rs1411313532	intron_variant,genic_upstream_transcript_variant
rs1411368297	intron_variant
rs1411439592	intron_variant,genic_upstream_transcript_variant
rs1411454216	intron_variant
rs1411536567	intron_variant,genic_downstream_transcript_variant
rs1411577078	downstream_transcript_variant,intron_variant,genic_downstream_transcript_variant
rs1411616925	intron_variant,genic_upstream_transcript_variant

rs1411618983	intron_variant,genic_upstream_transcript_variant
rs1411623614	intron_variant
rs1411664502	intron_variant,genic_downstream_transcript_variant
rs1411665844	intron_variant,genic_downstream_transcript_variant
rs1411677371	intron_variant,genic_upstream_transcript_variant
rs1411713265	intron_variant
rs1411774859	intron_variant,genic_downstream_transcript_variant
rs1411794757	intron_variant
rs1411806901	intron_variant
rs1411809982	intron_variant
rs1411811436	intron_variant,genic_upstream_transcript_variant
rs1411813755	intron_variant
rs1411849844	intron_variant
rs1411904121	intron_variant
rs1411926720	intron_variant
rs1411940340	intron_variant,genic_upstream_transcript_variant
rs1411954415	intron_variant
rs1411994476	intron_variant,genic_upstream_transcript_variant
rs1412005782	downstream_transcript_variant,500B_downstream_variant
rs1412009024	intron_variant,genic_upstream_transcript_variant
rs1412049970	intron_variant,genic_upstream_transcript_variant
rs1412055504	intron_variant,genic_upstream_transcript_variant,upstream_transcript_variant
rs1412071052	intron_variant
rs1412104062	intron_variant
rs1412196289	2KB_upstream_variant,intron_variant,genic_upstream_transcript_variant,upstream_transcript_variant
rs1412211697	intron_variant,genic_upstream_transcript_variant
rs1412217570	intron_variant,genic_downstream_transcript_variant
rs1412226416	intron_variant,genic_upstream_transcript_variant
rs1412244178	intron_variant
rs1412310413	intron_variant,genic_upstream_transcript_variant
rs1412332689	intron_variant
rs1412347312	intron_variant,genic_upstream_transcript_variant
rs1412353826	intron_variant
rs1412427532	intron_variant
rs1412439892	intron_variant,genic_downstream_transcript_variant
rs1412506058	intron_variant,genic_upstream_transcript_variant
rs1412529742	intron_variant
rs1412575531	intron_variant,genic_downstream_transcript_variant
rs1412584391	intron_variant
rs1412591012	intron_variant,genic_downstream_transcript_variant
rs1412665799	intron_variant
rs1412671547	intron_variant,genic_upstream_transcript_variant
rs1412675219	intron_variant,genic_upstream_transcript_variant
rs1412704223	intron_variant
rs1412713259	intron_variant,genic_upstream_transcript_variant
rs1412771681	intron_variant
rs1412842857	intron_variant
rs1412869677	intron_variant,genic_downstream_transcript_variant
rs1412888386	intron_variant
rs1412894899	intron_variant
rs1412969314	intron_variant,genic_upstream_transcript_variant
rs1413004030	intron_variant,genic_downstream_transcript_variant
rs1413004914	intron_variant,genic_upstream_transcript_variant
rs1413043253	intron_variant
rs1413117182	intron_variant
rs1413135472	intron_variant
rs1413162402	genic_downstream_transcript_variant,non_coding_transcript_variant,3_prime_UTR_variant
rs1413175228	intron_variant,genic_upstream_transcript_variant
rs1413209214	missense_variant,non_coding_transcript_variant,genic_downstream_transcript_variant,coding_sequence_variant
rs1413287662	intron_variant
rs1413292436	intron_variant,genic_upstream_transcript_variant,upstream_transcript_variant
rs1413298285	genic_downstream_transcript_variant,non_coding_transcript_variant,3_prime_UTR_variant
rs1413320599	intron_variant,5_prime_UTR_variant
rs1413345204	intron_variant
rs1413350895	intron_variant,genic_upstream_transcript_variant
rs1413359619	intron_variant,genic_upstream_transcript_variant
rs1413422494	intron_variant,genic_downstream_transcript_variant
rs1413430606	intron_variant
rs1413432875	intron_variant,genic_upstream_transcript_variant
rs1413463929	intron_variant
rs1413494415	intron_variant,genic_downstream_transcript_variant
rs1413533878	intron_variant
rs1413587945	intron_variant,genic_upstream_transcript_variant
rs1413659885	intron_variant

rs1413702777	intron_variant,genic_upstream_transcript_variant
rs1413717778	intron_variant,genic_downstream_transcript_variant
rs1413753927	intron_variant
rs1413777203	intron_variant
rs1413827855	intron_variant,genic_upstream_transcript_variant
rs1413830523	intron_variant,genic_upstream_transcript_variant
rs1413851397	intron_variant,genic_upstream_transcript_variant
rs1413852638	intron_variant
rs1413935839	intron_variant,genic_upstream_transcript_variant
rs1413956294	intron_variant,genic_downstream_transcript_variant
rs1413967054	intron_variant
rs1414000639	intron_variant,genic_upstream_transcript_variant
rs1414035564	2KB_upstream_variant,intron_variant,genic_upstream_transcript_variant,upstream_transcript_variant
rs1414055722	intron_variant
rs1414063006	intron_variant,genic_upstream_transcript_variant
rs1414067460	intron_variant,genic_downstream_transcript_variant
rs1414093360	intron_variant,genic_upstream_transcript_variant
rs1414130627	intron_variant
rs1414169070	intron_variant,genic_upstream_transcript_variant
rs1414187784	intron_variant,genic_upstream_transcript_variant
rs1414192612	intron_variant
rs1414201606	intron_variant
rs1414209879	intron_variant
rs1414244136	intron_variant
rs1414265289	intron_variant,genic_upstream_transcript_variant
rs1414281296	intron_variant
rs1414304044	intron_variant
rs1414312808	intron_variant,genic_downstream_transcript_variant
rs1414314940	intron_variant
rs1414341893	intron_variant,genic_upstream_transcript_variant
rs1414437811	intron_variant
rs1414486907	intron_variant
rs1414509890	intron_variant,genic_upstream_transcript_variant
rs1414517251	intron_variant,genic_upstream_transcript_variant
rs1414553866	intron_variant,genic_upstream_transcript_variant
rs1414565987	intron_variant
rs1414661984	genic_downstream_transcript_variant,non_coding_transcript_variant,3_prime_UTR_variant
rs1414667581	intron_variant
rs1414682710	intron_variant
rs1414776945	genic_downstream_transcript_variant,non_coding_transcript_variant,3_prime_UTR_variant
rs1414785019	intron_variant,genic_upstream_transcript_variant
rs1414787493	intron_variant
rs1414818661	intron_variant,genic_upstream_transcript_variant
rs1414841619	intron_variant
rs1414849742	intron_variant,genic_upstream_transcript_variant
rs1414873885	intron_variant,genic_downstream_transcript_variant
rs1414891437	intron_variant,genic_upstream_transcript_variant
rs1414944239	intron_variant,genic_upstream_transcript_variant
rs1414952126	intron_variant
rs1414976520	downstream_transcript_variant,intron_variant,genic_downstream_transcript_variant
rs1414996029	intron_variant,genic_upstream_transcript_variant
rs1415015816	intron_variant,genic_downstream_transcript_variant
rs1415033985	intron_variant,genic_downstream_transcript_variant
rs1415059851	intron_variant,genic_upstream_transcript_variant
rs1415060644	intron_variant
rs1415126344	intron_variant
rs1415131410	intron_variant
rs1415181720	intron_variant,genic_upstream_transcript_variant
rs1415202298	intron_variant,genic_upstream_transcript_variant
rs1415212740	intron_variant
rs1415240684	intron_variant
rs1415284898	intron_variant,genic_upstream_transcript_variant
rs1415289824	intron_variant
rs1415297092	intron_variant,genic_upstream_transcript_variant
rs1415333678	intron_variant,genic_upstream_transcript_variant
rs1415353248	intron_variant
rs1415419069	intron_variant,genic_downstream_transcript_variant
rs1415422010	intron_variant
rs1415464623	intron_variant
rs1415468628	intron_variant
rs1415469556	intron_variant,genic_upstream_transcript_variant
rs1415493708	intron_variant
rs1415495980	intron_variant,genic_upstream_transcript_variant
rs1415499942	2KB_upstream_variant,intron_variant,genic_upstream_transcript_variant,upstream_transcript_variant

rs1415519001	intron_variant
rs1415521320	intron_variant
rs1415535318	intron_variant
rs1415559854	intron_variant
rs1415579387	genic_downstream_transcript_variant,non_coding_transcript_variant,3_prime_UTR_variant
rs1415593157	intron_variant,genic_upstream_transcript_variant
rs1415676228	intron_variant,genic_upstream_transcript_variant
rs1415684928	intron_variant
rs1415736844	genic_downstream_transcript_variant,non_coding_transcript_variant,3_prime_UTR_variant
rs1415758134	intron_variant
rs1415764094	intron_variant,genic_upstream_transcript_variant,upstream_transcript_variant
rs1415800304	intron_variant,genic_downstream_transcript_variant
rs1415814379	intron_variant,genic_upstream_transcript_variant,upstream_transcript_variant
rs1415832639	intron_variant
rs1415850287	intron_variant
rs1415907396	intron_variant
rs1415911374	intron_variant,genic_upstream_transcript_variant
rs1415917924	intron_variant,genic_downstream_transcript_variant
rs1415968874	intron_variant
rs1416039394	intron_variant,genic_upstream_transcript_variant
rs1416063440	intron_variant,genic_upstream_transcript_variant
rs1416119674	intron_variant
rs1416153919	intron_variant
rs1416163597	intron_variant,genic_upstream_transcript_variant
rs1416195351	intron_variant,genic_upstream_transcript_variant
rs1416195665	intron_variant
rs1416255127	intron_variant,genic_upstream_transcript_variant
rs1416278844	intron_variant,genic_upstream_transcript_variant,upstream_transcript_variant
rs1416331696	intron_variant,genic_downstream_transcript_variant
rs1416338233	intron_variant,genic_upstream_transcript_variant
rs1416381155	intron_variant,genic_downstream_transcript_variant
rs1416396818	intron_variant,genic_downstream_transcript_variant
rs1416405144	intron_variant
rs1416430988	intron_variant,genic_downstream_transcript_variant
rs1416492385	intron_variant,genic_upstream_transcript_variant
rs1416493228	intron_variant,genic_downstream_transcript_variant
rs1416550430	intron_variant,genic_upstream_transcript_variant
rs1416603911	intron_variant,genic_upstream_transcript_variant
rs1416616452	intron_variant
rs1416626197	intron_variant,genic_downstream_transcript_variant
rs1416649025	intron_variant,genic_upstream_transcript_variant
rs1416687412	2KB_upstream_variant,intron_variant,genic_upstream_transcript_variant,upstream_transcript_variant
rs1416737759	intron_variant,genic_downstream_transcript_variant
rs1416771369	intron_variant,genic_downstream_transcript_variant
rs1416796673	intron_variant
rs1416818072	intron_variant,genic_downstream_transcript_variant
rs1416840107	intron_variant
rs1416849925	intron_variant
rs1416864415	intron_variant,genic_downstream_transcript_variant
rs1416896139	intron_variant,genic_upstream_transcript_variant
rs1416902625	intron_variant,genic_downstream_transcript_variant
rs1416923559	intron_variant,genic_downstream_transcript_variant
rs1416926865	intron_variant
rs1416957972	intron_variant
rs1416982830	intron_variant
rs1417000432	intron_variant,genic_upstream_transcript_variant
rs1417002950	intron_variant,genic_downstream_transcript_variant
rs1417010955	intron_variant
rs1417029911	intron_variant
rs1417042360	intron_variant,genic_downstream_transcript_variant
rs1417128991	intron_variant,genic_upstream_transcript_variant
rs1417138027	stop_gained,coding_sequence_variant,intron_variant,genic_upstream_transcript_variant,non_coding_transcript_variant
rs1417141852	intron_variant,genic_upstream_transcript_variant
rs1417153919	intron_variant,genic_downstream_transcript_variant
rs1417157107	intron_variant,genic_upstream_transcript_variant
rs1417185865	intron_variant
rs1417205891	intron_variant
rs1417223181	intron_variant
rs1417240082	intron_variant,genic_upstream_transcript_variant,upstream_transcript_variant
rs1417266255	intron_variant,genic_downstream_transcript_variant
rs1417274697	genic_upstream_transcript_variant,non_coding_transcript_variant,5_prime_UTR_variant,intron_variant
rs1417290378	intron_variant
rs1417328102	intron_variant,genic_downstream_transcript_variant
rs1417404534	intron_variant

rs1417411527	intron_variant
rs1417414218	intron_variant
rs1417415848	intron_variant,genic_upstream_transcript_variant
rs1417419562	intron_variant
rs1417429880	intron_variant,genic_upstream_transcript_variant
rs1417433919	missense_variant,non_coding_transcript_variant,coding_sequence_variant
rs1417443780	intron_variant
rs1417467674	intron_variant,genic_downstream_transcript_variant
rs1417488476	intron_variant,genic_upstream_transcript_variant
rs1417509860	intron_variant
rs1417517849	intron_variant,genic_upstream_transcript_variant,upstream_transcript_variant
rs1417527868	intron_variant,genic_upstream_transcript_variant,upstream_transcript_variant
rs1417567438	intron_variant,genic_upstream_transcript_variant,upstream_transcript_variant
rs1417571863	intron_variant,genic_upstream_transcript_variant
rs1417604262	intron_variant,genic_upstream_transcript_variant
rs1417616796	intron_variant
rs1417635383	intron_variant,genic_upstream_transcript_variant
rs1417667928	intron_variant
rs1417757346	intron_variant,genic_downstream_transcript_variant
rs1417767899	intron_variant
rs1417771839	intron_variant,genic_downstream_transcript_variant
rs1417783618	intron_variant
rs1417814993	intron_variant
rs1417862132	intron_variant
rs1417871928	intron_variant,genic_upstream_transcript_variant
rs1417900862	intron_variant,genic_upstream_transcript_variant
rs1417944268	intron_variant
rs1417953926	intron_variant,genic_downstream_transcript_variant
rs1417975136	missense_variant,non_coding_transcript_variant,coding_sequence_variant
rs1418009551	intron_variant,genic_upstream_transcript_variant
rs1418012091	intron_variant,genic_downstream_transcript_variant
rs1418018774	intron_variant,genic_downstream_transcript_variant
rs1418035767	intron_variant,genic_upstream_transcript_variant
rs1418077355	intron_variant
rs1418138444	intron_variant
rs1418168831	intron_variant
rs1418238539	intron_variant,genic_upstream_transcript_variant
rs1418239233	intron_variant
rs1418262887	intron_variant,genic_downstream_transcript_variant
rs1418275091	intron_variant
rs1418283441	intron_variant,genic_upstream_transcript_variant
rs1418288016	intron_variant,genic_upstream_transcript_variant,upstream_transcript_variant
rs1418345175	intron_variant,genic_upstream_transcript_variant
rs1418351074	intron_variant,genic_upstream_transcript_variant
rs1418362983	intron_variant
rs1418399135	intron_variant,genic_upstream_transcript_variant
rs1418419682	intron_variant,genic_downstream_transcript_variant
rs1418461542	intron_variant,genic_upstream_transcript_variant
rs1418493673	intron_variant,genic_upstream_transcript_variant
rs1418510745	intron_variant,genic_upstream_transcript_variant
rs1418531157	intron_variant,genic_upstream_transcript_variant
rs1418533620	intron_variant,genic_downstream_transcript_variant
rs1418555935	intron_variant,genic_upstream_transcript_variant
rs1418577875	intron_variant,genic_upstream_transcript_variant
rs1418598096	intron_variant,genic_upstream_transcript_variant
rs1418603627	intron_variant
rs1418609772	intron_variant
rs1418620881	intron_variant
rs1418651877	intron_variant,genic_downstream_transcript_variant
rs1418680525	intron_variant
rs1418710509	intron_variant,genic_upstream_transcript_variant
rs1418723360	intron_variant
rs1418767454	intron_variant,genic_upstream_transcript_variant
rs1418795078	intron_variant,genic_downstream_transcript_variant
rs1418803874	intron_variant,genic_upstream_transcript_variant
rs1418808482	intron_variant,genic_upstream_transcript_variant
rs1418834705	intron_variant
rs1418845572	intron_variant
rs1418863217	intron_variant,genic_upstream_transcript_variant
rs1418872940	intron_variant
rs1418885343	intron_variant,genic_upstream_transcript_variant
rs1418960498	intron_variant,genic_upstream_transcript_variant
rs1419026302	intron_variant,genic_upstream_transcript_variant
rs1419026666	intron_variant,genic_upstream_transcript_variant

rs1419027249	intron_variant,genic_downstream_transcript_variant
rs1419084654	intron_variant,genic_upstream_transcript_variant
rs1419096620	intron_variant
rs1419119600	intron_variant,genic_upstream_transcript_variant
rs1419137122	intron_variant
rs1419160204	intron_variant
rs1419168165	intron_variant
rs1419168836	intron_variant,genic_upstream_transcript_variant
rs1419204302	intron_variant
rs1419221783	intron_variant,genic_upstream_transcript_variant
rs1419243895	intron_variant,genic_upstream_transcript_variant
rs1419255057	intron_variant
rs1419287447	intron_variant
rs1419331097	intron_variant,genic_upstream_transcript_variant
rs1419361329	intron_variant,genic_upstream_transcript_variant
rs1419368770	intron_variant
rs1419375528	intron_variant
rs1419398628	intron_variant,genic_downstream_transcript_variant
rs1419410377	intron_variant
rs1419458980	intron_variant,genic_upstream_transcript_variant
rs1419522549	intron_variant
rs1419577403	intron_variant
rs1419596550	intron_variant,genic_downstream_transcript_variant
rs1419597462	intron_variant
rs1419601909	intron_variant
rs1419618357	intron_variant,genic_downstream_transcript_variant
rs1419651726	intron_variant,genic_upstream_transcript_variant
rs1419669081	intron_variant,genic_upstream_transcript_variant
rs1419705812	intron_variant,genic_upstream_transcript_variant
rs1419794459	intron_variant
rs1419813849	intron_variant
rs1419818689	intron_variant,genic_upstream_transcript_variant
rs1419868871	intron_variant
rs1419913735	intron_variant,genic_upstream_transcript_variant
rs1419921839	intron_variant,genic_downstream_transcript_variant
rs1419922081	intron_variant
rs1419978372	intron_variant,genic_upstream_transcript_variant
rs1419991097	intron_variant,genic_downstream_transcript_variant
rs1419994293	intron_variant
rs1420029545	intron_variant
rs1420051245	intron_variant
rs1420064646	intron_variant
rs1420084269	intron_variant,genic_upstream_transcript_variant
rs1420111639	intron_variant,genic_upstream_transcript_variant
rs1420122499	intron_variant,genic_downstream_transcript_variant
rs1420174204	intron_variant,genic_upstream_transcript_variant
rs1420178599	intron_variant,genic_downstream_transcript_variant
rs1420201065	intron_variant,genic_downstream_transcript_variant
rs1420220785	intron_variant
rs1420240934	missense_variant,non_coding_transcript_variant,genic_downstream_transcript_variant,coding_sequence_variant
rs1420250296	intron_variant,genic_upstream_transcript_variant
rs1420317257	intron_variant,genic_upstream_transcript_variant
rs1420370492	2KB_upstream_variant,intron_variant,genic_upstream_transcript_variant,upstream_transcript_variant
rs1420442950	intron_variant,genic_upstream_transcript_variant
rs1420479342	intron_variant,genic_upstream_transcript_variant
rs1420519819	intron_variant,genic_downstream_transcript_variant
rs1420538067	intron_variant
rs1420550735	intron_variant,genic_upstream_transcript_variant
rs1420555136	intron_variant
rs1420562525	intron_variant,genic_upstream_transcript_variant
rs1420569440	intron_variant
rs1420586253	intron_variant,genic_upstream_transcript_variant
rs1420587961	intron_variant
rs1420595847	intron_variant,genic_upstream_transcript_variant
rs1420649725	coding_sequence_variant,intron_variant,genic_upstream_transcript_variant,non_coding_transcript_variant,frameshift_variant
rs1420667736	intron_variant
rs1420676728	intron_variant,genic_upstream_transcript_variant
rs1420685246	intron_variant
rs1420685394	intron_variant,genic_upstream_transcript_variant
rs1420738047	intron_variant,genic_upstream_transcript_variant
rs1420749689	intron_variant
rs1420776400	intron_variant,genic_upstream_transcript_variant
rs1420821729	intron_variant,genic_downstream_transcript_variant
rs1420840589	intron_variant,genic_upstream_transcript_variant,upstream_transcript_variant

rs1420851997	intron_variant,genic_upstream_transcript_variant
rs1420875221	intron_variant
rs1420885936	intron_variant
rs1420890847	intron_variant,genic_downstream_transcript_variant
rs1420917514	intron_variant
rs1420930337	intron_variant
rs1420935255	intron_variant,genic_upstream_transcript_variant
rs1420950572	intron_variant,genic_upstream_transcript_variant
rs1420950882	intron_variant,genic_downstream_transcript_variant
rs1420975433	intron_variant,genic_upstream_transcript_variant
rs1420979872	intron_variant,genic_downstream_transcript_variant
rs1421027559	intron_variant,genic_downstream_transcript_variant
rs1421028269	genic_downstream_transcript_variant,non_coding_transcript_variant,3_prime_UTR_variant
rs1421043791	intron_variant,genic_downstream_transcript_variant
rs1421059305	intron_variant,genic_downstream_transcript_variant
rs1421102466	intron_variant,genic_upstream_transcript_variant
rs1421136204	intron_variant,genic_upstream_transcript_variant
rs1421189692	intron_variant
rs1421224397	intron_variant,genic_upstream_transcript_variant,upstream_transcript_variant
rs1421232917	intron_variant,genic_downstream_transcript_variant
rs1421237443	2KB_upstream_variant,intron_variant,genic_upstream_transcript_variant,upstream_transcript_variant
rs1421243179	genic_downstream_transcript_variant,non_coding_transcript_variant,3_prime_UTR_variant
rs1421259979	intron_variant
rs1421290095	intron_variant,genic_downstream_transcript_variant
rs1421298994	intron_variant,genic_upstream_transcript_variant
rs1421300666	intron_variant
rs1421300978	intron_variant,genic_upstream_transcript_variant
rs1421337303	intron_variant,genic_upstream_transcript_variant
rs1421345469	intron_variant,genic_downstream_transcript_variant
rs1421453556	intron_variant,genic_upstream_transcript_variant
rs1421455633	intron_variant
rs1421461518	intron_variant
rs1421468883	2KB_upstream_variant,intron_variant,genic_upstream_transcript_variant,upstream_transcript_variant
rs1421571216	intron_variant,genic_upstream_transcript_variant,upstream_transcript_variant
rs1421571748	intron_variant,genic_upstream_transcript_variant
rs1421597231	intron_variant,genic_upstream_transcript_variant
rs1421598688	intron_variant,genic_downstream_transcript_variant
rs1421652355	intron_variant,genic_upstream_transcript_variant
rs1421661336	intron_variant,genic_downstream_transcript_variant
rs1421718195	intron_variant,genic_upstream_transcript_variant
rs1421766875	intron_variant,genic_upstream_transcript_variant
rs1421774051	intron_variant,genic_upstream_transcript_variant
rs1421811939	intron_variant,genic_upstream_transcript_variant,upstream_transcript_variant
rs1421818782	intron_variant
rs1421828891	intron_variant,genic_upstream_transcript_variant
rs1421868121	intron_variant,genic_upstream_transcript_variant,upstream_transcript_variant
rs1421880147	intron_variant
rs1421897564	intron_variant,genic_upstream_transcript_variant
rs1421921787	genic_upstream_transcript_variant,intron_variant
rs1421928126	genic_upstream_transcript_variant,intron_variant
rs1421939985	genic_downstream_transcript_variant,intron_variant
rs1421944473	genic_upstream_transcript_variant,intron_variant
rs1421980959	intron_variant
rs1421989477	genic_upstream_transcript_variant,intron_variant
rs1422016142	genic_upstream_transcript_variant,intron_variant
rs1422029911	intron_variant
rs1422036465	genic_downstream_transcript_variant,intron_variant
rs1422037755	intron_variant
rs1422046181	genic_upstream_transcript_variant,intron_variant
rs1422047757	intron_variant
rs1422062331	genic_upstream_transcript_variant,intron_variant
rs1422102640	genic_downstream_transcript_variant,intron_variant
rs1422124075	genic_upstream_transcript_variant,intron_variant
rs1422160320	genic_upstream_transcript_variant,intron_variant
rs1422185735	intron_variant
rs1422193544	genic_upstream_transcript_variant,intron_variant
rs1422208527	intron_variant
rs1422229702	genic_upstream_transcript_variant,upstream_transcript_variant,2KB_upstream_variant,intron_variant
rs1422234081	genic_upstream_transcript_variant,intron_variant
rs1422278455	genic_upstream_transcript_variant,intron_variant
rs1422321062	genic_upstream_transcript_variant,upstream_transcript_variant,2KB_upstream_variant,intron_variant
rs1422359365	intron_variant
rs1422375699	downstream_transcript_variant,genic_downstream_transcript_variant,intron_variant
rs1422392149	genic_upstream_transcript_variant,intron_variant

rs1422414887	genic_upstream_transcript_variant,intron_variant
rs1422463275	genic_upstream_transcript_variant,intron_variant
rs1422469241	intron_variant
rs1422486932	genic_upstream_transcript_variant,intron_variant
rs1422540708	intron_variant
rs1422586440	genic_upstream_transcript_variant,upstream_transcript_variant,2KB_upstream_variant,intron_variant
rs1422639311	intron_variant
rs1422659577	genic_upstream_transcript_variant,intron_variant
rs1422700090	genic_upstream_transcript_variant,5_prime_UTR_variant,non_coding_transcript_variant,intron_variant
rs1422718497	genic_upstream_transcript_variant,intron_variant
rs1422722057	intron_variant
rs1422749892	intron_variant
rs1422752731	genic_upstream_transcript_variant,intron_variant
rs1422791220	genic_downstream_transcript_variant,intron_variant
rs1422820847	downstream_transcript_variant,500B_downstream_variant
rs1422825655	intron_variant
rs1422853030	genic_upstream_transcript_variant,upstream_transcript_variant,2KB_upstream_variant,intron_variant
rs1422865395	genic_upstream_transcript_variant,intron_variant
rs1422885739	intron_variant
rs1422918531	intron_variant
rs1422933094	intron_variant
rs1422966258	coding_sequence_variant,synonymous_variant,genic_downstream_transcript_variant,non_coding_transcript_variant
rs1422973668	genic_upstream_transcript_variant,upstream_transcript_variant,2KB_upstream_variant,intron_variant
rs1423043295	genic_upstream_transcript_variant,intron_variant
rs1423052070	genic_upstream_transcript_variant,intron_variant
rs1423151923	intron_variant
rs1423267991	genic_downstream_transcript_variant,intron_variant
rs1423270706	intron_variant
rs1423310746	intron_variant
rs1423340324	genic_downstream_transcript_variant,intron_variant
rs1423372708	genic_upstream_transcript_variant,5_prime_UTR_variant,non_coding_transcript_variant,intron_variant
rs1423379004	genic_downstream_transcript_variant,intron_variant
rs1423396047	genic_upstream_transcript_variant,intron_variant
rs1423406383	genic_upstream_transcript_variant,intron_variant
rs1423408175	intron_variant
rs1423425645	intron_variant
rs1423429918	genic_downstream_transcript_variant,intron_variant
rs1423465410	genic_upstream_transcript_variant,intron_variant
rs1423536772	intron_variant
rs1423553466	intron_variant
rs1423577251	intron_variant
rs1423610031	intron_variant
rs1423631417	intron_variant
rs1423679133	genic_upstream_transcript_variant,upstream_transcript_variant,intron_variant
rs1423684140	genic_upstream_transcript_variant,upstream_transcript_variant,2KB_upstream_variant,intron_variant
rs1423699789	coding_sequence_variant,missense_variant,genic_downstream_transcript_variant,non_coding_transcript_variant
rs1423723960	intron_variant
rs1423764257	intron_variant
rs1423803709	genic_upstream_transcript_variant,intron_variant
rs1423856263	intron_variant
rs1423881085	genic_downstream_transcript_variant,non_coding_transcript_variant,3_prime_UTR_variant
rs1423908022	genic_upstream_transcript_variant,intron_variant
rs1423924503	intron_variant
rs1423935763	genic_downstream_transcript_variant,intron_variant
rs1424015283	genic_downstream_transcript_variant,intron_variant
rs1424041665	genic_downstream_transcript_variant,intron_variant
rs1424055148	intron_variant
rs1424109025	intron_variant
rs1424136579	genic_downstream_transcript_variant,intron_variant
rs1424157003	genic_downstream_transcript_variant,intron_variant
rs1424162180	intron_variant
rs1424232690	genic_upstream_transcript_variant,intron_variant
rs1424279963	genic_downstream_transcript_variant,intron_variant
rs1424317470	genic_upstream_transcript_variant,upstream_transcript_variant,intron_variant
rs1424351823	genic_upstream_transcript_variant,intron_variant
rs1424370270	intron_variant
rs1424395698	genic_upstream_transcript_variant,intron_variant
rs1424400050	genic_upstream_transcript_variant,intron_variant
rs1424402480	genic_upstream_transcript_variant,intron_variant
rs1424420830	coding_sequence_variant,synonymous_variant,non_coding_transcript_variant
rs1424426229	genic_upstream_transcript_variant,upstream_transcript_variant,intron_variant
rs1424428516	intron_variant
rs1424447851	genic_downstream_transcript_variant,intron_variant
rs1424455189	genic_downstream_transcript_variant,intron_variant

rs1424473948	genic_downstream_transcript_variant,intron_variant
rs1424508319	genic_upstream_transcript_variant,intron_variant
rs1424516651	intron_variant
rs1424517036	genic_upstream_transcript_variant,intron_variant
rs1424563606	intron_variant
rs1424567821	coding_sequence_variant,synonymous_variant,non_coding_transcript_variant
rs1424569066	intron_variant
rs1424601648	genic_upstream_transcript_variant,intron_variant
rs1424615590	intron_variant
rs1424627981	intron_variant
rs1424632016	intron_variant
rs1424660115	intron_variant
rs1424681622	genic_downstream_transcript_variant,intron_variant
rs1424713187	intron_variant
rs1424731779	genic_upstream_transcript_variant,intron_variant
rs1424745032	genic_downstream_transcript_variant,non_coding_transcript_variant,3_prime_UTR_variant
rs1424746837	intron_variant
rs1424850440	genic_downstream_transcript_variant,intron_variant
rs1424851494	intron_variant
rs1424874459	intron_variant
rs1424911152	genic_upstream_transcript_variant,intron_variant
rs1424919208	genic_downstream_transcript_variant,intron_variant
rs1424939179	downstream_transcript_variant,500B_downstream_variant
rs1424953403	genic_upstream_transcript_variant,intron_variant
rs1424956700	intron_variant
rs1424956927	genic_downstream_transcript_variant,intron_variant
rs1424967818	genic_downstream_transcript_variant,non_coding_transcript_variant,3_prime_UTR_variant
rs1424996653	genic_upstream_transcript_variant,intron_variant
rs1424997203	intron_variant
rs1425054192	genic_upstream_transcript_variant,intron_variant
rs1425071186	5_prime_UTR_variant,intron_variant
rs1425078815	genic_upstream_transcript_variant,intron_variant
rs1425078851	intron_variant
rs1425084051	coding_sequence_variant,synonymous_variant,non_coding_transcript_variant
rs1425098169	intron_variant
rs1425157972	genic_upstream_transcript_variant,intron_variant
rs1425161140	genic_downstream_transcript_variant,intron_variant
rs1425173302	intron_variant
rs1425177681	genic_upstream_transcript_variant,intron_variant
rs1425183711	genic_downstream_transcript_variant,non_coding_transcript_variant,3_prime_UTR_variant
rs1425213675	intron_variant
rs1425232145	genic_downstream_transcript_variant,intron_variant
rs1425256990	genic_upstream_transcript_variant,intron_variant
rs1425296429	genic_downstream_transcript_variant,non_coding_transcript_variant,3_prime_UTR_variant
rs1425344967	genic_upstream_transcript_variant,intron_variant
rs1425347698	intron_variant
rs1425360911	intron_variant
rs1425371623	genic_upstream_transcript_variant,intron_variant
rs1425389056	genic_downstream_transcript_variant,splice_donor_variant
rs1425417534	genic_upstream_transcript_variant,upstream_transcript_variant,2KB_upstream_variant,intron_variant
rs1425433171	genic_upstream_transcript_variant,intron_variant
rs1425468795	intron_variant
rs1425473151	genic_downstream_transcript_variant,intron_variant
rs1425485583	intron_variant
rs1425512798	genic_downstream_transcript_variant,intron_variant
rs1425514359	intron_variant
rs1425514760	genic_upstream_transcript_variant,intron_variant
rs1425540494	intron_variant
rs1425587628	intron_variant
rs1425611692	genic_upstream_transcript_variant,intron_variant
rs1425619832	intron_variant
rs1425631297	genic_upstream_transcript_variant,intron_variant
rs1425636516	intron_variant
rs1425669712	genic_upstream_transcript_variant,intron_variant
rs1425691673	genic_upstream_transcript_variant,intron_variant
rs1425695998	genic_upstream_transcript_variant,intron_variant
rs1425701220	genic_downstream_transcript_variant,intron_variant
rs1425719355	genic_upstream_transcript_variant,intron_variant
rs1425738644	genic_upstream_transcript_variant,intron_variant
rs1425894686	intron_variant
rs1425902060	genic_upstream_transcript_variant,intron_variant
rs1425921810	genic_upstream_transcript_variant,intron_variant
rs1425939385	genic_upstream_transcript_variant,intron_variant
rs1425950145	genic_upstream_transcript_variant,intron_variant

rs1425956326	genic_upstream_transcript_variant,intron_variant
rs1426017203	genic_upstream_transcript_variant,upstream_transcript_variant,2KB_upstream_variant,intron_variant
rs1426039432	intron_variant
rs1426051548	genic_downstream_transcript_variant,intron_variant
rs1426096893	intron_variant
rs1426102387	intron_variant
rs1426132123	genic_upstream_transcript_variant,intron_variant
rs1426134519	genic_upstream_transcript_variant,upstream_transcript_variant,2KB_upstream_variant,intron_variant
rs1426135286	genic_upstream_transcript_variant,intron_variant
rs1426164750	intron_variant
rs1426177924	intron_variant
rs1426224427	genic_downstream_transcript_variant,intron_variant
rs1426253787	intron_variant
rs1426299544	intron_variant
rs1426323596	genic_downstream_transcript_variant,intron_variant
rs1426327621	genic_upstream_transcript_variant,intron_variant
rs1426334478	genic_downstream_transcript_variant,intron_variant
rs1426363944	intron_variant
rs1426422026	intron_variant
rs1426435984	intron_variant
rs1426445660	genic_downstream_transcript_variant,intron_variant
rs1426452513	genic_upstream_transcript_variant,5_prime_UTR_variant,non_coding_transcript_variant,intron_variant
rs1426453605	genic_downstream_transcript_variant,intron_variant
rs1426454117	intron_variant
rs1426575875	genic_upstream_transcript_variant,intron_variant
rs1426603349	genic_upstream_transcript_variant,intron_variant
rs1426640689	genic_upstream_transcript_variant,intron_variant
rs1426680947	genic_downstream_transcript_variant,intron_variant
rs1426681604	intron_variant
rs1426683856	genic_downstream_transcript_variant,intron_variant
rs1426713575	genic_upstream_transcript_variant,intron_variant
rs1426729399	genic_upstream_transcript_variant,intron_variant
rs1426807525	intron_variant
rs1426808306	genic_downstream_transcript_variant,non_coding_transcript_variant,3_prime_UTR_variant
rs1426863619	genic_upstream_transcript_variant,intron_variant
rs1426870666	genic_upstream_transcript_variant,upstream_transcript_variant,2KB_upstream_variant,intron_variant
rs1426917904	intron_variant
rs1426940620	genic_upstream_transcript_variant,intron_variant
rs1426951783	intron_variant
rs1426960183	genic_upstream_transcript_variant,intron_variant
rs1426989076	genic_upstream_transcript_variant,intron_variant
rs1426996276	intron_variant
rs1427003410	intron_variant
rs1427015348	genic_upstream_transcript_variant,intron_variant
rs1427017943	intron_variant
rs1427023783	genic_upstream_transcript_variant,intron_variant
rs1427039058	genic_upstream_transcript_variant,intron_variant
rs1427051207	coding_sequence_variant,missense_variant,non_coding_transcript_variant
rs1427060717	genic_downstream_transcript_variant,intron_variant
rs1427064585	intron_variant
rs1427094129	intron_variant
rs1427148191	genic_downstream_transcript_variant,intron_variant
rs1427153432	genic_upstream_transcript_variant,intron_variant
rs1427165500	intron_variant
rs1427183385	genic_upstream_transcript_variant,intron_variant
rs1427218909	intron_variant
rs1427223521	genic_upstream_transcript_variant,intron_variant
rs1427239676	coding_sequence_variant,missense_variant,non_coding_transcript_variant
rs1427267429	genic_upstream_transcript_variant,upstream_transcript_variant,intron_variant
rs1427287929	genic_upstream_transcript_variant,intron_variant
rs1427309623	genic_downstream_transcript_variant,intron_variant
rs1427373132	genic_upstream_transcript_variant,intron_variant
rs1427395776	genic_upstream_transcript_variant,intron_variant
rs1427404223	genic_upstream_transcript_variant,intron_variant
rs1427489606	genic_upstream_transcript_variant,intron_variant
rs1427564702	intron_variant
rs1427687115	intron_variant
rs1427715778	genic_upstream_transcript_variant,intron_variant
rs1427717143	intron_variant
rs1427749123	genic_upstream_transcript_variant,intron_variant
rs1427793414	intron_variant
rs1427834943	genic_upstream_transcript_variant,intron_variant
rs1427848823	genic_upstream_transcript_variant,intron_variant
rs1427883520	intron_variant

rs1427891995	genic_upstream_transcript_variant,intron_variant
rs1427912768	genic_downstream_transcript_variant,intron_variant
rs1427951492	genic_upstream_transcript_variant,intron_variant
rs1427990010	genic_upstream_transcript_variant,intron_variant
rs1427992367	genic_upstream_transcript_variant,intron_variant
rs1427996719	intron_variant
rs1428042601	genic_upstream_transcript_variant,intron_variant
rs1428051614	genic_upstream_transcript_variant,intron_variant
rs1428062120	genic_downstream_transcript_variant,intron_variant
rs1428065826	intron_variant
rs1428089393	genic_downstream_transcript_variant,intron_variant
rs1428090710	genic_upstream_transcript_variant,intron_variant
rs1428091730	genic_upstream_transcript_variant,intron_variant
rs1428094597	intron_variant
rs1428175627	intron_variant
rs1428199094	intron_variant
rs1428199845	genic_upstream_transcript_variant,intron_variant
rs1428211011	genic_upstream_transcript_variant,intron_variant
rs1428258959	genic_upstream_transcript_variant,intron_variant
rs1428279685	genic_upstream_transcript_variant,intron_variant
rs1428289208	genic_upstream_transcript_variant,intron_variant
rs1428414746	genic_upstream_transcript_variant,intron_variant
rs1428428166	intron_variant
rs1428428504	genic_upstream_transcript_variant,intron_variant
rs1428439255	intron_variant
rs1428457234	genic_upstream_transcript_variant,intron_variant
rs1428490730	intron_variant
rs1428523797	intron_variant
rs1428537533	genic_upstream_transcript_variant,intron_variant
rs1428629301	genic_downstream_transcript_variant,intron_variant
rs1428633135	genic_upstream_transcript_variant,intron_variant
rs1428654191	intron_variant
rs1428681889	genic_upstream_transcript_variant,intron_variant
rs1428717336	genic_downstream_transcript_variant,intron_variant
rs1428744596	genic_upstream_transcript_variant,intron_variant
rs1428777432	intron_variant
rs1428785637	genic_upstream_transcript_variant,intron_variant
rs1428791579	genic_upstream_transcript_variant,upstream_transcript_variant,2KB_upstream_variant,intron_variant
rs1428792722	genic_downstream_transcript_variant,intron_variant
rs1428866401	genic_upstream_transcript_variant,intron_variant
rs1428873877	genic_upstream_transcript_variant,intron_variant
rs1428878376	intron_variant
rs1428884788	genic_downstream_transcript_variant,intron_variant
rs1428885379	genic_downstream_transcript_variant,intron_variant
rs1428916155	intron_variant
rs1428918859	genic_upstream_transcript_variant,intron_variant
rs1428926640	genic_downstream_transcript_variant,non_coding_transcript_variant,3_prime_UTR_variant
rs1428965297	genic_upstream_transcript_variant,intron_variant
rs1428996841	intron_variant
rs1428998113	genic_upstream_transcript_variant,intron_variant
rs1428999050	genic_downstream_transcript_variant,intron_variant
rs1429090220	genic_downstream_transcript_variant,intron_variant
rs1429098348	intron_variant
rs1429100216	intron_variant
rs1429147596	downstream_transcript_variant,genic_downstream_transcript_variant,intron_variant
rs1429154398	genic_upstream_transcript_variant,intron_variant
rs1429219082	intron_variant
rs1429240484	genic_downstream_transcript_variant,intron_variant
rs1429304283	genic_upstream_transcript_variant,intron_variant
rs1429312825	intron_variant
rs1429327764	genic_upstream_transcript_variant,intron_variant
rs1429360492	intron_variant
rs1429361957	intron_variant
rs1429395225	genic_downstream_transcript_variant,intron_variant
rs1429407600	genic_upstream_transcript_variant,intron_variant
rs1429414605	5_prime_UTR_variant,intron_variant
rs1429445746	intron_variant
rs1429484639	genic_downstream_transcript_variant,intron_variant
rs1429527617	intron_variant
rs1429581340	intron_variant
rs1429593043	intron_variant
rs1429613195	genic_downstream_transcript_variant,intron_variant
rs1429682005	genic_upstream_transcript_variant,intron_variant
rs1429686802	genic_upstream_transcript_variant,intron_variant

rs1429690128	genic_upstream_transcript_variant,intron_variant
rs1429694270	genic_upstream_transcript_variant,intron_variant
rs1429701261	genic_upstream_transcript_variant,intron_variant
rs1429712436	intron_variant
rs1429728612	genic_upstream_transcript_variant,intron_variant
rs1429735929	genic_upstream_transcript_variant,intron_variant
rs1429743118	intron_variant
rs1429750625	genic_upstream_transcript_variant,intron_variant
rs1429754742	intron_variant
rs1429777376	intron_variant
rs1429777881	genic_downstream_transcript_variant,intron_variant
rs1429801044	intron_variant
rs1429840711	intron_variant
rs1429876848	intron_variant
rs1429915038	genic_downstream_transcript_variant,intron_variant
rs1429952049	intron_variant
rs1429974687	intron_variant
rs1430004750	genic_downstream_transcript_variant,intron_variant
rs1430034857	intron_variant
rs1430047058	genic_upstream_transcript_variant,intron_variant
rs1430076534	genic_upstream_transcript_variant,intron_variant
rs1430116275	5_prime_UTR_variant,intron_variant
rs1430124406	genic_downstream_transcript_variant,intron_variant
rs1430170795	genic_upstream_transcript_variant,intron_variant
rs1430187255	intron_variant
rs1430219666	intron_variant
rs1430227714	intron_variant
rs1430246761	genic_upstream_transcript_variant,intron_variant
rs1430264533	genic_downstream_transcript_variant,intron_variant
rs1430284792	intron_variant
rs1430294176	genic_downstream_transcript_variant,intron_variant
rs1430371443	genic_upstream_transcript_variant,intron_variant
rs1430379388	intron_variant
rs1430424200	genic_upstream_transcript_variant,intron_variant
rs1430477113	intron_variant
rs1430481480	genic_downstream_transcript_variant,non_coding_transcript_variant,3_prime_UTR_variant
rs1430483205	intron_variant
rs1430496165	genic_upstream_transcript_variant,upstream_transcript_variant,intron_variant
rs1430500690	intron_variant
rs1430505386	genic_downstream_transcript_variant,intron_variant
rs1430525432	genic_downstream_transcript_variant,intron_variant
rs1430582650	genic_downstream_transcript_variant,intron_variant
rs1430640070	genic_downstream_transcript_variant,intron_variant
rs1430661132	intron_variant
rs1430698695	genic_downstream_transcript_variant,non_coding_transcript_variant,3_prime_UTR_variant
rs1430720632	genic_downstream_transcript_variant,intron_variant
rs1430726033	genic_upstream_transcript_variant,non_coding_transcript_variant,coding_sequence_variant,synonymous_variant,intron_variant
rs1430782500	genic_upstream_transcript_variant,upstream_transcript_variant,2KB_upstream_variant,intron_variant
rs1430816522	intron_variant
rs1430828427	intron_variant
rs1430833139	intron_variant
rs1430867292	genic_downstream_transcript_variant,intron_variant
rs1430882194	genic_downstream_transcript_variant,intron_variant
rs1430891140	intron_variant
rs1430924636	genic_downstream_transcript_variant,intron_variant
rs1430944198	intron_variant
rs1430970282	genic_upstream_transcript_variant,intron_variant
rs1430978343	genic_downstream_transcript_variant,intron_variant
rs1430982560	genic_downstream_transcript_variant,intron_variant
rs1431019652	genic_downstream_transcript_variant,intron_variant
rs1431068760	genic_upstream_transcript_variant,intron_variant
rs1431107005	genic_upstream_transcript_variant,intron_variant
rs1431136569	genic_downstream_transcript_variant,intron_variant
rs1431172313	genic_upstream_transcript_variant,intron_variant
rs1431181347	genic_upstream_transcript_variant,upstream_transcript_variant,2KB_upstream_variant,intron_variant
rs1431226697	genic_upstream_transcript_variant,intron_variant
rs1431252742	genic_upstream_transcript_variant,intron_variant
rs1431271646	genic_downstream_transcript_variant,intron_variant
rs1431333870	genic_upstream_transcript_variant,intron_variant
rs1431343572	intron_variant
rs1431415059	genic_downstream_transcript_variant,intron_variant
rs1431448460	genic_upstream_transcript_variant,intron_variant
rs1431482280	coding_sequence_variant,missense_variant,non_coding_transcript_variant
rs1431487293	genic_upstream_transcript_variant,intron_variant

rs1431488064	genic_downstream_transcript_variant,intron_variant
rs1431616521	intron_variant
rs1431683899	coding_sequence_variant,missense_variant,genic_downstream_transcript_variant,non_coding_transcript_variant
rs1431684129	intron_variant
rs1431701159	genic_upstream_transcript_variant,intron_variant
rs1431732603	genic_upstream_transcript_variant,upstream_transcript_variant,intron_variant
rs1431751725	intron_variant
rs1431764584	intron_variant
rs1431785150	genic_upstream_transcript_variant,intron_variant
rs1431833042	genic_upstream_transcript_variant,intron_variant
rs1431918006	genic_downstream_transcript_variant,intron_variant
rs1431986735	genic_upstream_transcript_variant,intron_variant
rs1432045313	intron_variant
rs1432057531	genic_downstream_transcript_variant,non_coding_transcript_variant,3_prime_UTR_variant
rs1432099602	genic_upstream_transcript_variant,intron_variant
rs1432139158	genic_downstream_transcript_variant,intron_variant
rs1432224890	genic_downstream_transcript_variant,intron_variant
rs1432258525	intron_variant
rs1432276025	genic_downstream_transcript_variant,intron_variant
rs1432314969	genic_upstream_transcript_variant,intron_variant
rs1432382362	genic_downstream_transcript_variant,intron_variant
rs1432419896	intron_variant
rs1432469613	genic_upstream_transcript_variant,intron_variant
rs1432477966	intron_variant
rs1432479365	genic_upstream_transcript_variant,intron_variant
rs1432581826	genic_downstream_transcript_variant,intron_variant
rs1432597172	genic_downstream_transcript_variant,intron_variant
rs1432720848	intron_variant
rs1432750820	intron_variant
rs1432759228	intron_variant
rs1432782215	intron_variant
rs1432789061	intron_variant
rs1432798894	genic_upstream_transcript_variant,intron_variant
rs1432802260	genic_upstream_transcript_variant,intron_variant
rs1432834620	genic_downstream_transcript_variant,intron_variant
rs1432841316	genic_upstream_transcript_variant,intron_variant
rs1432854351	genic_upstream_transcript_variant,intron_variant
rs1432863997	genic_upstream_transcript_variant,intron_variant
rs1432931968	intron_variant
rs1432943392	intron_variant
rs1432947073	genic_upstream_transcript_variant,intron_variant
rs1432971085	intron_variant
rs1432977273	genic_upstream_transcript_variant,non_coding_transcript_variant,coding_sequence_variant,synonymous_variant,intron_variant
rs1432983845	genic_downstream_transcript_variant,non_coding_transcript_variant,3_prime_UTR_variant
rs1433019092	genic_upstream_transcript_variant,intron_variant
rs1433029332	intron_variant
rs1433097066	genic_downstream_transcript_variant,non_coding_transcript_variant,3_prime_UTR_variant
rs1433112753	genic_upstream_transcript_variant,intron_variant
rs1433173146	genic_upstream_transcript_variant,intron_variant
rs1433239747	genic_upstream_transcript_variant,intron_variant
rs1433249274	genic_upstream_transcript_variant,intron_variant
rs1433255882	genic_upstream_transcript_variant,intron_variant
rs1433275705	genic_upstream_transcript_variant,intron_variant
rs1433311001	genic_upstream_transcript_variant,intron_variant
rs1433324747	genic_upstream_transcript_variant,intron_variant
rs1433379712	intron_variant
rs1433409607	genic_downstream_transcript_variant,intron_variant
rs1433417502	genic_upstream_transcript_variant,upstream_transcript_variant,2KB_upstream_variant,intron_variant
rs1433429704	intron_variant
rs1433460756	intron_variant
rs1433483763	coding_sequence_variant,synonymous_variant,non_coding_transcript_variant
rs1433545256	genic_upstream_transcript_variant,upstream_transcript_variant,2KB_upstream_variant,intron_variant
rs1433555166	genic_upstream_transcript_variant,intron_variant
rs1433575702	genic_upstream_transcript_variant,upstream_transcript_variant,intron_variant
rs1433581161	intron_variant
rs1433586437	genic_downstream_transcript_variant,intron_variant
rs1433613909	genic_upstream_transcript_variant,intron_variant
rs1433658806	intron_variant
rs1433662300	intron_variant
rs1433702089	genic_upstream_transcript_variant,non_coding_transcript_variant,coding_sequence_variant,synonymous_variant,intron_variant
rs1433727901	intron_variant
rs1433746577	5_prime_UTR_variant,intron_variant
rs1433787524	intron_variant
rs1433796696	genic_downstream_transcript_variant,intron_variant

rs1433913527	genic_downstream_transcript_variant,intron_variant
rs1433921987	intron_variant
rs1433979717	intron_variant
rs1433989329	genic_downstream_transcript_variant,intron_variant
rs1434020791	genic_upstream_transcript_variant,upstream_transcript_variant,intron_variant
rs1434058198	intron_variant
rs1434074032	genic_downstream_transcript_variant,intron_variant
rs1434074310	intron_variant
rs1434134253	intron_variant
rs1434138487	genic_upstream_transcript_variant,intron_variant
rs1434193803	intron_variant
rs1434197998	intron_variant
rs1434237673	genic_downstream_transcript_variant,intron_variant
rs1434248114	coding_sequence_variant,missense_variant,genic_downstream_transcript_variant,non_coding_transcript_variant
rs1434262092	genic_downstream_transcript_variant,intron_variant
rs1434279870	intron_variant
rs1434283880	genic_downstream_transcript_variant,intron_variant
rs1434320249	intron_variant
rs1434345427	intron_variant
rs1434433577	genic_upstream_transcript_variant,upstream_transcript_variant,intron_variant
rs1434438828	genic_downstream_transcript_variant,intron_variant
rs1434486821	genic_upstream_transcript_variant,upstream_transcript_variant,intron_variant
rs1434580290	intron_variant,genic_downstream_transcript_variant
rs1434588278	intron_variant,genic_upstream_transcript_variant
rs1434602666	intron_variant
rs1434605030	intron_variant,genic_upstream_transcript_variant
rs1434614334	intron_variant
rs1434660458	intron_variant,genic_downstream_transcript_variant
rs1434680090	intron_variant
rs1434698879	intron_variant
rs1434744538	intron_variant
rs1434805683	intron_variant,genic_upstream_transcript_variant,2KB_upstream_variant,upstream_transcript_variant
rs1434851727	intron_variant,genic_downstream_transcript_variant
rs1434861014	intron_variant
rs1434896991	intron_variant
rs1434930087	intron_variant,genic_upstream_transcript_variant
rs1434970057	intron_variant
rs1434974815	intron_variant,genic_downstream_transcript_variant
rs1434979740	intron_variant
rs1435075239	intron_variant
rs1435104476	intron_variant,genic_upstream_transcript_variant
rs1435107106	intron_variant
rs1435124766	intron_variant
rs1435134493	intron_variant
rs1435146982	intron_variant,genic_upstream_transcript_variant
rs1435162492	intron_variant
rs1435187597	intron_variant,genic_upstream_transcript_variant
rs1435210043	intron_variant
rs1435274497	intron_variant,genic_upstream_transcript_variant
rs1435293339	intron_variant,genic_upstream_transcript_variant
rs1435338452	intron_variant,genic_upstream_transcript_variant
rs1435360462	intron_variant
rs1435397103	intron_variant,genic_upstream_transcript_variant,upstream_transcript_variant
rs1435406118	intron_variant
rs1435432761	intron_variant,genic_upstream_transcript_variant
rs1435486183	intron_variant,genic_upstream_transcript_variant,upstream_transcript_variant
rs1435510422	intron_variant,genic_upstream_transcript_variant
rs1435539302	intron_variant,genic_upstream_transcript_variant
rs1435541899	intron_variant,genic_upstream_transcript_variant
rs1435570388	intron_variant
rs1435600269	intron_variant,genic_upstream_transcript_variant
rs1435624973	intron_variant,genic_upstream_transcript_variant
rs1435631237	intron_variant,genic_upstream_transcript_variant
rs1435631699	intron_variant,genic_upstream_transcript_variant
rs1435697337	intron_variant
rs1435714248	intron_variant,genic_upstream_transcript_variant
rs1435735529	intron_variant
rs1435764115	intron_variant,genic_upstream_transcript_variant
rs1435798557	intron_variant,genic_upstream_transcript_variant
rs1435843312	intron_variant,genic_upstream_transcript_variant
rs1435868740	intron_variant,genic_downstream_transcript_variant
rs1435875772	intron_variant,genic_upstream_transcript_variant
rs1435883486	intron_variant,genic_upstream_transcript_variant
rs1435921405	intron_variant,genic_upstream_transcript_variant

rs1435967874	intron_variant,genic_upstream_transcript_variant
rs1436010421	intron_variant
rs1436051571	intron_variant,genic_upstream_transcript_variant,2KB_upstream_variant,upstream_transcript_variant
rs1436117328	intron_variant,genic_downstream_transcript_variant
rs1436134181	intron_variant
rs1436181089	intron_variant,genic_upstream_transcript_variant
rs1436187525	intron_variant,genic_upstream_transcript_variant
rs1436230982	intron_variant
rs1436249154	intron_variant
rs1436336741	intron_variant,genic_upstream_transcript_variant
rs1436403535	intron_variant,genic_upstream_transcript_variant
rs1436456332	intron_variant,genic_upstream_transcript_variant
rs1436460176	intron_variant,genic_downstream_transcript_variant
rs1436506169	intron_variant
rs1436508117	intron_variant
rs1436513176	intron_variant,genic_upstream_transcript_variant
rs1436519586	intron_variant,genic_upstream_transcript_variant
rs1436523254	intron_variant,genic_upstream_transcript_variant
rs1436542970	intron_variant,genic_downstream_transcript_variant
rs1436551337	intron_variant,genic_downstream_transcript_variant
rs1436551644	intron_variant,genic_upstream_transcript_variant
rs1436605019	intron_variant
rs1436639704	intron_variant,genic_upstream_transcript_variant
rs1436659830	intron_variant
rs1436663348	intron_variant
rs1436677870	intron_variant
rs1436693947	intron_variant,genic_upstream_transcript_variant
rs1436709607	intron_variant,genic_upstream_transcript_variant
rs1436747215	intron_variant,genic_downstream_transcript_variant
rs1436759130	intron_variant,genic_upstream_transcript_variant
rs1436772987	intron_variant
rs1436809741	intron_variant,genic_downstream_transcript_variant
rs1436841470	intron_variant,genic_upstream_transcript_variant,upstream_transcript_variant
rs1436860773	intron_variant,genic_downstream_transcript_variant
rs1436861547	intron_variant
rs1436866079	intron_variant,genic_downstream_transcript_variant
rs1436926892	5_prime_UTR_variant,intron_variant
rs1436933061	intron_variant,genic_upstream_transcript_variant
rs1437002570	intron_variant
rs1437011530	intron_variant,genic_upstream_transcript_variant
rs1437060610	intron_variant,genic_downstream_transcript_variant
rs1437100491	intron_variant
rs1437108664	intron_variant,genic_upstream_transcript_variant
rs1437117565	intron_variant,genic_downstream_transcript_variant
rs1437118480	intron_variant
rs1437131858	intron_variant,genic_upstream_transcript_variant,2KB_upstream_variant,upstream_transcript_variant
rs1437134190	intron_variant
rs1437167107	intron_variant
rs1437220287	genic_downstream_transcript_variant,non_coding_transcript_variant,3_prime_UTR_variant
rs1437234110	intron_variant,genic_upstream_transcript_variant
rs1437274566	intron_variant,genic_downstream_transcript_variant
rs1437284090	intron_variant
rs1437315426	intron_variant
rs1437378529	intron_variant
rs1437392330	intron_variant
rs1437396710	intron_variant
rs1437431989	intron_variant,genic_upstream_transcript_variant,non_coding_transcript_variant
rs1437444957	intron_variant,genic_upstream_transcript_variant
rs1437452362	intron_variant,genic_upstream_transcript_variant
rs1437468055	intron_variant
rs1437473226	intron_variant
rs1437483672	intron_variant
rs1437485082	intron_variant
rs1437532300	intron_variant
rs1437549281	intron_variant
rs1437565357	intron_variant,genic_upstream_transcript_variant,upstream_transcript_variant
rs1437572268	intron_variant
rs1437596213	intron_variant,genic_upstream_transcript_variant
rs1437609354	intron_variant
rs1437624012	intron_variant,genic_upstream_transcript_variant,upstream_transcript_variant
rs1437629428	intron_variant,genic_downstream_transcript_variant
rs1437669799	intron_variant,genic_upstream_transcript_variant
rs1437691322	intron_variant,genic_downstream_transcript_variant
rs1437708425	intron_variant

rs1437716134	intron_variant,genic_upstream_transcript_variant
rs1437736682	intron_variant
rs1437749474	intron_variant
rs1437767109	intron_variant
rs1437779258	intron_variant,downstream_transcript_variant,genic_downstream_transcript_variant
rs1437808223	intron_variant,genic_upstream_transcript_variant
rs1437839721	intron_variant,genic_upstream_transcript_variant
rs1437862028	intron_variant,genic_upstream_transcript_variant
rs1437881445	intron_variant,genic_downstream_transcript_variant
rs1437909870	intron_variant
rs1437944478	intron_variant,genic_upstream_transcript_variant
rs1438001518	intron_variant,genic_upstream_transcript_variant
rs1438043003	intron_variant,genic_upstream_transcript_variant
rs1438050335	intron_variant,genic_upstream_transcript_variant
rs1438102219	intron_variant,genic_downstream_transcript_variant
rs1438115727	intron_variant,genic_upstream_transcript_variant
rs1438131275	intron_variant,genic_upstream_transcript_variant
rs1438151978	intron_variant,genic_downstream_transcript_variant
rs1438184442	intron_variant,genic_downstream_transcript_variant
rs1438192610	intron_variant,genic_upstream_transcript_variant
rs1438198866	intron_variant
rs1438228501	intron_variant,genic_upstream_transcript_variant
rs1438232601	intron_variant,genic_downstream_transcript_variant
rs1438240355	intron_variant,genic_upstream_transcript_variant,upstream_transcript_variant
rs1438286391	intron_variant,genic_upstream_transcript_variant
rs1438310753	intron_variant,genic_upstream_transcript_variant
rs1438452116	intron_variant,genic_upstream_transcript_variant
rs1438473828	intron_variant,genic_downstream_transcript_variant
rs1438485081	intron_variant
rs1438510439	intron_variant,genic_upstream_transcript_variant,2KB_upstream_variant,upstream_transcript_variant
rs1438528358	intron_variant,genic_upstream_transcript_variant
rs1438536030	intron_variant,genic_upstream_transcript_variant,2KB_upstream_variant,upstream_transcript_variant
rs1438546473	intron_variant,genic_upstream_transcript_variant
rs1438559140	intron_variant
rs1438615066	intron_variant,genic_downstream_transcript_variant
rs1438649843	intron_variant,genic_upstream_transcript_variant
rs1438708725	intron_variant
rs1438720312	intron_variant,genic_upstream_transcript_variant
rs1438748954	intron_variant,genic_downstream_transcript_variant
rs1438759460	intron_variant,genic_downstream_transcript_variant
rs1438765468	intron_variant,genic_downstream_transcript_variant
rs1438879342	intron_variant
rs1438905380	intron_variant,genic_upstream_transcript_variant
rs1438908777	genic_downstream_transcript_variant,non_coding_transcript_variant,3_prime_UTR_variant
rs1438952536	intron_variant,genic_downstream_transcript_variant
rs1438963952	intron_variant,genic_upstream_transcript_variant
rs1438967407	intron_variant
rs1438978997	intron_variant,genic_downstream_transcript_variant
rs1439099499	intron_variant,genic_downstream_transcript_variant
rs1439105959	intron_variant,genic_upstream_transcript_variant
rs1439113822	intron_variant,genic_upstream_transcript_variant,2KB_upstream_variant,upstream_transcript_variant
rs1439179452	intron_variant
rs1439219677	intron_variant,genic_upstream_transcript_variant
rs1439231722	intron_variant
rs1439255593	intron_variant
rs1439279034	intron_variant
rs1439328802	intron_variant,genic_upstream_transcript_variant
rs1439365648	intron_variant,genic_upstream_transcript_variant,upstream_transcript_variant
rs1439381016	intron_variant,genic_upstream_transcript_variant
rs1439382703	intron_variant,genic_upstream_transcript_variant
rs1439436813	intron_variant,genic_upstream_transcript_variant
rs1439445236	intron_variant
rs1439457079	intron_variant
rs1439494694	intron_variant
rs1439511174	intron_variant,genic_downstream_transcript_variant
rs1439535149	intron_variant
rs1439557706	intron_variant
rs1439566047	intron_variant
rs1439617369	intron_variant,genic_upstream_transcript_variant
rs1439628192	intron_variant
rs1439665841	intron_variant
rs1439670618	intron_variant
rs1439670837	intron_variant,genic_downstream_transcript_variant
rs1439703750	intron_variant,genic_upstream_transcript_variant

rs1439708952	intron_variant,genic_downstream_transcript_variant
rs1439709860	intron_variant
rs1439721481	intron_variant,genic_upstream_transcript_variant
rs1439770968	intron_variant
rs1439849928	intron_variant,genic_upstream_transcript_variant,2KB_upstream_variant,upstream_transcript_variant
rs1439852002	intron_variant,genic_upstream_transcript_variant
rs1439905873	intron_variant,genic_upstream_transcript_variant
rs1439916111	intron_variant,genic_upstream_transcript_variant,upstream_transcript_variant
rs1439952494	intron_variant
rs1439968925	intron_variant,genic_upstream_transcript_variant
rs1439994290	intron_variant,genic_upstream_transcript_variant
rs1440009774	intron_variant,genic_upstream_transcript_variant
rs1440010692	intron_variant,genic_upstream_transcript_variant
rs1440030328	intron_variant
rs1440046371	intron_variant,genic_upstream_transcript_variant
rs1440046822	intron_variant,genic_upstream_transcript_variant
rs1440134288	intron_variant
rs1440156485	intron_variant,genic_upstream_transcript_variant
rs1440158497	intron_variant,genic_upstream_transcript_variant
rs1440179169	intron_variant,genic_upstream_transcript_variant
rs1440198420	intron_variant
rs1440205140	intron_variant,genic_downstream_transcript_variant
rs1440277517	intron_variant
rs1440290046	intron_variant,genic_upstream_transcript_variant
rs1440301773	intron_variant,genic_upstream_transcript_variant
rs1440302599	intron_variant
rs1440380089	intron_variant
rs1440413164	intron_variant
rs1440423028	intron_variant
rs1440463360	intron_variant,genic_upstream_transcript_variant,2KB_upstream_variant,upstream_transcript_variant
rs1440464907	intron_variant,genic_downstream_transcript_variant
rs1440486392	intron_variant
rs1440490811	intron_variant,genic_upstream_transcript_variant
rs1440546304	intron_variant,genic_upstream_transcript_variant
rs1440584800	intron_variant,genic_downstream_transcript_variant
rs1440645474	intron_variant,genic_upstream_transcript_variant
rs1440659811	intron_variant
rs1440666097	genic_downstream_transcript_variant,non_coding_transcript_variant,3_prime_UTR_variant
rs1440668430	intron_variant
rs1440698145	intron_variant
rs1440705422	intron_variant,genic_upstream_transcript_variant
rs1440733976	intron_variant,genic_downstream_transcript_variant
rs1440741109	intron_variant,genic_upstream_transcript_variant
rs1440791383	intron_variant
rs1440807671	intron_variant
rs1440821150	intron_variant
rs1440825320	intron_variant,genic_downstream_transcript_variant
rs1440844359	intron_variant
rs1440851397	intron_variant,genic_upstream_transcript_variant,2KB_upstream_variant,upstream_transcript_variant
rs1440878752	intron_variant,genic_upstream_transcript_variant
rs1440891595	intron_variant,genic_upstream_transcript_variant,2KB_upstream_variant,upstream_transcript_variant
rs1440894147	intron_variant,genic_downstream_transcript_variant
rs1440897950	intron_variant,genic_upstream_transcript_variant
rs1440959096	intron_variant,genic_downstream_transcript_variant
rs1440995066	intron_variant,genic_downstream_transcript_variant
rs1441004116	intron_variant
rs1441024422	intron_variant
rs1441065788	intron_variant,genic_downstream_transcript_variant
rs1441134243	intron_variant,genic_upstream_transcript_variant
rs1441143099	intron_variant
rs1441160738	intron_variant,genic_downstream_transcript_variant
rs1441201201	intron_variant
rs1441252473	intron_variant,genic_upstream_transcript_variant
rs1441258804	intron_variant,genic_upstream_transcript_variant
rs1441339856	intron_variant
rs1441341168	intron_variant,genic_upstream_transcript_variant,upstream_transcript_variant
rs1441343210	intron_variant,genic_upstream_transcript_variant
rs1441360849	intron_variant
rs1441366936	intron_variant,genic_upstream_transcript_variant
rs1441437317	intron_variant,genic_downstream_transcript_variant
rs1441439318	intron_variant,genic_upstream_transcript_variant
rs1441530431	intron_variant,genic_upstream_transcript_variant
rs1441533020	intron_variant
rs1441538198	intron_variant

rs1441557156	intron_variant,genic_downstream_transcript_variant
rs1441571339	intron_variant
rs1441571719	intron_variant
rs1441592074	intron_variant
rs1441608528	intron_variant
rs1441630311	intron_variant,genic_upstream_transcript_variant
rs1441664087	intron_variant,genic_upstream_transcript_variant
rs1441677642	intron_variant
rs1441696183	intron_variant,genic_downstream_transcript_variant
rs1441703570	intron_variant
rs1441716383	intron_variant,genic_upstream_transcript_variant
rs1441773448	intron_variant,genic_upstream_transcript_variant
rs1441837192	intron_variant,genic_upstream_transcript_variant
rs1441845537	intron_variant
rs1441905805	intron_variant
rs1441966958	intron_variant
rs1441987522	intron_variant
rs1442007237	intron_variant,genic_upstream_transcript_variant
rs1442009256	intron_variant,genic_downstream_transcript_variant
rs1442033161	coding_sequence_variant,non_coding_transcript_variant,genic_downstream_transcript_variant,missense_variant
rs1442056998	intron_variant
rs1442064323	intron_variant,genic_downstream_transcript_variant
rs1442133748	intron_variant,genic_upstream_transcript_variant
rs1442179237	intron_variant,genic_upstream_transcript_variant
rs1442196158	intron_variant
rs1442233904	intron_variant,genic_upstream_transcript_variant
rs1442282293	intron_variant,genic_downstream_transcript_variant
rs1442291961	intron_variant,genic_upstream_transcript_variant
rs1442314015	intron_variant,genic_upstream_transcript_variant,upstream_transcript_variant
rs1442356969	intron_variant
rs1442361796	intron_variant,genic_upstream_transcript_variant
rs1442364503	intron_variant,genic_upstream_transcript_variant
rs1442388655	intron_variant,downstream_transcript_variant,genic_downstream_transcript_variant
rs1442439194	intron_variant,genic_downstream_transcript_variant
rs1442468446	intron_variant
rs1442485111	intron_variant,genic_upstream_transcript_variant
rs1442497632	intron_variant
rs1442519055	intron_variant,genic_upstream_transcript_variant
rs1442521366	intron_variant
rs1442545694	intron_variant,genic_upstream_transcript_variant
rs1442570633	intron_variant,genic_upstream_transcript_variant
rs1442616328	5_prime_UTR_variant,intron_variant,genic_upstream_transcript_variant,upstream_transcript_variant
rs1442684871	intron_variant,genic_upstream_transcript_variant
rs1442699108	intron_variant,genic_upstream_transcript_variant
rs1442702228	intron_variant
rs1442730113	intron_variant,genic_upstream_transcript_variant
rs1442758267	intron_variant,genic_downstream_transcript_variant
rs1442779830	intron_variant
rs1442787118	intron_variant,genic_upstream_transcript_variant
rs1442797154	intron_variant,genic_upstream_transcript_variant,2KB_upstream_variant,upstream_transcript_variant
rs1442851708	intron_variant,genic_downstream_transcript_variant
rs1442854793	intron_variant,genic_upstream_transcript_variant
rs1442859159	intron_variant,genic_upstream_transcript_variant
rs1442868774	intron_variant,genic_upstream_transcript_variant
rs1442900099	intron_variant
rs1442929368	intron_variant
rs1442968412	intron_variant,genic_upstream_transcript_variant
rs1442968796	intron_variant
rs1442985593	intron_variant,genic_upstream_transcript_variant
rs1442995520	intron_variant,genic_upstream_transcript_variant
rs1443017896	intron_variant,genic_downstream_transcript_variant
rs1443038219	intron_variant,genic_upstream_transcript_variant
rs1443061456	intron_variant,genic_upstream_transcript_variant
rs1443073415	intron_variant,genic_downstream_transcript_variant
rs1443100284	intron_variant,genic_upstream_transcript_variant
rs1443106939	intron_variant,genic_downstream_transcript_variant
rs1443163908	intron_variant,genic_upstream_transcript_variant
rs1443169565	intron_variant,genic_downstream_transcript_variant
rs1443206945	intron_variant
rs1443262966	intron_variant,genic_upstream_transcript_variant
rs1443288289	intron_variant
rs1443296924	intron_variant,genic_upstream_transcript_variant
rs1443355410	intron_variant,genic_upstream_transcript_variant,upstream_transcript_variant
rs1443357429	intron_variant

rs1443404024	intron_variant,downstream_transcript_variant,genic_downstream_transcript_variant
rs1443449604	intron_variant
rs1443450013	genic_downstream_transcript_variant,non_coding_transcript_variant,3_prime_UTR_variant
rs1443482824	intron_variant,genic_upstream_transcript_variant
rs1443564966	genic_downstream_transcript_variant,non_coding_transcript_variant,3_prime_UTR_variant
rs1443595867	intron_variant,genic_upstream_transcript_variant
rs1443613503	intron_variant
rs1443632184	intron_variant,genic_upstream_transcript_variant
rs1443654497	intron_variant
rs1443657580	intron_variant,genic_upstream_transcript_variant
rs1443684031	5_prime_UTR_variant,intron_variant,genic_upstream_transcript_variant,upstream_transcript_variant
rs1443710471	intron_variant,genic_upstream_transcript_variant
rs1443710904	intron_variant,genic_upstream_transcript_variant
rs1443754216	intron_variant
rs1443767620	intron_variant,genic_downstream_transcript_variant
rs1443786286	intron_variant
rs1443853339	intron_variant,genic_upstream_transcript_variant,upstream_transcript_variant
rs1443857547	intron_variant
rs1443874447	intron_variant,genic_upstream_transcript_variant
rs1443946973	intron_variant,genic_downstream_transcript_variant
rs1444047447	intron_variant
rs1444088056	intron_variant
rs1444099719	intron_variant,genic_upstream_transcript_variant,2KB_upstream_variant,upstream_transcript_variant
rs1444124149	intron_variant
rs1444228421	intron_variant,genic_upstream_transcript_variant
rs1444257484	intron_variant,genic_downstream_transcript_variant
rs1444294291	intron_variant
rs1444303132	intron_variant,genic_downstream_transcript_variant
rs1444304903	intron_variant,genic_upstream_transcript_variant
rs1444311107	intron_variant
rs1444318186	intron_variant
rs1444366603	intron_variant,genic_upstream_transcript_variant,2KB_upstream_variant,upstream_transcript_variant
rs1444375628	intron_variant,genic_downstream_transcript_variant
rs1444382256	intron_variant
rs1444437446	intron_variant
rs1444503446	intron_variant,genic_upstream_transcript_variant
rs1444523758	intron_variant,genic_upstream_transcript_variant
rs1444542934	intron_variant
rs1444552480	intron_variant,genic_downstream_transcript_variant
rs1444558418	intron_variant
rs1444586008	intron_variant
rs1444598035	intron_variant,genic_upstream_transcript_variant
rs1444619207	intron_variant,genic_upstream_transcript_variant,2KB_upstream_variant,upstream_transcript_variant
rs1444624376	intron_variant
rs1444658555	intron_variant
rs1444670424	intron_variant,genic_downstream_transcript_variant
rs1444686986	intron_variant,genic_downstream_transcript_variant
rs1444710732	intron_variant,genic_upstream_transcript_variant
rs1444748563	intron_variant,genic_upstream_transcript_variant,upstream_transcript_variant
rs1444786251	intron_variant,genic_upstream_transcript_variant,upstream_transcript_variant
rs1444795762	genic_downstream_transcript_variant,non_coding_transcript_variant,3_prime_UTR_variant
rs1444852042	5_prime_UTR_variant,intron_variant,genic_upstream_transcript_variant,upstream_transcript_variant
rs1444862137	intron_variant,genic_upstream_transcript_variant
rs1444880948	genic_downstream_transcript_variant,non_coding_transcript_variant,3_prime_UTR_variant
rs1444923949	intron_variant
rs1444954230	intron_variant,genic_upstream_transcript_variant
rs1444984092	intron_variant
rs1444994776	intron_variant,genic_downstream_transcript_variant
rs1445021007	intron_variant
rs1445021763	intron_variant
rs1445031347	intron_variant,genic_upstream_transcript_variant
rs1445075043	intron_variant
rs1445075418	intron_variant
rs1445121623	coding_sequence_variant,synonymous_variant,non_coding_transcript_variant
rs1445147879	intron_variant,genic_upstream_transcript_variant
rs1445223680	intron_variant,genic_downstream_transcript_variant
rs1445281475	intron_variant,genic_upstream_transcript_variant
rs1445305898	intron_variant,genic_upstream_transcript_variant
rs1445307071	intron_variant,genic_downstream_transcript_variant
rs1445317593	intron_variant,genic_upstream_transcript_variant
rs1445324911	intron_variant,genic_downstream_transcript_variant
rs1445326512	intron_variant,genic_upstream_transcript_variant,upstream_transcript_variant
rs1445335059	intron_variant,genic_upstream_transcript_variant
rs1445404260	intron_variant,genic_downstream_transcript_variant

rs1445405600	intron_variant,genic_upstream_transcript_variant,2KB_upstream_variant,upstream_transcript_variant
rs1445427882	intron_variant
rs1445431223	intron_variant
rs1445453647	intron_variant
rs1445487679	intron_variant,genic_downstream_transcript_variant
rs1445515686	intron_variant,genic_upstream_transcript_variant
rs1445558360	intron_variant,genic_downstream_transcript_variant
rs1445587801	intron_variant,genic_downstream_transcript_variant
rs1445611867	intron_variant
rs1445644732	intron_variant
rs1445653912	intron_variant,genic_upstream_transcript_variant
rs1445656851	intron_variant
rs1445711582	intron_variant,genic_downstream_transcript_variant
rs1445772904	intron_variant
rs1445787783	intron_variant,genic_upstream_transcript_variant
rs1445794545	intron_variant,genic_upstream_transcript_variant,2KB_upstream_variant,upstream_transcript_variant
rs1445823660	intron_variant
rs1445866185	intron_variant,genic_downstream_transcript_variant
rs1445927959	intron_variant,genic_downstream_transcript_variant
rs1445936618	intron_variant,genic_downstream_transcript_variant
rs1445948621	intron_variant,genic_upstream_transcript_variant
rs1445950878	intron_variant,genic_upstream_transcript_variant
rs1445964223	intron_variant,genic_upstream_transcript_variant
rs1445967834	intron_variant,genic_upstream_transcript_variant
rs1446006228	intron_variant
rs1446059878	intron_variant
rs1446104968	intron_variant
rs1446112030	intron_variant,genic_upstream_transcript_variant,2KB_upstream_variant,upstream_transcript_variant
rs1446128287	intron_variant,genic_upstream_transcript_variant
rs1446170462	intron_variant,genic_downstream_transcript_variant
rs1446221027	intron_variant
rs1446254278	intron_variant,genic_downstream_transcript_variant
rs1446280624	intron_variant,genic_upstream_transcript_variant
rs1446315437	intron_variant
rs1446336684	intron_variant,genic_upstream_transcript_variant
rs1446376609	intron_variant
rs1446394707	intron_variant
rs1446417281	intron_variant,genic_upstream_transcript_variant
rs1446420652	intron_variant
rs1446423671	intron_variant,genic_downstream_transcript_variant
rs1446451700	intron_variant,genic_upstream_transcript_variant
rs1446467818	intron_variant
rs1446529483	intron_variant,genic_upstream_transcript_variant
rs1446544246	intron_variant,genic_downstream_transcript_variant
rs1446546769	intron_variant,genic_upstream_transcript_variant,upstream_transcript_variant
rs1446575309	intron_variant,genic_downstream_transcript_variant
rs1446601186	intron_variant,genic_upstream_transcript_variant
rs1446697799	intron_variant
rs1446741457	intron_variant,genic_upstream_transcript_variant
rs1446742412	intron_variant,genic_upstream_transcript_variant
rs1446763129	intron_variant,genic_upstream_transcript_variant
rs1446799912	intron_variant
rs1446825274	intron_variant,genic_downstream_transcript_variant
rs1446827883	intron_variant,genic_upstream_transcript_variant,2KB_upstream_variant,upstream_transcript_variant
rs1446837174	intron_variant
rs1446850277	intron_variant,genic_downstream_transcript_variant
rs1446878610	intron_variant,genic_upstream_transcript_variant,2KB_upstream_variant,upstream_transcript_variant
rs1446950110	intron_variant,genic_downstream_transcript_variant
rs1446952689	intron_variant,genic_upstream_transcript_variant
rs1446999225	intron_variant,genic_upstream_transcript_variant
rs1447077979	intron_variant,genic_upstream_transcript_variant
rs1447085665	genic_downstream_transcript_variant,intron_variant
rs1447117276	genic_downstream_transcript_variant,intron_variant
rs1447171584	intron_variant,genic_upstream_transcript_variant
rs1447178674	intron_variant
rs1447194968	intron_variant,genic_upstream_transcript_variant
rs1447241911	genic_downstream_transcript_variant,intron_variant
rs1447294010	genic_downstream_transcript_variant,non_coding_transcript_variant,3_prime_UTR_variant
rs1447302126	intron_variant,2KB_upstream_variant,upstream_transcript_variant,genic_upstream_transcript_variant
rs1447303234	intron_variant,upstream_transcript_variant,genic_upstream_transcript_variant
rs1447304750	intron_variant
rs1447310321	genic_downstream_transcript_variant,intron_variant
rs1447334920	intron_variant,genic_upstream_transcript_variant
rs1447373165	genic_downstream_transcript_variant,intron_variant

rs1447406290	genic_downstream_transcript_variant,intron_variant
rs1447409236	intron_variant,genic_upstream_transcript_variant
rs1447422345	intron_variant,genic_upstream_transcript_variant
rs1447446605	intron_variant,genic_upstream_transcript_variant
rs1447461127	intron_variant,genic_upstream_transcript_variant
rs1447502492	intron_variant,genic_upstream_transcript_variant
rs1447515910	intron_variant,genic_upstream_transcript_variant
rs1447525785	intron_variant,genic_upstream_transcript_variant
rs1447529225	intron_variant,genic_upstream_transcript_variant
rs1447563757	intron_variant,genic_upstream_transcript_variant
rs1447584197	intron_variant,genic_upstream_transcript_variant
rs1447616518	genic_downstream_transcript_variant,non_coding_transcript_variant,3_prime_UTR_variant
rs1447657097	intron_variant,2KB_upstream_variant,upstream_transcript_variant,genic_upstream_transcript_variant
rs1447669310	intron_variant
rs1447694084	genic_downstream_transcript_variant,intron_variant
rs1447787530	intron_variant,genic_upstream_transcript_variant
rs1447818720	intron_variant
rs1447823042	intron_variant,genic_upstream_transcript_variant
rs1447872309	intron_variant,genic_upstream_transcript_variant
rs1447920824	intron_variant,genic_upstream_transcript_variant
rs1447957434	intron_variant,genic_upstream_transcript_variant
rs1448004021	intron_variant
rs1448034553	intron_variant
rs1448040800	intron_variant,genic_upstream_transcript_variant
rs1448051974	intron_variant,upstream_transcript_variant,genic_upstream_transcript_variant
rs1448096556	intron_variant
rs1448102098	genic_downstream_transcript_variant,intron_variant
rs1448163855	intron_variant,upstream_transcript_variant,genic_upstream_transcript_variant
rs1448224184	intron_variant,genic_upstream_transcript_variant
rs1448240893	intron_variant
rs1448249129	intron_variant
rs1448273895	intron_variant
rs1448319901	intron_variant
rs1448332767	intron_variant
rs1448355118	intron_variant
rs1448421810	intron_variant,genic_upstream_transcript_variant
rs1448472584	intron_variant
rs1448485621	intron_variant,genic_upstream_transcript_variant
rs1448486609	intron_variant
rs1448503614	intron_variant
rs1448515729	intron_variant
rs1448515913	genic_downstream_transcript_variant,intron_variant
rs1448518903	intron_variant
rs1448539890	intron_variant
rs1448549940	intron_variant
rs1448556741	intron_variant
rs1448571208	intron_variant,genic_upstream_transcript_variant
rs1448577527	intron_variant,genic_upstream_transcript_variant
rs1448600869	genic_downstream_transcript_variant,intron_variant
rs1448605219	intron_variant,genic_upstream_transcript_variant
rs1448658512	intron_variant,genic_upstream_transcript_variant
rs1448665377	genic_downstream_transcript_variant,intron_variant
rs1448668865	intron_variant
rs1448671801	intron_variant
rs1448706064	intron_variant
rs1448706226	intron_variant
rs1448735974	intron_variant
rs1448753961	intron_variant,genic_upstream_transcript_variant
rs1448760775	intron_variant,genic_upstream_transcript_variant
rs1448780839	intron_variant,genic_upstream_transcript_variant
rs1448782491	intron_variant
rs1448783324	intron_variant
rs1448874389	intron_variant
rs1448931895	intron_variant,genic_upstream_transcript_variant
rs1448941622	intron_variant,2KB_upstream_variant,upstream_transcript_variant,genic_upstream_transcript_variant
rs1448959323	intron_variant
rs1448963785	intron_variant
rs1448965564	intron_variant,genic_upstream_transcript_variant
rs1449041011	intron_variant,genic_upstream_transcript_variant
rs1449094867	genic_downstream_transcript_variant,intron_variant
rs1449125475	genic_downstream_transcript_variant,intron_variant
rs1449127420	intron_variant,2KB_upstream_variant,upstream_transcript_variant,genic_upstream_transcript_variant
rs1449145121	intron_variant
rs1449147155	intron_variant,genic_upstream_transcript_variant

rs1449149070	intron_variant
rs1449149302	intron_variant,genic_upstream_transcript_variant
rs1449154388	intron_variant,genic_upstream_transcript_variant
rs1449201119	intron_variant
rs1449226043	intron_variant,genic_upstream_transcript_variant
rs1449257647	intron_variant,genic_upstream_transcript_variant
rs1449267886	intron_variant
rs1449298285	intron_variant,genic_upstream_transcript_variant
rs1449312008	genic_downstream_transcript_variant,intron_variant
rs1449342390	intron_variant,genic_upstream_transcript_variant
rs1449398596	intron_variant,genic_upstream_transcript_variant
rs1449416038	intron_variant,genic_upstream_transcript_variant
rs1449434628	intron_variant
rs1449529740	genic_downstream_transcript_variant,intron_variant,downstream_transcript_variant
rs1449530733	intron_variant,2KB_upstream_variant,upstream_transcript_variant,genic_upstream_transcript_variant
rs1449534827	intron_variant
rs1449543084	genic_downstream_transcript_variant,intron_variant
rs1449633106	genic_downstream_transcript_variant,intron_variant
rs1449637503	intron_variant
rs1449674619	intron_variant
rs1449682996	genic_downstream_transcript_variant,intron_variant
rs1449688510	intron_variant
rs1449690750	intron_variant
rs1449699458	genic_downstream_transcript_variant,intron_variant
rs1449726871	intron_variant
rs1449775526	genic_downstream_transcript_variant,intron_variant
rs1449793434	genic_downstream_transcript_variant,intron_variant
rs1449800720	intron_variant,genic_upstream_transcript_variant
rs1449810308	intron_variant
rs1449817462	intron_variant,genic_upstream_transcript_variant
rs1449847710	genic_downstream_transcript_variant,intron_variant
rs1449898097	intron_variant,genic_upstream_transcript_variant
rs1449945439	intron_variant,genic_upstream_transcript_variant
rs1449952552	genic_downstream_transcript_variant,non_coding_transcript_variant,3_prime_UTR_variant
rs1449996119	intron_variant,genic_upstream_transcript_variant
rs1450017360	genic_downstream_transcript_variant,intron_variant
rs1450024714	intron_variant
rs1450027274	intron_variant,upstream_transcript_variant,genic_upstream_transcript_variant
rs1450046927	500B_downstream_variant,downstream_transcript_variant
rs1450056874	intron_variant
rs1450063873	intron_variant
rs1450083317	intron_variant,genic_upstream_transcript_variant
rs1450131513	intron_variant
rs1450150968	intron_variant
rs1450154086	intron_variant
rs1450224009	intron_variant
rs1450255910	5_prime_UTR_variant,intron_variant,upstream_transcript_variant,genic_upstream_transcript_variant
rs1450258853	intron_variant,genic_upstream_transcript_variant
rs1450265791	genic_downstream_transcript_variant,intron_variant
rs1450279142	genic_downstream_transcript_variant,non_coding_transcript_variant,3_prime_UTR_variant
rs1450330667	genic_downstream_transcript_variant,intron_variant
rs1450392975	intron_variant,upstream_transcript_variant,genic_upstream_transcript_variant
rs1450412441	intron_variant
rs1450443192	intron_variant
rs1450448149	intron_variant
rs1450453005	genic_downstream_transcript_variant,intron_variant
rs1450478794	intron_variant
rs1450502953	intron_variant,genic_upstream_transcript_variant
rs1450504771	intron_variant
rs1450538144	intron_variant
rs1450556347	intron_variant,genic_upstream_transcript_variant
rs1450588377	intron_variant,genic_upstream_transcript_variant
rs1450604409	intron_variant,genic_upstream_transcript_variant
rs1450608134	intron_variant
rs1450651777	intron_variant
rs1450651953	intron_variant,genic_upstream_transcript_variant
rs1450659866	intron_variant
rs1450709644	intron_variant,genic_upstream_transcript_variant
rs1450755090	intron_variant,genic_upstream_transcript_variant
rs1450776979	intron_variant
rs1450792127	intron_variant,genic_upstream_transcript_variant
rs1450801067	intron_variant,genic_upstream_transcript_variant
rs1450806065	genic_downstream_transcript_variant,intron_variant
rs1450863705	intron_variant,genic_upstream_transcript_variant

rs1450885120	intron_variant,genic_upstream_transcript_variant
rs1450906177	intron_variant,genic_upstream_transcript_variant,synonymous_variant,coding_sequence_variant,non_coding_transcript_variant
rs1450909776	intron_variant,genic_upstream_transcript_variant
rs1450943645	genic_downstream_transcript_variant,intron_variant
rs1450943988	intron_variant
rs1450949503	genic_downstream_transcript_variant,intron_variant
rs1451000137	intron_variant,genic_upstream_transcript_variant
rs1451060545	intron_variant
rs1451064728	intron_variant
rs1451071290	intron_variant,upstream_transcript_variant,genic_upstream_transcript_variant
rs1451202062	intron_variant,genic_upstream_transcript_variant
rs1451287840	intron_variant,genic_upstream_transcript_variant
rs1451347802	intron_variant,2KB_upstream_variant,upstream_transcript_variant,genic_upstream_transcript_variant
rs1451363712	intron_variant
rs1451394426	intron_variant,upstream_transcript_variant,genic_upstream_transcript_variant
rs1451443095	intron_variant
rs1451458323	intron_variant,genic_upstream_transcript_variant
rs1451472823	genic_downstream_transcript_variant,intron_variant
rs1451475764	intron_variant
rs1451501864	intron_variant
rs1451515707	intron_variant
rs1451523237	intron_variant,genic_upstream_transcript_variant
rs1451534403	intron_variant,genic_upstream_transcript_variant
rs1451549590	intron_variant,genic_upstream_transcript_variant
rs1451553302	intron_variant
rs1451640811	intron_variant,genic_upstream_transcript_variant
rs1451662563	intron_variant
rs1451701062	intron_variant,genic_upstream_transcript_variant
rs1451716414	intron_variant
rs1451718723	coding_sequence_variant,synonymous_variant,non_coding_transcript_variant
rs1451749445	genic_downstream_transcript_variant,intron_variant
rs1451771202	genic_downstream_transcript_variant,non_coding_transcript_variant,3_prime_UTR_variant
rs1451777369	genic_downstream_transcript_variant,intron_variant
rs1451793261	intron_variant,genic_upstream_transcript_variant
rs1451812191	intron_variant,genic_upstream_transcript_variant
rs1451866705	intron_variant,genic_upstream_transcript_variant
rs1451866776	intron_variant
rs1451903478	intron_variant
rs1451919915	genic_downstream_transcript_variant,intron_variant
rs1451935647	intron_variant,upstream_transcript_variant,genic_upstream_transcript_variant
rs1451943747	genic_downstream_transcript_variant,intron_variant
rs1451954069	coding_sequence_variant,non_coding_transcript_variant,missense_variant
rs1451980413	intron_variant
rs1451988777	intron_variant,upstream_transcript_variant,genic_upstream_transcript_variant
rs1452056062	intron_variant
rs1452066232	intron_variant,genic_upstream_transcript_variant
rs1452071962	genic_downstream_transcript_variant,intron_variant
rs1452115032	genic_downstream_transcript_variant,non_coding_transcript_variant,3_prime_UTR_variant
rs1452172997	intron_variant
rs1452219207	genic_downstream_transcript_variant,intron_variant
rs1452252502	intron_variant,genic_upstream_transcript_variant
rs1452257344	intron_variant,genic_upstream_transcript_variant
rs1452286279	intron_variant,genic_upstream_transcript_variant
rs1452288384	intron_variant,genic_upstream_transcript_variant
rs1452303039	intron_variant,genic_upstream_transcript_variant
rs1452325741	intron_variant
rs1452340452	intron_variant,genic_upstream_transcript_variant
rs1452381402	intron_variant,genic_upstream_transcript_variant
rs1452416856	intron_variant
rs1452461998	intron_variant
rs1452465768	intron_variant,genic_upstream_transcript_variant
rs1452475408	genic_downstream_transcript_variant,intron_variant
rs1452501755	intron_variant,genic_upstream_transcript_variant
rs1452513990	intron_variant,genic_upstream_transcript_variant
rs1452604186	intron_variant,genic_upstream_transcript_variant
rs1452674834	genic_downstream_transcript_variant,intron_variant
rs1452679711	intron_variant,genic_upstream_transcript_variant
rs1452722843	intron_variant,upstream_transcript_variant,genic_upstream_transcript_variant
rs1452727189	intron_variant
rs1452737690	intron_variant,genic_upstream_transcript_variant
rs1452785858	intron_variant
rs1452848504	genic_downstream_transcript_variant,coding_sequence_variant,synonymous_variant,non_coding_transcript_variant
rs1452850048	intron_variant,genic_upstream_transcript_variant
rs1452904952	intron_variant,genic_upstream_transcript_variant

rs1452909359	intron_variant,genic_upstream_transcript_variant
rs1452924621	intron_variant,genic_upstream_transcript_variant
rs1452981617	intron_variant,genic_upstream_transcript_variant
rs1453037299	genic_downstream_transcript_variant,intron_variant
rs1453069187	genic_downstream_transcript_variant,intron_variant
rs1453122081	intron_variant,genic_upstream_transcript_variant
rs1453128167	intron_variant,genic_upstream_transcript_variant
rs1453194027	intron_variant
rs1453200681	intron_variant
rs1453239888	intron_variant,genic_upstream_transcript_variant
rs1453250797	intron_variant,2KB_upstream_variant,upstream_transcript_variant,genic_upstream_transcript_variant
rs1453316667	intron_variant,genic_upstream_transcript_variant
rs1453324511	intron_variant,genic_upstream_transcript_variant
rs1453372149	genic_downstream_transcript_variant,intron_variant
rs1453378035	500B_downstream_variant,downstream_transcript_variant
rs1453432004	genic_downstream_transcript_variant,intron_variant
rs1453485464	intron_variant,genic_upstream_transcript_variant
rs1453571428	intron_variant,genic_upstream_transcript_variant
rs1453594722	intron_variant,genic_upstream_transcript_variant
rs1453638954	intron_variant,genic_upstream_transcript_variant
rs1453666408	genic_downstream_transcript_variant,intron_variant
rs1453682136	intron_variant,2KB_upstream_variant,upstream_transcript_variant,genic_upstream_transcript_variant
rs1453772877	genic_downstream_transcript_variant,intron_variant
rs1453811189	intron_variant
rs1453820430	intron_variant,genic_upstream_transcript_variant
rs1453825641	intron_variant,upstream_transcript_variant,genic_upstream_transcript_variant
rs1453838209	intron_variant,genic_upstream_transcript_variant
rs1453851167	genic_downstream_transcript_variant,intron_variant
rs1453890954	intron_variant
rs1453935875	genic_downstream_transcript_variant,intron_variant
rs1453947690	intron_variant,genic_upstream_transcript_variant
rs1453963656	genic_downstream_transcript_variant,intron_variant
rs1453984764	intron_variant,genic_upstream_transcript_variant
rs1454009222	intron_variant
rs1454011180	intron_variant
rs1454025306	intron_variant,genic_upstream_transcript_variant
rs1454036960	intron_variant
rs1454042338	intron_variant
rs1454053311	intron_variant,genic_upstream_transcript_variant
rs1454064898	intron_variant
rs1454066619	intron_variant
rs1454105035	genic_downstream_transcript_variant,intron_variant
rs1454113950	genic_downstream_transcript_variant,intron_variant
rs1454123766	genic_downstream_transcript_variant,intron_variant
rs1454167413	intron_variant,genic_upstream_transcript_variant
rs1454197784	intron_variant,genic_upstream_transcript_variant
rs1454232556	intron_variant,genic_upstream_transcript_variant
rs1454242502	intron_variant
rs1454307589	intron_variant
rs1454315937	genic_downstream_transcript_variant,intron_variant
rs1454338135	intron_variant
rs1454342760	intron_variant,upstream_transcript_variant,genic_upstream_transcript_variant
rs1454345522	intron_variant
rs1454362470	intron_variant,genic_upstream_transcript_variant
rs1454400050	genic_downstream_transcript_variant,intron_variant
rs1454420859	intron_variant
rs1454429595	intron_variant
rs1454435277	intron_variant,genic_upstream_transcript_variant
rs1454437590	intron_variant,genic_upstream_transcript_variant
rs1454442669	intron_variant,2KB_upstream_variant,upstream_transcript_variant,genic_upstream_transcript_variant
rs1454450050	intron_variant
rs1454509768	intron_variant,genic_upstream_transcript_variant
rs1454552203	intron_variant
rs1454559850	intron_variant,genic_upstream_transcript_variant
rs1454565678	intron_variant
rs1454569952	genic_downstream_transcript_variant,intron_variant
rs1454592385	intron_variant
rs1454605173	intron_variant
rs1454611094	intron_variant
rs1454634763	intron_variant
rs1454652222	intron_variant,genic_upstream_transcript_variant
rs1454689300	intron_variant
rs1454696095	intron_variant
rs1454700091	intron_variant

rs1454725828	intron_variant
rs1454726204	intron_variant,genic_upstream_transcript_variant
rs1454742119	intron_variant
rs1454746602	intron_variant,genic_upstream_transcript_variant
rs1454764056	intron_variant,genic_upstream_transcript_variant
rs1454778500	intron_variant,genic_upstream_transcript_variant
rs1454828524	genic_downstream_transcript_variant,intron_variant
rs1454854904	intron_variant,genic_upstream_transcript_variant
rs1454856933	intron_variant,genic_upstream_transcript_variant,synonymous_variant,coding_sequence_variant,non_coding_transcript_variant
rs1454887572	intron_variant
rs1454902932	intron_variant,genic_upstream_transcript_variant
rs1454915019	intron_variant,genic_upstream_transcript_variant
rs1454916473	intron_variant,genic_upstream_transcript_variant
rs1455038932	genic_downstream_transcript_variant,intron_variant
rs1455054818	intron_variant,genic_upstream_transcript_variant
rs1455055677	genic_downstream_transcript_variant,intron_variant
rs1455072332	intron_variant,genic_upstream_transcript_variant
rs1455089355	intron_variant
rs1455092032	intron_variant,genic_upstream_transcript_variant
rs1455102575	intron_variant,genic_upstream_transcript_variant
rs1455148545	genic_downstream_transcript_variant,intron_variant
rs1455220918	intron_variant
rs1455335078	intron_variant
rs1455337200	intron_variant,genic_upstream_transcript_variant
rs1455342795	intron_variant
rs1455399445	intron_variant,genic_upstream_transcript_variant
rs1455412759	intron_variant
rs1455440121	intron_variant
rs1455475973	genic_downstream_transcript_variant,intron_variant
rs1455509087	genic_downstream_transcript_variant,intron_variant
rs1455527878	coding_sequence_variant,synonymous_variant,non_coding_transcript_variant
rs1455541728	intron_variant
rs1455546970	intron_variant,genic_upstream_transcript_variant
rs1455577304	intron_variant
rs1455599443	intron_variant
rs1455609129	intron_variant
rs1455696653	intron_variant
rs1455716880	intron_variant
rs1455727329	intron_variant,genic_upstream_transcript_variant
rs1455728819	intron_variant,genic_upstream_transcript_variant
rs1455734334	intron_variant
rs1455751731	intron_variant
rs1455771100	genic_downstream_transcript_variant,intron_variant
rs1455829705	intron_variant,genic_upstream_transcript_variant
rs1455867541	genic_downstream_transcript_variant,intron_variant
rs1455899454	intron_variant
rs1455914162	intron_variant
rs1455962884	intron_variant
rs1456007207	intron_variant,genic_upstream_transcript_variant
rs1456010271	intron_variant
rs1456036205	intron_variant
rs1456075748	intron_variant,genic_upstream_transcript_variant
rs1456082939	genic_downstream_transcript_variant,intron_variant
rs1456120233	intron_variant
rs1456121494	intron_variant
rs1456188718	intron_variant
rs1456217214	intron_variant
rs1456240074	intron_variant,genic_upstream_transcript_variant
rs1456248194	intron_variant
rs1456332887	intron_variant,genic_upstream_transcript_variant
rs1456349482	intron_variant
rs1456379339	intron_variant,genic_upstream_transcript_variant
rs1456398392	genic_downstream_transcript_variant,intron_variant
rs1456415035	intron_variant
rs1456450139	genic_downstream_transcript_variant,intron_variant
rs1456451910	intron_variant,genic_upstream_transcript_variant
rs1456474931	intron_variant,genic_upstream_transcript_variant
rs1456502970	genic_downstream_transcript_variant,intron_variant
rs1456544879	genic_downstream_transcript_variant,intron_variant
rs1456591854	intron_variant,upstream_transcript_variant,genic_upstream_transcript_variant
rs1456593956	intron_variant,genic_upstream_transcript_variant
rs1456615404	intron_variant,genic_upstream_transcript_variant
rs1456633688	genic_downstream_transcript_variant,intron_variant
rs1456662778	intron_variant,genic_upstream_transcript_variant

rs1456668497	intron_variant,genic_upstream_transcript_variant
rs1456677272	intron_variant,genic_upstream_transcript_variant
rs1456699763	intron_variant
rs1456761381	intron_variant,2KB_upstream_variant,upstream_transcript_variant,genic_upstream_transcript_variant
rs1456765623	intron_variant
rs1456776453	intron_variant,2KB_upstream_variant,upstream_transcript_variant,genic_upstream_transcript_variant
rs1456788838	genic_downstream_transcript_variant,intron_variant
rs1456789058	intron_variant,genic_upstream_transcript_variant
rs1456819862	intron_variant,genic_upstream_transcript_variant
rs1456842279	intron_variant,genic_upstream_transcript_variant
rs1456865507	genic_downstream_transcript_variant,intron_variant,downstream_transcript_variant
rs1456880751	genic_downstream_transcript_variant,intron_variant
rs1456899150	intron_variant
rs1456902764	intron_variant,genic_upstream_transcript_variant
rs1456907776	intron_variant,genic_upstream_transcript_variant
rs1456939127	intron_variant
rs1456960633	intron_variant,genic_upstream_transcript_variant
rs1456968649	genic_downstream_transcript_variant,intron_variant
rs1456977872	intron_variant
rs1456978140	genic_downstream_transcript_variant,intron_variant
rs1456997400	intron_variant,genic_upstream_transcript_variant
rs1457025042	intron_variant
rs1457060964	500B_downstream_variant,downstream_transcript_variant
rs1457066927	intron_variant,genic_upstream_transcript_variant
rs1457111670	genic_downstream_transcript_variant,intron_variant
rs1457169800	intron_variant
rs1457206771	intron_variant
rs1457252451	intron_variant,upstream_transcript_variant,genic_upstream_transcript_variant
rs1457279681	intron_variant
rs1457324787	intron_variant,genic_upstream_transcript_variant
rs1457348805	genic_downstream_transcript_variant,intron_variant
rs1457426432	intron_variant
rs1457433423	intron_variant
rs1457446217	genic_downstream_transcript_variant,intron_variant
rs1457473351	intron_variant
rs1457500190	intron_variant
rs1457523478	intron_variant,genic_upstream_transcript_variant
rs1457526363	intron_variant,2KB_upstream_variant,upstream_transcript_variant,genic_upstream_transcript_variant
rs1457531643	intron_variant,genic_upstream_transcript_variant
rs1457534574	intron_variant,upstream_transcript_variant,genic_upstream_transcript_variant
rs1457545983	intron_variant,genic_upstream_transcript_variant
rs1457558684	intron_variant,genic_upstream_transcript_variant
rs1457577974	intron_variant
rs1457582738	intron_variant,upstream_transcript_variant,genic_upstream_transcript_variant
rs1457604133	intron_variant
rs1457629940	intron_variant
rs1457650213	genic_downstream_transcript_variant,intron_variant
rs1457664022	intron_variant,genic_upstream_transcript_variant
rs1457667010	intron_variant
rs1457715246	intron_variant
rs1457740665	intron_variant
rs1457776664	intron_variant
rs1457789894	intron_variant
rs1457792033	genic_downstream_transcript_variant,intron_variant
rs1457836967	genic_downstream_transcript_variant,intron_variant
rs1457881364	genic_downstream_transcript_variant,intron_variant
rs1457889926	genic_downstream_transcript_variant,intron_variant
rs1457904308	intron_variant
rs1457916654	intron_variant,upstream_transcript_variant,genic_upstream_transcript_variant
rs1457936745	intron_variant
rs1457960193	intron_variant,genic_upstream_transcript_variant
rs1457965285	genic_downstream_transcript_variant,intron_variant
rs1457971276	intron_variant
rs1458035670	genic_downstream_transcript_variant,non_coding_transcript_variant,3_prime_UTR_variant
rs1458061908	genic_downstream_transcript_variant,intron_variant,downstream_transcript_variant
rs1458077342	intron_variant,genic_upstream_transcript_variant
rs1458102755	intron_variant,2KB_upstream_variant,upstream_transcript_variant,genic_upstream_transcript_variant
rs1458104635	genic_downstream_transcript_variant,intron_variant
rs1458106027	intron_variant,genic_upstream_transcript_variant
rs1458190393	intron_variant,genic_upstream_transcript_variant
rs1458191852	intron_variant,genic_upstream_transcript_variant
rs1458218813	genic_downstream_transcript_variant,intron_variant
rs1458250645	intron_variant,genic_upstream_transcript_variant
rs1458257166	intron_variant

rs1458278491	genic_downstream_transcript_variant,intron_variant
rs1458299033	intron_variant,genic_upstream_transcript_variant
rs1458326953	intron_variant
rs1458354819	intron_variant,genic_upstream_transcript_variant
rs1458425078	intron_variant,genic_upstream_transcript_variant
rs1458442819	intron_variant
rs1458459728	intron_variant,genic_upstream_transcript_variant
rs1458491351	intron_variant
rs1458548931	intron_variant
rs1458596964	genic_downstream_transcript_variant,intron_variant
rs1458620350	intron_variant
rs1458650684	intron_variant,genic_upstream_transcript_variant
rs1458672778	intron_variant
rs1458682141	intron_variant
rs1458720632	intron_variant,genic_upstream_transcript_variant
rs1458739376	intron_variant
rs1458759372	genic_downstream_transcript_variant,intron_variant
rs1458854305	intron_variant
rs1458860221	intron_variant,upstream_transcript_variant,genic_upstream_transcript_variant
rs1458862165	intron_variant,genic_upstream_transcript_variant
rs1459015617	intron_variant
rs1459032065	intron_variant
rs1459052591	intron_variant
rs1459081145	intron_variant,genic_upstream_transcript_variant
rs1459090140	intron_variant
rs1459101565	intron_variant,genic_upstream_transcript_variant
rs1459113154	intron_variant
rs1459146190	intron_variant,genic_upstream_transcript_variant
rs1459235229	intron_variant,upstream_transcript_variant,genic_upstream_transcript_variant
rs1459249174	500B_downstream_variant,downstream_transcript_variant
rs1459262751	intron_variant
rs1459288836	genic_downstream_transcript_variant,intron_variant
rs1459310685	intron_variant,genic_upstream_transcript_variant
rs1459318675	intron_variant,genic_upstream_transcript_variant
rs1459348352	intron_variant
rs1459351012	intron_variant,upstream_transcript_variant,genic_upstream_transcript_variant
rs1459375512	genic_downstream_transcript_variant,coding_sequence_variant,non_coding_transcript_variant,missense_variant
rs1459408373	intron_variant
rs1459409168	intron_variant
rs1459425630	genic_downstream_transcript_variant,intron_variant
rs1459428771	intron_variant
rs1459438681	intron_variant,genic_upstream_transcript_variant
rs1459452972	intron_variant,genic_upstream_transcript_variant
rs1459492041	intron_variant,genic_upstream_transcript_variant
rs1459500207	intron_variant,genic_upstream_transcript_variant
rs1459548991	intron_variant
rs1459576718	intron_variant,genic_upstream_transcript_variant
rs1459642168	intron_variant,genic_upstream_transcript_variant
rs1459679818	intron_variant,genic_upstream_transcript_variant
rs1459690936	intron_variant
rs1459696201	intron_variant,genic_upstream_transcript_variant
rs1459703001	intron_variant,genic_upstream_transcript_variant
rs1459709973	intron_variant
rs1459727668	intron_variant,genic_upstream_transcript_variant
rs1459745698	intron_variant,genic_upstream_transcript_variant
rs1459801507	intron_variant,genic_upstream_transcript_variant
rs1459864578	intron_variant,genic_downstream_transcript_variant
rs1459867959	intron_variant,genic_upstream_transcript_variant
rs1459868654	intron_variant,genic_upstream_transcript_variant
rs1459878786	intron_variant
rs1459900088	intron_variant
rs1459906012	intron_variant,genic_upstream_transcript_variant
rs1459908279	intron_variant,genic_downstream_transcript_variant
rs1459925605	intron_variant,genic_downstream_transcript_variant
rs1459947806	intron_variant,genic_downstream_transcript_variant
rs1459961926	intron_variant,genic_upstream_transcript_variant
rs1459974065	intron_variant
rs1460020143	intron_variant,genic_upstream_transcript_variant
rs1460044267	intron_variant
rs1460047648	intron_variant,genic_downstream_transcript_variant
rs1460084890	intron_variant
rs1460098943	intron_variant
rs1460172025	intron_variant
rs1460222627	intron_variant,genic_downstream_transcript_variant

rs1460245148	intron_variant,genic_upstream_transcript_variant
rs1460270870	intron_variant
rs1460297952	coding_sequence_variant,intron_variant,missense_variant
rs1460325676	intron_variant,genic_downstream_transcript_variant
rs1460328831	intron_variant,genic_upstream_transcript_variant
rs1460387627	intron_variant,genic_upstream_transcript_variant
rs1460393960	intron_variant,genic_upstream_transcript_variant
rs1460414489	intron_variant
rs1460434937	intron_variant,upstream_transcript_variant,genic_upstream_transcript_variant
rs1460441653	intron_variant
rs1460459693	intron_variant
rs1460546570	intron_variant,genic_downstream_transcript_variant
rs1460602287	intron_variant,genic_upstream_transcript_variant
rs1460606924	intron_variant,genic_upstream_transcript_variant
rs1460639979	intron_variant,genic_upstream_transcript_variant
rs1460661862	intron_variant
rs1460689739	intron_variant
rs1460720526	intron_variant,genic_upstream_transcript_variant
rs1460733918	intron_variant
rs1460750267	intron_variant
rs1460770759	intron_variant,genic_upstream_transcript_variant
rs1460815408	intron_variant,genic_downstream_transcript_variant
rs1460864330	intron_variant,genic_upstream_transcript_variant
rs1460932389	intron_variant
rs1460950810	intron_variant,genic_upstream_transcript_variant
rs1460985634	intron_variant
rs1461000972	intron_variant
rs1461026483	intron_variant
rs1461034570	intron_variant
rs1461051123	intron_variant,genic_upstream_transcript_variant
rs1461079558	intron_variant
rs1461086304	intron_variant,genic_upstream_transcript_variant
rs1461130710	intron_variant,upstream_transcript_variant,genic_upstream_transcript_variant
rs1461137727	3_prime_UTR_variant,genic_downstream_transcript_variant,non_coding_transcript_variant
rs1461169405	intron_variant,upstream_transcript_variant,genic_upstream_transcript_variant
rs1461311002	intron_variant,genic_upstream_transcript_variant
rs1461326800	intron_variant,genic_upstream_transcript_variant
rs1461342072	intron_variant,genic_upstream_transcript_variant
rs1461351151	intron_variant,genic_upstream_transcript_variant
rs1461386436	intron_variant,genic_downstream_transcript_variant
rs1461414732	intron_variant,genic_upstream_transcript_variant
rs1461424951	intron_variant,genic_upstream_transcript_variant
rs1461430743	intron_variant,genic_downstream_transcript_variant
rs1461455526	non_coding_transcript_variant,intron_variant,genic_upstream_transcript_variant,synonymous_variant,coding_sequence_variant
rs1461473738	intron_variant,genic_upstream_transcript_variant
rs1461508181	intron_variant,genic_downstream_transcript_variant
rs1461530441	intron_variant
rs1461541407	intron_variant,genic_upstream_transcript_variant
rs1461605425	intron_variant,genic_upstream_transcript_variant
rs1461621226	intron_variant,genic_downstream_transcript_variant
rs1461639647	intron_variant
rs1461653958	intron_variant,genic_upstream_transcript_variant
rs1461693818	intron_variant,genic_upstream_transcript_variant
rs1461699564	intron_variant,2KB_upstream_variant,upstream_transcript_variant,genic_upstream_transcript_variant
rs1461726778	intron_variant,genic_upstream_transcript_variant
rs1461791962	non_coding_transcript_variant,intron_variant,genic_upstream_transcript_variant,synonymous_variant,coding_sequence_variant
rs1461800856	intron_variant,genic_upstream_transcript_variant
rs1461833225	intron_variant
rs1461839589	intron_variant,genic_upstream_transcript_variant
rs1461873101	intron_variant,genic_upstream_transcript_variant
rs1461888701	intron_variant,genic_upstream_transcript_variant
rs1461889338	intron_variant
rs1461891295	intron_variant
rs1461919388	5_prime_UTR_variant,intron_variant,upstream_transcript_variant,genic_upstream_transcript_variant
rs1461941704	intron_variant,genic_upstream_transcript_variant
rs1461948240	intron_variant,genic_upstream_transcript_variant
rs1461952988	intron_variant,upstream_transcript_variant,genic_upstream_transcript_variant
rs1461990383	intron_variant
rs1462002255	intron_variant,genic_upstream_transcript_variant
rs1462010873	intron_variant,genic_downstream_transcript_variant
rs1462042758	3_prime_UTR_variant,genic_downstream_transcript_variant,non_coding_transcript_variant
rs1462107934	intron_variant
rs1462131430	intron_variant
rs1462170996	intron_variant

rs1462216470	intron_variant
rs1462227665	intron_variant,genic_upstream_transcript_variant
rs1462261092	intron_variant
rs1462274166	intron_variant,genic_downstream_transcript_variant
rs1462275481	2KB_upstream_variant,intron_variant,upstream_transcript_variant,genic_upstream_transcript_variant
rs1462280421	500B_downstream_variant,downstream_transcript_variant
rs1462304982	intron_variant
rs1462324155	intron_variant,genic_upstream_transcript_variant
rs1462326115	intron_variant,genic_downstream_transcript_variant
rs1462364783	intron_variant
rs1462370046	intron_variant,genic_downstream_transcript_variant
rs1462389004	intron_variant
rs1462419939	intron_variant,genic_upstream_transcript_variant
rs1462442337	intron_variant,genic_upstream_transcript_variant
rs1462451118	intron_variant
rs1462464114	intron_variant,genic_upstream_transcript_variant
rs1462479969	intron_variant,upstream_transcript_variant,genic_upstream_transcript_variant
rs1462523690	intron_variant,genic_downstream_transcript_variant
rs1462531956	intron_variant,genic_downstream_transcript_variant
rs1462563802	intron_variant
rs1462571988	intron_variant,genic_upstream_transcript_variant
rs1462572854	intron_variant,genic_downstream_transcript_variant
rs1462595458	intron_variant,genic_downstream_transcript_variant
rs1462596481	intron_variant,genic_upstream_transcript_variant
rs1462668736	intron_variant
rs1462679646	intron_variant,genic_downstream_transcript_variant
rs1462682247	intron_variant
rs1462685299	intron_variant,genic_upstream_transcript_variant
rs1462747176	intron_variant,genic_downstream_transcript_variant
rs1462782163	intron_variant,genic_upstream_transcript_variant
rs1462827498	intron_variant
rs1462846133	intron_variant,genic_downstream_transcript_variant
rs1462872866	intron_variant,genic_upstream_transcript_variant
rs1462878254	intron_variant,genic_upstream_transcript_variant
rs1462908828	intron_variant,genic_upstream_transcript_variant
rs1462915645	intron_variant,genic_upstream_transcript_variant
rs1462926492	intron_variant,genic_upstream_transcript_variant
rs1462979460	intron_variant,genic_upstream_transcript_variant
rs1462983013	intron_variant,genic_upstream_transcript_variant
rs1463006655	intron_variant
rs1463061458	intron_variant,genic_upstream_transcript_variant
rs1463103391	intron_variant
rs1463107773	intron_variant
rs1463110673	intron_variant
rs1463156721	intron_variant,genic_upstream_transcript_variant
rs1463163813	intron_variant,genic_upstream_transcript_variant
rs1463170671	intron_variant,genic_upstream_transcript_variant
rs1463189325	intron_variant,genic_downstream_transcript_variant
rs1463192221	intron_variant,genic_downstream_transcript_variant
rs1463304790	intron_variant
rs1463307187	intron_variant,genic_upstream_transcript_variant
rs1463326088	intron_variant,genic_upstream_transcript_variant
rs1463365000	intron_variant,genic_upstream_transcript_variant
rs1463385341	intron_variant,genic_upstream_transcript_variant
rs1463388512	intron_variant,genic_downstream_transcript_variant
rs1463408020	intron_variant,genic_upstream_transcript_variant
rs1463417292	intron_variant
rs1463454709	intron_variant,genic_upstream_transcript_variant
rs1463467244	intron_variant
rs1463556588	intron_variant
rs1463686070	intron_variant
rs1463706195	intron_variant
rs1463710285	intron_variant
rs1463746323	intron_variant
rs1463785268	intron_variant,genic_upstream_transcript_variant
rs1463803326	intron_variant,upstream_transcript_variant,genic_upstream_transcript_variant
rs1463831768	intron_variant
rs1463832071	intron_variant,genic_upstream_transcript_variant
rs1463842202	intron_variant,genic_upstream_transcript_variant
rs1463844879	intron_variant,genic_downstream_transcript_variant
rs1463875922	intron_variant
rs1463895850	intron_variant,genic_upstream_transcript_variant
rs1463969183	intron_variant
rs1463971718	intron_variant,genic_upstream_transcript_variant

rs1464006797	intron_variant,genic_upstream_transcript_variant
rs1464044444	intron_variant,genic_upstream_transcript_variant
rs1464115678	intron_variant
rs1464129513	intron_variant,genic_upstream_transcript_variant
rs1464243770	intron_variant,genic_downstream_transcript_variant
rs1464294611	2KB_upstream_variant,intron_variant,upstream_transcript_variant,genic_upstream_transcript_variant
rs1464298377	intron_variant,genic_downstream_transcript_variant
rs1464316950	coding_sequence_variant,genic_downstream_transcript_variant,missense_variant,non_coding_transcript_variant
rs1464364125	intron_variant
rs1464366727	intron_variant,genic_downstream_transcript_variant
rs1464367699	intron_variant,2KB_upstream_variant,upstream_transcript_variant,genic_upstream_transcript_variant
rs1464377766	intron_variant,genic_upstream_transcript_variant
rs1464412119	intron_variant
rs1464480155	intron_variant,genic_upstream_transcript_variant
rs1464494783	intron_variant,genic_upstream_transcript_variant
rs1464512782	intron_variant
rs1464528009	intron_variant,genic_upstream_transcript_variant
rs1464539654	intron_variant
rs1464599991	500B_downstream_variant,downstream_transcript_variant
rs1464675965	intron_variant,upstream_transcript_variant,genic_upstream_transcript_variant
rs1464710160	intron_variant
rs1464710536	intron_variant,genic_downstream_transcript_variant
rs1464741838	intron_variant,genic_upstream_transcript_variant
rs1464774656	intron_variant,upstream_transcript_variant,genic_upstream_transcript_variant
rs1464794785	intron_variant,genic_upstream_transcript_variant
rs1464806790	intron_variant,genic_downstream_transcript_variant
rs1464813176	intron_variant,genic_upstream_transcript_variant
rs1464841967	intron_variant,genic_upstream_transcript_variant
rs1464883835	intron_variant
rs1464902800	intron_variant,genic_downstream_transcript_variant
rs1464905123	intron_variant
rs1464940213	intron_variant,genic_downstream_transcript_variant
rs1464940411	intron_variant,genic_upstream_transcript_variant
rs1464971320	intron_variant,upstream_transcript_variant,genic_upstream_transcript_variant
rs1464974627	intron_variant
rs1465023725	intron_variant,genic_upstream_transcript_variant
rs1465027918	intron_variant,genic_upstream_transcript_variant
rs1465039698	intron_variant,2KB_upstream_variant,upstream_transcript_variant,genic_upstream_transcript_variant
rs1465041382	intron_variant,genic_downstream_transcript_variant
rs1465075389	2KB_upstream_variant,intron_variant,upstream_transcript_variant,genic_upstream_transcript_variant
rs1465075762	intron_variant,genic_upstream_transcript_variant
rs1465086873	intron_variant,2KB_upstream_variant,upstream_transcript_variant,genic_upstream_transcript_variant
rs1465154711	intron_variant,genic_upstream_transcript_variant
rs1465169162	intron_variant,genic_downstream_transcript_variant
rs1465205432	intron_variant,genic_downstream_transcript_variant
rs1465205467	intron_variant
rs1465226832	intron_variant,genic_downstream_transcript_variant
rs1465257935	intron_variant
rs1465265706	intron_variant,genic_downstream_transcript_variant
rs1465274653	intron_variant,genic_downstream_transcript_variant
rs1465288758	intron_variant,genic_upstream_transcript_variant
rs1465343954	intron_variant,genic_upstream_transcript_variant
rs1465362793	intron_variant,genic_upstream_transcript_variant
rs1465396774	intron_variant,genic_upstream_transcript_variant
rs1465400690	intron_variant,genic_upstream_transcript_variant
rs1465422519	intron_variant
rs1465439333	intron_variant
rs1465444752	intron_variant,genic_upstream_transcript_variant
rs1465488249	5_prime_UTR_variant,intron_variant
rs1465556948	intron_variant,genic_downstream_transcript_variant
rs1465558657	intron_variant,genic_upstream_transcript_variant
rs1465558921	intron_variant,genic_upstream_transcript_variant
rs1465589330	intron_variant,genic_downstream_transcript_variant
rs1465616849	intron_variant
rs1465639408	2KB_upstream_variant,intron_variant,upstream_transcript_variant,genic_upstream_transcript_variant
rs1465694041	intron_variant,genic_upstream_transcript_variant
rs1465710400	intron_variant,genic_upstream_transcript_variant
rs1465772712	coding_sequence_variant,intron_variant,missense_variant
rs1465774539	intron_variant,genic_downstream_transcript_variant
rs1465783185	intron_variant
rs1465840199	intron_variant,genic_upstream_transcript_variant
rs1465914503	intron_variant,genic_downstream_transcript_variant
rs1465922987	intron_variant
rs1465955052	intron_variant,genic_downstream_transcript_variant

rs1465972466	intron_variant
rs1466016328	intron_variant
rs1466067583	intron_variant,upstream_transcript_variant,genic_upstream_transcript_variant
rs1466079200	3_prime_UTR_variant,genic_downstream_transcript_variant,non_coding_transcript_variant
rs1466109590	intron_variant
rs1466119538	intron_variant,genic_upstream_transcript_variant
rs1466141075	intron_variant,genic_downstream_transcript_variant
rs1466155160	intron_variant,genic_upstream_transcript_variant
rs1466176468	intron_variant,genic_downstream_transcript_variant
rs1466185958	intron_variant
rs1466203731	intron_variant,genic_upstream_transcript_variant
rs1466206092	intron_variant,genic_upstream_transcript_variant
rs1466220589	intron_variant,genic_upstream_transcript_variant
rs1466239143	intron_variant
rs1466243339	intron_variant
rs1466259366	intron_variant,genic_downstream_transcript_variant
rs1466280159	intron_variant,genic_downstream_transcript_variant
rs1466292233	intron_variant,genic_upstream_transcript_variant
rs1466329205	intron_variant,upstream_transcript_variant,genic_upstream_transcript_variant
rs1466330032	intron_variant,genic_upstream_transcript_variant
rs1466380514	intron_variant
rs1466390869	intron_variant
rs1466407812	intron_variant,genic_upstream_transcript_variant
rs1466427644	intron_variant,genic_upstream_transcript_variant
rs1466473303	intron_variant
rs1466501881	intron_variant,genic_upstream_transcript_variant
rs1466561661	intron_variant
rs1466617948	intron_variant,genic_downstream_transcript_variant
rs1466644366	intron_variant,genic_upstream_transcript_variant
rs1466653458	intron_variant
rs1466655581	intron_variant
rs1466661428	intron_variant,genic_upstream_transcript_variant
rs1466665259	intron_variant,genic_downstream_transcript_variant
rs1466711316	intron_variant,genic_downstream_transcript_variant
rs1466745130	intron_variant,genic_downstream_transcript_variant
rs1466796044	intron_variant,genic_upstream_transcript_variant
rs1466797520	intron_variant,genic_downstream_transcript_variant
rs1466835849	intron_variant,genic_downstream_transcript_variant
rs1466838059	intron_variant,genic_upstream_transcript_variant
rs1466851761	intron_variant
rs1466861999	intron_variant,genic_downstream_transcript_variant
rs1466876676	intron_variant,genic_downstream_transcript_variant
rs1466900141	intron_variant
rs1466908355	intron_variant
rs1466920538	intron_variant,genic_upstream_transcript_variant
rs1466954854	intron_variant
rs1466975132	coding_sequence_variant,genic_downstream_transcript_variant,missense_variant,non_coding_transcript_variant
rs1466981738	intron_variant,genic_upstream_transcript_variant
rs1466990384	intron_variant,genic_upstream_transcript_variant
rs1467034893	intron_variant
rs1467080418	intron_variant
rs1467122527	intron_variant,genic_upstream_transcript_variant
rs1467148213	intron_variant
rs1467182961	intron_variant,genic_downstream_transcript_variant
rs1467201832	intron_variant
rs1467232376	intron_variant,genic_upstream_transcript_variant
rs1467257264	intron_variant,genic_downstream_transcript_variant
rs1467270255	intron_variant,genic_upstream_transcript_variant
rs1467285006	intron_variant,genic_upstream_transcript_variant
rs1467363280	intron_variant
rs1467378333	intron_variant,genic_upstream_transcript_variant
rs1467386450	intron_variant,genic_downstream_transcript_variant
rs1467390719	intron_variant
rs1467400378	intron_variant
rs1467450377	intron_variant
rs1467473539	2KB_upstream_variant,intron_variant,upstream_transcript_variant,genic_upstream_transcript_variant
rs1467502349	3_prime_UTR_variant,genic_downstream_transcript_variant,non_coding_transcript_variant
rs1467558620	intron_variant,genic_upstream_transcript_variant
rs1467580409	intron_variant
rs1467591850	intron_variant
rs1467641584	intron_variant,genic_upstream_transcript_variant
rs1467685481	intron_variant
rs1467708351	intron_variant
rs1467747056	intron_variant

rs1467752811	2KB_upstream_variant,intron_variant,upstream_transcript_variant,genic_upstream_transcript_variant
rs1467770793	intron_variant,genic_upstream_transcript_variant
rs1467807406	intron_variant
rs1467841989	intron_variant,genic_downstream_transcript_variant
rs1467905809	intron_variant,genic_upstream_transcript_variant
rs1467908146	intron_variant
rs1467925436	intron_variant,genic_upstream_transcript_variant
rs1467932717	intron_variant,genic_upstream_transcript_variant
rs1467947805	intron_variant,genic_upstream_transcript_variant
rs1467970764	intron_variant
rs1467993050	intron_variant,genic_upstream_transcript_variant
rs1468013917	intron_variant
rs1468117649	intron_variant,genic_upstream_transcript_variant
rs1468122493	intron_variant,genic_upstream_transcript_variant
rs1468142470	intron_variant,genic_downstream_transcript_variant
rs1468150901	intron_variant
rs1468181151	intron_variant,genic_upstream_transcript_variant
rs1468218545	intron_variant
rs1468238923	intron_variant,genic_upstream_transcript_variant
rs1468282623	intron_variant,genic_downstream_transcript_variant
rs1468288017	intron_variant,genic_upstream_transcript_variant
rs1468308844	intron_variant
rs1468318098	intron_variant,genic_upstream_transcript_variant
rs1468397049	intron_variant
rs1468407496	intron_variant,genic_upstream_transcript_variant
rs1468459468	non_coding_transcript_variant,intron_variant,genic_upstream_transcript_variant,missense_variant,coding_sequence_variant
rs1468465677	intron_variant,genic_upstream_transcript_variant
rs1468466503	intron_variant,genic_upstream_transcript_variant
rs1468580382	intron_variant,upstream_transcript_variant,genic_upstream_transcript_variant
rs1468619955	intron_variant,genic_downstream_transcript_variant
rs1468629302	intron_variant,genic_upstream_transcript_variant
rs1468629590	intron_variant,genic_upstream_transcript_variant
rs1468646080	intron_variant,genic_downstream_transcript_variant
rs1468670132	intron_variant
rs1468675612	intron_variant,genic_upstream_transcript_variant
rs1468694351	intron_variant,genic_upstream_transcript_variant
rs1468750037	intron_variant,genic_upstream_transcript_variant
rs1468761215	500B_downstream_variant,downstream_transcript_variant
rs1468784627	intron_variant,genic_downstream_transcript_variant
rs1468842953	intron_variant,genic_upstream_transcript_variant
rs1468858960	intron_variant,genic_downstream_transcript_variant
rs1468883069	intron_variant,genic_downstream_transcript_variant
rs1468900124	intron_variant,genic_upstream_transcript_variant
rs1468907294	intron_variant,genic_downstream_transcript_variant
rs1468908651	intron_variant,genic_upstream_transcript_variant
rs1468922880	intron_variant,genic_downstream_transcript_variant
rs1468927264	intron_variant
rs1468932446	intron_variant,genic_upstream_transcript_variant
rs1468969943	intron_variant,genic_upstream_transcript_variant
rs1468997280	intron_variant,genic_upstream_transcript_variant
rs1469017841	intron_variant,genic_downstream_transcript_variant
rs1469053155	intron_variant,genic_upstream_transcript_variant
rs1469085272	intron_variant,genic_upstream_transcript_variant
rs1469114061	intron_variant
rs1469137149	intron_variant
rs1469145170	intron_variant,genic_upstream_transcript_variant
rs1469157034	intron_variant,genic_upstream_transcript_variant
rs1469206152	intron_variant
rs1469240995	intron_variant,genic_downstream_transcript_variant
rs1469256390	intron_variant,genic_upstream_transcript_variant
rs1469268827	intron_variant,genic_upstream_transcript_variant
rs1469296848	intron_variant,genic_upstream_transcript_variant
rs1469352267	intron_variant
rs1469375833	intron_variant
rs1469412433	intron_variant,genic_upstream_transcript_variant
rs1469424373	intron_variant
rs1469436690	3_prime_UTR_variant,genic_downstream_transcript_variant,non_coding_transcript_variant
rs1469467557	intron_variant,genic_upstream_transcript_variant
rs1469473186	intron_variant,genic_upstream_transcript_variant
rs1469495263	intron_variant,genic_upstream_transcript_variant
rs1469501265	intron_variant,genic_downstream_transcript_variant
rs1469502447	intron_variant,genic_upstream_transcript_variant
rs1469533408	intron_variant
rs1469574734	intron_variant,genic_upstream_transcript_variant

rs1469589161	intron_variant
rs1469699400	intron_variant
rs1469731299	intron_variant
rs1469731457	intron_variant
rs1469731536	intron_variant,genic_upstream_transcript_variant
rs1469799175	intron_variant,genic_downstream_transcript_variant
rs1469858336	intron_variant
rs1469980580	intron_variant
rs1469985468	intron_variant,genic_upstream_transcript_variant
rs1470052672	intron_variant
rs1470068059	intron_variant,genic_downstream_transcript_variant
rs1470092273	intron_variant,genic_downstream_transcript_variant
rs1470104314	intron_variant
rs1470120029	intron_variant,genic_upstream_transcript_variant
rs1470135868	intron_variant,genic_downstream_transcript_variant
rs1470170471	intron_variant,genic_upstream_transcript_variant
rs1470176691	2KB_upstream_variant,intron_variant,upstream_transcript_variant,genic_upstream_transcript_variant
rs1470219848	intron_variant,genic_upstream_transcript_variant
rs1470253374	intron_variant,genic_downstream_transcript_variant
rs1470263389	intron_variant,genic_upstream_transcript_variant
rs1470295493	intron_variant,genic_upstream_transcript_variant
rs1470300650	intron_variant,genic_downstream_transcript_variant
rs1470352352	intron_variant
rs1470422251	intron_variant,genic_downstream_transcript_variant
rs1470428804	intron_variant,genic_upstream_transcript_variant
rs1470441125	intron_variant,genic_downstream_transcript_variant
rs1470450401	intron_variant,genic_downstream_transcript_variant
rs1470531889	intron_variant,genic_upstream_transcript_variant
rs1470547247	intron_variant
rs1470549606	intron_variant
rs1470581064	intron_variant
rs1470586005	intron_variant,genic_upstream_transcript_variant
rs1470609134	intron_variant,genic_upstream_transcript_variant
rs1470615388	intron_variant,genic_upstream_transcript_variant
rs1470617695	intron_variant,genic_upstream_transcript_variant
rs1470727099	intron_variant
rs1470738104	intron_variant
rs1470742926	intron_variant
rs1470763390	5_prime_UTR_variant,intron_variant,upstream_transcript_variant,genic_upstream_transcript_variant
rs1470774539	intron_variant
rs1470792435	intron_variant,genic_downstream_transcript_variant
rs1470843083	intron_variant,upstream_transcript_variant,genic_upstream_transcript_variant
rs1470854234	intron_variant
rs1470868620	intron_variant,genic_downstream_transcript_variant
rs1470875305	intron_variant
rs1470883300	intron_variant
rs1470927368	intron_variant
rs1470938144	intron_variant
rs1470949600	intron_variant,genic_upstream_transcript_variant
rs1470951861	intron_variant
rs1470998796	intron_variant,genic_upstream_transcript_variant
rs1471017193	intron_variant,upstream_transcript_variant,genic_upstream_transcript_variant
rs1471027579	intron_variant,genic_upstream_transcript_variant
rs1471052912	intron_variant
rs1471058108	intron_variant,genic_upstream_transcript_variant
rs1471072231	intron_variant,genic_downstream_transcript_variant
rs1471084645	intron_variant
rs1471095987	intron_variant
rs1471124167	intron_variant,genic_upstream_transcript_variant
rs1471165985	intron_variant
rs1471197190	intron_variant,genic_upstream_transcript_variant
rs1471202319	intron_variant,genic_upstream_transcript_variant
rs1471206255	intron_variant,genic_upstream_transcript_variant
rs1471217530	intron_variant
rs1471243231	intron_variant
rs1471266287	intron_variant,genic_downstream_transcript_variant
rs1471297164	intron_variant,genic_upstream_transcript_variant
rs1471303322	intron_variant,genic_upstream_transcript_variant
rs1471316786	intron_variant
rs1471339022	intron_variant,genic_downstream_transcript_variant
rs1471370270	intron_variant,genic_downstream_transcript_variant
rs1471375204	intron_variant
rs1471402205	intron_variant
rs1471412621	intron_variant,genic_upstream_transcript_variant

rs1471443861	intron_variant,genic_upstream_transcript_variant
rs1471475217	intron_variant,genic_upstream_transcript_variant
rs1471526693	intron_variant,genic_upstream_transcript_variant
rs1471550637	intron_variant,genic_upstream_transcript_variant
rs1471565340	intron_variant
rs1471596756	intron_variant
rs1471617198	intron_variant
rs1471629689	intron_variant,genic_downstream_transcript_variant
rs1471634317	intron_variant,genic_upstream_transcript_variant
rs1471639389	intron_variant,genic_downstream_transcript_variant
rs1471660917	intron_variant,genic_upstream_transcript_variant
rs1471705418	intron_variant
rs1471715092	intron_variant
rs1471757345	intron_variant,genic_downstream_transcript_variant
rs1471758147	intron_variant
rs1471765530	intron_variant
rs1471771231	non_coding_transcript_variant,intron_variant,genic_upstream_transcript_variant,missense_variant,coding_sequence_variant
rs1471801414	intron_variant,genic_upstream_transcript_variant
rs1471829083	intron_variant,genic_upstream_transcript_variant
rs1471872109	intron_variant,genic_upstream_transcript_variant
rs1471885354	intron_variant,genic_upstream_transcript_variant
rs1471888493	intron_variant
rs1471890312	intron_variant
rs1471919853	intron_variant,genic_downstream_transcript_variant
rs1471923598	intron_variant,genic_upstream_transcript_variant
rs1471946232	intron_variant
rs1471949329	intron_variant
rs1471962014	intron_variant,upstream_transcript_variant,genic_upstream_transcript_variant
rs1471993320	intron_variant
rs1472014282	intron_variant,genic_upstream_transcript_variant
rs1472030890	intron_variant,genic_upstream_transcript_variant
rs1472040277	intron_variant,genic_downstream_transcript_variant
rs1472107669	intron_variant,genic_upstream_transcript_variant
rs1472112096	intron_variant,genic_downstream_transcript_variant
rs1472133668	intron_variant
rs1472155004	intron_variant
rs1472158181	intron_variant,genic_upstream_transcript_variant
rs1472165769	intron_variant,genic_downstream_transcript_variant
rs1472200730	intron_variant,genic_downstream_transcript_variant
rs1472239992	intron_variant
rs1472254090	3_prime_UTR_variant,genic_downstream_transcript_variant,non_coding_transcript_variant
rs1472301553	intron_variant
rs1472310046	intron_variant
rs1472312326	intron_variant,genic_upstream_transcript_variant
rs1472337574	intron_variant,genic_downstream_transcript_variant
rs1472363479	intron_variant,genic_upstream_transcript_variant
rs1472373417	intron_variant,genic_downstream_transcript_variant
rs1472379337	intron_variant,genic_upstream_transcript_variant
rs1472417343	intron_variant,genic_upstream_transcript_variant
rs1472439557	intron_variant,genic_upstream_transcript_variant
rs1472471154	intron_variant,genic_upstream_transcript_variant
rs1472521190	intron_variant,genic_upstream_transcript_variant,upstream_transcript_variant
rs1472597977	intron_variant,genic_upstream_transcript_variant
rs1472721419	intron_variant,2KB_upstream_variant,genic_upstream_transcript_variant,upstream_transcript_variant
rs1472749202	intron_variant
rs1472776181	intron_variant,genic_upstream_transcript_variant
rs1472796639	intron_variant,genic_upstream_transcript_variant
rs1472812687	intron_variant
rs1472818144	intron_variant
rs1472825053	intron_variant,genic_downstream_transcript_variant
rs1472848287	intron_variant
rs1472894791	intron_variant,genic_upstream_transcript_variant
rs1472908014	intron_variant,genic_upstream_transcript_variant
rs1472946515	intron_variant,genic_upstream_transcript_variant
rs1472948931	intron_variant
rs1472955637	intron_variant
rs1472964154	intron_variant,genic_upstream_transcript_variant
rs1472988780	intron_variant,genic_downstream_transcript_variant
rs1473009267	intron_variant,genic_upstream_transcript_variant
rs1473024577	intron_variant,genic_downstream_transcript_variant
rs1473030685	intron_variant
rs1473108158	intron_variant,genic_upstream_transcript_variant
rs1473110442	intron_variant
rs1473172801	intron_variant

rs1473194512	intron_variant
rs1473203091	intron_variant,genic_downstream_transcript_variant
rs1473211467	intron_variant
rs1473212769	intron_variant,downstream_transcript_variant,genic_downstream_transcript_variant
rs1473283918	intron_variant
rs1473293845	intron_variant,genic_upstream_transcript_variant
rs1473331089	intron_variant,genic_upstream_transcript_variant,upstream_transcript_variant
rs1473331187	intron_variant,genic_upstream_transcript_variant
rs1473372031	intron_variant,genic_upstream_transcript_variant
rs1473375943	intron_variant
rs1473383342	intron_variant
rs1473401025	intron_variant,genic_upstream_transcript_variant
rs1473409460	intron_variant,genic_upstream_transcript_variant
rs1473416923	intron_variant,genic_upstream_transcript_variant
rs1473470938	intron_variant,genic_downstream_transcript_variant
rs1473509273	intron_variant
rs1473563032	intron_variant,genic_upstream_transcript_variant,upstream_transcript_variant
rs1473599659	intron_variant,genic_downstream_transcript_variant
rs1473666614	intron_variant
rs1473725374	intron_variant,genic_downstream_transcript_variant
rs1473725949	intron_variant
rs1473751270	intron_variant
rs1473797220	intron_variant,genic_downstream_transcript_variant
rs1473904760	intron_variant,genic_downstream_transcript_variant
rs1473931446	intron_variant,genic_upstream_transcript_variant
rs1474014047	intron_variant
rs1474057520	intron_variant
rs1474092743	intron_variant,genic_upstream_transcript_variant
rs1474104670	intron_variant
rs1474112787	intron_variant,genic_upstream_transcript_variant,upstream_transcript_variant
rs1474129454	intron_variant,genic_upstream_transcript_variant
rs1474137238	intron_variant,genic_upstream_transcript_variant
rs1474207174	intron_variant,genic_upstream_transcript_variant
rs1474230051	intron_variant,genic_upstream_transcript_variant
rs1474271700	intron_variant,genic_downstream_transcript_variant
rs1474325414	intron_variant,genic_upstream_transcript_variant
rs1474352691	intron_variant,genic_upstream_transcript_variant
rs1474395856	intron_variant,genic_downstream_transcript_variant
rs1474428051	intron_variant
rs1474469603	intron_variant,genic_downstream_transcript_variant
rs1474481016	intron_variant
rs1474524549	intron_variant
rs1474554445	intron_variant,genic_upstream_transcript_variant,2KB_upstream_variant,upstream_transcript_variant
rs1474563324	intron_variant
rs1474571914	intron_variant,genic_upstream_transcript_variant
rs1474648846	intron_variant,genic_upstream_transcript_variant
rs1474685739	intron_variant,genic_upstream_transcript_variant
rs1474751718	intron_variant,genic_downstream_transcript_variant
rs1474779476	intron_variant,genic_downstream_transcript_variant
rs1474796371	intron_variant,genic_upstream_transcript_variant
rs1474822316	intron_variant,genic_upstream_transcript_variant
rs1474834778	intron_variant,genic_downstream_transcript_variant
rs1474855429	intron_variant
rs1474872589	intron_variant
rs1474882149	intron_variant,genic_downstream_transcript_variant
rs1474882221	intron_variant
rs1474905647	intron_variant,genic_downstream_transcript_variant
rs1474916773	intron_variant,genic_downstream_transcript_variant
rs1474965042	intron_variant
rs1474970042	intron_variant,genic_upstream_transcript_variant
rs1474993228	intron_variant
rs1475034564	intron_variant,genic_downstream_transcript_variant
rs1475042140	intron_variant
rs1475063748	intron_variant
rs1475076306	intron_variant,genic_upstream_transcript_variant,2KB_upstream_variant,upstream_transcript_variant
rs1475083268	intron_variant,genic_upstream_transcript_variant
rs1475092028	intron_variant
rs1475118987	intron_variant
rs1475127248	intron_variant,genic_downstream_transcript_variant
rs1475143584	intron_variant
rs1475149954	intron_variant,genic_upstream_transcript_variant
rs1475200939	intron_variant,genic_upstream_transcript_variant
rs1475241559	intron_variant,genic_upstream_transcript_variant,upstream_transcript_variant
rs1475250577	intron_variant,genic_downstream_transcript_variant

rs1475253659	intron_variant,genic_downstream_transcript_variant
rs1475257408	intron_variant,genic_upstream_transcript_variant
rs1475263734	intron_variant
rs1475295308	intron_variant
rs1475309527	intron_variant,genic_upstream_transcript_variant
rs1475335367	intron_variant,genic_upstream_transcript_variant
rs1475340254	intron_variant
rs1475341195	non_coding_transcript_variant,3_prime_UTR_variant,genic_downstream_transcript_variant
rs1475364488	intron_variant,genic_upstream_transcript_variant
rs1475386083	intron_variant,genic_downstream_transcript_variant
rs1475388757	intron_variant,genic_downstream_transcript_variant
rs1475417421	intron_variant,genic_downstream_transcript_variant
rs1475452714	intron_variant,genic_downstream_transcript_variant
rs1475460487	intron_variant,2KB_upstream_variant,genic_upstream_transcript_variant,upstream_transcript_variant
rs1475475983	intron_variant,genic_upstream_transcript_variant
rs1475488646	intron_variant,genic_downstream_transcript_variant
rs1475504718	intron_variant
rs1475523402	intron_variant
rs1475551041	intron_variant,genic_upstream_transcript_variant
rs1475558388	intron_variant,genic_upstream_transcript_variant,upstream_transcript_variant
rs1475568194	intron_variant
rs1475593851	intron_variant,genic_upstream_transcript_variant
rs1475623284	intron_variant,genic_upstream_transcript_variant
rs1475700186	intron_variant,genic_upstream_transcript_variant,2KB_upstream_variant,upstream_transcript_variant
rs1475706415	intron_variant,genic_upstream_transcript_variant
rs1475746759	intron_variant
rs1475792756	intron_variant,genic_upstream_transcript_variant
rs1475793487	missense_variant,non_coding_transcript_variant,5_prime_UTR_variant,intron_variant,genic_upstream_transcript_variant,coding_sequence_variant
rs1475849844	intron_variant,genic_upstream_transcript_variant
rs1475864834	intron_variant,genic_upstream_transcript_variant
rs1475879333	intron_variant,genic_downstream_transcript_variant
rs1475899463	intron_variant
rs1475906870	intron_variant,genic_downstream_transcript_variant
rs1475953534	intron_variant
rs1475963796	intron_variant
rs1475972083	intron_variant,genic_downstream_transcript_variant
rs1475974266	intron_variant,genic_upstream_transcript_variant
rs1475982538	intron_variant,genic_upstream_transcript_variant
rs1475996706	intron_variant,genic_upstream_transcript_variant
rs1476047036	intron_variant,genic_upstream_transcript_variant
rs1476090447	intron_variant,genic_upstream_transcript_variant
rs1476091589	intron_variant,genic_upstream_transcript_variant
rs1476123942	intron_variant,genic_upstream_transcript_variant
rs1476137150	intron_variant
rs1476167998	intron_variant,genic_upstream_transcript_variant
rs1476171184	intron_variant,genic_downstream_transcript_variant
rs1476174048	intron_variant,genic_upstream_transcript_variant
rs1476181476	non_coding_transcript_variant,synonymous_variant,intron_variant,genic_upstream_transcript_variant,coding_sequence_variant
rs1476211668	intron_variant
rs1476230479	intron_variant,genic_upstream_transcript_variant
rs1476243774	intron_variant
rs1476250568	intron_variant,genic_upstream_transcript_variant
rs1476298502	intron_variant
rs1476318059	intron_variant,genic_upstream_transcript_variant
rs1476359557	intron_variant,genic_downstream_transcript_variant
rs1476376319	intron_variant
rs1476392094	intron_variant,genic_upstream_transcript_variant
rs1476422089	intron_variant
rs1476486510	intron_variant
rs1476503072	intron_variant,genic_upstream_transcript_variant
rs1476527441	intron_variant,genic_downstream_transcript_variant
rs1476535640	intron_variant
rs1476539762	intron_variant
rs1476580184	intron_variant,genic_upstream_transcript_variant
rs1476599154	intron_variant,genic_upstream_transcript_variant
rs1476621236	intron_variant
rs1476632186	intron_variant
rs1476663078	intron_variant
rs1476695921	intron_variant
rs1476723911	intron_variant,genic_downstream_transcript_variant
rs1476747674	intron_variant
rs1476751135	intron_variant
rs1476752378	intron_variant,genic_downstream_transcript_variant
rs1476793520	intron_variant,genic_downstream_transcript_variant

rs1476808645	intron_variant
rs1476831644	intron_variant
rs1476901924	intron_variant,genic_downstream_transcript_variant
rs1476905200	intron_variant
rs1476907147	intron_variant
rs1476913593	intron_variant,genic_upstream_transcript_variant,2KB_upstream_variant,upstream_transcript_variant
rs1476962956	intron_variant
rs1476968208	intron_variant,genic_upstream_transcript_variant
rs1477011944	intron_variant
rs1477030491	intron_variant,genic_upstream_transcript_variant
rs1477045700	intron_variant,genic_upstream_transcript_variant
rs1477052573	intron_variant
rs1477068130	intron_variant,genic_upstream_transcript_variant
rs1477086510	intron_variant
rs1477100442	intron_variant,genic_upstream_transcript_variant,upstream_transcript_variant
rs1477129221	intron_variant
rs1477203465	intron_variant
rs1477206868	intron_variant,genic_upstream_transcript_variant
rs1477211170	intron_variant,2KB_upstream_variant,genic_upstream_transcript_variant,upstream_transcript_variant
rs1477241899	intron_variant
rs1477242339	intron_variant
rs1477248197	intron_variant
rs1477334210	intron_variant,genic_downstream_transcript_variant
rs1477411494	intron_variant
rs1477416653	intron_variant
rs1477440268	intron_variant,genic_upstream_transcript_variant,upstream_transcript_variant
rs1477450780	intron_variant,genic_upstream_transcript_variant
rs1477525299	intron_variant
rs1477577123	intron_variant
rs1477602415	intron_variant,genic_upstream_transcript_variant
rs1477627936	intron_variant,genic_upstream_transcript_variant
rs1477730068	intron_variant,genic_downstream_transcript_variant
rs1477780615	intron_variant,genic_upstream_transcript_variant
rs1477798277	intron_variant,genic_downstream_transcript_variant
rs1477804860	intron_variant,genic_upstream_transcript_variant
rs1477805888	intron_variant,genic_upstream_transcript_variant
rs1477858346	intron_variant,genic_upstream_transcript_variant
rs1477863411	intron_variant,downstream_transcript_variant,genic_downstream_transcript_variant
rs1477873614	intron_variant,genic_upstream_transcript_variant,5_prime_UTR_variant,upstream_transcript_variant
rs1477900424	intron_variant,genic_upstream_transcript_variant,2KB_upstream_variant,upstream_transcript_variant
rs1477903538	intron_variant,genic_upstream_transcript_variant
rs1477934215	intron_variant,genic_upstream_transcript_variant
rs1477950465	intron_variant,genic_upstream_transcript_variant
rs1477958599	intron_variant
rs1477971577	intron_variant,genic_upstream_transcript_variant,2KB_upstream_variant,upstream_transcript_variant
rs1477982508	intron_variant
rs1477999566	intron_variant,genic_downstream_transcript_variant
rs1478014787	intron_variant,genic_upstream_transcript_variant
rs1478034359	intron_variant,genic_upstream_transcript_variant
rs1478054694	intron_variant,genic_upstream_transcript_variant,2KB_upstream_variant,upstream_transcript_variant
rs1478064081	intron_variant
rs1478076619	intron_variant
rs1478094108	intron_variant
rs1478123754	intron_variant
rs1478141567	intron_variant,genic_upstream_transcript_variant
rs1478165479	intron_variant,genic_upstream_transcript_variant
rs1478169977	intron_variant
rs1478171934	intron_variant
rs1478177098	intron_variant
rs1478193657	intron_variant,genic_upstream_transcript_variant
rs1478196236	intron_variant
rs1478211081	intron_variant
rs1478240562	intron_variant
rs1478245295	intron_variant,genic_upstream_transcript_variant
rs1478309709	intron_variant
rs1478341454	intron_variant,genic_upstream_transcript_variant
rs1478342550	intron_variant,genic_downstream_transcript_variant
rs1478354144	intron_variant,genic_upstream_transcript_variant,upstream_transcript_variant
rs1478365783	intron_variant
rs1478387407	intron_variant,genic_downstream_transcript_variant
rs1478413316	intron_variant,genic_downstream_transcript_variant
rs1478417758	intron_variant,genic_upstream_transcript_variant,upstream_transcript_variant
rs1478445084	intron_variant,genic_upstream_transcript_variant
rs1478459069	intron_variant,genic_upstream_transcript_variant

rs1478460600	intron_variant,genic_upstream_transcript_variant
rs1478529145	intron_variant,genic_downstream_transcript_variant
rs1478539017	intron_variant,genic_downstream_transcript_variant
rs1478575668	intron_variant
rs1478591342	intron_variant
rs1478616291	intron_variant,genic_upstream_transcript_variant
rs1478651227	intron_variant,genic_upstream_transcript_variant
rs1478713190	intron_variant,genic_upstream_transcript_variant
rs1478714826	intron_variant,genic_downstream_transcript_variant
rs1478724742	intron_variant,genic_upstream_transcript_variant
rs1478751211	intron_variant,genic_upstream_transcript_variant
rs1478756815	intron_variant
rs1478813703	intron_variant,genic_upstream_transcript_variant
rs1478821526	intron_variant,genic_upstream_transcript_variant
rs1478857697	intron_variant,genic_upstream_transcript_variant
rs1478883319	intron_variant
rs1478887285	intron_variant,genic_upstream_transcript_variant
rs1478934235	intron_variant,genic_upstream_transcript_variant
rs1478944328	downstream_transcript_variant,500B_downstream_variant
rs1478952827	intron_variant,genic_upstream_transcript_variant
rs1479053902	intron_variant
rs1479092984	non_coding_transcript_variant,3_prime_UTR_variant,genic_downstream_transcript_variant
rs1479103687	intron_variant
rs1479124638	intron_variant,genic_downstream_transcript_variant
rs1479175339	intron_variant,5_prime_UTR_variant
rs1479217784	intron_variant,genic_upstream_transcript_variant
rs1479224444	intron_variant
rs1479258351	intron_variant,genic_upstream_transcript_variant,2KB_upstream_variant,upstream_transcript_variant
rs1479284540	intron_variant
rs1479315080	intron_variant,genic_downstream_transcript_variant
rs1479375451	intron_variant,genic_downstream_transcript_variant
rs1479405538	intron_variant,genic_upstream_transcript_variant
rs1479425814	intron_variant
rs1479427022	intron_variant
rs1479431690	intron_variant
rs1479443815	intron_variant,2KB_upstream_variant,genic_upstream_transcript_variant,upstream_transcript_variant
rs1479460697	intron_variant,genic_upstream_transcript_variant
rs1479471931	intron_variant
rs1479473084	intron_variant
rs1479499793	intron_variant
rs1479570605	intron_variant
rs1479625993	intron_variant
rs1479662080	intron_variant
rs1479674474	intron_variant,genic_upstream_transcript_variant
rs1479677265	intron_variant,genic_upstream_transcript_variant
rs1479691783	intron_variant,genic_downstream_transcript_variant
rs1479699442	intron_variant
rs1479757854	non_coding_transcript_variant,3_prime_UTR_variant,genic_downstream_transcript_variant
rs1479781564	intron_variant,genic_upstream_transcript_variant,upstream_transcript_variant
rs1479812968	intron_variant,genic_upstream_transcript_variant
rs1479821329	intron_variant,genic_upstream_transcript_variant,upstream_transcript_variant
rs1479823060	intron_variant
rs1479841425	intron_variant,genic_downstream_transcript_variant
rs1479863093	intron_variant,genic_downstream_transcript_variant
rs1479866206	intron_variant,genic_downstream_transcript_variant
rs1479888151	intron_variant,genic_downstream_transcript_variant
rs1479922279	missense_variant,non_coding_transcript_variant,coding_sequence_variant,genic_downstream_transcript_variant
rs1479947015	intron_variant
rs1479961269	intron_variant
rs1479977605	intron_variant,genic_upstream_transcript_variant
rs1480021172	intron_variant,genic_upstream_transcript_variant
rs1480082286	intron_variant
rs1480155482	intron_variant,genic_upstream_transcript_variant
rs1480164174	intron_variant,genic_upstream_transcript_variant
rs1480198497	intron_variant,genic_upstream_transcript_variant
rs1480199513	intron_variant,genic_upstream_transcript_variant
rs1480203867	intron_variant
rs1480205274	intron_variant,genic_upstream_transcript_variant
rs1480269845	intron_variant,genic_upstream_transcript_variant
rs1480302028	intron_variant,genic_upstream_transcript_variant,upstream_transcript_variant
rs1480374159	intron_variant,genic_upstream_transcript_variant,2KB_upstream_variant,upstream_transcript_variant
rs1480415008	intron_variant,genic_downstream_transcript_variant
rs1480447352	intron_variant
rs1480456685	intron_variant,genic_upstream_transcript_variant

rs1480483503	intron_variant,genic_upstream_transcript_variant
rs1480494573	intron_variant,genic_upstream_transcript_variant
rs1480510695	intron_variant
rs1480558829	intron_variant
rs1480561281	intron_variant
rs1480618977	intron_variant,genic_upstream_transcript_variant
rs1480651071	intron_variant
rs1480726688	intron_variant
rs1480743073	intron_variant
rs1480745255	intron_variant,genic_upstream_transcript_variant
rs1480748322	intron_variant,genic_upstream_transcript_variant
rs1480779494	intron_variant,genic_upstream_transcript_variant
rs1480820481	intron_variant,genic_upstream_transcript_variant
rs1480822757	intron_variant,genic_upstream_transcript_variant
rs1480832177	intron_variant
rs1480836467	intron_variant
rs1480847553	intron_variant
rs1480850996	intron_variant,genic_upstream_transcript_variant
rs1480855284	intron_variant,genic_upstream_transcript_variant
rs1480897321	intron_variant,genic_upstream_transcript_variant
rs1480944800	intron_variant
rs1480949752	intron_variant,genic_upstream_transcript_variant
rs1480957914	intron_variant
rs1480982760	intron_variant,genic_upstream_transcript_variant
rs1480992030	intron_variant,genic_upstream_transcript_variant,5_prime_UTR_variant,non_coding_transcript_variant
rs1481001006	intron_variant
rs1481027601	intron_variant
rs1481045075	intron_variant,genic_upstream_transcript_variant
rs1481056466	intron_variant
rs1481069158	intron_variant
rs1481079082	intron_variant,genic_upstream_transcript_variant
rs1481127155	intron_variant
rs1481160556	intron_variant
rs1481186576	intron_variant,genic_downstream_transcript_variant
rs1481204970	intron_variant
rs1481307133	intron_variant,genic_upstream_transcript_variant
rs1481340712	intron_variant
rs1481343753	intron_variant,genic_upstream_transcript_variant
rs1481399950	intron_variant,genic_upstream_transcript_variant
rs1481546200	intron_variant
rs1481565195	intron_variant,genic_upstream_transcript_variant
rs1481604698	intron_variant,genic_upstream_transcript_variant
rs1481637404	non_coding_transcript_variant,3_prime_UTR_variant,genic_downstream_transcript_variant
rs1481649459	intron_variant,genic_upstream_transcript_variant
rs1481670311	missense_variant,coding_sequence_variant,intron_variant
rs1481695346	intron_variant,genic_upstream_transcript_variant,upstream_transcript_variant
rs1481706317	intron_variant,genic_upstream_transcript_variant
rs1481712626	intron_variant
rs1481767625	intron_variant
rs1481790173	intron_variant,genic_upstream_transcript_variant
rs1481812764	missense_variant,coding_sequence_variant,non_coding_transcript_variant
rs1481816899	intron_variant,genic_upstream_transcript_variant
rs1481822057	intron_variant,genic_downstream_transcript_variant
rs1481851508	intron_variant,genic_downstream_transcript_variant
rs1481859788	intron_variant,genic_upstream_transcript_variant
rs1481891266	intron_variant
rs1481953607	intron_variant,genic_upstream_transcript_variant
rs1481988270	intron_variant,genic_upstream_transcript_variant
rs1482024699	intron_variant,genic_upstream_transcript_variant
rs1482038148	intron_variant
rs1482052342	intron_variant,genic_upstream_transcript_variant
rs1482112531	intron_variant
rs1482114846	intron_variant,genic_upstream_transcript_variant
rs1482157538	intron_variant
rs1482163003	intron_variant
rs1482172028	intron_variant,genic_upstream_transcript_variant
rs1482189818	intron_variant,genic_downstream_transcript_variant
rs1482220350	intron_variant,genic_upstream_transcript_variant,upstream_transcript_variant
rs1482274809	intron_variant,genic_upstream_transcript_variant
rs1482291678	intron_variant
rs1482314442	intron_variant,genic_downstream_transcript_variant
rs1482338664	intron_variant,genic_upstream_transcript_variant,5_prime_UTR_variant,non_coding_transcript_variant
rs1482344585	intron_variant
rs1482345480	intron_variant,genic_upstream_transcript_variant

rs1482387416	intron_variant,genic_upstream_transcript_variant
rs1482388846	intron_variant,genic_downstream_transcript_variant
rs1482401194	intron_variant,genic_upstream_transcript_variant
rs1482429940	intron_variant,genic_downstream_transcript_variant
rs1482441537	intron_variant,genic_upstream_transcript_variant
rs1482456002	intron_variant,genic_upstream_transcript_variant,upstream_transcript_variant
rs1482464682	intron_variant
rs1482488279	intron_variant,5_prime_UTR_variant
rs1482504802	intron_variant,genic_upstream_transcript_variant
rs1482519148	intron_variant,genic_downstream_transcript_variant
rs1482585796	intron_variant,genic_upstream_transcript_variant
rs1482618203	intron_variant,genic_upstream_transcript_variant,2KB_upstream_variant,upstream_transcript_variant
rs1482625026	intron_variant
rs1482666895	non_coding_transcript_variant,3_prime_UTR_variant,genic_downstream_transcript_variant
rs1482673878	intron_variant,genic_downstream_transcript_variant
rs1482718503	intron_variant,genic_upstream_transcript_variant
rs1482747692	intron_variant,genic_upstream_transcript_variant
rs1482750356	intron_variant,genic_downstream_transcript_variant
rs1482751478	intron_variant
rs1482855774	intron_variant,genic_upstream_transcript_variant
rs1482876977	intron_variant
rs1482902407	intron_variant
rs1482936987	intron_variant,genic_upstream_transcript_variant
rs1482946975	intron_variant
rs1482953958	intron_variant,genic_upstream_transcript_variant
rs1482975755	intron_variant
rs1483011496	intron_variant,genic_downstream_transcript_variant
rs1483019341	intron_variant
rs1483068972	intron_variant,genic_downstream_transcript_variant
rs1483082485	intron_variant,genic_upstream_transcript_variant,upstream_transcript_variant
rs1483096799	intron_variant
rs1483116946	intron_variant,genic_downstream_transcript_variant
rs1483124041	intron_variant,genic_upstream_transcript_variant
rs1483135845	intron_variant,genic_downstream_transcript_variant
rs1483143808	intron_variant,genic_upstream_transcript_variant,upstream_transcript_variant
rs1483145083	intron_variant,genic_downstream_transcript_variant
rs1483145890	intron_variant
rs1483147202	intron_variant
rs1483201933	non_coding_transcript_variant,3_prime_UTR_variant,genic_downstream_transcript_variant
rs1483265267	intron_variant,genic_downstream_transcript_variant
rs1483304067	non_coding_transcript_variant,3_prime_UTR_variant,genic_downstream_transcript_variant
rs1483322076	intron_variant
rs1483403135	intron_variant
rs1483523557	intron_variant,genic_downstream_transcript_variant
rs1483548843	intron_variant
rs1483667562	intron_variant,downstream_transcript_variant,genic_downstream_transcript_variant
rs1483667995	intron_variant,genic_downstream_transcript_variant
rs1483737501	intron_variant,genic_downstream_transcript_variant
rs1483777546	intron_variant,genic_downstream_transcript_variant
rs1483782606	intron_variant,genic_upstream_transcript_variant
rs1483807850	intron_variant,genic_downstream_transcript_variant
rs1483846521	intron_variant
rs1483850580	missense_variant,non_coding_transcript_variant,intron_variant,genic_upstream_transcript_variant,coding_sequence_variant
rs1483859186	intron_variant
rs1483893840	non_coding_transcript_variant,3_prime_UTR_variant,genic_downstream_transcript_variant
rs1483903823	intron_variant
rs1483914768	intron_variant,genic_upstream_transcript_variant
rs1483943290	intron_variant,genic_upstream_transcript_variant
rs1483987790	intron_variant
rs1483988942	intron_variant
rs1484022965	intron_variant,genic_upstream_transcript_variant
rs1484034938	intron_variant,genic_upstream_transcript_variant
rs1484043712	intron_variant
rs1484082824	intron_variant
rs1484094975	intron_variant,genic_upstream_transcript_variant
rs1484124953	intron_variant,genic_upstream_transcript_variant,upstream_transcript_variant
rs1484157408	intron_variant,genic_downstream_transcript_variant
rs1484167928	intron_variant
rs1484171938	intron_variant,genic_upstream_transcript_variant
rs1484179965	intron_variant
rs1484224007	intron_variant,genic_upstream_transcript_variant
rs1484233569	intron_variant,genic_downstream_transcript_variant
rs1484267213	intron_variant
rs1484279382	intron_variant

rs1484279423	intron_variant,genic_upstream_transcript_variant
rs1484283669	intron_variant
rs1484290026	intron_variant,genic_upstream_transcript_variant
rs1484298284	intron_variant
rs1484299385	non_coding_transcript_variant,3_prime_UTR_variant,genic_downstream_transcript_variant
rs1484337568	intron_variant,genic_upstream_transcript_variant
rs1484351132	intron_variant,genic_upstream_transcript_variant
rs1484366894	intron_variant,genic_upstream_transcript_variant
rs1484384402	intron_variant,genic_upstream_transcript_variant
rs1484396440	intron_variant
rs1484409688	intron_variant
rs1484419753	intron_variant,genic_downstream_transcript_variant
rs1484423527	intron_variant
rs1484461683	intron_variant,2KB_upstream_variant,genic_upstream_transcript_variant,upstream_transcript_variant
rs1484481969	intron_variant,genic_downstream_transcript_variant
rs1484486848	intron_variant
rs1484493045	intron_variant
rs1484539884	intron_variant
rs1484563123	intron_variant,genic_upstream_transcript_variant
rs1484565023	intron_variant,genic_upstream_transcript_variant
rs1484568893	intron_variant,genic_upstream_transcript_variant
rs1484581761	intron_variant,genic_downstream_transcript_variant
rs1484625554	intron_variant
rs1484673628	intron_variant
rs1484686832	intron_variant
rs1484728338	intron_variant,genic_upstream_transcript_variant
rs1484732727	missense_variant,coding_sequence_variant,non_coding_transcript_variant
rs1484737027	non_coding_transcript_variant,coding_sequence_variant,synonymous_variant,genic_downstream_transcript_variant
rs1484767175	intron_variant
rs1484776874	intron_variant
rs1484792652	intron_variant,genic_upstream_transcript_variant
rs1484799408	missense_variant,non_coding_transcript_variant,coding_sequence_variant,genic_downstream_transcript_variant
rs1484801445	intron_variant
rs1484808932	intron_variant,genic_downstream_transcript_variant
rs1484811726	intron_variant,genic_upstream_transcript_variant
rs1484857617	intron_variant
rs1484861329	intron_variant,genic_downstream_transcript_variant
rs1484863610	intron_variant,genic_downstream_transcript_variant
rs1484887472	intron_variant
rs1484956847	intron_variant
rs1485033998	intron_variant
rs1485068410	intron_variant
rs1485105398	genic_downstream_transcript_variant,missense_variant,non_coding_transcript_variant,coding_sequence_variant
rs1485159955	intron_variant
rs1485164082	genic_upstream_transcript_variant,intron_variant
rs1485192946	genic_downstream_transcript_variant,intron_variant
rs1485195354	genic_upstream_transcript_variant,intron_variant
rs1485249108	genic_downstream_transcript_variant,non_coding_transcript_variant,3_prime_UTR_variant
rs1485294106	intron_variant
rs1485296990	genic_upstream_transcript_variant,intron_variant
rs1485304834	genic_upstream_transcript_variant,intron_variant
rs1485305255	genic_downstream_transcript_variant,intron_variant
rs1485375712	genic_upstream_transcript_variant,intron_variant
rs1485377537	genic_upstream_transcript_variant,intron_variant
rs1485442575	intron_variant
rs1485446369	intron_variant
rs1485456789	intron_variant
rs1485458008	genic_upstream_transcript_variant,intron_variant
rs1485468643	genic_upstream_transcript_variant,intron_variant
rs1485473904	intron_variant
rs1485501492	genic_downstream_transcript_variant,intron_variant
rs1485525072	genic_upstream_transcript_variant,intron_variant
rs1485548871	genic_upstream_transcript_variant,intron_variant
rs1485554043	intron_variant
rs1485554731	genic_upstream_transcript_variant,intron_variant
rs1485644444	genic_downstream_transcript_variant,non_coding_transcript_variant,3_prime_UTR_variant
rs1485666145	genic_upstream_transcript_variant,intron_variant
rs1485683999	intron_variant
rs1485695046	genic_upstream_transcript_variant,intron_variant
rs1485712615	genic_upstream_transcript_variant,intron_variant
rs1485732821	genic_upstream_transcript_variant,intron_variant
rs1485770040	genic_upstream_transcript_variant,intron_variant
rs1485771696	genic_upstream_transcript_variant,intron_variant
rs1485797167	genic_upstream_transcript_variant,intron_variant

rs1485825339	genic_upstream_transcript_variant,intron_variant
rs1485849146	genic_upstream_transcript_variant,intron_variant
rs1485850067	genic_downstream_transcript_variant,intron_variant
rs1485891606	missense_variant,non_coding_transcript_variant,coding_sequence_variant
rs1485918578	intron_variant
rs1485957399	intron_variant
rs1485957477	intron_variant
rs1485967265	intron_variant
rs1485988100	genic_upstream_transcript_variant,intron_variant
rs1486002520	genic_upstream_transcript_variant,intron_variant
rs1486006809	intron_variant
rs1486026021	genic_downstream_transcript_variant,intron_variant
rs1486029629	genic_upstream_transcript_variant,intron_variant
rs1486081428	intron_variant
rs1486141035	intron_variant
rs1486183446	intron_variant
rs1486187704	non_coding_transcript_variant,coding_sequence_variant,intron_variant,genic_upstream_transcript_variant,missense_variant
rs1486200433	genic_upstream_transcript_variant,intron_variant
rs1486213925	intron_variant
rs1486220479	genic_upstream_transcript_variant,intron_variant
rs1486250433	genic_upstream_transcript_variant,intron_variant
rs1486265593	genic_upstream_transcript_variant,intron_variant
rs1486289504	genic_downstream_transcript_variant,intron_variant
rs1486293799	genic_downstream_transcript_variant,non_coding_transcript_variant,3_prime_UTR_variant
rs1486294607	genic_upstream_transcript_variant,intron_variant
rs1486325271	genic_upstream_transcript_variant,intron_variant
rs1486326736	genic_upstream_transcript_variant,intron_variant
rs1486343767	genic_downstream_transcript_variant,intron_variant
rs1486406169	genic_upstream_transcript_variant,intron_variant
rs1486415695	genic_upstream_transcript_variant,upstream_transcript_variant,intron_variant,2KB_upstream_variant
rs1486418391	genic_downstream_transcript_variant,intron_variant
rs1486435271	genic_downstream_transcript_variant,intron_variant
rs1486444216	genic_upstream_transcript_variant,intron_variant
rs1486505250	intron_variant
rs1486514176	intron_variant
rs1486526150	genic_upstream_transcript_variant,intron_variant
rs1486594566	genic_upstream_transcript_variant,intron_variant
rs1486638297	intron_variant
rs1486639307	genic_upstream_transcript_variant,intron_variant
rs1486676965	intron_variant
rs1486738970	genic_upstream_transcript_variant,intron_variant
rs1486748346	genic_upstream_transcript_variant,intron_variant
rs1486797401	genic_upstream_transcript_variant,intron_variant
rs1486811946	intron_variant
rs1486827646	genic_downstream_transcript_variant,intron_variant
rs1486860429	genic_downstream_transcript_variant,intron_variant
rs1486892295	genic_downstream_transcript_variant,intron_variant
rs1486901783	intron_variant
rs1486985704	genic_downstream_transcript_variant,intron_variant
rs1486995542	genic_upstream_transcript_variant,upstream_transcript_variant,intron_variant,2KB_upstream_variant
rs1487027676	genic_downstream_transcript_variant,intron_variant
rs1487084701	genic_upstream_transcript_variant,intron_variant
rs1487118687	genic_upstream_transcript_variant,intron_variant
rs1487144303	intron_variant
rs1487148040	genic_downstream_transcript_variant,intron_variant
rs1487149931	genic_downstream_transcript_variant,intron_variant
rs1487161467	genic_upstream_transcript_variant,intron_variant
rs1487236608	genic_downstream_transcript_variant,intron_variant
rs1487292146	non_coding_transcript_variant,coding_sequence_variant,synonymous_variant
rs1487331990	genic_upstream_transcript_variant,intron_variant
rs1487375846	genic_downstream_transcript_variant,intron_variant
rs1487386232	genic_upstream_transcript_variant,intron_variant
rs1487401905	intron_variant
rs1487456192	genic_upstream_transcript_variant,intron_variant
rs1487466833	genic_upstream_transcript_variant,intron_variant
rs1487490849	genic_downstream_transcript_variant,intron_variant
rs1487528185	genic_upstream_transcript_variant,intron_variant
rs1487559453	genic_upstream_transcript_variant,intron_variant
rs1487595476	genic_upstream_transcript_variant,intron_variant
rs1487640889	genic_upstream_transcript_variant,intron_variant
rs1487666216	genic_downstream_transcript_variant,intron_variant
rs1487676944	upstream_transcript_variant,genic_upstream_transcript_variant,intron_variant,2KB_upstream_variant
rs1487691757	intron_variant
rs1487699547	genic_upstream_transcript_variant,intron_variant

rs1487714050	intron_variant
rs1487721453	genic_upstream_transcript_variant,upstream_transcript_variant,intron_variant
rs1487748127	upstream_transcript_variant,genic_upstream_transcript_variant,intron_variant,2KB_upstream_variant
rs1487779386	intron_variant
rs1487839363	intron_variant
rs1487920418	genic_upstream_transcript_variant,intron_variant
rs1487959142	genic_upstream_transcript_variant,upstream_transcript_variant,intron_variant
rs1487974399	intron_variant
rs1487995995	intron_variant
rs1488031501	genic_upstream_transcript_variant,intron_variant
rs1488070621	genic_downstream_transcript_variant,intron_variant
rs1488087347	genic_upstream_transcript_variant,intron_variant
rs1488102819	genic_downstream_transcript_variant,intron_variant
rs1488110360	genic_downstream_transcript_variant,intron_variant
rs1488111638	genic_downstream_transcript_variant,intron_variant
rs1488126201	intron_variant
rs1488182212	intron_variant
rs1488211616	missense_variant,non_coding_transcript_variant,coding_sequence_variant
rs1488260179	intron_variant
rs1488275528	intron_variant
rs1488289029	genic_upstream_transcript_variant,intron_variant
rs1488296990	genic_upstream_transcript_variant,intron_variant
rs1488319546	genic_downstream_transcript_variant,intron_variant
rs1488320755	intron_variant
rs1488322291	genic_downstream_transcript_variant,non_coding_transcript_variant,coding_sequence_variant,synonymous_variant
rs1488332951	genic_upstream_transcript_variant,upstream_transcript_variant,intron_variant,2KB_upstream_variant
rs1488332998	genic_downstream_transcript_variant,non_coding_transcript_variant,coding_sequence_variant,synonymous_variant
rs1488348455	genic_upstream_transcript_variant,intron_variant
rs1488355403	intron_variant
rs1488416448	genic_upstream_transcript_variant,intron_variant
rs1488461185	intron_variant
rs1488496268	genic_upstream_transcript_variant,intron_variant
rs1488526370	intron_variant
rs1488556868	intron_variant
rs1488564485	genic_downstream_transcript_variant,intron_variant
rs1488677626	genic_upstream_transcript_variant,intron_variant
rs1488732493	genic_downstream_transcript_variant,intron_variant
rs1488772874	intron_variant
rs1488784901	genic_downstream_transcript_variant,intron_variant
rs1488940496	genic_upstream_transcript_variant,intron_variant
rs1488941434	genic_upstream_transcript_variant,upstream_transcript_variant,intron_variant
rs1488977324	genic_upstream_transcript_variant,intron_variant
rs1488984139	genic_upstream_transcript_variant,upstream_transcript_variant,intron_variant,2KB_upstream_variant
rs1488986790	genic_downstream_transcript_variant,intron_variant
rs1488996959	genic_downstream_transcript_variant,intron_variant
rs1489023588	genic_upstream_transcript_variant,intron_variant
rs1489056306	intron_variant
rs1489077118	intron_variant
rs1489148201	genic_upstream_transcript_variant,intron_variant
rs1489151494	intron_variant
rs1489196782	intron_variant,upstream_transcript_variant,genic_upstream_transcript_variant,5_prime_UTR_variant
rs1489221263	intron_variant
rs1489223290	genic_upstream_transcript_variant,intron_variant
rs1489267774	intron_variant
rs1489281067	intron_variant
rs1489290055	genic_upstream_transcript_variant,upstream_transcript_variant,intron_variant
rs1489413308	intron_variant
rs1489426486	genic_upstream_transcript_variant,intron_variant
rs1489429146	genic_upstream_transcript_variant,intron_variant
rs1489472611	intron_variant
rs1489523164	genic_upstream_transcript_variant,intron_variant
rs1489529728	intron_variant
rs1489530780	genic_upstream_transcript_variant,intron_variant
rs1489541755	intron_variant
rs1489622764	intron_variant
rs1489655456	upstream_transcript_variant,genic_upstream_transcript_variant,intron_variant,2KB_upstream_variant
rs1489667657	intron_variant
rs1489704560	genic_upstream_transcript_variant,intron_variant
rs1489753985	intron_variant
rs1489759350	genic_upstream_transcript_variant,intron_variant
rs1489785801	intron_variant
rs1489802656	genic_downstream_transcript_variant,intron_variant
rs1489815721	intron_variant
rs1489822317	intron_variant

rs1489828270	intron_variant
rs1489861340	genic_upstream_transcript_variant,intron_variant
rs1489877229	genic_downstream_transcript_variant,non_coding_transcript_variant,3_prime_UTR_variant
rs1489878653	intron_variant
rs1489888731	intron_variant
rs1489931768	genic_downstream_transcript_variant,non_coding_transcript_variant,3_prime_UTR_variant
rs1489946351	missense_variant,non_coding_transcript_variant,coding_sequence_variant,5_prime_UTR_variant
rs1489948003	genic_upstream_transcript_variant,intron_variant
rs1489963596	intron_variant
rs1489970758	genic_upstream_transcript_variant,intron_variant
rs1489985110	genic_downstream_transcript_variant,non_coding_transcript_variant,3_prime_UTR_variant
rs1490057524	genic_downstream_transcript_variant,intron_variant
rs1490098958	genic_downstream_transcript_variant,intron_variant
rs1490106004	intron_variant
rs1490136874	intron_variant
rs1490167478	intron_variant
rs1490205154	intron_variant
rs1490267005	genic_upstream_transcript_variant,intron_variant
rs1490305096	genic_downstream_transcript_variant,intron_variant
rs1490327083	intron_variant
rs1490355177	intron_variant
rs1490367520	intron_variant
rs1490378773	genic_downstream_transcript_variant,intron_variant
rs1490380382	intron_variant
rs1490389467	genic_upstream_transcript_variant,intron_variant
rs1490424505	genic_downstream_transcript_variant,intron_variant
rs1490432889	intron_variant,5_prime_UTR_variant
rs1490460158	genic_upstream_transcript_variant,intron_variant
rs1490469428	genic_upstream_transcript_variant,intron_variant
rs1490493501	intron_variant
rs1490502361	genic_upstream_transcript_variant,intron_variant
rs1490503554	genic_downstream_transcript_variant,intron_variant
rs1490548078	intron_variant
rs1490584480	genic_downstream_transcript_variant,intron_variant
rs1490643773	intron_variant
rs1490648488	non_coding_transcript_variant,coding_sequence_variant,intron_variant,genic_upstream_transcript_variant,missense_variant
rs1490689992	genic_upstream_transcript_variant,intron_variant
rs1490692146	genic_upstream_transcript_variant,upstream_transcript_variant,intron_variant
rs1490747391	genic_upstream_transcript_variant,intron_variant
rs1490777570	genic_downstream_transcript_variant,intron_variant
rs1490779561	intron_variant
rs1490801483	genic_downstream_transcript_variant,missense_variant,non_coding_transcript_variant,coding_sequence_variant
rs1490812519	genic_upstream_transcript_variant,intron_variant
rs1490830596	genic_upstream_transcript_variant,intron_variant
rs1490873465	downstream_transcript_variant,genic_downstream_transcript_variant,intron_variant
rs1490881009	genic_upstream_transcript_variant,intron_variant
rs1490908032	genic_upstream_transcript_variant,intron_variant
rs1490914718	intron_variant
rs1490940194	intron_variant
rs1490959455	genic_upstream_transcript_variant,intron_variant
rs1490980598	intron_variant
rs1490992022	genic_upstream_transcript_variant,intron_variant
rs1490997489	intron_variant
rs1490998532	intron_variant
rs1491004001	genic_upstream_transcript_variant,upstream_transcript_variant,intron_variant
rs1491080305	intron_variant
rs1491183574	genic_upstream_transcript_variant,intron_variant
rs1491198455	genic_upstream_transcript_variant,intron_variant
rs1491236394	intron_variant
rs1491250836	genic_upstream_transcript_variant,intron_variant
rs1491263929	intron_variant
rs1491272300	genic_upstream_transcript_variant,intron_variant
rs1491272551	genic_upstream_transcript_variant,intron_variant
rs1491273986	intron_variant
rs1491325346	genic_upstream_transcript_variant,intron_variant
rs1491328304	intron_variant
rs1491332747	intron_variant
rs1491409307	genic_upstream_transcript_variant,upstream_transcript_variant,intron_variant,2KB_upstream_variant
rs1491422628	genic_upstream_transcript_variant,intron_variant
rs1491477387	intron_variant
rs1491535985	intron_variant
rs1491556630	genic_upstream_transcript_variant,intron_variant
rs1554370467	genic_upstream_transcript_variant,upstream_transcript_variant,intron_variant,2KB_upstream_variant
rs1554371931	intron_variant,non_coding_transcript_variant,genic_upstream_transcript_variant,5_prime_UTR_variant

rs1554373039	genic_upstream_transcript_variant,intron_variant
rs1554374560	genic_upstream_transcript_variant,intron_variant
rs1554375655	upstream_transcript_variant,genic_upstream_transcript_variant,intron_variant,2KB_upstream_variant
rs1554382033	genic_upstream_transcript_variant,intron_variant
rs1554382050	genic_upstream_transcript_variant,intron_variant
rs1554382319	genic_upstream_transcript_variant,intron_variant
rs1554382816	genic_upstream_transcript_variant,intron_variant
rs1554382817	genic_upstream_transcript_variant,intron_variant
rs1554382859	genic_upstream_transcript_variant,intron_variant
rs1554383010	genic_upstream_transcript_variant,intron_variant
rs1554383445	genic_upstream_transcript_variant,intron_variant
rs1554383538	genic_upstream_transcript_variant,intron_variant
rs1554385239	genic_upstream_transcript_variant,intron_variant
rs1554386634	splice_acceptor_variant,intron_variant
rs1554386858	intron_variant
rs1554386877	intron_variant
rs1554386898	intron_variant
rs1554387050	intron_variant
rs1554387069	intron_variant
rs1554387086	intron_variant
rs1554391274	non_coding_transcript_variant,coding_sequence_variant,frameshift_variant
rs1554392227	intron_variant
rs1554393535	intron_variant
rs1554394689	intron_variant
rs1554394700	intron_variant
rs1554394709	intron_variant
rs1554394720	intron_variant
rs1554394721	intron_variant
rs1554394727	intron_variant
rs1554394731	intron_variant
rs1554394775	intron_variant
rs1554394801	intron_variant
rs1554394802	intron_variant
rs1554394813	intron_variant
rs1554394820	intron_variant
rs1554394822	intron_variant
rs1554394824	intron_variant
rs1554394838	intron_variant
rs1554394839	intron_variant
rs1554397034	intron_variant
rs1554397514	intron_variant
rs1554397555	intron_variant
rs1554397556	intron_variant
rs1554398325	genic_downstream_transcript_variant,intron_variant
rs1554400829	genic_downstream_transcript_variant,intron_variant
rs1554402191	genic_downstream_transcript_variant,intron_variant
rs1562866405	genic_upstream_transcript_variant,upstream_transcript_variant,2KB_upstream_variant,intron_variant
rs1562867126	genic_upstream_transcript_variant,upstream_transcript_variant,2KB_upstream_variant,intron_variant
rs1562867391	genic_upstream_transcript_variant,upstream_transcript_variant,2KB_upstream_variant,intron_variant
rs1562867911	genic_upstream_transcript_variant,upstream_transcript_variant,2KB_upstream_variant,intron_variant
rs1562867920	genic_upstream_transcript_variant,upstream_transcript_variant,2KB_upstream_variant,intron_variant
rs1562868374	genic_upstream_transcript_variant,upstream_transcript_variant,2KB_upstream_variant,intron_variant
rs1562868825	genic_upstream_transcript_variant,intron_variant
rs1562868964	genic_upstream_transcript_variant,intron_variant
rs1562868994	genic_upstream_transcript_variant,intron_variant
rs1562869598	genic_upstream_transcript_variant,intron_variant
rs1562869714	genic_upstream_transcript_variant,intron_variant
rs1562869810	genic_upstream_transcript_variant,intron_variant
rs1562869841	genic_upstream_transcript_variant,intron_variant
rs1562869933	genic_upstream_transcript_variant,intron_variant
rs1562870100	genic_upstream_transcript_variant,intron_variant
rs1562870148	genic_upstream_transcript_variant,intron_variant
rs1562870176	genic_upstream_transcript_variant,intron_variant
rs1562870236	genic_upstream_transcript_variant,intron_variant
rs1562870260	genic_upstream_transcript_variant,intron_variant
rs1562870403	genic_upstream_transcript_variant,intron_variant
rs1562870873	genic_upstream_transcript_variant,intron_variant
rs1562871120	genic_upstream_transcript_variant,intron_variant
rs1562871181	genic_upstream_transcript_variant,intron_variant
rs1562871480	genic_upstream_transcript_variant,intron_variant
rs1562871800	genic_upstream_transcript_variant,intron_variant
rs1562872000	genic_upstream_transcript_variant,intron_variant
rs1562872014	genic_upstream_transcript_variant,intron_variant
rs1562872029	genic_upstream_transcript_variant,intron_variant

rs1562872086	genic_upstream_transcript_variant,intron_variant
rs1562872180	genic_upstream_transcript_variant,intron_variant
rs1562872185	genic_upstream_transcript_variant,intron_variant
rs1562872385	genic_upstream_transcript_variant,intron_variant
rs1562872415	genic_upstream_transcript_variant,intron_variant
rs1562872539	genic_upstream_transcript_variant,intron_variant
rs1562872603	genic_upstream_transcript_variant,intron_variant
rs1562872638	genic_upstream_transcript_variant,intron_variant
rs1562872821	genic_upstream_transcript_variant,intron_variant
rs1562873119	genic_upstream_transcript_variant,intron_variant
rs1562873181	genic_upstream_transcript_variant,intron_variant
rs1562873191	genic_upstream_transcript_variant,intron_variant
rs1562873312	genic_upstream_transcript_variant,intron_variant
rs1562873331	genic_upstream_transcript_variant,intron_variant
rs1562873349	genic_upstream_transcript_variant,intron_variant
rs1562873377	genic_upstream_transcript_variant,intron_variant
rs1562873513	genic_upstream_transcript_variant,intron_variant
rs1562873578	genic_upstream_transcript_variant,intron_variant
rs1562873620	genic_upstream_transcript_variant,intron_variant
rs1562873627	genic_upstream_transcript_variant,intron_variant
rs1562873650	genic_upstream_transcript_variant,intron_variant
rs1562873708	genic_upstream_transcript_variant,intron_variant
rs1562874163	genic_upstream_transcript_variant,intron_variant
rs1562874287	genic_upstream_transcript_variant,intron_variant
rs1562874309	genic_upstream_transcript_variant,intron_variant
rs1562874312	genic_upstream_transcript_variant,intron_variant
rs1562874461	genic_upstream_transcript_variant,intron_variant
rs1562874506	genic_upstream_transcript_variant,intron_variant
rs1562874512	genic_upstream_transcript_variant,intron_variant
rs1562874697	genic_upstream_transcript_variant,intron_variant
rs1562874718	genic_upstream_transcript_variant,intron_variant
rs1562874827	genic_upstream_transcript_variant,intron_variant
rs1562874928	genic_upstream_transcript_variant,intron_variant
rs1562875055	genic_upstream_transcript_variant,intron_variant
rs1562875120	genic_upstream_transcript_variant,intron_variant
rs1562875124	genic_upstream_transcript_variant,intron_variant
rs1562875418	genic_upstream_transcript_variant,intron_variant
rs1562875597	genic_upstream_transcript_variant,intron_variant
rs1562875702	genic_upstream_transcript_variant,intron_variant
rs1562875813	genic_upstream_transcript_variant,intron_variant
rs1562875884	genic_upstream_transcript_variant,intron_variant
rs1562875890	genic_upstream_transcript_variant,intron_variant
rs1562876043	genic_upstream_transcript_variant,intron_variant
rs1562876250	genic_upstream_transcript_variant,intron_variant
rs1562876413	genic_upstream_transcript_variant,intron_variant
rs1562876440	genic_upstream_transcript_variant,intron_variant
rs1562876563	genic_upstream_transcript_variant,intron_variant
rs1562876657	genic_upstream_transcript_variant,intron_variant
rs1562876703	genic_upstream_transcript_variant,intron_variant
rs1562876768	non_coding_transcript_variant,genic_upstream_transcript_variant,intron_variant
rs1562877218	genic_upstream_transcript_variant,upstream_transcript_variant,2KB_upstream_variant,intron_variant
rs1562877271	genic_upstream_transcript_variant,upstream_transcript_variant,2KB_upstream_variant,intron_variant
rs1562877760	genic_upstream_transcript_variant,upstream_transcript_variant,2KB_upstream_variant,intron_variant
rs1562877860	genic_upstream_transcript_variant,upstream_transcript_variant,2KB_upstream_variant,intron_variant
rs1562877941	genic_upstream_transcript_variant,intron_variant
rs1562878317	genic_upstream_transcript_variant,intron_variant
rs1562878494	genic_upstream_transcript_variant,intron_variant
rs1562878579	genic_upstream_transcript_variant,intron_variant
rs1562878648	genic_upstream_transcript_variant,intron_variant
rs1562878852	genic_upstream_transcript_variant,intron_variant
rs1562879308	genic_upstream_transcript_variant,intron_variant
rs1562879579	genic_upstream_transcript_variant,intron_variant
rs1562879629	genic_upstream_transcript_variant,intron_variant
rs1562879653	genic_upstream_transcript_variant,intron_variant
rs1562879671	genic_upstream_transcript_variant,intron_variant
rs1562880167	genic_upstream_transcript_variant,intron_variant
rs1562880214	genic_upstream_transcript_variant,intron_variant
rs1562880482	genic_upstream_transcript_variant,intron_variant
rs1562880735	genic_upstream_transcript_variant,intron_variant
rs1562880987	genic_upstream_transcript_variant,intron_variant
rs1562881143	genic_upstream_transcript_variant,intron_variant
rs1562881166	genic_upstream_transcript_variant,intron_variant
rs1562881382	genic_upstream_transcript_variant,intron_variant
rs1562881467	genic_upstream_transcript_variant,intron_variant

rs1562881749	genic_upstream_transcript_variant,intron_variant
rs1562881851	genic_upstream_transcript_variant,intron_variant
rs1562881899	genic_upstream_transcript_variant,intron_variant
rs1562882027	genic_upstream_transcript_variant,intron_variant
rs1562882050	genic_upstream_transcript_variant,intron_variant
rs1562882350	genic_upstream_transcript_variant,intron_variant
rs1562882352	genic_upstream_transcript_variant,intron_variant
rs1562882674	genic_upstream_transcript_variant,intron_variant
rs1562882676	genic_upstream_transcript_variant,intron_variant
rs1562882697	genic_upstream_transcript_variant,intron_variant
rs1562882830	coding_sequence_variant,intron_variant,genic_upstream_transcript_variant,non_coding_transcript_variant,missense_variant
rs1562883265	coding_sequence_variant,intron_variant,genic_upstream_transcript_variant,non_coding_transcript_variant,missense_variant
rs1562884742	genic_upstream_transcript_variant,intron_variant
rs1562884981	genic_upstream_transcript_variant,intron_variant
rs1562885437	genic_upstream_transcript_variant,intron_variant
rs1562885456	genic_upstream_transcript_variant,intron_variant
rs1562885561	genic_upstream_transcript_variant,intron_variant
rs1562885637	genic_upstream_transcript_variant,intron_variant
rs1562885641	genic_upstream_transcript_variant,intron_variant
rs1562885829	genic_upstream_transcript_variant,intron_variant
rs1562885837	genic_upstream_transcript_variant,intron_variant
rs1562886075	genic_upstream_transcript_variant,intron_variant
rs1562886255	genic_upstream_transcript_variant,intron_variant
rs1562886259	genic_upstream_transcript_variant,intron_variant
rs1562886914	genic_upstream_transcript_variant,intron_variant
rs1562886942	genic_upstream_transcript_variant,intron_variant
rs1562887059	genic_upstream_transcript_variant,intron_variant
rs1562887231	genic_upstream_transcript_variant,intron_variant
rs1562887591	genic_upstream_transcript_variant,intron_variant
rs1562887594	genic_upstream_transcript_variant,intron_variant
rs1562887913	genic_upstream_transcript_variant,intron_variant
rs1562888013	genic_upstream_transcript_variant,intron_variant
rs1562888065	genic_upstream_transcript_variant,intron_variant
rs1562888120	genic_upstream_transcript_variant,intron_variant
rs1562888436	genic_upstream_transcript_variant,intron_variant
rs1562888513	genic_upstream_transcript_variant,intron_variant
rs1562888525	genic_upstream_transcript_variant,intron_variant
rs1562888617	genic_upstream_transcript_variant,intron_variant
rs1562888856	genic_upstream_transcript_variant,intron_variant
rs1562889270	genic_upstream_transcript_variant,intron_variant
rs1562889393	genic_upstream_transcript_variant,intron_variant
rs1562889561	genic_upstream_transcript_variant,intron_variant
rs1562890195	genic_upstream_transcript_variant,intron_variant
rs1562890225	genic_upstream_transcript_variant,intron_variant
rs1562890235	genic_upstream_transcript_variant,intron_variant
rs1562890263	genic_upstream_transcript_variant,intron_variant
rs1562890414	genic_upstream_transcript_variant,intron_variant
rs1562890446	genic_upstream_transcript_variant,intron_variant
rs1562890521	genic_upstream_transcript_variant,intron_variant
rs1562890877	genic_upstream_transcript_variant,intron_variant
rs1562891333	genic_upstream_transcript_variant,intron_variant
rs1562891429	genic_upstream_transcript_variant,intron_variant
rs1562891845	genic_upstream_transcript_variant,intron_variant
rs1562892133	genic_upstream_transcript_variant,intron_variant
rs1562892167	genic_upstream_transcript_variant,intron_variant
rs1562892621	genic_upstream_transcript_variant,intron_variant
rs1562892637	genic_upstream_transcript_variant,intron_variant
rs1562892822	genic_upstream_transcript_variant,intron_variant
rs1562892872	genic_upstream_transcript_variant,intron_variant
rs1562892876	genic_upstream_transcript_variant,intron_variant
rs1562892881	genic_upstream_transcript_variant,intron_variant
rs1562892884	genic_upstream_transcript_variant,intron_variant
rs1562892886	genic_upstream_transcript_variant,intron_variant
rs1562892891	genic_upstream_transcript_variant,intron_variant
rs1562892905	genic_upstream_transcript_variant,intron_variant
rs1562892906	genic_upstream_transcript_variant,intron_variant
rs1562892929	genic_upstream_transcript_variant,intron_variant
rs1562892977	genic_upstream_transcript_variant,intron_variant
rs1562893020	genic_upstream_transcript_variant,intron_variant
rs1562893200	genic_upstream_transcript_variant,intron_variant
rs1562893257	genic_upstream_transcript_variant,intron_variant
rs1562893262	genic_upstream_transcript_variant,intron_variant
rs1562893269	genic_upstream_transcript_variant,intron_variant
rs1562893276	genic_upstream_transcript_variant,intron_variant

rs1562893296	genic_upstream_transcript_variant,intron_variant
rs1562893331	genic_upstream_transcript_variant,intron_variant
rs1562893353	genic_upstream_transcript_variant,intron_variant
rs1562893465	genic_upstream_transcript_variant,intron_variant
rs1562893483	genic_upstream_transcript_variant,intron_variant
rs1562893487	genic_upstream_transcript_variant,intron_variant
rs1562893503	genic_upstream_transcript_variant,intron_variant
rs1562893564	genic_upstream_transcript_variant,intron_variant
rs1562893586	genic_upstream_transcript_variant,intron_variant
rs1562893734	genic_upstream_transcript_variant,intron_variant
rs1562893884	genic_upstream_transcript_variant,intron_variant
rs1562893927	genic_upstream_transcript_variant,intron_variant
rs1562893936	genic_upstream_transcript_variant,intron_variant
rs1562894011	genic_upstream_transcript_variant,intron_variant
rs1562894017	genic_upstream_transcript_variant,intron_variant
rs1562894021	genic_upstream_transcript_variant,intron_variant
rs1562894569	genic_upstream_transcript_variant,intron_variant
rs1562894852	genic_upstream_transcript_variant,intron_variant
rs1562895608	genic_upstream_transcript_variant,intron_variant
rs1562896019	genic_upstream_transcript_variant,intron_variant
rs1562896440	genic_upstream_transcript_variant,intron_variant
rs1562896473	genic_upstream_transcript_variant,intron_variant
rs1562896507	genic_upstream_transcript_variant,intron_variant
rs1562896538	genic_upstream_transcript_variant,intron_variant
rs1562897212	genic_upstream_transcript_variant,upstream_transcript_variant,intron_variant
rs1562897447	genic_upstream_transcript_variant,upstream_transcript_variant,intron_variant
rs1562897462	genic_upstream_transcript_variant,upstream_transcript_variant,intron_variant
rs1562897479	genic_upstream_transcript_variant,upstream_transcript_variant,intron_variant
rs1562897980	genic_upstream_transcript_variant,upstream_transcript_variant,intron_variant
rs1562897985	genic_upstream_transcript_variant,upstream_transcript_variant,intron_variant
rs1562898127	genic_upstream_transcript_variant,upstream_transcript_variant,intron_variant
rs1562898196	genic_upstream_transcript_variant,upstream_transcript_variant,intron_variant
rs1562898821	intron_variant
rs1562898891	intron_variant
rs1562898924	intron_variant
rs1562898986	intron_variant
rs1562899184	intron_variant
rs1562899199	intron_variant
rs1562899215	intron_variant
rs1562899258	intron_variant
rs1562899276	intron_variant
rs1562899305	intron_variant
rs1562899728	intron_variant
rs1562899734	intron_variant
rs1562899950	intron_variant
rs1562899954	intron_variant
rs1562899991	intron_variant
rs1562899995	intron_variant
rs1562899999	intron_variant
rs1562900004	intron_variant
rs1562900009	intron_variant
rs1562900031	intron_variant
rs1562900036	intron_variant
rs1562900039	intron_variant
rs1562900047	intron_variant
rs1562900050	intron_variant
rs1562900060	intron_variant
rs1562900069	intron_variant
rs1562900074	intron_variant
rs1562900076	intron_variant
rs1562900086	intron_variant
rs1562900093	intron_variant
rs1562900103	intron_variant
rs1562900114	intron_variant
rs1562900129	intron_variant
rs1562900140	intron_variant
rs1562900150	intron_variant
rs1562900322	intron_variant
rs1562900324	intron_variant
rs1562900379	intron_variant
rs1562900483	intron_variant
rs1562900645	intron_variant
rs1562900741	intron_variant
rs1562900802	intron_variant

rs1562900906	intron_variant
rs1562900955	intron_variant
rs1562901126	intron_variant
rs1562901222	intron_variant
rs1562901383	intron_variant
rs1562901534	intron_variant
rs1562901713	intron_variant
rs1562901784	intron_variant
rs1562901805	intron_variant
rs1562902229	intron_variant
rs1562902310	intron_variant
rs1562902628	intron_variant
rs1562902858	intron_variant
rs1562903048	intron_variant
rs1562903059	intron_variant
rs1562903156	intron_variant
rs1562903209	intron_variant
rs1562903360	intron_variant
rs1562903420	intron_variant
rs1562904008	intron_variant
rs1562904406	intron_variant
rs1562904581	intron_variant
rs1562904757	intron_variant
rs1562905068	intron_variant
rs1562905306	intron_variant
rs1562905343	intron_variant
rs1562905348	intron_variant
rs1562906008	intron_variant
rs1562906302	intron_variant
rs1562906342	intron_variant
rs1562906651	intron_variant
rs1562906807	intron_variant
rs1562906933	intron_variant
rs1562907636	intron_variant
rs1562907652	intron_variant
rs1562907696	intron_variant
rs1562907804	intron_variant
rs1562908955	intron_variant
rs1562909033	intron_variant
rs1562909039	intron_variant
rs1562909309	intron_variant
rs1562909383	intron_variant
rs1562909812	intron_variant
rs1562909857	intron_variant
rs1562910451	intron_variant
rs1562910687	intron_variant
rs1562910964	intron_variant
rs1562911011	intron_variant
rs1562911360	intron_variant
rs1562911422	intron_variant
rs1562911438	intron_variant
rs1562911631	intron_variant
rs1562911655	intron_variant
rs1562912049	intron_variant
rs1562912280	intron_variant
rs1562912590	intron_variant
rs1562912594	intron_variant
rs1562912769	intron_variant
rs1562912865	intron_variant
rs1562913003	intron_variant
rs1562913521	intron_variant
rs1562913803	intron_variant
rs1562914022	intron_variant
rs1562914209	intron_variant
rs1562914271	intron_variant
rs1562914552	intron_variant
rs1562914675	intron_variant
rs1562914685	intron_variant
rs1562914841	intron_variant
rs1562915243	intron_variant
rs1562915466	intron_variant
rs1562915597	intron_variant
rs1562915794	intron_variant
rs1562915829	intron_variant

rs1562916358	intron_variant
rs1562916431	intron_variant
rs1562916788	intron_variant
rs1562917125	intron_variant
rs1562917328	intron_variant
rs1562917586	intron_variant
rs1562917648	intron_variant
rs1562917680	intron_variant
rs1562917743	intron_variant
rs1562917788	intron_variant
rs1562917790	intron_variant
rs1562917854	intron_variant
rs1562917867	intron_variant
rs1562917974	intron_variant
rs1562918093	intron_variant
rs1562918197	intron_variant
rs1562918213	intron_variant
rs1562918566	intron_variant
rs1562918762	intron_variant
rs1562918975	intron_variant
rs1562919034	intron_variant
rs1562919386	intron_variant
rs1562919517	intron_variant
rs1562919559	intron_variant
rs1562919602	intron_variant
rs1562919610	intron_variant
rs1562919625	intron_variant
rs1562919640	intron_variant
rs1562919689	intron_variant
rs1562919738	intron_variant
rs1562919804	intron_variant
rs1562919894	intron_variant
rs1562919896	intron_variant
rs1562920030	intron_variant
rs1562920036	intron_variant
rs1562920128	intron_variant
rs1562920359	synonymous_variant,coding_sequence_variant,non_coding_transcript_variant
rs1562920677	intron_variant
rs1562921050	intron_variant
rs1562921094	intron_variant
rs1562921431	intron_variant
rs1562921499	intron_variant
rs1562921911	intron_variant
rs1562921924	intron_variant
rs1562922022	missense_variant,coding_sequence_variant,non_coding_transcript_variant
rs1562922253	missense_variant,coding_sequence_variant,non_coding_transcript_variant
rs1562922669	intron_variant
rs1562923703	intron_variant
rs1562923792	intron_variant
rs1562923819	intron_variant
rs1562924533	intron_variant
rs1562924669	intron_variant
rs1562924727	intron_variant
rs1562924831	intron_variant
rs1562924868	intron_variant
rs1562925061	intron_variant
rs1562925134	intron_variant
rs1562926029	intron_variant
rs1562926053	intron_variant
rs1562926113	intron_variant
rs1562926295	intron_variant
rs1562926307	intron_variant
rs1562926461	intron_variant
rs1562926627	intron_variant
rs1562926678	intron_variant
rs1562926751	intron_variant
rs1562926885	intron_variant
rs1562926945	intron_variant
rs1562926979	intron_variant
rs1562927052	intron_variant
rs1562927065	intron_variant
rs1562927169	intron_variant
rs1562927440	intron_variant
rs1562927465	intron_variant

rs1562927678	intron_variant
rs1562927973	intron_variant
rs1562928114	intron_variant
rs1562928143	intron_variant
rs1562928697	intron_variant
rs1562928755	intron_variant
rs1562928807	intron_variant
rs1562928904	intron_variant
rs1562929016	intron_variant
rs1562929055	intron_variant
rs1562929516	genic_downstream_transcript_variant,downstream_transcript_variant,intron_variant
rs1562929842	genic_downstream_transcript_variant,intron_variant
rs1562930141	genic_downstream_transcript_variant,missense_variant,coding_sequence_variant,non_coding_transcript_variant
rs1562930219	genic_downstream_transcript_variant,intron_variant
rs1562930513	genic_downstream_transcript_variant,intron_variant
rs1562930925	genic_downstream_transcript_variant,intron_variant
rs1562930956	genic_downstream_transcript_variant,intron_variant
rs1562930960	genic_downstream_transcript_variant,intron_variant
rs1562931082	genic_downstream_transcript_variant,intron_variant
rs1562931131	genic_downstream_transcript_variant,intron_variant
rs1562931172	genic_downstream_transcript_variant,intron_variant
rs1562932354	genic_downstream_transcript_variant,intron_variant
rs1562932357	genic_downstream_transcript_variant,intron_variant
rs1562932482	genic_downstream_transcript_variant,intron_variant
rs1562932697	genic_downstream_transcript_variant,intron_variant
rs1562933004	genic_downstream_transcript_variant,missense_variant,coding_sequence_variant,non_coding_transcript_variant
rs1562933166	genic_downstream_transcript_variant,intron_variant
rs1562933203	genic_downstream_transcript_variant,intron_variant
rs1562933276	genic_downstream_transcript_variant,intron_variant
rs1562933315	genic_downstream_transcript_variant,intron_variant
rs1562933363	genic_downstream_transcript_variant,intron_variant
rs1562933551	genic_downstream_transcript_variant,intron_variant
rs1562933913	genic_downstream_transcript_variant,intron_variant
rs1562934146	genic_downstream_transcript_variant,intron_variant
rs1562934272	genic_downstream_transcript_variant,intron_variant
rs1562934319	genic_downstream_transcript_variant,intron_variant
rs1562934605	genic_downstream_transcript_variant,intron_variant
rs1562934664	genic_downstream_transcript_variant,intron_variant
rs1562934691	genic_downstream_transcript_variant,intron_variant
rs1562934698	genic_downstream_transcript_variant,intron_variant
rs1562934901	genic_downstream_transcript_variant,intron_variant
rs1562934971	genic_downstream_transcript_variant,intron_variant
rs1562935085	genic_downstream_transcript_variant,intron_variant
rs1562935120	genic_downstream_transcript_variant,intron_variant
rs1562935219	genic_downstream_transcript_variant,missense_variant,coding_sequence_variant,non_coding_transcript_variant
rs1562935564	genic_downstream_transcript_variant,intron_variant
rs1562935587	genic_downstream_transcript_variant,intron_variant
rs1562935598	genic_downstream_transcript_variant,intron_variant
rs1562935618	genic_downstream_transcript_variant,intron_variant
rs1562935745	genic_downstream_transcript_variant,intron_variant
rs1562936028	genic_downstream_transcript_variant,intron_variant
rs1562936153	genic_downstream_transcript_variant,intron_variant
rs1562936186	genic_downstream_transcript_variant,intron_variant
rs1562936226	genic_downstream_transcript_variant,intron_variant
rs1562936415	genic_downstream_transcript_variant,intron_variant
rs1562936510	genic_downstream_transcript_variant,intron_variant
rs1562936663	genic_downstream_transcript_variant,intron_variant
rs1562936683	genic_downstream_transcript_variant,intron_variant
rs1562936705	genic_downstream_transcript_variant,intron_variant
rs1562936753	genic_downstream_transcript_variant,intron_variant
rs1562936789	genic_downstream_transcript_variant,intron_variant
rs1562936921	genic_downstream_transcript_variant,intron_variant
rs1562936983	genic_downstream_transcript_variant,intron_variant
rs1562936991	genic_downstream_transcript_variant,intron_variant
rs1562937101	genic_downstream_transcript_variant,intron_variant
rs1562937122	genic_downstream_transcript_variant,intron_variant
rs1562937410	genic_downstream_transcript_variant,intron_variant
rs1562937543	genic_downstream_transcript_variant,intron_variant
rs1562937647	genic_downstream_transcript_variant,intron_variant
rs1562937909	genic_downstream_transcript_variant,intron_variant
rs1562938053	genic_downstream_transcript_variant,intron_variant
rs1562938097	genic_downstream_transcript_variant,intron_variant
rs1562938314	genic_downstream_transcript_variant,intron_variant
rs1562938353	genic_downstream_transcript_variant,intron_variant

rs1562938432	genic_downstream_transcript_variant,intron_variant
rs1562938530	genic_downstream_transcript_variant,intron_variant
rs1562938544	genic_downstream_transcript_variant,intron_variant
rs1562939003	genic_downstream_transcript_variant,intron_variant
rs1562939357	genic_downstream_transcript_variant,intron_variant
rs1562939781	genic_downstream_transcript_variant,intron_variant
rs1562939869	genic_downstream_transcript_variant,intron_variant
rs1562940591	genic_downstream_transcript_variant,intron_variant
rs1562941062	genic_downstream_transcript_variant,intron_variant
rs1562941124	genic_downstream_transcript_variant,intron_variant
rs1562941151	genic_downstream_transcript_variant,intron_variant
rs1562941196	genic_downstream_transcript_variant,intron_variant
rs1562941267	genic_downstream_transcript_variant,intron_variant
rs1562941361	genic_downstream_transcript_variant,missense_variant,coding_sequence_variant,non_coding_transcript_variant
rs1562941498	genic_downstream_transcript_variant,intron_variant
rs1562941664	genic_downstream_transcript_variant,synonymous_variant,coding_sequence_variant,non_coding_transcript_variant
rs1562942257	genic_downstream_transcript_variant,3_prime_UTR_variant,non_coding_transcript_variant
rs1562942263	genic_downstream_transcript_variant,3_prime_UTR_variant,non_coding_transcript_variant
rs1562942543	genic_downstream_transcript_variant,3_prime_UTR_variant,non_coding_transcript_variant
rs1562942650	genic_downstream_transcript_variant,3_prime_UTR_variant,non_coding_transcript_variant
rs1562943805	500B_downstream_variant,downstream_transcript_variant
rs1584835829	genic_upstream_transcript_variant,intron_variant,2KB_upstream_variant,upstream_transcript_variant
rs1584836238	genic_upstream_transcript_variant,intron_variant,2KB_upstream_variant,upstream_transcript_variant
rs1584836408	genic_upstream_transcript_variant,intron_variant,2KB_upstream_variant,upstream_transcript_variant
rs1584836545	genic_upstream_transcript_variant,intron_variant,2KB_upstream_variant,upstream_transcript_variant
rs1584837439	genic_upstream_transcript_variant,intron_variant,2KB_upstream_variant,upstream_transcript_variant
rs1584837825	genic_upstream_transcript_variant,intron_variant,2KB_upstream_variant,upstream_transcript_variant
rs1584838434	genic_upstream_transcript_variant,intron_variant,2KB_upstream_variant,upstream_transcript_variant
rs1584838920	genic_upstream_transcript_variant,intron_variant,2KB_upstream_variant,upstream_transcript_variant
rs1584839092	genic_upstream_transcript_variant,intron_variant,2KB_upstream_variant,upstream_transcript_variant
rs1584839269	genic_upstream_transcript_variant,intron_variant,2KB_upstream_variant,upstream_transcript_variant
rs1584839414	genic_upstream_transcript_variant,intron_variant,2KB_upstream_variant,upstream_transcript_variant
rs1584839626	genic_upstream_transcript_variant,intron_variant,2KB_upstream_variant,upstream_transcript_variant
rs1584839876	genic_upstream_transcript_variant,intron_variant,2KB_upstream_variant,upstream_transcript_variant
rs1584839924	genic_upstream_transcript_variant,intron_variant,2KB_upstream_variant,upstream_transcript_variant
rs1584840141	genic_upstream_transcript_variant,intron_variant,2KB_upstream_variant,upstream_transcript_variant
rs1584840151	genic_upstream_transcript_variant,intron_variant,2KB_upstream_variant,upstream_transcript_variant
rs1584840227	genic_upstream_transcript_variant,intron_variant,5_prime_UTR_variant,upstream_transcript_variant
rs1584840274	genic_upstream_transcript_variant,intron_variant,5_prime_UTR_variant,upstream_transcript_variant
rs1584840330	genic_upstream_transcript_variant,intron_variant,5_prime_UTR_variant,upstream_transcript_variant
rs1584840343	genic_upstream_transcript_variant,intron_variant,5_prime_UTR_variant,upstream_transcript_variant
rs1584840391	genic_upstream_transcript_variant,intron_variant,5_prime_UTR_variant,upstream_transcript_variant
rs1584840711	genic_upstream_transcript_variant,intron_variant,non_coding_transcript_variant,5_prime_UTR_variant
rs1584840757	genic_upstream_transcript_variant,intron_variant,splice_donor_variant
rs1584840957	genic_upstream_transcript_variant,intron_variant
rs1584840983	genic_upstream_transcript_variant,intron_variant
rs1584841015	genic_upstream_transcript_variant,intron_variant
rs1584841024	genic_upstream_transcript_variant,intron_variant
rs1584841086	genic_upstream_transcript_variant,intron_variant
rs1584841509	genic_upstream_transcript_variant,intron_variant
rs1584841588	genic_upstream_transcript_variant,intron_variant
rs1584841597	genic_upstream_transcript_variant,intron_variant
rs1584841709	genic_upstream_transcript_variant,intron_variant
rs1584841801	genic_upstream_transcript_variant,intron_variant
rs1584841928	genic_upstream_transcript_variant,intron_variant
rs1584842029	genic_upstream_transcript_variant,intron_variant
rs1584842468	genic_upstream_transcript_variant,intron_variant
rs1584842624	genic_upstream_transcript_variant,intron_variant
rs1584842780	genic_upstream_transcript_variant,intron_variant
rs1584842800	genic_upstream_transcript_variant,intron_variant
rs1584843286	genic_upstream_transcript_variant,intron_variant
rs1584843754	genic_upstream_transcript_variant,intron_variant
rs1584843874	genic_upstream_transcript_variant,intron_variant
rs1584844045	genic_upstream_transcript_variant,intron_variant
rs1584844169	genic_upstream_transcript_variant,intron_variant
rs1584844215	genic_upstream_transcript_variant,intron_variant
rs1584844252	genic_upstream_transcript_variant,intron_variant
rs1584844690	genic_upstream_transcript_variant,intron_variant
rs1584845134	genic_upstream_transcript_variant,intron_variant
rs1584845244	genic_upstream_transcript_variant,intron_variant
rs1584845326	genic_upstream_transcript_variant,intron_variant
rs1584845846	genic_upstream_transcript_variant,intron_variant
rs1584845878	genic_upstream_transcript_variant,intron_variant
rs1584846176	genic_upstream_transcript_variant,intron_variant

rs1584846204	genic_upstream_transcript_variant,intron_variant
rs1584846212	genic_upstream_transcript_variant,intron_variant
rs1584846232	genic_upstream_transcript_variant,intron_variant
rs1584846724	genic_upstream_transcript_variant,intron_variant
rs1584846837	genic_upstream_transcript_variant,intron_variant
rs1584846863	genic_upstream_transcript_variant,intron_variant
rs1584846881	genic_upstream_transcript_variant,intron_variant
rs1584847024	genic_upstream_transcript_variant,intron_variant
rs1584847225	genic_upstream_transcript_variant,intron_variant
rs1584847515	genic_upstream_transcript_variant,intron_variant
rs1584847685	genic_upstream_transcript_variant,intron_variant
rs1584847730	genic_upstream_transcript_variant,intron_variant
rs1584847861	genic_upstream_transcript_variant,intron_variant
rs1584848017	genic_upstream_transcript_variant,intron_variant
rs1584848239	genic_upstream_transcript_variant,intron_variant
rs1584848272	genic_upstream_transcript_variant,intron_variant
rs1584848554	genic_upstream_transcript_variant,intron_variant
rs1584848580	genic_upstream_transcript_variant,intron_variant
rs1584848627	genic_upstream_transcript_variant,intron_variant
rs1584848904	genic_upstream_transcript_variant,intron_variant
rs1584848945	genic_upstream_transcript_variant,intron_variant
rs1584848964	genic_upstream_transcript_variant,intron_variant
rs1584849464	genic_upstream_transcript_variant,intron_variant
rs1584849492	genic_upstream_transcript_variant,intron_variant
rs1584849777	genic_upstream_transcript_variant,intron_variant
rs1584849832	genic_upstream_transcript_variant,intron_variant
rs1584849997	genic_upstream_transcript_variant,intron_variant
rs1584850057	genic_upstream_transcript_variant,intron_variant
rs1584850226	genic_upstream_transcript_variant,intron_variant
rs1584850259	genic_upstream_transcript_variant,intron_variant
rs1584850302	genic_upstream_transcript_variant,intron_variant
rs1584850701	genic_upstream_transcript_variant,intron_variant
rs1584850828	genic_upstream_transcript_variant,intron_variant
rs1584850875	genic_upstream_transcript_variant,intron_variant
rs1584851031	genic_upstream_transcript_variant,intron_variant
rs1584851088	genic_upstream_transcript_variant,intron_variant
rs1584851329	genic_upstream_transcript_variant,intron_variant
rs1584851344	genic_upstream_transcript_variant,intron_variant
rs1584851401	genic_upstream_transcript_variant,intron_variant
rs1584851623	genic_upstream_transcript_variant,intron_variant
rs1584851830	genic_upstream_transcript_variant,intron_variant
rs1584851836	genic_upstream_transcript_variant,intron_variant
rs1584851840	genic_upstream_transcript_variant,intron_variant
rs1584851922	genic_upstream_transcript_variant,intron_variant
rs1584852799	genic_upstream_transcript_variant,intron_variant
rs1584852838	genic_upstream_transcript_variant,intron_variant
rs1584853122	genic_upstream_transcript_variant,intron_variant
rs1584853184	genic_upstream_transcript_variant,intron_variant
rs1584853259	genic_upstream_transcript_variant,intron_variant
rs1584853346	genic_upstream_transcript_variant,intron_variant
rs1584853772	genic_upstream_transcript_variant,intron_variant
rs1584853921	genic_upstream_transcript_variant,intron_variant
rs1584853958	genic_upstream_transcript_variant,intron_variant
rs1584854075	genic_upstream_transcript_variant,intron_variant
rs1584854325	genic_upstream_transcript_variant,intron_variant
rs1584854844	genic_upstream_transcript_variant,intron_variant
rs1584855057	genic_upstream_transcript_variant,intron_variant
rs1584855070	genic_upstream_transcript_variant,intron_variant
rs1584855101	genic_upstream_transcript_variant,intron_variant
rs1584855118	genic_upstream_transcript_variant,intron_variant
rs1584855123	genic_upstream_transcript_variant,intron_variant
rs1584855130	genic_upstream_transcript_variant,intron_variant
rs1584855171	genic_upstream_transcript_variant,intron_variant
rs1584855194	genic_upstream_transcript_variant,intron_variant
rs1584855204	genic_upstream_transcript_variant,intron_variant
rs1584855211	genic_upstream_transcript_variant,intron_variant
rs1584855216	genic_upstream_transcript_variant,intron_variant
rs1584855222	genic_upstream_transcript_variant,intron_variant
rs1584855261	genic_upstream_transcript_variant,intron_variant
rs1584855438	genic_upstream_transcript_variant,intron_variant
rs1584855537	genic_upstream_transcript_variant,intron_variant
rs1584855569	genic_upstream_transcript_variant,intron_variant
rs1584855924	genic_upstream_transcript_variant,intron_variant
rs1584856033	genic_upstream_transcript_variant,intron_variant

rs1584856354	genic_upstream_transcript_variant,intron_variant
rs1584856376	genic_upstream_transcript_variant,intron_variant
rs1584856466	genic_upstream_transcript_variant,intron_variant
rs1584856558	genic_upstream_transcript_variant,intron_variant
rs1584856894	genic_upstream_transcript_variant,intron_variant
rs1584857140	genic_upstream_transcript_variant,intron_variant
rs1584857241	genic_upstream_transcript_variant,intron_variant
rs1584857671	genic_upstream_transcript_variant,intron_variant
rs1584858607	genic_upstream_transcript_variant,intron_variant
rs1584858645	genic_upstream_transcript_variant,intron_variant
rs1584858959	genic_upstream_transcript_variant,intron_variant
rs1584859238	genic_upstream_transcript_variant,intron_variant
rs1584859373	genic_upstream_transcript_variant,intron_variant
rs1584859452	genic_upstream_transcript_variant,intron_variant
rs1584859549	genic_upstream_transcript_variant,intron_variant
rs1584859803	genic_upstream_transcript_variant,intron_variant
rs1584859862	genic_upstream_transcript_variant,intron_variant
rs1584860153	genic_upstream_transcript_variant,intron_variant
rs1584860192	genic_upstream_transcript_variant,intron_variant
rs1584860281	genic_upstream_transcript_variant,intron_variant
rs1584860298	genic_upstream_transcript_variant,intron_variant
rs1584860337	genic_upstream_transcript_variant,intron_variant
rs1584860504	genic_upstream_transcript_variant,intron_variant
rs1584860597	genic_upstream_transcript_variant,intron_variant
rs1584860791	genic_upstream_transcript_variant,intron_variant
rs1584860939	genic_upstream_transcript_variant,intron_variant
rs1584860978	genic_upstream_transcript_variant,intron_variant
rs1584861074	genic_upstream_transcript_variant,intron_variant
rs1584861093	genic_upstream_transcript_variant,intron_variant
rs1584861100	genic_upstream_transcript_variant,intron_variant
rs1584861107	genic_upstream_transcript_variant,intron_variant
rs1584861184	genic_upstream_transcript_variant,intron_variant
rs1584861304	genic_upstream_transcript_variant,intron_variant
rs1584861337	genic_upstream_transcript_variant,intron_variant
rs1584861409	genic_upstream_transcript_variant,intron_variant
rs1584861445	genic_upstream_transcript_variant,intron_variant
rs1584861493	genic_upstream_transcript_variant,intron_variant
rs1584861618	genic_upstream_transcript_variant,intron_variant
rs1584862039	genic_upstream_transcript_variant,intron_variant
rs1584862067	genic_upstream_transcript_variant,intron_variant
rs1584862171	genic_upstream_transcript_variant,intron_variant
rs1584862296	genic_upstream_transcript_variant,intron_variant
rs1584862464	genic_upstream_transcript_variant,intron_variant
rs1584862645	genic_upstream_transcript_variant,intron_variant
rs1584862757	genic_upstream_transcript_variant,intron_variant
rs1584862974	genic_upstream_transcript_variant,intron_variant
rs1584863191	genic_upstream_transcript_variant,intron_variant
rs1584863202	genic_upstream_transcript_variant,intron_variant
rs1584863277	genic_upstream_transcript_variant,intron_variant
rs1584863341	genic_upstream_transcript_variant,intron_variant
rs1584863620	genic_upstream_transcript_variant,intron_variant
rs1584863653	genic_upstream_transcript_variant,intron_variant
rs1584863693	genic_upstream_transcript_variant,intron_variant
rs1584863833	genic_upstream_transcript_variant,non_coding_transcript_variant,intron_variant
rs1584863896	genic_upstream_transcript_variant,2KB_upstream_variant,intron_variant,upstream_transcript_variant
rs1584864256	genic_upstream_transcript_variant,2KB_upstream_variant,intron_variant,upstream_transcript_variant
rs1584864279	genic_upstream_transcript_variant,2KB_upstream_variant,intron_variant,upstream_transcript_variant
rs1584864407	genic_upstream_transcript_variant,2KB_upstream_variant,intron_variant,upstream_transcript_variant
rs1584864449	genic_upstream_transcript_variant,2KB_upstream_variant,intron_variant,upstream_transcript_variant
rs1584864721	genic_upstream_transcript_variant,2KB_upstream_variant,intron_variant,upstream_transcript_variant
rs1584864726	genic_upstream_transcript_variant,2KB_upstream_variant,intron_variant,upstream_transcript_variant
rs1584865367	genic_upstream_transcript_variant,2KB_upstream_variant,intron_variant,upstream_transcript_variant
rs1584865439	genic_upstream_transcript_variant,2KB_upstream_variant,intron_variant,upstream_transcript_variant
rs1584865451	genic_upstream_transcript_variant,2KB_upstream_variant,intron_variant,upstream_transcript_variant
rs1584865459	genic_upstream_transcript_variant,2KB_upstream_variant,intron_variant,upstream_transcript_variant
rs1584865517	genic_upstream_transcript_variant,2KB_upstream_variant,intron_variant,upstream_transcript_variant
rs1584865523	genic_upstream_transcript_variant,2KB_upstream_variant,intron_variant,upstream_transcript_variant
rs1584865567	genic_upstream_transcript_variant,2KB_upstream_variant,intron_variant,upstream_transcript_variant
rs1584865807	genic_upstream_transcript_variant,2KB_upstream_variant,intron_variant,upstream_transcript_variant
rs1584865823	genic_upstream_transcript_variant,2KB_upstream_variant,intron_variant,upstream_transcript_variant
rs1584865927	genic_upstream_transcript_variant,2KB_upstream_variant,intron_variant,upstream_transcript_variant
rs1584865931	genic_upstream_transcript_variant,2KB_upstream_variant,intron_variant,upstream_transcript_variant
rs1584865941	genic_upstream_transcript_variant,2KB_upstream_variant,intron_variant,upstream_transcript_variant
rs1584866091	genic_upstream_transcript_variant,2KB_upstream_variant,intron_variant,upstream_transcript_variant

rs1584866193	genic_upstream_transcript_variant,2KB_upstream_variant,intron_variant,upstream_transcript_variant
rs1584866299	genic_upstream_transcript_variant,2KB_upstream_variant,intron_variant,upstream_transcript_variant
rs1584866315	genic_upstream_transcript_variant,2KB_upstream_variant,intron_variant,upstream_transcript_variant
rs1584866394	genic_upstream_transcript_variant,2KB_upstream_variant,intron_variant,upstream_transcript_variant
rs1584866399	genic_upstream_transcript_variant,2KB_upstream_variant,intron_variant,upstream_transcript_variant
rs1584866419	genic_upstream_transcript_variant,2KB_upstream_variant,intron_variant,upstream_transcript_variant
rs1584866439	genic_upstream_transcript_variant,2KB_upstream_variant,intron_variant,upstream_transcript_variant
rs1584866709	genic_upstream_transcript_variant,intron_variant
rs1584866981	genic_upstream_transcript_variant,intron_variant
rs1584867183	genic_upstream_transcript_variant,intron_variant
rs1584867195	genic_upstream_transcript_variant,intron_variant
rs1584867444	genic_upstream_transcript_variant,intron_variant
rs1584867552	genic_upstream_transcript_variant,intron_variant
rs1584867608	genic_upstream_transcript_variant,intron_variant
rs1584867844	genic_upstream_transcript_variant,intron_variant
rs1584867915	genic_upstream_transcript_variant,intron_variant
rs1584868154	genic_upstream_transcript_variant,intron_variant
rs1584868162	genic_upstream_transcript_variant,intron_variant
rs1584868291	genic_upstream_transcript_variant,intron_variant
rs1584868464	genic_upstream_transcript_variant,intron_variant
rs1584868523	genic_upstream_transcript_variant,intron_variant
rs1584868919	genic_upstream_transcript_variant,intron_variant
rs1584869022	genic_upstream_transcript_variant,intron_variant
rs1584869103	genic_upstream_transcript_variant,intron_variant
rs1584869532	genic_upstream_transcript_variant,intron_variant
rs1584869686	genic_upstream_transcript_variant,intron_variant
rs1584869731	genic_upstream_transcript_variant,intron_variant
rs1584869791	genic_upstream_transcript_variant,intron_variant
rs1584869845	genic_upstream_transcript_variant,intron_variant
rs1584869926	genic_upstream_transcript_variant,intron_variant
rs1584869951	genic_upstream_transcript_variant,intron_variant
rs1584870161	genic_upstream_transcript_variant,intron_variant
rs1584870237	genic_upstream_transcript_variant,intron_variant
rs1584870297	genic_upstream_transcript_variant,intron_variant
rs1584870504	genic_upstream_transcript_variant,intron_variant
rs1584870585	genic_upstream_transcript_variant,intron_variant
rs1584870630	genic_upstream_transcript_variant,intron_variant
rs1584870751	genic_upstream_transcript_variant,intron_variant
rs1584870779	genic_upstream_transcript_variant,intron_variant
rs1584870903	genic_upstream_transcript_variant,intron_variant
rs1584870955	genic_upstream_transcript_variant,intron_variant
rs1584871046	genic_upstream_transcript_variant,intron_variant
rs1584871079	genic_upstream_transcript_variant,intron_variant
rs1584871083	genic_upstream_transcript_variant,intron_variant
rs1584871089	genic_upstream_transcript_variant,intron_variant
rs1584871112	genic_upstream_transcript_variant,intron_variant
rs1584871170	genic_upstream_transcript_variant,intron_variant
rs1584871439	genic_upstream_transcript_variant,intron_variant
rs1584871505	genic_upstream_transcript_variant,intron_variant
rs1584871695	genic_upstream_transcript_variant,intron_variant
rs1584871877	genic_upstream_transcript_variant,intron_variant
rs1584871945	genic_upstream_transcript_variant,intron_variant
rs1584872001	genic_upstream_transcript_variant,intron_variant
rs1584872029	genic_upstream_transcript_variant,intron_variant
rs1584872150	genic_upstream_transcript_variant,intron_variant
rs1584872235	genic_upstream_transcript_variant,intron_variant
rs1584872274	genic_upstream_transcript_variant,intron_variant
rs1584872310	genic_upstream_transcript_variant,intron_variant
rs1584872358	genic_upstream_transcript_variant,intron_variant
rs1584872483	genic_upstream_transcript_variant,intron_variant
rs1584872500	genic_upstream_transcript_variant,intron_variant
rs1584872532	genic_upstream_transcript_variant,intron_variant
rs1584872872	genic_upstream_transcript_variant,intron_variant
rs1584873215	genic_upstream_transcript_variant,intron_variant
rs1584873279	genic_upstream_transcript_variant,intron_variant
rs1584873438	genic_upstream_transcript_variant,intron_variant
rs1584873570	genic_upstream_transcript_variant,intron_variant
rs1584873688	genic_upstream_transcript_variant,intron_variant
rs1584873707	genic_upstream_transcript_variant,intron_variant
rs1584873735	genic_upstream_transcript_variant,intron_variant
rs1584873783	genic_upstream_transcript_variant,intron_variant
rs1584873795	genic_upstream_transcript_variant,intron_variant
rs1584873859	genic_upstream_transcript_variant,intron_variant
rs1584874120	genic_upstream_transcript_variant,intron_variant

rs1584874233	genic_upstream_transcript_variant,intron_variant
rs1584874339	genic_upstream_transcript_variant,intron_variant
rs1584874394	genic_upstream_transcript_variant,intron_variant
rs1584874427	genic_upstream_transcript_variant,intron_variant
rs1584874525	genic_upstream_transcript_variant,intron_variant
rs1584874555	genic_upstream_transcript_variant,intron_variant
rs1584874773	genic_upstream_transcript_variant,intron_variant
rs1584874912	genic_upstream_transcript_variant,intron_variant
rs1584875095	genic_upstream_transcript_variant,intron_variant
rs1584875118	genic_upstream_transcript_variant,intron_variant
rs1584875152	genic_upstream_transcript_variant,intron_variant
rs1584875288	genic_upstream_transcript_variant,intron_variant
rs1584875304	genic_upstream_transcript_variant,intron_variant
rs1584875406	genic_upstream_transcript_variant,intron_variant
rs1584875799	genic_upstream_transcript_variant,intron_variant,coding_sequence_variant,non_coding_transcript_variant,missense_variant
rs1584876047	genic_upstream_transcript_variant,intron_variant,coding_sequence_variant,non_coding_transcript_variant,synonymous_variant
rs1584876483	genic_upstream_transcript_variant,intron_variant,coding_sequence_variant,non_coding_transcript_variant,missense_variant
rs1584876493	genic_upstream_transcript_variant,intron_variant,coding_sequence_variant,non_coding_transcript_variant,missense_variant
rs1584876709	genic_upstream_transcript_variant,intron_variant,coding_sequence_variant,non_coding_transcript_variant,missense_variant
rs1584876931	genic_upstream_transcript_variant,intron_variant,coding_sequence_variant,non_coding_transcript_variant,missense_variant
rs1584877029	genic_upstream_transcript_variant,intron_variant,coding_sequence_variant,non_coding_transcript_variant,missense_variant
rs1584877949	genic_upstream_transcript_variant,intron_variant,frameshift_variant,coding_sequence_variant,non_coding_transcript_variant
rs1584878015	genic_upstream_transcript_variant,intron_variant,frameshift_variant,coding_sequence_variant,non_coding_transcript_variant
rs1584878120	genic_upstream_transcript_variant,intron_variant,coding_sequence_variant,non_coding_transcript_variant,missense_variant
rs1584878883	genic_upstream_transcript_variant,intron_variant
rs1584879051	genic_upstream_transcript_variant,intron_variant
rs1584879072	genic_upstream_transcript_variant,intron_variant
rs1584879201	genic_upstream_transcript_variant,intron_variant
rs1584879371	genic_upstream_transcript_variant,intron_variant
rs1584879562	genic_upstream_transcript_variant,intron_variant
rs1584879597	genic_upstream_transcript_variant,intron_variant
rs1584879643	genic_upstream_transcript_variant,intron_variant
rs1584879748	genic_upstream_transcript_variant,intron_variant
rs1584879846	genic_upstream_transcript_variant,intron_variant
rs1584879861	genic_upstream_transcript_variant,intron_variant
rs1584880314	genic_upstream_transcript_variant,intron_variant
rs1584880639	genic_upstream_transcript_variant,intron_variant
rs1584880650	genic_upstream_transcript_variant,intron_variant
rs1584880661	genic_upstream_transcript_variant,intron_variant
rs1584880666	genic_upstream_transcript_variant,intron_variant
rs1584880673	genic_upstream_transcript_variant,intron_variant
rs1584881124	genic_upstream_transcript_variant,intron_variant
rs1584881144	genic_upstream_transcript_variant,intron_variant
rs1584881235	genic_upstream_transcript_variant,intron_variant
rs1584881260	genic_upstream_transcript_variant,intron_variant
rs1584881304	genic_upstream_transcript_variant,intron_variant
rs1584881351	genic_upstream_transcript_variant,intron_variant
rs1584881372	genic_upstream_transcript_variant,intron_variant
rs1584881505	genic_upstream_transcript_variant,intron_variant
rs1584881597	genic_upstream_transcript_variant,intron_variant
rs1584881636	genic_upstream_transcript_variant,intron_variant
rs1584881775	genic_upstream_transcript_variant,intron_variant
rs1584882046	genic_upstream_transcript_variant,intron_variant
rs1584882175	genic_upstream_transcript_variant,intron_variant
rs1584882308	genic_upstream_transcript_variant,intron_variant
rs1584882408	genic_upstream_transcript_variant,intron_variant
rs1584882422	genic_upstream_transcript_variant,intron_variant
rs1584882531	genic_upstream_transcript_variant,intron_variant
rs1584882594	genic_upstream_transcript_variant,intron_variant
rs1584882728	genic_upstream_transcript_variant,intron_variant
rs1584882852	genic_upstream_transcript_variant,intron_variant
rs1584883328	genic_upstream_transcript_variant,intron_variant
rs1584883420	genic_upstream_transcript_variant,intron_variant
rs1584883568	genic_upstream_transcript_variant,intron_variant
rs1584883654	genic_upstream_transcript_variant,intron_variant
rs1584883794	genic_upstream_transcript_variant,intron_variant
rs1584883895	genic_upstream_transcript_variant,intron_variant
rs1584883984	genic_upstream_transcript_variant,intron_variant
rs1584884374	genic_upstream_transcript_variant,intron_variant
rs1584884459	genic_upstream_transcript_variant,intron_variant
rs1584884468	genic_upstream_transcript_variant,intron_variant
rs1584884490	genic_upstream_transcript_variant,intron_variant
rs1584884770	genic_upstream_transcript_variant,intron_variant
rs1584884844	genic_upstream_transcript_variant,intron_variant

rs1584885023	genic_upstream_transcript_variant,intron_variant
rs1584885193	genic_upstream_transcript_variant,intron_variant
rs1584885250	genic_upstream_transcript_variant,intron_variant
rs1584885273	genic_upstream_transcript_variant,intron_variant
rs1584885456	genic_upstream_transcript_variant,intron_variant
rs1584885627	genic_upstream_transcript_variant,intron_variant
rs1584885665	genic_upstream_transcript_variant,intron_variant
rs1584885671	genic_upstream_transcript_variant,intron_variant
rs1584885826	genic_upstream_transcript_variant,intron_variant
rs1584885951	genic_upstream_transcript_variant,intron_variant
rs1584886266	genic_upstream_transcript_variant,intron_variant
rs1584886316	genic_upstream_transcript_variant,intron_variant
rs1584886534	genic_upstream_transcript_variant,intron_variant
rs1584886647	genic_upstream_transcript_variant,intron_variant
rs1584886699	genic_upstream_transcript_variant,intron_variant
rs1584886791	genic_upstream_transcript_variant,intron_variant
rs1584886965	genic_upstream_transcript_variant,intron_variant
rs1584887003	genic_upstream_transcript_variant,intron_variant
rs1584887020	genic_upstream_transcript_variant,intron_variant
rs1584887175	genic_upstream_transcript_variant,intron_variant
rs1584887355	genic_upstream_transcript_variant,intron_variant
rs1584887543	genic_upstream_transcript_variant,intron_variant
rs1584888316	genic_upstream_transcript_variant,intron_variant
rs1584888460	genic_upstream_transcript_variant,intron_variant
rs1584888465	genic_upstream_transcript_variant,intron_variant
rs1584888719	genic_upstream_transcript_variant,intron_variant
rs1584888808	genic_upstream_transcript_variant,intron_variant
rs1584888861	genic_upstream_transcript_variant,intron_variant
rs1584888885	genic_upstream_transcript_variant,intron_variant
rs1584889036	genic_upstream_transcript_variant,intron_variant
rs1584889051	genic_upstream_transcript_variant,intron_variant
rs1584889056	genic_upstream_transcript_variant,intron_variant
rs1584889199	genic_upstream_transcript_variant,intron_variant
rs1584889219	genic_upstream_transcript_variant,intron_variant
rs1584889266	genic_upstream_transcript_variant,intron_variant
rs1584889330	genic_upstream_transcript_variant,intron_variant
rs1584889368	genic_upstream_transcript_variant,intron_variant
rs1584889498	genic_upstream_transcript_variant,intron_variant
rs1584889607	genic_upstream_transcript_variant,intron_variant
rs1584889616	genic_upstream_transcript_variant,intron_variant
rs1584889623	genic_upstream_transcript_variant,intron_variant
rs1584889875	genic_upstream_transcript_variant,intron_variant
rs1584889966	genic_upstream_transcript_variant,intron_variant
rs1584890020	genic_upstream_transcript_variant,intron_variant
rs1584890150	genic_upstream_transcript_variant,intron_variant
rs1584890168	genic_upstream_transcript_variant,intron_variant
rs1584890182	genic_upstream_transcript_variant,intron_variant
rs1584890291	genic_upstream_transcript_variant,intron_variant
rs1584890334	genic_upstream_transcript_variant,intron_variant
rs1584890434	genic_upstream_transcript_variant,intron_variant
rs1584890447	genic_upstream_transcript_variant,intron_variant
rs1584890498	genic_upstream_transcript_variant,intron_variant
rs1584890660	genic_upstream_transcript_variant,intron_variant
rs1584891009	genic_upstream_transcript_variant,intron_variant
rs1584891551	genic_upstream_transcript_variant,intron_variant
rs1584891809	genic_upstream_transcript_variant,intron_variant
rs1584891826	genic_upstream_transcript_variant,intron_variant
rs1584891849	genic_upstream_transcript_variant,intron_variant
rs1584891874	genic_upstream_transcript_variant,intron_variant
rs1584891947	genic_upstream_transcript_variant,intron_variant
rs1584891988	genic_upstream_transcript_variant,intron_variant
rs1584892108	genic_upstream_transcript_variant,intron_variant
rs1584892224	genic_upstream_transcript_variant,intron_variant
rs1584892349	genic_upstream_transcript_variant,intron_variant
rs1584892396	genic_upstream_transcript_variant,intron_variant
rs1584892398	genic_upstream_transcript_variant,intron_variant
rs1584892477	genic_upstream_transcript_variant,intron_variant
rs1584892586	genic_upstream_transcript_variant,intron_variant
rs1584892695	genic_upstream_transcript_variant,intron_variant
rs1584892702	genic_upstream_transcript_variant,intron_variant
rs1584892775	genic_upstream_transcript_variant,intron_variant
rs1584893074	genic_upstream_transcript_variant,intron_variant
rs1584893261	genic_upstream_transcript_variant,intron_variant
rs1584893715	genic_upstream_transcript_variant,intron_variant

rs1584893736	genic_upstream_transcript_variant,intron_variant
rs1584893834	genic_upstream_transcript_variant,intron_variant
rs1584894335	genic_upstream_transcript_variant,intron_variant
rs1584894365	genic_upstream_transcript_variant,intron_variant
rs1584894580	genic_upstream_transcript_variant,intron_variant
rs1584895590	genic_upstream_transcript_variant,intron_variant
rs1584895646	genic_upstream_transcript_variant,intron_variant
rs1584895687	genic_upstream_transcript_variant,intron_variant
rs1584895723	genic_upstream_transcript_variant,intron_variant
rs1584895973	genic_upstream_transcript_variant,intron_variant
rs1584896097	genic_upstream_transcript_variant,intron_variant
rs1584896312	genic_upstream_transcript_variant,intron_variant
rs1584896319	genic_upstream_transcript_variant,intron_variant
rs1584896333	genic_upstream_transcript_variant,intron_variant
rs1584896447	genic_upstream_transcript_variant,intron_variant
rs1584896539	genic_upstream_transcript_variant,intron_variant
rs1584896948	genic_upstream_transcript_variant,intron_variant
rs1584897057	genic_upstream_transcript_variant,intron_variant
rs1584897345	genic_upstream_transcript_variant,intron_variant
rs1584897421	genic_upstream_transcript_variant,intron_variant
rs1584897435	genic_upstream_transcript_variant,intron_variant
rs1584897463	genic_upstream_transcript_variant,intron_variant
rs1584897481	genic_upstream_transcript_variant,intron_variant
rs1584897540	genic_upstream_transcript_variant,intron_variant
rs1584897616	genic_upstream_transcript_variant,intron_variant
rs1584897694	genic_upstream_transcript_variant,intron_variant
rs1584897836	genic_upstream_transcript_variant,intron_variant
rs1584897888	genic_upstream_transcript_variant,intron_variant
rs1584897916	genic_upstream_transcript_variant,intron_variant
rs1584898266	genic_upstream_transcript_variant,intron_variant
rs1584898880	genic_upstream_transcript_variant,intron_variant
rs1584898964	genic_upstream_transcript_variant,intron_variant
rs1584898983	genic_upstream_transcript_variant,intron_variant
rs1584899051	genic_upstream_transcript_variant,intron_variant
rs1584899150	genic_upstream_transcript_variant,intron_variant
rs1584899230	genic_upstream_transcript_variant,intron_variant
rs1584899285	genic_upstream_transcript_variant,intron_variant
rs1584899306	genic_upstream_transcript_variant,intron_variant
rs1584899411	genic_upstream_transcript_variant,intron_variant
rs1584899511	genic_upstream_transcript_variant,intron_variant
rs1584899557	genic_upstream_transcript_variant,intron_variant
rs1584899566	genic_upstream_transcript_variant,intron_variant
rs1584899929	genic_upstream_transcript_variant,intron_variant
rs1584899948	genic_upstream_transcript_variant,intron_variant
rs1584899959	genic_upstream_transcript_variant,intron_variant
rs1584899994	genic_upstream_transcript_variant,intron_variant
rs1584900001	genic_upstream_transcript_variant,intron_variant
rs1584900012	genic_upstream_transcript_variant,intron_variant
rs1584900016	genic_upstream_transcript_variant,intron_variant
rs1584900020	genic_upstream_transcript_variant,intron_variant
rs1584900025	genic_upstream_transcript_variant,intron_variant
rs1584900037	genic_upstream_transcript_variant,intron_variant
rs1584900047	genic_upstream_transcript_variant,intron_variant
rs1584900060	genic_upstream_transcript_variant,intron_variant
rs1584900075	genic_upstream_transcript_variant,intron_variant
rs1584900127	genic_upstream_transcript_variant,intron_variant
rs1584900206	genic_upstream_transcript_variant,intron_variant
rs1584900243	genic_upstream_transcript_variant,intron_variant
rs1584900389	genic_upstream_transcript_variant,intron_variant
rs1584900439	genic_upstream_transcript_variant,intron_variant
rs1584900459	genic_upstream_transcript_variant,intron_variant
rs1584900465	genic_upstream_transcript_variant,intron_variant
rs1584900585	genic_upstream_transcript_variant,intron_variant
rs1584900624	genic_upstream_transcript_variant,intron_variant
rs1584900674	genic_upstream_transcript_variant,intron_variant
rs1584900741	genic_upstream_transcript_variant,intron_variant
rs1584900752	genic_upstream_transcript_variant,intron_variant
rs1584900800	genic_upstream_transcript_variant,intron_variant
rs1584900825	genic_upstream_transcript_variant,intron_variant
rs1584900832	genic_upstream_transcript_variant,intron_variant
rs1584900848	genic_upstream_transcript_variant,intron_variant
rs1584900974	genic_upstream_transcript_variant,intron_variant
rs1584901034	genic_upstream_transcript_variant,intron_variant
rs1584901097	genic_upstream_transcript_variant,intron_variant

rs1584901101	genic_upstream_transcript_variant,intron_variant
rs1584901139	genic_upstream_transcript_variant,intron_variant
rs1584901248	genic_upstream_transcript_variant,intron_variant
rs1584901262	genic_upstream_transcript_variant,intron_variant
rs1584901269	genic_upstream_transcript_variant,intron_variant
rs1584901302	genic_upstream_transcript_variant,intron_variant
rs1584901330	genic_upstream_transcript_variant,intron_variant
rs1584901353	genic_upstream_transcript_variant,intron_variant
rs1584901378	genic_upstream_transcript_variant,intron_variant
rs1584901478	genic_upstream_transcript_variant,intron_variant
rs1584901700	genic_upstream_transcript_variant,intron_variant
rs1584901714	genic_upstream_transcript_variant,intron_variant
rs1584901845	genic_upstream_transcript_variant,intron_variant
rs1584901941	genic_upstream_transcript_variant,intron_variant
rs1584902044	genic_upstream_transcript_variant,intron_variant
rs1584902098	genic_upstream_transcript_variant,intron_variant
rs1584902314	genic_upstream_transcript_variant,intron_variant
rs1584902565	genic_upstream_transcript_variant,intron_variant
rs1584902574	genic_upstream_transcript_variant,intron_variant
rs1584902651	genic_upstream_transcript_variant,intron_variant,upstream_transcript_variant
rs1584902717	genic_upstream_transcript_variant,intron_variant,upstream_transcript_variant
rs1584902841	genic_upstream_transcript_variant,intron_variant,upstream_transcript_variant
rs1584903048	upstream_transcript_variant,genic_upstream_transcript_variant,intron_variant
rs1584903081	upstream_transcript_variant,genic_upstream_transcript_variant,intron_variant
rs1584903114	upstream_transcript_variant,genic_upstream_transcript_variant,intron_variant
rs1584903247	upstream_transcript_variant,genic_upstream_transcript_variant,intron_variant
rs1584903276	upstream_transcript_variant,genic_upstream_transcript_variant,intron_variant
rs1584903534	upstream_transcript_variant,genic_upstream_transcript_variant,intron_variant
rs1584903742	upstream_transcript_variant,genic_upstream_transcript_variant,intron_variant
rs1584903974	upstream_transcript_variant,genic_upstream_transcript_variant,intron_variant
rs1584904110	upstream_transcript_variant,genic_upstream_transcript_variant,intron_variant
rs1584904180	upstream_transcript_variant,genic_upstream_transcript_variant,intron_variant
rs1584904214	upstream_transcript_variant,genic_upstream_transcript_variant,intron_variant
rs1584904239	upstream_transcript_variant,genic_upstream_transcript_variant,intron_variant
rs1584904258	upstream_transcript_variant,genic_upstream_transcript_variant,intron_variant
rs1584904300	upstream_transcript_variant,genic_upstream_transcript_variant,intron_variant
rs1584904323	upstream_transcript_variant,genic_upstream_transcript_variant,intron_variant
rs1584904383	upstream_transcript_variant,genic_upstream_transcript_variant,intron_variant
rs1584904442	upstream_transcript_variant,genic_upstream_transcript_variant,intron_variant
rs1584904512	upstream_transcript_variant,genic_upstream_transcript_variant,intron_variant
rs1584904542	upstream_transcript_variant,genic_upstream_transcript_variant,intron_variant
rs1584904554	upstream_transcript_variant,genic_upstream_transcript_variant,intron_variant
rs1584904592	upstream_transcript_variant,genic_upstream_transcript_variant,intron_variant
rs1584904724	upstream_transcript_variant,genic_upstream_transcript_variant,intron_variant
rs1584904733	upstream_transcript_variant,genic_upstream_transcript_variant,intron_variant
rs1584904749	upstream_transcript_variant,genic_upstream_transcript_variant,intron_variant
rs1584904753	upstream_transcript_variant,genic_upstream_transcript_variant,intron_variant
rs1584904845	upstream_transcript_variant,genic_upstream_transcript_variant,intron_variant
rs1584904883	upstream_transcript_variant,genic_upstream_transcript_variant,intron_variant
rs1584904987	upstream_transcript_variant,genic_upstream_transcript_variant,intron_variant
rs1584905037	upstream_transcript_variant,genic_upstream_transcript_variant,intron_variant
rs1584905066	upstream_transcript_variant,genic_upstream_transcript_variant,intron_variant
rs1584905080	upstream_transcript_variant,genic_upstream_transcript_variant,intron_variant
rs1584905084	upstream_transcript_variant,genic_upstream_transcript_variant,intron_variant
rs1584905120	upstream_transcript_variant,genic_upstream_transcript_variant,intron_variant
rs1584905139	upstream_transcript_variant,genic_upstream_transcript_variant,intron_variant
rs1584905211	upstream_transcript_variant,genic_upstream_transcript_variant,intron_variant
rs1584905214	upstream_transcript_variant,genic_upstream_transcript_variant,intron_variant
rs1584905343	5_prime_UTR_variant,intron_variant
rs1584905364	5_prime_UTR_variant,intron_variant
rs1584905480	intron_variant
rs1584905692	intron_variant
rs1584905721	intron_variant
rs1584905908	intron_variant
rs1584906041	intron_variant
rs1584906052	intron_variant
rs1584906566	intron_variant
rs1584906591	intron_variant
rs1584906732	intron_variant
rs1584907086	intron_variant
rs1584907405	intron_variant
rs1584907413	intron_variant
rs1584907693	intron_variant
rs1584907872	intron_variant

rs1584907910	intron_variant
rs1584907935	intron_variant
rs1584908047	intron_variant
rs1584908061	intron_variant
rs1584908066	intron_variant
rs1584908080	intron_variant
rs1584908105	intron_variant
rs1584908141	intron_variant
rs1584908219	intron_variant
rs1584908366	intron_variant
rs1584908457	intron_variant
rs1584908468	intron_variant
rs1584908508	intron_variant
rs1584908557	intron_variant
rs1584908988	intron_variant
rs1584908996	intron_variant
rs1584909029	intron_variant
rs1584909160	intron_variant
rs1584909266	intron_variant
rs1584909293	intron_variant
rs1584909496	intron_variant
rs1584909735	intron_variant
rs1584909773	intron_variant
rs1584909883	intron_variant
rs1584910158	intron_variant
rs1584910513	intron_variant
rs1584910586	intron_variant
rs1584910674	intron_variant
rs1584910749	intron_variant
rs1584910815	intron_variant
rs1584910833	intron_variant
rs1584910998	intron_variant
rs1584911047	intron_variant
rs1584911106	intron_variant
rs1584911152	intron_variant
rs1584911308	intron_variant
rs1584911329	intron_variant
rs1584911357	intron_variant
rs1584911514	intron_variant
rs1584911580	intron_variant
rs1584911924	intron_variant
rs1584912078	intron_variant
rs1584912123	intron_variant
rs1584912170	intron_variant
rs1584912209	intron_variant
rs1584912324	intron_variant
rs1584912502	intron_variant
rs1584912540	intron_variant
rs1584912675	intron_variant
rs1584912718	intron_variant
rs1584912740	intron_variant
rs1584912771	intron_variant
rs1584912774	intron_variant
rs1584912784	intron_variant
rs1584913105	intron_variant
rs1584913367	intron_variant
rs1584913501	intron_variant
rs1584913590	intron_variant
rs1584913605	intron_variant
rs1584913702	intron_variant
rs1584914083	non_coding_transcript_variant,5_prime_UTR_variant,coding_sequence_variant,missense_variant
rs1584914352	non_coding_transcript_variant,coding_sequence_variant,missense_variant
rs1584915468	intron_variant
rs1584915494	intron_variant
rs1584915689	intron_variant
rs1584915709	intron_variant
rs1584915813	intron_variant
rs1584915831	intron_variant
rs1584916231	intron_variant
rs1584916396	intron_variant
rs1584916414	intron_variant
rs1584916523	intron_variant
rs1584916589	intron_variant
rs1584916652	intron_variant

rs1584916715	intron_variant
rs1584916762	intron_variant
rs1584916766	intron_variant
rs1584916778	intron_variant
rs1584916927	intron_variant
rs1584917071	intron_variant
rs1584917082	intron_variant
rs1584917181	intron_variant
rs1584917356	intron_variant
rs1584917476	intron_variant
rs1584917547	intron_variant
rs1584917558	intron_variant
rs1584917631	intron_variant
rs1584917674	intron_variant
rs1584917721	intron_variant
rs1584917737	intron_variant
rs1584917932	intron_variant
rs1584917987	intron_variant
rs1584918099	intron_variant
rs1584918125	intron_variant
rs1584918161	intron_variant
rs1584918167	intron_variant
rs1584918238	intron_variant
rs1584918320	intron_variant
rs1584918343	intron_variant
rs1584918549	intron_variant
rs1584918804	intron_variant
rs1584918830	intron_variant
rs1584919061	intron_variant
rs1584919293	intron_variant
rs1584919302	intron_variant
rs1584919324	intron_variant
rs1584919380	intron_variant
rs1584919467	intron_variant
rs1584919474	intron_variant
rs1584919500	intron_variant
rs1584919560	intron_variant
rs1584919626	intron_variant
rs1584919840	intron_variant
rs1584920331	intron_variant
rs1584920334	intron_variant
rs1584920456	intron_variant
rs1584920606	intron_variant
rs1584920676	intron_variant
rs1584920701	intron_variant
rs1584920846	intron_variant
rs1584920910	intron_variant
rs1584920951	intron_variant
rs1584921175	intron_variant
rs1584921254	intron_variant
rs1584921370	intron_variant
rs1584921403	intron_variant
rs1584921441	intron_variant
rs1584921531	intron_variant
rs1584921636	intron_variant
rs1584921698	intron_variant
rs1584922248	splice_donor_variant
rs1584922544	intron_variant
rs1584922594	intron_variant
rs1584922941	intron_variant
rs1584923252	non_coding_transcript_variant,inframe_indel,coding_sequence_variant
rs1584923277	non_coding_transcript_variant,frameshift_variant,coding_sequence_variant
rs1584923682	intron_variant
rs1584923701	intron_variant
rs1584923747	intron_variant
rs1584923794	intron_variant
rs1584924106	intron_variant
rs1584924330	intron_variant
rs1584924496	intron_variant
rs1584924511	intron_variant
rs1584924572	intron_variant
rs1584924723	intron_variant
rs1584924907	intron_variant
rs1584925202	intron_variant

rs1584925248	intron_variant
rs1584925302	intron_variant
rs1584925457	intron_variant
rs1584925724	intron_variant
rs1584925749	intron_variant
rs1584925880	intron_variant
rs1584925901	intron_variant
rs1584926141	intron_variant
rs1584926179	intron_variant
rs1584926295	intron_variant
rs1584926304	intron_variant
rs1584926470	intron_variant
rs1584926499	intron_variant
rs1584926632	intron_variant
rs1584927086	intron_variant
rs1584927158	intron_variant
rs1584927163	intron_variant
rs1584927350	intron_variant
rs1584927491	intron_variant
rs1584927514	intron_variant
rs1584927589	intron_variant
rs1584927643	intron_variant
rs1584927720	intron_variant
rs1584927750	intron_variant
rs1584928042	intron_variant
rs1584928247	intron_variant
rs1584928318	intron_variant
rs1584928355	intron_variant
rs1584928496	intron_variant
rs1584928605	intron_variant
rs1584928811	intron_variant
rs1584928946	intron_variant
rs1584929012	intron_variant
rs1584929078	intron_variant
rs1584929287	intron_variant
rs1584929301	intron_variant
rs1584929327	intron_variant
rs1584929344	intron_variant
rs1584929356	intron_variant
rs1584929361	intron_variant
rs1584929560	intron_variant
rs1584929635	intron_variant
rs1584929758	intron_variant
rs1584929815	intron_variant
rs1584929835	intron_variant
rs1584930019	intron_variant
rs1584930049	intron_variant
rs1584930169	intron_variant
rs1584930393	intron_variant
rs1584930551	intron_variant
rs1584930874	intron_variant
rs1584930923	intron_variant
rs1584931002	intron_variant
rs1584931010	intron_variant
rs1584931014	intron_variant
rs1584931126	intron_variant
rs1584931386	intron_variant
rs1584931538	intron_variant
rs1584931774	intron_variant
rs1584931956	intron_variant
rs1584931961	intron_variant
rs1584932035	intron_variant
rs1584932208	intron_variant
rs1584932228	intron_variant
rs1584932234	intron_variant
rs1584932243	intron_variant
rs1584932246	intron_variant
rs1584932445	intron_variant
rs1584932602	intron_variant
rs1584932817	intron_variant
rs1584932829	intron_variant
rs1584932887	intron_variant
rs1584932937	intron_variant
rs1584932968	intron_variant

rs1584933065	intron_variant
rs1584933084	intron_variant
rs1584933116	intron_variant
rs1584933166	intron_variant
rs1584933169	intron_variant
rs1584933638	intron_variant
rs1584933755	intron_variant
rs1584933780	intron_variant
rs1584933806	intron_variant
rs1584933862	intron_variant
rs1584933993	intron_variant
rs1584934004	intron_variant
rs1584934092	intron_variant
rs1584934099	intron_variant
rs1584934136	intron_variant
rs1584934361	intron_variant
rs1584934460	intron_variant
rs1584934480	intron_variant
rs1584934499	intron_variant
rs1584934508	intron_variant
rs1584934526	intron_variant
rs1584934534	intron_variant
rs1584934556	intron_variant
rs1584934786	intron_variant
rs1584934821	intron_variant
rs1584934938	intron_variant
rs1584934971	intron_variant
rs1584934979	intron_variant
rs1584934998	intron_variant
rs1584935002	intron_variant
rs1584935158	intron_variant
rs1584935226	intron_variant
rs1584935230	intron_variant
rs1584935377	intron_variant
rs1584935601	intron_variant
rs1584935682	intron_variant
rs1584935785	intron_variant
rs1584935845	intron_variant
rs1584936017	intron_variant
rs1584936070	intron_variant
rs1584936262	intron_variant
rs1584936349	intron_variant
rs1584936462	intron_variant
rs1584936498	intron_variant
rs1584936859	intron_variant
rs1584936992	intron_variant
rs1584937022	intron_variant
rs1584937032	intron_variant
rs1584937132	intron_variant
rs1584937148	intron_variant
rs1584937263	intron_variant
rs1584937287	intron_variant
rs1584937304	intron_variant
rs1584937328	intron_variant
rs1584937359	intron_variant
rs1584937420	intron_variant
rs1584937493	intron_variant
rs1584937504	intron_variant
rs1584937551	intron_variant
rs1584937784	intron_variant
rs1584937998	intron_variant
rs1584938226	intron_variant
rs1584938269	intron_variant
rs1584938352	intron_variant
rs1584938398	intron_variant
rs1584938415	intron_variant
rs1584938785	intron_variant
rs1584938817	intron_variant
rs1584939376	intron_variant
rs1584939390	intron_variant
rs1584939403	intron_variant
rs1584939580	intron_variant
rs1584939596	intron_variant
rs1584939762	intron_variant

rs1584939949	intron_variant
rs1584940397	intron_variant
rs1584940765	intron_variant
rs1584940807	intron_variant
rs1584940904	intron_variant
rs1584941072	intron_variant
rs1584941399	intron_variant
rs1584941706	non_coding_transcript_variant,coding_sequence_variant,missense_variant
rs1584941722	non_coding_transcript_variant,synonymous_variant,coding_sequence_variant
rs1584942154	intron_variant
rs1584942168	intron_variant
rs1584942436	intron_variant
rs1584942727	non_coding_transcript_variant,synonymous_variant,coding_sequence_variant
rs1584943119	intron_variant
rs1584943187	intron_variant
rs1584943266	intron_variant
rs1584943308	intron_variant
rs1584943661	missense_variant,intron_variant,coding_sequence_variant
rs1584943686	synonymous_variant,intron_variant,coding_sequence_variant
rs1584944191	intron_variant
rs1584944213	intron_variant
rs1584944357	intron_variant
rs1584944815	intron_variant
rs1584944871	intron_variant
rs1584945169	intron_variant
rs1584945240	intron_variant
rs1584945253	intron_variant
rs1584945336	intron_variant
rs1584945362	intron_variant
rs1584945407	intron_variant
rs1584945419	intron_variant
rs1584945466	intron_variant
rs1584945471	intron_variant
rs1584945497	intron_variant
rs1584945552	intron_variant
rs1584945579	intron_variant
rs1584945626	intron_variant
rs1584945708	intron_variant
rs1584945798	intron_variant
rs1584945938	intron_variant
rs1584945972	intron_variant
rs1584946365	intron_variant
rs1584946490	intron_variant
rs1584946566	intron_variant
rs1584947168	non_coding_transcript_variant,synonymous_variant,coding_sequence_variant
rs1584947519	intron_variant
rs1584947683	intron_variant
rs1584948052	intron_variant
rs1584948364	intron_variant
rs1584948401	intron_variant
rs1584948434	intron_variant
rs1584948594	intron_variant
rs1584948679	intron_variant
rs1584948754	intron_variant
rs1584948798	intron_variant
rs1584948850	intron_variant
rs1584948868	intron_variant
rs1584948881	intron_variant
rs1584948961	intron_variant
rs1584948982	intron_variant
rs1584948996	intron_variant
rs1584949026	intron_variant
rs1584949033	intron_variant
rs1584949056	intron_variant
rs1584949161	intron_variant
rs1584949501	intron_variant
rs1584949973	intron_variant
rs1584950020	intron_variant
rs1584950042	intron_variant
rs1584950133	intron_variant
rs1584950162	intron_variant
rs1584950472	intron_variant
rs1584950509	intron_variant
rs1584950579	intron_variant

rs1584950593	intron_variant
rs1584950634	intron_variant
rs1584950640	intron_variant
rs1584950685	intron_variant
rs1584950700	intron_variant
rs1584950725	intron_variant
rs1584950762	intron_variant
rs1584950776	intron_variant
rs1584950843	intron_variant
rs1584950994	intron_variant
rs1584951022	intron_variant
rs1584951093	intron_variant
rs1584951202	intron_variant
rs1584951321	intron_variant
rs1584951333	intron_variant
rs1584951356	intron_variant
rs1584951768	intron_variant
rs1584951782	intron_variant
rs1584951833	intron_variant
rs1584951915	intron_variant
rs1584952076	intron_variant
rs1584952135	intron_variant
rs1584952374	intron_variant
rs1584952493	intron_variant
rs1584952549	intron_variant
rs1584952560	intron_variant
rs1584952613	intron_variant
rs1584952685	intron_variant
rs1584952835	intron_variant
rs1584952840	intron_variant
rs1584953009	intron_variant
rs1584953099	intron_variant
rs1584953148	non_coding_transcript_variant,splice_acceptor_variant,intron_variant,coding_sequence_variant
rs1584953779	genic_downstream_transcript_variant,intron_variant,downstream_transcript_variant
rs1584953834	genic_downstream_transcript_variant,intron_variant,downstream_transcript_variant
rs1584953940	genic_downstream_transcript_variant,intron_variant,downstream_transcript_variant
rs1584954106	genic_downstream_transcript_variant,intron_variant
rs1584954154	genic_downstream_transcript_variant,intron_variant
rs1584954252	genic_downstream_transcript_variant,intron_variant
rs1584954269	genic_downstream_transcript_variant,intron_variant
rs1584954309	genic_downstream_transcript_variant,intron_variant
rs1584954332	genic_downstream_transcript_variant,intron_variant
rs1584954591	genic_downstream_transcript_variant,intron_variant
rs1584954801	genic_downstream_transcript_variant,intron_variant
rs1584954822	genic_downstream_transcript_variant,intron_variant
rs1584955164	genic_downstream_transcript_variant,intron_variant
rs1584955564	genic_downstream_transcript_variant,intron_variant
rs1584955607	genic_downstream_transcript_variant,intron_variant
rs1584955693	genic_downstream_transcript_variant,intron_variant
rs1584955859	genic_downstream_transcript_variant,intron_variant
rs1584956027	genic_downstream_transcript_variant,intron_variant
rs1584956105	genic_downstream_transcript_variant,intron_variant
rs1584956141	genic_downstream_transcript_variant,intron_variant
rs1584956176	genic_downstream_transcript_variant,intron_variant
rs1584956261	genic_downstream_transcript_variant,intron_variant
rs1584956339	genic_downstream_transcript_variant,intron_variant
rs1584956342	genic_downstream_transcript_variant,intron_variant
rs1584956475	genic_downstream_transcript_variant,intron_variant
rs1584956623	genic_downstream_transcript_variant,intron_variant
rs1584956638	genic_downstream_transcript_variant,intron_variant
rs1584956668	genic_downstream_transcript_variant,intron_variant
rs1584956705	genic_downstream_transcript_variant,intron_variant
rs1584956714	genic_downstream_transcript_variant,intron_variant
rs1584956805	genic_downstream_transcript_variant,intron_variant
rs1584956831	genic_downstream_transcript_variant,intron_variant
rs1584956925	genic_downstream_transcript_variant,intron_variant
rs1584956956	genic_downstream_transcript_variant,intron_variant
rs1584956971	genic_downstream_transcript_variant,intron_variant
rs1584957057	genic_downstream_transcript_variant,intron_variant
rs1584957107	genic_downstream_transcript_variant,intron_variant
rs1584957136	genic_downstream_transcript_variant,intron_variant
rs1584957254	genic_downstream_transcript_variant,intron_variant
rs1584957268	genic_downstream_transcript_variant,intron_variant
rs1584957411	genic_downstream_transcript_variant,intron_variant

rs1584957421	genic_downstream_transcript_variant,intron_variant
rs1584957501	genic_downstream_transcript_variant,intron_variant
rs1584957524	genic_downstream_transcript_variant,intron_variant
rs1584957539	genic_downstream_transcript_variant,intron_variant
rs1584957583	genic_downstream_transcript_variant,intron_variant
rs1584958297	genic_downstream_transcript_variant,intron_variant
rs1584958306	genic_downstream_transcript_variant,intron_variant
rs1584958311	genic_downstream_transcript_variant,intron_variant
rs1584958321	genic_downstream_transcript_variant,intron_variant
rs1584958388	genic_downstream_transcript_variant,intron_variant
rs1584958410	genic_downstream_transcript_variant,intron_variant
rs1584958460	genic_downstream_transcript_variant,intron_variant
rs1584958560	genic_downstream_transcript_variant,intron_variant
rs1584958681	genic_downstream_transcript_variant,intron_variant
rs1584958836	genic_downstream_transcript_variant,intron_variant
rs1584958925	genic_downstream_transcript_variant,intron_variant
rs1584958978	genic_downstream_transcript_variant,intron_variant
rs1584959016	genic_downstream_transcript_variant,intron_variant
rs1584959020	genic_downstream_transcript_variant,intron_variant
rs1584959025	genic_downstream_transcript_variant,intron_variant
rs1584959028	genic_downstream_transcript_variant,intron_variant
rs1584959064	genic_downstream_transcript_variant,intron_variant
rs1584959172	genic_downstream_transcript_variant,intron_variant
rs1584959308	genic_downstream_transcript_variant,intron_variant
rs1584959388	genic_downstream_transcript_variant,intron_variant
rs1584959582	genic_downstream_transcript_variant,intron_variant
rs1584959679	genic_downstream_transcript_variant,intron_variant
rs1584959687	genic_downstream_transcript_variant,intron_variant
rs1584959733	genic_downstream_transcript_variant,intron_variant
rs1584960165	genic_downstream_transcript_variant,intron_variant
rs1584960960	genic_downstream_transcript_variant,intron_variant
rs1584961463	genic_downstream_transcript_variant,intron_variant
rs1584961506	genic_downstream_transcript_variant,intron_variant
rs1584961525	genic_downstream_transcript_variant,intron_variant
rs1584961664	genic_downstream_transcript_variant,intron_variant
rs1584961700	genic_downstream_transcript_variant,intron_variant
rs1584961975	genic_downstream_transcript_variant,intron_variant
rs1584962162	genic_downstream_transcript_variant,intron_variant
rs1584962196	genic_downstream_transcript_variant,intron_variant
rs1584962202	genic_downstream_transcript_variant,intron_variant
rs1584962206	genic_downstream_transcript_variant,intron_variant
rs1584962247	genic_downstream_transcript_variant,intron_variant
rs1584962402	genic_downstream_transcript_variant,intron_variant
rs1584962481	genic_downstream_transcript_variant,intron_variant
rs1584962642	genic_downstream_transcript_variant,intron_variant
rs1584962880	genic_downstream_transcript_variant,intron_variant
rs1584962883	genic_downstream_transcript_variant,intron_variant
rs1584962904	genic_downstream_transcript_variant,intron_variant
rs1584962912	genic_downstream_transcript_variant,intron_variant
rs1584963106	genic_downstream_transcript_variant,intron_variant
rs1584963132	genic_downstream_transcript_variant,intron_variant
rs1584963221	genic_downstream_transcript_variant,intron_variant
rs1584963244	genic_downstream_transcript_variant,intron_variant
rs1584963342	genic_downstream_transcript_variant,intron_variant
rs1584963393	genic_downstream_transcript_variant,intron_variant
rs1584963454	genic_downstream_transcript_variant,intron_variant
rs1584963483	genic_downstream_transcript_variant,intron_variant
rs1584963516	genic_downstream_transcript_variant,intron_variant
rs1584963584	genic_downstream_transcript_variant,intron_variant
rs1584963606	genic_downstream_transcript_variant,intron_variant
rs1584963677	genic_downstream_transcript_variant,intron_variant
rs1584963681	genic_downstream_transcript_variant,intron_variant
rs1584963819	genic_downstream_transcript_variant,intron_variant
rs1584963832	genic_downstream_transcript_variant,intron_variant
rs1584963859	genic_downstream_transcript_variant,intron_variant
rs1584963871	genic_downstream_transcript_variant,intron_variant
rs1584964019	genic_downstream_transcript_variant,intron_variant
rs1584964033	genic_downstream_transcript_variant,intron_variant
rs1584964082	genic_downstream_transcript_variant,intron_variant
rs1584964128	genic_downstream_transcript_variant,intron_variant
rs1584964474	genic_downstream_transcript_variant,intron_variant
rs1584964619	genic_downstream_transcript_variant,intron_variant
rs1584964668	genic_downstream_transcript_variant,intron_variant
rs1584965258	genic_downstream_transcript_variant,intron_variant

rs1584965325	genic_downstream_transcript_variant,intron_variant
rs1584965593	synonymous_variant,non_coding_transcript_variant,genic_downstream_transcript_variant,coding_sequence_variant
rs1584965830	genic_downstream_transcript_variant,intron_variant
rs1584966160	genic_downstream_transcript_variant,intron_variant
rs1584966230	genic_downstream_transcript_variant,intron_variant
rs1584966255	genic_downstream_transcript_variant,intron_variant
rs1584966317	genic_downstream_transcript_variant,intron_variant
rs1584966453	genic_downstream_transcript_variant,intron_variant
rs1584966477	genic_downstream_transcript_variant,intron_variant
rs1584966489	genic_downstream_transcript_variant,intron_variant
rs1584966942	genic_downstream_transcript_variant,intron_variant
rs1584966951	genic_downstream_transcript_variant,intron_variant
rs1584966961	genic_downstream_transcript_variant,intron_variant
rs1584967168	genic_downstream_transcript_variant,intron_variant
rs1584967216	genic_downstream_transcript_variant,intron_variant
rs1584967286	genic_downstream_transcript_variant,intron_variant
rs1584967377	genic_downstream_transcript_variant,intron_variant
rs1584967542	genic_downstream_transcript_variant,intron_variant
rs1584967671	genic_downstream_transcript_variant,intron_variant
rs1584967712	genic_downstream_transcript_variant,intron_variant
rs1584967761	genic_downstream_transcript_variant,intron_variant
rs1584967808	genic_downstream_transcript_variant,intron_variant
rs1584967840	genic_downstream_transcript_variant,intron_variant
rs1584967937	genic_downstream_transcript_variant,intron_variant
rs1584967979	genic_downstream_transcript_variant,intron_variant
rs1584968087	genic_downstream_transcript_variant,intron_variant
rs1584968345	genic_downstream_transcript_variant,intron_variant
rs1584969084	genic_downstream_transcript_variant,intron_variant
rs1584969139	genic_downstream_transcript_variant,intron_variant
rs1584969145	genic_downstream_transcript_variant,intron_variant
rs1584969158	genic_downstream_transcript_variant,intron_variant
rs1584969181	genic_downstream_transcript_variant,intron_variant
rs1584969268	genic_downstream_transcript_variant,intron_variant
rs1584969394	genic_downstream_transcript_variant,intron_variant
rs1584969467	genic_downstream_transcript_variant,intron_variant
rs1584969557	genic_downstream_transcript_variant,intron_variant
rs1584969618	genic_downstream_transcript_variant,intron_variant
rs1584969620	genic_downstream_transcript_variant,intron_variant
rs1584969669	genic_downstream_transcript_variant,intron_variant
rs1584969717	genic_downstream_transcript_variant,intron_variant
rs1584969747	genic_downstream_transcript_variant,intron_variant
rs1584969890	genic_downstream_transcript_variant,intron_variant
rs1584969933	genic_downstream_transcript_variant,intron_variant
rs1584969976	genic_downstream_transcript_variant,intron_variant
rs1584970068	genic_downstream_transcript_variant,intron_variant
rs1584970343	genic_downstream_transcript_variant,intron_variant
rs1584970506	genic_downstream_transcript_variant,intron_variant
rs1584970541	genic_downstream_transcript_variant,intron_variant
rs1584971043	genic_downstream_transcript_variant,intron_variant
rs1584971091	genic_downstream_transcript_variant,intron_variant
rs1584971247	genic_downstream_transcript_variant,intron_variant
rs1584971493	genic_downstream_transcript_variant,intron_variant
rs1584971529	genic_downstream_transcript_variant,intron_variant
rs1584971554	genic_downstream_transcript_variant,intron_variant
rs1584971657	genic_downstream_transcript_variant,intron_variant
rs1584971780	genic_downstream_transcript_variant,intron_variant
rs1584972021	genic_downstream_transcript_variant,intron_variant
rs1584972040	genic_downstream_transcript_variant,intron_variant
rs1584972127	genic_downstream_transcript_variant,intron_variant
rs1584972433	genic_downstream_transcript_variant,intron_variant
rs1584972646	genic_downstream_transcript_variant,intron_variant
rs1584972889	genic_downstream_transcript_variant,intron_variant
rs1584973089	genic_downstream_transcript_variant,intron_variant
rs1584973110	genic_downstream_transcript_variant,intron_variant
rs1584973132	genic_downstream_transcript_variant,intron_variant
rs1584973148	genic_downstream_transcript_variant,intron_variant
rs1584973243	genic_downstream_transcript_variant,intron_variant
rs1584973276	genic_downstream_transcript_variant,intron_variant
rs1584973282	genic_downstream_transcript_variant,intron_variant
rs1584973446	genic_downstream_transcript_variant,intron_variant
rs1584973721	genic_downstream_transcript_variant,intron_variant
rs1584973751	genic_downstream_transcript_variant,intron_variant
rs1584973758	genic_downstream_transcript_variant,intron_variant
rs1584973760	genic_downstream_transcript_variant,intron_variant

rs1584973914	genic_downstream_transcript_variant,intron_variant
rs1584973985	genic_downstream_transcript_variant,intron_variant
rs1584974006	genic_downstream_transcript_variant,intron_variant
rs1584974050	genic_downstream_transcript_variant,intron_variant
rs1584974153	genic_downstream_transcript_variant,intron_variant
rs1584974209	genic_downstream_transcript_variant,intron_variant
rs1584974266	genic_downstream_transcript_variant,intron_variant
rs1584974554	genic_downstream_transcript_variant,intron_variant
rs1584974640	genic_downstream_transcript_variant,intron_variant
rs1584974721	genic_downstream_transcript_variant,intron_variant
rs1584974729	genic_downstream_transcript_variant,intron_variant
rs1584974811	genic_downstream_transcript_variant,intron_variant
rs1584974911	genic_downstream_transcript_variant,intron_variant
rs1584975040	genic_downstream_transcript_variant,intron_variant
rs1584975076	genic_downstream_transcript_variant,intron_variant
rs1584975134	genic_downstream_transcript_variant,intron_variant
rs1584975157	genic_downstream_transcript_variant,intron_variant
rs1584975190	genic_downstream_transcript_variant,intron_variant
rs1584975192	genic_downstream_transcript_variant,intron_variant
rs1584975196	genic_downstream_transcript_variant,intron_variant
rs1584975215	genic_downstream_transcript_variant,intron_variant
rs1584975373	genic_downstream_transcript_variant,intron_variant
rs1584975425	genic_downstream_transcript_variant,intron_variant
rs1584975538	genic_downstream_transcript_variant,intron_variant
rs1584975618	genic_downstream_transcript_variant,intron_variant
rs1584975691	genic_downstream_transcript_variant,intron_variant
rs1584975800	genic_downstream_transcript_variant,intron_variant
rs1584975882	genic_downstream_transcript_variant,intron_variant
rs1584975926	genic_downstream_transcript_variant,intron_variant
rs1584975991	genic_downstream_transcript_variant,intron_variant
rs1584976019	genic_downstream_transcript_variant,intron_variant
rs1584976096	genic_downstream_transcript_variant,intron_variant
rs1584976137	genic_downstream_transcript_variant,intron_variant
rs1584976143	genic_downstream_transcript_variant,intron_variant
rs1584976199	genic_downstream_transcript_variant,intron_variant
rs1584976241	genic_downstream_transcript_variant,intron_variant
rs1584976305	genic_downstream_transcript_variant,intron_variant
rs1584976322	genic_downstream_transcript_variant,intron_variant
rs1584976347	genic_downstream_transcript_variant,intron_variant
rs1584976405	genic_downstream_transcript_variant,intron_variant
rs1584976507	genic_downstream_transcript_variant,intron_variant
rs1584976769	genic_downstream_transcript_variant,intron_variant
rs1584976998	genic_downstream_transcript_variant,intron_variant
rs1584977207	genic_downstream_transcript_variant,intron_variant
rs1584977348	genic_downstream_transcript_variant,intron_variant
rs1584977385	genic_downstream_transcript_variant,intron_variant
rs1584977402	genic_downstream_transcript_variant,intron_variant
rs1584977551	genic_downstream_transcript_variant,intron_variant
rs1584977864	non_coding_transcript_variant,genic_downstream_transcript_variant,coding_sequence_variant,missense_variant
rs1584977903	synonymous_variant,non_coding_transcript_variant,genic_downstream_transcript_variant,coding_sequence_variant
rs1584978256	non_coding_transcript_variant,genic_downstream_transcript_variant,coding_sequence_variant,missense_variant
rs1584978380	non_coding_transcript_variant,genic_downstream_transcript_variant,coding_sequence_variant,missense_variant
rs1584978763	3_prime_UTR_variant,non_coding_transcript_variant,genic_downstream_transcript_variant
rs1584978945	3_prime_UTR_variant,non_coding_transcript_variant,genic_downstream_transcript_variant
rs1584979030	3_prime_UTR_variant,non_coding_transcript_variant,genic_downstream_transcript_variant
rs1584979138	3_prime_UTR_variant,non_coding_transcript_variant,genic_downstream_transcript_variant
rs1584979142	3_prime_UTR_variant,non_coding_transcript_variant,genic_downstream_transcript_variant
rs1584979189	3_prime_UTR_variant,non_coding_transcript_variant,genic_downstream_transcript_variant
rs1584979209	3_prime_UTR_variant,non_coding_transcript_variant,genic_downstream_transcript_variant
rs1584979254	3_prime_UTR_variant,non_coding_transcript_variant,genic_downstream_transcript_variant
rs1584979504	3_prime_UTR_variant,non_coding_transcript_variant,genic_downstream_transcript_variant
rs1584979523	3_prime_UTR_variant,non_coding_transcript_variant,genic_downstream_transcript_variant
rs1584979580	3_prime_UTR_variant,non_coding_transcript_variant,genic_downstream_transcript_variant
rs1584979626	3_prime_UTR_variant,non_coding_transcript_variant,genic_downstream_transcript_variant
rs1584979681	3_prime_UTR_variant,non_coding_transcript_variant,genic_downstream_transcript_variant
rs1584979865	3_prime_UTR_variant,non_coding_transcript_variant,genic_downstream_transcript_variant
rs1584979998	3_prime_UTR_variant,non_coding_transcript_variant,genic_downstream_transcript_variant
rs1584980548	3_prime_UTR_variant,non_coding_transcript_variant,genic_downstream_transcript_variant
rs1584980609	3_prime_UTR_variant,non_coding_transcript_variant,genic_downstream_transcript_variant
rs1584980703	3_prime_UTR_variant,non_coding_transcript_variant,genic_downstream_transcript_variant
rs1584980870	500B_downstream_variant,downstream_transcript_variant
rs1584980878	500B_downstream_variant,downstream_transcript_variant
rs1584981035	500B_downstream_variant,downstream_transcript_variant
rs1584981082	500B_downstream_variant,downstream_transcript_variant

rs1584981085	500B_downstream_variant,downstream_transcript_variant
rs1584981095	500B_downstream_variant,downstream_transcript_variant
rs1584981125	500B_downstream_variant,downstream_transcript_variant
rs1584981129	500B_downstream_variant,downstream_transcript_variant
rs1645496432	intron_variant
rs1645508828	synonymous_variant,non_coding_transcript_variant,genic_downstream_transcript_variant,coding_sequence_variant
rs1646127058	genic_upstream_transcript_variant,intron_variant
rs1646403099	intron_variant
rs1646403177	intron_variant
rs1646455636	genic_downstream_transcript_variant,intron_variant
rs1646623252	genic_downstream_transcript_variant,intron_variant
rs1647529618	upstream_transcript_variant,genic_upstream_transcript_variant,2KB_upstream_variant,intron_variant
rs1648874305	genic_upstream_transcript_variant,intron_variant
rs1648946997	intron_variant
rs1649526292	genic_downstream_transcript_variant,intron_variant
rs1649849364	intron_variant
rs1650133288	intron_variant
rs1650392731	intron_variant
rs1652523961	genic_upstream_transcript_variant,intron_variant
rs1652648037	intron_variant
rs1652827909	intron_variant
rs1654489186	genic_downstream_transcript_variant,intron_variant
rs1654988362	genic_upstream_transcript_variant,intron_variant
rs1654994152	intron_variant
rs1655095635	intron_variant
rs1655275278	genic_upstream_transcript_variant,intron_variant
rs1655304316	genic_upstream_transcript_variant,intron_variant
rs1656492171	genic_upstream_transcript_variant,intron_variant
rs1656840813	intron_variant
rs1658404070	genic_upstream_transcript_variant,intron_variant
rs1660551555	genic_upstream_transcript_variant,intron_variant
rs1660843275	genic_upstream_transcript_variant,intron_variant
rs1661046565	genic_upstream_transcript_variant,intron_variant
rs1661652345	intron_variant
rs1662524128	genic_downstream_transcript_variant,intron_variant
rs1664007137	genic_upstream_transcript_variant,intron_variant
rs1664365779	genic_upstream_transcript_variant,intron_variant
rs1665271449	genic_downstream_transcript_variant,intron_variant
rs1665508086	intron_variant
rs1666941242	genic_upstream_transcript_variant,intron_variant
rs1668055239	genic_downstream_transcript_variant,intron_variant,downstream_transcript_variant
rs1669440686	genic_downstream_transcript_variant,intron_variant
rs1669457827	genic_upstream_transcript_variant,intron_variant
rs1672390373	genic_upstream_transcript_variant,intron_variant
rs1672521299	intron_variant
rs1674934575	intron_variant
rs1676151911	intron_variant
rs1678612564	upstream_transcript_variant,genic_upstream_transcript_variant,2KB_upstream_variant,intron_variant
rs1678767768	genic_upstream_transcript_variant,intron_variant
rs1678867450	genic_upstream_transcript_variant,intron_variant
rs1680382158	intron_variant
rs1681108648	intron_variant
rs1682060253	intron_variant
rs1682765553	genic_upstream_transcript_variant,intron_variant
rs1683695276	genic_downstream_transcript_variant,intron_variant
rs1684242372	intron_variant
rs1684361337	genic_downstream_transcript_variant,intron_variant
rs1684524095	intron_variant
rs1685693591	intron_variant
rs1688397396	genic_downstream_transcript_variant,intron_variant
rs1688606612	genic_downstream_transcript_variant,intron_variant
rs1688744375	intron_variant
rs1689030042	genic_downstream_transcript_variant,intron_variant
rs1689179092	genic_downstream_transcript_variant,intron_variant
rs1690406619	genic_upstream_transcript_variant,intron_variant
rs1692328774	genic_upstream_transcript_variant,intron_variant
rs1692773356	genic_upstream_transcript_variant,intron_variant
rs1692922937	genic_upstream_transcript_variant,intron_variant
rs1693598836	genic_downstream_transcript_variant,intron_variant
rs1694388457	intron_variant
rs1695231098	intron_variant
rs1695512272	genic_downstream_transcript_variant,intron_variant
rs1698847415	genic_upstream_transcript_variant,intron_variant
rs1701447957	genic_upstream_transcript_variant,intron_variant

rs1702912130	intron_variant
rs1704008086	intron_variant
rs1704970940	genic_upstream_transcript_variant,intron_variant
rs1706963329	genic_upstream_transcript_variant,intron_variant
rs1707201288	upstream_transcript_variant,genic_upstream_transcript_variant,2KB_upstream_variant,intron_variant
rs1707959800	intron_variant
rs1711772762	genic_upstream_transcript_variant,intron_variant
rs1711960030	intron_variant
rs1712019828	genic_downstream_transcript_variant,intron_variant
rs1712386071	genic_upstream_transcript_variant,intron_variant
rs1712654787	intron_variant
rs1712966987	genic_downstream_transcript_variant,intron_variant
rs1713589107	genic_upstream_transcript_variant,intron_variant
rs1713771180	genic_downstream_transcript_variant,intron_variant
rs1714103615	genic_downstream_transcript_variant,intron_variant
rs1714591383	genic_upstream_transcript_variant,intron_variant
rs1715363822	genic_upstream_transcript_variant,intron_variant
rs1715634851	intron_variant
rs1715673540	intron_variant
rs1716371690	genic_upstream_transcript_variant,intron_variant
rs1716850916	genic_upstream_transcript_variant,intron_variant
rs1717986038	genic_upstream_transcript_variant,intron_variant
rs1719152365	genic_downstream_transcript_variant,intron_variant
rs1719354080	intron_variant
rs1720243676	intron_variant
rs1720871472	genic_downstream_transcript_variant,intron_variant
rs1721073585	intron_variant
rs1721362395	intron_variant
rs1721467232	genic_upstream_transcript_variant,intron_variant
rs1721487899	genic_upstream_transcript_variant,intron_variant
rs1723085671	genic_downstream_transcript_variant,intron_variant
rs1724400632	genic_upstream_transcript_variant,intron_variant
rs1725698487	genic_downstream_transcript_variant,intron_variant
rs1725943535	intron_variant
rs1726082370	intron_variant
rs1726108177	intron_variant
rs1726763857	genic_upstream_transcript_variant,intron_variant
rs1726872350	genic_downstream_transcript_variant,intron_variant
rs1727254617	intron_variant
rs1727708968	intron_variant
rs1727877907	genic_downstream_transcript_variant,intron_variant
rs1728885982	genic_upstream_transcript_variant,intron_variant
rs1729115849	genic_downstream_transcript_variant,intron_variant
rs1729129984	genic_downstream_transcript_variant,intron_variant
rs1730323315	genic_downstream_transcript_variant,intron_variant
rs1730685847	genic_downstream_transcript_variant,intron_variant
rs1731289326	intron_variant
rs1731334633	genic_upstream_transcript_variant,intron_variant
rs1731659259	intron_variant
rs1734273456	intron_variant
rs1735402544	genic_downstream_transcript_variant,intron_variant
rs1736075344	genic_downstream_transcript_variant,intron_variant
rs1737516075	genic_downstream_transcript_variant,intron_variant
rs1738058012	genic_upstream_transcript_variant,intron_variant
rs1738732277	intron_variant
rs1740878901	genic_upstream_transcript_variant,intron_variant
rs1741968136	intron_variant
rs1742204166	intron_variant
rs1743159624	genic_downstream_transcript_variant,intron_variant
rs1743672414	genic_upstream_transcript_variant,intron_variant
rs1743806511	intron_variant
rs1746726236	intron_variant
rs1747290638	intron_variant
rs1747426052	genic_downstream_transcript_variant,intron_variant
rs1748735219	intron_variant
rs1749051112	genic_downstream_transcript_variant,intron_variant
rs1749556196	genic_downstream_transcript_variant,intron_variant
rs1749711852	3_prime_UTR_variant,non_coding_transcript_variant,genic_downstream_transcript_variant
rs1750446163	intron_variant
rs1750863753	intron_variant
rs1752448925	intron_variant
rs1752883917	intron_variant
rs1752908905	genic_upstream_transcript_variant,intron_variant
rs1753214477	genic_downstream_transcript_variant,intron_variant

rs1753706571	genic_downstream_transcript_variant,intron_variant
rs1755422856	intron_variant
rs1757169023	intron_variant
rs1757600891	genic_upstream_transcript_variant,intron_variant
rs1759312246	intron_variant
rs1759371114	genic_downstream_transcript_variant,intron_variant
rs1760144555	intron_variant
rs1760609999	intron_variant
rs1760651316	intron_variant
rs1762287006	intron_variant
rs1762695209	genic_downstream_transcript_variant,intron_variant,downstream_transcript_variant
rs1763165100	genic_upstream_transcript_variant,intron_variant
rs1763506894	upstream_transcript_variant,genic_upstream_transcript_variant,intron_variant
rs1763614466	genic_upstream_transcript_variant,intron_variant
rs1763652746	intron_variant
rs1763652952	intron_variant
rs1764264355	intron_variant
rs1764636282	genic_downstream_transcript_variant,intron_variant
rs1764836666	genic_downstream_transcript_variant,intron_variant
rs1765944368	genic_downstream_transcript_variant,intron_variant
rs1765990862	genic_upstream_transcript_variant,intron_variant
rs1766653855	intron_variant
rs1767673479	genic_upstream_transcript_variant,intron_variant
rs1768033134	genic_upstream_transcript_variant,intron_variant
rs1769516371	upstream_transcript_variant,genic_upstream_transcript_variant,2KB_upstream_variant,intron_variant
rs1771619509	genic_downstream_transcript_variant,intron_variant
rs1771911315	genic_upstream_transcript_variant,intron_variant
rs1772411131	genic_upstream_transcript_variant,intron_variant
rs1773477147	genic_upstream_transcript_variant,intron_variant
rs1773679224	intron_variant
rs1777664349	genic_upstream_transcript_variant,intron_variant
rs1779530026	genic_upstream_transcript_variant,intron_variant
rs1779826919	intron_variant
rs1781949106	intron_variant
rs1782077179	genic_upstream_transcript_variant,intron_variant
rs1783584590	intron_variant
rs1783939164	genic_upstream_transcript_variant,intron_variant
rs1784838145	intron_variant
rs1785742443	genic_upstream_transcript_variant,intron_variant
rs1786480671	upstream_transcript_variant,intron_variant,genic_upstream_transcript_variant
rs1787702786	genic_downstream_transcript_variant,intron_variant
rs1788717613	genic_upstream_transcript_variant,intron_variant
rs1789989960	intron_variant,genic_downstream_transcript_variant
rs1790046799	5_prime_UTR_variant,non_coding_transcript_variant,genic_upstream_transcript_variant,intron_variant
rs1790207647	genic_downstream_transcript_variant,intron_variant
rs1791464119	coding_sequence_variant,intron_variant,synonymous_variant,non_coding_transcript_variant,genic_upstream_transcript_variant
rs1791464380	coding_sequence_variant,intron_variant,synonymous_variant,non_coding_transcript_variant,genic_upstream_transcript_variant
rs1791464742	coding_sequence_variant,missense_variant,intron_variant,non_coding_transcript_variant,genic_upstream_transcript_variant
rs1791466202	coding_sequence_variant,missense_variant,intron_variant,non_coding_transcript_variant,genic_upstream_transcript_variant
rs1791467122	coding_sequence_variant,missense_variant,intron_variant,non_coding_transcript_variant,genic_upstream_transcript_variant
rs1791469321	coding_sequence_variant,missense_variant,intron_variant,non_coding_transcript_variant,genic_upstream_transcript_variant
rs1791469817	coding_sequence_variant,missense_variant,intron_variant,non_coding_transcript_variant,genic_upstream_transcript_variant
rs1791473061	coding_sequence_variant,missense_variant,intron_variant,non_coding_transcript_variant,genic_upstream_transcript_variant
rs1791473786	coding_sequence_variant,missense_variant,intron_variant,non_coding_transcript_variant,genic_upstream_transcript_variant
rs1791476896	coding_sequence_variant,missense_variant,intron_variant,non_coding_transcript_variant,genic_upstream_transcript_variant
rs1791478666	coding_sequence_variant,missense_variant,intron_variant,non_coding_transcript_variant,genic_upstream_transcript_variant
rs1791479941	coding_sequence_variant,intron_variant,synonymous_variant,non_coding_transcript_variant,genic_upstream_transcript_variant
rs1791486595	coding_sequence_variant,missense_variant,intron_variant,non_coding_transcript_variant,genic_upstream_transcript_variant
rs1791488808	coding_sequence_variant,intron_variant,synonymous_variant,non_coding_transcript_variant,genic_upstream_transcript_variant
rs1791489014	coding_sequence_variant,missense_variant,intron_variant,non_coding_transcript_variant,genic_upstream_transcript_variant
rs1791489742	coding_sequence_variant,intron_variant,synonymous_variant,non_coding_transcript_variant,genic_upstream_transcript_variant
rs1791491463	coding_sequence_variant,missense_variant,intron_variant,non_coding_transcript_variant,genic_upstream_transcript_variant
rs1791491672	coding_sequence_variant,missense_variant,intron_variant,non_coding_transcript_variant,genic_upstream_transcript_variant
rs1791494214	coding_sequence_variant,intron_variant,synonymous_variant,non_coding_transcript_variant,genic_upstream_transcript_variant
rs1791494311	coding_sequence_variant,frameshift_variant,intron_variant,non_coding_transcript_variant,genic_upstream_transcript_variant
rs1791496321	coding_sequence_variant,missense_variant,intron_variant,non_coding_transcript_variant,genic_upstream_transcript_variant
rs1791503123	coding_sequence_variant,intron_variant,synonymous_variant,non_coding_transcript_variant,genic_upstream_transcript_variant
rs1791503359	coding_sequence_variant,missense_variant,intron_variant,non_coding_transcript_variant,genic_upstream_transcript_variant
rs1791505633	coding_sequence_variant,missense_variant,intron_variant,non_coding_transcript_variant,genic_upstream_transcript_variant
rs1791505734	coding_sequence_variant,intron_variant,synonymous_variant,non_coding_transcript_variant,genic_upstream_transcript_variant
rs1791507812	coding_sequence_variant,frameshift_variant,intron_variant,non_coding_transcript_variant,genic_upstream_transcript_variant
rs1791507929	coding_sequence_variant,intron_variant,synonymous_variant,non_coding_transcript_variant,genic_upstream_transcript_variant
rs1791508132	coding_sequence_variant,missense_variant,intron_variant,non_coding_transcript_variant,genic_upstream_transcript_variant
rs1791510135	coding_sequence_variant,missense_variant,intron_variant,non_coding_transcript_variant,genic_upstream_transcript_variant

rs1791510907	coding_sequence_variant,missense_variant,intron_variant,non_coding_transcript_variant,genic_upstream_transcript_variant
rs1791512125	coding_sequence_variant,missense_variant,intron_variant,non_coding_transcript_variant,genic_upstream_transcript_variant
rs1791512313	coding_sequence_variant,missense_variant,intron_variant,non_coding_transcript_variant,genic_upstream_transcript_variant
rs1791512753	coding_sequence_variant,missense_variant,intron_variant,non_coding_transcript_variant,genic_upstream_transcript_variant
rs1791514332	coding_sequence_variant,frameshift_variant,intron_variant,non_coding_transcript_variant,genic_upstream_transcript_variant
rs1791514789	coding_sequence_variant,missense_variant,intron_variant,non_coding_transcript_variant,genic_upstream_transcript_variant
rs1791515202	coding_sequence_variant,intron_variant,synonymous_variant,non_coding_transcript_variant,genic_upstream_transcript_variant
rs1791516586	coding_sequence_variant,missense_variant,intron_variant,non_coding_transcript_variant,genic_upstream_transcript_variant
rs1791522244	coding_sequence_variant,missense_variant,intron_variant,non_coding_transcript_variant,genic_upstream_transcript_variant
rs1791522568	coding_sequence_variant,missense_variant,intron_variant,non_coding_transcript_variant,genic_upstream_transcript_variant
rs1791524001	coding_sequence_variant,missense_variant,intron_variant,non_coding_transcript_variant,genic_upstream_transcript_variant
rs1791524108	coding_sequence_variant,missense_variant,intron_variant,non_coding_transcript_variant,genic_upstream_transcript_variant
rs1791529199	intron_variant,genic_upstream_transcript_variant
rs1791530978	intron_variant,genic_upstream_transcript_variant
rs1791531151	intron_variant,genic_upstream_transcript_variant
rs1791531229	intron_variant,genic_upstream_transcript_variant
rs1791531544	intron_variant,genic_upstream_transcript_variant
rs1791531919	intron_variant,genic_upstream_transcript_variant
rs1791532084	intron_variant,genic_upstream_transcript_variant
rs1791533971	intron_variant,genic_upstream_transcript_variant
rs1791534330	intron_variant,genic_upstream_transcript_variant
rs1791534698	intron_variant,genic_upstream_transcript_variant
rs1791534775	intron_variant,genic_upstream_transcript_variant
rs1791534845	intron_variant,genic_upstream_transcript_variant
rs1791535028	intron_variant,genic_upstream_transcript_variant
rs1791535122	intron_variant,genic_upstream_transcript_variant
rs1791538199	intron_variant,genic_upstream_transcript_variant
rs1791538350	intron_variant,genic_upstream_transcript_variant
rs1791538662	intron_variant,genic_upstream_transcript_variant
rs1791538986	intron_variant,genic_upstream_transcript_variant
rs1791539144	intron_variant,genic_upstream_transcript_variant
rs1791539432	intron_variant,genic_upstream_transcript_variant
rs1791539511	intron_variant,genic_upstream_transcript_variant
rs1791539600	intron_variant,genic_upstream_transcript_variant
rs1791539681	intron_variant,genic_upstream_transcript_variant
rs1791539852	intron_variant,genic_upstream_transcript_variant
rs1791539954	intron_variant,genic_upstream_transcript_variant
rs1791540235	intron_variant,genic_upstream_transcript_variant
rs1791540648	intron_variant,genic_upstream_transcript_variant
rs1791540895	intron_variant,genic_upstream_transcript_variant
rs1791540998	intron_variant,genic_upstream_transcript_variant
rs1791541083	intron_variant,genic_upstream_transcript_variant
rs1791542567	intron_variant,genic_upstream_transcript_variant
rs1791542646	intron_variant,genic_upstream_transcript_variant
rs1791543224	intron_variant,genic_upstream_transcript_variant
rs1791543319	intron_variant,genic_upstream_transcript_variant
rs1791544060	intron_variant,genic_upstream_transcript_variant
rs1791544299	intron_variant,genic_upstream_transcript_variant
rs1791544798	intron_variant,genic_upstream_transcript_variant
rs1791545171	intron_variant,genic_upstream_transcript_variant
rs1791545278	intron_variant,genic_upstream_transcript_variant
rs1791545711	intron_variant,genic_upstream_transcript_variant
rs1791545912	intron_variant,genic_upstream_transcript_variant
rs1791545997	intron_variant,genic_upstream_transcript_variant
rs1791546451	intron_variant,genic_upstream_transcript_variant
rs1791546888	intron_variant,genic_upstream_transcript_variant
rs1791547430	intron_variant,genic_upstream_transcript_variant
rs1791547737	intron_variant,genic_upstream_transcript_variant
rs1791547831	intron_variant,genic_upstream_transcript_variant
rs1791548155	intron_variant,genic_upstream_transcript_variant
rs1791548410	intron_variant,genic_upstream_transcript_variant
rs1791548794	intron_variant,genic_upstream_transcript_variant
rs1791548892	intron_variant,genic_upstream_transcript_variant
rs1791549001	intron_variant,genic_upstream_transcript_variant
rs1791549559	intron_variant,genic_upstream_transcript_variant
rs1791549704	intron_variant,genic_upstream_transcript_variant
rs1791549869	intron_variant,genic_upstream_transcript_variant
rs1791549951	intron_variant,genic_upstream_transcript_variant
rs1791550192	intron_variant,genic_upstream_transcript_variant
rs1791550284	intron_variant,genic_upstream_transcript_variant
rs1791550365	intron_variant,genic_upstream_transcript_variant
rs1791550878	intron_variant,genic_upstream_transcript_variant
rs1791551367	intron_variant,genic_upstream_transcript_variant
rs1791552392	intron_variant,genic_upstream_transcript_variant

rs1791552476	intron_variant,genic_upstream_transcript_variant
rs1791552551	intron_variant,genic_upstream_transcript_variant
rs1791552821	intron_variant,genic_upstream_transcript_variant
rs1791553094	intron_variant,genic_upstream_transcript_variant
rs1791553220	intron_variant,genic_upstream_transcript_variant
rs1791553352	intron_variant,genic_upstream_transcript_variant
rs1791553644	intron_variant,genic_upstream_transcript_variant
rs1791553991	intron_variant,genic_upstream_transcript_variant
rs1791554131	intron_variant,genic_upstream_transcript_variant
rs1791554633	intron_variant,genic_upstream_transcript_variant
rs1791555272	intron_variant,genic_upstream_transcript_variant
rs1791555410	intron_variant,genic_upstream_transcript_variant
rs1791555725	intron_variant,genic_upstream_transcript_variant
rs1791556896	intron_variant,genic_upstream_transcript_variant
rs1791557038	intron_variant,genic_upstream_transcript_variant
rs1791557379	intron_variant,genic_upstream_transcript_variant
rs1791557543	intron_variant,genic_upstream_transcript_variant
rs1791557916	intron_variant,genic_upstream_transcript_variant
rs1791558006	intron_variant,genic_upstream_transcript_variant
rs1791558297	intron_variant,genic_upstream_transcript_variant
rs1791558700	intron_variant,genic_upstream_transcript_variant
rs1791558912	intron_variant,genic_upstream_transcript_variant
rs1791559186	intron_variant,genic_upstream_transcript_variant
rs1791559790	intron_variant,genic_upstream_transcript_variant
rs1791559878	intron_variant,genic_upstream_transcript_variant
rs1791559966	intron_variant,genic_upstream_transcript_variant
rs1791560060	intron_variant,genic_upstream_transcript_variant
rs1791560156	intron_variant,genic_upstream_transcript_variant
rs1791560422	intron_variant,genic_upstream_transcript_variant
rs1791560689	intron_variant,genic_upstream_transcript_variant
rs1791560824	intron_variant,genic_upstream_transcript_variant
rs1791561112	intron_variant,genic_upstream_transcript_variant
rs1791561476	intron_variant,genic_upstream_transcript_variant
rs1791561755	intron_variant,genic_upstream_transcript_variant
rs1791561903	intron_variant,genic_upstream_transcript_variant
rs1791562361	intron_variant,genic_upstream_transcript_variant
rs1791562717	intron_variant,genic_upstream_transcript_variant
rs1791563307	intron_variant,genic_upstream_transcript_variant
rs1791563510	intron_variant,genic_upstream_transcript_variant
rs1791563587	intron_variant,genic_upstream_transcript_variant
rs1791563666	intron_variant,genic_upstream_transcript_variant
rs1791563879	intron_variant,genic_upstream_transcript_variant
rs1791564218	intron_variant,genic_upstream_transcript_variant
rs1791564306	intron_variant,genic_upstream_transcript_variant
rs1791564436	intron_variant,genic_upstream_transcript_variant
rs1791564598	intron_variant,genic_upstream_transcript_variant
rs1791565168	intron_variant,genic_upstream_transcript_variant
rs1791565595	intron_variant,genic_upstream_transcript_variant
rs1791565893	intron_variant,genic_upstream_transcript_variant
rs1791566539	intron_variant,genic_upstream_transcript_variant
rs1791566780	intron_variant,genic_upstream_transcript_variant
rs1791567348	intron_variant,genic_upstream_transcript_variant
rs1791567442	intron_variant,genic_upstream_transcript_variant
rs1791571014	intron_variant,genic_upstream_transcript_variant
rs1791571974	intron_variant,genic_upstream_transcript_variant
rs1791572151	intron_variant,genic_upstream_transcript_variant
rs1791572560	intron_variant,genic_upstream_transcript_variant
rs1791572673	intron_variant,genic_upstream_transcript_variant
rs1791573427	intron_variant,genic_upstream_transcript_variant
rs1791573773	intron_variant,genic_upstream_transcript_variant
rs1791574327	intron_variant,genic_upstream_transcript_variant
rs1791574508	intron_variant,genic_upstream_transcript_variant
rs1791574810	intron_variant,genic_upstream_transcript_variant
rs1791574889	intron_variant,genic_upstream_transcript_variant
rs1791575044	intron_variant,genic_upstream_transcript_variant
rs1791575987	intron_variant,genic_upstream_transcript_variant
rs1791576078	intron_variant,genic_upstream_transcript_variant
rs1791576283	intron_variant,genic_upstream_transcript_variant
rs1791576473	intron_variant,genic_upstream_transcript_variant
rs1791577079	intron_variant,genic_upstream_transcript_variant
rs1791577471	intron_variant,genic_upstream_transcript_variant
rs1791577638	intron_variant,genic_upstream_transcript_variant
rs1791577735	intron_variant,genic_upstream_transcript_variant
rs1791578196	intron_variant,genic_upstream_transcript_variant

rs1791578370	intron_variant,genic_upstream_transcript_variant
rs1791578980	intron_variant,genic_upstream_transcript_variant
rs1791579146	intron_variant,genic_upstream_transcript_variant
rs1791579229	intron_variant,genic_upstream_transcript_variant
rs1791579667	intron_variant,genic_upstream_transcript_variant
rs1791580443	intron_variant,genic_upstream_transcript_variant
rs1791581819	intron_variant,genic_upstream_transcript_variant
rs1791581954	intron_variant,genic_upstream_transcript_variant
rs1791582098	intron_variant,genic_upstream_transcript_variant
rs1791582240	intron_variant,genic_upstream_transcript_variant
rs1791582816	intron_variant,genic_upstream_transcript_variant
rs1791582991	intron_variant,genic_upstream_transcript_variant
rs1791583313	intron_variant,genic_upstream_transcript_variant
rs1791583611	intron_variant,genic_upstream_transcript_variant
rs1791584014	intron_variant,genic_upstream_transcript_variant
rs1791586264	intron_variant,genic_upstream_transcript_variant
rs1791586360	intron_variant,genic_upstream_transcript_variant
rs1791586574	intron_variant,genic_upstream_transcript_variant
rs1791586651	intron_variant,genic_upstream_transcript_variant
rs1791586940	intron_variant,genic_upstream_transcript_variant
rs1791587045	intron_variant,genic_upstream_transcript_variant
rs1791587142	intron_variant,genic_upstream_transcript_variant
rs1791587560	intron_variant,genic_upstream_transcript_variant
rs1791587728	intron_variant,genic_upstream_transcript_variant
rs1791587814	intron_variant,genic_upstream_transcript_variant
rs1791588182	intron_variant,genic_upstream_transcript_variant
rs1791589273	intron_variant,genic_upstream_transcript_variant
rs1791589465	intron_variant,genic_upstream_transcript_variant
rs1791589553	intron_variant,genic_upstream_transcript_variant
rs1791589646	intron_variant,genic_upstream_transcript_variant
rs1791589803	intron_variant,genic_upstream_transcript_variant
rs1791589899	intron_variant,genic_upstream_transcript_variant
rs1791590080	intron_variant,genic_upstream_transcript_variant
rs1791590172	intron_variant,genic_upstream_transcript_variant
rs1791590624	intron_variant,genic_upstream_transcript_variant
rs1791590726	intron_variant,genic_upstream_transcript_variant
rs1791591909	intron_variant,genic_upstream_transcript_variant
rs1791592001	intron_variant,genic_upstream_transcript_variant
rs1791592089	intron_variant,genic_upstream_transcript_variant
rs1791592187	intron_variant,genic_upstream_transcript_variant
rs1791592434	intron_variant,genic_upstream_transcript_variant
rs1791592529	intron_variant,genic_upstream_transcript_variant
rs1791592927	intron_variant,genic_upstream_transcript_variant
rs1791592999	intron_variant,genic_upstream_transcript_variant
rs1791593077	intron_variant,genic_upstream_transcript_variant
rs1791593291	intron_variant,genic_upstream_transcript_variant
rs1791593618	intron_variant,genic_upstream_transcript_variant
rs1791593720	intron_variant,genic_upstream_transcript_variant
rs1791593804	intron_variant,genic_upstream_transcript_variant
rs1791593900	intron_variant,genic_upstream_transcript_variant
rs1791594104	intron_variant,genic_upstream_transcript_variant
rs1791594256	intron_variant,genic_upstream_transcript_variant
rs1791594669	intron_variant,genic_upstream_transcript_variant
rs1791595375	intron_variant,genic_upstream_transcript_variant
rs1791595456	intron_variant,genic_upstream_transcript_variant
rs1791596288	intron_variant,genic_upstream_transcript_variant
rs1791596698	intron_variant,genic_upstream_transcript_variant
rs1791597046	intron_variant,genic_upstream_transcript_variant
rs1791597228	intron_variant,genic_upstream_transcript_variant
rs1791597786	intron_variant,genic_upstream_transcript_variant
rs1791597961	intron_variant,genic_upstream_transcript_variant
rs1791598485	intron_variant,genic_upstream_transcript_variant
rs1791598570	intron_variant,genic_upstream_transcript_variant
rs1791598947	intron_variant,genic_upstream_transcript_variant
rs1791599355	intron_variant,genic_upstream_transcript_variant
rs1791599546	intron_variant,genic_upstream_transcript_variant
rs1791600037	intron_variant,genic_upstream_transcript_variant
rs1791600126	intron_variant,genic_upstream_transcript_variant
rs1791600561	intron_variant,genic_upstream_transcript_variant
rs1791600867	intron_variant,genic_upstream_transcript_variant
rs1791602297	intron_variant,genic_upstream_transcript_variant
rs1791602369	intron_variant,genic_upstream_transcript_variant
rs1791605569	intron_variant,genic_upstream_transcript_variant
rs1791606004	intron_variant,genic_upstream_transcript_variant

rs1791606254	intron_variant,genic_upstream_transcript_variant
rs1791606345	intron_variant,genic_upstream_transcript_variant
rs1791606457	intron_variant,genic_upstream_transcript_variant
rs1791606797	intron_variant,genic_upstream_transcript_variant
rs1791607119	intron_variant,genic_upstream_transcript_variant
rs1791607739	intron_variant,genic_upstream_transcript_variant
rs1791607913	intron_variant,genic_upstream_transcript_variant
rs1791608015	intron_variant,genic_upstream_transcript_variant
rs1791608497	intron_variant,genic_upstream_transcript_variant
rs1791608604	intron_variant,genic_upstream_transcript_variant
rs1791608687	intron_variant,genic_upstream_transcript_variant
rs1791608772	intron_variant,genic_upstream_transcript_variant
rs1791608975	intron_variant,genic_upstream_transcript_variant
rs1791609060	intron_variant,genic_upstream_transcript_variant
rs1791611599	intron_variant,genic_upstream_transcript_variant
rs1791611787	intron_variant,genic_upstream_transcript_variant
rs1791613170	intron_variant,genic_upstream_transcript_variant
rs1791613742	intron_variant,genic_upstream_transcript_variant
rs1791613925	intron_variant,genic_upstream_transcript_variant
rs1791614654	intron_variant,genic_upstream_transcript_variant
rs1791615022	intron_variant,genic_upstream_transcript_variant
rs1791615181	intron_variant,genic_upstream_transcript_variant
rs1791615263	intron_variant,genic_upstream_transcript_variant
rs1791615348	intron_variant,genic_upstream_transcript_variant
rs1791615431	intron_variant,genic_upstream_transcript_variant
rs1791615521	intron_variant,genic_upstream_transcript_variant
rs1791615602	intron_variant,genic_upstream_transcript_variant
rs1791615763	intron_variant,genic_upstream_transcript_variant
rs1791615846	intron_variant,genic_upstream_transcript_variant
rs1791616340	intron_variant,genic_upstream_transcript_variant
rs1791616422	intron_variant,genic_upstream_transcript_variant
rs1791616795	intron_variant,genic_upstream_transcript_variant
rs1791617631	intron_variant,genic_upstream_transcript_variant
rs1791617931	intron_variant,genic_upstream_transcript_variant
rs1791618004	intron_variant,genic_upstream_transcript_variant
rs1791618298	intron_variant,genic_upstream_transcript_variant
rs1791618581	intron_variant,genic_upstream_transcript_variant
rs1791618684	intron_variant,genic_upstream_transcript_variant
rs1791618780	intron_variant,genic_upstream_transcript_variant
rs1791618873	intron_variant,genic_upstream_transcript_variant
rs1791619237	intron_variant,genic_upstream_transcript_variant
rs1791619550	intron_variant,genic_upstream_transcript_variant
rs1791620348	intron_variant,genic_upstream_transcript_variant
rs1791620441	intron_variant,genic_upstream_transcript_variant
rs1791621563	intron_variant,genic_upstream_transcript_variant
rs1791621905	intron_variant,genic_upstream_transcript_variant
rs1791622186	intron_variant,genic_upstream_transcript_variant
rs1791622277	intron_variant,genic_upstream_transcript_variant
rs1791622350	intron_variant,genic_upstream_transcript_variant
rs1791622766	intron_variant,genic_upstream_transcript_variant
rs1791622882	intron_variant,genic_upstream_transcript_variant
rs1791623150	intron_variant,genic_upstream_transcript_variant
rs1791623572	intron_variant,genic_upstream_transcript_variant
rs1791623865	intron_variant,genic_upstream_transcript_variant
rs1791624825	intron_variant,genic_upstream_transcript_variant
rs1791625105	intron_variant,genic_upstream_transcript_variant
rs1791626123	intron_variant,genic_upstream_transcript_variant
rs1791626326	intron_variant,genic_upstream_transcript_variant
rs1791626414	intron_variant,genic_upstream_transcript_variant
rs1791627179	intron_variant,genic_upstream_transcript_variant
rs1791627564	intron_variant,genic_upstream_transcript_variant
rs1791627907	intron_variant,genic_upstream_transcript_variant
rs1791628436	intron_variant,genic_upstream_transcript_variant
rs1791628583	intron_variant,genic_upstream_transcript_variant
rs1791629317	intron_variant,genic_upstream_transcript_variant
rs1791630039	intron_variant,genic_upstream_transcript_variant
rs1791630829	intron_variant,genic_upstream_transcript_variant
rs1791630966	intron_variant,genic_upstream_transcript_variant
rs1791631718	intron_variant,genic_upstream_transcript_variant
rs1791632255	intron_variant,genic_upstream_transcript_variant
rs1791632351	intron_variant,genic_upstream_transcript_variant
rs1791632443	intron_variant,genic_upstream_transcript_variant
rs1791632544	intron_variant,genic_upstream_transcript_variant
rs1791632655	intron_variant,genic_upstream_transcript_variant

rs1791633626	intron_variant,genic_upstream_transcript_variant
rs1791633724	intron_variant,genic_upstream_transcript_variant
rs1791633821	intron_variant,genic_upstream_transcript_variant
rs1791634007	intron_variant,genic_upstream_transcript_variant
rs1791634176	intron_variant,genic_upstream_transcript_variant
rs1791634526	intron_variant,genic_upstream_transcript_variant
rs1791634968	intron_variant,genic_upstream_transcript_variant
rs1791635430	intron_variant,genic_upstream_transcript_variant
rs1791635924	intron_variant,genic_upstream_transcript_variant
rs1791636024	intron_variant,genic_upstream_transcript_variant
rs1791637233	intron_variant,genic_upstream_transcript_variant
rs1791637315	intron_variant,genic_upstream_transcript_variant
rs1791637416	intron_variant,genic_upstream_transcript_variant
rs1791637509	intron_variant,genic_upstream_transcript_variant
rs1791638022	intron_variant,genic_upstream_transcript_variant
rs1791638407	intron_variant,genic_upstream_transcript_variant
rs1791638688	intron_variant,genic_upstream_transcript_variant
rs1791638774	intron_variant,genic_upstream_transcript_variant
rs1791640504	intron_variant,genic_upstream_transcript_variant
rs1791640823	intron_variant,genic_upstream_transcript_variant
rs1791641607	intron_variant,genic_upstream_transcript_variant
rs1791641756	intron_variant,genic_upstream_transcript_variant
rs1791642322	intron_variant,genic_upstream_transcript_variant
rs1791642426	intron_variant,genic_upstream_transcript_variant
rs1791643528	intron_variant,genic_upstream_transcript_variant
rs1791643630	intron_variant,genic_upstream_transcript_variant
rs1791643921	intron_variant,genic_upstream_transcript_variant
rs1791644030	intron_variant,genic_upstream_transcript_variant
rs1791644235	intron_variant,genic_upstream_transcript_variant
rs1791644345	intron_variant,genic_upstream_transcript_variant
rs1791644563	intron_variant,genic_upstream_transcript_variant
rs1791644702	intron_variant,genic_upstream_transcript_variant
rs1791644844	intron_variant,genic_upstream_transcript_variant
rs1791645137	intron_variant,genic_upstream_transcript_variant
rs1791645578	intron_variant,genic_upstream_transcript_variant
rs1791645879	intron_variant,genic_upstream_transcript_variant
rs1791646028	intron_variant,genic_upstream_transcript_variant
rs1791646317	intron_variant,genic_upstream_transcript_variant
rs1791648789	intron_variant,genic_upstream_transcript_variant
rs1791649628	intron_variant,genic_upstream_transcript_variant
rs1791649814	intron_variant,genic_upstream_transcript_variant
rs1791650157	intron_variant,genic_upstream_transcript_variant
rs1791650257	intron_variant,genic_upstream_transcript_variant
rs1791650463	intron_variant,genic_upstream_transcript_variant
rs1791650743	intron_variant,genic_upstream_transcript_variant
rs1791650830	intron_variant,genic_upstream_transcript_variant
rs1791650920	intron_variant,genic_upstream_transcript_variant
rs1791651111	intron_variant,genic_upstream_transcript_variant
rs1791651203	intron_variant,genic_upstream_transcript_variant
rs1791651714	intron_variant,genic_upstream_transcript_variant
rs1791652376	intron_variant,genic_upstream_transcript_variant
rs1791652880	intron_variant,genic_upstream_transcript_variant
rs1791652968	intron_variant,genic_upstream_transcript_variant
rs1791653067	intron_variant,genic_upstream_transcript_variant
rs1791653523	intron_variant,genic_upstream_transcript_variant
rs1791653747	intron_variant,genic_upstream_transcript_variant
rs1791654215	intron_variant,genic_upstream_transcript_variant
rs1791654533	intron_variant,genic_upstream_transcript_variant
rs1791654842	intron_variant,genic_upstream_transcript_variant
rs1791654941	intron_variant,genic_upstream_transcript_variant
rs1791655037	intron_variant,genic_upstream_transcript_variant
rs1791655206	intron_variant,genic_upstream_transcript_variant
rs1791655487	intron_variant,genic_upstream_transcript_variant
rs1791655663	intron_variant,genic_upstream_transcript_variant
rs1791655745	intron_variant,genic_upstream_transcript_variant
rs1791656014	intron_variant,genic_upstream_transcript_variant
rs1791656513	intron_variant,genic_upstream_transcript_variant
rs1791661168	intron_variant,genic_upstream_transcript_variant
rs1791661356	intron_variant,genic_upstream_transcript_variant
rs1791661808	intron_variant,genic_upstream_transcript_variant
rs1791662110	intron_variant,genic_upstream_transcript_variant
rs1791662252	intron_variant,genic_upstream_transcript_variant
rs1791662415	intron_variant,genic_upstream_transcript_variant
rs1791663799	intron_variant,genic_upstream_transcript_variant

rs1791664809	intron_variant,genic_upstream_transcript_variant
rs1791665087	intron_variant,genic_upstream_transcript_variant
rs1791665236	intron_variant,genic_upstream_transcript_variant
rs1791665899	intron_variant,genic_upstream_transcript_variant
rs1791666042	intron_variant,genic_upstream_transcript_variant
rs1791666174	intron_variant,genic_upstream_transcript_variant
rs1791666457	intron_variant,genic_upstream_transcript_variant
rs1791666558	intron_variant,genic_upstream_transcript_variant
rs1791666839	intron_variant,genic_upstream_transcript_variant
rs1791667142	intron_variant,genic_upstream_transcript_variant
rs1791667233	intron_variant,genic_upstream_transcript_variant
rs1791667428	intron_variant,genic_upstream_transcript_variant
rs1791667617	intron_variant,genic_upstream_transcript_variant
rs1791668126	intron_variant,genic_upstream_transcript_variant
rs1791668217	intron_variant,genic_upstream_transcript_variant
rs1791668421	intron_variant,genic_upstream_transcript_variant
rs1791668956	intron_variant,genic_upstream_transcript_variant
rs1791669067	intron_variant,genic_upstream_transcript_variant
rs1791669400	intron_variant,genic_upstream_transcript_variant
rs1791669570	intron_variant,genic_upstream_transcript_variant
rs1791669666	intron_variant,genic_upstream_transcript_variant
rs1791669754	intron_variant,genic_upstream_transcript_variant
rs1791670879	intron_variant,genic_upstream_transcript_variant
rs1791671027	intron_variant,genic_upstream_transcript_variant
rs1791671133	intron_variant,genic_upstream_transcript_variant
rs1791671312	intron_variant,genic_upstream_transcript_variant
rs1791671501	intron_variant,genic_upstream_transcript_variant
rs1791671854	intron_variant,genic_upstream_transcript_variant
rs1791671948	intron_variant,genic_upstream_transcript_variant
rs1791672040	intron_variant,genic_upstream_transcript_variant
rs1791672129	intron_variant,genic_upstream_transcript_variant
rs1791672243	intron_variant,genic_upstream_transcript_variant
rs1791672323	intron_variant,genic_upstream_transcript_variant
rs1791672878	intron_variant,genic_upstream_transcript_variant
rs1791672966	intron_variant,genic_upstream_transcript_variant
rs1791673041	intron_variant,genic_upstream_transcript_variant
rs1791673130	intron_variant,genic_upstream_transcript_variant
rs1791673231	intron_variant,genic_upstream_transcript_variant
rs1791674084	intron_variant,genic_upstream_transcript_variant
rs1791674347	intron_variant,genic_upstream_transcript_variant
rs1791674785	intron_variant,genic_upstream_transcript_variant
rs1791674882	intron_variant,genic_upstream_transcript_variant
rs1791674981	intron_variant,genic_upstream_transcript_variant
rs1791675273	intron_variant,genic_upstream_transcript_variant
rs1791675488	intron_variant,genic_upstream_transcript_variant
rs1791675715	intron_variant,genic_upstream_transcript_variant
rs1791676006	intron_variant,genic_upstream_transcript_variant
rs1791676280	intron_variant,genic_upstream_transcript_variant
rs1791677360	intron_variant,genic_upstream_transcript_variant
rs1791677445	intron_variant,genic_upstream_transcript_variant
rs1791677547	intron_variant,genic_upstream_transcript_variant
rs1791678005	intron_variant,genic_upstream_transcript_variant
rs1791678495	intron_variant,genic_upstream_transcript_variant
rs1791678996	intron_variant,genic_upstream_transcript_variant
rs1791679705	intron_variant,genic_upstream_transcript_variant
rs1791680202	intron_variant,genic_upstream_transcript_variant
rs1791680633	intron_variant,genic_upstream_transcript_variant
rs1791680828	intron_variant,genic_upstream_transcript_variant
rs1791680916	intron_variant,genic_upstream_transcript_variant
rs1791681131	intron_variant,genic_upstream_transcript_variant
rs1791681629	intron_variant,genic_upstream_transcript_variant
rs1791682159	intron_variant,genic_upstream_transcript_variant
rs1791682410	intron_variant,genic_upstream_transcript_variant
rs1791682492	intron_variant,genic_upstream_transcript_variant
rs1791682673	intron_variant,genic_upstream_transcript_variant
rs1791682761	intron_variant,genic_upstream_transcript_variant
rs1791688935	intron_variant,genic_upstream_transcript_variant
rs1791689451	intron_variant,genic_upstream_transcript_variant
rs1791689877	intron_variant,genic_upstream_transcript_variant
rs1791690240	intron_variant,genic_upstream_transcript_variant
rs1791690686	intron_variant,genic_upstream_transcript_variant
rs1791692291	intron_variant,genic_upstream_transcript_variant
rs1791692571	intron_variant,genic_upstream_transcript_variant
rs1791692890	intron_variant,genic_upstream_transcript_variant

rs1791693031	intron_variant,genic_upstream_transcript_variant
rs1791693173	intron_variant,genic_upstream_transcript_variant
rs1791697133	intron_variant,genic_upstream_transcript_variant
rs1791698248	intron_variant,genic_upstream_transcript_variant
rs1791698405	intron_variant,genic_upstream_transcript_variant
rs1791699068	intron_variant,genic_upstream_transcript_variant
rs1791699420	intron_variant,genic_upstream_transcript_variant
rs1791699591	intron_variant,genic_upstream_transcript_variant
rs1791699748	intron_variant,genic_upstream_transcript_variant
rs1791699888	intron_variant,genic_upstream_transcript_variant
rs1791701372	intron_variant,genic_upstream_transcript_variant
rs1791701538	intron_variant,genic_upstream_transcript_variant
rs1791701837	intron_variant,genic_upstream_transcript_variant
rs1791701980	intron_variant,genic_upstream_transcript_variant
rs1791702247	intron_variant,genic_upstream_transcript_variant
rs1791702382	intron_variant,genic_upstream_transcript_variant
rs1791702687	intron_variant,genic_upstream_transcript_variant
rs1791703834	intron_variant,genic_upstream_transcript_variant
rs1791703970	intron_variant,genic_upstream_transcript_variant
rs1791704764	intron_variant,genic_upstream_transcript_variant
rs1791705082	intron_variant,genic_upstream_transcript_variant
rs1791705383	intron_variant,genic_upstream_transcript_variant
rs1791706177	intron_variant,genic_upstream_transcript_variant
rs1791706340	intron_variant,genic_upstream_transcript_variant
rs1791707335	intron_variant,genic_upstream_transcript_variant
rs1791707498	intron_variant,genic_upstream_transcript_variant
rs1791707666	intron_variant,genic_upstream_transcript_variant
rs1791708124	intron_variant,genic_upstream_transcript_variant
rs1791708289	intron_variant,genic_upstream_transcript_variant
rs1791708433	intron_variant,genic_upstream_transcript_variant
rs1791708927	intron_variant,genic_upstream_transcript_variant
rs1791709438	intron_variant,genic_upstream_transcript_variant
rs1791709750	intron_variant,genic_upstream_transcript_variant
rs1791710077	intron_variant,genic_upstream_transcript_variant
rs1791711002	intron_variant,genic_upstream_transcript_variant
rs1791711168	intron_variant,genic_upstream_transcript_variant
rs1791711997	intron_variant,genic_upstream_transcript_variant
rs1791712352	intron_variant,genic_upstream_transcript_variant
rs1791712788	intron_variant,genic_upstream_transcript_variant
rs1791712952	intron_variant,genic_upstream_transcript_variant
rs1791713117	intron_variant,genic_upstream_transcript_variant
rs1791713255	intron_variant,genic_upstream_transcript_variant
rs1791714447	intron_variant,genic_upstream_transcript_variant
rs1791716105	intron_variant,genic_upstream_transcript_variant
rs1791717684	intron_variant,genic_upstream_transcript_variant
rs1791717876	intron_variant,genic_upstream_transcript_variant
rs1791719562	intron_variant,genic_upstream_transcript_variant
rs1791719741	intron_variant,genic_upstream_transcript_variant
rs1791719898	intron_variant,genic_upstream_transcript_variant
rs1791720034	intron_variant,genic_upstream_transcript_variant
rs1791720179	intron_variant,genic_upstream_transcript_variant
rs1791720474	intron_variant,genic_upstream_transcript_variant
rs1791720979	intron_variant,genic_upstream_transcript_variant
rs1791721134	intron_variant,genic_upstream_transcript_variant
rs1791721277	intron_variant,genic_upstream_transcript_variant
rs1791721423	intron_variant,genic_upstream_transcript_variant
rs1791721578	intron_variant,genic_upstream_transcript_variant
rs1791722811	intron_variant,genic_upstream_transcript_variant
rs1791723180	intron_variant,genic_upstream_transcript_variant
rs1791723616	intron_variant,genic_upstream_transcript_variant
rs1791724064	intron_variant,genic_upstream_transcript_variant
rs1791724279	intron_variant,genic_upstream_transcript_variant
rs1791724674	intron_variant,genic_upstream_transcript_variant
rs1791725265	intron_variant,genic_upstream_transcript_variant
rs1791725700	intron_variant,genic_upstream_transcript_variant
rs1791725837	intron_variant,genic_upstream_transcript_variant
rs1791726531	intron_variant,genic_upstream_transcript_variant
rs1791727076	intron_variant,genic_upstream_transcript_variant
rs1791728110	intron_variant,genic_upstream_transcript_variant
rs1791728407	intron_variant,genic_upstream_transcript_variant
rs1791728618	intron_variant,genic_upstream_transcript_variant
rs1791729321	intron_variant,genic_upstream_transcript_variant
rs1791729522	intron_variant,genic_upstream_transcript_variant
rs1791729884	intron_variant,genic_upstream_transcript_variant

rs1791730779	intron_variant,genic_upstream_transcript_variant
rs1791730887	intron_variant,genic_upstream_transcript_variant
rs1791731506	intron_variant,genic_upstream_transcript_variant
rs1791731680	intron_variant,genic_upstream_transcript_variant
rs1791731858	intron_variant,genic_upstream_transcript_variant
rs1791732058	intron_variant,genic_upstream_transcript_variant
rs1791732590	intron_variant,genic_upstream_transcript_variant
rs1791732686	intron_variant,genic_upstream_transcript_variant
rs1791733198	intron_variant,genic_upstream_transcript_variant
rs1791733572	intron_variant,genic_upstream_transcript_variant
rs1791733676	intron_variant,genic_upstream_transcript_variant
rs1791734087	intron_variant,genic_upstream_transcript_variant
rs1791734196	intron_variant,genic_upstream_transcript_variant
rs1791734315	intron_variant,genic_upstream_transcript_variant
rs1791735246	intron_variant,genic_upstream_transcript_variant
rs1791735471	intron_variant,genic_upstream_transcript_variant
rs1791736001	intron_variant,genic_upstream_transcript_variant
rs1791736761	intron_variant,genic_upstream_transcript_variant
rs1791736967	intron_variant,genic_upstream_transcript_variant
rs1791737203	intron_variant,genic_upstream_transcript_variant
rs1791737313	intron_variant,genic_upstream_transcript_variant
rs1791737797	intron_variant,genic_upstream_transcript_variant
rs1791738027	intron_variant,genic_upstream_transcript_variant
rs1791738125	intron_variant,genic_upstream_transcript_variant
rs1791739431	intron_variant,genic_upstream_transcript_variant
rs1791739508	intron_variant,genic_upstream_transcript_variant
rs1791739592	intron_variant,genic_upstream_transcript_variant
rs1791739687	intron_variant,genic_upstream_transcript_variant
rs1791739783	intron_variant,genic_upstream_transcript_variant
rs1791740112	intron_variant,genic_upstream_transcript_variant
rs1791740792	intron_variant,genic_upstream_transcript_variant
rs1791741113	intron_variant,genic_upstream_transcript_variant
rs1791741266	intron_variant,genic_upstream_transcript_variant
rs1791742135	intron_variant,genic_upstream_transcript_variant
rs1791742906	intron_variant,genic_upstream_transcript_variant
rs1791743046	intron_variant,genic_upstream_transcript_variant
rs1791744145	intron_variant,genic_upstream_transcript_variant
rs1791744311	intron_variant,genic_upstream_transcript_variant
rs1791745054	intron_variant,genic_upstream_transcript_variant
rs1791745486	intron_variant,genic_upstream_transcript_variant
rs1791747023	intron_variant,genic_upstream_transcript_variant
rs1791747893	intron_variant,genic_upstream_transcript_variant
rs1791748221	intron_variant,genic_upstream_transcript_variant
rs1791748783	intron_variant,genic_upstream_transcript_variant
rs1791748940	intron_variant,genic_upstream_transcript_variant
rs1791749209	intron_variant,genic_upstream_transcript_variant
rs1791750020	intron_variant,genic_upstream_transcript_variant
rs1791750520	intron_variant,genic_upstream_transcript_variant
rs1791750669	intron_variant,genic_upstream_transcript_variant
rs1791751158	intron_variant,genic_upstream_transcript_variant
rs1791751660	intron_variant,genic_upstream_transcript_variant
rs1791751827	intron_variant,genic_upstream_transcript_variant
rs1791751999	intron_variant,genic_upstream_transcript_variant
rs1791754144	intron_variant,genic_upstream_transcript_variant
rs1791754859	intron_variant,genic_upstream_transcript_variant
rs1791755195	intron_variant,genic_upstream_transcript_variant
rs1791755659	intron_variant,genic_upstream_transcript_variant
rs1791756283	intron_variant,genic_upstream_transcript_variant
rs1791756379	intron_variant,genic_upstream_transcript_variant
rs1791756481	intron_variant,genic_upstream_transcript_variant
rs1791757907	intron_variant,genic_upstream_transcript_variant
rs1791757997	intron_variant,genic_upstream_transcript_variant
rs1791758190	intron_variant,genic_upstream_transcript_variant
rs1791758755	intron_variant,genic_upstream_transcript_variant
rs1791758924	intron_variant,genic_upstream_transcript_variant
rs1791759118	intron_variant,genic_upstream_transcript_variant
rs1791759299	intron_variant,genic_upstream_transcript_variant
rs1791759706	intron_variant,genic_upstream_transcript_variant
rs1791759802	intron_variant,genic_upstream_transcript_variant
rs1791759908	intron_variant,genic_upstream_transcript_variant
rs1791759995	intron_variant,genic_upstream_transcript_variant
rs1791760083	intron_variant,genic_upstream_transcript_variant
rs1791760360	intron_variant,genic_upstream_transcript_variant
rs1791760447	intron_variant,genic_upstream_transcript_variant

rs1791760597	intron_variant,genic_upstream_transcript_variant
rs1791760699	intron_variant,genic_upstream_transcript_variant
rs1791762027	intron_variant,genic_upstream_transcript_variant
rs1791763608	intron_variant,genic_upstream_transcript_variant
rs1791764429	intron_variant,genic_upstream_transcript_variant
rs1791764956	intron_variant,genic_upstream_transcript_variant
rs1791765300	intron_variant,genic_upstream_transcript_variant
rs1791765852	intron_variant,genic_upstream_transcript_variant
rs1791766158	intron_variant,genic_upstream_transcript_variant
rs1791767006	intron_variant,genic_upstream_transcript_variant
rs1791767206	intron_variant,genic_upstream_transcript_variant
rs1791767304	intron_variant,genic_upstream_transcript_variant
rs1791768416	intron_variant,genic_upstream_transcript_variant
rs1791769242	intron_variant,genic_upstream_transcript_variant
rs1791769713	intron_variant,genic_upstream_transcript_variant
rs1791770014	intron_variant,genic_upstream_transcript_variant
rs1791770385	intron_variant,genic_upstream_transcript_variant
rs1791770656	intron_variant,genic_upstream_transcript_variant
rs1791770804	intron_variant,genic_upstream_transcript_variant
rs1791770997	intron_variant,genic_upstream_transcript_variant
rs1791771611	intron_variant,genic_upstream_transcript_variant
rs1791771888	intron_variant,genic_upstream_transcript_variant
rs1791772010	intron_variant,genic_upstream_transcript_variant
rs1791772653	intron_variant,genic_upstream_transcript_variant
rs1791772804	intron_variant,genic_upstream_transcript_variant
rs1791772927	intron_variant,genic_upstream_transcript_variant
rs1791773052	intron_variant,genic_upstream_transcript_variant
rs1791773185	intron_variant,genic_upstream_transcript_variant
rs1791773443	intron_variant,genic_upstream_transcript_variant
rs1791773529	intron_variant,genic_upstream_transcript_variant
rs1791773721	intron_variant,genic_upstream_transcript_variant
rs1791773813	intron_variant,genic_upstream_transcript_variant
rs1791773891	intron_variant,genic_upstream_transcript_variant
rs1791774011	intron_variant,genic_upstream_transcript_variant
rs1791774669	intron_variant,genic_upstream_transcript_variant
rs1791775681	intron_variant,genic_upstream_transcript_variant
rs1791775824	intron_variant,genic_upstream_transcript_variant
rs1791776708	intron_variant,genic_upstream_transcript_variant
rs1791776902	intron_variant,genic_upstream_transcript_variant
rs1791777156	intron_variant,genic_upstream_transcript_variant
rs1791777517	intron_variant,genic_upstream_transcript_variant
rs1791777824	intron_variant,genic_upstream_transcript_variant
rs1791778164	intron_variant,genic_upstream_transcript_variant
rs1791778304	intron_variant,genic_upstream_transcript_variant
rs1791779498	intron_variant,genic_upstream_transcript_variant
rs1791779964	intron_variant,genic_upstream_transcript_variant
rs1791780411	intron_variant,genic_upstream_transcript_variant
rs1791780809	intron_variant,genic_upstream_transcript_variant
rs1791780915	intron_variant,genic_upstream_transcript_variant
rs1791780988	intron_variant,genic_upstream_transcript_variant
rs1791781228	intron_variant,genic_upstream_transcript_variant
rs1791781525	intron_variant,genic_upstream_transcript_variant
rs1791781665	intron_variant,genic_upstream_transcript_variant
rs1791781974	intron_variant,genic_upstream_transcript_variant
rs1791782572	intron_variant,genic_upstream_transcript_variant
rs1791783412	intron_variant,genic_upstream_transcript_variant
rs1791783557	intron_variant,genic_upstream_transcript_variant
rs1791784277	intron_variant,genic_upstream_transcript_variant
rs1791784743	intron_variant,genic_upstream_transcript_variant
rs1791785035	intron_variant,genic_upstream_transcript_variant
rs1791785944	intron_variant,genic_upstream_transcript_variant
rs1791786084	intron_variant,genic_upstream_transcript_variant
rs1791786241	intron_variant,genic_upstream_transcript_variant
rs1791787251	intron_variant,genic_upstream_transcript_variant
rs1791787631	intron_variant,genic_upstream_transcript_variant
rs1791787779	intron_variant,genic_upstream_transcript_variant
rs1791788358	intron_variant,genic_upstream_transcript_variant
rs1791788630	intron_variant,genic_upstream_transcript_variant
rs1791788781	intron_variant,genic_upstream_transcript_variant
rs1791788922	intron_variant,genic_upstream_transcript_variant
rs1791789257	intron_variant,genic_upstream_transcript_variant
rs1791790859	intron_variant,genic_upstream_transcript_variant
rs1791792307	intron_variant,genic_upstream_transcript_variant
rs1791792623	intron_variant,genic_upstream_transcript_variant

rs1791792848	intron_variant,genic_upstream_transcript_variant
rs1791792946	intron_variant,genic_upstream_transcript_variant
rs1791793318	intron_variant,genic_upstream_transcript_variant
rs1791793460	intron_variant,genic_upstream_transcript_variant
rs1791793764	intron_variant,genic_upstream_transcript_variant
rs1791794072	intron_variant,genic_upstream_transcript_variant
rs1791794845	intron_variant,genic_upstream_transcript_variant
rs1791795804	intron_variant,genic_upstream_transcript_variant
rs1791796306	intron_variant,genic_upstream_transcript_variant
rs1791796455	intron_variant,genic_upstream_transcript_variant
rs1791796780	intron_variant,genic_upstream_transcript_variant
rs1791797075	intron_variant,genic_upstream_transcript_variant
rs1791797437	intron_variant,genic_upstream_transcript_variant
rs1791797692	intron_variant,genic_upstream_transcript_variant
rs1791797936	intron_variant,genic_upstream_transcript_variant
rs1791798381	intron_variant,genic_upstream_transcript_variant
rs1791799574	intron_variant,genic_upstream_transcript_variant
rs1791800167	intron_variant,genic_upstream_transcript_variant
rs1791801018	intron_variant,genic_upstream_transcript_variant
rs1791803650	intron_variant,genic_upstream_transcript_variant
rs1791804037	intron_variant,genic_upstream_transcript_variant
rs1791804359	intron_variant,genic_upstream_transcript_variant
rs1791804656	intron_variant,genic_upstream_transcript_variant
rs1791805298	intron_variant,genic_upstream_transcript_variant
rs1791805391	intron_variant,genic_upstream_transcript_variant
rs1791805596	intron_variant,genic_upstream_transcript_variant
rs1791805884	intron_variant,genic_upstream_transcript_variant
rs1791806002	intron_variant,genic_upstream_transcript_variant
rs1791806098	intron_variant,genic_upstream_transcript_variant
rs1791806188	intron_variant,genic_upstream_transcript_variant
rs1791806287	intron_variant,genic_upstream_transcript_variant
rs1791806442	intron_variant,genic_upstream_transcript_variant
rs1791806605	intron_variant,genic_upstream_transcript_variant
rs1791806909	intron_variant,genic_upstream_transcript_variant
rs1791807092	intron_variant,genic_upstream_transcript_variant
rs1791807193	intron_variant,genic_upstream_transcript_variant
rs1791807765	intron_variant,genic_upstream_transcript_variant
rs1791808326	intron_variant,genic_upstream_transcript_variant
rs1791809108	intron_variant,genic_upstream_transcript_variant
rs1791809196	intron_variant,genic_upstream_transcript_variant
rs1791809297	intron_variant,genic_upstream_transcript_variant
rs1791810318	intron_variant,genic_upstream_transcript_variant
rs1791810417	intron_variant,genic_upstream_transcript_variant
rs1791810514	intron_variant,genic_upstream_transcript_variant
rs1791810615	intron_variant,genic_upstream_transcript_variant
rs1791810972	intron_variant,genic_upstream_transcript_variant
rs1791811459	intron_variant,genic_upstream_transcript_variant
rs1791811558	intron_variant,genic_upstream_transcript_variant
rs1791811668	intron_variant,genic_upstream_transcript_variant
rs1791811867	intron_variant,genic_upstream_transcript_variant
rs1791812191	intron_variant,genic_upstream_transcript_variant
rs1791812716	intron_variant,genic_upstream_transcript_variant
rs1791814007	intron_variant,genic_upstream_transcript_variant
rs1791814158	intron_variant,genic_upstream_transcript_variant
rs1791815309	intron_variant,genic_upstream_transcript_variant
rs1791815966	intron_variant,genic_upstream_transcript_variant
rs1791816119	intron_variant,genic_upstream_transcript_variant
rs1791817005	intron_variant,genic_upstream_transcript_variant
rs1791817954	intron_variant,genic_upstream_transcript_variant
rs1791818099	intron_variant,genic_upstream_transcript_variant
rs1791818426	intron_variant,genic_upstream_transcript_variant
rs1791818589	intron_variant,genic_upstream_transcript_variant
rs1791819935	intron_variant,genic_upstream_transcript_variant
rs1791820624	intron_variant,genic_upstream_transcript_variant
rs1791820742	intron_variant,genic_upstream_transcript_variant
rs1791820845	intron_variant,genic_upstream_transcript_variant
rs1791821174	intron_variant,genic_upstream_transcript_variant
rs1791821392	intron_variant,genic_upstream_transcript_variant
rs1791821563	intron_variant,genic_upstream_transcript_variant
rs1791821672	intron_variant,genic_upstream_transcript_variant
rs1791821860	intron_variant,genic_upstream_transcript_variant
rs1791822080	intron_variant,genic_upstream_transcript_variant
rs1791822375	intron_variant,genic_upstream_transcript_variant
rs1791822523	intron_variant,genic_upstream_transcript_variant

rs1791822961	intron_variant,genic_upstream_transcript_variant
rs1791823077	intron_variant,genic_upstream_transcript_variant
rs1791823479	intron_variant,genic_upstream_transcript_variant
rs1791824149	intron_variant,genic_upstream_transcript_variant
rs1791824372	intron_variant,genic_upstream_transcript_variant
rs1791825137	intron_variant,genic_upstream_transcript_variant
rs1791825455	intron_variant,genic_upstream_transcript_variant
rs1791825553	intron_variant,genic_upstream_transcript_variant
rs1791825655	intron_variant,genic_upstream_transcript_variant
rs1791825819	intron_variant,genic_upstream_transcript_variant
rs1791826011	intron_variant,genic_upstream_transcript_variant
rs1791826636	intron_variant,genic_upstream_transcript_variant
rs1791827025	intron_variant,genic_upstream_transcript_variant
rs1791827243	intron_variant,genic_upstream_transcript_variant
rs1791827325	intron_variant,genic_upstream_transcript_variant
rs1791827825	intron_variant,genic_upstream_transcript_variant
rs1791828034	intron_variant,genic_upstream_transcript_variant
rs1791828542	intron_variant,genic_upstream_transcript_variant
rs1791828704	intron_variant,genic_upstream_transcript_variant
rs1791829163	intron_variant,genic_upstream_transcript_variant
rs1791829655	intron_variant,genic_upstream_transcript_variant
rs1791829802	intron_variant,genic_upstream_transcript_variant
rs1791830081	intron_variant,genic_upstream_transcript_variant
rs1791830229	intron_variant,genic_upstream_transcript_variant
rs1791830692	intron_variant,genic_upstream_transcript_variant
rs1791830928	intron_variant,genic_upstream_transcript_variant
rs1791831413	intron_variant,genic_upstream_transcript_variant
rs1791831508	intron_variant,genic_upstream_transcript_variant
rs1791831611	intron_variant,genic_upstream_transcript_variant
rs1791832182	intron_variant,genic_upstream_transcript_variant
rs1791832291	intron_variant,genic_upstream_transcript_variant
rs1791832571	intron_variant,genic_upstream_transcript_variant
rs1791833284	intron_variant,genic_upstream_transcript_variant
rs1791834215	intron_variant,genic_upstream_transcript_variant
rs1791834365	intron_variant,genic_upstream_transcript_variant
rs1791834914	intron_variant,genic_upstream_transcript_variant
rs1791835170	intron_variant,genic_upstream_transcript_variant
rs1791835400	intron_variant,genic_upstream_transcript_variant
rs1791835501	intron_variant,genic_upstream_transcript_variant
rs1791835889	intron_variant,genic_upstream_transcript_variant
rs1791836144	intron_variant,genic_upstream_transcript_variant
rs1791836238	intron_variant,genic_upstream_transcript_variant
rs1791836330	intron_variant,genic_upstream_transcript_variant
rs1791836870	intron_variant,genic_upstream_transcript_variant
rs1791837488	intron_variant,genic_upstream_transcript_variant
rs1791838077	intron_variant,genic_upstream_transcript_variant
rs1791838177	intron_variant,genic_upstream_transcript_variant
rs1791838287	intron_variant,genic_upstream_transcript_variant
rs1791838387	intron_variant,genic_upstream_transcript_variant
rs1791838713	intron_variant,genic_upstream_transcript_variant
rs1791838942	intron_variant,genic_upstream_transcript_variant
rs1791839167	intron_variant,genic_upstream_transcript_variant
rs1791839389	intron_variant,genic_upstream_transcript_variant
rs1791839575	intron_variant,genic_upstream_transcript_variant
rs1791842885	intron_variant,genic_upstream_transcript_variant
rs1791843094	intron_variant,genic_upstream_transcript_variant
rs1791843727	intron_variant,genic_upstream_transcript_variant
rs1791843975	intron_variant,genic_upstream_transcript_variant
rs1791844085	intron_variant,genic_upstream_transcript_variant
rs1791844374	intron_variant,genic_upstream_transcript_variant
rs1791844849	intron_variant,genic_upstream_transcript_variant
rs1791844972	intron_variant,genic_upstream_transcript_variant
rs1791845059	intron_variant,genic_upstream_transcript_variant
rs1791845228	intron_variant,genic_upstream_transcript_variant
rs1791845455	intron_variant,genic_upstream_transcript_variant
rs1791845827	intron_variant,genic_upstream_transcript_variant
rs1791846685	intron_variant,genic_upstream_transcript_variant
rs1791846877	intron_variant,genic_upstream_transcript_variant
rs1791846982	intron_variant,genic_upstream_transcript_variant
rs1791847092	intron_variant,genic_upstream_transcript_variant
rs1791847310	intron_variant,genic_upstream_transcript_variant
rs1791847518	intron_variant,genic_upstream_transcript_variant
rs1791847796	intron_variant,genic_upstream_transcript_variant
rs1791848081	intron_variant,genic_upstream_transcript_variant

rs1791848889	intron_variant,genic_upstream_transcript_variant
rs1791849222	intron_variant,genic_upstream_transcript_variant
rs1791849314	intron_variant,genic_upstream_transcript_variant
rs1791849416	intron_variant,genic_upstream_transcript_variant
rs1791849521	intron_variant,genic_upstream_transcript_variant
rs1791849610	intron_variant,genic_upstream_transcript_variant
rs1791849717	intron_variant,genic_upstream_transcript_variant
rs1791850957	intron_variant,genic_upstream_transcript_variant
rs1791851157	intron_variant,genic_upstream_transcript_variant
rs1791851249	intron_variant,genic_upstream_transcript_variant
rs1791851437	intron_variant,genic_upstream_transcript_variant
rs1791851795	intron_variant,genic_upstream_transcript_variant
rs1791851891	intron_variant,genic_upstream_transcript_variant
rs1791851998	intron_variant,genic_upstream_transcript_variant
rs1791852092	intron_variant,genic_upstream_transcript_variant
rs1791852434	intron_variant,genic_upstream_transcript_variant
rs1791852836	intron_variant,genic_upstream_transcript_variant
rs1791852942	intron_variant,genic_upstream_transcript_variant
rs1791853053	intron_variant,genic_upstream_transcript_variant
rs1791853458	intron_variant,genic_upstream_transcript_variant
rs1791853548	intron_variant,genic_upstream_transcript_variant
rs1791853655	intron_variant,genic_upstream_transcript_variant
rs1791853749	intron_variant,genic_upstream_transcript_variant
rs1791853853	intron_variant,genic_upstream_transcript_variant
rs1791853950	intron_variant,genic_upstream_transcript_variant
rs1791854149	intron_variant,genic_upstream_transcript_variant
rs1791854501	intron_variant,genic_upstream_transcript_variant
rs1791854742	intron_variant,genic_upstream_transcript_variant
rs1791854862	intron_variant,genic_upstream_transcript_variant
rs1791855698	intron_variant,genic_upstream_transcript_variant
rs1791855882	intron_variant,genic_upstream_transcript_variant
rs1791855998	intron_variant,genic_upstream_transcript_variant
rs1791856486	intron_variant,genic_upstream_transcript_variant
rs1791857112	intron_variant,genic_upstream_transcript_variant
rs1791857414	intron_variant,genic_upstream_transcript_variant
rs1791857505	intron_variant,genic_upstream_transcript_variant
rs1791857914	intron_variant,genic_upstream_transcript_variant
rs1791858369	intron_variant,genic_upstream_transcript_variant
rs1791858968	intron_variant,genic_upstream_transcript_variant
rs1791859129	intron_variant,genic_upstream_transcript_variant
rs1791859303	intron_variant,genic_upstream_transcript_variant
rs1791859383	intron_variant,genic_upstream_transcript_variant
rs1791859478	intron_variant,genic_upstream_transcript_variant
rs1791859563	intron_variant,genic_upstream_transcript_variant
rs1791859666	intron_variant,genic_upstream_transcript_variant
rs1791859763	intron_variant,genic_upstream_transcript_variant
rs1791859866	intron_variant,genic_upstream_transcript_variant
rs1791860566	intron_variant,genic_upstream_transcript_variant
rs1791860857	intron_variant,genic_upstream_transcript_variant
rs1791861184	intron_variant,genic_upstream_transcript_variant
rs1791861283	intron_variant,genic_upstream_transcript_variant
rs1791861621	intron_variant,genic_upstream_transcript_variant
rs1791861936	intron_variant,genic_upstream_transcript_variant
rs1791862583	intron_variant,genic_upstream_transcript_variant
rs1791864047	intron_variant,genic_upstream_transcript_variant
rs1791864142	intron_variant,genic_upstream_transcript_variant
rs1791864247	intron_variant,genic_upstream_transcript_variant
rs1791864466	intron_variant,genic_upstream_transcript_variant
rs1791864891	intron_variant,genic_upstream_transcript_variant
rs1791865001	intron_variant,genic_upstream_transcript_variant
rs1791865210	intron_variant,genic_upstream_transcript_variant
rs1791865315	intron_variant,genic_upstream_transcript_variant
rs1791865414	intron_variant,genic_upstream_transcript_variant
rs1791866099	intron_variant,genic_upstream_transcript_variant
rs1791866602	intron_variant,genic_upstream_transcript_variant
rs1791866802	intron_variant,genic_upstream_transcript_variant
rs1791866900	intron_variant,genic_upstream_transcript_variant
rs1791867327	intron_variant,genic_upstream_transcript_variant
rs1791867428	intron_variant,genic_upstream_transcript_variant
rs1791867520	intron_variant,genic_upstream_transcript_variant
rs1791867765	intron_variant,genic_upstream_transcript_variant
rs1791868047	intron_variant,genic_upstream_transcript_variant
rs1791868584	intron_variant,genic_upstream_transcript_variant
rs1791868917	intron_variant,genic_upstream_transcript_variant

rs1791869140	intron_variant,genic_upstream_transcript_variant
rs1791869438	intron_variant,genic_upstream_transcript_variant
rs1791869603	intron_variant,genic_upstream_transcript_variant
rs1791869684	intron_variant,genic_upstream_transcript_variant
rs1791869764	intron_variant,genic_upstream_transcript_variant
rs1791869869	intron_variant,genic_upstream_transcript_variant
rs1791870236	intron_variant,genic_upstream_transcript_variant
rs1791870345	intron_variant,genic_upstream_transcript_variant
rs1791870498	intron_variant,genic_upstream_transcript_variant
rs1791871429	intron_variant,genic_upstream_transcript_variant
rs1791872054	intron_variant,genic_upstream_transcript_variant
rs1791872732	intron_variant,genic_upstream_transcript_variant
rs1791873239	intron_variant,genic_upstream_transcript_variant
rs1791873439	intron_variant,genic_upstream_transcript_variant
rs1791873568	intron_variant,genic_upstream_transcript_variant
rs1791874381	intron_variant,genic_upstream_transcript_variant
rs1791874576	intron_variant,genic_upstream_transcript_variant
rs1791874773	intron_variant,genic_upstream_transcript_variant
rs1791874961	intron_variant,genic_upstream_transcript_variant
rs1791875058	intron_variant,genic_upstream_transcript_variant
rs1791875446	intron_variant,genic_upstream_transcript_variant
rs1791875776	intron_variant,genic_upstream_transcript_variant
rs1791875979	intron_variant,genic_upstream_transcript_variant
rs1791876583	intron_variant,genic_upstream_transcript_variant
rs1791877025	intron_variant,genic_upstream_transcript_variant
rs1791877136	intron_variant,genic_upstream_transcript_variant
rs1791877288	intron_variant,genic_upstream_transcript_variant
rs1791877699	intron_variant,genic_upstream_transcript_variant
rs1791877892	intron_variant,genic_upstream_transcript_variant
rs1791877970	intron_variant,genic_upstream_transcript_variant
rs1791878535	intron_variant,genic_upstream_transcript_variant
rs1791878707	intron_variant,genic_upstream_transcript_variant
rs1791878904	intron_variant,genic_upstream_transcript_variant
rs1791879197	intron_variant,genic_upstream_transcript_variant
rs1791879521	intron_variant,genic_upstream_transcript_variant
rs1791880007	intron_variant,genic_upstream_transcript_variant
rs1791880266	intron_variant,genic_upstream_transcript_variant
rs1791880354	intron_variant,genic_upstream_transcript_variant
rs1791880780	intron_variant,genic_upstream_transcript_variant
rs1791880880	intron_variant,genic_upstream_transcript_variant
rs1791881065	intron_variant,genic_upstream_transcript_variant
rs1791881896	intron_variant,genic_upstream_transcript_variant
rs1791882487	intron_variant,genic_upstream_transcript_variant
rs1791882785	intron_variant,genic_upstream_transcript_variant
rs1791883377	intron_variant,genic_upstream_transcript_variant
rs1791883802	intron_variant,genic_upstream_transcript_variant
rs1791884032	intron_variant,genic_upstream_transcript_variant
rs1791884119	intron_variant,genic_upstream_transcript_variant
rs1791884333	intron_variant,genic_upstream_transcript_variant
rs1791884455	intron_variant,genic_upstream_transcript_variant
rs1791884555	intron_variant,genic_upstream_transcript_variant
rs1791884740	intron_variant,genic_upstream_transcript_variant
rs1791884827	intron_variant,genic_upstream_transcript_variant
rs1791885420	intron_variant,genic_upstream_transcript_variant
rs1791885536	intron_variant,genic_upstream_transcript_variant
rs1791885640	intron_variant,genic_upstream_transcript_variant
rs1791885869	intron_variant,genic_upstream_transcript_variant
rs1791885962	intron_variant,genic_upstream_transcript_variant
rs1791886394	intron_variant,genic_upstream_transcript_variant
rs1791886713	intron_variant,genic_upstream_transcript_variant
rs1791887037	intron_variant,genic_upstream_transcript_variant
rs1791887366	intron_variant,genic_upstream_transcript_variant
rs1791887541	intron_variant,genic_upstream_transcript_variant
rs1791887999	intron_variant,genic_upstream_transcript_variant
rs1791888157	intron_variant,genic_upstream_transcript_variant
rs1791888986	intron_variant,genic_upstream_transcript_variant
rs1791889115	intron_variant,genic_upstream_transcript_variant
rs1791890420	intron_variant,genic_upstream_transcript_variant
rs1791890526	intron_variant,genic_upstream_transcript_variant
rs1791891113	intron_variant,genic_upstream_transcript_variant
rs1791891424	intron_variant,genic_upstream_transcript_variant
rs1791891524	intron_variant,genic_upstream_transcript_variant
rs1791891719	intron_variant,genic_upstream_transcript_variant
rs1791892102	intron_variant,genic_upstream_transcript_variant

rs1791892535	intron_variant,genic_upstream_transcript_variant
rs1791892664	intron_variant,genic_upstream_transcript_variant
rs1791893173	intron_variant,genic_upstream_transcript_variant
rs1791893592	intron_variant,genic_upstream_transcript_variant
rs1791893878	intron_variant,genic_upstream_transcript_variant
rs1791893980	intron_variant,genic_upstream_transcript_variant
rs1791894191	intron_variant,genic_upstream_transcript_variant
rs1791894634	intron_variant,genic_upstream_transcript_variant
rs1791895203	intron_variant,genic_upstream_transcript_variant
rs1791895293	intron_variant,genic_upstream_transcript_variant
rs1791896026	intron_variant,genic_upstream_transcript_variant
rs1791896219	intron_variant,genic_upstream_transcript_variant
rs1791896762	intron_variant,genic_upstream_transcript_variant
rs1791896892	intron_variant,genic_upstream_transcript_variant
rs1791896991	intron_variant,genic_upstream_transcript_variant
rs1791897083	intron_variant,genic_upstream_transcript_variant
rs1791897176	intron_variant,genic_upstream_transcript_variant
rs1791897263	intron_variant,genic_upstream_transcript_variant
rs1791897572	intron_variant,genic_upstream_transcript_variant
rs1791897669	intron_variant,genic_upstream_transcript_variant
rs1791897757	intron_variant,genic_upstream_transcript_variant
rs1791897956	intron_variant,genic_upstream_transcript_variant
rs1791898482	intron_variant,genic_upstream_transcript_variant
rs1791898840	intron_variant,genic_upstream_transcript_variant
rs1791899305	intron_variant,genic_upstream_transcript_variant
rs1791899429	intron_variant,genic_upstream_transcript_variant
rs1791899524	intron_variant,genic_upstream_transcript_variant
rs1791899730	intron_variant,genic_upstream_transcript_variant
rs1791900145	intron_variant,genic_upstream_transcript_variant
rs1791900493	intron_variant,genic_upstream_transcript_variant
rs1791900791	intron_variant,genic_upstream_transcript_variant
rs1791900888	intron_variant,genic_upstream_transcript_variant
rs1791901108	intron_variant,genic_upstream_transcript_variant
rs1791901408	intron_variant,genic_upstream_transcript_variant
rs1791901613	intron_variant,genic_upstream_transcript_variant
rs1791901719	intron_variant,genic_upstream_transcript_variant
rs1791902197	intron_variant,genic_upstream_transcript_variant
rs1791902719	intron_variant,genic_upstream_transcript_variant
rs1791903137	intron_variant,genic_upstream_transcript_variant
rs1791903229	intron_variant,genic_upstream_transcript_variant
rs1791903799	intron_variant,genic_upstream_transcript_variant
rs1791903976	intron_variant,genic_upstream_transcript_variant
rs1791904082	intron_variant,genic_upstream_transcript_variant
rs1791904367	intron_variant,genic_upstream_transcript_variant
rs1791904561	intron_variant,genic_upstream_transcript_variant
rs1791904742	intron_variant,genic_upstream_transcript_variant
rs1791905026	intron_variant,genic_upstream_transcript_variant
rs1791905133	intron_variant,genic_upstream_transcript_variant
rs1791905361	intron_variant,genic_upstream_transcript_variant
rs1791905467	intron_variant,genic_upstream_transcript_variant
rs1791905819	intron_variant,genic_upstream_transcript_variant
rs1791905923	intron_variant,genic_upstream_transcript_variant
rs1791906915	intron_variant,genic_upstream_transcript_variant
rs1791907245	intron_variant,genic_upstream_transcript_variant
rs1791907409	intron_variant,genic_upstream_transcript_variant
rs1791907754	intron_variant,genic_upstream_transcript_variant
rs1791907909	intron_variant,genic_upstream_transcript_variant
rs1791908875	intron_variant,genic_upstream_transcript_variant
rs1791909058	intron_variant,genic_upstream_transcript_variant
rs1791909519	intron_variant,genic_upstream_transcript_variant
rs1791909821	intron_variant,genic_upstream_transcript_variant
rs1791910804	intron_variant,genic_upstream_transcript_variant
rs1791911099	intron_variant,genic_upstream_transcript_variant
rs1791911373	intron_variant,genic_upstream_transcript_variant
rs1791911833	intron_variant,genic_upstream_transcript_variant
rs1791912018	intron_variant,genic_upstream_transcript_variant
rs1791912456	intron_variant,genic_upstream_transcript_variant
rs1791913074	intron_variant,genic_upstream_transcript_variant
rs1791913216	intron_variant,genic_upstream_transcript_variant
rs1791913728	intron_variant,genic_upstream_transcript_variant
rs1791914387	intron_variant,genic_upstream_transcript_variant
rs1791914710	intron_variant,genic_upstream_transcript_variant
rs1791914837	intron_variant,genic_upstream_transcript_variant
rs1791915337	intron_variant,genic_upstream_transcript_variant

rs1791915792	intron_variant,genic_upstream_transcript_variant
rs1791915932	intron_variant,genic_upstream_transcript_variant
rs1791916393	intron_variant,genic_upstream_transcript_variant
rs1791916490	intron_variant,genic_upstream_transcript_variant
rs1791916579	intron_variant,genic_upstream_transcript_variant
rs1791917035	intron_variant,genic_upstream_transcript_variant
rs1791918152	intron_variant,genic_upstream_transcript_variant
rs1791918286	intron_variant,genic_upstream_transcript_variant
rs1791920051	intron_variant,genic_upstream_transcript_variant
rs1791921180	intron_variant,genic_upstream_transcript_variant
rs1791921522	intron_variant,genic_upstream_transcript_variant
rs1791921945	intron_variant,genic_upstream_transcript_variant
rs1791922174	intron_variant,genic_upstream_transcript_variant
rs1791922829	intron_variant,genic_upstream_transcript_variant
rs1791922972	intron_variant,genic_upstream_transcript_variant
rs1791923275	intron_variant,genic_upstream_transcript_variant
rs1791923387	intron_variant,genic_upstream_transcript_variant
rs1791923567	intron_variant,genic_upstream_transcript_variant
rs1791924311	intron_variant,genic_upstream_transcript_variant
rs1791924392	intron_variant,genic_upstream_transcript_variant
rs1791924602	intron_variant,genic_upstream_transcript_variant
rs1791925134	intron_variant,genic_upstream_transcript_variant
rs1791925412	intron_variant,genic_upstream_transcript_variant
rs1791925523	intron_variant,genic_upstream_transcript_variant
rs1791925860	intron_variant,genic_upstream_transcript_variant
rs1791926343	intron_variant,genic_upstream_transcript_variant
rs1791926434	intron_variant,genic_upstream_transcript_variant
rs1791926516	intron_variant,genic_upstream_transcript_variant
rs1791926697	intron_variant,genic_upstream_transcript_variant
rs1791927097	intron_variant,genic_upstream_transcript_variant
rs1791927911	intron_variant,genic_upstream_transcript_variant
rs1791928546	intron_variant,genic_upstream_transcript_variant
rs1791928723	intron_variant,genic_upstream_transcript_variant
rs1791929030	intron_variant,genic_upstream_transcript_variant
rs1791929212	intron_variant,genic_upstream_transcript_variant
rs1791929317	intron_variant,genic_upstream_transcript_variant
rs1791929392	intron_variant,genic_upstream_transcript_variant
rs1791930153	intron_variant,genic_upstream_transcript_variant
rs1791930359	intron_variant,genic_upstream_transcript_variant
rs1791930442	intron_variant,genic_upstream_transcript_variant
rs1791930978	intron_variant,genic_upstream_transcript_variant
rs1791931066	intron_variant,genic_upstream_transcript_variant
rs1791931156	intron_variant,genic_upstream_transcript_variant
rs1791931616	intron_variant,genic_upstream_transcript_variant
rs1791932031	intron_variant,genic_upstream_transcript_variant
rs1791932164	intron_variant,genic_upstream_transcript_variant
rs1791932317	intron_variant,genic_upstream_transcript_variant
rs1791933015	intron_variant,genic_upstream_transcript_variant
rs1791933811	intron_variant,genic_upstream_transcript_variant
rs1791934444	intron_variant,genic_upstream_transcript_variant
rs1791935099	intron_variant,genic_upstream_transcript_variant
rs1791935282	intron_variant,genic_upstream_transcript_variant
rs1791935903	intron_variant,genic_upstream_transcript_variant
rs1791936290	intron_variant,genic_upstream_transcript_variant
rs1791936531	intron_variant,genic_upstream_transcript_variant
rs1791936880	intron_variant,genic_upstream_transcript_variant
rs1791936984	intron_variant,genic_upstream_transcript_variant
rs1791937102	intron_variant,genic_upstream_transcript_variant
rs1791937201	intron_variant,genic_upstream_transcript_variant
rs1791937664	intron_variant,genic_upstream_transcript_variant
rs1791937763	intron_variant,genic_upstream_transcript_variant
rs1791937870	intron_variant,genic_upstream_transcript_variant
rs1791938253	intron_variant,genic_upstream_transcript_variant
rs1791939676	intron_variant,genic_upstream_transcript_variant
rs1791940741	intron_variant,genic_upstream_transcript_variant
rs1791941350	intron_variant,genic_upstream_transcript_variant
rs1791942228	intron_variant,genic_upstream_transcript_variant
rs1791942388	intron_variant,genic_upstream_transcript_variant
rs1791942923	intron_variant,genic_upstream_transcript_variant
rs1791943425	intron_variant,genic_upstream_transcript_variant
rs1791944016	intron_variant,genic_upstream_transcript_variant
rs1791944330	intron_variant,genic_upstream_transcript_variant
rs1791944576	intron_variant,genic_upstream_transcript_variant
rs1791944905	intron_variant,genic_upstream_transcript_variant

rs1791945427	intron_variant,genic_upstream_transcript_variant
rs1791945836	intron_variant,genic_upstream_transcript_variant
rs1791946356	intron_variant,genic_upstream_transcript_variant
rs1791946508	intron_variant,genic_upstream_transcript_variant
rs1791947224	intron_variant,genic_upstream_transcript_variant
rs1791947794	intron_variant,genic_upstream_transcript_variant
rs1791947990	intron_variant,genic_upstream_transcript_variant
rs1791948316	intron_variant,genic_upstream_transcript_variant
rs1791948789	intron_variant,genic_upstream_transcript_variant
rs1791950309	intron_variant,genic_upstream_transcript_variant
rs1791950450	intron_variant,genic_upstream_transcript_variant
rs1791951203	intron_variant,genic_upstream_transcript_variant
rs1791951831	intron_variant,genic_upstream_transcript_variant
rs1791952309	intron_variant,genic_upstream_transcript_variant
rs1791953291	intron_variant,genic_upstream_transcript_variant
rs1791953452	intron_variant,genic_upstream_transcript_variant
rs1791954209	intron_variant,genic_upstream_transcript_variant
rs1791954367	intron_variant,genic_upstream_transcript_variant
rs1791954542	intron_variant,genic_upstream_transcript_variant
rs1791954707	intron_variant,genic_upstream_transcript_variant
rs1791955539	intron_variant,genic_upstream_transcript_variant
rs1791955846	intron_variant,genic_upstream_transcript_variant
rs1791957400	intron_variant,genic_upstream_transcript_variant
rs1791957721	intron_variant,genic_upstream_transcript_variant
rs1791958024	intron_variant,genic_upstream_transcript_variant
rs1791958182	intron_variant,genic_upstream_transcript_variant
rs1791958356	intron_variant,genic_upstream_transcript_variant
rs1791958513	intron_variant,genic_upstream_transcript_variant
rs1791958661	intron_variant,genic_upstream_transcript_variant
rs1791959187	intron_variant,genic_upstream_transcript_variant
rs1791959323	intron_variant,genic_upstream_transcript_variant
rs1791959610	intron_variant,genic_upstream_transcript_variant
rs1791959928	intron_variant,genic_upstream_transcript_variant
rs1791961675	intron_variant,genic_upstream_transcript_variant
rs1791961829	intron_variant,genic_upstream_transcript_variant
rs1791962707	intron_variant,genic_upstream_transcript_variant
rs1791962862	intron_variant,genic_upstream_transcript_variant
rs1791963158	intron_variant,genic_upstream_transcript_variant
rs1791963671	intron_variant,genic_upstream_transcript_variant
rs1791964153	intron_variant,genic_upstream_transcript_variant
rs1791964743	intron_variant,genic_upstream_transcript_variant
rs1791965050	intron_variant,genic_upstream_transcript_variant
rs1791966992	intron_variant,genic_upstream_transcript_variant
rs1791967139	intron_variant,genic_upstream_transcript_variant
rs1791967248	intron_variant,genic_upstream_transcript_variant
rs1791967531	intron_variant,genic_upstream_transcript_variant
rs1791968213	intron_variant,genic_upstream_transcript_variant
rs1791968383	intron_variant,genic_upstream_transcript_variant
rs1791968823	intron_variant,genic_upstream_transcript_variant
rs1791969198	intron_variant,genic_upstream_transcript_variant
rs1791969763	intron_variant,genic_upstream_transcript_variant
rs1791969843	intron_variant,genic_upstream_transcript_variant
rs1791970238	intron_variant,genic_upstream_transcript_variant
rs1791970421	intron_variant,genic_upstream_transcript_variant
rs1791970795	intron_variant,genic_upstream_transcript_variant
rs1791971199	intron_variant,genic_upstream_transcript_variant
rs1791971488	intron_variant,genic_upstream_transcript_variant
rs1791971587	intron_variant,genic_upstream_transcript_variant
rs1791971874	intron_variant,genic_upstream_transcript_variant
rs1791971964	intron_variant,genic_upstream_transcript_variant
rs1791972260	intron_variant,genic_upstream_transcript_variant
rs1791972436	intron_variant,genic_upstream_transcript_variant
rs1791972744	intron_variant,genic_upstream_transcript_variant
rs1791972837	intron_variant,genic_upstream_transcript_variant
rs1791973042	intron_variant,genic_upstream_transcript_variant
rs1791973337	intron_variant,genic_upstream_transcript_variant
rs1791973431	intron_variant,genic_upstream_transcript_variant
rs1791973886	intron_variant,genic_upstream_transcript_variant
rs1791974112	intron_variant,genic_upstream_transcript_variant
rs1791974394	intron_variant,genic_upstream_transcript_variant
rs1791974850	intron_variant,genic_upstream_transcript_variant
rs1791978362	intron_variant,genic_upstream_transcript_variant
rs1791978689	intron_variant,genic_upstream_transcript_variant
rs1791978882	intron_variant,genic_upstream_transcript_variant

rs1791979990	intron_variant,genic_upstream_transcript_variant
rs1791980736	intron_variant,genic_upstream_transcript_variant
rs1791980898	intron_variant,genic_upstream_transcript_variant
rs1791981059	intron_variant,genic_upstream_transcript_variant
rs1791981219	intron_variant,genic_upstream_transcript_variant
rs1791982223	intron_variant,genic_upstream_transcript_variant
rs1791983028	intron_variant,genic_upstream_transcript_variant
rs1791983450	intron_variant,genic_upstream_transcript_variant
rs1791983547	intron_variant,genic_upstream_transcript_variant
rs1791983840	intron_variant,genic_upstream_transcript_variant
rs1791984037	intron_variant,genic_upstream_transcript_variant
rs1791984753	intron_variant,genic_upstream_transcript_variant
rs1791984852	intron_variant,genic_upstream_transcript_variant
rs1791985370	intron_variant,genic_upstream_transcript_variant
rs1791985460	intron_variant,genic_upstream_transcript_variant
rs1791985561	intron_variant,genic_upstream_transcript_variant
rs1791985653	intron_variant,genic_upstream_transcript_variant
rs1791985746	intron_variant,genic_upstream_transcript_variant
rs1791985889	intron_variant,genic_upstream_transcript_variant
rs1791987577	intron_variant,genic_upstream_transcript_variant
rs1791988071	intron_variant,genic_upstream_transcript_variant
rs1791988158	intron_variant,genic_upstream_transcript_variant
rs1791988277	intron_variant,genic_upstream_transcript_variant
rs1791988431	intron_variant,genic_upstream_transcript_variant
rs1791988964	intron_variant,genic_upstream_transcript_variant
rs1791989070	intron_variant,genic_upstream_transcript_variant
rs1791989181	intron_variant,genic_upstream_transcript_variant
rs1791989853	intron_variant,genic_upstream_transcript_variant
rs1791990064	intron_variant,genic_upstream_transcript_variant
rs1791990441	intron_variant,genic_upstream_transcript_variant
rs1791990741	intron_variant,genic_upstream_transcript_variant
rs1791991161	intron_variant,genic_upstream_transcript_variant
rs1791991456	intron_variant,genic_upstream_transcript_variant
rs1791991706	intron_variant,genic_upstream_transcript_variant
rs1791991803	intron_variant,genic_upstream_transcript_variant
rs1791992026	intron_variant,genic_upstream_transcript_variant
rs1791992134	intron_variant,genic_upstream_transcript_variant
rs1791992304	intron_variant,genic_upstream_transcript_variant
rs1791992389	intron_variant,genic_upstream_transcript_variant
rs1791996653	intron_variant,genic_upstream_transcript_variant
rs1791996952	intron_variant,genic_upstream_transcript_variant
rs1791997100	intron_variant,genic_upstream_transcript_variant
rs1791997538	intron_variant,genic_upstream_transcript_variant
rs1791997844	intron_variant,genic_upstream_transcript_variant
rs1791998186	intron_variant,genic_upstream_transcript_variant
rs1791998363	intron_variant,genic_upstream_transcript_variant
rs1791998520	intron_variant,genic_upstream_transcript_variant
rs1791998674	intron_variant,genic_upstream_transcript_variant
rs1791999333	intron_variant,genic_upstream_transcript_variant
rs1791999489	intron_variant,genic_upstream_transcript_variant
rs1792000547	intron_variant,genic_upstream_transcript_variant
rs1792000703	intron_variant,genic_upstream_transcript_variant
rs1792001075	intron_variant,genic_upstream_transcript_variant
rs1792001379	intron_variant,genic_upstream_transcript_variant
rs1792003116	intron_variant,genic_upstream_transcript_variant
rs1792003288	intron_variant,genic_upstream_transcript_variant
rs1792003924	intron_variant,genic_upstream_transcript_variant
rs1792004062	intron_variant,genic_upstream_transcript_variant
rs1792004219	intron_variant,genic_upstream_transcript_variant
rs1792004878	intron_variant,genic_upstream_transcript_variant
rs1792007125	intron_variant,genic_upstream_transcript_variant
rs1792007497	intron_variant,genic_upstream_transcript_variant
rs1792007755	intron_variant,genic_upstream_transcript_variant
rs1792007863	intron_variant,genic_upstream_transcript_variant
rs1792008552	intron_variant,genic_upstream_transcript_variant
rs1792008878	intron_variant,genic_upstream_transcript_variant
rs1792009236	intron_variant,genic_upstream_transcript_variant
rs1792009575	intron_variant,genic_upstream_transcript_variant
rs1792009882	intron_variant,genic_upstream_transcript_variant
rs1792010194	intron_variant,genic_upstream_transcript_variant
rs1792010506	intron_variant,genic_upstream_transcript_variant
rs1792010843	intron_variant,genic_upstream_transcript_variant
rs1792010985	intron_variant,genic_upstream_transcript_variant
rs1792011274	intron_variant,genic_upstream_transcript_variant

rs1792011390	intron_variant,genic_upstream_transcript_variant
rs1792011577	intron_variant,genic_upstream_transcript_variant
rs1792011701	intron_variant,genic_upstream_transcript_variant
rs1792012678	intron_variant,genic_upstream_transcript_variant
rs1792012919	intron_variant,genic_upstream_transcript_variant
rs1792013138	intron_variant,genic_upstream_transcript_variant
rs1792013911	intron_variant,genic_upstream_transcript_variant
rs1792014424	intron_variant,genic_upstream_transcript_variant
rs1792014610	intron_variant,genic_upstream_transcript_variant
rs1792014955	intron_variant,genic_upstream_transcript_variant
rs1792015150	intron_variant,genic_upstream_transcript_variant
rs1792016176	intron_variant,genic_upstream_transcript_variant
rs1792016808	intron_variant,genic_upstream_transcript_variant
rs1792017060	intron_variant,genic_upstream_transcript_variant
rs1792017319	intron_variant,genic_upstream_transcript_variant
rs1792017736	intron_variant,genic_upstream_transcript_variant
rs1792017856	intron_variant,genic_upstream_transcript_variant
rs1792018188	intron_variant,genic_upstream_transcript_variant
rs1792018802	intron_variant,genic_upstream_transcript_variant
rs1792019950	intron_variant,genic_upstream_transcript_variant
rs1792020581	intron_variant,genic_upstream_transcript_variant
rs1792021718	intron_variant,genic_upstream_transcript_variant
rs1792021882	intron_variant,genic_upstream_transcript_variant
rs1792022336	intron_variant,genic_upstream_transcript_variant
rs1792022630	intron_variant,genic_upstream_transcript_variant
rs1792022721	intron_variant,genic_upstream_transcript_variant
rs1792023020	intron_variant,genic_upstream_transcript_variant
rs1792023229	intron_variant,genic_upstream_transcript_variant
rs1792023335	intron_variant,genic_upstream_transcript_variant
rs1792023999	intron_variant,genic_upstream_transcript_variant
rs1792024190	intron_variant,genic_upstream_transcript_variant
rs1792024728	intron_variant,genic_upstream_transcript_variant
rs1792025225	intron_variant,genic_upstream_transcript_variant
rs1792025302	intron_variant,genic_upstream_transcript_variant
rs1792025522	intron_variant,genic_upstream_transcript_variant
rs1792025714	intron_variant,genic_upstream_transcript_variant
rs1792026265	intron_variant,genic_upstream_transcript_variant
rs1792026374	intron_variant,genic_upstream_transcript_variant
rs1792026449	intron_variant,genic_upstream_transcript_variant
rs1792026543	intron_variant,genic_upstream_transcript_variant
rs1792026629	intron_variant,genic_upstream_transcript_variant
rs1792026723	intron_variant,genic_upstream_transcript_variant
rs1792026826	intron_variant,genic_upstream_transcript_variant
rs1792026992	intron_variant,genic_upstream_transcript_variant
rs1792027185	intron_variant,genic_upstream_transcript_variant
rs1792027416	intron_variant,genic_upstream_transcript_variant
rs1792027909	intron_variant,genic_upstream_transcript_variant
rs1792028002	intron_variant,genic_upstream_transcript_variant
rs1792028111	intron_variant,genic_upstream_transcript_variant
rs1792028441	intron_variant,genic_upstream_transcript_variant
rs1792028626	intron_variant,genic_upstream_transcript_variant
rs1792028894	intron_variant,genic_upstream_transcript_variant
rs1792028995	intron_variant,genic_upstream_transcript_variant
rs1792029324	intron_variant,genic_upstream_transcript_variant
rs1792030050	intron_variant,genic_upstream_transcript_variant
rs1792030261	intron_variant,genic_upstream_transcript_variant
rs1792030463	intron_variant,genic_upstream_transcript_variant
rs1792030548	intron_variant,genic_upstream_transcript_variant
rs1792030727	intron_variant,genic_upstream_transcript_variant
rs1792030922	intron_variant,genic_upstream_transcript_variant
rs1792031067	intron_variant,genic_upstream_transcript_variant
rs1792031273	intron_variant,genic_upstream_transcript_variant
rs1792031381	intron_variant,genic_upstream_transcript_variant
rs1792031488	intron_variant,genic_upstream_transcript_variant
rs1792032259	intron_variant,genic_upstream_transcript_variant
rs1792032478	intron_variant,genic_upstream_transcript_variant
rs1792032886	intron_variant,genic_upstream_transcript_variant
rs1792033745	intron_variant,genic_upstream_transcript_variant
rs1792034079	intron_variant,genic_upstream_transcript_variant
rs1792035274	intron_variant,genic_upstream_transcript_variant
rs1792035562	intron_variant,genic_upstream_transcript_variant
rs1792035884	intron_variant,genic_upstream_transcript_variant
rs1792036143	intron_variant,genic_upstream_transcript_variant
rs1792036229	intron_variant,genic_upstream_transcript_variant

rs1792036510	intron_variant,genic_upstream_transcript_variant
rs1792037010	intron_variant,genic_upstream_transcript_variant
rs1792037330	intron_variant,genic_upstream_transcript_variant
rs1792037998	intron_variant,genic_upstream_transcript_variant
rs1792038149	intron_variant,genic_upstream_transcript_variant
rs1792038555	intron_variant,genic_upstream_transcript_variant
rs1792039076	intron_variant,genic_upstream_transcript_variant
rs1792039375	intron_variant,genic_upstream_transcript_variant
rs1792039472	intron_variant,genic_upstream_transcript_variant
rs1792039564	intron_variant,genic_upstream_transcript_variant
rs1792039657	intron_variant,genic_upstream_transcript_variant
rs1792039738	intron_variant,genic_upstream_transcript_variant
rs1792039821	intron_variant,genic_upstream_transcript_variant
rs1792040211	intron_variant,genic_upstream_transcript_variant
rs1792040491	intron_variant,genic_upstream_transcript_variant
rs1792040583	intron_variant,genic_upstream_transcript_variant
rs1792040780	intron_variant,genic_upstream_transcript_variant
rs1792040882	intron_variant,genic_upstream_transcript_variant
rs1792041148	intron_variant,genic_upstream_transcript_variant
rs1792041239	intron_variant,genic_upstream_transcript_variant
rs1792041320	intron_variant,genic_upstream_transcript_variant
rs1792041395	intron_variant,genic_upstream_transcript_variant
rs1792041724	intron_variant,genic_upstream_transcript_variant
rs1792042388	intron_variant,genic_upstream_transcript_variant
rs1792042480	intron_variant,genic_upstream_transcript_variant
rs1792042674	intron_variant,genic_upstream_transcript_variant
rs1792042881	intron_variant,genic_upstream_transcript_variant
rs1792043054	intron_variant,genic_upstream_transcript_variant
rs1792043342	intron_variant,genic_upstream_transcript_variant
rs1792043434	intron_variant,genic_upstream_transcript_variant
rs1792043585	intron_variant,genic_upstream_transcript_variant
rs1792043771	intron_variant,genic_upstream_transcript_variant
rs1792044698	intron_variant,genic_upstream_transcript_variant
rs1792044886	intron_variant,genic_upstream_transcript_variant
rs1792045550	intron_variant,genic_upstream_transcript_variant
rs1792045638	intron_variant,genic_upstream_transcript_variant
rs1792045822	intron_variant,genic_upstream_transcript_variant
rs1792046115	intron_variant,genic_upstream_transcript_variant
rs1792046233	intron_variant,genic_upstream_transcript_variant
rs1792046320	intron_variant,genic_upstream_transcript_variant
rs1792046409	intron_variant,genic_upstream_transcript_variant
rs1792046722	intron_variant,genic_upstream_transcript_variant
rs1792046814	intron_variant,genic_upstream_transcript_variant
rs1792047100	intron_variant,genic_upstream_transcript_variant
rs1792047185	intron_variant,genic_upstream_transcript_variant
rs1792047269	intron_variant,genic_upstream_transcript_variant
rs1792047365	intron_variant,genic_upstream_transcript_variant
rs1792047591	intron_variant,genic_upstream_transcript_variant
rs1792048011	intron_variant,genic_upstream_transcript_variant
rs1792048576	intron_variant,genic_upstream_transcript_variant
rs1792048662	intron_variant,genic_upstream_transcript_variant
rs1792049170	intron_variant,genic_upstream_transcript_variant
rs1792049260	intron_variant,genic_upstream_transcript_variant
rs1792049446	intron_variant,genic_upstream_transcript_variant
rs1792049625	intron_variant,genic_upstream_transcript_variant
rs1792050626	intron_variant,genic_upstream_transcript_variant
rs1792050829	intron_variant,genic_upstream_transcript_variant
rs1792051061	intron_variant,genic_upstream_transcript_variant
rs1792051246	intron_variant,genic_upstream_transcript_variant
rs1792051671	intron_variant,genic_upstream_transcript_variant
rs1792052106	intron_variant,genic_upstream_transcript_variant
rs1792052326	intron_variant,genic_upstream_transcript_variant
rs1792052419	intron_variant,genic_upstream_transcript_variant
rs1792052510	intron_variant,genic_upstream_transcript_variant
rs1792052605	intron_variant,genic_upstream_transcript_variant
rs1792052691	intron_variant,genic_upstream_transcript_variant
rs1792053632	intron_variant,genic_upstream_transcript_variant
rs1792053833	intron_variant,genic_upstream_transcript_variant
rs1792054637	intron_variant,genic_upstream_transcript_variant
rs1792055228	intron_variant,genic_upstream_transcript_variant
rs1792055408	intron_variant,genic_upstream_transcript_variant
rs1792055594	intron_variant,genic_upstream_transcript_variant
rs1792055684	intron_variant,genic_upstream_transcript_variant
rs1792055795	intron_variant,genic_upstream_transcript_variant

rs1792055892	intron_variant,genic_upstream_transcript_variant
rs1792056088	intron_variant,genic_upstream_transcript_variant
rs1792056175	intron_variant,genic_upstream_transcript_variant
rs1792056265	intron_variant,genic_upstream_transcript_variant
rs1792056585	intron_variant,genic_upstream_transcript_variant
rs1792056719	intron_variant,genic_upstream_transcript_variant
rs1792056903	intron_variant,genic_upstream_transcript_variant
rs1792057213	intron_variant,genic_upstream_transcript_variant
rs1792057328	intron_variant,genic_upstream_transcript_variant
rs1792057433	intron_variant,genic_upstream_transcript_variant
rs1792057636	intron_variant,genic_upstream_transcript_variant
rs1792057912	intron_variant,genic_upstream_transcript_variant
rs1792058199	intron_variant,genic_upstream_transcript_variant
rs1792058290	intron_variant,genic_upstream_transcript_variant
rs1792059210	intron_variant,genic_upstream_transcript_variant
rs1792059430	intron_variant,genic_upstream_transcript_variant
rs1792060002	intron_variant,genic_upstream_transcript_variant
rs1792060543	intron_variant,genic_upstream_transcript_variant
rs1792062127	intron_variant,genic_upstream_transcript_variant
rs1792063033	intron_variant,genic_upstream_transcript_variant
rs1792063337	intron_variant,genic_upstream_transcript_variant
rs1792064287	intron_variant,genic_upstream_transcript_variant
rs1792064480	intron_variant,genic_upstream_transcript_variant
rs1792064562	intron_variant,genic_upstream_transcript_variant
rs1792064824	intron_variant,genic_upstream_transcript_variant
rs1792065499	intron_variant,genic_upstream_transcript_variant
rs1792066066	intron_variant,genic_upstream_transcript_variant
rs1792066575	intron_variant,genic_upstream_transcript_variant
rs1792067408	intron_variant,genic_upstream_transcript_variant
rs1792067508	intron_variant,genic_upstream_transcript_variant
rs1792067987	intron_variant,genic_upstream_transcript_variant
rs1792068277	intron_variant,genic_upstream_transcript_variant
rs1792068369	intron_variant,genic_upstream_transcript_variant
rs1792068567	intron_variant,genic_upstream_transcript_variant
rs1792068670	intron_variant,genic_upstream_transcript_variant
rs1792068764	intron_variant,genic_upstream_transcript_variant
rs1792068858	intron_variant,genic_upstream_transcript_variant
rs1792068943	intron_variant,genic_upstream_transcript_variant
rs1792070527	intron_variant,genic_upstream_transcript_variant
rs1792072687	intron_variant,genic_upstream_transcript_variant
rs1792072861	intron_variant,genic_upstream_transcript_variant
rs1792073135	intron_variant,genic_upstream_transcript_variant
rs1792073303	intron_variant,genic_upstream_transcript_variant
rs1792073736	intron_variant,genic_upstream_transcript_variant
rs1792073891	intron_variant,genic_upstream_transcript_variant
rs1792074047	intron_variant,genic_upstream_transcript_variant
rs1792074700	intron_variant,genic_upstream_transcript_variant
rs1792075966	intron_variant,genic_upstream_transcript_variant
rs1792077676	intron_variant,genic_upstream_transcript_variant
rs1792078038	intron_variant,genic_upstream_transcript_variant
rs1792078161	intron_variant,genic_upstream_transcript_variant
rs1792078267	intron_variant,genic_upstream_transcript_variant
rs1792078572	intron_variant,genic_upstream_transcript_variant
rs1792078674	intron_variant,genic_upstream_transcript_variant
rs1792078953	intron_variant,genic_upstream_transcript_variant
rs1792079045	intron_variant,genic_upstream_transcript_variant
rs1792079254	intron_variant,genic_upstream_transcript_variant
rs1792079509	intron_variant,genic_upstream_transcript_variant
rs1792079713	intron_variant,genic_upstream_transcript_variant
rs1792080014	intron_variant,genic_upstream_transcript_variant
rs1792080113	intron_variant,genic_upstream_transcript_variant
rs1792080303	intron_variant,genic_upstream_transcript_variant
rs1792080380	intron_variant,genic_upstream_transcript_variant
rs1792080557	intron_variant,genic_upstream_transcript_variant
rs1792080666	intron_variant,genic_upstream_transcript_variant
rs1792080917	intron_variant,genic_upstream_transcript_variant
rs1792081085	intron_variant,genic_upstream_transcript_variant
rs1792081249	intron_variant,genic_upstream_transcript_variant
rs1792081445	intron_variant,genic_upstream_transcript_variant
rs1792081529	intron_variant,genic_upstream_transcript_variant
rs1792081616	intron_variant,genic_upstream_transcript_variant
rs1792081921	intron_variant,genic_upstream_transcript_variant
rs1792082024	intron_variant,genic_upstream_transcript_variant
rs1792082136	intron_variant,genic_upstream_transcript_variant

rs1792082463	intron_variant,genic_upstream_transcript_variant
rs1792082647	intron_variant,genic_upstream_transcript_variant
rs1792082757	intron_variant,genic_upstream_transcript_variant
rs1792082833	intron_variant,genic_upstream_transcript_variant
rs1792083446	intron_variant,genic_upstream_transcript_variant
rs1792083921	intron_variant,genic_upstream_transcript_variant
rs1792084122	intron_variant,genic_upstream_transcript_variant
rs1792084238	intron_variant,genic_upstream_transcript_variant
rs1792084812	intron_variant,genic_upstream_transcript_variant
rs1792084900	intron_variant,genic_upstream_transcript_variant
rs1792084993	intron_variant,genic_upstream_transcript_variant
rs1792085100	intron_variant,genic_upstream_transcript_variant
rs1792085194	intron_variant,genic_upstream_transcript_variant
rs1792085288	intron_variant,genic_upstream_transcript_variant
rs1792085486	intron_variant,genic_upstream_transcript_variant
rs1792086077	intron_variant,genic_upstream_transcript_variant
rs1792086533	intron_variant,genic_upstream_transcript_variant
rs1792086732	intron_variant,genic_upstream_transcript_variant
rs1792086925	intron_variant,genic_upstream_transcript_variant
rs1792087022	intron_variant,genic_upstream_transcript_variant
rs1792087134	intron_variant,genic_upstream_transcript_variant
rs1792087222	intron_variant,genic_upstream_transcript_variant
rs1792087324	intron_variant,genic_upstream_transcript_variant
rs1792087863	intron_variant,genic_upstream_transcript_variant
rs1792088186	intron_variant,genic_upstream_transcript_variant
rs1792088507	intron_variant,genic_upstream_transcript_variant
rs1792089168	intron_variant,genic_upstream_transcript_variant
rs1792089468	intron_variant,genic_upstream_transcript_variant
rs1792089924	intron_variant,genic_upstream_transcript_variant
rs1792090093	intron_variant,genic_upstream_transcript_variant
rs1792090306	intron_variant,genic_upstream_transcript_variant
rs1792091480	intron_variant,genic_upstream_transcript_variant
rs1792091648	intron_variant,genic_upstream_transcript_variant
rs1792092307	intron_variant,genic_upstream_transcript_variant
rs1792092460	intron_variant,genic_upstream_transcript_variant
rs1792092619	intron_variant,genic_upstream_transcript_variant
rs1792092742	intron_variant,genic_upstream_transcript_variant
rs1792093447	intron_variant,genic_upstream_transcript_variant
rs1792093582	intron_variant,genic_upstream_transcript_variant
rs1792094580	intron_variant,genic_upstream_transcript_variant
rs1792094902	intron_variant,genic_upstream_transcript_variant
rs1792095050	intron_variant,genic_upstream_transcript_variant
rs1792095218	intron_variant,genic_upstream_transcript_variant
rs1792095360	intron_variant,genic_upstream_transcript_variant
rs1792095509	intron_variant,genic_upstream_transcript_variant
rs1792096308	intron_variant,genic_upstream_transcript_variant
rs1792096440	intron_variant,genic_upstream_transcript_variant
rs1792096562	intron_variant,genic_upstream_transcript_variant
rs1792096850	intron_variant,genic_upstream_transcript_variant
rs1792096988	intron_variant,genic_upstream_transcript_variant
rs1792097143	intron_variant,genic_upstream_transcript_variant
rs1792097281	intron_variant,genic_upstream_transcript_variant
rs1792097419	intron_variant,genic_upstream_transcript_variant
rs1792097733	intron_variant,genic_upstream_transcript_variant
rs1792097907	intron_variant,genic_upstream_transcript_variant
rs1792098586	intron_variant,genic_upstream_transcript_variant
rs1792098908	intron_variant,genic_upstream_transcript_variant
rs1792099686	intron_variant,genic_upstream_transcript_variant
rs1792099770	intron_variant,genic_upstream_transcript_variant
rs1792100043	intron_variant,genic_upstream_transcript_variant
rs1792100140	intron_variant,genic_upstream_transcript_variant
rs1792100325	intron_variant,genic_upstream_transcript_variant
rs1792100437	intron_variant,genic_upstream_transcript_variant
rs1792100694	intron_variant,genic_upstream_transcript_variant
rs1792100808	intron_variant,genic_upstream_transcript_variant
rs1792101094	intron_variant,genic_upstream_transcript_variant
rs1792101297	intron_variant,genic_upstream_transcript_variant
rs1792101376	intron_variant,genic_upstream_transcript_variant
rs1792101470	intron_variant,genic_upstream_transcript_variant
rs1792101554	intron_variant,genic_upstream_transcript_variant
rs1792101662	intron_variant,genic_upstream_transcript_variant
rs1792101744	intron_variant,genic_upstream_transcript_variant
rs1792102312	intron_variant,genic_upstream_transcript_variant
rs1792102988	intron_variant,genic_upstream_transcript_variant

rs1792103624	intron_variant,genic_upstream_transcript_variant
rs1792103691	intron_variant,genic_upstream_transcript_variant
rs1792103762	intron_variant,genic_upstream_transcript_variant
rs1792103916	intron_variant,genic_upstream_transcript_variant
rs1792104272	intron_variant,genic_upstream_transcript_variant
rs1792105223	intron_variant,genic_upstream_transcript_variant
rs1792105442	intron_variant,genic_upstream_transcript_variant
rs1792106410	intron_variant,genic_upstream_transcript_variant
rs1792106598	intron_variant,genic_upstream_transcript_variant
rs1792107472	intron_variant,genic_upstream_transcript_variant
rs1792107714	intron_variant,genic_upstream_transcript_variant
rs1792107991	intron_variant,genic_upstream_transcript_variant
rs1792108090	intron_variant,genic_upstream_transcript_variant
rs1792108181	intron_variant,genic_upstream_transcript_variant
rs1792108277	intron_variant,genic_upstream_transcript_variant
rs1792108626	intron_variant,genic_upstream_transcript_variant
rs1792108816	intron_variant,genic_upstream_transcript_variant
rs1792108913	intron_variant,genic_upstream_transcript_variant
rs1792108989	intron_variant,genic_upstream_transcript_variant
rs1792109091	intron_variant,genic_upstream_transcript_variant
rs1792109430	intron_variant,genic_upstream_transcript_variant
rs1792109531	intron_variant,genic_upstream_transcript_variant
rs1792109740	intron_variant,genic_upstream_transcript_variant
rs1792109829	intron_variant,genic_upstream_transcript_variant
rs1792110118	intron_variant,genic_upstream_transcript_variant
rs1792110437	intron_variant,genic_upstream_transcript_variant
rs1792111093	intron_variant,genic_upstream_transcript_variant
rs1792111180	intron_variant,genic_upstream_transcript_variant
rs1792111490	intron_variant,genic_upstream_transcript_variant
rs1792111591	intron_variant,genic_upstream_transcript_variant
rs1792111808	intron_variant,genic_upstream_transcript_variant
rs1792112145	intron_variant,genic_upstream_transcript_variant
rs1792112580	intron_variant,genic_upstream_transcript_variant
rs1792112683	intron_variant,genic_upstream_transcript_variant
rs1792112971	intron_variant,genic_upstream_transcript_variant
rs1792113063	intron_variant,genic_upstream_transcript_variant
rs1792113515	intron_variant,genic_upstream_transcript_variant
rs1792113624	intron_variant,genic_upstream_transcript_variant
rs1792113964	intron_variant,genic_upstream_transcript_variant
rs1792114159	intron_variant,genic_upstream_transcript_variant
rs1792114239	intron_variant,genic_upstream_transcript_variant
rs1792114561	intron_variant,genic_upstream_transcript_variant
rs1792114903	intron_variant,genic_upstream_transcript_variant
rs1792115000	intron_variant,genic_upstream_transcript_variant
rs1792115185	intron_variant,genic_upstream_transcript_variant
rs1792115690	intron_variant,genic_upstream_transcript_variant
rs1792116224	intron_variant,genic_upstream_transcript_variant
rs1792116312	intron_variant,genic_upstream_transcript_variant
rs1792117408	intron_variant,genic_upstream_transcript_variant
rs1792117644	intron_variant,genic_upstream_transcript_variant
rs1792117831	intron_variant,genic_upstream_transcript_variant
rs1792118010	intron_variant,genic_upstream_transcript_variant
rs1792118097	intron_variant,genic_upstream_transcript_variant
rs1792118385	intron_variant,genic_upstream_transcript_variant
rs1792118882	intron_variant,genic_upstream_transcript_variant
rs1792119799	intron_variant,genic_upstream_transcript_variant
rs1792119889	intron_variant,genic_upstream_transcript_variant
rs1792119982	intron_variant,genic_upstream_transcript_variant
rs1792120306	intron_variant,genic_upstream_transcript_variant
rs1792120395	intron_variant,genic_upstream_transcript_variant
rs1792120614	intron_variant,genic_upstream_transcript_variant
rs1792121587	intron_variant,genic_upstream_transcript_variant
rs1792121662	intron_variant,genic_upstream_transcript_variant
rs1792121981	intron_variant,genic_upstream_transcript_variant
rs1792122055	intron_variant,genic_upstream_transcript_variant
rs1792122168	intron_variant,genic_upstream_transcript_variant
rs1792124748	intron_variant,genic_upstream_transcript_variant
rs1792124850	intron_variant,genic_upstream_transcript_variant
rs1792125228	intron_variant,genic_upstream_transcript_variant
rs1792125333	intron_variant,genic_upstream_transcript_variant
rs1792125446	intron_variant,genic_upstream_transcript_variant
rs1792126756	intron_variant,genic_upstream_transcript_variant
rs1792127701	intron_variant,genic_upstream_transcript_variant
rs1792127992	intron_variant,genic_upstream_transcript_variant

rs1792128150	intron_variant,genic_upstream_transcript_variant
rs1792128539	intron_variant,genic_upstream_transcript_variant
rs1792129040	intron_variant,genic_upstream_transcript_variant
rs1792129249	intron_variant,genic_upstream_transcript_variant
rs1792129474	intron_variant,genic_upstream_transcript_variant
rs1792130066	intron_variant,genic_upstream_transcript_variant
rs1792130222	intron_variant,genic_upstream_transcript_variant
rs1792130414	intron_variant,genic_upstream_transcript_variant
rs1792130495	intron_variant,genic_upstream_transcript_variant
rs1792130589	intron_variant,genic_upstream_transcript_variant
rs1792131090	intron_variant,genic_upstream_transcript_variant
rs1792131322	intron_variant,genic_upstream_transcript_variant
rs1792131628	intron_variant,genic_upstream_transcript_variant
rs1792132184	intron_variant,genic_upstream_transcript_variant
rs1792132394	intron_variant,genic_upstream_transcript_variant
rs1792132506	intron_variant,genic_upstream_transcript_variant
rs1792132597	intron_variant,genic_upstream_transcript_variant
rs1792132917	intron_variant,genic_upstream_transcript_variant
rs1792133063	intron_variant,genic_upstream_transcript_variant
rs1792133352	intron_variant,genic_upstream_transcript_variant
rs1792133602	intron_variant,genic_upstream_transcript_variant
rs1792133886	intron_variant,genic_upstream_transcript_variant
rs1792134482	intron_variant,genic_upstream_transcript_variant
rs1792134599	intron_variant,genic_upstream_transcript_variant
rs1792134885	intron_variant,genic_upstream_transcript_variant
rs1792135000	intron_variant,genic_upstream_transcript_variant
rs1792135463	intron_variant,genic_upstream_transcript_variant
rs1792135616	intron_variant,genic_upstream_transcript_variant
rs1792135756	intron_variant,genic_upstream_transcript_variant
rs1792136059	intron_variant,genic_upstream_transcript_variant
rs1792136330	intron_variant,genic_upstream_transcript_variant
rs1792137072	intron_variant,genic_upstream_transcript_variant
rs1792137171	intron_variant,genic_upstream_transcript_variant
rs1792137394	intron_variant,genic_upstream_transcript_variant
rs1792137969	intron_variant,genic_upstream_transcript_variant
rs1792138286	intron_variant,genic_upstream_transcript_variant
rs1792138431	intron_variant,genic_upstream_transcript_variant
rs1792138875	intron_variant,genic_upstream_transcript_variant
rs1792139001	intron_variant,genic_upstream_transcript_variant
rs1792139157	intron_variant,genic_upstream_transcript_variant
rs1792139908	intron_variant,genic_upstream_transcript_variant
rs1792140084	intron_variant,genic_upstream_transcript_variant
rs1792140298	intron_variant,genic_upstream_transcript_variant
rs1792140851	intron_variant,genic_upstream_transcript_variant
rs1792141146	intron_variant,genic_upstream_transcript_variant
rs1792141435	intron_variant,genic_upstream_transcript_variant
rs1792142739	intron_variant,genic_upstream_transcript_variant
rs1792143239	intron_variant,genic_upstream_transcript_variant
rs1792143378	intron_variant,genic_upstream_transcript_variant
rs1792143528	intron_variant,genic_upstream_transcript_variant
rs1792143807	intron_variant,genic_upstream_transcript_variant
rs1792143955	intron_variant,genic_upstream_transcript_variant
rs1792144233	intron_variant,genic_upstream_transcript_variant
rs1792144620	intron_variant,genic_upstream_transcript_variant
rs1792145483	intron_variant,genic_upstream_transcript_variant
rs1792146013	intron_variant,genic_upstream_transcript_variant
rs1792146227	intron_variant,genic_upstream_transcript_variant
rs1792146327	intron_variant,genic_upstream_transcript_variant
rs1792146502	intron_variant,genic_upstream_transcript_variant
rs1792146588	intron_variant,genic_upstream_transcript_variant
rs1792146665	intron_variant,genic_upstream_transcript_variant
rs1792147456	intron_variant,genic_upstream_transcript_variant
rs1792147788	intron_variant,genic_upstream_transcript_variant
rs1792148075	intron_variant,genic_upstream_transcript_variant
rs1792149073	intron_variant,genic_upstream_transcript_variant
rs1792149289	intron_variant,genic_upstream_transcript_variant
rs1792149497	intron_variant,genic_upstream_transcript_variant
rs1792149606	intron_variant,genic_upstream_transcript_variant
rs1792149711	intron_variant,genic_upstream_transcript_variant
rs1792149815	intron_variant,genic_upstream_transcript_variant
rs1792149918	intron_variant,genic_upstream_transcript_variant
rs1792150129	intron_variant,genic_upstream_transcript_variant
rs1792150810	intron_variant,genic_upstream_transcript_variant
rs1792150902	intron_variant,genic_upstream_transcript_variant

rs1792151390	intron_variant,genic_upstream_transcript_variant
rs1792151759	intron_variant,genic_upstream_transcript_variant
rs1792151846	intron_variant,genic_upstream_transcript_variant
rs1792152231	intron_variant,genic_upstream_transcript_variant
rs1792152422	intron_variant,genic_upstream_transcript_variant
rs1792152558	intron_variant,genic_upstream_transcript_variant
rs1792153180	intron_variant,genic_upstream_transcript_variant
rs1792153548	intron_variant,genic_upstream_transcript_variant
rs1792153792	intron_variant,genic_upstream_transcript_variant
rs1792153914	intron_variant,genic_upstream_transcript_variant
rs1792154021	intron_variant,genic_upstream_transcript_variant
rs1792154134	intron_variant,genic_upstream_transcript_variant
rs1792154903	intron_variant,genic_upstream_transcript_variant
rs1792155196	intron_variant,genic_upstream_transcript_variant
rs1792155746	intron_variant,genic_upstream_transcript_variant
rs1792156595	intron_variant,genic_upstream_transcript_variant
rs1792156733	intron_variant,genic_upstream_transcript_variant
rs1792157228	intron_variant,genic_upstream_transcript_variant
rs1792157320	intron_variant,genic_upstream_transcript_variant
rs1792157434	intron_variant,genic_upstream_transcript_variant
rs1792158675	intron_variant,genic_upstream_transcript_variant
rs1792158781	intron_variant,genic_upstream_transcript_variant
rs1792159335	intron_variant,genic_upstream_transcript_variant
rs1792159434	intron_variant,genic_upstream_transcript_variant
rs1792160046	intron_variant,genic_upstream_transcript_variant
rs1792160404	intron_variant,genic_upstream_transcript_variant
rs1792160505	intron_variant,genic_upstream_transcript_variant
rs1792160588	intron_variant,genic_upstream_transcript_variant
rs1792161019	intron_variant,genic_upstream_transcript_variant
rs1792162235	intron_variant,genic_upstream_transcript_variant
rs1792162557	intron_variant,genic_upstream_transcript_variant
rs1792163545	intron_variant,genic_upstream_transcript_variant
rs1792164750	intron_variant,genic_upstream_transcript_variant
rs1792165043	intron_variant,genic_upstream_transcript_variant
rs1792166601	intron_variant,genic_upstream_transcript_variant
rs1792167492	intron_variant,genic_upstream_transcript_variant
rs1792168120	intron_variant,genic_upstream_transcript_variant
rs1792168546	intron_variant,genic_upstream_transcript_variant
rs1792169038	intron_variant,genic_upstream_transcript_variant
rs1792169245	intron_variant,genic_upstream_transcript_variant
rs1792169400	intron_variant,genic_upstream_transcript_variant
rs1792169591	intron_variant,genic_upstream_transcript_variant
rs1792170141	intron_variant,genic_upstream_transcript_variant
rs1792170348	intron_variant,genic_upstream_transcript_variant
rs1792171226	intron_variant,genic_upstream_transcript_variant
rs1792171518	intron_variant,genic_upstream_transcript_variant
rs1792171863	intron_variant,genic_upstream_transcript_variant
rs1792172368	intron_variant,genic_upstream_transcript_variant
rs1792172515	intron_variant,genic_upstream_transcript_variant
rs1792172664	intron_variant,genic_upstream_transcript_variant
rs1792173858	intron_variant,genic_upstream_transcript_variant
rs1792174181	intron_variant,genic_upstream_transcript_variant
rs1792175605	intron_variant,genic_upstream_transcript_variant
rs1792175761	intron_variant,genic_upstream_transcript_variant
rs1792175899	intron_variant,genic_upstream_transcript_variant
rs1792176247	intron_variant,genic_upstream_transcript_variant
rs1792176356	intron_variant,genic_upstream_transcript_variant
rs1792176669	intron_variant,genic_upstream_transcript_variant
rs1792176806	intron_variant,genic_upstream_transcript_variant
rs1792176916	intron_variant,genic_upstream_transcript_variant
rs1792177120	intron_variant,genic_upstream_transcript_variant
rs1792178038	intron_variant,genic_upstream_transcript_variant
rs1792178907	intron_variant,genic_upstream_transcript_variant
rs1792179060	intron_variant,genic_upstream_transcript_variant
rs1792179591	intron_variant,genic_upstream_transcript_variant
rs1792180333	intron_variant,genic_upstream_transcript_variant
rs1792180528	intron_variant,genic_upstream_transcript_variant
rs1792180897	intron_variant,genic_upstream_transcript_variant
rs1792181005	intron_variant,genic_upstream_transcript_variant
rs1792181218	intron_variant,genic_upstream_transcript_variant
rs1792181762	intron_variant,genic_upstream_transcript_variant
rs1792182520	intron_variant,genic_upstream_transcript_variant
rs1792183169	intron_variant,genic_upstream_transcript_variant
rs1792183652	intron_variant,genic_upstream_transcript_variant

rs1792183878	intron_variant,genic_upstream_transcript_variant
rs1792183985	intron_variant,genic_upstream_transcript_variant
rs1792185386	intron_variant,genic_upstream_transcript_variant
rs1792186287	intron_variant,genic_upstream_transcript_variant
rs1792186378	intron_variant,genic_upstream_transcript_variant
rs1792186457	intron_variant,genic_upstream_transcript_variant
rs1792186828	intron_variant,genic_upstream_transcript_variant
rs1792187078	intron_variant,genic_upstream_transcript_variant
rs1792187282	intron_variant,genic_upstream_transcript_variant
rs1792187396	intron_variant,genic_upstream_transcript_variant
rs1792187577	intron_variant,genic_upstream_transcript_variant
rs1792187785	intron_variant,genic_upstream_transcript_variant
rs1792187881	intron_variant,genic_upstream_transcript_variant
rs1792187984	intron_variant,genic_upstream_transcript_variant
rs1792188114	intron_variant,genic_upstream_transcript_variant
rs1792188422	intron_variant,genic_upstream_transcript_variant
rs1792188589	intron_variant,genic_upstream_transcript_variant
rs1792189118	intron_variant,genic_upstream_transcript_variant
rs1792189473	intron_variant,genic_upstream_transcript_variant
rs1792190197	intron_variant,genic_upstream_transcript_variant
rs1792190344	intron_variant,genic_upstream_transcript_variant
rs1792190652	intron_variant,genic_upstream_transcript_variant
rs1792192053	intron_variant,genic_upstream_transcript_variant
rs1792192811	intron_variant,genic_upstream_transcript_variant
rs1792192938	intron_variant,genic_upstream_transcript_variant
rs1792193041	intron_variant,genic_upstream_transcript_variant
rs1792193368	intron_variant,genic_upstream_transcript_variant
rs1792193649	intron_variant,genic_upstream_transcript_variant
rs1792193904	intron_variant,genic_upstream_transcript_variant
rs1792194270	intron_variant,genic_upstream_transcript_variant
rs1792194406	intron_variant,genic_upstream_transcript_variant
rs1792194859	intron_variant,genic_upstream_transcript_variant
rs1792194995	intron_variant,genic_upstream_transcript_variant
rs1792195133	intron_variant,genic_upstream_transcript_variant
rs1792195326	intron_variant,genic_upstream_transcript_variant
rs1792195431	intron_variant,genic_upstream_transcript_variant
rs1792195743	intron_variant,genic_upstream_transcript_variant
rs1792195824	intron_variant,genic_upstream_transcript_variant
rs1792195912	intron_variant,genic_upstream_transcript_variant
rs1792196009	intron_variant,genic_upstream_transcript_variant
rs1792196119	intron_variant,genic_upstream_transcript_variant
rs1792196223	intron_variant,genic_upstream_transcript_variant
rs1792196396	intron_variant,genic_upstream_transcript_variant
rs1792196567	intron_variant,genic_upstream_transcript_variant
rs1792196793	intron_variant,genic_upstream_transcript_variant
rs1792197040	intron_variant,genic_upstream_transcript_variant
rs1792197176	intron_variant,genic_upstream_transcript_variant
rs1792197332	intron_variant,genic_upstream_transcript_variant
rs1792197614	intron_variant,genic_upstream_transcript_variant
rs1792197777	intron_variant,genic_upstream_transcript_variant
rs1792198092	intron_variant,genic_upstream_transcript_variant
rs1792198254	intron_variant,genic_upstream_transcript_variant
rs1792198372	intron_variant,genic_upstream_transcript_variant
rs1792198474	intron_variant,genic_upstream_transcript_variant
rs1792199073	intron_variant,genic_upstream_transcript_variant
rs1792199286	intron_variant,genic_upstream_transcript_variant
rs1792199515	intron_variant,genic_upstream_transcript_variant
rs1792199651	intron_variant,genic_upstream_transcript_variant
rs1792199808	intron_variant,genic_upstream_transcript_variant
rs1792200134	intron_variant,genic_upstream_transcript_variant
rs1792200975	intron_variant,genic_upstream_transcript_variant
rs1792201144	intron_variant,genic_upstream_transcript_variant
rs1792201295	intron_variant,genic_upstream_transcript_variant
rs1792201824	intron_variant,genic_upstream_transcript_variant
rs1792202009	intron_variant,genic_upstream_transcript_variant
rs1792202621	intron_variant,genic_upstream_transcript_variant
rs1792202759	intron_variant,genic_upstream_transcript_variant
rs1792203074	intron_variant,genic_upstream_transcript_variant
rs1792203271	intron_variant,genic_upstream_transcript_variant
rs1792203418	intron_variant,genic_upstream_transcript_variant
rs1792203729	intron_variant,genic_upstream_transcript_variant
rs1792204042	intron_variant,genic_upstream_transcript_variant
rs1792204239	intron_variant,genic_upstream_transcript_variant
rs1792204687	intron_variant,genic_upstream_transcript_variant

rs1792204811	intron_variant,genic_upstream_transcript_variant
rs1792205127	intron_variant,genic_upstream_transcript_variant
rs1792205400	intron_variant,genic_upstream_transcript_variant
rs1792206088	intron_variant,genic_upstream_transcript_variant
rs1792207148	intron_variant,genic_upstream_transcript_variant
rs1792207360	intron_variant,genic_upstream_transcript_variant
rs1792207452	intron_variant,genic_upstream_transcript_variant
rs1792207544	intron_variant,genic_upstream_transcript_variant
rs1792208003	intron_variant,genic_upstream_transcript_variant
rs1792208685	intron_variant,genic_upstream_transcript_variant
rs1792208840	intron_variant,genic_upstream_transcript_variant
rs1792208990	intron_variant,genic_upstream_transcript_variant
rs1792209899	intron_variant,genic_upstream_transcript_variant
rs1792209993	intron_variant,genic_upstream_transcript_variant
rs1792210193	intron_variant,genic_upstream_transcript_variant
rs1792210459	intron_variant,genic_upstream_transcript_variant
rs1792210536	intron_variant,genic_upstream_transcript_variant
rs1792210620	intron_variant,genic_upstream_transcript_variant
rs1792210986	intron_variant,genic_upstream_transcript_variant
rs1792211076	intron_variant,genic_upstream_transcript_variant
rs1792211787	intron_variant,genic_upstream_transcript_variant
rs1792212137	intron_variant,genic_upstream_transcript_variant
rs1792213090	intron_variant,genic_upstream_transcript_variant
rs1792213176	intron_variant,genic_upstream_transcript_variant
rs1792213369	intron_variant,genic_upstream_transcript_variant
rs1792213500	intron_variant,genic_upstream_transcript_variant
rs1792213817	intron_variant,genic_upstream_transcript_variant
rs1792214128	intron_variant,genic_upstream_transcript_variant
rs1792214239	intron_variant,genic_upstream_transcript_variant
rs1792214475	intron_variant,genic_upstream_transcript_variant
rs1792214664	intron_variant,genic_upstream_transcript_variant
rs1792214883	intron_variant,genic_upstream_transcript_variant
rs1792214977	intron_variant,genic_upstream_transcript_variant
rs1792215060	intron_variant,genic_upstream_transcript_variant
rs1792215492	intron_variant,genic_upstream_transcript_variant
rs1792215585	intron_variant,genic_upstream_transcript_variant
rs1792215677	intron_variant,genic_upstream_transcript_variant
rs1792215756	intron_variant,genic_upstream_transcript_variant
rs1792215949	intron_variant,genic_upstream_transcript_variant
rs1792216045	intron_variant,genic_upstream_transcript_variant
rs1792216197	intron_variant,genic_upstream_transcript_variant
rs1792216606	intron_variant,genic_upstream_transcript_variant
rs1792217248	intron_variant,genic_upstream_transcript_variant
rs1792217401	intron_variant,genic_upstream_transcript_variant
rs1792217910	intron_variant,genic_upstream_transcript_variant
rs1792218436	intron_variant,genic_upstream_transcript_variant
rs1792218765	intron_variant,genic_upstream_transcript_variant
rs1792218935	intron_variant,genic_upstream_transcript_variant
rs1792219481	intron_variant,genic_upstream_transcript_variant
rs1792219587	intron_variant,genic_upstream_transcript_variant
rs1792219679	intron_variant,genic_upstream_transcript_variant
rs1792220667	intron_variant,genic_upstream_transcript_variant
rs1792221244	intron_variant,genic_upstream_transcript_variant
rs1792221972	intron_variant,genic_upstream_transcript_variant
rs1792222175	intron_variant,genic_upstream_transcript_variant
rs1792222627	intron_variant,genic_upstream_transcript_variant
rs1792223169	intron_variant,genic_upstream_transcript_variant
rs1792223387	intron_variant,genic_upstream_transcript_variant
rs1792223479	intron_variant,genic_upstream_transcript_variant
rs1792223701	intron_variant,genic_upstream_transcript_variant
rs1792223793	intron_variant,genic_upstream_transcript_variant
rs1792223923	intron_variant,genic_upstream_transcript_variant
rs1792224339	intron_variant,genic_upstream_transcript_variant
rs1792224997	intron_variant,genic_upstream_transcript_variant
rs1792225134	intron_variant,genic_upstream_transcript_variant
rs1792225399	intron_variant,genic_upstream_transcript_variant
rs1792225726	intron_variant,genic_upstream_transcript_variant
rs1792225897	intron_variant,genic_upstream_transcript_variant
rs1792226056	intron_variant,genic_upstream_transcript_variant
rs1792226295	intron_variant,genic_upstream_transcript_variant
rs1792226727	intron_variant,genic_upstream_transcript_variant
rs1792226888	intron_variant,genic_upstream_transcript_variant
rs1792226971	intron_variant,genic_upstream_transcript_variant
rs1792227079	intron_variant,genic_upstream_transcript_variant

rs1792227176	intron_variant,genic_upstream_transcript_variant
rs1792228043	intron_variant,genic_upstream_transcript_variant
rs1792228131	intron_variant,genic_upstream_transcript_variant
rs1792228364	intron_variant,genic_upstream_transcript_variant
rs1792229275	intron_variant,genic_upstream_transcript_variant
rs1792229498	intron_variant,genic_upstream_transcript_variant
rs1792229811	intron_variant,genic_upstream_transcript_variant
rs1792229929	intron_variant,genic_upstream_transcript_variant
rs1792230218	intron_variant,genic_upstream_transcript_variant
rs1792231296	intron_variant,genic_upstream_transcript_variant
rs1792231489	intron_variant,genic_upstream_transcript_variant
rs1792231694	intron_variant,genic_upstream_transcript_variant
rs1792231809	intron_variant,genic_upstream_transcript_variant
rs1792232046	intron_variant,genic_upstream_transcript_variant
rs1792232455	intron_variant,genic_upstream_transcript_variant
rs1792233081	intron_variant,genic_upstream_transcript_variant
rs1792233195	intron_variant,genic_upstream_transcript_variant
rs1792233381	intron_variant,genic_upstream_transcript_variant
rs1792233483	intron_variant,genic_upstream_transcript_variant
rs1792233575	intron_variant,genic_upstream_transcript_variant
rs1792234080	intron_variant,genic_upstream_transcript_variant
rs1792234175	intron_variant,genic_upstream_transcript_variant
rs1792234272	intron_variant,genic_upstream_transcript_variant
rs1792234361	intron_variant,genic_upstream_transcript_variant
rs1792234783	intron_variant,genic_upstream_transcript_variant
rs1792234904	intron_variant,genic_upstream_transcript_variant
rs1792235282	intron_variant,genic_upstream_transcript_variant
rs1792235372	intron_variant,genic_upstream_transcript_variant
rs1792235466	intron_variant,genic_upstream_transcript_variant
rs1792235860	intron_variant,genic_upstream_transcript_variant
rs1792236729	intron_variant,genic_upstream_transcript_variant
rs1792236829	intron_variant,genic_upstream_transcript_variant
rs1792236967	intron_variant,genic_upstream_transcript_variant
rs1792237295	intron_variant,genic_upstream_transcript_variant
rs1792237399	intron_variant,genic_upstream_transcript_variant
rs1792237866	intron_variant,genic_upstream_transcript_variant
rs1792237953	intron_variant,genic_upstream_transcript_variant
rs1792238143	intron_variant,genic_upstream_transcript_variant
rs1792238526	intron_variant,genic_upstream_transcript_variant
rs1792238612	intron_variant,genic_upstream_transcript_variant
rs1792238870	intron_variant,genic_upstream_transcript_variant
rs1792239028	intron_variant,genic_upstream_transcript_variant
rs1792239157	intron_variant,genic_upstream_transcript_variant
rs1792239361	intron_variant,genic_upstream_transcript_variant
rs1792239456	intron_variant,genic_upstream_transcript_variant
rs1792239557	intron_variant,genic_upstream_transcript_variant
rs1792239661	intron_variant,genic_upstream_transcript_variant
rs1792240070	intron_variant,genic_upstream_transcript_variant
rs1792240174	intron_variant,genic_upstream_transcript_variant
rs1792240574	intron_variant,genic_upstream_transcript_variant
rs1792240714	intron_variant,genic_upstream_transcript_variant
rs1792240804	intron_variant,genic_upstream_transcript_variant
rs1792240897	intron_variant,genic_upstream_transcript_variant
rs1792241015	intron_variant,genic_upstream_transcript_variant
rs1792241334	intron_variant,genic_upstream_transcript_variant
rs1792241605	intron_variant,genic_upstream_transcript_variant
rs1792241715	intron_variant,genic_upstream_transcript_variant
rs1792242209	intron_variant,genic_upstream_transcript_variant
rs1792242317	intron_variant,genic_upstream_transcript_variant
rs1792242462	intron_variant,genic_upstream_transcript_variant
rs1792242549	intron_variant,genic_upstream_transcript_variant
rs1792243233	intron_variant,genic_upstream_transcript_variant
rs1792243435	intron_variant,genic_upstream_transcript_variant
rs1792243612	intron_variant,genic_upstream_transcript_variant
rs1792243943	intron_variant,genic_upstream_transcript_variant
rs1792244150	intron_variant,genic_upstream_transcript_variant
rs1792244459	intron_variant,genic_upstream_transcript_variant
rs1792244560	intron_variant,genic_upstream_transcript_variant
rs1792244718	intron_variant,genic_upstream_transcript_variant
rs1792244825	intron_variant,genic_upstream_transcript_variant
rs1792244913	intron_variant,genic_upstream_transcript_variant
rs1792245010	intron_variant,genic_upstream_transcript_variant
rs1792245304	intron_variant,genic_upstream_transcript_variant
rs1792245544	intron_variant,genic_upstream_transcript_variant

rs1792245763	intron_variant,genic_upstream_transcript_variant
rs1792245962	intron_variant,genic_upstream_transcript_variant
rs1792246265	intron_variant,genic_upstream_transcript_variant
rs1792246357	intron_variant,genic_upstream_transcript_variant
rs1792246445	intron_variant,genic_upstream_transcript_variant
rs1792246802	intron_variant,genic_upstream_transcript_variant
rs1792247718	intron_variant,genic_upstream_transcript_variant
rs1792248101	intron_variant,genic_upstream_transcript_variant
rs1792248939	intron_variant,genic_upstream_transcript_variant
rs1792249460	intron_variant,genic_upstream_transcript_variant
rs1792249715	intron_variant,genic_upstream_transcript_variant
rs1792249807	intron_variant,genic_upstream_transcript_variant
rs1792250032	intron_variant,genic_upstream_transcript_variant
rs1792250122	intron_variant,genic_upstream_transcript_variant
rs1792250451	intron_variant,genic_upstream_transcript_variant
rs1792251733	intron_variant,genic_upstream_transcript_variant
rs1792252080	intron_variant,genic_upstream_transcript_variant
rs1792252226	intron_variant,genic_upstream_transcript_variant
rs1792252361	intron_variant,genic_upstream_transcript_variant
rs1792252697	intron_variant,genic_upstream_transcript_variant
rs1792252788	intron_variant,genic_upstream_transcript_variant
rs1792253151	intron_variant,genic_upstream_transcript_variant
rs1792253398	intron_variant,genic_upstream_transcript_variant
rs1792253971	intron_variant,genic_upstream_transcript_variant
rs1792254510	intron_variant,genic_upstream_transcript_variant
rs1792255288	intron_variant,genic_upstream_transcript_variant
rs1792255407	intron_variant,genic_upstream_transcript_variant
rs1792255558	intron_variant,genic_upstream_transcript_variant
rs1792255711	intron_variant,genic_upstream_transcript_variant
rs1792256367	intron_variant,genic_upstream_transcript_variant
rs1792256515	intron_variant,genic_upstream_transcript_variant
rs1792256661	intron_variant,genic_upstream_transcript_variant
rs1792257286	intron_variant,genic_upstream_transcript_variant
rs1792258329	intron_variant,genic_upstream_transcript_variant
rs1792258428	intron_variant,genic_upstream_transcript_variant
rs1792259056	intron_variant,genic_upstream_transcript_variant
rs1792259201	intron_variant,genic_upstream_transcript_variant
rs1792260116	intron_variant,genic_upstream_transcript_variant
rs1792260283	intron_variant,genic_upstream_transcript_variant
rs1792261221	intron_variant,genic_upstream_transcript_variant
rs1792261580	intron_variant,genic_upstream_transcript_variant
rs1792262689	intron_variant,genic_upstream_transcript_variant
rs1792262985	intron_variant,genic_upstream_transcript_variant
rs1792263576	intron_variant,genic_upstream_transcript_variant
rs1792263665	intron_variant,genic_upstream_transcript_variant
rs1792263915	intron_variant,genic_upstream_transcript_variant
rs1792264390	intron_variant,genic_upstream_transcript_variant
rs1792264936	intron_variant,genic_upstream_transcript_variant
rs1792265186	intron_variant,genic_upstream_transcript_variant
rs1792268339	intron_variant,genic_upstream_transcript_variant
rs1792268663	intron_variant,genic_upstream_transcript_variant
rs1792269307	intron_variant,genic_upstream_transcript_variant
rs1792269453	intron_variant,genic_upstream_transcript_variant
rs1792269611	intron_variant,genic_upstream_transcript_variant
rs1792269777	intron_variant,genic_upstream_transcript_variant
rs1792270467	intron_variant,genic_upstream_transcript_variant
rs1792270575	intron_variant,genic_upstream_transcript_variant
rs1792270844	intron_variant,genic_upstream_transcript_variant
rs1792270954	intron_variant,genic_upstream_transcript_variant
rs1792271408	intron_variant,genic_upstream_transcript_variant
rs1792271714	intron_variant,genic_upstream_transcript_variant
rs1792272723	intron_variant,genic_upstream_transcript_variant
rs1792272926	intron_variant,genic_upstream_transcript_variant
rs1792273114	intron_variant,genic_upstream_transcript_variant
rs1792273210	intron_variant,genic_upstream_transcript_variant
rs1792273578	intron_variant,genic_upstream_transcript_variant
rs1792273676	intron_variant,genic_upstream_transcript_variant
rs1792274174	intron_variant,genic_upstream_transcript_variant
rs1792275031	intron_variant,genic_upstream_transcript_variant
rs1792275267	intron_variant,genic_upstream_transcript_variant
rs1792275832	intron_variant,genic_upstream_transcript_variant
rs1792276030	intron_variant,genic_upstream_transcript_variant
rs1792276129	intron_variant,genic_upstream_transcript_variant
rs1792276568	intron_variant,genic_upstream_transcript_variant

rs1792276656	intron_variant,genic_upstream_transcript_variant
rs1792276963	intron_variant,genic_upstream_transcript_variant
rs1792277707	intron_variant,genic_upstream_transcript_variant
rs1792278022	intron_variant,genic_upstream_transcript_variant
rs1792278108	intron_variant,genic_upstream_transcript_variant
rs1792278400	intron_variant,genic_upstream_transcript_variant
rs1792279091	intron_variant,genic_upstream_transcript_variant
rs1792279491	intron_variant,genic_upstream_transcript_variant
rs1792279805	intron_variant,genic_upstream_transcript_variant
rs1792279953	intron_variant,genic_upstream_transcript_variant
rs1792280124	intron_variant,genic_upstream_transcript_variant
rs1792280666	intron_variant,genic_upstream_transcript_variant
rs1792281764	intron_variant,genic_upstream_transcript_variant
rs1792282240	intron_variant,genic_upstream_transcript_variant
rs1792282561	intron_variant,genic_upstream_transcript_variant
rs1792282872	intron_variant,genic_upstream_transcript_variant
rs1792283373	intron_variant,genic_upstream_transcript_variant
rs1792283543	intron_variant,genic_upstream_transcript_variant
rs1792284373	intron_variant,genic_upstream_transcript_variant
rs1792284513	intron_variant,genic_upstream_transcript_variant
rs1792284667	intron_variant,genic_upstream_transcript_variant
rs1792285010	intron_variant,genic_upstream_transcript_variant
rs1792285485	intron_variant,genic_upstream_transcript_variant
rs1792285632	intron_variant,genic_upstream_transcript_variant
rs1792286247	intron_variant,genic_upstream_transcript_variant
rs1792286408	intron_variant,genic_upstream_transcript_variant
rs1792287435	intron_variant,genic_upstream_transcript_variant
rs1792287498	intron_variant,genic_upstream_transcript_variant
rs1792287599	intron_variant,genic_upstream_transcript_variant
rs1792287812	intron_variant,genic_upstream_transcript_variant
rs1792287921	intron_variant,genic_upstream_transcript_variant
rs1792288429	intron_variant,genic_upstream_transcript_variant
rs1792288576	intron_variant,genic_upstream_transcript_variant
rs1792289763	intron_variant,genic_upstream_transcript_variant
rs1792289933	intron_variant,genic_upstream_transcript_variant
rs1792290245	intron_variant,genic_upstream_transcript_variant
rs1792290453	intron_variant,genic_upstream_transcript_variant
rs1792291891	intron_variant,genic_upstream_transcript_variant
rs1792292057	intron_variant,genic_upstream_transcript_variant
rs1792292909	intron_variant,genic_upstream_transcript_variant
rs1792296344	intron_variant,genic_upstream_transcript_variant
rs1792296669	intron_variant,genic_upstream_transcript_variant
rs1792296820	intron_variant,genic_upstream_transcript_variant
rs1792296964	intron_variant,genic_upstream_transcript_variant
rs1792297102	intron_variant,genic_upstream_transcript_variant
rs1792298259	intron_variant,genic_upstream_transcript_variant
rs1792298762	intron_variant,genic_upstream_transcript_variant
rs1792299299	intron_variant,genic_upstream_transcript_variant
rs1792299396	intron_variant,genic_upstream_transcript_variant
rs1792299675	intron_variant,genic_upstream_transcript_variant
rs1792300512	intron_variant,genic_upstream_transcript_variant
rs1792300622	intron_variant,genic_upstream_transcript_variant
rs1792300877	intron_variant,genic_upstream_transcript_variant
rs1792301038	intron_variant,genic_upstream_transcript_variant
rs1792301520	intron_variant,genic_upstream_transcript_variant
rs1792301722	intron_variant,genic_upstream_transcript_variant
rs1792302514	intron_variant,genic_upstream_transcript_variant
rs1792303057	intron_variant,genic_upstream_transcript_variant
rs1792304977	intron_variant,genic_upstream_transcript_variant
rs1792305453	intron_variant,genic_upstream_transcript_variant
rs1792306095	intron_variant,genic_upstream_transcript_variant
rs1792307056	intron_variant,genic_upstream_transcript_variant
rs1792308029	intron_variant,genic_upstream_transcript_variant
rs1792308353	intron_variant,genic_upstream_transcript_variant
rs1792309795	intron_variant,genic_upstream_transcript_variant
rs1792309997	intron_variant,genic_upstream_transcript_variant
rs1792310301	intron_variant,genic_upstream_transcript_variant
rs1792310388	intron_variant,genic_upstream_transcript_variant
rs1792310480	intron_variant,genic_upstream_transcript_variant
rs1792310855	intron_variant,genic_upstream_transcript_variant
rs1792310949	intron_variant,genic_upstream_transcript_variant
rs1792311170	intron_variant,genic_upstream_transcript_variant
rs1792311786	intron_variant,genic_upstream_transcript_variant
rs1792311869	intron_variant,genic_upstream_transcript_variant

rs1792312154	intron_variant,genic_upstream_transcript_variant
rs1792312249	intron_variant,genic_upstream_transcript_variant
rs1792312628	intron_variant,genic_upstream_transcript_variant
rs1792312727	intron_variant,genic_upstream_transcript_variant
rs1792313098	intron_variant,genic_upstream_transcript_variant
rs1792313451	intron_variant,genic_upstream_transcript_variant
rs1792313971	intron_variant,genic_upstream_transcript_variant
rs1792314071	intron_variant,genic_upstream_transcript_variant
rs1792314460	intron_variant,genic_upstream_transcript_variant
rs1792314974	intron_variant,genic_upstream_transcript_variant
rs1792315071	intron_variant,genic_upstream_transcript_variant
rs1792315166	intron_variant,genic_upstream_transcript_variant
rs1792315247	intron_variant,genic_upstream_transcript_variant
rs1792315341	intron_variant,genic_upstream_transcript_variant
rs1792315625	intron_variant,genic_upstream_transcript_variant
rs1792315716	intron_variant,genic_upstream_transcript_variant
rs1792315792	intron_variant,genic_upstream_transcript_variant
rs1792315892	intron_variant,genic_upstream_transcript_variant
rs1792316431	intron_variant,genic_upstream_transcript_variant
rs1792316518	intron_variant,genic_upstream_transcript_variant
rs1792316593	intron_variant,genic_upstream_transcript_variant
rs1792316695	intron_variant,genic_upstream_transcript_variant
rs1792316855	intron_variant,genic_upstream_transcript_variant
rs1792316950	intron_variant,genic_upstream_transcript_variant
rs1792317126	intron_variant,genic_upstream_transcript_variant
rs1792317493	intron_variant,genic_upstream_transcript_variant
rs1792318199	intron_variant,genic_upstream_transcript_variant
rs1792318331	intron_variant,genic_upstream_transcript_variant
rs1792318454	intron_variant,genic_upstream_transcript_variant
rs1792318576	intron_variant,genic_upstream_transcript_variant
rs1792319260	intron_variant,genic_upstream_transcript_variant
rs1792319712	intron_variant,genic_upstream_transcript_variant
rs1792319898	intron_variant,genic_upstream_transcript_variant
rs1792320081	intron_variant,genic_upstream_transcript_variant
rs1792320192	intron_variant,genic_upstream_transcript_variant
rs1792320423	intron_variant,genic_upstream_transcript_variant
rs1792320611	intron_variant,genic_upstream_transcript_variant
rs1792320715	intron_variant,genic_upstream_transcript_variant
rs1792321288	intron_variant,genic_upstream_transcript_variant
rs1792321367	intron_variant,genic_upstream_transcript_variant
rs1792321643	intron_variant,genic_upstream_transcript_variant
rs1792321902	intron_variant,genic_upstream_transcript_variant
rs1792322573	intron_variant,genic_upstream_transcript_variant
rs1792322666	intron_variant,genic_upstream_transcript_variant
rs1792322863	intron_variant,genic_upstream_transcript_variant
rs1792323797	intron_variant,genic_upstream_transcript_variant
rs1792324091	intron_variant,genic_upstream_transcript_variant
rs1792324256	intron_variant,genic_upstream_transcript_variant
rs1792324330	intron_variant,genic_upstream_transcript_variant
rs1792324433	intron_variant,genic_upstream_transcript_variant
rs1792324516	intron_variant,genic_upstream_transcript_variant
rs1792324995	intron_variant,genic_upstream_transcript_variant
rs1792325357	intron_variant,genic_upstream_transcript_variant
rs1792325862	intron_variant,genic_upstream_transcript_variant
rs1792325937	intron_variant,genic_upstream_transcript_variant
rs1792326039	intron_variant,genic_upstream_transcript_variant
rs1792326153	intron_variant,genic_upstream_transcript_variant
rs1792326557	intron_variant,genic_upstream_transcript_variant
rs1792326885	intron_variant,genic_upstream_transcript_variant
rs1792326974	intron_variant,genic_upstream_transcript_variant
rs1792327185	intron_variant,genic_upstream_transcript_variant
rs1792327752	intron_variant,genic_upstream_transcript_variant
rs1792327842	intron_variant,genic_upstream_transcript_variant
rs1792328047	intron_variant,genic_upstream_transcript_variant
rs1792328227	intron_variant,genic_upstream_transcript_variant
rs1792328678	intron_variant,genic_upstream_transcript_variant
rs1792328773	intron_variant,genic_upstream_transcript_variant
rs1792329181	intron_variant,genic_upstream_transcript_variant
rs1792329366	intron_variant,genic_upstream_transcript_variant
rs1792329592	intron_variant,genic_upstream_transcript_variant
rs1792329999	intron_variant,genic_upstream_transcript_variant
rs1792330385	intron_variant,genic_upstream_transcript_variant
rs1792331394	intron_variant,genic_upstream_transcript_variant
rs1792331737	intron_variant,genic_upstream_transcript_variant

rs1792331834	intron_variant,genic_upstream_transcript_variant
rs1792332009	intron_variant,genic_upstream_transcript_variant
rs1792332408	intron_variant,genic_upstream_transcript_variant
rs1792333457	intron_variant,genic_upstream_transcript_variant
rs1792333751	intron_variant,genic_upstream_transcript_variant
rs1792334157	intron_variant,genic_upstream_transcript_variant
rs1792334246	intron_variant,genic_upstream_transcript_variant
rs1792334424	intron_variant,genic_upstream_transcript_variant
rs1792335594	intron_variant,genic_upstream_transcript_variant
rs1792336219	intron_variant,genic_upstream_transcript_variant
rs1792336304	intron_variant,genic_upstream_transcript_variant
rs1792336587	intron_variant,genic_upstream_transcript_variant
rs1792336682	intron_variant,genic_upstream_transcript_variant
rs1792337031	intron_variant,genic_upstream_transcript_variant
rs1792337232	intron_variant,genic_upstream_transcript_variant
rs1792337320	intron_variant,genic_upstream_transcript_variant
rs1792337471	intron_variant,genic_upstream_transcript_variant
rs1792337773	intron_variant,genic_upstream_transcript_variant
rs1792338260	intron_variant,genic_upstream_transcript_variant
rs1792338591	intron_variant,genic_upstream_transcript_variant
rs1792338941	intron_variant,genic_upstream_transcript_variant
rs1792339092	intron_variant,genic_upstream_transcript_variant
rs1792339372	intron_variant,genic_upstream_transcript_variant
rs1792339571	intron_variant,genic_upstream_transcript_variant
rs1792339656	intron_variant,genic_upstream_transcript_variant
rs1792339809	intron_variant,genic_upstream_transcript_variant
rs1792340839	intron_variant,genic_upstream_transcript_variant
rs1792341110	intron_variant,genic_upstream_transcript_variant
rs1792341656	intron_variant,genic_upstream_transcript_variant
rs1792342502	intron_variant,genic_upstream_transcript_variant
rs1792342584	intron_variant,genic_upstream_transcript_variant
rs1792343305	intron_variant,genic_upstream_transcript_variant
rs1792343408	intron_variant,genic_upstream_transcript_variant
rs1792344226	intron_variant,genic_upstream_transcript_variant
rs1792344413	intron_variant,genic_upstream_transcript_variant
rs1792345094	intron_variant,genic_upstream_transcript_variant
rs1792345500	intron_variant,genic_upstream_transcript_variant
rs1792345580	intron_variant,genic_upstream_transcript_variant
rs1792346480	intron_variant,genic_upstream_transcript_variant
rs1792346764	intron_variant,genic_upstream_transcript_variant
rs1792346961	intron_variant,genic_upstream_transcript_variant
rs1792347474	intron_variant,genic_upstream_transcript_variant
rs1792347571	intron_variant,genic_upstream_transcript_variant
rs1792347818	intron_variant,genic_upstream_transcript_variant
rs1792348414	intron_variant,genic_upstream_transcript_variant
rs1792348502	intron_variant,genic_upstream_transcript_variant
rs1792348587	intron_variant,genic_upstream_transcript_variant
rs1792349297	intron_variant,genic_upstream_transcript_variant
rs1792349401	intron_variant,genic_upstream_transcript_variant
rs1792349560	intron_variant,genic_upstream_transcript_variant
rs1792349946	intron_variant,genic_upstream_transcript_variant
rs1792350032	intron_variant,genic_upstream_transcript_variant
rs1792350265	intron_variant,genic_upstream_transcript_variant
rs1792350438	intron_variant,genic_upstream_transcript_variant
rs1792350788	intron_variant,genic_upstream_transcript_variant
rs1792350879	intron_variant,genic_upstream_transcript_variant
rs1792351926	intron_variant,genic_upstream_transcript_variant
rs1792352128	intron_variant,genic_upstream_transcript_variant
rs1792352981	intron_variant,genic_upstream_transcript_variant
rs1792353195	intron_variant,genic_upstream_transcript_variant
rs1792353597	intron_variant,genic_upstream_transcript_variant
rs1792353685	intron_variant,genic_upstream_transcript_variant
rs1792353977	intron_variant,genic_upstream_transcript_variant
rs1792354077	intron_variant,genic_upstream_transcript_variant
rs1792354303	intron_variant,genic_upstream_transcript_variant
rs1792354760	intron_variant,genic_upstream_transcript_variant
rs1792354931	intron_variant,genic_upstream_transcript_variant
rs1792355492	intron_variant,genic_upstream_transcript_variant
rs1792355769	intron_variant,genic_upstream_transcript_variant
rs1792355858	intron_variant,genic_upstream_transcript_variant
rs1792356316	intron_variant,genic_upstream_transcript_variant
rs1792356412	intron_variant,genic_upstream_transcript_variant
rs1792357763	intron_variant,genic_upstream_transcript_variant
rs1792358770	intron_variant,genic_upstream_transcript_variant

rs1792359481	intron_variant,genic_upstream_transcript_variant
rs1792359629	intron_variant,genic_upstream_transcript_variant
rs1792360259	intron_variant,genic_upstream_transcript_variant
rs1792361895	intron_variant,genic_upstream_transcript_variant
rs1792362064	intron_variant,genic_upstream_transcript_variant
rs1792362561	intron_variant,genic_upstream_transcript_variant
rs1792362717	intron_variant,genic_upstream_transcript_variant
rs1792364512	intron_variant,genic_upstream_transcript_variant
rs1792364974	intron_variant,genic_upstream_transcript_variant
rs1792365061	intron_variant,genic_upstream_transcript_variant
rs1792365165	intron_variant,genic_upstream_transcript_variant
rs1792365260	intron_variant,genic_upstream_transcript_variant
rs1792365441	intron_variant,genic_upstream_transcript_variant
rs1792365799	intron_variant,genic_upstream_transcript_variant
rs1792366097	intron_variant,genic_upstream_transcript_variant
rs1792366198	intron_variant,genic_upstream_transcript_variant
rs1792367020	intron_variant,genic_upstream_transcript_variant
rs1792367364	intron_variant,genic_upstream_transcript_variant
rs1792367511	intron_variant,genic_upstream_transcript_variant
rs1792368183	intron_variant,genic_upstream_transcript_variant
rs1792368338	intron_variant,genic_upstream_transcript_variant
rs1792368621	intron_variant,genic_upstream_transcript_variant
rs1792369128	intron_variant,genic_upstream_transcript_variant
rs1792369452	intron_variant,genic_upstream_transcript_variant
rs1792369849	intron_variant,genic_upstream_transcript_variant
rs1792370262	intron_variant,genic_upstream_transcript_variant
rs1792370397	intron_variant,genic_upstream_transcript_variant
rs1792370550	intron_variant,genic_upstream_transcript_variant
rs1792371148	intron_variant,genic_upstream_transcript_variant
rs1792371743	intron_variant,genic_upstream_transcript_variant
rs1792373243	intron_variant,genic_upstream_transcript_variant
rs1792374596	intron_variant,genic_upstream_transcript_variant
rs1792374764	intron_variant,genic_upstream_transcript_variant
rs1792375295	intron_variant,genic_upstream_transcript_variant
rs1792375447	intron_variant,genic_upstream_transcript_variant
rs1792376035	intron_variant,genic_upstream_transcript_variant
rs1792376116	intron_variant,genic_upstream_transcript_variant
rs1792376619	intron_variant,genic_upstream_transcript_variant
rs1792377180	intron_variant,genic_upstream_transcript_variant
rs1792377822	intron_variant,genic_upstream_transcript_variant
rs1792378508	intron_variant,genic_upstream_transcript_variant
rs1792378991	intron_variant,genic_upstream_transcript_variant
rs1792379194	intron_variant,genic_upstream_transcript_variant
rs1792379288	intron_variant,genic_upstream_transcript_variant
rs1792379536	intron_variant,genic_upstream_transcript_variant
rs1792380203	intron_variant,genic_upstream_transcript_variant
rs1792380334	intron_variant,genic_upstream_transcript_variant
rs1792380524	intron_variant,genic_upstream_transcript_variant
rs1792380735	intron_variant,genic_upstream_transcript_variant
rs1792381479	intron_variant,genic_upstream_transcript_variant
rs1792381597	intron_variant,genic_upstream_transcript_variant
rs1792381683	intron_variant,genic_upstream_transcript_variant
rs1792381976	intron_variant,genic_upstream_transcript_variant
rs1792382064	intron_variant,genic_upstream_transcript_variant
rs1792382480	intron_variant,genic_upstream_transcript_variant
rs1792382758	intron_variant,genic_upstream_transcript_variant
rs1792383067	intron_variant,genic_upstream_transcript_variant
rs1792383291	intron_variant,genic_upstream_transcript_variant
rs1792383587	intron_variant,genic_upstream_transcript_variant
rs1792383686	intron_variant,genic_upstream_transcript_variant
rs1792383880	intron_variant,genic_upstream_transcript_variant
rs1792384030	intron_variant,genic_upstream_transcript_variant
rs1792384251	intron_variant,genic_upstream_transcript_variant
rs1792384716	intron_variant,genic_upstream_transcript_variant
rs1792384999	intron_variant,genic_upstream_transcript_variant
rs1792385092	intron_variant,genic_upstream_transcript_variant
rs1792385185	intron_variant,genic_upstream_transcript_variant
rs1792385273	intron_variant,genic_upstream_transcript_variant
rs1792385886	intron_variant,genic_upstream_transcript_variant
rs1792385979	intron_variant,genic_upstream_transcript_variant
rs1792386182	intron_variant,genic_upstream_transcript_variant
rs1792386402	intron_variant,genic_upstream_transcript_variant
rs1792386631	intron_variant,genic_upstream_transcript_variant
rs1792387272	intron_variant,genic_upstream_transcript_variant

rs1792387371	intron_variant,genic_upstream_transcript_variant
rs1792388029	intron_variant,genic_upstream_transcript_variant
rs1792388281	intron_variant,genic_upstream_transcript_variant
rs1792389222	intron_variant,genic_upstream_transcript_variant
rs1792389865	intron_variant,genic_upstream_transcript_variant
rs1792390374	intron_variant,genic_upstream_transcript_variant
rs1792391042	intron_variant,genic_upstream_transcript_variant
rs1792391432	intron_variant,genic_upstream_transcript_variant
rs1792391581	intron_variant,genic_upstream_transcript_variant
rs1792392205	intron_variant,genic_upstream_transcript_variant
rs1792392522	intron_variant,genic_upstream_transcript_variant
rs1792392892	intron_variant,genic_upstream_transcript_variant
rs1792393220	intron_variant,genic_upstream_transcript_variant
rs1792393387	intron_variant,genic_upstream_transcript_variant
rs1792393754	intron_variant,genic_upstream_transcript_variant
rs1792393903	intron_variant,genic_upstream_transcript_variant
rs1792393996	intron_variant,genic_upstream_transcript_variant
rs1792394101	intron_variant,genic_upstream_transcript_variant
rs1792394197	intron_variant,genic_upstream_transcript_variant
rs1792394322	intron_variant,genic_upstream_transcript_variant
rs1792394877	intron_variant,genic_upstream_transcript_variant
rs1792395096	intron_variant,genic_upstream_transcript_variant
rs1792395412	intron_variant,genic_upstream_transcript_variant
rs1792395784	intron_variant,genic_upstream_transcript_variant
rs1792395880	intron_variant,genic_upstream_transcript_variant
rs1792396509	intron_variant,genic_upstream_transcript_variant
rs1792396615	intron_variant,genic_upstream_transcript_variant
rs1792397077	intron_variant,genic_upstream_transcript_variant
rs1792397429	intron_variant,genic_upstream_transcript_variant
rs1792397736	intron_variant,genic_upstream_transcript_variant
rs1792398279	intron_variant,genic_upstream_transcript_variant
rs1792398605	intron_variant,genic_upstream_transcript_variant
rs1792398710	intron_variant,genic_upstream_transcript_variant
rs1792398811	intron_variant,genic_upstream_transcript_variant
rs1792399569	intron_variant,genic_upstream_transcript_variant
rs1792401293	intron_variant,genic_upstream_transcript_variant
rs1792401362	intron_variant,genic_upstream_transcript_variant
rs1792401701	intron_variant,genic_upstream_transcript_variant
rs1792402667	intron_variant,genic_upstream_transcript_variant
rs1792403223	intron_variant,genic_upstream_transcript_variant
rs1792403837	intron_variant,genic_upstream_transcript_variant
rs1792404028	intron_variant,genic_upstream_transcript_variant
rs1792404392	intron_variant,genic_upstream_transcript_variant
rs1792404779	intron_variant,genic_upstream_transcript_variant
rs1792405225	intron_variant,genic_upstream_transcript_variant
rs1792405490	intron_variant,genic_upstream_transcript_variant
rs1792406019	intron_variant,genic_upstream_transcript_variant
rs1792406228	intron_variant,genic_upstream_transcript_variant
rs1792406327	intron_variant,genic_upstream_transcript_variant
rs1792406432	intron_variant,genic_upstream_transcript_variant
rs1792406514	intron_variant,genic_upstream_transcript_variant
rs1792406623	intron_variant,genic_upstream_transcript_variant
rs1792407203	intron_variant,genic_upstream_transcript_variant
rs1792407446	intron_variant,genic_upstream_transcript_variant
rs1792407965	intron_variant,genic_upstream_transcript_variant
rs1792408178	intron_variant,genic_upstream_transcript_variant
rs1792408571	intron_variant,genic_upstream_transcript_variant
rs1792408662	intron_variant,genic_upstream_transcript_variant
rs1792409063	intron_variant,genic_upstream_transcript_variant
rs1792409168	intron_variant,genic_upstream_transcript_variant
rs1792409261	intron_variant,genic_upstream_transcript_variant
rs1792409545	intron_variant,genic_upstream_transcript_variant
rs1792409647	intron_variant,genic_upstream_transcript_variant
rs1792411092	intron_variant,genic_upstream_transcript_variant
rs1792411635	intron_variant,genic_upstream_transcript_variant
rs1792412511	intron_variant,genic_upstream_transcript_variant
rs1792412811	intron_variant,genic_upstream_transcript_variant
rs1792413858	intron_variant,genic_upstream_transcript_variant
rs1792413982	intron_variant,genic_upstream_transcript_variant
rs1792414075	intron_variant,genic_upstream_transcript_variant
rs1792414861	intron_variant,genic_upstream_transcript_variant
rs1792414955	intron_variant,genic_upstream_transcript_variant
rs1792415186	intron_variant,genic_upstream_transcript_variant
rs1792415390	intron_variant,genic_upstream_transcript_variant

rs1792415583	intron_variant,genic_upstream_transcript_variant
rs1792415950	intron_variant,genic_upstream_transcript_variant
rs1792416546	intron_variant,genic_upstream_transcript_variant
rs1792416868	intron_variant,genic_upstream_transcript_variant
rs1792417202	intron_variant,genic_upstream_transcript_variant
rs1792417437	intron_variant,genic_upstream_transcript_variant
rs1792417923	intron_variant,genic_upstream_transcript_variant
rs1792418141	intron_variant,genic_upstream_transcript_variant
rs1792418552	intron_variant,genic_upstream_transcript_variant
rs1792418858	intron_variant,genic_upstream_transcript_variant
rs1792419070	intron_variant,genic_upstream_transcript_variant
rs1792419196	intron_variant,genic_upstream_transcript_variant
rs1792419848	intron_variant,genic_upstream_transcript_variant
rs1792419951	intron_variant,genic_upstream_transcript_variant
rs1792421306	intron_variant,genic_upstream_transcript_variant
rs1792421407	intron_variant,genic_upstream_transcript_variant
rs1792421506	intron_variant,genic_upstream_transcript_variant
rs1792421919	intron_variant,genic_upstream_transcript_variant
rs1792422132	intron_variant,genic_upstream_transcript_variant
rs1792422310	intron_variant,genic_upstream_transcript_variant
rs1792422408	intron_variant,genic_upstream_transcript_variant
rs1792422490	intron_variant,genic_upstream_transcript_variant
rs1792422695	intron_variant,genic_upstream_transcript_variant
rs1792423064	intron_variant,genic_upstream_transcript_variant
rs1792423825	intron_variant,genic_upstream_transcript_variant
rs1792424106	intron_variant,genic_upstream_transcript_variant
rs1792424319	intron_variant,genic_upstream_transcript_variant
rs1792424446	intron_variant,genic_upstream_transcript_variant
rs1792424548	intron_variant,genic_upstream_transcript_variant
rs1792425205	intron_variant,genic_upstream_transcript_variant
rs1792425402	intron_variant,genic_upstream_transcript_variant
rs1792425844	intron_variant,genic_upstream_transcript_variant
rs1792426225	intron_variant,genic_upstream_transcript_variant
rs1792426525	intron_variant,genic_upstream_transcript_variant
rs1792426909	intron_variant,genic_upstream_transcript_variant
rs1792427125	intron_variant,genic_upstream_transcript_variant
rs1792427211	intron_variant,genic_upstream_transcript_variant
rs1792427474	intron_variant,genic_upstream_transcript_variant
rs1792427937	intron_variant,genic_upstream_transcript_variant
rs1792428027	intron_variant,genic_upstream_transcript_variant
rs1792428247	intron_variant,genic_upstream_transcript_variant
rs1792428573	intron_variant,genic_upstream_transcript_variant
rs1792428706	intron_variant,genic_upstream_transcript_variant
rs1792429049	intron_variant,genic_upstream_transcript_variant
rs1792429304	intron_variant,genic_upstream_transcript_variant
rs1792429727	intron_variant,genic_upstream_transcript_variant
rs1792430161	intron_variant,genic_upstream_transcript_variant
rs1792430434	intron_variant,genic_upstream_transcript_variant
rs1792430729	intron_variant,genic_upstream_transcript_variant
rs1792431086	intron_variant,genic_upstream_transcript_variant
rs1792431764	intron_variant,genic_upstream_transcript_variant
rs1792431874	intron_variant,genic_upstream_transcript_variant
rs1792432154	intron_variant,genic_upstream_transcript_variant
rs1792432262	intron_variant,genic_upstream_transcript_variant
rs1792432908	intron_variant,genic_upstream_transcript_variant
rs1792433063	intron_variant,genic_upstream_transcript_variant
rs1792433842	intron_variant,genic_upstream_transcript_variant
rs1792434219	intron_variant,genic_upstream_transcript_variant
rs1792434540	intron_variant,genic_upstream_transcript_variant
rs1792434897	intron_variant,genic_upstream_transcript_variant
rs1792435882	intron_variant,genic_upstream_transcript_variant
rs1792436007	intron_variant,genic_upstream_transcript_variant
rs1792436508	intron_variant,genic_upstream_transcript_variant
rs1792437116	intron_variant,genic_upstream_transcript_variant
rs1792437219	intron_variant,genic_upstream_transcript_variant
rs1792437539	intron_variant,genic_upstream_transcript_variant
rs1792437935	intron_variant,genic_upstream_transcript_variant
rs1792438238	intron_variant,genic_upstream_transcript_variant
rs1792438453	intron_variant,genic_upstream_transcript_variant
rs1792438692	intron_variant,genic_upstream_transcript_variant
rs1792438798	intron_variant,genic_upstream_transcript_variant
rs1792439618	intron_variant,genic_upstream_transcript_variant
rs1792439725	intron_variant,genic_upstream_transcript_variant
rs1792439821	intron_variant,genic_upstream_transcript_variant

rs1792439926	intron_variant,genic_upstream_transcript_variant
rs1792440024	intron_variant,genic_upstream_transcript_variant
rs1792440231	intron_variant,genic_upstream_transcript_variant
rs1792440344	intron_variant,genic_upstream_transcript_variant
rs1792440786	intron_variant,genic_upstream_transcript_variant
rs1792440995	intron_variant,genic_upstream_transcript_variant
rs1792441456	intron_variant,genic_upstream_transcript_variant
rs1792441955	intron_variant,genic_upstream_transcript_variant
rs1792442182	intron_variant,genic_upstream_transcript_variant
rs1792442338	intron_variant,genic_upstream_transcript_variant
rs1792442518	intron_variant,genic_upstream_transcript_variant
rs1792443362	intron_variant,genic_upstream_transcript_variant
rs1792443554	intron_variant,genic_upstream_transcript_variant
rs1792443708	intron_variant,genic_upstream_transcript_variant
rs1792444247	intron_variant,genic_upstream_transcript_variant
rs1792444795	intron_variant,genic_upstream_transcript_variant
rs1792445227	intron_variant,genic_upstream_transcript_variant
rs1792445997	intron_variant,genic_upstream_transcript_variant
rs1792446245	intron_variant,genic_upstream_transcript_variant
rs1792446354	intron_variant,genic_upstream_transcript_variant
rs1792447078	intron_variant,genic_upstream_transcript_variant
rs1792447421	intron_variant,genic_upstream_transcript_variant
rs1792447539	intron_variant,genic_upstream_transcript_variant
rs1792449042	intron_variant,genic_upstream_transcript_variant
rs1792449565	intron_variant,genic_upstream_transcript_variant
rs1792449787	intron_variant,genic_upstream_transcript_variant
rs1792449996	intron_variant,genic_upstream_transcript_variant
rs1792450555	intron_variant,genic_upstream_transcript_variant
rs1792450692	intron_variant,genic_upstream_transcript_variant
rs1792450805	intron_variant,genic_upstream_transcript_variant
rs1792450901	intron_variant,genic_upstream_transcript_variant
rs1792450996	intron_variant,genic_upstream_transcript_variant
rs1792451102	intron_variant,genic_upstream_transcript_variant
rs1792451552	intron_variant,genic_upstream_transcript_variant
rs1792451671	intron_variant,genic_upstream_transcript_variant
rs1792451786	intron_variant,genic_upstream_transcript_variant
rs1792452443	intron_variant,genic_upstream_transcript_variant
rs1792452547	intron_variant,genic_upstream_transcript_variant
rs1792452835	intron_variant,genic_upstream_transcript_variant
rs1792453601	intron_variant,genic_upstream_transcript_variant
rs1792453998	intron_variant,genic_upstream_transcript_variant
rs1792454342	intron_variant,genic_upstream_transcript_variant
rs1792454458	intron_variant,genic_upstream_transcript_variant
rs1792454795	intron_variant,genic_upstream_transcript_variant
rs1792455189	intron_variant,genic_upstream_transcript_variant
rs1792455558	intron_variant,genic_upstream_transcript_variant
rs1792456406	intron_variant,genic_upstream_transcript_variant
rs1792456543	intron_variant,genic_upstream_transcript_variant
rs1792457281	intron_variant,genic_upstream_transcript_variant
rs1792457388	intron_variant,genic_upstream_transcript_variant
rs1792457600	intron_variant,genic_upstream_transcript_variant
rs1792457710	intron_variant,genic_upstream_transcript_variant
rs1792457823	intron_variant,genic_upstream_transcript_variant
rs1792458728	intron_variant,genic_upstream_transcript_variant
rs1792458839	intron_variant,genic_upstream_transcript_variant
rs1792458941	intron_variant,genic_upstream_transcript_variant
rs1792459049	intron_variant,genic_upstream_transcript_variant
rs1792459259	intron_variant,genic_upstream_transcript_variant
rs1792459618	intron_variant,genic_upstream_transcript_variant
rs1792459706	intron_variant,genic_upstream_transcript_variant
rs1792459809	intron_variant,genic_upstream_transcript_variant
rs1792460668	intron_variant,genic_upstream_transcript_variant
rs1792461106	intron_variant,genic_upstream_transcript_variant
rs1792461209	intron_variant,genic_upstream_transcript_variant
rs1792461307	intron_variant,genic_upstream_transcript_variant
rs1792461526	intron_variant,genic_upstream_transcript_variant
rs1792461772	intron_variant,genic_upstream_transcript_variant
rs1792461985	intron_variant,genic_upstream_transcript_variant
rs1792462317	intron_variant,genic_upstream_transcript_variant
rs1792462417	intron_variant,genic_upstream_transcript_variant
rs1792462508	intron_variant,genic_upstream_transcript_variant
rs1792462728	intron_variant,genic_upstream_transcript_variant
rs1792462843	intron_variant,genic_upstream_transcript_variant
rs1792463166	intron_variant,genic_upstream_transcript_variant

rs1792463543	intron_variant,genic_upstream_transcript_variant
rs1792463767	intron_variant,genic_upstream_transcript_variant
rs1792464668	intron_variant,genic_upstream_transcript_variant
rs1792465033	intron_variant,genic_upstream_transcript_variant
rs1792465169	intron_variant,genic_upstream_transcript_variant
rs1792465460	intron_variant,genic_upstream_transcript_variant
rs1792465561	intron_variant,genic_upstream_transcript_variant
rs1792466070	intron_variant,genic_upstream_transcript_variant
rs1792466469	intron_variant,genic_upstream_transcript_variant
rs1792466670	intron_variant,genic_upstream_transcript_variant
rs1792466983	intron_variant,genic_upstream_transcript_variant
rs1792467208	intron_variant,genic_upstream_transcript_variant
rs1792467422	intron_variant,genic_upstream_transcript_variant
rs1792467517	intron_variant,genic_upstream_transcript_variant
rs1792467617	intron_variant,genic_upstream_transcript_variant
rs1792467738	intron_variant,genic_upstream_transcript_variant
rs1792468059	intron_variant,genic_upstream_transcript_variant
rs1792468431	intron_variant,genic_upstream_transcript_variant
rs1792469004	intron_variant,genic_upstream_transcript_variant
rs1792469328	intron_variant,genic_upstream_transcript_variant
rs1792469686	intron_variant,genic_upstream_transcript_variant
rs1792469781	intron_variant,genic_upstream_transcript_variant
rs1792470683	intron_variant,genic_upstream_transcript_variant
rs1792470787	intron_variant,genic_upstream_transcript_variant
rs1792470893	intron_variant,genic_upstream_transcript_variant
rs1792470992	intron_variant,genic_upstream_transcript_variant
rs1792471191	intron_variant,genic_upstream_transcript_variant
rs1792471295	intron_variant,genic_upstream_transcript_variant
rs1792471398	intron_variant,genic_upstream_transcript_variant
rs1792471525	intron_variant,genic_upstream_transcript_variant
rs1792472184	intron_variant,genic_upstream_transcript_variant
rs1792472281	intron_variant,genic_upstream_transcript_variant
rs1792472612	intron_variant,genic_upstream_transcript_variant
rs1792473115	intron_variant,genic_upstream_transcript_variant
rs1792473220	intron_variant,genic_upstream_transcript_variant
rs1792473657	intron_variant,genic_upstream_transcript_variant
rs1792473865	intron_variant,genic_upstream_transcript_variant
rs1792474031	intron_variant,genic_upstream_transcript_variant
rs1792474422	intron_variant,genic_upstream_transcript_variant
rs1792474534	intron_variant,genic_upstream_transcript_variant
rs1792474640	intron_variant,genic_upstream_transcript_variant
rs1792474973	intron_variant,genic_upstream_transcript_variant
rs1792475097	intron_variant,genic_upstream_transcript_variant
rs1792475483	intron_variant,genic_upstream_transcript_variant
rs1792475579	intron_variant,genic_upstream_transcript_variant
rs1792475806	intron_variant,genic_upstream_transcript_variant
rs1792476129	intron_variant,genic_upstream_transcript_variant
rs1792476880	intron_variant,genic_upstream_transcript_variant
rs1792476974	intron_variant,genic_upstream_transcript_variant
rs1792477264	intron_variant,genic_upstream_transcript_variant
rs1792477366	intron_variant,genic_upstream_transcript_variant
rs1792478136	intron_variant,genic_upstream_transcript_variant
rs1792478574	intron_variant,genic_upstream_transcript_variant
rs1792479290	intron_variant,genic_upstream_transcript_variant
rs1792479465	intron_variant,genic_upstream_transcript_variant
rs1792479950	intron_variant,genic_upstream_transcript_variant
rs1792480124	intron_variant,genic_upstream_transcript_variant
rs1792480474	intron_variant,genic_upstream_transcript_variant
rs1792480656	intron_variant,genic_upstream_transcript_variant
rs1792481031	intron_variant,genic_upstream_transcript_variant
rs1792481269	intron_variant,genic_upstream_transcript_variant
rs1792482008	intron_variant,genic_upstream_transcript_variant
rs1792482321	intron_variant,genic_upstream_transcript_variant
rs1792482516	intron_variant,genic_upstream_transcript_variant
rs1792482617	intron_variant,genic_upstream_transcript_variant
rs1792482836	intron_variant,genic_upstream_transcript_variant
rs1792483073	intron_variant,genic_upstream_transcript_variant
rs1792483279	intron_variant,genic_upstream_transcript_variant
rs1792483385	intron_variant,genic_upstream_transcript_variant
rs1792483767	intron_variant,genic_upstream_transcript_variant
rs1792483954	intron_variant,genic_upstream_transcript_variant
rs1792484054	intron_variant,genic_upstream_transcript_variant
rs1792484154	intron_variant,genic_upstream_transcript_variant
rs1792484474	intron_variant,genic_upstream_transcript_variant

rs1792484577	intron_variant,genic_upstream_transcript_variant
rs1792484810	intron_variant,genic_upstream_transcript_variant
rs1792484926	intron_variant,genic_upstream_transcript_variant
rs1792485328	intron_variant,genic_upstream_transcript_variant
rs1792485422	intron_variant,genic_upstream_transcript_variant
rs1792485543	intron_variant,genic_upstream_transcript_variant
rs1792486139	intron_variant,genic_upstream_transcript_variant
rs1792486485	intron_variant,genic_upstream_transcript_variant
rs1792486587	intron_variant,genic_upstream_transcript_variant
rs1792486686	intron_variant,genic_upstream_transcript_variant
rs1792487009	intron_variant,genic_upstream_transcript_variant
rs1792487549	intron_variant,genic_upstream_transcript_variant
rs1792487872	intron_variant,genic_upstream_transcript_variant
rs1792488170	intron_variant,genic_upstream_transcript_variant
rs1792488347	intron_variant,genic_upstream_transcript_variant
rs1792488626	intron_variant,genic_upstream_transcript_variant
rs1792488855	intron_variant,genic_upstream_transcript_variant
rs1792488953	intron_variant,genic_upstream_transcript_variant
rs1792489197	intron_variant,genic_upstream_transcript_variant
rs1792489412	intron_variant,genic_upstream_transcript_variant
rs1792489967	intron_variant,genic_upstream_transcript_variant
rs1792490068	intron_variant,genic_upstream_transcript_variant
rs1792490704	intron_variant,genic_upstream_transcript_variant
rs1792491021	intron_variant,genic_upstream_transcript_variant
rs1792491581	intron_variant,genic_upstream_transcript_variant
rs1792491690	intron_variant,genic_upstream_transcript_variant
rs1792491999	intron_variant,genic_upstream_transcript_variant
rs1792492283	intron_variant,genic_upstream_transcript_variant
rs1792492466	intron_variant,genic_upstream_transcript_variant
rs1792492833	intron_variant,genic_upstream_transcript_variant
rs1792493254	intron_variant,genic_upstream_transcript_variant
rs1792493352	intron_variant,genic_upstream_transcript_variant
rs1792493452	intron_variant,genic_upstream_transcript_variant
rs1792493546	intron_variant,genic_upstream_transcript_variant
rs1792493652	intron_variant,genic_upstream_transcript_variant
rs1792493767	intron_variant,genic_upstream_transcript_variant
rs1792494348	intron_variant,genic_upstream_transcript_variant
rs1792494734	intron_variant,genic_upstream_transcript_variant
rs1792494837	intron_variant,genic_upstream_transcript_variant
rs1792494942	intron_variant,genic_upstream_transcript_variant
rs1792496936	intron_variant,genic_upstream_transcript_variant
rs1792497110	intron_variant,genic_upstream_transcript_variant
rs1792497182	intron_variant,genic_upstream_transcript_variant
rs1792497320	intron_variant,genic_upstream_transcript_variant
rs1792497825	intron_variant,genic_upstream_transcript_variant
rs1792497922	intron_variant,genic_upstream_transcript_variant
rs1792498054	intron_variant,genic_upstream_transcript_variant
rs1792498147	intron_variant,genic_upstream_transcript_variant
rs1792498359	intron_variant,genic_upstream_transcript_variant
rs1792498857	intron_variant,genic_upstream_transcript_variant
rs1792499914	intron_variant,genic_upstream_transcript_variant
rs1792500037	intron_variant,genic_upstream_transcript_variant
rs1792500179	intron_variant,genic_upstream_transcript_variant
rs1792500350	intron_variant,genic_upstream_transcript_variant
rs1792500673	intron_variant,genic_upstream_transcript_variant
rs1792500787	intron_variant,genic_upstream_transcript_variant
rs1792501979	intron_variant,genic_upstream_transcript_variant
rs1792502495	intron_variant,genic_upstream_transcript_variant
rs1792502828	intron_variant,genic_upstream_transcript_variant
rs1792503563	intron_variant,genic_upstream_transcript_variant
rs1792503698	intron_variant,genic_upstream_transcript_variant
rs1792503879	intron_variant,genic_upstream_transcript_variant
rs1792504343	intron_variant,genic_upstream_transcript_variant
rs1792504505	intron_variant,genic_upstream_transcript_variant
rs1792505863	intron_variant,genic_upstream_transcript_variant
rs1792506019	intron_variant,genic_upstream_transcript_variant
rs1792506848	intron_variant,genic_upstream_transcript_variant
rs1792507063	intron_variant,genic_upstream_transcript_variant
rs1792507678	intron_variant,genic_upstream_transcript_variant
rs1792507814	intron_variant,genic_upstream_transcript_variant
rs1792508650	intron_variant,genic_upstream_transcript_variant
rs1792508954	intron_variant,genic_upstream_transcript_variant
rs1792509363	intron_variant,genic_upstream_transcript_variant
rs1792509518	intron_variant,genic_upstream_transcript_variant

rs1792509876	intron_variant,genic_upstream_transcript_variant
rs1792510668	intron_variant,genic_upstream_transcript_variant
rs1792511132	intron_variant,genic_upstream_transcript_variant
rs1792511563	intron_variant,genic_upstream_transcript_variant
rs1792511708	intron_variant,genic_upstream_transcript_variant
rs1792511860	intron_variant,genic_upstream_transcript_variant
rs1792512306	intron_variant,genic_upstream_transcript_variant
rs1792512639	intron_variant,genic_upstream_transcript_variant
rs1792513723	intron_variant,genic_upstream_transcript_variant
rs1792513864	intron_variant,genic_upstream_transcript_variant
rs1792514511	intron_variant,upstream_transcript_variant,genic_upstream_transcript_variant
rs1792514839	intron_variant,upstream_transcript_variant,genic_upstream_transcript_variant
rs1792515139	intron_variant,upstream_transcript_variant,genic_upstream_transcript_variant
rs1792515466	intron_variant,upstream_transcript_variant,genic_upstream_transcript_variant
rs1792515776	intron_variant,upstream_transcript_variant,genic_upstream_transcript_variant
rs1792516061	intron_variant,upstream_transcript_variant,genic_upstream_transcript_variant
rs1792516511	intron_variant,upstream_transcript_variant,genic_upstream_transcript_variant
rs1792516784	intron_variant,upstream_transcript_variant,genic_upstream_transcript_variant
rs1792516931	intron_variant,upstream_transcript_variant,genic_upstream_transcript_variant
rs1792517420	intron_variant,upstream_transcript_variant,genic_upstream_transcript_variant
rs1792518147	intron_variant,upstream_transcript_variant,genic_upstream_transcript_variant
rs1792518648	intron_variant,upstream_transcript_variant,genic_upstream_transcript_variant
rs1792519322	intron_variant,upstream_transcript_variant,genic_upstream_transcript_variant
rs1792519566	intron_variant,upstream_transcript_variant,genic_upstream_transcript_variant
rs1792519906	intron_variant,upstream_transcript_variant,genic_upstream_transcript_variant
rs1792520261	intron_variant,upstream_transcript_variant,genic_upstream_transcript_variant
rs1792520576	intron_variant,upstream_transcript_variant,genic_upstream_transcript_variant
rs1792520892	intron_variant,upstream_transcript_variant,genic_upstream_transcript_variant
rs1792521099	intron_variant,upstream_transcript_variant,genic_upstream_transcript_variant
rs1792521441	intron_variant,upstream_transcript_variant,genic_upstream_transcript_variant
rs1792522044	intron_variant,upstream_transcript_variant,genic_upstream_transcript_variant
rs1792522645	intron_variant,upstream_transcript_variant,genic_upstream_transcript_variant
rs1792522747	intron_variant,upstream_transcript_variant,genic_upstream_transcript_variant
rs1792522854	intron_variant,upstream_transcript_variant,genic_upstream_transcript_variant
rs1792522966	intron_variant,upstream_transcript_variant,genic_upstream_transcript_variant
rs1792523344	intron_variant,upstream_transcript_variant,genic_upstream_transcript_variant
rs1792523444	intron_variant,upstream_transcript_variant,genic_upstream_transcript_variant
rs1792523671	intron_variant,upstream_transcript_variant,genic_upstream_transcript_variant
rs1792523766	intron_variant,upstream_transcript_variant,genic_upstream_transcript_variant
rs1792523859	intron_variant,upstream_transcript_variant,genic_upstream_transcript_variant
rs1792524394	intron_variant,upstream_transcript_variant,genic_upstream_transcript_variant
rs1792524643	intron_variant,upstream_transcript_variant,genic_upstream_transcript_variant
rs1792524987	intron_variant,upstream_transcript_variant,genic_upstream_transcript_variant
rs1792525085	intron_variant,upstream_transcript_variant,genic_upstream_transcript_variant
rs1792525194	intron_variant,upstream_transcript_variant,genic_upstream_transcript_variant
rs1792525314	intron_variant,upstream_transcript_variant,genic_upstream_transcript_variant
rs1792525595	intron_variant,upstream_transcript_variant,genic_upstream_transcript_variant
rs1792527256	intron_variant,upstream_transcript_variant,genic_upstream_transcript_variant
rs1792527592	intron_variant,upstream_transcript_variant,genic_upstream_transcript_variant
rs1792527695	intron_variant,upstream_transcript_variant,genic_upstream_transcript_variant
rs1792527817	intron_variant,upstream_transcript_variant,genic_upstream_transcript_variant
rs1792528199	intron_variant,upstream_transcript_variant,genic_upstream_transcript_variant
rs1792528345	intron_variant,upstream_transcript_variant,genic_upstream_transcript_variant
rs1792528651	intron_variant,upstream_transcript_variant,genic_upstream_transcript_variant
rs1792529434	intron_variant,upstream_transcript_variant,genic_upstream_transcript_variant
rs1792530011	intron_variant,upstream_transcript_variant,genic_upstream_transcript_variant
rs1792530475	intron_variant,upstream_transcript_variant,genic_upstream_transcript_variant
rs1792530710	intron_variant,upstream_transcript_variant,genic_upstream_transcript_variant
rs1792530814	intron_variant,upstream_transcript_variant,genic_upstream_transcript_variant
rs1792531129	intron_variant,upstream_transcript_variant,genic_upstream_transcript_variant
rs1792531242	intron_variant,upstream_transcript_variant,genic_upstream_transcript_variant
rs1792531643	intron_variant,upstream_transcript_variant,genic_upstream_transcript_variant
rs1792532024	intron_variant,upstream_transcript_variant,genic_upstream_transcript_variant
rs1792532602	intron_variant,upstream_transcript_variant,genic_upstream_transcript_variant
rs1792532835	intron_variant,upstream_transcript_variant,genic_upstream_transcript_variant
rs1792532948	intron_variant,upstream_transcript_variant,genic_upstream_transcript_variant
rs1792533190	intron_variant,upstream_transcript_variant,genic_upstream_transcript_variant
rs1792533539	intron_variant,upstream_transcript_variant,genic_upstream_transcript_variant
rs1792533648	intron_variant,upstream_transcript_variant,genic_upstream_transcript_variant
rs1792533994	intron_variant,upstream_transcript_variant,genic_upstream_transcript_variant
rs1792534101	intron_variant,upstream_transcript_variant,genic_upstream_transcript_variant
rs1792534329	intron_variant,upstream_transcript_variant,genic_upstream_transcript_variant
rs1792534450	intron_variant,upstream_transcript_variant,genic_upstream_transcript_variant
rs1792534724	intron_variant,upstream_transcript_variant,genic_upstream_transcript_variant

rs1792535004	intron_variant,upstream_transcript_variant,genic_upstream_transcript_variant
rs1792535091	intron_variant,upstream_transcript_variant,genic_upstream_transcript_variant
rs1792535260	intron_variant,upstream_transcript_variant,genic_upstream_transcript_variant
rs1792535433	intron_variant,upstream_transcript_variant,genic_upstream_transcript_variant
rs1792535777	intron_variant,upstream_transcript_variant,genic_upstream_transcript_variant
rs1792536042	intron_variant,upstream_transcript_variant,genic_upstream_transcript_variant
rs1792536261	intron_variant,upstream_transcript_variant,genic_upstream_transcript_variant
rs1792536365	intron_variant,upstream_transcript_variant,genic_upstream_transcript_variant
rs1792536826	intron_variant,upstream_transcript_variant,genic_upstream_transcript_variant
rs1792537318	intron_variant,upstream_transcript_variant,genic_upstream_transcript_variant
rs1792537549	intron_variant,upstream_transcript_variant,genic_upstream_transcript_variant
rs1792537646	intron_variant,upstream_transcript_variant,genic_upstream_transcript_variant
rs1792537873	intron_variant,upstream_transcript_variant,genic_upstream_transcript_variant
rs1792538218	intron_variant,upstream_transcript_variant,genic_upstream_transcript_variant
rs1792538322	intron_variant,upstream_transcript_variant,genic_upstream_transcript_variant
rs1792538477	intron_variant,upstream_transcript_variant,genic_upstream_transcript_variant
rs1792539624	intron_variant,upstream_transcript_variant,genic_upstream_transcript_variant
rs1792541212	intron_variant,upstream_transcript_variant,genic_upstream_transcript_variant
rs1792541316	intron_variant,upstream_transcript_variant,genic_upstream_transcript_variant
rs1792541997	intron_variant,upstream_transcript_variant,genic_upstream_transcript_variant
rs1792542402	intron_variant,upstream_transcript_variant,genic_upstream_transcript_variant
rs1792542693	intron_variant,upstream_transcript_variant,genic_upstream_transcript_variant
rs1792543230	intron_variant,upstream_transcript_variant,genic_upstream_transcript_variant
rs1792543445	intron_variant,upstream_transcript_variant,genic_upstream_transcript_variant
rs1792543555	intron_variant,upstream_transcript_variant,genic_upstream_transcript_variant
rs1792544593	intron_variant,upstream_transcript_variant,genic_upstream_transcript_variant
rs1792544701	intron_variant,upstream_transcript_variant,genic_upstream_transcript_variant
rs1792544806	intron_variant,upstream_transcript_variant,genic_upstream_transcript_variant
rs1792545150	intron_variant,upstream_transcript_variant,genic_upstream_transcript_variant
rs1792545259	intron_variant,upstream_transcript_variant,genic_upstream_transcript_variant
rs1792545363	intron_variant,upstream_transcript_variant,genic_upstream_transcript_variant
rs1792545471	intron_variant,upstream_transcript_variant,genic_upstream_transcript_variant
rs1792545684	intron_variant,upstream_transcript_variant,genic_upstream_transcript_variant
rs1792545894	intron_variant,upstream_transcript_variant,genic_upstream_transcript_variant
rs1792546221	intron_variant,upstream_transcript_variant,genic_upstream_transcript_variant
rs1792547152	intron_variant,upstream_transcript_variant,genic_upstream_transcript_variant
rs1792547353	intron_variant,upstream_transcript_variant,genic_upstream_transcript_variant
rs1792547470	intron_variant,upstream_transcript_variant,genic_upstream_transcript_variant
rs1792547675	intron_variant,upstream_transcript_variant,genic_upstream_transcript_variant
rs1792547783	intron_variant,upstream_transcript_variant,genic_upstream_transcript_variant
rs1792547894	intron_variant,upstream_transcript_variant,genic_upstream_transcript_variant
rs1792549432	intron_variant,upstream_transcript_variant,genic_upstream_transcript_variant
rs1792549988	intron_variant,upstream_transcript_variant,genic_upstream_transcript_variant
rs1792550345	intron_variant,upstream_transcript_variant,genic_upstream_transcript_variant
rs1792550460	intron_variant,upstream_transcript_variant,genic_upstream_transcript_variant
rs1792550545	intron_variant,upstream_transcript_variant,genic_upstream_transcript_variant
rs1792550641	intron_variant,upstream_transcript_variant,genic_upstream_transcript_variant
rs1792551996	intron_variant,upstream_transcript_variant,genic_upstream_transcript_variant
rs1792553053	intron_variant,upstream_transcript_variant,genic_upstream_transcript_variant
rs1792553388	intron_variant,upstream_transcript_variant,genic_upstream_transcript_variant
rs1792554228	intron_variant,upstream_transcript_variant,genic_upstream_transcript_variant
rs1792554763	intron_variant,upstream_transcript_variant,genic_upstream_transcript_variant
rs1792554969	intron_variant,upstream_transcript_variant,genic_upstream_transcript_variant
rs1792555061	intron_variant,upstream_transcript_variant,genic_upstream_transcript_variant
rs1792555159	intron_variant,upstream_transcript_variant,genic_upstream_transcript_variant
rs1792555785	intron_variant,upstream_transcript_variant,genic_upstream_transcript_variant
rs1792556396	intron_variant,upstream_transcript_variant,genic_upstream_transcript_variant
rs1792556628	intron_variant,upstream_transcript_variant,genic_upstream_transcript_variant
rs1792556741	intron_variant,upstream_transcript_variant,genic_upstream_transcript_variant
rs1792556834	intron_variant,upstream_transcript_variant,genic_upstream_transcript_variant
rs1792557290	intron_variant,upstream_transcript_variant,genic_upstream_transcript_variant
rs1792557394	intron_variant,upstream_transcript_variant,genic_upstream_transcript_variant
rs1792558117	intron_variant,upstream_transcript_variant,genic_upstream_transcript_variant
rs1792558216	intron_variant,upstream_transcript_variant,genic_upstream_transcript_variant
rs1792558920	intron_variant,upstream_transcript_variant,genic_upstream_transcript_variant
rs1792559384	intron_variant,upstream_transcript_variant,genic_upstream_transcript_variant
rs1792559702	intron_variant,upstream_transcript_variant,genic_upstream_transcript_variant
rs1792560108	intron_variant,upstream_transcript_variant,genic_upstream_transcript_variant
rs1792560950	intron_variant,upstream_transcript_variant,genic_upstream_transcript_variant
rs1792561337	intron_variant,upstream_transcript_variant,genic_upstream_transcript_variant
rs1792561453	intron_variant,upstream_transcript_variant,genic_upstream_transcript_variant
rs1792561905	intron_variant,upstream_transcript_variant,genic_upstream_transcript_variant
rs1792562214	intron_variant,upstream_transcript_variant,genic_upstream_transcript_variant
rs1792562293	intron_variant,upstream_transcript_variant,genic_upstream_transcript_variant

rs1792562538	intron_variant,upstream_transcript_variant,genic_upstream_transcript_variant
rs1792563052	intron_variant,upstream_transcript_variant,genic_upstream_transcript_variant
rs1792563476	intron_variant,upstream_transcript_variant,genic_upstream_transcript_variant
rs1792564184	intron_variant,upstream_transcript_variant,genic_upstream_transcript_variant
rs1792564497	intron_variant,upstream_transcript_variant,genic_upstream_transcript_variant
rs1792564587	intron_variant,upstream_transcript_variant,genic_upstream_transcript_variant
rs1792564837	intron_variant,upstream_transcript_variant,genic_upstream_transcript_variant
rs1792564939	intron_variant,upstream_transcript_variant,genic_upstream_transcript_variant
rs1792565348	intron_variant,upstream_transcript_variant,genic_upstream_transcript_variant
rs1792565588	intron_variant,upstream_transcript_variant,genic_upstream_transcript_variant
rs1792565799	intron_variant,upstream_transcript_variant,genic_upstream_transcript_variant
rs1792566154	intron_variant,upstream_transcript_variant,genic_upstream_transcript_variant
rs1792566256	intron_variant,upstream_transcript_variant,genic_upstream_transcript_variant
rs1792566519	intron_variant,upstream_transcript_variant,genic_upstream_transcript_variant
rs1792567076	intron_variant,upstream_transcript_variant,genic_upstream_transcript_variant
rs1792567442	intron_variant,upstream_transcript_variant,genic_upstream_transcript_variant
rs1792568197	intron_variant,upstream_transcript_variant,genic_upstream_transcript_variant
rs1792568296	intron_variant,upstream_transcript_variant,genic_upstream_transcript_variant
rs1792568839	intron_variant,upstream_transcript_variant,genic_upstream_transcript_variant
rs1792568940	intron_variant,upstream_transcript_variant,genic_upstream_transcript_variant
rs1792569048	intron_variant,upstream_transcript_variant,genic_upstream_transcript_variant
rs1792569147	intron_variant,upstream_transcript_variant,genic_upstream_transcript_variant
rs1792569365	intron_variant,upstream_transcript_variant,genic_upstream_transcript_variant
rs1792569477	intron_variant,upstream_transcript_variant,genic_upstream_transcript_variant
rs1792569602	intron_variant,upstream_transcript_variant,genic_upstream_transcript_variant
rs1792570123	intron_variant,upstream_transcript_variant,genic_upstream_transcript_variant
rs1792570361	intron_variant,upstream_transcript_variant,genic_upstream_transcript_variant
rs1792570470	intron_variant,upstream_transcript_variant,genic_upstream_transcript_variant
rs1792570574	intron_variant,upstream_transcript_variant,genic_upstream_transcript_variant
rs1792570672	intron_variant,upstream_transcript_variant,genic_upstream_transcript_variant
rs1792570864	intron_variant,upstream_transcript_variant,genic_upstream_transcript_variant
rs1792570966	intron_variant,upstream_transcript_variant,genic_upstream_transcript_variant
rs1792571089	intron_variant,upstream_transcript_variant,genic_upstream_transcript_variant
rs1792571208	intron_variant,upstream_transcript_variant,genic_upstream_transcript_variant
rs1792572294	intron_variant,upstream_transcript_variant,genic_upstream_transcript_variant
rs1792572410	intron_variant,upstream_transcript_variant,genic_upstream_transcript_variant
rs1792573092	intron_variant,upstream_transcript_variant,genic_upstream_transcript_variant
rs1792573252	intron_variant,upstream_transcript_variant,genic_upstream_transcript_variant
rs1792573593	intron_variant,upstream_transcript_variant,genic_upstream_transcript_variant
rs1792574302	intron_variant,upstream_transcript_variant,genic_upstream_transcript_variant
rs1792574408	intron_variant,upstream_transcript_variant,genic_upstream_transcript_variant
rs1792574768	intron_variant,upstream_transcript_variant,genic_upstream_transcript_variant
rs1792575069	intron_variant,upstream_transcript_variant,genic_upstream_transcript_variant
rs1792575256	intron_variant,upstream_transcript_variant,genic_upstream_transcript_variant
rs1792575369	intron_variant,upstream_transcript_variant,genic_upstream_transcript_variant
rs1792575703	intron_variant,upstream_transcript_variant,genic_upstream_transcript_variant
rs1792575808	intron_variant,upstream_transcript_variant,genic_upstream_transcript_variant
rs1792576277	intron_variant,upstream_transcript_variant,genic_upstream_transcript_variant
rs1792576731	intron_variant,upstream_transcript_variant,genic_upstream_transcript_variant
rs1792576825	intron_variant,upstream_transcript_variant,genic_upstream_transcript_variant
rs1792576917	intron_variant,upstream_transcript_variant,genic_upstream_transcript_variant
rs1792577139	intron_variant,upstream_transcript_variant,genic_upstream_transcript_variant
rs1792577345	intron_variant,upstream_transcript_variant,genic_upstream_transcript_variant
rs1792577635	intron_variant,upstream_transcript_variant,genic_upstream_transcript_variant
rs1792577749	intron_variant,upstream_transcript_variant,genic_upstream_transcript_variant
rs1792578017	intron_variant,upstream_transcript_variant,genic_upstream_transcript_variant
rs1792578724	intron_variant,upstream_transcript_variant,genic_upstream_transcript_variant
rs1792578927	intron_variant,upstream_transcript_variant,genic_upstream_transcript_variant
rs1792579090	intron_variant,upstream_transcript_variant,genic_upstream_transcript_variant
rs1792579192	intron_variant,upstream_transcript_variant,genic_upstream_transcript_variant
rs1792579505	intron_variant,upstream_transcript_variant,genic_upstream_transcript_variant
rs1792581120	intron_variant,upstream_transcript_variant,genic_upstream_transcript_variant
rs1792581215	intron_variant,upstream_transcript_variant,genic_upstream_transcript_variant
rs1792581321	intron_variant,upstream_transcript_variant,genic_upstream_transcript_variant
rs1792581429	intron_variant,upstream_transcript_variant,genic_upstream_transcript_variant
rs1792582083	intron_variant,upstream_transcript_variant,genic_upstream_transcript_variant
rs1792582184	intron_variant,upstream_transcript_variant,genic_upstream_transcript_variant
rs1792582284	intron_variant,upstream_transcript_variant,genic_upstream_transcript_variant
rs1792582966	intron_variant,upstream_transcript_variant,genic_upstream_transcript_variant
rs1792583326	intron_variant,upstream_transcript_variant,genic_upstream_transcript_variant
rs1792584374	intron_variant,upstream_transcript_variant,genic_upstream_transcript_variant
rs1792585716	intron_variant,upstream_transcript_variant,genic_upstream_transcript_variant
rs1792586544	intron_variant,upstream_transcript_variant,genic_upstream_transcript_variant
rs1792587081	intron_variant,upstream_transcript_variant,genic_upstream_transcript_variant

rs1792587311	intron_variant,upstream_transcript_variant,genic_upstream_transcript_variant
rs1792587521	intron_variant,upstream_transcript_variant,genic_upstream_transcript_variant
rs1792588073	intron_variant,upstream_transcript_variant,genic_upstream_transcript_variant
rs1792588601	intron_variant,upstream_transcript_variant,genic_upstream_transcript_variant
rs1792588822	intron_variant,upstream_transcript_variant,genic_upstream_transcript_variant
rs1792589032	intron_variant,upstream_transcript_variant,genic_upstream_transcript_variant
rs1792589144	intron_variant,upstream_transcript_variant,genic_upstream_transcript_variant
rs1792589527	intron_variant,upstream_transcript_variant,genic_upstream_transcript_variant
rs1792589853	intron_variant,upstream_transcript_variant,genic_upstream_transcript_variant
rs1792590195	intron_variant,upstream_transcript_variant,genic_upstream_transcript_variant
rs1792590271	intron_variant,upstream_transcript_variant,genic_upstream_transcript_variant
rs1792590482	intron_variant,upstream_transcript_variant,genic_upstream_transcript_variant
rs1792595200	intron_variant,upstream_transcript_variant,genic_upstream_transcript_variant
rs1792595457	intron_variant,upstream_transcript_variant,genic_upstream_transcript_variant
rs1792595843	intron_variant,upstream_transcript_variant,genic_upstream_transcript_variant
rs1792595957	intron_variant,upstream_transcript_variant,genic_upstream_transcript_variant
rs1792596182	intron_variant,upstream_transcript_variant,genic_upstream_transcript_variant
rs1792596780	intron_variant,upstream_transcript_variant,genic_upstream_transcript_variant
rs1792596921	intron_variant,upstream_transcript_variant,genic_upstream_transcript_variant
rs1792597357	intron_variant,upstream_transcript_variant,genic_upstream_transcript_variant
rs1792597723	intron_variant,upstream_transcript_variant,genic_upstream_transcript_variant
rs1792598362	intron_variant,upstream_transcript_variant,genic_upstream_transcript_variant
rs1792598930	intron_variant,upstream_transcript_variant,genic_upstream_transcript_variant
rs1792599458	intron_variant,upstream_transcript_variant,genic_upstream_transcript_variant
rs1792599551	intron_variant,upstream_transcript_variant,genic_upstream_transcript_variant
rs1792599690	intron_variant,upstream_transcript_variant,genic_upstream_transcript_variant
rs1792599803	intron_variant,upstream_transcript_variant,genic_upstream_transcript_variant
rs1792600157	intron_variant,upstream_transcript_variant,genic_upstream_transcript_variant
rs1792600291	intron_variant,upstream_transcript_variant,genic_upstream_transcript_variant
rs1792600409	intron_variant,upstream_transcript_variant,genic_upstream_transcript_variant
rs1792600516	intron_variant,upstream_transcript_variant,genic_upstream_transcript_variant
rs1792600740	intron_variant,upstream_transcript_variant,genic_upstream_transcript_variant
rs1792600953	intron_variant,upstream_transcript_variant,genic_upstream_transcript_variant
rs1792601189	intron_variant,upstream_transcript_variant,genic_upstream_transcript_variant
rs1792601313	intron_variant,upstream_transcript_variant,genic_upstream_transcript_variant
rs1792601431	intron_variant,upstream_transcript_variant,genic_upstream_transcript_variant
rs1792602006	intron_variant,upstream_transcript_variant,genic_upstream_transcript_variant
rs1792603455	intron_variant,upstream_transcript_variant,genic_upstream_transcript_variant
rs1792603722	intron_variant,upstream_transcript_variant,genic_upstream_transcript_variant
rs1792604009	intron_variant,upstream_transcript_variant,genic_upstream_transcript_variant
rs1792604285	intron_variant,upstream_transcript_variant,genic_upstream_transcript_variant
rs1792604398	intron_variant,upstream_transcript_variant,genic_upstream_transcript_variant
rs1792604509	intron_variant,upstream_transcript_variant,genic_upstream_transcript_variant
rs1792604621	intron_variant,upstream_transcript_variant,genic_upstream_transcript_variant
rs1792604737	intron_variant,upstream_transcript_variant,genic_upstream_transcript_variant
rs1792604824	intron_variant,upstream_transcript_variant,genic_upstream_transcript_variant
rs1792606584	intron_variant,upstream_transcript_variant,genic_upstream_transcript_variant
rs1792606998	intron_variant,upstream_transcript_variant,genic_upstream_transcript_variant
rs1792607444	intron_variant,upstream_transcript_variant,genic_upstream_transcript_variant
rs1792607554	intron_variant,upstream_transcript_variant,genic_upstream_transcript_variant
rs1792608272	intron_variant,upstream_transcript_variant,genic_upstream_transcript_variant
rs1792608696	intron_variant,upstream_transcript_variant,genic_upstream_transcript_variant
rs1792608977	intron_variant,upstream_transcript_variant,genic_upstream_transcript_variant
rs1792609333	intron_variant,upstream_transcript_variant,genic_upstream_transcript_variant
rs1792609425	intron_variant,upstream_transcript_variant,genic_upstream_transcript_variant
rs1792610026	intron_variant,upstream_transcript_variant,genic_upstream_transcript_variant
rs1792610639	intron_variant,upstream_transcript_variant,genic_upstream_transcript_variant
rs1792611199	intron_variant,upstream_transcript_variant,genic_upstream_transcript_variant
rs1792611726	intron_variant,upstream_transcript_variant,genic_upstream_transcript_variant
rs1792611829	intron_variant,upstream_transcript_variant,genic_upstream_transcript_variant
rs1792612137	intron_variant,upstream_transcript_variant,genic_upstream_transcript_variant
rs1792612230	intron_variant,upstream_transcript_variant,genic_upstream_transcript_variant
rs1792612482	intron_variant,upstream_transcript_variant,genic_upstream_transcript_variant
rs1792612711	intron_variant,upstream_transcript_variant,genic_upstream_transcript_variant
rs1792613186	intron_variant,upstream_transcript_variant,genic_upstream_transcript_variant
rs1792613414	intron_variant,upstream_transcript_variant,genic_upstream_transcript_variant
rs1792613646	intron_variant,upstream_transcript_variant,genic_upstream_transcript_variant
rs1792613784	intron_variant,upstream_transcript_variant,genic_upstream_transcript_variant
rs1792613896	intron_variant,upstream_transcript_variant,genic_upstream_transcript_variant
rs1792614108	intron_variant,upstream_transcript_variant,genic_upstream_transcript_variant
rs1792614200	intron_variant,upstream_transcript_variant,genic_upstream_transcript_variant
rs1792614310	intron_variant,upstream_transcript_variant,genic_upstream_transcript_variant
rs1792615039	intron_variant,upstream_transcript_variant,genic_upstream_transcript_variant
rs1792616143	intron_variant,upstream_transcript_variant,genic_upstream_transcript_variant

rs1792616757	intron_variant,upstream_transcript_variant,genic_upstream_transcript_variant
rs1792617398	intron_variant,upstream_transcript_variant,genic_upstream_transcript_variant
rs1792617723	intron_variant,upstream_transcript_variant,genic_upstream_transcript_variant
rs1792618085	intron_variant,upstream_transcript_variant,genic_upstream_transcript_variant
rs1792618203	intron_variant,upstream_transcript_variant,genic_upstream_transcript_variant
rs1792619340	intron_variant,upstream_transcript_variant,genic_upstream_transcript_variant
rs1792619508	intron_variant,upstream_transcript_variant,genic_upstream_transcript_variant
rs1792620552	intron_variant,upstream_transcript_variant,genic_upstream_transcript_variant
rs1792621006	intron_variant,upstream_transcript_variant,genic_upstream_transcript_variant
rs1792621267	intron_variant,upstream_transcript_variant,genic_upstream_transcript_variant
rs1792621732	intron_variant,upstream_transcript_variant,genic_upstream_transcript_variant
rs1792622176	intron_variant,upstream_transcript_variant,genic_upstream_transcript_variant
rs1792622410	intron_variant,upstream_transcript_variant,genic_upstream_transcript_variant
rs1792623003	intron_variant,upstream_transcript_variant,genic_upstream_transcript_variant
rs1792624068	intron_variant,upstream_transcript_variant,genic_upstream_transcript_variant
rs1792624284	intron_variant,upstream_transcript_variant,genic_upstream_transcript_variant
rs1792624553	intron_variant,upstream_transcript_variant,genic_upstream_transcript_variant
rs1792626061	intron_variant,5_prime_UTR_variant
rs1792626166	intron_variant,5_prime_UTR_variant
rs1792626415	intron_variant,5_prime_UTR_variant
rs1792626612	intron_variant,5_prime_UTR_variant
rs1792627095	intron_variant,5_prime_UTR_variant
rs1792627706	intron_variant,5_prime_UTR_variant
rs1792628695	intron_variant,5_prime_UTR_variant
rs1792629622	intron_variant,5_prime_UTR_variant
rs1792630502	intron_variant
rs1792631795	intron_variant
rs1792632424	intron_variant
rs1792632962	intron_variant
rs1792633373	intron_variant
rs1792633884	intron_variant
rs1792634598	intron_variant
rs1792635484	intron_variant
rs1792636111	intron_variant
rs1792637115	intron_variant
rs1792638240	intron_variant
rs1792638740	intron_variant
rs1792640328	intron_variant
rs1792641234	intron_variant
rs1792641585	intron_variant
rs1792641935	intron_variant
rs1792642100	intron_variant
rs1792642813	intron_variant
rs1792643004	intron_variant
rs1792644490	intron_variant
rs1792647040	intron_variant
rs1792647722	intron_variant
rs1792647914	intron_variant
rs1792648090	intron_variant
rs1792648257	intron_variant
rs1792651411	intron_variant
rs1792651712	intron_variant
rs1792651887	intron_variant
rs1792652580	intron_variant
rs1792652755	intron_variant
rs1792653883	intron_variant
rs1792654508	intron_variant
rs1792655591	intron_variant
rs1792658168	intron_variant
rs1792658500	intron_variant
rs1792658863	intron_variant
rs1792659217	intron_variant
rs1792659720	intron_variant
rs1792659906	intron_variant
rs1792660357	intron_variant
rs1792660472	intron_variant
rs1792660794	intron_variant
rs1792661134	intron_variant
rs1792661667	intron_variant
rs1792661999	intron_variant
rs1792662089	intron_variant
rs1792662190	intron_variant
rs1792662566	intron_variant
rs1792662666	intron_variant

rs1792663008	intron_variant
rs1792663219	intron_variant
rs1792663321	intron_variant
rs1792663529	intron_variant
rs1792663637	intron_variant
rs1792663836	intron_variant
rs1792663949	intron_variant
rs1792664051	intron_variant
rs1792664257	intron_variant
rs1792664681	intron_variant
rs1792665053	intron_variant
rs1792665860	intron_variant
rs1792666513	intron_variant
rs1792666742	intron_variant
rs1792668228	intron_variant
rs1792669049	intron_variant
rs1792669605	intron_variant
rs1792669685	intron_variant
rs1792669894	intron_variant
rs1792670007	intron_variant
rs1792670102	intron_variant
rs1792670225	intron_variant
rs1792670757	intron_variant
rs1792672739	intron_variant
rs1792673996	intron_variant,5_prime_UTR_variant,splice_acceptor_variant
rs1792674332	intron_variant,5_prime_UTR_variant
rs1792674788	intron_variant,5_prime_UTR_variant
rs1792675788	intron_variant,5_prime_UTR_variant
rs1792676482	intron_variant,5_prime_UTR_variant
rs1792677192	intron_variant,5_prime_UTR_variant
rs1792677679	intron_variant,5_prime_UTR_variant
rs1792678593	intron_variant,5_prime_UTR_variant
rs1792678748	intron_variant,5_prime_UTR_variant
rs1792678897	intron_variant,5_prime_UTR_variant
rs1792679313	intron_variant,splice_donor_variant
rs1792680528	intron_variant
rs1792681674	intron_variant
rs1792681840	intron_variant
rs1792682206	intron_variant
rs1792682317	intron_variant
rs1792682425	intron_variant
rs1792682528	intron_variant
rs1792682871	intron_variant
rs1792683068	intron_variant
rs1792683734	intron_variant
rs1792684067	intron_variant
rs1792684946	intron_variant
rs1792685043	intron_variant
rs1792685276	intron_variant
rs1792685494	intron_variant
rs1792685800	intron_variant
rs1792686003	intron_variant
rs1792686121	intron_variant
rs1792686495	intron_variant
rs1792686604	intron_variant
rs1792686954	intron_variant
rs1792687186	intron_variant
rs1792687481	intron_variant
rs1792687788	intron_variant
rs1792687891	intron_variant
rs1792688201	intron_variant
rs1792688299	intron_variant
rs1792688404	intron_variant
rs1792689485	intron_variant
rs1792689733	intron_variant
rs1792690076	intron_variant
rs1792690790	intron_variant
rs1792690890	intron_variant
rs1792691071	intron_variant
rs1792691226	intron_variant
rs1792691386	intron_variant
rs1792691558	intron_variant
rs1792691898	intron_variant
rs1792692244	intron_variant

rs1792692564	intron_variant
rs1792693202	intron_variant
rs1792693506	intron_variant
rs1792693657	intron_variant
rs1792693818	intron_variant
rs1792694189	intron_variant
rs1792694523	intron_variant
rs1792696236	intron_variant
rs1792696387	intron_variant
rs1792696749	intron_variant
rs1792697109	intron_variant
rs1792697598	intron_variant
rs1792697762	intron_variant
rs1792698621	intron_variant
rs1792698823	intron_variant
rs1792699338	intron_variant
rs1792699524	intron_variant
rs1792699869	intron_variant
rs1792700247	intron_variant
rs1792700821	intron_variant
rs1792701254	intron_variant
rs1792702271	intron_variant
rs1792702496	intron_variant
rs1792702695	intron_variant
rs1792702887	intron_variant
rs1792703051	intron_variant
rs1792703223	intron_variant
rs1792703432	intron_variant
rs1792704539	intron_variant
rs1792705520	intron_variant
rs1792706742	intron_variant
rs1792706932	intron_variant
rs1792707243	intron_variant
rs1792707803	intron_variant
rs1792707990	intron_variant
rs1792708166	intron_variant
rs1792709557	intron_variant
rs1792709710	intron_variant
rs1792711017	intron_variant
rs1792711792	intron_variant
rs1792712557	intron_variant
rs1792714020	intron_variant
rs1792714384	intron_variant
rs1792716320	intron_variant
rs1792716618	intron_variant
rs1792716734	intron_variant
rs1792716859	intron_variant
rs1792717206	intron_variant
rs1792717590	intron_variant
rs1792717701	intron_variant
rs1792717882	intron_variant
rs1792718297	intron_variant
rs1792720144	intron_variant
rs1792721814	intron_variant
rs1792722122	intron_variant
rs1792722546	intron_variant
rs1792723531	intron_variant
rs1792724323	intron_variant
rs1792724689	intron_variant
rs1792724859	intron_variant
rs1792725447	intron_variant
rs1792726012	intron_variant
rs1792726183	intron_variant
rs1792726353	intron_variant
rs1792726949	intron_variant
rs1792727158	intron_variant
rs1792727998	intron_variant
rs1792728794	intron_variant
rs1792729347	intron_variant
rs1792729722	intron_variant
rs1792730076	intron_variant
rs1792730247	intron_variant
rs1792730535	intron_variant
rs1792730924	intron_variant

rs1792731093	intron_variant
rs1792731520	intron_variant
rs1792731896	intron_variant
rs1792732107	intron_variant
rs1792732205	intron_variant
rs1792732306	intron_variant
rs1792732783	intron_variant
rs1792732907	intron_variant
rs1792734200	intron_variant
rs1792734310	intron_variant
rs1792734709	intron_variant
rs1792734887	intron_variant
rs1792735009	intron_variant
rs1792735314	intron_variant
rs1792735610	intron_variant
rs1792735828	intron_variant
rs1792736036	intron_variant
rs1792736886	intron_variant
rs1792737101	intron_variant
rs1792737331	intron_variant
rs1792738071	intron_variant
rs1792738165	intron_variant
rs1792738266	intron_variant
rs1792738934	intron_variant
rs1792739548	intron_variant
rs1792739654	intron_variant
rs1792740004	intron_variant
rs1792740258	intron_variant
rs1792740892	intron_variant
rs1792741274	intron_variant
rs1792741924	intron_variant
rs1792742014	intron_variant
rs1792742109	intron_variant
rs1792742206	intron_variant
rs1792742390	intron_variant
rs1792742667	intron_variant
rs1792742769	intron_variant
rs1792742953	intron_variant
rs1792743060	intron_variant
rs1792743151	intron_variant
rs1792743330	intron_variant
rs1792743440	intron_variant
rs1792743605	intron_variant
rs1792743740	intron_variant
rs1792744047	intron_variant
rs1792744559	intron_variant
rs1792744820	intron_variant
rs1792744992	intron_variant
rs1792745108	intron_variant
rs1792745219	intron_variant
rs1792745508	intron_variant
rs1792745900	intron_variant
rs1792746431	intron_variant
rs1792746797	intron_variant
rs1792747093	intron_variant
rs1792747258	intron_variant
rs1792747693	intron_variant
rs1792747900	intron_variant
rs1792748325	intron_variant
rs1792748902	intron_variant
rs1792749041	intron_variant
rs1792749149	intron_variant
rs1792749302	intron_variant
rs1792749510	intron_variant
rs1792750818	intron_variant
rs1792751452	intron_variant
rs1792752309	intron_variant
rs1792755618	intron_variant
rs1792755920	intron_variant
rs1792756069	intron_variant
rs1792756194	intron_variant
rs1792756299	intron_variant
rs1792756408	intron_variant
rs1792756632	intron_variant

rs1792756732	intron_variant
rs1792756841	intron_variant
rs1792757002	intron_variant
rs1792757255	intron_variant
rs1792757483	intron_variant
rs1792757715	intron_variant
rs1792757916	intron_variant
rs1792758001	intron_variant
rs1792758255	intron_variant
rs1792758352	intron_variant
rs1792758445	intron_variant
rs1792758555	intron_variant
rs1792758690	intron_variant
rs1792758921	intron_variant
rs1792759058	intron_variant
rs1792759165	intron_variant
rs1792759363	intron_variant
rs1792759466	intron_variant
rs1792760062	intron_variant
rs1792760352	intron_variant
rs1792760481	intron_variant
rs1792760744	intron_variant
rs1792760842	intron_variant
rs1792760991	intron_variant
rs1792761274	intron_variant
rs1792761442	intron_variant
rs1792761748	intron_variant
rs1792761914	intron_variant
rs1792762129	intron_variant
rs1792762297	intron_variant
rs1792762465	intron_variant
rs1792762877	intron_variant
rs1792763128	intron_variant
rs1792763269	intron_variant
rs1792763552	intron_variant
rs1792763730	intron_variant
rs1792763918	intron_variant
rs1792764255	intron_variant
rs1792764485	intron_variant
rs1792764562	intron_variant
rs1792764802	intron_variant
rs1792764915	intron_variant
rs1792765702	intron_variant
rs1792765879	intron_variant
rs1792766006	intron_variant
rs1792766281	intron_variant
rs1792766412	intron_variant
rs1792766529	intron_variant
rs1792766636	intron_variant
rs1792766876	intron_variant
rs1792766990	intron_variant
rs1792767111	intron_variant
rs1792767528	intron_variant
rs1792767733	intron_variant
rs1792767956	intron_variant
rs1792768236	intron_variant
rs1792769203	intron_variant
rs1792769666	intron_variant
rs1792769753	intron_variant
rs1792769964	intron_variant
rs1792770069	intron_variant
rs1792770232	intron_variant
rs1792770345	intron_variant
rs1792770463	intron_variant
rs1792771059	intron_variant
rs1792771259	intron_variant
rs1792771372	intron_variant
rs1792772117	intron_variant
rs1792772229	intron_variant
rs1792772348	intron_variant
rs1792772454	intron_variant
rs1792772583	intron_variant
rs1792772735	intron_variant
rs1792773539	intron_variant

rs1792773630	intron_variant
rs1792774247	intron_variant
rs1792774352	intron_variant
rs1792775070	intron_variant
rs1792775453	intron_variant
rs1792775567	intron_variant
rs1792775665	intron_variant
rs1792775795	intron_variant
rs1792776256	intron_variant
rs1792777495	intron_variant
rs1792778566	intron_variant
rs1792778743	intron_variant
rs1792778856	intron_variant
rs1792778973	intron_variant
rs1792779678	intron_variant
rs1792779988	intron_variant
rs1792780978	intron_variant
rs1792781176	intron_variant
rs1792781391	intron_variant
rs1792781488	intron_variant
rs1792782116	intron_variant
rs1792782345	intron_variant
rs1792782764	intron_variant
rs1792783038	intron_variant
rs1792783485	intron_variant
rs1792784490	intron_variant
rs1792784619	intron_variant
rs1792785397	intron_variant
rs1792785611	intron_variant
rs1792786191	intron_variant
rs1792786297	intron_variant
rs1792786546	intron_variant
rs1792786665	intron_variant
rs1792786864	intron_variant
rs1792788843	intron_variant
rs1792789203	intron_variant
rs1792789644	intron_variant
rs1792789757	intron_variant
rs1792789876	intron_variant
rs1792790159	intron_variant
rs1792790637	intron_variant
rs1792791768	intron_variant
rs1792792109	intron_variant
rs1792792280	intron_variant
rs1792792444	intron_variant
rs1792792659	intron_variant
rs1792792901	intron_variant
rs1792793061	intron_variant
rs1792794205	intron_variant
rs1792794699	intron_variant
rs1792797820	intron_variant
rs1792799755	intron_variant
rs1792801339	intron_variant
rs1792801889	intron_variant
rs1792803445	intron_variant
rs1792803808	intron_variant
rs1792804687	intron_variant
rs1792805246	intron_variant
rs1792806365	intron_variant
rs1792806703	intron_variant
rs1792806892	intron_variant
rs1792807076	intron_variant
rs1792807434	intron_variant
rs1792807537	intron_variant
rs1792807634	intron_variant
rs1792808171	intron_variant
rs1792808433	intron_variant
rs1792808716	intron_variant
rs1792808847	intron_variant
rs1792808977	intron_variant
rs1792809223	intron_variant
rs1792809328	intron_variant
rs1792809808	intron_variant
rs1792809920	intron_variant

rs1792810589	intron_variant
rs1792810684	intron_variant
rs1792810960	intron_variant
rs1792811065	intron_variant
rs1792811383	intron_variant
rs1792811595	intron_variant
rs1792812211	intron_variant
rs1792812314	intron_variant
rs1792812412	intron_variant
rs1792813029	intron_variant
rs1792813194	intron_variant
rs1792813343	intron_variant
rs1792813653	intron_variant
rs1792813859	intron_variant
rs1792814658	intron_variant
rs1792814948	intron_variant
rs1792815033	intron_variant
rs1792815148	intron_variant
rs1792815246	intron_variant
rs1792815353	intron_variant
rs1792815562	intron_variant
rs1792815897	intron_variant
rs1792817018	intron_variant
rs1792817778	intron_variant
rs1792818094	intron_variant
rs1792818585	intron_variant
rs1792819435	intron_variant
rs1792820298	intron_variant
rs1792820738	intron_variant
rs1792820913	intron_variant
rs1792821083	intron_variant
rs1792821237	intron_variant
rs1792821388	intron_variant
rs1792821553	intron_variant
rs1792821844	intron_variant
rs1792822160	intron_variant
rs1792822610	intron_variant
rs1792822913	intron_variant
rs1792823054	intron_variant
rs1792823225	intron_variant
rs1792823387	intron_variant
rs1792824172	intron_variant
rs1792824311	intron_variant
rs1792824473	intron_variant
rs1792824634	intron_variant
rs1792824799	intron_variant
rs1792825869	intron_variant
rs1792826153	intron_variant
rs1792826299	intron_variant
rs1792828299	intron_variant
rs1792828456	intron_variant
rs1792828832	intron_variant
rs1792829908	intron_variant
rs1792830714	intron_variant
rs1792830885	intron_variant
rs1792831160	intron_variant
rs1792832106	intron_variant
rs1792832206	intron_variant
rs1792832571	intron_variant
rs1792832669	intron_variant
rs1792832778	intron_variant
rs1792832879	intron_variant
rs1792833753	intron_variant
rs1792833858	intron_variant
rs1792833960	intron_variant
rs1792834482	intron_variant
rs1792834599	intron_variant
rs1792834689	intron_variant
rs1792834794	intron_variant
rs1792835034	intron_variant
rs1792835380	intron_variant
rs1792835648	intron_variant
rs1792835753	intron_variant
rs1792835835	intron_variant

rs1792836032	intron_variant
rs1792836346	intron_variant
rs1792836670	intron_variant
rs1792836818	intron_variant
rs1792837173	intron_variant
rs1792837516	intron_variant
rs1792837826	intron_variant
rs1792837908	intron_variant
rs1792838007	intron_variant
rs1792838460	intron_variant
rs1792838880	intron_variant
rs1792839130	intron_variant
rs1792839366	intron_variant
rs1792840464	intron_variant
rs1792840812	intron_variant
rs1792841089	intron_variant
rs1792841403	intron_variant
rs1792841553	intron_variant
rs1792841881	intron_variant
rs1792842214	intron_variant
rs1792842390	intron_variant
rs1792842961	intron_variant
rs1792843277	intron_variant
rs1792843428	intron_variant
rs1792844133	intron_variant
rs1792844781	intron_variant
rs1792845142	intron_variant
rs1792845845	intron_variant
rs1792846310	intron_variant
rs1792846456	intron_variant
rs1792846783	intron_variant
rs1792846920	intron_variant
rs1792847028	intron_variant
rs1792847119	intron_variant
rs1792847229	intron_variant
rs1792847635	intron_variant
rs1792847939	intron_variant
rs1792848038	intron_variant
rs1792848132	intron_variant
rs1792848578	intron_variant
rs1792849530	intron_variant
rs1792849781	intron_variant
rs1792850123	intron_variant
rs1792850359	intron_variant
rs1792850561	intron_variant
rs1792850919	intron_variant
rs1792851012	intron_variant
rs1792851219	intron_variant
rs1792851319	intron_variant
rs1792851962	intron_variant
rs1792852522	intron_variant
rs1792852710	intron_variant
rs1792852926	intron_variant
rs1792853393	intron_variant
rs1792853505	intron_variant
rs1792853875	intron_variant
rs1792853971	intron_variant
rs1792854470	intron_variant
rs1792854795	intron_variant
rs1792854981	intron_variant
rs1792855694	intron_variant
rs1792855889	intron_variant
rs1792856205	intron_variant
rs1792856294	intron_variant
rs1792856385	intron_variant
rs1792856699	intron_variant
rs1792856940	intron_variant
rs1792857158	intron_variant
rs1792857261	intron_variant
rs1792857460	intron_variant
rs1792857546	intron_variant
rs1792857637	intron_variant
rs1792857736	intron_variant
rs1792857929	intron_variant

rs1792858391	intron_variant
rs1792858694	intron_variant
rs1792858785	intron_variant
rs1792858889	intron_variant
rs1792858988	intron_variant
rs1792859335	intron_variant
rs1792859550	intron_variant
rs1792859762	intron_variant
rs1792859964	intron_variant
rs1792860817	intron_variant
rs1792861031	intron_variant
rs1792861501	intron_variant
rs1792861833	intron_variant
rs1792862350	intron_variant
rs1792862948	intron_variant
rs1792863116	intron_variant
rs1792863780	intron_variant
rs1792864114	intron_variant
rs1792864755	intron_variant
rs1792865483	intron_variant
rs1792865586	intron_variant
rs1792866767	intron_variant
rs1792866970	intron_variant
rs1792867158	intron_variant
rs1792867249	intron_variant
rs1792868826	intron_variant
rs1792868987	intron_variant
rs1792869578	intron_variant
rs1792869754	intron_variant
rs1792869930	intron_variant
rs1792870073	intron_variant
rs1792870276	intron_variant
rs1792870370	intron_variant
rs1792870569	intron_variant
rs1792870906	intron_variant
rs1792871551	intron_variant
rs1792871724	intron_variant
rs1792872692	intron_variant
rs1792872802	intron_variant
rs1792872933	intron_variant
rs1792873047	intron_variant
rs1792873221	intron_variant
rs1792874176	intron_variant
rs1792874340	intron_variant
rs1792874425	intron_variant
rs1792874914	intron_variant
rs1792875112	intron_variant
rs1792875509	intron_variant
rs1792875627	intron_variant
rs1792876053	intron_variant
rs1792876376	intron_variant
rs1792876495	intron_variant
rs1792876775	intron_variant
rs1792877842	intron_variant
rs1792878200	intron_variant
rs1792878393	intron_variant
rs1792879373	intron_variant
rs1792879476	intron_variant
rs1792879880	intron_variant
rs1792879983	intron_variant
rs1792880179	intron_variant
rs1792880274	intron_variant
rs1792880740	intron_variant
rs1792881052	intron_variant
rs1792881456	intron_variant
rs1792881552	intron_variant
rs1792881951	intron_variant
rs1792882264	intron_variant
rs1792882482	intron_variant
rs1792882677	intron_variant
rs1792883048	intron_variant
rs1792883276	intron_variant
rs1792883579	intron_variant
rs1792883671	intron_variant

rs1792885424	intron_variant
rs1792885530	intron_variant
rs1792885732	intron_variant
rs1792886268	intron_variant
rs1792886370	intron_variant
rs1792886464	intron_variant
rs1792887010	intron_variant
rs1792887224	intron_variant
rs1792887486	intron_variant
rs1792887575	intron_variant
rs1792887880	intron_variant
rs1792888352	intron_variant
rs1792888449	intron_variant
rs1792888660	intron_variant
rs1792888850	intron_variant
rs1792888966	intron_variant
rs1792889530	intron_variant
rs1792890018	intron_variant
rs1792890219	intron_variant
rs1792890318	intron_variant
rs1792890519	intron_variant
rs1792890619	intron_variant
rs1792890710	intron_variant
rs1792890806	intron_variant
rs1792890909	intron_variant
rs1792891013	intron_variant
rs1792891304	intron_variant
rs1792891715	intron_variant
rs1792891926	intron_variant
rs1792892014	intron_variant
rs1792892103	intron_variant
rs1792892336	intron_variant
rs1792892674	intron_variant
rs1792893000	intron_variant
rs1792893717	intron_variant
rs1792893814	intron_variant
rs1792893911	intron_variant
rs1792894124	intron_variant
rs1792894326	intron_variant
rs1792894422	intron_variant
rs1792894633	intron_variant
rs1792895091	intron_variant
rs1792895589	intron_variant
rs1792895711	intron_variant
rs1792895790	intron_variant
rs1792896518	intron_variant
rs1792896958	intron_variant
rs1792897068	intron_variant
rs1792897165	intron_variant
rs1792897406	intron_variant
rs1792897879	intron_variant
rs1792898371	intron_variant
rs1792898521	intron_variant
rs1792898864	intron_variant
rs1792899358	intron_variant
rs1792899591	intron_variant
rs1792900091	intron_variant
rs1792900773	intron_variant
rs1792901118	intron_variant
rs1792901353	intron_variant
rs1792901910	intron_variant
rs1792902720	intron_variant
rs1792902898	intron_variant
rs1792903429	intron_variant
rs1792903713	intron_variant
rs1792904328	intron_variant
rs1792905070	intron_variant
rs1792905291	intron_variant
rs1792906338	intron_variant
rs1792906548	intron_variant
rs1792906963	intron_variant
rs1792907063	intron_variant
rs1792907160	intron_variant
rs1792907249	intron_variant

rs1792907357	intron_variant
rs1792907466	intron_variant
rs1792908063	intron_variant
rs1792908176	intron_variant
rs1792908647	intron_variant
rs1792909873	intron_variant
rs1792910016	intron_variant
rs1792910121	intron_variant
rs1792910194	intron_variant,2KB_upstream_variant,upstream_transcript_variant,genic_upstream_transcript_variant
rs1792910834	intron_variant
rs1792911343	intron_variant
rs1792912124	intron_variant
rs1792912371	intron_variant
rs1792912772	intron_variant
rs1792912935	intron_variant
rs1792913103	intron_variant
rs1792913269	intron_variant
rs1792914184	intron_variant
rs1792914501	intron_variant
rs1792914696	intron_variant
rs1792914905	intron_variant
rs1792915015	intron_variant
rs1792915147	intron_variant
rs1792915331	intron_variant
rs1792916111	intron_variant
rs1792916854	intron_variant
rs1792917222	intron_variant
rs1792917381	intron_variant
rs1792917761	intron_variant
rs1792918184	intron_variant
rs1792918882	intron_variant
rs1792919212	intron_variant
rs1792920256	intron_variant
rs1792920619	intron_variant
rs1792921436	intron_variant
rs1792921697	intron_variant
rs1792921858	intron_variant
rs1792922550	intron_variant
rs1792923245	intron_variant
rs1792924084	intron_variant
rs1792924267	intron_variant
rs1792924433	intron_variant
rs1792925984	intron_variant
rs1792926122	intron_variant
rs1792926310	intron_variant
rs1792926493	intron_variant
rs1792926662	intron_variant
rs1792926826	intron_variant
rs1792927003	intron_variant
rs1792927610	intron_variant
rs1792928924	intron_variant
rs1792929108	intron_variant
rs1792929281	intron_variant
rs1792930257	intron_variant
rs1792931520	intron_variant
rs1792931709	intron_variant
rs1792931898	intron_variant
rs1792932086	intron_variant
rs1792932437	intron_variant
rs1792932599	intron_variant
rs1792933187	intron_variant
rs1792933975	intron_variant
rs1792934307	intron_variant
rs1792934479	intron_variant
rs1792934638	intron_variant
rs1792934804	intron_variant
rs1792934959	intron_variant
rs1792935318	intron_variant
rs1792935644	intron_variant
rs1792936579	intron_variant
rs1792937372	intron_variant
rs1792937534	intron_variant
rs1792937699	intron_variant,genic_downstream_transcript_variant
rs1792938792	intron_variant

rs1792939423	intron_variant
rs1792940337	intron_variant
rs1792940951	intron_variant
rs1792941060	intron_variant
rs1792941717	intron_variant
rs1792941826	intron_variant
rs1792942530	intron_variant
rs1792942872	intron_variant
rs1792942969	intron_variant
rs1792943686	intron_variant
rs1792943793	intron_variant
rs1792944020	intron_variant
rs1792944331	intron_variant
rs1792945110	intron_variant
rs1792945280	intron_variant
rs1792945453	intron_variant
rs1792946149	intron_variant
rs1792946516	intron_variant
rs1792946781	intron_variant
rs1792946891	intron_variant
rs1792947199	intron_variant
rs1792947527	intron_variant
rs1792947828	intron_variant
rs1792948013	intron_variant
rs1792948697	intron_variant
rs1792949063	intron_variant
rs1792949175	intron_variant
rs1792949826	intron_variant
rs1792950477	intron_variant
rs1792951214	intron_variant
rs1792951417	intron_variant
rs1792951715	intron_variant
rs1792951806	intron_variant
rs1792951913	intron_variant
rs1792952402	intron_variant
rs1792953662	intron_variant
rs1792953769	intron_variant
rs1792954514	intron_variant
rs1792954722	intron_variant
rs1792955312	intron_variant
rs1792955431	intron_variant
rs1792955913	intron_variant
rs1792956632	intron_variant
rs1792956803	intron_variant
rs1792957166	intron_variant
rs1792957620	intron_variant
rs1792957731	intron_variant
rs1792959138	intron_variant
rs1792959629	intron_variant
rs1792959851	intron_variant
rs1792959958	intron_variant
rs1792960293	intron_variant
rs1792960959	intron_variant
rs1792961069	intron_variant
rs1792961167	intron_variant
rs1792961270	intron_variant
rs1792961368	intron_variant
rs1792962717	intron_variant
rs1792963330	intron_variant
rs1792964431	intron_variant
rs1792964822	intron_variant
rs1792965369	intron_variant
rs1792965476	intron_variant
rs1792965797	intron_variant
rs1792965926	intron_variant
rs1792966269	intron_variant
rs1792966495	intron_variant
rs1792966977	intron_variant
rs1792967542	intron_variant
rs1792967901	intron_variant
rs1792968124	intron_variant
rs1792968245	intron_variant
rs1792968348	intron_variant
rs1792969471	intron_variant

rs1792969573	intron_variant
rs1792969865	intron_variant
rs1792969976	intron_variant
rs1792970340	intron_variant
rs1792970443	intron_variant
rs1792970690	intron_variant
rs1792970812	intron_variant
rs1792970919	intron_variant
rs1792971018	intron_variant
rs1792971248	intron_variant
rs1792971573	intron_variant
rs1792971672	intron_variant
rs1792971773	intron_variant
rs1792971869	intron_variant
rs1792972602	intron_variant
rs1792973093	intron_variant
rs1792973194	intron_variant
rs1792973501	intron_variant
rs1792973750	intron_variant
rs1792973853	intron_variant
rs1792975347	intron_variant
rs1792975661	intron_variant
rs1792975799	intron_variant
rs1792975964	intron_variant
rs1792976313	intron_variant
rs1792976465	intron_variant
rs1792976601	intron_variant
rs1792976771	intron_variant
rs1792977855	intron_variant
rs1792979220	intron_variant
rs1792980073	intron_variant
rs1792980303	intron_variant
rs1792980388	intron_variant
rs1792980686	intron_variant
rs1792981177	intron_variant
rs1792981600	intron_variant
rs1792981866	intron_variant
rs1792982084	intron_variant
rs1792982306	intron_variant
rs1792982428	intron_variant
rs1792982535	intron_variant
rs1792982632	intron_variant
rs1792982992	intron_variant
rs1792983158	intron_variant
rs1792983623	intron_variant
rs1792983961	intron_variant
rs1792984163	intron_variant
rs1792984267	intron_variant
rs1792985039	intron_variant
rs1792985142	intron_variant
rs1792985348	intron_variant
rs1792985463	intron_variant
rs1792986226	intron_variant
rs1792986341	intron_variant
rs1792986557	intron_variant
rs1792986649	intron_variant
rs1792986993	intron_variant
rs1792987106	intron_variant
rs1792987356	intron_variant
rs1792987476	intron_variant
rs1792987597	intron_variant
rs1792988132	intron_variant
rs1792988494	intron_variant
rs1792988705	intron_variant
rs1792989501	intron_variant
rs1792989672	intron_variant
rs1792990031	intron_variant
rs1792990870	intron_variant
rs1792991051	intron_variant
rs1792991219	intron_variant
rs1792991658	intron_variant
rs1792991767	intron_variant
rs1792992006	intron_variant
rs1792992309	intron_variant

rs1792992407	intron_variant
rs1792992643	intron_variant
rs1792992770	intron_variant
rs1792993019	intron_variant
rs1792993239	intron_variant
rs1792993859	intron_variant
rs1792994952	intron_variant
rs1792995265	intron_variant
rs1792995614	intron_variant
rs1792995722	intron_variant
rs1792995960	intron_variant
rs1792996064	intron_variant
rs1792996496	intron_variant
rs1792996607	intron_variant
rs1792996718	intron_variant
rs1792996937	intron_variant
rs1792997394	intron_variant
rs1792997519	intron_variant
rs1792997625	intron_variant
rs1792998172	intron_variant
rs1792998734	intron_variant
rs1792998941	intron_variant
rs1792999169	intron_variant
rs1793001123	intron_variant
rs1793001232	intron_variant
rs1793001426	intron_variant
rs1793001545	intron_variant
rs1793002074	intron_variant
rs1793002189	intron_variant
rs1793002311	intron_variant
rs1793002849	intron_variant
rs1793003154	intron_variant
rs1793003267	intron_variant
rs1793003372	intron_variant
rs1793003471	intron_variant
rs1793003654	intron_variant
rs1793003773	intron_variant
rs1793004346	intron_variant
rs1793004908	intron_variant
rs1793011439	5_prime_UTR_variant,non_coding_transcript_variant,coding_sequence_variant,missense_variant
rs1793013042	5_prime_UTR_variant,non_coding_transcript_variant,coding_sequence_variant,missense_variant
rs1793018571	non_coding_transcript_variant,coding_sequence_variant,missense_variant
rs1793018682	non_coding_transcript_variant,coding_sequence_variant,missense_variant
rs1793022221	non_coding_transcript_variant,coding_sequence_variant,missense_variant
rs1793022650	intron_variant
rs1793023897	intron_variant
rs1793024398	intron_variant
rs1793025135	intron_variant
rs1793025645	intron_variant
rs1793028822	intron_variant
rs1793028979	intron_variant
rs1793029302	intron_variant
rs1793029896	intron_variant
rs1793030595	intron_variant
rs1793031371	intron_variant
rs1793031763	intron_variant
rs1793032670	intron_variant
rs1793032788	intron_variant
rs1793032968	intron_variant
rs1793033064	intron_variant
rs1793033376	intron_variant
rs1793034274	intron_variant
rs1793034402	intron_variant
rs1793034515	intron_variant
rs1793035301	intron_variant
rs1793035552	intron_variant
rs1793035997	intron_variant
rs1793036473	intron_variant
rs1793036615	intron_variant
rs1793037490	intron_variant
rs1793037832	intron_variant
rs1793038010	intron_variant
rs1793038171	intron_variant
rs1793038907	intron_variant

rs1793039096	intron_variant
rs1793040437	intron_variant
rs1793040543	intron_variant
rs1793040665	intron_variant
rs1793041289	intron_variant
rs1793042245	intron_variant
rs1793042410	intron_variant
rs1793042595	intron_variant
rs1793042926	intron_variant
rs1793043155	intron_variant
rs1793043400	intron_variant
rs1793043615	intron_variant
rs1793043724	intron_variant
rs1793043842	intron_variant
rs1793044347	intron_variant
rs1793044855	intron_variant
rs1793045858	intron_variant
rs1793046379	intron_variant
rs1793046490	intron_variant
rs1793047303	intron_variant
rs1793047521	intron_variant
rs1793047633	intron_variant
rs1793047966	intron_variant
rs1793048092	intron_variant
rs1793048210	intron_variant
rs1793048741	intron_variant
rs1793048857	intron_variant
rs1793048991	intron_variant
rs1793049194	intron_variant
rs1793049314	intron_variant
rs1793049481	intron_variant
rs1793049665	intron_variant
rs1793050087	intron_variant
rs1793050268	intron_variant
rs1793051777	intron_variant
rs1793053054	intron_variant
rs1793053934	intron_variant
rs1793054114	intron_variant
rs1793054278	intron_variant
rs1793054578	intron_variant
rs1793054945	intron_variant
rs1793055116	intron_variant
rs1793055461	intron_variant
rs1793055819	intron_variant
rs1793055979	intron_variant
rs1793056213	intron_variant
rs1793056395	intron_variant
rs1793056740	intron_variant
rs1793056889	intron_variant
rs1793057246	intron_variant
rs1793057424	intron_variant
rs1793057751	intron_variant
rs1793059577	intron_variant
rs1793060122	intron_variant
rs1793060947	intron_variant
rs1793061051	intron_variant
rs1793061711	intron_variant
rs1793062251	intron_variant
rs1793062400	intron_variant
rs1793062759	intron_variant
rs1793063307	intron_variant
rs1793063614	intron_variant
rs1793064341	intron_variant
rs1793065173	intron_variant
rs1793065501	intron_variant
rs1793066515	intron_variant
rs1793066615	intron_variant
rs1793066721	intron_variant
rs1793067019	intron_variant
rs1793067476	intron_variant
rs1793067584	intron_variant
rs1793067888	intron_variant
rs1793068243	intron_variant
rs1793068690	intron_variant

rs1793069924	intron_variant
rs1793070010	intron_variant
rs1793070220	intron_variant
rs1793070313	intron_variant
rs1793070873	intron_variant
rs1793070987	intron_variant
rs1793071303	intron_variant
rs1793071418	intron_variant
rs1793071526	intron_variant
rs1793071854	intron_variant
rs1793071964	intron_variant
rs1793072158	intron_variant
rs1793072245	intron_variant
rs1793072663	intron_variant
rs1793072843	intron_variant
rs1793073157	intron_variant
rs1793073267	intron_variant
rs1793074172	intron_variant
rs1793074336	intron_variant
rs1793074521	intron_variant
rs1793074848	intron_variant
rs1793076774	intron_variant
rs1793077247	intron_variant
rs1793077388	intron_variant
rs1793077693	intron_variant
rs1793077980	intron_variant
rs1793078475	intron_variant
rs1793079456	intron_variant
rs1793079624	intron_variant
rs1793079770	intron_variant
rs1793079930	intron_variant
rs1793080854	intron_variant
rs1793081132	intron_variant
rs1793082056	intron_variant
rs1793082723	intron_variant
rs1793083022	intron_variant
rs1793083529	intron_variant
rs1793084274	intron_variant
rs1793084586	intron_variant
rs1793084916	intron_variant
rs1793085764	intron_variant
rs1793085896	intron_variant
rs1793085989	intron_variant
rs1793086510	intron_variant
rs1793086681	intron_variant
rs1793086825	intron_variant
rs1793087734	intron_variant
rs1793088071	intron_variant
rs1793088242	intron_variant
rs1793088543	intron_variant
rs1793088880	intron_variant
rs1793089401	intron_variant
rs1793089881	intron_variant
rs1793090222	intron_variant
rs1793091510	intron_variant
rs1793091679	intron_variant
rs1793091828	intron_variant
rs1793091986	intron_variant
rs1793092157	intron_variant
rs1793092787	intron_variant
rs1793092926	intron_variant
rs1793093250	intron_variant
rs1793093676	intron_variant
rs1793094071	intron_variant
rs1793094493	intron_variant
rs1793094570	intron_variant
rs1793094788	intron_variant
rs1793095914	intron_variant
rs1793096040	intron_variant
rs1793096148	intron_variant
rs1793096705	intron_variant
rs1793097023	intron_variant
rs1793097309	intron_variant
rs1793097510	intron_variant

rs1793097893	intron_variant
rs1793097998	intron_variant
rs1793098093	intron_variant
rs1793098186	intron_variant
rs1793098490	intron_variant
rs1793098593	intron_variant
rs1793098879	intron_variant
rs1793098983	intron_variant
rs1793099319	intron_variant
rs1793099566	intron_variant
rs1793099668	intron_variant
rs1793099829	intron_variant
rs1793100711	intron_variant
rs1793100882	intron_variant
rs1793101066	intron_variant
rs1793101222	intron_variant
rs1793103028	intron_variant
rs1793103160	intron_variant
rs1793103465	intron_variant
rs1793103802	intron_variant
rs1793104266	intron_variant
rs1793104396	intron_variant
rs1793104615	intron_variant
rs1793104955	intron_variant
rs1793105058	intron_variant
rs1793105144	intron_variant
rs1793105906	intron_variant
rs1793106131	intron_variant
rs1793106226	intron_variant
rs1793106938	intron_variant
rs1793107048	intron_variant
rs1793108088	intron_variant
rs1793108293	intron_variant
rs1793108398	intron_variant
rs1793108958	intron_variant
rs1793109857	intron_variant
rs1793110793	intron_variant
rs1793111353	intron_variant
rs1793112098	intron_variant
rs1793112212	intron_variant
rs1793112621	intron_variant
rs1793113782	intron_variant
rs1793113882	intron_variant
rs1793114668	intron_variant
rs1793114878	intron_variant
rs1793115006	intron_variant
rs1793115124	intron_variant
rs1793115550	intron_variant
rs1793115638	intron_variant
rs1793116041	intron_variant
rs1793116135	intron_variant
rs1793116227	intron_variant
rs1793116536	intron_variant
rs1793116917	intron_variant
rs1793117755	intron_variant
rs1793117964	intron_variant
rs1793118052	intron_variant
rs1793118260	intron_variant
rs1793118883	intron_variant
rs1793118972	intron_variant
rs1793119176	intron_variant
rs1793119494	intron_variant
rs1793119866	intron_variant
rs1793119967	intron_variant
rs1793120227	intron_variant
rs1793120631	intron_variant
rs1793120732	intron_variant
rs1793120844	intron_variant
rs1793121431	intron_variant
rs1793122060	intron_variant
rs1793122154	intron_variant
rs1793122263	intron_variant
rs1793122348	intron_variant
rs1793122461	intron_variant

rs1793122562	intron_variant
rs1793122675	intron_variant
rs1793122783	intron_variant
rs1793122959	intron_variant
rs1793123054	intron_variant
rs1793124126	intron_variant
rs1793124623	intron_variant
rs1793124718	intron_variant
rs1793124842	intron_variant
rs1793125790	intron_variant
rs1793125908	intron_variant
rs1793126341	intron_variant
rs1793126558	intron_variant
rs1793127327	intron_variant
rs1793127448	intron_variant
rs1793129542	intron_variant
rs1793129637	intron_variant
rs1793129729	intron_variant
rs1793130056	intron_variant
rs1793130267	intron_variant
rs1793130595	intron_variant
rs1793131031	intron_variant
rs1793131528	intron_variant
rs1793132129	intron_variant
rs1793132299	intron_variant
rs1793132456	intron_variant
rs1793132632	intron_variant
rs1793132773	intron_variant
rs1793133055	intron_variant
rs1793133211	intron_variant
rs1793133380	intron_variant
rs1793133547	intron_variant
rs1793133682	intron_variant
rs1793133860	intron_variant
rs1793133962	intron_variant
rs1793134363	intron_variant
rs1793134676	intron_variant
rs1793134778	intron_variant
rs1793135260	intron_variant
rs1793136058	intron_variant
rs1793136397	intron_variant
rs1793137032	intron_variant
rs1793137453	intron_variant
rs1793137707	intron_variant
rs1793137809	intron_variant
rs1793138115	intron_variant
rs1793138232	intron_variant
rs1793138537	intron_variant
rs1793138634	intron_variant
rs1793138831	intron_variant
rs1793139140	intron_variant
rs1793140093	intron_variant
rs1793140961	intron_variant
rs1793141141	intron_variant
rs1793141338	intron_variant
rs1793141833	intron_variant
rs1793142091	intron_variant
rs1793142410	intron_variant
rs1793142497	intron_variant
rs1793142712	intron_variant
rs1793143048	intron_variant
rs1793143541	intron_variant
rs1793143643	intron_variant
rs1793143757	intron_variant
rs1793143838	intron_variant
rs1793143941	intron_variant
rs1793144596	intron_variant
rs1793145517	intron_variant
rs1793145854	intron_variant
rs1793145942	intron_variant
rs1793146220	intron_variant
rs1793146332	intron_variant
rs1793146751	intron_variant
rs1793146852	intron_variant

rs1793147162	intron_variant
rs1793147247	intron_variant
rs1793147358	intron_variant
rs1793147761	intron_variant
rs1793147847	intron_variant
rs1793147944	intron_variant
rs1793148138	intron_variant
rs1793148900	intron_variant
rs1793149488	intron_variant
rs1793149697	intron_variant
rs1793150944	intron_variant
rs1793151082	intron_variant
rs1793151761	intron_variant
rs1793152328	intron_variant
rs1793152412	intron_variant
rs1793152728	intron_variant
rs1793152919	intron_variant
rs1793153182	intron_variant
rs1793154178	intron_variant
rs1793154268	intron_variant
rs1793154546	intron_variant
rs1793154654	intron_variant
rs1793155085	intron_variant
rs1793155285	intron_variant
rs1793155608	intron_variant
rs1793155817	intron_variant
rs1793156577	intron_variant
rs1793156786	intron_variant
rs1793156888	intron_variant
rs1793157235	intron_variant
rs1793157328	intron_variant
rs1793157429	intron_variant
rs1793157536	intron_variant
rs1793158115	intron_variant
rs1793158420	intron_variant
rs1793158523	intron_variant
rs1793159092	intron_variant
rs1793159193	intron_variant
rs1793159282	intron_variant
rs1793159640	intron_variant
rs1793160277	intron_variant
rs1793160768	intron_variant
rs1793160888	intron_variant
rs1793161135	intron_variant
rs1793161477	intron_variant
rs1793161868	intron_variant
rs1793162143	intron_variant
rs1793162572	intron_variant
rs1793163218	intron_variant
rs1793165724	intron_variant
rs1793166062	intron_variant
rs1793166359	intron_variant
rs1793166444	intron_variant
rs1793166658	intron_variant
rs1793166885	intron_variant
rs1793167397	intron_variant
rs1793167477	intron_variant
rs1793167572	intron_variant
rs1793169344	intron_variant
rs1793170176	intron_variant
rs1793170552	intron_variant
rs1793170741	intron_variant
rs1793170910	intron_variant
rs1793171042	intron_variant
rs1793171152	intron_variant
rs1793171573	intron_variant
rs1793172119	intron_variant
rs1793172223	intron_variant
rs1793172423	intron_variant
rs1793172527	intron_variant
rs1793172620	intron_variant
rs1793172834	intron_variant
rs1793172934	intron_variant
rs1793173054	intron_variant

rs1793173632	intron_variant
rs1793173846	intron_variant
rs1793174268	intron_variant
rs1793174509	intron_variant
rs1793174837	intron_variant
rs1793174959	intron_variant
rs1793175254	intron_variant
rs1793175868	intron_variant
rs1793175974	intron_variant
rs1793176271	intron_variant
rs1793176373	intron_variant
rs1793176468	intron_variant
rs1793176795	intron_variant
rs1793177135	intron_variant
rs1793177352	intron_variant
rs1793177893	intron_variant
rs1793178124	intron_variant
rs1793179071	intron_variant
rs1793179374	intron_variant
rs1793179460	intron_variant
rs1793179557	intron_variant
rs1793179646	intron_variant
rs1793179858	intron_variant
rs1793179958	intron_variant
rs1793180057	intron_variant
rs1793181035	intron_variant
rs1793181144	intron_variant
rs1793181230	intron_variant
rs1793181340	intron_variant
rs1793181589	intron_variant
rs1793182550	intron_variant
rs1793182761	intron_variant
rs1793182858	intron_variant
rs1793182944	intron_variant
rs1793183042	intron_variant
rs1793183362	intron_variant
rs1793183463	intron_variant
rs1793183893	intron_variant
rs1793184118	intron_variant
rs1793184231	intron_variant
rs1793184777	intron_variant
rs1793184872	intron_variant
rs1793184980	intron_variant
rs1793185086	intron_variant
rs1793185189	intron_variant
rs1793185705	intron_variant
rs1793185944	intron_variant
rs1793186147	intron_variant
rs1793186552	intron_variant
rs1793186662	intron_variant
rs1793186802	intron_variant
rs1793187002	intron_variant
rs1793187243	intron_variant
rs1793187646	intron_variant
rs1793188122	intron_variant
rs1793188276	intron_variant
rs1793189289	intron_variant
rs1793191569	intron_variant
rs1793191630	intron_variant
rs1793192079	intron_variant
rs1793192388	intron_variant
rs1793192674	intron_variant
rs1793193213	intron_variant
rs1793193346	intron_variant
rs1793193557	intron_variant
rs1793194651	intron_variant
rs1793194841	intron_variant
rs1793195040	intron_variant
rs1793195616	intron_variant
rs1793195945	intron_variant
rs1793196055	intron_variant
rs1793196517	intron_variant
rs1793197587	intron_variant
rs1793197810	intron_variant

rs1793197924	intron_variant
rs1793198947	intron_variant
rs1793199047	intron_variant
rs1793199148	intron_variant
rs1793199240	intron_variant
rs1793199819	intron_variant
rs1793200356	intron_variant
rs1793201042	intron_variant
rs1793201362	intron_variant
rs1793201730	intron_variant
rs1793201901	intron_variant
rs1793202209	intron_variant
rs1793202303	intron_variant
rs1793202411	intron_variant
rs1793202772	intron_variant
rs1793203072	intron_variant
rs1793203162	intron_variant
rs1793203381	intron_variant
rs1793203485	intron_variant
rs1793203862	intron_variant
rs1793204527	intron_variant
rs1793204608	intron_variant
rs1793204705	intron_variant
rs1793204806	intron_variant
rs1793204893	intron_variant
rs1793204989	intron_variant
rs1793205277	intron_variant
rs1793205549	intron_variant
rs1793205769	intron_variant
rs1793205969	intron_variant
rs1793206336	intron_variant
rs1793206421	intron_variant
rs1793207273	intron_variant
rs1793207464	intron_variant
rs1793207556	intron_variant
rs1793207659	intron_variant
rs1793207984	intron_variant
rs1793208839	intron_variant
rs1793209066	intron_variant
rs1793209256	intron_variant
rs1793209385	intron_variant
rs1793209484	intron_variant
rs1793209668	intron_variant
rs1793209961	intron_variant
rs1793210470	intron_variant
rs1793210550	intron_variant
rs1793210694	intron_variant
rs1793210889	intron_variant
rs1793211319	intron_variant
rs1793211878	intron_variant
rs1793211974	intron_variant
rs1793212063	intron_variant
rs1793212409	intron_variant
rs1793212690	intron_variant
rs1793212977	intron_variant
rs1793213067	intron_variant
rs1793213161	intron_variant
rs1793213464	intron_variant
rs1793213753	intron_variant
rs1793213933	intron_variant
rs1793214012	intron_variant
rs1793214308	intron_variant
rs1793214626	intron_variant
rs1793215323	intron_variant
rs1793215533	intron_variant
rs1793215739	intron_variant
rs1793216061	intron_variant
rs1793216398	intron_variant
rs1793216788	intron_variant
rs1793217129	intron_variant
rs1793217901	intron_variant
rs1793218014	intron_variant
rs1793219002	intron_variant
rs1793219168	intron_variant

rs1793219502	intron_variant
rs1793219638	intron_variant
rs1793219965	intron_variant
rs1793220121	intron_variant
rs1793220503	intron_variant
rs1793221291	intron_variant
rs1793221453	intron_variant
rs1793221621	intron_variant
rs1793221788	intron_variant
rs1793221937	intron_variant
rs1793223305	intron_variant
rs1793223619	intron_variant
rs1793224264	intron_variant
rs1793224771	intron_variant
rs1793225111	intron_variant
rs1793225758	intron_variant
rs1793226642	intron_variant
rs1793227300	intron_variant
rs1793227626	intron_variant
rs1793227930	intron_variant
rs1793228231	intron_variant
rs1793228401	intron_variant
rs1793228754	intron_variant
rs1793228919	intron_variant
rs1793230020	intron_variant
rs1793230179	intron_variant
rs1793230357	intron_variant
rs1793231200	intron_variant
rs1793231509	intron_variant
rs1793232189	intron_variant
rs1793232492	intron_variant
rs1793233709	intron_variant
rs1793234044	intron_variant
rs1793234181	intron_variant
rs1793235192	intron_variant
rs1793235679	intron_variant
rs1793235785	intron_variant
rs1793236343	intron_variant
rs1793237066	intron_variant
rs1793237169	intron_variant
rs1793237277	intron_variant
rs1793237609	intron_variant
rs1793237903	intron_variant
rs1793238013	intron_variant
rs1793238293	intron_variant
rs1793238712	intron_variant
rs1793238840	intron_variant
rs1793239445	intron_variant
rs1793239625	intron_variant
rs1793239996	intron_variant
rs1793240081	intron_variant
rs1793240209	intron_variant
rs1793240371	intron_variant
rs1793241012	intron_variant
rs1793241136	intron_variant
rs1793241300	intron_variant
rs1793241449	intron_variant
rs1793242131	intron_variant
rs1793242220	intron_variant
rs1793242302	intron_variant
rs1793242470	intron_variant
rs1793242641	intron_variant
rs1793243156	intron_variant
rs1793243622	intron_variant
rs1793244209	intron_variant
rs1793244346	intron_variant
rs1793244632	intron_variant
rs1793246189	intron_variant
rs1793246325	intron_variant
rs1793246598	intron_variant
rs1793246759	intron_variant
rs1793247207	intron_variant
rs1793247334	intron_variant
rs1793248173	intron_variant

rs1793248490	intron_variant
rs1793248990	intron_variant
rs1793249611	intron_variant
rs1793249929	intron_variant
rs1793250090	intron_variant
rs1793250558	intron_variant
rs1793250864	intron_variant
rs1793251408	intron_variant
rs1793251620	intron_variant
rs1793251969	intron_variant
rs1793252513	intron_variant
rs1793252678	intron_variant
rs1793253318	intron_variant
rs1793253471	intron_variant
rs1793253836	intron_variant
rs1793254314	intron_variant
rs1793256449	intron_variant
rs1793257457	intron_variant
rs1793257732	intron_variant
rs1793258041	intron_variant
rs1793258207	intron_variant
rs1793258548	intron_variant
rs1793259388	intron_variant
rs1793260091	intron_variant
rs1793260551	intron_variant
rs1793260692	intron_variant
rs1793262043	intron_variant
rs1793262397	intron_variant
rs1793262501	intron_variant
rs1793262601	intron_variant
rs1793262839	intron_variant
rs1793264478	intron_variant
rs1793265173	intron_variant
rs1793266341	intron_variant
rs1793266951	intron_variant
rs1793267046	intron_variant
rs1793267158	intron_variant
rs1793267297	intron_variant
rs1793267545	intron_variant
rs1793268055	intron_variant
rs1793269347	intron_variant
rs1793269722	intron_variant
rs1793269904	intron_variant
rs1793270074	intron_variant
rs1793270253	intron_variant
rs1793272827	intron_variant
rs1793273269	intron_variant
rs1793273753	intron_variant
rs1793274288	intron_variant
rs1793275957	intron_variant
rs1793276140	intron_variant
rs1793276313	intron_variant
rs1793276644	intron_variant
rs1793276802	intron_variant
rs1793277403	intron_variant
rs1793278999	intron_variant
rs1793279326	intron_variant
rs1793280408	intron_variant
rs1793281288	intron_variant
rs1793281651	intron_variant
rs1793282367	intron_variant
rs1793282724	intron_variant
rs1793283054	intron_variant
rs1793283538	intron_variant
rs1793283674	intron_variant
rs1793284016	intron_variant
rs1793284240	intron_variant
rs1793284344	intron_variant
rs1793284898	intron_variant
rs1793285502	intron_variant
rs1793287287	intron_variant
rs1793288011	intron_variant
rs1793289837	intron_variant
rs1793290016	intron_variant

rs1793290236	intron_variant
rs1793291137	intron_variant
rs1793291465	intron_variant
rs1793291693	intron_variant
rs1793291821	intron_variant
rs1793292062	intron_variant
rs1793292279	intron_variant
rs1793292372	intron_variant
rs1793292575	intron_variant
rs1793292669	intron_variant
rs1793292989	intron_variant
rs1793293393	intron_variant
rs1793294405	intron_variant
rs1793294721	intron_variant
rs1793295088	intron_variant
rs1793295406	intron_variant
rs1793296144	intron_variant
rs1793296328	intron_variant
rs1793296956	intron_variant
rs1793297078	intron_variant
rs1793297219	intron_variant
rs1793297360	intron_variant
rs1793297733	intron_variant
rs1793298421	intron_variant
rs1793298985	intron_variant
rs1793299273	intron_variant
rs1793299484	intron_variant
rs1793299688	intron_variant
rs1793299794	intron_variant
rs1793299898	intron_variant
rs1793300238	intron_variant
rs1793300473	intron_variant
rs1793300602	intron_variant
rs1793300702	intron_variant
rs1793300798	intron_variant
rs1793301015	intron_variant
rs1793301188	intron_variant
rs1793301707	intron_variant
rs1793301877	intron_variant
rs1793303387	intron_variant
rs1793303862	intron_variant
rs1793311164	intron_variant
rs1793311356	intron_variant
rs1793311788	intron_variant
rs1793313050	intron_variant
rs1793313385	intron_variant
rs1793313568	intron_variant
rs1793313956	intron_variant
rs1793314333	intron_variant
rs1793314451	intron_variant
rs1793314687	intron_variant
rs1793315175	intron_variant
rs1793315282	intron_variant
rs1793316274	intron_variant
rs1793316361	intron_variant
rs1793316610	intron_variant
rs1793316773	intron_variant
rs1793317166	intron_variant
rs1793317665	intron_variant
rs1793318010	intron_variant
rs1793318902	intron_variant
rs1793319293	intron_variant
rs1793320317	intron_variant
rs1793320651	intron_variant
rs1793320771	intron_variant
rs1793320908	intron_variant
rs1793321407	intron_variant
rs1793321747	intron_variant
rs1793321915	intron_variant
rs1793322454	intron_variant
rs1793322619	intron_variant
rs1793323438	intron_variant
rs1793323774	intron_variant
rs1793324092	intron_variant

rs1793324240	intron_variant
rs1793324392	intron_variant
rs1793324882	intron_variant
rs1793325057	intron_variant
rs1793325397	intron_variant
rs1793325749	intron_variant
rs1793326529	intron_variant
rs1793327246	intron_variant
rs1793327403	intron_variant
rs1793328624	intron_variant
rs1793328792	intron_variant
rs1793329315	intron_variant
rs1793329993	intron_variant
rs1793331348	intron_variant
rs1793331989	intron_variant
rs1793332323	intron_variant
rs1793332480	intron_variant
rs1793332664	intron_variant
rs1793332827	intron_variant
rs1793333203	intron_variant
rs1793335672	intron_variant
rs1793336213	intron_variant
rs1793336670	intron_variant
rs1793337413	intron_variant
rs1793337755	intron_variant
rs1793338258	intron_variant
rs1793338422	intron_variant
rs1793338586	intron_variant
rs1793339449	intron_variant
rs1793339625	intron_variant
rs1793340450	intron_variant
rs1793341015	intron_variant
rs1793341550	intron_variant
rs1793341724	intron_variant
rs1793341891	intron_variant
rs1793342593	intron_variant
rs1793342689	intron_variant
rs1793342845	intron_variant
rs1793342967	intron_variant
rs1793343135	intron_variant
rs1793343638	intron_variant
rs1793343748	intron_variant
rs1793344399	intron_variant
rs1793344623	intron_variant
rs1793344728	intron_variant
rs1793344829	intron_variant
rs1793346006	intron_variant
rs1793346812	intron_variant
rs1793346916	intron_variant
rs1793347034	intron_variant
rs1793347391	intron_variant
rs1793347592	intron_variant
rs1793347802	intron_variant
rs1793348086	intron_variant
rs1793348184	intron_variant
rs1793348378	intron_variant
rs1793348489	intron_variant
rs1793348943	intron_variant
rs1793349040	intron_variant
rs1793349756	intron_variant
rs1793349863	intron_variant
rs1793349962	intron_variant
rs1793350150	intron_variant
rs1793350538	intron_variant
rs1793351454	intron_variant
rs1793351911	intron_variant
rs1793352340	intron_variant
rs1793352588	intron_variant
rs1793352802	intron_variant
rs1793352905	intron_variant
rs1793353039	intron_variant
rs1793353766	intron_variant
rs1793353967	intron_variant
rs1793354830	intron_variant

rs1793355031	intron_variant
rs1793355422	intron_variant
rs1793355517	intron_variant
rs1793355598	intron_variant
rs1793355992	intron_variant
rs1793356194	intron_variant
rs1793356700	intron_variant
rs1793357039	intron_variant
rs1793357138	intron_variant
rs1793357339	intron_variant
rs1793358083	intron_variant
rs1793358186	intron_variant
rs1793358391	intron_variant
rs1793358487	intron_variant
rs1793358582	intron_variant
rs1793358778	intron_variant
rs1793359543	intron_variant
rs1793359810	intron_variant
rs1793360555	intron_variant
rs1793360883	intron_variant
rs1793361015	intron_variant
rs1793361588	intron_variant
rs1793362566	intron_variant
rs1793364401	intron_variant
rs1793364507	intron_variant
rs1793364737	intron_variant
rs1793365015	intron_variant
rs1793366150	intron_variant
rs1793366347	intron_variant
rs1793366627	intron_variant
rs1793366724	intron_variant
rs1793366822	intron_variant
rs1793366936	intron_variant
rs1793367190	intron_variant
rs1793367425	intron_variant
rs1793367675	intron_variant
rs1793367771	intron_variant
rs1793368399	intron_variant
rs1793368866	intron_variant
rs1793369093	intron_variant
rs1793369223	intron_variant
rs1793369380	intron_variant
rs1793370288	intron_variant
rs1793370992	intron_variant
rs1793371323	intron_variant
rs1793371649	intron_variant
rs1793372229	intron_variant
rs1793372385	intron_variant
rs1793372534	intron_variant
rs1793373294	intron_variant
rs1793373485	intron_variant
rs1793373596	intron_variant
rs1793373759	intron_variant
rs1793374549	intron_variant
rs1793374649	intron_variant
rs1793375186	intron_variant
rs1793375292	intron_variant
rs1793375404	intron_variant
rs1793378689	non_coding_transcript_variant,coding_sequence_variant,missense_variant
rs1793379665	non_coding_transcript_variant,coding_sequence_variant,missense_variant
rs1793379901	synonymous_variant,non_coding_transcript_variant,coding_sequence_variant
rs1793380314	stop_gained,non_coding_transcript_variant,coding_sequence_variant
rs1793382118	non_coding_transcript_variant,coding_sequence_variant,missense_variant
rs1793383550	synonymous_variant,non_coding_transcript_variant,coding_sequence_variant
rs1793386405	non_coding_transcript_variant,coding_sequence_variant,missense_variant
rs1793386951	intron_variant
rs1793387320	intron_variant
rs1793388710	intron_variant
rs1793389661	intron_variant
rs1793389859	intron_variant
rs1793390211	intron_variant
rs1793390892	intron_variant
rs1793391307	intron_variant
rs1793391740	intron_variant

rs1793392185	intron_variant
rs1793392387	intron_variant
rs1793392503	intron_variant
rs1793392715	intron_variant
rs1793393027	intron_variant
rs1793393794	intron_variant
rs1793394829	intron_variant
rs1793394941	intron_variant
rs1793395050	intron_variant
rs1793395265	intron_variant
rs1793395792	intron_variant
rs1793395882	intron_variant
rs1793396086	intron_variant
rs1793397079	intron_variant
rs1793397273	intron_variant
rs1793397750	intron_variant
rs1793399087	intron_variant
rs1793399419	intron_variant
rs1793400059	intron_variant
rs1793400381	intron_variant
rs1793401182	intron_variant
rs1793401283	intron_variant
rs1793401392	intron_variant
rs1793401604	intron_variant
rs1793403146	intron_variant
rs1793403352	intron_variant
rs1793404010	intron_variant
rs1793404123	intron_variant
rs1793404309	intron_variant
rs1793404469	intron_variant
rs1793404599	intron_variant
rs1793404715	intron_variant
rs1793404949	intron_variant
rs1793405065	intron_variant
rs1793405264	intron_variant
rs1793405467	intron_variant
rs1793405677	intron_variant
rs1793406030	intron_variant
rs1793406115	intron_variant
rs1793406226	intron_variant
rs1793406506	intron_variant
rs1793406607	intron_variant
rs1793406978	intron_variant
rs1793407286	intron_variant
rs1793407396	intron_variant
rs1793407599	intron_variant
rs1793408044	intron_variant
rs1793408296	intron_variant
rs1793408596	intron_variant
rs1793408697	intron_variant
rs1793409252	intron_variant
rs1793409666	intron_variant
rs1793409810	intron_variant
rs1793410267	intron_variant
rs1793410812	intron_variant
rs1793411050	intron_variant
rs1793411650	intron_variant
rs1793411906	intron_variant
rs1793412085	intron_variant
rs1793412194	intron_variant
rs1793412298	intron_variant
rs1793412602	intron_variant
rs1793414330	intron_variant
rs1793415550	intron_variant
rs1793415901	intron_variant
rs1793415997	intron_variant
rs1793416195	intron_variant
rs1793416384	intron_variant
rs1793416503	intron_variant
rs1793417127	intron_variant
rs1793417875	non_coding_transcript_variant,coding_sequence_variant,missense_variant
rs1793418268	frameshift_variant,non_coding_transcript_variant,coding_sequence_variant
rs1793418893	non_coding_transcript_variant,coding_sequence_variant,missense_variant
rs1793420750	non_coding_transcript_variant,coding_sequence_variant,missense_variant

rs1793421740	non_coding_transcript_variant,coding_sequence_variant,missense_variant
rs1793422861	stop_gained,non_coding_transcript_variant,coding_sequence_variant
rs1793429138	non_coding_transcript_variant,coding_sequence_variant,missense_variant
rs1793429447	splice_donor_variant
rs1793431001	intron_variant
rs1793431467	intron_variant
rs1793432087	intron_variant
rs1793432617	intron_variant
rs1793433395	intron_variant
rs1793434714	intron_variant
rs1793434810	intron_variant
rs1793435799	intron_variant
rs1793436087	intron_variant
rs1793438424	intron_variant
rs1793438871	intron_variant
rs1793439766	intron_variant
rs1793439953	intron_variant
rs1793440048	intron_variant
rs1793440390	intron_variant
rs1793440482	intron_variant
rs1793440573	intron_variant
rs1793440674	intron_variant
rs1793441066	intron_variant
rs1793442008	intron_variant
rs1793443673	intron_variant
rs1793443772	intron_variant
rs1793443956	intron_variant
rs1793444324	intron_variant
rs1793444527	intron_variant
rs1793444625	intron_variant
rs1793444714	intron_variant
rs1793444917	intron_variant
rs1793445364	intron_variant
rs1793446495	intron_variant
rs1793446633	intron_variant
rs1793446849	intron_variant
rs1793447059	intron_variant
rs1793448491	intron_variant
rs1793448640	intron_variant
rs1793448792	intron_variant
rs1793448948	intron_variant
rs1793449118	intron_variant
rs1793449403	intron_variant
rs1793449568	intron_variant
rs1793449716	intron_variant
rs1793450021	intron_variant
rs1793450115	intron_variant
rs1793450200	intron_variant
rs1793450874	intron_variant
rs1793452535	intron_variant
rs1793452630	intron_variant
rs1793452974	intron_variant
rs1793453066	intron_variant
rs1793453279	intron_variant
rs1793453531	intron_variant
rs1793453866	intron_variant
rs1793453973	intron_variant
rs1793454365	intron_variant
rs1793455187	intron_variant
rs1793455295	intron_variant
rs1793456334	intron_variant
rs1793456524	intron_variant
rs1793456728	intron_variant
rs1793457222	intron_variant
rs1793458177	intron_variant
rs1793458498	intron_variant
rs1793458973	intron_variant
rs1793459071	intron_variant
rs1793459421	intron_variant
rs1793459737	intron_variant
rs1793459873	intron_variant
rs1793460214	intron_variant
rs1793461272	intron_variant
rs1793461723	intron_variant

rs1793462031	intron_variant
rs1793463531	intron_variant
rs1793463818	intron_variant
rs1793463926	intron_variant
rs1793464205	intron_variant
rs1793464994	intron_variant
rs1793465699	intron_variant
rs1793466014	intron_variant
rs1793466670	intron_variant
rs1793467141	intron_variant
rs1793468879	intron_variant
rs1793469370	intron_variant
rs1793470264	intron_variant
rs1793470607	intron_variant
rs1793470715	intron_variant
rs1793470925	intron_variant
rs1793471154	intron_variant
rs1793471452	intron_variant
rs1793471554	intron_variant
rs1793471774	intron_variant
rs1793471866	intron_variant
rs1793472264	intron_variant
rs1793473989	intron_variant
rs1793474159	intron_variant
rs1793474520	intron_variant
rs1793474949	intron_variant
rs1793475642	intron_variant
rs1793475746	intron_variant
rs1793475818	intron_variant
rs1793476024	intron_variant
rs1793476543	intron_variant
rs1793476662	intron_variant
rs1793476908	intron_variant
rs1793477069	intron_variant
rs1793477806	intron_variant
rs1793477953	intron_variant
rs1793478108	intron_variant
rs1793478249	intron_variant
rs1793478806	intron_variant
rs1793480109	intron_variant
rs1793480699	intron_variant
rs1793481499	intron_variant
rs1793481596	intron_variant
rs1793481698	intron_variant
rs1793481896	intron_variant
rs1793481997	intron_variant
rs1793482193	intron_variant
rs1793482312	intron_variant
rs1793482432	intron_variant
rs1793483054	intron_variant
rs1793483141	intron_variant
rs1793483329	intron_variant
rs1793483522	intron_variant
rs1793483611	intron_variant
rs1793484223	intron_variant
rs1793484641	intron_variant
rs1793485103	intron_variant
rs1793485694	intron_variant
rs1793486753	intron_variant
rs1793487190	intron_variant
rs1793487346	intron_variant
rs1793487996	intron_variant
rs1793488273	intron_variant
rs1793488630	intron_variant
rs1793489274	intron_variant
rs1793489429	intron_variant
rs1793489750	intron_variant
rs1793489916	intron_variant
rs1793490278	intron_variant
rs1793490770	intron_variant
rs1793491246	intron_variant
rs1793491612	intron_variant
rs1793491774	intron_variant
rs1793492127	intron_variant

rs1793492592	intron_variant
rs1793492761	intron_variant
rs1793493340	intron_variant
rs1793493950	intron_variant
rs1793494459	intron_variant
rs1793494616	intron_variant
rs1793494946	intron_variant
rs1793495114	intron_variant
rs1793495457	intron_variant
rs1793495651	intron_variant
rs1793495801	intron_variant
rs1793495958	intron_variant
rs1793496109	intron_variant
rs1793497197	intron_variant
rs1793498311	intron_variant
rs1793498497	intron_variant
rs1793499053	intron_variant
rs1793499379	intron_variant
rs1793499766	intron_variant
rs1793499978	intron_variant
rs1793500248	intron_variant
rs1793500476	intron_variant
rs1793500928	intron_variant
rs1793501690	intron_variant
rs1793502333	intron_variant
rs1793502678	intron_variant
rs1793502843	intron_variant
rs1793503600	intron_variant
rs1793503781	intron_variant
rs1793503956	intron_variant
rs1793504466	intron_variant
rs1793504923	intron_variant
rs1793505141	intron_variant
rs1793505229	intron_variant
rs1793505346	intron_variant
rs1793505844	intron_variant
rs1793506160	intron_variant
rs1793506259	intron_variant
rs1793506978	intron_variant
rs1793507141	intron_variant
rs1793507238	intron_variant
rs1793507339	intron_variant
rs1793507570	intron_variant
rs1793507663	intron_variant
rs1793507885	intron_variant
rs1793508423	intron_variant
rs1793508539	intron_variant
rs1793508645	intron_variant
rs1793508800	intron_variant
rs1793510002	intron_variant
rs1793510336	intron_variant
rs1793510499	intron_variant
rs1793510927	intron_variant
rs1793511080	intron_variant
rs1793511377	intron_variant
rs1793511740	intron_variant
rs1793512182	intron_variant
rs1793513313	intron_variant
rs1793513468	intron_variant
rs1793513921	intron_variant
rs1793514481	intron_variant
rs1793514783	intron_variant
rs1793515126	intron_variant
rs1793515955	intron_variant
rs1793516087	intron_variant
rs1793516741	intron_variant
rs1793517112	intron_variant
rs1793517753	intron_variant
rs1793517913	intron_variant
rs1793518377	intron_variant
rs1793518880	intron_variant
rs1793519035	intron_variant
rs1793519563	intron_variant
rs1793519881	intron_variant

rs1793520261	intron_variant
rs1793520419	intron_variant
rs1793521010	intron_variant
rs1793521175	intron_variant
rs1793521573	intron_variant
rs1793521899	intron_variant
rs1793522085	intron_variant
rs1793522166	intron_variant
rs1793522663	intron_variant
rs1793522756	intron_variant
rs1793522857	intron_variant
rs1793523059	intron_variant
rs1793523749	intron_variant
rs1793523855	intron_variant
rs1793525002	intron_variant
rs1793525249	intron_variant
rs1793525471	intron_variant
rs1793526402	intron_variant
rs1793526506	intron_variant
rs1793526599	intron_variant
rs1793526957	intron_variant
rs1793527061	intron_variant
rs1793527382	intron_variant
rs1793527493	intron_variant
rs1793527594	intron_variant
rs1793527670	intron_variant
rs1793527854	intron_variant
rs1793527942	intron_variant
rs1793528149	intron_variant
rs1793528428	intron_variant
rs1793528536	intron_variant
rs1793529489	intron_variant
rs1793529591	intron_variant
rs1793529725	intron_variant
rs1793529812	intron_variant
rs1793530197	intron_variant
rs1793531262	intron_variant
rs1793531569	intron_variant
rs1793531667	intron_variant
rs1793532032	intron_variant
rs1793534158	intron_variant
rs1793534572	intron_variant
rs1793534688	intron_variant
rs1793535006	intron_variant
rs1793535487	intron_variant
rs1793535572	intron_variant
rs1793535810	intron_variant
rs1793536036	intron_variant
rs1793536796	intron_variant
rs1793537595	intron_variant
rs1793537676	intron_variant
rs1793538017	intron_variant
rs1793538513	intron_variant
rs1793538610	intron_variant
rs1793538709	intron_variant
rs1793539127	intron_variant
rs1793539470	intron_variant
rs1793539662	intron_variant
rs1793540442	intron_variant
rs1793540830	intron_variant
rs1793540925	intron_variant
rs1793541219	intron_variant
rs1793541295	intron_variant
rs1793541383	intron_variant
rs1793541488	intron_variant
rs1793541711	intron_variant
rs1793541910	intron_variant
rs1793542371	intron_variant
rs1793542480	intron_variant
rs1793542585	intron_variant
rs1793542817	intron_variant
rs1793543041	intron_variant
rs1793543488	intron_variant
rs1793543945	intron_variant

rs1793544022	intron_variant
rs1793544379	intron_variant
rs1793544564	intron_variant
rs1793544666	intron_variant
rs1793544773	intron_variant
rs1793545403	intron_variant
rs1793545796	intron_variant
rs1793546206	intron_variant
rs1793546313	intron_variant
rs1793546713	intron_variant
rs1793547045	intron_variant
rs1793547264	intron_variant
rs1793547545	intron_variant
rs1793547936	intron_variant
rs1793548158	intron_variant
rs1793548257	intron_variant
rs1793548461	intron_variant
rs1793550108	intron_variant
rs1793550428	intron_variant
rs1793550524	intron_variant
rs1793550854	intron_variant
rs1793551078	intron_variant
rs1793551187	intron_variant
rs1793551925	intron_variant
rs1793552459	intron_variant
rs1793553621	intron_variant
rs1793553847	intron_variant
rs1793554885	intron_variant
rs1793555294	intron_variant
rs1793555714	intron_variant
rs1793555910	intron_variant
rs1793556396	intron_variant
rs1793556592	intron_variant
rs1793557010	intron_variant
rs1793557110	intron_variant
rs1793557225	intron_variant
rs1793557327	intron_variant
rs1793557429	intron_variant
rs1793557808	intron_variant
rs1793558070	intron_variant
rs1793558525	intron_variant
rs1793558672	intron_variant
rs1793558773	intron_variant
rs1793559817	intron_variant
rs1793559908	intron_variant
rs1793560229	intron_variant
rs1793560517	intron_variant
rs1793560627	intron_variant
rs1793561491	intron_variant
rs1793561796	intron_variant
rs1793562028	intron_variant
rs1793562209	intron_variant
rs1793562865	intron_variant
rs1793563060	intron_variant
rs1793563157	intron_variant
rs1793563965	intron_variant
rs1793564533	intron_variant
rs1793564725	intron_variant
rs1793565461	intron_variant
rs1793565901	intron_variant
rs1793566438	intron_variant
rs1793566860	intron_variant
rs1793566957	intron_variant
rs1793567282	intron_variant
rs1793567483	intron_variant
rs1793567675	intron_variant
rs1793567778	intron_variant
rs1793567876	intron_variant
rs1793568091	intron_variant
rs1793568557	intron_variant
rs1793568932	intron_variant
rs1793569149	intron_variant
rs1793569359	intron_variant
rs1793569470	intron_variant

rs1793569580	intron_variant
rs1793569973	intron_variant
rs1793570392	intron_variant
rs1793570495	intron_variant
rs1793570647	intron_variant
rs1793570799	intron_variant
rs1793571795	intron_variant
rs1793572106	intron_variant
rs1793572452	intron_variant
rs1793572616	intron_variant
rs1793572756	intron_variant
rs1793572934	intron_variant
rs1793573079	intron_variant
rs1793573240	intron_variant
rs1793573380	intron_variant
rs1793574055	intron_variant
rs1793574738	intron_variant
rs1793575490	intron_variant
rs1793576152	intron_variant
rs1793576295	intron_variant
rs1793576451	intron_variant
rs1793577725	intron_variant
rs1793578066	intron_variant
rs1793578357	intron_variant
rs1793578512	intron_variant
rs1793578704	intron_variant
rs1793578802	intron_variant
rs1793579538	intron_variant
rs1793579807	intron_variant
rs1793580005	intron_variant
rs1793580810	intron_variant
rs1793581336	intron_variant
rs1793581715	intron_variant
rs1793582157	intron_variant
rs1793582371	intron_variant
rs1793583308	intron_variant
rs1793584495	intron_variant
rs1793584599	intron_variant
rs1793584699	intron_variant
rs1793584833	intron_variant
rs1793584926	intron_variant
rs1793585032	intron_variant
rs1793585584	intron_variant
rs1793585793	intron_variant
rs1793586335	intron_variant
rs1793586484	intron_variant
rs1793587074	intron_variant
rs1793587251	intron_variant
rs1793587428	intron_variant
rs1793588163	intron_variant
rs1793588826	intron_variant
rs1793589021	intron_variant
rs1793589131	intron_variant
rs1793589439	intron_variant
rs1793589743	intron_variant
rs1793589911	intron_variant
rs1793590933	intron_variant
rs1793591084	intron_variant
rs1793591585	intron_variant
rs1793593735	intron_variant
rs1793594100	intron_variant
rs1793594309	intron_variant
rs1793594777	intron_variant
rs1793595160	intron_variant
rs1793595323	intron_variant
rs1793595535	intron_variant
rs1793595859	intron_variant
rs1793595988	intron_variant
rs1793596331	intron_variant
rs1793596644	intron_variant
rs1793596803	intron_variant
rs1793596942	intron_variant
rs1793597067	intron_variant
rs1793597739	intron_variant

rs1793597903	intron_variant
rs1793598263	intron_variant
rs1793599384	intron_variant
rs1793599551	intron_variant
rs1793600427	intron_variant
rs1793600604	intron_variant
rs1793600779	intron_variant
rs1793600932	intron_variant
rs1793601090	intron_variant
rs1793601237	intron_variant
rs1793601729	intron_variant
rs1793601914	intron_variant
rs1793602454	intron_variant
rs1793602957	intron_variant
rs1793603598	intron_variant
rs1793604573	intron_variant
rs1793604872	intron_variant
rs1793605037	intron_variant
rs1793606190	intron_variant
rs1793606381	intron_variant
rs1793606572	intron_variant
rs1793608283	intron_variant
rs1793608366	intron_variant
rs1793608568	intron_variant
rs1793608857	intron_variant
rs1793608949	intron_variant
rs1793609656	intron_variant
rs1793609772	intron_variant
rs1793610565	intron_variant
rs1793610663	intron_variant
rs1793610766	intron_variant
rs1793611340	intron_variant
rs1793611855	intron_variant
rs1793612057	intron_variant
rs1793612287	intron_variant
rs1793613329	intron_variant
rs1793613510	intron_variant
rs1793613612	intron_variant
rs1793613808	intron_variant
rs1793614049	intron_variant
rs1793614440	intron_variant
rs1793614546	intron_variant
rs1793614628	intron_variant
rs1793615241	intron_variant
rs1793615606	intron_variant
rs1793615904	intron_variant
rs1793616577	intron_variant
rs1793617147	intron_variant
rs1793617500	intron_variant
rs1793618014	intron_variant
rs1793618803	intron_variant
rs1793619077	intron_variant
rs1793619318	intron_variant
rs1793619795	intron_variant
rs1793619903	intron_variant
rs1793619988	intron_variant
rs1793620313	intron_variant
rs1793620734	intron_variant
rs1793621134	intron_variant
rs1793621561	intron_variant
rs1793621664	intron_variant
rs1793621775	intron_variant
rs1793622207	intron_variant
rs1793622420	intron_variant
rs1793622512	intron_variant
rs1793622614	intron_variant
rs1793623097	intron_variant
rs1793623308	intron_variant
rs1793623697	intron_variant
rs1793623905	intron_variant
rs1793624108	intron_variant
rs1793624328	intron_variant
rs1793624399	intron_variant
rs1793624686	intron_variant

rs1793624745	intron_variant
rs1793624896	intron_variant
rs1793625199	intron_variant
rs1793625488	intron_variant
rs1793625573	intron_variant
rs1793626027	intron_variant
rs1793626118	intron_variant
rs1793626219	intron_variant
rs1793626825	intron_variant
rs1793627058	intron_variant
rs1793627133	intron_variant
rs1793627305	intron_variant
rs1793628058	intron_variant
rs1793628283	intron_variant
rs1793631588	intron_variant
rs1793631882	intron_variant
rs1793631983	intron_variant
rs1793632221	intron_variant
rs1793632322	intron_variant
rs1793633581	intron_variant
rs1793633893	intron_variant
rs1793633998	intron_variant
rs1793635374	intron_variant
rs1793635547	intron_variant
rs1793636522	intron_variant
rs1793636719	intron_variant
rs1793637060	intron_variant
rs1793637511	intron_variant
rs1793637700	intron_variant
rs1793637836	intron_variant
rs1793638732	intron_variant
rs1793639732	intron_variant
rs1793639878	intron_variant
rs1793640565	intron_variant
rs1793640733	intron_variant
rs1793640883	intron_variant
rs1793641043	intron_variant
rs1793641488	intron_variant
rs1793643061	intron_variant
rs1793643140	intron_variant
rs1793643383	intron_variant
rs1793643465	intron_variant
rs1793643576	intron_variant
rs1793644019	intron_variant
rs1793645131	intron_variant
rs1793645236	intron_variant
rs1793645480	intron_variant
rs1793645834	intron_variant
rs1793646210	intron_variant
rs1793647549	intron_variant
rs1793647787	intron_variant
rs1793648632	intron_variant
rs1793649506	intron_variant
rs1793650709	intron_variant
rs1793651235	intron_variant
rs1793651786	intron_variant
rs1793652254	intron_variant
rs1793652419	intron_variant
rs1793652934	intron_variant
rs1793653895	intron_variant
rs1793654032	intron_variant
rs1793654192	intron_variant
rs1793655572	intron_variant
rs1793655850	intron_variant
rs1793655969	intron_variant
rs1793656427	intron_variant
rs1793656903	intron_variant
rs1793657049	intron_variant
rs1793657333	intron_variant
rs1793657600	intron_variant
rs1793657787	intron_variant
rs1793657934	intron_variant
rs1793658098	intron_variant
rs1793658225	intron_variant

rs1793658377	intron_variant
rs1793658675	intron_variant
rs1793658799	intron_variant
rs1793658906	intron_variant
rs1793659026	intron_variant
rs1793659143	intron_variant
rs1793659630	intron_variant
rs1793660233	intron_variant
rs1793660882	intron_variant
rs1793661639	intron_variant
rs1793661940	intron_variant
rs1793662081	intron_variant
rs1793662229	intron_variant
rs1793662395	intron_variant
rs1793662561	intron_variant
rs1793663244	intron_variant
rs1793663468	intron_variant
rs1793663920	intron_variant
rs1793664081	intron_variant
rs1793664246	intron_variant
rs1793665287	intron_variant
rs1793665566	intron_variant
rs1793665969	intron_variant
rs1793666450	intron_variant
rs1793667098	intron_variant
rs1793667271	intron_variant
rs1793667587	intron_variant
rs1793667757	intron_variant
rs1793668076	intron_variant
rs1793668185	intron_variant
rs1793668546	intron_variant
rs1793668813	intron_variant
rs1793669296	intron_variant
rs1793669412	intron_variant
rs1793669585	intron_variant
rs1793670143	intron_variant
rs1793671126	intron_variant
rs1793671501	intron_variant
rs1793671601	intron_variant
rs1793672110	intron_variant
rs1793672217	intron_variant
rs1793672596	intron_variant
rs1793672931	intron_variant
rs1793673073	intron_variant
rs1793673179	intron_variant
rs1793673256	intron_variant
rs1793673456	intron_variant
rs1793673752	intron_variant
rs1793674040	intron_variant
rs1793674423	intron_variant
rs1793674656	intron_variant
rs1793674855	intron_variant
rs1793675127	intron_variant
rs1793675217	intron_variant
rs1793675453	intron_variant
rs1793675615	intron_variant
rs1793675698	intron_variant
rs1793675793	intron_variant
rs1793675871	intron_variant
rs1793676051	intron_variant
rs1793676303	intron_variant
rs1793676436	intron_variant
rs1793676582	intron_variant
rs1793676717	intron_variant
rs1793677028	intron_variant
rs1793677566	intron_variant
rs1793677852	intron_variant
rs1793679747	intron_variant
rs1793680560	intron_variant
rs1793681066	intron_variant
rs1793682004	intron_variant
rs1793682143	intron_variant
rs1793682310	intron_variant
rs1793682519	intron_variant

rs1793682679	intron_variant
rs1793682823	intron_variant
rs1793683021	intron_variant
rs1793683496	intron_variant
rs1793683609	intron_variant
rs1793684022	intron_variant
rs1793684269	intron_variant
rs1793684403	intron_variant
rs1793684524	intron_variant
rs1793684658	intron_variant
rs1793684777	intron_variant
rs1793685172	intron_variant
rs1793685275	intron_variant
rs1793685389	intron_variant
rs1793685957	intron_variant
rs1793686093	intron_variant
rs1793686267	intron_variant
rs1793686599	intron_variant
rs1793686755	intron_variant
rs1793686897	intron_variant
rs1793687419	intron_variant
rs1793688338	intron_variant
rs1793688830	intron_variant
rs1793690500	intron_variant
rs1793691179	intron_variant
rs1793691923	intron_variant
rs1793692256	intron_variant
rs1793692588	intron_variant
rs1793693028	intron_variant
rs1793693725	intron_variant
rs1793693856	intron_variant
rs1793694037	intron_variant
rs1793694324	intron_variant
rs1793694467	intron_variant
rs1793694787	intron_variant
rs1793695306	intron_variant
rs1793695614	intron_variant
rs1793695779	intron_variant
rs1793696184	intron_variant
rs1793696265	intron_variant
rs1793696356	intron_variant
rs1793696645	intron_variant
rs1793696844	intron_variant
rs1793697448	intron_variant
rs1793697738	intron_variant
rs1793697834	intron_variant
rs1793697950	intron_variant
rs1793698112	intron_variant
rs1793698358	intron_variant
rs1793698565	intron_variant
rs1793698642	intron_variant
rs1793699119	intron_variant
rs1793699822	intron_variant
rs1793700258	intron_variant
rs1793700630	intron_variant
rs1793701279	intron_variant
rs1793701379	intron_variant
rs1793701490	intron_variant
rs1793701714	intron_variant
rs1793701819	intron_variant
rs1793701947	intron_variant
rs1793702753	intron_variant
rs1793702866	intron_variant
rs1793703198	intron_variant
rs1793703860	intron_variant
rs1793704215	intron_variant
rs1793704596	intron_variant
rs1793704879	intron_variant
rs1793705320	intron_variant
rs1793705406	intron_variant
rs1793706209	intron_variant
rs1793706438	intron_variant
rs1793706648	intron_variant
rs1793707036	intron_variant

rs1793707742	intron_variant
rs1793708066	intron_variant
rs1793708354	intron_variant
rs1793708448	intron_variant
rs1793708623	intron_variant
rs1793708793	intron_variant
rs1793708885	intron_variant
rs1793708984	intron_variant
rs1793709701	intron_variant
rs1793709810	intron_variant
rs1793709903	intron_variant
rs1793710034	intron_variant
rs1793710457	intron_variant
rs1793710984	intron_variant
rs1793711066	intron_variant
rs1793711197	intron_variant
rs1793711299	intron_variant
rs1793711508	intron_variant
rs1793712499	intron_variant
rs1793712679	intron_variant
rs1793712777	intron_variant
rs1793713418	intron_variant
rs1793713770	intron_variant
rs1793714097	intron_variant
rs1793714241	intron_variant
rs1793714583	intron_variant
rs1793715096	intron_variant
rs1793715406	intron_variant
rs1793715595	intron_variant
rs1793716256	intron_variant
rs1793716769	intron_variant
rs1793717506	intron_variant
rs1793717889	intron_variant
rs1793717989	intron_variant
rs1793718093	intron_variant
rs1793718339	intron_variant
rs1793718545	intron_variant
rs1793719004	intron_variant
rs1793719143	intron_variant
rs1793719705	intron_variant
rs1793720645	intron_variant
rs1793720833	intron_variant
rs1793721239	intron_variant
rs1793721960	intron_variant
rs1793722484	intron_variant
rs1793722670	intron_variant
rs1793722785	intron_variant
rs1793723019	intron_variant
rs1793723230	intron_variant
rs1793723618	intron_variant
rs1793723718	intron_variant
rs1793723824	intron_variant
rs1793723936	intron_variant
rs1793724041	intron_variant
rs1793724341	intron_variant
rs1793724436	intron_variant
rs1793724540	intron_variant
rs1793725371	intron_variant
rs1793725689	intron_variant
rs1793725944	intron_variant
rs1793727780	intron_variant
rs1793728058	intron_variant
rs1793728413	intron_variant
rs1793728620	intron_variant
rs1793729270	intron_variant
rs1793729541	intron_variant
rs1793729969	intron_variant
rs1793730062	intron_variant
rs1793730503	intron_variant
rs1793730975	intron_variant
rs1793731177	intron_variant
rs1793731523	intron_variant
rs1793731931	intron_variant
rs1793732029	intron_variant

rs1793733189	intron_variant
rs1793733486	intron_variant
rs1793733896	intron_variant
rs1793733990	intron_variant
rs1793734094	intron_variant
rs1793734187	intron_variant
rs1793734294	intron_variant
rs1793734874	intron_variant
rs1793735383	intron_variant
rs1793735769	intron_variant
rs1793735842	intron_variant
rs1793736138	intron_variant
rs1793737597	intron_variant
rs1793738144	intron_variant
rs1793738295	intron_variant
rs1793738569	intron_variant
rs1793738933	intron_variant
rs1793739217	intron_variant
rs1793739287	intron_variant
rs1793739791	intron_variant
rs1793739874	intron_variant
rs1793739975	intron_variant
rs1793740202	intron_variant
rs1793740311	intron_variant
rs1793740500	intron_variant
rs1793740931	intron_variant
rs1793741042	intron_variant
rs1793741151	intron_variant
rs1793741251	intron_variant
rs1793741456	intron_variant
rs1793741634	intron_variant
rs1793741856	intron_variant
rs1793741963	intron_variant
rs1793743179	intron_variant
rs1793743750	intron_variant
rs1793744280	intron_variant
rs1793744821	intron_variant
rs1793744935	intron_variant
rs1793745291	intron_variant
rs1793745492	intron_variant
rs1793745612	intron_variant
rs1793746074	intron_variant
rs1793746389	intron_variant
rs1793746546	intron_variant
rs1793747213	intron_variant
rs1793748482	intron_variant
rs1793748611	intron_variant
rs1793748852	intron_variant
rs1793749582	intron_variant
rs1793749696	intron_variant
rs1793749809	intron_variant
rs1793750362	intron_variant
rs1793750603	intron_variant
rs1793751156	intron_variant
rs1793751249	intron_variant
rs1793751354	intron_variant
rs1793751575	intron_variant
rs1793752172	intron_variant
rs1793752259	intron_variant
rs1793752415	intron_variant
rs1793752589	intron_variant
rs1793753294	intron_variant
rs1793753435	intron_variant
rs1793753714	intron_variant
rs1793753959	intron_variant
rs1793754160	intron_variant
rs1793754802	intron_variant
rs1793755133	intron_variant
rs1793755824	intron_variant
rs1793756254	intron_variant
rs1793756373	intron_variant
rs1793756528	intron_variant
rs1793758156	intron_variant
rs1793758368	intron_variant

rs1793761146	intron_variant
rs1793761493	intron_variant
rs1793761671	intron_variant
rs1793761996	intron_variant
rs1793763126	intron_variant
rs1793763492	intron_variant
rs1793763956	intron_variant
rs1793764257	intron_variant
rs1793764415	intron_variant
rs1793764717	intron_variant
rs1793765225	intron_variant
rs1793765386	intron_variant
rs1793766885	intron_variant
rs1793767013	intron_variant
rs1793767149	intron_variant
rs1793767892	intron_variant
rs1793768699	intron_variant
rs1793768867	intron_variant
rs1793769987	intron_variant
rs1793771648	intron_variant
rs1793772117	intron_variant
rs1793772238	intron_variant
rs1793772796	intron_variant
rs1793773069	intron_variant
rs1793773176	intron_variant
rs1793773616	intron_variant
rs1793774086	intron_variant
rs1793774193	intron_variant
rs1793774310	intron_variant
rs1793774418	intron_variant
rs1793774675	intron_variant
rs1793776072	intron_variant
rs1793776178	intron_variant
rs1793776802	intron_variant
rs1793776901	intron_variant
rs1793777403	intron_variant
rs1793777510	intron_variant
rs1793777799	intron_variant
rs1793778108	intron_variant
rs1793778339	intron_variant
rs1793778546	intron_variant
rs1793778633	intron_variant
rs1793778727	intron_variant
rs1793778827	intron_variant
rs1793779160	intron_variant
rs1793779330	intron_variant
rs1793779495	intron_variant
rs1793779869	intron_variant
rs1793780449	intron_variant
rs1793781135	intron_variant
rs1793781303	intron_variant
rs1793781615	intron_variant
rs1793781775	intron_variant
rs1793782376	intron_variant
rs1793782704	intron_variant
rs1793782864	intron_variant
rs1793783544	intron_variant
rs1793783728	intron_variant
rs1793784197	intron_variant
rs1793785481	intron_variant
rs1793786048	intron_variant
rs1793786380	intron_variant
rs1793786520	intron_variant
rs1793786666	intron_variant
rs1793787287	intron_variant
rs1793787592	intron_variant
rs1793787753	intron_variant
rs1793788347	intron_variant
rs1793788640	intron_variant
rs1793788783	intron_variant
rs1793789107	intron_variant
rs1793789394	intron_variant
rs1793789541	intron_variant
rs1793790003	intron_variant

rs1793790481	intron_variant
rs1793791299	intron_variant
rs1793791405	intron_variant
rs1793791570	intron_variant
rs1793792613	intron_variant
rs1793792903	intron_variant
rs1793793044	intron_variant
rs1793796074	intron_variant
rs1793796251	intron_variant
rs1793796686	intron_variant
rs1793797505	intron_variant
rs1793798111	intron_variant
rs1793798589	intron_variant
rs1793798751	intron_variant
rs1793799472	intron_variant
rs1793799617	intron_variant
rs1793799790	intron_variant
rs1793800149	intron_variant
rs1793800276	intron_variant
rs1793801743	intron_variant
rs1793801901	intron_variant
rs1793802362	intron_variant
rs1793802526	intron_variant
rs1793802687	intron_variant
rs1793803006	intron_variant
rs1793803187	intron_variant
rs1793804098	intron_variant
rs1793804260	intron_variant
rs1793804555	intron_variant
rs1793804819	intron_variant
rs1793805267	intron_variant
rs1793805428	intron_variant
rs1793805596	intron_variant
rs1793805743	intron_variant
rs1793805907	intron_variant
rs1793806069	intron_variant
rs1793806679	intron_variant
rs1793806801	intron_variant
rs1793806985	intron_variant
rs1793807302	intron_variant
rs1793807445	intron_variant
rs1793808229	intron_variant
rs1793809153	intron_variant
rs1793809305	intron_variant
rs1793809621	intron_variant
rs1793809880	intron_variant
rs1793809994	intron_variant
rs1793810630	intron_variant
rs1793810977	intron_variant
rs1793812005	intron_variant
rs1793812151	intron_variant
rs1793814480	intron_variant
rs1793814634	intron_variant
rs1793814790	intron_variant
rs1793814936	intron_variant
rs1793816532	intron_variant
rs1793816683	intron_variant
rs1793816908	intron_variant
rs1793817735	intron_variant
rs1793818023	intron_variant
rs1793818410	intron_variant
rs1793818782	intron_variant
rs1793819496	intron_variant
rs1793820069	intron_variant
rs1793820168	intron_variant
rs1793820264	intron_variant
rs1793820645	intron_variant
rs1793820741	intron_variant
rs1793820827	intron_variant
rs1793820934	intron_variant
rs1793821034	intron_variant
rs1793821698	intron_variant
rs1793821820	intron_variant
rs1793822323	intron_variant

rs1793822414	intron_variant
rs1793822624	intron_variant
rs1793822832	intron_variant
rs1793823376	intron_variant
rs1793823558	intron_variant
rs1793823648	intron_variant
rs1793823745	intron_variant
rs1793823964	intron_variant
rs1793824060	intron_variant
rs1793824847	intron_variant
rs1793825133	intron_variant
rs1793825436	intron_variant
rs1793825546	intron_variant
rs1793825844	intron_variant
rs1793826133	intron_variant
rs1793826583	intron_variant
rs1793826867	intron_variant
rs1793827307	intron_variant
rs1793827720	intron_variant
rs1793828216	intron_variant
rs1793828319	intron_variant
rs1793828397	intron_variant
rs1793828595	intron_variant
rs1793828878	intron_variant
rs1793828965	intron_variant
rs1793829066	intron_variant
rs1793829152	intron_variant
rs1793829692	intron_variant
rs1793829791	intron_variant
rs1793829891	intron_variant
rs1793830204	intron_variant
rs1793830559	intron_variant
rs1793830817	intron_variant
rs1793831029	intron_variant
rs1793831323	intron_variant
rs1793831760	intron_variant
rs1793831835	intron_variant
rs1793832298	intron_variant
rs1793832587	intron_variant
rs1793832993	intron_variant
rs1793833796	intron_variant
rs1793833890	intron_variant
rs1793834390	intron_variant
rs1793834494	intron_variant
rs1793834590	intron_variant
rs1793834694	intron_variant
rs1793834785	intron_variant
rs1793835313	intron_variant
rs1793835511	intron_variant
rs1793836227	intron_variant
rs1793836922	intron_variant
rs1793837015	intron_variant
rs1793837535	intron_variant
rs1793837641	intron_variant
rs1793838483	intron_variant
rs1793838683	intron_variant
rs1793838788	intron_variant
rs1793838882	intron_variant
rs1793839221	intron_variant
rs1793839766	intron_variant
rs1793840143	intron_variant
rs1793840245	intron_variant
rs1793840692	intron_variant
rs1793840993	intron_variant
rs1793841187	intron_variant
rs1793842160	intron_variant
rs1793842512	intron_variant
rs1793843211	intron_variant
rs1793843420	intron_variant
rs1793843615	intron_variant
rs1793843924	intron_variant
rs1793844022	intron_variant
rs1793844449	intron_variant
rs1793845031	intron_variant

rs1793845263	intron_variant
rs1793845380	intron_variant
rs1793845497	intron_variant
rs1793845597	intron_variant
rs1793845798	intron_variant
rs1793846104	intron_variant
rs1793846230	intron_variant
rs1793846495	intron_variant
rs1793846741	intron_variant
rs1793846851	intron_variant
rs1793846947	intron_variant
rs1793847045	intron_variant
rs1793847161	intron_variant
rs1793847464	intron_variant
rs1793847561	intron_variant
rs1793847906	intron_variant
rs1793848026	intron_variant
rs1793848494	intron_variant
rs1793848607	intron_variant
rs1793848863	intron_variant
rs1793851226	intron_variant
rs1793851702	intron_variant
rs1793852198	intron_variant
rs1793852395	intron_variant
rs1793852836	intron_variant
rs1793853044	intron_variant
rs1793853151	intron_variant
rs1793853251	intron_variant
rs1793853786	intron_variant
rs1793854375	intron_variant
rs1793854688	intron_variant
rs1793854882	intron_variant
rs1793855702	intron_variant
rs1793855942	intron_variant
rs1793856035	intron_variant
rs1793856141	intron_variant
rs1793856425	intron_variant
rs1793856635	intron_variant
rs1793857766	intron_variant
rs1793859131	intron_variant
rs1793859234	intron_variant
rs1793859335	intron_variant
rs1793859697	intron_variant
rs1793859808	intron_variant
rs1793860158	intron_variant
rs1793860722	intron_variant
rs1793860832	intron_variant
rs1793861033	intron_variant
rs1793861163	intron_variant
rs1793861256	intron_variant
rs1793861471	intron_variant
rs1793861567	intron_variant
rs1793862217	intron_variant
rs1793862395	intron_variant
rs1793862719	intron_variant
rs1793863026	intron_variant
rs1793863652	intron_variant
rs1793863891	intron_variant
rs1793864083	intron_variant
rs1793864397	intron_variant
rs1793865388	intron_variant
rs1793865985	intron_variant
rs1793866495	intron_variant
rs1793866888	intron_variant
rs1793867481	intron_variant
rs1793867597	intron_variant
rs1793868705	intron_variant
rs1793868816	intron_variant
rs1793869401	intron_variant
rs1793869534	intron_variant
rs1793869633	intron_variant
rs1793869732	intron_variant
rs1793869954	intron_variant
rs1793870059	intron_variant

rs1793870278	intron_variant
rs1793870390	intron_variant
rs1793870489	intron_variant
rs1793870588	intron_variant
rs1793871089	intron_variant
rs1793871413	intron_variant
rs1793872441	intron_variant
rs1793872552	intron_variant
rs1793872774	intron_variant
rs1793872884	intron_variant
rs1793872992	intron_variant
rs1793873385	intron_variant
rs1793873482	intron_variant
rs1793873890	intron_variant
rs1793874091	intron_variant
rs1793874181	intron_variant
rs1793874484	intron_variant
rs1793874680	intron_variant
rs1793874784	intron_variant
rs1793874884	intron_variant
rs1793874972	intron_variant
rs1793875368	intron_variant
rs1793875476	intron_variant
rs1793875578	intron_variant
rs1793875752	intron_variant
rs1793876148	intron_variant
rs1793876283	intron_variant
rs1793876379	intron_variant
rs1793876716	intron_variant
rs1793876827	intron_variant
rs1793877310	intron_variant
rs1793877401	intron_variant
rs1793878194	intron_variant
rs1793878302	intron_variant
rs1793878613	intron_variant
rs1793878705	intron_variant
rs1793879100	intron_variant
rs1793880248	intron_variant
rs1793880353	intron_variant
rs1793880446	intron_variant
rs1793880674	intron_variant
rs1793880874	intron_variant
rs1793881002	intron_variant
rs1793881736	intron_variant
rs1793881950	intron_variant
rs1793882230	intron_variant
rs1793882338	intron_variant
rs1793882538	intron_variant
rs1793882732	intron_variant
rs1793882846	intron_variant
rs1793882941	intron_variant
rs1793883064	intron_variant
rs1793883612	intron_variant
rs1793884197	intron_variant
rs1793884302	intron_variant
rs1793884780	intron_variant
rs1793886085	intron_variant
rs1793886407	intron_variant
rs1793886730	intron_variant
rs1793887486	intron_variant
rs1793888265	intron_variant
rs1793888418	intron_variant
rs1793888649	intron_variant
rs1793889064	intron_variant
rs1793889213	intron_variant
rs1793890243	intron_variant
rs1793890675	intron_variant
rs1793891477	intron_variant
rs1793891573	intron_variant
rs1793892202	intron_variant
rs1793892353	intron_variant
rs1793892500	intron_variant
rs1793892670	intron_variant
rs1793892804	intron_variant

rs1793893437	intron_variant
rs1793894610	intron_variant
rs1793894889	intron_variant
rs1793895048	intron_variant
rs1793895324	intron_variant
rs1793895807	intron_variant
rs1793896148	intron_variant
rs1793896907	intron_variant
rs1793897236	intron_variant
rs1793897553	intron_variant
rs1793897709	intron_variant
rs1793898236	intron_variant
rs1793898770	intron_variant
rs1793900422	intron_variant
rs1793900571	intron_variant
rs1793900860	intron_variant
rs1793901005	intron_variant
rs1793901538	intron_variant
rs1793902017	intron_variant
rs1793902494	intron_variant
rs1793902827	intron_variant
rs1793902977	intron_variant
rs1793903139	intron_variant
rs1793903299	intron_variant
rs1793903462	intron_variant
rs1793903875	intron_variant
rs1793903981	intron_variant
rs1793904764	intron_variant
rs1793905058	intron_variant
rs1793905242	intron_variant
rs1793905431	intron_variant
rs1793905518	intron_variant
rs1793905608	intron_variant
rs1793906284	intron_variant
rs1793906776	intron_variant
rs1793907382	intron_variant
rs1793907571	intron_variant
rs1793907772	intron_variant
rs1793908412	intron_variant
rs1793908520	intron_variant
rs1793909069	intron_variant
rs1793909495	intron_variant
rs1793909625	intron_variant
rs1793910181	intron_variant
rs1793910513	intron_variant
rs1793911023	intron_variant
rs1793911630	intron_variant
rs1793912647	intron_variant
rs1793912840	intron_variant
rs1793913157	intron_variant
rs1793913248	intron_variant
rs1793913341	intron_variant
rs1793914869	intron_variant
rs1793916030	intron_variant
rs1793916494	intron_variant
rs1793917087	intron_variant
rs1793917540	intron_variant
rs1793918195	intron_variant
rs1793918820	intron_variant
rs1793920687	intron_variant
rs1793920839	intron_variant
rs1793921140	intron_variant
rs1793922902	intron_variant
rs1793925321	intron_variant
rs1793925611	intron_variant
rs1793926151	intron_variant
rs1793926292	intron_variant
rs1793926447	intron_variant
rs1793926924	intron_variant
rs1793927085	intron_variant
rs1793927241	intron_variant
rs1793927387	intron_variant
rs1793927544	intron_variant
rs1793927875	intron_variant

rs1793928790	intron_variant
rs1793928914	intron_variant
rs1793930645	intron_variant
rs1793931195	intron_variant
rs1793931277	intron_variant
rs1793931493	intron_variant
rs1793931817	intron_variant
rs1793932705	intron_variant
rs1793933016	intron_variant
rs1793933823	intron_variant
rs1793933966	intron_variant
rs1793934561	intron_variant
rs1793934657	intron_variant
rs1793934817	intron_variant
rs1793935084	intron_variant
rs1793935565	intron_variant
rs1793935736	intron_variant
rs1793936004	intron_variant
rs1793936149	intron_variant
rs1793936536	intron_variant
rs1793936632	intron_variant
rs1793936721	intron_variant
rs1793936906	intron_variant
rs1793937342	intron_variant
rs1793938146	intron_variant
rs1793938298	intron_variant
rs1793938480	intron_variant
rs1793938760	intron_variant
rs1793939217	intron_variant
rs1793939291	intron_variant
rs1793940564	intron_variant
rs1793941092	intron_variant
rs1793941398	intron_variant
rs1793942186	intron_variant
rs1793942542	intron_variant
rs1793942989	intron_variant
rs1793943129	intron_variant
rs1793943580	intron_variant
rs1793944516	intron_variant
rs1793945044	intron_variant
rs1793945240	intron_variant
rs1793945679	intron_variant
rs1793946109	intron_variant
rs1793946778	intron_variant
rs1793946865	intron_variant
rs1793947040	intron_variant
rs1793947493	intron_variant
rs1793947829	intron_variant
rs1793947925	intron_variant
rs1793948016	intron_variant
rs1793948198	intron_variant
rs1793948292	intron_variant
rs1793948778	intron_variant
rs1793948942	intron_variant
rs1793949076	intron_variant
rs1793949226	intron_variant
rs1793949766	intron_variant
rs1793949909	intron_variant
rs1793950074	intron_variant
rs1793950228	intron_variant
rs1793950499	intron_variant
rs1793950649	intron_variant
rs1793951149	intron_variant
rs1793951312	intron_variant
rs1793951876	intron_variant
rs1793951996	intron_variant
rs1793952124	intron_variant
rs1793952498	intron_variant
rs1793952600	intron_variant
rs1793952694	intron_variant
rs1793952780	intron_variant
rs1793953018	intron_variant
rs1793953207	intron_variant
rs1793953514	intron_variant

rs1793954198	intron_variant
rs1793954675	intron_variant
rs1793954761	intron_variant
rs1793954925	intron_variant
rs1793955159	intron_variant
rs1793955268	intron_variant
rs1793955341	intron_variant
rs1793955437	intron_variant
rs1793955972	intron_variant
rs1793956145	intron_variant
rs1793956451	intron_variant
rs1793956728	intron_variant
rs1793956815	intron_variant
rs1793958228	intron_variant
rs1793958549	intron_variant
rs1793959106	intron_variant
rs1793959580	intron_variant
rs1793960846	intron_variant
rs1793961425	intron_variant
rs1793961884	intron_variant
rs1793962028	intron_variant
rs1793962334	intron_variant
rs1793962757	intron_variant
rs1793963059	intron_variant
rs1793963242	intron_variant
rs1793964395	intron_variant
rs1793964505	intron_variant
rs1793964676	intron_variant
rs1793965945	intron_variant
rs1793966439	intron_variant
rs1793966580	intron_variant
rs1793966863	intron_variant
rs1793967024	intron_variant
rs1793967159	intron_variant
rs1793967302	intron_variant
rs1793967456	intron_variant
rs1793968117	intron_variant
rs1793968255	intron_variant
rs1793968399	intron_variant
rs1793968540	intron_variant
rs1793969226	intron_variant
rs1793969425	intron_variant
rs1793970097	intron_variant
rs1793971501	intron_variant
rs1793971846	intron_variant
rs1793973513	intron_variant
rs1793975645	intron_variant
rs1793975805	intron_variant
rs1793976295	intron_variant
rs1793976850	intron_variant
rs1793977121	intron_variant
rs1793977383	intron_variant
rs1793977699	intron_variant
rs1793978120	intron_variant
rs1793978188	intron_variant
rs1793979871	intron_variant
rs1793980039	intron_variant
rs1793980976	intron_variant
rs1793981464	intron_variant
rs1793981609	intron_variant
rs1793982173	intron_variant
rs1793982322	intron_variant
rs1793982823	intron_variant
rs1793982970	intron_variant
rs1793983649	intron_variant
rs1793983801	intron_variant
rs1793984575	intron_variant
rs1793984728	intron_variant
rs1793985189	intron_variant
rs1793985657	intron_variant
rs1793986740	intron_variant
rs1793986884	intron_variant
rs1793987183	intron_variant
rs1793987440	intron_variant

rs1793987769	intron_variant
rs1793987979	intron_variant
rs1793988285	intron_variant
rs1793988562	intron_variant
rs1793988659	intron_variant
rs1793989184	intron_variant
rs1793989383	intron_variant
rs1793989473	intron_variant
rs1793989999	intron_variant
rs1793990638	intron_variant
rs1793991608	intron_variant
rs1793991774	intron_variant
rs1793991944	intron_variant
rs1793992118	intron_variant
rs1793992853	intron_variant
rs1793992936	intron_variant
rs1793993129	intron_variant
rs1793993305	intron_variant
rs1793993493	intron_variant
rs1793993663	intron_variant
rs1793993944	intron_variant
rs1793994573	intron_variant
rs1793994750	intron_variant
rs1793994839	intron_variant
rs1793995215	intron_variant
rs1793995417	intron_variant
rs1793995630	intron_variant
rs1793995728	intron_variant
rs1793996448	intron_variant
rs1793997065	intron_variant
rs1793997220	intron_variant
rs1793997377	intron_variant
rs1793997675	intron_variant
rs1793997929	intron_variant
rs1793998944	intron_variant
rs1793999258	intron_variant
rs1793999944	intron_variant
rs1794000089	intron_variant
rs1794000386	intron_variant
rs1794000533	intron_variant
rs1794001447	intron_variant
rs1794002235	intron_variant
rs1794002906	intron_variant
rs1794003060	intron_variant
rs1794003977	intron_variant
rs1794004465	intron_variant
rs1794004771	intron_variant
rs1794004913	intron_variant
rs1794005228	intron_variant
rs1794005395	intron_variant
rs1794005540	intron_variant
rs1794005822	intron_variant
rs1794006440	intron_variant
rs1794006551	intron_variant
rs1794006714	intron_variant
rs1794006859	intron_variant
rs1794007007	intron_variant
rs1794007265	intron_variant
rs1794007417	intron_variant
rs1794007736	intron_variant
rs1794008026	intron_variant
rs1794008866	intron_variant
rs1794008976	intron_variant
rs1794009532	intron_variant
rs1794009639	intron_variant
rs1794009831	intron_variant
rs1794010051	intron_variant
rs1794010354	intron_variant
rs1794010559	intron_variant
rs1794010790	intron_variant
rs1794011002	intron_variant
rs1794011499	intron_variant
rs1794011631	intron_variant
rs1794012153	intron_variant

rs1794012326	intron_variant
rs1794012545	intron_variant
rs1794012655	intron_variant
rs1794013404	intron_variant
rs1794014206	intron_variant
rs1794014395	intron_variant
rs1794014874	intron_variant
rs1794015695	intron_variant
rs1794015844	intron_variant
rs1794016731	intron_variant
rs1794017414	intron_variant
rs1794017675	intron_variant
rs1794018313	intron_variant
rs1794018648	intron_variant
rs1794018823	intron_variant
rs1794018920	intron_variant
rs1794019361	intron_variant
rs1794019877	intron_variant
rs1794020243	intron_variant
rs1794020335	intron_variant
rs1794020416	intron_variant
rs1794021035	intron_variant
rs1794021208	intron_variant
rs1794021294	intron_variant
rs1794021385	intron_variant
rs1794022172	intron_variant
rs1794022264	intron_variant
rs1794022521	intron_variant
rs1794023073	intron_variant
rs1794023243	intron_variant
rs1794023322	intron_variant
rs1794024080	intron_variant
rs1794024348	intron_variant
rs1794024543	intron_variant
rs1794024878	intron_variant
rs1794024959	intron_variant
rs1794025057	intron_variant
rs1794025146	intron_variant
rs1794025442	intron_variant
rs1794025532	intron_variant
rs1794025854	intron_variant
rs1794026333	intron_variant
rs1794027330	intron_variant
rs1794027432	intron_variant
rs1794027518	intron_variant
rs1794028260	intron_variant
rs1794028813	intron_variant
rs1794029262	intron_variant
rs1794030023	intron_variant
rs1794030210	intron_variant
rs1794031560	intron_variant
rs1794032030	intron_variant
rs1794032113	intron_variant
rs1794032217	intron_variant
rs1794032499	intron_variant
rs1794033090	intron_variant
rs1794033325	intron_variant
rs1794033643	intron_variant
rs1794033957	intron_variant
rs1794034048	intron_variant
rs1794034861	intron_variant
rs1794035067	intron_variant
rs1794035365	intron_variant
rs1794035726	intron_variant
rs1794036077	intron_variant
rs1794036369	intron_variant
rs1794036655	intron_variant
rs1794036979	intron_variant
rs1794037317	intron_variant
rs1794037583	intron_variant
rs1794037987	intron_variant
rs1794038499	intron_variant
rs1794038590	intron_variant
rs1794038696	intron_variant

rs1794038974	intron_variant
rs1794039293	intron_variant
rs1794041012	intron_variant
rs1794041311	intron_variant
rs1794041405	intron_variant
rs1794041718	intron_variant
rs1794041815	intron_variant
rs1794041908	intron_variant
rs1794042004	intron_variant
rs1794042667	intron_variant
rs1794042888	intron_variant
rs1794043035	intron_variant
rs1794043834	intron_variant
rs1794044006	intron_variant
rs1794044162	intron_variant
rs1794044640	intron_variant
rs1794045120	intron_variant
rs1794045433	intron_variant
rs1794045582	intron_variant
rs1794045744	intron_variant
rs1794045890	intron_variant
rs1794046204	intron_variant
rs1794046351	intron_variant
rs1794047791	intron_variant
rs1794047960	intron_variant
rs1794048100	intron_variant
rs1794048780	intron_variant
rs1794048941	intron_variant
rs1794049525	intron_variant
rs1794049675	intron_variant
rs1794049929	intron_variant
rs1794050230	intron_variant
rs1794050665	intron_variant
rs1794051031	intron_variant
rs1794051168	intron_variant
rs1794051867	intron_variant
rs1794052006	intron_variant
rs1794052326	intron_variant
rs1794052419	intron_variant
rs1794052610	intron_variant
rs1794053196	intron_variant
rs1794053476	intron_variant
rs1794053576	intron_variant
rs1794054042	intron_variant
rs1794054552	intron_variant
rs1794054792	intron_variant
rs1794054886	intron_variant
rs1794055352	intron_variant
rs1794055956	intron_variant
rs1794056207	intron_variant
rs1794056708	intron_variant
rs1794056909	intron_variant
rs1794057003	intron_variant
rs1794057646	intron_variant
rs1794057753	intron_variant
rs1794058029	intron_variant
rs1794058224	intron_variant
rs1794058342	intron_variant
rs1794058435	intron_variant
rs1794058527	intron_variant
rs1794058833	intron_variant
rs1794058957	intron_variant
rs1794059368	intron_variant
rs1794059466	intron_variant
rs1794059578	intron_variant
rs1794059675	intron_variant
rs1794060165	intron_variant
rs1794060779	intron_variant
rs1794061059	intron_variant
rs1794061196	intron_variant
rs1794063443	intron_variant
rs1794064111	intron_variant
rs1794064777	intron_variant
rs1794065056	intron_variant

rs1794065336	intron_variant
rs1794065909	intron_variant
rs1794065998	intron_variant
rs1794066871	intron_variant
rs1794067208	intron_variant
rs1794068153	intron_variant
rs1794068359	intron_variant
rs1794068462	intron_variant
rs1794069046	intron_variant
rs1794069144	intron_variant
rs1794069234	intron_variant
rs1794069324	intron_variant
rs1794069579	intron_variant
rs1794070337	intron_variant
rs1794070546	intron_variant
rs1794070904	intron_variant
rs1794070994	intron_variant
rs1794071136	intron_variant
rs1794071215	intron_variant
rs1794071292	intron_variant
rs1794071448	intron_variant
rs1794072524	intron_variant
rs1794072857	intron_variant
rs1794072936	intron_variant
rs1794073059	intron_variant
rs1794073259	intron_variant
rs1794073361	intron_variant
rs1794073555	intron_variant
rs1794073728	intron_variant
rs1794073830	intron_variant
rs1794073966	intron_variant
rs1794074057	intron_variant
rs1794074300	intron_variant
rs1794074463	intron_variant
rs1794074624	intron_variant
rs1794074790	intron_variant
rs1794075203	intron_variant
rs1794075676	intron_variant
rs1794075878	intron_variant
rs1794076021	intron_variant
rs1794076404	intron_variant
rs1794076565	intron_variant
rs1794076921	intron_variant
rs1794077476	intron_variant
rs1794077574	intron_variant
rs1794077913	intron_variant
rs1794078249	intron_variant
rs1794078350	intron_variant
rs1794078559	intron_variant
rs1794078657	intron_variant
rs1794078743	intron_variant
rs1794079067	intron_variant
rs1794079401	intron_variant
rs1794079720	intron_variant
rs1794079835	intron_variant
rs1794080273	intron_variant
rs1794080358	intron_variant
rs1794080957	intron_variant
rs1794081253	intron_variant
rs1794081350	intron_variant
rs1794081556	intron_variant
rs1794081951	intron_variant
rs1794083279	intron_variant
rs1794083535	intron_variant
rs1794083624	intron_variant
rs1794083739	intron_variant
rs1794083844	intron_variant
rs1794083984	intron_variant
rs1794084414	intron_variant
rs1794084631	intron_variant
rs1794085051	intron_variant
rs1794085355	intron_variant
rs1794085451	intron_variant
rs1794085538	intron_variant

rs1794085817	intron_variant
rs1794086084	intron_variant
rs1794086192	intron_variant
rs1794086273	intron_variant
rs1794087020	intron_variant
rs1794087144	intron_variant
rs1794087253	intron_variant
rs1794087461	intron_variant
rs1794087576	intron_variant
rs1794087682	intron_variant
rs1794087979	intron_variant
rs1794088080	intron_variant
rs1794088369	intron_variant
rs1794089253	intron_variant
rs1794089956	intron_variant
rs1794090344	intron_variant
rs1794090643	intron_variant
rs1794090818	intron_variant
rs1794091570	intron_variant
rs1794091928	intron_variant
rs1794092060	intron_variant
rs1794093214	intron_variant
rs1794093371	intron_variant
rs1794093909	intron_variant
rs1794095150	intron_variant
rs1794096335	intron_variant
rs1794097089	intron_variant
rs1794097262	intron_variant
rs1794097406	intron_variant
rs1794097916	intron_variant
rs1794098078	intron_variant
rs1794098240	intron_variant
rs1794098364	intron_variant
rs1794098689	intron_variant
rs1794098835	intron_variant
rs1794099333	intron_variant
rs1794100515	intron_variant
rs1794100675	intron_variant
rs1794101697	intron_variant
rs1794102003	intron_variant
rs1794102777	intron_variant
rs1794103053	intron_variant
rs1794103789	intron_variant
rs1794103961	intron_variant
rs1794104081	intron_variant
rs1794104197	intron_variant
rs1794104298	intron_variant
rs1794104405	intron_variant
rs1794104507	intron_variant
rs1794104598	intron_variant
rs1794104712	intron_variant
rs1794104801	intron_variant
rs1794104894	intron_variant
rs1794105005	intron_variant
rs1794105121	intron_variant
rs1794105862	intron_variant
rs1794106066	intron_variant
rs1794106160	intron_variant
rs1794106261	intron_variant
rs1794106582	intron_variant
rs1794106793	intron_variant
rs1794106907	intron_variant
rs1794107584	intron_variant
rs1794107772	intron_variant
rs1794108075	intron_variant
rs1794108182	intron_variant
rs1794108376	intron_variant
rs1794108490	intron_variant
rs1794108803	intron_variant
rs1794109000	intron_variant
rs1794109103	intron_variant
rs1794109297	intron_variant
rs1794109702	intron_variant
rs1794109872	intron_variant

rs1794110378	intron_variant
rs1794110564	intron_variant
rs1794110767	intron_variant
rs1794111006	intron_variant
rs1794111335	intron_variant
rs1794111437	intron_variant
rs1794111565	intron_variant
rs1794112245	intron_variant
rs1794112482	intron_variant
rs1794112574	intron_variant
rs1794112897	intron_variant
rs1794113170	intron_variant
rs1794113374	intron_variant
rs1794113474	intron_variant
rs1794113568	intron_variant
rs1794113919	intron_variant
rs1794114130	intron_variant
rs1794114328	intron_variant
rs1794114577	intron_variant
rs1794114845	intron_variant
rs1794115485	intron_variant
rs1794115622	intron_variant
rs1794115795	intron_variant
rs1794115947	intron_variant
rs1794116072	intron_variant
rs1794116331	intron_variant
rs1794116443	intron_variant
rs1794116537	intron_variant
rs1794116626	intron_variant
rs1794116725	intron_variant
rs1794116956	intron_variant
rs1794117057	intron_variant
rs1794117176	intron_variant
rs1794117282	intron_variant
rs1794117405	intron_variant
rs1794117507	intron_variant
rs1794117622	intron_variant
rs1794117727	intron_variant
rs1794117806	intron_variant
rs1794117919	intron_variant
rs1794118280	intron_variant
rs1794118421	intron_variant
rs1794118518	intron_variant
rs1794118736	intron_variant
rs1794119218	intron_variant
rs1794119556	intron_variant
rs1794119691	intron_variant
rs1794119822	intron_variant
rs1794119967	intron_variant
rs1794120093	intron_variant
rs1794120299	intron_variant
rs1794120415	intron_variant
rs1794120521	intron_variant
rs1794120774	intron_variant
rs1794121383	intron_variant
rs1794121477	intron_variant
rs1794121735	intron_variant
rs1794122187	intron_variant
rs1794122437	intron_variant
rs1794122768	intron_variant
rs1794122884	intron_variant
rs1794125070	intron_variant
rs1794125168	intron_variant
rs1794125408	intron_variant
rs1794126110	intron_variant
rs1794126645	intron_variant
rs1794126725	intron_variant
rs1794126811	intron_variant
rs1794126914	intron_variant
rs1794127092	intron_variant
rs1794128124	intron_variant
rs1794128242	intron_variant
rs1794128328	intron_variant
rs1794128429	intron_variant

rs1794128548	intron_variant
rs1794128645	intron_variant
rs1794129336	intron_variant
rs1794129590	intron_variant
rs1794129693	intron_variant
rs1794130726	intron_variant
rs1794130844	intron_variant
rs1794130990	intron_variant
rs1794131070	intron_variant
rs1794131303	intron_variant
rs1794131517	intron_variant
rs1794131623	intron_variant
rs1794131740	intron_variant
rs1794132463	intron_variant
rs1794132959	intron_variant
rs1794134702	intron_variant
rs1794134805	intron_variant
rs1794135372	intron_variant
rs1794135825	intron_variant
rs1794136761	intron_variant
rs1794140926	coding_sequence_variant,non_coding_transcript_variant,missense_variant
rs1794141772	coding_sequence_variant,non_coding_transcript_variant,missense_variant
rs1794142537	coding_sequence_variant,non_coding_transcript_variant,missense_variant
rs1794142644	coding_sequence_variant,non_coding_transcript_variant,synonymous_variant
rs1794142770	coding_sequence_variant,non_coding_transcript_variant,missense_variant
rs1794143799	coding_sequence_variant,non_coding_transcript_variant,missense_variant
rs1794146524	intron_variant
rs1794148115	intron_variant
rs1794148303	intron_variant
rs1794148391	intron_variant
rs1794148490	intron_variant
rs1794148761	intron_variant
rs1794149087	intron_variant
rs1794149273	intron_variant
rs1794149543	intron_variant
rs1794150426	intron_variant
rs1794150529	intron_variant
rs1794150624	intron_variant
rs1794151625	intron_variant
rs1794151740	intron_variant
rs1794151833	intron_variant
rs1794152027	intron_variant
rs1794152844	intron_variant
rs1794153299	intron_variant
rs1794153775	intron_variant
rs1794153917	intron_variant
rs1794154202	intron_variant
rs1794154476	intron_variant
rs1794154792	intron_variant
rs1794155537	intron_variant
rs1794155678	intron_variant
rs1794155843	intron_variant
rs1794156010	intron_variant
rs1794156349	intron_variant
rs1794156876	intron_variant
rs1794157028	intron_variant
rs1794158200	intron_variant
rs1794158406	intron_variant
rs1794158496	intron_variant
rs1794158600	intron_variant
rs1794158702	intron_variant
rs1794158854	intron_variant
rs1794158997	intron_variant
rs1794160669	intron_variant
rs1794160907	intron_variant
rs1794161084	intron_variant
rs1794161465	intron_variant
rs1794161630	intron_variant
rs1794161974	intron_variant
rs1794162134	intron_variant
rs1794162271	intron_variant
rs1794162592	intron_variant
rs1794162895	intron_variant
rs1794163646	intron_variant

rs1794163794	intron_variant
rs1794163940	intron_variant
rs1794166050	intron_variant
rs1794166464	intron_variant
rs1794166552	intron_variant
rs1794166656	intron_variant
rs1794166885	intron_variant
rs1794167070	intron_variant
rs1794167362	intron_variant
rs1794167543	intron_variant
rs1794167725	intron_variant
rs1794167940	intron_variant
rs1794168032	intron_variant
rs1794168239	intron_variant
rs1794168535	intron_variant
rs1794168864	intron_variant
rs1794169109	intron_variant
rs1794169214	intron_variant
rs1794169412	intron_variant
rs1794169744	intron_variant
rs1794169913	intron_variant
rs1794170279	intron_variant
rs1794170369	intron_variant
rs1794170468	intron_variant
rs1794170660	intron_variant
rs1794171230	intron_variant
rs1794171331	intron_variant
rs1794172068	intron_variant
rs1794172366	intron_variant
rs1794172455	intron_variant
rs1794172706	intron_variant
rs1794174124	intron_variant
rs1794175795	intron_variant
rs1794175883	intron_variant
rs1794176598	intron_variant
rs1794176707	intron_variant
rs1794176897	intron_variant
rs1794177189	intron_variant
rs1794177286	intron_variant
rs1794177591	intron_variant
rs1794178049	intron_variant
rs1794178155	intron_variant
rs1794178554	intron_variant
rs1794178830	intron_variant
rs1794178917	intron_variant
rs1794179140	intron_variant
rs1794179457	intron_variant
rs1794179549	intron_variant
rs1794180359	intron_variant
rs1794180875	intron_variant
rs1794181824	intron_variant
rs1794181964	intron_variant
rs1794182097	intron_variant
rs1794182627	intron_variant
rs1794182778	intron_variant
rs1794183837	intron_variant
rs1794184028	intron_variant
rs1794184220	intron_variant
rs1794184392	intron_variant
rs1794185106	intron_variant
rs1794185195	intron_variant
rs1794185376	intron_variant
rs1794185709	intron_variant
rs1794185847	intron_variant
rs1794185978	intron_variant
rs1794186307	intron_variant
rs1794186459	intron_variant
rs1794187627	intron_variant
rs1794187788	intron_variant
rs1794187951	intron_variant
rs1794188505	intron_variant
rs1794188652	intron_variant
rs1794189599	intron_variant
rs1794189933	intron_variant

rs1794190619	intron_variant
rs1794190783	intron_variant
rs1794191302	intron_variant
rs1794191439	intron_variant
rs1794191619	intron_variant
rs1794191776	intron_variant
rs1794192733	intron_variant
rs1794194051	intron_variant
rs1794194140	intron_variant
rs1794194237	intron_variant
rs1794194334	intron_variant
rs1794194969	intron_variant
rs1794195177	intron_variant
rs1794195661	intron_variant
rs1794196058	intron_variant
rs1794196234	intron_variant
rs1794196321	intron_variant
rs1794196411	intron_variant
rs1794196675	intron_variant
rs1794197114	intron_variant
rs1794198414	intron_variant
rs1794199422	intron_variant
rs1794199522	intron_variant
rs1794199847	intron_variant
rs1794200052	intron_variant
rs1794200330	intron_variant
rs1794201102	intron_variant
rs1794201291	intron_variant
rs1794201398	intron_variant
rs1794202063	intron_variant
rs1794202311	intron_variant
rs1794202503	intron_variant
rs1794202588	intron_variant
rs1794202768	intron_variant
rs1794202869	intron_variant
rs1794202955	intron_variant
rs1794203845	intron_variant
rs1794203935	intron_variant
rs1794204119	intron_variant
rs1794204229	intron_variant
rs1794204526	intron_variant
rs1794204736	intron_variant
rs1794204839	intron_variant
rs1794205998	intron_variant
rs1794206444	intron_variant
rs1794206564	intron_variant
rs1794206840	intron_variant
rs1794207511	intron_variant
rs1794208499	intron_variant
rs1794208705	intron_variant
rs1794209019	intron_variant
rs1794209830	intron_variant
rs1794209943	intron_variant
rs1794210063	intron_variant
rs1794210168	intron_variant
rs1794210273	intron_variant
rs1794210374	intron_variant
rs1794210527	intron_variant
rs1794210999	intron_variant
rs1794211291	intron_variant
rs1794211945	intron_variant
rs1794212293	intron_variant
rs1794212419	intron_variant
rs1794212947	intron_variant
rs1794213032	intron_variant
rs1794213387	intron_variant
rs1794214049	intron_variant
rs1794215154	intron_variant
rs1794215480	intron_variant
rs1794215608	intron_variant
rs1794215954	intron_variant
rs1794216049	intron_variant
rs1794216378	intron_variant
rs1794216558	intron_variant

rs1794217263	intron_variant
rs1794217655	intron_variant
rs1794217838	intron_variant
rs1794218432	intron_variant
rs1794220984	coding_sequence_variant,non_coding_transcript_variant,missense_variant
rs1794222447	coding_sequence_variant,non_coding_transcript_variant,synonymous_variant
rs1794223640	coding_sequence_variant,non_coding_transcript_variant,missense_variant
rs1794225549	coding_sequence_variant,stop_gained,non_coding_transcript_variant
rs1794227312	splice_donor_variant
rs1794228357	intron_variant
rs1794231327	intron_variant
rs1794232818	intron_variant
rs1794234130	intron_variant
rs1794234483	intron_variant
rs1794241396	coding_sequence_variant,non_coding_transcript_variant,synonymous_variant
rs1794241813	coding_sequence_variant,non_coding_transcript_variant,missense_variant
rs1794243276	coding_sequence_variant,non_coding_transcript_variant,synonymous_variant
rs1794248741	intron_variant
rs1794248908	intron_variant
rs1794250761	intron_variant
rs1794250977	intron_variant
rs1794251260	intron_variant
rs1794251450	intron_variant
rs1794252027	intron_variant
rs1794252118	intron_variant
rs1794252216	intron_variant
rs1794252534	intron_variant
rs1794252907	intron_variant
rs1794253087	intron_variant
rs1794253484	intron_variant
rs1794253570	intron_variant
rs1794253669	intron_variant
rs1794254060	intron_variant
rs1794254808	intron_variant
rs1794255174	intron_variant
rs1794255401	intron_variant
rs1794255498	intron_variant
rs1794255604	intron_variant
rs1794255690	intron_variant
rs1794256148	intron_variant
rs1794256246	intron_variant
rs1794256344	intron_variant
rs1794256554	intron_variant
rs1794256963	intron_variant
rs1794257793	intron_variant
rs1794257901	intron_variant
rs1794258134	intron_variant
rs1794258241	intron_variant
rs1794258352	intron_variant
rs1794258682	intron_variant
rs1794259567	intron_variant
rs1794260611	intron_variant
rs1794261318	intron_variant
rs1794261478	intron_variant
rs1794261838	intron_variant
rs1794262496	intron_variant
rs1794263341	intron_variant
rs1794263448	intron_variant
rs1794264121	intron_variant
rs1794264942	intron_variant
rs1794265193	intron_variant
rs1794265293	intron_variant
rs1794265401	intron_variant
rs1794265646	intron_variant
rs1794265737	intron_variant
rs1794266107	intron_variant
rs1794266331	intron_variant
rs1794266706	intron_variant
rs1794266806	intron_variant
rs1794266924	intron_variant
rs1794267132	intron_variant
rs1794267250	intron_variant
rs1794267997	intron_variant
rs1794268098	intron_variant

rs1794268306	intron_variant
rs1794268392	intron_variant
rs1794269055	intron_variant
rs1794269267	intron_variant
rs1794269374	intron_variant
rs1794269617	intron_variant
rs1794270876	intron_variant
rs1794270969	intron_variant
rs1794275099	coding_sequence_variant,non_coding_transcript_variant,missense_variant
rs1794275336	coding_sequence_variant,non_coding_transcript_variant,missense_variant
rs1794276678	coding_sequence_variant,non_coding_transcript_variant,missense_variant
rs1794277835	coding_sequence_variant,non_coding_transcript_variant,synonymous_variant
rs1794279085	coding_sequence_variant,non_coding_transcript_variant,synonymous_variant
rs1794279787	coding_sequence_variant,non_coding_transcript_variant,synonymous_variant
rs1794280473	intron_variant
rs1794280566	intron_variant
rs1794280747	intron_variant
rs1794280939	intron_variant
rs1794281262	intron_variant
rs1794282209	intron_variant
rs1794282631	intron_variant
rs1794283102	intron_variant
rs1794283309	intron_variant
rs1794283547	intron_variant
rs1794283629	intron_variant
rs1794283718	intron_variant
rs1794284220	intron_variant
rs1794284441	intron_variant
rs1794284679	intron_variant
rs1794285007	intron_variant
rs1794285633	intron_variant
rs1794285839	intron_variant
rs1794286026	intron_variant
rs1794286139	intron_variant
rs1794286585	intron_variant
rs1794287028	intron_variant
rs1794287117	intron_variant
rs1794287334	intron_variant
rs1794287656	intron_variant
rs1794287754	intron_variant
rs1794287943	intron_variant
rs1794288135	intron_variant
rs1794288242	intron_variant
rs1794288348	intron_variant
rs1794288450	intron_variant
rs1794288583	intron_variant
rs1794288773	intron_variant
rs1794289050	intron_variant
rs1794289409	intron_variant
rs1794289677	intron_variant
rs1794289918	intron_variant
rs1794290488	intron_variant
rs1794291315	intron_variant
rs1794292084	intron_variant
rs1794292211	intron_variant
rs1794292511	intron_variant
rs1794294577	intron_variant
rs1794294929	intron_variant
rs1794295329	intron_variant
rs1794295626	intron_variant
rs1794295726	intron_variant
rs1794295926	intron_variant
rs1794296379	intron_variant
rs1794296619	intron_variant
rs1794296785	intron_variant
rs1794297069	intron_variant
rs1794297209	intron_variant
rs1794297367	intron_variant
rs1794298829	intron_variant
rs1794300421	intron_variant
rs1794300837	intron_variant
rs1794301325	intron_variant
rs1794301692	intron_variant
rs1794302168	intron_variant

rs1794302546	intron_variant
rs1794302646	intron_variant
rs1794303398	intron_variant
rs1794304017	intron_variant
rs1794304127	intron_variant
rs1794304425	intron_variant
rs1794304627	intron_variant
rs1794305217	intron_variant
rs1794305411	intron_variant
rs1794306292	intron_variant
rs1794306386	intron_variant
rs1794306643	intron_variant
rs1794307491	coding_sequence_variant,intron_variant,synonymous_variant
rs1794307675	coding_sequence_variant,intron_variant,synonymous_variant
rs1794308172	coding_sequence_variant,intron_variant,missense_variant
rs1794308770	coding_sequence_variant,intron_variant,synonymous_variant
rs1794308869	coding_sequence_variant,intron_variant,missense_variant
rs1794309667	coding_sequence_variant,intron_variant,synonymous_variant
rs1794310643	coding_sequence_variant,intron_variant,synonymous_variant
rs1794310980	coding_sequence_variant,intron_variant,synonymous_variant
rs1794311792	coding_sequence_variant,intron_variant,synonymous_variant
rs1794311908	coding_sequence_variant,intron_variant,frameshift_variant
rs1794314081	intron_variant
rs1794314760	intron_variant
rs1794314992	intron_variant
rs1794316644	intron_variant
rs1794317060	intron_variant
rs1794317152	intron_variant
rs1794317242	intron_variant
rs1794318084	intron_variant
rs1794318272	intron_variant
rs1794318390	intron_variant
rs1794318508	intron_variant
rs1794319134	intron_variant
rs1794319223	intron_variant
rs1794319627	intron_variant
rs1794319796	intron_variant
rs1794320104	intron_variant
rs1794320597	intron_variant
rs1794320954	intron_variant
rs1794321142	intron_variant
rs1794321619	intron_variant
rs1794321706	intron_variant
rs1794321903	intron_variant
rs1794324023	intron_variant
rs1794324115	intron_variant
rs1794324498	intron_variant
rs1794324717	intron_variant
rs1794324801	intron_variant
rs1794325064	intron_variant
rs1794325173	intron_variant
rs1794325615	intron_variant
rs1794326143	intron_variant
rs1794327004	intron_variant
rs1794327197	intron_variant
rs1794327494	intron_variant
rs1794327792	intron_variant
rs1794327890	intron_variant
rs1794328384	intron_variant
rs1794328476	intron_variant
rs1794328657	intron_variant
rs1794328840	intron_variant
rs1794328938	intron_variant
rs1794329333	intron_variant
rs1794329468	intron_variant
rs1794329696	intron_variant
rs1794330203	intron_variant
rs1794330898	intron_variant
rs1794331874	intron_variant
rs1794332011	intron_variant
rs1794332161	intron_variant
rs1794332285	intron_variant
rs1794332402	intron_variant
rs1794332686	intron_variant

rs1794333224	intron_variant
rs1794335672	intron_variant
rs1794335991	intron_variant
rs1794338828	intron_variant
rs1794338992	intron_variant
rs1794339180	intron_variant
rs1794339280	intron_variant
rs1794339377	intron_variant
rs1794339458	intron_variant
rs1794339563	intron_variant
rs1794339652	intron_variant
rs1794339815	intron_variant
rs1794340239	intron_variant
rs1794340340	intron_variant
rs1794341084	intron_variant
rs1794341223	intron_variant
rs1794341928	intron_variant
rs1794342068	intron_variant
rs1794342207	intron_variant
rs1794342768	intron_variant
rs1794343810	intron_variant
rs1794344107	intron_variant
rs1794344268	intron_variant
rs1794344398	intron_variant
rs1794344827	intron_variant
rs1794345471	intron_variant
rs1794345752	intron_variant
rs1794346520	intron_variant
rs1794346795	intron_variant
rs1794347079	intron_variant
rs1794347839	intron_variant
rs1794348789	intron_variant
rs1794348887	intron_variant
rs1794349547	intron_variant
rs1794349678	intron_variant
rs1794350379	intron_variant
rs1794350529	intron_variant
rs1794350660	intron_variant
rs1794351513	intron_variant
rs1794351893	intron_variant
rs1794357189	intron_variant
rs1794357515	intron_variant
rs1794357669	intron_variant
rs1794358509	intron_variant
rs1794359274	intron_variant
rs1794359907	intron_variant
rs1794360424	intron_variant
rs1794360735	intron_variant
rs1794361107	intron_variant
rs1794361919	intron_variant
rs1794363429	intron_variant
rs1794363845	intron_variant
rs1794363972	intron_variant
rs1794364100	intron_variant
rs1794364251	intron_variant
rs1794364901	intron_variant
rs1794365283	intron_variant
rs1794365410	intron_variant
rs1794365647	intron_variant
rs1794365763	intron_variant
rs1794365861	intron_variant
rs1794365957	intron_variant
rs1794366070	intron_variant
rs1794366462	intron_variant
rs1794366936	intron_variant
rs1794367057	intron_variant
rs1794367359	intron_variant
rs1794367587	intron_variant
rs1794368164	intron_variant
rs1794368565	intron_variant
rs1794368719	intron_variant
rs1794370491	intron_variant
rs1794370980	intron_variant
rs1794371268	intron_variant

rs1794371541	intron_variant
rs1794371655	intron_variant
rs1794371973	intron_variant
rs1794372746	intron_variant
rs1794373050	intron_variant
rs1794373385	intron_variant
rs1794373593	intron_variant
rs1794373718	intron_variant
rs1794373990	intron_variant
rs1794374218	intron_variant
rs1794374780	intron_variant
rs1794375495	intron_variant
rs1794375841	intron_variant
rs1794378353	intron_variant
rs1794378680	intron_variant
rs1794379006	intron_variant
rs1794379672	intron_variant
rs1794379776	intron_variant
rs1794379876	intron_variant
rs1794380079	intron_variant
rs1794380360	intron_variant
rs1794380527	intron_variant
rs1794380816	intron_variant
rs1794381019	intron_variant
rs1794381235	intron_variant
rs1794381336	intron_variant
rs1794381667	intron_variant
rs1794381874	intron_variant
rs1794381962	intron_variant
rs1794382134	intron_variant
rs1794382229	intron_variant
rs1794382333	intron_variant
rs1794382634	intron_variant
rs1794382736	intron_variant
rs1794382928	intron_variant
rs1794383013	intron_variant
rs1794383103	intron_variant
rs1794383214	intron_variant
rs1794383436	intron_variant
rs1794383537	intron_variant
rs1794383623	intron_variant
rs1794383845	intron_variant
rs1794384022	intron_variant
rs1794384430	intron_variant
rs1794384508	intron_variant
rs1794384741	intron_variant
rs1794384828	intron_variant
rs1794385121	intron_variant
rs1794385532	intron_variant
rs1794385862	intron_variant
rs1794385959	intron_variant
rs1794386046	intron_variant
rs1794386345	intron_variant
rs1794387054	intron_variant
rs1794387419	intron_variant
rs1794387727	intron_variant
rs1794388414	intron_variant
rs1794388729	intron_variant
rs1794389308	intron_variant
rs1794389573	intron_variant
rs1794389757	intron_variant
rs1794390143	intron_variant
rs1794390657	intron_variant
rs1794390837	intron_variant
rs1794391056	intron_variant
rs1794391252	intron_variant
rs1794391653	intron_variant
rs1794391971	intron_variant
rs1794392212	intron_variant
rs1794392319	intron_variant
rs1794392405	intron_variant
rs1794392649	intron_variant
rs1794392754	intron_variant
rs1794392948	intron_variant

rs1794393174	intron_variant
rs1794393360	intron_variant
rs1794393635	intron_variant
rs1794393730	intron_variant
rs1794394013	intron_variant
rs1794395272	intron_variant
rs1794395356	intron_variant
rs1794395439	intron_variant
rs1794395532	intron_variant
rs1794395728	intron_variant
rs1794395831	intron_variant
rs1794395930	intron_variant
rs1794396629	intron_variant
rs1794397127	intron_variant
rs1794397424	intron_variant
rs1794397564	intron_variant
rs1794397704	intron_variant
rs1794397846	intron_variant
rs1794397984	intron_variant
rs1794398463	intron_variant
rs1794398617	intron_variant
rs1794398946	intron_variant
rs1794399332	intron_variant
rs1794399549	intron_variant
rs1794400019	intron_variant
rs1794400243	intron_variant
rs1794400693	intron_variant
rs1794401329	intron_variant
rs1794401421	intron_variant
rs1794401524	intron_variant
rs1794401630	intron_variant
rs1794402140	intron_variant
rs1794402674	intron_variant
rs1794402774	intron_variant
rs1794402965	intron_variant
rs1794403464	intron_variant
rs1794403700	intron_variant
rs1794403794	intron_variant
rs1794403996	intron_variant
rs1794404447	intron_variant
rs1794404551	intron_variant
rs1794404656	intron_variant
rs1794404744	intron_variant
rs1794406425	intron_variant
rs1794406647	intron_variant
rs1794407010	intron_variant
rs1794408102	intron_variant
rs1794408200	intron_variant
rs1794408491	intron_variant
rs1794408658	intron_variant
rs1794409010	intron_variant
rs1794409170	intron_variant
rs1794410464	intron_variant
rs1794410957	intron_variant
rs1794411114	intron_variant
rs1794411426	intron_variant
rs1794411695	intron_variant
rs1794411836	intron_variant
rs1794412467	intron_variant
rs1794413000	intron_variant
rs1794414338	intron_variant
rs1794414503	intron_variant
rs1794414762	intron_variant
rs1794415436	intron_variant
rs1794415841	intron_variant
rs1794415989	intron_variant
rs1794416710	intron_variant
rs1794417347	intron_variant
rs1794417487	intron_variant
rs1794417675	intron_variant
rs1794417819	intron_variant
rs1794417936	intron_variant
rs1794418318	intron_variant
rs1794419664	intron_variant

rs1794420315	intron_variant
rs1794420415	intron_variant
rs1794420555	intron_variant
rs1794421455	intron_variant
rs1794421809	intron_variant
rs1794422088	intron_variant
rs1794422431	intron_variant
rs1794422575	intron_variant
rs1794423442	intron_variant
rs1794423595	intron_variant
rs1794423772	intron_variant
rs1794424107	intron_variant
rs1794424277	intron_variant
rs1794424441	intron_variant
rs1794424600	intron_variant
rs1794424972	intron_variant
rs1794425702	intron_variant
rs1794426158	intron_variant
rs1794428013	intron_variant
rs1794429179	intron_variant
rs1794429311	intron_variant
rs1794429582	intron_variant
rs1794430101	intron_variant
rs1794430314	intron_variant
rs1794430644	intron_variant
rs1794431327	intron_variant
rs1794431424	intron_variant
rs1794431715	intron_variant
rs1794432256	intron_variant
rs1794432748	intron_variant
rs1794432851	intron_variant
rs1794432938	intron_variant
rs1794433402	intron_variant
rs1794433671	intron_variant
rs1794433778	intron_variant
rs1794433887	intron_variant
rs1794434060	intron_variant
rs1794434156	intron_variant
rs1794434459	intron_variant
rs1794434566	intron_variant
rs1794434742	intron_variant
rs1794434918	intron_variant
rs1794435013	intron_variant
rs1794435115	intron_variant
rs1794435220	intron_variant
rs1794435656	intron_variant
rs1794435956	intron_variant
rs1794436594	intron_variant
rs1794436686	intron_variant
rs1794436974	intron_variant
rs1794437078	intron_variant
rs1794437265	intron_variant
rs1794437739	intron_variant
rs1794437902	intron_variant
rs1794438097	intron_variant
rs1794438378	intron_variant
rs1794438496	intron_variant
rs1794438762	intron_variant
rs1794438860	intron_variant
rs1794438951	intron_variant
rs1794439279	intron_variant
rs1794439374	intron_variant
rs1794439465	intron_variant
rs1794439860	intron_variant
rs1794440081	intron_variant
rs1794440281	intron_variant
rs1794440473	intron_variant
rs1794440560	intron_variant
rs1794440754	intron_variant
rs1794440932	intron_variant
rs1794441102	intron_variant
rs1794441185	intron_variant
rs1794442009	intron_variant
rs1794442195	intron_variant

rs1794442823	intron_variant
rs1794442922	intron_variant
rs1794443409	intron_variant
rs1794443592	intron_variant
rs1794443774	intron_variant
rs1794444360	intron_variant
rs1794445608	intron_variant
rs1794445724	intron_variant
rs1794445915	intron_variant
rs1794446111	intron_variant
rs1794446515	intron_variant
rs1794447035	intron_variant
rs1794447336	intron_variant
rs1794447443	intron_variant
rs1794447893	intron_variant
rs1794448160	intron_variant
rs1794448307	intron_variant
rs1794450027	intron_variant
rs1794450336	intron_variant
rs1794450489	intron_variant
rs1794450649	intron_variant
rs1794450984	intron_variant
rs1794451136	intron_variant
rs1794451288	intron_variant
rs1794451834	intron_variant
rs1794452416	intron_variant
rs1794452564	intron_variant
rs1794452709	intron_variant
rs1794452845	intron_variant
rs1794453140	intron_variant
rs1794454418	intron_variant
rs1794455304	intron_variant
rs1794457657	coding_sequence_variant,non_coding_transcript_variant,missense_variant
rs1794458829	coding_sequence_variant,non_coding_transcript_variant,synonymous_variant
rs1794459560	coding_sequence_variant,non_coding_transcript_variant,missense_variant
rs1794460713	coding_sequence_variant,non_coding_transcript_variant,missense_variant
rs1794462604	coding_sequence_variant,non_coding_transcript_variant,synonymous_variant
rs1794464524	coding_sequence_variant,non_coding_transcript_variant,synonymous_variant
rs1794466783	coding_sequence_variant,non_coding_transcript_variant,frameshift_variant
rs1794467654	coding_sequence_variant,non_coding_transcript_variant,missense_variant
rs1794476422	intron_variant
rs1794476570	intron_variant
rs1794476856	intron_variant
rs1794478554	intron_variant
rs1794480401	intron_variant
rs1794480879	intron_variant
rs1794481193	intron_variant
rs1794481491	intron_variant
rs1794482461	intron_variant
rs1794482623	intron_variant
rs1794482725	genic_upstream_transcript_variant,intron_variant
rs1794483362	intron_variant
rs1794484054	intron_variant
rs1794484186	intron_variant
rs1794485368	intron_variant
rs1794485676	intron_variant
rs1794485959	intron_variant
rs1794486214	intron_variant
rs1794486774	intron_variant
rs1794487739	intron_variant
rs1794488320	intron_variant
rs1794488423	intron_variant
rs1794489080	intron_variant
rs1794489219	intron_variant
rs1794490399	intron_variant
rs1794490519	intron_variant
rs1794490641	intron_variant
rs1794491321	intron_variant
rs1794491648	intron_variant
rs1794491978	intron_variant
rs1794492121	intron_variant
rs1794494931	intron_variant
rs1794495082	intron_variant
rs1794495858	intron_variant

rs1794496341	intron_variant
rs1794496781	intron_variant
rs1794496976	intron_variant
rs1794497184	intron_variant
rs1794497505	intron_variant
rs1794497640	intron_variant
rs1794498145	intron_variant
rs1794498433	intron_variant
rs1794498585	intron_variant
rs1794498901	intron_variant
rs1794499561	intron_variant
rs1794500061	intron_variant
rs1794500244	intron_variant
rs1794500395	intron_variant
rs1794500863	intron_variant
rs1794500995	intron_variant
rs1794501845	intron_variant
rs1794502000	intron_variant
rs1794503504	intron_variant
rs1794503813	intron_variant
rs1794503947	intron_variant
rs1794504143	intron_variant
rs1794504428	intron_variant
rs1794504845	intron_variant
rs1794505030	intron_variant
rs1794505326	intron_variant
rs1794505469	intron_variant
rs1794505758	intron_variant
rs1794506066	intron_variant
rs1794506563	intron_variant
rs1794507057	intron_variant
rs1794507191	intron_variant
rs1794507832	intron_variant
rs1794508108	intron_variant
rs1794508423	intron_variant
rs1794508562	intron_variant
rs1794508863	intron_variant
rs1794509634	intron_variant
rs1794509920	intron_variant
rs1794510842	intron_variant
rs1794510967	intron_variant
rs1794511755	intron_variant
rs1794512057	intron_variant
rs1794512175	intron_variant
rs1794512993	intron_variant
rs1794513147	intron_variant
rs1794513438	intron_variant
rs1794514552	intron_variant
rs1794515313	intron_variant
rs1794515594	intron_variant
rs1794516347	intron_variant
rs1794516703	intron_variant
rs1794516845	intron_variant
rs1794516982	intron_variant
rs1794517127	intron_variant
rs1794517267	intron_variant
rs1794517785	intron_variant
rs1794517951	intron_variant
rs1794519859	intron_variant
rs1794520007	intron_variant
rs1794520923	intron_variant
rs1794521821	intron_variant
rs1794523573	intron_variant
rs1794523710	intron_variant
rs1794523847	intron_variant
rs1794524471	intron_variant
rs1794524618	intron_variant
rs1794525356	intron_variant
rs1794525513	intron_variant
rs1794525829	intron_variant
rs1794526634	intron_variant
rs1794526994	intron_variant
rs1794528178	intron_variant
rs1794529591	intron_variant

rs1794529738	intron_variant
rs1794529861	intron_variant
rs1794530717	intron_variant
rs1794530843	intron_variant
rs1794530997	intron_variant
rs1794531513	intron_variant
rs1794531640	intron_variant
rs1794532279	intron_variant
rs1794533550	intron_variant
rs1794534079	intron_variant
rs1794534246	intron_variant
rs1794536915	intron_variant
rs1794537631	intron_variant
rs1794537788	intron_variant
rs1794538099	intron_variant
rs1794538590	intron_variant
rs1794538765	intron_variant
rs1794538919	intron_variant
rs1794540493	intron_variant
rs1794540653	intron_variant
rs1794540820	intron_variant
rs1794540981	intron_variant
rs1794541316	intron_variant
rs1794543545	intron_variant
rs1794543694	intron_variant
rs1794543866	intron_variant
rs1794544413	intron_variant
rs1794547073	intron_variant
rs1794547230	intron_variant
rs1794547698	intron_variant
rs1794548036	intron_variant
rs1794548348	intron_variant
rs1794548700	intron_variant
rs1794549026	intron_variant
rs1794549440	intron_variant
rs1794549762	intron_variant
rs1794550415	intron_variant
rs1794551176	intron_variant
rs1794551632	intron_variant
rs1794552102	intron_variant
rs1794552255	intron_variant
rs1794552415	intron_variant
rs1794552582	intron_variant
rs1794553417	intron_variant
rs1794553764	intron_variant
rs1794553940	intron_variant
rs1794555176	intron_variant
rs1794555329	intron_variant
rs1794556340	intron_variant
rs1794556511	intron_variant
rs1794557108	intron_variant
rs1794557267	intron_variant
rs1794557823	intron_variant
rs1794557985	intron_variant
rs1794558132	intron_variant
rs1794558554	intron_variant
rs1794559160	intron_variant
rs1794559451	intron_variant
rs1794559743	intron_variant
rs1794560280	intron_variant
rs1794560419	intron_variant
rs1794560808	intron_variant
rs1794561460	intron_variant
rs1794561605	intron_variant
rs1794561788	intron_variant
rs1794561972	intron_variant
rs1794562155	intron_variant
rs1794562366	intron_variant
rs1794562467	intron_variant
rs1794562748	intron_variant
rs1794563213	intron_variant
rs1794563355	intron_variant
rs1794563852	intron_variant
rs1794563993	intron_variant

rs1794564483	intron_variant
rs1794564566	intron_variant
rs1794564757	intron_variant
rs1794565220	intron_variant
rs1794565773	intron_variant
rs1794566709	intron_variant
rs1794567033	intron_variant
rs1794567212	intron_variant
rs1794567594	intron_variant
rs1794567910	intron_variant
rs1794568190	intron_variant
rs1794568487	intron_variant
rs1794568797	intron_variant
rs1794568891	intron_variant
rs1794568991	intron_variant
rs1794569267	intron_variant
rs1794569555	intron_variant
rs1794569723	intron_variant
rs1794569846	intron_variant
rs1794570141	intron_variant
rs1794570507	intron_variant
rs1794570700	intron_variant
rs1794570801	intron_variant
rs1794570965	intron_variant
rs1794571209	intron_variant
rs1794571292	intron_variant
rs1794571376	intron_variant
rs1794571782	intron_variant
rs1794571944	intron_variant
rs1794572259	intron_variant
rs1794573039	intron_variant
rs1794573750	intron_variant
rs1794573883	intron_variant
rs1794574315	intron_variant
rs1794574408	intron_variant
rs1794574571	intron_variant
rs1794574756	intron_variant
rs1794574974	intron_variant
rs1794575193	intron_variant
rs1794575277	intron_variant
rs1794575470	intron_variant
rs1794575563	intron_variant
rs1794575764	intron_variant
rs1794575845	intron_variant
rs1794576355	intron_variant
rs1794576446	intron_variant
rs1794576833	intron_variant
rs1794577117	intron_variant
rs1794580169	intron_variant
rs1794580457	intron_variant
rs1794581052	intron_variant
rs1794581146	intron_variant
rs1794584342	intron_variant
rs1794584549	intron_variant
rs1794585472	intron_variant
rs1794585575	intron_variant
rs1794586629	intron_variant
rs1794586934	intron_variant
rs1794587215	intron_variant
rs1794588195	intron_variant
rs1794588645	intron_variant
rs1794588840	intron_variant
rs1794589034	intron_variant
rs1794589235	intron_variant
rs1794590155	intron_variant
rs1794590562	intron_variant
rs1794590873	intron_variant
rs1794591069	intron_variant
rs1794591447	intron_variant
rs1794591940	intron_variant
rs1794592090	intron_variant
rs1794592373	intron_variant
rs1794592517	intron_variant
rs1794592802	intron_variant

rs1794593856	intron_variant
rs1794593946	intron_variant
rs1794594439	intron_variant
rs1794594592	intron_variant
rs1794594693	intron_variant
rs1794594942	intron_variant
rs1794595420	intron_variant
rs1794595524	intron_variant
rs1794595810	intron_variant
rs1794595907	intron_variant
rs1794596551	intron_variant
rs1794596876	intron_variant
rs1794598137	intron_variant
rs1794598579	intron_variant
rs1794598891	intron_variant
rs1794599159	intron_variant
rs1794599901	intron_variant
rs1794600105	intron_variant
rs1794600205	intron_variant
rs1794600969	intron_variant
rs1794601122	intron_variant
rs1794601548	intron_variant
rs1794601655	intron_variant
rs1794602295	intron_variant
rs1794602411	intron_variant
rs1794602498	intron_variant
rs1794602711	intron_variant
rs1794602816	intron_variant
rs1794602936	intron_variant
rs1794603119	intron_variant
rs1794603227	intron_variant
rs1794603433	intron_variant
rs1794603718	intron_variant
rs1794603816	intron_variant
rs1794604109	intron_variant
rs1794604251	intron_variant
rs1794604412	intron_variant
rs1794604560	intron_variant
rs1794604872	intron_variant
rs1794605025	intron_variant
rs1794605182	intron_variant
rs1794605341	intron_variant
rs1794606029	intron_variant
rs1794606507	intron_variant
rs1794606875	intron_variant
rs1794607443	intron_variant
rs1794607534	intron_variant
rs1794607649	intron_variant
rs1794608095	intron_variant
rs1794608669	intron_variant
rs1794608788	intron_variant
rs1794608923	intron_variant
rs1794609061	intron_variant
rs1794609202	intron_variant
rs1794609757	intron_variant
rs1794609893	intron_variant
rs1794610648	intron_variant
rs1794610767	intron_variant
rs1794612284	intron_variant
rs1794613376	intron_variant
rs1794613510	intron_variant
rs1794614367	intron_variant
rs1794614501	intron_variant
rs1794614735	intron_variant
rs1794615066	intron_variant
rs1794615168	intron_variant
rs1794615508	intron_variant
rs1794615654	intron_variant
rs1794616075	intron_variant
rs1794617261	intron_variant
rs1794617724	intron_variant
rs1794618041	intron_variant
rs1794618168	intron_variant
rs1794618276	intron_variant

rs1794618479	intron_variant
rs1794618574	intron_variant
rs1794619417	intron_variant
rs1794619538	intron_variant
rs1794620318	intron_variant
rs1794620418	intron_variant
rs1794621151	intron_variant
rs1794621258	intron_variant
rs1794621384	intron_variant
rs1794621498	intron_variant
rs1794621630	intron_variant
rs1794622350	intron_variant
rs1794622625	intron_variant
rs1794622998	intron_variant
rs1794623122	intron_variant
rs1794623250	intron_variant
rs1794623362	intron_variant
rs1794623457	intron_variant
rs1794623650	intron_variant
rs1794623917	intron_variant
rs1794624446	intron_variant
rs1794624536	intron_variant
rs1794625039	intron_variant
rs1794625196	intron_variant
rs1794625329	intron_variant
rs1794625474	intron_variant
rs1794626776	intron_variant
rs1794627168	intron_variant
rs1794628094	intron_variant
rs1794628230	intron_variant
rs1794628376	intron_variant
rs1794629008	intron_variant
rs1794629249	intron_variant
rs1794629745	intron_variant
rs1794630697	intron_variant
rs1794631441	intron_variant
rs1794631568	intron_variant
rs1794632008	intron_variant
rs1794632085	intron_variant
rs1794632445	intron_variant
rs1794632552	intron_variant
rs1794632818	intron_variant
rs1794633117	intron_variant
rs1794633239	intron_variant
rs1794633388	intron_variant
rs1794633531	intron_variant
rs1794634369	intron_variant
rs1794634510	intron_variant
rs1794634973	intron_variant
rs1794635721	intron_variant
rs1794635890	intron_variant
rs1794636055	intron_variant
rs1794636140	intron_variant
rs1794636729	intron_variant
rs1794637418	intron_variant
rs1794637506	intron_variant
rs1794637759	intron_variant
rs1794637874	intron_variant
rs1794638489	intron_variant
rs1794639131	intron_variant
rs1794639668	intron_variant
rs1794639763	intron_variant
rs1794640006	intron_variant
rs1794640402	intron_variant
rs1794640481	intron_variant
rs1794640816	intron_variant
rs1794640913	intron_variant
rs1794641096	intron_variant
rs1794641178	intron_variant
rs1794641534	intron_variant
rs1794642068	intron_variant
rs1794642160	intron_variant
rs1794642258	intron_variant
rs1794642676	intron_variant

rs1794642879	intron_variant
rs1794643046	intron_variant
rs1794643153	intron_variant
rs1794643214	intron_variant
rs1794643724	intron_variant
rs1794644418	intron_variant
rs1794644854	intron_variant
rs1794645357	intron_variant
rs1794645531	intron_variant
rs1794645923	intron_variant
rs1794646025	intron_variant
rs1794646111	intron_variant
rs1794646278	intron_variant
rs1794646501	intron_variant
rs1794646841	intron_variant
rs1794646922	intron_variant
rs1794647023	intron_variant
rs1794647130	intron_variant
rs1794647408	intron_variant
rs1794648016	intron_variant
rs1794648597	intron_variant
rs1794648681	intron_variant
rs1794648907	intron_variant
rs1794649256	intron_variant
rs1794649406	intron_variant
rs1794649672	intron_variant
rs1794649837	intron_variant
rs1794649956	intron_variant
rs1794650038	intron_variant
rs1794650127	intron_variant
rs1794650326	intron_variant
rs1794650807	intron_variant
rs1794650975	intron_variant
rs1794651489	intron_variant
rs1794651569	intron_variant
rs1794651702	intron_variant
rs1794651837	intron_variant
rs1794651974	intron_variant
rs1794654124	intron_variant
rs1794654273	intron_variant
rs1794654449	intron_variant
rs1794654592	intron_variant
rs1794656689	intron_variant
rs1794658097	intron_variant
rs1794658335	intron_variant
rs1794659090	intron_variant
rs1794659544	intron_variant
rs1794659824	intron_variant
rs1794660835	intron_variant
rs1794660952	intron_variant
rs1794661220	intron_variant
rs1794661757	intron_variant
rs1794662470	intron_variant
rs1794662602	intron_variant
rs1794663242	intron_variant
rs1794663379	intron_variant
rs1794663644	intron_variant
rs1794663762	intron_variant
rs1794663905	intron_variant
rs1794664475	intron_variant
rs1794664929	intron_variant
rs1794665163	intron_variant
rs1794666339	intron_variant
rs1794666478	intron_variant
rs1794666867	intron_variant
rs1794667679	intron_variant
rs1794667986	intron_variant
rs1794668559	intron_variant
rs1794669241	intron_variant
rs1794669751	intron_variant
rs1794670212	intron_variant
rs1794670683	intron_variant
rs1794670810	intron_variant
rs1794670929	intron_variant

rs1794671177	intron_variant
rs1794671310	intron_variant
rs1794671557	intron_variant
rs1794671767	intron_variant
rs1794671935	intron_variant
rs1794672187	intron_variant
rs1794672317	intron_variant
rs1794672401	intron_variant
rs1794672494	intron_variant
rs1794672597	intron_variant
rs1794673002	intron_variant
rs1794673082	intron_variant
rs1794673243	intron_variant
rs1794673333	intron_variant
rs1794673569	intron_variant
rs1794673760	intron_variant
rs1794673845	intron_variant
rs1794674460	intron_variant
rs1794674529	intron_variant
rs1794674623	intron_variant
rs1794675074	intron_variant
rs1794675485	intron_variant
rs1794675632	intron_variant
rs1794676357	intron_variant
rs1794677164	intron_variant
rs1794677715	intron_variant
rs1794677796	intron_variant
rs1794678152	intron_variant
rs1794678211	intron_variant
rs1794678299	intron_variant
rs1794678448	intron_variant
rs1794678510	intron_variant
rs1794678735	intron_variant
rs1794679030	intron_variant
rs1794679295	intron_variant
rs1794679458	intron_variant
rs1794679822	intron_variant
rs1794680288	intron_variant
rs1794680446	intron_variant
rs1794681334	intron_variant
rs1794681640	intron_variant
rs1794682032	intron_variant
rs1794682268	intron_variant
rs1794682343	intron_variant
rs1794682419	intron_variant
rs1794682536	intron_variant
rs1794682615	intron_variant
rs1794682999	intron_variant
rs1794684150	intron_variant
rs1794684239	intron_variant
rs1794684571	intron_variant
rs1794684646	intron_variant
rs1794684731	intron_variant
rs1794684990	intron_variant
rs1794685369	intron_variant
rs1794685555	intron_variant
rs1794685743	intron_variant
rs1794685999	intron_variant
rs1794686132	intron_variant
rs1794688891	intron_variant
rs1794689075	intron_variant
rs1794689251	intron_variant
rs1794689353	intron_variant
rs1794689837	intron_variant
rs1794690090	intron_variant
rs1794690214	intron_variant
rs1794690837	intron_variant
rs1794691261	intron_variant
rs1794691979	intron_variant
rs1794692568	intron_variant
rs1794693037	intron_variant
rs1794693469	intron_variant
rs1794694403	intron_variant
rs1794694529	intron_variant

rs1794694668	intron_variant
rs1794694786	intron_variant
rs1794695030	intron_variant
rs1794696367	intron_variant
rs1794696967	intron_variant
rs1794697111	intron_variant
rs1794697243	intron_variant
rs1794697673	intron_variant
rs1794697815	intron_variant
rs1794698256	intron_variant
rs1794698413	intron_variant
rs1794699278	intron_variant
rs1794699431	intron_variant
rs1794699848	intron_variant
rs1794700465	intron_variant
rs1794701208	intron_variant
rs1794701351	intron_variant
rs1794702854	intron_variant
rs1794703168	intron_variant
rs1794703304	intron_variant
rs1794703583	intron_variant
rs1794704526	intron_variant
rs1794704664	intron_variant
rs1794704786	intron_variant
rs1794705073	intron_variant
rs1794705439	intron_variant
rs1794705586	intron_variant
rs1794706206	intron_variant
rs1794706354	intron_variant
rs1794706443	intron_variant
rs1794706642	intron_variant
rs1794707273	intron_variant
rs1794707357	intron_variant
rs1794707663	intron_variant
rs1794707855	intron_variant
rs1794708098	intron_variant
rs1794708828	intron_variant
rs1794709588	intron_variant
rs1794709844	intron_variant
rs1794710087	intron_variant
rs1794710184	intron_variant
rs1794710278	intron_variant
rs1794710478	intron_variant
rs1794710875	intron_variant
rs1794711012	intron_variant
rs1794711305	intron_variant
rs1794711469	intron_variant
rs1794711545	intron_variant
rs1794711614	intron_variant
rs1794711711	intron_variant
rs1794711876	intron_variant
rs1794712133	intron_variant
rs1794712295	intron_variant
rs1794713424	intron_variant
rs1794713956	intron_variant
rs1794714434	intron_variant
rs1794714686	intron_variant
rs1794715057	intron_variant
rs1794715227	intron_variant
rs1794715396	intron_variant
rs1794715636	intron_variant
rs1794715725	intron_variant
rs1794715832	intron_variant
rs1794716500	intron_variant
rs1794716637	intron_variant
rs1794717054	intron_variant
rs1794717906	intron_variant
rs1794718807	intron_variant
rs1794719998	intron_variant
rs1794720121	intron_variant
rs1794720268	intron_variant
rs1794720574	intron_variant
rs1794721412	intron_variant
rs1794721547	intron_variant

rs1794721835	intron_variant
rs1794722005	intron_variant
rs1794722934	intron_variant
rs1794723431	intron_variant
rs1794723986	intron_variant
rs1794724591	intron_variant
rs1794724707	intron_variant
rs1794725272	intron_variant
rs1794725557	intron_variant
rs1794726836	intron_variant
rs1794727187	intron_variant
rs1794728628	intron_variant
rs1794728763	intron_variant
rs1794728902	intron_variant
rs1794729055	intron_variant
rs1794729189	intron_variant
rs1794730631	intron_variant
rs1794730782	intron_variant
rs1794730888	intron_variant
rs1794731398	intron_variant
rs1794732032	intron_variant
rs1794732321	intron_variant
rs1794732438	intron_variant
rs1794733548	intron_variant
rs1794734727	intron_variant
rs1794736239	intron_variant
rs1794736550	intron_variant
rs1794736839	intron_variant
rs1794738603	intron_variant
rs1794739082	intron_variant
rs1794739225	intron_variant
rs1794739368	intron_variant
rs1794739647	intron_variant
rs1794739799	intron_variant
rs1794739936	intron_variant
rs1794740064	intron_variant
rs1794740333	intron_variant
rs1794741583	intron_variant
rs1794741882	intron_variant
rs1794742197	intron_variant
rs1794742372	intron_variant
rs1794742601	intron_variant
rs1794742681	intron_variant
rs1794742870	intron_variant
rs1794742949	intron_variant
rs1794743031	intron_variant
rs1794743267	intron_variant
rs1794743351	intron_variant
rs1794743434	intron_variant
rs1794743596	intron_variant
rs1794743869	intron_variant
rs1794744050	intron_variant
rs1794744165	intron_variant
rs1794744343	intron_variant
rs1794745141	intron_variant
rs1794745831	intron_variant
rs1794746114	intron_variant
rs1794746358	intron_variant
rs1794746738	intron_variant
rs1794747757	intron_variant
rs1794747900	intron_variant
rs1794748073	intron_variant
rs1794748394	intron_variant
rs1794750104	intron_variant
rs1794750557	intron_variant
rs1794750845	intron_variant
rs1794750981	intron_variant
rs1794751529	intron_variant
rs1794751796	intron_variant
rs1794752240	intron_variant
rs1794752351	intron_variant
rs1794752470	intron_variant
rs1794752714	intron_variant
rs1794752853	intron_variant

rs1794753421	intron_variant
rs1794753600	intron_variant
rs1794753723	intron_variant
rs1794753858	intron_variant
rs1794754113	intron_variant
rs1794754234	intron_variant
rs1794754832	intron_variant
rs1794755653	intron_variant
rs1794755924	intron_variant
rs1794756581	intron_variant
rs1794756970	intron_variant
rs1794757095	intron_variant
rs1794757260	intron_variant
rs1794757514	intron_variant
rs1794757635	intron_variant
rs1794758367	intron_variant
rs1794758636	intron_variant
rs1794758767	intron_variant
rs1794759196	intron_variant
rs1794759907	intron_variant
rs1794759996	intron_variant
rs1794760250	intron_variant
rs1794760420	intron_variant
rs1794760994	intron_variant
rs1794761081	intron_variant
rs1794761398	intron_variant
rs1794761635	intron_variant
rs1794761809	intron_variant
rs1794762171	intron_variant
rs1794762268	intron_variant
rs1794765724	intron_variant
rs1794765824	intron_variant
rs1794765931	intron_variant
rs1794766796	coding_sequence_variant,non_coding_transcript_variant,missense_variant
rs1794767087	coding_sequence_variant,non_coding_transcript_variant,frameshift_variant
rs1794768151	coding_sequence_variant,non_coding_transcript_variant,synonymous_variant
rs1794768272	coding_sequence_variant,non_coding_transcript_variant,missense_variant
rs1794768537	coding_sequence_variant,non_coding_transcript_variant,missense_variant
rs1794768724	coding_sequence_variant,non_coding_transcript_variant,missense_variant
rs1794770224	coding_sequence_variant,non_coding_transcript_variant,synonymous_variant
rs1794770427	coding_sequence_variant,non_coding_transcript_variant,missense_variant
rs1794771881	coding_sequence_variant,non_coding_transcript_variant,missense_variant
rs1794775921	coding_sequence_variant,intron_variant,synonymous_variant
rs1794776689	coding_sequence_variant,intron_variant,synonymous_variant
rs1794778060	downstream_transcript_variant,intron_variant,genic_downstream_transcript_variant
rs1794778600	downstream_transcript_variant,intron_variant,genic_downstream_transcript_variant
rs1794778844	downstream_transcript_variant,intron_variant,genic_downstream_transcript_variant
rs1794779137	downstream_transcript_variant,intron_variant,genic_downstream_transcript_variant
rs1794779849	downstream_transcript_variant,intron_variant,genic_downstream_transcript_variant
rs1794779984	downstream_transcript_variant,intron_variant,genic_downstream_transcript_variant
rs1794780054	downstream_transcript_variant,intron_variant,genic_downstream_transcript_variant
rs1794780130	downstream_transcript_variant,intron_variant,genic_downstream_transcript_variant
rs1794780349	downstream_transcript_variant,intron_variant,genic_downstream_transcript_variant
rs1794780931	downstream_transcript_variant,intron_variant,genic_downstream_transcript_variant
rs1794781082	downstream_transcript_variant,intron_variant,genic_downstream_transcript_variant
rs1794781325	downstream_transcript_variant,intron_variant,genic_downstream_transcript_variant
rs1794781403	downstream_transcript_variant,intron_variant,genic_downstream_transcript_variant
rs1794781626	downstream_transcript_variant,intron_variant,genic_downstream_transcript_variant
rs1794781814	downstream_transcript_variant,intron_variant,genic_downstream_transcript_variant
rs1794782455	downstream_transcript_variant,intron_variant,genic_downstream_transcript_variant
rs1794782627	downstream_transcript_variant,intron_variant,genic_downstream_transcript_variant
rs1794783234	downstream_transcript_variant,intron_variant,genic_downstream_transcript_variant
rs1794783816	downstream_transcript_variant,intron_variant,genic_downstream_transcript_variant
rs1794784157	downstream_transcript_variant,intron_variant,genic_downstream_transcript_variant
rs1794784232	downstream_transcript_variant,intron_variant,genic_downstream_transcript_variant
rs1794784717	downstream_transcript_variant,intron_variant,genic_downstream_transcript_variant
rs1794784787	downstream_transcript_variant,intron_variant,genic_downstream_transcript_variant
rs1794784876	downstream_transcript_variant,intron_variant,genic_downstream_transcript_variant
rs1794784962	downstream_transcript_variant,intron_variant,genic_downstream_transcript_variant
rs1794785047	downstream_transcript_variant,intron_variant,genic_downstream_transcript_variant
rs1794785126	downstream_transcript_variant,intron_variant,genic_downstream_transcript_variant
rs1794785643	downstream_transcript_variant,intron_variant,genic_downstream_transcript_variant
rs1794785772	downstream_transcript_variant,intron_variant,genic_downstream_transcript_variant
rs1794786022	downstream_transcript_variant,intron_variant,genic_downstream_transcript_variant

rs1794786267	downstream_transcript_variant,intron_variant,genic_downstream_transcript_variant
rs1794786396	downstream_transcript_variant,intron_variant,genic_downstream_transcript_variant
rs1794786763	downstream_transcript_variant,intron_variant,genic_downstream_transcript_variant
rs1794786893	downstream_transcript_variant,intron_variant,genic_downstream_transcript_variant
rs1794787533	downstream_transcript_variant,intron_variant,genic_downstream_transcript_variant
rs1794787641	downstream_transcript_variant,intron_variant,genic_downstream_transcript_variant
rs1794787732	downstream_transcript_variant,intron_variant,genic_downstream_transcript_variant
rs1794787800	downstream_transcript_variant,intron_variant,genic_downstream_transcript_variant
rs1794787886	downstream_transcript_variant,intron_variant,genic_downstream_transcript_variant
rs1794787964	downstream_transcript_variant,intron_variant,genic_downstream_transcript_variant
rs1794788215	downstream_transcript_variant,intron_variant,genic_downstream_transcript_variant
rs1794788385	downstream_transcript_variant,intron_variant,genic_downstream_transcript_variant
rs1794788471	downstream_transcript_variant,intron_variant,genic_downstream_transcript_variant
rs1794788650	downstream_transcript_variant,intron_variant,genic_downstream_transcript_variant
rs1794788877	downstream_transcript_variant,intron_variant,genic_downstream_transcript_variant
rs1794788976	downstream_transcript_variant,intron_variant,genic_downstream_transcript_variant
rs1794789472	downstream_transcript_variant,intron_variant,genic_downstream_transcript_variant
rs1794789632	downstream_transcript_variant,intron_variant,genic_downstream_transcript_variant
rs1794790255	downstream_transcript_variant,intron_variant,genic_downstream_transcript_variant
rs1794790434	downstream_transcript_variant,intron_variant,genic_downstream_transcript_variant
rs1794790577	downstream_transcript_variant,intron_variant,genic_downstream_transcript_variant
rs1794791189	downstream_transcript_variant,intron_variant,genic_downstream_transcript_variant
rs1794791443	downstream_transcript_variant,intron_variant,genic_downstream_transcript_variant
rs1794791687	downstream_transcript_variant,intron_variant,genic_downstream_transcript_variant
rs1794791844	downstream_transcript_variant,intron_variant,genic_downstream_transcript_variant
rs1794791932	downstream_transcript_variant,intron_variant,genic_downstream_transcript_variant
rs1794792096	downstream_transcript_variant,intron_variant,genic_downstream_transcript_variant
rs1794792261	downstream_transcript_variant,intron_variant,genic_downstream_transcript_variant
rs1794792403	downstream_transcript_variant,intron_variant,genic_downstream_transcript_variant
rs1794793439	intron_variant,genic_downstream_transcript_variant
rs1794793519	intron_variant,genic_downstream_transcript_variant
rs1794793777	intron_variant,genic_downstream_transcript_variant
rs1794794029	intron_variant,genic_downstream_transcript_variant
rs1794794280	intron_variant,genic_downstream_transcript_variant
rs1794794901	intron_variant,genic_downstream_transcript_variant
rs1794795037	intron_variant,genic_downstream_transcript_variant
rs1794795290	intron_variant,genic_downstream_transcript_variant
rs1794795520	intron_variant,genic_downstream_transcript_variant
rs1794795622	intron_variant,genic_downstream_transcript_variant
rs1794796011	intron_variant,genic_downstream_transcript_variant
rs1794796129	intron_variant,genic_downstream_transcript_variant
rs1794796261	intron_variant,genic_downstream_transcript_variant
rs1794796378	intron_variant,genic_downstream_transcript_variant
rs1794796507	intron_variant,genic_downstream_transcript_variant
rs1794796780	intron_variant,genic_downstream_transcript_variant
rs1794797523	intron_variant,genic_downstream_transcript_variant
rs1794798115	intron_variant,genic_downstream_transcript_variant
rs1794798245	intron_variant,genic_downstream_transcript_variant
rs1794798369	intron_variant,genic_downstream_transcript_variant
rs1794798497	intron_variant,genic_downstream_transcript_variant
rs1794798774	intron_variant,genic_downstream_transcript_variant
rs1794799382	intron_variant,genic_downstream_transcript_variant
rs1794799985	intron_variant,genic_downstream_transcript_variant
rs1794800065	intron_variant,genic_downstream_transcript_variant
rs1794800236	intron_variant,genic_downstream_transcript_variant
rs1794800364	intron_variant,genic_downstream_transcript_variant
rs1794800846	intron_variant,genic_downstream_transcript_variant
rs1794801443	intron_variant,genic_downstream_transcript_variant
rs1794801670	intron_variant,genic_downstream_transcript_variant
rs1794801759	intron_variant,genic_downstream_transcript_variant
rs1794802118	intron_variant,genic_downstream_transcript_variant
rs1794802239	intron_variant,genic_downstream_transcript_variant
rs1794802419	intron_variant,genic_downstream_transcript_variant
rs1794802611	intron_variant,genic_downstream_transcript_variant
rs1794802781	intron_variant,genic_downstream_transcript_variant
rs1794802858	intron_variant,genic_downstream_transcript_variant
rs1794802959	intron_variant,genic_downstream_transcript_variant
rs1794803296	intron_variant,genic_downstream_transcript_variant
rs1794803393	intron_variant,genic_downstream_transcript_variant
rs1794803749	intron_variant,genic_downstream_transcript_variant
rs1794803948	intron_variant,genic_downstream_transcript_variant
rs1794804037	intron_variant,genic_downstream_transcript_variant
rs1794804115	intron_variant,genic_downstream_transcript_variant
rs1794804193	intron_variant,genic_downstream_transcript_variant

rs1794804291	intron_variant,genic_downstream_transcript_variant
rs1794804365	intron_variant,genic_downstream_transcript_variant
rs1794804768	intron_variant,genic_downstream_transcript_variant
rs1794805297	intron_variant,genic_downstream_transcript_variant
rs1794805535	intron_variant,genic_downstream_transcript_variant
rs1794805919	intron_variant,genic_downstream_transcript_variant
rs1794806737	intron_variant,genic_downstream_transcript_variant
rs1794806891	intron_variant,genic_downstream_transcript_variant
rs1794807073	intron_variant,genic_downstream_transcript_variant
rs1794807147	intron_variant,genic_downstream_transcript_variant
rs1794807220	intron_variant,genic_downstream_transcript_variant
rs1794807573	intron_variant,genic_downstream_transcript_variant
rs1794807649	intron_variant,genic_downstream_transcript_variant
rs1794807815	intron_variant,genic_downstream_transcript_variant
rs1794807888	intron_variant,genic_downstream_transcript_variant
rs1794808307	intron_variant,genic_downstream_transcript_variant
rs1794808393	intron_variant,genic_downstream_transcript_variant
rs1794808601	intron_variant,genic_downstream_transcript_variant
rs1794809283	intron_variant,genic_downstream_transcript_variant
rs1794809349	intron_variant,genic_downstream_transcript_variant
rs1794809767	intron_variant,genic_downstream_transcript_variant
rs1794810727	intron_variant,genic_downstream_transcript_variant
rs1794810790	intron_variant,genic_downstream_transcript_variant
rs1794810997	intron_variant,genic_downstream_transcript_variant
rs1794811341	intron_variant,genic_downstream_transcript_variant
rs1794811422	intron_variant,genic_downstream_transcript_variant
rs1794811573	intron_variant,genic_downstream_transcript_variant
rs1794811655	intron_variant,genic_downstream_transcript_variant
rs1794811719	intron_variant,genic_downstream_transcript_variant
rs1794811814	intron_variant,genic_downstream_transcript_variant
rs1794811893	intron_variant,genic_downstream_transcript_variant
rs1794812048	intron_variant,genic_downstream_transcript_variant
rs1794812137	intron_variant,genic_downstream_transcript_variant
rs1794812539	intron_variant,genic_downstream_transcript_variant
rs1794812826	intron_variant,genic_downstream_transcript_variant
rs1794813424	intron_variant,genic_downstream_transcript_variant
rs1794814114	intron_variant,genic_downstream_transcript_variant
rs1794814338	intron_variant,genic_downstream_transcript_variant
rs1794814715	intron_variant,genic_downstream_transcript_variant
rs1794814789	intron_variant,genic_downstream_transcript_variant
rs1794815017	intron_variant,genic_downstream_transcript_variant
rs1794815385	intron_variant,genic_downstream_transcript_variant
rs1794815461	intron_variant,genic_downstream_transcript_variant
rs1794815769	intron_variant,genic_downstream_transcript_variant
rs1794816033	intron_variant,genic_downstream_transcript_variant
rs1794816581	intron_variant,genic_downstream_transcript_variant
rs1794816833	intron_variant,genic_downstream_transcript_variant
rs1794816949	intron_variant,genic_downstream_transcript_variant
rs1794817537	intron_variant,genic_downstream_transcript_variant
rs1794818202	intron_variant,genic_downstream_transcript_variant
rs1794818444	intron_variant,genic_downstream_transcript_variant
rs1794818713	intron_variant,genic_downstream_transcript_variant
rs1794818786	intron_variant,genic_downstream_transcript_variant
rs1794818952	intron_variant,genic_downstream_transcript_variant
rs1794819042	intron_variant,genic_downstream_transcript_variant
rs1794819128	intron_variant,genic_downstream_transcript_variant
rs1794819362	intron_variant,genic_downstream_transcript_variant
rs1794819592	intron_variant,genic_downstream_transcript_variant
rs1794819668	intron_variant,genic_downstream_transcript_variant
rs1794819902	intron_variant,genic_downstream_transcript_variant
rs1794820327	intron_variant,genic_downstream_transcript_variant
rs1794820408	intron_variant,genic_downstream_transcript_variant
rs1794822721	intron_variant,genic_downstream_transcript_variant
rs1794823444	intron_variant,genic_downstream_transcript_variant
rs1794823996	intron_variant,genic_downstream_transcript_variant
rs1794824119	intron_variant,genic_downstream_transcript_variant
rs1794824552	intron_variant,genic_downstream_transcript_variant
rs1794825610	intron_variant,genic_downstream_transcript_variant
rs1794825734	intron_variant,genic_downstream_transcript_variant
rs1794826423	intron_variant,genic_downstream_transcript_variant
rs1794826779	intron_variant,genic_downstream_transcript_variant
rs1794828878	intron_variant,genic_downstream_transcript_variant
rs1794829007	intron_variant,genic_downstream_transcript_variant
rs1794829199	intron_variant,genic_downstream_transcript_variant

rs1794829325	intron_variant,genic_downstream_transcript_variant
rs1794829593	intron_variant,genic_downstream_transcript_variant
rs1794831188	non_coding_transcript_variant,coding_sequence_variant,synonymous_variant,genic_downstream_transcript_variant
rs1794832170	coding_sequence_variant,synonymous_variant,non_coding_transcript_variant,genic_downstream_transcript_variant,missense_variant
rs1794832630	inframe_insertion,non_coding_transcript_variant,coding_sequence_variant,genic_downstream_transcript_variant
rs1794835436	missense_variant,non_coding_transcript_variant,coding_sequence_variant,genic_downstream_transcript_variant
rs1794836962	missense_variant,non_coding_transcript_variant,coding_sequence_variant,genic_downstream_transcript_variant
rs1794837434	missense_variant,non_coding_transcript_variant,coding_sequence_variant,genic_downstream_transcript_variant
rs1794837837	intron_variant,genic_downstream_transcript_variant
rs1794838240	intron_variant,genic_downstream_transcript_variant
rs1794838581	intron_variant,genic_downstream_transcript_variant
rs1794839355	intron_variant,genic_downstream_transcript_variant
rs1794839540	intron_variant,genic_downstream_transcript_variant
rs1794839735	intron_variant,genic_downstream_transcript_variant
rs1794840496	intron_variant,genic_downstream_transcript_variant
rs1794841027	intron_variant,genic_downstream_transcript_variant
rs1794841574	intron_variant,genic_downstream_transcript_variant
rs1794841711	intron_variant,genic_downstream_transcript_variant
rs1794841785	intron_variant,genic_downstream_transcript_variant
rs1794841857	intron_variant,genic_downstream_transcript_variant
rs1794841949	intron_variant,genic_downstream_transcript_variant
rs1794842204	intron_variant,genic_downstream_transcript_variant
rs1794843162	intron_variant,genic_downstream_transcript_variant
rs1794843490	intron_variant,genic_downstream_transcript_variant
rs1794843845	intron_variant,genic_downstream_transcript_variant
rs1794844139	intron_variant,genic_downstream_transcript_variant
rs1794844412	intron_variant,genic_downstream_transcript_variant
rs1794844480	intron_variant,genic_downstream_transcript_variant
rs1794844741	intron_variant,genic_downstream_transcript_variant
rs1794844835	intron_variant,genic_downstream_transcript_variant
rs1794845146	intron_variant,genic_downstream_transcript_variant
rs1794846913	missense_variant,non_coding_transcript_variant,coding_sequence_variant,genic_downstream_transcript_variant
rs1794846997	missense_variant,non_coding_transcript_variant,coding_sequence_variant,genic_downstream_transcript_variant
rs1794848550	missense_variant,non_coding_transcript_variant,coding_sequence_variant,genic_downstream_transcript_variant
rs1794850310	missense_variant,non_coding_transcript_variant,coding_sequence_variant,genic_downstream_transcript_variant
rs1794850395	missense_variant,non_coding_transcript_variant,coding_sequence_variant,genic_downstream_transcript_variant
rs1794850590	missense_variant,non_coding_transcript_variant,coding_sequence_variant,genic_downstream_transcript_variant
rs1794852692	intron_variant,genic_downstream_transcript_variant
rs1794852947	intron_variant,genic_downstream_transcript_variant
rs1794854824	intron_variant,genic_downstream_transcript_variant
rs1794855225	intron_variant,genic_downstream_transcript_variant
rs1794855574	intron_variant,genic_downstream_transcript_variant
rs1794855658	intron_variant,genic_downstream_transcript_variant
rs1794856301	intron_variant,genic_downstream_transcript_variant
rs1794856386	intron_variant,genic_downstream_transcript_variant
rs1794856462	intron_variant,genic_downstream_transcript_variant
rs1794856529	intron_variant,genic_downstream_transcript_variant
rs1794856656	intron_variant,genic_downstream_transcript_variant
rs1794856719	intron_variant,genic_downstream_transcript_variant
rs1794857073	intron_variant,genic_downstream_transcript_variant
rs1794857193	intron_variant,genic_downstream_transcript_variant
rs1794857368	intron_variant,genic_downstream_transcript_variant
rs1794857438	intron_variant,genic_downstream_transcript_variant
rs1794857850	intron_variant,genic_downstream_transcript_variant
rs1794858256	intron_variant,genic_downstream_transcript_variant
rs1794858410	intron_variant,genic_downstream_transcript_variant
rs1794858488	intron_variant,genic_downstream_transcript_variant
rs1794858570	intron_variant,genic_downstream_transcript_variant
rs1794858632	intron_variant,genic_downstream_transcript_variant
rs1794858789	intron_variant,genic_downstream_transcript_variant
rs1794859028	intron_variant,genic_downstream_transcript_variant
rs1794859104	intron_variant,genic_downstream_transcript_variant
rs1794859254	intron_variant,genic_downstream_transcript_variant
rs1794859332	intron_variant,genic_downstream_transcript_variant
rs1794859482	intron_variant,genic_downstream_transcript_variant
rs1794859562	intron_variant,genic_downstream_transcript_variant
rs1794859813	intron_variant,genic_downstream_transcript_variant
rs1794860081	intron_variant,genic_downstream_transcript_variant
rs1794860153	intron_variant,genic_downstream_transcript_variant
rs1794860314	intron_variant,genic_downstream_transcript_variant
rs1794860386	intron_variant,genic_downstream_transcript_variant
rs1794860454	intron_variant,genic_downstream_transcript_variant
rs1794860535	intron_variant,genic_downstream_transcript_variant
rs1794860675	intron_variant,genic_downstream_transcript_variant

rs1794861140	intron_variant,genic_downstream_transcript_variant
rs1794861362	intron_variant,genic_downstream_transcript_variant
rs1794861424	intron_variant,genic_downstream_transcript_variant
rs1794861582	intron_variant,genic_downstream_transcript_variant
rs1794861756	intron_variant,genic_downstream_transcript_variant
rs1794861988	intron_variant,genic_downstream_transcript_variant
rs1794862145	intron_variant,genic_downstream_transcript_variant
rs1794862217	intron_variant,genic_downstream_transcript_variant
rs1794862283	intron_variant,genic_downstream_transcript_variant
rs1794862695	intron_variant,genic_downstream_transcript_variant
rs1794863042	intron_variant,genic_downstream_transcript_variant
rs1794863126	intron_variant,genic_downstream_transcript_variant
rs1794863285	intron_variant,genic_downstream_transcript_variant
rs1794863470	intron_variant,genic_downstream_transcript_variant
rs1794863691	intron_variant,genic_downstream_transcript_variant
rs1794863830	intron_variant,genic_downstream_transcript_variant
rs1794863920	intron_variant,genic_downstream_transcript_variant
rs1794863995	intron_variant,genic_downstream_transcript_variant
rs1794864150	intron_variant,genic_downstream_transcript_variant
rs1794864884	intron_variant,genic_downstream_transcript_variant
rs1794865017	intron_variant,genic_downstream_transcript_variant
rs1794866219	intron_variant,genic_downstream_transcript_variant
rs1794866476	intron_variant,genic_downstream_transcript_variant
rs1794866613	intron_variant,genic_downstream_transcript_variant
rs1794866865	intron_variant,genic_downstream_transcript_variant
rs1794867576	intron_variant,genic_downstream_transcript_variant
rs1794867912	intron_variant,genic_downstream_transcript_variant
rs1794868250	intron_variant,genic_downstream_transcript_variant
rs1794868626	intron_variant,genic_downstream_transcript_variant
rs1794869174	intron_variant,genic_downstream_transcript_variant
rs1794869643	intron_variant,genic_downstream_transcript_variant
rs1794869714	intron_variant,genic_downstream_transcript_variant
rs1794870279	intron_variant,genic_downstream_transcript_variant
rs1794870527	intron_variant,genic_downstream_transcript_variant
rs1794870940	intron_variant,genic_downstream_transcript_variant
rs1794871051	intron_variant,genic_downstream_transcript_variant
rs1794871179	intron_variant,genic_downstream_transcript_variant
rs1794871556	intron_variant,genic_downstream_transcript_variant
rs1794872079	intron_variant,genic_downstream_transcript_variant
rs1794872366	intron_variant,genic_downstream_transcript_variant
rs1794872696	intron_variant,genic_downstream_transcript_variant
rs1794872961	intron_variant,genic_downstream_transcript_variant
rs1794873045	intron_variant,genic_downstream_transcript_variant
rs1794873341	intron_variant,genic_downstream_transcript_variant
rs1794873431	intron_variant,genic_downstream_transcript_variant
rs1794873759	intron_variant,genic_downstream_transcript_variant
rs1794873854	intron_variant,genic_downstream_transcript_variant
rs1794874018	intron_variant,genic_downstream_transcript_variant
rs1794874319	intron_variant,genic_downstream_transcript_variant
rs1794874393	intron_variant,genic_downstream_transcript_variant
rs1794874658	intron_variant,genic_downstream_transcript_variant
rs1794874730	intron_variant,genic_downstream_transcript_variant
rs1794875218	intron_variant,genic_downstream_transcript_variant
rs1794875305	intron_variant,genic_downstream_transcript_variant
rs1794875394	intron_variant,genic_downstream_transcript_variant
rs1794875465	intron_variant,genic_downstream_transcript_variant
rs1794875533	intron_variant,genic_downstream_transcript_variant
rs1794875760	intron_variant,genic_downstream_transcript_variant
rs1794875849	intron_variant,genic_downstream_transcript_variant
rs1794876016	intron_variant,genic_downstream_transcript_variant
rs1794876328	intron_variant,genic_downstream_transcript_variant
rs1794876646	intron_variant,genic_downstream_transcript_variant
rs1794876875	intron_variant,genic_downstream_transcript_variant
rs1794876959	intron_variant,genic_downstream_transcript_variant
rs1794877215	intron_variant,genic_downstream_transcript_variant
rs1794878191	intron_variant,genic_downstream_transcript_variant
rs1794879050	intron_variant,genic_downstream_transcript_variant
rs1794879193	intron_variant,genic_downstream_transcript_variant
rs1794879437	intron_variant,genic_downstream_transcript_variant
rs1794879694	intron_variant,genic_downstream_transcript_variant
rs1794880144	intron_variant,genic_downstream_transcript_variant
rs1794880394	intron_variant,genic_downstream_transcript_variant
rs1794880508	intron_variant,genic_downstream_transcript_variant
rs1794880626	intron_variant,genic_downstream_transcript_variant

rs1794880876	intron_variant,genic_downstream_transcript_variant
rs1794881105	intron_variant,genic_downstream_transcript_variant
rs1794882592	intron_variant,genic_downstream_transcript_variant
rs1794883010	intron_variant,genic_downstream_transcript_variant
rs1794883870	intron_variant,genic_downstream_transcript_variant
rs1794884114	intron_variant,genic_downstream_transcript_variant
rs1794884352	intron_variant,genic_downstream_transcript_variant
rs1794884778	intron_variant,genic_downstream_transcript_variant
rs1794885332	intron_variant,genic_downstream_transcript_variant
rs1794885441	intron_variant,genic_downstream_transcript_variant
rs1794885510	intron_variant,genic_downstream_transcript_variant
rs1794885676	intron_variant,genic_downstream_transcript_variant
rs1794885976	intron_variant,genic_downstream_transcript_variant
rs1794886060	intron_variant,genic_downstream_transcript_variant
rs1794886227	intron_variant,genic_downstream_transcript_variant
rs1794886365	intron_variant,genic_downstream_transcript_variant
rs1794886464	intron_variant,genic_downstream_transcript_variant
rs1794886637	intron_variant,genic_downstream_transcript_variant
rs1794886856	intron_variant,genic_downstream_transcript_variant
rs1794886928	intron_variant,genic_downstream_transcript_variant
rs1794887018	intron_variant,genic_downstream_transcript_variant
rs1794887659	intron_variant,genic_downstream_transcript_variant
rs1794888099	intron_variant,genic_downstream_transcript_variant
rs1794888226	intron_variant,genic_downstream_transcript_variant
rs1794888311	intron_variant,genic_downstream_transcript_variant
rs1794888962	intron_variant,genic_downstream_transcript_variant
rs1794889051	intron_variant,genic_downstream_transcript_variant
rs1794889432	intron_variant,genic_downstream_transcript_variant
rs1794889611	intron_variant,genic_downstream_transcript_variant
rs1794889886	intron_variant,genic_downstream_transcript_variant
rs1794890042	intron_variant,genic_downstream_transcript_variant
rs1794890524	intron_variant,genic_downstream_transcript_variant
rs1794890705	intron_variant,genic_downstream_transcript_variant
rs1794890865	intron_variant,genic_downstream_transcript_variant
rs1794891034	intron_variant,genic_downstream_transcript_variant
rs1794891283	intron_variant,genic_downstream_transcript_variant
rs1794891361	intron_variant,genic_downstream_transcript_variant
rs1794891677	intron_variant,genic_downstream_transcript_variant
rs1794891764	intron_variant,genic_downstream_transcript_variant
rs1794891915	intron_variant,genic_downstream_transcript_variant
rs1794892014	intron_variant,genic_downstream_transcript_variant
rs1794892097	intron_variant,genic_downstream_transcript_variant
rs1794892350	intron_variant,genic_downstream_transcript_variant
rs1794892433	intron_variant,genic_downstream_transcript_variant
rs1794893237	intron_variant,genic_downstream_transcript_variant
rs1794893314	intron_variant,genic_downstream_transcript_variant
rs1794893998	intron_variant,genic_downstream_transcript_variant
rs1794894407	intron_variant,genic_downstream_transcript_variant
rs1794895397	intron_variant,genic_downstream_transcript_variant
rs1794895730	intron_variant,genic_downstream_transcript_variant
rs1794896059	intron_variant,genic_downstream_transcript_variant
rs1794896301	intron_variant,genic_downstream_transcript_variant
rs1794896541	intron_variant,genic_downstream_transcript_variant
rs1794896891	intron_variant,genic_downstream_transcript_variant
rs1794897165	intron_variant,genic_downstream_transcript_variant
rs1794897277	intron_variant,genic_downstream_transcript_variant
rs1794898837	intron_variant,genic_downstream_transcript_variant
rs1794899293	intron_variant,genic_downstream_transcript_variant
rs1794899747	intron_variant,genic_downstream_transcript_variant
rs1794900757	intron_variant,genic_downstream_transcript_variant
rs1794900862	intron_variant,genic_downstream_transcript_variant
rs1794902059	intron_variant,genic_downstream_transcript_variant
rs1794902165	intron_variant,genic_downstream_transcript_variant
rs1794902253	intron_variant,genic_downstream_transcript_variant
rs1794902647	intron_variant,genic_downstream_transcript_variant
rs1794902765	intron_variant,genic_downstream_transcript_variant
rs1794902868	intron_variant,genic_downstream_transcript_variant
rs1794903389	intron_variant,genic_downstream_transcript_variant
rs1794903499	intron_variant,genic_downstream_transcript_variant
rs1794903613	intron_variant,genic_downstream_transcript_variant
rs1794903728	intron_variant,genic_downstream_transcript_variant
rs1794904104	intron_variant,genic_downstream_transcript_variant
rs1794904227	intron_variant,genic_downstream_transcript_variant
rs1794905461	intron_variant,genic_downstream_transcript_variant

rs1794905819	intron_variant,genic_downstream_transcript_variant
rs1794906575	intron_variant,genic_downstream_transcript_variant
rs1794906733	intron_variant,genic_downstream_transcript_variant
rs1794906844	intron_variant,genic_downstream_transcript_variant
rs1794906957	intron_variant,genic_downstream_transcript_variant
rs1794907432	intron_variant,genic_downstream_transcript_variant
rs1794908043	intron_variant,genic_downstream_transcript_variant
rs1794908119	intron_variant,genic_downstream_transcript_variant
rs1794908340	intron_variant,genic_downstream_transcript_variant
rs1794908496	intron_variant,genic_downstream_transcript_variant
rs1794908566	intron_variant,genic_downstream_transcript_variant
rs1794908624	intron_variant,genic_downstream_transcript_variant
rs1794908768	intron_variant,genic_downstream_transcript_variant
rs1794908936	intron_variant,genic_downstream_transcript_variant
rs1794909011	intron_variant,genic_downstream_transcript_variant
rs1794909244	intron_variant,genic_downstream_transcript_variant
rs1794909316	intron_variant,genic_downstream_transcript_variant
rs1794909463	intron_variant,genic_downstream_transcript_variant
rs1794909683	intron_variant,genic_downstream_transcript_variant
rs1794909769	intron_variant,genic_downstream_transcript_variant
rs1794909925	intron_variant,genic_downstream_transcript_variant
rs1794910515	intron_variant,genic_downstream_transcript_variant
rs1794911081	intron_variant,genic_downstream_transcript_variant
rs1794911162	intron_variant,genic_downstream_transcript_variant
rs1794911338	intron_variant,genic_downstream_transcript_variant
rs1794912031	intron_variant,genic_downstream_transcript_variant
rs1794912094	intron_variant,genic_downstream_transcript_variant
rs1794912293	intron_variant,genic_downstream_transcript_variant
rs1794912363	intron_variant,genic_downstream_transcript_variant
rs1794912966	intron_variant,genic_downstream_transcript_variant
rs1794913094	intron_variant,genic_downstream_transcript_variant
rs1794913170	intron_variant,genic_downstream_transcript_variant
rs1794913485	intron_variant,genic_downstream_transcript_variant
rs1794913547	intron_variant,genic_downstream_transcript_variant
rs1794913904	intron_variant,genic_downstream_transcript_variant
rs1794914394	intron_variant,genic_downstream_transcript_variant
rs1794914647	intron_variant,genic_downstream_transcript_variant
rs1794914741	intron_variant,genic_downstream_transcript_variant
rs1794914890	intron_variant,genic_downstream_transcript_variant
rs1794914969	intron_variant,genic_downstream_transcript_variant
rs1794915510	intron_variant,genic_downstream_transcript_variant
rs1794915597	intron_variant,genic_downstream_transcript_variant
rs1794915665	intron_variant,genic_downstream_transcript_variant
rs1794915753	intron_variant,genic_downstream_transcript_variant
rs1794916785	intron_variant,genic_downstream_transcript_variant
rs1794916908	intron_variant,genic_downstream_transcript_variant
rs1794917277	intron_variant,genic_downstream_transcript_variant
rs1794917771	intron_variant,genic_downstream_transcript_variant
rs1794918274	intron_variant,genic_downstream_transcript_variant
rs1794918389	intron_variant,genic_downstream_transcript_variant
rs1794918534	intron_variant,genic_downstream_transcript_variant
rs1794918765	intron_variant,genic_downstream_transcript_variant
rs1794918885	intron_variant,genic_downstream_transcript_variant
rs1794919012	intron_variant,genic_downstream_transcript_variant
rs1794919263	intron_variant,genic_downstream_transcript_variant
rs1794919385	intron_variant,genic_downstream_transcript_variant
rs1794919502	intron_variant,genic_downstream_transcript_variant
rs1794919768	intron_variant,genic_downstream_transcript_variant
rs1794920284	intron_variant,genic_downstream_transcript_variant
rs1794920905	intron_variant,genic_downstream_transcript_variant
rs1794921126	intron_variant,genic_downstream_transcript_variant
rs1794921628	intron_variant,genic_downstream_transcript_variant
rs1794922126	intron_variant,genic_downstream_transcript_variant
rs1794922402	intron_variant,genic_downstream_transcript_variant
rs1794922579	intron_variant,genic_downstream_transcript_variant
rs1794922761	intron_variant,genic_downstream_transcript_variant
rs1794923198	intron_variant,genic_downstream_transcript_variant
rs1794923264	intron_variant,genic_downstream_transcript_variant
rs1794923445	intron_variant,genic_downstream_transcript_variant
rs1794924084	intron_variant,genic_downstream_transcript_variant
rs1794924259	intron_variant,genic_downstream_transcript_variant
rs1794924345	intron_variant,genic_downstream_transcript_variant
rs1794924425	intron_variant,genic_downstream_transcript_variant
rs1794924493	intron_variant,genic_downstream_transcript_variant

rs1794924966	intron_variant,genic_downstream_transcript_variant
rs1794925377	intron_variant,genic_downstream_transcript_variant
rs1794925529	intron_variant,genic_downstream_transcript_variant
rs1794925898	intron_variant,genic_downstream_transcript_variant
rs1794925975	intron_variant,genic_downstream_transcript_variant
rs1794926372	intron_variant,genic_downstream_transcript_variant
rs1794926626	intron_variant,genic_downstream_transcript_variant
rs1794927134	intron_variant,genic_downstream_transcript_variant
rs1794927398	intron_variant,genic_downstream_transcript_variant
rs1794927481	intron_variant,genic_downstream_transcript_variant
rs1794927556	intron_variant,genic_downstream_transcript_variant
rs1794927870	intron_variant,genic_downstream_transcript_variant
rs1794928915	intron_variant,genic_downstream_transcript_variant
rs1794929190	intron_variant,genic_downstream_transcript_variant
rs1794929327	intron_variant,genic_downstream_transcript_variant
rs1794929484	intron_variant,genic_downstream_transcript_variant
rs1794929709	intron_variant,genic_downstream_transcript_variant
rs1794929911	intron_variant,genic_downstream_transcript_variant
rs1794930045	intron_variant,genic_downstream_transcript_variant
rs1794930871	intron_variant,genic_downstream_transcript_variant
rs1794930999	intron_variant,genic_downstream_transcript_variant
rs1794932085	intron_variant,genic_downstream_transcript_variant
rs1794932332	intron_variant,genic_downstream_transcript_variant
rs1794932695	intron_variant,genic_downstream_transcript_variant
rs1794933303	intron_variant,genic_downstream_transcript_variant
rs1794933550	intron_variant,genic_downstream_transcript_variant
rs1794934820	intron_variant,genic_downstream_transcript_variant
rs1794935269	intron_variant,genic_downstream_transcript_variant
rs1794935411	intron_variant,genic_downstream_transcript_variant
rs1794936198	intron_variant,genic_downstream_transcript_variant
rs1794936985	intron_variant,genic_downstream_transcript_variant
rs1794937102	intron_variant,genic_downstream_transcript_variant
rs1794937360	intron_variant,genic_downstream_transcript_variant
rs1794937606	intron_variant,genic_downstream_transcript_variant
rs1794938210	intron_variant,genic_downstream_transcript_variant
rs1794939005	intron_variant,genic_downstream_transcript_variant
rs1794939118	intron_variant,genic_downstream_transcript_variant
rs1794939349	intron_variant,genic_downstream_transcript_variant
rs1794939839	intron_variant,genic_downstream_transcript_variant
rs1794940066	intron_variant,genic_downstream_transcript_variant
rs1794940204	intron_variant,genic_downstream_transcript_variant
rs1794940691	intron_variant,genic_downstream_transcript_variant
rs1794940958	intron_variant,genic_downstream_transcript_variant
rs1794941249	intron_variant,genic_downstream_transcript_variant
rs1794941511	intron_variant,genic_downstream_transcript_variant
rs1794941919	intron_variant,genic_downstream_transcript_variant
rs1794942220	intron_variant,genic_downstream_transcript_variant
rs1794942340	intron_variant,genic_downstream_transcript_variant
rs1794942486	intron_variant,genic_downstream_transcript_variant
rs1794942605	intron_variant,genic_downstream_transcript_variant
rs1794943856	intron_variant,genic_downstream_transcript_variant
rs1794944566	intron_variant,genic_downstream_transcript_variant
rs1794944693	intron_variant,genic_downstream_transcript_variant
rs1794946463	non_coding_transcript_variant,coding_sequence_variant,synonymous_variant,genic_downstream_transcript_variant
rs1794949535	missense_variant,non_coding_transcript_variant,coding_sequence_variant,genic_downstream_transcript_variant
rs1794949971	missense_variant,non_coding_transcript_variant,coding_sequence_variant,genic_downstream_transcript_variant
rs1794950502	missense_variant,non_coding_transcript_variant,coding_sequence_variant,genic_downstream_transcript_variant
rs1794951727	missense_variant,non_coding_transcript_variant,coding_sequence_variant,genic_downstream_transcript_variant
rs1794951926	non_coding_transcript_variant,coding_sequence_variant,synonymous_variant,genic_downstream_transcript_variant
rs1794952295	non_coding_transcript_variant,coding_sequence_variant,synonymous_variant,genic_downstream_transcript_variant
rs1794953165	missense_variant,non_coding_transcript_variant,coding_sequence_variant,genic_downstream_transcript_variant
rs1794953674	non_coding_transcript_variant,coding_sequence_variant,synonymous_variant,genic_downstream_transcript_variant
rs1794954190	non_coding_transcript_variant,coding_sequence_variant,synonymous_variant,genic_downstream_transcript_variant
rs1794955817	intron_variant,genic_downstream_transcript_variant
rs1794955980	intron_variant,genic_downstream_transcript_variant
rs1794956405	intron_variant,genic_downstream_transcript_variant
rs1794956483	intron_variant,genic_downstream_transcript_variant
rs1794956863	intron_variant,genic_downstream_transcript_variant
rs1794957557	intron_variant,genic_downstream_transcript_variant
rs1794957631	intron_variant,genic_downstream_transcript_variant
rs1794957712	intron_variant,genic_downstream_transcript_variant
rs1794957804	intron_variant,genic_downstream_transcript_variant
rs1794958030	intron_variant,genic_downstream_transcript_variant
rs1794958196	intron_variant,genic_downstream_transcript_variant

rs1794958275	intron_variant,genic_downstream_transcript_variant
rs1794958417	intron_variant,genic_downstream_transcript_variant
rs1794958493	intron_variant,genic_downstream_transcript_variant
rs1794958562	intron_variant,genic_downstream_transcript_variant
rs1794958799	intron_variant,genic_downstream_transcript_variant
rs1794958883	intron_variant,genic_downstream_transcript_variant
rs1794959257	intron_variant,genic_downstream_transcript_variant
rs1794959827	intron_variant,genic_downstream_transcript_variant
rs1794959900	intron_variant,genic_downstream_transcript_variant
rs1794960307	intron_variant,genic_downstream_transcript_variant
rs1794960551	intron_variant,genic_downstream_transcript_variant
rs1794961256	intron_variant,genic_downstream_transcript_variant
rs1794961476	intron_variant,genic_downstream_transcript_variant
rs1794961552	intron_variant,genic_downstream_transcript_variant
rs1794962228	intron_variant,genic_downstream_transcript_variant
rs1794962307	intron_variant,genic_downstream_transcript_variant
rs1794962374	intron_variant,genic_downstream_transcript_variant
rs1794962433	intron_variant,genic_downstream_transcript_variant
rs1794962508	intron_variant,genic_downstream_transcript_variant
rs1794963019	intron_variant,genic_downstream_transcript_variant
rs1794963096	intron_variant,genic_downstream_transcript_variant
rs1794963155	intron_variant,genic_downstream_transcript_variant
rs1794963297	intron_variant,genic_downstream_transcript_variant
rs1794963639	intron_variant,genic_downstream_transcript_variant
rs1794963798	intron_variant,genic_downstream_transcript_variant
rs1794963868	intron_variant,genic_downstream_transcript_variant
rs1794963939	intron_variant,genic_downstream_transcript_variant
rs1794964742	intron_variant,genic_downstream_transcript_variant
rs1794965031	intron_variant,genic_downstream_transcript_variant
rs1794965244	intron_variant,genic_downstream_transcript_variant
rs1794965449	intron_variant,genic_downstream_transcript_variant
rs1794965580	intron_variant,genic_downstream_transcript_variant
rs1794965725	intron_variant,genic_downstream_transcript_variant
rs1794965784	intron_variant,genic_downstream_transcript_variant
rs1794966107	intron_variant,genic_downstream_transcript_variant
rs1794966222	intron_variant,genic_downstream_transcript_variant
rs1794966343	intron_variant,genic_downstream_transcript_variant
rs1794966820	intron_variant,genic_downstream_transcript_variant
rs1794967186	intron_variant,genic_downstream_transcript_variant
rs1794967461	intron_variant,genic_downstream_transcript_variant
rs1794967610	intron_variant,genic_downstream_transcript_variant
rs1794967966	intron_variant,genic_downstream_transcript_variant
rs1794968110	intron_variant,genic_downstream_transcript_variant
rs1794968478	intron_variant,genic_downstream_transcript_variant
rs1794968626	intron_variant,genic_downstream_transcript_variant
rs1794968881	intron_variant,genic_downstream_transcript_variant
rs1794968962	intron_variant,genic_downstream_transcript_variant
rs1794969046	intron_variant,genic_downstream_transcript_variant
rs1794969214	intron_variant,genic_downstream_transcript_variant
rs1794969287	intron_variant,genic_downstream_transcript_variant
rs1794969355	intron_variant,genic_downstream_transcript_variant
rs1794969430	intron_variant,genic_downstream_transcript_variant
rs1794969655	intron_variant,genic_downstream_transcript_variant
rs1794970039	intron_variant,genic_downstream_transcript_variant
rs1794970153	intron_variant,genic_downstream_transcript_variant
rs1794970394	intron_variant,genic_downstream_transcript_variant
rs1794970979	intron_variant,genic_downstream_transcript_variant
rs1794971114	intron_variant,genic_downstream_transcript_variant
rs1794971191	intron_variant,genic_downstream_transcript_variant
rs1794971268	intron_variant,genic_downstream_transcript_variant
rs1794971384	intron_variant,genic_downstream_transcript_variant
rs1794971919	intron_variant,genic_downstream_transcript_variant
rs1794972143	intron_variant,genic_downstream_transcript_variant
rs1794972388	intron_variant,genic_downstream_transcript_variant
rs1794972921	intron_variant,genic_downstream_transcript_variant
rs1794973453	intron_variant,genic_downstream_transcript_variant
rs1794973694	intron_variant,genic_downstream_transcript_variant
rs1794973822	intron_variant,genic_downstream_transcript_variant
rs1794973949	intron_variant,genic_downstream_transcript_variant
rs1794974071	intron_variant,genic_downstream_transcript_variant
rs1794974721	intron_variant,genic_downstream_transcript_variant
rs1794975420	intron_variant,genic_downstream_transcript_variant
rs1794975815	intron_variant,genic_downstream_transcript_variant
rs1794975963	intron_variant,genic_downstream_transcript_variant

rs1794976089	intron_variant,genic_downstream_transcript_variant
rs1794978824	intron_variant,genic_downstream_transcript_variant
rs1794979076	intron_variant,genic_downstream_transcript_variant
rs1794979442	intron_variant,genic_downstream_transcript_variant
rs1794979527	intron_variant,genic_downstream_transcript_variant
rs1794979597	intron_variant,genic_downstream_transcript_variant
rs1794979763	intron_variant,genic_downstream_transcript_variant
rs1794979861	intron_variant,genic_downstream_transcript_variant
rs1794979950	intron_variant,genic_downstream_transcript_variant
rs1794980111	intron_variant,genic_downstream_transcript_variant
rs1794980374	intron_variant,genic_downstream_transcript_variant
rs1794980519	intron_variant,genic_downstream_transcript_variant
rs1794980769	intron_variant,genic_downstream_transcript_variant
rs1794981105	intron_variant,genic_downstream_transcript_variant
rs1794981175	intron_variant,genic_downstream_transcript_variant
rs1794981687	intron_variant,genic_downstream_transcript_variant
rs1794982717	intron_variant,genic_downstream_transcript_variant
rs1794982977	intron_variant,genic_downstream_transcript_variant
rs1794984605	intron_variant,genic_downstream_transcript_variant
rs1794984681	intron_variant,genic_downstream_transcript_variant
rs1794985057	intron_variant,genic_downstream_transcript_variant
rs1794986187	intron_variant,genic_downstream_transcript_variant
rs1794987691	intron_variant,genic_downstream_transcript_variant
rs1794987878	intron_variant,genic_downstream_transcript_variant
rs1794988070	intron_variant,genic_downstream_transcript_variant
rs1794988167	intron_variant,genic_downstream_transcript_variant
rs1794988329	intron_variant,genic_downstream_transcript_variant
rs1794988481	intron_variant,genic_downstream_transcript_variant
rs1794988698	intron_variant,genic_downstream_transcript_variant
rs1794988864	intron_variant,genic_downstream_transcript_variant
rs1794989146	intron_variant,genic_downstream_transcript_variant
rs1794989416	intron_variant,genic_downstream_transcript_variant
rs1794989559	intron_variant,genic_downstream_transcript_variant
rs1794989633	intron_variant,genic_downstream_transcript_variant
rs1794990388	intron_variant,genic_downstream_transcript_variant
rs1794990848	intron_variant,genic_downstream_transcript_variant
rs1794990934	intron_variant,genic_downstream_transcript_variant
rs1794991196	intron_variant,genic_downstream_transcript_variant
rs1794991283	intron_variant,genic_downstream_transcript_variant
rs1794991361	intron_variant,genic_downstream_transcript_variant
rs1794991550	intron_variant,genic_downstream_transcript_variant
rs1794991624	intron_variant,genic_downstream_transcript_variant
rs1794991702	intron_variant,genic_downstream_transcript_variant
rs1794991898	intron_variant,genic_downstream_transcript_variant
rs1794992252	intron_variant,genic_downstream_transcript_variant
rs1794993091	intron_variant,genic_downstream_transcript_variant
rs1794993428	intron_variant,genic_downstream_transcript_variant
rs1794994248	intron_variant,genic_downstream_transcript_variant
rs1794994975	intron_variant,genic_downstream_transcript_variant
rs1794995638	intron_variant,genic_downstream_transcript_variant
rs1794995724	intron_variant,genic_downstream_transcript_variant
rs1794996234	intron_variant,genic_downstream_transcript_variant
rs1794996336	intron_variant,genic_downstream_transcript_variant
rs1794996524	intron_variant,genic_downstream_transcript_variant
rs1794996819	intron_variant,genic_downstream_transcript_variant
rs1794996984	intron_variant,genic_downstream_transcript_variant
rs1794997561	intron_variant,genic_downstream_transcript_variant
rs1794997745	intron_variant,genic_downstream_transcript_variant
rs1794998410	intron_variant,genic_downstream_transcript_variant
rs1794998618	intron_variant,genic_downstream_transcript_variant
rs1794998813	intron_variant,genic_downstream_transcript_variant
rs1794999193	intron_variant,genic_downstream_transcript_variant
rs1794999432	intron_variant,genic_downstream_transcript_variant
rs1794999508	intron_variant,genic_downstream_transcript_variant
rs1794999699	intron_variant,genic_downstream_transcript_variant
rs1795000604	intron_variant,genic_downstream_transcript_variant
rs1795001241	intron_variant,genic_downstream_transcript_variant
rs1795001574	intron_variant,genic_downstream_transcript_variant
rs1795001906	intron_variant,genic_downstream_transcript_variant
rs1795002010	intron_variant,genic_downstream_transcript_variant
rs1795002208	intron_variant,genic_downstream_transcript_variant
rs1795002553	intron_variant,genic_downstream_transcript_variant
rs1795002812	intron_variant,genic_downstream_transcript_variant
rs1795002888	intron_variant,genic_downstream_transcript_variant

rs1795003061	intron_variant,genic_downstream_transcript_variant
rs1795003131	intron_variant,genic_downstream_transcript_variant
rs1795003319	intron_variant,genic_downstream_transcript_variant
rs1795003399	intron_variant,genic_downstream_transcript_variant
rs1795003479	intron_variant,genic_downstream_transcript_variant
rs1795003859	intron_variant,genic_downstream_transcript_variant
rs1795003943	intron_variant,genic_downstream_transcript_variant
rs1795004034	intron_variant,genic_downstream_transcript_variant
rs1795004347	intron_variant,genic_downstream_transcript_variant
rs1795004522	intron_variant,genic_downstream_transcript_variant
rs1795005242	intron_variant,genic_downstream_transcript_variant
rs1795005403	intron_variant,genic_downstream_transcript_variant
rs1795005642	intron_variant,genic_downstream_transcript_variant
rs1795005837	intron_variant,genic_downstream_transcript_variant
rs1795006073	intron_variant,genic_downstream_transcript_variant
rs1795006159	intron_variant,genic_downstream_transcript_variant
rs1795007006	intron_variant,genic_downstream_transcript_variant
rs1795007145	intron_variant,genic_downstream_transcript_variant
rs1795007474	intron_variant,genic_downstream_transcript_variant
rs1795008061	intron_variant,genic_downstream_transcript_variant
rs1795008725	intron_variant,genic_downstream_transcript_variant
rs1795008815	intron_variant,genic_downstream_transcript_variant
rs1795009296	intron_variant,genic_downstream_transcript_variant
rs1795009373	intron_variant,genic_downstream_transcript_variant
rs1795009447	intron_variant,genic_downstream_transcript_variant
rs1795009885	intron_variant,genic_downstream_transcript_variant
rs1795010172	intron_variant,genic_downstream_transcript_variant
rs1795010344	intron_variant,genic_downstream_transcript_variant
rs1795010440	intron_variant,genic_downstream_transcript_variant
rs1795010566	intron_variant,genic_downstream_transcript_variant
rs1795010754	intron_variant,genic_downstream_transcript_variant
rs1795010830	intron_variant,genic_downstream_transcript_variant
rs1795010926	intron_variant,genic_downstream_transcript_variant
rs1795012036	intron_variant,genic_downstream_transcript_variant
rs1795012168	intron_variant,genic_downstream_transcript_variant
rs1795013159	intron_variant,genic_downstream_transcript_variant
rs1795013283	intron_variant,genic_downstream_transcript_variant
rs1795013930	intron_variant,genic_downstream_transcript_variant
rs1795014017	intron_variant,genic_downstream_transcript_variant
rs1795014250	intron_variant,genic_downstream_transcript_variant
rs1795014503	intron_variant,genic_downstream_transcript_variant
rs1795015127	intron_variant,genic_downstream_transcript_variant
rs1795015364	intron_variant,genic_downstream_transcript_variant
rs1795015886	intron_variant,genic_downstream_transcript_variant
rs1795016018	intron_variant,genic_downstream_transcript_variant
rs1795016369	intron_variant,genic_downstream_transcript_variant
rs1795016510	intron_variant,genic_downstream_transcript_variant
rs1795016656	intron_variant,genic_downstream_transcript_variant
rs1795016930	intron_variant,genic_downstream_transcript_variant
rs1795017319	intron_variant,genic_downstream_transcript_variant
rs1795017447	intron_variant,genic_downstream_transcript_variant
rs1795017572	intron_variant,genic_downstream_transcript_variant
rs1795017707	intron_variant,genic_downstream_transcript_variant
rs1795018711	intron_variant,genic_downstream_transcript_variant
rs1795019058	intron_variant,genic_downstream_transcript_variant
rs1795019638	intron_variant,genic_downstream_transcript_variant
rs1795020075	intron_variant,genic_downstream_transcript_variant
rs1795020355	intron_variant,genic_downstream_transcript_variant
rs1795020673	intron_variant,genic_downstream_transcript_variant
rs1795021351	intron_variant,genic_downstream_transcript_variant
rs1795021466	intron_variant,genic_downstream_transcript_variant
rs1795021648	intron_variant,genic_downstream_transcript_variant
rs1795021850	intron_variant,genic_downstream_transcript_variant
rs1795022089	intron_variant,genic_downstream_transcript_variant
rs1795022199	intron_variant,genic_downstream_transcript_variant
rs1795022751	intron_variant,genic_downstream_transcript_variant
rs1795023335	intron_variant,genic_downstream_transcript_variant
rs1795023734	intron_variant,genic_downstream_transcript_variant
rs1795024227	intron_variant,genic_downstream_transcript_variant
rs1795024895	intron_variant,genic_downstream_transcript_variant
rs1795025066	intron_variant,genic_downstream_transcript_variant
rs1795027070	intron_variant,genic_downstream_transcript_variant
rs1795027353	intron_variant,genic_downstream_transcript_variant
rs1795027494	intron_variant,genic_downstream_transcript_variant

rs1795027871	splice_acceptor_variant,genic_downstream_transcript_variant
rs1795031717	intron_variant,genic_downstream_transcript_variant
rs1795033012	intron_variant,genic_downstream_transcript_variant
rs1795033266	intron_variant,genic_downstream_transcript_variant
rs1795033434	intron_variant,genic_downstream_transcript_variant
rs1795033515	intron_variant,genic_downstream_transcript_variant
rs1795034169	intron_variant,genic_downstream_transcript_variant
rs1795034419	intron_variant,genic_downstream_transcript_variant
rs1795034516	intron_variant,genic_downstream_transcript_variant
rs1795034625	intron_variant,genic_downstream_transcript_variant
rs1795034956	intron_variant,genic_downstream_transcript_variant
rs1795035120	intron_variant,genic_downstream_transcript_variant
rs1795035288	intron_variant,genic_downstream_transcript_variant
rs1795035377	intron_variant,genic_downstream_transcript_variant
rs1795035455	intron_variant,genic_downstream_transcript_variant
rs1795035694	intron_variant,genic_downstream_transcript_variant
rs1795035775	intron_variant,genic_downstream_transcript_variant
rs1795035861	intron_variant,genic_downstream_transcript_variant
rs1795036123	intron_variant,genic_downstream_transcript_variant
rs1795036209	intron_variant,genic_downstream_transcript_variant
rs1795036333	intron_variant,genic_downstream_transcript_variant
rs1795036582	intron_variant,genic_downstream_transcript_variant
rs1795036717	intron_variant,genic_downstream_transcript_variant
rs1795036840	intron_variant,genic_downstream_transcript_variant
rs1795037470	intron_variant,genic_downstream_transcript_variant
rs1795037563	intron_variant,genic_downstream_transcript_variant
rs1795037658	intron_variant,genic_downstream_transcript_variant
rs1795037956	intron_variant,genic_downstream_transcript_variant
rs1795038179	intron_variant,genic_downstream_transcript_variant
rs1795038712	intron_variant,genic_downstream_transcript_variant
rs1795038783	intron_variant,genic_downstream_transcript_variant
rs1795039016	intron_variant,genic_downstream_transcript_variant
rs1795039374	intron_variant,genic_downstream_transcript_variant
rs1795040245	intron_variant,genic_downstream_transcript_variant
rs1795040388	intron_variant,genic_downstream_transcript_variant
rs1795040466	intron_variant,genic_downstream_transcript_variant
rs1795041170	intron_variant,genic_downstream_transcript_variant
rs1795041295	intron_variant,genic_downstream_transcript_variant
rs1795041417	intron_variant,genic_downstream_transcript_variant
rs1795041620	intron_variant,genic_downstream_transcript_variant
rs1795041851	intron_variant,genic_downstream_transcript_variant
rs1795041931	intron_variant,genic_downstream_transcript_variant
rs1795042938	intron_variant,genic_downstream_transcript_variant
rs1795043329	intron_variant,genic_downstream_transcript_variant
rs1795043398	intron_variant,genic_downstream_transcript_variant
rs1795043572	intron_variant,genic_downstream_transcript_variant
rs1795043650	intron_variant,genic_downstream_transcript_variant
rs1795044119	intron_variant,genic_downstream_transcript_variant
rs1795044539	intron_variant,genic_downstream_transcript_variant
rs1795044936	intron_variant,genic_downstream_transcript_variant
rs1795045287	intron_variant,genic_downstream_transcript_variant
rs1795045715	intron_variant,genic_downstream_transcript_variant
rs1795045992	intron_variant,genic_downstream_transcript_variant
rs1795046428	intron_variant,genic_downstream_transcript_variant
rs1795046879	intron_variant,genic_downstream_transcript_variant
rs1795047330	intron_variant,genic_downstream_transcript_variant
rs1795047552	intron_variant,genic_downstream_transcript_variant
rs1795047911	intron_variant,genic_downstream_transcript_variant
rs1795048064	intron_variant,genic_downstream_transcript_variant
rs1795048494	intron_variant,genic_downstream_transcript_variant
rs1795048651	intron_variant,genic_downstream_transcript_variant
rs1795048953	intron_variant,genic_downstream_transcript_variant
rs1795049030	intron_variant,genic_downstream_transcript_variant
rs1795049185	intron_variant,genic_downstream_transcript_variant
rs1795049261	intron_variant,genic_downstream_transcript_variant
rs1795049321	intron_variant,genic_downstream_transcript_variant
rs1795049541	intron_variant,genic_downstream_transcript_variant
rs1795049716	intron_variant,genic_downstream_transcript_variant
rs1795050111	intron_variant,genic_downstream_transcript_variant
rs1795050184	intron_variant,genic_downstream_transcript_variant
rs1795050533	intron_variant,genic_downstream_transcript_variant
rs1795050601	intron_variant,genic_downstream_transcript_variant
rs1795050681	intron_variant,genic_downstream_transcript_variant
rs1795050994	intron_variant,genic_downstream_transcript_variant

rs1795051235	intron_variant,genic_downstream_transcript_variant
rs1795051320	intron_variant,genic_downstream_transcript_variant
rs1795051823	intron_variant,genic_downstream_transcript_variant
rs1795052016	intron_variant,genic_downstream_transcript_variant
rs1795052573	intron_variant,genic_downstream_transcript_variant
rs1795052919	intron_variant,genic_downstream_transcript_variant
rs1795052995	intron_variant,genic_downstream_transcript_variant
rs1795053077	intron_variant,genic_downstream_transcript_variant
rs1795053224	intron_variant,genic_downstream_transcript_variant
rs1795053296	intron_variant,genic_downstream_transcript_variant
rs1795053353	intron_variant,genic_downstream_transcript_variant
rs1795053670	intron_variant,genic_downstream_transcript_variant
rs1795053811	intron_variant,genic_downstream_transcript_variant
rs1795054217	intron_variant,genic_downstream_transcript_variant
rs1795054366	intron_variant,genic_downstream_transcript_variant
rs1795054660	intron_variant,genic_downstream_transcript_variant
rs1795054724	intron_variant,genic_downstream_transcript_variant
rs1795054798	intron_variant,genic_downstream_transcript_variant
rs1795054873	intron_variant,genic_downstream_transcript_variant
rs1795054953	intron_variant,genic_downstream_transcript_variant
rs1795055158	intron_variant,genic_downstream_transcript_variant
rs1795055218	intron_variant,genic_downstream_transcript_variant
rs1795055624	intron_variant,genic_downstream_transcript_variant
rs1795055697	intron_variant,genic_downstream_transcript_variant
rs1795056105	intron_variant,genic_downstream_transcript_variant
rs1795056184	intron_variant,genic_downstream_transcript_variant
rs1795056336	intron_variant,genic_downstream_transcript_variant
rs1795056541	intron_variant,genic_downstream_transcript_variant
rs1795056742	intron_variant,genic_downstream_transcript_variant
rs1795057208	intron_variant,genic_downstream_transcript_variant
rs1795057276	intron_variant,genic_downstream_transcript_variant
rs1795057721	intron_variant,genic_downstream_transcript_variant
rs1795057871	intron_variant,genic_downstream_transcript_variant
rs1795058117	intron_variant,genic_downstream_transcript_variant
rs1795058234	intron_variant,genic_downstream_transcript_variant
rs1795058575	intron_variant,genic_downstream_transcript_variant
rs1795058840	intron_variant,genic_downstream_transcript_variant
rs1795058912	intron_variant,genic_downstream_transcript_variant
rs1795059248	intron_variant,genic_downstream_transcript_variant
rs1795059341	intron_variant,genic_downstream_transcript_variant
rs1795059827	intron_variant,genic_downstream_transcript_variant
rs1795060000	intron_variant,genic_downstream_transcript_variant
rs1795060419	intron_variant,genic_downstream_transcript_variant
rs1795060536	intron_variant,genic_downstream_transcript_variant
rs1795060776	intron_variant,genic_downstream_transcript_variant
rs1795061419	intron_variant,genic_downstream_transcript_variant
rs1795061804	intron_variant,genic_downstream_transcript_variant
rs1795062056	intron_variant,genic_downstream_transcript_variant
rs1795062587	intron_variant,genic_downstream_transcript_variant
rs1795062881	intron_variant,genic_downstream_transcript_variant
rs1795063485	intron_variant,genic_downstream_transcript_variant
rs1795063618	intron_variant,genic_downstream_transcript_variant
rs1795063871	intron_variant,genic_downstream_transcript_variant
rs1795064250	intron_variant,genic_downstream_transcript_variant
rs1795064556	intron_variant,genic_downstream_transcript_variant
rs1795065571	intron_variant,genic_downstream_transcript_variant
rs1795066067	non_coding_transcript_variant,coding_sequence_variant,synonymous_variant,genic_downstream_transcript_variant
rs1795066858	missense_variant,non_coding_transcript_variant,coding_sequence_variant,genic_downstream_transcript_variant
rs1795068335	missense_variant,non_coding_transcript_variant,coding_sequence_variant,genic_downstream_transcript_variant
rs1795069074	missense_variant,non_coding_transcript_variant,coding_sequence_variant,genic_downstream_transcript_variant
rs1795069423	missense_variant,non_coding_transcript_variant,coding_sequence_variant,genic_downstream_transcript_variant
rs1795070786	missense_variant,non_coding_transcript_variant,coding_sequence_variant,genic_downstream_transcript_variant
rs1795071709	missense_variant,non_coding_transcript_variant,coding_sequence_variant,genic_downstream_transcript_variant
rs1795075053	intron_variant,genic_downstream_transcript_variant
rs1795075491	intron_variant,genic_downstream_transcript_variant
rs1795076008	intron_variant,genic_downstream_transcript_variant
rs1795076363	intron_variant,genic_downstream_transcript_variant
rs1795076492	intron_variant,genic_downstream_transcript_variant
rs1795077582	intron_variant,genic_downstream_transcript_variant
rs1795077695	intron_variant,genic_downstream_transcript_variant
rs1795078037	intron_variant,genic_downstream_transcript_variant
rs1795078172	intron_variant,genic_downstream_transcript_variant
rs1795079116	intron_variant,genic_downstream_transcript_variant
rs1795079222	intron_variant,genic_downstream_transcript_variant

rs1795079581	intron_variant,genic_downstream_transcript_variant
rs1795079986	intron_variant,genic_downstream_transcript_variant
rs1795080464	intron_variant,genic_downstream_transcript_variant
rs1795080542	intron_variant,genic_downstream_transcript_variant
rs1795081265	intron_variant,genic_downstream_transcript_variant
rs1795081339	intron_variant,genic_downstream_transcript_variant
rs1795081579	intron_variant,genic_downstream_transcript_variant
rs1795081666	intron_variant,genic_downstream_transcript_variant
rs1795081739	intron_variant,genic_downstream_transcript_variant
rs1795081814	intron_variant,genic_downstream_transcript_variant
rs1795081893	intron_variant,genic_downstream_transcript_variant
rs1795082019	intron_variant,genic_downstream_transcript_variant
rs1795082087	intron_variant,genic_downstream_transcript_variant
rs1795082327	intron_variant,genic_downstream_transcript_variant
rs1795082588	intron_variant,genic_downstream_transcript_variant
rs1795082668	intron_variant,genic_downstream_transcript_variant
rs1795082731	intron_variant,genic_downstream_transcript_variant
rs1795082808	intron_variant,genic_downstream_transcript_variant
rs1795082917	intron_variant,genic_downstream_transcript_variant
rs1795083001	intron_variant,genic_downstream_transcript_variant
rs1795083078	intron_variant,genic_downstream_transcript_variant
rs1795083151	intron_variant,genic_downstream_transcript_variant
rs1795083851	intron_variant,genic_downstream_transcript_variant
rs1795084423	intron_variant,genic_downstream_transcript_variant
rs1795084690	intron_variant,genic_downstream_transcript_variant
rs1795084926	intron_variant,genic_downstream_transcript_variant
rs1795085835	intron_variant,genic_downstream_transcript_variant
rs1795085953	intron_variant,genic_downstream_transcript_variant
rs1795086024	intron_variant,genic_downstream_transcript_variant
rs1795086280	intron_variant,genic_downstream_transcript_variant
rs1795086349	intron_variant,genic_downstream_transcript_variant
rs1795086434	intron_variant,genic_downstream_transcript_variant
rs1795086598	intron_variant,genic_downstream_transcript_variant
rs1795087121	intron_variant,genic_downstream_transcript_variant
rs1795087794	intron_variant,genic_downstream_transcript_variant
rs1795087965	intron_variant,genic_downstream_transcript_variant
rs1795088420	intron_variant,genic_downstream_transcript_variant
rs1795088843	intron_variant,genic_downstream_transcript_variant
rs1795089780	intron_variant,genic_downstream_transcript_variant
rs1795089982	intron_variant,genic_downstream_transcript_variant
rs1795090667	intron_variant,genic_downstream_transcript_variant
rs1795090888	intron_variant,genic_downstream_transcript_variant
rs1795091576	intron_variant,genic_downstream_transcript_variant
rs1795091642	intron_variant,genic_downstream_transcript_variant
rs1795091812	intron_variant,genic_downstream_transcript_variant
rs1795091898	intron_variant,genic_downstream_transcript_variant
rs1795092054	intron_variant,genic_downstream_transcript_variant
rs1795092139	intron_variant,genic_downstream_transcript_variant
rs1795092214	intron_variant,genic_downstream_transcript_variant
rs1795092284	intron_variant,genic_downstream_transcript_variant
rs1795092664	intron_variant,genic_downstream_transcript_variant
rs1795092864	intron_variant,genic_downstream_transcript_variant
rs1795092946	intron_variant,genic_downstream_transcript_variant
rs1795093197	intron_variant,genic_downstream_transcript_variant
rs1795093524	intron_variant,genic_downstream_transcript_variant
rs1795093602	intron_variant,genic_downstream_transcript_variant
rs1795094145	intron_variant,genic_downstream_transcript_variant
rs1795094469	intron_variant,genic_downstream_transcript_variant
rs1795094547	intron_variant,genic_downstream_transcript_variant
rs1795094831	intron_variant,genic_downstream_transcript_variant
rs1795094930	intron_variant,genic_downstream_transcript_variant
rs1795095276	intron_variant,genic_downstream_transcript_variant
rs1795095491	intron_variant,genic_downstream_transcript_variant
rs1795095809	intron_variant,genic_downstream_transcript_variant
rs1795095976	intron_variant,genic_downstream_transcript_variant
rs1795096047	intron_variant,genic_downstream_transcript_variant
rs1795097339	intron_variant,genic_downstream_transcript_variant
rs1795097775	intron_variant,genic_downstream_transcript_variant
rs1795098019	intron_variant,genic_downstream_transcript_variant
rs1795098145	intron_variant,genic_downstream_transcript_variant
rs1795098371	intron_variant,genic_downstream_transcript_variant
rs1795098436	intron_variant,genic_downstream_transcript_variant
rs1795098778	intron_variant,genic_downstream_transcript_variant
rs1795099495	intron_variant,genic_downstream_transcript_variant

rs1795099573	intron_variant,genic_downstream_transcript_variant
rs1795099948	intron_variant,genic_downstream_transcript_variant
rs1795100106	intron_variant,genic_downstream_transcript_variant
rs1795100454	intron_variant,genic_downstream_transcript_variant
rs1795100738	intron_variant,genic_downstream_transcript_variant
rs1795100811	intron_variant,genic_downstream_transcript_variant
rs1795100978	intron_variant,genic_downstream_transcript_variant
rs1795101233	intron_variant,genic_downstream_transcript_variant
rs1795101302	intron_variant,genic_downstream_transcript_variant
rs1795101467	intron_variant,genic_downstream_transcript_variant
rs1795101645	intron_variant,genic_downstream_transcript_variant
rs1795101937	intron_variant,genic_downstream_transcript_variant
rs1795102026	intron_variant,genic_downstream_transcript_variant
rs1795102263	intron_variant,genic_downstream_transcript_variant
rs1795102666	intron_variant,genic_downstream_transcript_variant
rs1795102924	intron_variant,genic_downstream_transcript_variant
rs1795103502	intron_variant,genic_downstream_transcript_variant
rs1795103592	intron_variant,genic_downstream_transcript_variant
rs1795104197	intron_variant,genic_downstream_transcript_variant
rs1795104335	intron_variant,genic_downstream_transcript_variant
rs1795104414	intron_variant,genic_downstream_transcript_variant
rs1795104495	intron_variant,genic_downstream_transcript_variant
rs1795104691	intron_variant,genic_downstream_transcript_variant
rs1795105010	intron_variant,genic_downstream_transcript_variant
rs1795105747	intron_variant,genic_downstream_transcript_variant
rs1795105837	intron_variant,genic_downstream_transcript_variant
rs1795105931	intron_variant,genic_downstream_transcript_variant
rs1795106273	intron_variant,genic_downstream_transcript_variant
rs1795106365	intron_variant,genic_downstream_transcript_variant
rs1795106945	intron_variant,genic_downstream_transcript_variant
rs1795107241	intron_variant,genic_downstream_transcript_variant
rs1795107635	intron_variant,genic_downstream_transcript_variant
rs1795107948	intron_variant,genic_downstream_transcript_variant
rs1795108092	intron_variant,genic_downstream_transcript_variant
rs1795108165	intron_variant,genic_downstream_transcript_variant
rs1795108242	intron_variant,genic_downstream_transcript_variant
rs1795108480	intron_variant,genic_downstream_transcript_variant
rs1795108546	intron_variant,genic_downstream_transcript_variant
rs1795108628	intron_variant,genic_downstream_transcript_variant
rs1795108699	intron_variant,genic_downstream_transcript_variant
rs1795108861	intron_variant,genic_downstream_transcript_variant
rs1795109432	intron_variant,genic_downstream_transcript_variant
rs1795109496	intron_variant,genic_downstream_transcript_variant
rs1795109633	intron_variant,genic_downstream_transcript_variant
rs1795109800	intron_variant,genic_downstream_transcript_variant
rs1795110291	intron_variant,genic_downstream_transcript_variant
rs1795110539	intron_variant,genic_downstream_transcript_variant
rs1795110705	intron_variant,genic_downstream_transcript_variant
rs1795110782	intron_variant,genic_downstream_transcript_variant
rs1795111300	intron_variant,genic_downstream_transcript_variant
rs1795111365	intron_variant,genic_downstream_transcript_variant
rs1795111731	intron_variant,genic_downstream_transcript_variant
rs1795111793	intron_variant,genic_downstream_transcript_variant
rs1795111852	intron_variant,genic_downstream_transcript_variant
rs1795112226	intron_variant,genic_downstream_transcript_variant
rs1795112342	intron_variant,genic_downstream_transcript_variant
rs1795112470	intron_variant,genic_downstream_transcript_variant
rs1795112626	intron_variant,genic_downstream_transcript_variant
rs1795113019	intron_variant,genic_downstream_transcript_variant
rs1795113115	intron_variant,genic_downstream_transcript_variant
rs1795113356	intron_variant,genic_downstream_transcript_variant
rs1795113463	intron_variant,genic_downstream_transcript_variant
rs1795113746	intron_variant,genic_downstream_transcript_variant
rs1795113855	intron_variant,genic_downstream_transcript_variant
rs1795114348	intron_variant,genic_downstream_transcript_variant
rs1795114464	intron_variant,genic_downstream_transcript_variant
rs1795116156	intron_variant,genic_downstream_transcript_variant
rs1795116486	intron_variant,genic_downstream_transcript_variant
rs1795116603	intron_variant,genic_downstream_transcript_variant
rs1795116851	intron_variant,genic_downstream_transcript_variant
rs1795117415	intron_variant,genic_downstream_transcript_variant
rs1795117899	intron_variant,genic_downstream_transcript_variant
rs1795117987	intron_variant,genic_downstream_transcript_variant
rs1795118504	intron_variant,genic_downstream_transcript_variant

rs1795118601	intron_variant,genic_downstream_transcript_variant
rs1795118790	intron_variant,genic_downstream_transcript_variant
rs1795118959	intron_variant,genic_downstream_transcript_variant
rs1795119821	intron_variant,genic_downstream_transcript_variant
rs1795120077	intron_variant,genic_downstream_transcript_variant
rs1795120349	intron_variant,genic_downstream_transcript_variant
rs1795120944	intron_variant,genic_downstream_transcript_variant
rs1795121011	intron_variant,genic_downstream_transcript_variant
rs1795121165	intron_variant,genic_downstream_transcript_variant
rs1795121241	intron_variant,genic_downstream_transcript_variant
rs1795121996	intron_variant,genic_downstream_transcript_variant
rs1795122161	intron_variant,genic_downstream_transcript_variant
rs1795123136	intron_variant,genic_downstream_transcript_variant
rs1795123383	intron_variant,genic_downstream_transcript_variant
rs1795123511	intron_variant,genic_downstream_transcript_variant
rs1795123585	intron_variant,genic_downstream_transcript_variant
rs1795123797	intron_variant,genic_downstream_transcript_variant
rs1795123876	intron_variant,genic_downstream_transcript_variant
rs1795124243	intron_variant,genic_downstream_transcript_variant
rs1795124346	intron_variant,genic_downstream_transcript_variant
rs1795124799	intron_variant,genic_downstream_transcript_variant
rs1795124901	intron_variant,genic_downstream_transcript_variant
rs1795125224	intron_variant,genic_downstream_transcript_variant
rs1795125370	intron_variant,genic_downstream_transcript_variant
rs1795125526	intron_variant,genic_downstream_transcript_variant
rs1795125600	intron_variant,genic_downstream_transcript_variant
rs1795125947	intron_variant,genic_downstream_transcript_variant
rs1795126285	intron_variant,genic_downstream_transcript_variant
rs1795126650	intron_variant,genic_downstream_transcript_variant
rs1795126883	intron_variant,genic_downstream_transcript_variant
rs1795126970	intron_variant,genic_downstream_transcript_variant
rs1795127421	intron_variant,genic_downstream_transcript_variant
rs1795127581	intron_variant,genic_downstream_transcript_variant
rs1795127666	intron_variant,genic_downstream_transcript_variant
rs1795127923	intron_variant,genic_downstream_transcript_variant
rs1795128006	intron_variant,genic_downstream_transcript_variant
rs1795128118	intron_variant,genic_downstream_transcript_variant
rs1795128672	intron_variant,genic_downstream_transcript_variant
rs1795128862	intron_variant,genic_downstream_transcript_variant
rs1795129425	intron_variant,genic_downstream_transcript_variant
rs1795129759	intron_variant,genic_downstream_transcript_variant
rs1795129828	intron_variant,genic_downstream_transcript_variant
rs1795129897	intron_variant,genic_downstream_transcript_variant
rs1795129989	intron_variant,genic_downstream_transcript_variant
rs1795130174	intron_variant,genic_downstream_transcript_variant
rs1795130521	intron_variant,genic_downstream_transcript_variant
rs1795131008	intron_variant,genic_downstream_transcript_variant
rs1795131088	intron_variant,genic_downstream_transcript_variant
rs1795131873	intron_variant,genic_downstream_transcript_variant
rs1795131954	intron_variant,genic_downstream_transcript_variant
rs1795132083	intron_variant,genic_downstream_transcript_variant
rs1795132561	intron_variant,genic_downstream_transcript_variant
rs1795132638	intron_variant,genic_downstream_transcript_variant
rs1795132771	intron_variant,genic_downstream_transcript_variant
rs1795132916	intron_variant,genic_downstream_transcript_variant
rs1795132980	intron_variant,genic_downstream_transcript_variant
rs1795133132	intron_variant,genic_downstream_transcript_variant
rs1795133359	intron_variant,genic_downstream_transcript_variant
rs1795134241	intron_variant,genic_downstream_transcript_variant
rs1795134365	intron_variant,genic_downstream_transcript_variant
rs1795134733	intron_variant,genic_downstream_transcript_variant
rs1795134822	intron_variant,genic_downstream_transcript_variant
rs1795135059	intron_variant,genic_downstream_transcript_variant
rs1795135301	intron_variant,genic_downstream_transcript_variant
rs1795135425	intron_variant,genic_downstream_transcript_variant
rs1795135576	intron_variant,genic_downstream_transcript_variant
rs1795135680	intron_variant,genic_downstream_transcript_variant
rs1795136135	intron_variant,genic_downstream_transcript_variant
rs1795136552	intron_variant,genic_downstream_transcript_variant
rs1795136700	intron_variant,genic_downstream_transcript_variant
rs1795136758	intron_variant,genic_downstream_transcript_variant
rs1795137400	intron_variant,genic_downstream_transcript_variant
rs1795137639	intron_variant,genic_downstream_transcript_variant
rs1795138188	intron_variant,genic_downstream_transcript_variant

rs1795138696	intron_variant,genic_downstream_transcript_variant
rs1795138776	intron_variant,genic_downstream_transcript_variant
rs1795139106	intron_variant,genic_downstream_transcript_variant
rs1795139398	intron_variant,genic_downstream_transcript_variant
rs1795139828	intron_variant,genic_downstream_transcript_variant
rs1795140056	intron_variant,genic_downstream_transcript_variant
rs1795140189	intron_variant,genic_downstream_transcript_variant
rs1795140300	intron_variant,genic_downstream_transcript_variant
rs1795140702	intron_variant,genic_downstream_transcript_variant
rs1795140939	intron_variant,genic_downstream_transcript_variant
rs1795141190	intron_variant,genic_downstream_transcript_variant
rs1795141269	intron_variant,genic_downstream_transcript_variant
rs1795141344	intron_variant,genic_downstream_transcript_variant
rs1795141821	intron_variant,genic_downstream_transcript_variant
rs1795141991	intron_variant,genic_downstream_transcript_variant
rs1795142945	intron_variant,genic_downstream_transcript_variant
rs1795143027	intron_variant,genic_downstream_transcript_variant
rs1795143604	intron_variant,genic_downstream_transcript_variant
rs1795143831	intron_variant,genic_downstream_transcript_variant
rs1795144112	intron_variant,genic_downstream_transcript_variant
rs1795144179	intron_variant,genic_downstream_transcript_variant
rs1795144355	intron_variant,genic_downstream_transcript_variant
rs1795144450	intron_variant,genic_downstream_transcript_variant
rs1795144716	intron_variant,genic_downstream_transcript_variant
rs1795144967	intron_variant,genic_downstream_transcript_variant
rs1795145037	intron_variant,genic_downstream_transcript_variant
rs1795145209	intron_variant,genic_downstream_transcript_variant
rs1795145288	intron_variant,genic_downstream_transcript_variant
rs1795145620	intron_variant,genic_downstream_transcript_variant
rs1795145833	intron_variant,genic_downstream_transcript_variant
rs1795146309	intron_variant,genic_downstream_transcript_variant
rs1795146529	intron_variant,genic_downstream_transcript_variant
rs1795146614	intron_variant,genic_downstream_transcript_variant
rs1795147113	intron_variant,genic_downstream_transcript_variant
rs1795147408	intron_variant,genic_downstream_transcript_variant
rs1795147760	intron_variant,genic_downstream_transcript_variant
rs1795148006	intron_variant,genic_downstream_transcript_variant
rs1795148077	intron_variant,genic_downstream_transcript_variant
rs1795148163	intron_variant,genic_downstream_transcript_variant
rs1795148249	intron_variant,genic_downstream_transcript_variant
rs1795148829	intron_variant,genic_downstream_transcript_variant
rs1795148957	intron_variant,genic_downstream_transcript_variant
rs1795149031	intron_variant,genic_downstream_transcript_variant
rs1795149498	intron_variant,genic_downstream_transcript_variant
rs1795149888	intron_variant,genic_downstream_transcript_variant
rs1795150455	intron_variant,genic_downstream_transcript_variant
rs1795150672	intron_variant,genic_downstream_transcript_variant
rs1795150841	intron_variant,genic_downstream_transcript_variant
rs1795151169	intron_variant,genic_downstream_transcript_variant
rs1795151261	intron_variant,genic_downstream_transcript_variant
rs1795151347	intron_variant,genic_downstream_transcript_variant
rs1795151539	intron_variant,genic_downstream_transcript_variant
rs1795151629	intron_variant,genic_downstream_transcript_variant
rs1795151769	intron_variant,genic_downstream_transcript_variant
rs1795151986	intron_variant,genic_downstream_transcript_variant
rs1795152051	intron_variant,genic_downstream_transcript_variant
rs1795152136	intron_variant,genic_downstream_transcript_variant
rs1795152598	intron_variant,genic_downstream_transcript_variant
rs1795152664	intron_variant,genic_downstream_transcript_variant
rs1795152819	intron_variant,genic_downstream_transcript_variant
rs1795152976	intron_variant,genic_downstream_transcript_variant
rs1795153135	intron_variant,genic_downstream_transcript_variant
rs1795153201	intron_variant,genic_downstream_transcript_variant
rs1795153361	intron_variant,genic_downstream_transcript_variant
rs1795153788	intron_variant,genic_downstream_transcript_variant
rs1795153990	intron_variant,genic_downstream_transcript_variant
rs1795154459	intron_variant,genic_downstream_transcript_variant
rs1795154543	intron_variant,genic_downstream_transcript_variant
rs1795154680	intron_variant,genic_downstream_transcript_variant
rs1795154763	intron_variant,genic_downstream_transcript_variant
rs1795154928	intron_variant,genic_downstream_transcript_variant
rs1795155350	intron_variant,genic_downstream_transcript_variant
rs1795155424	intron_variant,genic_downstream_transcript_variant
rs1795155497	intron_variant,genic_downstream_transcript_variant

rs1795155579	intron_variant,genic_downstream_transcript_variant
rs1795155697	intron_variant,genic_downstream_transcript_variant
rs1795155947	intron_variant,genic_downstream_transcript_variant
rs1795156065	intron_variant,genic_downstream_transcript_variant
rs1795156166	intron_variant,genic_downstream_transcript_variant
rs1795156738	intron_variant,genic_downstream_transcript_variant
rs1795157100	intron_variant,genic_downstream_transcript_variant
rs1795157377	intron_variant,genic_downstream_transcript_variant
rs1795157664	intron_variant,genic_downstream_transcript_variant
rs1795158469	intron_variant,genic_downstream_transcript_variant
rs1795158566	intron_variant,genic_downstream_transcript_variant
rs1795158887	intron_variant,genic_downstream_transcript_variant
rs1795158965	intron_variant,genic_downstream_transcript_variant
rs1795159050	intron_variant,genic_downstream_transcript_variant
rs1795159136	intron_variant,genic_downstream_transcript_variant
rs1795159286	intron_variant,genic_downstream_transcript_variant
rs1795159363	intron_variant,genic_downstream_transcript_variant
rs1795159431	intron_variant,genic_downstream_transcript_variant
rs1795160028	intron_variant,genic_downstream_transcript_variant
rs1795160242	intron_variant,genic_downstream_transcript_variant
rs1795160459	intron_variant,genic_downstream_transcript_variant
rs1795160713	intron_variant,genic_downstream_transcript_variant
rs1795160784	intron_variant,genic_downstream_transcript_variant
rs1795160868	intron_variant,genic_downstream_transcript_variant
rs1795161064	intron_variant,genic_downstream_transcript_variant
rs1795161172	intron_variant,genic_downstream_transcript_variant
rs1795161254	intron_variant,genic_downstream_transcript_variant
rs1795161528	intron_variant,genic_downstream_transcript_variant
rs1795161607	intron_variant,genic_downstream_transcript_variant
rs1795161687	intron_variant,genic_downstream_transcript_variant
rs1795161764	intron_variant,genic_downstream_transcript_variant
rs1795162118	intron_variant,genic_downstream_transcript_variant
rs1795162274	intron_variant,genic_downstream_transcript_variant
rs1795163142	intron_variant,genic_downstream_transcript_variant
rs1795163233	intron_variant,genic_downstream_transcript_variant
rs1795163717	intron_variant,genic_downstream_transcript_variant
rs1795164827	intron_variant,genic_downstream_transcript_variant
rs1795164957	intron_variant,genic_downstream_transcript_variant
rs1795165254	intron_variant,genic_downstream_transcript_variant
rs1795165365	intron_variant,genic_downstream_transcript_variant
rs1795165484	intron_variant,genic_downstream_transcript_variant
rs1795165611	intron_variant,genic_downstream_transcript_variant
rs1795167058	intron_variant,genic_downstream_transcript_variant
rs1795167291	intron_variant,genic_downstream_transcript_variant
rs1795167509	intron_variant,genic_downstream_transcript_variant
rs1795168576	intron_variant,genic_downstream_transcript_variant
rs1795169393	intron_variant,genic_downstream_transcript_variant
rs1795169635	intron_variant,genic_downstream_transcript_variant
rs1795170219	missense_variant,non_coding_transcript_variant,coding_sequence_variant,genic_downstream_transcript_variant
rs1795170374	non_coding_transcript_variant,coding_sequence_variant,synonymous_variant,genic_downstream_transcript_variant
rs1795171447	missense_variant,non_coding_transcript_variant,coding_sequence_variant,genic_downstream_transcript_variant
rs1795171887	missense_variant,non_coding_transcript_variant,coding_sequence_variant,genic_downstream_transcript_variant
rs1795173431	missense_variant,non_coding_transcript_variant,coding_sequence_variant,genic_downstream_transcript_variant
rs1795174174	non_coding_transcript_variant,coding_sequence_variant,synonymous_variant,genic_downstream_transcript_variant
rs1795175313	missense_variant,non_coding_transcript_variant,coding_sequence_variant,genic_downstream_transcript_variant
rs1795175502	missense_variant,non_coding_transcript_variant,coding_sequence_variant,genic_downstream_transcript_variant
rs1795175584	intron_variant,genic_downstream_transcript_variant
rs1795176147	intron_variant,genic_downstream_transcript_variant
rs1795176748	intron_variant,genic_downstream_transcript_variant
rs1795176838	intron_variant,genic_downstream_transcript_variant
rs1795176909	intron_variant,genic_downstream_transcript_variant
rs1795176970	intron_variant,genic_downstream_transcript_variant
rs1795177119	intron_variant,genic_downstream_transcript_variant
rs1795177250	intron_variant,genic_downstream_transcript_variant
rs1795177906	intron_variant,genic_downstream_transcript_variant
rs1795178086	intron_variant,genic_downstream_transcript_variant
rs1795178158	intron_variant,genic_downstream_transcript_variant
rs1795178374	intron_variant,genic_downstream_transcript_variant
rs1795178586	intron_variant,genic_downstream_transcript_variant
rs1795179194	intron_variant,genic_downstream_transcript_variant
rs1795180019	intron_variant,genic_downstream_transcript_variant
rs1795180169	intron_variant,genic_downstream_transcript_variant
rs1795180335	intron_variant,genic_downstream_transcript_variant
rs1795180634	intron_variant,genic_downstream_transcript_variant

rs1795181038	intron_variant,genic_downstream_transcript_variant
rs1795181194	intron_variant,genic_downstream_transcript_variant
rs1795181531	intron_variant,genic_downstream_transcript_variant
rs1795181615	intron_variant,genic_downstream_transcript_variant
rs1795181730	intron_variant,genic_downstream_transcript_variant
rs1795182252	intron_variant,genic_downstream_transcript_variant
rs1795182361	intron_variant,genic_downstream_transcript_variant
rs1795182484	intron_variant,genic_downstream_transcript_variant
rs1795182725	intron_variant,genic_downstream_transcript_variant
rs1795182832	intron_variant,genic_downstream_transcript_variant
rs1795183054	intron_variant,genic_downstream_transcript_variant
rs1795183353	intron_variant,genic_downstream_transcript_variant
rs1795183423	intron_variant,genic_downstream_transcript_variant
rs1795183580	intron_variant,genic_downstream_transcript_variant
rs1795184160	intron_variant,genic_downstream_transcript_variant
rs1795184837	intron_variant,genic_downstream_transcript_variant
rs1795184970	intron_variant,genic_downstream_transcript_variant
rs1795185358	intron_variant,genic_downstream_transcript_variant
rs1795186172	intron_variant,genic_downstream_transcript_variant
rs1795186428	intron_variant,genic_downstream_transcript_variant
rs1795186534	intron_variant,genic_downstream_transcript_variant
rs1795186784	intron_variant,genic_downstream_transcript_variant
rs1795186909	intron_variant,genic_downstream_transcript_variant
rs1795187049	intron_variant,genic_downstream_transcript_variant
rs1795187531	intron_variant,genic_downstream_transcript_variant
rs1795187950	intron_variant,genic_downstream_transcript_variant
rs1795188187	intron_variant,genic_downstream_transcript_variant
rs1795188628	intron_variant,genic_downstream_transcript_variant
rs1795188799	intron_variant,genic_downstream_transcript_variant
rs1795189329	intron_variant,genic_downstream_transcript_variant
rs1795189894	intron_variant,genic_downstream_transcript_variant
rs1795189970	intron_variant,genic_downstream_transcript_variant
rs1795190189	intron_variant,genic_downstream_transcript_variant
rs1795190582	intron_variant,genic_downstream_transcript_variant
rs1795190801	intron_variant,genic_downstream_transcript_variant
rs1795191203	intron_variant,genic_downstream_transcript_variant
rs1795191464	intron_variant,genic_downstream_transcript_variant
rs1795191610	intron_variant,genic_downstream_transcript_variant
rs1795191692	intron_variant,genic_downstream_transcript_variant
rs1795191836	intron_variant,genic_downstream_transcript_variant
rs1795192012	intron_variant,genic_downstream_transcript_variant
rs1795192183	intron_variant,genic_downstream_transcript_variant
rs1795192698	intron_variant,genic_downstream_transcript_variant
rs1795193169	intron_variant,genic_downstream_transcript_variant
rs1795193862	intron_variant,genic_downstream_transcript_variant
rs1795194001	intron_variant,genic_downstream_transcript_variant
rs1795194159	intron_variant,genic_downstream_transcript_variant
rs1795194247	intron_variant,genic_downstream_transcript_variant
rs1795194407	intron_variant,genic_downstream_transcript_variant
rs1795194654	intron_variant,genic_downstream_transcript_variant
rs1795194820	intron_variant,genic_downstream_transcript_variant
rs1795194988	intron_variant,genic_downstream_transcript_variant
rs1795195071	intron_variant,genic_downstream_transcript_variant
rs1795195345	intron_variant,genic_downstream_transcript_variant
rs1795195533	intron_variant,genic_downstream_transcript_variant
rs1795196550	intron_variant,genic_downstream_transcript_variant
rs1795196657	intron_variant,genic_downstream_transcript_variant
rs1795196907	intron_variant,genic_downstream_transcript_variant
rs1795197108	intron_variant,genic_downstream_transcript_variant
rs1795197195	intron_variant,genic_downstream_transcript_variant
rs1795197659	intron_variant,genic_downstream_transcript_variant
rs1795198389	intron_variant,genic_downstream_transcript_variant
rs1795198595	intron_variant,genic_downstream_transcript_variant
rs1795198670	intron_variant,genic_downstream_transcript_variant
rs1795198991	intron_variant,genic_downstream_transcript_variant
rs1795199070	intron_variant,genic_downstream_transcript_variant
rs1795199282	intron_variant,genic_downstream_transcript_variant
rs1795199375	intron_variant,genic_downstream_transcript_variant
rs1795199574	intron_variant,genic_downstream_transcript_variant
rs1795199817	intron_variant,genic_downstream_transcript_variant
rs1795199886	intron_variant,genic_downstream_transcript_variant
rs1795199965	intron_variant,genic_downstream_transcript_variant
rs1795200030	intron_variant,genic_downstream_transcript_variant
rs1795200389	intron_variant,genic_downstream_transcript_variant

rs1795200476	intron_variant,genic_downstream_transcript_variant
rs1795200561	intron_variant,genic_downstream_transcript_variant
rs1795200640	intron_variant,genic_downstream_transcript_variant
rs1795200716	intron_variant,genic_downstream_transcript_variant
rs1795200812	intron_variant,genic_downstream_transcript_variant
rs1795201338	intron_variant,genic_downstream_transcript_variant
rs1795201903	intron_variant,genic_downstream_transcript_variant
rs1795201970	intron_variant,genic_downstream_transcript_variant
rs1795202458	intron_variant,genic_downstream_transcript_variant
rs1795202664	intron_variant,genic_downstream_transcript_variant
rs1795203184	intron_variant,genic_downstream_transcript_variant
rs1795203252	intron_variant,genic_downstream_transcript_variant
rs1795203486	intron_variant,genic_downstream_transcript_variant
rs1795203899	intron_variant,genic_downstream_transcript_variant
rs1795204148	intron_variant,genic_downstream_transcript_variant
rs1795204283	intron_variant,genic_downstream_transcript_variant
rs1795204348	intron_variant,genic_downstream_transcript_variant
rs1795205130	intron_variant,genic_downstream_transcript_variant
rs1795206092	intron_variant,genic_downstream_transcript_variant
rs1795206173	intron_variant,genic_downstream_transcript_variant
rs1795206620	intron_variant,genic_downstream_transcript_variant
rs1795206855	missense_variant,non_coding_transcript_variant,coding_sequence_variant,genic_downstream_transcript_variant
rs1795207276	non_coding_transcript_variant,coding_sequence_variant,synonymous_variant,genic_downstream_transcript_variant
rs1795208410	missense_variant,non_coding_transcript_variant,coding_sequence_variant,genic_downstream_transcript_variant
rs1795213844	missense_variant,non_coding_transcript_variant,coding_sequence_variant,genic_downstream_transcript_variant
rs1795213981	non_coding_transcript_variant,coding_sequence_variant,synonymous_variant,genic_downstream_transcript_variant
rs1795214378	non_coding_transcript_variant,coding_sequence_variant,synonymous_variant,genic_downstream_transcript_variant
rs1795215629	intron_variant,genic_downstream_transcript_variant
rs1795215784	intron_variant,genic_downstream_transcript_variant
rs1795215896	intron_variant,genic_downstream_transcript_variant
rs1795216248	intron_variant,genic_downstream_transcript_variant
rs1795216364	intron_variant,genic_downstream_transcript_variant
rs1795216655	intron_variant,genic_downstream_transcript_variant
rs1795216784	intron_variant,genic_downstream_transcript_variant
rs1795217543	intron_variant,genic_downstream_transcript_variant
rs1795217686	intron_variant,genic_downstream_transcript_variant
rs1795217915	intron_variant,genic_downstream_transcript_variant
rs1795219069	intron_variant,genic_downstream_transcript_variant
rs1795219139	intron_variant,genic_downstream_transcript_variant
rs1795219372	intron_variant,genic_downstream_transcript_variant
rs1795219445	intron_variant,genic_downstream_transcript_variant
rs1795219820	intron_variant,genic_downstream_transcript_variant
rs1795220190	intron_variant,genic_downstream_transcript_variant
rs1795220206	intron_variant
rs1795220329	intron_variant,genic_downstream_transcript_variant
rs1795220411	intron_variant,genic_downstream_transcript_variant
rs1795220480	intron_variant,genic_downstream_transcript_variant
rs1795220623	intron_variant,genic_downstream_transcript_variant
rs1795221294	intron_variant,genic_downstream_transcript_variant
rs1795221676	intron_variant,genic_downstream_transcript_variant
rs1795221757	intron_variant,genic_downstream_transcript_variant
rs1795221836	intron_variant,genic_downstream_transcript_variant
rs1795221904	intron_variant,genic_downstream_transcript_variant
rs1795222033	intron_variant,genic_downstream_transcript_variant
rs1795222107	intron_variant,genic_downstream_transcript_variant
rs1795222752	intron_variant,genic_downstream_transcript_variant
rs1795222813	intron_variant,genic_downstream_transcript_variant
rs1795223532	intron_variant,genic_downstream_transcript_variant
rs1795223606	intron_variant,genic_downstream_transcript_variant
rs1795223736	intron_variant,genic_downstream_transcript_variant
rs1795224199	intron_variant,genic_downstream_transcript_variant
rs1795224530	intron_variant,genic_downstream_transcript_variant
rs1795224798	intron_variant,genic_downstream_transcript_variant
rs1795225266	intron_variant,genic_downstream_transcript_variant
rs1795225348	intron_variant,genic_downstream_transcript_variant
rs1795226034	intron_variant,genic_downstream_transcript_variant
rs1795226305	intron_variant,genic_downstream_transcript_variant
rs1795227205	intron_variant,genic_downstream_transcript_variant
rs1795227290	intron_variant,genic_downstream_transcript_variant
rs1795227360	intron_variant,genic_downstream_transcript_variant
rs1795227451	intron_variant,genic_downstream_transcript_variant
rs1795227714	intron_variant,genic_downstream_transcript_variant
rs1795227794	intron_variant,genic_downstream_transcript_variant
rs1795228051	intron_variant,genic_downstream_transcript_variant

rs1795228631	intron_variant,genic_downstream_transcript_variant
rs1795229230	intron_variant,genic_downstream_transcript_variant
rs1795229305	intron_variant,genic_downstream_transcript_variant
rs1795229471	intron_variant,genic_downstream_transcript_variant
rs1795230065	intron_variant,genic_downstream_transcript_variant
rs1795230391	intron_variant,genic_downstream_transcript_variant
rs1795230568	intron_variant,genic_downstream_transcript_variant
rs1795230647	intron_variant,genic_downstream_transcript_variant
rs1795230720	intron_variant,genic_downstream_transcript_variant
rs1795230880	intron_variant,genic_downstream_transcript_variant
rs1795231134	intron_variant,genic_downstream_transcript_variant
rs1795231439	intron_variant,genic_downstream_transcript_variant
rs1795231912	intron_variant,genic_downstream_transcript_variant
rs1795232590	intron_variant,genic_downstream_transcript_variant
rs1795232679	intron_variant,genic_downstream_transcript_variant
rs1795232755	intron_variant,genic_downstream_transcript_variant
rs1795233979	intron_variant,genic_downstream_transcript_variant
rs1795234423	intron_variant,genic_downstream_transcript_variant
rs1795234679	intron_variant,genic_downstream_transcript_variant
rs1795235240	intron_variant,genic_downstream_transcript_variant
rs1795235556	intron_variant,genic_downstream_transcript_variant
rs1795235629	intron_variant,genic_downstream_transcript_variant
rs1795236093	intron_variant,genic_downstream_transcript_variant
rs1795236591	intron_variant,genic_downstream_transcript_variant
rs1795236807	intron_variant,genic_downstream_transcript_variant
rs1795242027	intron_variant,genic_downstream_transcript_variant
rs1795242132	intron_variant,genic_downstream_transcript_variant
rs1795242258	intron_variant,genic_downstream_transcript_variant
rs1795242375	intron_variant,genic_downstream_transcript_variant
rs1795242482	intron_variant,genic_downstream_transcript_variant
rs1795242710	intron_variant,genic_downstream_transcript_variant
rs1795242828	intron_variant,genic_downstream_transcript_variant
rs1795243389	intron_variant,genic_downstream_transcript_variant
rs1795244082	intron_variant,genic_downstream_transcript_variant
rs1795244185	intron_variant,genic_downstream_transcript_variant
rs1795244412	intron_variant,genic_downstream_transcript_variant
rs1795245072	intron_variant,genic_downstream_transcript_variant
rs1795245167	intron_variant,genic_downstream_transcript_variant
rs1795245583	intron_variant,genic_downstream_transcript_variant
rs1795245669	intron_variant,genic_downstream_transcript_variant
rs1795246264	intron_variant,genic_downstream_transcript_variant
rs1795246335	intron_variant,genic_downstream_transcript_variant
rs1795246584	intron_variant,genic_downstream_transcript_variant
rs1795247012	intron_variant,genic_downstream_transcript_variant
rs1795247726	intron_variant,genic_downstream_transcript_variant
rs1795248405	intron_variant,genic_downstream_transcript_variant
rs1795248544	intron_variant,genic_downstream_transcript_variant
rs1795249208	intron_variant,genic_downstream_transcript_variant
rs1795249507	intron_variant,genic_downstream_transcript_variant
rs1795249791	intron_variant,genic_downstream_transcript_variant
rs1795250247	intron_variant,genic_downstream_transcript_variant
rs1795250365	intron_variant,genic_downstream_transcript_variant
rs1795250854	intron_variant,genic_downstream_transcript_variant
rs1795251350	intron_variant,genic_downstream_transcript_variant
rs1795252194	intron_variant,genic_downstream_transcript_variant
rs1795252453	intron_variant,genic_downstream_transcript_variant
rs1795252531	intron_variant,genic_downstream_transcript_variant
rs1795252851	intron_variant,genic_downstream_transcript_variant
rs1795253323	intron_variant,genic_downstream_transcript_variant
rs1795253653	intron_variant,genic_downstream_transcript_variant
rs1795253874	intron_variant,genic_downstream_transcript_variant
rs1795254059	intron_variant,genic_downstream_transcript_variant
rs1795254330	intron_variant,genic_downstream_transcript_variant
rs1795254477	intron_variant,genic_downstream_transcript_variant
rs1795254797	intron_variant,genic_downstream_transcript_variant
rs1795254960	intron_variant,genic_downstream_transcript_variant
rs1795255233	intron_variant,genic_downstream_transcript_variant
rs1795255308	intron_variant,genic_downstream_transcript_variant
rs1795256001	intron_variant,genic_downstream_transcript_variant
rs1795256079	intron_variant,genic_downstream_transcript_variant
rs1795256252	intron_variant,genic_downstream_transcript_variant
rs1795256432	intron_variant,genic_downstream_transcript_variant
rs1795256524	intron_variant,genic_downstream_transcript_variant
rs1795256730	intron_variant,genic_downstream_transcript_variant

rs1795256898	intron_variant,genic_downstream_transcript_variant
rs1795257079	intron_variant,genic_downstream_transcript_variant
rs1795257384	intron_variant,genic_downstream_transcript_variant
rs1795257458	intron_variant,genic_downstream_transcript_variant
rs1795258070	intron_variant,genic_downstream_transcript_variant
rs1795258168	intron_variant,genic_downstream_transcript_variant
rs1795258413	intron_variant,genic_downstream_transcript_variant
rs1795258642	intron_variant,genic_downstream_transcript_variant
rs1795259643	intron_variant,genic_downstream_transcript_variant
rs1795259809	intron_variant,genic_downstream_transcript_variant
rs1795260192	intron_variant,genic_downstream_transcript_variant
rs1795260273	intron_variant,genic_downstream_transcript_variant
rs1795260417	intron_variant,genic_downstream_transcript_variant
rs1795260582	intron_variant,genic_downstream_transcript_variant
rs1795260921	intron_variant,genic_downstream_transcript_variant
rs1795261436	intron_variant,genic_downstream_transcript_variant
rs1795261870	intron_variant,genic_downstream_transcript_variant
rs1795262181	intron_variant,genic_downstream_transcript_variant
rs1795262349	intron_variant,genic_downstream_transcript_variant
rs1795262468	intron_variant,genic_downstream_transcript_variant
rs1795262831	intron_variant,genic_downstream_transcript_variant
rs1795263646	intron_variant,genic_downstream_transcript_variant
rs1795263866	intron_variant,genic_downstream_transcript_variant
rs1795264260	intron_variant,genic_downstream_transcript_variant
rs1795264500	intron_variant,genic_downstream_transcript_variant
rs1795265145	intron_variant,genic_downstream_transcript_variant
rs1795265236	intron_variant,genic_downstream_transcript_variant
rs1795265586	intron_variant,genic_downstream_transcript_variant
rs1795266035	intron_variant,genic_downstream_transcript_variant
rs1795266507	intron_variant,genic_downstream_transcript_variant
rs1795266773	intron_variant,genic_downstream_transcript_variant
rs1795266851	intron_variant,genic_downstream_transcript_variant
rs1795267007	intron_variant,genic_downstream_transcript_variant
rs1795267174	intron_variant,genic_downstream_transcript_variant
rs1795267509	intron_variant,genic_downstream_transcript_variant
rs1795267682	intron_variant,genic_downstream_transcript_variant
rs1795267850	intron_variant,genic_downstream_transcript_variant
rs1795267933	intron_variant,genic_downstream_transcript_variant
rs1795268132	intron_variant,genic_downstream_transcript_variant
rs1795268211	intron_variant,genic_downstream_transcript_variant
rs1795268285	intron_variant,genic_downstream_transcript_variant
rs1795268369	intron_variant,genic_downstream_transcript_variant
rs1795268459	intron_variant,genic_downstream_transcript_variant
rs1795268606	intron_variant,genic_downstream_transcript_variant
rs1795268683	intron_variant,genic_downstream_transcript_variant
rs1795268825	intron_variant,genic_downstream_transcript_variant
rs1795268910	intron_variant,genic_downstream_transcript_variant
rs1795269575	intron_variant,genic_downstream_transcript_variant
rs1795269648	intron_variant,genic_downstream_transcript_variant
rs1795270911	intron_variant,genic_downstream_transcript_variant
rs1795271112	intron_variant,genic_downstream_transcript_variant
rs1795271204	intron_variant,genic_downstream_transcript_variant
rs1795272492	intron_variant,genic_downstream_transcript_variant
rs1795272969	intron_variant,genic_downstream_transcript_variant
rs1795273373	intron_variant,genic_downstream_transcript_variant
rs1795273624	intron_variant,genic_downstream_transcript_variant
rs1795273910	intron_variant,genic_downstream_transcript_variant
rs1795274637	intron_variant,genic_downstream_transcript_variant
rs1795274879	intron_variant,genic_downstream_transcript_variant
rs1795275158	intron_variant,genic_downstream_transcript_variant
rs1795275375	intron_variant,genic_downstream_transcript_variant
rs1795275643	intron_variant,genic_downstream_transcript_variant
rs1795275768	intron_variant,genic_downstream_transcript_variant
rs1795275911	intron_variant,genic_downstream_transcript_variant
rs1795276316	intron_variant,genic_downstream_transcript_variant
rs1795277799	intron_variant,genic_downstream_transcript_variant
rs1795278002	intron_variant,genic_downstream_transcript_variant
rs1795278070	intron_variant,genic_downstream_transcript_variant
rs1795278423	intron_variant,genic_downstream_transcript_variant
rs1795278561	intron_variant,genic_downstream_transcript_variant
rs1795279016	intron_variant,genic_downstream_transcript_variant
rs1795279306	intron_variant,genic_downstream_transcript_variant
rs1795279378	intron_variant,genic_downstream_transcript_variant
rs1795279544	intron_variant,genic_downstream_transcript_variant

rs1795279700	intron_variant,genic_downstream_transcript_variant
rs1795279775	intron_variant,genic_downstream_transcript_variant
rs1795279867	intron_variant,genic_downstream_transcript_variant
rs1795279953	intron_variant,genic_downstream_transcript_variant
rs1795280025	intron_variant,genic_downstream_transcript_variant
rs1795280257	intron_variant,genic_downstream_transcript_variant
rs1795280398	intron_variant,genic_downstream_transcript_variant
rs1795280461	intron_variant,genic_downstream_transcript_variant
rs1795280597	intron_variant,genic_downstream_transcript_variant
rs1795280781	intron_variant,genic_downstream_transcript_variant
rs1795280855	intron_variant,genic_downstream_transcript_variant
rs1795281006	intron_variant,genic_downstream_transcript_variant
rs1795281259	intron_variant,genic_downstream_transcript_variant
rs1795281406	intron_variant,genic_downstream_transcript_variant
rs1795281650	intron_variant,genic_downstream_transcript_variant
rs1795282550	intron_variant,genic_downstream_transcript_variant
rs1795282982	intron_variant,genic_downstream_transcript_variant
rs1795283071	intron_variant,genic_downstream_transcript_variant
rs1795283571	intron_variant,genic_downstream_transcript_variant
rs1795283723	intron_variant,genic_downstream_transcript_variant
rs1795284301	intron_variant,genic_downstream_transcript_variant
rs1795284627	intron_variant,genic_downstream_transcript_variant
rs1795284700	intron_variant,genic_downstream_transcript_variant
rs1795284769	intron_variant,genic_downstream_transcript_variant
rs1795284917	intron_variant,genic_downstream_transcript_variant
rs1795285593	intron_variant,genic_downstream_transcript_variant
rs1795285803	intron_variant,genic_downstream_transcript_variant
rs1795286047	intron_variant,genic_downstream_transcript_variant
rs1795286322	intron_variant,genic_downstream_transcript_variant
rs1795286428	intron_variant,genic_downstream_transcript_variant
rs1795286644	intron_variant,genic_downstream_transcript_variant
rs1795286894	intron_variant,genic_downstream_transcript_variant
rs1795287020	intron_variant,genic_downstream_transcript_variant
rs1795287147	intron_variant,genic_downstream_transcript_variant
rs1795287702	intron_variant,genic_downstream_transcript_variant
rs1795289087	intron_variant,genic_downstream_transcript_variant
rs1795289198	intron_variant,genic_downstream_transcript_variant
rs1795289285	intron_variant,genic_downstream_transcript_variant
rs1795289657	intron_variant,genic_downstream_transcript_variant
rs1795290022	intron_variant,genic_downstream_transcript_variant
rs1795290457	intron_variant,genic_downstream_transcript_variant
rs1795290747	intron_variant,genic_downstream_transcript_variant
rs1795290834	intron_variant,genic_downstream_transcript_variant
rs1795290922	intron_variant,genic_downstream_transcript_variant
rs1795291444	intron_variant,genic_downstream_transcript_variant
rs1795291542	intron_variant,genic_downstream_transcript_variant
rs1795291723	intron_variant,genic_downstream_transcript_variant
rs1795291822	intron_variant,genic_downstream_transcript_variant
rs1795291903	intron_variant,genic_downstream_transcript_variant
rs1795292005	intron_variant,genic_downstream_transcript_variant
rs1795292133	intron_variant,genic_downstream_transcript_variant
rs1795292249	intron_variant,genic_downstream_transcript_variant
rs1795292454	intron_variant,genic_downstream_transcript_variant
rs1795292533	intron_variant,genic_downstream_transcript_variant
rs1795292619	intron_variant,genic_downstream_transcript_variant
rs1795292708	intron_variant,genic_downstream_transcript_variant
rs1795293005	intron_variant,genic_downstream_transcript_variant
rs1795293197	intron_variant,genic_downstream_transcript_variant
rs1795293285	intron_variant,genic_downstream_transcript_variant
rs1795293449	intron_variant,genic_downstream_transcript_variant
rs1795294326	intron_variant,genic_downstream_transcript_variant
rs1795294522	intron_variant,genic_downstream_transcript_variant
rs1795295758	intron_variant,genic_downstream_transcript_variant
rs1795295838	intron_variant,genic_downstream_transcript_variant
rs1795295919	intron_variant,genic_downstream_transcript_variant
rs1795296367	intron_variant,genic_downstream_transcript_variant
rs1795296456	intron_variant,genic_downstream_transcript_variant
rs1795296681	intron_variant,genic_downstream_transcript_variant
rs1795297351	intron_variant,genic_downstream_transcript_variant
rs1795297684	intron_variant,genic_downstream_transcript_variant
rs1795297834	intron_variant,genic_downstream_transcript_variant
rs1795298981	intron_variant,genic_downstream_transcript_variant
rs1795299109	intron_variant,genic_downstream_transcript_variant
rs1795299234	intron_variant,genic_downstream_transcript_variant

rs1795299382	intron_variant,genic_downstream_transcript_variant
rs1795299838	intron_variant,genic_downstream_transcript_variant
rs1795299975	intron_variant,genic_downstream_transcript_variant
rs1795300678	intron_variant,genic_downstream_transcript_variant
rs1795301088	intron_variant,genic_downstream_transcript_variant
rs1795301366	intron_variant,genic_downstream_transcript_variant
rs1795301506	intron_variant,genic_downstream_transcript_variant
rs1795301640	intron_variant,genic_downstream_transcript_variant
rs1795301858	intron_variant,genic_downstream_transcript_variant
rs1795302725	intron_variant,genic_downstream_transcript_variant
rs1795303078	intron_variant,genic_downstream_transcript_variant
rs1795303180	intron_variant,genic_downstream_transcript_variant
rs1795303390	intron_variant,genic_downstream_transcript_variant
rs1795303980	intron_variant,genic_downstream_transcript_variant
rs1795304440	intron_variant,genic_downstream_transcript_variant
rs1795304529	intron_variant,genic_downstream_transcript_variant
rs1795304625	intron_variant,genic_downstream_transcript_variant
rs1795304714	intron_variant,genic_downstream_transcript_variant
rs1795305234	intron_variant,genic_downstream_transcript_variant
rs1795305319	intron_variant,genic_downstream_transcript_variant
rs1795305823	intron_variant,genic_downstream_transcript_variant
rs1795305900	intron_variant,genic_downstream_transcript_variant
rs1795305970	intron_variant,genic_downstream_transcript_variant
rs1795306098	intron_variant,genic_downstream_transcript_variant
rs1795307283	intron_variant,genic_downstream_transcript_variant
rs1795308214	intron_variant,genic_downstream_transcript_variant
rs1795308640	intron_variant,genic_downstream_transcript_variant
rs1795309232	intron_variant,genic_downstream_transcript_variant
rs1795309666	intron_variant,genic_downstream_transcript_variant
rs1795309807	intron_variant,genic_downstream_transcript_variant
rs1795309953	intron_variant,genic_downstream_transcript_variant
rs1795310190	intron_variant,genic_downstream_transcript_variant
rs1795311054	intron_variant,genic_downstream_transcript_variant
rs1795312642	intron_variant,genic_downstream_transcript_variant
rs1795312790	intron_variant,genic_downstream_transcript_variant
rs1795313220	intron_variant,genic_downstream_transcript_variant
rs1795313356	intron_variant,genic_downstream_transcript_variant
rs1795313464	intron_variant,genic_downstream_transcript_variant
rs1795313578	intron_variant,genic_downstream_transcript_variant
rs1795314784	intron_variant,genic_downstream_transcript_variant
rs1795314884	intron_variant,genic_downstream_transcript_variant
rs1795315477	intron_variant,genic_downstream_transcript_variant
rs1795315691	intron_variant,genic_downstream_transcript_variant
rs1795315956	intron_variant,genic_downstream_transcript_variant
rs1795316131	intron_variant,genic_downstream_transcript_variant
rs1795316328	intron_variant,genic_downstream_transcript_variant
rs1795316662	intron_variant,genic_downstream_transcript_variant
rs1795317118	intron_variant,genic_downstream_transcript_variant
rs1795317220	intron_variant,genic_downstream_transcript_variant
rs1795317286	intron_variant,genic_downstream_transcript_variant
rs1795317367	intron_variant,genic_downstream_transcript_variant
rs1795317447	intron_variant,genic_downstream_transcript_variant
rs1795317519	intron_variant,genic_downstream_transcript_variant
rs1795317588	intron_variant,genic_downstream_transcript_variant
rs1795318197	intron_variant,genic_downstream_transcript_variant
rs1795318460	intron_variant,genic_downstream_transcript_variant
rs1795318746	intron_variant,genic_downstream_transcript_variant
rs1795319127	intron_variant,genic_downstream_transcript_variant
rs1795319354	intron_variant,genic_downstream_transcript_variant
rs1795319610	intron_variant,genic_downstream_transcript_variant
rs1795319925	intron_variant,genic_downstream_transcript_variant
rs1795320081	intron_variant,genic_downstream_transcript_variant
rs1795320165	intron_variant,genic_downstream_transcript_variant
rs1795320401	intron_variant,genic_downstream_transcript_variant
rs1795320737	intron_variant,genic_downstream_transcript_variant
rs1795320821	intron_variant,genic_downstream_transcript_variant
rs1795320932	intron_variant,genic_downstream_transcript_variant
rs1795321049	intron_variant,genic_downstream_transcript_variant
rs1795321156	intron_variant,genic_downstream_transcript_variant
rs1795321269	intron_variant,genic_downstream_transcript_variant
rs1795321633	intron_variant,genic_downstream_transcript_variant
rs1795321811	intron_variant,genic_downstream_transcript_variant
rs1795321878	intron_variant,genic_downstream_transcript_variant
rs1795322614	intron_variant,genic_downstream_transcript_variant

rs1795323137	intron_variant,genic_downstream_transcript_variant
rs1795323401	intron_variant,genic_downstream_transcript_variant
rs1795325520	intron_variant,genic_downstream_transcript_variant
rs1795325752	intron_variant,genic_downstream_transcript_variant
rs1795326012	intron_variant,genic_downstream_transcript_variant
rs1795326376	intron_variant,genic_downstream_transcript_variant
rs1795326931	intron_variant,genic_downstream_transcript_variant
rs1795327048	intron_variant,genic_downstream_transcript_variant
rs1795327337	intron_variant,genic_downstream_transcript_variant
rs1795327474	intron_variant,genic_downstream_transcript_variant
rs1795327839	intron_variant,genic_downstream_transcript_variant
rs1795327962	intron_variant,genic_downstream_transcript_variant
rs1795328801	intron_variant,genic_downstream_transcript_variant
rs1795329234	intron_variant,genic_downstream_transcript_variant
rs1795329510	intron_variant,genic_downstream_transcript_variant
rs1795329587	intron_variant,genic_downstream_transcript_variant
rs1795329664	intron_variant,genic_downstream_transcript_variant
rs1795330115	intron_variant,genic_downstream_transcript_variant
rs1795330403	intron_variant,genic_downstream_transcript_variant
rs1795330517	intron_variant,genic_downstream_transcript_variant
rs1795330634	intron_variant,genic_downstream_transcript_variant
rs1795331193	intron_variant,genic_downstream_transcript_variant
rs1795331352	intron_variant,genic_downstream_transcript_variant
rs1795331647	intron_variant,genic_downstream_transcript_variant
rs1795331780	intron_variant,genic_downstream_transcript_variant
rs1795332201	intron_variant,genic_downstream_transcript_variant
rs1795332586	intron_variant,genic_downstream_transcript_variant
rs1795332880	intron_variant,genic_downstream_transcript_variant
rs1795333204	intron_variant,genic_downstream_transcript_variant
rs1795333274	intron_variant,genic_downstream_transcript_variant
rs1795333361	intron_variant,genic_downstream_transcript_variant
rs1795333456	intron_variant,genic_downstream_transcript_variant
rs1795333979	intron_variant,genic_downstream_transcript_variant
rs1795334150	intron_variant,genic_downstream_transcript_variant
rs1795334911	intron_variant,genic_downstream_transcript_variant
rs1795335149	intron_variant,genic_downstream_transcript_variant
rs1795335423	intron_variant,genic_downstream_transcript_variant
rs1795335715	intron_variant,genic_downstream_transcript_variant
rs1795335789	intron_variant,genic_downstream_transcript_variant
rs1795335873	intron_variant,genic_downstream_transcript_variant
rs1795336553	intron_variant,genic_downstream_transcript_variant
rs1795337016	intron_variant,genic_downstream_transcript_variant
rs1795337184	intron_variant,genic_downstream_transcript_variant
rs1795337287	intron_variant,genic_downstream_transcript_variant
rs1795337377	intron_variant,genic_downstream_transcript_variant
rs1795337631	intron_variant,genic_downstream_transcript_variant
rs1795337930	intron_variant,genic_downstream_transcript_variant
rs1795338182	intron_variant,genic_downstream_transcript_variant
rs1795338257	intron_variant,genic_downstream_transcript_variant
rs1795338575	intron_variant,genic_downstream_transcript_variant
rs1795338751	intron_variant,genic_downstream_transcript_variant
rs1795339198	intron_variant,genic_downstream_transcript_variant
rs1795339784	intron_variant,genic_downstream_transcript_variant
rs1795340085	intron_variant,genic_downstream_transcript_variant
rs1795340175	intron_variant,genic_downstream_transcript_variant
rs1795340329	intron_variant,genic_downstream_transcript_variant
rs1795340413	intron_variant,genic_downstream_transcript_variant
rs1795340502	intron_variant,genic_downstream_transcript_variant
rs1795340708	intron_variant,genic_downstream_transcript_variant
rs1795341339	intron_variant,genic_downstream_transcript_variant
rs1795341961	intron_variant,genic_downstream_transcript_variant
rs1795342022	intron_variant,genic_downstream_transcript_variant
rs1795342373	intron_variant,genic_downstream_transcript_variant
rs1795342686	intron_variant,genic_downstream_transcript_variant
rs1795342803	intron_variant,genic_downstream_transcript_variant
rs1795343460	intron_variant,genic_downstream_transcript_variant
rs1795344201	intron_variant,genic_downstream_transcript_variant
rs1795344575	intron_variant,genic_downstream_transcript_variant
rs1795344863	intron_variant,genic_downstream_transcript_variant
rs1795345205	intron_variant,genic_downstream_transcript_variant
rs1795345347	intron_variant,genic_downstream_transcript_variant
rs1795345584	intron_variant,genic_downstream_transcript_variant
rs1795345709	intron_variant,genic_downstream_transcript_variant
rs1795345826	intron_variant,genic_downstream_transcript_variant

rs1795346066	intron_variant,genic_downstream_transcript_variant
rs1795346700	intron_variant,genic_downstream_transcript_variant
rs1795346916	intron_variant,genic_downstream_transcript_variant
rs1795347011	intron_variant,genic_downstream_transcript_variant
rs1795347424	intron_variant,genic_downstream_transcript_variant
rs1795347647	intron_variant,genic_downstream_transcript_variant
rs1795347759	intron_variant,genic_downstream_transcript_variant
rs1795347888	intron_variant,genic_downstream_transcript_variant
rs1795348036	intron_variant,genic_downstream_transcript_variant
rs1795348328	intron_variant,genic_downstream_transcript_variant
rs1795349327	intron_variant,genic_downstream_transcript_variant
rs1795350411	intron_variant,genic_downstream_transcript_variant
rs1795350528	intron_variant,genic_downstream_transcript_variant
rs1795350728	intron_variant,genic_downstream_transcript_variant
rs1795350870	intron_variant,genic_downstream_transcript_variant
rs1795351345	intron_variant,genic_downstream_transcript_variant
rs1795351463	intron_variant,genic_downstream_transcript_variant
rs1795351753	intron_variant,genic_downstream_transcript_variant
rs1795352134	intron_variant,genic_downstream_transcript_variant
rs1795352251	intron_variant,genic_downstream_transcript_variant
rs1795352754	intron_variant,genic_downstream_transcript_variant
rs1795352988	intron_variant,genic_downstream_transcript_variant
rs1795353643	intron_variant,genic_downstream_transcript_variant
rs1795353899	intron_variant,genic_downstream_transcript_variant
rs1795354010	intron_variant,genic_downstream_transcript_variant
rs1795354129	intron_variant,genic_downstream_transcript_variant
rs1795354257	intron_variant,genic_downstream_transcript_variant
rs1795354319	intron_variant,genic_downstream_transcript_variant
rs1795354614	intron_variant,genic_downstream_transcript_variant
rs1795355302	intron_variant,genic_downstream_transcript_variant
rs1795355436	intron_variant,genic_downstream_transcript_variant
rs1795355555	intron_variant,genic_downstream_transcript_variant
rs1795355899	intron_variant,genic_downstream_transcript_variant
rs1795355992	intron_variant,genic_downstream_transcript_variant
rs1795356352	intron_variant,genic_downstream_transcript_variant
rs1795357103	intron_variant,genic_downstream_transcript_variant
rs1795357270	intron_variant,genic_downstream_transcript_variant
rs1795357377	intron_variant,genic_downstream_transcript_variant
rs1795357494	intron_variant,genic_downstream_transcript_variant
rs1795358297	intron_variant,genic_downstream_transcript_variant
rs1795358378	intron_variant,genic_downstream_transcript_variant
rs1795358503	intron_variant,genic_downstream_transcript_variant
rs1795358669	intron_variant,genic_downstream_transcript_variant
rs1795358957	intron_variant,genic_downstream_transcript_variant
rs1795359197	intron_variant,genic_downstream_transcript_variant
rs1795359453	intron_variant,genic_downstream_transcript_variant
rs1795359986	intron_variant,genic_downstream_transcript_variant
rs1795360348	intron_variant,genic_downstream_transcript_variant
rs1795360740	intron_variant,genic_downstream_transcript_variant
rs1795360825	intron_variant,genic_downstream_transcript_variant
rs1795361546	intron_variant,genic_downstream_transcript_variant
rs1795361701	intron_variant,genic_downstream_transcript_variant
rs1795361944	intron_variant,genic_downstream_transcript_variant
rs1795362047	intron_variant,genic_downstream_transcript_variant
rs1795362394	intron_variant,genic_downstream_transcript_variant
rs1795362519	intron_variant,genic_downstream_transcript_variant
rs1795362811	intron_variant,genic_downstream_transcript_variant
rs1795363071	intron_variant,genic_downstream_transcript_variant
rs1795363187	intron_variant,genic_downstream_transcript_variant
rs1795363330	intron_variant,genic_downstream_transcript_variant
rs1795363454	intron_variant,genic_downstream_transcript_variant
rs1795363582	intron_variant,genic_downstream_transcript_variant
rs1795363826	intron_variant,genic_downstream_transcript_variant
rs1795364595	intron_variant,genic_downstream_transcript_variant
rs1795364689	intron_variant,genic_downstream_transcript_variant
rs1795365015	intron_variant,genic_downstream_transcript_variant
rs1795365389	intron_variant,genic_downstream_transcript_variant
rs1795365481	intron_variant,genic_downstream_transcript_variant
rs1795365808	intron_variant,genic_downstream_transcript_variant
rs1795365892	intron_variant,genic_downstream_transcript_variant
rs1795366193	intron_variant,genic_downstream_transcript_variant
rs1795366591	intron_variant,genic_downstream_transcript_variant
rs1795366971	intron_variant,genic_downstream_transcript_variant
rs1795367159	intron_variant,genic_downstream_transcript_variant

rs1795367262	intron_variant,genic_downstream_transcript_variant
rs1795367791	intron_variant,genic_downstream_transcript_variant
rs1795367875	intron_variant,genic_downstream_transcript_variant
rs1795368139	intron_variant,genic_downstream_transcript_variant
rs1795368258	intron_variant,genic_downstream_transcript_variant
rs1795368449	intron_variant,genic_downstream_transcript_variant
rs1795368821	intron_variant,genic_downstream_transcript_variant
rs1795369104	intron_variant,genic_downstream_transcript_variant
rs1795369363	intron_variant,genic_downstream_transcript_variant
rs1795369440	intron_variant,genic_downstream_transcript_variant
rs1795369524	intron_variant,genic_downstream_transcript_variant
rs1795369691	intron_variant,genic_downstream_transcript_variant
rs1795369850	intron_variant,genic_downstream_transcript_variant
rs1795370120	intron_variant,genic_downstream_transcript_variant
rs1795370185	intron_variant,genic_downstream_transcript_variant
rs1795370254	intron_variant,genic_downstream_transcript_variant
rs1795370327	intron_variant,genic_downstream_transcript_variant
rs1795370710	intron_variant,genic_downstream_transcript_variant
rs1795371284	intron_variant,genic_downstream_transcript_variant
rs1795371571	intron_variant,genic_downstream_transcript_variant
rs1795371706	intron_variant,genic_downstream_transcript_variant
rs1795371956	intron_variant,genic_downstream_transcript_variant
rs1795372198	intron_variant,genic_downstream_transcript_variant
rs1795372286	intron_variant,genic_downstream_transcript_variant
rs1795372443	intron_variant,genic_downstream_transcript_variant
rs1795372523	intron_variant,genic_downstream_transcript_variant
rs1795372599	intron_variant,genic_downstream_transcript_variant
rs1795372671	intron_variant,genic_downstream_transcript_variant
rs1795373187	intron_variant,genic_downstream_transcript_variant
rs1795373379	intron_variant,genic_downstream_transcript_variant
rs1795373956	intron_variant,genic_downstream_transcript_variant
rs1795374040	intron_variant,genic_downstream_transcript_variant
rs1795374474	intron_variant,genic_downstream_transcript_variant
rs1795374672	intron_variant,genic_downstream_transcript_variant
rs1795374749	intron_variant,genic_downstream_transcript_variant
rs1795374831	intron_variant,genic_downstream_transcript_variant
rs1795375190	intron_variant,genic_downstream_transcript_variant
rs1795375344	intron_variant,genic_downstream_transcript_variant
rs1795375579	intron_variant,genic_downstream_transcript_variant
rs1795375653	intron_variant,genic_downstream_transcript_variant
rs1795375736	intron_variant,genic_downstream_transcript_variant
rs1795375875	intron_variant,genic_downstream_transcript_variant
rs1795376036	intron_variant,genic_downstream_transcript_variant
rs1795376213	intron_variant,genic_downstream_transcript_variant
rs1795376297	intron_variant,genic_downstream_transcript_variant
rs1795376373	intron_variant,genic_downstream_transcript_variant
rs1795376751	intron_variant,genic_downstream_transcript_variant
rs1795376904	intron_variant,genic_downstream_transcript_variant
rs1795376993	intron_variant,genic_downstream_transcript_variant
rs1795377075	intron_variant,genic_downstream_transcript_variant
rs1795377166	intron_variant,genic_downstream_transcript_variant
rs1795377269	intron_variant,genic_downstream_transcript_variant
rs1795377374	intron_variant,genic_downstream_transcript_variant
rs1795377456	intron_variant,genic_downstream_transcript_variant
rs1795377537	intron_variant,genic_downstream_transcript_variant
rs1795377696	intron_variant,genic_downstream_transcript_variant
rs1795377801	intron_variant,genic_downstream_transcript_variant
rs1795377896	intron_variant,genic_downstream_transcript_variant
rs1795378032	intron_variant,genic_downstream_transcript_variant
rs1795378436	intron_variant,genic_downstream_transcript_variant
rs1795378672	intron_variant,genic_downstream_transcript_variant
rs1795378839	intron_variant,genic_downstream_transcript_variant
rs1795379184	intron_variant,genic_downstream_transcript_variant
rs1795379264	intron_variant,genic_downstream_transcript_variant
rs1795379630	intron_variant,genic_downstream_transcript_variant
rs1795379783	intron_variant,genic_downstream_transcript_variant
rs1795379867	intron_variant,genic_downstream_transcript_variant
rs1795380221	intron_variant,genic_downstream_transcript_variant
rs1795380393	intron_variant,genic_downstream_transcript_variant
rs1795380461	intron_variant,genic_downstream_transcript_variant
rs1795380603	intron_variant,genic_downstream_transcript_variant
rs1795380863	intron_variant,genic_downstream_transcript_variant
rs1795381021	intron_variant,genic_downstream_transcript_variant
rs1795381097	intron_variant,genic_downstream_transcript_variant

rs1795381181	intron_variant,genic_downstream_transcript_variant
rs1795381415	intron_variant,genic_downstream_transcript_variant
rs1795381505	intron_variant,genic_downstream_transcript_variant
rs1795381800	intron_variant,genic_downstream_transcript_variant
rs1795382042	intron_variant,genic_downstream_transcript_variant
rs1795382124	intron_variant,genic_downstream_transcript_variant
rs1795382208	intron_variant,genic_downstream_transcript_variant
rs1795382445	intron_variant,genic_downstream_transcript_variant
rs1795382660	intron_variant,genic_downstream_transcript_variant
rs1795382756	intron_variant,genic_downstream_transcript_variant
rs1795383503	intron_variant,genic_downstream_transcript_variant
rs1795383581	intron_variant,genic_downstream_transcript_variant
rs1795383726	intron_variant,genic_downstream_transcript_variant
rs1795383809	intron_variant,genic_downstream_transcript_variant
rs1795383890	intron_variant,genic_downstream_transcript_variant
rs1795384353	intron_variant,genic_downstream_transcript_variant
rs1795384506	intron_variant,genic_downstream_transcript_variant
rs1795385247	intron_variant,genic_downstream_transcript_variant
rs1795385329	intron_variant,genic_downstream_transcript_variant
rs1795386042	intron_variant,genic_downstream_transcript_variant
rs1795386211	intron_variant,genic_downstream_transcript_variant
rs1795387499	intron_variant,genic_downstream_transcript_variant
rs1795390324	intron_variant,genic_downstream_transcript_variant
rs1795390438	intron_variant,genic_downstream_transcript_variant
rs1795390813	intron_variant,genic_downstream_transcript_variant
rs1795390903	intron_variant,genic_downstream_transcript_variant
rs1795391450	intron_variant,genic_downstream_transcript_variant
rs1795391809	intron_variant,genic_downstream_transcript_variant
rs1795392086	intron_variant,genic_downstream_transcript_variant
rs1795393082	intron_variant,genic_downstream_transcript_variant
rs1795393431	intron_variant,genic_downstream_transcript_variant
rs1795394100	intron_variant,genic_downstream_transcript_variant
rs1795394223	intron_variant,genic_downstream_transcript_variant
rs1795395091	intron_variant,genic_downstream_transcript_variant
rs1795396184	intron_variant,genic_downstream_transcript_variant
rs1795398918	intron_variant,genic_downstream_transcript_variant
rs1795399113	intron_variant,genic_downstream_transcript_variant
rs1795399431	intron_variant,genic_downstream_transcript_variant
rs1795399695	intron_variant,genic_downstream_transcript_variant
rs1795400127	intron_variant,genic_downstream_transcript_variant
rs1795400215	intron_variant,genic_downstream_transcript_variant
rs1795400404	intron_variant,genic_downstream_transcript_variant
rs1795400810	intron_variant,genic_downstream_transcript_variant
rs1795401099	intron_variant,genic_downstream_transcript_variant
rs1795401352	intron_variant,genic_downstream_transcript_variant
rs1795401646	intron_variant,genic_downstream_transcript_variant
rs1795402004	intron_variant,genic_downstream_transcript_variant
rs1795402615	intron_variant,genic_downstream_transcript_variant
rs1795402801	intron_variant,genic_downstream_transcript_variant
rs1795402911	intron_variant,genic_downstream_transcript_variant
rs1795403160	intron_variant,genic_downstream_transcript_variant
rs1795403718	intron_variant,genic_downstream_transcript_variant
rs1795404103	intron_variant,genic_downstream_transcript_variant
rs1795404347	intron_variant,genic_downstream_transcript_variant
rs1795404837	intron_variant,genic_downstream_transcript_variant
rs1795405332	intron_variant,genic_downstream_transcript_variant
rs1795405697	intron_variant,genic_downstream_transcript_variant
rs1795406017	intron_variant,genic_downstream_transcript_variant
rs1795406260	intron_variant,genic_downstream_transcript_variant
rs1795406399	intron_variant,genic_downstream_transcript_variant
rs1795406485	intron_variant,genic_downstream_transcript_variant
rs1795406796	intron_variant,genic_downstream_transcript_variant
rs1795406975	intron_variant,genic_downstream_transcript_variant
rs1795407150	intron_variant,genic_downstream_transcript_variant
rs1795407345	intron_variant,genic_downstream_transcript_variant
rs1795407595	intron_variant,genic_downstream_transcript_variant
rs1795407673	intron_variant,genic_downstream_transcript_variant
rs1795407819	intron_variant,genic_downstream_transcript_variant
rs1795408301	intron_variant,genic_downstream_transcript_variant
rs1795408685	intron_variant,genic_downstream_transcript_variant
rs1795409000	intron_variant,genic_downstream_transcript_variant
rs1795409089	intron_variant,genic_downstream_transcript_variant
rs1795409267	intron_variant,genic_downstream_transcript_variant
rs1795409728	intron_variant,genic_downstream_transcript_variant

rs1795410056	intron_variant,genic_downstream_transcript_variant
rs1795410255	intron_variant,genic_downstream_transcript_variant
rs1795410445	intron_variant,genic_downstream_transcript_variant
rs1795410914	intron_variant,genic_downstream_transcript_variant
rs1795412007	intron_variant,genic_downstream_transcript_variant
rs1795412181	intron_variant,genic_downstream_transcript_variant
rs1795412483	intron_variant,genic_downstream_transcript_variant
rs1795412742	intron_variant,genic_downstream_transcript_variant
rs1795412926	intron_variant,genic_downstream_transcript_variant
rs1795413910	intron_variant,genic_downstream_transcript_variant
rs1795413997	intron_variant,genic_downstream_transcript_variant
rs1795414087	intron_variant,genic_downstream_transcript_variant
rs1795414200	intron_variant,genic_downstream_transcript_variant
rs1795414751	intron_variant,genic_downstream_transcript_variant
rs1795414831	intron_variant,genic_downstream_transcript_variant
rs1795415221	intron_variant,genic_downstream_transcript_variant
rs1795415311	intron_variant,genic_downstream_transcript_variant
rs1795415399	intron_variant,genic_downstream_transcript_variant
rs1795416007	intron_variant,genic_downstream_transcript_variant
rs1795416547	intron_variant,genic_downstream_transcript_variant
rs1795416898	intron_variant,genic_downstream_transcript_variant
rs1795417133	intron_variant,genic_downstream_transcript_variant
rs1795418262	intron_variant,genic_downstream_transcript_variant
rs1795418548	intron_variant,genic_downstream_transcript_variant
rs1795418709	intron_variant,genic_downstream_transcript_variant
rs1795418789	intron_variant,genic_downstream_transcript_variant
rs1795419692	intron_variant,genic_downstream_transcript_variant
rs1795420414	intron_variant,genic_downstream_transcript_variant
rs1795420741	intron_variant,genic_downstream_transcript_variant
rs1795421243	intron_variant,genic_downstream_transcript_variant
rs1795421330	intron_variant,genic_downstream_transcript_variant
rs1795421435	intron_variant,genic_downstream_transcript_variant
rs1795421620	intron_variant,genic_downstream_transcript_variant
rs1795421948	intron_variant,genic_downstream_transcript_variant
rs1795422107	intron_variant,genic_downstream_transcript_variant
rs1795422359	intron_variant,genic_downstream_transcript_variant
rs1795422443	intron_variant,genic_downstream_transcript_variant
rs1795422643	intron_variant,genic_downstream_transcript_variant
rs1795422954	intron_variant,genic_downstream_transcript_variant
rs1795423036	intron_variant,genic_downstream_transcript_variant
rs1795423116	intron_variant,genic_downstream_transcript_variant
rs1795423484	intron_variant,genic_downstream_transcript_variant
rs1795423568	intron_variant,genic_downstream_transcript_variant
rs1795423654	intron_variant,genic_downstream_transcript_variant
rs1795424101	intron_variant,genic_downstream_transcript_variant
rs1795425027	intron_variant,genic_downstream_transcript_variant
rs1795425123	intron_variant,genic_downstream_transcript_variant
rs1795425207	intron_variant,genic_downstream_transcript_variant
rs1795425794	intron_variant,genic_downstream_transcript_variant
rs1795426194	intron_variant,genic_downstream_transcript_variant
rs1795426395	intron_variant,genic_downstream_transcript_variant
rs1795426794	intron_variant,genic_downstream_transcript_variant
rs1795426895	intron_variant,genic_downstream_transcript_variant
rs1795426966	intron_variant,genic_downstream_transcript_variant
rs1795427118	intron_variant,genic_downstream_transcript_variant
rs1795427421	intron_variant,genic_downstream_transcript_variant
rs1795428556	intron_variant,genic_downstream_transcript_variant
rs1795429105	intron_variant,genic_downstream_transcript_variant
rs1795429190	intron_variant,genic_downstream_transcript_variant
rs1795429451	intron_variant,genic_downstream_transcript_variant
rs1795429613	intron_variant,genic_downstream_transcript_variant
rs1795429716	intron_variant,genic_downstream_transcript_variant
rs1795429810	intron_variant,genic_downstream_transcript_variant
rs1795430316	intron_variant,genic_downstream_transcript_variant
rs1795430625	intron_variant,genic_downstream_transcript_variant
rs1795430829	intron_variant,genic_downstream_transcript_variant
rs1795430912	intron_variant,genic_downstream_transcript_variant
rs1795431007	intron_variant,genic_downstream_transcript_variant
rs1795431296	intron_variant,genic_downstream_transcript_variant
rs1795431754	intron_variant,genic_downstream_transcript_variant
rs1795431924	intron_variant,genic_downstream_transcript_variant
rs1795432159	intron_variant,genic_downstream_transcript_variant
rs1795432392	intron_variant,genic_downstream_transcript_variant
rs1795432768	intron_variant,genic_downstream_transcript_variant

rs1795432858	intron_variant,genic_downstream_transcript_variant
rs1795433411	intron_variant,genic_downstream_transcript_variant
rs1795433502	intron_variant,genic_downstream_transcript_variant
rs1795433736	intron_variant,genic_downstream_transcript_variant
rs1795433811	intron_variant,genic_downstream_transcript_variant
rs1795434055	intron_variant,genic_downstream_transcript_variant
rs1795434259	intron_variant,genic_downstream_transcript_variant
rs1795435262	intron_variant,genic_downstream_transcript_variant
rs1795435331	intron_variant,genic_downstream_transcript_variant
rs1795435720	intron_variant,genic_downstream_transcript_variant
rs1795435970	intron_variant,genic_downstream_transcript_variant
rs1795436071	intron_variant,genic_downstream_transcript_variant
rs1795436157	intron_variant,genic_downstream_transcript_variant
rs1795436233	intron_variant,genic_downstream_transcript_variant
rs1795436379	intron_variant,genic_downstream_transcript_variant
rs1795436682	intron_variant,genic_downstream_transcript_variant
rs1795436955	intron_variant,genic_downstream_transcript_variant
rs1795437042	intron_variant,genic_downstream_transcript_variant
rs1795437207	intron_variant,genic_downstream_transcript_variant
rs1795437355	intron_variant,genic_downstream_transcript_variant
rs1795437562	intron_variant,genic_downstream_transcript_variant
rs1795437657	intron_variant,genic_downstream_transcript_variant
rs1795437885	intron_variant,genic_downstream_transcript_variant
rs1795438043	intron_variant,genic_downstream_transcript_variant
rs1795438231	intron_variant,genic_downstream_transcript_variant
rs1795438338	intron_variant,genic_downstream_transcript_variant
rs1795438500	intron_variant,genic_downstream_transcript_variant
rs1795438688	intron_variant,genic_downstream_transcript_variant
rs1795438887	intron_variant,genic_downstream_transcript_variant
rs1795438981	intron_variant,genic_downstream_transcript_variant
rs1795439149	intron_variant,genic_downstream_transcript_variant
rs1795439449	intron_variant,genic_downstream_transcript_variant
rs1795439737	intron_variant,genic_downstream_transcript_variant
rs1795439858	intron_variant,genic_downstream_transcript_variant
rs1795440256	intron_variant,genic_downstream_transcript_variant
rs1795440427	intron_variant,genic_downstream_transcript_variant
rs1795440622	intron_variant,genic_downstream_transcript_variant
rs1795440882	intron_variant,genic_downstream_transcript_variant
rs1795441008	intron_variant,genic_downstream_transcript_variant
rs1795441152	intron_variant,genic_downstream_transcript_variant
rs1795441269	intron_variant,genic_downstream_transcript_variant
rs1795442169	intron_variant,genic_downstream_transcript_variant
rs1795442311	intron_variant,genic_downstream_transcript_variant
rs1795442612	intron_variant,genic_downstream_transcript_variant
rs1795442888	intron_variant,genic_downstream_transcript_variant
rs1795443330	intron_variant,genic_downstream_transcript_variant
rs1795443798	intron_variant,genic_downstream_transcript_variant
rs1795444083	intron_variant,genic_downstream_transcript_variant
rs1795444217	intron_variant,genic_downstream_transcript_variant
rs1795445504	intron_variant,genic_downstream_transcript_variant
rs1795445621	intron_variant,genic_downstream_transcript_variant
rs1795445746	intron_variant,genic_downstream_transcript_variant
rs1795445968	intron_variant,genic_downstream_transcript_variant
rs1795446105	intron_variant,genic_downstream_transcript_variant
rs1795447081	intron_variant,genic_downstream_transcript_variant
rs1795447386	intron_variant,genic_downstream_transcript_variant
rs1795447503	intron_variant,genic_downstream_transcript_variant
rs1795447737	intron_variant,genic_downstream_transcript_variant
rs1795447990	intron_variant,genic_downstream_transcript_variant
rs1795448531	intron_variant,genic_downstream_transcript_variant
rs1795449204	intron_variant,genic_downstream_transcript_variant
rs1795449666	intron_variant,genic_downstream_transcript_variant
rs1795449904	intron_variant,genic_downstream_transcript_variant
rs1795450045	intron_variant,genic_downstream_transcript_variant
rs1795450913	intron_variant,genic_downstream_transcript_variant
rs1795451151	intron_variant,genic_downstream_transcript_variant
rs1795451278	intron_variant,genic_downstream_transcript_variant
rs1795451808	intron_variant,genic_downstream_transcript_variant
rs1795452186	intron_variant,genic_downstream_transcript_variant
rs1795452284	intron_variant,genic_downstream_transcript_variant
rs1795452621	intron_variant,genic_downstream_transcript_variant
rs1795452739	intron_variant,genic_downstream_transcript_variant
rs1795453183	intron_variant,genic_downstream_transcript_variant
rs1795453584	intron_variant,genic_upstream_transcript_variant

rs1795453754	intron_variant,genic_downstream_transcript_variant
rs1795453867	intron_variant,genic_downstream_transcript_variant
rs1795453963	intron_variant,genic_downstream_transcript_variant
rs1795454455	intron_variant,genic_downstream_transcript_variant
rs1795455386	intron_variant,genic_downstream_transcript_variant
rs1795455740	intron_variant,genic_downstream_transcript_variant
rs1795456014	intron_variant,genic_downstream_transcript_variant
rs1795456144	intron_variant,genic_downstream_transcript_variant
rs1795456742	intron_variant,genic_downstream_transcript_variant
rs1795456881	intron_variant,genic_downstream_transcript_variant
rs1795456972	intron_variant,genic_downstream_transcript_variant
rs1795457569	intron_variant,genic_downstream_transcript_variant
rs1795457815	intron_variant,genic_downstream_transcript_variant
rs1795458759	intron_variant,genic_downstream_transcript_variant
rs1795459779	intron_variant,genic_downstream_transcript_variant
rs1795459924	intron_variant,genic_downstream_transcript_variant
rs1795460229	intron_variant,genic_downstream_transcript_variant
rs1795460526	intron_variant,genic_downstream_transcript_variant
rs1795461198	intron_variant,genic_downstream_transcript_variant
rs1795461325	intron_variant,genic_downstream_transcript_variant
rs1795462027	intron_variant,genic_downstream_transcript_variant
rs1795462171	intron_variant,genic_downstream_transcript_variant
rs1795462725	intron_variant,genic_downstream_transcript_variant
rs1795462879	intron_variant,genic_downstream_transcript_variant
rs1795463180	intron_variant,genic_downstream_transcript_variant
rs1795463316	intron_variant,genic_downstream_transcript_variant
rs1795463461	intron_variant,genic_downstream_transcript_variant
rs1795465375	intron_variant,genic_downstream_transcript_variant
rs1795465660	intron_variant,genic_downstream_transcript_variant
rs1795466432	intron_variant,genic_downstream_transcript_variant
rs1795466802	intron_variant,genic_downstream_transcript_variant
rs1795468445	intron_variant,genic_downstream_transcript_variant
rs1795469185	intron_variant,genic_downstream_transcript_variant
rs1795469336	intron_variant,genic_downstream_transcript_variant
rs1795470214	intron_variant,genic_downstream_transcript_variant
rs1795470751	intron_variant,genic_downstream_transcript_variant
rs1795470886	intron_variant,genic_downstream_transcript_variant
rs1795471261	intron_variant,genic_downstream_transcript_variant
rs1795471633	intron_variant,genic_downstream_transcript_variant
rs1795472085	intron_variant,genic_downstream_transcript_variant
rs1795472494	intron_variant,genic_downstream_transcript_variant
rs1795472761	intron_variant,genic_downstream_transcript_variant
rs1795473026	intron_variant,genic_downstream_transcript_variant
rs1795473112	intron_variant,genic_downstream_transcript_variant
rs1795473179	intron_variant,genic_downstream_transcript_variant
rs1795473895	intron_variant,genic_downstream_transcript_variant
rs1795474321	intron_variant,genic_downstream_transcript_variant
rs1795474408	intron_variant,genic_downstream_transcript_variant
rs1795474523	intron_variant,genic_downstream_transcript_variant
rs1795475100	intron_variant,genic_downstream_transcript_variant
rs1795475166	intron_variant,genic_downstream_transcript_variant
rs1795475759	intron_variant,genic_downstream_transcript_variant
rs1795476416	intron_variant,genic_downstream_transcript_variant
rs1795476757	intron_variant,genic_downstream_transcript_variant
rs1795477057	intron_variant,genic_downstream_transcript_variant
rs1795477129	intron_variant,genic_downstream_transcript_variant
rs1795477415	intron_variant,genic_downstream_transcript_variant
rs1795477525	intron_variant,genic_downstream_transcript_variant
rs1795477599	intron_variant,genic_downstream_transcript_variant
rs1795477881	intron_variant,genic_downstream_transcript_variant
rs1795477964	intron_variant,genic_downstream_transcript_variant
rs1795478055	intron_variant,genic_downstream_transcript_variant
rs1795478196	intron_variant,genic_downstream_transcript_variant
rs1795479978	intron_variant,genic_downstream_transcript_variant
rs1795480101	intron_variant,genic_downstream_transcript_variant
rs1795480745	intron_variant,genic_downstream_transcript_variant
rs1795481014	intron_variant,genic_downstream_transcript_variant
rs1795481390	intron_variant,genic_downstream_transcript_variant
rs1795481473	intron_variant,genic_downstream_transcript_variant
rs1795481559	intron_variant,genic_downstream_transcript_variant
rs1795481727	intron_variant,genic_downstream_transcript_variant
rs1795481882	intron_variant,genic_downstream_transcript_variant
rs1795482064	intron_variant,genic_downstream_transcript_variant
rs1795482557	intron_variant,genic_downstream_transcript_variant

rs1795482911	intron_variant,genic_downstream_transcript_variant
rs1795483074	intron_variant,genic_downstream_transcript_variant
rs1795483416	intron_variant,genic_downstream_transcript_variant
rs1795483556	intron_variant,genic_downstream_transcript_variant
rs1795483801	intron_variant,genic_downstream_transcript_variant
rs1795484059	intron_variant,genic_downstream_transcript_variant
rs1795484353	intron_variant,genic_downstream_transcript_variant
rs1795484631	intron_variant,genic_downstream_transcript_variant
rs1795484711	intron_variant,genic_downstream_transcript_variant
rs1795485062	intron_variant,genic_downstream_transcript_variant
rs1795485329	intron_variant,genic_downstream_transcript_variant
rs1795485458	intron_variant,genic_downstream_transcript_variant
rs1795485850	intron_variant,genic_downstream_transcript_variant
rs1795487710	intron_variant,genic_downstream_transcript_variant
rs1795488522	intron_variant,genic_downstream_transcript_variant
rs1795488785	intron_variant,genic_downstream_transcript_variant
rs1795489118	intron_variant,genic_downstream_transcript_variant
rs1795489377	intron_variant,genic_downstream_transcript_variant
rs1795489474	intron_variant,genic_downstream_transcript_variant
rs1795489582	intron_variant,genic_downstream_transcript_variant
rs1795489827	intron_variant,genic_downstream_transcript_variant
rs1795489949	intron_variant,genic_downstream_transcript_variant
rs1795490175	intron_variant,genic_downstream_transcript_variant
rs1795490989	intron_variant,genic_downstream_transcript_variant
rs1795491117	intron_variant,genic_downstream_transcript_variant
rs1795492133	intron_variant,genic_downstream_transcript_variant
rs1795492771	intron_variant,genic_downstream_transcript_variant
rs1795493080	intron_variant,genic_downstream_transcript_variant
rs1795493440	intron_variant,genic_downstream_transcript_variant
rs1795493700	intron_variant,genic_downstream_transcript_variant
rs1795493948	intron_variant,genic_downstream_transcript_variant
rs1795494105	intron_variant,genic_downstream_transcript_variant
rs1795494248	intron_variant,genic_downstream_transcript_variant
rs1795494332	intron_variant,genic_downstream_transcript_variant
rs1795494649	intron_variant,genic_downstream_transcript_variant
rs1795494941	intron_variant,genic_downstream_transcript_variant
rs1795495095	intron_variant,genic_downstream_transcript_variant
rs1795495444	intron_variant,genic_downstream_transcript_variant
rs1795495666	intron_variant,genic_downstream_transcript_variant
rs1795497536	intron_variant,genic_downstream_transcript_variant
rs1795497846	intron_variant,genic_downstream_transcript_variant
rs1795498121	intron_variant,genic_downstream_transcript_variant
rs1795498630	intron_variant,genic_downstream_transcript_variant
rs1795498892	intron_variant,genic_downstream_transcript_variant
rs1795499021	intron_variant,genic_downstream_transcript_variant
rs1795499149	intron_variant,genic_downstream_transcript_variant
rs1795499292	intron_variant,genic_downstream_transcript_variant
rs1795499388	intron_variant,genic_downstream_transcript_variant
rs1795500160	intron_variant,genic_downstream_transcript_variant
rs1795500257	intron_variant,genic_downstream_transcript_variant
rs1795500622	intron_variant,genic_downstream_transcript_variant
rs1795500746	intron_variant,genic_downstream_transcript_variant
rs1795501357	intron_variant,genic_downstream_transcript_variant
rs1795502604	intron_variant,genic_downstream_transcript_variant
rs1795503475	intron_variant,genic_downstream_transcript_variant
rs1795504628	intron_variant,genic_downstream_transcript_variant
rs1795504951	intron_variant,genic_downstream_transcript_variant
rs1795506213	intron_variant,genic_downstream_transcript_variant
rs1795506344	intron_variant,genic_downstream_transcript_variant
rs1795506901	intron_variant,genic_downstream_transcript_variant
rs1795507291	intron_variant,genic_downstream_transcript_variant
rs1795508399	intron_variant,genic_downstream_transcript_variant
rs1795509273	intron_variant,genic_downstream_transcript_variant
rs1795509398	intron_variant,genic_downstream_transcript_variant
rs1795509951	intron_variant,genic_downstream_transcript_variant
rs1795510538	intron_variant,genic_downstream_transcript_variant
rs1795511638	intron_variant,genic_downstream_transcript_variant
rs1795511748	intron_variant,genic_downstream_transcript_variant
rs1795511878	intron_variant,genic_downstream_transcript_variant
rs1795511993	intron_variant,genic_downstream_transcript_variant
rs1795512319	intron_variant,genic_downstream_transcript_variant
rs1795512621	intron_variant,genic_downstream_transcript_variant
rs1795512769	intron_variant,genic_downstream_transcript_variant
rs1795513287	intron_variant,genic_downstream_transcript_variant

rs1795513533	intron_variant,genic_downstream_transcript_variant
rs1795514160	intron_variant,genic_downstream_transcript_variant
rs1795514302	intron_variant,genic_downstream_transcript_variant
rs1795514836	intron_variant,genic_downstream_transcript_variant
rs1795515147	intron_variant,genic_downstream_transcript_variant
rs1795515587	intron_variant,genic_downstream_transcript_variant
rs1795515976	intron_variant,genic_downstream_transcript_variant
rs1795516466	intron_variant,genic_downstream_transcript_variant
rs1795517052	intron_variant,genic_downstream_transcript_variant
rs1795517124	intron_variant,genic_downstream_transcript_variant
rs1795517196	intron_variant,genic_downstream_transcript_variant
rs1795517358	intron_variant,genic_downstream_transcript_variant
rs1795517931	intron_variant,genic_downstream_transcript_variant
rs1795518339	intron_variant,genic_downstream_transcript_variant
rs1795518445	intron_variant,genic_downstream_transcript_variant
rs1795520166	intron_variant,genic_downstream_transcript_variant
rs1795520542	intron_variant,genic_downstream_transcript_variant
rs1795520737	intron_variant,genic_downstream_transcript_variant
rs1795521475	intron_variant,genic_downstream_transcript_variant
rs1795521617	intron_variant,genic_downstream_transcript_variant
rs1795521716	intron_variant,genic_downstream_transcript_variant
rs1795522033	intron_variant,genic_downstream_transcript_variant
rs1795522258	intron_variant,genic_downstream_transcript_variant
rs1795522420	intron_variant,genic_downstream_transcript_variant
rs1795522888	intron_variant,genic_downstream_transcript_variant
rs1795523361	intron_variant,genic_downstream_transcript_variant
rs1795523462	intron_variant,genic_downstream_transcript_variant
rs1795523818	intron_variant,genic_downstream_transcript_variant
rs1795524050	intron_variant,genic_downstream_transcript_variant
rs1795524315	intron_variant,genic_downstream_transcript_variant
rs1795524473	intron_variant,genic_downstream_transcript_variant
rs1795524578	intron_variant,genic_downstream_transcript_variant
rs1795524666	intron_variant,genic_downstream_transcript_variant
rs1795524732	intron_variant,genic_downstream_transcript_variant
rs1795524907	intron_variant,genic_downstream_transcript_variant
rs1795525891	intron_variant,genic_downstream_transcript_variant
rs1795526194	intron_variant,genic_downstream_transcript_variant
rs1795526819	intron_variant,genic_downstream_transcript_variant
rs1795526898	intron_variant,genic_downstream_transcript_variant
rs1795526976	intron_variant,genic_downstream_transcript_variant
rs1795527061	intron_variant,genic_downstream_transcript_variant
rs1795527179	intron_variant,genic_downstream_transcript_variant
rs1795527435	intron_variant,genic_downstream_transcript_variant
rs1795528304	intron_variant,genic_downstream_transcript_variant
rs1795528479	intron_variant,genic_downstream_transcript_variant
rs1795528880	intron_variant,genic_downstream_transcript_variant
rs1795529090	intron_variant,genic_downstream_transcript_variant
rs1795529185	intron_variant,genic_downstream_transcript_variant
rs1795529554	intron_variant,genic_downstream_transcript_variant
rs1795529724	intron_variant,genic_downstream_transcript_variant
rs1795529803	intron_variant,genic_downstream_transcript_variant
rs1795529891	intron_variant,genic_downstream_transcript_variant
rs1795531009	intron_variant,genic_downstream_transcript_variant
rs1795531947	intron_variant,genic_downstream_transcript_variant
rs1795532396	intron_variant,genic_downstream_transcript_variant
rs1795533101	intron_variant,genic_downstream_transcript_variant
rs1795533224	intron_variant,genic_downstream_transcript_variant
rs1795533364	intron_variant,genic_downstream_transcript_variant
rs1795533638	intron_variant,genic_downstream_transcript_variant
rs1795535166	intron_variant,genic_downstream_transcript_variant
rs1795535309	intron_variant,genic_downstream_transcript_variant
rs1795535587	intron_variant,genic_downstream_transcript_variant
rs1795535721	intron_variant,genic_downstream_transcript_variant
rs1795537280	intron_variant,genic_downstream_transcript_variant
rs1795537570	intron_variant,genic_downstream_transcript_variant
rs1795537662	intron_variant,genic_downstream_transcript_variant
rs1795537852	intron_variant,genic_downstream_transcript_variant
rs1795538013	intron_variant,genic_downstream_transcript_variant
rs1795538201	intron_variant,genic_downstream_transcript_variant
rs1795538483	intron_variant,genic_downstream_transcript_variant
rs1795538754	intron_variant,genic_downstream_transcript_variant
rs1795538847	intron_variant,genic_downstream_transcript_variant
rs1795539021	intron_variant,genic_downstream_transcript_variant
rs1795539262	intron_variant,genic_downstream_transcript_variant

rs1795539343	intron_variant,genic_downstream_transcript_variant
rs1795539508	intron_variant,genic_downstream_transcript_variant
rs1795539584	intron_variant,genic_downstream_transcript_variant
rs1795539839	intron_variant,genic_downstream_transcript_variant
rs1795539921	intron_variant,genic_downstream_transcript_variant
rs1795540184	intron_variant,genic_downstream_transcript_variant
rs1795540267	intron_variant,genic_downstream_transcript_variant
rs1795540442	intron_variant,genic_downstream_transcript_variant
rs1795540540	intron_variant,genic_downstream_transcript_variant
rs1795540633	intron_variant,genic_downstream_transcript_variant
rs1795540909	intron_variant,genic_downstream_transcript_variant
rs1795541009	intron_variant,genic_downstream_transcript_variant
rs1795541178	intron_variant,genic_downstream_transcript_variant
rs1795541450	intron_variant,genic_downstream_transcript_variant
rs1795542003	intron_variant,genic_downstream_transcript_variant
rs1795542503	intron_variant,genic_downstream_transcript_variant
rs1795542790	intron_variant,genic_downstream_transcript_variant
rs1795543332	intron_variant,genic_downstream_transcript_variant
rs1795543527	intron_variant,genic_downstream_transcript_variant
rs1795543606	intron_variant,genic_downstream_transcript_variant
rs1795543773	intron_variant,genic_downstream_transcript_variant
rs1795544079	intron_variant,genic_downstream_transcript_variant
rs1795544879	intron_variant,genic_downstream_transcript_variant
rs1795544974	intron_variant,genic_downstream_transcript_variant
rs1795545301	intron_variant,genic_downstream_transcript_variant
rs1795545396	intron_variant,genic_downstream_transcript_variant
rs1795545503	intron_variant,genic_downstream_transcript_variant
rs1795545608	intron_variant,genic_downstream_transcript_variant
rs1795546009	intron_variant,genic_downstream_transcript_variant
rs1795546180	intron_variant,genic_downstream_transcript_variant
rs1795546468	intron_variant,genic_downstream_transcript_variant
rs1795546643	intron_variant,genic_downstream_transcript_variant
rs1795546727	intron_variant,genic_downstream_transcript_variant
rs1795546829	intron_variant,genic_downstream_transcript_variant
rs1795547140	intron_variant,genic_downstream_transcript_variant
rs1795547691	intron_variant,genic_downstream_transcript_variant
rs1795547969	intron_variant,genic_downstream_transcript_variant
rs1795548519	intron_variant,genic_downstream_transcript_variant
rs1795548840	intron_variant,genic_downstream_transcript_variant
rs1795548976	intron_variant,genic_downstream_transcript_variant
rs1795549391	intron_variant,genic_downstream_transcript_variant
rs1795549661	intron_variant,genic_downstream_transcript_variant
rs1795549760	intron_variant,genic_downstream_transcript_variant
rs1795549855	intron_variant,genic_downstream_transcript_variant
rs1795550440	intron_variant,genic_downstream_transcript_variant
rs1795550528	intron_variant,genic_downstream_transcript_variant
rs1795550609	intron_variant,genic_downstream_transcript_variant
rs1795550826	intron_variant,genic_downstream_transcript_variant
rs1795550920	intron_variant,genic_downstream_transcript_variant
rs1795551338	intron_variant,genic_downstream_transcript_variant
rs1795551427	intron_variant,genic_downstream_transcript_variant
rs1795551516	intron_variant,genic_downstream_transcript_variant
rs1795551897	intron_variant,genic_downstream_transcript_variant
rs1795552004	intron_variant,genic_downstream_transcript_variant
rs1795552565	intron_variant,genic_downstream_transcript_variant
rs1795552705	intron_variant,genic_downstream_transcript_variant
rs1795552853	intron_variant,genic_downstream_transcript_variant
rs1795553133	intron_variant,genic_downstream_transcript_variant
rs1795553228	intron_variant,genic_downstream_transcript_variant
rs1795553342	intron_variant,genic_downstream_transcript_variant
rs1795553596	intron_variant,genic_downstream_transcript_variant
rs1795553793	intron_variant,genic_downstream_transcript_variant
rs1795554003	intron_variant,genic_downstream_transcript_variant
rs1795554991	intron_variant,genic_downstream_transcript_variant
rs1795555198	intron_variant,genic_downstream_transcript_variant
rs1795555291	intron_variant,genic_downstream_transcript_variant
rs1795555497	intron_variant,genic_downstream_transcript_variant
rs1795555728	intron_variant,genic_downstream_transcript_variant
rs1795556030	intron_variant,genic_downstream_transcript_variant
rs1795556533	intron_variant,genic_downstream_transcript_variant
rs1795556872	intron_variant,genic_downstream_transcript_variant
rs1795557014	intron_variant,genic_downstream_transcript_variant
rs1795557159	intron_variant,genic_downstream_transcript_variant
rs1795558017	intron_variant,genic_downstream_transcript_variant

rs1795558279	intron_variant,genic_downstream_transcript_variant
rs1795558776	intron_variant,genic_downstream_transcript_variant
rs1795558954	intron_variant,genic_downstream_transcript_variant
rs1795559135	intron_variant,genic_downstream_transcript_variant
rs1795559354	intron_variant,genic_downstream_transcript_variant
rs1795559807	intron_variant,genic_downstream_transcript_variant
rs1795560065	intron_variant,genic_downstream_transcript_variant
rs1795560663	intron_variant,genic_downstream_transcript_variant
rs1795561129	intron_variant,genic_downstream_transcript_variant
rs1795561794	intron_variant,genic_downstream_transcript_variant
rs1795562214	intron_variant,genic_downstream_transcript_variant
rs1795562455	intron_variant,genic_downstream_transcript_variant
rs1795562536	intron_variant,genic_downstream_transcript_variant
rs1795563153	intron_variant,genic_downstream_transcript_variant
rs1795563567	intron_variant,genic_downstream_transcript_variant
rs1795564118	intron_variant,genic_downstream_transcript_variant
rs1795564680	intron_variant,genic_downstream_transcript_variant
rs1795564952	intron_variant,genic_downstream_transcript_variant
rs1795565070	intron_variant,genic_downstream_transcript_variant
rs1795565380	intron_variant,genic_downstream_transcript_variant
rs1795565871	intron_variant,genic_downstream_transcript_variant
rs1795565951	intron_variant,genic_downstream_transcript_variant
rs1795566034	intron_variant,genic_downstream_transcript_variant
rs1795566180	intron_variant,genic_downstream_transcript_variant
rs1795567121	intron_variant,genic_downstream_transcript_variant
rs1795567281	intron_variant,genic_downstream_transcript_variant
rs1795567430	intron_variant,genic_downstream_transcript_variant
rs1795567646	intron_variant,genic_downstream_transcript_variant
rs1795567715	intron_variant,genic_downstream_transcript_variant
rs1795568020	intron_variant,genic_downstream_transcript_variant
rs1795568084	intron_variant,genic_downstream_transcript_variant
rs1795568562	intron_variant,genic_downstream_transcript_variant
rs1795568857	intron_variant,genic_downstream_transcript_variant
rs1795569099	intron_variant,genic_downstream_transcript_variant
rs1795569972	intron_variant,genic_downstream_transcript_variant
rs1795570585	intron_variant,genic_downstream_transcript_variant
rs1795570723	intron_variant,genic_downstream_transcript_variant
rs1795570805	intron_variant,genic_downstream_transcript_variant
rs1795571106	intron_variant,genic_downstream_transcript_variant
rs1795571283	intron_variant,genic_downstream_transcript_variant
rs1795571611	intron_variant,genic_downstream_transcript_variant
rs1795571897	intron_variant,genic_downstream_transcript_variant
rs1795572174	intron_variant,genic_downstream_transcript_variant
rs1795572400	intron_variant,genic_downstream_transcript_variant
rs1795573084	intron_variant,genic_downstream_transcript_variant
rs1795573180	intron_variant,genic_downstream_transcript_variant
rs1795573549	intron_variant,genic_downstream_transcript_variant
rs1795573817	intron_variant,genic_downstream_transcript_variant
rs1795574070	intron_variant,genic_downstream_transcript_variant
rs1795574170	intron_variant,genic_downstream_transcript_variant
rs1795574388	intron_variant,genic_downstream_transcript_variant
rs1795576130	intron_variant,genic_downstream_transcript_variant
rs1795576294	intron_variant,genic_downstream_transcript_variant
rs1795576396	intron_variant,genic_downstream_transcript_variant
rs1795576766	intron_variant,genic_downstream_transcript_variant
rs1795577584	intron_variant,genic_downstream_transcript_variant
rs1795577930	intron_variant,genic_downstream_transcript_variant
rs1795578094	intron_variant,genic_downstream_transcript_variant
rs1795578260	intron_variant,genic_downstream_transcript_variant
rs1795578343	intron_variant,genic_downstream_transcript_variant
rs1795578522	intron_variant,genic_downstream_transcript_variant
rs1795578697	intron_variant,genic_downstream_transcript_variant
rs1795578791	intron_variant,genic_downstream_transcript_variant
rs1795578899	intron_variant,genic_downstream_transcript_variant
rs1795579348	intron_variant,genic_downstream_transcript_variant
rs1795579435	intron_variant,genic_downstream_transcript_variant
rs1795579509	intron_variant,genic_downstream_transcript_variant
rs1795579917	intron_variant,genic_downstream_transcript_variant
rs1795580075	intron_variant,genic_downstream_transcript_variant
rs1795580167	intron_variant,genic_downstream_transcript_variant
rs1795580693	intron_variant,genic_downstream_transcript_variant
rs1795580781	intron_variant,genic_downstream_transcript_variant
rs1795580864	intron_variant,genic_downstream_transcript_variant
rs1795581138	intron_variant,genic_downstream_transcript_variant

rs1795581264	intron_variant,genic_downstream_transcript_variant
rs1795581599	intron_variant,genic_downstream_transcript_variant
rs1795581775	intron_variant,genic_downstream_transcript_variant
rs1795581850	intron_variant,genic_downstream_transcript_variant
rs1795582223	intron_variant,genic_downstream_transcript_variant
rs1795582412	intron_variant,genic_downstream_transcript_variant
rs1795582607	intron_variant,genic_downstream_transcript_variant
rs1795582798	intron_variant,genic_downstream_transcript_variant
rs1795582884	intron_variant,genic_downstream_transcript_variant
rs1795582963	intron_variant,genic_downstream_transcript_variant
rs1795583211	intron_variant,genic_downstream_transcript_variant
rs1795583291	intron_variant,genic_downstream_transcript_variant
rs1795583370	intron_variant,genic_downstream_transcript_variant
rs1795583448	intron_variant,genic_downstream_transcript_variant
rs1795584159	intron_variant,genic_downstream_transcript_variant
rs1795584493	intron_variant,genic_downstream_transcript_variant
rs1795585857	intron_variant,genic_downstream_transcript_variant
rs1795586613	intron_variant,genic_downstream_transcript_variant
rs1795587038	intron_variant,genic_downstream_transcript_variant
rs1795587825	intron_variant,genic_downstream_transcript_variant
rs1795588294	intron_variant,genic_downstream_transcript_variant
rs1795588645	intron_variant,genic_downstream_transcript_variant
rs1795590337	intron_variant,genic_downstream_transcript_variant
rs1795591103	intron_variant,genic_downstream_transcript_variant
rs1795591233	intron_variant,genic_downstream_transcript_variant
rs1795591373	intron_variant,genic_downstream_transcript_variant
rs1795592817	intron_variant,genic_downstream_transcript_variant
rs1795592941	intron_variant,genic_downstream_transcript_variant
rs1795593570	intron_variant,genic_downstream_transcript_variant
rs1795594103	intron_variant,genic_downstream_transcript_variant
rs1795594172	intron_variant,genic_downstream_transcript_variant
rs1795594232	intron_variant,genic_downstream_transcript_variant
rs1795594444	intron_variant,genic_downstream_transcript_variant
rs1795595147	intron_variant,genic_downstream_transcript_variant
rs1795595223	intron_variant,genic_downstream_transcript_variant
rs1795595493	intron_variant,genic_downstream_transcript_variant
rs1795595569	intron_variant,genic_downstream_transcript_variant
rs1795595695	intron_variant,genic_downstream_transcript_variant
rs1795595775	intron_variant,genic_downstream_transcript_variant
rs1795596071	intron_variant,genic_downstream_transcript_variant
rs1795596155	intron_variant,genic_downstream_transcript_variant
rs1795596246	intron_variant,genic_downstream_transcript_variant
rs1795596709	intron_variant,genic_downstream_transcript_variant
rs1795597724	intron_variant,genic_downstream_transcript_variant
rs1795597983	intron_variant,genic_downstream_transcript_variant
rs1795598066	intron_variant,genic_downstream_transcript_variant
rs1795598243	intron_variant,genic_downstream_transcript_variant
rs1795598589	intron_variant,genic_downstream_transcript_variant
rs1795599348	intron_variant,genic_downstream_transcript_variant
rs1795599649	intron_variant,genic_downstream_transcript_variant
rs1795600012	intron_variant,genic_downstream_transcript_variant
rs1795600198	intron_variant,genic_downstream_transcript_variant
rs1795600266	intron_variant,genic_downstream_transcript_variant
rs1795600363	intron_variant,genic_downstream_transcript_variant
rs1795600477	intron_variant,genic_downstream_transcript_variant
rs1795600636	intron_variant,genic_downstream_transcript_variant
rs1795600809	intron_variant,genic_downstream_transcript_variant
rs1795601208	intron_variant,genic_downstream_transcript_variant
rs1795601288	intron_variant,genic_downstream_transcript_variant
rs1795601460	intron_variant,genic_downstream_transcript_variant
rs1795601567	intron_variant,genic_downstream_transcript_variant
rs1795601838	intron_variant,genic_downstream_transcript_variant
rs1795602018	intron_variant,genic_downstream_transcript_variant
rs1795602233	intron_variant,genic_downstream_transcript_variant
rs1795602566	intron_variant,genic_downstream_transcript_variant
rs1795602655	intron_variant,genic_downstream_transcript_variant
rs1795602985	intron_variant,genic_downstream_transcript_variant
rs1795603242	intron_variant,genic_downstream_transcript_variant
rs1795603483	intron_variant,genic_downstream_transcript_variant
rs1795603553	intron_variant,genic_downstream_transcript_variant
rs1795604095	intron_variant,genic_downstream_transcript_variant
rs1795605547	intron_variant,genic_downstream_transcript_variant
rs1795605632	intron_variant,genic_downstream_transcript_variant
rs1795605736	intron_variant,genic_downstream_transcript_variant

rs1795605985	intron_variant,genic_downstream_transcript_variant
rs1795606873	intron_variant,genic_downstream_transcript_variant
rs1795607138	intron_variant,genic_downstream_transcript_variant
rs1795607209	intron_variant,genic_downstream_transcript_variant
rs1795607289	intron_variant,genic_downstream_transcript_variant
rs1795607612	intron_variant,genic_downstream_transcript_variant
rs1795607790	intron_variant,genic_downstream_transcript_variant
rs1795607973	intron_variant,genic_downstream_transcript_variant
rs1795608151	intron_variant,genic_downstream_transcript_variant
rs1795608255	intron_variant,genic_downstream_transcript_variant
rs1795608435	intron_variant,genic_downstream_transcript_variant
rs1795608644	intron_variant,genic_downstream_transcript_variant
rs1795608986	intron_variant,genic_downstream_transcript_variant
rs1795609079	intron_variant,genic_downstream_transcript_variant
rs1795609393	intron_variant,genic_downstream_transcript_variant
rs1795609487	intron_variant,genic_downstream_transcript_variant
rs1795609668	intron_variant,genic_downstream_transcript_variant
rs1795609766	intron_variant,genic_downstream_transcript_variant
rs1795610418	intron_variant,genic_downstream_transcript_variant
rs1795610979	intron_variant,genic_downstream_transcript_variant
rs1795611171	intron_variant,genic_downstream_transcript_variant
rs1795611258	intron_variant,genic_downstream_transcript_variant
rs1795611352	intron_variant,genic_downstream_transcript_variant
rs1795611539	intron_variant,genic_downstream_transcript_variant
rs1795611631	intron_variant,genic_downstream_transcript_variant
rs1795611843	intron_variant,genic_downstream_transcript_variant
rs1795611935	intron_variant,genic_downstream_transcript_variant
rs1795612029	intron_variant,genic_downstream_transcript_variant
rs1795612537	intron_variant,genic_downstream_transcript_variant
rs1795612713	intron_variant,genic_downstream_transcript_variant
rs1795613387	intron_variant,genic_downstream_transcript_variant
rs1795613560	intron_variant,genic_downstream_transcript_variant
rs1795613656	intron_variant,genic_downstream_transcript_variant
rs1795614110	intron_variant,genic_downstream_transcript_variant
rs1795614403	intron_variant,genic_downstream_transcript_variant
rs1795614491	intron_variant,genic_downstream_transcript_variant
rs1795614680	intron_variant,genic_downstream_transcript_variant
rs1795614768	intron_variant,genic_downstream_transcript_variant
rs1795614990	intron_variant,genic_downstream_transcript_variant
rs1795615160	intron_variant,genic_downstream_transcript_variant
rs1795615349	intron_variant,genic_downstream_transcript_variant
rs1795615431	intron_variant,genic_downstream_transcript_variant
rs1795615588	intron_variant,genic_downstream_transcript_variant
rs1795615697	intron_variant,genic_downstream_transcript_variant
rs1795615781	intron_variant,genic_downstream_transcript_variant
rs1795615975	intron_variant,genic_downstream_transcript_variant
rs1795616294	intron_variant,genic_downstream_transcript_variant
rs1795616368	intron_variant,genic_downstream_transcript_variant
rs1795616642	intron_variant,genic_downstream_transcript_variant
rs1795616799	intron_variant,genic_downstream_transcript_variant
rs1795617258	intron_variant,genic_downstream_transcript_variant
rs1795617341	intron_variant,genic_downstream_transcript_variant
rs1795617851	intron_variant,genic_downstream_transcript_variant
rs1795618229	intron_variant,genic_downstream_transcript_variant
rs1795618319	intron_variant,genic_downstream_transcript_variant
rs1795618502	intron_variant,genic_downstream_transcript_variant
rs1795618851	intron_variant,genic_downstream_transcript_variant
rs1795619141	intron_variant,genic_downstream_transcript_variant
rs1795619894	intron_variant,genic_downstream_transcript_variant
rs1795620404	intron_variant,genic_downstream_transcript_variant
rs1795620651	intron_variant,genic_downstream_transcript_variant
rs1795620820	intron_variant,genic_downstream_transcript_variant
rs1795620905	intron_variant,genic_downstream_transcript_variant
rs1795621223	intron_variant,genic_downstream_transcript_variant
rs1795621692	intron_variant,genic_downstream_transcript_variant
rs1795621799	intron_variant,genic_downstream_transcript_variant
rs1795622003	intron_variant,genic_downstream_transcript_variant
rs1795622592	intron_variant,genic_downstream_transcript_variant
rs1795623182	intron_variant,genic_downstream_transcript_variant
rs1795623367	intron_variant,genic_downstream_transcript_variant
rs1795623537	intron_variant,genic_downstream_transcript_variant
rs1795623619	intron_variant,genic_downstream_transcript_variant
rs1795623909	intron_variant,genic_downstream_transcript_variant
rs1795623990	intron_variant,genic_downstream_transcript_variant

rs1795624068	intron_variant,genic_downstream_transcript_variant
rs1795624153	intron_variant,genic_downstream_transcript_variant
rs1795624457	intron_variant,genic_downstream_transcript_variant
rs1795624529	intron_variant,genic_downstream_transcript_variant
rs1795624699	intron_variant,genic_downstream_transcript_variant
rs1795624777	intron_variant,genic_downstream_transcript_variant
rs1795625284	intron_variant,genic_downstream_transcript_variant
rs1795625544	intron_variant,genic_downstream_transcript_variant
rs1795625723	intron_variant,genic_downstream_transcript_variant
rs1795626010	intron_variant,genic_downstream_transcript_variant
rs1795626221	intron_variant,genic_downstream_transcript_variant
rs1795626875	intron_variant,genic_downstream_transcript_variant
rs1795626967	intron_variant,genic_downstream_transcript_variant
rs1795627159	intron_variant,genic_downstream_transcript_variant
rs1795627267	intron_variant,genic_downstream_transcript_variant
rs1795627362	intron_variant,genic_downstream_transcript_variant
rs1795627468	intron_variant,genic_downstream_transcript_variant
rs1795627929	intron_variant,genic_downstream_transcript_variant
rs1795628325	intron_variant,genic_downstream_transcript_variant
rs1795628748	intron_variant,genic_downstream_transcript_variant
rs1795628933	intron_variant,genic_downstream_transcript_variant
rs1795629097	intron_variant,genic_downstream_transcript_variant
rs1795630265	intron_variant,genic_downstream_transcript_variant
rs1795630518	intron_variant,genic_downstream_transcript_variant
rs1795630800	intron_variant,genic_downstream_transcript_variant
rs1795630978	intron_variant,genic_downstream_transcript_variant
rs1795631064	intron_variant,genic_downstream_transcript_variant
rs1795631146	intron_variant,genic_downstream_transcript_variant
rs1795631304	intron_variant,genic_downstream_transcript_variant
rs1795631707	intron_variant,genic_downstream_transcript_variant
rs1795631974	intron_variant,genic_downstream_transcript_variant
rs1795632156	intron_variant,genic_downstream_transcript_variant
rs1795632353	intron_variant,genic_downstream_transcript_variant
rs1795632539	intron_variant,genic_downstream_transcript_variant
rs1795632735	intron_variant,genic_downstream_transcript_variant
rs1795632822	intron_variant,genic_downstream_transcript_variant
rs1795634589	intron_variant,genic_downstream_transcript_variant
rs1795634857	intron_variant,genic_downstream_transcript_variant
rs1795635046	intron_variant,genic_downstream_transcript_variant
rs1795635138	intron_variant,genic_downstream_transcript_variant
rs1795635252	intron_variant,genic_downstream_transcript_variant
rs1795635466	intron_variant,genic_downstream_transcript_variant
rs1795635641	intron_variant,genic_downstream_transcript_variant
rs1795635725	intron_variant,genic_downstream_transcript_variant
rs1795635920	intron_variant,genic_downstream_transcript_variant
rs1795636445	intron_variant,genic_downstream_transcript_variant
rs1795636546	intron_variant,genic_downstream_transcript_variant
rs1795636631	intron_variant,genic_downstream_transcript_variant
rs1795637007	intron_variant,genic_downstream_transcript_variant
rs1795637228	intron_variant,genic_downstream_transcript_variant
rs1795637354	intron_variant,genic_downstream_transcript_variant
rs1795637441	intron_variant,genic_downstream_transcript_variant
rs1795637725	intron_variant,genic_downstream_transcript_variant
rs1795637810	intron_variant,genic_downstream_transcript_variant
rs1795638335	intron_variant,genic_downstream_transcript_variant
rs1795638428	intron_variant,genic_downstream_transcript_variant
rs1795638538	intron_variant,genic_downstream_transcript_variant
rs1795638989	intron_variant,genic_downstream_transcript_variant
rs1795639078	intron_variant,genic_downstream_transcript_variant
rs1795639337	intron_variant,genic_downstream_transcript_variant
rs1795639545	intron_variant,genic_downstream_transcript_variant
rs1795639756	intron_variant,genic_downstream_transcript_variant
rs1795639964	intron_variant,genic_downstream_transcript_variant
rs1795640235	intron_variant,genic_downstream_transcript_variant
rs1795640329	intron_variant,genic_downstream_transcript_variant
rs1795640613	intron_variant,genic_downstream_transcript_variant
rs1795640796	intron_variant,genic_downstream_transcript_variant
rs1795641082	intron_variant,genic_downstream_transcript_variant
rs1795641303	intron_variant,genic_downstream_transcript_variant
rs1795641725	intron_variant,genic_downstream_transcript_variant
rs1795641820	intron_variant,genic_downstream_transcript_variant
rs1795642095	intron_variant,genic_downstream_transcript_variant
rs1795642368	intron_variant,genic_downstream_transcript_variant
rs1795642570	intron_variant,genic_downstream_transcript_variant

rs1795642645	intron_variant,genic_downstream_transcript_variant
rs1795642738	intron_variant,genic_downstream_transcript_variant
rs1795642836	intron_variant,genic_downstream_transcript_variant
rs1795643248	intron_variant,genic_downstream_transcript_variant
rs1795643338	intron_variant,genic_downstream_transcript_variant
rs1795644014	intron_variant,genic_downstream_transcript_variant
rs1795644095	intron_variant,genic_downstream_transcript_variant
rs1795644889	intron_variant,genic_downstream_transcript_variant
rs1795645051	intron_variant,genic_downstream_transcript_variant
rs1795646165	missense_variant,non_coding_transcript_variant,coding_sequence_variant,genic_downstream_transcript_variant
rs1795646491	missense_variant,non_coding_transcript_variant,coding_sequence_variant,genic_downstream_transcript_variant
rs1795646988	missense_variant,non_coding_transcript_variant,coding_sequence_variant,genic_downstream_transcript_variant
rs1795647105	non_coding_transcript_variant,coding_sequence_variant,synonymous_variant,genic_downstream_transcript_variant
rs1795648934	non_coding_transcript_variant,coding_sequence_variant,synonymous_variant,genic_downstream_transcript_variant
rs1795650693	intron_variant,genic_downstream_transcript_variant
rs1795651185	intron_variant,genic_downstream_transcript_variant
rs1795651247	intron_variant,genic_downstream_transcript_variant
rs1795651441	intron_variant,genic_downstream_transcript_variant
rs1795651834	intron_variant,genic_downstream_transcript_variant
rs1795652218	intron_variant,genic_downstream_transcript_variant
rs1795652334	intron_variant,genic_downstream_transcript_variant
rs1795653182	intron_variant,genic_downstream_transcript_variant
rs1795653967	intron_variant,genic_downstream_transcript_variant
rs1795656686	missense_variant,non_coding_transcript_variant,coding_sequence_variant,genic_downstream_transcript_variant
rs1795657946	missense_variant,non_coding_transcript_variant,coding_sequence_variant,genic_downstream_transcript_variant
rs1795659339	missense_variant,non_coding_transcript_variant,coding_sequence_variant,genic_downstream_transcript_variant
rs1795660204	missense_variant,non_coding_transcript_variant,coding_sequence_variant,genic_downstream_transcript_variant
rs1795660296	non_coding_transcript_variant,coding_sequence_variant,synonymous_variant,genic_downstream_transcript_variant
rs1795662590	missense_variant,non_coding_transcript_variant,coding_sequence_variant,genic_downstream_transcript_variant
rs1795663226	non_coding_transcript_variant,coding_sequence_variant,synonymous_variant,genic_downstream_transcript_variant
rs1795663624	non_coding_transcript_variant,coding_sequence_variant,synonymous_variant,genic_downstream_transcript_variant
rs1795663778	non_coding_transcript_variant,coding_sequence_variant,synonymous_variant,genic_downstream_transcript_variant
rs1795665686	missense_variant,non_coding_transcript_variant,coding_sequence_variant,genic_downstream_transcript_variant
rs1795667792	missense_variant,non_coding_transcript_variant,coding_sequence_variant,genic_downstream_transcript_variant
rs1795668596	genic_downstream_transcript_variant,non_coding_transcript_variant,3_prime_UTR_variant
rs1795668695	genic_downstream_transcript_variant,non_coding_transcript_variant,3_prime_UTR_variant
rs1795670453	genic_downstream_transcript_variant,non_coding_transcript_variant,3_prime_UTR_variant
rs1795670736	genic_downstream_transcript_variant,non_coding_transcript_variant,3_prime_UTR_variant
rs1795670946	genic_downstream_transcript_variant,non_coding_transcript_variant,3_prime_UTR_variant
rs1795671080	genic_downstream_transcript_variant,non_coding_transcript_variant,3_prime_UTR_variant
rs1795671195	genic_downstream_transcript_variant,non_coding_transcript_variant,3_prime_UTR_variant
rs1795671637	genic_downstream_transcript_variant,non_coding_transcript_variant,3_prime_UTR_variant
rs1795671916	genic_downstream_transcript_variant,non_coding_transcript_variant,3_prime_UTR_variant
rs1795672161	genic_downstream_transcript_variant,non_coding_transcript_variant,3_prime_UTR_variant
rs1795672298	genic_downstream_transcript_variant,non_coding_transcript_variant,3_prime_UTR_variant
rs1795673197	genic_downstream_transcript_variant,non_coding_transcript_variant,3_prime_UTR_variant
rs1795673482	genic_downstream_transcript_variant,non_coding_transcript_variant,3_prime_UTR_variant
rs1795674301	genic_downstream_transcript_variant,non_coding_transcript_variant,3_prime_UTR_variant
rs1795674412	genic_downstream_transcript_variant,non_coding_transcript_variant,3_prime_UTR_variant
rs1795674507	genic_downstream_transcript_variant,non_coding_transcript_variant,3_prime_UTR_variant
rs1795674715	genic_downstream_transcript_variant,non_coding_transcript_variant,3_prime_UTR_variant
rs1795674938	genic_downstream_transcript_variant,non_coding_transcript_variant,3_prime_UTR_variant
rs1795675034	genic_downstream_transcript_variant,non_coding_transcript_variant,3_prime_UTR_variant
rs1795675254	genic_downstream_transcript_variant,non_coding_transcript_variant,3_prime_UTR_variant
rs1795675558	genic_downstream_transcript_variant,non_coding_transcript_variant,3_prime_UTR_variant
rs1795676076	genic_downstream_transcript_variant,non_coding_transcript_variant,3_prime_UTR_variant
rs1795676264	genic_downstream_transcript_variant,non_coding_transcript_variant,3_prime_UTR_variant
rs1795677233	genic_downstream_transcript_variant,non_coding_transcript_variant,3_prime_UTR_variant
rs1795677701	genic_downstream_transcript_variant,non_coding_transcript_variant,3_prime_UTR_variant
rs1795678015	genic_downstream_transcript_variant,non_coding_transcript_variant,3_prime_UTR_variant
rs1795678503	genic_downstream_transcript_variant,non_coding_transcript_variant,3_prime_UTR_variant
rs1795678838	genic_downstream_transcript_variant,non_coding_transcript_variant,3_prime_UTR_variant
rs1795678960	genic_downstream_transcript_variant,non_coding_transcript_variant,3_prime_UTR_variant
rs1795679660	genic_downstream_transcript_variant,non_coding_transcript_variant,3_prime_UTR_variant
rs1795679864	genic_downstream_transcript_variant,non_coding_transcript_variant,3_prime_UTR_variant
rs1795680484	genic_downstream_transcript_variant,non_coding_transcript_variant,3_prime_UTR_variant
rs1795680695	genic_downstream_transcript_variant,non_coding_transcript_variant,3_prime_UTR_variant
rs1795681292	genic_downstream_transcript_variant,non_coding_transcript_variant,3_prime_UTR_variant
rs1795681394	genic_downstream_transcript_variant,non_coding_transcript_variant,3_prime_UTR_variant
rs1795681606	genic_downstream_transcript_variant,non_coding_transcript_variant,3_prime_UTR_variant
rs1795681700	genic_downstream_transcript_variant,non_coding_transcript_variant,3_prime_UTR_variant
rs1795681785	genic_downstream_transcript_variant,non_coding_transcript_variant,3_prime_UTR_variant
rs1795681878	genic_downstream_transcript_variant,non_coding_transcript_variant,3_prime_UTR_variant
rs1795682369	genic_downstream_transcript_variant,non_coding_transcript_variant,3_prime_UTR_variant

rs1795682468	genic_downstream_transcript_variant,non_coding_transcript_variant,3_prime_UTR_variant
rs1795682555	genic_downstream_transcript_variant,non_coding_transcript_variant,3_prime_UTR_variant
rs1795682653	genic_downstream_transcript_variant,non_coding_transcript_variant,3_prime_UTR_variant
rs1795683327	genic_downstream_transcript_variant,non_coding_transcript_variant,3_prime_UTR_variant
rs1795683764	genic_downstream_transcript_variant,non_coding_transcript_variant,3_prime_UTR_variant
rs1795683869	genic_downstream_transcript_variant,non_coding_transcript_variant,3_prime_UTR_variant
rs1795684296	genic_downstream_transcript_variant,non_coding_transcript_variant,3_prime_UTR_variant
rs1795685061	genic_downstream_transcript_variant,non_coding_transcript_variant,3_prime_UTR_variant
rs1795685392	genic_downstream_transcript_variant,non_coding_transcript_variant,3_prime_UTR_variant
rs1795685539	genic_downstream_transcript_variant,non_coding_transcript_variant,3_prime_UTR_variant
rs1795686035	genic_downstream_transcript_variant,non_coding_transcript_variant,3_prime_UTR_variant
rs1795686142	genic_downstream_transcript_variant,non_coding_transcript_variant,3_prime_UTR_variant
rs1795686248	genic_downstream_transcript_variant,non_coding_transcript_variant,3_prime_UTR_variant
rs1795686396	genic_downstream_transcript_variant,non_coding_transcript_variant,3_prime_UTR_variant
rs1795686567	genic_downstream_transcript_variant,non_coding_transcript_variant,3_prime_UTR_variant
rs1795687094	genic_downstream_transcript_variant,non_coding_transcript_variant,3_prime_UTR_variant
rs1795687741	genic_downstream_transcript_variant,non_coding_transcript_variant,3_prime_UTR_variant
rs1795688017	genic_downstream_transcript_variant,non_coding_transcript_variant,3_prime_UTR_variant
rs1795688385	genic_downstream_transcript_variant,non_coding_transcript_variant,3_prime_UTR_variant
rs1795688467	genic_downstream_transcript_variant,non_coding_transcript_variant,3_prime_UTR_variant
rs1795689136	genic_downstream_transcript_variant,non_coding_transcript_variant,3_prime_UTR_variant
rs1795689717	genic_downstream_transcript_variant,non_coding_transcript_variant,3_prime_UTR_variant
rs1795689936	genic_downstream_transcript_variant,non_coding_transcript_variant,3_prime_UTR_variant
rs1795690049	genic_downstream_transcript_variant,non_coding_transcript_variant,3_prime_UTR_variant
rs1795690512	genic_downstream_transcript_variant,non_coding_transcript_variant,3_prime_UTR_variant
rs1795690760	genic_downstream_transcript_variant,non_coding_transcript_variant,3_prime_UTR_variant
rs1795691544	genic_downstream_transcript_variant,non_coding_transcript_variant,3_prime_UTR_variant
rs1795691993	genic_downstream_transcript_variant,non_coding_transcript_variant,3_prime_UTR_variant
rs1795692570	genic_downstream_transcript_variant,non_coding_transcript_variant,3_prime_UTR_variant
rs1795693121	genic_downstream_transcript_variant,non_coding_transcript_variant,3_prime_UTR_variant
rs1795693511	genic_downstream_transcript_variant,non_coding_transcript_variant,3_prime_UTR_variant
rs1795693592	genic_downstream_transcript_variant,non_coding_transcript_variant,3_prime_UTR_variant
rs1795693867	genic_downstream_transcript_variant,non_coding_transcript_variant,3_prime_UTR_variant
rs1795693955	genic_downstream_transcript_variant,non_coding_transcript_variant,3_prime_UTR_variant
rs1795695189	genic_downstream_transcript_variant,non_coding_transcript_variant,3_prime_UTR_variant
rs1795695936	genic_downstream_transcript_variant,non_coding_transcript_variant,3_prime_UTR_variant
rs1795696100	genic_downstream_transcript_variant,non_coding_transcript_variant,3_prime_UTR_variant
rs1795696267	genic_downstream_transcript_variant,non_coding_transcript_variant,3_prime_UTR_variant
rs1795696333	genic_downstream_transcript_variant,non_coding_transcript_variant,3_prime_UTR_variant
rs1795696419	genic_downstream_transcript_variant,non_coding_transcript_variant,3_prime_UTR_variant
rs1795696498	genic_downstream_transcript_variant,non_coding_transcript_variant,3_prime_UTR_variant
rs1795696641	genic_downstream_transcript_variant,non_coding_transcript_variant,3_prime_UTR_variant
rs1795696804	genic_downstream_transcript_variant,non_coding_transcript_variant,3_prime_UTR_variant
rs1795697436	genic_downstream_transcript_variant,non_coding_transcript_variant,3_prime_UTR_variant
rs1795697512	genic_downstream_transcript_variant,non_coding_transcript_variant,3_prime_UTR_variant
rs1795697721	genic_downstream_transcript_variant,non_coding_transcript_variant,3_prime_UTR_variant
rs1795697810	genic_downstream_transcript_variant,non_coding_transcript_variant,3_prime_UTR_variant
rs1795698724	genic_downstream_transcript_variant,non_coding_transcript_variant,3_prime_UTR_variant
rs1795698805	genic_downstream_transcript_variant,non_coding_transcript_variant,3_prime_UTR_variant
rs1795698884	genic_downstream_transcript_variant,non_coding_transcript_variant,3_prime_UTR_variant
rs1795699597	genic_downstream_transcript_variant,non_coding_transcript_variant,3_prime_UTR_variant
rs1795699678	genic_downstream_transcript_variant,non_coding_transcript_variant,3_prime_UTR_variant
rs1795699742	genic_downstream_transcript_variant,non_coding_transcript_variant,3_prime_UTR_variant
rs1795699817	genic_downstream_transcript_variant,non_coding_transcript_variant,3_prime_UTR_variant
rs1795700043	genic_downstream_transcript_variant,non_coding_transcript_variant,3_prime_UTR_variant
rs1795700214	genic_downstream_transcript_variant,non_coding_transcript_variant,3_prime_UTR_variant
rs1795700289	genic_downstream_transcript_variant,non_coding_transcript_variant,3_prime_UTR_variant
rs1795700547	genic_downstream_transcript_variant,non_coding_transcript_variant,3_prime_UTR_variant
rs1795700856	genic_downstream_transcript_variant,non_coding_transcript_variant,3_prime_UTR_variant
rs1795700939	genic_downstream_transcript_variant,non_coding_transcript_variant,3_prime_UTR_variant
rs1795701008	genic_downstream_transcript_variant,non_coding_transcript_variant,3_prime_UTR_variant
rs1795701069	genic_downstream_transcript_variant,non_coding_transcript_variant,3_prime_UTR_variant
rs1795701286	genic_downstream_transcript_variant,non_coding_transcript_variant,3_prime_UTR_variant
rs1795702167	genic_downstream_transcript_variant,non_coding_transcript_variant,3_prime_UTR_variant
rs1795702237	genic_downstream_transcript_variant,non_coding_transcript_variant,3_prime_UTR_variant
rs1795702520	genic_downstream_transcript_variant,non_coding_transcript_variant,3_prime_UTR_variant
rs1795702617	genic_downstream_transcript_variant,non_coding_transcript_variant,3_prime_UTR_variant
rs1795702770	genic_downstream_transcript_variant,non_coding_transcript_variant,3_prime_UTR_variant
rs1795703017	genic_downstream_transcript_variant,non_coding_transcript_variant,3_prime_UTR_variant
rs1795703181	genic_downstream_transcript_variant,non_coding_transcript_variant,3_prime_UTR_variant
rs1795703252	genic_downstream_transcript_variant,non_coding_transcript_variant,3_prime_UTR_variant
rs1795703871	genic_downstream_transcript_variant,non_coding_transcript_variant,3_prime_UTR_variant
rs1795703935	genic_downstream_transcript_variant,non_coding_transcript_variant,3_prime_UTR_variant
rs1795704010	genic_downstream_transcript_variant,non_coding_transcript_variant,3_prime_UTR_variant

rs1795704160	genic_downstream_transcript_variant,non_coding_transcript_variant,3_prime_UTR_variant
rs1795704245	genic_downstream_transcript_variant,non_coding_transcript_variant,3_prime_UTR_variant
rs1795704331	genic_downstream_transcript_variant,non_coding_transcript_variant,3_prime_UTR_variant
rs1795704653	genic_downstream_transcript_variant,non_coding_transcript_variant,3_prime_UTR_variant
rs1795704744	genic_downstream_transcript_variant,non_coding_transcript_variant,3_prime_UTR_variant
rs1795705369	genic_downstream_transcript_variant,non_coding_transcript_variant,3_prime_UTR_variant
rs1795705602	genic_downstream_transcript_variant,non_coding_transcript_variant,3_prime_UTR_variant
rs1795705674	genic_downstream_transcript_variant,non_coding_transcript_variant,3_prime_UTR_variant
rs1795705751	genic_downstream_transcript_variant,non_coding_transcript_variant,3_prime_UTR_variant
rs1795706424	genic_downstream_transcript_variant,non_coding_transcript_variant,3_prime_UTR_variant
rs1795706495	genic_downstream_transcript_variant,non_coding_transcript_variant,3_prime_UTR_variant
rs1795706713	genic_downstream_transcript_variant,non_coding_transcript_variant,3_prime_UTR_variant
rs1795706802	genic_downstream_transcript_variant,non_coding_transcript_variant,3_prime_UTR_variant
rs1795706869	genic_downstream_transcript_variant,non_coding_transcript_variant,3_prime_UTR_variant
rs1795707031	genic_downstream_transcript_variant,non_coding_transcript_variant,3_prime_UTR_variant
rs1795707100	genic_downstream_transcript_variant,non_coding_transcript_variant,3_prime_UTR_variant
rs1795707231	genic_downstream_transcript_variant,non_coding_transcript_variant,3_prime_UTR_variant
rs1795707303	genic_downstream_transcript_variant,non_coding_transcript_variant,3_prime_UTR_variant
rs1795707907	genic_downstream_transcript_variant,non_coding_transcript_variant,3_prime_UTR_variant
rs1795708123	genic_downstream_transcript_variant,non_coding_transcript_variant,3_prime_UTR_variant
rs1795708491	genic_downstream_transcript_variant,non_coding_transcript_variant,3_prime_UTR_variant
rs1795708851	genic_downstream_transcript_variant,non_coding_transcript_variant,3_prime_UTR_variant
rs1795709794	genic_downstream_transcript_variant,non_coding_transcript_variant,3_prime_UTR_variant
rs1795709965	genic_downstream_transcript_variant,non_coding_transcript_variant,3_prime_UTR_variant
rs1795710035	genic_downstream_transcript_variant,non_coding_transcript_variant,3_prime_UTR_variant
rs1795710172	genic_downstream_transcript_variant,non_coding_transcript_variant,3_prime_UTR_variant
rs1795710384	genic_downstream_transcript_variant,non_coding_transcript_variant,3_prime_UTR_variant
rs1795710535	genic_downstream_transcript_variant,non_coding_transcript_variant,3_prime_UTR_variant
rs1795710882	genic_downstream_transcript_variant,non_coding_transcript_variant,3_prime_UTR_variant
rs1795710956	genic_downstream_transcript_variant,non_coding_transcript_variant,3_prime_UTR_variant
rs1795711528	genic_downstream_transcript_variant,non_coding_transcript_variant,3_prime_UTR_variant
rs1795711767	genic_downstream_transcript_variant,non_coding_transcript_variant,3_prime_UTR_variant
rs1795712170	genic_downstream_transcript_variant,non_coding_transcript_variant,3_prime_UTR_variant
rs1795712330	genic_downstream_transcript_variant,non_coding_transcript_variant,3_prime_UTR_variant
rs1795712684	genic_downstream_transcript_variant,non_coding_transcript_variant,3_prime_UTR_variant
rs1795712787	genic_downstream_transcript_variant,non_coding_transcript_variant,3_prime_UTR_variant
rs1795712972	genic_downstream_transcript_variant,non_coding_transcript_variant,3_prime_UTR_variant
rs1795713112	genic_downstream_transcript_variant,non_coding_transcript_variant,3_prime_UTR_variant
rs1795713181	genic_downstream_transcript_variant,non_coding_transcript_variant,3_prime_UTR_variant
rs1795713338	genic_downstream_transcript_variant,non_coding_transcript_variant,3_prime_UTR_variant
rs1795713432	genic_downstream_transcript_variant,non_coding_transcript_variant,3_prime_UTR_variant
rs1795713540	genic_downstream_transcript_variant,non_coding_transcript_variant,3_prime_UTR_variant
rs1795713867	genic_downstream_transcript_variant,non_coding_transcript_variant,3_prime_UTR_variant
rs1795713942	genic_downstream_transcript_variant,non_coding_transcript_variant,3_prime_UTR_variant
rs1795714740	genic_downstream_transcript_variant,non_coding_transcript_variant,3_prime_UTR_variant
rs1795714822	genic_downstream_transcript_variant,non_coding_transcript_variant,3_prime_UTR_variant
rs1795714894	genic_downstream_transcript_variant,non_coding_transcript_variant,3_prime_UTR_variant
rs1795714975	genic_downstream_transcript_variant,non_coding_transcript_variant,3_prime_UTR_variant
rs1795715049	genic_downstream_transcript_variant,non_coding_transcript_variant,3_prime_UTR_variant
rs1795715193	genic_downstream_transcript_variant,non_coding_transcript_variant,3_prime_UTR_variant
rs1795716043	genic_downstream_transcript_variant,non_coding_transcript_variant,3_prime_UTR_variant
rs1795716376	genic_downstream_transcript_variant,non_coding_transcript_variant,3_prime_UTR_variant
rs1795716476	genic_downstream_transcript_variant,non_coding_transcript_variant,3_prime_UTR_variant
rs1795716563	genic_downstream_transcript_variant,non_coding_transcript_variant,3_prime_UTR_variant
rs1795716790	genic_downstream_transcript_variant,non_coding_transcript_variant,3_prime_UTR_variant
rs1795716859	genic_downstream_transcript_variant,non_coding_transcript_variant,3_prime_UTR_variant
rs1795717092	genic_downstream_transcript_variant,non_coding_transcript_variant,3_prime_UTR_variant
rs1795717875	genic_downstream_transcript_variant,non_coding_transcript_variant,3_prime_UTR_variant
rs1795718104	genic_downstream_transcript_variant,non_coding_transcript_variant,3_prime_UTR_variant
rs1795718218	genic_downstream_transcript_variant,non_coding_transcript_variant,3_prime_UTR_variant
rs1795718528	genic_downstream_transcript_variant,non_coding_transcript_variant,3_prime_UTR_variant
rs1795718765	genic_downstream_transcript_variant,non_coding_transcript_variant,3_prime_UTR_variant
rs1795718843	genic_downstream_transcript_variant,non_coding_transcript_variant,3_prime_UTR_variant
rs1795719607	genic_downstream_transcript_variant,non_coding_transcript_variant,3_prime_UTR_variant
rs1795719737	genic_downstream_transcript_variant,non_coding_transcript_variant,3_prime_UTR_variant
rs1795720891	genic_downstream_transcript_variant,non_coding_transcript_variant,3_prime_UTR_variant
rs1795720975	genic_downstream_transcript_variant,non_coding_transcript_variant,3_prime_UTR_variant
rs1795721331	genic_downstream_transcript_variant,non_coding_transcript_variant,3_prime_UTR_variant
rs1795722095	genic_downstream_transcript_variant,non_coding_transcript_variant,3_prime_UTR_variant
rs1795722503	genic_downstream_transcript_variant,non_coding_transcript_variant,3_prime_UTR_variant
rs1795722560	genic_downstream_transcript_variant,non_coding_transcript_variant,3_prime_UTR_variant
rs1795722634	genic_downstream_transcript_variant,non_coding_transcript_variant,3_prime_UTR_variant
rs1795722860	genic_downstream_transcript_variant,non_coding_transcript_variant,3_prime_UTR_variant
rs1795723032	genic_downstream_transcript_variant,non_coding_transcript_variant,3_prime_UTR_variant

rs1795723263	genic_downstream_transcript_variant,non_coding_transcript_variant,3_prime_UTR_variant
rs1795723407	genic_downstream_transcript_variant,non_coding_transcript_variant,3_prime_UTR_variant
rs1795723500	genic_downstream_transcript_variant,non_coding_transcript_variant,3_prime_UTR_variant
rs1795723661	genic_downstream_transcript_variant,non_coding_transcript_variant,3_prime_UTR_variant
rs1795723815	genic_downstream_transcript_variant,non_coding_transcript_variant,3_prime_UTR_variant
rs1795723886	genic_downstream_transcript_variant,non_coding_transcript_variant,3_prime_UTR_variant
rs1795723976	genic_downstream_transcript_variant,non_coding_transcript_variant,3_prime_UTR_variant
rs1795724056	genic_downstream_transcript_variant,non_coding_transcript_variant,3_prime_UTR_variant
rs1795724560	genic_downstream_transcript_variant,non_coding_transcript_variant,3_prime_UTR_variant
rs1795724682	genic_downstream_transcript_variant,non_coding_transcript_variant,3_prime_UTR_variant
rs1795724960	genic_downstream_transcript_variant,non_coding_transcript_variant,3_prime_UTR_variant
rs1795725067	genic_downstream_transcript_variant,non_coding_transcript_variant,3_prime_UTR_variant
rs1795725172	genic_downstream_transcript_variant,non_coding_transcript_variant,3_prime_UTR_variant
rs1795726179	genic_downstream_transcript_variant,non_coding_transcript_variant,3_prime_UTR_variant
rs1795726396	genic_downstream_transcript_variant,non_coding_transcript_variant,3_prime_UTR_variant
rs1795727341	genic_downstream_transcript_variant,non_coding_transcript_variant,3_prime_UTR_variant
rs1795728069	genic_downstream_transcript_variant,non_coding_transcript_variant,3_prime_UTR_variant
rs1795728183	genic_downstream_transcript_variant,non_coding_transcript_variant,3_prime_UTR_variant
rs1795728567	genic_downstream_transcript_variant,non_coding_transcript_variant,3_prime_UTR_variant
rs1795729013	genic_downstream_transcript_variant,non_coding_transcript_variant,3_prime_UTR_variant
rs1795729350	genic_downstream_transcript_variant,non_coding_transcript_variant,3_prime_UTR_variant
rs1795729779	genic_downstream_transcript_variant,non_coding_transcript_variant,3_prime_UTR_variant
rs1795729922	genic_downstream_transcript_variant,non_coding_transcript_variant,3_prime_UTR_variant
rs1795730140	genic_downstream_transcript_variant,non_coding_transcript_variant,3_prime_UTR_variant
rs1795730261	genic_downstream_transcript_variant,non_coding_transcript_variant,3_prime_UTR_variant
rs1795730739	genic_downstream_transcript_variant,non_coding_transcript_variant,3_prime_UTR_variant
rs1795730829	genic_downstream_transcript_variant,non_coding_transcript_variant,3_prime_UTR_variant
rs1795731143	genic_downstream_transcript_variant,non_coding_transcript_variant,3_prime_UTR_variant
rs1795731327	genic_downstream_transcript_variant,non_coding_transcript_variant,3_prime_UTR_variant
rs1795731625	genic_downstream_transcript_variant,non_coding_transcript_variant,3_prime_UTR_variant
rs1795731701	genic_downstream_transcript_variant,non_coding_transcript_variant,3_prime_UTR_variant
rs1795731787	genic_downstream_transcript_variant,non_coding_transcript_variant,3_prime_UTR_variant
rs1795731871	genic_downstream_transcript_variant,non_coding_transcript_variant,3_prime_UTR_variant
rs1795731955	genic_downstream_transcript_variant,non_coding_transcript_variant,3_prime_UTR_variant
rs1795732032	genic_downstream_transcript_variant,non_coding_transcript_variant,3_prime_UTR_variant
rs1795732106	genic_downstream_transcript_variant,non_coding_transcript_variant,3_prime_UTR_variant
rs1795732187	genic_downstream_transcript_variant,non_coding_transcript_variant,3_prime_UTR_variant
rs1795732255	genic_downstream_transcript_variant,non_coding_transcript_variant,3_prime_UTR_variant
rs1795732334	genic_downstream_transcript_variant,non_coding_transcript_variant,3_prime_UTR_variant
rs1795732417	genic_downstream_transcript_variant,non_coding_transcript_variant,3_prime_UTR_variant
rs1795732582	genic_downstream_transcript_variant,non_coding_transcript_variant,3_prime_UTR_variant
rs1795733190	genic_downstream_transcript_variant,non_coding_transcript_variant,3_prime_UTR_variant
rs1795733281	genic_downstream_transcript_variant,non_coding_transcript_variant,3_prime_UTR_variant
rs1795733357	genic_downstream_transcript_variant,non_coding_transcript_variant,3_prime_UTR_variant
rs1795733917	genic_downstream_transcript_variant,non_coding_transcript_variant,3_prime_UTR_variant
rs1795734151	genic_downstream_transcript_variant,non_coding_transcript_variant,3_prime_UTR_variant
rs1795734220	genic_downstream_transcript_variant,non_coding_transcript_variant,3_prime_UTR_variant
rs1795734371	genic_downstream_transcript_variant,non_coding_transcript_variant,3_prime_UTR_variant
rs1795734685	genic_downstream_transcript_variant,non_coding_transcript_variant,3_prime_UTR_variant
rs1795734763	genic_downstream_transcript_variant,non_coding_transcript_variant,3_prime_UTR_variant
rs1795734834	genic_downstream_transcript_variant,non_coding_transcript_variant,3_prime_UTR_variant
rs1795734902	genic_downstream_transcript_variant,non_coding_transcript_variant,3_prime_UTR_variant
rs1795735495	genic_downstream_transcript_variant,non_coding_transcript_variant,3_prime_UTR_variant
rs1795735663	genic_downstream_transcript_variant,non_coding_transcript_variant,3_prime_UTR_variant
rs1795736315	genic_downstream_transcript_variant,non_coding_transcript_variant,3_prime_UTR_variant
rs1795736393	genic_downstream_transcript_variant,non_coding_transcript_variant,3_prime_UTR_variant
rs1795736471	genic_downstream_transcript_variant,non_coding_transcript_variant,3_prime_UTR_variant
rs1795736549	genic_downstream_transcript_variant,non_coding_transcript_variant,3_prime_UTR_variant
rs1795737202	genic_downstream_transcript_variant,non_coding_transcript_variant,3_prime_UTR_variant
rs1795737460	genic_downstream_transcript_variant,non_coding_transcript_variant,3_prime_UTR_variant
rs1795737535	genic_downstream_transcript_variant,non_coding_transcript_variant,3_prime_UTR_variant
rs1795737680	genic_downstream_transcript_variant,non_coding_transcript_variant,3_prime_UTR_variant
rs1795737760	genic_downstream_transcript_variant,non_coding_transcript_variant,3_prime_UTR_variant
rs1795738066	genic_downstream_transcript_variant,non_coding_transcript_variant,3_prime_UTR_variant
rs1795738157	genic_downstream_transcript_variant,non_coding_transcript_variant,3_prime_UTR_variant
rs1795738311	genic_downstream_transcript_variant,non_coding_transcript_variant,3_prime_UTR_variant
rs1795738566	genic_downstream_transcript_variant,non_coding_transcript_variant,3_prime_UTR_variant
rs1795738657	genic_downstream_transcript_variant,non_coding_transcript_variant,3_prime_UTR_variant
rs1795738745	genic_downstream_transcript_variant,non_coding_transcript_variant,3_prime_UTR_variant
rs1795739160	genic_downstream_transcript_variant,non_coding_transcript_variant,3_prime_UTR_variant
rs1795739303	genic_downstream_transcript_variant,non_coding_transcript_variant,3_prime_UTR_variant
rs1795739941	genic_downstream_transcript_variant,non_coding_transcript_variant,3_prime_UTR_variant
rs1795740036	downstream_transcript_variant,non_coding_transcript_variant,3_prime_UTR_variant,genic_downstream_transcript_variant
rs1795740198	downstream_transcript_variant,non_coding_transcript_variant,3_prime_UTR_variant,genic_downstream_transcript_variant

rs1795740498	downstream_transcript_variant,500B_downstream_variant
rs1795740585	downstream_transcript_variant,500B_downstream_variant
rs1795740687	downstream_transcript_variant,500B_downstream_variant
rs1795740961	downstream_transcript_variant,500B_downstream_variant
rs1795741395	downstream_transcript_variant,500B_downstream_variant
rs1795741577	downstream_transcript_variant,500B_downstream_variant
rs1795741649	downstream_transcript_variant,500B_downstream_variant
rs1795741861	downstream_transcript_variant,500B_downstream_variant
rs1795742580	downstream_transcript_variant,500B_downstream_variant
rs1795742668	downstream_transcript_variant,500B_downstream_variant
rs1795742747	downstream_transcript_variant,500B_downstream_variant
rs1795743319	downstream_transcript_variant,500B_downstream_variant
rs1795743655	downstream_transcript_variant,500B_downstream_variant
rs1795743762	downstream_transcript_variant,500B_downstream_variant
rs1795743870	downstream_transcript_variant,500B_downstream_variant
rs1795744066	downstream_transcript_variant,500B_downstream_variant
rs1795744276	downstream_transcript_variant,500B_downstream_variant
rs1795744365	downstream_transcript_variant,500B_downstream_variant
rs1795744745	downstream_transcript_variant,500B_downstream_variant
rs1795744847	downstream_transcript_variant,500B_downstream_variant
rs1795744927	downstream_transcript_variant,500B_downstream_variant
rs1795745139	downstream_transcript_variant,500B_downstream_variant
rs1795745215	downstream_transcript_variant,500B_downstream_variant
rs1795746642	downstream_transcript_variant,500B_downstream_variant
rs1795746739	downstream_transcript_variant,500B_downstream_variant
rs1795747969	downstream_transcript_variant,500B_downstream_variant
rs1795748517	downstream_transcript_variant,500B_downstream_variant
rs1795748906	downstream_transcript_variant,500B_downstream_variant
rs1795749862	downstream_transcript_variant,500B_downstream_variant
rs1795750193	downstream_transcript_variant,500B_downstream_variant
rs1795750845	downstream_transcript_variant,500B_downstream_variant
rs1795751041	downstream_transcript_variant,500B_downstream_variant
rs1795751481	downstream_transcript_variant,500B_downstream_variant
rs1795751722	downstream_transcript_variant,500B_downstream_variant
rs1795751891	downstream_transcript_variant,500B_downstream_variant
rs1795751971	downstream_transcript_variant,500B_downstream_variant
rs1795752049	downstream_transcript_variant,500B_downstream_variant
rs1795752209	downstream_transcript_variant,500B_downstream_variant
rs1795752276	downstream_transcript_variant,500B_downstream_variant
rs1795752362	downstream_transcript_variant,500B_downstream_variant
rs1795752461	downstream_transcript_variant,500B_downstream_variant
rs1795752558	downstream_transcript_variant,500B_downstream_variant
rs1795752769	downstream_transcript_variant,500B_downstream_variant
rs1795753006	downstream_transcript_variant,500B_downstream_variant
rs1795753199	downstream_transcript_variant,500B_downstream_variant
rs1795753644	downstream_transcript_variant,500B_downstream_variant
rs1795753731	downstream_transcript_variant,500B_downstream_variant
rs1795753806	downstream_transcript_variant,500B_downstream_variant
rs1795754281	downstream_transcript_variant,500B_downstream_variant
rs1795754701	downstream_transcript_variant,500B_downstream_variant
rs1795754812	downstream_transcript_variant,500B_downstream_variant
rs1795754988	downstream_transcript_variant,500B_downstream_variant
rs1795755630	downstream_transcript_variant,500B_downstream_variant
rs1795755825	downstream_transcript_variant,500B_downstream_variant
rs1795755930	downstream_transcript_variant,500B_downstream_variant
rs1795756012	downstream_transcript_variant,500B_downstream_variant
rs1795756432	downstream_transcript_variant,500B_downstream_variant
rs1795756658	downstream_transcript_variant,500B_downstream_variant
rs1795756754	downstream_transcript_variant,500B_downstream_variant
rs1795756924	downstream_transcript_variant,500B_downstream_variant
rs1795757023	downstream_transcript_variant,500B_downstream_variant
rs1795757109	downstream_transcript_variant,500B_downstream_variant
rs1795757269	downstream_transcript_variant,500B_downstream_variant
rs1795757357	downstream_transcript_variant,500B_downstream_variant
rs1795757687	downstream_transcript_variant,500B_downstream_variant
rs1795757762	downstream_transcript_variant,500B_downstream_variant
rs1795757851	downstream_transcript_variant,500B_downstream_variant
rs1795758176	downstream_transcript_variant,500B_downstream_variant
rs1795934707	genic_upstream_transcript_variant,2KB_upstream_variant,upstream_transcript_variant,intron_variant
rs1795935424	genic_upstream_transcript_variant,2KB_upstream_variant,upstream_transcript_variant,intron_variant
rs1795935978	genic_upstream_transcript_variant,2KB_upstream_variant,upstream_transcript_variant,intron_variant
rs1795936060	genic_upstream_transcript_variant,2KB_upstream_variant,upstream_transcript_variant,intron_variant
rs1795936139	genic_upstream_transcript_variant,2KB_upstream_variant,upstream_transcript_variant,intron_variant
rs1795936500	genic_upstream_transcript_variant,2KB_upstream_variant,upstream_transcript_variant,intron_variant

rs1795936762	genic_upstream_transcript_variant,2KB_upstream_variant,upstream_transcript_variant,intron_variant
rs1795937175	genic_upstream_transcript_variant,2KB_upstream_variant,upstream_transcript_variant,intron_variant
rs1795937343	genic_upstream_transcript_variant,2KB_upstream_variant,upstream_transcript_variant,intron_variant
rs1795937586	genic_upstream_transcript_variant,2KB_upstream_variant,upstream_transcript_variant,intron_variant
rs1795937798	genic_upstream_transcript_variant,2KB_upstream_variant,upstream_transcript_variant,intron_variant
rs1795937886	genic_upstream_transcript_variant,2KB_upstream_variant,upstream_transcript_variant,intron_variant
rs1795938019	genic_upstream_transcript_variant,2KB_upstream_variant,upstream_transcript_variant,intron_variant
rs1795938330	genic_upstream_transcript_variant,2KB_upstream_variant,upstream_transcript_variant,intron_variant
rs1795938390	genic_upstream_transcript_variant,2KB_upstream_variant,upstream_transcript_variant,intron_variant
rs1795938491	genic_upstream_transcript_variant,2KB_upstream_variant,upstream_transcript_variant,intron_variant
rs1795938742	genic_upstream_transcript_variant,2KB_upstream_variant,upstream_transcript_variant,intron_variant
rs1795938847	genic_upstream_transcript_variant,2KB_upstream_variant,upstream_transcript_variant,intron_variant
rs1795938959	genic_upstream_transcript_variant,2KB_upstream_variant,upstream_transcript_variant,intron_variant
rs1795939090	genic_upstream_transcript_variant,2KB_upstream_variant,upstream_transcript_variant,intron_variant
rs1795939147	genic_upstream_transcript_variant,2KB_upstream_variant,upstream_transcript_variant,intron_variant
rs1795939197	genic_upstream_transcript_variant,2KB_upstream_variant,upstream_transcript_variant,intron_variant
rs1795939586	genic_upstream_transcript_variant,2KB_upstream_variant,upstream_transcript_variant,intron_variant
rs1795939684	genic_upstream_transcript_variant,2KB_upstream_variant,upstream_transcript_variant,intron_variant
rs1795940319	genic_upstream_transcript_variant,2KB_upstream_variant,upstream_transcript_variant,intron_variant
rs1795940410	genic_upstream_transcript_variant,2KB_upstream_variant,upstream_transcript_variant,intron_variant
rs1795940501	genic_upstream_transcript_variant,2KB_upstream_variant,upstream_transcript_variant,intron_variant
rs1795940836	genic_upstream_transcript_variant,2KB_upstream_variant,upstream_transcript_variant,intron_variant
rs1795941185	genic_upstream_transcript_variant,2KB_upstream_variant,upstream_transcript_variant,intron_variant
rs1795941624	genic_upstream_transcript_variant,2KB_upstream_variant,upstream_transcript_variant,intron_variant
rs1795942339	genic_upstream_transcript_variant,2KB_upstream_variant,upstream_transcript_variant,intron_variant
rs1795942448	genic_upstream_transcript_variant,2KB_upstream_variant,upstream_transcript_variant,intron_variant
rs1795943000	genic_upstream_transcript_variant,2KB_upstream_variant,upstream_transcript_variant,intron_variant
rs1795943631	genic_upstream_transcript_variant,2KB_upstream_variant,upstream_transcript_variant,intron_variant
rs1795943722	genic_upstream_transcript_variant,2KB_upstream_variant,upstream_transcript_variant,intron_variant
rs1795943895	genic_upstream_transcript_variant,2KB_upstream_variant,upstream_transcript_variant,intron_variant
rs1795944188	genic_upstream_transcript_variant,2KB_upstream_variant,upstream_transcript_variant,intron_variant
rs1795944336	genic_upstream_transcript_variant,2KB_upstream_variant,upstream_transcript_variant,intron_variant
rs1795944622	genic_upstream_transcript_variant,2KB_upstream_variant,upstream_transcript_variant,intron_variant
rs1795944760	genic_upstream_transcript_variant,2KB_upstream_variant,upstream_transcript_variant,intron_variant
rs1795945084	genic_upstream_transcript_variant,2KB_upstream_variant,upstream_transcript_variant,intron_variant
rs1795945754	genic_upstream_transcript_variant,2KB_upstream_variant,upstream_transcript_variant,intron_variant
rs1795945808	genic_upstream_transcript_variant,2KB_upstream_variant,upstream_transcript_variant,intron_variant
rs1795945958	genic_upstream_transcript_variant,2KB_upstream_variant,upstream_transcript_variant,intron_variant
rs1795946147	genic_upstream_transcript_variant,2KB_upstream_variant,upstream_transcript_variant,intron_variant
rs1795946375	genic_upstream_transcript_variant,2KB_upstream_variant,upstream_transcript_variant,intron_variant
rs1795946915	genic_upstream_transcript_variant,2KB_upstream_variant,upstream_transcript_variant,intron_variant
rs1795946969	genic_upstream_transcript_variant,2KB_upstream_variant,upstream_transcript_variant,intron_variant
rs1795947117	genic_upstream_transcript_variant,2KB_upstream_variant,upstream_transcript_variant,intron_variant
rs1795947326	genic_upstream_transcript_variant,2KB_upstream_variant,upstream_transcript_variant,intron_variant
rs1795947475	genic_upstream_transcript_variant,2KB_upstream_variant,upstream_transcript_variant,intron_variant
rs1795947546	genic_upstream_transcript_variant,2KB_upstream_variant,upstream_transcript_variant,intron_variant
rs1795947668	genic_upstream_transcript_variant,2KB_upstream_variant,upstream_transcript_variant,intron_variant
rs1795947791	genic_upstream_transcript_variant,2KB_upstream_variant,upstream_transcript_variant,intron_variant
rs1795947977	genic_upstream_transcript_variant,2KB_upstream_variant,upstream_transcript_variant,intron_variant
rs1795948099	genic_upstream_transcript_variant,2KB_upstream_variant,upstream_transcript_variant,intron_variant
rs1795948340	genic_upstream_transcript_variant,2KB_upstream_variant,upstream_transcript_variant,intron_variant
rs1795948624	genic_upstream_transcript_variant,2KB_upstream_variant,upstream_transcript_variant,intron_variant
rs1795948725	genic_upstream_transcript_variant,2KB_upstream_variant,upstream_transcript_variant,intron_variant
rs1795949351	genic_upstream_transcript_variant,2KB_upstream_variant,upstream_transcript_variant,intron_variant
rs1795950141	genic_upstream_transcript_variant,2KB_upstream_variant,upstream_transcript_variant,intron_variant
rs1795950250	genic_upstream_transcript_variant,2KB_upstream_variant,upstream_transcript_variant,intron_variant
rs1795950513	genic_upstream_transcript_variant,2KB_upstream_variant,upstream_transcript_variant,intron_variant
rs1795950914	genic_upstream_transcript_variant,2KB_upstream_variant,upstream_transcript_variant,intron_variant
rs1795951918	genic_upstream_transcript_variant,2KB_upstream_variant,upstream_transcript_variant,intron_variant
rs1795951976	genic_upstream_transcript_variant,2KB_upstream_variant,upstream_transcript_variant,intron_variant
rs1795952196	genic_upstream_transcript_variant,2KB_upstream_variant,upstream_transcript_variant,intron_variant
rs1795952259	genic_upstream_transcript_variant,2KB_upstream_variant,upstream_transcript_variant,intron_variant
rs1795952319	genic_upstream_transcript_variant,2KB_upstream_variant,upstream_transcript_variant,intron_variant
rs1795952379	genic_upstream_transcript_variant,2KB_upstream_variant,upstream_transcript_variant,intron_variant
rs1795952433	genic_upstream_transcript_variant,2KB_upstream_variant,upstream_transcript_variant,intron_variant
rs1795952488	genic_upstream_transcript_variant,2KB_upstream_variant,upstream_transcript_variant,intron_variant
rs1795952721	genic_upstream_transcript_variant,2KB_upstream_variant,upstream_transcript_variant,intron_variant
rs1795952776	genic_upstream_transcript_variant,2KB_upstream_variant,upstream_transcript_variant,intron_variant
rs1795956074	genic_upstream_transcript_variant,2KB_upstream_variant,upstream_transcript_variant,intron_variant
rs1795956201	genic_upstream_transcript_variant,2KB_upstream_variant,upstream_transcript_variant,intron_variant
rs1795956510	genic_upstream_transcript_variant,2KB_upstream_variant,upstream_transcript_variant,intron_variant
rs1795957076	genic_upstream_transcript_variant,2KB_upstream_variant,upstream_transcript_variant,intron_variant
rs1795957140	genic_upstream_transcript_variant,2KB_upstream_variant,upstream_transcript_variant,intron_variant
rs1795957400	genic_upstream_transcript_variant,2KB_upstream_variant,upstream_transcript_variant,intron_variant

rs1795957757	genic_upstream_transcript_variant,2KB_upstream_variant,upstream_transcript_variant,intron_variant
rs1795957892	genic_upstream_transcript_variant,2KB_upstream_variant,upstream_transcript_variant,intron_variant
rs1795957949	genic_upstream_transcript_variant,2KB_upstream_variant,upstream_transcript_variant,intron_variant
rs1795957999	genic_upstream_transcript_variant,2KB_upstream_variant,upstream_transcript_variant,intron_variant
rs1795958074	genic_upstream_transcript_variant,2KB_upstream_variant,upstream_transcript_variant,intron_variant
rs1795958206	genic_upstream_transcript_variant,2KB_upstream_variant,upstream_transcript_variant,intron_variant
rs1795958335	genic_upstream_transcript_variant,2KB_upstream_variant,upstream_transcript_variant,intron_variant
rs1795958386	genic_upstream_transcript_variant,2KB_upstream_variant,upstream_transcript_variant,intron_variant
rs1795958681	genic_upstream_transcript_variant,2KB_upstream_variant,upstream_transcript_variant,intron_variant
rs1795958907	genic_upstream_transcript_variant,2KB_upstream_variant,upstream_transcript_variant,intron_variant
rs1795959348	genic_upstream_transcript_variant,2KB_upstream_variant,upstream_transcript_variant,intron_variant
rs1795959653	genic_upstream_transcript_variant,2KB_upstream_variant,upstream_transcript_variant,intron_variant
rs1795959751	genic_upstream_transcript_variant,2KB_upstream_variant,upstream_transcript_variant,intron_variant
rs1795959865	genic_upstream_transcript_variant,2KB_upstream_variant,upstream_transcript_variant,intron_variant
rs1795961146	genic_upstream_transcript_variant,2KB_upstream_variant,upstream_transcript_variant,intron_variant
rs1795961550	genic_upstream_transcript_variant,2KB_upstream_variant,upstream_transcript_variant,intron_variant
rs1795961650	genic_upstream_transcript_variant,2KB_upstream_variant,upstream_transcript_variant,intron_variant
rs1795961757	genic_upstream_transcript_variant,2KB_upstream_variant,upstream_transcript_variant,intron_variant
rs1795961859	genic_upstream_transcript_variant,2KB_upstream_variant,upstream_transcript_variant,intron_variant
rs1795962403	genic_upstream_transcript_variant,2KB_upstream_variant,upstream_transcript_variant,intron_variant
rs1795962706	genic_upstream_transcript_variant,2KB_upstream_variant,upstream_transcript_variant,intron_variant
rs1795962826	genic_upstream_transcript_variant,2KB_upstream_variant,upstream_transcript_variant,intron_variant
rs1795963324	genic_upstream_transcript_variant,2KB_upstream_variant,upstream_transcript_variant,intron_variant
rs1795963535	genic_upstream_transcript_variant,2KB_upstream_variant,upstream_transcript_variant,intron_variant
rs1795963602	genic_upstream_transcript_variant,2KB_upstream_variant,upstream_transcript_variant,intron_variant
rs1795963725	genic_upstream_transcript_variant,2KB_upstream_variant,upstream_transcript_variant,intron_variant
rs1795963783	genic_upstream_transcript_variant,2KB_upstream_variant,upstream_transcript_variant,intron_variant
rs1795963923	genic_upstream_transcript_variant,2KB_upstream_variant,upstream_transcript_variant,intron_variant
rs1795964467	genic_upstream_transcript_variant,2KB_upstream_variant,upstream_transcript_variant,intron_variant
rs1795964779	genic_upstream_transcript_variant,2KB_upstream_variant,upstream_transcript_variant,intron_variant
rs1795964849	genic_upstream_transcript_variant,2KB_upstream_variant,upstream_transcript_variant,intron_variant
rs1795964910	genic_upstream_transcript_variant,2KB_upstream_variant,upstream_transcript_variant,intron_variant
rs1795964983	genic_upstream_transcript_variant,2KB_upstream_variant,upstream_transcript_variant,intron_variant
rs1795965175	genic_upstream_transcript_variant,2KB_upstream_variant,upstream_transcript_variant,intron_variant
rs1795965310	genic_upstream_transcript_variant,2KB_upstream_variant,upstream_transcript_variant,intron_variant
rs1795965380	genic_upstream_transcript_variant,2KB_upstream_variant,upstream_transcript_variant,intron_variant
rs1795965442	genic_upstream_transcript_variant,2KB_upstream_variant,upstream_transcript_variant,intron_variant
rs1795965491	genic_upstream_transcript_variant,2KB_upstream_variant,upstream_transcript_variant,intron_variant
rs1795965639	genic_upstream_transcript_variant,2KB_upstream_variant,upstream_transcript_variant,intron_variant
rs1795966064	genic_upstream_transcript_variant,2KB_upstream_variant,upstream_transcript_variant,intron_variant
rs1795966145	genic_upstream_transcript_variant,2KB_upstream_variant,upstream_transcript_variant,intron_variant
rs1795966207	genic_upstream_transcript_variant,2KB_upstream_variant,upstream_transcript_variant,intron_variant
rs1795966347	genic_upstream_transcript_variant,2KB_upstream_variant,upstream_transcript_variant,intron_variant
rs1795966547	genic_upstream_transcript_variant,2KB_upstream_variant,upstream_transcript_variant,intron_variant
rs1795966688	genic_upstream_transcript_variant,2KB_upstream_variant,upstream_transcript_variant,intron_variant
rs1795966901	genic_upstream_transcript_variant,2KB_upstream_variant,upstream_transcript_variant,intron_variant
rs1795966957	genic_upstream_transcript_variant,2KB_upstream_variant,upstream_transcript_variant,intron_variant
rs1795967284	genic_upstream_transcript_variant,2KB_upstream_variant,upstream_transcript_variant,intron_variant
rs1795967358	genic_upstream_transcript_variant,2KB_upstream_variant,upstream_transcript_variant,intron_variant
rs1795967610	genic_upstream_transcript_variant,2KB_upstream_variant,upstream_transcript_variant,intron_variant
rs1795967673	genic_upstream_transcript_variant,2KB_upstream_variant,upstream_transcript_variant,intron_variant
rs1795967811	genic_upstream_transcript_variant,2KB_upstream_variant,upstream_transcript_variant,intron_variant
rs1795967877	genic_upstream_transcript_variant,2KB_upstream_variant,upstream_transcript_variant,intron_variant
rs1795968139	genic_upstream_transcript_variant,2KB_upstream_variant,upstream_transcript_variant,intron_variant
rs1795968355	genic_upstream_transcript_variant,2KB_upstream_variant,upstream_transcript_variant,intron_variant
rs1795969686	genic_upstream_transcript_variant,2KB_upstream_variant,upstream_transcript_variant,intron_variant
rs1795969982	genic_upstream_transcript_variant,2KB_upstream_variant,upstream_transcript_variant,intron_variant
rs1795970498	genic_upstream_transcript_variant,2KB_upstream_variant,upstream_transcript_variant,intron_variant
rs1795970735	genic_upstream_transcript_variant,2KB_upstream_variant,upstream_transcript_variant,intron_variant
rs1795970789	genic_upstream_transcript_variant,2KB_upstream_variant,upstream_transcript_variant,intron_variant
rs1795970940	genic_upstream_transcript_variant,2KB_upstream_variant,upstream_transcript_variant,intron_variant
rs1795971068	genic_upstream_transcript_variant,2KB_upstream_variant,upstream_transcript_variant,intron_variant
rs1795971140	genic_upstream_transcript_variant,2KB_upstream_variant,upstream_transcript_variant,intron_variant
rs1795971211	genic_upstream_transcript_variant,2KB_upstream_variant,upstream_transcript_variant,intron_variant
rs1795971270	genic_upstream_transcript_variant,2KB_upstream_variant,upstream_transcript_variant,intron_variant
rs1795971341	genic_upstream_transcript_variant,2KB_upstream_variant,upstream_transcript_variant,intron_variant
rs1795971467	genic_upstream_transcript_variant,2KB_upstream_variant,upstream_transcript_variant,intron_variant
rs1795971543	genic_upstream_transcript_variant,2KB_upstream_variant,upstream_transcript_variant,intron_variant
rs1795971615	genic_upstream_transcript_variant,2KB_upstream_variant,upstream_transcript_variant,intron_variant
rs1795971670	genic_upstream_transcript_variant,2KB_upstream_variant,upstream_transcript_variant,intron_variant
rs1795971804	genic_upstream_transcript_variant,2KB_upstream_variant,upstream_transcript_variant,intron_variant
rs1795971921	genic_upstream_transcript_variant,2KB_upstream_variant,upstream_transcript_variant,intron_variant
rs1795971984	genic_upstream_transcript_variant,2KB_upstream_variant,upstream_transcript_variant,intron_variant
rs1795972749	genic_upstream_transcript_variant,2KB_upstream_variant,upstream_transcript_variant,intron_variant

rs1795972957	genic_upstream_transcript_variant,2KB_upstream_variant,upstream_transcript_variant,intron_variant
rs1795973594	genic_upstream_transcript_variant,2KB_upstream_variant,upstream_transcript_variant,intron_variant
rs1795973654	genic_upstream_transcript_variant,2KB_upstream_variant,upstream_transcript_variant,intron_variant
rs1795973904	genic_upstream_transcript_variant,2KB_upstream_variant,upstream_transcript_variant,intron_variant
rs1795974094	genic_upstream_transcript_variant,2KB_upstream_variant,upstream_transcript_variant,intron_variant
rs1795974522	genic_upstream_transcript_variant,2KB_upstream_variant,upstream_transcript_variant,intron_variant
rs1795974781	genic_upstream_transcript_variant,2KB_upstream_variant,upstream_transcript_variant,intron_variant
rs1795974851	genic_upstream_transcript_variant,2KB_upstream_variant,upstream_transcript_variant,intron_variant
rs1795974911	genic_upstream_transcript_variant,2KB_upstream_variant,upstream_transcript_variant,intron_variant
rs1795975276	genic_upstream_transcript_variant,2KB_upstream_variant,upstream_transcript_variant,intron_variant
rs1795975616	genic_upstream_transcript_variant,2KB_upstream_variant,upstream_transcript_variant,intron_variant
rs1795975995	genic_upstream_transcript_variant,2KB_upstream_variant,upstream_transcript_variant,intron_variant
rs1795976078	genic_upstream_transcript_variant,2KB_upstream_variant,upstream_transcript_variant,intron_variant
rs1795976203	genic_upstream_transcript_variant,2KB_upstream_variant,upstream_transcript_variant,intron_variant
rs1795976311	genic_upstream_transcript_variant,2KB_upstream_variant,upstream_transcript_variant,intron_variant
rs1795976365	genic_upstream_transcript_variant,2KB_upstream_variant,upstream_transcript_variant,intron_variant
rs1795976599	genic_upstream_transcript_variant,2KB_upstream_variant,upstream_transcript_variant,intron_variant
rs1795976936	genic_upstream_transcript_variant,2KB_upstream_variant,upstream_transcript_variant,intron_variant
rs1795977173	genic_upstream_transcript_variant,2KB_upstream_variant,upstream_transcript_variant,intron_variant
rs1795978055	genic_upstream_transcript_variant,2KB_upstream_variant,upstream_transcript_variant,intron_variant
rs1795978166	genic_upstream_transcript_variant,2KB_upstream_variant,upstream_transcript_variant,intron_variant
rs1795978224	genic_upstream_transcript_variant,2KB_upstream_variant,upstream_transcript_variant,intron_variant
rs1795978285	genic_upstream_transcript_variant,2KB_upstream_variant,upstream_transcript_variant,intron_variant
rs1795978345	genic_upstream_transcript_variant,2KB_upstream_variant,upstream_transcript_variant,intron_variant
rs1795978437	genic_upstream_transcript_variant,2KB_upstream_variant,upstream_transcript_variant,intron_variant
rs1795978568	genic_upstream_transcript_variant,2KB_upstream_variant,upstream_transcript_variant,intron_variant
rs1795978623	genic_upstream_transcript_variant,2KB_upstream_variant,upstream_transcript_variant,intron_variant
rs1795978975	genic_upstream_transcript_variant,2KB_upstream_variant,upstream_transcript_variant,intron_variant
rs1795979025	genic_upstream_transcript_variant,2KB_upstream_variant,upstream_transcript_variant,intron_variant
rs1795979183	genic_upstream_transcript_variant,2KB_upstream_variant,upstream_transcript_variant,intron_variant
rs1795979340	genic_upstream_transcript_variant,2KB_upstream_variant,upstream_transcript_variant,intron_variant
rs1795979392	genic_upstream_transcript_variant,2KB_upstream_variant,upstream_transcript_variant,intron_variant
rs1795979514	genic_upstream_transcript_variant,2KB_upstream_variant,upstream_transcript_variant,intron_variant
rs1795979633	genic_upstream_transcript_variant,2KB_upstream_variant,upstream_transcript_variant,intron_variant
rs1795979744	genic_upstream_transcript_variant,2KB_upstream_variant,upstream_transcript_variant,intron_variant
rs1795979984	genic_upstream_transcript_variant,2KB_upstream_variant,upstream_transcript_variant,intron_variant
rs1795980175	genic_upstream_transcript_variant,2KB_upstream_variant,upstream_transcript_variant,intron_variant
rs1795980472	genic_upstream_transcript_variant,2KB_upstream_variant,upstream_transcript_variant,intron_variant
rs1795980607	genic_upstream_transcript_variant,2KB_upstream_variant,upstream_transcript_variant,intron_variant
rs1795980737	genic_upstream_transcript_variant,2KB_upstream_variant,upstream_transcript_variant,intron_variant
rs1795980921	genic_upstream_transcript_variant,2KB_upstream_variant,upstream_transcript_variant,intron_variant
rs1795980981	genic_upstream_transcript_variant,2KB_upstream_variant,upstream_transcript_variant,intron_variant
rs1795981179	genic_upstream_transcript_variant,2KB_upstream_variant,upstream_transcript_variant,intron_variant
rs1795981370	genic_upstream_transcript_variant,2KB_upstream_variant,upstream_transcript_variant,intron_variant
rs1795981613	genic_upstream_transcript_variant,2KB_upstream_variant,upstream_transcript_variant,intron_variant
rs1795981982	genic_upstream_transcript_variant,2KB_upstream_variant,upstream_transcript_variant,intron_variant
rs1795982130	genic_upstream_transcript_variant,2KB_upstream_variant,upstream_transcript_variant,intron_variant
rs1795982224	genic_upstream_transcript_variant,2KB_upstream_variant,upstream_transcript_variant,intron_variant
rs1795982307	genic_upstream_transcript_variant,2KB_upstream_variant,upstream_transcript_variant,intron_variant
rs1795982486	genic_upstream_transcript_variant,2KB_upstream_variant,upstream_transcript_variant,intron_variant
rs1795982577	genic_upstream_transcript_variant,2KB_upstream_variant,upstream_transcript_variant,intron_variant
rs1795982676	genic_upstream_transcript_variant,2KB_upstream_variant,upstream_transcript_variant,intron_variant
rs1795983452	genic_upstream_transcript_variant,2KB_upstream_variant,upstream_transcript_variant,intron_variant
rs1795983496	genic_upstream_transcript_variant,2KB_upstream_variant,upstream_transcript_variant,intron_variant
rs1795983721	genic_upstream_transcript_variant,2KB_upstream_variant,upstream_transcript_variant,intron_variant
rs1795984103	genic_upstream_transcript_variant,2KB_upstream_variant,upstream_transcript_variant,intron_variant
rs1795984481	genic_upstream_transcript_variant,2KB_upstream_variant,upstream_transcript_variant,intron_variant
rs1795984704	genic_upstream_transcript_variant,2KB_upstream_variant,upstream_transcript_variant,intron_variant
rs1795984752	genic_upstream_transcript_variant,2KB_upstream_variant,upstream_transcript_variant,intron_variant
rs1795985065	genic_upstream_transcript_variant,2KB_upstream_variant,upstream_transcript_variant,intron_variant
rs1795985650	genic_upstream_transcript_variant,2KB_upstream_variant,upstream_transcript_variant,intron_variant
rs1795985704	genic_upstream_transcript_variant,2KB_upstream_variant,upstream_transcript_variant,intron_variant
rs1795986085	genic_upstream_transcript_variant,2KB_upstream_variant,upstream_transcript_variant,intron_variant
rs1795986163	genic_upstream_transcript_variant,2KB_upstream_variant,upstream_transcript_variant,intron_variant
rs1795986718	genic_upstream_transcript_variant,2KB_upstream_variant,upstream_transcript_variant,intron_variant
rs1795986824	genic_upstream_transcript_variant,2KB_upstream_variant,upstream_transcript_variant,intron_variant
rs1795986880	genic_upstream_transcript_variant,2KB_upstream_variant,upstream_transcript_variant,intron_variant
rs1795986937	genic_upstream_transcript_variant,2KB_upstream_variant,upstream_transcript_variant,intron_variant
rs1795987118	genic_upstream_transcript_variant,2KB_upstream_variant,upstream_transcript_variant,intron_variant
rs1795987229	genic_upstream_transcript_variant,2KB_upstream_variant,upstream_transcript_variant,intron_variant
rs1795987354	genic_upstream_transcript_variant,2KB_upstream_variant,upstream_transcript_variant,intron_variant
rs1795987414	genic_upstream_transcript_variant,2KB_upstream_variant,upstream_transcript_variant,intron_variant
rs1795987709	genic_upstream_transcript_variant,2KB_upstream_variant,upstream_transcript_variant,intron_variant
rs1795987825	genic_upstream_transcript_variant,2KB_upstream_variant,upstream_transcript_variant,intron_variant

rs1795988045	genic_upstream_transcript_variant,2KB_upstream_variant,upstream_transcript_variant,intron_variant
rs1795988097	genic_upstream_transcript_variant,2KB_upstream_variant,upstream_transcript_variant,intron_variant
rs1795988203	genic_upstream_transcript_variant,2KB_upstream_variant,upstream_transcript_variant,intron_variant
rs1795988258	genic_upstream_transcript_variant,2KB_upstream_variant,upstream_transcript_variant,intron_variant
rs1795988441	genic_upstream_transcript_variant,2KB_upstream_variant,upstream_transcript_variant,intron_variant
rs1795988511	genic_upstream_transcript_variant,2KB_upstream_variant,upstream_transcript_variant,intron_variant
rs1795988564	genic_upstream_transcript_variant,2KB_upstream_variant,upstream_transcript_variant,intron_variant
rs1795988657	genic_upstream_transcript_variant,2KB_upstream_variant,upstream_transcript_variant,intron_variant
rs1795988757	genic_upstream_transcript_variant,2KB_upstream_variant,upstream_transcript_variant,intron_variant
rs1795989033	genic_upstream_transcript_variant,2KB_upstream_variant,upstream_transcript_variant,intron_variant
rs1795989206	genic_upstream_transcript_variant,2KB_upstream_variant,upstream_transcript_variant,intron_variant
rs1795989597	genic_upstream_transcript_variant,2KB_upstream_variant,upstream_transcript_variant,intron_variant
rs1795989699	genic_upstream_transcript_variant,2KB_upstream_variant,upstream_transcript_variant,intron_variant
rs1795989785	genic_upstream_transcript_variant,2KB_upstream_variant,upstream_transcript_variant,intron_variant
rs1795989876	genic_upstream_transcript_variant,2KB_upstream_variant,upstream_transcript_variant,intron_variant
rs1795990039	genic_upstream_transcript_variant,2KB_upstream_variant,upstream_transcript_variant,intron_variant
rs1795990139	genic_upstream_transcript_variant,2KB_upstream_variant,upstream_transcript_variant,intron_variant
rs1795990575	genic_upstream_transcript_variant,2KB_upstream_variant,upstream_transcript_variant,intron_variant
rs1795990669	genic_upstream_transcript_variant,2KB_upstream_variant,upstream_transcript_variant,intron_variant
rs1795990743	genic_upstream_transcript_variant,2KB_upstream_variant,upstream_transcript_variant,intron_variant
rs1795991010	genic_upstream_transcript_variant,2KB_upstream_variant,upstream_transcript_variant,intron_variant
rs1795991692	genic_upstream_transcript_variant,2KB_upstream_variant,upstream_transcript_variant,intron_variant
rs1795992973	genic_upstream_transcript_variant,2KB_upstream_variant,upstream_transcript_variant,intron_variant
rs1795993054	genic_upstream_transcript_variant,2KB_upstream_variant,upstream_transcript_variant,intron_variant
rs1795993253	genic_upstream_transcript_variant,2KB_upstream_variant,upstream_transcript_variant,intron_variant
rs1795993358	genic_upstream_transcript_variant,2KB_upstream_variant,upstream_transcript_variant,intron_variant
rs1795993641	genic_upstream_transcript_variant,2KB_upstream_variant,upstream_transcript_variant,intron_variant
rs1795994024	genic_upstream_transcript_variant,2KB_upstream_variant,upstream_transcript_variant,intron_variant
rs1795994189	genic_upstream_transcript_variant,2KB_upstream_variant,upstream_transcript_variant,intron_variant
rs1795994298	genic_upstream_transcript_variant,2KB_upstream_variant,upstream_transcript_variant,intron_variant
rs1795994456	genic_upstream_transcript_variant,2KB_upstream_variant,upstream_transcript_variant,intron_variant
rs1795994690	genic_upstream_transcript_variant,2KB_upstream_variant,upstream_transcript_variant,intron_variant
rs1795994824	genic_upstream_transcript_variant,2KB_upstream_variant,upstream_transcript_variant,intron_variant
rs1795994884	genic_upstream_transcript_variant,2KB_upstream_variant,upstream_transcript_variant,intron_variant
rs1795994952	genic_upstream_transcript_variant,2KB_upstream_variant,upstream_transcript_variant,intron_variant
rs1795995006	genic_upstream_transcript_variant,2KB_upstream_variant,upstream_transcript_variant,intron_variant
rs1795995322	genic_upstream_transcript_variant,2KB_upstream_variant,upstream_transcript_variant,intron_variant
rs1795995532	genic_upstream_transcript_variant,2KB_upstream_variant,upstream_transcript_variant,intron_variant
rs1795995660	genic_upstream_transcript_variant,2KB_upstream_variant,upstream_transcript_variant,intron_variant
rs1795995735	genic_upstream_transcript_variant,2KB_upstream_variant,upstream_transcript_variant,intron_variant
rs1795996601	genic_upstream_transcript_variant,2KB_upstream_variant,upstream_transcript_variant,intron_variant
rs1795996673	genic_upstream_transcript_variant,2KB_upstream_variant,upstream_transcript_variant,intron_variant
rs1795996846	genic_upstream_transcript_variant,2KB_upstream_variant,5_prime_UTR_variant,intron_variant,upstream_transcript_variant
rs1795996953	5_prime_UTR_variant,genic_upstream_transcript_variant,intron_variant,upstream_transcript_variant
rs1795997028	5_prime_UTR_variant,genic_upstream_transcript_variant,intron_variant,upstream_transcript_variant
rs1795997093	5_prime_UTR_variant,genic_upstream_transcript_variant,intron_variant,upstream_transcript_variant
rs1795997166	5_prime_UTR_variant,genic_upstream_transcript_variant,intron_variant,upstream_transcript_variant
rs1795997236	5_prime_UTR_variant,genic_upstream_transcript_variant,intron_variant,upstream_transcript_variant
rs1795997311	5_prime_UTR_variant,genic_upstream_transcript_variant,intron_variant,upstream_transcript_variant
rs1795997759	5_prime_UTR_variant,genic_upstream_transcript_variant,intron_variant,upstream_transcript_variant
rs1795997830	5_prime_UTR_variant,genic_upstream_transcript_variant,intron_variant,upstream_transcript_variant
rs1795997986	5_prime_UTR_variant,genic_upstream_transcript_variant,intron_variant,upstream_transcript_variant
rs1795998058	5_prime_UTR_variant,genic_upstream_transcript_variant,intron_variant,upstream_transcript_variant
rs1795998420	5_prime_UTR_variant,genic_upstream_transcript_variant,intron_variant,upstream_transcript_variant
rs1795998486	5_prime_UTR_variant,genic_upstream_transcript_variant,intron_variant,upstream_transcript_variant
rs1795998653	5_prime_UTR_variant,genic_upstream_transcript_variant,intron_variant,upstream_transcript_variant
rs1795999507	5_prime_UTR_variant,genic_upstream_transcript_variant,intron_variant,upstream_transcript_variant
rs1795999759	5_prime_UTR_variant,genic_upstream_transcript_variant,intron_variant,upstream_transcript_variant
rs1795999968	5_prime_UTR_variant,genic_upstream_transcript_variant,intron_variant,upstream_transcript_variant
rs1796000145	5_prime_UTR_variant,genic_upstream_transcript_variant,intron_variant,upstream_transcript_variant
rs1796000312	5_prime_UTR_variant,genic_upstream_transcript_variant,intron_variant,upstream_transcript_variant
rs1796000687	5_prime_UTR_variant,genic_upstream_transcript_variant,intron_variant,upstream_transcript_variant
rs1796000854	5_prime_UTR_variant,genic_upstream_transcript_variant,intron_variant,upstream_transcript_variant
rs1796000945	5_prime_UTR_variant,genic_upstream_transcript_variant,intron_variant,upstream_transcript_variant
rs1796001007	5_prime_UTR_variant,genic_upstream_transcript_variant,intron_variant,upstream_transcript_variant
rs1796001084	5_prime_UTR_variant,genic_upstream_transcript_variant,intron_variant,upstream_transcript_variant
rs1796001369	5_prime_UTR_variant,genic_upstream_transcript_variant,intron_variant,upstream_transcript_variant
rs1796001438	5_prime_UTR_variant,genic_upstream_transcript_variant,intron_variant,upstream_transcript_variant
rs1796001741	5_prime_UTR_variant,genic_upstream_transcript_variant,intron_variant,upstream_transcript_variant
rs1796002012	5_prime_UTR_variant,genic_upstream_transcript_variant,intron_variant,upstream_transcript_variant
rs1796002162	5_prime_UTR_variant,genic_upstream_transcript_variant,intron_variant,upstream_transcript_variant
rs1796002233	5_prime_UTR_variant,genic_upstream_transcript_variant,intron_variant,upstream_transcript_variant
rs1796002470	5_prime_UTR_variant,genic_upstream_transcript_variant,intron_variant,upstream_transcript_variant
rs1796002562	5_prime_UTR_variant,genic_upstream_transcript_variant,intron_variant,upstream_transcript_variant

rs1796002824	5_prime_UTR_variant,genic_upstream_transcript_variant,intron_variant,upstream_transcript_variant
rs1796002920	5_prime_UTR_variant,genic_upstream_transcript_variant,intron_variant,upstream_transcript_variant
rs1796003682	5_prime_UTR_variant,genic_upstream_transcript_variant,intron_variant,upstream_transcript_variant
rs1796003852	5_prime_UTR_variant,genic_upstream_transcript_variant,intron_variant,upstream_transcript_variant
rs1796004034	5_prime_UTR_variant,genic_upstream_transcript_variant,intron_variant,upstream_transcript_variant
rs1796004130	5_prime_UTR_variant,genic_upstream_transcript_variant,intron_variant,upstream_transcript_variant
rs1796004301	5_prime_UTR_variant,genic_upstream_transcript_variant,intron_variant,upstream_transcript_variant
rs1796004388	5_prime_UTR_variant,genic_upstream_transcript_variant,intron_variant,upstream_transcript_variant
rs1796004644	5_prime_UTR_variant,genic_upstream_transcript_variant,intron_variant,upstream_transcript_variant
rs1796004739	5_prime_UTR_variant,genic_upstream_transcript_variant,intron_variant,upstream_transcript_variant
rs1796005245	5_prime_UTR_variant,genic_upstream_transcript_variant,non_coding_transcript_variant,intron_variant
rs1796005347	5_prime_UTR_variant,genic_upstream_transcript_variant,non_coding_transcript_variant,intron_variant
rs1796006612	5_prime_UTR_variant,genic_upstream_transcript_variant,non_coding_transcript_variant,intron_variant
rs1796007054	5_prime_UTR_variant,genic_upstream_transcript_variant,non_coding_transcript_variant,intron_variant
rs1796007142	5_prime_UTR_variant,genic_upstream_transcript_variant,non_coding_transcript_variant,intron_variant
rs1796007334	5_prime_UTR_variant,genic_upstream_transcript_variant,non_coding_transcript_variant,intron_variant
rs1796007430	5_prime_UTR_variant,genic_upstream_transcript_variant,non_coding_transcript_variant,intron_variant
rs1796007691	5_prime_UTR_variant,genic_upstream_transcript_variant,non_coding_transcript_variant,intron_variant
rs1796008035	5_prime_UTR_variant,genic_upstream_transcript_variant,non_coding_transcript_variant,intron_variant
rs1796008207	5_prime_UTR_variant,genic_upstream_transcript_variant,non_coding_transcript_variant,intron_variant
rs1796008901	5_prime_UTR_variant,genic_upstream_transcript_variant,non_coding_transcript_variant,intron_variant
rs1796009214	5_prime_UTR_variant,genic_upstream_transcript_variant,non_coding_transcript_variant,intron_variant
rs1796009596	5_prime_UTR_variant,genic_upstream_transcript_variant,non_coding_transcript_variant,intron_variant
rs1796009689	5_prime_UTR_variant,genic_upstream_transcript_variant,non_coding_transcript_variant,intron_variant
rs1796009868	5_prime_UTR_variant,genic_upstream_transcript_variant,non_coding_transcript_variant,intron_variant
rs1796010164	5_prime_UTR_variant,genic_upstream_transcript_variant,non_coding_transcript_variant,intron_variant
rs1796010473	5_prime_UTR_variant,genic_upstream_transcript_variant,non_coding_transcript_variant,intron_variant
rs1796010665	5_prime_UTR_variant,genic_upstream_transcript_variant,non_coding_transcript_variant,intron_variant
rs1796010865	5_prime_UTR_variant,genic_upstream_transcript_variant,non_coding_transcript_variant,intron_variant
rs1796011993	5_prime_UTR_variant,genic_upstream_transcript_variant,non_coding_transcript_variant,intron_variant
rs1796012083	5_prime_UTR_variant,genic_upstream_transcript_variant,non_coding_transcript_variant,intron_variant
rs1796012428	5_prime_UTR_variant,genic_upstream_transcript_variant,non_coding_transcript_variant,intron_variant
rs1796012538	5_prime_UTR_variant,genic_upstream_transcript_variant,non_coding_transcript_variant,intron_variant
rs1796012714	5_prime_UTR_variant,genic_upstream_transcript_variant,non_coding_transcript_variant,intron_variant
rs1796012802	5_prime_UTR_variant,genic_upstream_transcript_variant,non_coding_transcript_variant,intron_variant
rs1796013096	5_prime_UTR_variant,genic_upstream_transcript_variant,non_coding_transcript_variant,intron_variant
rs1796013252	5_prime_UTR_variant,genic_upstream_transcript_variant,non_coding_transcript_variant,intron_variant
rs1796013528	genic_upstream_transcript_variant,intron_variant
rs1796013612	genic_upstream_transcript_variant,intron_variant
rs1796013842	genic_upstream_transcript_variant,intron_variant
rs1796014047	genic_upstream_transcript_variant,intron_variant
rs1796014151	genic_upstream_transcript_variant,intron_variant
rs1796014260	genic_upstream_transcript_variant,intron_variant
rs1796014447	genic_upstream_transcript_variant,intron_variant
rs1796014548	genic_upstream_transcript_variant,intron_variant
rs1796015185	genic_upstream_transcript_variant,intron_variant
rs1796015294	genic_upstream_transcript_variant,intron_variant
rs1796015395	genic_upstream_transcript_variant,intron_variant
rs1796015822	genic_upstream_transcript_variant,intron_variant
rs1796015923	genic_upstream_transcript_variant,intron_variant
rs1796015987	genic_upstream_transcript_variant,intron_variant
rs1796016257	genic_upstream_transcript_variant,intron_variant
rs1796016380	genic_upstream_transcript_variant,intron_variant
rs1796016475	genic_upstream_transcript_variant,intron_variant
rs1796017151	genic_upstream_transcript_variant,intron_variant
rs1796017231	genic_upstream_transcript_variant,intron_variant
rs1796017481	genic_upstream_transcript_variant,intron_variant
rs1796018027	genic_upstream_transcript_variant,intron_variant
rs1796018641	genic_upstream_transcript_variant,intron_variant
rs1796018737	genic_upstream_transcript_variant,intron_variant
rs1796019228	genic_upstream_transcript_variant,intron_variant
rs1796019329	genic_upstream_transcript_variant,intron_variant
rs1796020002	genic_upstream_transcript_variant,intron_variant
rs1796020870	genic_upstream_transcript_variant,intron_variant
rs1796021222	genic_upstream_transcript_variant,intron_variant
rs1796021986	genic_upstream_transcript_variant,intron_variant
rs1796022089	genic_upstream_transcript_variant,intron_variant
rs1796022188	genic_upstream_transcript_variant,intron_variant
rs1796022425	genic_upstream_transcript_variant,intron_variant
rs1796022516	genic_upstream_transcript_variant,intron_variant
rs1796022605	genic_upstream_transcript_variant,intron_variant
rs1796022892	genic_upstream_transcript_variant,intron_variant
rs1796023005	genic_upstream_transcript_variant,intron_variant
rs1796023099	genic_upstream_transcript_variant,intron_variant

rs1796023190	genic_upstream_transcript_variant,intron_variant
rs1796023401	genic_upstream_transcript_variant,intron_variant
rs1796023627	genic_upstream_transcript_variant,intron_variant
rs1796023810	genic_upstream_transcript_variant,intron_variant
rs1796023981	genic_upstream_transcript_variant,intron_variant
rs1796024247	genic_upstream_transcript_variant,intron_variant
rs1796024631	genic_upstream_transcript_variant,intron_variant
rs1796024823	genic_upstream_transcript_variant,intron_variant
rs1796025202	genic_upstream_transcript_variant,intron_variant
rs1796025378	genic_upstream_transcript_variant,intron_variant
rs1796025549	genic_upstream_transcript_variant,intron_variant
rs1796025857	genic_upstream_transcript_variant,intron_variant
rs1796025936	genic_upstream_transcript_variant,intron_variant
rs1796026284	genic_upstream_transcript_variant,intron_variant
rs1796026466	genic_upstream_transcript_variant,intron_variant
rs1796026652	genic_upstream_transcript_variant,intron_variant
rs1796026948	genic_upstream_transcript_variant,intron_variant
rs1796027033	genic_upstream_transcript_variant,intron_variant
rs1796027228	genic_upstream_transcript_variant,intron_variant
rs1796027334	genic_upstream_transcript_variant,intron_variant
rs1796027420	genic_upstream_transcript_variant,intron_variant
rs1796027505	genic_upstream_transcript_variant,intron_variant
rs1796027924	genic_upstream_transcript_variant,intron_variant
rs1796028330	genic_upstream_transcript_variant,intron_variant
rs1796028811	genic_upstream_transcript_variant,intron_variant
rs1796029046	genic_upstream_transcript_variant,intron_variant
rs1796030397	genic_upstream_transcript_variant,intron_variant
rs1796030687	genic_upstream_transcript_variant,intron_variant
rs1796030987	genic_upstream_transcript_variant,intron_variant
rs1796031752	genic_upstream_transcript_variant,intron_variant
rs1796032797	genic_upstream_transcript_variant,intron_variant
rs1796033079	genic_upstream_transcript_variant,intron_variant
rs1796033392	genic_upstream_transcript_variant,intron_variant
rs1796033523	genic_upstream_transcript_variant,intron_variant
rs1796033627	genic_upstream_transcript_variant,intron_variant
rs1796034276	genic_upstream_transcript_variant,intron_variant
rs1796034735	genic_upstream_transcript_variant,intron_variant
rs1796036086	genic_upstream_transcript_variant,intron_variant
rs1796036598	genic_upstream_transcript_variant,intron_variant
rs1796036787	genic_upstream_transcript_variant,intron_variant
rs1796037296	genic_upstream_transcript_variant,intron_variant
rs1796037395	genic_upstream_transcript_variant,intron_variant
rs1796037488	genic_upstream_transcript_variant,intron_variant
rs1796038010	genic_upstream_transcript_variant,intron_variant
rs1796038331	genic_upstream_transcript_variant,intron_variant
rs1796038640	genic_upstream_transcript_variant,intron_variant
rs1796038852	genic_upstream_transcript_variant,intron_variant
rs1796039070	genic_upstream_transcript_variant,intron_variant
rs1796039240	genic_upstream_transcript_variant,intron_variant
rs1796039801	genic_upstream_transcript_variant,intron_variant
rs1796040648	genic_upstream_transcript_variant,intron_variant
rs1796041070	genic_upstream_transcript_variant,intron_variant
rs1796041424	genic_upstream_transcript_variant,intron_variant
rs1796041512	genic_upstream_transcript_variant,intron_variant
rs1796041955	genic_upstream_transcript_variant,intron_variant
rs1796042807	genic_upstream_transcript_variant,intron_variant
rs1796043374	genic_upstream_transcript_variant,intron_variant
rs1796043578	genic_upstream_transcript_variant,intron_variant
rs1796043830	genic_upstream_transcript_variant,intron_variant
rs1796044127	genic_upstream_transcript_variant,intron_variant
rs1796044645	genic_upstream_transcript_variant,intron_variant
rs1796044857	genic_upstream_transcript_variant,intron_variant
rs1796045656	genic_upstream_transcript_variant,intron_variant
rs1796046141	genic_upstream_transcript_variant,intron_variant
rs1796046758	genic_upstream_transcript_variant,intron_variant
rs1796046868	genic_upstream_transcript_variant,intron_variant
rs1796047070	genic_upstream_transcript_variant,intron_variant
rs1796047209	genic_upstream_transcript_variant,intron_variant
rs1796047395	genic_upstream_transcript_variant,intron_variant
rs1796047605	genic_upstream_transcript_variant,intron_variant
rs1796048868	genic_upstream_transcript_variant,intron_variant
rs1796049026	genic_upstream_transcript_variant,intron_variant
rs1796049363	genic_upstream_transcript_variant,intron_variant
rs1796050208	genic_upstream_transcript_variant,intron_variant

rs1796050950	genic_upstream_transcript_variant,intron_variant
rs1796051227	genic_upstream_transcript_variant,intron_variant
rs1796051393	genic_upstream_transcript_variant,intron_variant
rs1796051559	genic_upstream_transcript_variant,intron_variant
rs1796052289	genic_upstream_transcript_variant,intron_variant
rs1796052445	genic_upstream_transcript_variant,intron_variant
rs1796052988	genic_upstream_transcript_variant,intron_variant
rs1796053166	genic_upstream_transcript_variant,intron_variant
rs1796053579	genic_upstream_transcript_variant,intron_variant
rs1796054423	genic_upstream_transcript_variant,intron_variant
rs1796054577	genic_upstream_transcript_variant,intron_variant
rs1796055262	genic_upstream_transcript_variant,intron_variant
rs1796055426	genic_upstream_transcript_variant,intron_variant
rs1796056173	genic_upstream_transcript_variant,intron_variant
rs1796056826	genic_upstream_transcript_variant,intron_variant
rs1796057101	genic_upstream_transcript_variant,intron_variant
rs1796057594	genic_upstream_transcript_variant,intron_variant
rs1796057710	genic_upstream_transcript_variant,intron_variant
rs1796057834	genic_upstream_transcript_variant,intron_variant
rs1796058155	genic_upstream_transcript_variant,intron_variant
rs1796058897	genic_upstream_transcript_variant,intron_variant
rs1796059099	genic_upstream_transcript_variant,intron_variant
rs1796059183	genic_upstream_transcript_variant,intron_variant
rs1796059277	genic_upstream_transcript_variant,intron_variant
rs1796059933	genic_upstream_transcript_variant,intron_variant
rs1796060048	genic_upstream_transcript_variant,intron_variant
rs1796060137	genic_upstream_transcript_variant,intron_variant
rs1796060527	genic_upstream_transcript_variant,intron_variant
rs1796062324	genic_upstream_transcript_variant,intron_variant
rs1796062889	genic_upstream_transcript_variant,intron_variant
rs1796063313	genic_upstream_transcript_variant,intron_variant
rs1796063399	genic_upstream_transcript_variant,intron_variant
rs1796063497	genic_upstream_transcript_variant,intron_variant
rs1796063828	genic_upstream_transcript_variant,intron_variant
rs1796064366	genic_upstream_transcript_variant,intron_variant
rs1796064697	genic_upstream_transcript_variant,intron_variant
rs1796064869	genic_upstream_transcript_variant,intron_variant
rs1796065202	genic_upstream_transcript_variant,intron_variant
rs1796065286	genic_upstream_transcript_variant,intron_variant
rs1796065384	genic_upstream_transcript_variant,intron_variant
rs1796065490	genic_upstream_transcript_variant,intron_variant
rs1796065673	genic_upstream_transcript_variant,intron_variant
rs1796066069	genic_upstream_transcript_variant,intron_variant
rs1796066169	genic_upstream_transcript_variant,intron_variant
rs1796066734	genic_upstream_transcript_variant,intron_variant
rs1796066840	genic_upstream_transcript_variant,intron_variant
rs1796067590	genic_upstream_transcript_variant,intron_variant
rs1796068274	genic_upstream_transcript_variant,intron_variant
rs1796068692	genic_upstream_transcript_variant,intron_variant
rs1796068808	genic_upstream_transcript_variant,intron_variant
rs1796068908	genic_upstream_transcript_variant,intron_variant
rs1796070322	genic_upstream_transcript_variant,intron_variant
rs1796070420	genic_upstream_transcript_variant,intron_variant
rs1796070498	genic_upstream_transcript_variant,intron_variant
rs1796070595	genic_upstream_transcript_variant,intron_variant
rs1796070686	genic_upstream_transcript_variant,intron_variant
rs1796070776	genic_upstream_transcript_variant,intron_variant
rs1796071652	genic_upstream_transcript_variant,intron_variant
rs1796072425	genic_upstream_transcript_variant,intron_variant
rs1796072525	genic_upstream_transcript_variant,intron_variant
rs1796072758	genic_upstream_transcript_variant,intron_variant
rs1796072902	genic_upstream_transcript_variant,intron_variant
rs1796072999	genic_upstream_transcript_variant,intron_variant
rs1796073191	genic_upstream_transcript_variant,intron_variant
rs1796073284	genic_upstream_transcript_variant,intron_variant
rs1796073500	genic_upstream_transcript_variant,intron_variant
rs1796073724	genic_upstream_transcript_variant,intron_variant
rs1796073832	genic_upstream_transcript_variant,intron_variant
rs1796074224	genic_upstream_transcript_variant,intron_variant
rs1796074331	genic_upstream_transcript_variant,intron_variant
rs1796075722	genic_upstream_transcript_variant,intron_variant
rs1796077093	genic_upstream_transcript_variant,intron_variant
rs1796077222	genic_upstream_transcript_variant,intron_variant
rs1796077525	genic_upstream_transcript_variant,intron_variant

rs1796077734	genic_upstream_transcript_variant,intron_variant
rs1796077834	genic_upstream_transcript_variant,intron_variant
rs1796078030	genic_upstream_transcript_variant,intron_variant
rs1796078258	genic_upstream_transcript_variant,intron_variant
rs1796078393	genic_upstream_transcript_variant,intron_variant
rs1796078684	genic_upstream_transcript_variant,intron_variant
rs1796078808	genic_upstream_transcript_variant,intron_variant
rs1796079419	genic_upstream_transcript_variant,intron_variant
rs1796079514	genic_upstream_transcript_variant,intron_variant
rs1796079609	genic_upstream_transcript_variant,intron_variant
rs1796079694	genic_upstream_transcript_variant,intron_variant
rs1796079794	genic_upstream_transcript_variant,intron_variant
rs1796079892	genic_upstream_transcript_variant,intron_variant
rs1796080118	genic_upstream_transcript_variant,intron_variant
rs1796080229	genic_upstream_transcript_variant,intron_variant
rs1796080333	genic_upstream_transcript_variant,intron_variant
rs1796080880	genic_upstream_transcript_variant,intron_variant
rs1796081770	genic_upstream_transcript_variant,intron_variant
rs1796082203	genic_upstream_transcript_variant,intron_variant
rs1796083464	genic_upstream_transcript_variant,intron_variant
rs1796083583	genic_upstream_transcript_variant,intron_variant
rs1796084607	genic_upstream_transcript_variant,intron_variant
rs1796084715	genic_upstream_transcript_variant,intron_variant
rs1796084822	genic_upstream_transcript_variant,intron_variant
rs1796085152	genic_upstream_transcript_variant,intron_variant
rs1796085536	genic_upstream_transcript_variant,intron_variant
rs1796085639	genic_upstream_transcript_variant,intron_variant
rs1796085858	genic_upstream_transcript_variant,intron_variant
rs1796086374	genic_upstream_transcript_variant,intron_variant
rs1796086471	genic_upstream_transcript_variant,intron_variant
rs1796086750	genic_upstream_transcript_variant,intron_variant
rs1796086952	genic_upstream_transcript_variant,intron_variant
rs1796087387	genic_upstream_transcript_variant,intron_variant
rs1796088074	genic_upstream_transcript_variant,intron_variant
rs1796088176	genic_upstream_transcript_variant,intron_variant
rs1796088299	genic_upstream_transcript_variant,intron_variant
rs1796088863	genic_upstream_transcript_variant,intron_variant
rs1796088967	genic_upstream_transcript_variant,intron_variant
rs1796089187	genic_upstream_transcript_variant,intron_variant
rs1796089305	genic_upstream_transcript_variant,intron_variant
rs1796089549	genic_upstream_transcript_variant,intron_variant
rs1796090386	genic_upstream_transcript_variant,intron_variant
rs1796090502	genic_upstream_transcript_variant,intron_variant
rs1796091547	genic_upstream_transcript_variant,intron_variant
rs1796092210	genic_upstream_transcript_variant,intron_variant
rs1796092334	genic_upstream_transcript_variant,intron_variant
rs1796093247	genic_upstream_transcript_variant,intron_variant
rs1796093990	genic_upstream_transcript_variant,intron_variant
rs1796094164	genic_upstream_transcript_variant,intron_variant
rs1796094950	genic_upstream_transcript_variant,intron_variant
rs1796095927	genic_upstream_transcript_variant,intron_variant
rs1796097205	genic_upstream_transcript_variant,intron_variant
rs1796097413	genic_upstream_transcript_variant,intron_variant
rs1796097512	genic_upstream_transcript_variant,intron_variant
rs1796097620	genic_upstream_transcript_variant,intron_variant
rs1796097831	genic_upstream_transcript_variant,intron_variant
rs1796097960	genic_upstream_transcript_variant,intron_variant
rs1796098181	genic_upstream_transcript_variant,intron_variant
rs1796098595	genic_upstream_transcript_variant,intron_variant
rs1796099469	genic_upstream_transcript_variant,intron_variant
rs1796100211	genic_upstream_transcript_variant,intron_variant
rs1796100354	genic_upstream_transcript_variant,intron_variant
rs1796100458	genic_upstream_transcript_variant,intron_variant
rs1796100693	genic_upstream_transcript_variant,intron_variant
rs1796100794	genic_upstream_transcript_variant,intron_variant
rs1796101265	genic_upstream_transcript_variant,intron_variant
rs1796101520	genic_upstream_transcript_variant,intron_variant
rs1796101846	genic_upstream_transcript_variant,intron_variant
rs1796102429	genic_upstream_transcript_variant,intron_variant
rs1796102529	genic_upstream_transcript_variant,intron_variant
rs1796102752	genic_upstream_transcript_variant,intron_variant
rs1796102978	genic_upstream_transcript_variant,intron_variant
rs1796103300	genic_upstream_transcript_variant,intron_variant
rs1796103404	genic_upstream_transcript_variant,intron_variant

rs1796103654	genic_upstream_transcript_variant,intron_variant
rs1796103823	genic_upstream_transcript_variant,intron_variant
rs1796104602	genic_upstream_transcript_variant,intron_variant
rs1796104763	genic_upstream_transcript_variant,intron_variant
rs1796106755	genic_upstream_transcript_variant,intron_variant
rs1796107072	genic_upstream_transcript_variant,intron_variant
rs1796107223	genic_upstream_transcript_variant,intron_variant
rs1796107554	genic_upstream_transcript_variant,intron_variant
rs1796108052	genic_upstream_transcript_variant,intron_variant
rs1796108348	genic_upstream_transcript_variant,intron_variant
rs1796108691	genic_upstream_transcript_variant,intron_variant
rs1796109480	genic_upstream_transcript_variant,intron_variant
rs1796109579	genic_upstream_transcript_variant,intron_variant
rs1796109686	genic_upstream_transcript_variant,intron_variant
rs1796109789	genic_upstream_transcript_variant,intron_variant
rs1796109967	genic_upstream_transcript_variant,intron_variant
rs1796110168	genic_upstream_transcript_variant,intron_variant
rs1796111359	genic_upstream_transcript_variant,intron_variant
rs1796111469	genic_upstream_transcript_variant,intron_variant
rs1796111575	genic_upstream_transcript_variant,intron_variant
rs1796111697	genic_upstream_transcript_variant,intron_variant
rs1796112245	genic_upstream_transcript_variant,intron_variant
rs1796112356	genic_upstream_transcript_variant,intron_variant
rs1796112461	genic_upstream_transcript_variant,intron_variant
rs1796112571	genic_upstream_transcript_variant,intron_variant
rs1796112896	genic_upstream_transcript_variant,intron_variant
rs1796113484	genic_upstream_transcript_variant,intron_variant
rs1796114031	genic_upstream_transcript_variant,intron_variant
rs1796114862	genic_upstream_transcript_variant,intron_variant
rs1796115073	genic_upstream_transcript_variant,intron_variant
rs1796116167	genic_upstream_transcript_variant,intron_variant
rs1796116935	genic_upstream_transcript_variant,intron_variant
rs1796117303	genic_upstream_transcript_variant,intron_variant
rs1796117724	genic_upstream_transcript_variant,intron_variant
rs1796117810	genic_upstream_transcript_variant,intron_variant
rs1796118169	genic_upstream_transcript_variant,intron_variant
rs1796118285	genic_upstream_transcript_variant,intron_variant
rs1796118631	genic_upstream_transcript_variant,intron_variant
rs1796118733	genic_upstream_transcript_variant,intron_variant
rs1796118832	genic_upstream_transcript_variant,intron_variant
rs1796119051	genic_upstream_transcript_variant,intron_variant
rs1796119984	genic_upstream_transcript_variant,intron_variant
rs1796120327	genic_upstream_transcript_variant,intron_variant
rs1796120432	genic_upstream_transcript_variant,intron_variant
rs1796120684	genic_upstream_transcript_variant,intron_variant
rs1796120787	genic_upstream_transcript_variant,intron_variant
rs1796120982	genic_upstream_transcript_variant,intron_variant
rs1796121206	genic_upstream_transcript_variant,intron_variant
rs1796121291	genic_upstream_transcript_variant,intron_variant
rs1796121401	genic_upstream_transcript_variant,intron_variant
rs1796122389	genic_upstream_transcript_variant,intron_variant
rs1796122699	genic_upstream_transcript_variant,intron_variant
rs1796122797	genic_upstream_transcript_variant,intron_variant
rs1796122997	genic_upstream_transcript_variant,intron_variant
rs1796123087	genic_upstream_transcript_variant,intron_variant
rs1796123197	genic_upstream_transcript_variant,intron_variant
rs1796123924	genic_upstream_transcript_variant,intron_variant
rs1796124433	genic_upstream_transcript_variant,intron_variant
rs1796124536	genic_upstream_transcript_variant,intron_variant
rs1796124967	genic_upstream_transcript_variant,intron_variant
rs1796125057	genic_upstream_transcript_variant,intron_variant
rs1796125145	genic_upstream_transcript_variant,intron_variant
rs1796125566	genic_upstream_transcript_variant,intron_variant
rs1796125929	genic_upstream_transcript_variant,intron_variant
rs1796126541	genic_upstream_transcript_variant,intron_variant
rs1796126763	genic_upstream_transcript_variant,intron_variant
rs1796126968	genic_upstream_transcript_variant,intron_variant
rs1796127474	genic_upstream_transcript_variant,intron_variant
rs1796127871	genic_upstream_transcript_variant,intron_variant
rs1796128910	genic_upstream_transcript_variant,intron_variant
rs1796129062	genic_upstream_transcript_variant,intron_variant
rs1796130848	genic_upstream_transcript_variant,intron_variant
rs1796131007	genic_upstream_transcript_variant,intron_variant
rs1796131163	genic_upstream_transcript_variant,intron_variant

rs1796131621	genic_upstream_transcript_variant,intron_variant
rs1796131788	genic_upstream_transcript_variant,intron_variant
rs1796132198	genic_upstream_transcript_variant,intron_variant
rs1796132553	genic_upstream_transcript_variant,intron_variant
rs1796133345	genic_upstream_transcript_variant,intron_variant
rs1796133553	genic_upstream_transcript_variant,intron_variant
rs1796133654	genic_upstream_transcript_variant,intron_variant
rs1796134018	genic_upstream_transcript_variant,intron_variant
rs1796134310	genic_upstream_transcript_variant,intron_variant
rs1796135183	genic_upstream_transcript_variant,intron_variant
rs1796135298	genic_upstream_transcript_variant,intron_variant
rs1796135704	genic_upstream_transcript_variant,intron_variant
rs1796136622	genic_upstream_transcript_variant,intron_variant
rs1796136855	genic_upstream_transcript_variant,intron_variant
rs1796137211	genic_upstream_transcript_variant,intron_variant
rs1796137301	genic_upstream_transcript_variant,intron_variant
rs1796137416	genic_upstream_transcript_variant,intron_variant
rs1796137509	genic_upstream_transcript_variant,intron_variant
rs1796137825	genic_upstream_transcript_variant,intron_variant
rs1796138170	genic_upstream_transcript_variant,intron_variant
rs1796138590	genic_upstream_transcript_variant,intron_variant
rs1796138699	genic_upstream_transcript_variant,intron_variant
rs1796139320	genic_upstream_transcript_variant,intron_variant
rs1796140129	genic_upstream_transcript_variant,intron_variant
rs1796140835	genic_upstream_transcript_variant,intron_variant
rs1796141320	genic_upstream_transcript_variant,intron_variant
rs1796141533	genic_upstream_transcript_variant,intron_variant
rs1796141810	genic_upstream_transcript_variant,intron_variant
rs1796142109	genic_upstream_transcript_variant,intron_variant
rs1796142961	genic_upstream_transcript_variant,intron_variant
rs1796143426	genic_upstream_transcript_variant,intron_variant
rs1796143669	genic_upstream_transcript_variant,intron_variant
rs1796143912	genic_upstream_transcript_variant,intron_variant
rs1796144398	genic_upstream_transcript_variant,intron_variant
rs1796144509	genic_upstream_transcript_variant,intron_variant
rs1796144576	genic_upstream_transcript_variant,intron_variant
rs1796144785	genic_upstream_transcript_variant,intron_variant
rs1796146262	genic_upstream_transcript_variant,intron_variant
rs1796146601	genic_upstream_transcript_variant,intron_variant
rs1796146795	genic_upstream_transcript_variant,intron_variant
rs1796146892	genic_upstream_transcript_variant,intron_variant
rs1796147685	genic_upstream_transcript_variant,intron_variant
rs1796148315	genic_upstream_transcript_variant,intron_variant
rs1796148421	genic_upstream_transcript_variant,intron_variant
rs1796149528	genic_upstream_transcript_variant,intron_variant
rs1796149616	genic_upstream_transcript_variant,intron_variant
rs1796149989	genic_upstream_transcript_variant,intron_variant
rs1796150223	genic_upstream_transcript_variant,intron_variant
rs1796150422	genic_upstream_transcript_variant,intron_variant
rs1796150520	genic_upstream_transcript_variant,intron_variant
rs1796150613	genic_upstream_transcript_variant,intron_variant
rs1796150946	genic_upstream_transcript_variant,intron_variant
rs1796151260	genic_upstream_transcript_variant,intron_variant
rs1796151479	genic_upstream_transcript_variant,intron_variant
rs1796151583	genic_upstream_transcript_variant,intron_variant
rs1796152693	genic_upstream_transcript_variant,intron_variant
rs1796152927	genic_upstream_transcript_variant,intron_variant
rs1796153022	genic_upstream_transcript_variant,intron_variant
rs1796153239	genic_upstream_transcript_variant,intron_variant
rs1796154667	genic_upstream_transcript_variant,intron_variant
rs1796154873	genic_upstream_transcript_variant,intron_variant
rs1796155067	genic_upstream_transcript_variant,intron_variant
rs1796155166	genic_upstream_transcript_variant,intron_variant
rs1796155373	genic_upstream_transcript_variant,intron_variant
rs1796155546	genic_upstream_transcript_variant,intron_variant
rs1796155650	genic_upstream_transcript_variant,intron_variant
rs1796155764	genic_upstream_transcript_variant,intron_variant
rs1796156760	genic_upstream_transcript_variant,intron_variant
rs1796156860	genic_upstream_transcript_variant,intron_variant
rs1796156951	genic_upstream_transcript_variant,intron_variant
rs1796157051	genic_upstream_transcript_variant,intron_variant
rs1796157280	genic_upstream_transcript_variant,intron_variant
rs1796157389	genic_upstream_transcript_variant,intron_variant
rs1796157593	genic_upstream_transcript_variant,intron_variant

rs1796157683	genic_upstream_transcript_variant,intron_variant
rs1796157781	genic_upstream_transcript_variant,intron_variant
rs1796157888	genic_upstream_transcript_variant,intron_variant
rs1796157977	genic_upstream_transcript_variant,intron_variant
rs1796158271	genic_upstream_transcript_variant,intron_variant
rs1796158459	genic_upstream_transcript_variant,intron_variant
rs1796158559	genic_upstream_transcript_variant,intron_variant
rs1796158936	genic_upstream_transcript_variant,intron_variant
rs1796159292	genic_upstream_transcript_variant,intron_variant
rs1796159386	genic_upstream_transcript_variant,intron_variant
rs1796161031	genic_upstream_transcript_variant,intron_variant
rs1796161243	genic_upstream_transcript_variant,intron_variant
rs1796161330	genic_upstream_transcript_variant,intron_variant
rs1796161428	genic_upstream_transcript_variant,intron_variant
rs1796161524	genic_upstream_transcript_variant,intron_variant
rs1796161803	genic_upstream_transcript_variant,intron_variant
rs1796161907	genic_upstream_transcript_variant,intron_variant
rs1796162361	genic_upstream_transcript_variant,intron_variant
rs1796162554	genic_upstream_transcript_variant,intron_variant
rs1796162654	genic_upstream_transcript_variant,intron_variant
rs1796163361	genic_upstream_transcript_variant,intron_variant
rs1796163466	genic_upstream_transcript_variant,intron_variant
rs1796163559	genic_upstream_transcript_variant,intron_variant
rs1796163668	genic_upstream_transcript_variant,intron_variant
rs1796164204	genic_upstream_transcript_variant,intron_variant
rs1796164296	genic_upstream_transcript_variant,intron_variant
rs1796164402	genic_upstream_transcript_variant,intron_variant
rs1796164522	genic_upstream_transcript_variant,intron_variant
rs1796164620	genic_upstream_transcript_variant,intron_variant
rs1796164845	genic_upstream_transcript_variant,intron_variant
rs1796165493	genic_upstream_transcript_variant,intron_variant
rs1796165699	genic_upstream_transcript_variant,intron_variant
rs1796166232	genic_upstream_transcript_variant,intron_variant
rs1796166446	genic_upstream_transcript_variant,intron_variant
rs1796166548	genic_upstream_transcript_variant,intron_variant
rs1796166659	genic_upstream_transcript_variant,intron_variant
rs1796166752	genic_upstream_transcript_variant,intron_variant
rs1796166862	genic_upstream_transcript_variant,intron_variant
rs1796167224	genic_upstream_transcript_variant,intron_variant
rs1796167357	genic_upstream_transcript_variant,intron_variant
rs1796167658	genic_upstream_transcript_variant,intron_variant
rs1796167754	genic_upstream_transcript_variant,intron_variant
rs1796168592	genic_upstream_transcript_variant,intron_variant
rs1796168691	genic_upstream_transcript_variant,intron_variant
rs1796169012	genic_upstream_transcript_variant,intron_variant
rs1796169121	genic_upstream_transcript_variant,intron_variant
rs1796169230	genic_upstream_transcript_variant,intron_variant
rs1796169327	genic_upstream_transcript_variant,intron_variant
rs1796169680	genic_upstream_transcript_variant,intron_variant
rs1796169788	genic_upstream_transcript_variant,intron_variant
rs1796170832	genic_upstream_transcript_variant,intron_variant
rs1796171145	genic_upstream_transcript_variant,intron_variant
rs1796171337	genic_upstream_transcript_variant,intron_variant
rs1796171461	genic_upstream_transcript_variant,intron_variant
rs1796171564	genic_upstream_transcript_variant,intron_variant
rs1796171751	genic_upstream_transcript_variant,intron_variant
rs1796172047	genic_upstream_transcript_variant,intron_variant
rs1796172583	genic_upstream_transcript_variant,intron_variant
rs1796172880	genic_upstream_transcript_variant,intron_variant
rs1796173154	genic_upstream_transcript_variant,intron_variant
rs1796173346	genic_upstream_transcript_variant,intron_variant
rs1796173662	genic_upstream_transcript_variant,intron_variant
rs1796174005	genic_upstream_transcript_variant,intron_variant
rs1796174198	genic_upstream_transcript_variant,intron_variant
rs1796174286	genic_upstream_transcript_variant,intron_variant
rs1796174389	genic_upstream_transcript_variant,intron_variant
rs1796174662	genic_upstream_transcript_variant,intron_variant
rs1796174847	genic_upstream_transcript_variant,intron_variant
rs1796175165	genic_upstream_transcript_variant,intron_variant
rs1796175404	genic_upstream_transcript_variant,intron_variant
rs1796175730	genic_upstream_transcript_variant,intron_variant
rs1796175934	genic_upstream_transcript_variant,intron_variant
rs1796176146	genic_upstream_transcript_variant,intron_variant
rs1796176426	genic_upstream_transcript_variant,intron_variant

rs1796177060	genic_upstream_transcript_variant,intron_variant
rs1796177160	genic_upstream_transcript_variant,intron_variant
rs1796177258	genic_upstream_transcript_variant,intron_variant
rs1796178372	genic_upstream_transcript_variant,intron_variant
rs1796178762	genic_upstream_transcript_variant,intron_variant
rs1796179046	genic_upstream_transcript_variant,intron_variant
rs1796179476	genic_upstream_transcript_variant,intron_variant
rs1796179730	genic_upstream_transcript_variant,intron_variant
rs1796179914	genic_upstream_transcript_variant,intron_variant
rs1796180222	genic_upstream_transcript_variant,intron_variant
rs1796180720	genic_upstream_transcript_variant,intron_variant
rs1796182180	genic_upstream_transcript_variant,intron_variant
rs1796182277	genic_upstream_transcript_variant,intron_variant
rs1796182392	genic_upstream_transcript_variant,intron_variant
rs1796183710	genic_upstream_transcript_variant,intron_variant
rs1796183960	genic_upstream_transcript_variant,intron_variant
rs1796184353	genic_upstream_transcript_variant,intron_variant
rs1796185024	genic_upstream_transcript_variant,intron_variant
rs1796185233	genic_upstream_transcript_variant,intron_variant
rs1796185873	genic_upstream_transcript_variant,intron_variant
rs1796186095	genic_upstream_transcript_variant,intron_variant
rs1796186931	genic_upstream_transcript_variant,intron_variant
rs1796187252	genic_upstream_transcript_variant,intron_variant
rs1796187457	genic_upstream_transcript_variant,intron_variant
rs1796188464	genic_upstream_transcript_variant,intron_variant
rs1796188720	genic_upstream_transcript_variant,intron_variant
rs1796189300	genic_upstream_transcript_variant,intron_variant
rs1796189543	genic_upstream_transcript_variant,intron_variant
rs1796189767	genic_upstream_transcript_variant,intron_variant
rs1796189964	genic_upstream_transcript_variant,intron_variant
rs1796190194	genic_upstream_transcript_variant,intron_variant
rs1796190564	genic_upstream_transcript_variant,intron_variant
rs1796190792	genic_upstream_transcript_variant,intron_variant
rs1796191032	genic_upstream_transcript_variant,intron_variant
rs1796191154	genic_upstream_transcript_variant,intron_variant
rs1796191251	genic_upstream_transcript_variant,intron_variant
rs1796191365	genic_upstream_transcript_variant,intron_variant
rs1796191759	genic_upstream_transcript_variant,intron_variant
rs1796191868	genic_upstream_transcript_variant,intron_variant
rs1796191978	genic_upstream_transcript_variant,intron_variant
rs1796192251	genic_upstream_transcript_variant,intron_variant
rs1796192700	genic_upstream_transcript_variant,intron_variant
rs1796192804	genic_upstream_transcript_variant,intron_variant
rs1796192999	genic_upstream_transcript_variant,intron_variant
rs1796193288	genic_upstream_transcript_variant,intron_variant
rs1796193385	genic_upstream_transcript_variant,intron_variant
rs1796193493	genic_upstream_transcript_variant,intron_variant
rs1796193598	genic_upstream_transcript_variant,intron_variant
rs1796194174	genic_upstream_transcript_variant,intron_variant
rs1796194270	genic_upstream_transcript_variant,intron_variant
rs1796194727	genic_upstream_transcript_variant,intron_variant
rs1796195265	genic_upstream_transcript_variant,intron_variant
rs1796196109	genic_upstream_transcript_variant,intron_variant
rs1796196210	genic_upstream_transcript_variant,intron_variant
rs1796196317	genic_upstream_transcript_variant,intron_variant
rs1796196872	genic_upstream_transcript_variant,intron_variant
rs1796197084	genic_upstream_transcript_variant,intron_variant
rs1796197366	genic_upstream_transcript_variant,intron_variant
rs1796197869	genic_upstream_transcript_variant,intron_variant
rs1796198286	genic_upstream_transcript_variant,intron_variant
rs1796198806	genic_upstream_transcript_variant,intron_variant
rs1796198900	genic_upstream_transcript_variant,intron_variant
rs1796199368	genic_upstream_transcript_variant,intron_variant
rs1796199572	genic_upstream_transcript_variant,intron_variant
rs1796199659	genic_upstream_transcript_variant,intron_variant
rs1796200001	genic_upstream_transcript_variant,intron_variant
rs1796200108	genic_upstream_transcript_variant,intron_variant
rs1796201268	genic_upstream_transcript_variant,intron_variant
rs1796201375	genic_upstream_transcript_variant,intron_variant
rs1796202086	genic_upstream_transcript_variant,intron_variant
rs1796202191	genic_upstream_transcript_variant,intron_variant
rs1796202292	genic_upstream_transcript_variant,intron_variant
rs1796202408	genic_upstream_transcript_variant,intron_variant
rs1796202618	genic_upstream_transcript_variant,intron_variant

rs1796202842	genic_upstream_transcript_variant,intron_variant
rs1796203138	genic_upstream_transcript_variant,intron_variant
rs1796203679	genic_upstream_transcript_variant,intron_variant
rs1796203969	genic_upstream_transcript_variant,intron_variant
rs1796204791	genic_upstream_transcript_variant,intron_variant
rs1796205339	genic_upstream_transcript_variant,intron_variant
rs1796205579	genic_upstream_transcript_variant,intron_variant
rs1796205677	genic_upstream_transcript_variant,intron_variant
rs1796205777	genic_upstream_transcript_variant,intron_variant
rs1796206236	genic_upstream_transcript_variant,intron_variant
rs1796207412	genic_upstream_transcript_variant,intron_variant
rs1796207847	genic_upstream_transcript_variant,intron_variant
rs1796208095	genic_upstream_transcript_variant,intron_variant
rs1796208206	genic_upstream_transcript_variant,intron_variant
rs1796208675	genic_upstream_transcript_variant,intron_variant
rs1796209123	genic_upstream_transcript_variant,intron_variant
rs1796209333	genic_upstream_transcript_variant,intron_variant
rs1796210017	genic_upstream_transcript_variant,intron_variant
rs1796210132	genic_upstream_transcript_variant,intron_variant
rs1796210230	genic_upstream_transcript_variant,intron_variant
rs1796210635	genic_upstream_transcript_variant,intron_variant
rs1796210845	genic_upstream_transcript_variant,intron_variant
rs1796210934	genic_upstream_transcript_variant,intron_variant
rs1796211039	genic_upstream_transcript_variant,intron_variant
rs1796211964	genic_upstream_transcript_variant,intron_variant
rs1796212165	genic_upstream_transcript_variant,intron_variant
rs1796212590	genic_upstream_transcript_variant,intron_variant
rs1796213362	genic_upstream_transcript_variant,intron_variant
rs1796213598	genic_upstream_transcript_variant,intron_variant
rs1796213722	genic_upstream_transcript_variant,intron_variant
rs1796214024	genic_upstream_transcript_variant,intron_variant
rs1796214339	genic_upstream_transcript_variant,intron_variant
rs1796214459	genic_upstream_transcript_variant,intron_variant
rs1796215424	genic_upstream_transcript_variant,intron_variant
rs1796215610	genic_upstream_transcript_variant,intron_variant
rs1796215806	genic_upstream_transcript_variant,intron_variant
rs1796215909	genic_upstream_transcript_variant,intron_variant
rs1796216222	genic_upstream_transcript_variant,intron_variant
rs1796216668	genic_upstream_transcript_variant,intron_variant
rs1796216758	genic_upstream_transcript_variant,intron_variant
rs1796217134	genic_upstream_transcript_variant,intron_variant
rs1796217600	genic_upstream_transcript_variant,intron_variant
rs1796218237	genic_upstream_transcript_variant,intron_variant
rs1796219017	genic_upstream_transcript_variant,intron_variant
rs1796219253	genic_upstream_transcript_variant,intron_variant
rs1796219483	genic_upstream_transcript_variant,intron_variant
rs1796219831	genic_upstream_transcript_variant,intron_variant
rs1796220256	genic_upstream_transcript_variant,intron_variant
rs1796220939	genic_upstream_transcript_variant,intron_variant
rs1796221804	genic_upstream_transcript_variant,intron_variant
rs1796221979	genic_upstream_transcript_variant,intron_variant
rs1796222088	genic_upstream_transcript_variant,intron_variant
rs1796222193	genic_upstream_transcript_variant,intron_variant
rs1796222300	genic_upstream_transcript_variant,intron_variant
rs1796222404	genic_upstream_transcript_variant,intron_variant
rs1796222634	genic_upstream_transcript_variant,intron_variant
rs1796222771	genic_upstream_transcript_variant,intron_variant
rs1796223010	genic_upstream_transcript_variant,intron_variant
rs1796223210	genic_upstream_transcript_variant,intron_variant
rs1796223672	genic_upstream_transcript_variant,intron_variant
rs1796223775	genic_upstream_transcript_variant,intron_variant
rs1796223870	genic_upstream_transcript_variant,intron_variant
rs1796223966	genic_upstream_transcript_variant,intron_variant
rs1796224432	genic_upstream_transcript_variant,intron_variant
rs1796225344	genic_upstream_transcript_variant,intron_variant
rs1796225871	genic_upstream_transcript_variant,intron_variant
rs1796226049	genic_upstream_transcript_variant,intron_variant
rs1796226589	genic_upstream_transcript_variant,intron_variant
rs1796226779	genic_upstream_transcript_variant,intron_variant
rs1796227075	genic_upstream_transcript_variant,intron_variant
rs1796227632	genic_upstream_transcript_variant,intron_variant
rs1796227715	genic_upstream_transcript_variant,intron_variant
rs1796227817	genic_upstream_transcript_variant,intron_variant
rs1796227919	genic_upstream_transcript_variant,intron_variant

rs1796228500	genic_upstream_transcript_variant,intron_variant
rs1796228637	genic_upstream_transcript_variant,intron_variant
rs1796229045	genic_upstream_transcript_variant,intron_variant
rs1796229140	genic_upstream_transcript_variant,intron_variant
rs1796229610	genic_upstream_transcript_variant,intron_variant
rs1796229830	genic_upstream_transcript_variant,intron_variant
rs1796229916	genic_upstream_transcript_variant,intron_variant
rs1796230014	genic_upstream_transcript_variant,intron_variant
rs1796230440	genic_upstream_transcript_variant,intron_variant
rs1796230531	genic_upstream_transcript_variant,intron_variant
rs1796231026	genic_upstream_transcript_variant,intron_variant
rs1796231520	genic_upstream_transcript_variant,intron_variant
rs1796231628	genic_upstream_transcript_variant,intron_variant
rs1796231921	genic_upstream_transcript_variant,intron_variant
rs1796232026	genic_upstream_transcript_variant,intron_variant
rs1796232592	genic_upstream_transcript_variant,intron_variant
rs1796232889	genic_upstream_transcript_variant,intron_variant
rs1796233294	genic_upstream_transcript_variant,intron_variant
rs1796233636	genic_upstream_transcript_variant,intron_variant
rs1796233730	genic_upstream_transcript_variant,intron_variant
rs1796233847	genic_upstream_transcript_variant,intron_variant
rs1796234218	genic_upstream_transcript_variant,intron_variant
rs1796234396	genic_upstream_transcript_variant,intron_variant
rs1796234512	genic_upstream_transcript_variant,intron_variant
rs1796234644	genic_upstream_transcript_variant,intron_variant
rs1796235115	genic_upstream_transcript_variant,intron_variant
rs1796235312	genic_upstream_transcript_variant,intron_variant
rs1796236126	genic_upstream_transcript_variant,intron_variant
rs1796236854	genic_upstream_transcript_variant,intron_variant
rs1796237872	genic_upstream_transcript_variant,intron_variant
rs1796238405	genic_upstream_transcript_variant,intron_variant
rs1796238596	genic_upstream_transcript_variant,intron_variant
rs1796239122	genic_upstream_transcript_variant,intron_variant
rs1796239421	genic_upstream_transcript_variant,intron_variant
rs1796239917	genic_upstream_transcript_variant,intron_variant
rs1796240624	genic_upstream_transcript_variant,intron_variant
rs1796240852	genic_upstream_transcript_variant,intron_variant
rs1796241044	genic_upstream_transcript_variant,intron_variant
rs1796241143	genic_upstream_transcript_variant,intron_variant
rs1796241236	genic_upstream_transcript_variant,intron_variant
rs1796241325	genic_upstream_transcript_variant,intron_variant
rs1796241429	genic_upstream_transcript_variant,intron_variant
rs1796241831	genic_upstream_transcript_variant,intron_variant
rs1796242150	genic_upstream_transcript_variant,intron_variant
rs1796242243	genic_upstream_transcript_variant,intron_variant
rs1796242547	genic_upstream_transcript_variant,intron_variant
rs1796242972	genic_upstream_transcript_variant,intron_variant
rs1796243118	genic_upstream_transcript_variant,intron_variant
rs1796243416	genic_upstream_transcript_variant,intron_variant
rs1796243803	genic_upstream_transcript_variant,intron_variant
rs1796244232	genic_upstream_transcript_variant,intron_variant
rs1796244524	genic_upstream_transcript_variant,intron_variant
rs1796244718	genic_upstream_transcript_variant,intron_variant
rs1796244819	genic_upstream_transcript_variant,intron_variant
rs1796245335	genic_upstream_transcript_variant,intron_variant
rs1796245411	genic_upstream_transcript_variant,intron_variant
rs1796246536	genic_upstream_transcript_variant,intron_variant
rs1796246816	genic_upstream_transcript_variant,intron_variant
rs1796246903	genic_upstream_transcript_variant,intron_variant
rs1796247534	genic_upstream_transcript_variant,intron_variant
rs1796247728	genic_upstream_transcript_variant,intron_variant
rs1796248013	genic_upstream_transcript_variant,intron_variant
rs1796248336	genic_upstream_transcript_variant,intron_variant
rs1796248424	genic_upstream_transcript_variant,intron_variant
rs1796248662	genic_upstream_transcript_variant,intron_variant
rs1796248750	genic_upstream_transcript_variant,intron_variant
rs1796248848	genic_upstream_transcript_variant,intron_variant
rs1796249031	genic_upstream_transcript_variant,intron_variant
rs1796249202	genic_upstream_transcript_variant,intron_variant
rs1796249299	genic_upstream_transcript_variant,intron_variant
rs1796250302	genic_upstream_transcript_variant,intron_variant
rs1796250404	genic_upstream_transcript_variant,intron_variant
rs1796250502	genic_upstream_transcript_variant,intron_variant
rs1796250613	genic_upstream_transcript_variant,intron_variant

rs1796250725	genic_upstream_transcript_variant,intron_variant
rs1796251415	genic_upstream_transcript_variant,intron_variant
rs1796251502	genic_upstream_transcript_variant,intron_variant
rs1796251603	genic_upstream_transcript_variant,intron_variant
rs1796251908	genic_upstream_transcript_variant,intron_variant
rs1796252288	genic_upstream_transcript_variant,intron_variant
rs1796252568	genic_upstream_transcript_variant,intron_variant
rs1796252928	genic_upstream_transcript_variant,intron_variant
rs1796253245	genic_upstream_transcript_variant,intron_variant
rs1796253679	genic_upstream_transcript_variant,intron_variant
rs1796253768	genic_upstream_transcript_variant,intron_variant
rs1796254034	genic_upstream_transcript_variant,intron_variant
rs1796254115	genic_upstream_transcript_variant,intron_variant
rs1796254429	genic_upstream_transcript_variant,intron_variant
rs1796254625	genic_upstream_transcript_variant,intron_variant
rs1796254716	genic_upstream_transcript_variant,intron_variant
rs1796255029	genic_upstream_transcript_variant,intron_variant
rs1796255142	genic_upstream_transcript_variant,intron_variant
rs1796256348	genic_upstream_transcript_variant,intron_variant
rs1796256456	genic_upstream_transcript_variant,intron_variant
rs1796256715	genic_upstream_transcript_variant,intron_variant
rs1796256821	genic_upstream_transcript_variant,intron_variant
rs1796256926	genic_upstream_transcript_variant,intron_variant
rs1796257129	genic_upstream_transcript_variant,intron_variant
rs1796257952	genic_upstream_transcript_variant,intron_variant
rs1796258063	genic_upstream_transcript_variant,intron_variant
rs1796258148	genic_upstream_transcript_variant,intron_variant
rs1796258255	genic_upstream_transcript_variant,intron_variant
rs1796258432	genic_upstream_transcript_variant,intron_variant
rs1796259787	genic_upstream_transcript_variant,intron_variant
rs1796260094	genic_upstream_transcript_variant,intron_variant
rs1796260188	genic_upstream_transcript_variant,intron_variant
rs1796260436	genic_upstream_transcript_variant,intron_variant
rs1796260796	genic_upstream_transcript_variant,intron_variant
rs1796261100	genic_upstream_transcript_variant,intron_variant
rs1796261245	genic_upstream_transcript_variant,intron_variant
rs1796261394	genic_upstream_transcript_variant,intron_variant
rs1796261543	genic_upstream_transcript_variant,intron_variant
rs1796262269	genic_upstream_transcript_variant,intron_variant
rs1796262362	genic_upstream_transcript_variant,intron_variant
rs1796262651	genic_upstream_transcript_variant,intron_variant
rs1796262763	genic_upstream_transcript_variant,intron_variant
rs1796263678	genic_upstream_transcript_variant,intron_variant
rs1796263768	genic_upstream_transcript_variant,intron_variant
rs1796263864	genic_upstream_transcript_variant,intron_variant
rs1796264262	genic_upstream_transcript_variant,intron_variant
rs1796264358	genic_upstream_transcript_variant,intron_variant
rs1796265133	genic_upstream_transcript_variant,intron_variant
rs1796265294	genic_upstream_transcript_variant,intron_variant
rs1796266598	genic_upstream_transcript_variant,intron_variant
rs1796266878	genic_upstream_transcript_variant,intron_variant
rs1796267041	genic_upstream_transcript_variant,intron_variant
rs1796267142	genic_upstream_transcript_variant,intron_variant
rs1796267574	genic_upstream_transcript_variant,intron_variant
rs1796267855	genic_upstream_transcript_variant,intron_variant
rs1796268272	genic_upstream_transcript_variant,intron_variant
rs1796268636	genic_upstream_transcript_variant,intron_variant
rs1796268844	genic_upstream_transcript_variant,intron_variant
rs1796269390	genic_upstream_transcript_variant,intron_variant
rs1796269613	genic_upstream_transcript_variant,intron_variant
rs1796269701	genic_upstream_transcript_variant,intron_variant
rs1796269893	genic_upstream_transcript_variant,intron_variant
rs1796270114	genic_upstream_transcript_variant,intron_variant
rs1796270216	genic_upstream_transcript_variant,intron_variant
rs1796270328	genic_upstream_transcript_variant,intron_variant
rs1796271294	genic_upstream_transcript_variant,intron_variant
rs1796271802	genic_upstream_transcript_variant,intron_variant
rs1796271950	genic_upstream_transcript_variant,intron_variant
rs1796272061	genic_upstream_transcript_variant,intron_variant
rs1796272852	genic_upstream_transcript_variant,intron_variant
rs1796272946	genic_upstream_transcript_variant,intron_variant
rs1796273154	genic_upstream_transcript_variant,intron_variant
rs1796273631	genic_upstream_transcript_variant,intron_variant
rs1796273720	genic_upstream_transcript_variant,intron_variant

rs1796273913	genic_upstream_transcript_variant,intron_variant
rs1796274657	genic_upstream_transcript_variant,intron_variant
rs1796274760	genic_upstream_transcript_variant,intron_variant
rs1796274940	genic_upstream_transcript_variant,intron_variant
rs1796275091	genic_upstream_transcript_variant,intron_variant
rs1796275275	genic_upstream_transcript_variant,intron_variant
rs1796275607	genic_upstream_transcript_variant,intron_variant
rs1796276209	genic_upstream_transcript_variant,intron_variant
rs1796277046	genic_upstream_transcript_variant,intron_variant
rs1796277966	genic_upstream_transcript_variant,intron_variant
rs1796278083	genic_upstream_transcript_variant,intron_variant
rs1796278415	genic_upstream_transcript_variant,intron_variant
rs1796278864	genic_upstream_transcript_variant,intron_variant
rs1796279465	genic_upstream_transcript_variant,intron_variant
rs1796279681	genic_upstream_transcript_variant,intron_variant
rs1796280119	genic_upstream_transcript_variant,intron_variant
rs1796280216	genic_upstream_transcript_variant,intron_variant
rs1796280332	genic_upstream_transcript_variant,intron_variant
rs1796280670	genic_upstream_transcript_variant,intron_variant
rs1796280982	genic_upstream_transcript_variant,intron_variant
rs1796281108	genic_upstream_transcript_variant,intron_variant
rs1796281768	genic_upstream_transcript_variant,intron_variant
rs1796282607	genic_upstream_transcript_variant,intron_variant
rs1796282853	genic_upstream_transcript_variant,intron_variant
rs1796283179	genic_upstream_transcript_variant,intron_variant
rs1796283561	genic_upstream_transcript_variant,intron_variant
rs1796283916	genic_upstream_transcript_variant,intron_variant
rs1796284364	genic_upstream_transcript_variant,intron_variant
rs1796284458	genic_upstream_transcript_variant,intron_variant
rs1796284935	genic_upstream_transcript_variant,intron_variant
rs1796285056	genic_upstream_transcript_variant,intron_variant
rs1796285174	genic_upstream_transcript_variant,intron_variant
rs1796285281	genic_upstream_transcript_variant,intron_variant
rs1796285380	genic_upstream_transcript_variant,intron_variant
rs1796286222	genic_upstream_transcript_variant,intron_variant
rs1796286538	genic_upstream_transcript_variant,intron_variant
rs1796286759	genic_upstream_transcript_variant,intron_variant
rs1796287569	genic_upstream_transcript_variant,intron_variant
rs1796287673	genic_upstream_transcript_variant,intron_variant
rs1796287818	genic_upstream_transcript_variant,intron_variant
rs1796287948	genic_upstream_transcript_variant,intron_variant
rs1796289560	genic_upstream_transcript_variant,intron_variant
rs1796289772	genic_upstream_transcript_variant,intron_variant
rs1796289964	genic_upstream_transcript_variant,intron_variant
rs1796290093	genic_upstream_transcript_variant,intron_variant
rs1796290526	genic_upstream_transcript_variant,intron_variant
rs1796290850	genic_upstream_transcript_variant,intron_variant
rs1796291063	genic_upstream_transcript_variant,intron_variant
rs1796291165	genic_upstream_transcript_variant,intron_variant
rs1796292000	genic_upstream_transcript_variant,intron_variant
rs1796292105	genic_upstream_transcript_variant,intron_variant
rs1796292214	genic_upstream_transcript_variant,intron_variant
rs1796292311	genic_upstream_transcript_variant,intron_variant
rs1796292531	genic_upstream_transcript_variant,intron_variant
rs1796292631	genic_upstream_transcript_variant,intron_variant
rs1796292735	genic_upstream_transcript_variant,intron_variant
rs1796292816	genic_upstream_transcript_variant,intron_variant
rs1796293016	genic_upstream_transcript_variant,intron_variant
rs1796294082	genic_upstream_transcript_variant,intron_variant
rs1796294601	genic_upstream_transcript_variant,intron_variant
rs1796294694	genic_upstream_transcript_variant,intron_variant
rs1796294802	genic_upstream_transcript_variant,intron_variant
rs1796295651	genic_upstream_transcript_variant,intron_variant
rs1796295817	genic_upstream_transcript_variant,intron_variant
rs1796296322	genic_upstream_transcript_variant,intron_variant
rs1796296481	genic_upstream_transcript_variant,intron_variant
rs1796297453	genic_upstream_transcript_variant,intron_variant
rs1796297796	genic_upstream_transcript_variant,intron_variant
rs1796298149	genic_upstream_transcript_variant,intron_variant
rs1796298800	genic_upstream_transcript_variant,intron_variant
rs1796298959	genic_upstream_transcript_variant,intron_variant
rs1796299268	genic_upstream_transcript_variant,intron_variant
rs1796299423	genic_upstream_transcript_variant,intron_variant
rs1796299640	genic_upstream_transcript_variant,intron_variant

rs1796300058	genic_upstream_transcript_variant,intron_variant
rs1796300243	genic_upstream_transcript_variant,intron_variant
rs1796300423	genic_upstream_transcript_variant,intron_variant
rs1796300682	genic_upstream_transcript_variant,intron_variant
rs1796300902	genic_upstream_transcript_variant,intron_variant
rs1796301021	genic_upstream_transcript_variant,intron_variant
rs1796301198	genic_upstream_transcript_variant,intron_variant
rs1796302978	genic_upstream_transcript_variant,intron_variant
rs1796303111	genic_upstream_transcript_variant,intron_variant
rs1796303383	genic_upstream_transcript_variant,intron_variant
rs1796303530	genic_upstream_transcript_variant,intron_variant
rs1796304639	genic_upstream_transcript_variant,intron_variant
rs1796305018	genic_upstream_transcript_variant,intron_variant
rs1796305986	genic_upstream_transcript_variant,intron_variant
rs1796306071	genic_upstream_transcript_variant,intron_variant
rs1796306450	genic_upstream_transcript_variant,intron_variant
rs1796307300	genic_upstream_transcript_variant,intron_variant
rs1796307603	genic_upstream_transcript_variant,intron_variant
rs1796307711	genic_upstream_transcript_variant,intron_variant
rs1796308089	genic_upstream_transcript_variant,intron_variant
rs1796308660	genic_upstream_transcript_variant,intron_variant
rs1796308755	genic_upstream_transcript_variant,intron_variant
rs1796309311	genic_upstream_transcript_variant,intron_variant
rs1796309506	genic_upstream_transcript_variant,intron_variant
rs1796309705	genic_upstream_transcript_variant,intron_variant
rs1796309894	genic_upstream_transcript_variant,intron_variant
rs1796310207	genic_upstream_transcript_variant,intron_variant
rs1796310669	genic_upstream_transcript_variant,intron_variant
rs1796310769	genic_upstream_transcript_variant,intron_variant
rs1796311414	genic_upstream_transcript_variant,intron_variant
rs1796311504	genic_upstream_transcript_variant,intron_variant
rs1796311919	genic_upstream_transcript_variant,intron_variant
rs1796312424	genic_upstream_transcript_variant,intron_variant
rs1796313107	genic_upstream_transcript_variant,intron_variant
rs1796313461	genic_upstream_transcript_variant,intron_variant
rs1796313564	genic_upstream_transcript_variant,intron_variant
rs1796313753	genic_upstream_transcript_variant,intron_variant
rs1796314120	genic_upstream_transcript_variant,intron_variant
rs1796314770	genic_upstream_transcript_variant,intron_variant
rs1796315400	genic_upstream_transcript_variant,intron_variant
rs1796315498	genic_upstream_transcript_variant,intron_variant
rs1796315593	genic_upstream_transcript_variant,intron_variant
rs1796317140	genic_upstream_transcript_variant,intron_variant
rs1796317240	genic_upstream_transcript_variant,intron_variant
rs1796317714	genic_upstream_transcript_variant,intron_variant
rs1796318307	genic_upstream_transcript_variant,intron_variant
rs1796318464	genic_upstream_transcript_variant,intron_variant
rs1796319424	genic_upstream_transcript_variant,intron_variant
rs1796320042	genic_upstream_transcript_variant,intron_variant
rs1796320329	genic_upstream_transcript_variant,intron_variant
rs1796320473	genic_upstream_transcript_variant,intron_variant
rs1796320625	genic_upstream_transcript_variant,intron_variant
rs1796320803	genic_upstream_transcript_variant,intron_variant
rs1796321126	genic_upstream_transcript_variant,intron_variant
rs1796321434	genic_upstream_transcript_variant,intron_variant
rs1796321725	genic_upstream_transcript_variant,intron_variant
rs1796322474	genic_upstream_transcript_variant,intron_variant
rs1796322822	genic_upstream_transcript_variant,intron_variant
rs1796322978	genic_upstream_transcript_variant,intron_variant
rs1796323326	genic_upstream_transcript_variant,intron_variant
rs1796323762	genic_upstream_transcript_variant,intron_variant
rs1796323886	genic_upstream_transcript_variant,intron_variant
rs1796324357	genic_upstream_transcript_variant,intron_variant
rs1796324493	genic_upstream_transcript_variant,intron_variant
rs1796325114	genic_upstream_transcript_variant,intron_variant
rs1796325239	genic_upstream_transcript_variant,intron_variant
rs1796325678	genic_upstream_transcript_variant,intron_variant
rs1796325928	genic_upstream_transcript_variant,intron_variant
rs1796326459	genic_upstream_transcript_variant,intron_variant
rs1796327089	genic_upstream_transcript_variant,intron_variant
rs1796328099	genic_upstream_transcript_variant,intron_variant
rs1796328180	genic_upstream_transcript_variant,intron_variant
rs1796328849	genic_upstream_transcript_variant,intron_variant
rs1796329322	genic_upstream_transcript_variant,intron_variant

rs1796329592	genic_upstream_transcript_variant,intron_variant
rs1796330237	genic_upstream_transcript_variant,intron_variant
rs1796330374	genic_upstream_transcript_variant,intron_variant
rs1796331319	genic_upstream_transcript_variant,intron_variant
rs1796331476	genic_upstream_transcript_variant,intron_variant
rs1796331778	genic_upstream_transcript_variant,intron_variant
rs1796332247	genic_upstream_transcript_variant,intron_variant
rs1796332689	genic_upstream_transcript_variant,intron_variant
rs1796332986	genic_upstream_transcript_variant,intron_variant
rs1796333255	genic_upstream_transcript_variant,intron_variant
rs1796333375	genic_upstream_transcript_variant,intron_variant
rs1796333654	genic_upstream_transcript_variant,intron_variant
rs1796333797	genic_upstream_transcript_variant,intron_variant
rs1796334139	genic_upstream_transcript_variant,intron_variant
rs1796335195	genic_upstream_transcript_variant,intron_variant
rs1796335354	genic_upstream_transcript_variant,intron_variant
rs1796335692	genic_upstream_transcript_variant,intron_variant
rs1796336355	genic_upstream_transcript_variant,intron_variant
rs1796336649	genic_upstream_transcript_variant,intron_variant
rs1796337944	genic_upstream_transcript_variant,intron_variant
rs1796338100	genic_upstream_transcript_variant,intron_variant
rs1796338236	genic_upstream_transcript_variant,intron_variant
rs1796338759	genic_upstream_transcript_variant,intron_variant
rs1796338913	genic_upstream_transcript_variant,intron_variant
rs1796339751	genic_upstream_transcript_variant,intron_variant
rs1796339839	genic_upstream_transcript_variant,intron_variant
rs1796339930	genic_upstream_transcript_variant,intron_variant
rs1796340135	genic_upstream_transcript_variant,intron_variant
rs1796340322	genic_upstream_transcript_variant,intron_variant
rs1796340431	genic_upstream_transcript_variant,intron_variant
rs1796340547	genic_upstream_transcript_variant,intron_variant
rs1796340894	genic_upstream_transcript_variant,intron_variant
rs1796340983	genic_upstream_transcript_variant,intron_variant
rs1796341437	genic_upstream_transcript_variant,intron_variant
rs1796341541	genic_upstream_transcript_variant,intron_variant
rs1796342090	genic_upstream_transcript_variant,intron_variant
rs1796342300	genic_upstream_transcript_variant,intron_variant
rs1796342394	genic_upstream_transcript_variant,intron_variant
rs1796342778	genic_upstream_transcript_variant,intron_variant
rs1796342913	genic_upstream_transcript_variant,intron_variant
rs1796343154	genic_upstream_transcript_variant,intron_variant
rs1796343264	genic_upstream_transcript_variant,intron_variant
rs1796343744	genic_upstream_transcript_variant,intron_variant
rs1796343922	genic_upstream_transcript_variant,intron_variant
rs1796344023	genic_upstream_transcript_variant,intron_variant
rs1796344450	genic_upstream_transcript_variant,intron_variant
rs1796344537	genic_upstream_transcript_variant,intron_variant
rs1796345082	genic_upstream_transcript_variant,intron_variant
rs1796345173	genic_upstream_transcript_variant,intron_variant
rs1796345743	genic_upstream_transcript_variant,intron_variant
rs1796346503	genic_upstream_transcript_variant,intron_variant
rs1796346948	genic_upstream_transcript_variant,intron_variant
rs1796347254	genic_upstream_transcript_variant,intron_variant
rs1796347559	genic_upstream_transcript_variant,intron_variant
rs1796347733	genic_upstream_transcript_variant,intron_variant
rs1796348619	genic_upstream_transcript_variant,intron_variant
rs1796349073	genic_upstream_transcript_variant,intron_variant
rs1796349972	genic_upstream_transcript_variant,intron_variant
rs1796350179	genic_upstream_transcript_variant,intron_variant
rs1796350290	genic_upstream_transcript_variant,intron_variant
rs1796352407	genic_upstream_transcript_variant,intron_variant
rs1796353138	genic_upstream_transcript_variant,intron_variant
rs1796353558	genic_upstream_transcript_variant,intron_variant
rs1796353969	genic_upstream_transcript_variant,intron_variant
rs1796354450	genic_upstream_transcript_variant,intron_variant
rs1796355348	genic_upstream_transcript_variant,intron_variant
rs1796355824	genic_upstream_transcript_variant,intron_variant
rs1796356028	genic_upstream_transcript_variant,intron_variant
rs1796356258	genic_upstream_transcript_variant,intron_variant
rs1796356338	genic_upstream_transcript_variant,intron_variant
rs1796356614	genic_upstream_transcript_variant,intron_variant
rs1796356789	genic_upstream_transcript_variant,intron_variant
rs1796356941	genic_upstream_transcript_variant,intron_variant
rs1796357345	genic_upstream_transcript_variant,intron_variant

rs1796358502	genic_upstream_transcript_variant,intron_variant
rs1796359098	genic_upstream_transcript_variant,intron_variant
rs1796359268	genic_upstream_transcript_variant,intron_variant
rs1796359452	genic_upstream_transcript_variant,intron_variant
rs1796359699	genic_upstream_transcript_variant,intron_variant
rs1796360067	genic_upstream_transcript_variant,intron_variant
rs1796360341	genic_upstream_transcript_variant,intron_variant
rs1796360424	genic_upstream_transcript_variant,intron_variant
rs1796360746	genic_upstream_transcript_variant,intron_variant
rs1796360983	genic_upstream_transcript_variant,intron_variant
rs1796361370	genic_upstream_transcript_variant,intron_variant
rs1796361470	genic_upstream_transcript_variant,intron_variant
rs1796361572	genic_upstream_transcript_variant,intron_variant
rs1796361794	genic_upstream_transcript_variant,intron_variant
rs1796361907	genic_upstream_transcript_variant,intron_variant
rs1796362244	genic_upstream_transcript_variant,intron_variant
rs1796363065	genic_upstream_transcript_variant,intron_variant
rs1796363264	genic_upstream_transcript_variant,intron_variant
rs1796363453	genic_upstream_transcript_variant,intron_variant
rs1796364089	genic_upstream_transcript_variant,intron_variant
rs1796364602	genic_upstream_transcript_variant,intron_variant
rs1796364699	genic_upstream_transcript_variant,intron_variant
rs1796365748	genic_upstream_transcript_variant,intron_variant
rs1796365846	genic_upstream_transcript_variant,intron_variant
rs1796366356	genic_upstream_transcript_variant,intron_variant
rs1796366519	genic_upstream_transcript_variant,intron_variant
rs1796367787	genic_upstream_transcript_variant,intron_variant
rs1796367901	genic_upstream_transcript_variant,intron_variant
rs1796368012	genic_upstream_transcript_variant,intron_variant
rs1796368343	genic_upstream_transcript_variant,intron_variant
rs1796368484	genic_upstream_transcript_variant,intron_variant
rs1796368634	genic_upstream_transcript_variant,intron_variant
rs1796369298	genic_upstream_transcript_variant,intron_variant
rs1796370291	genic_upstream_transcript_variant,intron_variant
rs1796370434	genic_upstream_transcript_variant,intron_variant
rs1796370790	genic_upstream_transcript_variant,intron_variant
rs1796370901	genic_upstream_transcript_variant,intron_variant
rs1796371215	genic_upstream_transcript_variant,intron_variant
rs1796371529	genic_upstream_transcript_variant,intron_variant
rs1796371951	genic_upstream_transcript_variant,intron_variant
rs1796372157	genic_upstream_transcript_variant,intron_variant
rs1796372270	genic_upstream_transcript_variant,intron_variant
rs1796372575	genic_upstream_transcript_variant,intron_variant
rs1796372978	genic_upstream_transcript_variant,intron_variant
rs1796373183	genic_upstream_transcript_variant,intron_variant
rs1796373300	genic_upstream_transcript_variant,intron_variant
rs1796373620	genic_upstream_transcript_variant,intron_variant
rs1796373945	genic_upstream_transcript_variant,intron_variant
rs1796374142	genic_upstream_transcript_variant,intron_variant
rs1796374236	genic_upstream_transcript_variant,intron_variant
rs1796374598	genic_upstream_transcript_variant,intron_variant
rs1796374970	genic_upstream_transcript_variant,intron_variant
rs1796375844	genic_upstream_transcript_variant,intron_variant
rs1796376362	genic_upstream_transcript_variant,intron_variant
rs1796376706	genic_upstream_transcript_variant,intron_variant
rs1796376929	genic_upstream_transcript_variant,intron_variant
rs1796377195	genic_upstream_transcript_variant,intron_variant
rs1796377488	genic_upstream_transcript_variant,intron_variant
rs1796378295	genic_upstream_transcript_variant,intron_variant
rs1796378498	genic_upstream_transcript_variant,intron_variant
rs1796379189	genic_upstream_transcript_variant,intron_variant
rs1796379276	genic_upstream_transcript_variant,intron_variant
rs1796379577	genic_upstream_transcript_variant,intron_variant
rs1796379838	genic_upstream_transcript_variant,intron_variant
rs1796379936	genic_upstream_transcript_variant,intron_variant
rs1796380349	genic_upstream_transcript_variant,intron_variant
rs1796380452	genic_upstream_transcript_variant,intron_variant
rs1796380692	genic_upstream_transcript_variant,intron_variant
rs1796380785	genic_upstream_transcript_variant,intron_variant
rs1796381076	genic_upstream_transcript_variant,intron_variant
rs1796381393	genic_upstream_transcript_variant,intron_variant
rs1796382021	genic_upstream_transcript_variant,intron_variant
rs1796382240	genic_upstream_transcript_variant,intron_variant
rs1796382359	genic_upstream_transcript_variant,intron_variant

rs1796382933	genic_upstream_transcript_variant,intron_variant
rs1796383939	genic_upstream_transcript_variant,intron_variant
rs1796384058	genic_upstream_transcript_variant,intron_variant
rs1796384166	genic_upstream_transcript_variant,intron_variant
rs1796384261	genic_upstream_transcript_variant,intron_variant
rs1796384479	genic_upstream_transcript_variant,intron_variant
rs1796384648	genic_upstream_transcript_variant,intron_variant
rs1796384757	genic_upstream_transcript_variant,intron_variant
rs1796384849	genic_upstream_transcript_variant,intron_variant
rs1796384947	genic_upstream_transcript_variant,intron_variant
rs1796385055	genic_upstream_transcript_variant,intron_variant
rs1796385536	genic_upstream_transcript_variant,intron_variant
rs1796385872	genic_upstream_transcript_variant,intron_variant
rs1796386081	genic_upstream_transcript_variant,intron_variant
rs1796386480	genic_upstream_transcript_variant,intron_variant
rs1796386967	genic_upstream_transcript_variant,intron_variant
rs1796387891	genic_upstream_transcript_variant,intron_variant
rs1796387997	genic_upstream_transcript_variant,intron_variant
rs1796388223	genic_upstream_transcript_variant,intron_variant
rs1796388544	genic_upstream_transcript_variant,intron_variant
rs1796388835	genic_upstream_transcript_variant,intron_variant
rs1796388933	genic_upstream_transcript_variant,intron_variant
rs1796389140	genic_upstream_transcript_variant,intron_variant
rs1796389354	genic_upstream_transcript_variant,intron_variant
rs1796389460	genic_upstream_transcript_variant,intron_variant
rs1796389547	genic_upstream_transcript_variant,intron_variant
rs1796389749	genic_upstream_transcript_variant,intron_variant
rs1796390255	genic_upstream_transcript_variant,intron_variant
rs1796391179	genic_upstream_transcript_variant,intron_variant
rs1796391385	genic_upstream_transcript_variant,intron_variant
rs1796391515	genic_upstream_transcript_variant,intron_variant
rs1796391662	genic_upstream_transcript_variant,intron_variant
rs1796391857	genic_upstream_transcript_variant,intron_variant
rs1796392088	genic_upstream_transcript_variant,intron_variant
rs1796392252	genic_upstream_transcript_variant,intron_variant
rs1796392544	genic_upstream_transcript_variant,intron_variant
rs1796392648	genic_upstream_transcript_variant,intron_variant
rs1796392861	genic_upstream_transcript_variant,intron_variant
rs1796393251	genic_upstream_transcript_variant,intron_variant
rs1796393559	genic_upstream_transcript_variant,intron_variant
rs1796393730	genic_upstream_transcript_variant,intron_variant
rs1796394038	genic_upstream_transcript_variant,intron_variant
rs1796394309	genic_upstream_transcript_variant,intron_variant
rs1796394400	genic_upstream_transcript_variant,intron_variant
rs1796394510	genic_upstream_transcript_variant,intron_variant
rs1796394698	genic_upstream_transcript_variant,intron_variant
rs1796394793	genic_upstream_transcript_variant,intron_variant
rs1796394896	genic_upstream_transcript_variant,intron_variant
rs1796395089	genic_upstream_transcript_variant,intron_variant
rs1796395197	genic_upstream_transcript_variant,intron_variant
rs1796395386	genic_upstream_transcript_variant,intron_variant
rs1796395615	genic_upstream_transcript_variant,intron_variant
rs1796396902	genic_upstream_transcript_variant,intron_variant
rs1796397051	genic_upstream_transcript_variant,intron_variant
rs1796397281	genic_upstream_transcript_variant,intron_variant
rs1796397702	genic_upstream_transcript_variant,intron_variant
rs1796398059	genic_upstream_transcript_variant,intron_variant
rs1796399898	genic_upstream_transcript_variant,intron_variant
rs1796400067	genic_upstream_transcript_variant,intron_variant
rs1796400448	genic_upstream_transcript_variant,intron_variant
rs1796400600	genic_upstream_transcript_variant,intron_variant
rs1796401001	genic_upstream_transcript_variant,intron_variant
rs1796401872	genic_upstream_transcript_variant,intron_variant
rs1796402379	genic_upstream_transcript_variant,intron_variant
rs1796402587	genic_upstream_transcript_variant,intron_variant
rs1796402933	genic_upstream_transcript_variant,intron_variant
rs1796403273	genic_upstream_transcript_variant,intron_variant
rs1796403783	genic_upstream_transcript_variant,intron_variant
rs1796403910	genic_upstream_transcript_variant,intron_variant
rs1796404291	genic_upstream_transcript_variant,intron_variant
rs1796404466	genic_upstream_transcript_variant,intron_variant
rs1796404794	genic_upstream_transcript_variant,intron_variant
rs1796405267	genic_upstream_transcript_variant,intron_variant
rs1796405429	genic_upstream_transcript_variant,intron_variant

rs1796405739	genic_upstream_transcript_variant,intron_variant
rs1796405898	genic_upstream_transcript_variant,intron_variant
rs1796406070	genic_upstream_transcript_variant,intron_variant
rs1796406465	genic_upstream_transcript_variant,intron_variant
rs1796406621	genic_upstream_transcript_variant,intron_variant
rs1796406991	genic_upstream_transcript_variant,intron_variant
rs1796407105	genic_upstream_transcript_variant,intron_variant
rs1796407434	genic_upstream_transcript_variant,intron_variant
rs1796407767	genic_upstream_transcript_variant,intron_variant
rs1796407857	genic_upstream_transcript_variant,intron_variant
rs1796408078	genic_upstream_transcript_variant,intron_variant
rs1796408634	genic_upstream_transcript_variant,intron_variant
rs1796409046	genic_upstream_transcript_variant,intron_variant
rs1796409334	genic_upstream_transcript_variant,intron_variant
rs1796410151	genic_upstream_transcript_variant,intron_variant
rs1796410368	genic_upstream_transcript_variant,intron_variant
rs1796410477	genic_upstream_transcript_variant,intron_variant
rs1796410989	genic_upstream_transcript_variant,intron_variant
rs1796411219	genic_upstream_transcript_variant,intron_variant
rs1796411847	genic_upstream_transcript_variant,intron_variant
rs1796412441	genic_upstream_transcript_variant,intron_variant
rs1796412664	genic_upstream_transcript_variant,intron_variant
rs1796412761	genic_upstream_transcript_variant,intron_variant
rs1796412869	genic_upstream_transcript_variant,intron_variant
rs1796412983	genic_upstream_transcript_variant,intron_variant
rs1796413096	genic_upstream_transcript_variant,intron_variant
rs1796413314	genic_upstream_transcript_variant,intron_variant
rs1796413554	genic_upstream_transcript_variant,intron_variant
rs1796413836	genic_upstream_transcript_variant,intron_variant
rs1796414052	genic_upstream_transcript_variant,intron_variant
rs1796414531	genic_upstream_transcript_variant,intron_variant
rs1796414634	genic_upstream_transcript_variant,intron_variant
rs1796414748	genic_upstream_transcript_variant,intron_variant
rs1796414865	genic_upstream_transcript_variant,intron_variant
rs1796415647	genic_upstream_transcript_variant,intron_variant
rs1796416362	genic_upstream_transcript_variant,intron_variant
rs1796416459	genic_upstream_transcript_variant,intron_variant
rs1796416670	genic_upstream_transcript_variant,intron_variant
rs1796417150	genic_upstream_transcript_variant,intron_variant
rs1796417718	genic_upstream_transcript_variant,intron_variant
rs1796417791	genic_upstream_transcript_variant,intron_variant
rs1796418229	genic_upstream_transcript_variant,intron_variant
rs1796418348	genic_upstream_transcript_variant,intron_variant
rs1796418730	genic_upstream_transcript_variant,intron_variant
rs1796418939	genic_upstream_transcript_variant,intron_variant
rs1796419126	genic_upstream_transcript_variant,intron_variant
rs1796419474	genic_upstream_transcript_variant,intron_variant
rs1796419681	genic_upstream_transcript_variant,intron_variant
rs1796419951	genic_upstream_transcript_variant,intron_variant
rs1796420049	genic_upstream_transcript_variant,intron_variant
rs1796420150	genic_upstream_transcript_variant,intron_variant
rs1796420240	genic_upstream_transcript_variant,intron_variant
rs1796420554	genic_upstream_transcript_variant,intron_variant
rs1796421735	genic_upstream_transcript_variant,intron_variant
rs1796421841	genic_upstream_transcript_variant,intron_variant
rs1796421949	genic_upstream_transcript_variant,intron_variant
rs1796422084	genic_upstream_transcript_variant,intron_variant
rs1796422370	genic_upstream_transcript_variant,intron_variant
rs1796422963	genic_upstream_transcript_variant,intron_variant
rs1796423317	genic_upstream_transcript_variant,intron_variant
rs1796424020	genic_upstream_transcript_variant,intron_variant
rs1796424457	genic_upstream_transcript_variant,intron_variant
rs1796424547	genic_upstream_transcript_variant,intron_variant
rs1796424667	genic_upstream_transcript_variant,intron_variant
rs1796424765	genic_upstream_transcript_variant,intron_variant
rs1796425135	genic_upstream_transcript_variant,intron_variant
rs1796425423	genic_upstream_transcript_variant,intron_variant
rs1796425539	genic_upstream_transcript_variant,intron_variant
rs1796425735	genic_upstream_transcript_variant,intron_variant
rs1796425833	genic_upstream_transcript_variant,intron_variant
rs1796426120	genic_upstream_transcript_variant,intron_variant
rs1796426462	genic_upstream_transcript_variant,intron_variant
rs1796426659	genic_upstream_transcript_variant,intron_variant
rs1796426769	genic_upstream_transcript_variant,intron_variant

rs1796426981	genic_upstream_transcript_variant,intron_variant
rs1796427074	genic_upstream_transcript_variant,intron_variant
rs1796427955	genic_upstream_transcript_variant,intron_variant
rs1796428447	genic_upstream_transcript_variant,intron_variant
rs1796429057	genic_upstream_transcript_variant,intron_variant
rs1796429140	genic_upstream_transcript_variant,intron_variant
rs1796429352	genic_upstream_transcript_variant,intron_variant
rs1796429879	genic_upstream_transcript_variant,intron_variant
rs1796429973	genic_upstream_transcript_variant,intron_variant
rs1796430168	genic_upstream_transcript_variant,intron_variant
rs1796430284	genic_upstream_transcript_variant,intron_variant
rs1796430639	genic_upstream_transcript_variant,intron_variant
rs1796430851	genic_upstream_transcript_variant,intron_variant
rs1796431247	genic_upstream_transcript_variant,intron_variant
rs1796431583	genic_upstream_transcript_variant,intron_variant
rs1796431772	genic_upstream_transcript_variant,intron_variant
rs1796432397	genic_upstream_transcript_variant,intron_variant
rs1796432591	genic_upstream_transcript_variant,intron_variant
rs1796432792	genic_upstream_transcript_variant,intron_variant
rs1796433043	genic_upstream_transcript_variant,intron_variant
rs1796433143	genic_upstream_transcript_variant,intron_variant
rs1796433329	genic_upstream_transcript_variant,intron_variant
rs1796433435	genic_upstream_transcript_variant,intron_variant
rs1796433653	genic_upstream_transcript_variant,intron_variant
rs1796433837	genic_upstream_transcript_variant,intron_variant
rs1796434309	genic_upstream_transcript_variant,intron_variant
rs1796434512	genic_upstream_transcript_variant,intron_variant
rs1796434619	genic_upstream_transcript_variant,intron_variant
rs1796434850	genic_upstream_transcript_variant,intron_variant
rs1796434942	genic_upstream_transcript_variant,intron_variant
rs1796435179	genic_upstream_transcript_variant,intron_variant
rs1796435268	genic_upstream_transcript_variant,intron_variant
rs1796435747	genic_upstream_transcript_variant,intron_variant
rs1796435846	genic_upstream_transcript_variant,intron_variant
rs1796435945	genic_upstream_transcript_variant,intron_variant
rs1796436037	genic_upstream_transcript_variant,intron_variant
rs1796436368	genic_upstream_transcript_variant,intron_variant
rs1796436693	genic_upstream_transcript_variant,intron_variant
rs1796437337	genic_upstream_transcript_variant,intron_variant
rs1796437434	genic_upstream_transcript_variant,intron_variant
rs1796437629	genic_upstream_transcript_variant,intron_variant
rs1796437900	genic_upstream_transcript_variant,intron_variant
rs1796438178	genic_upstream_transcript_variant,intron_variant
rs1796439394	genic_upstream_transcript_variant,intron_variant
rs1796440087	genic_upstream_transcript_variant,intron_variant
rs1796440407	genic_upstream_transcript_variant,intron_variant
rs1796440595	genic_upstream_transcript_variant,intron_variant
rs1796440692	genic_upstream_transcript_variant,intron_variant
rs1796440800	genic_upstream_transcript_variant,intron_variant
rs1796440915	genic_upstream_transcript_variant,intron_variant
rs1796441093	genic_upstream_transcript_variant,intron_variant
rs1796441194	genic_upstream_transcript_variant,intron_variant
rs1796441300	genic_upstream_transcript_variant,intron_variant
rs1796441382	genic_upstream_transcript_variant,intron_variant
rs1796441472	genic_upstream_transcript_variant,intron_variant
rs1796441561	genic_upstream_transcript_variant,intron_variant
rs1796441671	genic_upstream_transcript_variant,intron_variant
rs1796442133	genic_upstream_transcript_variant,intron_variant
rs1796442246	genic_upstream_transcript_variant,intron_variant
rs1796442375	genic_upstream_transcript_variant,intron_variant
rs1796443514	genic_upstream_transcript_variant,intron_variant
rs1796443620	genic_upstream_transcript_variant,intron_variant
rs1796443852	genic_upstream_transcript_variant,intron_variant
rs1796444406	genic_upstream_transcript_variant,intron_variant
rs1796444728	genic_upstream_transcript_variant,intron_variant
rs1796444831	genic_upstream_transcript_variant,intron_variant
rs1796445370	genic_upstream_transcript_variant,intron_variant
rs1796445841	genic_upstream_transcript_variant,intron_variant
rs1796446153	genic_upstream_transcript_variant,intron_variant
rs1796446243	genic_upstream_transcript_variant,intron_variant
rs1796446340	genic_upstream_transcript_variant,intron_variant
rs1796446533	genic_upstream_transcript_variant,intron_variant
rs1796447013	genic_upstream_transcript_variant,intron_variant
rs1796447200	genic_upstream_transcript_variant,intron_variant

rs1796447299	genic_upstream_transcript_variant,intron_variant
rs1796447577	genic_upstream_transcript_variant,intron_variant
rs1796447759	genic_upstream_transcript_variant,intron_variant
rs1796447952	genic_upstream_transcript_variant,intron_variant
rs1796448150	genic_upstream_transcript_variant,intron_variant
rs1796448357	genic_upstream_transcript_variant,intron_variant
rs1796448930	genic_upstream_transcript_variant,intron_variant
rs1796449250	genic_upstream_transcript_variant,intron_variant
rs1796450259	genic_upstream_transcript_variant,intron_variant
rs1796450590	genic_upstream_transcript_variant,intron_variant
rs1796450706	genic_upstream_transcript_variant,intron_variant
rs1796450930	genic_upstream_transcript_variant,intron_variant
rs1796451142	genic_upstream_transcript_variant,intron_variant
rs1796452121	genic_upstream_transcript_variant,intron_variant
rs1796452549	genic_upstream_transcript_variant,intron_variant
rs1796452771	genic_upstream_transcript_variant,intron_variant
rs1796452959	genic_upstream_transcript_variant,intron_variant
rs1796453536	genic_upstream_transcript_variant,intron_variant
rs1796453642	genic_upstream_transcript_variant,intron_variant
rs1796453760	genic_upstream_transcript_variant,intron_variant
rs1796453864	genic_upstream_transcript_variant,intron_variant
rs1796455361	genic_upstream_transcript_variant,intron_variant
rs1796455575	genic_upstream_transcript_variant,intron_variant
rs1796455691	genic_upstream_transcript_variant,intron_variant
rs1796456145	genic_upstream_transcript_variant,intron_variant
rs1796456222	genic_upstream_transcript_variant,intron_variant
rs1796456806	genic_upstream_transcript_variant,intron_variant
rs1796457084	genic_upstream_transcript_variant,intron_variant
rs1796457511	genic_upstream_transcript_variant,intron_variant
rs1796457847	genic_upstream_transcript_variant,intron_variant
rs1796459022	genic_upstream_transcript_variant,intron_variant
rs1796461078	genic_upstream_transcript_variant,intron_variant
rs1796461656	genic_upstream_transcript_variant,intron_variant
rs1796462015	genic_upstream_transcript_variant,intron_variant
rs1796462162	genic_upstream_transcript_variant,intron_variant
rs1796462828	genic_upstream_transcript_variant,intron_variant
rs1796463096	genic_upstream_transcript_variant,intron_variant
rs1796464061	genic_upstream_transcript_variant,intron_variant
rs1796464164	genic_upstream_transcript_variant,intron_variant
rs1796464376	genic_upstream_transcript_variant,intron_variant
rs1796464586	genic_upstream_transcript_variant,intron_variant
rs1796465060	genic_upstream_transcript_variant,intron_variant
rs1796465294	genic_upstream_transcript_variant,intron_variant
rs1796465415	genic_upstream_transcript_variant,intron_variant
rs1796465965	genic_upstream_transcript_variant,intron_variant
rs1796466568	genic_upstream_transcript_variant,intron_variant
rs1796466679	genic_upstream_transcript_variant,intron_variant
rs1796466792	genic_upstream_transcript_variant,intron_variant
rs1796466910	genic_upstream_transcript_variant,intron_variant
rs1796467003	genic_upstream_transcript_variant,intron_variant
rs1796467217	genic_upstream_transcript_variant,intron_variant
rs1796467438	genic_upstream_transcript_variant,intron_variant
rs1796467551	genic_upstream_transcript_variant,intron_variant
rs1796467879	genic_upstream_transcript_variant,intron_variant
rs1796468237	genic_upstream_transcript_variant,intron_variant
rs1796468398	genic_upstream_transcript_variant,intron_variant
rs1796468745	genic_upstream_transcript_variant,intron_variant
rs1796469590	genic_upstream_transcript_variant,intron_variant
rs1796469760	genic_upstream_transcript_variant,intron_variant
rs1796470739	genic_upstream_transcript_variant,intron_variant
rs1796471693	genic_upstream_transcript_variant,intron_variant
rs1796471799	genic_upstream_transcript_variant,intron_variant
rs1796472890	genic_upstream_transcript_variant,intron_variant
rs1796473855	genic_upstream_transcript_variant,intron_variant
rs1796474724	genic_upstream_transcript_variant,intron_variant
rs1796474939	genic_upstream_transcript_variant,intron_variant
rs1796475191	genic_upstream_transcript_variant,intron_variant
rs1796475604	genic_upstream_transcript_variant,intron_variant
rs1796475793	genic_upstream_transcript_variant,intron_variant
rs1796475889	genic_upstream_transcript_variant,intron_variant
rs1796476805	genic_upstream_transcript_variant,intron_variant
rs1796476897	genic_upstream_transcript_variant,intron_variant
rs1796477336	genic_upstream_transcript_variant,intron_variant
rs1796477470	genic_upstream_transcript_variant,intron_variant

rs1796477800	genic_upstream_transcript_variant,intron_variant
rs1796477895	genic_upstream_transcript_variant,intron_variant
rs1796477996	genic_upstream_transcript_variant,intron_variant
rs1796478190	genic_upstream_transcript_variant,intron_variant
rs1796478500	genic_upstream_transcript_variant,intron_variant
rs1796478604	genic_upstream_transcript_variant,intron_variant
rs1796478807	genic_upstream_transcript_variant,intron_variant
rs1796479506	genic_upstream_transcript_variant,intron_variant
rs1796479915	genic_upstream_transcript_variant,intron_variant
rs1796480610	genic_upstream_transcript_variant,intron_variant
rs1796480737	genic_upstream_transcript_variant,intron_variant
rs1796480838	genic_upstream_transcript_variant,intron_variant
rs1796481244	genic_upstream_transcript_variant,intron_variant
rs1796481863	genic_upstream_transcript_variant,intron_variant
rs1796481964	genic_upstream_transcript_variant,intron_variant
rs1796482346	genic_upstream_transcript_variant,intron_variant
rs1796482474	genic_upstream_transcript_variant,intron_variant
rs1796483230	genic_upstream_transcript_variant,intron_variant
rs1796483413	genic_upstream_transcript_variant,intron_variant
rs1796483785	genic_upstream_transcript_variant,intron_variant
rs1796484250	genic_upstream_transcript_variant,intron_variant
rs1796484399	genic_upstream_transcript_variant,intron_variant
rs1796484561	genic_upstream_transcript_variant,intron_variant
rs1796485017	genic_upstream_transcript_variant,intron_variant
rs1796485164	genic_upstream_transcript_variant,intron_variant
rs1796485483	genic_upstream_transcript_variant,intron_variant
rs1796486107	genic_upstream_transcript_variant,intron_variant
rs1796486290	genic_upstream_transcript_variant,intron_variant
rs1796486736	genic_upstream_transcript_variant,intron_variant
rs1796486904	genic_upstream_transcript_variant,intron_variant
rs1796487229	genic_upstream_transcript_variant,intron_variant
rs1796487518	genic_upstream_transcript_variant,intron_variant
rs1796488149	genic_upstream_transcript_variant,intron_variant
rs1796488346	genic_upstream_transcript_variant,intron_variant
rs1796488654	genic_upstream_transcript_variant,intron_variant
rs1796489161	genic_upstream_transcript_variant,intron_variant
rs1796489367	genic_upstream_transcript_variant,intron_variant
rs1796489472	genic_upstream_transcript_variant,intron_variant
rs1796489600	genic_upstream_transcript_variant,intron_variant
rs1796489705	genic_upstream_transcript_variant,intron_variant
rs1796489957	genic_upstream_transcript_variant,intron_variant
rs1796490770	genic_upstream_transcript_variant,intron_variant
rs1796491382	genic_upstream_transcript_variant,intron_variant
rs1796491825	genic_upstream_transcript_variant,intron_variant
rs1796492029	genic_upstream_transcript_variant,intron_variant
rs1796492579	genic_upstream_transcript_variant,intron_variant
rs1796493954	genic_upstream_transcript_variant,intron_variant
rs1796494348	genic_upstream_transcript_variant,intron_variant
rs1796494450	genic_upstream_transcript_variant,intron_variant
rs1796495232	genic_upstream_transcript_variant,intron_variant
rs1796495594	genic_upstream_transcript_variant,intron_variant
rs1796495712	genic_upstream_transcript_variant,intron_variant
rs1796495829	genic_upstream_transcript_variant,intron_variant
rs1796495979	genic_upstream_transcript_variant,intron_variant
rs1796496752	genic_upstream_transcript_variant,intron_variant
rs1796496840	genic_upstream_transcript_variant,intron_variant
rs1796497179	genic_upstream_transcript_variant,intron_variant
rs1796497278	genic_upstream_transcript_variant,intron_variant
rs1796497633	genic_upstream_transcript_variant,intron_variant
rs1796498422	genic_upstream_transcript_variant,intron_variant
rs1796499397	genic_upstream_transcript_variant,intron_variant
rs1796499826	genic_upstream_transcript_variant,intron_variant
rs1796500067	genic_upstream_transcript_variant,intron_variant
rs1796500371	genic_upstream_transcript_variant,intron_variant
rs1796500463	genic_upstream_transcript_variant,intron_variant
rs1796500969	genic_upstream_transcript_variant,intron_variant
rs1796501062	genic_upstream_transcript_variant,intron_variant
rs1796501160	genic_upstream_transcript_variant,intron_variant
rs1796501646	genic_upstream_transcript_variant,intron_variant
rs1796501736	genic_upstream_transcript_variant,intron_variant
rs1796502175	genic_upstream_transcript_variant,intron_variant
rs1796502265	genic_upstream_transcript_variant,intron_variant
rs1796502585	genic_upstream_transcript_variant,intron_variant
rs1796502788	genic_upstream_transcript_variant,intron_variant

rs1796503276	genic_upstream_transcript_variant,intron_variant
rs1796503387	genic_upstream_transcript_variant,intron_variant
rs1796504425	genic_upstream_transcript_variant,intron_variant
rs1796504738	genic_upstream_transcript_variant,intron_variant
rs1796505586	genic_upstream_transcript_variant,intron_variant
rs1796505689	genic_upstream_transcript_variant,intron_variant
rs1796506384	genic_upstream_transcript_variant,intron_variant
rs1796506871	genic_upstream_transcript_variant,intron_variant
rs1796507560	genic_upstream_transcript_variant,intron_variant
rs1796507695	genic_upstream_transcript_variant,intron_variant
rs1796508368	genic_upstream_transcript_variant,intron_variant
rs1796508538	genic_upstream_transcript_variant,intron_variant
rs1796509304	genic_upstream_transcript_variant,intron_variant
rs1796509686	genic_upstream_transcript_variant,intron_variant
rs1796509835	genic_upstream_transcript_variant,intron_variant
rs1796509976	genic_upstream_transcript_variant,intron_variant
rs1796510686	genic_upstream_transcript_variant,intron_variant
rs1796511345	genic_upstream_transcript_variant,intron_variant
rs1796511761	genic_upstream_transcript_variant,intron_variant
rs1796512282	genic_upstream_transcript_variant,intron_variant
rs1796512459	genic_upstream_transcript_variant,intron_variant
rs1796512566	genic_upstream_transcript_variant,intron_variant
rs1796513405	genic_upstream_transcript_variant,intron_variant
rs1796513679	genic_upstream_transcript_variant,intron_variant
rs1796514151	genic_upstream_transcript_variant,intron_variant
rs1796514468	genic_upstream_transcript_variant,intron_variant
rs1796514821	genic_upstream_transcript_variant,intron_variant
rs1796514921	genic_upstream_transcript_variant,intron_variant
rs1796515120	genic_upstream_transcript_variant,intron_variant
rs1796515973	genic_upstream_transcript_variant,intron_variant
rs1796516473	genic_upstream_transcript_variant,intron_variant
rs1796516924	genic_upstream_transcript_variant,intron_variant
rs1796517122	genic_upstream_transcript_variant,intron_variant
rs1796517823	genic_upstream_transcript_variant,intron_variant
rs1796518444	genic_upstream_transcript_variant,intron_variant
rs1796518540	genic_upstream_transcript_variant,intron_variant
rs1796518631	genic_upstream_transcript_variant,intron_variant
rs1796518738	genic_upstream_transcript_variant,intron_variant
rs1796518865	genic_upstream_transcript_variant,intron_variant
rs1796519183	genic_upstream_transcript_variant,intron_variant
rs1796519530	genic_upstream_transcript_variant,intron_variant
rs1796520172	genic_upstream_transcript_variant,intron_variant
rs1796520478	genic_upstream_transcript_variant,intron_variant
rs1796520589	genic_upstream_transcript_variant,intron_variant
rs1796520925	genic_upstream_transcript_variant,intron_variant
rs1796521100	genic_upstream_transcript_variant,intron_variant
rs1796521195	genic_upstream_transcript_variant,intron_variant
rs1796521499	genic_upstream_transcript_variant,intron_variant
rs1796521756	genic_upstream_transcript_variant,intron_variant
rs1796521969	genic_upstream_transcript_variant,intron_variant
rs1796522059	genic_upstream_transcript_variant,intron_variant
rs1796522286	genic_upstream_transcript_variant,intron_variant
rs1796522382	genic_upstream_transcript_variant,intron_variant
rs1796522491	genic_upstream_transcript_variant,intron_variant
rs1796522838	genic_upstream_transcript_variant,intron_variant
rs1796523621	genic_upstream_transcript_variant,intron_variant
rs1796523728	genic_upstream_transcript_variant,intron_variant
rs1796524028	genic_upstream_transcript_variant,intron_variant
rs1796524238	genic_upstream_transcript_variant,intron_variant
rs1796524636	genic_upstream_transcript_variant,intron_variant
rs1796525092	genic_upstream_transcript_variant,intron_variant
rs1796525318	genic_upstream_transcript_variant,intron_variant
rs1796525656	genic_upstream_transcript_variant,intron_variant
rs1796525763	genic_upstream_transcript_variant,intron_variant
rs1796526357	genic_upstream_transcript_variant,intron_variant
rs1796526471	genic_upstream_transcript_variant,intron_variant
rs1796527506	genic_upstream_transcript_variant,intron_variant
rs1796527728	genic_upstream_transcript_variant,intron_variant
rs1796527936	genic_upstream_transcript_variant,intron_variant
rs1796528558	genic_upstream_transcript_variant,intron_variant
rs1796528897	genic_upstream_transcript_variant,intron_variant
rs1796528997	genic_upstream_transcript_variant,intron_variant
rs1796529192	genic_upstream_transcript_variant,intron_variant
rs1796529284	genic_upstream_transcript_variant,intron_variant

rs1796529696	genic_upstream_transcript_variant,intron_variant
rs1796529807	genic_upstream_transcript_variant,intron_variant
rs1796530319	genic_upstream_transcript_variant,intron_variant
rs1796530780	genic_upstream_transcript_variant,intron_variant
rs1796531135	genic_upstream_transcript_variant,intron_variant
rs1796531666	genic_upstream_transcript_variant,intron_variant
rs1796532121	genic_upstream_transcript_variant,intron_variant
rs1796532354	genic_upstream_transcript_variant,intron_variant
rs1796532469	genic_upstream_transcript_variant,intron_variant
rs1796532822	genic_upstream_transcript_variant,intron_variant
rs1796532934	genic_upstream_transcript_variant,intron_variant
rs1796533027	genic_upstream_transcript_variant,intron_variant
rs1796534320	genic_upstream_transcript_variant,intron_variant
rs1796535064	genic_upstream_transcript_variant,intron_variant
rs1796535230	genic_upstream_transcript_variant,intron_variant
rs1796535393	genic_upstream_transcript_variant,intron_variant
rs1796535561	genic_upstream_transcript_variant,intron_variant
rs1796535730	genic_upstream_transcript_variant,intron_variant
rs1796535893	genic_upstream_transcript_variant,intron_variant
rs1796536057	genic_upstream_transcript_variant,intron_variant
rs1796536406	genic_upstream_transcript_variant,intron_variant
rs1796536751	genic_upstream_transcript_variant,intron_variant
rs1796537496	genic_upstream_transcript_variant,intron_variant
rs1796537654	genic_upstream_transcript_variant,intron_variant
rs1796537992	genic_upstream_transcript_variant,intron_variant
rs1796538333	genic_upstream_transcript_variant,intron_variant
rs1796538575	genic_upstream_transcript_variant,intron_variant
rs1796539100	genic_upstream_transcript_variant,intron_variant
rs1796539310	genic_upstream_transcript_variant,intron_variant
rs1796539853	genic_upstream_transcript_variant,intron_variant
rs1796539954	genic_upstream_transcript_variant,intron_variant
rs1796540561	genic_upstream_transcript_variant,intron_variant
rs1796540667	genic_upstream_transcript_variant,intron_variant
rs1796540851	genic_upstream_transcript_variant,intron_variant
rs1796541124	genic_upstream_transcript_variant,intron_variant
rs1796541219	genic_upstream_transcript_variant,intron_variant
rs1796541318	genic_upstream_transcript_variant,intron_variant
rs1796541404	genic_upstream_transcript_variant,intron_variant
rs1796542303	genic_upstream_transcript_variant,intron_variant
rs1796542414	genic_upstream_transcript_variant,intron_variant
rs1796543058	genic_upstream_transcript_variant,intron_variant
rs1796543168	genic_upstream_transcript_variant,intron_variant
rs1796543262	genic_upstream_transcript_variant,intron_variant
rs1796543490	genic_upstream_transcript_variant,intron_variant
rs1796544000	genic_upstream_transcript_variant,intron_variant
rs1796545409	genic_upstream_transcript_variant,intron_variant
rs1796545575	genic_upstream_transcript_variant,intron_variant
rs1796545728	genic_upstream_transcript_variant,intron_variant
rs1796546216	genic_upstream_transcript_variant,intron_variant
rs1796546613	genic_upstream_transcript_variant,intron_variant
rs1796548181	genic_upstream_transcript_variant,intron_variant
rs1796548393	genic_upstream_transcript_variant,intron_variant
rs1796549447	genic_upstream_transcript_variant,intron_variant
rs1796550255	genic_upstream_transcript_variant,intron_variant
rs1796550965	genic_upstream_transcript_variant,intron_variant
rs1796551428	genic_upstream_transcript_variant,intron_variant
rs1796551584	genic_upstream_transcript_variant,intron_variant
rs1796551738	genic_upstream_transcript_variant,intron_variant
rs1796551873	genic_upstream_transcript_variant,intron_variant
rs1796552821	genic_upstream_transcript_variant,intron_variant
rs1796553393	genic_upstream_transcript_variant,intron_variant
rs1796553878	genic_upstream_transcript_variant,intron_variant
rs1796554331	genic_upstream_transcript_variant,intron_variant
rs1796554798	genic_upstream_transcript_variant,intron_variant
rs1796554964	genic_upstream_transcript_variant,intron_variant
rs1796555622	genic_upstream_transcript_variant,intron_variant
rs1796555781	genic_upstream_transcript_variant,intron_variant
rs1796555942	genic_upstream_transcript_variant,intron_variant
rs1796556109	genic_upstream_transcript_variant,intron_variant
rs1796556267	genic_upstream_transcript_variant,intron_variant
rs1796556554	genic_upstream_transcript_variant,intron_variant
rs1796556735	genic_upstream_transcript_variant,intron_variant
rs1796556883	genic_upstream_transcript_variant,intron_variant
rs1796557113	genic_upstream_transcript_variant,intron_variant

rs1796557211	genic_upstream_transcript_variant,intron_variant
rs1796557340	genic_upstream_transcript_variant,intron_variant
rs1796557443	genic_upstream_transcript_variant,intron_variant
rs1796557710	genic_upstream_transcript_variant,intron_variant
rs1796558041	genic_upstream_transcript_variant,intron_variant
rs1796558577	genic_upstream_transcript_variant,intron_variant
rs1796559353	genic_upstream_transcript_variant,intron_variant
rs1796559450	genic_upstream_transcript_variant,intron_variant
rs1796560028	genic_upstream_transcript_variant,intron_variant
rs1796560226	genic_upstream_transcript_variant,intron_variant
rs1796560328	genic_upstream_transcript_variant,intron_variant
rs1796560419	genic_upstream_transcript_variant,intron_variant
rs1796560611	genic_upstream_transcript_variant,intron_variant
rs1796560806	genic_upstream_transcript_variant,intron_variant
rs1796560910	genic_upstream_transcript_variant,intron_variant
rs1796561110	genic_upstream_transcript_variant,intron_variant
rs1796561730	genic_upstream_transcript_variant,intron_variant
rs1796561896	genic_upstream_transcript_variant,intron_variant
rs1796562063	genic_upstream_transcript_variant,intron_variant
rs1796562858	genic_upstream_transcript_variant,intron_variant
rs1796564470	genic_upstream_transcript_variant,intron_variant
rs1796564640	genic_upstream_transcript_variant,intron_variant
rs1796565731	genic_upstream_transcript_variant,intron_variant
rs1796565876	genic_upstream_transcript_variant,intron_variant
rs1796566257	genic_upstream_transcript_variant,intron_variant
rs1796566754	genic_upstream_transcript_variant,intron_variant
rs1796566921	genic_upstream_transcript_variant,intron_variant
rs1796567831	genic_upstream_transcript_variant,intron_variant
rs1796567978	genic_upstream_transcript_variant,intron_variant
rs1796568130	genic_upstream_transcript_variant,intron_variant
rs1796568283	genic_upstream_transcript_variant,intron_variant
rs1796568429	genic_upstream_transcript_variant,intron_variant
rs1796568844	genic_upstream_transcript_variant,intron_variant
rs1796569015	genic_upstream_transcript_variant,intron_variant
rs1796569738	genic_upstream_transcript_variant,intron_variant
rs1796570230	genic_upstream_transcript_variant,intron_variant
rs1796570384	genic_upstream_transcript_variant,intron_variant
rs1796571322	genic_upstream_transcript_variant,intron_variant
rs1796571504	genic_upstream_transcript_variant,intron_variant
rs1796571859	genic_upstream_transcript_variant,intron_variant
rs1796572216	genic_upstream_transcript_variant,intron_variant
rs1796572498	genic_upstream_transcript_variant,intron_variant
rs1796572846	genic_upstream_transcript_variant,intron_variant
rs1796573082	genic_upstream_transcript_variant,intron_variant
rs1796574072	genic_upstream_transcript_variant,intron_variant
rs1796574177	genic_upstream_transcript_variant,intron_variant
rs1796574743	genic_upstream_transcript_variant,intron_variant
rs1796575110	genic_upstream_transcript_variant,intron_variant
rs1796575418	genic_upstream_transcript_variant,intron_variant
rs1796575894	genic_upstream_transcript_variant,intron_variant
rs1796576686	genic_upstream_transcript_variant,intron_variant
rs1796576842	genic_upstream_transcript_variant,intron_variant
rs1796577668	genic_upstream_transcript_variant,intron_variant
rs1796577861	genic_upstream_transcript_variant,intron_variant
rs1796578236	genic_upstream_transcript_variant,intron_variant
rs1796578408	genic_upstream_transcript_variant,intron_variant
rs1796579293	genic_upstream_transcript_variant,intron_variant
rs1796580336	genic_upstream_transcript_variant,intron_variant
rs1796580681	genic_upstream_transcript_variant,intron_variant
rs1796580768	genic_upstream_transcript_variant,intron_variant
rs1796580877	genic_upstream_transcript_variant,intron_variant
rs1796580988	genic_upstream_transcript_variant,intron_variant
rs1796581236	genic_upstream_transcript_variant,intron_variant
rs1796581437	genic_upstream_transcript_variant,intron_variant
rs1796581744	genic_upstream_transcript_variant,intron_variant
rs1796582699	genic_upstream_transcript_variant,intron_variant
rs1796583208	genic_upstream_transcript_variant,intron_variant
rs1796584110	genic_upstream_transcript_variant,intron_variant
rs1796584865	genic_upstream_transcript_variant,intron_variant
rs1796585032	genic_upstream_transcript_variant,intron_variant
rs1796585276	genic_upstream_transcript_variant,intron_variant
rs1796586009	genic_upstream_transcript_variant,intron_variant
rs1796586567	genic_upstream_transcript_variant,intron_variant
rs1796586943	genic_upstream_transcript_variant,intron_variant

rs1796587038	genic_upstream_transcript_variant,intron_variant
rs1796587865	genic_upstream_transcript_variant,intron_variant
rs1796587992	genic_upstream_transcript_variant,intron_variant
rs1796588699	genic_upstream_transcript_variant,intron_variant
rs1796588962	genic_upstream_transcript_variant,intron_variant
rs1796589113	genic_upstream_transcript_variant,intron_variant
rs1796589774	genic_upstream_transcript_variant,intron_variant
rs1796590109	genic_upstream_transcript_variant,intron_variant
rs1796590340	genic_upstream_transcript_variant,intron_variant
rs1796590462	genic_upstream_transcript_variant,intron_variant
rs1796590689	genic_upstream_transcript_variant,intron_variant
rs1796590936	genic_upstream_transcript_variant,intron_variant
rs1796591264	genic_upstream_transcript_variant,intron_variant
rs1796591469	genic_upstream_transcript_variant,intron_variant
rs1796591697	genic_upstream_transcript_variant,intron_variant
rs1796591798	genic_upstream_transcript_variant,intron_variant
rs1796592347	genic_upstream_transcript_variant,intron_variant
rs1796592939	genic_upstream_transcript_variant,intron_variant
rs1796593056	genic_upstream_transcript_variant,intron_variant
rs1796593772	genic_upstream_transcript_variant,intron_variant
rs1796593878	genic_upstream_transcript_variant,intron_variant
rs1796593992	genic_upstream_transcript_variant,intron_variant
rs1796594408	genic_upstream_transcript_variant,intron_variant
rs1796595081	genic_upstream_transcript_variant,intron_variant
rs1796595307	genic_upstream_transcript_variant,intron_variant
rs1796595774	genic_upstream_transcript_variant,intron_variant
rs1796596226	genic_upstream_transcript_variant,intron_variant
rs1796596435	genic_upstream_transcript_variant,intron_variant
rs1796596654	genic_upstream_transcript_variant,intron_variant
rs1796596973	genic_upstream_transcript_variant,intron_variant
rs1796597082	genic_upstream_transcript_variant,intron_variant
rs1796597393	genic_upstream_transcript_variant,intron_variant
rs1796597495	genic_upstream_transcript_variant,intron_variant
rs1796597673	genic_upstream_transcript_variant,intron_variant
rs1796597827	genic_upstream_transcript_variant,intron_variant
rs1796597917	genic_upstream_transcript_variant,intron_variant
rs1796598000	genic_upstream_transcript_variant,intron_variant
rs1796598101	genic_upstream_transcript_variant,intron_variant
rs1796598196	genic_upstream_transcript_variant,intron_variant
rs1796598287	genic_upstream_transcript_variant,intron_variant
rs1796598892	genic_upstream_transcript_variant,intron_variant
rs1796599744	genic_upstream_transcript_variant,intron_variant
rs1796600138	genic_upstream_transcript_variant,intron_variant
rs1796600920	genic_upstream_transcript_variant,intron_variant
rs1796601516	genic_upstream_transcript_variant,intron_variant
rs1796602492	genic_upstream_transcript_variant,intron_variant
rs1796602923	genic_upstream_transcript_variant,intron_variant
rs1796603390	genic_upstream_transcript_variant,intron_variant
rs1796603611	genic_upstream_transcript_variant,intron_variant
rs1796603702	genic_upstream_transcript_variant,intron_variant
rs1796603805	genic_upstream_transcript_variant,intron_variant
rs1796604024	genic_upstream_transcript_variant,intron_variant
rs1796604134	genic_upstream_transcript_variant,intron_variant
rs1796604677	genic_upstream_transcript_variant,intron_variant
rs1796604792	genic_upstream_transcript_variant,intron_variant
rs1796604888	genic_upstream_transcript_variant,intron_variant
rs1796605483	genic_upstream_transcript_variant,intron_variant
rs1796605590	genic_upstream_transcript_variant,intron_variant
rs1796605813	genic_upstream_transcript_variant,intron_variant
rs1796606320	genic_upstream_transcript_variant,intron_variant
rs1796607056	genic_upstream_transcript_variant,intron_variant
rs1796607151	genic_upstream_transcript_variant,intron_variant
rs1796607241	genic_upstream_transcript_variant,intron_variant
rs1796607337	genic_upstream_transcript_variant,intron_variant
rs1796607727	genic_upstream_transcript_variant,intron_variant
rs1796609154	genic_upstream_transcript_variant,intron_variant
rs1796609253	genic_upstream_transcript_variant,intron_variant
rs1796609334	genic_upstream_transcript_variant,intron_variant
rs1796609928	genic_upstream_transcript_variant,intron_variant
rs1796610353	genic_upstream_transcript_variant,intron_variant
rs1796610882	genic_upstream_transcript_variant,intron_variant
rs1796611019	genic_upstream_transcript_variant,intron_variant
rs1796611296	genic_upstream_transcript_variant,intron_variant
rs1796612170	genic_upstream_transcript_variant,intron_variant

rs1796612420	genic_upstream_transcript_variant,intron_variant
rs1796612766	genic_upstream_transcript_variant,intron_variant
rs1796613252	genic_upstream_transcript_variant,intron_variant
rs1796613363	genic_upstream_transcript_variant,intron_variant
rs1796613469	genic_upstream_transcript_variant,intron_variant
rs1796614452	genic_upstream_transcript_variant,intron_variant
rs1796614860	genic_upstream_transcript_variant,intron_variant
rs1796615028	genic_upstream_transcript_variant,intron_variant
rs1796615209	genic_upstream_transcript_variant,intron_variant
rs1796615660	genic_upstream_transcript_variant,intron_variant
rs1796615745	genic_upstream_transcript_variant,intron_variant
rs1796615862	genic_upstream_transcript_variant,intron_variant
rs1796615934	genic_upstream_transcript_variant,intron_variant
rs1796616327	genic_upstream_transcript_variant,intron_variant
rs1796616735	genic_upstream_transcript_variant,intron_variant
rs1796616832	genic_upstream_transcript_variant,intron_variant
rs1796616942	genic_upstream_transcript_variant,intron_variant
rs1796617031	genic_upstream_transcript_variant,intron_variant
rs1796617409	genic_upstream_transcript_variant,intron_variant
rs1796617587	genic_upstream_transcript_variant,intron_variant
rs1796617786	genic_upstream_transcript_variant,intron_variant
rs1796618522	genic_upstream_transcript_variant,intron_variant
rs1796618752	genic_upstream_transcript_variant,intron_variant
rs1796618920	genic_upstream_transcript_variant,intron_variant
rs1796619506	genic_upstream_transcript_variant,intron_variant,non_coding_transcript_variant
rs1796620115	genic_upstream_transcript_variant,intron_variant,non_coding_transcript_variant
rs1796620576	genic_upstream_transcript_variant,intron_variant,non_coding_transcript_variant
rs1796620676	genic_upstream_transcript_variant,intron_variant,non_coding_transcript_variant
rs1796620764	genic_upstream_transcript_variant,intron_variant,non_coding_transcript_variant
rs1796620977	genic_upstream_transcript_variant,intron_variant,non_coding_transcript_variant
rs1796621064	genic_upstream_transcript_variant,intron_variant,non_coding_transcript_variant
rs1796621663	genic_upstream_transcript_variant,intron_variant,2KB_upstream_variant,upstream_transcript_variant
rs1796622007	genic_upstream_transcript_variant,intron_variant,2KB_upstream_variant,upstream_transcript_variant
rs1796622428	genic_upstream_transcript_variant,intron_variant,2KB_upstream_variant,upstream_transcript_variant
rs1796622649	genic_upstream_transcript_variant,intron_variant,2KB_upstream_variant,upstream_transcript_variant
rs1796622831	genic_upstream_transcript_variant,intron_variant,2KB_upstream_variant,upstream_transcript_variant
rs1796623061	genic_upstream_transcript_variant,intron_variant,2KB_upstream_variant,upstream_transcript_variant
rs1796623554	genic_upstream_transcript_variant,intron_variant,2KB_upstream_variant,upstream_transcript_variant
rs1796623863	genic_upstream_transcript_variant,intron_variant,2KB_upstream_variant,upstream_transcript_variant
rs1796624050	genic_upstream_transcript_variant,intron_variant,2KB_upstream_variant,upstream_transcript_variant
rs1796624333	genic_upstream_transcript_variant,intron_variant,2KB_upstream_variant,upstream_transcript_variant
rs1796624417	genic_upstream_transcript_variant,intron_variant,2KB_upstream_variant,upstream_transcript_variant
rs1796624926	genic_upstream_transcript_variant,intron_variant,2KB_upstream_variant,upstream_transcript_variant
rs1796625209	genic_upstream_transcript_variant,intron_variant,2KB_upstream_variant,upstream_transcript_variant
rs1796625294	genic_upstream_transcript_variant,intron_variant,2KB_upstream_variant,upstream_transcript_variant
rs1796625383	genic_upstream_transcript_variant,intron_variant,2KB_upstream_variant,upstream_transcript_variant
rs1796625557	genic_upstream_transcript_variant,intron_variant,2KB_upstream_variant,upstream_transcript_variant
rs1796625905	genic_upstream_transcript_variant,intron_variant,2KB_upstream_variant,upstream_transcript_variant
rs1796626003	genic_upstream_transcript_variant,intron_variant,2KB_upstream_variant,upstream_transcript_variant
rs1796626093	genic_upstream_transcript_variant,intron_variant,2KB_upstream_variant,upstream_transcript_variant
rs1796626488	genic_upstream_transcript_variant,intron_variant,2KB_upstream_variant,upstream_transcript_variant
rs1796626788	genic_upstream_transcript_variant,intron_variant,2KB_upstream_variant,upstream_transcript_variant
rs1796626990	genic_upstream_transcript_variant,intron_variant,2KB_upstream_variant,upstream_transcript_variant
rs1796627352	genic_upstream_transcript_variant,intron_variant,2KB_upstream_variant,upstream_transcript_variant
rs1796627444	genic_upstream_transcript_variant,intron_variant,2KB_upstream_variant,upstream_transcript_variant
rs1796627543	genic_upstream_transcript_variant,intron_variant,2KB_upstream_variant,upstream_transcript_variant
rs1796627644	genic_upstream_transcript_variant,intron_variant,2KB_upstream_variant,upstream_transcript_variant
rs1796627830	genic_upstream_transcript_variant,intron_variant,2KB_upstream_variant,upstream_transcript_variant
rs1796627915	genic_upstream_transcript_variant,intron_variant,2KB_upstream_variant,upstream_transcript_variant
rs1796627994	genic_upstream_transcript_variant,intron_variant,2KB_upstream_variant,upstream_transcript_variant
rs1796628201	genic_upstream_transcript_variant,intron_variant,2KB_upstream_variant,upstream_transcript_variant
rs1796628660	genic_upstream_transcript_variant,intron_variant,2KB_upstream_variant,upstream_transcript_variant
rs1796629161	genic_upstream_transcript_variant,intron_variant,2KB_upstream_variant,upstream_transcript_variant
rs1796629353	genic_upstream_transcript_variant,intron_variant,2KB_upstream_variant,upstream_transcript_variant
rs1796630544	genic_upstream_transcript_variant,intron_variant,2KB_upstream_variant,upstream_transcript_variant
rs1796630856	genic_upstream_transcript_variant,intron_variant,2KB_upstream_variant,upstream_transcript_variant
rs1796631214	genic_upstream_transcript_variant,intron_variant,2KB_upstream_variant,upstream_transcript_variant
rs1796631538	genic_upstream_transcript_variant,intron_variant,2KB_upstream_variant,upstream_transcript_variant
rs1796631871	genic_upstream_transcript_variant,intron_variant,2KB_upstream_variant,upstream_transcript_variant
rs1796632924	genic_upstream_transcript_variant,intron_variant,2KB_upstream_variant,upstream_transcript_variant
rs1796633313	genic_upstream_transcript_variant,intron_variant,2KB_upstream_variant,upstream_transcript_variant
rs1796633432	genic_upstream_transcript_variant,intron_variant,2KB_upstream_variant,upstream_transcript_variant
rs1796633858	genic_upstream_transcript_variant,intron_variant,2KB_upstream_variant,upstream_transcript_variant
rs1796650210	genic_upstream_transcript_variant,intron_variant,2KB_upstream_variant,upstream_transcript_variant

rs1796650329	genic_upstream_transcript_variant,intron_variant,2KB_upstream_variant,upstream_transcript_variant
rs1796650441	genic_upstream_transcript_variant,intron_variant,2KB_upstream_variant,upstream_transcript_variant
rs1796650562	genic_upstream_transcript_variant,intron_variant,2KB_upstream_variant,upstream_transcript_variant
rs1796651162	genic_upstream_transcript_variant,intron_variant,2KB_upstream_variant,upstream_transcript_variant
rs1796651444	genic_upstream_transcript_variant,intron_variant,2KB_upstream_variant,upstream_transcript_variant
rs1796651675	genic_upstream_transcript_variant,intron_variant,2KB_upstream_variant,upstream_transcript_variant
rs1796651934	genic_upstream_transcript_variant,intron_variant,2KB_upstream_variant,upstream_transcript_variant
rs1796652437	genic_upstream_transcript_variant,intron_variant,2KB_upstream_variant,upstream_transcript_variant
rs1796653517	genic_upstream_transcript_variant,intron_variant,2KB_upstream_variant,upstream_transcript_variant
rs1796653675	genic_upstream_transcript_variant,intron_variant,2KB_upstream_variant,upstream_transcript_variant
rs1796654161	genic_upstream_transcript_variant,intron_variant,2KB_upstream_variant,upstream_transcript_variant
rs1796654420	genic_upstream_transcript_variant,intron_variant,2KB_upstream_variant,upstream_transcript_variant
rs1796654525	genic_upstream_transcript_variant,intron_variant,2KB_upstream_variant,upstream_transcript_variant
rs1796654845	genic_upstream_transcript_variant,intron_variant,2KB_upstream_variant,upstream_transcript_variant
rs1796654963	genic_upstream_transcript_variant,intron_variant,2KB_upstream_variant,upstream_transcript_variant
rs1796655086	genic_upstream_transcript_variant,intron_variant,2KB_upstream_variant,upstream_transcript_variant
rs1796655453	genic_upstream_transcript_variant,intron_variant,2KB_upstream_variant,upstream_transcript_variant
rs1796655540	genic_upstream_transcript_variant,intron_variant,2KB_upstream_variant,upstream_transcript_variant
rs1796655660	genic_upstream_transcript_variant,intron_variant,2KB_upstream_variant,upstream_transcript_variant
rs1796655766	genic_upstream_transcript_variant,intron_variant,2KB_upstream_variant,upstream_transcript_variant
rs1796656203	genic_upstream_transcript_variant,intron_variant,2KB_upstream_variant,upstream_transcript_variant
rs1796656749	genic_upstream_transcript_variant,intron_variant,2KB_upstream_variant,upstream_transcript_variant
rs1796656997	genic_upstream_transcript_variant,intron_variant,2KB_upstream_variant,upstream_transcript_variant
rs1796657234	genic_upstream_transcript_variant,intron_variant,2KB_upstream_variant,upstream_transcript_variant
rs1796657766	genic_upstream_transcript_variant,intron_variant,2KB_upstream_variant,upstream_transcript_variant
rs1796658011	genic_upstream_transcript_variant,intron_variant,2KB_upstream_variant,upstream_transcript_variant
rs1796658109	genic_upstream_transcript_variant,intron_variant,2KB_upstream_variant,upstream_transcript_variant
rs1796658229	genic_upstream_transcript_variant,intron_variant,2KB_upstream_variant,upstream_transcript_variant
rs1796658722	genic_upstream_transcript_variant,intron_variant,2KB_upstream_variant,upstream_transcript_variant
rs1796658989	genic_upstream_transcript_variant,intron_variant,2KB_upstream_variant,upstream_transcript_variant
rs1796659310	genic_upstream_transcript_variant,intron_variant,2KB_upstream_variant,upstream_transcript_variant
rs1796659415	genic_upstream_transcript_variant,intron_variant,2KB_upstream_variant,upstream_transcript_variant
rs1796659513	genic_upstream_transcript_variant,intron_variant,2KB_upstream_variant,upstream_transcript_variant
rs1796660005	genic_upstream_transcript_variant,intron_variant,2KB_upstream_variant,upstream_transcript_variant
rs1796660246	genic_upstream_transcript_variant,intron_variant,2KB_upstream_variant,upstream_transcript_variant
rs1796660470	genic_upstream_transcript_variant,intron_variant,2KB_upstream_variant,upstream_transcript_variant
rs1796660608	genic_upstream_transcript_variant,intron_variant,2KB_upstream_variant,upstream_transcript_variant
rs1796661477	genic_upstream_transcript_variant,intron_variant,2KB_upstream_variant,upstream_transcript_variant
rs1796661735	genic_upstream_transcript_variant,intron_variant,2KB_upstream_variant,upstream_transcript_variant
rs1796661855	genic_upstream_transcript_variant,intron_variant,2KB_upstream_variant,upstream_transcript_variant
rs1796661974	genic_upstream_transcript_variant,intron_variant,2KB_upstream_variant,upstream_transcript_variant
rs1796662077	genic_upstream_transcript_variant,intron_variant,2KB_upstream_variant,upstream_transcript_variant
rs1796662185	genic_upstream_transcript_variant,intron_variant,2KB_upstream_variant,upstream_transcript_variant
rs1796662293	genic_upstream_transcript_variant,intron_variant,2KB_upstream_variant,upstream_transcript_variant
rs1796662405	genic_upstream_transcript_variant,intron_variant,2KB_upstream_variant,upstream_transcript_variant
rs1796662512	genic_upstream_transcript_variant,intron_variant,2KB_upstream_variant,upstream_transcript_variant
rs1796662776	genic_upstream_transcript_variant,intron_variant,2KB_upstream_variant,upstream_transcript_variant
rs1796663157	genic_upstream_transcript_variant,intron_variant,2KB_upstream_variant,upstream_transcript_variant
rs1796663648	genic_upstream_transcript_variant,intron_variant,2KB_upstream_variant,upstream_transcript_variant
rs1796663919	genic_upstream_transcript_variant,intron_variant,2KB_upstream_variant,upstream_transcript_variant
rs1796664415	genic_upstream_transcript_variant,intron_variant,2KB_upstream_variant,upstream_transcript_variant
rs1796664696	genic_upstream_transcript_variant,intron_variant,2KB_upstream_variant,upstream_transcript_variant
rs1796664814	genic_upstream_transcript_variant,intron_variant,2KB_upstream_variant,upstream_transcript_variant
rs1796665307	genic_upstream_transcript_variant,intron_variant,2KB_upstream_variant,upstream_transcript_variant
rs1796665732	genic_upstream_transcript_variant,intron_variant,2KB_upstream_variant,upstream_transcript_variant
rs1796666126	genic_upstream_transcript_variant,intron_variant,2KB_upstream_variant,upstream_transcript_variant
rs1796666615	genic_upstream_transcript_variant,intron_variant,2KB_upstream_variant,upstream_transcript_variant
rs1796666714	genic_upstream_transcript_variant,intron_variant,2KB_upstream_variant,upstream_transcript_variant
rs1796666823	genic_upstream_transcript_variant,intron_variant,2KB_upstream_variant,upstream_transcript_variant
rs1796667061	genic_upstream_transcript_variant,intron_variant,2KB_upstream_variant,upstream_transcript_variant
rs1796667402	genic_upstream_transcript_variant,intron_variant,2KB_upstream_variant,upstream_transcript_variant
rs1796667523	genic_upstream_transcript_variant,intron_variant,2KB_upstream_variant,upstream_transcript_variant
rs1796668252	genic_upstream_transcript_variant,intron_variant,2KB_upstream_variant,upstream_transcript_variant
rs1796668467	genic_upstream_transcript_variant,intron_variant,2KB_upstream_variant,upstream_transcript_variant
rs1796668565	genic_upstream_transcript_variant,intron_variant,2KB_upstream_variant,upstream_transcript_variant
rs1796669017	genic_upstream_transcript_variant,intron_variant,2KB_upstream_variant,upstream_transcript_variant
rs1796669358	genic_upstream_transcript_variant,intron_variant,2KB_upstream_variant,upstream_transcript_variant
rs1796669965	genic_upstream_transcript_variant,intron_variant,2KB_upstream_variant,upstream_transcript_variant
rs1796670266	genic_upstream_transcript_variant,intron_variant,2KB_upstream_variant,upstream_transcript_variant
rs1796670511	genic_upstream_transcript_variant,intron_variant,2KB_upstream_variant,upstream_transcript_variant
rs1796670772	genic_upstream_transcript_variant,intron_variant,2KB_upstream_variant,upstream_transcript_variant
rs1796671263	genic_upstream_transcript_variant,intron_variant,2KB_upstream_variant,upstream_transcript_variant
rs1796671630	genic_upstream_transcript_variant,intron_variant,2KB_upstream_variant,upstream_transcript_variant
rs1796671790	genic_upstream_transcript_variant,intron_variant,2KB_upstream_variant,upstream_transcript_variant

rs1796672007	genic_upstream_transcript_variant,intron_variant,2KB_upstream_variant,upstream_transcript_variant
rs1796673105	genic_upstream_transcript_variant,intron_variant,2KB_upstream_variant,upstream_transcript_variant
rs1796673809	genic_upstream_transcript_variant,intron_variant,2KB_upstream_variant,upstream_transcript_variant
rs1796673968	genic_upstream_transcript_variant,intron_variant,2KB_upstream_variant,upstream_transcript_variant
rs1796674135	genic_upstream_transcript_variant,intron_variant,2KB_upstream_variant,upstream_transcript_variant
rs1796674480	genic_upstream_transcript_variant,intron_variant,2KB_upstream_variant,upstream_transcript_variant
rs1796674628	genic_upstream_transcript_variant,intron_variant,2KB_upstream_variant,upstream_transcript_variant
rs1796674964	genic_upstream_transcript_variant,intron_variant,2KB_upstream_variant,upstream_transcript_variant
rs1796675352	genic_upstream_transcript_variant,intron_variant,2KB_upstream_variant,upstream_transcript_variant
rs1796675574	genic_upstream_transcript_variant,intron_variant,2KB_upstream_variant,upstream_transcript_variant
rs1796675672	genic_upstream_transcript_variant,intron_variant,2KB_upstream_variant,upstream_transcript_variant
rs1796675880	genic_upstream_transcript_variant,intron_variant,2KB_upstream_variant,upstream_transcript_variant
rs1796676092	genic_upstream_transcript_variant,intron_variant,2KB_upstream_variant,upstream_transcript_variant
rs1796676267	genic_upstream_transcript_variant,intron_variant,2KB_upstream_variant,upstream_transcript_variant
rs1796676384	genic_upstream_transcript_variant,intron_variant,2KB_upstream_variant,upstream_transcript_variant
rs1796676570	genic_upstream_transcript_variant,intron_variant,2KB_upstream_variant,upstream_transcript_variant
rs1796676675	genic_upstream_transcript_variant,intron_variant,2KB_upstream_variant,upstream_transcript_variant
rs1796677046	genic_upstream_transcript_variant,intron_variant,2KB_upstream_variant,upstream_transcript_variant
rs1796677264	genic_upstream_transcript_variant,intron_variant,2KB_upstream_variant,upstream_transcript_variant
rs1796677376	genic_upstream_transcript_variant,intron_variant,2KB_upstream_variant,upstream_transcript_variant
rs1796677673	genic_upstream_transcript_variant,intron_variant,2KB_upstream_variant,upstream_transcript_variant
rs1796678083	genic_upstream_transcript_variant,intron_variant,2KB_upstream_variant,upstream_transcript_variant
rs1796678751	genic_upstream_transcript_variant,intron_variant,2KB_upstream_variant,upstream_transcript_variant
rs1796679320	genic_upstream_transcript_variant,intron_variant,2KB_upstream_variant,upstream_transcript_variant
rs1796679596	genic_upstream_transcript_variant,intron_variant,2KB_upstream_variant,upstream_transcript_variant
rs1796679694	genic_upstream_transcript_variant,intron_variant,2KB_upstream_variant,upstream_transcript_variant
rs1796680062	genic_upstream_transcript_variant,intron_variant,2KB_upstream_variant,upstream_transcript_variant
rs1796680327	genic_upstream_transcript_variant,intron_variant,2KB_upstream_variant,upstream_transcript_variant
rs1796680422	genic_upstream_transcript_variant,intron_variant,2KB_upstream_variant,upstream_transcript_variant
rs1796680763	genic_upstream_transcript_variant,intron_variant,2KB_upstream_variant,upstream_transcript_variant
rs1796681478	genic_upstream_transcript_variant,intron_variant,2KB_upstream_variant,upstream_transcript_variant
rs1796681935	genic_upstream_transcript_variant,intron_variant,2KB_upstream_variant,upstream_transcript_variant
rs1796682028	genic_upstream_transcript_variant,intron_variant,2KB_upstream_variant,upstream_transcript_variant
rs1796682310	genic_upstream_transcript_variant,intron_variant,2KB_upstream_variant,upstream_transcript_variant
rs1796683216	genic_upstream_transcript_variant,intron_variant,2KB_upstream_variant,upstream_transcript_variant
rs1796683344	genic_upstream_transcript_variant,intron_variant,2KB_upstream_variant,upstream_transcript_variant
rs1796683978	genic_upstream_transcript_variant,intron_variant,2KB_upstream_variant,upstream_transcript_variant
rs1796684067	genic_upstream_transcript_variant,intron_variant,2KB_upstream_variant,upstream_transcript_variant
rs1796684266	genic_upstream_transcript_variant,intron_variant,2KB_upstream_variant,upstream_transcript_variant
rs1796684360	genic_upstream_transcript_variant,intron_variant,2KB_upstream_variant,upstream_transcript_variant
rs1796685043	genic_upstream_transcript_variant,intron_variant,2KB_upstream_variant,upstream_transcript_variant
rs1796685156	genic_upstream_transcript_variant,intron_variant,2KB_upstream_variant,upstream_transcript_variant
rs1796686238	genic_upstream_transcript_variant,intron_variant,2KB_upstream_variant,upstream_transcript_variant
rs1796686681	genic_upstream_transcript_variant,intron_variant,2KB_upstream_variant,upstream_transcript_variant
rs1796686890	genic_upstream_transcript_variant,intron_variant,2KB_upstream_variant,upstream_transcript_variant
rs1796687117	genic_upstream_transcript_variant,intron_variant,2KB_upstream_variant,upstream_transcript_variant
rs1796687762	genic_upstream_transcript_variant,intron_variant,2KB_upstream_variant,upstream_transcript_variant
rs1796687857	genic_upstream_transcript_variant,intron_variant,2KB_upstream_variant,upstream_transcript_variant
rs1796687962	genic_upstream_transcript_variant,intron_variant,2KB_upstream_variant,upstream_transcript_variant
rs1796688062	genic_upstream_transcript_variant,intron_variant,2KB_upstream_variant,upstream_transcript_variant
rs1796688578	genic_upstream_transcript_variant,intron_variant,2KB_upstream_variant,upstream_transcript_variant
rs1796688758	genic_upstream_transcript_variant,intron_variant,2KB_upstream_variant,upstream_transcript_variant
rs1796688852	genic_upstream_transcript_variant,intron_variant,2KB_upstream_variant,upstream_transcript_variant
rs1796689486	genic_upstream_transcript_variant,intron_variant,2KB_upstream_variant,upstream_transcript_variant
rs1796689775	genic_upstream_transcript_variant,intron_variant,2KB_upstream_variant,upstream_transcript_variant
rs1796691296	genic_upstream_transcript_variant,intron_variant,2KB_upstream_variant,upstream_transcript_variant
rs1796691485	genic_upstream_transcript_variant,intron_variant,2KB_upstream_variant,upstream_transcript_variant
rs1796691586	genic_upstream_transcript_variant,intron_variant,2KB_upstream_variant,upstream_transcript_variant
rs1796691688	genic_upstream_transcript_variant,intron_variant,2KB_upstream_variant,upstream_transcript_variant
rs1796692379	genic_upstream_transcript_variant,intron_variant,2KB_upstream_variant,upstream_transcript_variant
rs1796692503	genic_upstream_transcript_variant,intron_variant,2KB_upstream_variant,upstream_transcript_variant
rs1796692708	genic_upstream_transcript_variant,intron_variant,2KB_upstream_variant,upstream_transcript_variant
rs1796693757	genic_upstream_transcript_variant,intron_variant,2KB_upstream_variant,upstream_transcript_variant
rs1796693982	genic_upstream_transcript_variant,intron_variant,2KB_upstream_variant,upstream_transcript_variant
rs1796694162	genic_upstream_transcript_variant,intron_variant,2KB_upstream_variant,upstream_transcript_variant
rs1796694495	genic_upstream_transcript_variant,intron_variant,2KB_upstream_variant,upstream_transcript_variant
rs1796694951	genic_upstream_transcript_variant,intron_variant,2KB_upstream_variant,upstream_transcript_variant
rs1796695266	genic_upstream_transcript_variant,intron_variant,2KB_upstream_variant,upstream_transcript_variant
rs1796695387	genic_upstream_transcript_variant,intron_variant,2KB_upstream_variant,upstream_transcript_variant
rs1796695485	genic_upstream_transcript_variant,intron_variant,2KB_upstream_variant,upstream_transcript_variant
rs1796695584	genic_upstream_transcript_variant,intron_variant,2KB_upstream_variant,upstream_transcript_variant
rs1796695932	genic_upstream_transcript_variant,intron_variant,2KB_upstream_variant,upstream_transcript_variant
rs1796696470	genic_upstream_transcript_variant,intron_variant,2KB_upstream_variant,upstream_transcript_variant
rs1796696568	genic_upstream_transcript_variant,intron_variant,2KB_upstream_variant,upstream_transcript_variant

rs1796696972	genic_upstream_transcript_variant,intron_variant,2KB_upstream_variant,upstream_transcript_variant
rs1796697965	genic_upstream_transcript_variant,intron_variant,2KB_upstream_variant,upstream_transcript_variant
rs1796698066	genic_upstream_transcript_variant,intron_variant,2KB_upstream_variant,upstream_transcript_variant
rs1796700175	genic_upstream_transcript_variant,intron_variant,2KB_upstream_variant,upstream_transcript_variant
rs1796701064	genic_upstream_transcript_variant,intron_variant,2KB_upstream_variant,upstream_transcript_variant
rs1796701174	genic_upstream_transcript_variant,intron_variant,2KB_upstream_variant,upstream_transcript_variant
rs1796701733	genic_upstream_transcript_variant,intron_variant,2KB_upstream_variant,upstream_transcript_variant
rs1796701840	genic_upstream_transcript_variant,intron_variant,2KB_upstream_variant,upstream_transcript_variant
rs1796702053	genic_upstream_transcript_variant,intron_variant,2KB_upstream_variant,upstream_transcript_variant
rs1796702137	genic_upstream_transcript_variant,intron_variant,2KB_upstream_variant,upstream_transcript_variant
rs1796702228	genic_upstream_transcript_variant,intron_variant,2KB_upstream_variant,upstream_transcript_variant
rs1796702308	genic_upstream_transcript_variant,intron_variant,2KB_upstream_variant,upstream_transcript_variant
rs1796702412	genic_upstream_transcript_variant,intron_variant,2KB_upstream_variant,upstream_transcript_variant
rs1796702685	genic_upstream_transcript_variant,intron_variant,2KB_upstream_variant,upstream_transcript_variant
rs1796703069	genic_upstream_transcript_variant,intron_variant,2KB_upstream_variant,upstream_transcript_variant
rs1796703280	genic_upstream_transcript_variant,intron_variant,2KB_upstream_variant,upstream_transcript_variant
rs1796703604	genic_upstream_transcript_variant,intron_variant,2KB_upstream_variant,upstream_transcript_variant
rs1796703817	genic_upstream_transcript_variant,intron_variant,2KB_upstream_variant,upstream_transcript_variant
rs1796704035	genic_upstream_transcript_variant,intron_variant,2KB_upstream_variant,upstream_transcript_variant
rs1796704493	genic_upstream_transcript_variant,intron_variant,2KB_upstream_variant,upstream_transcript_variant
rs1796704594	genic_upstream_transcript_variant,intron_variant,2KB_upstream_variant,upstream_transcript_variant
rs1796704716	genic_upstream_transcript_variant,intron_variant,2KB_upstream_variant,upstream_transcript_variant
rs1796704819	genic_upstream_transcript_variant,intron_variant,2KB_upstream_variant,upstream_transcript_variant
rs1796705274	genic_upstream_transcript_variant,intron_variant,2KB_upstream_variant,upstream_transcript_variant
rs1796706098	genic_upstream_transcript_variant,intron_variant,2KB_upstream_variant,upstream_transcript_variant
rs1796707234	genic_upstream_transcript_variant,intron_variant,2KB_upstream_variant,upstream_transcript_variant
rs1796707497	genic_upstream_transcript_variant,intron_variant,2KB_upstream_variant,upstream_transcript_variant
rs1796707941	genic_upstream_transcript_variant,intron_variant,2KB_upstream_variant,upstream_transcript_variant
rs1796708039	genic_upstream_transcript_variant,intron_variant,2KB_upstream_variant,upstream_transcript_variant
rs1796708147	genic_upstream_transcript_variant,intron_variant,2KB_upstream_variant,upstream_transcript_variant
rs1796708744	genic_upstream_transcript_variant,intron_variant,2KB_upstream_variant,upstream_transcript_variant
rs1796708885	genic_upstream_transcript_variant,intron_variant,2KB_upstream_variant,upstream_transcript_variant
rs1796709037	genic_upstream_transcript_variant,intron_variant,2KB_upstream_variant,upstream_transcript_variant
rs1796709445	genic_upstream_transcript_variant,intron_variant,2KB_upstream_variant,upstream_transcript_variant
rs1796709688	genic_upstream_transcript_variant,intron_variant,2KB_upstream_variant,upstream_transcript_variant
rs1796710108	genic_upstream_transcript_variant,intron_variant,2KB_upstream_variant,upstream_transcript_variant
rs1796710333	genic_upstream_transcript_variant,intron_variant,2KB_upstream_variant,upstream_transcript_variant
rs1796711021	genic_upstream_transcript_variant,intron_variant,2KB_upstream_variant,upstream_transcript_variant
rs1796711763	genic_upstream_transcript_variant,intron_variant,2KB_upstream_variant,upstream_transcript_variant
rs1796711858	genic_upstream_transcript_variant,intron_variant,2KB_upstream_variant,upstream_transcript_variant
rs1796712419	genic_upstream_transcript_variant,intron_variant,2KB_upstream_variant,upstream_transcript_variant
rs1796712512	genic_upstream_transcript_variant,intron_variant,2KB_upstream_variant,upstream_transcript_variant
rs1796712619	genic_upstream_transcript_variant,intron_variant,2KB_upstream_variant,upstream_transcript_variant
rs1796713125	genic_upstream_transcript_variant,intron_variant,2KB_upstream_variant,upstream_transcript_variant
rs1796713615	genic_upstream_transcript_variant,intron_variant,2KB_upstream_variant,upstream_transcript_variant
rs1796713827	genic_upstream_transcript_variant,intron_variant,2KB_upstream_variant,upstream_transcript_variant
rs1796713917	genic_upstream_transcript_variant,intron_variant,2KB_upstream_variant,upstream_transcript_variant
rs1796714164	genic_upstream_transcript_variant,intron_variant,2KB_upstream_variant,upstream_transcript_variant
rs1796714273	genic_upstream_transcript_variant,intron_variant,2KB_upstream_variant,upstream_transcript_variant
rs1796714379	genic_upstream_transcript_variant,intron_variant,2KB_upstream_variant,upstream_transcript_variant
rs1796714537	genic_upstream_transcript_variant,intron_variant,2KB_upstream_variant,upstream_transcript_variant
rs1796715809	genic_upstream_transcript_variant,intron_variant,2KB_upstream_variant,upstream_transcript_variant
rs1796716384	genic_upstream_transcript_variant,intron_variant,2KB_upstream_variant,upstream_transcript_variant
rs1796716540	genic_upstream_transcript_variant,intron_variant,2KB_upstream_variant,upstream_transcript_variant
rs1796717522	genic_upstream_transcript_variant,intron_variant,2KB_upstream_variant,upstream_transcript_variant
rs1796717638	genic_upstream_transcript_variant,intron_variant,2KB_upstream_variant,upstream_transcript_variant
rs1796717821	genic_upstream_transcript_variant,intron_variant,2KB_upstream_variant,upstream_transcript_variant
rs1796717920	genic_upstream_transcript_variant,intron_variant,2KB_upstream_variant,upstream_transcript_variant
rs1796718495	genic_upstream_transcript_variant,intron_variant,2KB_upstream_variant,upstream_transcript_variant
rs1796718641	genic_upstream_transcript_variant,intron_variant,2KB_upstream_variant,upstream_transcript_variant
rs1796719846	genic_upstream_transcript_variant,intron_variant,2KB_upstream_variant,upstream_transcript_variant
rs1796720529	genic_upstream_transcript_variant,intron_variant,2KB_upstream_variant,upstream_transcript_variant
rs1796721676	genic_upstream_transcript_variant,intron_variant,2KB_upstream_variant,upstream_transcript_variant
rs1796721807	genic_upstream_transcript_variant,intron_variant,2KB_upstream_variant,upstream_transcript_variant
rs1796722188	genic_upstream_transcript_variant,intron_variant,2KB_upstream_variant,upstream_transcript_variant
rs1796722662	genic_upstream_transcript_variant,intron_variant,2KB_upstream_variant,upstream_transcript_variant
rs1796722821	genic_upstream_transcript_variant,intron_variant,2KB_upstream_variant,upstream_transcript_variant
rs1796722960	genic_upstream_transcript_variant,intron_variant,2KB_upstream_variant,upstream_transcript_variant
rs1796723354	genic_upstream_transcript_variant,intron_variant,2KB_upstream_variant,upstream_transcript_variant
rs1796724235	genic_upstream_transcript_variant,intron_variant,2KB_upstream_variant,upstream_transcript_variant
rs1796724377	genic_upstream_transcript_variant,intron_variant
rs1796724931	genic_upstream_transcript_variant,intron_variant
rs1796725028	genic_upstream_transcript_variant,intron_variant
rs1796725380	genic_upstream_transcript_variant,intron_variant

rs1796725486	genic_upstream_transcript_variant,intron_variant
rs1796725603	genic_upstream_transcript_variant,intron_variant
rs1796725715	genic_upstream_transcript_variant,intron_variant
rs1796725932	genic_upstream_transcript_variant,intron_variant
rs1796726376	genic_upstream_transcript_variant,intron_variant
rs1796728247	genic_upstream_transcript_variant,intron_variant
rs1796729483	genic_upstream_transcript_variant,intron_variant
rs1796730399	genic_upstream_transcript_variant,intron_variant
rs1796730499	genic_upstream_transcript_variant,intron_variant
rs1796730631	genic_upstream_transcript_variant,intron_variant
rs1796730751	genic_upstream_transcript_variant,intron_variant
rs1796731272	genic_upstream_transcript_variant,intron_variant
rs1796731407	genic_upstream_transcript_variant,intron_variant
rs1796731854	genic_upstream_transcript_variant,intron_variant
rs1796732411	genic_upstream_transcript_variant,intron_variant
rs1796732713	genic_upstream_transcript_variant,intron_variant
rs1796732991	genic_upstream_transcript_variant,intron_variant
rs1796733295	genic_upstream_transcript_variant,intron_variant
rs1796733508	genic_upstream_transcript_variant,intron_variant
rs1796733756	genic_upstream_transcript_variant,intron_variant
rs1796733881	genic_upstream_transcript_variant,intron_variant
rs1796734109	genic_upstream_transcript_variant,intron_variant
rs1796734308	genic_upstream_transcript_variant,intron_variant
rs1796734871	genic_upstream_transcript_variant,intron_variant
rs1796735092	genic_upstream_transcript_variant,intron_variant
rs1796735179	genic_upstream_transcript_variant,intron_variant
rs1796735668	genic_upstream_transcript_variant,intron_variant
rs1796735775	genic_upstream_transcript_variant,intron_variant
rs1796736121	genic_upstream_transcript_variant,intron_variant
rs1796736242	genic_upstream_transcript_variant,intron_variant
rs1796736457	genic_upstream_transcript_variant,intron_variant
rs1796736786	genic_upstream_transcript_variant,intron_variant
rs1796737085	genic_upstream_transcript_variant,intron_variant
rs1796737519	genic_upstream_transcript_variant,intron_variant
rs1796737856	genic_upstream_transcript_variant,intron_variant
rs1796738007	genic_upstream_transcript_variant,intron_variant
rs1796738415	genic_upstream_transcript_variant,intron_variant
rs1796738532	genic_upstream_transcript_variant,intron_variant
rs1796738794	genic_upstream_transcript_variant,intron_variant
rs1796738914	genic_upstream_transcript_variant,intron_variant
rs1796740867	genic_upstream_transcript_variant,intron_variant
rs1796741354	genic_upstream_transcript_variant,intron_variant
rs1796741460	genic_upstream_transcript_variant,intron_variant
rs1796741950	genic_upstream_transcript_variant,intron_variant
rs1796742046	genic_upstream_transcript_variant,intron_variant
rs1796742157	genic_upstream_transcript_variant,intron_variant
rs1796742247	genic_upstream_transcript_variant,intron_variant
rs1796742342	genic_upstream_transcript_variant,intron_variant
rs1796742634	genic_upstream_transcript_variant,intron_variant
rs1796742823	genic_upstream_transcript_variant,intron_variant
rs1796743137	genic_upstream_transcript_variant,intron_variant
rs1796743591	genic_upstream_transcript_variant,intron_variant
rs1796743796	genic_upstream_transcript_variant,intron_variant
rs1796744982	genic_upstream_transcript_variant,intron_variant
rs1796745066	genic_upstream_transcript_variant,intron_variant
rs1796745357	genic_upstream_transcript_variant,intron_variant
rs1796745468	genic_upstream_transcript_variant,intron_variant
rs1796745799	genic_upstream_transcript_variant,intron_variant
rs1796746489	genic_upstream_transcript_variant,intron_variant
rs1796747188	genic_upstream_transcript_variant,intron_variant
rs1796747362	genic_upstream_transcript_variant,intron_variant
rs1796748402	genic_upstream_transcript_variant,intron_variant
rs1796748865	genic_upstream_transcript_variant,intron_variant
rs1796749052	genic_upstream_transcript_variant,intron_variant
rs1796749135	genic_upstream_transcript_variant,intron_variant
rs1796749327	genic_upstream_transcript_variant,intron_variant
rs1796749532	genic_upstream_transcript_variant,intron_variant
rs1796749719	genic_upstream_transcript_variant,intron_variant
rs1796749813	genic_upstream_transcript_variant,intron_variant
rs1796750201	genic_upstream_transcript_variant,intron_variant
rs1796750315	genic_upstream_transcript_variant,intron_variant
rs1796750742	genic_upstream_transcript_variant,intron_variant
rs1796751412	genic_upstream_transcript_variant,intron_variant
rs1796751533	genic_upstream_transcript_variant,intron_variant

rs1796751855	genic_upstream_transcript_variant,intron_variant
rs1796752349	genic_upstream_transcript_variant,intron_variant
rs1796752517	genic_upstream_transcript_variant,intron_variant
rs1796752773	genic_upstream_transcript_variant,intron_variant
rs1796752914	genic_upstream_transcript_variant,intron_variant
rs1796753725	genic_upstream_transcript_variant,intron_variant
rs1796753887	genic_upstream_transcript_variant,intron_variant
rs1796754481	genic_upstream_transcript_variant,intron_variant
rs1796755259	genic_upstream_transcript_variant,intron_variant
rs1796755751	genic_upstream_transcript_variant,intron_variant
rs1796757218	genic_upstream_transcript_variant,intron_variant
rs1796757790	genic_upstream_transcript_variant,intron_variant
rs1796757871	genic_upstream_transcript_variant,intron_variant
rs1796758438	genic_upstream_transcript_variant,intron_variant
rs1796758540	genic_upstream_transcript_variant,intron_variant
rs1796758631	genic_upstream_transcript_variant,intron_variant
rs1796758723	genic_upstream_transcript_variant,intron_variant
rs1796760231	genic_upstream_transcript_variant,intron_variant
rs1796760327	genic_upstream_transcript_variant,intron_variant
rs1796760629	genic_upstream_transcript_variant,intron_variant
rs1796760709	genic_upstream_transcript_variant,intron_variant
rs1796760799	genic_upstream_transcript_variant,intron_variant
rs1796760885	genic_upstream_transcript_variant,intron_variant
rs1796760966	genic_upstream_transcript_variant,intron_variant
rs1796761051	genic_upstream_transcript_variant,intron_variant
rs1796761144	genic_upstream_transcript_variant,intron_variant
rs1796761535	genic_upstream_transcript_variant,intron_variant
rs1796761629	genic_upstream_transcript_variant,intron_variant
rs1796761833	genic_upstream_transcript_variant,intron_variant
rs1796761926	genic_upstream_transcript_variant,intron_variant
rs1796762005	genic_upstream_transcript_variant,intron_variant
rs1796762277	genic_upstream_transcript_variant,intron_variant
rs1796762367	genic_upstream_transcript_variant,intron_variant
rs1796762548	genic_upstream_transcript_variant,intron_variant
rs1796763246	genic_upstream_transcript_variant,intron_variant
rs1796763674	genic_upstream_transcript_variant,intron_variant
rs1796763874	genic_upstream_transcript_variant,intron_variant
rs1796763953	genic_upstream_transcript_variant,intron_variant
rs1796764039	genic_upstream_transcript_variant,intron_variant
rs1796764118	genic_upstream_transcript_variant,intron_variant
rs1796764187	genic_upstream_transcript_variant,intron_variant
rs1796765160	genic_upstream_transcript_variant,intron_variant
rs1796765267	genic_upstream_transcript_variant,intron_variant
rs1796765653	genic_upstream_transcript_variant,intron_variant
rs1796765914	genic_upstream_transcript_variant,intron_variant
rs1796766374	genic_upstream_transcript_variant,intron_variant
rs1796766538	genic_upstream_transcript_variant,intron_variant
rs1796767132	genic_upstream_transcript_variant,intron_variant
rs1796767391	genic_upstream_transcript_variant,intron_variant
rs1796767863	genic_upstream_transcript_variant,intron_variant
rs1796767950	genic_upstream_transcript_variant,intron_variant
rs1796768361	genic_upstream_transcript_variant,intron_variant
rs1796768647	genic_upstream_transcript_variant,intron_variant
rs1796768829	genic_upstream_transcript_variant,intron_variant
rs1796768905	genic_upstream_transcript_variant,intron_variant
rs1796769009	genic_upstream_transcript_variant,intron_variant
rs1796769169	genic_upstream_transcript_variant,intron_variant
rs1796769333	genic_upstream_transcript_variant,intron_variant
rs1796769796	genic_upstream_transcript_variant,intron_variant
rs1796770861	genic_upstream_transcript_variant,intron_variant
rs1796771058	genic_upstream_transcript_variant,intron_variant
rs1796771160	genic_upstream_transcript_variant,intron_variant
rs1796771417	genic_upstream_transcript_variant,intron_variant
rs1796771775	genic_upstream_transcript_variant,intron_variant
rs1796771856	genic_upstream_transcript_variant,intron_variant
rs1796771947	genic_upstream_transcript_variant,intron_variant
rs1796772044	genic_upstream_transcript_variant,intron_variant
rs1796772112	genic_upstream_transcript_variant,intron_variant
rs1796774096	genic_upstream_transcript_variant,intron_variant
rs1796774192	genic_upstream_transcript_variant,intron_variant
rs1796774546	genic_upstream_transcript_variant,intron_variant
rs1796774709	genic_upstream_transcript_variant,intron_variant
rs1796774971	genic_upstream_transcript_variant,intron_variant
rs1796775845	genic_upstream_transcript_variant,intron_variant

rs1796775925	genic_upstream_transcript_variant,intron_variant
rs1796776092	genic_upstream_transcript_variant,intron_variant
rs1796776276	genic_upstream_transcript_variant,intron_variant
rs1796776363	genic_upstream_transcript_variant,intron_variant
rs1796776441	genic_upstream_transcript_variant,intron_variant
rs1796776649	genic_upstream_transcript_variant,intron_variant
rs1796777243	genic_upstream_transcript_variant,intron_variant
rs1796777318	genic_upstream_transcript_variant,intron_variant
rs1796777662	genic_upstream_transcript_variant,intron_variant
rs1796777944	genic_upstream_transcript_variant,intron_variant
rs1796778058	genic_upstream_transcript_variant,intron_variant
rs1796778339	genic_upstream_transcript_variant,intron_variant
rs1796778502	genic_upstream_transcript_variant,intron_variant
rs1796778584	genic_upstream_transcript_variant,intron_variant
rs1796779116	genic_upstream_transcript_variant,intron_variant
rs1796779204	genic_upstream_transcript_variant,intron_variant
rs1796779292	genic_upstream_transcript_variant,intron_variant
rs1796779764	genic_upstream_transcript_variant,intron_variant
rs1796780034	genic_upstream_transcript_variant,intron_variant
rs1796780260	genic_upstream_transcript_variant,intron_variant
rs1796780345	genic_upstream_transcript_variant,intron_variant
rs1796780505	genic_upstream_transcript_variant,intron_variant
rs1796780582	genic_upstream_transcript_variant,intron_variant
rs1796780798	genic_upstream_transcript_variant,intron_variant
rs1796781719	genic_upstream_transcript_variant,intron_variant
rs1796781994	genic_upstream_transcript_variant,intron_variant
rs1796782249	genic_upstream_transcript_variant,intron_variant
rs1796782542	genic_upstream_transcript_variant,intron_variant
rs1796782719	genic_upstream_transcript_variant,intron_variant
rs1796783068	genic_upstream_transcript_variant,intron_variant
rs1796783223	genic_upstream_transcript_variant,intron_variant
rs1796783393	genic_upstream_transcript_variant,intron_variant
rs1796783558	genic_upstream_transcript_variant,intron_variant
rs1796783665	genic_upstream_transcript_variant,intron_variant
rs1796783758	genic_upstream_transcript_variant,intron_variant
rs1796784048	genic_upstream_transcript_variant,intron_variant
rs1796785891	genic_upstream_transcript_variant,intron_variant
rs1796785988	genic_upstream_transcript_variant,intron_variant
rs1796786204	genic_upstream_transcript_variant,intron_variant
rs1796786512	genic_upstream_transcript_variant,intron_variant
rs1796786697	genic_upstream_transcript_variant,intron_variant
rs1796786771	genic_upstream_transcript_variant,intron_variant
rs1796786953	genic_upstream_transcript_variant,intron_variant
rs1796787266	genic_upstream_transcript_variant,intron_variant
rs1796787356	genic_upstream_transcript_variant,intron_variant
rs1796787786	genic_upstream_transcript_variant,intron_variant
rs1796788090	genic_upstream_transcript_variant,intron_variant
rs1796789480	genic_upstream_transcript_variant,intron_variant
rs1796789647	genic_upstream_transcript_variant,intron_variant
rs1796789731	genic_upstream_transcript_variant,intron_variant
rs1796790624	genic_upstream_transcript_variant,intron_variant
rs1796790989	genic_upstream_transcript_variant,intron_variant
rs1796791091	genic_upstream_transcript_variant,intron_variant
rs1796791191	genic_upstream_transcript_variant,intron_variant
rs1796791384	genic_upstream_transcript_variant,intron_variant
rs1796791572	genic_upstream_transcript_variant,intron_variant
rs1796791644	genic_upstream_transcript_variant,intron_variant
rs1796791865	genic_upstream_transcript_variant,intron_variant
rs1796792320	genic_upstream_transcript_variant,intron_variant
rs1796792412	genic_upstream_transcript_variant,intron_variant
rs1796792580	genic_upstream_transcript_variant,intron_variant
rs1796792687	genic_upstream_transcript_variant,intron_variant
rs1796792901	genic_upstream_transcript_variant,intron_variant
rs1796793847	genic_upstream_transcript_variant,intron_variant
rs1796794012	genic_upstream_transcript_variant,intron_variant
rs1796796000	genic_upstream_transcript_variant,intron_variant
rs1796796471	genic_upstream_transcript_variant,intron_variant
rs1796796776	genic_upstream_transcript_variant,intron_variant
rs1796796869	genic_upstream_transcript_variant,intron_variant
rs1796796957	genic_upstream_transcript_variant,intron_variant
rs1796798093	genic_upstream_transcript_variant,intron_variant
rs1796798173	genic_upstream_transcript_variant,intron_variant
rs1796798267	genic_upstream_transcript_variant,intron_variant
rs1796798507	genic_upstream_transcript_variant,intron_variant

rs1796799303	genic_upstream_transcript_variant,intron_variant
rs1796799555	genic_upstream_transcript_variant,intron_variant
rs1796799998	genic_upstream_transcript_variant,intron_variant
rs1796800165	genic_upstream_transcript_variant,intron_variant
rs1796801025	genic_upstream_transcript_variant,intron_variant
rs1796801285	genic_upstream_transcript_variant,intron_variant
rs1796801467	genic_upstream_transcript_variant,intron_variant
rs1796801702	genic_upstream_transcript_variant,intron_variant
rs1796801873	genic_upstream_transcript_variant,intron_variant
rs1796801969	genic_upstream_transcript_variant,intron_variant
rs1796802804	genic_upstream_transcript_variant,intron_variant
rs1796803926	genic_upstream_transcript_variant,intron_variant
rs1796804287	genic_upstream_transcript_variant,intron_variant
rs1796804512	genic_upstream_transcript_variant,intron_variant
rs1796804939	genic_upstream_transcript_variant,intron_variant
rs1796805430	genic_upstream_transcript_variant,intron_variant
rs1796805602	genic_upstream_transcript_variant,intron_variant
rs1796805690	genic_upstream_transcript_variant,intron_variant
rs1796806542	genic_upstream_transcript_variant,intron_variant
rs1796806629	genic_upstream_transcript_variant,intron_variant
rs1796807295	genic_upstream_transcript_variant,intron_variant
rs1796807376	genic_upstream_transcript_variant,intron_variant
rs1796808205	genic_upstream_transcript_variant,intron_variant
rs1796808304	genic_upstream_transcript_variant,intron_variant
rs1796808659	genic_upstream_transcript_variant,intron_variant
rs1796808743	genic_upstream_transcript_variant,intron_variant
rs1796809195	genic_upstream_transcript_variant,intron_variant
rs1796809424	genic_upstream_transcript_variant,intron_variant
rs1796809497	genic_upstream_transcript_variant,intron_variant
rs1796810169	genic_upstream_transcript_variant,intron_variant
rs1796811125	genic_upstream_transcript_variant,intron_variant
rs1796811206	genic_upstream_transcript_variant,intron_variant
rs1796811289	genic_upstream_transcript_variant,intron_variant
rs1796812447	genic_upstream_transcript_variant,intron_variant
rs1796813381	genic_upstream_transcript_variant,intron_variant
rs1796813915	genic_upstream_transcript_variant,intron_variant
rs1796814203	genic_upstream_transcript_variant,intron_variant
rs1796814465	genic_upstream_transcript_variant,intron_variant
rs1796814896	genic_upstream_transcript_variant,intron_variant
rs1796815074	genic_upstream_transcript_variant,intron_variant
rs1796815232	genic_upstream_transcript_variant,intron_variant
rs1796815454	genic_upstream_transcript_variant,intron_variant
rs1796815675	genic_upstream_transcript_variant,intron_variant
rs1796816371	genic_upstream_transcript_variant,intron_variant
rs1796816601	genic_upstream_transcript_variant,intron_variant
rs1796816796	genic_upstream_transcript_variant,intron_variant
rs1796816888	genic_upstream_transcript_variant,intron_variant
rs1796817293	genic_upstream_transcript_variant,intron_variant
rs1796817392	genic_upstream_transcript_variant,intron_variant
rs1796817693	genic_upstream_transcript_variant,intron_variant
rs1796817791	genic_upstream_transcript_variant,intron_variant
rs1796817894	genic_upstream_transcript_variant,intron_variant
rs1796817981	genic_upstream_transcript_variant,intron_variant
rs1796818165	genic_upstream_transcript_variant,intron_variant
rs1796818770	genic_upstream_transcript_variant,intron_variant
rs1796818923	genic_upstream_transcript_variant,intron_variant
rs1796819065	genic_upstream_transcript_variant,intron_variant
rs1796819844	genic_upstream_transcript_variant,intron_variant
rs1796819996	genic_upstream_transcript_variant,intron_variant
rs1796820478	genic_upstream_transcript_variant,intron_variant
rs1796820633	genic_upstream_transcript_variant,intron_variant
rs1796820930	genic_upstream_transcript_variant,intron_variant
rs1796821058	genic_upstream_transcript_variant,intron_variant
rs1796821552	genic_upstream_transcript_variant,intron_variant
rs1796821698	genic_upstream_transcript_variant,intron_variant
rs1796821849	genic_upstream_transcript_variant,intron_variant
rs1796821982	genic_upstream_transcript_variant,intron_variant
rs1796822290	genic_upstream_transcript_variant,intron_variant
rs1796822598	genic_upstream_transcript_variant,intron_variant
rs1796822698	genic_upstream_transcript_variant,intron_variant
rs1796823242	genic_upstream_transcript_variant,intron_variant
rs1796823536	genic_upstream_transcript_variant,intron_variant
rs1796823823	genic_upstream_transcript_variant,intron_variant
rs1796823921	genic_upstream_transcript_variant,intron_variant

rs1796824117	genic_upstream_transcript_variant,intron_variant
rs1796824211	genic_upstream_transcript_variant,intron_variant
rs1796824310	genic_upstream_transcript_variant,intron_variant
rs1796824639	genic_upstream_transcript_variant,intron_variant
rs1796824736	genic_upstream_transcript_variant,intron_variant
rs1796825052	genic_upstream_transcript_variant,intron_variant
rs1796825253	genic_upstream_transcript_variant,intron_variant
rs1796825783	genic_upstream_transcript_variant,intron_variant
rs1796825938	genic_upstream_transcript_variant,intron_variant
rs1796826065	genic_upstream_transcript_variant,intron_variant
rs1796826203	genic_upstream_transcript_variant,intron_variant
rs1796826344	genic_upstream_transcript_variant,intron_variant
rs1796826999	genic_upstream_transcript_variant,intron_variant
rs1796827299	genic_upstream_transcript_variant,intron_variant
rs1796827432	genic_upstream_transcript_variant,intron_variant
rs1796828998	genic_upstream_transcript_variant,intron_variant
rs1796829127	genic_upstream_transcript_variant,intron_variant
rs1796829789	genic_upstream_transcript_variant,intron_variant
rs1796830066	genic_upstream_transcript_variant,intron_variant
rs1796830409	genic_upstream_transcript_variant,intron_variant
rs1796830547	genic_upstream_transcript_variant,intron_variant
rs1796830824	genic_upstream_transcript_variant,intron_variant
rs1796830978	genic_upstream_transcript_variant,intron_variant
rs1796831951	genic_upstream_transcript_variant,intron_variant
rs1796832135	genic_upstream_transcript_variant,intron_variant
rs1796832770	genic_upstream_transcript_variant,intron_variant
rs1796832886	genic_upstream_transcript_variant,intron_variant
rs1796833299	genic_upstream_transcript_variant,intron_variant
rs1796833463	genic_upstream_transcript_variant,intron_variant
rs1796834344	genic_upstream_transcript_variant,intron_variant
rs1796834774	genic_upstream_transcript_variant,intron_variant
rs1796834916	genic_upstream_transcript_variant,intron_variant
rs1796835215	genic_upstream_transcript_variant,intron_variant
rs1796835602	genic_upstream_transcript_variant,intron_variant
rs1796836352	genic_upstream_transcript_variant,intron_variant
rs1796836437	genic_upstream_transcript_variant,intron_variant
rs1796836631	genic_upstream_transcript_variant,intron_variant
rs1796836719	genic_upstream_transcript_variant,intron_variant
rs1796836801	genic_upstream_transcript_variant,intron_variant
rs1796836876	genic_upstream_transcript_variant,intron_variant
rs1796837166	genic_upstream_transcript_variant,intron_variant
rs1796837467	genic_upstream_transcript_variant,intron_variant
rs1796837558	genic_upstream_transcript_variant,intron_variant
rs1796837881	genic_upstream_transcript_variant,intron_variant
rs1796837971	genic_upstream_transcript_variant,intron_variant
rs1796838277	genic_upstream_transcript_variant,intron_variant
rs1796838485	genic_upstream_transcript_variant,intron_variant
rs1796838584	genic_upstream_transcript_variant,intron_variant
rs1796838790	genic_upstream_transcript_variant,intron_variant
rs1796839751	genic_upstream_transcript_variant,intron_variant
rs1796840120	genic_upstream_transcript_variant,intron_variant
rs1796840316	genic_upstream_transcript_variant,intron_variant
rs1796840601	genic_upstream_transcript_variant,intron_variant
rs1796840706	genic_upstream_transcript_variant,intron_variant
rs1796841332	genic_upstream_transcript_variant,intron_variant
rs1796841535	genic_upstream_transcript_variant,intron_variant
rs1796841957	genic_upstream_transcript_variant,intron_variant
rs1796842417	genic_upstream_transcript_variant,intron_variant
rs1796843277	genic_upstream_transcript_variant,intron_variant
rs1796843472	genic_upstream_transcript_variant,intron_variant
rs1796843667	genic_upstream_transcript_variant,intron_variant
rs1796844638	genic_upstream_transcript_variant,intron_variant
rs1796844794	genic_upstream_transcript_variant,intron_variant
rs1796845406	genic_upstream_transcript_variant,intron_variant
rs1796845559	genic_upstream_transcript_variant,intron_variant
rs1796845701	genic_upstream_transcript_variant,intron_variant
rs1796845850	genic_upstream_transcript_variant,intron_variant
rs1796846696	genic_upstream_transcript_variant,intron_variant
rs1796846856	genic_upstream_transcript_variant,intron_variant
rs1796847734	genic_upstream_transcript_variant,intron_variant
rs1796848034	genic_upstream_transcript_variant,intron_variant
rs1796848191	genic_upstream_transcript_variant,intron_variant
rs1796848474	genic_upstream_transcript_variant,intron_variant
rs1796848771	genic_upstream_transcript_variant,intron_variant

rs1796849575	genic_upstream_transcript_variant,intron_variant
rs1796849692	genic_upstream_transcript_variant,intron_variant
rs1796849826	genic_upstream_transcript_variant,intron_variant
rs1796851404	genic_upstream_transcript_variant,intron_variant
rs1796852312	genic_upstream_transcript_variant,intron_variant
rs1796852456	genic_upstream_transcript_variant,intron_variant
rs1796852607	genic_upstream_transcript_variant,intron_variant
rs1796853283	genic_upstream_transcript_variant,intron_variant
rs1796854122	genic_upstream_transcript_variant,intron_variant
rs1796854407	genic_upstream_transcript_variant,intron_variant
rs1796855145	genic_upstream_transcript_variant,intron_variant
rs1796855519	genic_upstream_transcript_variant,intron_variant
rs1796855723	genic_upstream_transcript_variant,intron_variant
rs1796855820	genic_upstream_transcript_variant,intron_variant
rs1796856169	genic_upstream_transcript_variant,intron_variant
rs1796856245	genic_upstream_transcript_variant,intron_variant
rs1796856516	genic_upstream_transcript_variant,intron_variant
rs1796856610	genic_upstream_transcript_variant,intron_variant
rs1796856695	genic_upstream_transcript_variant,intron_variant
rs1796856949	genic_upstream_transcript_variant,intron_variant
rs1796857044	genic_upstream_transcript_variant,intron_variant
rs1796857133	genic_upstream_transcript_variant,intron_variant
rs1796857306	genic_upstream_transcript_variant,intron_variant
rs1796857700	genic_upstream_transcript_variant,intron_variant
rs1796858356	genic_upstream_transcript_variant,intron_variant
rs1796858478	genic_upstream_transcript_variant,intron_variant
rs1796859551	genic_upstream_transcript_variant,intron_variant
rs1796859850	genic_upstream_transcript_variant,intron_variant
rs1796860484	genic_upstream_transcript_variant,intron_variant
rs1796860570	genic_upstream_transcript_variant,intron_variant
rs1796860650	genic_upstream_transcript_variant,intron_variant
rs1796860733	genic_upstream_transcript_variant,intron_variant
rs1796861343	genic_upstream_transcript_variant,intron_variant
rs1796861427	genic_upstream_transcript_variant,intron_variant
rs1796861638	genic_upstream_transcript_variant,intron_variant
rs1796862042	genic_upstream_transcript_variant,intron_variant
rs1796862375	genic_upstream_transcript_variant,intron_variant
rs1796862483	genic_upstream_transcript_variant,intron_variant
rs1796862682	genic_upstream_transcript_variant,intron_variant
rs1796863056	genic_upstream_transcript_variant,intron_variant
rs1796863217	genic_upstream_transcript_variant,intron_variant
rs1796863313	genic_upstream_transcript_variant,intron_variant
rs1796863824	genic_upstream_transcript_variant,intron_variant
rs1796863925	genic_upstream_transcript_variant,intron_variant
rs1796864019	genic_upstream_transcript_variant,intron_variant
rs1796864338	genic_upstream_transcript_variant,intron_variant
rs1796864675	genic_upstream_transcript_variant,intron_variant
rs1796864980	genic_upstream_transcript_variant,intron_variant
rs1796865133	genic_upstream_transcript_variant,intron_variant
rs1796866031	genic_upstream_transcript_variant,intron_variant
rs1796866328	genic_upstream_transcript_variant,intron_variant
rs1796866482	genic_upstream_transcript_variant,intron_variant
rs1796866646	genic_upstream_transcript_variant,intron_variant
rs1796866950	genic_upstream_transcript_variant,intron_variant
rs1796867385	genic_upstream_transcript_variant,intron_variant
rs1796867820	genic_upstream_transcript_variant,intron_variant
rs1796868260	genic_upstream_transcript_variant,intron_variant
rs1796868549	genic_upstream_transcript_variant,intron_variant
rs1796868686	genic_upstream_transcript_variant,intron_variant
rs1796868817	genic_upstream_transcript_variant,intron_variant
rs1796869138	genic_upstream_transcript_variant,intron_variant
rs1796869280	genic_upstream_transcript_variant,intron_variant
rs1796869603	genic_upstream_transcript_variant,intron_variant
rs1796869881	genic_upstream_transcript_variant,intron_variant
rs1796870244	genic_upstream_transcript_variant,intron_variant
rs1796870322	genic_upstream_transcript_variant,intron_variant
rs1796871160	genic_upstream_transcript_variant,intron_variant
rs1796872059	genic_upstream_transcript_variant,intron_variant
rs1796873415	genic_upstream_transcript_variant,intron_variant
rs1796873571	genic_upstream_transcript_variant,intron_variant
rs1796873848	genic_upstream_transcript_variant,intron_variant
rs1796874303	genic_upstream_transcript_variant,intron_variant
rs1796874431	genic_upstream_transcript_variant,intron_variant
rs1796874567	genic_upstream_transcript_variant,intron_variant

rs1796875012	genic_upstream_transcript_variant,intron_variant
rs1796875146	genic_upstream_transcript_variant,intron_variant
rs1796875265	genic_upstream_transcript_variant,intron_variant
rs1796875405	genic_upstream_transcript_variant,intron_variant
rs1796875852	genic_upstream_transcript_variant,intron_variant
rs1796876428	genic_upstream_transcript_variant,intron_variant
rs1796876797	genic_upstream_transcript_variant,intron_variant
rs1796876955	genic_upstream_transcript_variant,intron_variant
rs1796877094	genic_upstream_transcript_variant,intron_variant
rs1796877467	genic_upstream_transcript_variant,intron_variant
rs1796877641	genic_upstream_transcript_variant,intron_variant
rs1796877724	genic_upstream_transcript_variant,intron_variant
rs1796877809	genic_upstream_transcript_variant,intron_variant
rs1796878276	genic_upstream_transcript_variant,intron_variant
rs1796878574	genic_upstream_transcript_variant,intron_variant
rs1796878725	genic_upstream_transcript_variant,intron_variant
rs1796879512	genic_upstream_transcript_variant,intron_variant
rs1796879610	genic_upstream_transcript_variant,intron_variant
rs1796879887	genic_upstream_transcript_variant,intron_variant
rs1796880294	genic_upstream_transcript_variant,intron_variant
rs1796880810	genic_upstream_transcript_variant,intron_variant
rs1796880923	genic_upstream_transcript_variant,intron_variant
rs1796881009	genic_upstream_transcript_variant,intron_variant
rs1796881108	genic_upstream_transcript_variant,intron_variant
rs1796881384	genic_upstream_transcript_variant,intron_variant
rs1796881547	genic_upstream_transcript_variant,intron_variant
rs1796881673	genic_upstream_transcript_variant,intron_variant
rs1796881763	genic_upstream_transcript_variant,intron_variant
rs1796882001	genic_upstream_transcript_variant,intron_variant
rs1796882856	genic_upstream_transcript_variant,intron_variant
rs1796882943	genic_upstream_transcript_variant,intron_variant
rs1796883048	genic_upstream_transcript_variant,intron_variant
rs1796883396	genic_upstream_transcript_variant,intron_variant
rs1796884292	genic_upstream_transcript_variant,intron_variant
rs1796884716	genic_upstream_transcript_variant,intron_variant
rs1796884843	genic_upstream_transcript_variant,intron_variant
rs1796885007	genic_upstream_transcript_variant,intron_variant
rs1796885323	genic_upstream_transcript_variant,intron_variant
rs1796885586	genic_upstream_transcript_variant,intron_variant
rs1796886108	genic_upstream_transcript_variant,intron_variant
rs1796886228	genic_upstream_transcript_variant,intron_variant
rs1796886578	genic_upstream_transcript_variant,intron_variant
rs1796887021	genic_upstream_transcript_variant,intron_variant
rs1796887300	genic_upstream_transcript_variant,intron_variant
rs1796887733	genic_upstream_transcript_variant,intron_variant
rs1796887877	genic_upstream_transcript_variant,intron_variant
rs1796888228	genic_upstream_transcript_variant,intron_variant
rs1796889175	genic_upstream_transcript_variant,intron_variant
rs1796889315	genic_upstream_transcript_variant,intron_variant
rs1796889474	genic_upstream_transcript_variant,intron_variant
rs1796889604	genic_upstream_transcript_variant,intron_variant
rs1796890001	genic_upstream_transcript_variant,intron_variant
rs1796891781	genic_upstream_transcript_variant,intron_variant
rs1796892316	genic_upstream_transcript_variant,intron_variant
rs1796892788	genic_upstream_transcript_variant,intron_variant
rs1796893065	genic_upstream_transcript_variant,intron_variant
rs1796893982	genic_upstream_transcript_variant,intron_variant
rs1796894115	genic_upstream_transcript_variant,intron_variant
rs1796894611	genic_upstream_transcript_variant,intron_variant
rs1796894756	genic_upstream_transcript_variant,intron_variant
rs1796894889	genic_upstream_transcript_variant,intron_variant
rs1796895033	genic_upstream_transcript_variant,intron_variant
rs1796895327	genic_upstream_transcript_variant,intron_variant
rs1796895418	genic_upstream_transcript_variant,intron_variant
rs1796895700	genic_upstream_transcript_variant,intron_variant
rs1796896275	genic_upstream_transcript_variant,intron_variant
rs1796896606	genic_upstream_transcript_variant,intron_variant
rs1796896691	genic_upstream_transcript_variant,intron_variant
rs1796896871	genic_upstream_transcript_variant,intron_variant
rs1796897003	genic_upstream_transcript_variant,intron_variant
rs1796897103	genic_upstream_transcript_variant,intron_variant
rs1796898176	genic_upstream_transcript_variant,intron_variant
rs1796898334	genic_upstream_transcript_variant,intron_variant
rs1796898462	genic_upstream_transcript_variant,intron_variant

rs1796898917	genic_upstream_transcript_variant,intron_variant
rs1796900076	genic_upstream_transcript_variant,intron_variant
rs1796900250	genic_upstream_transcript_variant,intron_variant
rs1796901288	genic_upstream_transcript_variant,intron_variant
rs1796901555	genic_upstream_transcript_variant,intron_variant
rs1796901649	genic_upstream_transcript_variant,intron_variant
rs1796903386	genic_upstream_transcript_variant,intron_variant
rs1796903462	genic_upstream_transcript_variant,intron_variant
rs1796903759	genic_upstream_transcript_variant,intron_variant
rs1796903855	genic_upstream_transcript_variant,intron_variant
rs1796904177	genic_upstream_transcript_variant,intron_variant
rs1796904268	genic_upstream_transcript_variant,intron_variant
rs1796904340	genic_upstream_transcript_variant,intron_variant
rs1796904440	genic_upstream_transcript_variant,intron_variant
rs1796905755	genic_upstream_transcript_variant,intron_variant
rs1796906073	genic_upstream_transcript_variant,intron_variant
rs1796906213	genic_upstream_transcript_variant,intron_variant
rs1796906384	genic_upstream_transcript_variant,intron_variant
rs1796906539	genic_upstream_transcript_variant,intron_variant
rs1796907572	genic_upstream_transcript_variant,intron_variant
rs1796908003	genic_upstream_transcript_variant,intron_variant
rs1796908315	genic_upstream_transcript_variant,intron_variant
rs1796908692	genic_upstream_transcript_variant,intron_variant
rs1796908831	genic_upstream_transcript_variant,intron_variant
rs1796908954	genic_upstream_transcript_variant,intron_variant
rs1796909078	genic_upstream_transcript_variant,intron_variant
rs1796909198	genic_upstream_transcript_variant,intron_variant
rs1796909438	genic_upstream_transcript_variant,intron_variant
rs1796910003	genic_upstream_transcript_variant,intron_variant
rs1796910922	genic_upstream_transcript_variant,intron_variant
rs1796911351	genic_upstream_transcript_variant,intron_variant
rs1796912022	genic_upstream_transcript_variant,intron_variant
rs1796912712	genic_upstream_transcript_variant,intron_variant
rs1796914756	genic_upstream_transcript_variant,intron_variant
rs1796914937	genic_upstream_transcript_variant,intron_variant
rs1796915251	genic_upstream_transcript_variant,intron_variant
rs1796916621	genic_upstream_transcript_variant,intron_variant
rs1796917150	genic_upstream_transcript_variant,intron_variant
rs1796917233	genic_upstream_transcript_variant,intron_variant
rs1796917403	genic_upstream_transcript_variant,intron_variant
rs1796917496	genic_upstream_transcript_variant,intron_variant
rs1796917755	genic_upstream_transcript_variant,intron_variant
rs1796918005	genic_upstream_transcript_variant,intron_variant
rs1796918508	genic_upstream_transcript_variant,intron_variant
rs1796918596	genic_upstream_transcript_variant,intron_variant
rs1796918702	genic_upstream_transcript_variant,intron_variant
rs1796918877	genic_upstream_transcript_variant,intron_variant
rs1796919225	genic_upstream_transcript_variant,intron_variant
rs1796919489	genic_upstream_transcript_variant,intron_variant
rs1796919922	genic_upstream_transcript_variant,intron_variant
rs1796920003	genic_upstream_transcript_variant,intron_variant
rs1796920334	genic_upstream_transcript_variant,intron_variant
rs1796920424	genic_upstream_transcript_variant,intron_variant
rs1796920649	genic_upstream_transcript_variant,intron_variant
rs1796920740	genic_upstream_transcript_variant,intron_variant
rs1796920819	genic_upstream_transcript_variant,intron_variant
rs1796921253	genic_upstream_transcript_variant,intron_variant
rs1796921503	genic_upstream_transcript_variant,intron_variant
rs1796921859	genic_upstream_transcript_variant,intron_variant
rs1796921937	genic_upstream_transcript_variant,intron_variant
rs1796922113	genic_upstream_transcript_variant,intron_variant
rs1796922291	genic_upstream_transcript_variant,intron_variant
rs1796922651	genic_upstream_transcript_variant,intron_variant
rs1796923062	genic_upstream_transcript_variant,intron_variant
rs1796924183	genic_upstream_transcript_variant,intron_variant
rs1796924388	genic_upstream_transcript_variant,intron_variant
rs1796924506	genic_upstream_transcript_variant,intron_variant
rs1796924776	genic_upstream_transcript_variant,intron_variant
rs1796925170	genic_upstream_transcript_variant,intron_variant
rs1796925295	genic_upstream_transcript_variant,intron_variant
rs1796925718	genic_upstream_transcript_variant,intron_variant
rs1796925838	genic_upstream_transcript_variant,intron_variant
rs1796925972	genic_upstream_transcript_variant,intron_variant
rs1796926251	genic_upstream_transcript_variant,intron_variant

rs1796926671	genic_upstream_transcript_variant,intron_variant
rs1796926969	genic_upstream_transcript_variant,intron_variant
rs1796927180	genic_upstream_transcript_variant,intron_variant
rs1796927263	genic_upstream_transcript_variant,intron_variant
rs1796927349	genic_upstream_transcript_variant,intron_variant
rs1796927426	genic_upstream_transcript_variant,intron_variant
rs1796927894	genic_upstream_transcript_variant,intron_variant
rs1796928070	genic_upstream_transcript_variant,intron_variant
rs1796928487	genic_upstream_transcript_variant,intron_variant
rs1796929213	genic_upstream_transcript_variant,intron_variant
rs1796929296	genic_upstream_transcript_variant,intron_variant
rs1796930122	genic_upstream_transcript_variant,intron_variant
rs1796930216	genic_upstream_transcript_variant,intron_variant
rs1796930307	genic_upstream_transcript_variant,intron_variant
rs1796930525	genic_upstream_transcript_variant,intron_variant
rs1796930619	genic_upstream_transcript_variant,intron_variant
rs1796930798	genic_upstream_transcript_variant,intron_variant
rs1796930982	genic_upstream_transcript_variant,intron_variant
rs1796931091	genic_upstream_transcript_variant,intron_variant
rs1796931624	genic_upstream_transcript_variant,intron_variant
rs1796931841	genic_upstream_transcript_variant,intron_variant
rs1796931985	genic_upstream_transcript_variant,intron_variant
rs1796932112	genic_upstream_transcript_variant,intron_variant
rs1796932395	genic_upstream_transcript_variant,intron_variant
rs1796932538	genic_upstream_transcript_variant,intron_variant
rs1796932784	genic_upstream_transcript_variant,intron_variant
rs1796932915	genic_upstream_transcript_variant,intron_variant
rs1796933186	genic_upstream_transcript_variant,intron_variant
rs1796933569	genic_upstream_transcript_variant,intron_variant
rs1796933647	genic_upstream_transcript_variant,intron_variant
rs1796933911	genic_upstream_transcript_variant,intron_variant
rs1796934010	genic_upstream_transcript_variant,intron_variant
rs1796935167	genic_upstream_transcript_variant,intron_variant
rs1796935250	genic_upstream_transcript_variant,intron_variant
rs1796935337	genic_upstream_transcript_variant,intron_variant
rs1796935435	genic_upstream_transcript_variant,intron_variant
rs1796937329	genic_upstream_transcript_variant,intron_variant
rs1796937593	genic_upstream_transcript_variant,intron_variant
rs1796940110	5_prime_UTR_variant,genic_upstream_transcript_variant,intron_variant,non_coding_transcript_variant
rs1796940426	initiator_codon_variant,genic_upstream_transcript_variant,intron_variant,non_coding_transcript_variant,missense_variant
rs1796940635	coding_sequence_variant,genic_upstream_transcript_variant,intron_variant,non_coding_transcript_variant,missense_variant
rs1796940721	coding_sequence_variant,genic_upstream_transcript_variant,intron_variant,non_coding_transcript_variant,missense_variant
rs1796941936	genic_upstream_transcript_variant,intron_variant
rs1796942783	genic_upstream_transcript_variant,intron_variant
rs1796943067	genic_upstream_transcript_variant,intron_variant
rs1796943474	genic_upstream_transcript_variant,intron_variant
rs1796943600	genic_upstream_transcript_variant,intron_variant
rs1796944322	genic_upstream_transcript_variant,intron_variant
rs1796944865	genic_upstream_transcript_variant,intron_variant
rs1796945298	genic_upstream_transcript_variant,intron_variant
rs1796946619	genic_upstream_transcript_variant,intron_variant
rs1796947181	genic_upstream_transcript_variant,intron_variant
rs1796947502	genic_upstream_transcript_variant,intron_variant
rs1796947586	genic_upstream_transcript_variant,intron_variant
rs1796948119	genic_upstream_transcript_variant,intron_variant
rs1796948256	genic_upstream_transcript_variant,intron_variant
rs1796948367	genic_upstream_transcript_variant,intron_variant
rs1796948650	genic_upstream_transcript_variant,intron_variant
rs1796949286	genic_upstream_transcript_variant,intron_variant
rs1796950010	genic_upstream_transcript_variant,intron_variant
rs1796950151	genic_upstream_transcript_variant,intron_variant
rs1796950290	genic_upstream_transcript_variant,intron_variant
rs1796950424	genic_upstream_transcript_variant,intron_variant
rs1796950546	genic_upstream_transcript_variant,intron_variant
rs1796952051	genic_upstream_transcript_variant,intron_variant
rs1796952354	genic_upstream_transcript_variant,intron_variant
rs1796952504	genic_upstream_transcript_variant,intron_variant
rs1796953205	genic_upstream_transcript_variant,intron_variant
rs1796953416	genic_upstream_transcript_variant,intron_variant
rs1796953528	genic_upstream_transcript_variant,intron_variant
rs1796953796	intron_variant,genic_upstream_transcript_variant
rs1796953918	intron_variant,genic_upstream_transcript_variant
rs1796954069	intron_variant,genic_upstream_transcript_variant
rs1796954738	intron_variant,genic_upstream_transcript_variant

rs1796954872	intron_variant,genic_upstream_transcript_variant
rs1796955221	intron_variant,genic_upstream_transcript_variant
rs1796955841	intron_variant,genic_upstream_transcript_variant
rs1796956132	intron_variant,genic_upstream_transcript_variant
rs1796956255	intron_variant,genic_upstream_transcript_variant
rs1796956471	intron_variant,genic_upstream_transcript_variant
rs1796956992	intron_variant,genic_upstream_transcript_variant
rs1796957103	intron_variant,genic_upstream_transcript_variant
rs1796957382	intron_variant,genic_upstream_transcript_variant
rs1796958131	intron_variant,genic_upstream_transcript_variant
rs1796958223	intron_variant,genic_upstream_transcript_variant
rs1796958591	intron_variant,genic_upstream_transcript_variant
rs1796958684	intron_variant,genic_upstream_transcript_variant
rs1796958774	intron_variant,genic_upstream_transcript_variant
rs1796959069	intron_variant,genic_upstream_transcript_variant
rs1796959150	intron_variant,genic_upstream_transcript_variant
rs1796959826	intron_variant,genic_upstream_transcript_variant
rs1796959911	intron_variant,genic_upstream_transcript_variant
rs1796960134	intron_variant,genic_upstream_transcript_variant
rs1796960238	intron_variant,genic_upstream_transcript_variant
rs1796960609	intron_variant,genic_upstream_transcript_variant
rs1796960714	intron_variant,genic_upstream_transcript_variant
rs1796961641	intron_variant,genic_upstream_transcript_variant
rs1796961729	intron_variant,genic_upstream_transcript_variant
rs1796961916	intron_variant,genic_upstream_transcript_variant
rs1796962681	intron_variant,genic_upstream_transcript_variant
rs1796962783	intron_variant,genic_upstream_transcript_variant
rs1796962892	intron_variant,genic_upstream_transcript_variant
rs1796962972	intron_variant,genic_upstream_transcript_variant
rs1796963858	intron_variant,genic_upstream_transcript_variant
rs1796963942	intron_variant,genic_upstream_transcript_variant
rs1796964476	intron_variant,genic_upstream_transcript_variant
rs1796964669	intron_variant,genic_upstream_transcript_variant
rs1796964965	intron_variant,genic_upstream_transcript_variant
rs1796965143	intron_variant,genic_upstream_transcript_variant
rs1796965405	intron_variant,genic_upstream_transcript_variant
rs1796965783	intron_variant,genic_upstream_transcript_variant
rs1796966533	intron_variant,genic_upstream_transcript_variant
rs1796966623	intron_variant,genic_upstream_transcript_variant
rs1796966739	intron_variant,genic_upstream_transcript_variant
rs1796966837	intron_variant,genic_upstream_transcript_variant
rs1796967008	intron_variant,genic_upstream_transcript_variant
rs1796967890	intron_variant,genic_upstream_transcript_variant
rs1796967971	intron_variant,genic_upstream_transcript_variant
rs1796968194	intron_variant,genic_upstream_transcript_variant
rs1796968290	intron_variant,genic_upstream_transcript_variant
rs1796968485	intron_variant,genic_upstream_transcript_variant
rs1796968896	intron_variant,genic_upstream_transcript_variant
rs1796969416	intron_variant,genic_upstream_transcript_variant
rs1796969524	intron_variant,genic_upstream_transcript_variant
rs1796969606	intron_variant,genic_upstream_transcript_variant
rs1796969702	intron_variant,genic_upstream_transcript_variant
rs1796969998	intron_variant,genic_upstream_transcript_variant
rs1796970163	intron_variant,genic_upstream_transcript_variant
rs1796970470	intron_variant,genic_upstream_transcript_variant
rs1796970549	intron_variant,genic_upstream_transcript_variant
rs1796970662	intron_variant,genic_upstream_transcript_variant
rs1796970964	intron_variant,genic_upstream_transcript_variant
rs1796971237	intron_variant,genic_upstream_transcript_variant
rs1796971339	intron_variant,genic_upstream_transcript_variant
rs1796971733	intron_variant,genic_upstream_transcript_variant
rs1796971814	intron_variant,genic_upstream_transcript_variant
rs1796972610	intron_variant,genic_upstream_transcript_variant
rs1796973046	intron_variant,genic_upstream_transcript_variant
rs1796973512	intron_variant,genic_upstream_transcript_variant
rs1796974065	intron_variant,genic_upstream_transcript_variant
rs1796974408	intron_variant,genic_upstream_transcript_variant
rs1796974497	intron_variant,genic_upstream_transcript_variant
rs1796974745	intron_variant,genic_upstream_transcript_variant
rs1796975182	intron_variant,genic_upstream_transcript_variant
rs1796975290	intron_variant,genic_upstream_transcript_variant
rs1796975457	intron_variant,genic_upstream_transcript_variant
rs1796976244	intron_variant,genic_upstream_transcript_variant
rs1796976778	intron_variant,genic_upstream_transcript_variant

rs1796976860	intron_variant,genic_upstream_transcript_variant
rs1796977241	intron_variant,genic_upstream_transcript_variant
rs1796977362	intron_variant,genic_upstream_transcript_variant
rs1796977834	intron_variant,genic_upstream_transcript_variant
rs1796977901	intron_variant,genic_upstream_transcript_variant
rs1796977979	intron_variant,genic_upstream_transcript_variant
rs1796979784	intron_variant,genic_upstream_transcript_variant
rs1796980473	intron_variant,genic_upstream_transcript_variant
rs1796980552	intron_variant,genic_upstream_transcript_variant
rs1796980643	intron_variant,genic_upstream_transcript_variant
rs1796980737	intron_variant,genic_upstream_transcript_variant
rs1796981176	intron_variant,genic_upstream_transcript_variant
rs1796981261	intron_variant,genic_upstream_transcript_variant
rs1796981347	intron_variant,genic_upstream_transcript_variant
rs1796981580	intron_variant,genic_upstream_transcript_variant
rs1796981948	intron_variant,genic_upstream_transcript_variant
rs1796982038	intron_variant,genic_upstream_transcript_variant
rs1796982588	intron_variant,genic_upstream_transcript_variant
rs1796983469	intron_variant,genic_upstream_transcript_variant
rs1796983570	intron_variant,genic_upstream_transcript_variant
rs1796983776	intron_variant,genic_upstream_transcript_variant
rs1796983983	intron_variant,genic_upstream_transcript_variant
rs1796984254	intron_variant,genic_upstream_transcript_variant
rs1796984340	intron_variant,genic_upstream_transcript_variant
rs1796984413	intron_variant,genic_upstream_transcript_variant
rs1796984575	intron_variant,genic_upstream_transcript_variant
rs1796985287	intron_variant,genic_upstream_transcript_variant
rs1796985704	intron_variant,genic_upstream_transcript_variant
rs1796985976	intron_variant,genic_upstream_transcript_variant
rs1796986081	intron_variant,genic_upstream_transcript_variant
rs1796986468	intron_variant,genic_upstream_transcript_variant
rs1796986911	intron_variant,genic_upstream_transcript_variant
rs1796987071	intron_variant,genic_upstream_transcript_variant
rs1796987155	intron_variant,genic_upstream_transcript_variant
rs1796987454	intron_variant,genic_upstream_transcript_variant
rs1796987538	intron_variant,genic_upstream_transcript_variant
rs1796987618	intron_variant,genic_upstream_transcript_variant
rs1796987793	intron_variant,genic_upstream_transcript_variant
rs1796988041	intron_variant,genic_upstream_transcript_variant
rs1796988379	intron_variant,genic_upstream_transcript_variant
rs1796988488	intron_variant,genic_upstream_transcript_variant
rs1796988724	intron_variant,genic_upstream_transcript_variant
rs1796988818	intron_variant,genic_upstream_transcript_variant
rs1796989273	intron_variant,genic_upstream_transcript_variant
rs1796989444	intron_variant,genic_upstream_transcript_variant
rs1796989574	intron_variant,genic_upstream_transcript_variant
rs1796990344	intron_variant,genic_upstream_transcript_variant
rs1796990948	intron_variant,genic_upstream_transcript_variant
rs1796991790	intron_variant,genic_upstream_transcript_variant
rs1796991906	intron_variant,genic_upstream_transcript_variant
rs1796992292	intron_variant,genic_upstream_transcript_variant
rs1796992382	intron_variant,genic_upstream_transcript_variant
rs1796992464	intron_variant,genic_upstream_transcript_variant
rs1796992777	intron_variant,genic_upstream_transcript_variant
rs1796992961	intron_variant,genic_upstream_transcript_variant
rs1796993299	intron_variant,genic_upstream_transcript_variant
rs1796993710	intron_variant,genic_upstream_transcript_variant
rs1796993837	intron_variant,genic_upstream_transcript_variant
rs1796994549	intron_variant,genic_upstream_transcript_variant
rs1796995414	intron_variant,genic_upstream_transcript_variant
rs1796995794	intron_variant,genic_upstream_transcript_variant
rs1796995882	intron_variant,genic_upstream_transcript_variant
rs1796996314	intron_variant,genic_upstream_transcript_variant
rs1796996719	intron_variant,genic_upstream_transcript_variant
rs1796996907	intron_variant,genic_upstream_transcript_variant
rs1796997242	intron_variant,genic_upstream_transcript_variant
rs1796997775	intron_variant,genic_upstream_transcript_variant
rs1796997840	intron_variant,genic_upstream_transcript_variant
rs1796998466	intron_variant,genic_upstream_transcript_variant
rs1796998637	intron_variant,genic_upstream_transcript_variant
rs1796998708	intron_variant,genic_upstream_transcript_variant
rs1796998975	intron_variant,genic_upstream_transcript_variant
rs1796999159	intron_variant,genic_upstream_transcript_variant
rs1796999250	intron_variant,genic_upstream_transcript_variant

rs1796999460	intron_variant,genic_upstream_transcript_variant
rs1796999538	intron_variant,genic_upstream_transcript_variant
rs1796999812	intron_variant,genic_upstream_transcript_variant
rs1797000107	intron_variant,genic_upstream_transcript_variant
rs1797000191	intron_variant,genic_upstream_transcript_variant
rs1797000266	intron_variant,genic_upstream_transcript_variant
rs1797000446	intron_variant,genic_upstream_transcript_variant
rs1797000518	intron_variant,genic_upstream_transcript_variant
rs1797001286	intron_variant,genic_upstream_transcript_variant
rs1797001547	intron_variant,genic_upstream_transcript_variant
rs1797001624	intron_variant,genic_upstream_transcript_variant
rs1797001693	intron_variant,genic_upstream_transcript_variant
rs1797001778	intron_variant,genic_upstream_transcript_variant
rs1797002134	intron_variant,genic_upstream_transcript_variant
rs1797002625	intron_variant,genic_upstream_transcript_variant
rs1797002959	intron_variant,genic_upstream_transcript_variant
rs1797003121	intron_variant,genic_upstream_transcript_variant
rs1797003187	intron_variant,genic_upstream_transcript_variant
rs1797003680	intron_variant,genic_upstream_transcript_variant
rs1797003842	intron_variant,genic_upstream_transcript_variant
rs1797003918	intron_variant,genic_upstream_transcript_variant
rs1797004295	intron_variant,genic_upstream_transcript_variant
rs1797004494	intron_variant,genic_upstream_transcript_variant
rs1797004782	intron_variant,genic_upstream_transcript_variant
rs1797005147	intron_variant,genic_upstream_transcript_variant
rs1797005318	intron_variant,genic_upstream_transcript_variant
rs1797005883	intron_variant,genic_upstream_transcript_variant
rs1797006017	intron_variant,genic_upstream_transcript_variant
rs1797006161	intron_variant,genic_upstream_transcript_variant
rs1797006422	intron_variant,genic_upstream_transcript_variant
rs1797006556	intron_variant,genic_upstream_transcript_variant
rs1797006692	intron_variant,genic_upstream_transcript_variant
rs1797007315	intron_variant,genic_upstream_transcript_variant
rs1797007442	intron_variant,genic_upstream_transcript_variant
rs1797007734	intron_variant,genic_upstream_transcript_variant
rs1797007995	intron_variant,genic_upstream_transcript_variant
rs1797008269	intron_variant,genic_upstream_transcript_variant
rs1797008405	intron_variant,genic_upstream_transcript_variant
rs1797009237	intron_variant,genic_upstream_transcript_variant
rs1797009377	intron_variant,genic_upstream_transcript_variant
rs1797009509	intron_variant,genic_upstream_transcript_variant
rs1797010176	intron_variant,genic_upstream_transcript_variant
rs1797010401	intron_variant,genic_upstream_transcript_variant
rs1797010691	intron_variant,genic_upstream_transcript_variant
rs1797011145	intron_variant,genic_upstream_transcript_variant
rs1797012829	intron_variant,genic_upstream_transcript_variant
rs1797013137	intron_variant,genic_upstream_transcript_variant
rs1797013313	intron_variant,genic_upstream_transcript_variant
rs1797013445	intron_variant,genic_upstream_transcript_variant
rs1797013961	intron_variant,genic_upstream_transcript_variant
rs1797016104	intron_variant,genic_upstream_transcript_variant
rs1797016241	intron_variant,genic_upstream_transcript_variant
rs1797016519	intron_variant,genic_upstream_transcript_variant
rs1797016849	intron_variant,genic_upstream_transcript_variant
rs1797017343	intron_variant,genic_upstream_transcript_variant
rs1797018756	intron_variant,genic_upstream_transcript_variant
rs1797019066	intron_variant,genic_upstream_transcript_variant
rs1797019557	intron_variant,genic_upstream_transcript_variant
rs1797020463	intron_variant,genic_upstream_transcript_variant
rs1797020771	intron_variant,genic_upstream_transcript_variant
rs1797022050	intron_variant,genic_upstream_transcript_variant
rs1797022216	intron_variant,genic_upstream_transcript_variant
rs1797022364	intron_variant,genic_upstream_transcript_variant
rs1797022769	intron_variant,genic_upstream_transcript_variant
rs1797022977	intron_variant,genic_upstream_transcript_variant
rs1797023872	intron_variant,genic_upstream_transcript_variant
rs1797024974	intron_variant,genic_upstream_transcript_variant
rs1797025515	intron_variant,genic_upstream_transcript_variant
rs1797026029	intron_variant,genic_upstream_transcript_variant
rs1797026283	intron_variant,genic_upstream_transcript_variant
rs1797026807	intron_variant,genic_upstream_transcript_variant
rs1797027177	intron_variant,genic_upstream_transcript_variant
rs1797027588	intron_variant,genic_upstream_transcript_variant
rs1797027818	intron_variant,genic_upstream_transcript_variant

rs1797028483	intron_variant,genic_upstream_transcript_variant
rs1797029169	intron_variant,genic_upstream_transcript_variant
rs1797029694	intron_variant,genic_upstream_transcript_variant
rs1797030407	intron_variant,genic_upstream_transcript_variant
rs1797030529	intron_variant,genic_upstream_transcript_variant
rs1797030971	intron_variant,genic_upstream_transcript_variant
rs1797031702	intron_variant,genic_upstream_transcript_variant
rs1797031955	intron_variant,genic_upstream_transcript_variant
rs1797032078	intron_variant,genic_upstream_transcript_variant
rs1797032246	intron_variant,genic_upstream_transcript_variant
rs1797032393	intron_variant,genic_upstream_transcript_variant
rs1797032533	intron_variant,genic_upstream_transcript_variant
rs1797032862	intron_variant,genic_upstream_transcript_variant
rs1797033007	intron_variant,genic_upstream_transcript_variant
rs1797033257	intron_variant,genic_upstream_transcript_variant
rs1797033421	intron_variant,genic_upstream_transcript_variant
rs1797033995	intron_variant,genic_upstream_transcript_variant
rs1797034478	intron_variant,genic_upstream_transcript_variant
rs1797034567	intron_variant,genic_upstream_transcript_variant
rs1797034743	intron_variant,genic_upstream_transcript_variant
rs1797034820	intron_variant,genic_upstream_transcript_variant
rs1797034894	intron_variant,genic_upstream_transcript_variant
rs1797034984	intron_variant,genic_upstream_transcript_variant
rs1797035073	intron_variant,genic_upstream_transcript_variant
rs1797035147	intron_variant,genic_upstream_transcript_variant
rs1797035408	intron_variant,genic_upstream_transcript_variant
rs1797035493	intron_variant,genic_upstream_transcript_variant
rs1797035568	intron_variant,genic_upstream_transcript_variant
rs1797035931	intron_variant,genic_upstream_transcript_variant
rs1797036108	intron_variant,genic_upstream_transcript_variant
rs1797036643	intron_variant,genic_upstream_transcript_variant
rs1797036737	intron_variant,genic_upstream_transcript_variant
rs1797037578	intron_variant,genic_upstream_transcript_variant
rs1797037855	intron_variant,genic_upstream_transcript_variant
rs1797038004	intron_variant,genic_upstream_transcript_variant
rs1797038330	intron_variant,genic_upstream_transcript_variant
rs1797038732	intron_variant,genic_upstream_transcript_variant
rs1797039441	intron_variant,genic_upstream_transcript_variant
rs1797039523	intron_variant,genic_upstream_transcript_variant
rs1797039601	intron_variant,genic_upstream_transcript_variant
rs1797039684	intron_variant,genic_upstream_transcript_variant
rs1797039761	intron_variant,genic_upstream_transcript_variant
rs1797039843	intron_variant,genic_upstream_transcript_variant
rs1797040227	intron_variant,genic_upstream_transcript_variant
rs1797040612	intron_variant,genic_upstream_transcript_variant
rs1797040709	intron_variant,genic_upstream_transcript_variant
rs1797040787	intron_variant,genic_upstream_transcript_variant
rs1797041411	intron_variant,genic_upstream_transcript_variant
rs1797041595	intron_variant,genic_upstream_transcript_variant
rs1797041741	intron_variant,genic_upstream_transcript_variant
rs1797041824	intron_variant,genic_upstream_transcript_variant
rs1797042182	intron_variant,genic_upstream_transcript_variant
rs1797042247	intron_variant,genic_upstream_transcript_variant
rs1797042324	intron_variant,genic_upstream_transcript_variant
rs1797042502	intron_variant,genic_upstream_transcript_variant
rs1797042780	intron_variant,genic_upstream_transcript_variant
rs1797043154	intron_variant,genic_upstream_transcript_variant
rs1797043245	intron_variant,genic_upstream_transcript_variant
rs1797043392	intron_variant,genic_upstream_transcript_variant
rs1797043563	intron_variant,genic_upstream_transcript_variant
rs1797043755	intron_variant,genic_upstream_transcript_variant
rs1797043853	intron_variant,genic_upstream_transcript_variant
rs1797044049	intron_variant,genic_upstream_transcript_variant
rs1797044123	intron_variant,genic_upstream_transcript_variant
rs1797044305	intron_variant,genic_upstream_transcript_variant
rs1797044553	intron_variant,genic_upstream_transcript_variant
rs1797044719	intron_variant,genic_upstream_transcript_variant
rs1797045669	intron_variant,genic_upstream_transcript_variant
rs1797046285	intron_variant,genic_upstream_transcript_variant
rs1797046414	intron_variant,genic_upstream_transcript_variant
rs1797046560	intron_variant,genic_upstream_transcript_variant
rs1797046970	intron_variant,genic_upstream_transcript_variant
rs1797047114	intron_variant,genic_upstream_transcript_variant
rs1797047377	intron_variant,genic_upstream_transcript_variant

rs1797047490	intron_variant,genic_upstream_transcript_variant
rs1797047625	intron_variant,genic_upstream_transcript_variant
rs1797048080	intron_variant,genic_upstream_transcript_variant
rs1797048210	intron_variant,genic_upstream_transcript_variant
rs1797048326	intron_variant,genic_upstream_transcript_variant
rs1797048582	intron_variant,genic_upstream_transcript_variant
rs1797049175	intron_variant,genic_upstream_transcript_variant
rs1797050215	intron_variant,genic_upstream_transcript_variant
rs1797050312	intron_variant,genic_upstream_transcript_variant
rs1797050738	intron_variant,genic_upstream_transcript_variant
rs1797050833	intron_variant,genic_upstream_transcript_variant
rs1797050922	intron_variant,genic_upstream_transcript_variant
rs1797050988	intron_variant,genic_upstream_transcript_variant
rs1797051074	intron_variant,genic_upstream_transcript_variant
rs1797051149	intron_variant,genic_upstream_transcript_variant
rs1797051431	intron_variant,genic_upstream_transcript_variant
rs1797051817	intron_variant,genic_upstream_transcript_variant
rs1797052069	intron_variant,genic_upstream_transcript_variant
rs1797052151	intron_variant,genic_upstream_transcript_variant
rs1797052345	intron_variant,genic_upstream_transcript_variant
rs1797052584	intron_variant,genic_upstream_transcript_variant
rs1797052668	intron_variant,genic_upstream_transcript_variant
rs1797053198	intron_variant,genic_upstream_transcript_variant
rs1797053375	intron_variant,genic_upstream_transcript_variant
rs1797053552	intron_variant,genic_upstream_transcript_variant
rs1797053862	intron_variant,genic_upstream_transcript_variant
rs1797053954	intron_variant,genic_upstream_transcript_variant
rs1797054602	intron_variant,genic_upstream_transcript_variant
rs1797054690	intron_variant,genic_upstream_transcript_variant
rs1797055465	intron_variant,genic_upstream_transcript_variant
rs1797055724	intron_variant,genic_upstream_transcript_variant
rs1797056270	intron_variant,genic_upstream_transcript_variant
rs1797056345	intron_variant,genic_upstream_transcript_variant
rs1797056714	intron_variant,genic_upstream_transcript_variant
rs1797056811	intron_variant,genic_upstream_transcript_variant
rs1797057139	intron_variant,genic_upstream_transcript_variant
rs1797057208	intron_variant,genic_upstream_transcript_variant
rs1797057611	intron_variant,genic_upstream_transcript_variant
rs1797057885	intron_variant,genic_upstream_transcript_variant
rs1797058603	intron_variant,genic_upstream_transcript_variant
rs1797058676	intron_variant,genic_upstream_transcript_variant
rs1797058748	intron_variant,genic_upstream_transcript_variant
rs1797058820	intron_variant,genic_upstream_transcript_variant
rs1797058916	intron_variant,genic_upstream_transcript_variant
rs1797058997	intron_variant,genic_upstream_transcript_variant
rs1797059251	intron_variant,genic_upstream_transcript_variant
rs1797059419	intron_variant,genic_upstream_transcript_variant
rs1797060208	intron_variant,genic_upstream_transcript_variant
rs1797060297	intron_variant,genic_upstream_transcript_variant
rs1797060384	intron_variant,genic_upstream_transcript_variant
rs1797060657	intron_variant,genic_upstream_transcript_variant
rs1797061005	intron_variant,genic_upstream_transcript_variant
rs1797061092	intron_variant,genic_upstream_transcript_variant
rs1797061176	intron_variant,genic_upstream_transcript_variant
rs1797061251	intron_variant,genic_upstream_transcript_variant
rs1797062137	intron_variant,genic_upstream_transcript_variant
rs1797062283	intron_variant,genic_upstream_transcript_variant
rs1797062364	intron_variant,genic_upstream_transcript_variant
rs1797063885	coding_sequence_variant,missense_variant,5_prime_UTR_variant,genic_upstream_transcript_variant,intron_variant,non_coding_transcript_variant
rs1797064164	coding_sequence_variant,missense_variant,intron_variant,genic_upstream_transcript_variant,non_coding_transcript_variant
rs1797064273	synonymous_variant,coding_sequence_variant,intron_variant,genic_upstream_transcript_variant,non_coding_transcript_variant
rs1797066204	synonymous_variant,coding_sequence_variant,intron_variant,genic_upstream_transcript_variant,non_coding_transcript_variant
rs1797068456	coding_sequence_variant,missense_variant,intron_variant,genic_upstream_transcript_variant,non_coding_transcript_variant
rs1797069603	coding_sequence_variant,missense_variant,intron_variant,genic_upstream_transcript_variant,non_coding_transcript_variant
rs1800613522	downstream_transcript_variant,genic_downstream_transcript_variant,intron_variant
rs1803849437	genic_downstream_transcript_variant,intron_variant
rs1803861549	genic_downstream_transcript_variant,intron_variant
rs1809607683	intron_variant,genic_upstream_transcript_variant
rs1839718006	genic_upstream_transcript_variant,intron_variant,upstream_transcript_variant
rs1934022137	intron_variant
rs1934185185	intron_variant
rs1934525867	intron_variant,genic_upstream_transcript_variant
rs1939305037	intron_variant,genic_downstream_transcript_variant
rs1941940782	intron_variant

rs1944026033	intron_variant
rs2014578826	intron_variant,genic_upstream_transcript_variant
rs2033148705	intron_variant,genic_upstream_transcript_variant,upstream_transcript_variant
rs2043628095	intron_variant,genic_downstream_transcript_variant
rs2049759124	intron_variant,genic_upstream_transcript_variant
rs2052978422	intron_variant,genic_upstream_transcript_variant
rs2054507949	intron_variant,genic_upstream_transcript_variant
rs2059920872	intron_variant,genic_downstream_transcript_variant
rs2064223049	intron_variant
rs2070614684	intron_variant,genic_downstream_transcript_variant
rs2074658451	intron_variant,genic_downstream_transcript_variant
rs2076053855	intron_variant,genic_downstream_transcript_variant
rs2079148505	intron_variant,genic_upstream_transcript_variant,upstream_transcript_variant
rs2083522116	intron_variant
rs2087572095	intron_variant,genic_upstream_transcript_variant
rs2088633710	intron_variant,genic_downstream_transcript_variant
rs2096729957	intron_variant,genic_downstream_transcript_variant
rs16945	downstream_transcript_variant,500B_downstream_variant
rs16945	downstream_transcript_variant,500B_downstream_variant
rs16945	downstream_transcript_variant,500B_downstream_variant
rs16945	downstream_transcript_variant,500B_downstream_variant
rs28841	intron_variant
rs28841	intron_variant
rs38839	2KB_upstream_variant,intron_variant,upstream_transcript_variant,genic_upstream_transcript_variant
rs38839	2KB_upstream_variant,intron_variant,upstream_transcript_variant,genic_upstream_transcript_variant
rs38840	genic_upstream_transcript_variant,intron_variant
rs38840	genic_upstream_transcript_variant,intron_variant
rs38841	genic_upstream_transcript_variant,intron_variant
rs38841	genic_upstream_transcript_variant,intron_variant
rs38842	genic_upstream_transcript_variant,intron_variant
rs38843	genic_upstream_transcript_variant,intron_variant
rs38843	genic_upstream_transcript_variant,intron_variant
rs38844	genic_upstream_transcript_variant,intron_variant
rs38845	genic_upstream_transcript_variant,intron_variant
rs38846	genic_upstream_transcript_variant,intron_variant
rs38846	genic_upstream_transcript_variant,intron_variant
rs38847	genic_upstream_transcript_variant,intron_variant
rs38847	genic_upstream_transcript_variant,intron_variant
rs38849	genic_upstream_transcript_variant,intron_variant
rs38849	genic_upstream_transcript_variant,intron_variant
rs38850	genic_upstream_transcript_variant,intron_variant
rs38852	genic_upstream_transcript_variant,intron_variant
rs38853	genic_upstream_transcript_variant,intron_variant
rs38853	genic_upstream_transcript_variant,intron_variant
rs38854	genic_upstream_transcript_variant,intron_variant
rs38855	genic_upstream_transcript_variant,intron_variant
rs38857	intron_variant
rs38857	intron_variant
rs38857	intron_variant
rs38857	intron_variant
rs38858	intron_variant
rs38859	intron_variant
rs38860	intron_variant
rs38860	intron_variant
rs39747	genic_upstream_transcript_variant,intron_variant
rs40238	genic_upstream_transcript_variant,intron_variant
rs40238	genic_upstream_transcript_variant,intron_variant
rs40238	genic_upstream_transcript_variant,intron_variant
rs40239	genic_upstream_transcript_variant,intron_variant
rs40239	genic_upstream_transcript_variant,intron_variant
rs41735	genic_downstream_transcript_variant,intron_variant
rs41741	downstream_transcript_variant,500B_downstream_variant
rs41741	downstream_transcript_variant,500B_downstream_variant
rs41741	downstream_transcript_variant,500B_downstream_variant
rs183642	genic_downstream_transcript_variant,intron_variant
rs183642	genic_downstream_transcript_variant,intron_variant
rs183642	genic_downstream_transcript_variant,intron_variant
rs184953	2KB_upstream_variant,intron_variant,upstream_transcript_variant,genic_upstream_transcript_variant
rs193686	genic_downstream_transcript_variant,intron_variant
rs193687	genic_downstream_transcript_variant,intron_variant
rs193688	genic_downstream_transcript_variant,intron_variant
rs193688	genic_downstream_transcript_variant,intron_variant
rs714180	genic_upstream_transcript_variant,intron_variant
rs714180	genic_upstream_transcript_variant,intron_variant

rs722134	genic_upstream_transcript_variant,intron_variant
rs2237708	genic_upstream_transcript_variant,intron_variant
rs2237709	genic_upstream_transcript_variant,intron_variant
rs2237710	genic_upstream_transcript_variant,intron_variant
rs2237710	genic_upstream_transcript_variant,intron_variant
rs2237712	genic_upstream_transcript_variant,intron_variant
rs2237712	genic_upstream_transcript_variant,intron_variant
rs2237712	genic_upstream_transcript_variant,intron_variant
rs2237713	genic_upstream_transcript_variant,intron_variant
rs2237714	genic_upstream_transcript_variant,intron_variant
rs2237717	intron_variant
rs2237717	intron_variant
rs2237717	intron_variant
rs2283053	genic_downstream_transcript_variant,intron_variant
rs2299433	genic_upstream_transcript_variant,intron_variant
rs2299433	genic_upstream_transcript_variant,intron_variant
rs2299434	genic_upstream_transcript_variant,intron_variant
rs2299435	genic_upstream_transcript_variant,intron_variant
rs2299436	genic_upstream_transcript_variant,intron_variant
rs2299436	genic_upstream_transcript_variant,intron_variant
rs2299437	genic_upstream_transcript_variant,intron_variant
rs2299437	genic_upstream_transcript_variant,intron_variant
rs2299437	genic_upstream_transcript_variant,intron_variant
rs2299439	genic_downstream_transcript_variant,intron_variant
rs2299439	genic_downstream_transcript_variant,intron_variant
rs2299440	genic_downstream_transcript_variant,intron_variant
rs2402118	genic_downstream_transcript_variant,intron_variant
rs2402118	genic_downstream_transcript_variant,intron_variant
rs2402118	genic_downstream_transcript_variant,intron_variant
rs2402118	genic_downstream_transcript_variant,intron_variant
rs2402118	genic_downstream_transcript_variant,intron_variant
rs2896191	genic_upstream_transcript_variant,intron_variant
rs3031564	2KB_upstream_variant,intron_variant,upstream_transcript_variant,genic_upstream_transcript_variant
rs3031564	2KB_upstream_variant,intron_variant,upstream_transcript_variant,genic_upstream_transcript_variant
rs3031564	2KB_upstream_variant,intron_variant,upstream_transcript_variant,genic_upstream_transcript_variant
rs3031564	2KB_upstream_variant,intron_variant,upstream_transcript_variant,genic_upstream_transcript_variant
rs3031564	2KB_upstream_variant,intron_variant,upstream_transcript_variant,genic_upstream_transcript_variant
rs3031564	2KB_upstream_variant,intron_variant,upstream_transcript_variant,genic_upstream_transcript_variant
rs3031564	2KB_upstream_variant,intron_variant,upstream_transcript_variant,genic_upstream_transcript_variant
rs3031564	2KB_upstream_variant,intron_variant,upstream_transcript_variant,genic_upstream_transcript_variant
rs3031564	2KB_upstream_variant,intron_variant,upstream_transcript_variant,genic_upstream_transcript_variant
rs3807996	intron_variant
rs3840635	genic_upstream_transcript_variant,intron_variant
rs3840635	genic_upstream_transcript_variant,intron_variant
rs3840635	genic_upstream_transcript_variant,intron_variant
rs3840635	genic_upstream_transcript_variant,intron_variant
rs3840635	genic_upstream_transcript_variant,intron_variant
rs3840635	genic_upstream_transcript_variant,intron_variant
rs3840635	genic_upstream_transcript_variant,intron_variant
rs3840635	genic_upstream_transcript_variant,intron_variant
rs4727836	intron_variant
rs6466592	intron_variant
rs6466592	intron_variant
rs6466592	intron_variant
rs6945273	intron_variant
rs6946538	intron_variant
rs6947629	genic_downstream_transcript_variant,intron_variant
rs6949201	intron_variant
rs6949201	intron_variant
rs6965570	genic_upstream_transcript_variant,intron_variant
rs6978135	genic_downstream_transcript_variant,intron_variant
rs6978135	genic_downstream_transcript_variant,intron_variant
rs7804632	intron_variant
rs7810164	genic_upstream_transcript_variant,intron_variant
rs9641562	genic_upstream_transcript_variant,intron_variant
rs9641563	genic_upstream_transcript_variant,intron_variant
rs9641564	genic_upstream_transcript_variant,intron_variant
rs10215153	intron_variant
rs10223961	genic_upstream_transcript_variant,intron_variant
rs10232307	intron_variant
rs10235944	intron_variant
rs10243024	genic_upstream_transcript_variant,intron_variant
rs10243024	genic_upstream_transcript_variant,intron_variant
rs10271561	genic_upstream_transcript_variant,intron_variant

rs10279782	intron_variant
rs10487353	genic_upstream_transcript_variant,intron_variant
rs10674901	intron_variant
rs10674901	intron_variant
rs10674901	intron_variant
rs10674901	intron_variant
rs10674901	intron_variant
rs10674901	intron_variant
rs10674901	intron_variant
rs60728121	intron_variant
rs375694180	intron_variant
rs139335533	intron_variant
rs1491263538	genic_upstream_transcript_variant,intron_variant
rs398005977	genic_upstream_transcript_variant,intron_variant
rs397726974	genic_upstream_transcript_variant,intron_variant
rs71148328	genic_upstream_transcript_variant,intron_variant
rs1251370061	genic_downstream_transcript_variant,intron_variant
rs149992616	genic_downstream_transcript_variant,intron_variant
rs145607365	genic_downstream_transcript_variant,intron_variant
rs34757041	genic_downstream_transcript_variant,intron_variant
rs34756179	genic_downstream_transcript_variant,intron_variant
rs56530581	intron_variant,genic_upstream_transcript_variant
rs56494314	intron_variant,genic_upstream_transcript_variant
rs52803357	intron_variant,genic_upstream_transcript_variant
rs61574521	intron_variant,genic_upstream_transcript_variant
rs60635852	intron_variant
rs60230670	intron_variant,genic_downstream_transcript_variant
rs61168850	intron_variant
rs57391921	intron_variant
rs1487033029	intron_variant
rs1283035003	intron_variant
rs397702650	intron_variant
rs67225621	intron_variant
rs796631372	intron_variant
rs67063134	intron_variant
rs36012692	intron_variant
rs1437231732	2KB_upstream_variant,genic_upstream_transcript_variant,upstream_transcript_variant,intron_variant
rs1371410510	2KB_upstream_variant,genic_upstream_transcript_variant,upstream_transcript_variant,intron_variant
rs889249901	2KB_upstream_variant,genic_upstream_transcript_variant,upstream_transcript_variant,intron_variant
rs869176357	2KB_upstream_variant,genic_upstream_transcript_variant,upstream_transcript_variant,intron_variant
rs397897310	2KB_upstream_variant,genic_upstream_transcript_variant,upstream_transcript_variant,intron_variant
rs368522661	2KB_upstream_variant,genic_upstream_transcript_variant,upstream_transcript_variant,intron_variant
rs112424327	2KB_upstream_variant,genic_upstream_transcript_variant,upstream_transcript_variant,intron_variant
rs36222678	2KB_upstream_variant,genic_upstream_transcript_variant,upstream_transcript_variant,intron_variant
rs150357421	intron_variant,genic_upstream_transcript_variant
rs62470764	intron_variant,genic_downstream_transcript_variant
rs1491058896	intron_variant,genic_upstream_transcript_variant
rs1467890336	intron_variant,genic_upstream_transcript_variant
rs1258091127	intron_variant,genic_upstream_transcript_variant
rs1174825885	intron_variant,genic_upstream_transcript_variant
rs60570682	intron_variant,genic_upstream_transcript_variant
rs150308728	intron_variant,genic_upstream_transcript_variant
rs1491052470	intron_variant
rs796499649	intron_variant
rs765700347	intron_variant
rs372923004	intron_variant
rs146796918	intron_variant
rs72318699	intron_variant
rs1491296805	intron_variant
rs1445950074	intron_variant
rs1342380574	intron_variant
rs1306508011	intron_variant
rs1284068744	intron_variant
rs1256638755	intron_variant
rs1220779340	intron_variant
rs56211062	intron_variant
rs553425769	intron_variant
rs1476413470	intron_variant,genic_downstream_transcript_variant
rs1469062989	intron_variant,genic_downstream_transcript_variant
rs1441378861	intron_variant,genic_downstream_transcript_variant
rs1414970085	intron_variant,genic_downstream_transcript_variant
rs1393305754	intron_variant,genic_downstream_transcript_variant
rs1321921983	intron_variant,genic_downstream_transcript_variant
rs1284780498	intron_variant,genic_downstream_transcript_variant

rs1215833845	intron_variant,genic_downstream_transcript_variant
rs1177190697	intron_variant,genic_downstream_transcript_variant
rs1162319701	intron_variant,genic_downstream_transcript_variant
rs773395948	intron_variant,genic_downstream_transcript_variant
rs757359883	intron_variant,genic_downstream_transcript_variant
rs67413931	intron_variant,genic_downstream_transcript_variant
rs62470765	intron_variant,genic_downstream_transcript_variant
rs397974831	intron_variant
rs367847186	intron_variant
rs148286670	intron_variant
rs373660761	intron_variant
rs759615935	intron_variant,genic_upstream_transcript_variant
rs149077312	intron_variant,genic_upstream_transcript_variant
rs67054197	intron_variant,genic_upstream_transcript_variant
rs62469055	intron_variant,genic_upstream_transcript_variant,upstream_transcript_variant,2KB_upstream_variant
rs397820061	intron_variant
rs148173983	intron_variant
rs67670327	intron_variant
rs1474664678	intron_variant,genic_upstream_transcript_variant
rs1430338794	intron_variant,genic_upstream_transcript_variant
rs796979618	intron_variant,genic_upstream_transcript_variant
rs376932004	intron_variant,genic_upstream_transcript_variant
rs71780538	intron_variant,genic_upstream_transcript_variant
rs1158336212	intron_variant
rs146007827	intron_variant
rs1276223352	intron_variant
rs386467303	intron_variant
rs66625749	intron_variant,genic_upstream_transcript_variant
rs386493874	intron_variant,genic_upstream_transcript_variant
rs140282074	intron_variant,genic_upstream_transcript_variant,upstream_transcript_variant,2KB_upstream_variant
rs66712868	intron_variant,genic_upstream_transcript_variant,upstream_transcript_variant,2KB_upstream_variant
rs66712867	intron_variant,genic_upstream_transcript_variant,upstream_transcript_variant,2KB_upstream_variant
rs367840178	intron_variant
rs66849349	intron_variant
rs139235163	intron_variant,genic_downstream_transcript_variant
rs66907203	intron_variant,genic_downstream_transcript_variant
rs66904902	intron_variant,genic_downstream_transcript_variant
rs137935225	intron_variant,genic_upstream_transcript_variant
rs66908767	intron_variant,genic_upstream_transcript_variant
rs66908766	intron_variant,genic_upstream_transcript_variant
rs66908765	intron_variant,genic_upstream_transcript_variant
rs66908764	intron_variant,genic_upstream_transcript_variant
rs66908763	intron_variant,genic_upstream_transcript_variant
rs66908762	intron_variant,genic_upstream_transcript_variant
rs66908761	intron_variant,genic_upstream_transcript_variant
rs1401817436	intron_variant
rs775236929	intron_variant
rs566782532	intron_variant
rs376637658	intron_variant
rs373134711	intron_variant
rs146754820	intron_variant
rs66996051	intron_variant
rs67297108	intron_variant
rs67297107	intron_variant
rs1491494569	intron_variant
rs1392929067	intron_variant
rs1327962790	intron_variant
rs73716768	intron_variant,genic_downstream_transcript_variant
rs368105843	intron_variant
rs116390080	intron_variant
rs112842464	intron_variant
rs118074096	intron_variant,downstream_transcript_variant,genic_downstream_transcript_variant
rs386435000	intron_variant,genic_upstream_transcript_variant
rs372598144	genic_downstream_transcript_variant,intron_variant
rs1160458462	intron_variant,genic_upstream_transcript_variant
rs879871325	intron_variant,genic_upstream_transcript_variant
rs376254940	intron_variant,genic_upstream_transcript_variant
rs1445045860	intron_variant,genic_upstream_transcript_variant
rs1404826611	intron_variant,genic_upstream_transcript_variant
rs1403002235	intron_variant,genic_upstream_transcript_variant
rs1388731990	intron_variant,genic_upstream_transcript_variant
rs1336590190	intron_variant,genic_upstream_transcript_variant
rs1330226872	intron_variant,genic_upstream_transcript_variant
rs1165191012	intron_variant,genic_upstream_transcript_variant

rs369614565	intron_variant,genic_upstream_transcript_variant
rs527395917	intron_variant,genic_upstream_transcript_variant
rs368246294	intron_variant,genic_upstream_transcript_variant
rs778952866	intron_variant
rs560457746	intron_variant
rs377384694	intron_variant
rs765849621	intron_variant
rs1254058248	intron_variant
rs753014425	intron_variant,genic_upstream_transcript_variant
rs747715532	intron_variant,genic_upstream_transcript_variant
rs536870496	intron_variant,genic_upstream_transcript_variant
rs367766330	intron_variant,genic_upstream_transcript_variant
rs1491577926	intron_variant,genic_upstream_transcript_variant
rs1483576249	intron_variant,genic_upstream_transcript_variant
rs982830381	intron_variant
rs1415785967	intron_variant
rs796853155	intron_variant
rs554481521	intron_variant
rs375084330	intron_variant
rs1476851288	upstream_transcript_variant,genic_upstream_transcript_variant,intron_variant,2KB_upstream_variant
rs530064072	intron_variant,genic_upstream_transcript_variant
rs891590876	intron_variant
rs574203259	intron_variant,genic_upstream_transcript_variant
rs564884564	genic_downstream_transcript_variant,intron_variant
rs539133481	intron_variant,genic_upstream_transcript_variant
rs557854595	intron_variant
rs556754721	intron_variant
rs555448301	intron_variant
rs375583910	intron_variant
rs1405595391	intron_variant
rs546780063	genic_downstream_transcript_variant,intron_variant
rs1237807623	intron_variant,genic_upstream_transcript_variant
rs532372468	intron_variant
rs563904002	intron_variant
rs375900077	intron_variant
rs1270420095	intron_variant
rs929506599	intron_variant
rs779562192	intron_variant,genic_upstream_transcript_variant
rs1251699262	genic_downstream_transcript_variant,intron_variant
rs1176384808	genic_downstream_transcript_variant,intron_variant
rs955405496	genic_downstream_transcript_variant,intron_variant
rs1180390677	genic_downstream_transcript_variant,intron_variant
rs762702546	genic_downstream_transcript_variant,intron_variant
rs751660412	genic_downstream_transcript_variant,intron_variant
rs1291154390	genic_downstream_transcript_variant,intron_variant
rs559296135	genic_downstream_transcript_variant,intron_variant
rs1260106143	intron_variant,genic_upstream_transcript_variant
rs1001530884	intron_variant,genic_upstream_transcript_variant
rs1479875163	intron_variant,genic_upstream_transcript_variant
rs1424719466	intron_variant,genic_upstream_transcript_variant
rs1331878635	intron_variant,genic_upstream_transcript_variant
rs1284157953	intron_variant,genic_upstream_transcript_variant
rs1268774060	intron_variant,genic_upstream_transcript_variant
rs1260185170	intron_variant,genic_upstream_transcript_variant
rs1222997092	intron_variant,genic_upstream_transcript_variant
rs1203854088	intron_variant,genic_upstream_transcript_variant
rs779814452	intron_variant,genic_upstream_transcript_variant
rs1236934214	intron_variant,genic_upstream_transcript_variant
rs758446144	intron_variant,genic_upstream_transcript_variant
rs1434417107	upstream_transcript_variant,2KB_upstream_variant,intron_variant,genic_upstream_transcript_variant
rs1393611408	upstream_transcript_variant,2KB_upstream_variant,intron_variant,genic_upstream_transcript_variant
rs1376654538	upstream_transcript_variant,2KB_upstream_variant,intron_variant,genic_upstream_transcript_variant
rs1374776150	upstream_transcript_variant,2KB_upstream_variant,intron_variant,genic_upstream_transcript_variant
rs1367643446	upstream_transcript_variant,2KB_upstream_variant,intron_variant,genic_upstream_transcript_variant
rs1319493506	upstream_transcript_variant,2KB_upstream_variant,intron_variant,genic_upstream_transcript_variant
rs1293497134	upstream_transcript_variant,2KB_upstream_variant,intron_variant,genic_upstream_transcript_variant
rs1292999586	upstream_transcript_variant,2KB_upstream_variant,intron_variant,genic_upstream_transcript_variant
rs1247121839	upstream_transcript_variant,2KB_upstream_variant,intron_variant,genic_upstream_transcript_variant
rs1237445909	upstream_transcript_variant,2KB_upstream_variant,intron_variant,genic_upstream_transcript_variant
rs869038513	upstream_transcript_variant,2KB_upstream_variant,intron_variant,genic_upstream_transcript_variant
rs1419588982	intron_variant
rs969342789	intron_variant,genic_downstream_transcript_variant
rs1483681734	intron_variant
rs1469304512	intron_variant

rs1415523677	intron_variant
rs1250115564	intron_variant
rs1187893867	intron_variant
rs1184757751	intron_variant
rs759688352	intron_variant
rs1028222407	intron_variant,genic_downstream_transcript_variant
rs997205090	intron_variant,genic_downstream_transcript_variant
rs1219982298	genic_upstream_transcript_variant,intron_variant
rs747147150	genic_upstream_transcript_variant,intron_variant
rs1476725551	genic_downstream_transcript_variant,intron_variant
rs1379054262	genic_downstream_transcript_variant,intron_variant
rs1489276384	genic_upstream_transcript_variant,intron_variant
rs1357351645	intron_variant
rs1272360083	intron_variant
rs1362478455	intron_variant
rs776965898	intron_variant
rs1443751716	genic_upstream_transcript_variant,intron_variant
rs1423111949	genic_upstream_transcript_variant,intron_variant
rs1396483354	genic_upstream_transcript_variant,intron_variant
rs1387418033	genic_upstream_transcript_variant,intron_variant
rs1372560806	genic_upstream_transcript_variant,intron_variant
rs1357061293	genic_upstream_transcript_variant,intron_variant
rs1317046138	genic_upstream_transcript_variant,intron_variant
rs1308241827	genic_upstream_transcript_variant,intron_variant
rs1302195996	genic_upstream_transcript_variant,intron_variant
rs1295876989	genic_upstream_transcript_variant,intron_variant
rs1291389343	genic_upstream_transcript_variant,intron_variant
rs1218207781	genic_upstream_transcript_variant,intron_variant
rs1164648918	genic_upstream_transcript_variant,intron_variant
rs869245146	genic_upstream_transcript_variant,intron_variant
rs776764808	genic_upstream_transcript_variant,intron_variant
rs767255754	genic_upstream_transcript_variant,intron_variant
rs1397878507	genic_downstream_transcript_variant,intron_variant
rs1414615536	genic_upstream_transcript_variant,intron_variant
rs1403208144	genic_upstream_transcript_variant,intron_variant
rs779923249	genic_upstream_transcript_variant,intron_variant
rs1477521205	intron_variant
rs1376614750	upstream_transcript_variant,genic_upstream_transcript_variant,intron_variant
rs1350940512	intron_variant
rs1281420884	intron_variant
rs1005267440	intron_variant
rs1263984001	genic_upstream_transcript_variant,intron_variant,upstream_transcript_variant,2KB_upstream_variant
rs1239485971	genic_upstream_transcript_variant,intron_variant,upstream_transcript_variant,2KB_upstream_variant
rs781100494	intron_variant
rs758508055	intron_variant
rs1477104941	intron_variant,genic_upstream_transcript_variant
rs1467887598	intron_variant,genic_upstream_transcript_variant
rs1378624161	intron_variant,genic_upstream_transcript_variant
rs1193060790	intron_variant,genic_upstream_transcript_variant
rs1454142689	intron_variant
rs1380587769	intron_variant
rs1364431125	intron_variant
rs1289595028	intron_variant
rs796323792	intron_variant
rs1297779902	intron_variant
rs1262992194	intron_variant
rs897900498	genic_upstream_transcript_variant,intron_variant
rs879340516	genic_upstream_transcript_variant,intron_variant
rs773943998	genic_upstream_transcript_variant,intron_variant
rs1218946223	intron_variant
rs774060523	intron_variant
rs767196610	intron_variant
rs1486100144	intron_variant
rs869213398	intron_variant
rs1454614693	genic_upstream_transcript_variant,intron_variant
rs1450723281	intron_variant
rs1440305574	intron_variant
rs770913210	intron_variant
rs1300327003	intron_variant
rs1291061466	intron_variant
rs775107525	intron_variant
rs774612760	intron_variant
rs1438226662	upstream_transcript_variant,intron_variant,2KB_upstream_variant,genic_upstream_transcript_variant
rs1364293794	intron_variant,genic_upstream_transcript_variant

rs1305305708	intron_variant,genic_upstream_transcript_variant
rs1234778638	intron_variant,genic_upstream_transcript_variant
rs1035121935	intron_variant
rs1030391504	intron_variant
rs1277751279	intron_variant
rs1428375631	intron_variant
rs1053959932	genic_upstream_transcript_variant,intron_variant
rs1220990250	intron_variant
rs1471917133	intron_variant
rs1298367602	genic_downstream_transcript_variant,intron_variant
rs1162110960	genic_downstream_transcript_variant,intron_variant
rs1454527158	genic_upstream_transcript_variant,intron_variant
rs1431930868	intron_variant
rs1173997370	intron_variant,genic_downstream_transcript_variant
rs1446226785	genic_downstream_transcript_variant,intron_variant
rs1371091186	genic_upstream_transcript_variant,intron_variant
rs1026058071	genic_upstream_transcript_variant,intron_variant
rs1383653178	2KB_upstream_variant,genic_upstream_transcript_variant,upstream_transcript_variant,intron_variant
rs1319250184	intron_variant,genic_upstream_transcript_variant
rs1260104145	intron_variant,genic_upstream_transcript_variant
rs1439323260	intron_variant,genic_upstream_transcript_variant
rs1176732281	intron_variant
rs1222148712	genic_upstream_transcript_variant,intron_variant
rs1347850984	genic_upstream_transcript_variant,intron_variant
rs1290575282	intron_variant
rs1473698764	intron_variant,genic_upstream_transcript_variant
rs1352094674	intron_variant,genic_upstream_transcript_variant
rs1474626406	intron_variant,genic_upstream_transcript_variant
rs1239319469	intron_variant,genic_upstream_transcript_variant
rs1423900181	intron_variant,genic_downstream_transcript_variant
rs1390290820	intron_variant
rs1473009240	genic_upstream_transcript_variant,intron_variant
rs1405835625	genic_upstream_transcript_variant,intron_variant
rs1348283443	intron_variant
rs1343460709	intron_variant
rs1491167146	genic_upstream_transcript_variant,intron_variant
rs1271232546	genic_upstream_transcript_variant,intron_variant
rs1241475594	intron_variant
rs1433784914	genic_upstream_transcript_variant,intron_variant
rs1409683062	intron_variant,genic_upstream_transcript_variant
rs1482862733	intron_variant
rs1429447831	genic_upstream_transcript_variant,intron_variant
rs1313274502	intron_variant,genic_upstream_transcript_variant
rs1282557906	intron_variant
rs1281618684	genic_downstream_transcript_variant,intron_variant
rs1483523706	intron_variant,genic_upstream_transcript_variant
rs1443167352	intron_variant
rs1486388740	intron_variant
rs1445283858	intron_variant,genic_upstream_transcript_variant
rs1411671350	genic_upstream_transcript_variant,intron_variant
rs1456775137	genic_upstream_transcript_variant,intron_variant
rs1408600334	intron_variant
rs1429133598	intron_variant,genic_upstream_transcript_variant
rs1425169536	intron_variant
rs1407411602	intron_variant,genic_upstream_transcript_variant
rs1431986972	intron_variant,genic_upstream_transcript_variant
rs1353555511	intron_variant,genic_upstream_transcript_variant
rs1358885005	intron_variant,genic_upstream_transcript_variant
rs1405900321	intron_variant
rs1485525415	intron_variant
rs1491365122	intron_variant
rs1437620582	intron_variant
rs1411779972	intron_variant,genic_upstream_transcript_variant
rs1474273915	intron_variant
rs1454618665	intron_variant,genic_upstream_transcript_variant
rs1491147401	intron_variant
rs1436894873	genic_upstream_transcript_variant,intron_variant
rs1491184620	intron_variant
rs1491326782	intron_variant
rs753080687	genic_upstream_transcript_variant,intron_variant
rs1360262192	intron_variant
rs1162329827	intron_variant
