# Supplementary material for: An In silico Approach towards Finding the Cancer-Causing Mutations in Human MET Gene
Source: Int J Genomics. 2023 May 9;2023:9705159. doi: 10.1155/2023/9705159 (PMC10188262; doi:10.1155/2023/9705159)
Supplement: Supplementary Materials — Supplementary File 2: SIFT and PROVEAN results. Supplementary File 3: PolyPhen-2 prediction. Supplementary File 4: PANTHER-PSEP prediction. Supplementary File 5: I-Mutant 2.0 and MUpro prediction. Supplementary File 6: Deleterious nsSNPs of MET gene predicted by all computational tools. Supplementary File 7: Effects of nsSNPs on structural and functional properties of MET by MutPred2 server. Supplementary File 8: Conservancy scale of deleterious nsSNPs. Supplementary File 9: Position of deleterious nsSNPs in secondary structure. Supplementary File 10: Deleterious nsSNPs present in post-translational modification sites. Supplementary File 11: Effects of deleterious nsSNPs on different properties of MET protein. Supplementary File 12: Gene interactions data predicted by GeneMANIA. [file 9705159.f2.docx]

**SIFT and PROVEAN Prediction**

| **rsID** | **Amino Acid Substitution** | **SIFT Prediction** | **PROVEAN Prediction** |
| --- | --- | --- | --- |
|  |  |  |  |
| rs41736 | D1286E | TOLERATED | NEUTRAL |
| rs33917957 | N375S | TOLERATED | NEUTRAL |
| rs34349517 | L238S | DELETERIOUS | DELETERIOUS |
| rs34589476 | R970C | TOLERATED | NEUTRAL |
| rs35225896 | I316M | DELETERIOUS | NEUTRAL |
| rs35284565 | R218S | DELETERIOUS | NEUTRAL |
| rs35469582 | R143Q | DELETERIOUS | NEUTRAL |
| rs35601148 | T309P | TOLERATED | NEUTRAL |
| rs35776110 | A320V | DELETERIOUS | DELETERIOUS |
| rs45440991 | R793C | DELETERIOUS | NEUTRAL |
| rs45441497 | L402I | TOLERATED | NEUTRAL |
| rs45446492 | R731Q | TOLERATED | NEUTRAL |
| rs45460604 | Q559R | DELETERIOUS | NEUTRAL |
| rs45483396 | L211W | DELETERIOUS | NEUTRAL |
| rs45551737 | P239R | DELETERIOUS | DELETERIOUS |
| rs45553236 | R739H | TOLERATED | DELETERIOUS |
| rs45561544 | D981E | TOLERATED | NEUTRAL |
| rs45564937 | M1031V | TOLERATED | NEUTRAL |
| rs45575240 | G853C | DELETERIOUS | NEUTRAL |
| rs45578433 | A1363T | TOLERATED | NEUTRAL |
| rs45585831 | T495I | TOLERATED | NEUTRAL |
| rs45586239 | H633L | TOLERATED | NEUTRAL |
| rs45587940 | R739C | DELETERIOUS | DELETERIOUS |
| rs45592846 | T1096S | TOLERATED | NEUTRAL |
| rs45602940 | R591W | DELETERIOUS | DELETERIOUS |
| rs45607832 | R970H | TOLERATED | NEUTRAL |
| rs45612435 | R1022Q | TOLERATED | NEUTRAL |
| rs45628136 | Q1067K | TOLERATED | NEUTRAL |
| rs55985569 | E168D | TOLERATED | NEUTRAL |
| rs56311081 | S156L | DELETERIOUS | DELETERIOUS |
| rs56340719 | F343L | DELETERIOUS | NEUTRAL |
| rs56391007 | T992I | DELETERIOUS | NEUTRAL |
| rs77523018 | M362T | DELETERIOUS | NEUTRAL |
| rs80256822 | A48V | DELETERIOUS | DELETERIOUS |
| rs115574135 | H888Y | TOLERATED | NEUTRAL |
| rs118057172 | N856K | DELETERIOUS | NEUTRAL |
| rs121913243 | H1094R | DELETERIOUS | DELETERIOUS |
| rs121913244 | H1094Y | DELETERIOUS | DELETERIOUS |
| rs121913245 | M1250T | DELETERIOUS | DELETERIOUS |
| rs121913246 | Y1230C | DELETERIOUS | DELETERIOUS |
| rs121913247 | Y1230H | DELETERIOUS | DELETERIOUS |
| rs121913668 | M1131T | DELETERIOUS | DELETERIOUS |
| rs121913669 | V1188L | DELETERIOUS | DELETERIOUS |
| rs121913670 | V1220I | DELETERIOUS | NEUTRAL |
| rs121913671 | D1228N | DELETERIOUS | DELETERIOUS |
| rs121913673 | L1195V | DELETERIOUS | DELETERIOUS |
| rs121913675 | T1173I | TOLERATED | NEUTRAL |
| rs121913676 | M1250I | DELETERIOUS | DELETERIOUS |
| rs121913677 | K1244R | TOLERATED | NEUTRAL |
| rs146651797 | H1174Q | DELETERIOUS | NEUTRAL |
| rs180985111 | G24E | TOLERATED | DELETERIOUS |
| rs185301166 | R266S | DELETERIOUS | NEUTRAL |
| rs199502137 | T895M | DELETERIOUS | NEUTRAL |
| rs199542598 | F590L | TOLERATED | NEUTRAL |
| rs199643166 | R793H | TOLERATED | NEUTRAL |
| rs199701987 | V136I | TOLERATED | NEUTRAL |
| rs199736573 | P97A | TOLERATED | NEUTRAL |
| rs199761604 | S435N | TOLERATED | NEUTRAL |
| rs199763277 | R1166Q | DELETERIOUS | NEUTRAL |
| rs199771406 | S572N | TOLERATED | NEUTRAL |
| rs199771406 | S572T | TOLERATED | NEUTRAL |
| rs200016433 | A364T | DELETERIOUS | DELETERIOUS |
| rs200074800 | A347T | DELETERIOUS | DELETERIOUS |
| rs200218511 | G554R | TOLERATED | DELETERIOUS |
| rs200283364 | P534S | TOLERATED | NEUTRAL |
| rs200345440 | L674F | DELETERIOUS | NEUTRAL |
| rs200633053 | I850V | TOLERATED | NEUTRAL |
| rs200524064 | N795D | TOLERATED | NEUTRAL |
| rs200690492 | D340G | DELETERIOUS | DELETERIOUS |
| rs200740468 | E436Q | TOLERATED | NEUTRAL |
| rs200776610 | T222M | DELETERIOUS | DELETERIOUS |
| rs200819547 | D449N | TOLERATED | NEUTRAL |
| rs200861145 | S203T | TOLERATED | NEUTRAL |
| rs201037977 | G1119A | TOLERATED | NEUTRAL |
| rs201154533 | A411V | TOLERATED | NEUTRAL |
| rs201191014 | K350Q | TOLERATED | NEUTRAL |
| rs201271860 | T733I | DELETERIOUS | NEUTRAL |
| rs201274041 | R359Q | TOLERATED | NEUTRAL |
| rs201315884 | V37A | TOLERATED | NEUTRAL |
| rs201467281 | S323G | TOLERATED | NEUTRAL |
| rs201628326 | H394N | TOLERATED | NEUTRAL |
| rs201687037 | T301A | TOLERATED | NEUTRAL |
| rs201789039 | M431T | TOLERATED | NEUTRAL |
| rs201861645 | L604V | TOLERATED | NEUTRAL |
| rs201975130 | R580T | TOLERATED | NEUTRAL |
| rs201980687 | R413S | TOLERATED | NEUTRAL |
| rs202047059 | T67A | TOLERATED | NEUTRAL |
| rs202166889 | A1384G | TOLERATED | NEUTRAL |
| rs367628460 | R412C | TOLERATED | NEUTRAL |
| rs367634278 | T1126N | DELETERIOUS | NEUTRAL |
| rs368144654 | T273S | TOLERATED | NEUTRAL |
| rs368328347 | D94G | TOLERATED | NEUTRAL |
| rs368750834 | V111I | TOLERATED | NEUTRAL |
| rs368787826 | M822V | TOLERATED | NEUTRAL |
| rs368891381 | K876Q | TOLERATED | NEUTRAL |
| rs368942722 | H61D | TOLERATED | NEUTRAL |
| rs369312680 | R1336Q | TOLERATED | NEUTRAL |
| rs369758288 | M852K | TOLERATED | NEUTRAL |
| rs369838973 | R1170L | DELETERIOUS | DELETERIOUS |
| rs370314484 | Y369C | TOLERATED | NEUTRAL |
| rs370368651 | D1380E | DELETERIOUS | NEUTRAL |
| rs370499060 | E49A | TOLERATED | NEUTRAL |
| rs370529693 | P1073L | TOLERATED | DELETERIOUS |
| rs370767911 | N1374K | TOLERATED | NEUTRAL |
| rs370883654 | T555A | TOLERATED | NEUTRAL |
| rs371124109 | T511M | TOLERATED | NEUTRAL |
| rs371463233 | R412H | TOLERATED | NEUTRAL |
| rs371939364 | S794C | TOLERATED | NEUTRAL |
| rs372116735 | N620D | TOLERATED | NEUTRAL |
| rs372830789 | H1174R | TOLERATED | NEUTRAL |
| rs373030463 | N704D | TOLERATED | NEUTRAL |
| rs373312981 | G471E | TOLERATED | NEUTRAL |
| rs373419003 | P1027A | TOLERATED | NEUTRAL |
| rs373517956 | A1375S | TOLERATED | NEUTRAL |
| rs374050750 | A48T | TOLERATED | NEUTRAL |
| rs374383028 | N1353D | TOLERATED | NEUTRAL |
| rs374578653 | H58R | TOLERATED | DELETERIOUS |
| rs374733251 | T557A | TOLERATED | NEUTRAL |
| rs375353223 | M35L | TOLERATED | NEUTRAL |
| rs375391602 | R413H | TOLERATED | NEUTRAL |
| rs375395179 | S1141L | DELETERIOUS | DELETERIOUS |
| rs375523121 | H780R | TOLERATED | NEUTRAL |
| rs375576430 | S942L | TOLERATED | NEUTRAL |
| rs375951814 | T621I | TOLERATED | NEUTRAL |
| rs376097698 | L185I | TOLERATED | NEUTRAL |
| rs376104371 | L893I | TOLERATED | NEUTRAL |
| rs376243090 | F1012L | TOLERATED | NEUTRAL |
| rs376244358 | M35I | TOLERATED | NEUTRAL |
| rs376364468 | V427I | TOLERATED | NEUTRAL |
| rs376418811 | I1337M | TOLERATED | NEUTRAL |
| rs376459715 | S663L | TOLERATED | NEUTRAL |
| rs376589619 | P472S | TOLERATED | NEUTRAL |
| rs45450897 | I834M | TOLERATED | NEUTRAL |
| rs45471794 | R982M | TOLERATED | NEUTRAL |
| rs45516592 | N768D | TOLERATED | NEUTRAL |
| rs45531032 | I747V | TOLERATED | NEUTRAL |
| rs45532942 | D1265Y | DELETERIOUS | DELETERIOUS |
| rs45541232 | D1180N | DELETERIOUS | DELETERIOUS |
| rs45571834 | I1053T | DELETERIOUS | DELETERIOUS |
| rs45583838 | L1097V | DELETERIOUS | NEUTRAL |
| rs45595632 | T1261A | DELETERIOUS | DELETERIOUS |
| rs45604032 | G933R | TOLERATED | DELETERIOUS |
| rs45605635 | Y649C | TOLERATED | NEUTRAL |
| rs56361366 | S661R | DELETERIOUS | NEUTRAL |
| rs77651398 | N45S | TOLERATED | NEUTRAL |
| rs148526613 | N381T | TOLERATED | NEUTRAL |
| rs189810227 | D1377N | TOLERATED | NEUTRAL |
| rs199595181 | H148N | TOLERATED | NEUTRAL |
| rs199808716 | G757E | DELETERIOUS | DELETERIOUS |
| rs200023903 | T67I | TOLERATED | NEUTRAL |
| rs200269358 | N571T | TOLERATED | NEUTRAL |
| rs200341323 | N686K | DELETERIOUS | NEUTRAL |
| rs201090828 | L675V | TOLERATED | NEUTRAL |
| rs202110450 | H1136Q | DELETERIOUS | NEUTRAL |
| rs202135726 | N680K | TOLERATED | NEUTRAL |
| rs202236031 | L403M | DELETERIOUS | NEUTRAL |
| rs267601244 | P1285S | TOLERATED | DELETERIOUS |
| rs367722737 | S213L | DELETERIOUS | DELETERIOUS |
| rs368312290 | P1322A | TOLERATED | DELETERIOUS |
| rs371941340 | F96C | TOLERATED | NEUTRAL |
| rs372320153 | D77N | TOLERATED | NEUTRAL |
| rs372699296 | V836I | TOLERATED | NEUTRAL |
| rs376751279 | E1378V | TOLERATED | DELETERIOUS |
| rs376928397 | I799N | DELETERIOUS | DELETERIOUS |

**PolyPhen-2 Prediction**

| **rsID** | **Amino Acid Substitution** | **HumDiv** | | **HumVar** | |
| --- | --- | --- | --- | --- | --- |
|  |  | **Prediction** | **Score** | **Prediction** | **Score** |
| rs41736 | D1286E | PROBABLY DAMAGING | 0.997 | PROBABLY DAMAGING | 0.983 |
| rs33917957 | N375S | BENIGN | 0.038 | BENIGN | 0.010 |
| rs34349517 | L238S | PROBABLY DAMAGING | 1.00 | PROBABLY DAMAGING | 1.00 |
| rs34589476 | R970C | BENIGN | 0.026 | BENIGN | 0.010 |
| rs35225896 | I316M | PROBABLY DAMAGING | 0.990 | PROBABLY DAMAGING | 0.975 |
| rs35284565 | R218S | PROBABLY DAMAGING | 1.000 | PROBABLY DAMAGING | 1.000 |
| rs35469582 | R143Q | PROBABLY DAMAGING | 1.000 | PROBABLY DAMAGING | 0.999 |
| rs35601148 | T309P | BENIGN | 0.002 | BENIGN | 0.007 |
| rs35776110 | A320V | PROBABLY DAMAGING | 1.000 | PROBABLY DAMAGING | 0.997 |
| rs45440991 | R793C | PROBABLY DAMAGING | 1.000 | PROBABLY DAMAGING | 0.958 |
| rs45441497 | L402I | BENIGN | 0.232 | BENIGN | 0.356 |
| rs45446492 | R731Q | PROBABLY DAMAGING | 1.000 | POSSIBLY DAMAGING | 0.870 |
| rs45460604 | Q559R | BENIGN | 0.004 | BENIGN | 0.040 |
| rs45483396 | L211W | PROBABLY DAMAGING | 1.000 | PROBABLY DAMAGING | 0.994 |
| rs45551737 | P239R | PROBABLY DAMAGING | 1.000 | PROBABLY DAMAGING | 1.000 |
| rs45553236 | R739H | POSSIBLY DAMAGING | 0.868 | BENIGN | 0.255 |
| rs45561544 | D981E | PROBABLY DAMAGING | 0.995 | PROBABLY DAMAGING | 0.985 |
| rs45564937 | M1031V | BENIGN | 0.001 | BENIGN | 0.002 |
| rs45575240 | G853C | PROBABLY DAMAGING | 0.998 | POSSIBLY DAMAGING | 0.872 |
| rs45578433 | A1363T | POSSIBLY DAMAGING | 0.955 | BENIGN | 0.262 |
| rs45585831 | T495I | BENIGN | 0.023 | BENIGN | 0.048 |
| rs45586239 | H633L | BENIGN | 0.107 | BENIGN | 0.012 |
| rs45587940 | R739C | PROBABLY DAMAGING | 1.000 | PROBABLY DAMAGING | 0.944 |
| rs45592846 | T1096S | PROBABLY DAMAGING | 0.999 | PROBABLY DAMAGING | 1.000 |
| rs45602940 | R591W | PROBABLY DAMAGING | 0.995 | POSSIBLY DAMAGING | 0.802 |
| rs45607832 | R970H | PROBABLY DAMAGING | 0.995 | POSSIBLY DAMAGING | 0.796 |
| rs45612435 | R1022Q | PROBABLY DAMAGING | 0.999 | PROBABLY DAMAGING | 0.986 |
| rs45628136 | Q1067K | BENIGN | 0.083 | BENIGN | 0.019 |
| rs55985569 | E168D | POSSIBLY DAMAGING | 0.807 | POSSIBLY DAMAGING | 0.582 |
| rs56311081 | S156L | POSSIBLY DAMAGING | 0.858 | POSSIBLY DAMAGING | 0.548 |
| rs56340719 | F343L | POSSIBLY DAMAGING | 0.643 | BENIGN | 0.203 |
| rs56391007 | T992I | PROBABLY DAMAGING | 0.999 | PROBABLY DAMAGING | 0.997 |
| rs77523018 | M362T | BENIGN | 0.038 | BENIGN | 0.087 |
| rs80256822 | A48V | POSSIBLY DAMAGING | 0.758 | BENIGN | 0.069 |
| rs115574135 | H888Y | BENIGN | 0.000 | BENIGN | 0.000 |
| rs118057172 | N856K | POSSIBLY DAMAGING | 0.900 | BENIGN | 0.438 |
| rs121913243 | H1094R | PROBABLY DAMAGING | 1.000 | PROBABLY DAMAGING | 0.997 |
| rs121913244 | H1094Y | PROBABLY DAMAGING | 0.999 | PROBABLY DAMAGING | 0.997 |
| rs121913245 | M1250T | PROBABLY DAMAGING | 1.000 | PROBABLY DAMAGING | 0.998 |
| rs121913246 | Y1230C | PROBABLY DAMAGING | 1.000 | PROBABLY DAMAGING | 1.000 |
| rs121913247 | Y1230H | PROBABLY DAMAGING | 1.000 | PROBABLY DAMAGING | 1.000 |
| rs121913668 | M1131T | PROBABLY DAMAGING | 1.000 | PROBABLY DAMAGING | 0.998 |
| rs121913669 | V1188L | PROBABLY DAMAGING | 0.980 | PROBABLY DAMAGING | 0.977 |
| rs121913670 | V1220I | PROBABLY DAMAGING | 0.980 | PROBABLY DAMAGING | 0.977 |
| rs121913671 | D1228N | PROBABLY DAMAGING | 1.000 | PROBABLY DAMAGING | 0.999 |
| rs121913673 | L1195V | PROBABLY DAMAGING | 0.998 | PROBABLY DAMAGING | 0.998 |
| rs121913675 | T1173I | POSSIBLY DAMAGING | 0.956 | POSSIBLY DAMAGING | 0.590 |
| rs121913676 | M1250I | PROBABLY DAMAGING | 0.975 | PROBABLY DAMAGING | 0.968 |
| rs121913677 | K1244R | PROBABLY DAMAGING | 0.994 | PROBABLY DAMAGING | 0.995 |
| rs146651797 | H1174Q | PROBABLY DAMAGING | 1.000 | PROBABLY DAMAGING | 0.997 |
| rs180985111 | G24E | BENIGN | 0.017 | BENIGN | 0.023 |
| rs185301166 | R266S | POSSIBLY DAMAGING | 0.954 | PROBABLY DAMAGING | 0.925 |
| rs199502137 | T895M | PROBABLY DAMAGING | 0.996 | POSSIBLY DAMAGING | 0.749 |
| rs199542598 | F590L | BENIGN | 0.108 | BENIGN | 0.058 |
| rs199643166 | R793H | PROBABLY DAMAGING | 0.999 | PROBABLY DAMAGING | 0.944 |
| rs199701987 | V136I | POSSIBLY DAMAGING | 0.763 | BENIGN | 0.204 |
| rs199736573 | P97A | PROBABLY DAMAGING | 1.000 | PROBABLY DAMAGING | 1.000 |
| rs199761604 | S435N | BENIGN | 0.000 | BENIGN | 0.000 |
| rs199763277 | R1166Q | PROBABLY DAMAGING | 1.000 | PROBABLY DAMAGING | 0.999 |
| rs199771406 | S572N | BENIGN | 0.233 | BENIGN | 0.133 |
| rs199771406 | S572T | BENIGN | 0.001 | BENIGN | 0.003 |
| rs200016433 | A364T | PROBABLY DAMAGING | 1.000 | PROBABLY DAMAGING | 0.999 |
| rs200074800 | A347T | PROBABLY DAMAGING | 0.985 | POSSIBLY DAMAGING | 0.662 |
| rs200218511 | G554R | PROBABLY DAMAGING | 0.999 | PROBABLY DAMAGING | 0.942 |
| rs200283364 | P534S | BENIGN | 0.003 | BENIGN | 0.011 |
| rs200345440 | L674F | PROBABLY DAMAGING | 0.992 | PROBABLY DAMAGING | 0.922 |
| rs200633053 | I850V | BENIGN | 0.013 | BENIGN | 0.011 |
| rs200524064 | N795D | POSSIBLY DAMAGING | 0.608 | BENIGN | 0.185 |
| rs200690492 | D340G | PROBABLY DAMAGING | 1.000 | PROBABLY DAMAGING | 1.000 |
| rs200740468 | E436Q | BENIGN | 0.000 | BENIGN | 0.000 |
| rs200776610 | T222M | PROBABLY DAMAGING | 1.000 | PROBABLY DAMAGING | 1.000 |
| rs200819547 | D449N | BENIGN | 0.001 | BENIGN | 0.004 |
| rs200861145 | S203T | BENIGN | 0.043 | BENIGN | 0.041 |
| rs201037977 | G1119A | BENIGN | 0.428 | BENIGN | 0.260 |
| rs201154533 | A411V | BENIGN | 0.000 | BENIGN | 0.000 |
| rs201191014 | K350Q | BENIGN | 0.012 | BENIGN | 0.202 |
| rs201271860 | T733I | BENIGN | 0.001 | BENIGN | 0.003 |
| rs201274041 | R359Q | PROBABLY DAMAGING | 1.000 | PROBABLY DAMAGING | 0.919 |
| rs201315884 | V37A | BENIGN | 0.113 | BENIGN | 0.031 |
| rs201467281 | S323G | BENIGN | 0.015 | BENIGN | 0.002 |
| rs201628326 | H394N | PROBABLY DAMAGING | 0.977 | POSSIBLY DAMAGING | 0.810 |
| rs201687037 | T301A | PROBABLY DAMAGING | 0.997 | PROBABLY DAMAGING | 0.993 |
| rs201789039 | M431T | BENIGN | 0.001 | BENIGN | 0.004 |
| rs201861645 | L604V | BENIGN | 0.134 | BENIGN | 0.138 |
| rs201975130 | R580T | BENIGN | 0.001 | BENIGN | 0.002 |
| rs201980687 | R413S | BENIGN | 0.003 | BENIGN | 0.028 |
| rs202047059 | T67A | BENIGN | 0.040 | BENIGN | 0.036 |
| rs202166889 | A1384G | BENIGN | 0.000 | BENIGN | 0.000 |
| rs367628460 | R412C | PROBABLY DAMAGING | 1.000 | PROBABLY DAMAGING | 0.991 |
| rs367634278 | T1126N | PROBABLY DAMAGING | 0.966 | PROBABLY DAMAGING | 0.977 |
| rs368144654 | T273S | BENIGN | 0.003 | BENIGN | 0.148 |
| rs368328347 | D94G | BENIGN | 0.005 | BENIGN | 0.032 |
| rs368750834 | V111I | BENIGN | 0.000 | BENIGN | 0.000 |
| rs368787826 | M822V | BENIGN | 0.000 | BENIGN | 0.001 |
| rs368891381 | K876Q | PROBABLY DAMAGING | 0.999 | PROBABLY DAMAGING | 0.996 |
| rs368942722 | H61D | BENIGN | 0.141 | BENIGN | 0.230 |
| rs369312680 | R1336Q | PROBABLY DAMAGING | 0.902 | BENIGN | 0.189 |
| rs369758288 | M852K | BENIGN | 0.000 | BENIGN | 0.000 |
| rs369838973 | R1170L | PROBABLY DAMAGING | 1.000 | PROBABLY DAMAGING | 1.000 |
| rs370314484 | Y369C | PROBABLY DAMAGING | 0.998 | PROBABLY DAMAGING | 0.929 |
| rs370368651 | D1380E | POSSIBLY DAMAGING | 0.748 | BENIGN | 0.119 |
| rs370499060 | E49A | POSSIBLY DAMAGING | 0.527 | BENIGN | 0.349 |
| rs370529693 | P1073L | PROBABLY DAMAGING | 0.976 | POSSIBLY DAMAGING | 0.450 |
| rs370767911 | N1374K | BENIGN | 0.138 | BENIGN | 0.040 |
| rs370883654 | T555A | BENIGN | 0.000 | BENIGN | 0.003 |
| rs371124109 | T511M | PROBABLY DAMAGING | 1.000 | POSSIBLY DAMAGING | 0.831 |
| rs371463233 | R412H | BENIGN | 0.139 | BENIGN | 0.017 |
| rs371939364 | S794C | PROBABLY DAMAGING | 0.999 | PROBABLY DAMAGING | 0.956 |
| rs372116735 | N620D | PROBABLY DAMAGING | 0.990 | POSSIBLY DAMAGING | 0.855 |
| rs372830789 | H1174R | PROBABLY DAMAGING | 0.999 | PROBABLY DAMAGING | 0.990 |
| rs373030463 | N704D | BENIGN | 0.000 | BENIGN | 0.001 |
| rs373312981 | G471E | BENIGN | 0.001 | BENIGN | 0.004 |
| rs373419003 | P1027A | POSSIBLY DAMAGING | 0.883 | BENIGN | 0.268 |
| rs373517956 | A1375S | BENIGN | 0.001 | BENIGN | 0.001 |
| rs374050750 | A48T | BENIGN | 0.083 | BENIGN | 0.006 |
| rs374383028 | N1353D | PROBABLY DAMAGING | 0.999 | PROBABLY DAMAGING | 0.995 |
| rs374578653 | H58R | POSSIBLY DAMAGING | 0.699 | BENIGN | 0.235 |
| rs374733251 | T557A | PROBABLY DAMAGING | 0.968 | PROBABLY DAMAGING | 0.779 |
| rs375353223 | M35L | BENIGN | 0.319 | BENIGN | 0.075 |
| rs375391602 | R413H | BENIGN | 0.004 | BENIGN | 0.004 |
| rs375395179 | S1141L | PROBABLY DAMAGING | 1.000 | PROBABLY DAMAGING | 0.999 |
| rs375523121 | H780R | BENIGN | 0.001 | BENIGN | 0.004 |
| rs375576430 | S942L | BENIGN | 0.007 | BENIGN | 0.006 |
| rs375951814 | T621I | BENIGN | 0.115 | BENIGN | 0.031 |
| rs376097698 | L185I | PROBABLY DAMAGING | 0.999 | PROBABLY DAMAGING | 0.999 |
| rs376104371 | L893I | POSSIBLY DAMAGING | 0.819 | BENIGN | 0.355 |
| rs376243090 | F1012L | BENIGN | 0.240 | BENIGN | 0.090 |
| rs376244358 | M35I | POSSIBLY DAMAGING | 0.839 | BENIGN | 0.164 |
| rs376364468 | V427I | BENIGN | 0.003 | BENIGN | 0.004 |
| rs376418811 | I1337M | PROBABLY DAMAGING | 1.000 | PROBABLY DAMAGING | 1.000 |
| rs376459715 | S663L | PROBABLY DAMAGING | 0.970 | POSSIBLY DAMAGING | 0.512 |
| rs376589619 | P472S | BENIGN | 0.000 | BENIGN | 0.001 |
| rs45450897 | I834M | BENIGN | 0.306 | BENIGN | 0.278 |
| rs45471794 | R982M | PROBABLY DAMAGING | 1.000 | PROBABLY DAMAGING | 0.995 |
| rs45516592 | N768D | BENIGN | 0.004 | BENIGN | 0.014 |
| rs45531032 | I747V | BENIGN | 0.313 | BENIGN | 0.337 |
| rs45532942 | D1265Y | PROBABLY DAMAGING | 1.000 | PROBABLY DAMAGING | 1.000 |
| rs45541232 | D1180N | PROBABLY DAMAGING | 1.000 | PROBABLY DAMAGING | 0.999 |
| rs45571834 | I1053T | PROBABLY DAMAGING | 1.000 | PROBABLY DAMAGING | 1.000 |
| rs45583838 | L1097V | PROBABLY DAMAGING | 0.995 | PROBABLY DAMAGING | 0.996 |
| rs45595632 | T1261A | PROBABLY DAMAGING | 0.999 | PROBABLY DAMAGING | 0.996 |
| rs45604032 | G933R | POSSIBLY DAMAGING | 0.790 | BENIGN | 0.440 |
| rs45605635 | Y649C | PROBABLY DAMAGING | 0.997 | PROBABLY DAMAGING | 0.929 |
| rs56361366 | S661R | POSSIBLY DAMAGING | 0.926 | POSSIBLY DAMAGING | 0.605 |
| rs77651398 | N45S | PROBABLY DAMAGING | 0.988 | BENIGN | 0.285 |
| rs148526613 | N381T | POSSIBLY DAMAGING | 0.513 | BENIGN | 0.247 |
| rs189810227 | D1377N | BENIGN | 0.004 | BENIGN | 0.008 |
| rs199595181 | H148N | BENIGN | 0.087 | POSSIBLY DAMAGING | 0.521 |
| rs199808716 | G757E | PROBABLY DAMAGING | 1.000 | PROBABLY DAMAGING | 0.983 |
| rs200023903 | T67I | BENIGN | 0.000 | BENIGN | 0.001 |
| rs200269358 | N571T | BENIGN | 0.000 | BENIGN | 0.000 |
| rs200341323 | N686K | BENIGN | 0.011 | BENIGN | 0.013 |
| rs201090828 | L675V | BENIGN | 0.004 | BENIGN | 0.027 |
| rs202110450 | H1136Q | PROBABLY DAMAGING | 1.000 | PROBABLY DAMAGING | 0.999 |
| rs202135726 | N680K | BENIGN | 0.000 | BENIGN | 0.000 |
| rs202236031 | L403M | PROBABLY DAMAGING | 0.997 | PROBABLY DAMAGING | 0.964 |
| rs267601244 | P1285S | PROBABLY DAMAGING | 1.000 | PROBABLY DAMAGING | 0.999 |
| rs367722737 | S213L | PROBABLY DAMAGING | 1.000 | PROBABLY DAMAGING | 0.999 |
| rs368312290 | P1322A | PROBABLY DAMAGING | 1.000 | PROBABLY DAMAGING | 1.000 |
| rs371941340 | F96C | POSSIBLY DAMAGING | 0.950 | POSSIBLY DAMAGING | 0.847 |
| rs372320153 | D77N | BENIGN | 0.000 | BENIGN | 0.004 |
| rs372699296 | V836I | POSSIBLY DAMAGING | 0.789 | BENIGN | 0.339 |
| rs376751279 | E1378V | BENIGN | 0.000 | BENIGN | 0.001 |
| rs376928397 | I799N | POSSIBLY DAMAGING | 0.681 | BENIGN | 0.376 |

**PANTHER-PSEP Prediction**

| **rsID** | **Amino Acid Substitution** | **PANTHER Prediction** |
| --- | --- | --- |
|  |  |  |
| rs41736 | D1286E | PROBABLY DAMAGING |
| rs33917957 | N375S | PROBABLY DAMAGING |
| rs34349517 | L238S | PROBABLY DAMAGING |
| rs34589476 | R970C | POSSIBLY DAMAGING |
| rs35225896 | I316M | POSSIBLY DAMAGING |
| rs35284565 | R218S | PROBABLY DAMAGING |
| rs35469582 | R143Q | PROBABLY DAMAGING |
| rs35601148 | T309P | PROBABLY DAMAGING |
| rs35776110 | A320V | PROBABLY DAMAGING |
| rs45440991 | R793C | PROBABLY DAMAGING |
| rs45441497 | L402I | PROBABLY BENIGN |
| rs45446492 | R731Q | PROBABLY DAMAGING |
| rs45460604 | Q559R | PROBABLY BENIGN |
| rs45483396 | L211W | POSSIBLY DAMAGING |
| rs45551737 | P239R | PROBABLY DAMAGING |
| rs45553236 | R739H | PROBABLY BENIGN |
| rs45561544 | D981E | PROBABLY DAMAGING |
| rs45564937 | M1031V | PROBABLY BENIGN |
| rs45575240 | G853C | POSSIBLY DAMAGING |
| rs45578433 | A1363T | POSSIBLY DAMAGING |
| rs45585831 | T495I | PROBABLY BENIGN |
| rs45586239 | H633L | POSSIBLY DAMAGING |
| rs45587940 | R739C | PROBABLY BENIGN |
| rs45592846 | T1096S | PROBABLY DAMAGING |
| rs45602940 | R591W | POSSIBLY DAMAGING |
| rs45607832 | R970H | POSSIBLY DAMAGING |
| rs45612435 | R1022Q | PROBABLY DAMAGING |
| rs45628136 | Q1067K | POSSIBLY DAMAGING |
| rs55985569 | E168D | POSSIBLY DAMAGING |
| rs56311081 | S156L | PROBABLY DAMAGING |
| rs56340719 | F343L | PROBABLY BENIGN |
| rs56391007 | T992I | PROBABLY DAMAGING |
| rs77523018 | M362T | PROBABLY BENIGN |
| rs80256822 | A48V | POSSIBLY DAMAGING |
| rs115574135 | H888Y | POSSIBLY DAMAGING |
| rs118057172 | N856K | PROBABLY DAMAGING |
| rs121913243 | H1094R | PROBABLY DAMAGING |
| rs121913244 | H1094Y | PROBABLY DAMAGING |
| rs121913245 | M1250T | PROBABLY DAMAGING |
| rs121913246 | Y1230C | PROBABLY DAMAGING |
| rs121913247 | Y1230H | PROBABLY DAMAGING |
| rs121913668 | M1131T | PROBABLY DAMAGING |
| rs121913669 | V1188L | PROBABLY DAMAGING |
| rs121913670 | V1220I | PROBABLY DAMAGING |
| rs121913671 | D1228N | PROBABLY DAMAGING |
| rs121913673 | L1195V | PROBABLY DAMAGING |
| rs121913675 | T1173I | POSSIBLY DAMAGING |
| rs121913676 | M1250I | PROBABLY DAMAGING |
| rs121913677 | K1244R | PROBABLY DAMAGING |
| rs146651797 | H1174Q | PROBABLY DAMAGING |
| rs180985111 | G24E | PROBABLY DAMAGING |
| rs185301166 | R266S | PROBABLY DAMAGING |
| rs199502137 | T895M | POSSIBLY DAMAGING |
| rs199542598 | F590L | PROBABLY DAMAGING |
| rs199643166 | R793H | PROBABLY DAMAGING |
| rs199701987 | V136I | POSSIBLY DAMAGING |
| rs199736573 | P97A | PROBABLY DAMAGING |
| rs199761604 | S435N | PROBABLY BENIGN |
| rs199763277 | R1166Q | PROBABLY DAMAGING |
| rs199771406 | S572N | PROBABLY DAMAGING |
| rs199771406 | S572T | PROBABLY DAMAGING |
| rs200016433 | A364T | PROBABLY DAMAGING |
| rs200074800 | A347T | PROBABLY DAMAGING |
| rs200218511 | G554R | POSSIBLY DAMAGING |
| rs200283364 | P534S | PROBABLY BENIGN |
| rs200345440 | L674F | POSSIBLY DAMAGING |
| rs200633053 | I850V | POSSIBLY DAMAGING |
| rs200524064 | N795D | POSSIBLY DAMAGING |
| rs200690492 | D340G | PROBABLY DAMAGING |
| rs200740468 | E436Q | PROBABLY BENIGN |
| rs200776610 | T222M | PROBABLY DAMAGING |
| rs200819547 | D449N | PROBABLY BENIGN |
| rs200861145 | S203T | POSSIBLY DAMAGING |
| rs201037977 | G1119A | POSSIBLY DAMAGING |
| rs201154533 | A411V | POSSIBLY DAMAGING |
| rs201191014 | K350Q | PROBABLY DAMAGING |
| rs201271860 | T733I | PROBABLY BENIGN |
| rs201274041 | R359Q | POSSIBLY DAMAGING |
| rs201315884 | V37A | POSSIBLY DAMAGING |
| rs201467281 | S323G | POSSIBLY DAMAGING |
| rs201628326 | H394N | POSSIBLY DAMAGING |
| rs201687037 | T301A | PROBABLY DAMAGING |
| rs201789039 | M431T | PROBABLY DAMAGING |
| rs201861645 | L604V | POSSIBLY DAMAGING |
| rs201975130 | R580T | PROBABLY BENIGN |
| rs201980687 | R413S | PROBABLY BENIGN |
| rs202047059 | T67A | PROBABLY BENIGN |
| rs202166889 | A1384G | PROBABLY BENIGN |
| rs367628460 | R412C | POSSIBLY DAMAGING |
| rs367634278 | T1126N | POSSIBLY DAMAGING |
| rs368144654 | T273S | POSSIBLY DAMAGING |
| rs368328347 | D94G | POSSIBLY DAMAGING |
| rs368750834 | V111I | PROBABLY BENIGN |
| rs368787826 | M822V | PROBABLY BENIGN |
| rs368891381 | K876Q | PROBABLY DAMAGING |
| rs368942722 | H61D | POSSIBLY DAMAGING |
| rs369312680 | R1336Q | PROBABLY BENIGN |
| rs369758288 | M852K | PROBABLY BENIGN |
| rs369838973 | R1170L | PROBABLY DAMAGING |
| rs370314484 | Y369C | PROBABLY BENIGN |
| rs370368651 | D1380E | POSSIBLY DAMAGING |
| rs370499060 | E49A | POSSIBLY DAMAGING |
| rs370529693 | P1073L | POSSIBLY DAMAGING |
| rs370767911 | N1374K | PROBABLY DAMAGING |
| rs370883654 | T555A | PROBABLY BENIGN |
| rs371124109 | T511M | PROBABLY DAMAGING |
| rs371463233 | R412H | POSSIBLY DAMAGING |
| rs371939364 | S794C | POSSIBLY DAMAGING |
| rs372116735 | N620D | PROBABLY DAMAGING |
| rs372830789 | H1174R | PROBABLY DAMAGING |
| rs373030463 | N704D | PROBABLY BENIGN |
| rs373312981 | G471E | POSSIBLY DAMAGING |
| rs373419003 | P1027A | POSSIBLY DAMAGING |
| rs373517956 | A1375S | PROBABLY BENIGN |
| rs374050750 | A48T | POSSIBLY DAMAGING |
| rs374383028 | N1353D | PROBABLY DAMAGING |
| rs374578653 | H58R | POSSIBLY DAMAGING |
| rs374733251 | T557A | POSSIBLY DAMAGING |
| rs375353223 | M35L | PROBABLY DAMAGING |
| rs375391602 | R413H | PROBABLY BENIGN |
| rs375395179 | S1141L | PROBABLY DAMAGING |
| rs375523121 | H780R | PROBABLY BENIGN |
| rs375576430 | S942L | POSSIBLY DAMAGING |
| rs375951814 | T621I | PROBABLY BENIGN |
| rs376097698 | L185I | PROBABLY DAMAGING |
| rs376104371 | L893I | POSSIBLY DAMAGING |
| rs376243090 | F1012L | PROBABLY DAMAGING |
| rs376244358 | M35I | PROBABLY DAMAGING |
| rs376364468 | V427I | PROBABLY BENIGN |
| rs376418811 | I1337M | PROBABLY DAMAGING |
| rs376459715 | S663L | POSSIBLY DAMAGING |
| rs376589619 | P472S | PROBABLY BENIGN |
| rs45450897 | I834M | POSSIBLY DAMAGING |
| rs45471794 | R982M | PROBABLY DAMAGING |
| rs45516592 | N768D | PROBABLY DAMAGING |
| rs45531032 | I747V | PROBABLY DAMAGING |
| rs45532942 | D1265Y | PROBABLY DAMAGING |
| rs45541232 | D1180N | PROBABLY DAMAGING |
| rs45571834 | I1053T | PROBABLY DAMAGING |
| rs45583838 | L1097V | PROBABLY DAMAGING |
| rs45595632 | T1261A | PROBABLY DAMAGING |
| rs45604032 | G933R | PROBABLY DAMAGING |
| rs45605635 | Y649C | PROBABLY BENIGN |
| rs56361366 | S661R | PROBABLY BENIGN |
| rs77651398 | N45S | POSSIBLY DAMAGING |
| rs148526613 | N381T | POSSIBLY DAMAGING |
| rs189810227 | D1377N | PROBABLY BENIGN |
| rs199595181 | H148N | PROBABLY BENIGN |
| rs199808716 | G757E | PROBABLY DAMAGING |
| rs200023903 | T67I | PROBABLY BENIGN |
| rs200269358 | N571T | PROBABLY BENIGN |
| rs200341323 | N686K | POSSIBLY DAMAGING |
| rs201090828 | L675V | POSSIBLY DAMAGING |
| rs202110450 | H1136Q | PROBABLY DAMAGING |
| rs202135726 | N680K | PROBABLY BENIGN |
| rs202236031 | L403M | POSSIBLY DAMAGING |
| rs267601244 | P1285S | PROBABLY DAMAGING |
| rs367722737 | S213L | PROBABLY DAMAGING |
| rs368312290 | P1322A | PROBABLY DAMAGING |
| rs371941340 | F96C | PROBABLY BENIGN |
| rs372320153 | D77N | POSSIBLY DAMAGING |
| rs372699296 | V836I | PROBABLY DAMAGING |
| rs376751279 | E1378V | POSSIBLY DAMAGING |
| rs376928397 | I799N | PROBABLY DAMAGING |

**I-Mutant 2.0 and MUpro Prediction**

| **rsID** | **Amino Acid Substitution** | **I-Mutant 2.0** | | **MUpro** | |
| --- | --- | --- | --- | --- | --- |
|  |  | **Prediction** | **ΔΔG value** | **Prediction** | **ΔΔG value** |
| rs41736 | D1286E | Decrease | -0.58 | Decrease | -0.47064191 |
| rs33917957 | N375S | Decrease | -1.41 | Decrease | -1.2449739 |
| rs34349517 | L238S | Decrease | -3.29 | Decrease | -1.7819785 |
| rs34589476 | R970C | Decrease | -1.56 | Decrease | -0.63466933 |
| rs35225896 | I316M | Decrease | -1.37 | Decrease | -1.1752522 |
| rs35284565 | R218S | Decrease | -2.37 | Decrease | -1.7229467 |
| rs35469582 | R143Q | Decrease | -1.29 | Decrease | -0.64424568 |
| rs35601148 | T309P | Decrease | -3.38 | Decrease | -1.7014609 |
| rs35776110 | A320V | Decrease | -0.45 | Decrease | -0.73871906 |
| rs45440991 | R793C | Decrease | -0.64 | Decrease | -0.50788821 |
| rs45441497 | L402I | Decrease | -0.47 | Decrease | -1.0202772 |
| rs45446492 | R731Q | Decrease | -1.40 | Decrease | -1.0323208 |
| rs45460604 | Q559R | Decrease | -0.95 | Decrease | -0.2363657 |
| rs45483396 | L211W | Increase | 0.57 | Decrease | -1.2475528 |
| rs45551737 | P239R | Decrease | -0.99 | Decrease | -0.32881731 |
| rs45553236 | R739H | Decrease | -2.16 | Decrease | -1.0124361 |
| rs45561544 | D981E | Decrease | -0.76 | Decrease | -1.266302 |
| rs45564937 | M1031V | Decrease | -0.09 | Decrease | -1.0631076 |
| rs45575240 | G853C | Decrease | -0.25 | Decrease | -0.41928002 |
| rs45578433 | A1363T | Decrease | -0.96 | Decrease | -0.39642151 |
| rs45585831 | T495I | Decrease | -0.56 | Decrease | -0.26668503 |
| rs45586239 | H633L | Increase | 0.53 | Decrease | -0.096095566 |
| rs45587940 | R739C | Decrease | -0.82 | Decrease | -0.55216388 |
| rs45592846 | T1096S | Decrease | -0.66 | Decrease | -1.1898198 |
| rs45602940 | R591W | Decrease | -1.12 | Decrease | -0.76703795 |
| rs45607832 | R970H | Decrease | -2.08 | Decrease | -0.94960965 |
| rs45612435 | R1022Q | Decrease | -1.40 | Decrease | -0.60721832 |
| rs45628136 | Q1067K | Decrease | -0.10 | Decrease | -1.3638094 |
| rs55985569 | E168D | Decrease | -0.27 | Decrease | -0.60217382 |
| rs56311081 | S156L | Decrease | -0.14 | Increase | 0.3600935 |
| rs56340719 | F343L | Decrease | -2.68 | Decrease | -0.65629612 |
| rs56391007 | T992I | Increase | 0.21 | Increase | 0.05116573 |
| rs77523018 | M362T | Decrease | -1.51 | Decrease | -1.7133026 |
| rs80256822 | A48V | Decrease | -0.38 | Decrease | -0.3747182 |
| rs115574135 | H888Y | Increase | 1.20 | Decrease | -0.29403231 |
| rs118057172 | N856K | Decrease | -0.12 | Decrease | -1.1389299 |
| rs121913243 | H1094R | Decrease | -1.40 | Decrease | -0.45113944 |
| rs121913244 | H1094Y | Decrease | -0.34 | Decrease | -0.23100286 |
| rs121913245 | M1250T | Increase | 1.06 | Decrease | -1.4147514 |
| rs121913246 | Y1230C | Increase | 0.04 | Decrease | -1.6434112 |
| rs121913247 | Y1230H | Decrease | -1.73 | Decrease | -1.8945526 |
| rs121913668 | M1131T | Decrease | -1.21 | Decrease | -1.7033143 |
| rs121913669 | V1188L | Decrease | -1.82 | Decrease | -0.19291231 |
| rs121913670 | V1220I | Decrease | -1.39 | Decrease | -0.34372268 |
| rs121913671 | D1228N | Decrease | -1.34 | Decrease | -1.4521681 |
| rs121913673 | L1195V | Decrease | -1.22 | Decrease | -0.78586704 |
| rs121913675 | T1173I | Decrease | -2.46 | Decrease | -0.96579784 |
| rs121913676 | M1250I | Increase | 1.31 | Decrease | -0.59671157 |
| rs121913677 | K1244R | Decrease | -0.32 | Decrease | -0.26790801 |
| rs146651797 | H1174Q | Decrease | -1.55 | Decrease | -0.63189718 |
| rs180985111 | G24E | Decrease | -0.39 | Decrease | -0.89916858 |
| rs185301166 | R266S | Decrease | -1.55 | Decrease | -0.61625597 |
| rs199502137 | T895M | Decrease | -0.33 | Decrease | -0.27990537 |
| rs199542598 | F590L | Decrease | -2.96 | Decrease | -0.41402604 |
| rs199643166 | R793H | Decrease | -1.40 | Decrease | -0.88082 |
| rs199701987 | V136I | Decrease | -0.67 | Decrease | -0.48259271 |
| rs199736573 | P97A | Decrease | -2.02 | Decrease | -0.97126313 |
| rs199761604 | S435N | Decrease | -1.03 | Decrease | -0.19767504 |
| rs199763277 | R1166Q | Decrease | -1.54 | Decrease | -0.19767504 |
| rs199771406 | S572N | Decrease | -0.26 | Decrease | -0.45394493 |
| rs199771406 | S572T | Decrease | -1.35 | Decrease | -0.50611592 |
| rs200016433 | A364T | Decrease | -1.40 | Decrease | -1.1973565 |
| rs200074800 | A347T | Decrease | -1.66 | Decrease | -1.1973565 |
| rs200218511 | G554R | Decrease | -1.10 | Decrease | -0.53059519 |
| rs200283364 | P534S | Decrease | -0.90 | Decrease | -0.8803707 |
| rs200345440 | L674F | Decrease | -0.79 | Decrease | -0.80495212 |
| rs200633053 | I850V | Decrease | -0.59 | Decrease | -0.87574969 |
| rs200524064 | N795D | Decrease | -0.73 | Decrease | -0.68089162 |
| rs200690492 | D340G | Decrease | -2.20 | Decrease | -2.0429035 |
| rs200740468 | E436Q | Decrease | -0.82 | Decrease | -0.87607894 |
| rs200776610 | T222M | Decrease | -0.90 | Decrease | -0.69817409 |
| rs200819547 | D449N | Decrease | -2.70 | Decrease | -1.3411227 |
| rs200861145 | S203T | Decrease | -1.15 | Decrease | -0.60662339 |
| rs201037977 | G1119A | Decrease | -2.07 | Decrease | -1.1496808 |
| rs201154533 | A411V | Increase | 1.12 | Decrease | -0.42880162 |
| rs201191014 | K350Q | Decrease | -1.23 | Decrease | -0.57491186 |
| rs201271860 | T733I | Decrease | -0.95 | Decrease | -0.45187662 |
| rs201274041 | R359Q | Decrease | -1.12 | Decrease | -0.41437289 |
| rs201315884 | V37A | Decrease | -0.52 | Decrease | -1.8476443 |
| rs201467281 | S323G | Decrease | -0.59 | Decrease | -1.568492 |
| rs201628326 | H394N | Decrease | -1.00 | Decrease | -0.54247084 |
| rs201687037 | T301A | Decrease | -0.98 | Decrease | -0.51643105 |
| rs201789039 | M431T | Decrease | -0.71 | Decrease | -1.443167 |
| rs201861645 | L604V | Decrease | -0.41 | Decrease | -1.3463441 |
| rs201975130 | R580T | Decrease | -1.01 | Decrease | -0.38673067 |
| rs201980687 | R413S | Decrease | -2.18 | Decrease | -1.507355 |
| rs202047059 | T67A | Decrease | -1.25 | Decrease | -0.80153554 |
| rs202166889 | A1384G | Decrease | -0.93 | Decrease | -1.1548002 |
| rs367628460 | R412C | Decrease | -0.39 | Decrease | -1.1365864 |
| rs367634278 | T1126N | Decrease | -1.16 | Decrease | -0.77104845 |
| rs368144654 | T273S | Decrease | -0.57 | Decrease | -0.84074857 |
| rs368328347 | D94G | Decrease | -0.13 | Decrease | -0.97811334 |
| rs368750834 | V111I | Decrease | -0.91 | Decrease | -0.95037361 |
| rs368787826 | M822V | Increase | 0.50 | Decrease | -1.0530846 |
| rs368891381 | K876Q | Decrease | -1.24 | Decrease | -0.59110455 |
| rs368942722 | H61D | Decrease | -0.61 | Decrease | -0.41021632 |
| rs369312680 | R1336Q | Decrease | -1.86 | Decrease | -0.87277283 |
| rs369758288 | M852K | Decrease | -1.67 | Decrease | -1.7414081 |
| rs369838973 | R1170L | Decrease | -1.48 | Decrease | -0.22089626 |
| rs370314484 | Y369C | Increase | 0.68 | Decrease | -0.95249398 |
| rs370368651 | D1380E | Decrease | -0.40 | Decrease | -0.23467971 |
| rs370499060 | E49A | Decrease | -1.34 | Decrease | -0.65322941 |
| rs370529693 | P1073L | Decrease | -0.62 | Increase | 0.4210473 |
| rs370767911 | N1374K | Decrease | -0.90 | Decrease | -1.6389505 |
| rs370883654 | T555A | Decrease | -0.27 | Decrease | -1.1300476 |
| rs371124109 | T511M | Decrease | -0.75 | Decrease | -0.42400648 |
| rs371463233 | R412H | Decrease | -0.91 | Decrease | -1.522355 |
| rs371939364 | S794C | Decrease | -1.22 | Decrease | -1.522355 |
| rs372116735 | N620D | Increase | 0.50 | Decrease | -0.71408213 |
| rs372830789 | H1174R | Decrease | -1.02 | Decrease | -0.76871737 |
| rs373030463 | N704D | Decrease | -0.29 | Decrease | -0.92697745 |
| rs373312981 | G471E | Increase | 0.57 | Decrease | -0.57064022 |
| rs373419003 | P1027A | Decrease | -1.97 | Decrease | -1.2840794 |
| rs373517956 | A1375S | Decrease | -0.74 | Decrease | -1.2607403 |
| rs374050750 | A48T | Decrease | -1.69 | Decrease | -0.67828577 |
| rs374383028 | N1353D | Decrease | -0.97 | Decrease | -0.60926271 |
| rs374578653 | H58R | Decrease | -0.70 | Decrease | -0.39999154 |
| rs374733251 | T557A | Decrease | -0.37 | Decrease | -0.29719918 |
| rs375353223 | M35L | Decrease | -0.39 | Decrease | -0.94719299 |
| rs375391602 | R413H | Decrease | -0.66 | Decrease | -1.5459605 |
| rs375395179 | S1141L | Decrease | -1.51 | Decrease | -0.22501427 |
| rs375523121 | H780R | Decrease | -0.07 | Decrease | -0.52200469 |
| rs375576430 | S942L | Decrease | -0.86 | Increase | 0.23447337 |
| rs375951814 | T621I | Increase | 0.04 | Decrease | -0.3514325 |
| rs376097698 | L185I | Decrease | -1.24 | Decrease | -0.20746783 |
| rs376104371 | L893I | Decrease | -0.61 | Decrease | -0.42459199 |
| rs376243090 | F1012L | Decrease | -1.32 | Decrease | -0.80437823 |
| rs376244358 | M35I | Decrease | -0.47 | Decrease | -0.86467308 |
| rs376364468 | V427I | Decrease | -0.13 | Decrease | -1.1321991 |
| rs376418811 | I1337M | Decrease | -1.74 | Decrease | -0.9887432 |
| rs376459715 | S663L | Decrease | -0.59 | Decrease | -0.5467134 |
| rs376589619 | P472S | Decrease | -2.65 | Decrease | -0.8865637 |
| rs45450897 | I834M | Decrease | -1.80 | Decrease | -2.0987404 |
| rs45471794 | R982M | Decrease | -1.70 | Decrease | -0.60772829 |
| rs45516592 | N768D | Increase | 0.59 | Decrease | -1.4010759 |
| rs45531032 | I747V | Decrease | -1.44 | Decrease | -1.0526797 |
| rs45532942 | D1265Y | Increase | 0.22 | Decrease | -0.6690068 |
| rs45541232 | D1180N | Decrease | -0.58 | Decrease | -0.69380773 |
| rs45571834 | I1053T | Decrease | -3.91 | Decrease | -2.8875827 |
| rs45583838 | L1097V | Decrease | -1.75 | Decrease | -1.0068436 |
| rs45595632 | T1261A | Decrease | -1.59 | Decrease | -1.5390707 |
| rs45604032 | G933R | Decrease | -1.78 | Decrease | -0.59094222 |
| rs45605635 | Y649C | Increase | 0.34 | Decrease | -0.35709871 |
| rs56361366 | S661R | Decrease | -1.16 | Decrease | -0.27736488 |
| rs77651398 | N45S | Decrease | -1.32 | Decrease | -1.182256 |
| rs148526613 | N381T | Decrease | -1.31 | Decrease | -0.9790215 |
| rs189810227 | D1377N | Decrease | -2.63 | Decrease | -0.99949468 |
| rs199595181 | H148N | Decrease | -1.47 | Decrease | -0.58253587 |
| rs199808716 | G757E | Decrease | -0.07 | Decrease | -1.016227 |
| rs200023903 | T67I | Decrease | -0.35 | Decrease | -0.41050474 |
| rs200269358 | N571T | Decrease | -1.46 | Decrease | -0.31390648 |
| rs200341323 | N686K | Decrease | -0.90 | Decrease | -1.1572044 |
| rs201090828 | L675V | Decrease | -0.76 | Decrease | -0.71217739 |
| rs202110450 | H1136Q | Decrease | -1.01 | Decrease | -0.789679 |
| rs202135726 | N680K | Decrease | -0.37 | Decrease | -1.5064917 |
| rs202236031 | L403M | Increase | 0.37 | Decrease | -1.2045313 |
| rs267601244 | P1285S | Decrease | -2.53 | Decrease | -1.287108 |
| rs367722737 | S213L | Decrease | -0.89 | Increase | 0.063694092 |
| rs368312290 | P1322A | Decrease | -0.82 | Decrease | -1.1598329 |
| rs371941340 | F96C | Decrease | -1.24 | Decrease | -1.0309017 |
| rs372320153 | D77N | Decrease | -0.19 | Decrease | -0.75989872 |
| rs372699296 | V836I | Decrease | -1.03 | Decrease | -0.75217797 |
| rs376751279 | E1378V | Decrease | -0.31 | Decrease | -0.49051083 |
| rs376928397 | I799N | Decrease | -0.97 | Decrease | -1.6031408 |

**Deleterious nsSNPs of MET Gene Predicted by Six Computational Tools**

| **rsID** | **Amino Acid Substitution** | **SIFT** | **PROVEAN** | **PolyPhen-2** | **PATHER-PSEP** | **I-Mutant 2.0** | **MUpro** |
| --- | --- | --- | --- | --- | --- | --- | --- |
| rs34349517 | L238S | DELETERIOUS | DELETERIOUS | PROBABLY DAMAGING | PROBABLY DAMAGING | DECREASED STABILITY | DECREASED STABILITY |
| rs35776110 | A320V | DELETERIOUS | DELETERIOUS | PROBABLY DAMAGING | PROBABLY DAMAGING | DECREASED STABILITY | DECREASED STABILITY |
| rs45551737 | P239R | DELETERIOUS | DELETERIOUS | PROBABLY DAMAGING | PROBABLY DAMAGING | DECREASED STABILITY | DECREASED STABILITY |
| rs121913243 | H1094R | DELETERIOUS | DELETERIOUS | PROBABLY DAMAGING | PROBABLY DAMAGING | DECREASED STABILITY | DECREASED STABILITY |
| rs121913244 | H1094Y | DELETERIOUS | DELETERIOUS | PROBABLY DAMAGING | PROBABLY DAMAGING | DECREASED STABILITY | DECREASED STABILITY |
| rs121913247 | Y1230H | DELETERIOUS | DELETERIOUS | PROBABLY DAMAGING | PROBABLY DAMAGING | DECREASED STABILITY | DECREASED STABILITY |
| rs121913668 | M1131T | DELETERIOUS | DELETERIOUS | PROBABLY DAMAGING | PROBABLY DAMAGING | DECREASED STABILITY | DECREASED STABILITY |
| rs121913669 | V1188L | DELETERIOUS | DELETERIOUS | PROBABLY DAMAGING | PROBABLY DAMAGING | DECREASED STABILITY | DECREASED STABILITY |
| rs121913671 | D1228N | DELETERIOUS | DELETERIOUS | PROBABLY DAMAGING | PROBABLY DAMAGING | DECREASED STABILITY | DECREASED STABILITY |
| rs121913673 | L1195V | DELETERIOUS | DELETERIOUS | PROBABLY DAMAGING | PROBABLY DAMAGING | DECREASED STABILITY | DECREASED STABILITY |
| rs200016433 | A364T | DELETERIOUS | DELETERIOUS | PROBABLY DAMAGING | PROBABLY DAMAGING | DECREASED STABILITY | DECREASED STABILITY |
| rs200690492 | D340G | DELETERIOUS | DELETERIOUS | PROBABLY DAMAGING | PROBABLY DAMAGING | DECREASED STABILITY | DECREASED STABILITY |
| rs200776610 | T222M | DELETERIOUS | DELETERIOUS | PROBABLY DAMAGING | PROBABLY DAMAGING | DECREASED STABILITY | DECREASED STABILITY |
| rs375395179 | S1141L | DELETERIOUS | DELETERIOUS | PROBABLY DAMAGING | PROBABLY DAMAGING | DECREASED STABILITY | DECREASED STABILITY |
| rs45541232 | D1180N | DELETERIOUS | DELETERIOUS | PROBABLY DAMAGING | PROBABLY DAMAGING | DECREASED STABILITY | DECREASED STABILITY |
| rs45571834 | I1053T | DELETERIOUS | DELETERIOUS | PROBABLY DAMAGING | PROBABLY DAMAGING | DECREASED STABILITY | DECREASED STABILITY |
| rs45595632 | T1261A | DELETERIOUS | DELETERIOUS | PROBABLY DAMAGING | PROBABLY DAMAGING | DECREASED STABILITY | DECREASED STABILITY |
| rs199808716 | G757E | DELETERIOUS | DELETERIOUS | PROBABLY DAMAGING | PROBABLY DAMAGING | DECREASED STABILITY | DECREASED STABILITY |

**Effects of nsSNPs on structural & functional properties of MET by MutPred2 server**

| **rsID** | **Amino Acid Substitution** | **Probability of deleterious substitution** | **Top Features** | **Affected PROSITE and ELM Motifs** |
| --- | --- | --- | --- | --- |
| rs34349517 | L238S | 0.474 |  |  |
| rs35776110 | A320V | 0.808 | Altered Disordered interface (P=0.01) | ELME000155, ELME000239 |
|  |  |  | Loss of Relative solvent accessibility (P=0.03) |  |
|  |  |  | Gain of Allosteric site at Y321 (P=0.04) |  |
|  |  |  | Altered Transmembrane protein (P=0.02) |  |
| rs45551737 | P239R | 0.731 | Altered Transmembrane protein (P=0.0e+00) | None |
|  |  |  | Altered Ordered interface (P=0.04) |  |
|  |  |  | Gain of Helix (P=0.04) |  |
|  |  |  | Loss of Sulfation at Y234 (P=0.02) |  |
| rs121913243 | H1094R | 0.837 | Altered Metal binding (P=1.6e-04) | ELME000062, ELME000220, PS00107 |
|  |  |  | Gain of Strand (P=0.05) |  |
|  |  |  | Altered Transmembrane protein (P=8.3e-03) |  |
| rs121913244 | H1094Y | 0.817 | Altered Metal binding (P=6.3e-05) | ELME000020, ELME000120, ELME000220, PS00107 |
|  |  |  | Altered Ordered interface (P=6.3e-03) |  |
|  |  |  | Altered Transmembrane protein (P=0.02) |  |
| rs121913247 | Y1230H | 0.697 | Altered Metal binding (P=2.9e-03) | PS00007 |
|  |  |  | Altered Ordered interface (P=3.8e-03) |  |
|  |  |  | Loss of Relative solvent accessibility (P=0.02) |  |
|  |  |  | Loss of Allosteric site at R1227 (P=9.4e-03) |  |
|  |  |  | Loss of Phosphorylation at Y1234 (P=0.05) |  |
|  |  |  | Altered Transmembrane protein (9.0e-03) |  |
|  |  |  | Altered DNA binding (P=0.03) |  |
|  |  |  | Altered Stability (P=0.02) |  |
|  |  |  | Loss of Sulfation at Y1230 (P=0.02) |  |
| rs121913668 | M1131T | 0.923 | Loss of Helix (P= 0.03) | ELME000053 |
| rs121913669 | V1188L | 0.633 | Loss of Loop (P=0.04) | PS00008 |
|  |  |  | Gain of Acetylation at K1193 (P=0.01) |  |
|  |  |  | Loss of Allosteric site at M1192 (P=0.03) |  |
| rs121913671 | D1228N | 0.709 | Altered Ordered interface (P=0.05) | PS00007,  PS00008 |
|  |  |  | Loss of Relative solvent accessibility (P=0.03) |  |
|  |  |  | Gain of Allosteric site at R1227 (P=0.01) |  |
|  |  |  | Altered Transmembrane protein (P=0.01) |  |
|  |  |  | Altered Metal binding (P=0.05) |  |
|  |  |  | Loss of Sulfation at Y1230 (P=0.02) |  |
| rs121913673 | L1195V | 0.496 |  |  |
| rs200016433 | A364T | 0.478 |  |  |
| rs200690492 | D340G | 0.631 | Altered Metal binding (P=4.4e-03) | ELME000321, PS00008 |
|  |  |  | Loss of Relative solvent accessibility (P=5.3e-03) |  |
|  |  |  | Altered Transmembrane protein (P=1.5e-03) |  |
|  |  |  | Altered Stability (P=0.03) |  |
| rs200776610 | T222M | 0.317 |  |  |
| rs375395179 | S1141L | 0.668 |  | ELME000335 |
| rs45541232 | D1180N | 0.579 | Gain of Allosteric site at F1184 (P= 0.05) | ELME000106, ELME000220, PS00006 |
|  |  |  | Altered DNA binding (P=0.03) |  |
| rs45571834 | I1053T | 0.589 | Altered Stability (P=4.2e-04) | ELME000052, ELME000147 |
| rs45595632 | T1261A | 0.662 | Altered Metal binding (P=0.05) | ELME000053, ELME000146, ELME000220, ELME000239, PS00005 |
|  |  |  | Altered Transmembrane protein (P=0.03) |  |
|  |  |  | Altered Stability (P=0.05) |  |
| rs199808716 | G757E | 0.916 | Gain of Loop (P=5.2e-03) | ELME000053, ELME000063, ELME000085, ELME000147, PS00008 |
|  |  |  | Altered Transmembrane protein (P=0.02) |  |

**Conservancy scale of Deleterious nsSNPs**

| **rsID** | **Amino Acid Residue** | **Conservation scale** |
| --- | --- | --- |
| rs34349517 | L238 | 6 |
| rs35776110 | A320 | 9 |
| rs45551737 | P239 | 8 |
| rs121913243 | H1094 | 9 |
| rs121913244 | H1094 | 9 |
| rs121913247 | Y1230 | 6 |
| rs121913668 | M1131 | 9 |
| rs121913669 | V1188 | 9 |
| rs121913671 | D1228 | 9 |
| rs121913673 | L1195 | 9 |
| rs200016433 | A364 | 7 |
| rs200690492 | D340 | 5 |
| rs200776610 | T222 | 8 |
| rs375395179 | S1141 | 9 |
| rs45541232 | D1180 | 9 |
| rs45571834 | I1053 | 7 |
| rs45595632 | T1261 | 9 |
| rs199808716 | G757 | 9 |

**Position of Deleterious nsSNPs in secondary structure**

| **rsID** | **Amino Acid Residue** | **Secondary Structure Prediction** |
| --- | --- | --- |
| rs34349517 | L238 | Coil |
| rs35776110 | A320 | Helix |
| rs45551737 | P239 | Helix |
| rs121913243 | H1094 | Extracellular |
| rs121913244 | H1094 | Extracellular |
| rs121913247 | Y1230 | Coil |
| rs121913668 | M1131 | Helix |
| rs121913669 | V1188 | Helix |
| rs121913671 | D1228 | Extracellular |
| rs121913673 | L1195 | Helix |
| rs200016433 | A364 | Extracellular |
| rs200690492 | D340 | Coil |
| rs200776610 | T222 | Extracellular |
| rs375395179 | S1141 | Coil |
| rs45541232 | D1180 | Helix |
| rs45571834 | I1053 | Extracellular |
| rs45595632 | T1261 | Coil |
| rs199808716 | G757 | Coil |

**Deleterious nsSNPs present in Post Translational Modification Sites**

| **Amino Acid Residue** | **Post Translational Modification** | **Score** |
| --- | --- | --- |
| S966 | Phosphorylation | 0.811 |
| T977 | Phosphorylation | 0.873 |
| S985 | Phosphorylation | 0.654 |
| S988 | Phosphorylation | 0.838 |
| S990 | Phosphorylation | 0.908 |
| T992 | Phosphorylation | 0.602 |
| T993 | Phosphorylation | 0.566 |
| S997 | Phosphorylation | 0.877 |
| S1000 | Phosphorylation | 0.867 |
| Y1003 | Phosphorylation | 0.962 |
| S1032 | Phosphorylation | 0.597 |
| Y1230 | Phosphorylation | 0.966 |
| Y1234 | Phosphorylation | 0.969 |
| Y1235 | Phosphorylation | 0.97 |
| T1289 | Phosphorylation | 0.917 |
| Y1349 | Phosphorylation | 0.968 |
| Y1356 | Phosphorylation | 0.97 |
| Y1365 | Phosphorylation | 0.965 |
| S1370 | Phosphorylation | 0.742 |
| S1371 | Phosphorylation | 0.775 |
| S1385 | Phosphorylation | 0.76 |

**Effects of Deleterious nsSNPs on Different Properties of MET protein**

| **rsID** | **Amino Acid Substitution** | **Change of Property** |
| --- | --- | --- |
| rs34349517 | L238S | Smaller than wild type and less hydrophobic |
| rs35776110 | A320V | Bigger than wild type |
| rs45551737 | P239R | Bigger than wild type, neutral to positive, and  less hydrophobic |
| rs121913243 | H1094R | Bigger than wild type and neutral to positive |
| rs121913244 | H1094Y | Bigger than wild type and more hydrophobic |
| rs121913247 | Y1230H | Smaller than wild type and less hydrophobic |
| rs121913668 | M1131T | Smaller than wild type and less hydrophobic |
| rs121913669 | V1188L | Bigger than wild type |
| rs121913671 | D1228N | Negative to neutral |
| rs121913673 | L1195V | Smaller than wild type |
| rs200016433 | A364T | Bigger than wild type and less hydrophobic |
| rs200690492 | D340G | Smaller than wild type, negative to neutral, and more hydrophobic |
| rs200776610 | T222M | Bigger than wild type and more hydrophobic |
| rs375395179 | S1141L | Bigger than wild type and more hydrophobic |
| rs45541232 | D1180N | Negative to neutral |
| rs45571834 | I1053T | Smaller than wild type and less hydrophobic |
| rs45595632 | T1261A | Smaller than wild type and more hydrophobic |
| rs199808716 | G757E | Bigger than wild type, neutral to negative and less hydrophobic |

**Gene Interactions Data predicted by GeneMANIA**

| **Gene 1** | **Gene 2** | **Weight** | **Network group** | **Network** |
| --- | --- | --- | --- | --- |
| SEMA4D | CBL | 0.0052751745 | Co-expression | Wang-Maris-2006 |
| LECT2 | MET | 0.009972299 | Co-expression | Wang-Maris-2006 |
| FES | MET | 0.010145108 | Co-expression | Wang-Maris-2006 |
| FES | LECT2 | 0.004007075 | Co-expression | Wang-Maris-2006 |
| SH2B1 | PLXNB1 | 0.01966762 | Co-expression | Wang-Maris-2006 |
| SHD | MUC20 | 0.006650234 | Co-expression | Innocenti-Brown-2011 |
| SOCS1 | RND1 | 0.009348849 | Co-expression | Innocenti-Brown-2011 |
| RHOA | MET | 0.020991514 | Co-expression | Alizadeh-Staudt-2000 |
| SH2B1 | PLXNB1 | 0.0054434515 | Co-expression | Bild-Nevins-2006 B |
| SEMA4D | PTPN3 | 0.006899765 | Co-expression | Burington-Shaughnessy-2008 |
| SH2B2 | CBL | 0.0055336566 | Co-expression | Arijs-Rutgeerts-2009 |
| FES | PAX3 | 0.010089833 | Co-expression | Wu-Garvey-2007 |
| PTPN3 | HGF | 0.010065355 | Co-expression | Rosenwald-Staudt-2001 |
| RHOA | MET | 0.013719732 | Co-expression | Rosenwald-Staudt-2001 |
| SEMA4D | ARHGAP35 | 0.017395029 | Co-expression | Perou-Botstein-1999 |
| SH2B1 | ARHGAP35 | 0.016142916 | Co-expression | Perou-Botstein-1999 |
| CBL | MET | 0.7056816 | Co-localization | Chen-Huang-2014 |
| ARHGAP35 | CBL | 0.0009284352 | Genetic Interactions | Lin-Smith-2010 |
| PTPN3 | HGF | 0.0006943289 | Genetic Interactions | Lin-Smith-2010 |
| PTPN3 | CBL | 0.0005834258 | Genetic Interactions | Lin-Smith-2010 |
| SEMA4D | HGF | 0.0012218205 | Genetic Interactions | Lin-Smith-2010 |
| SEMA4D | CBL | 0.0010266626 | Genetic Interactions | Lin-Smith-2010 |
| LECT2 | ARHGAP35 | 0.0021164203 | Genetic Interactions | Lin-Smith-2010 |
| SOCS1 | HGF | 0.0011892909 | Genetic Interactions | Lin-Smith-2010 |
| SOCS1 | CBL | 0.0009993289 | Genetic Interactions | Lin-Smith-2010 |
| FES | RND1 | 0.0022266603 | Genetic Interactions | Lin-Smith-2010 |
| SH2B1 | CBL | 0.0023365403 | Genetic Interactions | Lin-Smith-2010 |
| HGF | MET | 0.011994241 | Pathway | Wu-Stein-2010 |
| CBL | MET | 0.0043845195 | Pathway | Wu-Stein-2010 |
| CBL | HGF | 0.007637407 | Pathway | Wu-Stein-2010 |
| MUC20 | MET | 0.070684865 | Pathway | Wu-Stein-2010 |
| MUC20 | HGF | 0.123126164 | Pathway | Wu-Stein-2010 |
| INPPL1 | MET | 0.023753842 | Pathway | Wu-Stein-2010 |
| INPPL1 | HGF | 0.041376885 | Pathway | Wu-Stein-2010 |
| PAX3 | MET | 0.06542567 | Pathway | Wu-Stein-2010 |
| PLXNB1 | SEMA4D | 0.21476543 | Pathway | Wu-Stein-2010 |
| RND1 | PLXNB1 | 0.09257329 | Pathway | Wu-Stein-2010 |
| SOCS1 | CBL | 0.005419993 | Pathway | Wu-Stein-2010 |
| GLMN | MET | 0.0866093 | Pathway | Wu-Stein-2010 |
| GLMN | HGF | 0.15086497 | Pathway | Wu-Stein-2010 |
| RHOA | ARHGAP35 | 0.010811585 | Pathway | Wu-Stein-2010 |
| HGF | MET | 0.017480196 | Pathway | NCI_NATURE |
| CBL | HGF | 0.0126832705 | Pathway | NCI_NATURE |
| MUC20 | MET | 0.14786214 | Pathway | NCI_NATURE |
| MUC20 | HGF | 0.15281369 | Pathway | NCI_NATURE |
| INPPL1 | MET | 0.19072096 | Pathway | NCI_NATURE |
| INPPL1 | HGF | 0.19710776 | Pathway | NCI_NATURE |
| PAX3 | MET | 0.32075316 | Pathway | NCI_NATURE |
| SH2B2 | CBL | 0.04579593 | Pathway | NCI_NATURE |
| RRAS | MET | 0.20801303 | Pathway | REACTOME |
| ARHGAP35 | MET | 0.30825618 | Pathway | REACTOME |
| SEMA4D | MET | 0.111992 | Pathway | REACTOME |
| SEMA4D | RRAS | 0.13380918 | Pathway | REACTOME |
| SEMA4D | ARHGAP35 | 0.1982929 | Pathway | REACTOME |
| RND1 | MET | 0.11574812 | Pathway | REACTOME |
| RND1 | RRAS | 0.13829704 | Pathway | REACTOME |
| RND1 | ARHGAP35 | 0.20494348 | Pathway | REACTOME |
| RND1 | SEMA4D | 0.074457645 | Pathway | REACTOME |
| FES | RRAS | 0.22573519 | Pathway | REACTOME |
| FES | RND1 | 0.12560955 | Pathway | REACTOME |
| RHOA | MET | 0.2228451 | Pathway | REACTOME |
| RHOA | SEMA4D | 0.14335026 | Pathway | REACTOME |
| RHOA | RND1 | 0.14815812 | Pathway | REACTOME |
| HGF | MET | 0.057538852 | Physical Interactions | Li-Fu-2017 |
| INPPL1 | MET | 0.07028273 | Physical Interactions | Leung-Jones-2014 |
| SHD | MET | 0.16810635 | Physical Interactions | Leung-Jones-2014 |
| SOCS1 | MET | 0.16810635 | Physical Interactions | Leung-Jones-2014 |
| FES | MET | 0.056807023 | Physical Interactions | Leung-Jones-2014 |
| SH2B2 | MET | 0.16810635 | Physical Interactions | Leung-Jones-2014 |
| SH2B1 | MET | 0.16810635 | Physical Interactions | Leung-Jones-2014 |
| RRAS | MET | 0.0826992 | Physical Interactions | IREF-reactome |
| ARHGAP35 | MET | 0.043608747 | Physical Interactions | IREF-reactome |
| SEMA4D | MET | 0.0651831 | Physical Interactions | IREF-reactome |
| SEMA4D | RRAS | 0.052488815 | Physical Interactions | IREF-reactome |
| SEMA4D | ARHGAP35 | 0.02767828 | Physical Interactions | IREF-reactome |
| PLXNB1 | MET | 0.07433277 | Physical Interactions | IREF-reactome |
| PLXNB1 | RRAS | 0.059856605 | Physical Interactions | IREF-reactome |
| PLXNB1 | ARHGAP35 | 0.031563446 | Physical Interactions | IREF-reactome |
| PLXNB1 | SEMA4D | 0.047178686 | Physical Interactions | IREF-reactome |
| RND1 | MET | 0.077295646 | Physical Interactions | IREF-reactome |
| RND1 | RRAS | 0.062242467 | Physical Interactions | IREF-reactome |
| RND1 | ARHGAP35 | 0.03282155 | Physical Interactions | IREF-reactome |
| RND1 | SEMA4D | 0.049059205 | Physical Interactions | IREF-reactome |
| RND1 | PLXNB1 | 0.055945583 | Physical Interactions | IREF-reactome |
| FES | MET | 0.061732966 | Physical Interactions | IREF-reactome |
| FES | RRAS | 0.049710587 | Physical Interactions | IREF-reactome |
| FES | SEMA4D | 0.039181642 | Physical Interactions | IREF-reactome |
| FES | PLXNB1 | 0.04468152 | Physical Interactions | IREF-reactome |
| FES | RND1 | 0.046462502 | Physical Interactions | IREF-reactome |
| SH2B2 | CBL | 0.0367465 | Physical Interactions | IREF-reactome |
| RHOA | MET | 0.019523786 | Physical Interactions | IREF-reactome |
| RHOA | ARHGAP35 | 0.008290259 | Physical Interactions | IREF-reactome |
| RHOA | SEMA4D | 0.012391661 | Physical Interactions | IREF-reactome |
| RHOA | PLXNB1 | 0.014131064 | Physical Interactions | IREF-reactome |
| RHOA | RND1 | 0.014694321 | Physical Interactions | IREF-reactome |
| RRAS | MET | 0.0826992 | Physical Interactions | Vastrik-Stein-2007 |
| ARHGAP35 | MET | 0.043608747 | Physical Interactions | Vastrik-Stein-2007 |
| SEMA4D | MET | 0.0651831 | Physical Interactions | Vastrik-Stein-2007 |
| SEMA4D | RRAS | 0.052488815 | Physical Interactions | Vastrik-Stein-2007 |
| SEMA4D | ARHGAP35 | 0.02767828 | Physical Interactions | Vastrik-Stein-2007 |
| PLXNB1 | MET | 0.07433277 | Physical Interactions | Vastrik-Stein-2007 |
| PLXNB1 | RRAS | 0.059856605 | Physical Interactions | Vastrik-Stein-2007 |
| PLXNB1 | ARHGAP35 | 0.031563446 | Physical Interactions | Vastrik-Stein-2007 |
| PLXNB1 | SEMA4D | 0.047178686 | Physical Interactions | Vastrik-Stein-2007 |
| RND1 | MET | 0.077295646 | Physical Interactions | Vastrik-Stein-2007 |
| RND1 | RRAS | 0.062242467 | Physical Interactions | Vastrik-Stein-2007 |
| RND1 | ARHGAP35 | 0.03282155 | Physical Interactions | Vastrik-Stein-2007 |
| RND1 | SEMA4D | 0.049059205 | Physical Interactions | Vastrik-Stein-2007 |
| RND1 | PLXNB1 | 0.055945583 | Physical Interactions | Vastrik-Stein-2007 |
| FES | MET | 0.061732966 | Physical Interactions | Vastrik-Stein-2007 |
| FES | RRAS | 0.049710587 | Physical Interactions | Vastrik-Stein-2007 |
| FES | SEMA4D | 0.039181642 | Physical Interactions | Vastrik-Stein-2007 |
| FES | PLXNB1 | 0.04468152 | Physical Interactions | Vastrik-Stein-2007 |
| FES | RND1 | 0.046462502 | Physical Interactions | Vastrik-Stein-2007 |
| SH2B2 | CBL | 0.0367465 | Physical Interactions | Vastrik-Stein-2007 |
| RHOA | MET | 0.019523786 | Physical Interactions | Vastrik-Stein-2007 |
| RHOA | ARHGAP35 | 0.008290259 | Physical Interactions | Vastrik-Stein-2007 |
| RHOA | SEMA4D | 0.012391661 | Physical Interactions | Vastrik-Stein-2007 |
| RHOA | PLXNB1 | 0.014131064 | Physical Interactions | Vastrik-Stein-2007 |
| RHOA | RND1 | 0.014694321 | Physical Interactions | Vastrik-Stein-2007 |
| PTPN3 | MET | 0.4694522 | Physical Interactions | Barr-Knapp-2009 |
| INPPL1 | CBL | 0.005671721 | Physical Interactions | Brehme-Superti-Furga-2009 |
| INPPL1 | CBL | 0.3064572 | Physical Interactions | IREF-quickgo |
| PLXNB1 | RRAS | 0.5578675 | Physical Interactions | IREF-quickgo |
| PLXNB1 | SEMA4D | 0.5578675 | Physical Interactions | IREF-quickgo |
| RND1 | PLXNB1 | 0.5578675 | Physical Interactions | IREF-quickgo |
| LECT2 | MET | 0.539355 | Physical Interactions | IREF-quickgo |
| GLMN | MET | 0.2233621 | Physical Interactions | IREF-quickgo |
| HGF | MET | 0.41173652 | Physical Interactions | IREF-dip |
| PLXNB1 | SEMA4D | 0.76536685 | Physical Interactions | IREF-dip |
| RND1 | ARHGAP35 | 0.76536685 | Physical Interactions | IREF-dip |
| RND1 | PLXNB1 | 0.41421357 | Physical Interactions | IREF-dip |
| GNG12 | MET | 0.7589716 | Physical Interactions | Havugimana-Emili-2012 |
| RND1 | PLXNB1 | 0.32323816 | Physical Interactions | IREF-uniprotpp |
| HGF | MET | 0.076532066 | Physical Interactions | BIOGRID-SMALL-SCALE-STUDIES |
| CBL | MET | 0.0066243554 | Physical Interactions | BIOGRID-SMALL-SCALE-STUDIES |
| INPPL1 | MET | 0.04551932 | Physical Interactions | BIOGRID-SMALL-SCALE-STUDIES |
| INPPL1 | CBL | 0.011946155 | Physical Interactions | BIOGRID-SMALL-SCALE-STUDIES |
| PLXNB1 | SEMA4D | 0.21083972 | Physical Interactions | BIOGRID-SMALL-SCALE-STUDIES |
| RND1 | ARHGAP35 | 0.05947727 | Physical Interactions | BIOGRID-SMALL-SCALE-STUDIES |
| RND1 | PLXNB1 | 0.040668495 | Physical Interactions | BIOGRID-SMALL-SCALE-STUDIES |
| SOCS1 | MET | 0.010417426 | Physical Interactions | BIOGRID-SMALL-SCALE-STUDIES |
| GLMN | MET | 0.06058516 | Physical Interactions | BIOGRID-SMALL-SCALE-STUDIES |
| SH2B2 | CBL | 0.016796818 | Physical Interactions | BIOGRID-SMALL-SCALE-STUDIES |
| SH2B2 | INPPL1 | 0.1154195 | Physical Interactions | BIOGRID-SMALL-SCALE-STUDIES |
| SH2B1 | SH2B2 | 0.14031015 | Physical Interactions | BIOGRID-SMALL-SCALE-STUDIES |
| RHOA | ARHGAP35 | 0.022769572 | Physical Interactions | BIOGRID-SMALL-SCALE-STUDIES |
| RHOA | PLXNB1 | 0.015569045 | Physical Interactions | BIOGRID-SMALL-SCALE-STUDIES |
| CBL | MET | 0.035542484 | Physical Interactions | IREF-mint |
| PLXNB1 | MET | 0.15904064 | Physical Interactions | IREF-mint |
| RND1 | PLXNB1 | 0.6295244 | Physical Interactions | IREF-mint |
| SH2B2 | CBL | 0.081416406 | Physical Interactions | IREF-mint |
| HGF | MET | 1 | Physical Interactions | IREF-corum |
| PLXNB1 | SEMA4D | 0.851885 | Physical Interactions | IREF-corum |
| HGF | MET | 0.57281905 | Physical Interactions | IREF-bind-translation |
| CBL | MET | 0.04929598 | Physical Interactions | IREF-bind-translation |
| INPPL1 | MET | 0.3229385 | Physical Interactions | IREF-bind-translation |
| RND1 | PLXNB1 | 0.88479984 | Physical Interactions | IREF-bind-translation |
| HGF | MET | 0.64922225 | Physical Interactions | IREF-bind |
| CBL | MET | 0.06676539 | Physical Interactions | IREF-bind |
| INPPL1 | MET | 0.3719139 | Physical Interactions | IREF-bind |
| RND1 | PLXNB1 | 1 | Physical Interactions | IREF-bind |
| HGF | MET | 0.013028996 | Physical Interactions | IREF-matrixdb |
| CBL | MET | 0.039134648 | Physical Interactions | IREF-matrixdb |
| INPPL1 | MET | 0.0680915 | Physical Interactions | IREF-matrixdb |
| PTPN3 | MET | 0.033827048 | Physical Interactions | IREF-matrixdb |
| PLXNB1 | MET | 0.012673709 | Physical Interactions | IREF-matrixdb |
| PLXNB1 | SEMA4D | 0.19407418 | Physical Interactions | IREF-matrixdb |
| RND1 | PLXNB1 | 0.4193324 | Physical Interactions | IREF-matrixdb |
| SHD | MET | 0.16414404 | Physical Interactions | IREF-matrixdb |
| SOCS1 | MET | 0.16414404 | Physical Interactions | IREF-matrixdb |
| FES | MET | 0.04173584 | Physical Interactions | IREF-matrixdb |
| SH2B2 | MET | 0.16414404 | Physical Interactions | IREF-matrixdb |
| SH2B1 | MET | 0.11172158 | Physical Interactions | IREF-matrixdb |
| CBL | MET | 0.039352205 | Physical Interactions | IREF-spike |
| PLXNB1 | MET | 0.17824732 | Physical Interactions | IREF-spike |
| HGF | MET | 0.051388726 | Physical Interactions | IREF-biogrid |
| CBL | MET | 0.0081320135 | Physical Interactions | IREF-biogrid |
| INPPL1 | MET | 0.014848727 | Physical Interactions | IREF-biogrid |
| INPPL1 | CBL | 0.004902164 | Physical Interactions | IREF-biogrid |
| GNG12 | MET | 0.046827503 | Physical Interactions | IREF-biogrid |
| RND1 | ARHGAP35 | 0.1482535 | Physical Interactions | IREF-biogrid |
| SOCS1 | MET | 0.014996564 | Physical Interactions | IREF-biogrid |
| SH2B2 | CBL | 0.026728062 | Physical Interactions | IREF-biogrid |
| SH2B2 | INPPL1 | 0.04880436 | Physical Interactions | IREF-biogrid |
| SH2B1 | SH2B2 | 0.1775593 | Physical Interactions | IREF-biogrid |
| RHOA | ARHGAP35 | 0.02260142 | Physical Interactions | IREF-biogrid |
| SEMA4D | MET | 0.104945496 | Predicted | Wu-Stein-2010 |
| PLXNB1 | MET | 0.1152263 | Predicted | Wu-Stein-2010 |
| RHOA | RND1 | 0.08313782 | Predicted | Wu-Stein-2010 |
| RND1 | PLXNB1 | 0.70710677 | Predicted | I2D-BioGRID-Rat2Human |
| MUC20 | MET | 0.7853231 | Predicted | I2D-BioGRID-Mouse2Human |
| SEMA4D | MET | 0.011310431 | Shared protein domains | INTERPRO |
| PLXNB1 | MET | 0.01303379 | Shared protein domains | INTERPRO |
| PLXNB1 | SEMA4D | 0.017338678 | Shared protein domains | INTERPRO |
| SOCS1 | SHD | 0.012974091 | Shared protein domains | INTERPRO |
| FES | MET | 0.005206651 | Shared protein domains | INTERPRO |
| SH2B2 | SHD | 0.016145678 | Shared protein domains | INTERPRO |
| SH2B2 | SOCS1 | 0.01636524 | Shared protein domains | INTERPRO |
| SH2B1 | SHD | 0.01613468 | Shared protein domains | INTERPRO |
| SH2B1 | SOCS1 | 0.016368506 | Shared protein domains | INTERPRO |
| SH2B1 | SH2B2 | 0.0643932 | Shared protein domains | INTERPRO |
| RHOA | RND1 | 0.019796582 | Shared protein domains | INTERPRO |
| SEMA4D | MET | 0.013703098 | Shared protein domains | PFAM |
| PLXNB1 | MET | 0.024387928 | Shared protein domains | PFAM |
| PLXNB1 | SEMA4D | 0.013346357 | Shared protein domains | PFAM |
| RND1 | RRAS | 0.007544877 | Shared protein domains | PFAM |
| SHD | INPPL1 | 0.008589158 | Shared protein domains | PFAM |
| SOCS1 | SHD | 0.010380636 | Shared protein domains | PFAM |
| FES | SHD | 0.008739008 | Shared protein domains | PFAM |
| SH2B2 | SHD | 0.011812418 | Shared protein domains | PFAM |
| SH2B1 | SHD | 0.011812418 | Shared protein domains | PFAM |
| SH2B1 | SH2B2 | 0.076938115 | Shared protein domains | PFAM |
| RHOA | RRAS | 0.007651891 | Shared protein domains | PFAM |
| RHOA | RND1 | 0.007544877 | Shared protein domains | PFAM |
